# Discovery of Potential Antiviral Compounds against Hendra Virus by Targeting Its Receptor-Binding Protein (G) Using Computational Approaches

**DOI:** 10.3390/molecules27020554

**Published:** 2022-01-16

**Authors:** Faisal Ahmad, Aqel Albutti, Muhammad Hamza Tariq, Ghufranud Din, Muhammad Tahir ul Qamar, Sajjad Ahmad

**Affiliations:** 1 National Center for Bioinformatics, Quaid-i-Azam University, Islamabad 45320, Pakistan; faisalahmad@bs.qau.edu.pk; 2 Department of Medical Biotechnology, College of Applied Medical Sciences, Qassim University, Buraydah 51452, Saudi Arabia; 3 Department of Biotechnology, Virtual University of Pakistan, Lahore 54000, Pakistan; hamza382@gmail.com; 4 Department of Medical Lab Technology, The University of Haripur, Haripur 22660, Pakistan; ghufran.uddin@uoh.edu.pk; 5 College of Life Science and Technology, Guangxi University, Nanning 530004, China; m.tahirulqamar@hotmail.com; 6 Department of Health and Biological Sciences, Abasyn University, Peshawar 25000, Pakistan

**Keywords:** Hendra virus, drug discovery, molecular docking, molecular dynamics simulations

## Abstract

Hendra virus (HeV) belongs to the paramyxoviridae family of viruses which is associated with the respiratory distress, neurological illness, and potential fatality of the affected individuals. So far, no competitive approved therapeutic substance is available for HeV. For that reason, the current research work was conducted to propose some novel compounds, by adopting a Computer Aided Drug Discovery approach, which could be used to combat HeV. The G attachment Glycoprotein (Ggp) of HeV was selected to achieve the primary objective of this study, as this protein makes the entry of HeV possible in the host cells. Briefly, a library of 6000 antiviral compounds was screened for potential drug-like properties, followed by the molecular docking of short-listed compounds with the Protein Data Bank (PDB) structure of Ggp. Docked complexes of top two hits, having maximum binding affinities with the active sites of Ggp, were further considered for molecular dynamic simulations of 200 ns to elucidate the results of molecular docking analysis. MD simulations and Molecular Mechanics Energies combined with the Generalized Born and Surface Area (MMGBSA) or Poisson–Boltzmann and Surface Area (MMPBSA) revealed that both docked complexes are stable in nature. Furthermore, the same methodology was used between lead compounds and HeV Ggp in complex with its functional receptor in human, Ephrin-B2. Surprisingly, no major differences were found in the results, which demonstrates that our identified compounds can also perform their action even when the Ggp is attached to the Ephrin-B2 ligand. Therefore, in light of all of these results, we strongly suggest that compounds (*S*)-5-(benzylcarbamoyl)-1-(2-(4-methyl-2-phenylpiperazin-1-yl)-2-oxoethyl)-6-oxo-3,6-dihydropyridin-1-ium-3-ide and 5-(cyclohexylcarbamoyl)-1-(2-((2-(3-fluorophenyl)-2-methylpropyl)amino)-2-oxoethyl)-6-oxo-3,6-dihydropyridin-1-ium-3-ide could be considered as potential therapeutic agents against HeV; however, further in vitro and in vivo experiments are required to validate this study.

## 1. Introduction

A growing number of emerging viral diseases is due to the natural viral reservoirs in different animals such as bats [1], and these zoonotic viruses are estimated to be responsible for 70% of infectious diseases [2]. One such pathogenic Zoonotic origin virus is Hendra virus (HeV), which was discovered in 1999. Soon after their discovery, fruit bats of the Pteropodidae family were found to be the natural host of HeV [3]. HeV belongs to the Paramyxoviruses family of viruses; however, several features distinguish them from other Paramyxoviruses, including unique specific 3′ leader and 5′ trailer sequences that act as the promoters to transcribe and replicate the genomic RNA, the existence of a highly conserved catalytic site sequence GDNE instead of the GDNQ sequence in the “Transcriptase Protein” [4,5], and the entire length of the genome. The HeV genome consists of 18,234 nucleotides (nt) and is approximately 15% longer than other genomes [4]. 

HeV is extremely pathogenic in nature, and several outbreaks in horses, pigs, and humans have been reported globally with well-characterized respiratory distress and neurological diseases. In humans, the estimated mortality rate due to HeV lies between 50 and 100%, making it one of the deadliest viruses of all times [6]. Although the total number of affected individuals is very small, the World Health Organization has placed HeV on the Blueprint priority disease list [7]. Diseases mentioned in this list have very little or no available treatments, whereas owing to their epidemic potential, they impart a serious public health risk. According to the HeV fact sheet available on the WHO official website, the first reported outbreak of HeV was in 1994 in the Brisbane suburb of Hendra, Australia; during the outbreak, 21 racehorses and two humans that were taking care of them were affected. Since July 2016, around 53 incidents associated with HeV have been reported, affecting more than 70 horses. All of these incidents were restricted to the north-eastern coastal region of Australia. During the incidences, seven humans were infected with Hendra virus during close contact while taking care of the horses or disposing of deceased animals. According to the report, the fatality rate due to Hendra virus in humans and horses is 60% and 80%, respectively [8]. However, an accurate mortality rate in equines cannot be determined as infected animals with a positive diagnosis of HeV are euthanized immediately [8]. 

The study is based on a hypothesis of a high fatality rate of this virus in the case of both animals and humans, which make it a high-risk group 4 pathogen, as no proper drug or vaccine has been approved as a therapeutic. Therefore a novel drug strategy is required to target its already identified protein with potent inhibitor molecules. 

Similar to many other zoonotic viruses, HeV does not transfer from bats to humans directly; rather, dates and palm sap that have been contaminated by the feces and saliva of bats provide a route for transmission in humans and therefore exhibit a considerable concern for the international public health community [9,10]. Humans infected with HeV usually demonstrate clinical symptoms of fatigue, headache, pneumonia, ataxia, vomiting, high fever, sore throat, and dry cough. Severe infection often results in respiratory distress, encephalitis, neurological diseases, and eventually death [11,12,13]. Currently, no approved drug is available against HeV, although a few anti-viral drugs (including Ribavirin and Chloroquine) have been reported to be effective against HeV; however, they only assist in the delay of the virus and do not prevent its deadly effects [1,2,3].

HeV, just like Nipah Virus, measles, canine distemper, and rinderpest, enters cells through surface glycoproteins that bind to the cell membrane of human receptors. The pathogenicity of HeV in the human body starts with the attachment of their G attachment glycoprotein (Ggp) with the Ephrin-B2, which acts as the cellular receptor of HeV in humans. Ephrin-B2 is a type II integral membrane protein with an *N*-terminal cytoplasmic tail, a single transmembrane helix, a stalk region, and a *C*-terminal six-bladed b-propeller. The crystal structures of HeV-Ggp in combination with the human Ephrin-B2 ligand reveal surprising characteristics of the selectivity of this interaction, which have been confirmed by biophysical investigations [14,15]. As Ggp is responsible for the attachment of HeV on the host cellular receptors, inhibiting Ggp will eventually prohibit the attachment of HeV with the human receptors Ephrin-B2, which will ultimately inhibit the pathogenesis of HeV. 

Many previous studies based on computer-aided drug discovery (CADD) against pathogens have also considered surface proteins as a therapeutic target of pathogens. For example, Shehroz et al., used CADD protocols to design a drug against the spike glycoprotein of SARS-CoV-2 [16]. Similarly, Anasir et al. and Dash et al., designed therapeutic substances against the surface glycoprotein of Dengue virus [17] and Ebola virus [18], respectively.

Recently, the use of bioinformatics tools has revolutionized the field of drug discovery, as with the aid of these computational methods, the therapeutic potential of a variety of substances of interest can be assessed against different diseases. This saves wet lab resources as well as time and finally proposes therapeutic substances with strong potential to act as a drug against the disease under consideration [19]. The proposed study also employed different computational approaches, such as virtual screening of chemical compounds, molecular docking, and molecular dynamics simulation analysis, to identify the “best hits” that will serve as a baseline for the pharmaceutical industry to combat HeV.

## 2. Methodology

### 2.1. Drug Target Selection and Preparation

The G attachment glycoprotein (Ggp) of HeV was selected to identify the potential inhibitors of HeV. To begin the research work, the three-dimensional structure of Ggp in complex with human Ephrin-B2 receptors was retrieved from the protein data bank (PDB) [20], under PDB ID: 2VSK. This structure was used in two distinct ways for further analysis; one in the native complex form and the other one in discrete Ggp form. The intact Ggp structure was separated from the docked complex through Discovery Studio 3.5v 2019 Vélizy-Villacoublay FRANCE [21]. The purpose of considering both the un-bounded and bounded Ggp structure was to identify the effect of inhibitors on Ggp in two different scenarios; when the virus exists in intact form and when the virus is attached to the host cellular receptors. In order to improve the quality of the selected models of the target proteins, energy minimization was done through UCSF Chimera 1.15v 2020 University of California USA. This software is also a powerful visualization software; hence, it was also considered to analyze the final prepared structures [22].

### 2.2. Small Molecule Preparation

Potential inhibitors against the target were gathered using the Asinex BioDesign database (http://www.asinex.com) antiviral library, on 22 July 2021. This library of small chemicals was further used in structure-based virtual screening. Prior to that, the library was screened for drug-like inhibitors first, followed by screening for lead-like inhibitors using the Swiss-ADME online tool (http://www.swissadme.ch/) on 23 July 2021 [23]. Firstly, investigations were made to check whether the compounds present in the library violated drug-like rules, namely, the Lipinski rule of five, Muegge rule, Egan rule, and Veber rule, each of which evaluates drug-like properties based on distinct parameters [24,25,26,27]. Additionally, different medicinal chemistry parameters, Pan Assay Interference (PAINS), Synthetic accessibility, and Brenk Alert, were also considered to short-list the compounds [28,29,30]. Compounds with no PAINS substructure, high synthetic accessibility, and minimum Brenk Alert were eventually considered to be the lead compounds. Compounds following all of the above-mentioned rules were further screened for Adsorption, Distribution, Metabolism, Excretion, and Toxicity (ADMET)-related properties [31]. Both HIA and BBB were estimated using a BOILED-EGG plot [32]. This plot examines the TPSA and LogP of the compound under consideration and predicts its pharmacokinetics with respect to BBB and HIA [32]. Another important parameter considered while estimating pharmacokinetics is solubility in water, as high solubility means even a small volume of the drug is sufficient for absorption within the body [33]. Moreover, Immunogenecity, Mutagenecity, AMES toxicity, and Pro-ToxII tests predictions were carried out to estimate the toxicity of the short-listed compounds.

Finally, a comparison between the drug-likeliness and non-drug-likeliness of the lead compounds was done through the molsoft online tool (http://molsoft.com/) on 25 July 2021, whose working is based on different physiochemical properties, including the molecular weight, number of HBDs, number of HBAs, MolLogP, MolLogS, MolPSA, MolVol, number of stereo centers, BBB score, and pKa of acidic and basic groups [34]. A total of 6000 compounds were isolated from the library after applying the aforementioned filters, with 3560 lead-like compounds being chosen for further study. Compounds with acceptable lead-likeness capabilities were then separated from the other compounds. The energy of these potent inhibitors was then minimized using the MMFF94 force field using Discovery Studio 2017.

### 2.3. Molecular Docking

Active site identification, ligand preparation, and molecular docking were the three main stages in the molecular docking approach. From a literature point of view, the active pocket residues reside among the loop region Glu119-Trp125 and other essential binding residues, i.e., Tyr255, Asp219 Tyr305, Lys357, Tyr508, Gln559, Tyr581, and Gln 579, that catalyze the hydrolysis of the alpha-(2->3)-, alpha-(2->6)-, alpha-(2->8)-glycosidic linkages of terminal sialic residues in oligosaccharides and glycoproteins [1]. The active cavity in the protein three-dimensional structure of molecule binding must be investigated for effective suppression. Appropriate active sites are classified according to their buriedness, size, shape, and hydrophobic considerations [35]. The active site for (PDB-2VSK) was identified from the literature review and was confirmed manually via sequence alignment of our target sequence. The active site residue confirmed during physical examinations was similar in comparison to the one reported in the literature.

Then, molecular docking was performed following the same protocol published in our previous studies [36,37,38] between 3560 lead-like compounds with the un-bounded structure of Ggp by using AutoDock Vina v.1.2.0 software CCSB California USA, whose working is based on the protocols of Genetic Optimization [39]. The best ligands against the target protein were classified based on their binding affinities. To visualize the docked protein complexes and the interactions that lead to ligand binding in detail, UCSF Chimera [21] and Visual Discovery studio (DS) [21] were applied. The top ten compounds were then docked against the complex of Ggp and Ephrin-B2 receptors to check their binding affinity with the bounded form of Ggp.

### 2.4. Molecular Dynamics (MD) Simulation

MD simulations were used to investigate the behavior of docked proteins using an explicit water molecules (TIP3P) water box with a size dimension of 8.0 Å having Na^+^ or Cl^−^, according to the solute charge to neutralize the system. During the system preparation, the protein was assembled using the pdb4amber tool, where its protonation state and missing residues were adjusted. Assisted Model Building with Energy Refinement (AMBER 16) software (University of California, San Francisco) was used, and several modules were applied for analysis [40]. The initial coordinates and topological files were generated using the TLeap package of (AMBER 16) software. The facility was built using the ff14SB force field. A constant temperature was achieved using the Berendsen Coupling Integration Algorithm. The Process TRAJectory (PTRAJ) module of (AMBER 16) was used to conduct the outcomes analysis. Xmgrace 5.1.25v University of Cambridge UK, was used to compute and visualize four attributes [41]. The same protocol was followed for MD simulations as published in our previous studies [36,37,38,42]. The simulation time was extended to 200 ns, and the root mean square deviation (RMSD), root mean square fluctuation and (RMSF), radius of gyration (Rg), and B-factor analyses were performed.

### 2.5. Radial Distribution Function

The radial distribution function, *g*(*r*), for a group of N atoms is the probability of finding an atom in a spherical volume of radius r [43]. In the current study, RDFs between amino acids in the vicinity of the pocket and ligand were plotted to gain insight into conformational variations induced by intermolecular interactions. Radial distribution functions (RDFs) were extracted using PTRAJ in order to quantify the density of a specified atom around another definite atom at an ideal distance for interaction. The expression for the radial distribution function is presented in the Equation (1).

*gr* = *ρijr*<*ρj*> = *nijr*<*ρj*>4*πr*Δ*r* (1)

In the above equation, *ρij* is the observed number density of one specified atom of the molecule (solvent) at a specified distance, (*r*), from one atom of the other molecule (solute). The function *g*(*r*) is the ratio between this observed number density *ρij* at the distance r and the average bulk atom number density of the solvent, *ρj*. This ratio is equal to the ratio between *nij*(*r*) and <*ρj*>4*πr*2Δ*r* where *nij* is the bin number of atoms in a spherical volume fragment, dependent on the bin width Δ*r*. In other words, the RDF provides the probability of finding the one specified atom of the solute in a specified volume, at a distance (*r*) from another specified atom of the solvent. The factor 4*πr*2Δ*r* gives the volume of a spherical shell with a thickness, Δ*r*, at a distance (*r*) from the chosen solute atom. The ratio between the number of solvent atoms found in this spherical volume (4*πr*2Δ*r*) at a distance (*r*) from the specified solute atom and the bulk density of solvent atoms (again molecules) gives the probability of finding the solvent atom at this distance from the solute atom as compared to a solute atom in bulk solution, which is referred to as the *g*(*r*) value. 

### 2.6. Binding Free Energy Calculations

The MMPBSA method was used to calculate the binding free energies against an average of 1000 frames of MD simulations [44] by calculating the difference among the individual free energy of the ligand and receptors and both in a single complex entity (Equation (2)):

Δ*G_bind_* = Δ*G_complex_* − Δ*G_receptor_* − Δ*G_ligand_* (2)

The ∆*G* in Equation (6) is Gibb’s free energy that is calculated by MMGB/PBSA for each term as shown in Equation (3):

Δ*G* = *E_gas_* + Δ*G_solv_* − *TS_solute_* (3)

where *T* depicts temperature and *S* demonstrates the entropy contribution to ligand binding, which was predicted through identified approximations. *E_gas_* shows the gas phase energy, which in most of the cases, represents the MM energy from the force field. *E_gas_* basically covers contributions from both internal energy and van der Waals’ interaction and electrostatic energies (Equation (4)):

*E_gas_* = *E_int_* + *E_ele_* + *E_vdw_* (4)

Another important value is that of Δ*G_solv_*, which is estimated through an implicit solvent model. Δ*G_solv_* considers both non-polar and electrostatic contributions (Equation (5)):

Δ*G_solv_* = Δ*G_ele_* + Δ*G_np_G_ele_* (5)

Equation (5) is the addition of electrostatic energy and polar solvation components (Equation (6)):

Δ*G_ele_PB*/*GB* = *E_ele_* + Δ*GPB*/*GB* (6)

In Equation (6), G_np_ is the non-electrostatic contribution and is proportional to the solvent-accessible surface area of the molecule (Equation (7)), which is calculated through the LCPO (Linear Combinations of Pairwise Overlaps) method [44] in MMPBSA with a water probe radius of 1.4 Å.

Δ*G_np_* = *γSAS* + *β* (7)

The standard values for constants *γ* and *β* in Equation (7) were used in this analysis, which are 0.0072 kcal/mol·Å^2^ and 0 kcal/mol, respectively, in MMGBSA and 0.0052 kcal/mol·Å^2^ and 0.92 kcal/mol, respectively, in MMPBSA.

### 2.7. WaterSwap Absolute Binding Free Energy Calculations

The WaterSwap technique from the Sire package2020.1.0 (University of Bristol), based on the explicit solvent model, was also ad used to further assess the binding free energies of our systems [45,46]. The working of this technique involves the swapping of ligand dimensions with an equal volume and size of the binding site-explicit water molecules, making it possible to calculate the free energy contribution of the water molecules, which are contemporaneous with the binding pocket of Ggp. To perform the WaterSwap analysis, trajectories from the last 10 ns MD simulations were considered, and in total, 1000 iterations were conducted. Four distinct methods of binding free energy calculation were considered for the estimation of absolute binding free energy, namely, thermodynamic integration (TI), free energy perturbation (FEP), quadrature-based integration of TI, and Bennett’s acceptance ratio (BAR) method. The final forecasted values of <1 kcal/mol were evaluated to be the ideal values, depicting the overall high stability in the studied complexes [47].

## 3. Results

### 3.1. Filtering Antiviral Chemical Libraries

The current study was aimed to identify the novel inhibitors of the Ggp of Hendra virus. For this purpose, a library of energy minimized 3560 antiviral compounds (http://www.asinex.com/?page_id=17) (accessed on 7 January 2022) (with acceptable drug-like properties) was docked with the active sites of the Ggp in two forms; Ggp unbounded and Ggp bounded with Ephrin-B2. Once the molecular docking was completed, antiviral compounds were ranked based on four different parameters, i.e., minimum Gibbs free energy, maximum occupancy of the binding pocket, strength of hydrogen bonding, and other possible non-covalent contacts. The top five best docked compounds were the same in both studied scenarios; their chemical structures and Gibbs free energies are shown in Table 1, which clearly indicates that these top five compounds had a free energy within the range of −9.7 kcal/mol and −7.4 kcal/mol.

**Figure 1:**
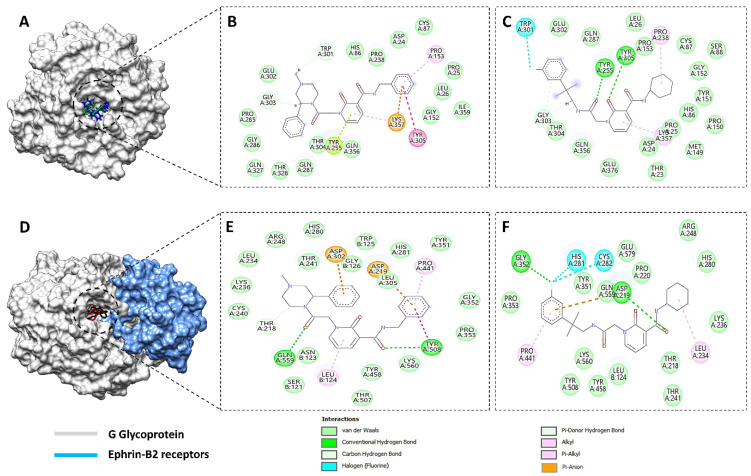
Binding conformation and chemical interaction network of top 1 and top 2 compounds within the binding pocket of unbounded and bounded Ggp with the Ephrin-B2 receptor. (**A**) Docked complex of unbounded Ggp with the top 1 (Green) and top 2 (Blue) inhibitors, (**B**) Interaction map of top inhibitor with the active residues of unbounded Ggp, (**C**) Interaction map of the top 2 inhibitor with the active residues of unbounded Ggp, (**D**) Docked complex of bounded Ggp with the top 1 (Red) and top 2 (Black) inhibitors, (**E**) Interaction map of the top inhibitor with the active residues of bounded Ggp, (**F**) Interaction map of the top 2 inhibitor with the active residues of bounded Ggp. The coordinates of the complexes are shown in Supplementary Materials.

Among these top five compounds, the two top-most compounds had a Gibbs free energy considerably lower than the others; these two antiviral agents are Top1 [(S)-5-(benzylcarbamoyl)-1-(2-(4-methyl-2-phenylpiperazin-1-yl)-2-oxoethyl)-6-oxo-3,6-dihydropyridin-1-ium-3-ide] and Top2 [5-(cyclohexylcarbamoyl)-1-(2-((2-(3-fluorophenyl)-2-methylpropyl)amino)-2-oxoethyl)-6-oxo-3,6-dihydropyridin-1-ium-3-ide]. The docking poses and binding interactions of these best docked compounds with the active residues of Ggp are shown in Figure 1.

All of the selected compounds, which were used for docking analysis, were previously validated for acceptable drug-like properties. Table 2 shows the physiochemical properties of the top two hits, which were evaluated by different rules of drug-likeliness. This table also states that these compounds have zero violation for all the commonly considered rules of drug-likeliness, i.e., Lipinski rule of 5, Veber rule, Muegge rule, and Egan rule. Furthermore, they also showed acceptable characteristics of medicinal chemistry, including Pan-assay interference compounds (PAINS), structure alert (brenk), and synthetic accessibility. The top two compounds also demonstrated normal parameters of Absorption, Distribution, Metabolism, Excretion and Toxicity (ADMET).

The BOILED-EGG plot is illustrated in Figure S1, according to which the analyzed compounds lie within the white region of the egg, depicting strong human intestinal absorption and incapability of crossing the blood–brain barrier, which is again a strongly desirable characteristic of a potential therapeutic substance. Likewise, other ADMET-related properties were normal in both of the candidate inhibitors (Table S1), with only 06 and 04 violations in the top 1 and top 2 hits, respectively. Finally, a comparison between the drug-like and non-drug-like properties was made through the molsoft online tool, the results of which also showed that the analyzed compounds had significantly more drug-like properties than non-drug-like ones (Figure S2).

### 3.2. MD Simulations

**Figure 2:**
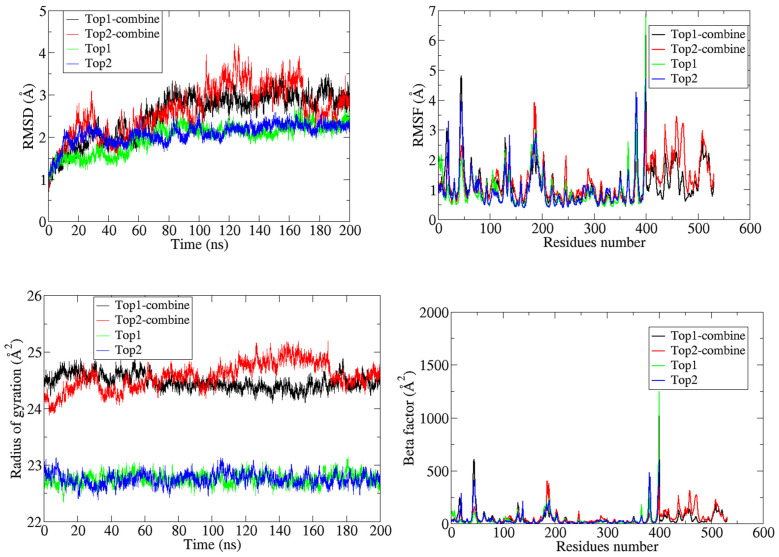
Superimposed RMSD, RMSF, Rg, and beta-factor analysis for the docked complexes of the top two hits during the 200 ns MD simulation, where Top1 and Top2 represent the simulation of the docked complex of the top 1 and top 2 compounds with the native Ggp of HEV, respectively, and Top1-combine and Top2-combine (form) show the docked complex of the top 1 and top 2 compounds with the discrete Ggp of HEV attached to Ephrin-B2 receptors.

The top two best categorized docked complexes were additionally considered for molecular dynamic simulations to validate the molecular docking results. MD simulations were performed for 200 ns, and the stability of the complex was estimated through different statistical parameters, i.e., root mean square fluctuation (RMSF), root mean square distribution (RMSD), beta-factor, and the radius of the gyration factor (Figure 2). Throughout the 200 ns simulations, both studied compounds maintained their stability within the docked site of individual Ggp and Ggp in complex with Ephrin-B2; therefore, all four were contemplated for further exploration. The structural integrity of the protein-ligand complex was estimated by calculating the RMSD value of the backbone of the docked complex. The average RMSD value of docked complexes with native Ggp was found to be lower than their respective docked complexes with Ggp bounded to Ephrin-B2 receptors. The mean RMSD of the top compound (attached to unbounded Ggp) was 1.9 Å, while this value was 2.1 Å for its docked complex with Ggp in complex with Ephrin-B2, with a displacement of 0.25 Å. Similarly, the average RMSD value of the top 2 compound bounded to Ggp and Ggp previously complexed with Ephrin-B2 receptors was 1.7 Å and 2.5Å, respectively, displaying a displacement of 0.9 Å.

To estimate the residual flexibility of the backbone of unbounded compounds and docked complexes, the RMSF value was calculated. There were no major fluctuations in the assessed structures. The average RMSF value for the top 1 bounded with Ggp, top 1 docked complex with Ggp and Ephrin-B2, top 2 bounded with Ggp, and top 2 docked complex with Ggp and Ephrin-B2 was 3.8 Å, 3.3 Å, 2.3 Å, and 2.2 Å, respectively. Furthermore, Rg values were determined to estimate the compactness of the protein structure. The average Rg value for the top compound with Ggp was 23 Å^2^, which was 24 Å^2^ for its docked complex with Ggp already attached to Ephrin-B2. A similar trend was seen for the top 2 compound, where the mean Rg value for the compound with Ggp alone was 23. Å^2^; however, for Ggp attached to Ephrin-B2 in complex with the top 2 compound, this value was 24.8 Å^2^.

**Figure 3:**
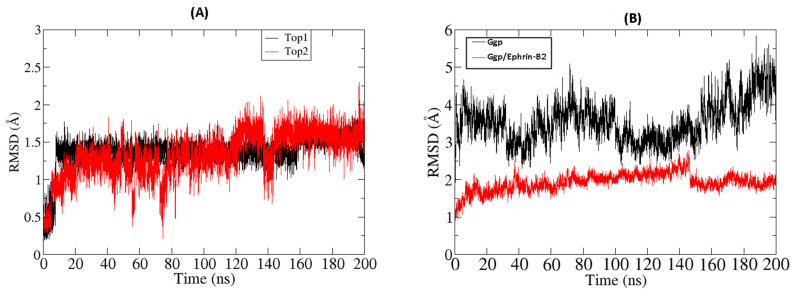
(**A**) RMSD of Top1 and Top2 inhibitors with a simulation time interval of 200 ns. (**B**) graphical representation of RMSD for single Ggp and in complex with Ephrin-B2.

To analyze the thermal disorders and additional residual flexibility, the beta factor was calculated. The mean values of the beta factor for the top 1 and top 2 compound, docked with Ggp, were 99 Å^2^ and 78 Å^2^, respectively, while the respective values were 81 Å^2^ and 75 Å^2^ for the receptor protein in complex with the Ephrin-B2 receptors. The stability of both inhibitors was inferred with a time frame of 200 ns. It was calculated that both inhibitors remained in pockets with no confirmatory changes and angular displacement. Thus, the average mean square deviation had a score of 1.3 Å for Top1 and 1.3 Å for Top2, respectively. Moreover, the RMSD for the single Ggp and Ggp in complex with Ephrin-B2 was calculated. The average root mean value for Ggp was 4.5 Å and 1.3 Å in complex with Ephrine-B2. In the meanwhile, average root mean square deviation of both inhibitors have been obtained, results shows a high stability with average RMSD of 1.5 Å for Top1 and 1.6 Å for Top2 inhibitor throughout simulations. Whereas, average RMSD calculated against single Ggp protein results in 4.8 Å, while in complex with Ephrin-B2 it has an average RMSD of 1.8 Å which make them stable along 200 ns time interval as shown in (Figure 3).

**Figure 4:**
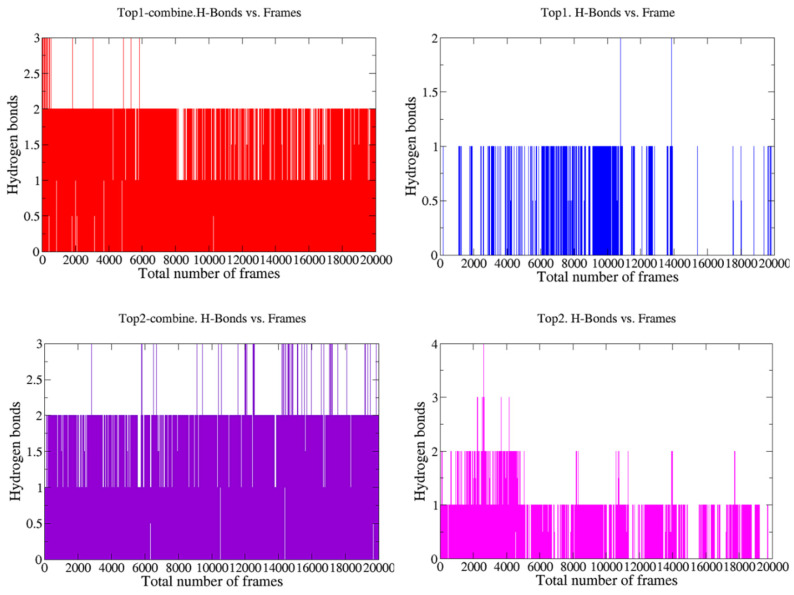
Hydrogen bond analysis of simulated dock complexes at different time intervals. Top1 and Top2 represent the hydrogen bond assessment of the top 1 and top 2 compounds bonded with the Ggp of HEV, respectively, while Top1-combine and Top2-combine show the hydrogen bond examination of the top 1 and top 2 compounds bonded with the Ggp of HEV attached to Ephrin-B2 receptors.

The stability and strength of hydrogen bonds, which were previously represented in Figure 1, were also analyzed during MD simulations, as their stability is directly associated with the flexibility and overall stability of the entire protein globular structure. Hydrogen bonding within the docked complex was assessed at two different time intervals (1 ns and 200 ns) to check whether the bonds present at the start of the simulations were sustained until the end of the 200 ns time interval. For the docked complex of the top compound with Ggp+ Ephrin-B2, two faces of hydrogen bonds were found at the receiver domain, on the amino acid residues of Tyr 255 (HH) and Lys 357 (HZ3). Similarly, the top 2 compound also showed two faces of H-bonds on the same residues of the receiver domain, i.e., Tyr 255 (HH) and Lys 357 (HZ1). To assess the different changes in the docked complexes, protein structures were downloaded at different time intervals. Frames for the whole systems at different intervals were evaluated for the movement of inhibitors at the binding site of the protein receptor along with the RMSD trajectories at the respective intervals. During the entire period of 200 ns, both hydrogen bonds in both of the studied top hits were found to be stable in nature, as shown by a stable environment throughout the analyzed trajectories at different time intervals (Figure 4). The top compound bonded with Ggp alone, demonstrating only one stable face of hydrogen bonds at the receiver domain, on the amino acid residue of Tyr 255 (HH). The top 2 compound showed three different hydrogen bonds with amino acids Tyr 255 (HH), Tyr 305 (H), and Thr 304 (HG1), with hydrogen bonds at Tyr 255 (HH) and Thr 304 (HG1) remaining constant for 200 ns whilst Tyr 305 (H) remained stable only for 100 ns.

#### 3.2.1. Radial Distribution Function (RDF)

**Figure 5:**
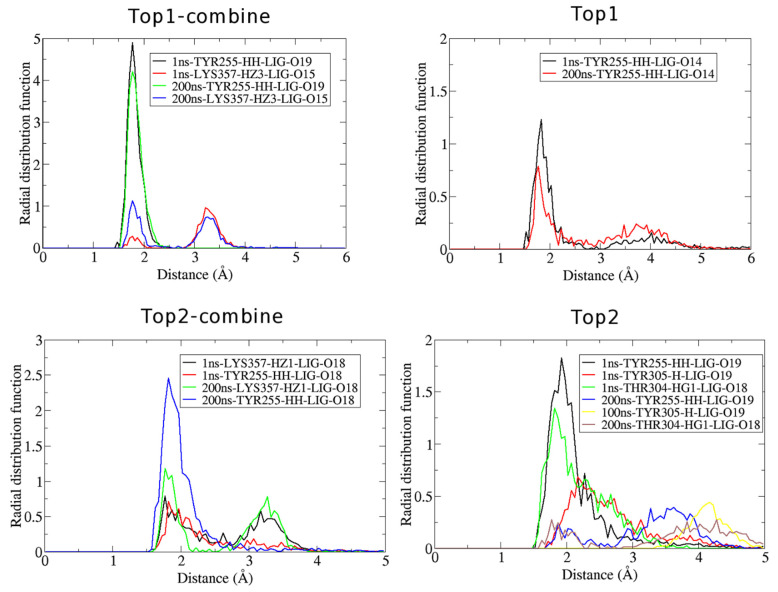
The radial distribution function of the docked complexes at 1 ns and 200 ns. Top1 depicts the RDF of the top 1 compound docked with Ggp, Top 2 represents the RDF of the top 2 compound docked with Ggp, Top1-combine shows the RDF of the docked complex of the top 1 compound with Ggp attached to Ephrin-B2 receptors and Top2-combine displays the RDF of the docked complex of the top 2 compound with Ggp attached to Ephrin-B2 receptors.

RDF analysis was performed to elucidate the distribution of atoms around the binding sites of the target protein. An in-house script in VMD was employed to point out the important residues of the target protein that can play an essential role in establishing hydrogen bonds with the ligand throughout the MD simulations. The RDF graphs were generated at both 1 ns and 200 ns to confirm whether the residues remained active throughout the simulations. Figure 5 depicts that in the complex of the top 1 inhibitor and Ggp+ Ephrin-B2, Tyr 255 and Lys 357 were the most vital residues of Ggp in forming the hydrogen bonds. Tyr 255 had an RDF value far higher than that of Lys 357 at both times, with an RDF value of 4.9 at 1 ns and 4.1 at 200 ns. This distribution was observed between the HH atom of Tyr 255 and the 19th oxygen of the top 1 compound, at a distance of 1.75 Å. The RDF plot of the Ggp+ Ephrin-B2 protein in complex with the top 2 compound represented that same residue involved in forming bonds with the ligand, i.e., Lys 357 and Tyr 255. However, at this time, Tyr 255 had a very high RDF value (2.5) at 200 ns compared to 1 ns (0.75). Distribution due to Tyr 255 was due to its HH atom and the 18th oxygen atom of the top 2 compound. Lys357 showed relatively different behavior; two different RDFs were seen at two different distances in the 1 ns and 200 ns simulations. During the simulation of 1 ns, an RDF of 0.75 was seen at a 1.75 Å distance followed by many fluctuating peaks with the last major peak at a 3.2 Å distance and a 0.6 RDF value. Similarly, in the 200 ns time frame, the first peak was visualized at a distance of 1.75 Å (RDF = 1.2) with an immediate fluctuation at 1.85 Å (1.1). Additionally, one more major peak was found at 3.3 Å (RDF = 0.75) followed by another instant peak at 3.45 Å (RDF = 0.5). All of these distributions were observed between the HZ1 residue of protein and the 18th oxygen of the ligand. For the docked complex of the top 1 compound and Ggp alone, only Tyr255 was found to be involved in hydrogen bonding with the inhibitor. This hydrogen bond represented an RDF value of 1.25 at 1 ns and 0.8 at 200 ns at a distance of around 0.8 Å and 0.77 Å, respectively. The HH atom of Tyr 255 and the 14th oxygen of the top 1 hit were reported to be responsible for this distribution. For the docked complex of the top 2 compound and Ggp protein alone, three different residues of Ggp were assessed to be responsible for hydrogen bonds, i.e., Tyr255, Thr304, and Tyr305. At 1 ns the RDF for these residues was 1.85 (2 Å), 1.3 (1.9 Å), and 0.65 (2.1 Å) respectively. However, there were so many fluctuations until 200 ns as seen in (Figure 5). For Tyr255, HH atoms and the 19th oxygen of the top 2 hit formed steady hydrogen bonds, and the same oxygen atom was identified to form hydrogen bonds with the H atom of tyr305. For Thr 304, the HG1 atom exhibited hydrogen bonding with the 18th oxygen atom of the ligand. Good stability was seen in all of these bonds at 200 ns with an RDF value of 0.85 (4.1 Å) for Tyr255 and 0.25 (at both 1.4 Å and 4.15 Å distance) for Thr304. For Tyr305, the RDF value was found to be 0.35 at 4.1 Å; however, this value was calculated at 100 ns as after that, this bond did not remain stable.

#### 3.2.2. Binding Free Energy Calculation

The binding free energies of both the top 1 and top 2 inhibitors in the active sites of Ggp and Ggp+ Ephrin-B2 are shown in Table 3. The free energy in the gas phase was the most favorable energy in both GB and PB, which was found in the entire system of all four cases, i.e., top 1-alone Ggp, top 2-alone Ggp, top 1-combined Ggp (glycoprotein in complex with human cell surface Ephrin-B2) top 2-combined Ggp with the total input of −20.3134 kcal/mol, −62.4830 kcal/mol, −204.43 kcal/mol, and −104.8816 kcal/mol, respectively. Van der Waals’ energy also added a large amount of energy to the docked complexes of the top hits with Ggp alone, accounting for −19.2174 kcal/mol for top 1-alone Ggp and −35.5087 kcal/mol for top 2-alone Ggp. Contrary to this, electrostatic energy imparted a second major contribution to the binding free energies of the top hits with a combined Ggp structure. Electrostatic energy accounted for −157.7020 kcal/mol and −60.6010 kcal/mol energy for the top 1-combined Ggp and Top2-combined Ggp complexes, respectively. For the Top1-combined Ggp (glycoprotein in complex with human cell surface Ephrin-B2) docked complex, the difference in values owing to the solvation energy was 168.2240 kcal/mol for MMGBSA and 173.7503 kcal/mol for MMPBSA, while this value was 67.5825 kcal/mol for MMGBSA and 76.1380 kcal/mol for MMPBSA for the Top2-combined Ggp (glycoprotein in complex with human cell surface Ephrin-B2) docked complex. For the Top1-Ggp complex, the difference in the solvation energy was 11.5228 kcal/mol for MMGBSA and 9.3210 kcal/mol for MMPBSA, whereas this value was 40.0763 kcal/mol for MMGBSA and 49.5926 kcal/mol for MMPBSA for the Top2-combined Ggp docked complex. Amino acids with a binding energy lower than 0 kcal/mol are considered hot-spot residues as they dominate other amino acids to maintain the binding free energy of the overall structure. Hot-spot amino acids in the docked complex of top 1-alone Ggp in GB include Ile 109 (−1) and Ala 253 (−1), whereas Ile 109 (−0.9) was the only residue reported to be a hot spot in PB. Likewise, residues in the top 2- alone Ggp docked complex for GB were Ile 109 (−1.7), Gly 155 (−1.3), Ile 235(−1), and Gly 303 (−1.1), and for PB, these residues were Ile 109 (−1.5), Lys 157 (−0.8), and Asp 307 (−1). As compared to this, hot-spot amino acids in the Top1-combined Ggp complex were ILE 375 (−1.4) and Leu 487 (−1.3) in PB but GLN 240 (−1), ILE 298 (−2), ARG 300 (−1.9), and PHE 353 (−3.75) in GB, whereas, for the Top2-combined Ggp structure, PRO 301 (−0.9), PHE 350 (−4.1) residues were reported to be hot spots in PB, and GLN 240 (−1.2), PRO 301 (−5.9), VAL 352 (−1.9), and Leu 487 (−1.7) were the hot-spot amino acids in GB.

### 3.3. WaterSwap Analysis

Water is considered an important solvent as its molecules serve as the interactive bridges between the protein and its inhibitor (ligand). Therefore, in the current experimental work, the explicit water model of WaterSwap was used to investigate the molecular details of ligand–water, protein–water, and protein interactions. Table 4 represents the free binding energy calculated using four different algorithms in WaterSwap analysis. The results show that the binding potential of all studied complexes was lower than 1 kcal/mol, which is acceptable as the standard value is <1 kcal/mol.

## 4. Discussion

HeV is one of the deadliest known viruses for humans with no approved therapeutic substance; therefore, we conducted this research work to propose lead compounds that could be used as future anti-HeV drugs. For this purpose, extensive CADD-based computational methods were adopted.

Firstly, the database of 6000 anti-viral compounds was screened for pharamcokinetics-based parameters, as used by many researchers in previously published studies [48,49,50]. Compounds having acceptable drug-like properties were considered for molecular docking analysis against Ggp of HeV through Autodock Vina software. This software has been used extensively to perform molecular docking analysis to propose potent inhibitors of deadly viruses [51,52,53]. The docking study revealed that compounds (*S*)-5-(benzylcarbamoyl)-1-(2-(4-methyl-2-phenylpiperazin-1-yl)-2-oxoethyl)-6-oxo-3,6-dihydropyridin-1-ium-3-ide and 5-(cyclohexylcarbamoyl)-1-(2-((2-(3-fluorophenyl)-2-methylpropyl)amino)-2-oxoethyl)-6-oxo-3,6-dihydropyridin-1-ium-3-ide had the maximum binding potential with the active residues of Ggp in both unbounded and bounded form. Hydrogen bonds formed by them not only assist in filtering the unrealistic poses in docking but also help in precise calculation of the binding energy [54]. Developed hydrogen bonds were found to be stable in MD simulation studies, which are considered an effective methodology to validate the results of molecular docking analysis, as shown in many previous studies [55,56,57]. Furthermore, the stability of the entire system was confirmed through RDF analysis. MMGBSA/MMPBSA was employed to evaluate the binding free energies, which in turn explains the strength of binding affinities between inhibitors and proteins [58]. The MMGBSA/MMPBSA method is well demonstrated in binding free estimation for antiviral inhibitors [38,59,60]. Data from this step indicated that the bounded ligand will not be easily displaced by the solvents and hence, the stability of docked complex will be sustained [59].

A major limitation in MMGBSA and MMPBSA is the creation of cavitation and great differences during double decoupling. To overcome this shortcoming, WaterSwap analysis was conducted to elucidate the stability of the docked complex to measure the absolute values of the binding free energy [46]. WaterSwap assessment indicated that the binding affinity of the studied complexes was higher than the standardized value of <1 kcal/mol. Many scientists have used the same methodology to forecast potential therapeutic compounds against different viruses such as SARS-CoV-2 [60], Dengue virus [61], and Nipah virus [62]. Therefore, CADD-based drug discovery appears to be capable of combating pathogens including viruses.

To the best of our knowledge, this is the first study to suggest antiviral compounds as the potential drugs against HeV. Although a subunit vaccine is under clinical trials in human [63] and immunoinformatics-based silico vaccines have recently been proposed by Kamthania et al., and Hossan et. al. [64,65], no anti-HeV oral drug has been reported or proposed before. Ribavirin and Chloroquine are the two anti-viral drugs that have been tested for their anti-HeV activity. Chloroquine (an anti-malarial drug) acts by inhibiting the critical proteolytic processing of HeV, whereas Ribavirin inhibits the replication of HeV [1,4]. Both of these drugs only delay the HeV infection; they do not eradicate the chances of virus-mediated death [66]. In contrast to this, our proposed compounds have a strong potential to inhibit the G glycoprotein, which makes the attachment of the virus possible with human receptors. Therefore, the current study indicates huge potential for drug discovery against HeV.

## 5. Conclusions

HeV infection is a global health issue that results in many neurological disorders. Due to the unavailability of any approved therapeutic option, it is necessary to identify some therapeutic methods to combat this harmful viral infection. Because of the enormous advantages, computer-aided drug discovery was employed to propose the lead compound against HeV. Our study proposed that (*S*)-5-(benzylcarbamoyl)-1-(2-(4-methyl-2-phenylpiperazin-1-yl)-2-oxoethyl)-6-oxo-3,6-dihydropyridin-1-ium-3-ide and 5-(cyclohexylcarbamoyl)-1-(2-((2-(3-fluorophenyl)-2-methylpropyl)amino)-2-oxoethyl)-6-oxo-3,6-dihydropyridin-1-ium-3-ide are the lead compounds with maximum binding affinity with the active sites of the G glycoprotein of HeV, even if HeV is attached to its human cellular receptors, thereby, inhibiting its pathogenesis. Further wet laboratory investigations are necessary to authorize the safety and efficiency of these lead compounds against HeV.

## Figures and Tables

**Table 1:** Binding energy of the top five compounds in terms of the Autodoc Vina scores and their interactions with the G glycoprotein receptor of Hendra Virus.

Compound Name	Chemical Structure	AutoDock Vina Score	
		G Attachement Glycoprotein	G Attachement Glycoprotein Complex with Ephrin-B2 Receptors
**Top1 (C_24_H_30_FN_3_O_3_)**	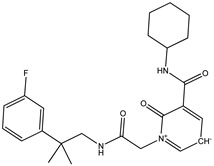	−9.7	−9.5
**Top2 (C_26_H_28_N_4_O_3_)**	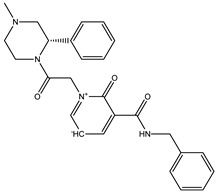	−9.4	−9.2
**Top3 (C_22_H_24_FN_5_O_3_)**	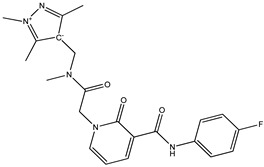	−8.6	−8.7
**Top4 (C_22_H_21_FN_4_O_3_)**	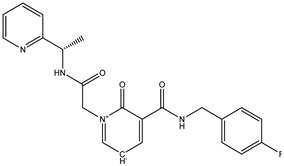	−8.2	−8.5
**Top5 (C_21_H_26_N_4_O_3_)**	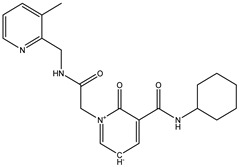	−7.9	−7.4

**Table 2:** Drug-likeliness and physiochemical properties of top 2 best docked compounds.

Compound ID	Physiochemical Properties					Drug-Likeliness (Violations for Different Rules)				Medicinal Chemistry		
	Molecular Weight (g/mol)	H-Bond Donor	H-Bond Acceptor	Topological Polar Surface Area (Å^2^)	Number of Rotatable Bonds	Lipinski Rule of 5	Veber Rule	Egan Rule	Muegge Rule	PAINS Alert	Brenk Alert	Synthetic Accessibility
**Top 1**	445.53	2	3	75.85	8	0	0	0	0	0	0	3.75
**Top 2**	427.51	2	4	80.20	9	0	0	0	0	0	0	3.29

**Table 3:** Binding energy of the different complexes; Top1 = bounded top 1 compound and Ggp, Top2 = bounded top 2 compound and Ggp, Top1-combine = docked complex of Top 1 compound and Ggp attached to the human receptor, Top2-combine = docked complex of top 2 compound and Ggp attached to the human receptor.

Components	Top1-Combine	Top1	Top2-Combine	Top2
	Energies (kcal/mol)			
**∆Evdw**	−46.7359	−19.2174	−44.2806	−35.5087
**∆Eele**	−157.7020	−1.0961	−60.6010	−26.9743
**∆EGB**	174.3174	13.9830	73.1834	44.9049
**∆Esurf**	−6.0934	−2.4602	−5.6009	−4.8286
**∆Ggas**	−204.4379	−20.3134	−104.8816	−62.4830
**∆Gsolv/GB**	168.2240	11.5228	67.5825	40.0763
**∆tot/GB**	−36.2139	−8.7906	−37.2991	−22.4067
**∆EPB**	178.3334	12.0337	80.8110	53.6368
**∆Gnpol**	−4.5831	−2.7127	−4.6730	−4.0441
**∆Gsolv/PB**	173.7503	9.3210	76.1380	49.5926
**∆tot/PB**	−30.6876	−10.9925	−28.7436	−12.8904
**∆tot/PB**	−30.6876	−10.9925	−28.7436	−12.8904

**Table 4:** Binding free energy calculation for the protein-inhibitor complex based on WaterSwap calculations; Top1 = Ggp with bounded top 1 inhibitor, Top2 = Ggp with bounded top 1 inhibitor, Top1-combine = complex of Ggp and human receptor in complex with the top 1 inhibitor, Top2-combine = complex of Ggp and human receptor in complex with the top 2 inhibitor.

Algorithm	Waterswap Energy (kcal/mol)			
	Top1-Combine	Top1	Top2-Combine	Top2
**Bennetts**	−27.69	−25.89	−35.53	−30.80
**FEP**	−24.75	−20.15	−33.23	−26.60
**TI**	−26.01	−23.05	−33.13	−30.20
**Quadrature**	−22.4	−21.4	−31.24	−23.6

## Data Availability

The data presented in this study are available within the article.

## Supplementary Materials

The following supporting information can be downloaded online. Figure S1: BOILED-Egg plot of the top two compounds with the best binding ability to the G glycoprotein of Hendra Virus, where molecule 1 and 2 code for the top 1 and top 2 compound in Table 1, respectively. Figure S2: Comparison of drug-like and non-drug-like properties of the top 1 (a) and top 2 (b) drug candidates. Table S1: Different parameters of ADMET properties upon which ADMETsar evaluates the pharmacokinetics properties of a compound. Normal properties are not highlighted while those that deviate from the normal range are highlighted in red.
