# Supplementary material for: Discovery of Potential Antiviral Compounds against Hendra Virus by Targeting Its Receptor-Binding Protein (G) Using Computational Approaches
Source: Molecules. 2022 Jan 16;27(2):554. doi: 10.3390/molecules27020554 (PMC8779602; doi:10.3390/molecules27020554)
Supplement: Supplementary file 1 [file molecules-27-00554-s001.zip › molecules-1485629-supplementary.pdf]

Supplementary Materials

Discovery of Potential Antiviral Compounds against Hendra Virus by Targeting Its Receptor–Binding Protein (G) Using Computational Approaches

Faisal Ahmad [1,†], Aqel Albutti [2,*,†], Muhammad Hamza Tariq [3,†], Ghufranud Din [4], Muhammad Tahir ul Qamar [5] and Sajjad Ahmad [6,*]

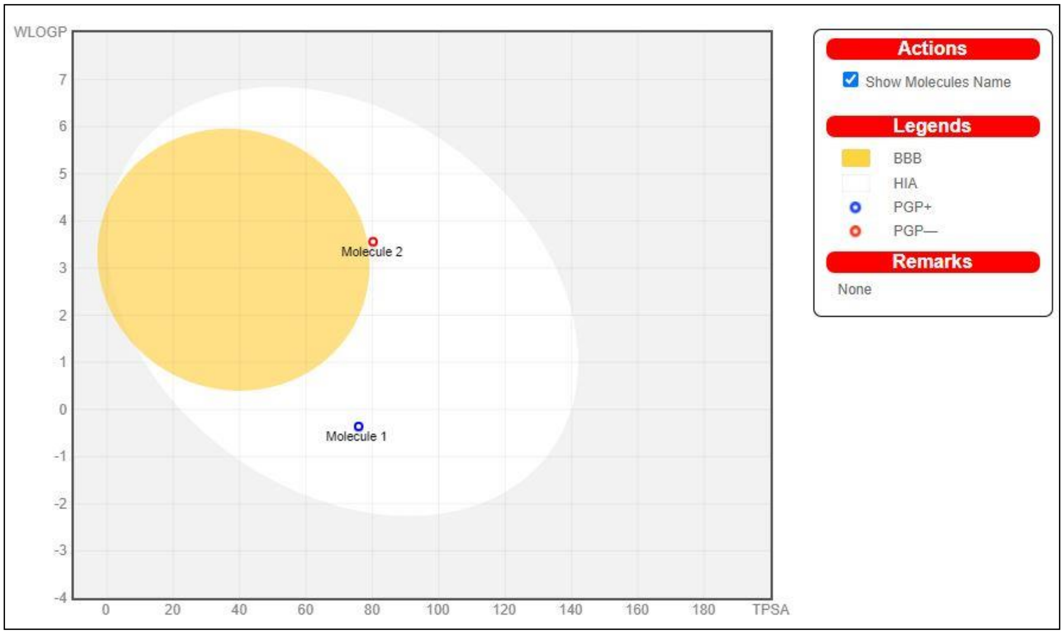

Figure S1. BOILED–Egg plot of top two compounds, having best binding capability to G Glycoprotein of Hendra Virus, where molecule 1 and 2 codes for Top 1 and Top 2 compound in Table 1 respectively.

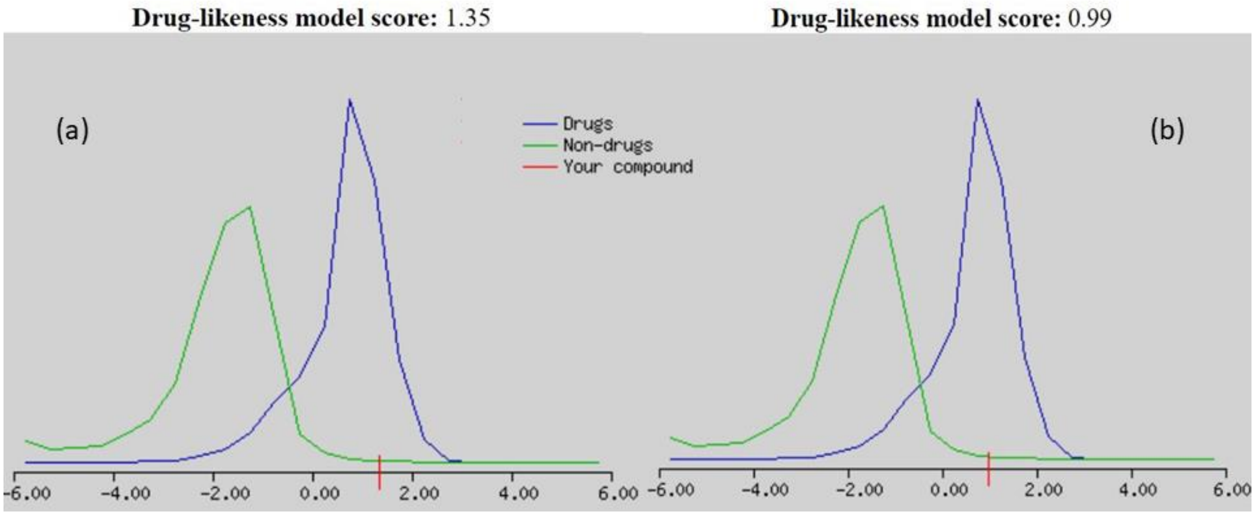

Figure S2. Comparison of drug–like and non–drug–like properties of top 1 (**a**) and top 2 (**b**) drug candidates.

Table S1. Different parameters of ADMET properties upon which ADMETsar evaluates pharmacokinetics properties of a compound. Normal properties are not highlighted while those which are deviating from the normal range are highlighted red.

Models	Ideal Case	Top 1	Top2
**Absorption**			
**Blood–Brain Barrier**	–ive	–ive	–ive
**Human Intestinal Absorption**	+ive	+ive	+ive
**Human Oral Bioavailability**	+ive	+ive	+ive
**Caco–2 Permeability**	+ive	+ive	+ive
**P–glycoprotein substrate**	–ive	–ive	–ive
**Metabolism**			
**Renal Organic Cation Transporter**	Non–Inhibitor	Non–Inhibitor	Non–Inhibitor
**CYP450 2C9 Substrate**	+ive	–ive	+ive
**CYP450 2D6 Substrate**	+ive	+ive	+ive
**CYP450 3A4 Substrate**	+ive	+ive	+ive
**CYP450 1A2 Inhibitor**	–ive	–ive	–ive
**CYP450 2C9 Inhibitor**	–ive	–ive	+ive

**CYP450 2D6 Inhibitor**	−ive	+ive	−ive
**CYP450 2C19 Inhibitor**	−ive	−ive	−ive
**CYP450 3A4 Inhibitor**	−ive	−ive	−ive
**CYP Inhibitor Promiscuity**	−ive	−ive	−ive
**Toxicity**			
**AMES Toxicity**	−ive	−ive	−ive
**Carcinogens**	−ive	−ive	−ive
**Human Ether−a−go−go−Related Gene Inhibition**	−ive	−ive	−ive
**AMES mutagenesis**	−ive	−ive	−ive
**Eye Irritation**	−ive	−ive	−ive
**Eye Corrosion**	−ive	−ive	+ive
**hepatotoxicity**	−ive	−ive	−ive
**Estrogen receptor binding**	−ive	−ive	−ive
**Androgen receptor binding**	−ive	−ive	−ive
**Thyroid receptor binding**	−ive	+ive	−ive
**Glucocorticoid receptor binding**	−ive	−ive	+ive
**Aromatase binding**	−ive	+ive	+ive
**Rat Acute Toxicity (mol/kg)**	−ive	−ive	−ive
**Total Violations**	00	04	04

Top1−coordinates

HEADER   CSD ENTRY

CRYST1   1.0000   1.0000   1.0000 90.00 90.00 90.00

SCALE1   1.000000 0.000000 0.000000   0.000000

SCALE2   0.000000 1.000000 0.000000   0.000000

SCALE3   0.000000 0.000000 1.000000   0.000000

ATOM   1 N   ILE A   1   32.653 −16.268 −1.319 1.00 0.00   N

ATOM	2	CA	ILE	A	1	32.633	−15.941	−2.763	1.00	0.00	C
ATOM	3	C	ILE	A	1	32.837	−14.441	−2.909	1.00	0.00	C
ATOM	4	O	ILE	A	1	32.290	−13.705	−2.093	1.00	0.00	O
ATOM	5	CB	ILE	A	1	31.347	−16.438	−3.468	1.00	0.00	C
ATOM	6	CG1	ILE	A	1	31.341	−16.009	−4.953	1.00	0.00	C
ATOM	7	CG2	ILE	A	1	30.059	−15.978	−2.757	1.00	0.00	C
ATOM	8	CD1	ILE	A	1	30.325	−16.768	−5.817	1.00	0.00	C
ATOM	9	HA	ILE	A	1	33.476	−16.435	−3.236	1.00	0.00	H
ATOM	10	HB	ILE	A	1	31.370	−17.529	−3.437	1.00	0.00	H
ATOM	11	HG12	ILE	A	1	31.130	−14.941	−5.030	1.00	0.00	H
ATOM	12	HG13	ILE	A	1	32.330	−16.191	−5.375	1.00	0.00	H
ATOM	13	HD11	ILE	A	1	30.420	−16.435	−6.849	1.00	0.00	H
ATOM	14	HD12	ILE	A	1	30.514	−17.841	−5.770	1.00	0.00	H
ATOM	15	HD13	ILE	A	1	29.306	−16.561	−5.490	1.00	0.00	H
ATOM	16	H1	ILE	A	1	32.449	−17.244	−1.163	1.00	0.00	H
ATOM	17	H2	ILE	A	1	31.970	−15.687	−0.848	1.00	0.00	H
ATOM	18	H3	ILE	A	1	33.568	−16.041	−0.940	1.00	0.00	H
ATOM	19	HG21	ILE	A	1	29.185	−16.396	−3.259	1.00	0.00	H
ATOM	20	HG22	ILE	A	1	29.968	−14.891	−2.784	1.00	0.00	H
ATOM	21	HG23	ILE	A	1	30.021	−16.320	−1.724	1.00	0.00	H
ATOM	22	N	CYS	A	2	33.675	−13.977	−3.840	1.00	0.00	N
ATOM	23	CA	CYS	A	2	33.809	−12.535	−4.055	1.00	0.00	C
ATOM	24	C	CYS	A	2	32.574	−11.948	−4.761	1.00	0.00	C
ATOM	25	O	CYS	A	2	32.343	−12.226	−5.937	1.00	0.00	O
ATOM	26	CB	CYS	A	2	35.092	−12.191	−4.815	1.00	0.00	C
ATOM	27	SG	CYS	A	2	35.277	−10.404	−5.017	1.00	0.00	S
ATOM	28	H	CYS	A	2	34.138	−14.619	−4.473	1.00	0.00	H
ATOM	29	HA	CYS	A	2	33.888	−12.061	−3.076	1.00	0.00	H
ATOM	30	HB2	CYS	A	2	35.060	−12.645	−5.806	1.00	0.00	H
ATOM	31	HB3	CYS	A	2	35.956	−12.576	−4.276	1.00	0.00	H

ATOM	32	N	LEU	A	3	31.841	−11.084	−4.056	1.00	0.00	N
ATOM	33	CA	LEU	A	3	30.699	−10.317	−4.576	1.00	0.00	C
ATOM	34	C	LEU	A	3	31.032	−8.834	−4.838	1.00	0.00	C
ATOM	35	O	LEU	A	3	30.234	−8.131	−5.451	1.00	0.00	O
ATOM	36	CB	LEU	A	3	29.536	−10.434	−3.572	1.00	0.00	C
ATOM	37	CG	LEU	A	3	29.029	−11.868	−3.324	1.00	0.00	C
ATOM	38	CD1	LEU	A	3	28.068	−11.866	−2.138	1.00	0.00	C
ATOM	39	CD2	LEU	A	3	28.323	−12.447	−4.551	1.00	0.00	C
ATOM	40	H	LEU	A	3	32.062	−10.971	−3.078	1.00	0.00	H
ATOM	41	HA	LEU	A	3	30.378	−10.733	−5.533	1.00	0.00	H
ATOM	42	HB2	LEU	A	3	29.865	−10.009	−2.622	1.00	0.00	H
ATOM	43	HB3	LEU	A	3	28.699	−9.829	−3.927	1.00	0.00	H
ATOM	44	HG	LEU	A	3	29.866	−12.517	−3.068	1.00	0.00	H
ATOM	45	HD11	LEU	A	3	27.641	−12.859	−1.999	1.00	0.00	H
ATOM	46	HD12	LEU	A	3	27.266	−11.150	−2.317	1.00	0.00	H
ATOM	47	HD13	LEU	A	3	28.600	−11.576	−1.232	1.00	0.00	H
ATOM	48	HD21	LEU	A	3	29.023	−12.547	−5.379	1.00	0.00	H
ATOM	49	HD22	LEU	A	3	27.501	−11.795	−4.847	1.00	0.00	H
ATOM	50	HD23	LEU	A	3	27.917	−13.432	−4.318	1.00	0.00	H
ATOM	51	N	GLN	A	4	32.174	−8.356	−4.332	1.00	0.00	N
ATOM	52	CA	GLN	A	4	32.625	−6.966	−4.438	1.00	0.00	C
ATOM	53	C	GLN	A	4	33.318	−6.704	−5.783	1.00	0.00	C
ATOM	54	O	GLN	A	4	34.098	−7.532	−6.258	1.00	0.00	O
ATOM	55	CB	GLN	A	4	33.568	−6.659	−3.256	1.00	0.00	C
ATOM	56	CG	GLN	A	4	33.907	−5.174	−3.041	1.00	0.00	C
ATOM	57	CD	GLN	A	4	32.715	−4.319	−2.625	1.00	0.00	C
ATOM	58	OE1	GLN	A	4	31.658	−4.797	−2.257	1.00	0.00	O
ATOM	59	NE2	GLN	A	4	32.833	−3.013	−2.682	1.00	0.00	N
ATOM	60	H	GLN	A	4	32.809	−9.008	−3.905	1.00	0.00	H
ATOM	61	HA	GLN	A	4	31.749	−6.318	−4.366	1.00	0.00	H

ATOM 62 HB2 GLN A 4 33.132 −7.049 −2.334 1.00 0.00 H

ATOM 63 HB3 GLN A 4 34.509 −7.178 −3.425 1.00 0.00 H

ATOM 64 HG2 GLN A 4 34.659 −5.101 −2.255 1.00 0.00 H

ATOM 65 HG3 GLN A 4 34.345 −4.761 −3.945 1.00 0.00 H

ATOM 66 HE21 GLN A 4 33.685 −2.587 −2.999 1.00 0.00 H

ATOM 67 HE22 GLN A 4 32.019 −2.489 −2.414 1.00 0.00 H

ATOM 68 N LYS A 5 33.179 −5.485 −6.311 1.00 0.00 N

ATOM 69 CA LYS A 5 33.993 −4.990 −7.427 1.00 0.00 C

ATOM 70 C LYS A 5 35.463 −4.805 −7.021 1.00 0.00 C

ATOM 71 O LYS A 5 35.744 −4.157 −6.016 1.00 0.00 O

ATOM 72 CB LYS A 5 33.343 −3.703 −7.956 1.00 0.00 C

ATOM 73 CG LYS A 5 34.110 −3.120 −9.144 1.00 0.00 C

ATOM 74 CD LYS A 5 33.252 −2.138 −9.952 1.00 0.00 C

ATOM 75 CE LYS A 5 34.025 −1.589 −11.158 1.00 0.00 C

ATOM 76 NZ LYS A 5 34.494 −2.678 −12.052 1.00 0.00 N

ATOM 77 H LYS A 5 32.506 −4.854 −5.897 1.00 0.00 H

ATOM 78 HA LYS A 5 33.964 −5.741 −8.213 1.00 0.00 H

ATOM 79 HB2 LYS A 5 32.329 −3.946 −8.275 1.00 0.00 H

ATOM 80 HB3 LYS A 5 33.285 −2.956 −7.162 1.00 0.00 H

ATOM 81 HG2 LYS A 5 35.010 −2.616 −8.788 1.00 0.00 H

ATOM 82 HG3 LYS A 5 34.402 −3.937 −9.795 1.00 0.00 H

ATOM 83 HD2 LYS A 5 32.350 −2.647 −10.299 1.00 0.00 H

ATOM 84 HD3 LYS A 5 32.950 −1.310 −9.308 1.00 0.00 H

ATOM 85 HE2 LYS A 5 34.877 −1.007 −10.794 1.00 0.00 H

ATOM 86 HE3 LYS A 5 33.363 −0.913 −11.708 1.00 0.00 H

ATOM 87 HZ1 LYS A 5 34.932 −2.323 −12.889 1.00 0.00 H

ATOM 88 HZ2 LYS A 5 35.137 −3.292 −11.566 1.00 0.00 H

ATOM 89 HZ3 LYS A 5 33.708 −3.272 −12.317 1.00 0.00 H

ATOM 90 N THR A 6 36.403 −5.279 −7.844 1.00 0.00 N

ATOM 91 CA THR A 6 37.856 −5.126 −7.630 1.00 0.00 C

ATOM 92 C THR A 6 38.647 −5.237 −8.944 1.00 0.00 C

ATOM 93 O THR A 6 38.111 −5.652 −9.971 1.00 0.00 O

ATOM 94 CB THR A 6 38.376 −6.122 −6.569 1.00 0.00 C

ATOM 95 OG1 THR A 6 39.739 −5.865 −6.316 1.00 0.00 O

ATOM 96 CG2 THR A 6 38.270 −7.592 −6.974 1.00 0.00 C

ATOM 97 H THR A 6 36.108 −5.742 −8.700 1.00 0.00 H

ATOM 98 HA THR A 6 38.038 −4.126 −7.236 1.00 0.00 H

ATOM 99 HB THR A 6 37.819 −5.975 −5.643 1.00 0.00 H

ATOM 100 HG1 THR A 6 40.069 −6.475 −5.641 1.00 0.00 H

ATOM 101 HG21 THR A 6 38.657 −8.210 −6.165 1.00 0.00 H

ATOM 102 HG22 THR A 6 37.224 −7.854 −7.143 1.00 0.00 H

ATOM 103 HG23 THR A 6 38.843 −7.785 −7.881 1.00 0.00 H

ATOM 104 N THR A 7 39.919 −4.835 −8.924 1.00 0.00 N

ATOM 105 CA THR A 7 40.904 −5.065 −9.996 1.00 0.00 C

ATOM 106 C THR A 7 41.967 −6.108 −9.622 1.00 0.00 C

ATOM 107 O THR A 7 42.910 −6.306 −10.383 1.00 0.00 O

ATOM 108 CB THR A 7 41.566 −3.750 −10.439 1.00 0.00 C

ATOM 109 OG1 THR A 7 42.236 −3.148 −9.357 1.00 0.00 O

ATOM 110 CG2 THR A 7 40.544 −2.746 −10.975 1.00 0.00 C

ATOM 111 H THR A 7 40.283 −4.513 −8.035 1.00 0.00 H

ATOM 112 HA THR A 7 40.392 −5.474 −10.866 1.00 0.00 H

ATOM 113 HB THR A 7 42.285 −3.957 −11.233 1.00 0.00 H

ATOM 114 HG21 THR A 7 41.069 −1.869 −11.353 1.00 0.00 H

ATOM 115 HG22 THR A 7 39.862 −2.438 −10.183 1.00 0.00 H

ATOM 116 HG23 THR A 7 39.978 −3.200 −11.789 1.00 0.00 H

ATOM 117 HG1 THR A 7 43.081 −3.597 −9.254 1.00 0.00 H

ATOM 118 N SER A 8 41.831 −6.792 −8.479 1.00 0.00 N

ATOM 119 CA SER A 8 42.648 −7.967 −8.139 1.00 0.00 C

ATOM 120 C SER A 8 42.492 −9.069 −9.196 1.00 0.00 C

ATOM 121 O SER A 8 41.383 −9.338 −9.659 1.00 0.00 O

ATOM 122 CB SER A 8 42.242 −8.541 −6.780 1.00 0.00 C
ATOM 123 OG SER A 8 42.255 −7.570 −5.754 1.00 0.00 O
ATOM 124 H SER A 8 41.087 −6.545 −7.842 1.00 0.00 H
ATOM 125 HA SER A 8 43.694 −7.668 −8.092 1.00 0.00 H
ATOM 126 HB2 SER A 8 41.236 −8.955 −6.849 1.00 0.00 H
ATOM 127 HB3 SER A 8 42.938 −9.339 −6.520 1.00 0.00 H
ATOM 128 HG SER A 8 42.544 −8.039 −4.942 1.00 0.00 H
ATOM 129 N THR A 9 43.545 −9.851 −9.436 1.00 0.00 N
ATOM 130 CA THR A 9 43.583 −10.945 −10.433 1.00 0.00 C
ATOM 131 C THR A 9 42.946 −12.254 −9.931 1.00 0.00 C
ATOM 132 O THR A 9 43.398 −13.354 −10.246 1.00 0.00 O
ATOM 133 CB THR A 9 45.011 −11.166 −10.966 1.00 0.00 C
ATOM 134 OG1 THR A 9 45.889 −11.607 −9.943 1.00 0.00 O
ATOM 135 CG2 THR A 9 45.595 −9.902 −11.599 1.00 0.00 C
ATOM 136 H THR A 9 44.359 −9.740 −8.843 1.00 0.00 H
ATOM 137 HA THR A 9 42.977 −10.631 −11.285 1.00 0.00 H
ATOM 138 HB THR A 9 44.970 −11.928 −11.748 1.00 0.00 H
ATOM 139 HG21 THR A 9 46.543 −10.143 −12.081 1.00 0.00 H
ATOM 140 HG22 THR A 9 45.764 −9.131 −10.848 1.00 0.00 H
ATOM 141 HG23 THR A 9 44.910 −9.517 −12.355 1.00 0.00 H
ATOM 142 HG1 THR A 9 45.581 −11.264 −9.081 1.00 0.00 H
ATOM 143 N ILE A 10 41.898 −12.159 −9.110 1.00 0.00 N
ATOM 144 CA ILE A 10 41.276 −13.273 −8.367 1.00 0.00 C
ATOM 145 C ILE A 10 40.673 −14.378 −9.256 1.00 0.00 C
ATOM 146 O ILE A 10 40.501 −15.519 −8.812 1.00 0.00 O
ATOM 147 CB ILE A 10 40.227 −12.716 −7.379 1.00 0.00 C
ATOM 148 CG1 ILE A 10 39.160 −11.831 −8.065 1.00 0.00 C
ATOM 149 CG2 ILE A 10 40.916 −12.004 −6.207 1.00 0.00 C
ATOM 150 CD1 ILE A 10 38.103 −11.279 −7.101 1.00 0.00 C
ATOM 151 H ILE A 10 41.584 −11.220 −8.901 1.00 0.00 H

ATOM	152	HA	ILE	A	10	42.056	−13.761	−7.783	1.00	0.00	H
ATOM	153	HB	ILE	A	10	39.721	−13.573	−6.948	1.00	0.00	H
ATOM	154	HG12	ILE	A	10	39.630	−10.980	−8.555	1.00	0.00	H
ATOM	155	HG13	ILE	A	10	38.654	−12.417	−8.832	1.00	0.00	H
ATOM	156	HG21	ILE	A	10	41.531	−11.184	−6.567	1.00	0.00	H
ATOM	157	HG22	ILE	A	10	41.560	−12.711	−5.682	1.00	0.00	H
ATOM	158	HG23	ILE	A	10	40.194	−11.632	−5.487	1.00	0.00	H
ATOM	159	HD11	ILE	A	10	37.733	−12.067	−6.449	1.00	0.00	H
ATOM	160	HD12	ILE	A	10	37.276	−10.863	−7.670	1.00	0.00	H
ATOM	161	HD13	ILE	A	10	38.533	−10.487	−6.492	1.00	0.00	H
ATOM	162	N	LEU	A	11	40.408	−14.076	−10.529	1.00	0.00	N
ATOM	163	CA	LEU	A	11	40.169	−15.065	−11.579	1.00	0.00	C
ATOM	164	C	LEU	A	11	41.513	−15.603	−12.095	1.00	0.00	C
ATOM	165	O	LEU	A	11	42.148	−14.989	−12.945	1.00	0.00	O
ATOM	166	CB	LEU	A	11	39.368	−14.411	−12.718	1.00	0.00	C
ATOM	167	CG	LEU	A	11	37.929	−14.021	−12.357	1.00	0.00	C
ATOM	168	CD1	LEU	A	11	37.406	−13.062	−13.424	1.00	0.00	C
ATOM	169	CD2	LEU	A	11	37.013	−15.243	−12.273	1.00	0.00	C
ATOM	170	H	LEU	A	11	40.670	−13.148	−10.830	1.00	0.00	H
ATOM	171	HA	LEU	A	11	39.597	−15.901	−11.178	1.00	0.00	H
ATOM	172	HB2	LEU	A	11	39.904	−13.514	−13.031	1.00	0.00	H
ATOM	173	HB3	LEU	A	11	39.339	−15.094	−13.567	1.00	0.00	H
ATOM	174	HG	LEU	A	11	37.916	−13.511	−11.394	1.00	0.00	H
ATOM	175	HD21	LEU	A	11	37.370	−15.933	−11.511	1.00	0.00	H
ATOM	176	HD22	LEU	A	11	36.005	−14.927	−12.014	1.00	0.00	H
ATOM	177	HD23	LEU	A	11	36.986	−15.747	−13.238	1.00	0.00	H
ATOM	178	HD11	LEU	A	11	37.965	−12.130	−13.364	1.00	0.00	H
ATOM	179	HD12	LEU	A	11	37.551	−13.487	−14.415	1.00	0.00	H
ATOM	180	HD13	LEU	A	11	36.350	−12.847	−13.270	1.00	0.00	H
ATOM	181	N	LYS	A	12	41.922	−16.783	−11.613	1.00	0.00	N

ATOM	182	CA	LYS	A	12	43.185	−17.445	−11.998	1.00	0.00	C
ATOM	183	C	LYS	A	12	42.898	−18.715	−12.827	1.00	0.00	C
ATOM	184	O	LYS	A	12	42.657	−19.768	−12.220	1.00	0.00	O
ATOM	185	CB	LYS	A	12	44.061	−17.686	−10.749	1.00	0.00	C
ATOM	186	CG	LYS	A	12	44.321	−16.380	−9.971	1.00	0.00	C
ATOM	187	CD	LYS	A	12	45.404	−16.508	−8.886	1.00	0.00	C
ATOM	188	CE	LYS	A	12	45.336	−15.373	−7.849	1.00	0.00	C
ATOM	189	NZ	LYS	A	12	45.434	−14.022	−8.445	1.00	0.00	N
ATOM	190	H	LYS	A	12	41.368	−17.191	−10.876	1.00	0.00	H
ATOM	191	HA	LYS	A	12	43.757	−16.763	−12.628	1.00	0.00	H
ATOM	192	HB2	LYS	A	12	43.575	−18.404	−10.086	1.00	0.00	H
ATOM	193	HB3	LYS	A	12	45.016	−18.102	−11.074	1.00	0.00	H
ATOM	194	HG2	LYS	A	12	44.634	−15.610	−10.677	1.00	0.00	H
ATOM	195	HG3	LYS	A	12	43.389	−16.064	−9.498	1.00	0.00	H
ATOM	196	HD2	LYS	A	12	45.268	−17.449	−8.353	1.00	0.00	H
ATOM	197	HD3	LYS	A	12	46.388	−16.518	−9.356	1.00	0.00	H
ATOM	198	HE2	LYS	A	12	44.382	−15.433	−7.315	1.00	0.00	H
ATOM	199	HE3	LYS	A	12	46.121	−15.499	−7.097	1.00	0.00	H
ATOM	200	HZ1	LYS	A	12	45.123	−13.338	−7.743	1.00	0.00	H
ATOM	201	HZ2	LYS	A	12	46.354	−13.750	−8.749	1.00	0.00	H
ATOM	202	HZ3	LYS	A	12	44.778	−13.890	−9.207	1.00	0.00	H
ATOM	203	N	PRO	A	13	42.721	−18.603	−14.161	1.00	0.00	N
ATOM	204	CA	PRO	A	13	42.212	−19.673	−15.031	1.00	0.00	C
ATOM	205	C	PRO	A	13	43.206	−20.839	−15.209	1.00	0.00	C
ATOM	206	O	PRO	A	13	44.265	−20.883	−14.575	1.00	0.00	O
ATOM	207	CB	PRO	A	13	41.911	−18.964	−16.367	1.00	0.00	C
ATOM	208	CG	PRO	A	13	42.965	−17.865	−16.425	1.00	0.00	C
ATOM	209	CD	PRO	A	13	43.020	−17.421	−14.967	1.00	0.00	C
ATOM	210	HA	PRO	A	13	41.282	−20.076	−14.631	1.00	0.00	H
ATOM	211	HB2	PRO	A	13	40.928	−18.497	−16.321	1.00	0.00	H

ATOM 212 HB3 PRO A 13 41.968 −19.622 −17.235 1.00 0.00 H
ATOM 213 HG2 PRO A 13 43.928 −18.283 −16.726 1.00 0.00 H
ATOM 214 HG3 PRO A 13 42.672 −17.049 −17.088 1.00 0.00 H
ATOM 215 HD2 PRO A 13 42.255 −16.665 −14.798 1.00 0.00 H
ATOM 216 HD3 PRO A 13 44.008 −17.015 −14.745 1.00 0.00 H
ATOM 217 N ARG A 14 42.865 −21.803 −16.072 1.00 0.00 N
ATOM 218 CA ARG A 14 43.782 −22.766 −16.709 1.00 0.00 C
ATOM 219 C ARG A 14 43.227 −23.218 −18.056 1.00 0.00 C
ATOM 220 O ARG A 14 42.066 −23.608 −18.128 1.00 0.00 O
ATOM 221 CB ARG A 14 44.005 −23.992 −15.797 1.00 0.00 C
ATOM 222 CG ARG A 14 45.386 −23.988 −15.114 1.00 0.00 C
ATOM 223 CD ARG A 14 45.287 −24.303 −13.619 1.00 0.00 C
ATOM 224 NE ARG A 14 44.662 −23.182 −12.895 1.00 0.00 N
ATOM 225 CZ ARG A 14 44.369 −23.138 −11.612 1.00 0.00 C
ATOM 226 NH1 ARG A 14 44.609 −24.144 −10.816 1.00 0.00 N
ATOM 227 NH2 ARG A 14 43.821 −22.076 −11.098 1.00 0.00 N
ATOM 228 H ARG A 14 41.901 −21.808 −16.395 1.00 0.00 H
ATOM 229 HA ARG A 14 44.734 −22.269 −16.907 1.00 0.00 H
ATOM 230 HB2 ARG A 14 43.937 −24.908 −16.388 1.00 0.00 H
ATOM 231 HB3 ARG A 14 43.203 −24.044 −15.057 1.00 0.00 H
ATOM 232 HG2 ARG A 14 46.014 −24.742 −15.592 1.00 0.00 H
ATOM 233 HG3 ARG A 14 45.879 −23.022 −15.230 1.00 0.00 H
ATOM 234 HD2 ARG A 14 44.706 −25.217 −13.484 1.00 0.00 H
ATOM 235 HD3 ARG A 14 46.296 −24.465 −13.233 1.00 0.00 H
ATOM 236 HE ARG A 14 44.494 −22.336 −13.437 1.00 0.00 H
ATOM 237 HH11 ARG A 14 45.013 −24.978 −11.206 1.00 0.00 H
ATOM 238 HH12 ARG A 14 44.382 −24.111 −9.841 1.00 0.00 H
ATOM 239 HH21 ARG A 14 43.557 −21.300 −11.706 1.00 0.00 H
ATOM 240 HH22 ARG A 14 43.639 −22.022 −10.120 1.00 0.00 H
ATOM 241 N LEU A 15 44.096 −23.335 −19.060 1.00 0.00 N

ATOM 242 CA LEU A 15 43.834 −24.121 −20.267 1.00 0.00 C
ATOM 243 C LEU A 15 43.809 −25.615 −19.898 1.00 0.00 C
ATOM 244 O LEU A 15 44.801 −26.138 −19.398 1.00 0.00 O
ATOM 245 CB LEU A 15 44.931 −23.783 −21.301 1.00 0.00 C
ATOM 246 CG LEU A 15 44.927 −24.630 −22.587 1.00 0.00 C
ATOM 247 CD1 LEU A 15 43.663 −24.422 −23.421 1.00 0.00 C
ATOM 248 CD2 LEU A 15 46.128 −24.262 −23.456 1.00 0.00 C
ATOM 249 H LEU A 15 45.048 −23.052 −18.898 1.00 0.00 H
ATOM 250 HA LEU A 15 42.862 −23.838 −20.677 1.00 0.00 H
ATOM 251 HB2 LEU A 15 45.902 −23.920 −20.820 1.00 0.00 H
ATOM 252 HB3 LEU A 15 44.840 −22.730 −21.573 1.00 0.00 H
ATOM 253 HG LEU A 15 45.005 −25.687 −22.333 1.00 0.00 H
ATOM 254 HD11 LEU A 15 43.740 −24.969 −24.360 1.00 0.00 H
ATOM 255 HD12 LEU A 15 43.535 −23.359 −23.634 1.00 0.00 H
ATOM 256 HD13 LEU A 15 42.792 −24.780 −22.880 1.00 0.00 H
ATOM 257 HD21 LEU A 15 47.052 −24.432 −22.903 1.00 0.00 H
ATOM 258 HD22 LEU A 15 46.077 −23.211 −23.744 1.00 0.00 H
ATOM 259 HD23 LEU A 15 46.145 −24.876 −24.356 1.00 0.00 H
ATOM 260 N ILE A 16 42.721 −26.314 −20.227 1.00 0.00 N
ATOM 261 CA ILE A 16 42.559 −27.771 −20.037 1.00 0.00 C
ATOM 262 C ILE A 16 42.486 −28.521 −21.383 1.00 0.00 C
ATOM 263 O ILE A 16 41.862 −29.576 −21.500 1.00 0.00 O
ATOM 264 CB ILE A 16 41.385 −28.092 −19.078 1.00 0.00 C
ATOM 265 CG1 ILE A 16 40.023 −27.655 −19.659 1.00 0.00 C
ATOM 266 CG2 ILE A 16 41.615 −27.485 −17.683 1.00 0.00 C
ATOM 267 CD1 ILE A 16 38.852 −28.479 −19.122 1.00 0.00 C
ATOM 268 H ILE A 16 41.935 −25.803 −20.625 1.00 0.00 H
ATOM 269 HA ILE A 16 43.458 −28.152 −19.551 1.00 0.00 H
ATOM 270 HB ILE A 16 41.368 −29.177 −18.952 1.00 0.00 H
ATOM 271 HG12 ILE A 16 39.852 −26.598 −19.454 1.00 0.00 H

```
ATOM    272 HG13 ILE A  16      40.038 −27.793 −20.735  1.00  0.00           H
ATOM    273 HG21 ILE A  16      42.589 −27.793 −17.302  1.00  0.00           H
ATOM    274 HG22 ILE A  16      41.572 −26.397 −17.726  1.00  0.00           H
ATOM    275 HG23 ILE A  16      40.849 −27.847 −16.995  1.00  0.00           H
ATOM    276 HD11 ILE A  16      39.016 −29.536 −19.331  1.00  0.00           H
ATOM    277 HD12 ILE A  16      37.935 −28.164 −19.622  1.00  0.00           H
ATOM    278 HD13 ILE A  16      38.746 −28.328 −18.049  1.00  0.00           H
ATOM    279  N   SER A  17      43.075 −27.945 −22.436  1.00  0.00           N
ATOM    280  CA  SER A  17      42.952 −28.358 −23.846  1.00  0.00           C
ATOM    281  C   SER A  17      41.504 −28.349 −24.343  1.00  0.00           C
ATOM    282  O   SER A  17      41.102 −27.443 −25.080  1.00  0.00           O
ATOM    283  CB  SER A  17      43.609 −29.723 −24.097  1.00  0.00           C
ATOM    284  OG  SER A  17      42.722 −30.772 −23.763  1.00  0.00           O
ATOM    285  H   SER A  17      43.557 −27.078 −22.252  1.00  0.00           H
ATOM    286  HA  SER A  17      43.496 −27.629 −24.446  1.00  0.00           H
ATOM    287  HB2 SER A  17      44.527 −29.810 −23.512  1.00  0.00           H
ATOM    288  HB3 SER A  17      43.861 −29.808 −25.155  1.00  0.00           H
ATOM    289  HG  SER A  17      42.459 −30.659 −22.829  1.00  0.00           H
ATOM    290  H   SER A  17      40.824 −29.147 −24.045  1.00  0.00           H
ATOM    291  N   GLU A  18      47.852 −30.099 −40.413  1.00  0.00           N
ATOM    292  CA  GLU A  18      47.877 −29.395 −41.699  1.00  0.00           C
ATOM    293  C   GLU A  18      46.935 −28.166 −41.646  1.00  0.00           C
ATOM    294  O   GLU A  18      46.825 −27.560 −40.581  1.00  0.00           O
ATOM    295  CB  GLU A  18      47.624 −30.413 −42.829  1.00  0.00           C
ATOM    296  CG  GLU A  18      48.078 −29.927 −44.224  1.00  0.00           C
ATOM    297  CD  GLU A  18      46.946 −29.884 −45.262  1.00  0.00           C
ATOM    298  OE1 GLU A  18      45.827 −29.477 −44.875  1.00  0.00           O
ATOM    299  OE2 GLU A  18      47.248 −30.083 −46.458  1.00  0.00           O
ATOM    300  HA  GLU A  18      48.884 −29.003 −41.835  1.00  0.00           H
ATOM    301  HB2 GLU A  18      46.574 −30.706 −42.827  1.00  0.00           H
```

ATOM	302	HB3	GLU	A	18	48.200	−31.314	−42.608	1.00	0.00	H
ATOM	303	HG2	GLU	A	18	48.520	−28.932	−44.139	1.00	0.00	H
ATOM	304	HG3	GLU	A	18	48.869	−30.595	−44.576	1.00	0.00	H
ATOM	305	H	GLU	A	18	47.471	−29.570	−39.635	1.00	0.00	H
ATOM	306	H	GLU	A	18	48.805	−30.358	−40.179	1.00	0.00	H
ATOM	307	N	GLY	A	19	46.356	−27.704	−42.760	1.00	0.00	N
ATOM	308	CA	GLY	A	19	45.510	−26.507	−42.816	1.00	0.00	C
ATOM	309	C	GLY	A	19	44.054	−26.823	−42.475	1.00	0.00	C
ATOM	310	O	GLY	A	19	43.267	−27.200	−43.348	1.00	0.00	O
ATOM	311	H	GLY	A	19	46.347	−28.301	−43.587	1.00	0.00	H
ATOM	312	HA2	GLY	A	19	45.886	−25.752	−42.125	1.00	0.00	H
ATOM	313	HA3	GLY	A	19	45.543	−26.100	−43.825	1.00	0.00	H
ATOM	314	N	VAL	A	20	43.654	−26.625	−41.218	1.00	0.00	N
ATOM	315	CA	VAL	A	20	42.374	−27.122	−40.679	1.00	0.00	C
ATOM	316	C	VAL	A	20	41.660	−26.060	−39.843	1.00	0.00	C
ATOM	317	O	VAL	A	20	42.298	−25.317	−39.105	1.00	0.00	O
ATOM	318	CB	VAL	A	20	42.614	−28.437	−39.897	1.00	0.00	C
ATOM	319	CG1	VAL	A	20	41.449	−28.895	−39.005	1.00	0.00	C
ATOM	320	CG2	VAL	A	20	42.910	−29.593	−40.867	1.00	0.00	C
ATOM	321	H	VAL	A	20	44.333	−26.237	−40.562	1.00	0.00	H
ATOM	322	HA	VAL	A	20	41.711	−27.349	−41.509	1.00	0.00	H
ATOM	323	HB	VAL	A	20	43.481	−28.303	−39.255	1.00	0.00	H
ATOM	324	HG21	VAL	A	20	43.075	−30.512	−40.306	1.00	0.00	H
ATOM	325	HG22	VAL	A	20	42.080	−29.712	−41.562	1.00	0.00	H
ATOM	326	HG23	VAL	A	20	43.818	−29.378	−41.433	1.00	0.00	H
ATOM	327	HG11	VAL	A	20	41.688	−29.855	−38.543	1.00	0.00	H
ATOM	328	HG12	VAL	A	20	40.539	−29.003	−39.591	1.00	0.00	H
ATOM	329	HG13	VAL	A	20	41.285	−28.176	−38.203	1.00	0.00	H
ATOM	330	N	CYS	A	21	40.324	−26.107	−39.823	1.00	0.00	N
ATOM	331	CA	CYS	A	21	39.523	−25.474	−38.775	1.00	0.00	C

ATOM	332	C	CYS	A	21	38.522	−26.450	−38.134	1.00	0.00	C
ATOM	333	O	CYS	A	21	37.795	−27.162	−38.826	1.00	0.00	O
ATOM	334	CB	CYS	A	21	38.843	−24.203	−39.296	1.00	0.00	C
ATOM	335	SG	CYS	A	21	37.836	−23.395	−38.020	1.00	0.00	S
ATOM	336	H	CYS	A	21	39.865	−26.774	−40.434	1.00	0.00	H
ATOM	337	HA	CYS	A	21	40.194	−25.155	−37.982	1.00	0.00	H
ATOM	338	HB2	CYS	A	21	39.624	−23.512	−39.620	1.00	0.00	H
ATOM	339	HB3	CYS	A	21	38.218	−24.450	−40.154	1.00	0.00	H
ATOM	340	N	ILE	A	22	38.426	−26.415	−36.806	1.00	0.00	N
ATOM	341	CA	ILE	A	22	37.385	−27.046	−35.989	1.00	0.00	C
ATOM	342	C	ILE	A	22	36.337	−25.982	−35.619	1.00	0.00	C
ATOM	343	O	ILE	A	22	36.694	−24.978	−35.011	1.00	0.00	O
ATOM	344	CB	ILE	A	22	38.038	−27.659	−34.727	1.00	0.00	C
ATOM	345	CG1	ILE	A	22	39.206	−28.632	−35.012	1.00	0.00	C
ATOM	346	CG2	ILE	A	22	36.981	−28.338	−33.852	1.00	0.00	C
ATOM	347	CD1	ILE	A	22	38.840	−29.866	−35.843	1.00	0.00	C
ATOM	348	H	ILE	A	22	39.069	−25.793	−36.321	1.00	0.00	H
ATOM	349	HA	ILE	A	22	36.892	−27.834	−36.557	1.00	0.00	H
ATOM	350	HB	ILE	A	22	38.456	−26.845	−34.143	1.00	0.00	H
ATOM	351	HG12	ILE	A	22	39.621	−28.972	−34.062	1.00	0.00	H
ATOM	352	HG13	ILE	A	22	40.004	−28.096	−35.530	1.00	0.00	H
ATOM	353	HG21	ILE	A	22	36.462	−29.086	−34.444	1.00	0.00	H
ATOM	354	HG22	ILE	A	22	36.261	−27.607	−33.482	1.00	0.00	H
ATOM	355	HG23	ILE	A	22	37.452	−28.819	−32.994	1.00	0.00	H
ATOM	356	HD11	ILE	A	22	39.740	−30.451	−36.037	1.00	0.00	H
ATOM	357	HD12	ILE	A	22	38.416	−29.547	−36.790	1.00	0.00	H
ATOM	358	HD13	ILE	A	22	38.124	−30.488	−35.308	1.00	0.00	H
ATOM	359	N	THR	A	23	35.061	−26.173	−35.973	1.00	0.00	N
ATOM	360	CA	THR	A	23	34.011	−25.139	−35.810	1.00	0.00	C
ATOM	361	C	THR	A	23	32.593	−25.736	−35.677	1.00	0.00	C

ATOM	362	O	THR	A	23	32.461	−26.906	−35.326	1.00	0.00	O
ATOM	363	CB	THR	A	23	34.154	−24.107	−36.947	1.00	0.00	C
ATOM	364	OG1	THR	A	23	33.341	−22.973	−36.758	1.00	0.00	O
ATOM	365	CG2	THR	A	23	33.810	−24.689	−38.317	1.00	0.00	C
ATOM	366	H	THR	A	23	34.825	−26.999	−36.512	1.00	0.00	H
ATOM	367	HA	THR	A	23	34.204	−24.604	−34.880	1.00	0.00	H
ATOM	368	HB	THR	A	23	35.187	−23.761	−36.969	1.00	0.00	H
ATOM	369	HG1	THR	A	23	33.888	−22.321	−36.243	1.00	0.00	H
ATOM	370	HG21	THR	A	23	33.941	−23.913	−39.065	1.00	0.00	H
ATOM	371	HG22	THR	A	23	32.777	−25.034	−38.338	1.00	0.00	H
ATOM	372	HG23	THR	A	23	34.480	−25.518	−38.544	1.00	0.00	H
ATOM	373	N	ASP	A	24	31.544	−24.905	−35.745	1.00	0.00	N
ATOM	374	CA	ASP	A	24	30.172	−25.150	−35.241	1.00	0.00	C
ATOM	375	C	ASP	A	24	30.130	−25.782	−33.823	1.00	0.00	C
ATOM	376	O	ASP	A	24	29.601	−26.890	−33.655	1.00	0.00	O
ATOM	377	CB	ASP	A	24	29.320	−25.911	−36.277	1.00	0.00	C
ATOM	378	CG	ASP	A	24	27.824	−25.942	−35.912	1.00	0.00	C
ATOM	379	OD1	ASP	A	24	27.267	−24.883	−35.555	1.00	0.00	O
ATOM	380	OD2	ASP	A	24	27.179	−26.974	−36.224	1.00	0.00	O
ATOM	381	H	ASP	A	24	31.771	−23.951	−36.009	1.00	0.00	H
ATOM	382	HA	ASP	A	24	29.708	−24.169	−35.132	1.00	0.00	H
ATOM	383	HB2	ASP	A	24	29.698	−26.930	−36.377	1.00	0.00	H
ATOM	384	HB3	ASP	A	24	29.425	−25.420	−37.246	1.00	0.00	H
ATOM	385	N	PRO	A	25	30.851	−25.203	−32.835	1.00	0.00	N
ATOM	386	CA	PRO	A	25	31.111	−25.870	−31.568	1.00	0.00	C
ATOM	387	C	PRO	A	25	29.889	−25.889	−30.640	1.00	0.00	C
ATOM	388	O	PRO	A	25	29.296	−24.848	−30.335	1.00	0.00	O
ATOM	389	CB	PRO	A	25	32.292	−25.131	−30.942	1.00	0.00	C
ATOM	390	CG	PRO	A	25	32.151	−23.713	−31.483	1.00	0.00	C
ATOM	391	CD	PRO	A	25	31.562	−23.927	−32.874	1.00	0.00	C

ATOM	392	HA	PRO	A	25	31.420	−26.894	−31.768	1.00	0.00	H
ATOM	393	HB2	PRO	A	25	32.249	−25.151	−29.856	1.00	0.00	H
ATOM	394	HB3	PRO	A	25	33.223	−25.568	−31.302	1.00	0.00	H
ATOM	395	HG2	PRO	A	25	31.458	−23.149	−30.863	1.00	0.00	H
ATOM	396	HG3	PRO	A	25	33.116	−23.213	−31.533	1.00	0.00	H
ATOM	397	HD2	PRO	A	25	32.390	−23.982	−33.577	1.00	0.00	H
ATOM	398	HD3	PRO	A	25	30.898	−23.104	−33.142	1.00	0.00	H
ATOM	399	N	LEU	A	26	29.713	−27.034	−29.981	1.00	0.00	N
ATOM	400	CA	LEU	A	26	28.813	−27.256	−28.849	1.00	0.00	C
ATOM	401	C	LEU	A	26	29.625	−27.688	−27.624	1.00	0.00	C
ATOM	402	O	LEU	A	26	30.477	−28.568	−27.731	1.00	0.00	O
ATOM	403	CB	LEU	A	26	27.745	−28.286	−29.272	1.00	0.00	C
ATOM	404	CG	LEU	A	26	26.740	−28.757	−28.193	1.00	0.00	C
ATOM	405	CD1	LEU	A	26	25.454	−29.220	−28.876	1.00	0.00	C
ATOM	406	CD2	LEU	A	26	27.221	−29.953	−27.368	1.00	0.00	C
ATOM	407	H	LEU	A	26	30.322	−27.806	−30.239	1.00	0.00	H
ATOM	408	HA	LEU	A	26	28.304	−26.323	−28.604	1.00	0.00	H
ATOM	409	HB2	LEU	A	26	27.186	−27.834	−30.093	1.00	0.00	H
ATOM	410	HB3	LEU	A	26	28.252	−29.158	−29.670	1.00	0.00	H
ATOM	411	HG	LEU	A	26	26.504	−27.928	−27.528	1.00	0.00	H
ATOM	412	HD21	LEU	A	26	26.458	−30.227	−26.639	1.00	0.00	H
ATOM	413	HD22	LEU	A	26	27.418	−30.808	−28.013	1.00	0.00	H
ATOM	414	HD23	LEU	A	26	28.118	−29.704	−26.811	1.00	0.00	H
ATOM	415	HD11	LEU	A	26	24.730	−29.544	−28.129	1.00	0.00	H
ATOM	416	HD12	LEU	A	26	25.658	−30.041	−29.563	1.00	0.00	H
ATOM	417	HD13	LEU	A	26	25.025	−28.386	−29.425	1.00	0.00	H
ATOM	418	N	LEU	A	27	29.206	−27.209	−26.454	1.00	0.00	N
ATOM	419	CA	LEU	A	27	29.513	−27.755	−25.133	1.00	0.00	C
ATOM	420	C	LEU	A	27	28.234	−27.672	−24.288	1.00	0.00	C
ATOM	421	O	LEU	A	27	27.495	−26.694	−24.406	1.00	0.00	O

ATOM	422	CB	LEU	A	27	30.689	−26.983	−24.496	1.00	0.00	C
ATOM	423	CG	LEU	A	27	31.011	−27.381	−23.038	1.00	0.00	C
ATOM	424	CD1	LEU	A	27	31.580	−28.798	−22.938	1.00	0.00	C
ATOM	425	CD2	LEU	A	27	32.040	−26.429	−22.435	1.00	0.00	C
ATOM	426	H	LEU	A	27	28.448	−26.543	−26.461	1.00	0.00	H
ATOM	427	HA	LEU	A	27	29.796	−28.802	−25.235	1.00	0.00	H
ATOM	428	HB2	LEU	A	27	31.579	−27.130	−25.106	1.00	0.00	H
ATOM	429	HB3	LEU	A	27	30.441	−25.921	−24.508	1.00	0.00	H
ATOM	430	HG	LEU	A	27	30.113	−27.312	−22.427	1.00	0.00	H
ATOM	431	HD11	LEU	A	27	30.841	−29.523	−23.270	1.00	0.00	H
ATOM	432	HD12	LEU	A	27	32.477	−28.893	−23.549	1.00	0.00	H
ATOM	433	HD13	LEU	A	27	31.822	−29.024	−21.898	1.00	0.00	H
ATOM	434	HD21	LEU	A	27	32.996	−26.535	−22.941	1.00	0.00	H
ATOM	435	HD22	LEU	A	27	32.160	−26.669	−21.380	1.00	0.00	H
ATOM	436	HD23	LEU	A	27	31.686	−25.400	−22.512	1.00	0.00	H
ATOM	437	N	ALA	A	28	28.009	−28.657	−23.427	1.00	0.00	N
ATOM	438	CA	ALA	A	28	26.996	−28.628	−22.378	1.00	0.00	C
ATOM	439	C	ALA	A	28	27.507	−29.389	−21.151	1.00	0.00	C
ATOM	440	O	ALA	A	28	28.128	−30.441	−21.322	1.00	0.00	O
ATOM	441	CB	ALA	A	28	25.713	−29.267	−22.919	1.00	0.00	C
ATOM	442	H	ALA	A	28	28.619	−29.466	−23.444	1.00	0.00	H
ATOM	443	HA	ALA	A	28	26.790	−27.596	−22.093	1.00	0.00	H
ATOM	444	HB1	ALA	A	28	24.916	−29.174	−22.180	1.00	0.00	H
ATOM	445	HB2	ALA	A	28	25.417	−28.773	−23.843	1.00	0.00	H
ATOM	446	HB3	ALA	A	28	25.883	−30.325	−23.121	1.00	0.00	H
ATOM	447	N	VAL	A	29	27.206	−28.915	−19.942	1.00	0.00	N
ATOM	448	CA	VAL	A	29	27.513	−29.596	−18.675	1.00	0.00	C
ATOM	449	C	VAL	A	29	26.244	−29.750	−17.836	1.00	0.00	C
ATOM	450	O	VAL	A	29	25.599	−28.771	−17.465	1.00	0.00	O
ATOM	451	CB	VAL	A	29	28.634	−28.874	−17.896	1.00	0.00	C

ATOM 452 CG1 VAL A 29 28.977 −29.598 −16.585 1.00 0.00 C
ATOM 453 CG2 VAL A 29 29.927 −28.768 −18.719 1.00 0.00 C
ATOM 454 H VAL A 29 26.836 −27.965 −19.892 1.00 0.00 H
ATOM 455 HA VAL A 29 27.875 −30.598 −18.899 1.00 0.00 H
ATOM 456 HB VAL A 29 28.304 −27.867 −17.648 1.00 0.00 H
ATOM 457 HG11 VAL A 29 28.121 −29.578 −15.911 1.00 0.00 H
ATOM 458 HG12 VAL A 29 29.809 −29.097 −16.090 1.00 0.00 H
ATOM 459 HG13 VAL A 29 29.251 −30.633 −16.790 1.00 0.00 H
ATOM 460 HG21 VAL A 29 30.706 −28.288 −18.128 1.00 0.00 H
ATOM 461 HG22 VAL A 29 30.257 −29.762 −19.023 1.00 0.00 H
ATOM 462 HG23 VAL A 29 29.751 −28.160 −19.608 1.00 0.00 H
ATOM 463 N ASP A 30 25.979 −30.976 −17.386 1.00 0.00 N
ATOM 464 CA ASP A 30 24.855 −31.337 −16.523 1.00 0.00 C
ATOM 465 C ASP A 30 25.327 −32.279 −15.413 1.00 0.00 C
ATOM 466 O ASP A 30 25.739 −33.407 −15.671 1.00 0.00 O
ATOM 467 CB ASP A 30 23.759 −32.039 −17.332 1.00 0.00 C
ATOM 468 CG ASP A 30 22.716 −31.107 −17.950 1.00 0.00 C
ATOM 469 OD1 ASP A 30 21.921 −30.639 −17.098 1.00 0.00 O
ATOM 470 OD2 ASP A 30 22.293 −31.477 −19.072 1.00 0.00 O
ATOM 471 H ASP A 30 26.607 −31.723 −17.667 1.00 0.00 H
ATOM 472 HA ASP A 30 24.427 −30.444 −16.065 1.00 0.00 H
ATOM 473 HB2 ASP A 30 23.226 −32.714 −16.659 1.00 0.00 H
ATOM 474 HB3 ASP A 30 24.223 −32.663 −18.098 1.00 0.00 H
ATOM 475 N ASN A 31 25.123 −31.875 −14.157 1.00 0.00 N
ATOM 476 CA ASN A 31 25.235 −32.733 −12.966 1.00 0.00 C
ATOM 477 C ASN A 31 26.608 −33.416 −12.719 1.00 0.00 C
ATOM 478 O ASN A 31 26.746 −34.096 −11.708 1.00 0.00 O
ATOM 479 CB ASN A 31 24.060 −33.742 −12.963 1.00 0.00 C
ATOM 480 CG ASN A 31 22.694 −33.090 −13.103 1.00 0.00 C
ATOM 481 OD1 ASN A 31 22.000 −33.203 −14.105 1.00 0.00 O

ATOM 482 ND2 ASN A 31 22.285 −32.321 −12.120 1.00 0.00 N
ATOM 483 H ASN A 31 24.757 −30.944 −14.033 1.00 0.00 H
ATOM 484 HA ASN A 31 25.105 −32.083 −12.102 1.00 0.00 H
ATOM 485 HB2 ASN A 31 24.189 −34.450 −13.781 1.00 0.00 H
ATOM 486 HB3 ASN A 31 24.078 −34.307 −12.031 1.00 0.00 H
ATOM 487 HD21 ASN A 31 22.831 −32.248 −11.281 1.00 0.00 H
ATOM 488 HD22 ASN A 31 21.389 −31.884 −12.224 1.00 0.00 H
ATOM 489 N GLY A 32 27.624 −33.166 −13.555 1.00 0.00 N
ATOM 490 CA GLY A 32 28.937 −33.834 −13.541 1.00 0.00 C
ATOM 491 C GLY A 32 29.290 −34.541 −14.861 1.00 0.00 C
ATOM 492 O GLY A 32 30.462 −34.810 −15.122 1.00 0.00 O
ATOM 493 H GLY A 32 27.401 −32.630 −14.381 1.00 0.00 H
ATOM 494 HA2 GLY A 32 28.961 −34.586 −12.751 1.00 0.00 H
ATOM 495 HA3 GLY A 32 29.713 −33.096 −13.333 1.00 0.00 H
ATOM 496 N PHE A 33 28.309 −34.709 −15.750 1.00 0.00 N
ATOM 497 CA PHE A 33 28.478 −35.166 −17.130 1.00 0.00 C
ATOM 498 C PHE A 33 28.590 −33.987 −18.105 1.00 0.00 C
ATOM 499 O PHE A 33 28.134 −32.882 −17.805 1.00 0.00 O
ATOM 500 CB PHE A 33 27.300 −36.078 −17.500 1.00 0.00 C
ATOM 501 CG PHE A 33 27.084 −37.199 −16.501 1.00 0.00 C
ATOM 502 CD1 PHE A 33 28.071 −38.188 −16.349 1.00 0.00 C
ATOM 503 CD2 PHE A 33 25.950 −37.211 −15.669 1.00 0.00 C
ATOM 504 CE1 PHE A 33 27.928 −39.190 −15.375 1.00 0.00 C
ATOM 505 CE2 PHE A 33 25.804 −38.216 −14.695 1.00 0.00 C
ATOM 506 CZ PHE A 33 26.791 −39.208 −14.550 1.00 0.00 C
ATOM 507 H PHE A 33 27.377 −34.401 −15.500 1.00 0.00 H
ATOM 508 HA PHE A 33 29.396 −35.748 −17.200 1.00 0.00 H
ATOM 509 HB2 PHE A 33 26.392 −35.478 −17.572 1.00 0.00 H
ATOM 510 HB3 PHE A 33 27.488 −36.518 −18.481 1.00 0.00 H
ATOM 511 HD1 PHE A 33 28.945 −38.169 −16.980 1.00 0.00 H

```
ATOM    512  HD2 PHE A  33      25.198 −36.440 −15.770  1.00  0.00           H
ATOM    513  HE1 PHE A  33      28.701 −39.938 −15.258  1.00  0.00           H
ATOM    514  HE2 PHE A  33      24.941 −38.221 −14.049  1.00  0.00           H
ATOM    515  HZ  PHE A  33      26.683 −39.976 −13.797  1.00  0.00           H
ATOM    516  N   PHE A  34      29.056 −34.251 −19.328  1.00  0.00           N
ATOM    517  CA  PHE A  34      29.075 −33.267 −20.415  1.00  0.00           C
ATOM    518  C   PHE A  34      28.679 −33.861 −21.773  1.00  0.00           C
ATOM    519  O   PHE A  34      28.847 −35.060 −22.012  1.00  0.00           O
ATOM    520  CB  PHE A  34      30.447 −32.569 −20.477  1.00  0.00           C
ATOM    521  CG  PHE A  34      31.565 −33.383 −21.107  1.00  0.00           C
ATOM    522  CD1 PHE A  34      31.845 −33.249 −22.480  1.00  0.00           C
ATOM    523  CD2 PHE A  34      32.323 −34.275 −20.326  1.00  0.00           C
ATOM    524  CE1 PHE A  34      32.858 −34.022 −23.075  1.00  0.00           C
ATOM    525  CE2 PHE A  34      33.350 −35.031 −20.916  1.00  0.00           C
ATOM    526  CZ  PHE A  34      33.609 −34.914 −22.291  1.00  0.00           C
ATOM    527  H   PHE A  34      29.396 −35.187 −19.526  1.00  0.00           H
ATOM    528  HA  PHE A  34      28.330 −32.510 −20.183  1.00  0.00           H
ATOM    529  HB2 PHE A  34      30.343 −31.653 −21.055  1.00  0.00           H
ATOM    530  HB3 PHE A  34      30.736 −32.267 −19.474  1.00  0.00           H
ATOM    531  HD1 PHE A  34      31.271 −32.560 −23.084  1.00  0.00           H
ATOM    532  HD2 PHE A  34      32.106 −34.395 −19.276  1.00  0.00           H
ATOM    533  HE1 PHE A  34      33.056 −33.932 −24.133  1.00  0.00           H
ATOM    534  HE2 PHE A  34      33.921 −35.719 −20.313  1.00  0.00           H
ATOM    535  HZ  PHE A  34      34.383 −35.513 −22.744  1.00  0.00           H
ATOM    536  N   ALA A  35      28.321 −32.971 −22.701  1.00  0.00           N
ATOM    537  CA  ALA A  35      28.228 −33.227 −24.137  1.00  0.00           C
ATOM    538  C   ALA A  35      29.031 −32.200 −24.947  1.00  0.00           C
ATOM    539  O   ALA A  35      29.260 −31.073 −24.511  1.00  0.00           O
ATOM    540  CB  ALA A  35      26.762 −33.245 −24.574  1.00  0.00           C
ATOM    541  H   ALA A  35      28.244 −32.004 −22.401  1.00  0.00           H
```

ATOM 542 HA ALA A 35 28.654 −34.206 −24.346 1.00 0.00 H
ATOM 543 HB1 ALA A 35 26.694 −33.401 −25.651 1.00 0.00 H
ATOM 544 HB2 ALA A 35 26.259 −34.063 −24.073 1.00 0.00 H
ATOM 545 HB3 ALA A 35 26.278 −32.303 −24.316 1.00 0.00 H
ATOM 546 N TYR A 36 29.423 −32.590 −26.158 1.00 0.00 N
ATOM 547 CA TYR A 36 30.275 −31.819 −27.057 1.00 0.00 C
ATOM 548 C TYR A 36 29.952 −32.124 −28.529 1.00 0.00 C
ATOM 549 O TYR A 36 29.590 −33.249 −28.875 1.00 0.00 O
ATOM 550 CB TYR A 36 31.739 −32.124 −26.712 1.00 0.00 C
ATOM 551 CG TYR A 36 32.755 −31.482 −27.635 1.00 0.00 C
ATOM 552 CD1 TYR A 36 33.206 −30.172 −27.384 1.00 0.00 C
ATOM 553 CD2 TYR A 36 33.223 −32.184 −28.763 1.00 0.00 C
ATOM 554 CE1 TYR A 36 34.138 −29.567 −28.250 1.00 0.00 C
ATOM 555 CE2 TYR A 36 34.147 −31.578 −29.633 1.00 0.00 C
ATOM 556 CZ TYR A 36 34.613 −30.270 −29.376 1.00 0.00 C
ATOM 557 OH TYR A 36 35.520 −29.692 −30.206 1.00 0.00 O
ATOM 558 H TYR A 36 29.238 −33.554 −26.420 1.00 0.00 H
ATOM 559 HA TYR A 36 30.104 −30.761 −26.882 1.00 0.00 H
ATOM 560 HB2 TYR A 36 31.879 −33.203 −26.732 1.00 0.00 H
ATOM 561 HB3 TYR A 36 31.935 −31.791 −25.691 1.00 0.00 H
ATOM 562 HD1 TYR A 36 32.819 −29.622 −26.536 1.00 0.00 H
ATOM 563 HD2 TYR A 36 32.859 −33.179 −28.970 1.00 0.00 H
ATOM 564 HE1 TYR A 36 34.481 −28.560 −28.069 1.00 0.00 H
ATOM 565 HE2 TYR A 36 34.499 −32.115 −30.498 1.00 0.00 H
ATOM 566 HH TYR A 36 35.796 −30.311 −30.902 1.00 0.00 H
ATOM 567 N SER A 37 30.176 −31.160 −29.424 1.00 0.00 N
ATOM 568 CA SER A 37 30.195 −31.384 −30.879 1.00 0.00 C
ATOM 569 C SER A 37 31.152 −30.421 −31.571 1.00 0.00 C
ATOM 570 O SER A 37 31.192 −29.246 −31.206 1.00 0.00 O
ATOM 571 CB SER A 37 28.771 −31.323 −31.479 1.00 0.00 C

ATOM 572 OG SER A 37 28.649 −30.497 −32.636 1.00 0.00 O
ATOM 573 H SER A 37 30.533 −30.273 −29.080 1.00 0.00 H
ATOM 574 HA SER A 37 30.581 −32.388 −31.051 1.00 0.00 H
ATOM 575 HB2 SER A 37 28.481 −32.337 −31.747 1.00 0.00 H
ATOM 576 HB3 SER A 37 28.061 −30.985 −30.726 1.00 0.00 H
ATOM 577 HG SER A 37 28.840 −29.586 −32.367 1.00 0.00 H
ATOM 578 N HIS A 38 31.698 −30.845 −32.714 1.00 0.00 N
ATOM 579 CA HIS A 38 32.311 −29.950 −33.701 1.00 0.00 C
ATOM 580 C HIS A 38 32.132 −30.462 −35.130 1.00 0.00 C
ATOM 581 O HIS A 38 31.985 −31.663 −35.359 1.00 0.00 O
ATOM 582 CB HIS A 38 33.795 −29.706 −33.368 1.00 0.00 C
ATOM 583 CG HIS A 38 34.754 −30.875 −33.523 1.00 0.00 C
ATOM 584 ND1 HIS A 38 35.715 −31.238 −32.574 1.00 0.00 N
ATOM 585 CD2 HIS A 38 35.013 −31.576 −34.670 1.00 0.00 C
ATOM 586 CE1 HIS A 38 36.486 −32.172 −33.150 1.00 0.00 C
ATOM 587 NE2 HIS A 38 36.083 −32.401 −34.408 1.00 0.00 N
ATOM 588 H HIS A 38 31.507 −31.799 −33.004 1.00 0.00 H
ATOM 589 HA HIS A 38 31.805 −28.980 −33.651 1.00 0.00 H
ATOM 590 HB2 HIS A 38 34.147 −28.900 −34.011 1.00 0.00 H
ATOM 591 HB3 HIS A 38 33.860 −29.337 −32.345 1.00 0.00 H
ATOM 592 HD2 HIS A 38 34.513 −31.467 −35.620 1.00 0.00 H
ATOM 593 HE1 HIS A 38 37.324 −32.661 −32.669 1.00 0.00 H
ATOM 594 HE2 HIS A 38 36.519 −33.044 −35.057 1.00 0.00 H
ATOM 595 N LEU A 39 32.341 −29.570 −36.093 1.00 0.00 N
ATOM 596 CA LEU A 39 32.568 −29.848 −37.506 1.00 0.00 C
ATOM 597 C LEU A 39 34.018 −29.492 −37.876 1.00 0.00 C
ATOM 598 O LEU A 39 34.403 −28.324 −37.860 1.00 0.00 O
ATOM 599 CB LEU A 39 31.531 −29.038 −38.307 1.00 0.00 C
ATOM 600 CG LEU A 39 31.610 −29.229 −39.830 1.00 0.00 C
ATOM 601 CD1 LEU A 39 31.315 −30.676 −40.228 1.00 0.00 C

ATOM 602 CD2 LEU A 39 30.582 −28.326 −40.513 1.00 0.00 C
ATOM 603 H LEU A 39 32.463 −28.604 −35.802 1.00 0.00 H
ATOM 604 HA LEU A 39 32.412 −30.907 −37.697 1.00 0.00 H
ATOM 605 HB2 LEU A 39 30.532 −29.316 −37.968 1.00 0.00 H
ATOM 606 HB3 LEU A 39 31.674 −27.979 −38.083 1.00 0.00 H
ATOM 607 HG LEU A 39 32.601 −28.953 −40.192 1.00 0.00 H
ATOM 608 HD11 LEU A 39 32.150 −31.314 −39.946 1.00 0.00 H
ATOM 609 HD12 LEU A 39 31.171 −30.743 −41.303 1.00 0.00 H
ATOM 610 HD13 LEU A 39 30.404 −31.020 −39.741 1.00 0.00 H
ATOM 611 HD21 LEU A 39 30.661 −28.427 −41.594 1.00 0.00 H
ATOM 612 HD22 LEU A 39 29.577 −28.611 −40.200 1.00 0.00 H
ATOM 613 HD23 LEU A 39 30.761 −27.289 −40.230 1.00 0.00 H
ATOM 614 N GLU A 40 34.829 −30.497 −38.201 1.00 0.00 N
ATOM 615 CA GLU A 40 36.172 −30.314 −38.761 1.00 0.00 C
ATOM 616 C GLU A 40 36.102 −29.971 −40.254 1.00 0.00 C
ATOM 617 O GLU A 40 35.412 −30.654 −41.019 1.00 0.00 O
ATOM 618 CB GLU A 40 37.008 −31.587 −38.537 1.00 0.00 C
ATOM 619 CG GLU A 40 38.426 −31.465 −39.123 1.00 0.00 C
ATOM 620 CD GLU A 40 39.310 −32.676 −38.824 1.00 0.00 C
ATOM 621 OE1 GLU A 40 38.843 −33.782 −39.175 1.00 0.00 O
ATOM 622 OE2 GLU A 40 40.538 −32.459 −38.884 1.00 0.00 O
ATOM 623 H GLU A 40 34.438 −31.433 −38.231 1.00 0.00 H
ATOM 624 HA GLU A 40 36.660 −29.484 −38.247 1.00 0.00 H
ATOM 625 HB2 GLU A 40 36.499 −32.422 −39.013 1.00 0.00 H
ATOM 626 HB3 GLU A 40 37.080 −31.782 −37.467 1.00 0.00 H
ATOM 627 HG2 GLU A 40 38.895 −30.563 −38.733 1.00 0.00 H
ATOM 628 HG3 GLU A 40 38.365 −31.355 −40.208 1.00 0.00 H
ATOM 629 N LYS A 41 36.969 −29.057 −40.697 1.00 0.00 N
ATOM 630 CA LYS A 41 37.115 −28.605 −42.084 1.00 0.00 C
ATOM 631 C LYS A 41 38.571 −28.599 −42.541 1.00 0.00 C

ATOM	632	O	LYS	A	41	39.459	−28.232	−41.779	1.00	0.00	O
ATOM	633	CB	LYS	A	41	36.532	−27.192	−42.215	1.00	0.00	C
ATOM	634	CG	LYS	A	41	35.000	−27.188	−42.132	1.00	0.00	C
ATOM	635	CD	LYS	A	41	34.505	−25.755	−41.942	1.00	0.00	C
ATOM	636	CE	LYS	A	41	32.984	−25.682	−42.087	1.00	0.00	C
ATOM	637	NZ	LYS	A	41	32.604	−25.277	−43.460	1.00	0.00	N
ATOM	638	H	LYS	A	41	37.493	−28.535	−39.997	1.00	0.00	H
ATOM	639	HA	LYS	A	41	36.563	−29.279	−42.730	1.00	0.00	H
ATOM	640	HB2	LYS	A	41	36.950	−26.561	−41.427	1.00	0.00	H
ATOM	641	HB3	LYS	A	41	36.825	−26.766	−43.175	1.00	0.00	H
ATOM	642	HG2	LYS	A	41	34.590	−27.616	−43.047	1.00	0.00	H
ATOM	643	HG3	LYS	A	41	34.661	−27.781	−41.283	1.00	0.00	H
ATOM	644	HD2	LYS	A	41	34.988	−25.087	−42.657	1.00	0.00	H
ATOM	645	HD3	LYS	A	41	34.781	−25.432	−40.939	1.00	0.00	H
ATOM	646	HE2	LYS	A	41	32.556	−26.654	−41.826	1.00	0.00	H
ATOM	647	HE3	LYS	A	41	32.605	−24.942	−41.374	1.00	0.00	H
ATOM	648	HZ1	LYS	A	41	31.619	−25.409	−43.624	1.00	0.00	H
ATOM	649	HZ2	LYS	A	41	32.785	−24.283	−43.584	1.00	0.00	H
ATOM	650	HZ3	LYS	A	41	33.148	−25.784	−44.157	1.00	0.00	H
ATOM	651	N	ILE	A	42	38.782	−28.846	−43.832	1.00	0.00	N
ATOM	652	CA	ILE	A	42	39.983	−28.435	−44.572	1.00	0.00	C
ATOM	653	C	ILE	A	42	39.874	−26.930	−44.849	1.00	0.00	C
ATOM	654	O	ILE	A	42	38.910	−26.489	−45.483	1.00	0.00	O
ATOM	655	CB	ILE	A	42	40.129	−29.225	−45.896	1.00	0.00	C
ATOM	656	CG1	ILE	A	42	40.051	−30.752	−45.664	1.00	0.00	C
ATOM	657	CG2	ILE	A	42	41.447	−28.838	−46.594	1.00	0.00	C
ATOM	658	CD1	ILE	A	42	40.092	−31.599	−46.942	1.00	0.00	C
ATOM	659	H	ILE	A	42	37.971	−29.107	−44.381	1.00	0.00	H
ATOM	660	HA	ILE	A	42	40.868	−28.617	−43.958	1.00	0.00	H
ATOM	661	HB	ILE	A	42	39.304	−28.947	−46.553	1.00	0.00	H

ATOM 662 HG12 ILE A 42 40.865 −31.058 −45.007 1.00 0.00 H
ATOM 663 HG13 ILE A 42 39.105 −30.985 −45.176 1.00 0.00 H
ATOM 664 HG21 ILE A 42 41.492 −27.765 −46.778 1.00 0.00 H
ATOM 665 HG22 ILE A 42 41.532 −29.335 −47.560 1.00 0.00 H
ATOM 666 HG23 ILE A 42 42.301 −29.122 −45.976 1.00 0.00 H
ATOM 667 HD11 ILE A 42 39.877 −32.639 −46.691 1.00 0.00 H
ATOM 668 HD12 ILE A 42 39.340 −31.244 −47.649 1.00 0.00 H
ATOM 669 HD13 ILE A 42 41.078 −31.552 −47.401 1.00 0.00 H
ATOM 670 N GLY A 43 40.853 −26.149 −44.400 1.00 0.00 N
ATOM 671 CA GLY A 43 40.871 −24.693 −44.530 1.00 0.00 C
ATOM 672 C GLY A 43 39.778 −23.973 −43.726 1.00 0.00 C
ATOM 673 O GLY A 43 39.364 −24.418 −42.658 1.00 0.00 O
ATOM 674 H GLY A 43 41.642 −26.589 −43.932 1.00 0.00 H
ATOM 675 HA2 GLY A 43 41.840 −24.322 −44.191 1.00 0.00 H
ATOM 676 HA3 GLY A 43 40.764 −24.438 −45.584 1.00 0.00 H
ATOM 677 N SER A 44 39.377 −22.792 −44.214 1.00 0.00 N
ATOM 678 CA SER A 44 38.618 −21.785 −43.448 1.00 0.00 C
ATOM 679 C SER A 44 37.361 −22.322 −42.740 1.00 0.00 C
ATOM 680 O SER A 44 36.545 −23.020 −43.338 1.00 0.00 O
ATOM 681 CB SER A 44 38.240 −20.607 −44.361 1.00 0.00 C
ATOM 682 OG SER A 44 37.289 −20.990 −45.342 1.00 0.00 O
ATOM 683 H SER A 44 39.825 −22.488 −45.062 1.00 0.00 H
ATOM 684 HA SER A 44 39.286 −21.399 −42.677 1.00 0.00 H
ATOM 685 HB2 SER A 44 39.138 −20.229 −44.853 1.00 0.00 H
ATOM 686 HB3 SER A 44 37.816 −19.804 −43.756 1.00 0.00 H
ATOM 687 HG SER A 44 37.612 −21.795 −45.763 1.00 0.00 H
ATOM 688 N CYS A 45 37.075 −21.793 −41.549 1.00 0.00 N
ATOM 689 CA CYS A 45 35.934 −22.219 −40.729 1.00 0.00 C
ATOM 690 C CYS A 45 34.570 −22.018 −41.415 1.00 0.00 C
ATOM 691 O CYS A 45 33.711 −22.895 −41.369 1.00 0.00 O

ATOM 692 CB CYS A 45 35.988 −21.445 −39.408 1.00 0.00 C
ATOM 693 SG CYS A 45 37.608 −21.463 −38.602 1.00 0.00 S
ATOM 694 H CYS A 45 37.786 −21.248 −41.082 1.00 0.00 H
ATOM 695 HA CYS A 45 36.045 −23.281 −40.508 1.00 0.00 H
ATOM 696 HB2 CYS A 45 35.253 −21.860 −38.715 1.00 0.00 H
ATOM 697 HB3 CYS A 45 35.720 −20.404 −39.589 1.00 0.00 H
ATOM 698 N THR A 46 34.412 −20.958 −42.212 1.00 0.00 N
ATOM 699 CA THR A 46 33.191 −20.738 −43.001 1.00 0.00 C
ATOM 700 C THR A 46 33.122 −21.675 −44.214 1.00 0.00 C
ATOM 701 O THR A 46 32.301 −22.593 −44.245 1.00 0.00 O
ATOM 702 CB THR A 46 33.042 −19.262 −43.407 1.00 0.00 C
ATOM 703 OG1 THR A 46 34.166 −18.816 −44.133 1.00 0.00 O
ATOM 704 CG2 THR A 46 32.906 −18.338 −42.199 1.00 0.00 C
ATOM 705 H THR A 46 35.167 −20.294 −42.291 1.00 0.00 H
ATOM 706 HA THR A 46 32.331 −20.983 −42.375 1.00 0.00 H
ATOM 707 HB THR A 46 32.152 −19.153 −44.028 1.00 0.00 H
ATOM 708 HG1 THR A 46 34.385 −17.941 −43.792 1.00 0.00 H
ATOM 709 HG21 THR A 46 32.720 −17.316 −42.530 1.00 0.00 H
ATOM 710 HG22 THR A 46 32.063 −18.658 −41.586 1.00 0.00 H
ATOM 711 HG23 THR A 46 33.810 −18.359 −41.587 1.00 0.00 H
ATOM 712 N ARG A 47 34.016 −21.517 −45.198 1.00 0.00 N
ATOM 713 CA ARG A 47 33.886 −22.128 −46.540 1.00 0.00 C
ATOM 714 C ARG A 47 34.731 −23.388 −46.768 1.00 0.00 C
ATOM 715 O ARG A 47 34.648 −23.979 −47.837 1.00 0.00 O
ATOM 716 CB ARG A 47 34.132 −21.052 −47.614 1.00 0.00 C
ATOM 717 CG ARG A 47 33.023 −19.987 −47.591 1.00 0.00 C
ATOM 718 CD ARG A 47 33.265 −18.883 −48.625 1.00 0.00 C
ATOM 719 NE ARG A 47 32.190 −17.870 −48.565 1.00 0.00 N
ATOM 720 CZ ARG A 47 32.310 −16.559 −48.697 1.00 0.00 C
ATOM 721 NH1 ARG A 47 33.448 −15.983 −48.965 1.00 0.00 N

ATOM	722	NH2	ARG	A	47	31.267	−15.790	−48.557	1.00	0.00	N
ATOM	723	H	ARG	A	47	34.702	−20.778	−45.071	1.00	0.00	H
ATOM	724	HA	ARG	A	47	32.861	−22.482	−46.665	1.00	0.00	H
ATOM	725	HB2	ARG	A	47	35.104	−20.584	−47.445	1.00	0.00	H
ATOM	726	HB3	ARG	A	47	34.140	−21.519	−48.601	1.00	0.00	H
ATOM	727	HG2	ARG	A	47	32.065	−20.467	−47.798	1.00	0.00	H
ATOM	728	HG3	ARG	A	47	32.975	−19.526	−46.604	1.00	0.00	H
ATOM	729	HD2	ARG	A	47	34.236	−18.430	−48.416	1.00	0.00	H
ATOM	730	HD3	ARG	A	47	33.293	−19.328	−49.622	1.00	0.00	H
ATOM	731	HE	ARG	A	47	31.265	−18.219	−48.377	1.00	0.00	H
ATOM	732	HH11	ARG	A	47	34.266	−16.554	−49.081	1.00	0.00	H
ATOM	733	HH12	ARG	A	47	33.516	−14.985	−49.046	1.00	0.00	H
ATOM	734	HH21	ARG	A	47	30.372	−16.184	−48.327	1.00	0.00	H
ATOM	735	HH22	ARG	A	47	31.361	−14.793	−48.633	1.00	0.00	H
ATOM	736	N	GLY	A	48	35.482	−23.836	−45.765	1.00	0.00	N
ATOM	737	CA	GLY	A	48	36.289	−25.056	−45.809	1.00	0.00	C
ATOM	738	C	GLY	A	48	35.466	−26.342	−45.930	1.00	0.00	C
ATOM	739	O	GLY	A	48	34.333	−26.424	−45.436	1.00	0.00	O
ATOM	740	H	GLY	A	48	35.528	−23.289	−44.912	1.00	0.00	H
ATOM	741	HA2	GLY	A	48	36.974	−24.997	−46.656	1.00	0.00	H
ATOM	742	HA3	GLY	A	48	36.888	−25.119	−44.900	1.00	0.00	H
ATOM	743	N	ILE	A	49	36.051	−27.347	−46.588	1.00	0.00	N
ATOM	744	CA	ILE	A	49	35.428	−28.644	−46.886	1.00	0.00	C
ATOM	745	C	ILE	A	49	35.351	−29.481	−45.608	1.00	0.00	C
ATOM	746	O	ILE	A	49	36.379	−29.747	−44.992	1.00	0.00	O
ATOM	747	CB	ILE	A	49	36.209	−29.404	−47.990	1.00	0.00	C
ATOM	748	CG1	ILE	A	49	36.279	−28.617	−49.321	1.00	0.00	C
ATOM	749	CG2	ILE	A	49	35.559	−30.776	−48.263	1.00	0.00	C
ATOM	750	CD1	ILE	A	49	37.525	−27.728	−49.447	1.00	0.00	C
ATOM	751	H	ILE	A	49	37.019	−27.217	−46.857	1.00	0.00	H

ATOM 752 HA ILE A 49 34.414 −28.467 −47.246 1.00 0.00 H
ATOM 753 HB ILE A 49 37.224 −29.595 −47.639 1.00 0.00 H
ATOM 754 HG12 ILE A 49 35.380 −28.011 −49.445 1.00 0.00 H
ATOM 755 HG13 ILE A 49 36.316 −29.319 −50.156 1.00 0.00 H
ATOM 756 HG21 ILE A 49 35.588 −31.407 −47.373 1.00 0.00 H
ATOM 757 HG22 ILE A 49 34.526 −30.654 −48.587 1.00 0.00 H
ATOM 758 HG23 ILE A 49 36.115 −31.301 −49.042 1.00 0.00 H
ATOM 759 HD11 ILE A 49 37.547 −27.284 −50.443 1.00 0.00 H
ATOM 760 HD12 ILE A 49 37.508 −26.926 −48.712 1.00 0.00 H
ATOM 761 HD13 ILE A 49 38.426 −28.328 −49.310 1.00 0.00 H
ATOM 762 N ALA A 50 34.159 −29.948 −45.237 1.00 0.00 N
ATOM 763 CA ALA A 50 33.963 −30.791 −44.057 1.00 0.00 C
ATOM 764 C ALA A 50 34.711 −32.136 −44.171 1.00 0.00 C
ATOM 765 O ALA A 50 34.429 −32.908 −45.085 1.00 0.00 O
ATOM 766 CB ALA A 50 32.458 −31.011 −43.871 1.00 0.00 C
ATOM 767 H ALA A 50 33.364 −29.765 −45.825 1.00 0.00 H
ATOM 768 HA ALA A 50 34.340 −30.256 −43.185 1.00 0.00 H
ATOM 769 HB1 ALA A 50 32.300 −31.663 −43.015 1.00 0.00 H
ATOM 770 HB2 ALA A 50 32.039 −31.494 −44.754 1.00 0.00 H
ATOM 771 HB3 ALA A 50 31.955 −30.058 −43.697 1.00 0.00 H
ATOM 772 N LYS A 51 35.543 −32.472 −43.175 1.00 0.00 N
ATOM 773 CA LYS A 51 36.100 −33.828 −42.993 1.00 0.00 C
ATOM 774 C LYS A 51 35.198 −34.708 −42.127 1.00 0.00 C
ATOM 775 O LYS A 51 34.843 −35.806 −42.535 1.00 0.00 O
ATOM 776 CB LYS A 51 37.523 −33.785 −42.404 1.00 0.00 C
ATOM 777 CG LYS A 51 38.551 −33.093 −43.320 1.00 0.00 C
ATOM 778 CD LYS A 51 39.938 −33.764 −43.288 1.00 0.00 C
ATOM 779 CE LYS A 51 40.584 −33.827 −41.897 1.00 0.00 C
ATOM 780 NZ LYS A 51 41.147 −32.528 −41.462 1.00 0.00 N
ATOM 781 H LYS A 51 35.676 −31.791 −42.433 1.00 0.00 H

ATOM 782 HA LYS A 51 36.151 −34.327 −43.963 1.00 0.00 H
ATOM 783 HB2 LYS A 51 37.837 −34.818 −42.235 1.00 0.00 H
ATOM 784 HB3 LYS A 51 37.507 −33.281 −41.438 1.00 0.00 H
ATOM 785 HG2 LYS A 51 38.194 −33.133 −44.350 1.00 0.00 H
ATOM 786 HG3 LYS A 51 38.639 −32.045 −43.031 1.00 0.00 H
ATOM 787 HD2 LYS A 51 39.821 −34.788 −43.650 1.00 0.00 H
ATOM 788 HD3 LYS A 51 40.608 −33.255 −43.981 1.00 0.00 H
ATOM 789 HE2 LYS A 51 39.834 −34.160 −41.172 1.00 0.00 H
ATOM 790 HE3 LYS A 51 41.379 −34.578 −41.904 1.00 0.00 H
ATOM 791 HZ1 LYS A 51 42.028 −32.307 −41.901 1.00 0.00 H
ATOM 792 HZ2 LYS A 51 40.487 −31.776 −41.593 1.00 0.00 H
ATOM 793 HZ3 LYS A 51 41.281 −32.563 −40.443 1.00 0.00 H
ATOM 794 N GLN A 52 34.765 −34.202 −40.970 1.00 0.00 N
ATOM 795 CA GLN A 52 33.913 −34.935 −40.026 1.00 0.00 C
ATOM 796 C GLN A 52 33.124 −33.981 −39.118 1.00 0.00 C
ATOM 797 O GLN A 52 33.687 −33.060 −38.532 1.00 0.00 O
ATOM 798 CB GLN A 52 34.737 −35.946 −39.201 1.00 0.00 C
ATOM 799 CG GLN A 52 35.890 −35.327 −38.396 1.00 0.00 C
ATOM 800 CD GLN A 52 36.834 −36.376 −37.825 1.00 0.00 C
ATOM 801 OE1 GLN A 52 36.428 −37.393 −37.287 1.00 0.00 O
ATOM 802 NE2 GLN A 52 38.126 −36.138 −37.865 1.00 0.00 N
ATOM 803 H GLN A 52 35.093 −33.285 −40.698 1.00 0.00 H
ATOM 804 HA GLN A 52 33.203 −35.526 −40.600 1.00 0.00 H
ATOM 805 HB2 GLN A 52 35.154 −36.686 −39.886 1.00 0.00 H
ATOM 806 HB3 GLN A 52 34.071 −36.470 −38.515 1.00 0.00 H
ATOM 807 HG2 GLN A 52 36.468 −34.685 −39.054 1.00 0.00 H
ATOM 808 HG3 GLN A 52 35.503 −34.722 −37.577 1.00 0.00 H
ATOM 809 HE21 GLN A 52 38.475 −35.301 −38.343 1.00 0.00 H
ATOM 810 HE22 GLN A 52 38.736 −36.845 −37.504 1.00 0.00 H
ATOM 811 N ARG A 53 31.837 −34.276 −38.908 1.00 0.00 N

ATOM 812 CA ARG A 53 31.103 −33.891 −37.696 1.00 0.00 C
ATOM 813 C ARG A 53 31.421 −34.950 −36.645 1.00 0.00 C
ATOM 814 O ARG A 53 31.179 −36.118 −36.926 1.00 0.00 O
ATOM 815 CB ARG A 53 29.593 −33.831 −38.012 1.00 0.00 C
ATOM 816 CG ARG A 53 28.643 −33.751 −36.794 1.00 0.00 C
ATOM 817 CD ARG A 53 28.634 −32.400 −36.071 1.00 0.00 C
ATOM 818 NE ARG A 53 28.074 −31.349 −36.937 1.00 0.00 N
ATOM 819 CZ ARG A 53 27.935 −30.070 −36.651 1.00 0.00 C
ATOM 820 NH1 ARG A 53 27.457 −29.278 −37.555 1.00 0.00 N
ATOM 821 NH2 ARG A 53 28.225 −29.537 −35.500 1.00 0.00 N
ATOM 822 H ARG A 53 31.490 −35.099 −39.380 1.00 0.00 H
ATOM 823 HA ARG A 53 31.443 −32.922 −37.336 1.00 0.00 H
ATOM 824 HB2 ARG A 53 29.333 −34.730 −38.567 1.00 0.00 H
ATOM 825 HB3 ARG A 53 29.404 −32.989 −38.678 1.00 0.00 H
ATOM 826 HG2 ARG A 53 27.629 −33.950 −37.139 1.00 0.00 H
ATOM 827 HG3 ARG A 53 28.891 −34.532 −36.077 1.00 0.00 H
ATOM 828 HD2 ARG A 53 29.647 −32.139 −35.769 1.00 0.00 H
ATOM 829 HD3 ARG A 53 28.023 −32.487 −35.170 1.00 0.00 H
ATOM 830 HE ARG A 53 27.794 −31.610 −37.868 1.00 0.00 H
ATOM 831 HH11 ARG A 53 27.018 −29.651 −38.389 1.00 0.00 H
ATOM 832 HH12 ARG A 53 27.295 −28.313 −37.259 1.00 0.00 H
ATOM 833 HH21 ARG A 53 28.509 −30.102 −34.712 1.00 0.00 H
ATOM 834 HH22 ARG A 53 28.038 −28.542 −35.384 1.00 0.00 H
ATOM 835 N ILE A 54 31.771 −34.554 −35.426 1.00 0.00 N
ATOM 836 CA ILE A 54 31.750 −35.456 −34.264 1.00 0.00 C
ATOM 837 C ILE A 54 30.752 −34.940 −33.232 1.00 0.00 C
ATOM 838 O ILE A 54 30.565 −33.728 −33.090 1.00 0.00 O
ATOM 839 CB ILE A 54 33.154 −35.736 −33.671 1.00 0.00 C
ATOM 840 CG1 ILE A 54 33.655 −34.642 −32.699 1.00 0.00 C
ATOM 841 CG2 ILE A 54 34.173 −36.033 −34.788 1.00 0.00 C

ATOM 842 CD1 ILE A 54 34.960 −35.011 −31.982 1.00 0.00 C
ATOM 843 H ILE A 54 31.884 −33.560 −35.253 1.00 0.00 H
ATOM 844 HA ILE A 54 31.375 −36.428 −34.589 1.00 0.00 H
ATOM 845 HB ILE A 54 33.054 −36.652 −33.084 1.00 0.00 H
ATOM 846 HG12 ILE A 54 32.912 −34.477 −31.918 1.00 0.00 H
ATOM 847 HG13 ILE A 54 33.786 −33.706 −33.240 1.00 0.00 H
ATOM 848 HG21 ILE A 54 35.075 −36.482 −34.372 1.00 0.00 H
ATOM 849 HG22 ILE A 54 34.437 −35.118 −35.316 1.00 0.00 H
ATOM 850 HG23 ILE A 54 33.748 −36.747 −35.495 1.00 0.00 H
ATOM 851 HD11 ILE A 54 34.847 −35.971 −31.477 1.00 0.00 H
ATOM 852 HD12 ILE A 54 35.193 −34.251 −31.239 1.00 0.00 H
ATOM 853 HD13 ILE A 54 35.784 −35.068 −32.692 1.00 0.00 H
ATOM 854 N ILE A 55 30.161 −35.864 −32.481 1.00 0.00 N
ATOM 855 CA ILE A 55 29.270 −35.602 −31.349 1.00 0.00 C
ATOM 856 C ILE A 55 29.651 −36.583 −30.237 1.00 0.00 C
ATOM 857 O ILE A 55 29.774 −37.775 −30.503 1.00 0.00 O
ATOM 858 CB ILE A 55 27.786 −35.752 −31.762 1.00 0.00 C
ATOM 859 CG1 ILE A 55 27.451 −35.005 −33.078 1.00 0.00 C
ATOM 860 CG2 ILE A 55 26.899 −35.273 −30.600 1.00 0.00 C
ATOM 861 CD1 ILE A 55 25.974 −35.032 −33.482 1.00 0.00 C
ATOM 862 H ILE A 55 30.328 −36.843 −32.708 1.00 0.00 H
ATOM 863 HA ILE A 55 29.433 −34.586 −30.993 1.00 0.00 H
ATOM 864 HB ILE A 55 27.583 −36.813 −31.926 1.00 0.00 H
ATOM 865 HG12 ILE A 55 28.007 −35.468 −33.894 1.00 0.00 H
ATOM 866 HG13 ILE A 55 27.764 −33.965 −33.001 1.00 0.00 H
ATOM 867 HD11 ILE A 55 25.867 −34.639 −34.494 1.00 0.00 H
ATOM 868 HD12 ILE A 55 25.603 −36.054 −33.453 1.00 0.00 H
ATOM 869 HD13 ILE A 55 25.387 −34.411 −32.805 1.00 0.00 H
ATOM 870 HG21 ILE A 55 27.131 −35.822 −29.687 1.00 0.00 H
ATOM 871 HG22 ILE A 55 25.855 −35.461 −30.840 1.00 0.00 H

ATOM 872 HG23 ILE A 55 27.050 −34.208 −30.425 1.00 0.00 H

ATOM 873 N GLY A 56 29.941 −36.108 −29.029 1.00 0.00 N

ATOM 874 CA GLY A 56 30.489 −36.947 −27.959 1.00 0.00 C

ATOM 875 C GLY A 56 30.115 −36.481 −26.558 1.00 0.00 C

ATOM 876 O GLY A 56 29.592 −35.380 −26.386 1.00 0.00 O

ATOM 877 H GLY A 56 29.799 −35.120 −28.836 1.00 0.00 H

ATOM 878 HA2 GLY A 56 31.574 −36.976 −28.033 1.00 0.00 H

ATOM 879 HA3 GLY A 56 30.113 −37.963 −28.070 1.00 0.00 H

ATOM 880 N VAL A 57 30.293 −37.367 −25.581 1.00 0.00 N

ATOM 881 CA VAL A 57 29.871 −37.190 −24.183 1.00 0.00 C

ATOM 882 C VAL A 57 30.884 −37.811 −23.218 1.00 0.00 C

ATOM 883 O VAL A 57 31.718 −38.636 −23.611 1.00 0.00 O

ATOM 884 CB VAL A 57 28.455 −37.762 −23.925 1.00 0.00 C

ATOM 885 CG1 VAL A 57 27.394 −37.155 −24.847 1.00 0.00 C

ATOM 886 CG2 VAL A 57 28.369 −39.280 −24.089 1.00 0.00 C

ATOM 887 H VAL A 57 30.743 −38.249 −25.813 1.00 0.00 H

ATOM 888 HA VAL A 57 29.846 −36.124 −23.967 1.00 0.00 H

ATOM 889 HB VAL A 57 28.169 −37.529 −22.898 1.00 0.00 H

ATOM 890 HG21 VAL A 57 29.077 −39.771 −23.422 1.00 0.00 H

ATOM 891 HG22 VAL A 57 27.368 −39.625 −23.831 1.00 0.00 H

ATOM 892 HG23 VAL A 57 28.588 −39.545 −25.122 1.00 0.00 H

ATOM 893 HG11 VAL A 57 27.337 −36.083 −24.677 1.00 0.00 H

ATOM 894 HG12 VAL A 57 27.634 −37.345 −25.889 1.00 0.00 H

ATOM 895 HG13 VAL A 57 26.430 −37.606 −24.636 1.00 0.00 H

ATOM 896 N GLY A 58 30.778 −37.455 −21.939 1.00 0.00 N

ATOM 897 CA GLY A 58 31.604 −38.039 −20.889 1.00 0.00 C

ATOM 898 C GLY A 58 31.428 −37.387 −19.522 1.00 0.00 C

ATOM 899 O GLY A 58 30.374 −36.828 −19.220 1.00 0.00 O

ATOM 900 H GLY A 58 30.100 −36.746 −21.679 1.00 0.00 H

ATOM 901 HA2 GLY A 58 31.372 −39.098 −20.788 1.00 0.00 H

ATOM 902 HA3 GLY A 58 32.642 −37.944 −21.189 1.00 0.00 H
ATOM 903 N GLU A 59 32.484 −37.431 −18.709 1.00 0.00 N
ATOM 904 CA GLU A 59 32.478 −37.059 −17.286 1.00 0.00 C
ATOM 905 C GLU A 59 33.516 −35.947 −16.996 1.00 0.00 C
ATOM 906 O GLU A 59 34.571 −35.895 −17.633 1.00 0.00 O
ATOM 907 CB GLU A 59 32.691 −38.324 −16.420 1.00 0.00 C
ATOM 908 CG GLU A 59 31.831 −39.527 −16.879 1.00 0.00 C
ATOM 909 CD GLU A 59 31.691 −40.658 −15.847 1.00 0.00 C
ATOM 910 OE1 GLU A 59 32.668 −40.913 −15.109 1.00 0.00 O
ATOM 911 OE2 GLU A 59 30.652 −41.363 −15.901 1.00 0.00 O
ATOM 912 H GLU A 59 33.330 −37.866 −19.064 1.00 0.00 H
ATOM 913 HA GLU A 59 31.497 −36.661 −17.025 1.00 0.00 H
ATOM 914 HB2 GLU A 59 33.742 −38.614 −16.457 1.00 0.00 H
ATOM 915 HB3 GLU A 59 32.436 −38.072 −15.390 1.00 0.00 H
ATOM 916 HG2 GLU A 59 30.836 −39.163 −17.128 1.00 0.00 H
ATOM 917 HG3 GLU A 59 32.264 −39.943 −17.792 1.00 0.00 H
ATOM 918 N VAL A 60 33.189 −34.980 −16.124 1.00 0.00 N
ATOM 919 CA VAL A 60 33.978 −33.742 −15.898 1.00 0.00 C
ATOM 920 C VAL A 60 34.874 −33.844 −14.647 1.00 0.00 C
ATOM 921 O VAL A 60 34.716 −33.103 −13.677 1.00 0.00 O
ATOM 922 CB VAL A 60 33.088 −32.474 −15.905 1.00 0.00 C
ATOM 923 CG1 VAL A 60 33.923 −31.183 −15.995 1.00 0.00 C
ATOM 924 CG2 VAL A 60 32.144 −32.432 −17.114 1.00 0.00 C
ATOM 925 H VAL A 60 32.287 −35.044 −15.658 1.00 0.00 H
ATOM 926 HA VAL A 60 34.658 −33.630 −16.740 1.00 0.00 H
ATOM 927 HB VAL A 60 32.481 −32.447 −14.999 1.00 0.00 H
ATOM 928 HG11 VAL A 60 33.262 −30.317 −16.001 1.00 0.00 H
ATOM 929 HG12 VAL A 60 34.526 −31.188 −16.903 1.00 0.00 H
ATOM 930 HG13 VAL A 60 34.583 −31.098 −15.135 1.00 0.00 H
ATOM 931 HG21 VAL A 60 31.554 −31.515 −17.093 1.00 0.00 H

ATOM 932 HG22 VAL A 60 32.724 −32.465 −18.036 1.00 0.00 H
ATOM 933 HG23 VAL A 60 31.452 −33.273 −17.087 1.00 0.00 H
ATOM 934 N LEU A 61 35.668 −34.914 −14.577 1.00 0.00 N
ATOM 935 CA LEU A 61 36.376 −35.344 −13.362 1.00 0.00 C
ATOM 936 C LEU A 61 37.560 −34.429 −12.982 1.00 0.00 C
ATOM 937 O LEU A 61 38.222 −33.833 −13.839 1.00 0.00 O
ATOM 938 CB LEU A 61 36.828 −36.815 −13.502 1.00 0.00 C
ATOM 939 CG LEU A 61 35.766 −37.806 −14.024 1.00 0.00 C
ATOM 940 CD1 LEU A 61 36.388 −39.192 −14.190 1.00 0.00 C
ATOM 941 CD2 LEU A 61 34.568 −37.915 −13.081 1.00 0.00 C
ATOM 942 H LEU A 61 35.735 −35.486 −15.405 1.00 0.00 H
ATOM 943 HA LEU A 61 35.664 −35.294 −12.536 1.00 0.00 H
ATOM 944 HB2 LEU A 61 37.679 −36.846 −14.176 1.00 0.00 H
ATOM 945 HB3 LEU A 61 37.179 −37.160 −12.528 1.00 0.00 H
ATOM 946 HG LEU A 61 35.416 −37.483 −15.004 1.00 0.00 H
ATOM 947 HD11 LEU A 61 35.634 −39.879 −14.583 1.00 0.00 H
ATOM 948 HD12 LEU A 61 36.743 −39.563 −13.230 1.00 0.00 H
ATOM 949 HD13 LEU A 61 37.217 −39.142 −14.896 1.00 0.00 H
ATOM 950 HD21 LEU A 61 34.899 −38.199 −12.083 1.00 0.00 H
ATOM 951 HD22 LEU A 61 34.039 −36.964 −13.037 1.00 0.00 H
ATOM 952 HD23 LEU A 61 33.879 −38.676 −13.455 1.00 0.00 H
ATOM 953 N ASP A 62 37.947 −34.452 −11.706 1.00 0.00 N
ATOM 954 CA ASP A 62 39.197 −33.879 −11.194 1.00 0.00 C
ATOM 955 C ASP A 62 40.246 −34.977 −10.893 1.00 0.00 C
ATOM 956 O ASP A 62 40.281 −36.019 −11.554 1.00 0.00 O
ATOM 957 CB ASP A 62 38.898 −32.980 −9.981 1.00 0.00 C
ATOM 958 CG ASP A 62 38.368 −33.705 −8.737 1.00 0.00 C
ATOM 959 OD1 ASP A 62 38.344 −33.038 −7.682 1.00 0.00 O
ATOM 960 OD2 ASP A 62 38.142 −34.932 −8.817 1.00 0.00 O
ATOM 961 H ASP A 62 37.426 −35.013 −11.041 1.00 0.00 H

ATOM	962	HA	ASP	A	62	39.624	−33.219	−11.941	1.00	0.00	H
ATOM	963	HB2	ASP	A	62	39.807	−32.441	−9.713	1.00	0.00	H
ATOM	964	HB3	ASP	A	62	38.176	−32.227	−10.297	1.00	0.00	H
ATOM	965	N	ARG	A	63	41.282	−34.592	−10.139	1.00	0.00	N
ATOM	966	CA	ARG	A	63	42.199	−35.449	−9.365	1.00	0.00	C
ATOM	967	C	ARG	A	63	42.535	−34.676	−8.076	1.00	0.00	C
ATOM	968	O	ARG	A	63	42.035	−33.574	−7.877	1.00	0.00	O
ATOM	969	CB	ARG	A	63	43.478	−35.822	−10.155	1.00	0.00	C
ATOM	970	CG	ARG	A	63	43.325	−36.720	−11.398	1.00	0.00	C
ATOM	971	CD	ARG	A	63	43.109	−36.074	−12.781	1.00	0.00	C
ATOM	972	NE	ARG	A	63	43.115	−34.596	−12.795	1.00	0.00	N
ATOM	973	CZ	ARG	A	63	42.185	−33.804	−13.287	1.00	0.00	C
ATOM	974	NH1	ARG	A	63	41.069	−34.260	−13.779	1.00	0.00	N
ATOM	975	NH2	ARG	A	63	42.342	−32.523	−13.233	1.00	0.00	N
ATOM	976	H	ARG	A	63	41.182	−33.660	−9.752	1.00	0.00	H
ATOM	977	HA	ARG	A	63	41.664	−36.357	−9.078	1.00	0.00	H
ATOM	978	HB2	ARG	A	63	44.113	−36.398	−9.481	1.00	0.00	H
ATOM	979	HB3	ARG	A	63	44.040	−34.925	−10.398	1.00	0.00	H
ATOM	980	HG2	ARG	A	63	44.245	−37.302	−11.479	1.00	0.00	H
ATOM	981	HG3	ARG	A	63	42.528	−37.440	−11.216	1.00	0.00	H
ATOM	982	HD2	ARG	A	63	42.175	−36.461	−13.186	1.00	0.00	H
ATOM	983	HD3	ARG	A	63	43.903	−36.420	−13.445	1.00	0.00	H
ATOM	984	HE	ARG	A	63	43.900	−34.109	−12.353	1.00	0.00	H
ATOM	985	HH11	ARG	A	63	40.810	−35.185	−13.468	1.00	0.00	H
ATOM	986	HH12	ARG	A	63	40.301	−33.623	−13.952	1.00	0.00	H
ATOM	987	HH21	ARG	A	63	43.032	−32.184	−12.557	1.00	0.00	H
ATOM	988	HH22	ARG	A	63	41.523	−31.932	−13.333	1.00	0.00	H
ATOM	989	N	GLY	A	64	43.596	−35.063	−7.362	1.00	0.00	N
ATOM	990	CA	GLY	A	64	44.177	−34.264	−6.269	1.00	0.00	C
ATOM	991	C	GLY	A	64	44.691	−32.858	−6.646	1.00	0.00	C

ATOM	992	O	GLY	A	64	44.932	−32.060	−5.744	1.00	0.00	O
ATOM	993	H	GLY	A	64	44.027	−35.941	−7.599	1.00	0.00	H
ATOM	994	HA2	GLY	A	64	43.419	−34.136	−5.494	1.00	0.00	H
ATOM	995	HA3	GLY	A	64	45.013	−34.813	−5.838	1.00	0.00	H
ATOM	996	N	ASP	A	65	44.792	−32.517	−7.939	1.00	0.00	N
ATOM	997	CA	ASP	A	65	45.024	−31.142	−8.429	1.00	0.00	C
ATOM	998	C	ASP	A	65	43.776	−30.227	−8.348	1.00	0.00	C
ATOM	999	O	ASP	A	65	43.903	−28.999	−8.368	1.00	0.00	O
ATOM	1000	CB	ASP	A	65	45.556	−31.212	−9.873	1.00	0.00	C
ATOM	1001	CG	ASP	A	65	44.576	−31.843	−10.870	1.00	0.00	C
ATOM	1002	OD1	ASP	A	65	44.908	−32.874	−11.493	1.00	0.00	O
ATOM	1003	OD2	ASP	A	65	43.435	−31.355	−11.002	1.00	0.00	O
ATOM	1004	H	ASP	A	65	44.545	−33.209	−8.628	1.00	0.00	H
ATOM	1005	HA	ASP	A	65	45.793	−30.680	−7.810	1.00	0.00	H
ATOM	1006	HB2	ASP	A	65	45.792	−30.201	−10.209	1.00	0.00	H
ATOM	1007	HB3	ASP	A	65	46.486	−31.785	−9.872	1.00	0.00	H
ATOM	1008	N	LYS	A	66	42.592	−30.831	−8.185	1.00	0.00	N
ATOM	1009	CA	LYS	A	66	41.267	−30.221	−7.978	1.00	0.00	C
ATOM	1010	C	LYS	A	66	40.803	−29.293	−9.107	1.00	0.00	C
ATOM	1011	O	LYS	A	66	40.002	−28.384	−8.881	1.00	0.00	O
ATOM	1012	CB	LYS	A	66	41.139	−29.639	−6.559	1.00	0.00	C
ATOM	1013	CG	LYS	A	66	41.758	−30.592	−5.520	1.00	0.00	C
ATOM	1014	CD	LYS	A	66	41.206	−30.392	−4.115	1.00	0.00	C
ATOM	1015	CE	LYS	A	66	41.977	−31.308	−3.158	1.00	0.00	C
ATOM	1016	NZ	LYS	A	66	41.169	−31.616	−1.960	1.00	0.00	N
ATOM	1017	H	LYS	A	66	42.608	−31.843	−8.160	1.00	0.00	H
ATOM	1018	HA	LYS	A	66	40.554	−31.049	−8.011	1.00	0.00	H
ATOM	1019	HB2	LYS	A	66	41.645	−28.672	−6.506	1.00	0.00	H
ATOM	1020	HB3	LYS	A	66	40.081	−29.492	−6.341	1.00	0.00	H
ATOM	1021	HG2	LYS	A	66	42.837	−30.439	−5.503	1.00	0.00	H

ATOM	1022	HG3	LYS	A	66	41.551	−31.625	−5.808	1.00	0.00	H
ATOM	1023	HD2	LYS	A	66	41.310	−29.351	−3.806	1.00	0.00	H
ATOM	1024	HD3	LYS	A	66	40.148	−30.660	−4.137	1.00	0.00	H
ATOM	1025	HE2	LYS	A	66	42.910	−30.813	−2.883	1.00	0.00	H
ATOM	1026	HE3	LYS	A	66	42.221	−32.236	−3.679	1.00	0.00	H
ATOM	1027	HZ1	LYS	A	66	41.682	−31.991	−1.183	1.00	0.00	H
ATOM	1028	HZ2	LYS	A	66	40.404	−32.260	−2.193	1.00	0.00	H
ATOM	1029	HZ3	LYS	A	66	40.584	−30.823	−1.686	1.00	0.00	H
ATOM	1030	N	VAL	A	67	41.310	−29.508	−10.324	1.00	0.00	N
ATOM	1031	CA	VAL	A	67	40.852	−28.860	−11.563	1.00	0.00	C
ATOM	1032	C	VAL	A	67	39.850	−29.779	−12.285	1.00	0.00	C
ATOM	1033	O	VAL	A	67	40.228	−30.898	−12.642	1.00	0.00	O
ATOM	1034	CB	VAL	A	67	42.044	−28.531	−12.488	1.00	0.00	C
ATOM	1035	CG1	VAL	A	67	41.593	−27.774	−13.744	1.00	0.00	C
ATOM	1036	CG2	VAL	A	67	43.100	−27.665	−11.783	1.00	0.00	C
ATOM	1037	H	VAL	A	67	42.002	−30.252	−10.416	1.00	0.00	H
ATOM	1038	HA	VAL	A	67	40.371	−27.920	−11.307	1.00	0.00	H
ATOM	1039	HB	VAL	A	67	42.526	−29.456	−12.803	1.00	0.00	H
ATOM	1040	HG11	VAL	A	67	42.458	−27.519	−14.357	1.00	0.00	H
ATOM	1041	HG12	VAL	A	67	41.057	−26.866	−13.470	1.00	0.00	H
ATOM	1042	HG13	VAL	A	67	40.941	−28.408	−14.345	1.00	0.00	H
ATOM	1043	HG21	VAL	A	67	43.904	−27.427	−12.479	1.00	0.00	H
ATOM	1044	HG22	VAL	A	67	42.645	−26.752	−11.404	1.00	0.00	H
ATOM	1045	HG23	VAL	A	67	43.532	−28.223	−10.949	1.00	0.00	H
ATOM	1046	N	PRO	A	68	38.633	−29.325	−12.636	1.00	0.00	N
ATOM	1047	CA	PRO	A	68	37.737	−30.098	−13.493	1.00	0.00	C
ATOM	1048	C	PRO	A	68	38.318	−30.227	−14.910	1.00	0.00	C
ATOM	1049	O	PRO	A	68	38.679	−29.220	−15.524	1.00	0.00	O
ATOM	1050	CB	PRO	A	68	36.395	−29.355	−13.495	1.00	0.00	C
ATOM	1051	CG	PRO	A	68	36.513	−28.293	−12.401	1.00	0.00	C

ATOM 1052 CD PRO A 68 38.013 −28.077 −12.239 1.00 0.00 C

ATOM 1053 HA PRO A 68 37.596 −31.090 −13.062 1.00 0.00 H

ATOM 1054 HB2 PRO A 68 36.233 −28.867 −14.455 1.00 0.00 H

ATOM 1055 HB3 PRO A 68 35.575 −30.041 −13.282 1.00 0.00 H

ATOM 1056 HG2 PRO A 68 36.099 −28.678 −11.470 1.00 0.00 H

ATOM 1057 HG3 PRO A 68 36.007 −27.370 −12.685 1.00 0.00 H

ATOM 1058 HD2 PRO A 68 38.237 −27.834 −11.201 1.00 0.00 H

ATOM 1059 HD3 PRO A 68 38.350 −27.280 −12.904 1.00 0.00 H

ATOM 1060 N SER A 69 38.291 −31.436 −15.468 1.00 0.00 N

ATOM 1061 CA SER A 69 38.810 −31.764 −16.805 1.00 0.00 C

ATOM 1062 C SER A 69 37.825 −32.657 −17.558 1.00 0.00 C

ATOM 1063 O SER A 69 37.126 −33.456 −16.944 1.00 0.00 O

ATOM 1064 CB SER A 69 40.164 −32.477 −16.705 1.00 0.00 C

ATOM 1065 OG SER A 69 41.124 −31.676 −16.042 1.00 0.00 O

ATOM 1066 H SER A 69 37.953 −32.215 −14.909 1.00 0.00 H

ATOM 1067 HA SER A 69 38.953 −30.846 −17.375 1.00 0.00 H

ATOM 1068 HB2 SER A 69 40.033 −33.410 −16.159 1.00 0.00 H

ATOM 1069 HB3 SER A 69 40.525 −32.711 −17.708 1.00 0.00 H

ATOM 1070 HG SER A 69 41.895 −32.215 −15.835 1.00 0.00 H

ATOM 1071 N MET A 70 37.794 −32.584 −18.893 1.00 0.00 N

ATOM 1072 CA MET A 70 36.953 −33.487 −19.689 1.00 0.00 C

ATOM 1073 C MET A 70 37.548 −34.907 −19.730 1.00 0.00 C

ATOM 1074 O MET A 70 38.711 −35.080 −20.102 1.00 0.00 O

ATOM 1075 CB MET A 70 36.684 −32.908 −21.096 1.00 0.00 C

ATOM 1076 CG MET A 70 37.801 −33.133 −22.127 1.00 0.00 C

ATOM 1077 SD MET A 70 37.475 −32.468 −23.780 1.00 0.00 S

ATOM 1078 CE MET A 70 38.727 −33.401 −24.703 1.00 0.00 C

ATOM 1079 H MET A 70 38.431 −31.965 −19.371 1.00 0.00 H

ATOM 1080 HA MET A 70 35.984 −33.556 −19.189 1.00 0.00 H

ATOM 1081 HB2 MET A 70 35.782 −33.380 −21.485 1.00 0.00 H

ATOM	1082	HB3	MET	A	70	36.486	−31.840	−21.012	1.00	0.00	H
ATOM	1083	HG2	MET	A	70	37.941	−34.206	−22.247	1.00	0.00	H
ATOM	1084	HG3	MET	A	70	38.733	−32.706	−21.750	1.00	0.00	H
ATOM	1085	HE1	MET	A	70	38.688	−33.121	−25.755	1.00	0.00	H
ATOM	1086	HE2	MET	A	70	38.528	−34.469	−24.609	1.00	0.00	H
ATOM	1087	HE3	MET	A	70	39.718	−33.184	−24.303	1.00	0.00	H
ATOM	1088	N	PHE	A	71	36.694	−35.919	−19.593	1.00	0.00	N
ATOM	1089	CA	PHE	A	71	36.996	−37.318	−19.900	1.00	0.00	C
ATOM	1090	C	PHE	A	71	35.923	−37.860	−20.845	1.00	0.00	C
ATOM	1091	O	PHE	A	71	34.844	−38.256	−20.410	1.00	0.00	O
ATOM	1092	CB	PHE	A	71	37.115	−38.135	−18.601	1.00	0.00	C
ATOM	1093	CG	PHE	A	71	38.319	−37.751	−17.761	1.00	0.00	C
ATOM	1094	CD1	PHE	A	71	39.526	−38.465	−17.878	1.00	0.00	C
ATOM	1095	CD2	PHE	A	71	38.246	−36.649	−16.892	1.00	0.00	C
ATOM	1096	CE1	PHE	A	71	40.654	−38.077	−17.131	1.00	0.00	C
ATOM	1097	CE2	PHE	A	71	39.374	−36.256	−16.155	1.00	0.00	C
ATOM	1098	CZ	PHE	A	71	40.578	−36.968	−16.270	1.00	0.00	C
ATOM	1099	H	PHE	A	71	35.799	−35.736	−19.144	1.00	0.00	H
ATOM	1100	HA	PHE	A	71	37.956	−37.389	−20.412	1.00	0.00	H
ATOM	1101	HB2	PHE	A	71	37.194	−39.193	−18.858	1.00	0.00	H
ATOM	1102	HB3	PHE	A	71	36.209	−38.014	−18.006	1.00	0.00	H
ATOM	1103	HD1	PHE	A	71	39.590	−39.311	−18.546	1.00	0.00	H
ATOM	1104	HD2	PHE	A	71	37.322	−36.093	−16.800	1.00	0.00	H
ATOM	1105	HE1	PHE	A	71	41.578	−38.629	−17.223	1.00	0.00	H
ATOM	1106	HE2	PHE	A	71	39.297	−35.410	−15.492	1.00	0.00	H
ATOM	1107	HZ	PHE	A	71	41.444	−36.666	−15.698	1.00	0.00	H
ATOM	1108	N	MET	A	72	36.182	−37.772	−22.156	1.00	0.00	N
ATOM	1109	CA	MET	A	72	35.320	−38.366	−23.189	1.00	0.00	C
ATOM	1110	C	MET	A	72	35.211	−39.877	−22.971	1.00	0.00	C
ATOM	1111	O	MET	A	72	36.225	−40.572	−22.988	1.00	0.00	O

ATOM	1112	CB	MET	A	72	35.903	−38.118	−24.591	1.00	0.00	C
ATOM	1113	CG	MET	A	72	35.610	−36.727	−25.155	1.00	0.00	C
ATOM	1114	SD	MET	A	72	33.900	−36.526	−25.726	1.00	0.00	S
ATOM	1115	CE	MET	A	72	34.105	−35.118	−26.845	1.00	0.00	C
ATOM	1116	H	MET	A	72	37.090	−37.441	−22.432	1.00	0.00	H
ATOM	1117	HA	MET	A	72	34.319	−37.937	−23.128	1.00	0.00	H
ATOM	1118	HB2	MET	A	72	35.486	−38.850	−25.286	1.00	0.00	H
ATOM	1119	HB3	MET	A	72	36.983	−38.271	−24.559	1.00	0.00	H
ATOM	1120	HG2	MET	A	72	35.848	−35.962	−24.415	1.00	0.00	H
ATOM	1121	HG3	MET	A	72	36.267	−36.581	−26.012	1.00	0.00	H
ATOM	1122	HE1	MET	A	72	33.158	−34.925	−27.345	1.00	0.00	H
ATOM	1123	HE2	MET	A	72	34.410	−34.236	−26.281	1.00	0.00	H
ATOM	1124	HE3	MET	A	72	34.861	−35.352	−27.596	1.00	0.00	H
ATOM	1125	N	THR	A	73	33.985	−40.388	−22.892	1.00	0.00	N
ATOM	1126	CA	THR	A	73	33.703	−41.828	−22.770	1.00	0.00	C
ATOM	1127	C	THR	A	73	33.118	−42.423	−24.053	1.00	0.00	C
ATOM	1128	O	THR	A	73	33.195	−43.634	−24.259	1.00	0.00	O
ATOM	1129	CB	THR	A	73	32.724	−42.089	−21.619	1.00	0.00	C
ATOM	1130	OG1	THR	A	73	31.494	−41.478	−21.922	1.00	0.00	O
ATOM	1131	CG2	THR	A	73	33.207	−41.549	−20.275	1.00	0.00	C
ATOM	1132	H	THR	A	73	33.187	−39.756	−22.898	1.00	0.00	H
ATOM	1133	HA	THR	A	73	34.624	−42.369	−22.551	1.00	0.00	H
ATOM	1134	HB	THR	A	73	32.587	−43.168	−21.532	1.00	0.00	H
ATOM	1135	HG1	THR	A	73	30.965	−41.424	−21.101	1.00	0.00	H
ATOM	1136	HG21	THR	A	73	34.183	−41.972	−20.046	1.00	0.00	H
ATOM	1137	HG22	THR	A	73	32.517	−41.855	−19.491	1.00	0.00	H
ATOM	1138	HG23	THR	A	73	33.283	−40.460	−20.285	1.00	0.00	H
ATOM	1139	N	ASN	A	74	32.483	−41.597	−24.893	1.00	0.00	N
ATOM	1140	CA	ASN	A	74	31.578	−42.048	−25.945	1.00	0.00	C
ATOM	1141	C	ASN	A	74	31.463	−40.993	−27.060	1.00	0.00	C

ATOM 1142 O ASN A 74 31.212 −39.822 −26.778 1.00 0.00 O
ATOM 1143 CB ASN A 74 30.227 −42.298 −25.260 1.00 0.00 C
ATOM 1144 CG ASN A 74 29.172 −42.951 −26.120 1.00 0.00 C
ATOM 1145 OD1 ASN A 74 29.389 −43.366 −27.247 1.00 0.00 O
ATOM 1146 ND2 ASN A 74 28.011 −43.150 −25.556 1.00 0.00 N
ATOM 1147 H ASN A 74 32.390 −40.631 −24.598 1.00 0.00 H
ATOM 1148 HA ASN A 74 31.949 −42.983 −26.369 1.00 0.00 H
ATOM 1149 HB2 ASN A 74 30.367 −42.925 −24.384 1.00 0.00 H
ATOM 1150 HB3 ASN A 74 29.841 −41.352 −24.901 1.00 0.00 H
ATOM 1151 HD21 ASN A 74 27.888 −42.863 −24.588 1.00 0.00 H
ATOM 1152 HD22 ASN A 74 27.297 −43.655 −26.062 1.00 0.00 H
ATOM 1153 N VAL A 75 31.651 −41.390 −28.325 1.00 0.00 N
ATOM 1154 CA VAL A 75 31.692 −40.484 −29.489 1.00 0.00 C
ATOM 1155 C VAL A 75 31.021 −41.130 −30.705 1.00 0.00 C
ATOM 1156 O VAL A 75 31.187 −42.324 −30.954 1.00 0.00 O
ATOM 1157 CB VAL A 75 33.138 −40.041 −29.824 1.00 0.00 C
ATOM 1158 CG1 VAL A 75 33.187 −39.020 −30.972 1.00 0.00 C
ATOM 1159 CG2 VAL A 75 33.861 −39.399 −28.628 1.00 0.00 C
ATOM 1160 H VAL A 75 31.775 −42.376 −28.507 1.00 0.00 H
ATOM 1161 HA VAL A 75 31.122 −39.589 −29.247 1.00 0.00 H
ATOM 1162 HB VAL A 75 33.709 −40.920 −30.126 1.00 0.00 H
ATOM 1163 HG11 VAL A 75 34.213 −38.694 −31.137 1.00 0.00 H
ATOM 1164 HG12 VAL A 75 32.835 −39.471 −31.899 1.00 0.00 H
ATOM 1165 HG13 VAL A 75 32.569 −38.154 −30.731 1.00 0.00 H
ATOM 1166 HG21 VAL A 75 34.853 −39.060 −28.926 1.00 0.00 H
ATOM 1167 HG22 VAL A 75 33.289 −38.551 −28.251 1.00 0.00 H
ATOM 1168 HG23 VAL A 75 33.989 −40.127 −27.828 1.00 0.00 H
ATOM 1169 N TRP A 76 30.305 −40.329 −31.493 1.00 0.00 N
ATOM 1170 CA TRP A 76 29.620 −40.710 −32.727 1.00 0.00 C
ATOM 1171 C TRP A 76 29.911 −39.713 −33.860 1.00 0.00 C

ATOM 1172 O TRP A 76 30.058 −38.510 −33.629 1.00 0.00 O
ATOM 1173 CB TRP A 76 28.120 −40.845 −32.440 1.00 0.00 C
ATOM 1174 CG TRP A 76 27.261 −41.178 −33.622 1.00 0.00 C
ATOM 1175 CD1 TRP A 76 27.079 −42.417 −34.126 1.00 0.00 C
ATOM 1176 CD2 TRP A 76 26.498 −40.280 −34.488 1.00 0.00 C
ATOM 1177 NE1 TRP A 76 26.255 −42.355 −35.232 1.00 0.00 N
ATOM 1178 CE2 TRP A 76 25.853 −41.063 −35.492 1.00 0.00 C
ATOM 1179 CE3 TRP A 76 26.269 −38.888 −34.515 1.00 0.00 C
ATOM 1180 CZ2 TRP A 76 25.015 −40.499 −36.466 1.00 0.00 C
ATOM 1181 CZ3 TRP A 76 25.418 −38.312 −35.478 1.00 0.00 C
ATOM 1182 CH2 TRP A 76 24.795 −39.112 −36.454 1.00 0.00 C
ATOM 1183 H TRP A 76 30.218 −39.356 −31.219 1.00 0.00 H
ATOM 1184 HA TRP A 76 29.987 −41.684 −33.050 1.00 0.00 H
ATOM 1185 HB2 TRP A 76 27.979 −41.617 −31.683 1.00 0.00 H
ATOM 1186 HB3 TRP A 76 27.763 −39.905 −32.017 1.00 0.00 H
ATOM 1187 HD1 TRP A 76 27.517 −43.322 −33.716 1.00 0.00 H
ATOM 1188 HE3 TRP A 76 26.745 −38.267 −33.771 1.00 0.00 H
ATOM 1189 HZ2 TRP A 76 24.538 −41.127 −37.203 1.00 0.00 H
ATOM 1190 HZ3 TRP A 76 25.234 −37.247 −35.465 1.00 0.00 H
ATOM 1191 HH2 TRP A 76 24.147 −38.660 −37.192 1.00 0.00 H
ATOM 1192 HE1 TRP A 76 26.002 −43.167 −35.775 1.00 0.00 H
ATOM 1193 N THR A 77 29.888 −40.215 −35.096 1.00 0.00 N
ATOM 1194 CA THR A 77 30.163 −39.469 −36.333 1.00 0.00 C
ATOM 1195 C THR A 77 29.091 −39.823 −37.373 1.00 0.00 C
ATOM 1196 O THR A 77 28.882 −41.013 −37.620 1.00 0.00 O
ATOM 1197 CB THR A 77 31.572 −39.809 −36.853 1.00 0.00 C
ATOM 1198 OG1 THR A 77 32.533 −39.288 −35.964 1.00 0.00 O
ATOM 1199 CG2 THR A 77 31.900 −39.246 −38.236 1.00 0.00 C
ATOM 1200 H THR A 77 29.656 −41.191 −35.202 1.00 0.00 H
ATOM 1201 HA THR A 77 30.150 −38.404 −36.123 1.00 0.00 H

ATOM 1202 HB THR A 77 31.685 −40.894 −36.892 1.00 0.00 H
ATOM 1203 HG1 THR A 77 33.399 −39.394 −36.366 1.00 0.00 H
ATOM 1204 HG21 THR A 77 32.943 −39.449 −38.479 1.00 0.00 H
ATOM 1205 HG22 THR A 77 31.275 −39.723 −38.991 1.00 0.00 H
ATOM 1206 HG23 THR A 77 31.731 −38.172 −38.257 1.00 0.00 H
ATOM 1207 N PRO A 78 28.410 −38.850 −38.013 1.00 0.00 N
ATOM 1208 CA PRO A 78 27.413 −39.143 −39.041 1.00 0.00 C
ATOM 1209 C PRO A 78 28.034 −39.780 −40.298 1.00 0.00 C
ATOM 1210 O PRO A 78 29.108 −39.345 −40.715 1.00 0.00 O
ATOM 1211 CB PRO A 78 26.752 −37.807 −39.395 1.00 0.00 C
ATOM 1212 CG PRO A 78 27.151 −36.851 −38.277 1.00 0.00 C
ATOM 1213 CD PRO A 78 28.472 −37.420 −37.769 1.00 0.00 C
ATOM 1214 HA PRO A 78 26.674 −39.813 −38.604 1.00 0.00 H
ATOM 1215 HB2 PRO A 78 25.669 −37.904 −39.471 1.00 0.00 H
ATOM 1216 HB3 PRO A 78 27.160 −37.431 −40.330 1.00 0.00 H
ATOM 1217 HG2 PRO A 78 27.265 −35.834 −38.650 1.00 0.00 H
ATOM 1218 HG3 PRO A 78 26.407 −36.877 −37.485 1.00 0.00 H
ATOM 1219 HD2 PRO A 78 28.585 −37.186 −36.710 1.00 0.00 H
ATOM 1220 HD3 PRO A 78 29.297 −37.007 −38.348 1.00 0.00 H
ATOM 1221 N PRO A 79 27.249 −40.545 −41.085 1.00 0.00 N
ATOM 1222 CA PRO A 79 27.665 −41.049 −42.401 1.00 0.00 C
ATOM 1223 C PRO A 79 27.792 −39.964 −43.491 1.00 0.00 C
ATOM 1224 O PRO A 79 28.243 −40.258 −44.595 1.00 0.00 O
ATOM 1225 CB PRO A 79 26.621 −42.110 −42.768 1.00 0.00 C
ATOM 1226 CG PRO A 79 25.361 −41.621 −42.057 1.00 0.00 C
ATOM 1227 CD PRO A 79 25.912 −41.025 −40.763 1.00 0.00 C
ATOM 1228 HA PRO A 79 28.640 −41.531 −42.310 1.00 0.00 H
ATOM 1229 HB2 PRO A 79 26.923 −43.072 −42.351 1.00 0.00 H
ATOM 1230 HB3 PRO A 79 26.472 −42.197 −43.845 1.00 0.00 H
ATOM 1231 HG2 PRO A 79 24.884 −40.839 −42.649 1.00 0.00 H

ATOM	1232	HG3	PRO	A	79	24.663	−42.437	−41.861	1.00	0.00	H
ATOM	1233	HD2	PRO	A	79	25.984	−41.802	−39.999	1.00	0.00	H
ATOM	1234	HD3	PRO	A	79	25.264	−40.218	−40.419	1.00	0.00	H
ATOM	1235	N	ASN	A	80	27.436	−38.707	−43.199	1.00	0.00	N
ATOM	1236	CA	ASN	A	80	27.847	−37.546	−43.987	1.00	0.00	C
ATOM	1237	C	ASN	A	80	28.017	−36.308	−43.075	1.00	0.00	C
ATOM	1238	O	ASN	A	80	27.078	−35.952	−42.360	1.00	0.00	O
ATOM	1239	CB	ASN	A	80	26.834	−37.295	−45.115	1.00	0.00	C
ATOM	1240	CG	ASN	A	80	27.359	−36.266	−46.097	1.00	0.00	C
ATOM	1241	OD1	ASN	A	80	27.651	−35.133	−45.750	1.00	0.00	O
ATOM	1242	ND2	ASN	A	80	27.756	−36.689	−47.273	1.00	0.00	N
ATOM	1243	H	ASN	A	80	27.096	−38.523	−42.269	1.00	0.00	H
ATOM	1244	HA	ASN	A	80	28.803	−37.788	−44.451	1.00	0.00	H
ATOM	1245	HB2	ASN	A	80	26.638	−38.228	−45.643	1.00	0.00	H
ATOM	1246	HB3	ASN	A	80	25.893	−36.935	−44.700	1.00	0.00	H
ATOM	1247	HD21	ASN	A	80	27.652	−37.655	−47.524	1.00	0.00	H
ATOM	1248	HD22	ASN	A	80	28.212	−36.014	−47.861	1.00	0.00	H
ATOM	1249	N	PRO	A	81	29.185	−35.636	−43.073	1.00	0.00	N
ATOM	1250	CA	PRO	A	81	29.442	−34.505	−42.182	1.00	0.00	C
ATOM	1251	C	PRO	A	81	28.842	−33.176	−42.669	1.00	0.00	C
ATOM	1252	O	PRO	A	81	28.701	−32.248	−41.878	1.00	0.00	O
ATOM	1253	CB	PRO	A	81	30.966	−34.435	−42.125	1.00	0.00	C
ATOM	1254	CG	PRO	A	81	31.396	−34.856	−43.528	1.00	0.00	C
ATOM	1255	CD	PRO	A	81	30.382	−35.955	−43.846	1.00	0.00	C
ATOM	1256	HA	PRO	A	81	29.050	−34.711	−41.185	1.00	0.00	H
ATOM	1257	HB2	PRO	A	81	31.305	−35.187	−41.416	1.00	0.00	H
ATOM	1258	HB3	PRO	A	81	31.335	−33.447	−41.851	1.00	0.00	H
ATOM	1259	HG2	PRO	A	81	31.279	−34.026	−44.224	1.00	0.00	H
ATOM	1260	HG3	PRO	A	81	32.418	−35.233	−43.547	1.00	0.00	H
ATOM	1261	HD2	PRO	A	81	30.777	−36.920	−43.522	1.00	0.00	H

ATOM 1262 HD3 PRO A 81 30.174 −35.972 −44.917 1.00 0.00 H
ATOM 1263 N SER A 82 28.540 −33.051 −43.965 1.00 0.00 N
ATOM 1264 CA SER A 82 28.065 −31.816 −44.599 1.00 0.00 C
ATOM 1265 C SER A 82 26.572 −31.547 −44.395 1.00 0.00 C
ATOM 1266 O SER A 82 26.124 −30.445 −44.696 1.00 0.00 O
ATOM 1267 CB SER A 82 28.344 −31.869 −46.106 1.00 0.00 C
ATOM 1268 OG SER A 82 29.722 −32.086 −46.353 1.00 0.00 O
ATOM 1269 H SER A 82 28.644 −33.861 −44.569 1.00 0.00 H
ATOM 1270 HA SER A 82 28.606 −30.970 −44.175 1.00 0.00 H
ATOM 1271 HB2 SER A 82 28.038 −30.929 −46.568 1.00 0.00 H
ATOM 1272 HB3 SER A 82 27.768 −32.679 −46.554 1.00 0.00 H
ATOM 1273 HG SER A 82 30.202 −31.285 −46.139 1.00 0.00 H
ATOM 1274 N THR A 83 25.807 −32.544 −43.940 1.00 0.00 N
ATOM 1275 CA THR A 83 24.331 −32.536 −43.904 1.00 0.00 C
ATOM 1276 C THR A 83 23.748 −32.495 −42.483 1.00 0.00 C
ATOM 1277 O THR A 83 22.591 −32.871 −42.299 1.00 0.00 O
ATOM 1278 CB THR A 83 23.777 −33.749 −44.679 1.00 0.00 C
ATOM 1279 OG1 THR A 83 24.192 −34.950 −44.067 1.00 0.00 O
ATOM 1280 CG2 THR A 83 24.235 −33.792 −46.137 1.00 0.00 C
ATOM 1281 H THR A 83 26.250 −33.443 −43.801 1.00 0.00 H
ATOM 1282 HA THR A 83 23.966 −31.639 −44.405 1.00 0.00 H
ATOM 1283 HB THR A 83 22.688 −33.719 −44.667 1.00 0.00 H
ATOM 1284 HG21 THR A 83 23.740 −34.620 −46.644 1.00 0.00 H
ATOM 1285 HG22 THR A 83 25.313 −33.932 −46.196 1.00 0.00 H
ATOM 1286 HG23 THR A 83 23.966 −32.859 −46.633 1.00 0.00 H
ATOM 1287 HG1 THR A 83 23.721 −35.017 −43.231 1.00 0.00 H
ATOM 1288 N ILE A 84 24.568 −32.200 −41.464 1.00 0.00 N
ATOM 1289 CA ILE A 84 24.195 −32.190 −40.036 1.00 0.00 C
ATOM 1290 C ILE A 84 24.608 −30.855 −39.401 1.00 0.00 C
ATOM 1291 O ILE A 84 25.792 −30.503 −39.420 1.00 0.00 O

ATOM 1292 CB ILE A 84 24.820 −33.401 −39.299 1.00 0.00 C
ATOM 1293 CG1 ILE A 84 24.274 −34.758 −39.803 1.00 0.00 C
ATOM 1294 CG2 ILE A 84 24.653 −33.303 −37.770 1.00 0.00 C
ATOM 1295 CD1 ILE A 84 22.788 −35.029 −39.526 1.00 0.00 C
ATOM 1296 H ILE A 84 25.499 −31.887 −41.700 1.00 0.00 H
ATOM 1297 HA ILE A 84 23.113 −32.261 −39.952 1.00 0.00 H
ATOM 1298 HB ILE A 84 25.892 −33.394 −39.503 1.00 0.00 H
ATOM 1299 HG12 ILE A 84 24.846 −35.554 −39.329 1.00 0.00 H
ATOM 1300 HG13 ILE A 84 24.449 −34.840 −40.876 1.00 0.00 H
ATOM 1301 HG21 ILE A 84 25.232 −32.469 −37.371 1.00 0.00 H
ATOM 1302 HG22 ILE A 84 23.604 −33.154 −37.515 1.00 0.00 H
ATOM 1303 HG23 ILE A 84 25.006 −34.218 −37.294 1.00 0.00 H
ATOM 1304 HD11 ILE A 84 22.513 −35.993 −39.954 1.00 0.00 H
ATOM 1305 HD12 ILE A 84 22.171 −34.255 −39.981 1.00 0.00 H
ATOM 1306 HD13 ILE A 84 22.598 −35.060 −38.453 1.00 0.00 H
ATOM 1307 N HIS A 85 23.664 −30.163 −38.755 1.00 0.00 N
ATOM 1308 CA HIS A 85 23.795 −28.751 −38.366 1.00 0.00 C
ATOM 1309 C HIS A 85 23.184 −28.450 −36.986 1.00 0.00 C
ATOM 1310 O HIS A 85 22.183 −29.059 −36.604 1.00 0.00 O
ATOM 1311 CB HIS A 85 23.124 −27.860 −39.432 1.00 0.00 C
ATOM 1312 CG HIS A 85 23.448 −28.223 −40.858 1.00 0.00 C
ATOM 1313 ND1 HIS A 85 24.583 −27.875 −41.551 1.00 0.00 N
ATOM 1314 CD2 HIS A 85 22.744 −29.094 −41.645 1.00 0.00 C
ATOM 1315 CE1 HIS A 85 24.558 −28.511 −42.733 1.00 0.00 C
ATOM 1316 NE2 HIS A 85 23.461 −29.275 −42.830 1.00 0.00 N
ATOM 1317 H HIS A 85 22.721 −30.544 −38.758 1.00 0.00 H
ATOM 1318 HA HIS A 85 24.851 −28.486 −38.326 1.00 0.00 H
ATOM 1319 HB2 HIS A 85 22.043 −27.908 −39.311 1.00 0.00 H
ATOM 1320 HB3 HIS A 85 23.430 −26.828 −39.265 1.00 0.00 H
ATOM 1321 HD2 HIS A 85 21.832 −29.605 −41.366 1.00 0.00 H

ATOM 1322 HE1 HIS A 85 25.318 −28.433 −43.500 1.00 0.00 H
ATOM 1323 HD1 HIS A 85 25.298 −27.238 −41.235 1.00 0.00 H
ATOM 1324 N HIS A 86 23.709 −27.410 −36.324 1.00 0.00 N
ATOM 1325 CA HIS A 86 23.065 −26.677 −35.219 1.00 0.00 C
ATOM 1326 C HIS A 86 22.537 −27.561 −34.072 1.00 0.00 C
ATOM 1327 O HIS A 86 21.420 −27.382 −33.588 1.00 0.00 O
ATOM 1328 CB HIS A 86 22.010 −25.694 −35.756 1.00 0.00 C
ATOM 1329 CG HIS A 86 22.453 −24.893 −36.956 1.00 0.00 C
ATOM 1330 ND1 HIS A 86 23.614 −24.157 −37.090 1.00 0.00 N
ATOM 1331 CD2 HIS A 86 21.812 −24.856 −38.164 1.00 0.00 C
ATOM 1332 CE1 HIS A 86 23.649 −23.667 −38.343 1.00 0.00 C
ATOM 1333 NE2 HIS A 86 22.577 −24.080 −39.036 1.00 0.00 N
ATOM 1334 H HIS A 86 24.543 −26.980 −36.704 1.00 0.00 H
ATOM 1335 HA HIS A 86 23.850 −26.068 −34.769 1.00 0.00 H
ATOM 1336 HB2 HIS A 86 21.117 −26.258 −36.032 1.00 0.00 H
ATOM 1337 HB3 HIS A 86 21.729 −25.004 −34.960 1.00 0.00 H
ATOM 1338 HD2 HIS A 86 20.892 −25.367 −38.409 1.00 0.00 H
ATOM 1339 HE1 HIS A 86 24.437 −23.046 −38.753 1.00 0.00 H
ATOM 1340 HD1 HIS A 86 24.350 −24.049 −36.403 1.00 0.00 H
ATOM 1341 N CYS A 87 23.316 −28.577 −33.692 1.00 0.00 N
ATOM 1342 CA CYS A 87 22.938 −29.526 −32.649 1.00 0.00 C
ATOM 1343 C CYS A 87 22.659 −28.844 −31.295 1.00 0.00 C
ATOM 1344 O CYS A 87 23.218 −27.791 −30.989 1.00 0.00 O
ATOM 1345 CB CYS A 87 24.023 −30.599 −32.526 1.00 0.00 C
ATOM 1346 SG CYS A 87 24.293 −31.608 −34.012 1.00 0.00 S
ATOM 1347 H CYS A 87 24.207 −28.690 −34.146 1.00 0.00 H
ATOM 1348 HA CYS A 87 22.022 −30.012 −32.969 1.00 0.00 H
ATOM 1349 HB2 CYS A 87 23.763 −31.270 −31.705 1.00 0.00 H
ATOM 1350 HB3 CYS A 87 24.961 −30.107 −32.267 1.00 0.00 H
ATOM 1351 N SER A 88 21.868 −29.506 −30.449 1.00 0.00 N

ATOM 1352 CA SER A 88 21.390 −28.989 −29.164 1.00 0.00 C
ATOM 1353 C SER A 88 21.182 −30.116 −28.155 1.00 0.00 C
ATOM 1354 O SER A 88 20.615 −31.137 −28.526 1.00 0.00 O
ATOM 1355 CB SER A 88 20.058 −28.282 −29.403 1.00 0.00 C
ATOM 1356 OG SER A 88 19.662 −27.658 −28.205 1.00 0.00 O
ATOM 1357 H SER A 88 21.394 −30.334 −30.803 1.00 0.00 H
ATOM 1358 HA SER A 88 22.121 −28.294 −28.760 1.00 0.00 H
ATOM 1359 HB2 SER A 88 20.176 −27.522 −30.177 1.00 0.00 H
ATOM 1360 HB3 SER A 88 19.301 −29.000 −29.711 1.00 0.00 H
ATOM 1361 HG SER A 88 20.285 −26.913 −28.103 1.00 0.00 H
ATOM 1362 N SER A 89 21.634 −29.979 −26.904 1.00 0.00 N
ATOM 1363 CA SER A 89 21.833 −31.136 −26.010 1.00 0.00 C
ATOM 1364 C SER A 89 21.270 −30.982 −24.589 1.00 0.00 C
ATOM 1365 O SER A 89 20.991 −29.875 −24.131 1.00 0.00 O
ATOM 1366 CB SER A 89 23.321 −31.493 −25.993 1.00 0.00 C
ATOM 1367 OG SER A 89 24.068 −30.503 −25.323 1.00 0.00 O
ATOM 1368 H SER A 89 22.062 −29.099 −26.636 1.00 0.00 H
ATOM 1369 HA SER A 89 21.318 −31.991 −26.438 1.00 0.00 H
ATOM 1370 HB2 SER A 89 23.459 −32.446 −25.482 1.00 0.00 H
ATOM 1371 HB3 SER A 89 23.684 −31.593 −27.016 1.00 0.00 H
ATOM 1372 HG SER A 89 23.939 −29.631 −25.743 1.00 0.00 H
ATOM 1373 N THR A 90 20.986 −32.108 −23.922 1.00 0.00 N
ATOM 1374 CA THR A 90 20.414 −32.200 −22.558 1.00 0.00 C
ATOM 1375 C THR A 90 20.697 −33.580 −21.939 1.00 0.00 C
ATOM 1376 O THR A 90 20.427 −34.594 −22.583 1.00 0.00 O
ATOM 1377 CB THR A 90 18.891 −31.980 −22.630 1.00 0.00 C
ATOM 1378 OG1 THR A 90 18.615 −30.636 −22.942 1.00 0.00 O
ATOM 1379 CG2 THR A 90 18.153 −32.261 −21.325 1.00 0.00 C
ATOM 1380 H THR A 90 21.140 −32.989 −24.406 1.00 0.00 H
ATOM 1381 HA THR A 90 20.850 −31.433 −21.915 1.00 0.00 H

ATOM 1382 HB THR A 90 18.473 −32.618 −23.410 1.00 0.00 H

ATOM 1383 HG1 THR A 90 19.369 −30.297 −23.452 1.00 0.00 H

ATOM 1384 HG21 THR A 90 18.583 −31.657 −20.526 1.00 0.00 H

ATOM 1385 HG22 THR A 90 18.222 −33.316 −21.064 1.00 0.00 H

ATOM 1386 HG23 THR A 90 17.100 −32.011 −21.456 1.00 0.00 H

ATOM 1387 N TYR A 91 21.145 −33.650 −20.677 1.00 0.00 N

ATOM 1388 CA TYR A 91 21.287 −34.919 −19.944 1.00 0.00 C

ATOM 1389 C TYR A 91 19.981 −35.370 −19.275 1.00 0.00 C

ATOM 1390 O TYR A 91 19.447 −34.636 −18.437 1.00 0.00 O

ATOM 1391 CB TYR A 91 22.385 −34.801 −18.881 1.00 0.00 C

ATOM 1392 CG TYR A 91 22.529 −36.011 −17.974 1.00 0.00 C

ATOM 1393 CD1 TYR A 91 23.097 −37.195 −18.477 1.00 0.00 C

ATOM 1394 CD2 TYR A 91 22.058 −35.969 −16.646 1.00 0.00 C

ATOM 1395 CE1 TYR A 91 23.179 −38.337 −17.660 1.00 0.00 C

ATOM 1396 CE2 TYR A 91 22.133 −37.110 −15.827 1.00 0.00 C

ATOM 1397 CZ TYR A 91 22.692 −38.299 −16.337 1.00 0.00 C

ATOM 1398 OH TYR A 91 22.755 −39.405 −15.553 1.00 0.00 O

ATOM 1399 H TYR A 91 21.417 −32.791 −20.195 1.00 0.00 H

ATOM 1400 HA TYR A 91 21.592 −35.697 −20.640 1.00 0.00 H

ATOM 1401 HB2 TYR A 91 22.147 −33.946 −18.261 1.00 0.00 H

ATOM 1402 HB3 TYR A 91 23.336 −34.614 −19.371 1.00 0.00 H

ATOM 1403 HD1 TYR A 91 23.459 −37.228 −19.496 1.00 0.00 H

ATOM 1404 HD2 TYR A 91 21.632 −35.066 −16.248 1.00 0.00 H

ATOM 1405 HE1 TYR A 91 23.618 −39.245 −18.038 1.00 0.00 H

ATOM 1406 HE2 TYR A 91 21.757 −37.080 −14.816 1.00 0.00 H

ATOM 1407 HH TYR A 91 22.729 −39.202 −14.618 1.00 0.00 H

ATOM 1408 N HIS A 92 19.656 −36.657 −19.398 1.00 0.00 N

ATOM 1409 CA HIS A 92 18.632 −37.365 −18.623 1.00 0.00 C

ATOM 1410 C HIS A 92 19.028 −38.845 −18.440 1.00 0.00 C

ATOM 1411 O HIS A 92 19.458 −39.490 −19.390 1.00 0.00 O

ATOM	1412	CB	HIS	A	92	17.262	−37.163	−19.304	1.00	0.00	C
ATOM	1413	CG	HIS	A	92	16.217	−38.180	−18.927	1.00	0.00	C
ATOM	1414	ND1	HIS	A	92	16.001	−39.401	−19.576	1.00	0.00	N
ATOM	1415	CD2	HIS	A	92	15.449	−38.127	−17.804	1.00	0.00	C
ATOM	1416	CE1	HIS	A	92	15.092	−40.046	−18.822	1.00	0.00	C
ATOM	1417	NE2	HIS	A	92	14.749	−39.308	−17.754	1.00	0.00	N
ATOM	1418	H	HIS	A	92	20.220	−37.236	−20.017	1.00	0.00	H
ATOM	1419	HA	HIS	A	92	18.572	−36.925	−17.630	1.00	0.00	H
ATOM	1420	HB2	HIS	A	92	16.889	−36.171	−19.051	1.00	0.00	H
ATOM	1421	HB3	HIS	A	92	17.380	−37.188	−20.384	1.00	0.00	H
ATOM	1422	HD2	HIS	A	92	15.440	−37.346	−17.059	1.00	0.00	H
ATOM	1423	HE1	HIS	A	92	14.743	−41.056	−18.995	1.00	0.00	H
ATOM	1424	HE2	HIS	A	92	14.274	−39.680	−16.923	1.00	0.00	H
ATOM	1425	N	GLU	A	93	19.054	−39.300	−17.181	1.00	0.00	N
ATOM	1426	CA	GLU	A	93	19.219	−40.703	−16.737	1.00	0.00	C
ATOM	1427	C	GLU	A	93	20.207	−41.561	−17.562	1.00	0.00	C
ATOM	1428	O	GLU	A	93	19.822	−42.365	−18.404	1.00	0.00	O
ATOM	1429	CB	GLU	A	93	17.835	−41.356	−16.555	1.00	0.00	C
ATOM	1430	CG	GLU	A	93	17.053	−40.651	−15.429	1.00	0.00	C
ATOM	1431	CD	GLU	A	93	15.624	−41.183	−15.266	1.00	0.00	C
ATOM	1432	OE1	GLU	A	93	15.458	−42.420	−15.209	1.00	0.00	O
ATOM	1433	OE2	GLU	A	93	14.707	−40.332	−15.183	1.00	0.00	O
ATOM	1434	H	GLU	A	93	18.656	−38.687	−16.491	1.00	0.00	H
ATOM	1435	HA	GLU	A	93	19.656	−40.670	−15.740	1.00	0.00	H
ATOM	1436	HB2	GLU	A	93	17.277	−41.300	−17.490	1.00	0.00	H
ATOM	1437	HB3	GLU	A	93	17.970	−42.404	−16.287	1.00	0.00	H
ATOM	1438	HG2	GLU	A	93	17.010	−39.577	−15.630	1.00	0.00	H
ATOM	1439	HG3	GLU	A	93	17.596	−40.782	−14.490	1.00	0.00	H
ATOM	1440	N	ASP	A	94	21.507	−41.383	−17.299	1.00	0.00	N
ATOM	1441	CA	ASP	A	94	22.657	−41.976	−18.005	1.00	0.00	C

ATOM	1442	C	ASP	A	94	22.823	−41.631	−19.496	1.00	0.00	C
ATOM	1443	O	ASP	A	94	23.880	−41.936	−20.052	1.00	0.00	O
ATOM	1444	CB	ASP	A	94	22.789	−43.492	−17.757	1.00	0.00	C
ATOM	1445	CG	ASP	A	94	23.340	−43.884	−16.386	1.00	0.00	C
ATOM	1446	OD1	ASP	A	94	24.017	−43.046	−15.742	1.00	0.00	O
ATOM	1447	OD2	ASP	A	94	23.561	−45.104	−16.227	1.00	0.00	O
ATOM	1448	H	ASP	A	94	21.744	−40.704	−16.588	1.00	0.00	H
ATOM	1449	HA	ASP	A	94	23.536	−41.525	−17.548	1.00	0.00	H
ATOM	1450	HB2	ASP	A	94	23.474	−43.906	−18.496	1.00	0.00	H
ATOM	1451	HB3	ASP	A	94	21.826	−43.980	−17.911	1.00	0.00	H
ATOM	1452	N	PHE	A	95	21.958	−40.813	−20.098	1.00	0.00	N
ATOM	1453	CA	PHE	A	95	22.097	−40.379	−21.491	1.00	0.00	C
ATOM	1454	C	PHE	A	95	22.221	−38.860	−21.615	1.00	0.00	C
ATOM	1455	O	PHE	A	95	21.340	−38.113	−21.195	1.00	0.00	O
ATOM	1456	CB	PHE	A	95	20.932	−40.910	−22.336	1.00	0.00	C
ATOM	1457	CG	PHE	A	95	20.602	−42.374	−22.111	1.00	0.00	C
ATOM	1458	CD1	PHE	A	95	21.484	−43.379	−22.547	1.00	0.00	C
ATOM	1459	CD2	PHE	A	95	19.443	−42.725	−21.395	1.00	0.00	C
ATOM	1460	CE1	PHE	A	95	21.209	−44.728	−22.267	1.00	0.00	C
ATOM	1461	CE2	PHE	A	95	19.175	−44.073	−21.102	1.00	0.00	C
ATOM	1462	CZ	PHE	A	95	20.058	−45.076	−21.538	1.00	0.00	C
ATOM	1463	H	PHE	A	95	21.096	−40.573	−19.613	1.00	0.00	H
ATOM	1464	HA	PHE	A	95	23.004	−40.810	−21.909	1.00	0.00	H
ATOM	1465	HB2	PHE	A	95	20.048	−40.322	−22.102	1.00	0.00	H
ATOM	1466	HB3	PHE	A	95	21.163	−40.751	−23.390	1.00	0.00	H
ATOM	1467	HD1	PHE	A	95	22.389	−43.114	−23.070	1.00	0.00	H
ATOM	1468	HD2	PHE	A	95	18.802	−41.953	−20.986	1.00	0.00	H
ATOM	1469	HE1	PHE	A	95	21.910	−45.489	−22.576	1.00	0.00	H
ATOM	1470	HE2	PHE	A	95	18.329	−44.319	−20.474	1.00	0.00	H
ATOM	1471	HZ	PHE	A	95	19.870	−46.104	−21.269	1.00	0.00	H

ATOM 1472 N TYR A 96 23.187 −38.399 −22.409 1.00 0.00 N
ATOM 1473 CA TYR A 96 23.070 −37.093 −23.052 1.00 0.00 C
ATOM 1474 C TYR A 96 22.317 −37.261 −24.378 1.00 0.00 C
ATOM 1475 O TYR A 96 22.768 −37.948 −25.301 1.00 0.00 O
ATOM 1476 CB TYR A 96 24.439 −36.415 −23.192 1.00 0.00 C
ATOM 1477 CG TYR A 96 24.534 −35.090 −22.456 1.00 0.00 C
ATOM 1478 CD1 TYR A 96 25.375 −34.950 −21.333 1.00 0.00 C
ATOM 1479 CD2 TYR A 96 23.805 −33.983 −22.933 1.00 0.00 C
ATOM 1480 CE1 TYR A 96 25.460 −33.709 −20.669 1.00 0.00 C
ATOM 1481 CE2 TYR A 96 23.900 −32.742 −22.276 1.00 0.00 C
ATOM 1482 CZ TYR A 96 24.709 −32.609 −21.131 1.00 0.00 C
ATOM 1483 OH TYR A 96 24.703 −31.442 −20.444 1.00 0.00 O
ATOM 1484 H TYR A 96 23.824 −39.070 −22.826 1.00 0.00 H
ATOM 1485 HA TYR A 96 22.469 −36.437 −22.424 1.00 0.00 H
ATOM 1486 HB2 TYR A 96 25.216 −37.080 −22.816 1.00 0.00 H
ATOM 1487 HB3 TYR A 96 24.639 −36.225 −24.248 1.00 0.00 H
ATOM 1488 HD1 TYR A 96 25.954 −35.788 −20.974 1.00 0.00 H
ATOM 1489 HD2 TYR A 96 23.179 −34.087 −23.807 1.00 0.00 H
ATOM 1490 HE1 TYR A 96 26.068 −33.594 −19.784 1.00 0.00 H
ATOM 1491 HE2 TYR A 96 23.364 −31.877 −22.634 1.00 0.00 H
ATOM 1492 HH TYR A 96 23.810 −31.267 −20.096 1.00 0.00 H
ATOM 1493 N TYR A 97 21.108 −36.709 −24.428 1.00 0.00 N
ATOM 1494 CA TYR A 97 20.309 −36.566 −25.640 1.00 0.00 C
ATOM 1495 C TYR A 97 20.795 −35.330 −26.407 1.00 0.00 C
ATOM 1496 O TYR A 97 21.166 −34.322 −25.800 1.00 0.00 O
ATOM 1497 CB TYR A 97 18.822 −36.437 −25.275 1.00 0.00 C
ATOM 1498 CG TYR A 97 18.215 −37.618 −24.529 1.00 0.00 C
ATOM 1499 CD1 TYR A 97 17.333 −38.505 −25.182 1.00 0.00 C
ATOM 1500 CD2 TYR A 97 18.480 −37.796 −23.156 1.00 0.00 C
ATOM 1501 CE1 TYR A 97 16.731 −39.566 −24.473 1.00 0.00 C

ATOM 1502 CE2 TYR A 97 17.884 −38.857 −22.451 1.00 0.00 C
ATOM 1503 CZ TYR A 97 17.000 −39.736 −23.099 1.00 0.00 C
ATOM 1504 OH TYR A 97 16.403 −40.728 −22.390 1.00 0.00 O
ATOM 1505 H TYR A 97 20.815 −36.155 −23.629 1.00 0.00 H
ATOM 1506 HA TYR A 97 20.436 −37.446 −26.270 1.00 0.00 H
ATOM 1507 HB2 TYR A 97 18.692 −35.542 −24.663 1.00 0.00 H
ATOM 1508 HB3 TYR A 97 18.261 −36.281 −26.194 1.00 0.00 H
ATOM 1509 HD1 TYR A 97 17.082 −38.350 −26.218 1.00 0.00 H
ATOM 1510 HD2 TYR A 97 19.132 −37.114 −22.625 1.00 0.00 H
ATOM 1511 HE1 TYR A 97 16.030 −40.230 −24.953 1.00 0.00 H
ATOM 1512 HE2 TYR A 97 18.101 −39.007 −21.404 1.00 0.00 H
ATOM 1513 HH TYR A 97 16.318 −40.466 −21.459 1.00 0.00 H
ATOM 1514 N THR A 98 20.795 −35.383 −27.739 1.00 0.00 N
ATOM 1515 CA THR A 98 21.269 −34.285 −28.588 1.00 0.00 C
ATOM 1516 C THR A 98 20.570 −34.264 −29.951 1.00 0.00 C
ATOM 1517 O THR A 98 20.570 −35.240 −30.698 1.00 0.00 O
ATOM 1518 CB THR A 98 22.803 −34.281 −28.676 1.00 0.00 C
ATOM 1519 OG1 THR A 98 23.254 −33.191 −29.446 1.00 0.00 O
ATOM 1520 CG2 THR A 98 23.419 −35.541 −29.275 1.00 0.00 C
ATOM 1521 H THR A 98 20.556 −36.259 −28.196 1.00 0.00 H
ATOM 1522 HA THR A 98 21.000 −33.368 −28.083 1.00 0.00 H
ATOM 1523 HB THR A 98 23.195 −34.164 −27.665 1.00 0.00 H
ATOM 1524 HG1 THR A 98 24.213 −33.218 −29.435 1.00 0.00 H
ATOM 1525 HG21 THR A 98 24.487 −35.544 −29.073 1.00 0.00 H
ATOM 1526 HG22 THR A 98 23.238 −35.572 −30.349 1.00 0.00 H
ATOM 1527 HG23 THR A 98 22.994 −36.428 −28.807 1.00 0.00 H
ATOM 1528 N LEU A 99 19.791 −33.206 −30.167 1.00 0.00 N
ATOM 1529 CA LEU A 99 18.923 −32.956 −31.312 1.00 0.00 C
ATOM 1530 C LEU A 99 19.660 −32.108 −32.361 1.00 0.00 C
ATOM 1531 O LEU A 99 20.055 −30.983 −32.065 1.00 0.00 O

ATOM 1532 CB LEU A 99 17.666 −32.258 −30.755 1.00 0.00 C
ATOM 1533 CG LEU A 99 16.650 −31.785 −31.805 1.00 0.00 C
ATOM 1534 CD1 LEU A 99 16.073 −32.946 −32.608 1.00 0.00 C
ATOM 1535 CD2 LEU A 99 15.499 −31.051 −31.120 1.00 0.00 C
ATOM 1536 H LEU A 99 19.866 −32.450 −29.494 1.00 0.00 H
ATOM 1537 HA LEU A 99 18.635 −33.903 −31.766 1.00 0.00 H
ATOM 1538 HB2 LEU A 99 17.166 −32.935 −30.061 1.00 0.00 H
ATOM 1539 HB3 LEU A 99 17.985 −31.383 −30.184 1.00 0.00 H
ATOM 1540 HG LEU A 99 17.130 −31.099 −32.495 1.00 0.00 H
ATOM 1541 HD11 LEU A 99 16.861 −33.447 −33.164 1.00 0.00 H
ATOM 1542 HD12 LEU A 99 15.591 −33.654 −31.935 1.00 0.00 H
ATOM 1543 HD13 LEU A 99 15.348 −32.563 −33.320 1.00 0.00 H
ATOM 1544 HD21 LEU A 99 15.886 −30.192 −30.570 1.00 0.00 H
ATOM 1545 HD22 LEU A 99 14.788 −30.701 −31.865 1.00 0.00 H
ATOM 1546 HD23 LEU A 99 14.996 −31.718 −30.420 1.00 0.00 H
ATOM 1547 N CYS A 100 19.743 −32.584 −33.603 1.00 0.00 N
ATOM 1548 CA CYS A 100 20.414 −31.916 −34.724 1.00 0.00 C
ATOM 1549 C CYS A 100 19.472 −31.741 −35.927 1.00 0.00 C
ATOM 1550 O CYS A 100 18.683 −32.636 −36.255 1.00 0.00 O
ATOM 1551 CB CYS A 100 21.642 −32.731 −35.147 1.00 0.00 C
ATOM 1552 SG CYS A 100 22.896 −33.093 −33.884 1.00 0.00 S
ATOM 1553 H CYS A 100 19.356 −33.504 −33.785 1.00 0.00 H
ATOM 1554 HA CYS A 100 20.748 −30.924 −34.419 1.00 0.00 H
ATOM 1555 HB2 CYS A 100 21.282 −33.691 −35.523 1.00 0.00 H
ATOM 1556 HB3 CYS A 100 22.131 −32.215 −35.973 1.00 0.00 H
ATOM 1557 N ALA A 101 19.672 −30.665 −36.687 1.00 0.00 N
ATOM 1558 CA ALA A 101 19.045 −30.474 −37.993 1.00 0.00 C
ATOM 1559 C ALA A 101 19.756 −31.302 −39.077 1.00 0.00 C
ATOM 1560 O ALA A 101 20.987 −31.284 −39.165 1.00 0.00 O
ATOM 1561 CB ALA A 101 19.064 −28.979 −38.318 1.00 0.00 C

ATOM 1562 H ALA A 101 20.419 −30.022 −36.439 1.00 0.00 H
ATOM 1563 HA ALA A 101 18.005 −30.799 −37.937 1.00 0.00 H
ATOM 1564 HB1 ALA A 101 20.088 −28.641 −38.460 1.00 0.00 H
ATOM 1565 HB2 ALA A 101 18.632 −28.425 −37.488 1.00 0.00 H
ATOM 1566 HB3 ALA A 101 18.491 −28.784 −39.225 1.00 0.00 H
ATOM 1567 N VAL A 102 18.984 −31.951 −39.952 1.00 0.00 N
ATOM 1568 CA VAL A 102 19.468 −32.679 −41.135 1.00 0.00 C
ATOM 1569 C VAL A 102 19.127 −31.875 −42.390 1.00 0.00 C
ATOM 1570 O VAL A 102 18.004 −31.390 −42.515 1.00 0.00 O
ATOM 1571 CB VAL A 102 18.851 −34.090 −41.228 1.00 0.00 C
ATOM 1572 CG1 VAL A 102 19.548 −34.932 −42.305 1.00 0.00 C
ATOM 1573 CG2 VAL A 102 18.906 −34.861 −39.904 1.00 0.00 C
ATOM 1574 H VAL A 102 17.974 −31.892 −39.849 1.00 0.00 H
ATOM 1575 HA VAL A 102 20.547 −32.785 −41.066 1.00 0.00 H
ATOM 1576 HB VAL A 102 17.804 −33.996 −41.505 1.00 0.00 H
ATOM 1577 HG11 VAL A 102 19.419 −34.475 −43.285 1.00 0.00 H
ATOM 1578 HG12 VAL A 102 20.613 −35.014 −42.082 1.00 0.00 H
ATOM 1579 HG13 VAL A 102 19.112 −35.929 −42.333 1.00 0.00 H
ATOM 1580 HG21 VAL A 102 18.294 −34.358 −39.155 1.00 0.00 H
ATOM 1581 HG22 VAL A 102 18.506 −35.865 −40.042 1.00 0.00 H
ATOM 1582 HG23 VAL A 102 19.933 −34.928 −39.548 1.00 0.00 H
ATOM 1583 N SER A 103 20.038 −31.756 −43.361 1.00 0.00 N
ATOM 1584 CA SER A 103 19.801 −30.930 −44.556 1.00 0.00 C
ATOM 1585 C SER A 103 20.377 −31.517 −45.842 1.00 0.00 C
ATOM 1586 O SER A 103 21.553 −31.866 −45.909 1.00 0.00 O
ATOM 1587 CB SER A 103 20.364 −29.529 −44.337 1.00 0.00 C
ATOM 1588 OG SER A 103 20.047 −28.697 −45.429 1.00 0.00 O
ATOM 1589 H SER A 103 20.962 −32.151 −43.212 1.00 0.00 H
ATOM 1590 HA SER A 103 18.727 −30.823 −44.707 1.00 0.00 H
ATOM 1591 HB2 SER A 103 19.955 −29.104 −43.421 1.00 0.00 H

ATOM 1592 HB3 SER A 103 21.444 −29.596 −44.254 1.00 0.00 H
ATOM 1593 HG SER A 103 20.532 −27.860 −45.253 1.00 0.00 H
ATOM 1594 N HIS A 104 19.592 −31.448 −46.923 1.00 0.00 N
ATOM 1595 CA HIS A 104 20.047 −31.680 −48.303 1.00 0.00 C
ATOM 1596 C HIS A 104 20.580 −30.412 −48.995 1.00 0.00 C
ATOM 1597 O HIS A 104 21.123 −30.503 −50.091 1.00 0.00 O
ATOM 1598 CB HIS A 104 18.896 −32.315 −49.116 1.00 0.00 C
ATOM 1599 CG HIS A 104 19.210 −33.639 −49.769 1.00 0.00 C
ATOM 1600 ND1 HIS A 104 18.514 −34.157 −50.861 1.00 0.00 N
ATOM 1601 CD2 HIS A 104 20.090 −34.589 −49.328 1.00 0.00 C
ATOM 1602 CE1 HIS A 104 19.014 −35.380 −51.082 1.00 0.00 C
ATOM 1603 NE2 HIS A 104 19.964 −35.666 −50.177 1.00 0.00 N
ATOM 1604 H HIS A 104 18.648 −31.116 −46.794 1.00 0.00 H
ATOM 1605 HA HIS A 104 20.896 −32.362 −48.281 1.00 0.00 H
ATOM 1606 HB2 HIS A 104 18.031 −32.474 −48.472 1.00 0.00 H
ATOM 1607 HB3 HIS A 104 18.579 −31.622 −49.895 1.00 0.00 H
ATOM 1608 HD2 HIS A 104 20.733 −34.519 −48.463 1.00 0.00 H
ATOM 1609 HE1 HIS A 104 18.682 −36.042 −51.872 1.00 0.00 H
ATOM 1610 HE2 HIS A 104 20.477 −36.535 −50.124 1.00 0.00 H
ATOM 1611 N VAL A 105 20.430 −29.234 −48.374 1.00 0.00 N
ATOM 1612 CA VAL A 105 20.701 −27.912 −48.983 1.00 0.00 C
ATOM 1613 C VAL A 105 21.814 −27.133 −48.266 1.00 0.00 C
ATOM 1614 O VAL A 105 22.018 −25.951 −48.527 1.00 0.00 O
ATOM 1615 CB VAL A 105 19.407 −27.081 −49.114 1.00 0.00 C
ATOM 1616 CG1 VAL A 105 18.363 −27.818 −49.964 1.00 0.00 C
ATOM 1617 CG2 VAL A 105 18.773 −26.731 −47.761 1.00 0.00 C
ATOM 1618 H VAL A 105 20.024 −29.237 −47.447 1.00 0.00 H
ATOM 1619 HA VAL A 105 21.073 −28.069 −49.995 1.00 0.00 H
ATOM 1620 HB VAL A 105 19.642 −26.149 −49.626 1.00 0.00 H
ATOM 1621 HG11 VAL A 105 18.790 −28.068 −50.935 1.00 0.00 H

ATOM 1622 HG12 VAL A 105 17.494 −27.184 −50.118 1.00 0.00 H
ATOM 1623 HG13 VAL A 105 18.036 −28.734 −49.472 1.00 0.00 H
ATOM 1624 HG21 VAL A 105 19.497 −26.221 −47.124 1.00 0.00 H
ATOM 1625 HG22 VAL A 105 18.422 −27.632 −47.261 1.00 0.00 H
ATOM 1626 HG23 VAL A 105 17.929 −26.063 −47.917 1.00 0.00 H
ATOM 1627 N GLY A 106 22.529 −27.778 −47.341 1.00 0.00 N
ATOM 1628 CA GLY A 106 23.466 −27.114 −46.437 1.00 0.00 C
ATOM 1629 C GLY A 106 22.743 −26.305 −45.354 1.00 0.00 C
ATOM 1630 O GLY A 106 21.701 −26.724 −44.861 1.00 0.00 O
ATOM 1631 H GLY A 106 22.290 −28.738 −47.150 1.00 0.00 H
ATOM 1632 HA2 GLY A 106 24.106 −26.445 −47.009 1.00 0.00 H
ATOM 1633 HA3 GLY A 106 24.092 −27.856 −45.945 1.00 0.00 H
ATOM 1634 N ASP A 107 23.335 −25.183 −44.949 1.00 0.00 N
ATOM 1635 CA ASP A 107 22.908 −24.343 −43.821 1.00 0.00 C
ATOM 1636 C ASP A 107 21.398 −23.977 −43.870 1.00 0.00 C
ATOM 1637 O ASP A 107 20.968 −23.246 −44.770 1.00 0.00 O
ATOM 1638 CB ASP A 107 23.832 −23.116 −43.789 1.00 0.00 C
ATOM 1639 CG ASP A 107 23.573 −22.135 −42.639 1.00 0.00 C
ATOM 1640 OD1 ASP A 107 22.392 −21.792 −42.418 1.00 0.00 O
ATOM 1641 OD2 ASP A 107 24.564 −21.501 −42.229 1.00 0.00 O
ATOM 1642 H ASP A 107 24.144 −24.864 −45.456 1.00 0.00 H
ATOM 1643 HA ASP A 107 23.109 −24.885 −42.898 1.00 0.00 H
ATOM 1644 HB2 ASP A 107 24.861 −23.470 −43.713 1.00 0.00 H
ATOM 1645 HB3 ASP A 107 23.740 −22.579 −44.733 1.00 0.00 H
ATOM 1646 N PRO A 108 20.582 −24.454 −42.905 1.00 0.00 N
ATOM 1647 CA PRO A 108 19.149 −24.176 −42.858 1.00 0.00 C
ATOM 1648 C PRO A 108 18.764 −22.710 −42.664 1.00 0.00 C
ATOM 1649 O PRO A 108 17.672 −22.324 −43.072 1.00 0.00 O
ATOM 1650 CB PRO A 108 18.629 −24.989 −41.675 1.00 0.00 C
ATOM 1651 CG PRO A 108 19.568 −26.185 −41.627 1.00 0.00 C

ATOM	1652	CD	PRO	A	108	20.900	−25.519	−41.956	1.00	0.00	C
ATOM	1653	HA	PRO	A	108	18.693	−24.544	−43.778	1.00	0.00	H
ATOM	1654	HB2	PRO	A	108	17.594	−25.279	−41.810	1.00	0.00	H
ATOM	1655	HB3	PRO	A	108	18.742	−24.404	−40.765	1.00	0.00	H
ATOM	1656	HG2	PRO	A	108	19.581	−26.661	−40.648	1.00	0.00	H
ATOM	1657	HG3	PRO	A	108	19.306	−26.898	−42.409	1.00	0.00	H
ATOM	1658	HD2	PRO	A	108	21.581	−26.261	−42.372	1.00	0.00	H
ATOM	1659	HD3	PRO	A	108	21.327	−25.081	−41.055	1.00	0.00	H
ATOM	1660	N	ILE	A	109	19.603	−21.880	−42.039	1.00	0.00	N
ATOM	1661	CA	ILE	A	109	19.321	−20.449	−41.843	1.00	0.00	C
ATOM	1662	C	ILE	A	109	19.552	−19.687	−43.150	1.00	0.00	C
ATOM	1663	O	ILE	A	109	18.659	−18.987	−43.635	1.00	0.00	O
ATOM	1664	CB	ILE	A	109	20.190	−19.891	−40.690	1.00	0.00	C
ATOM	1665	CG1	ILE	A	109	19.950	−20.631	−39.355	1.00	0.00	C
ATOM	1666	CG2	ILE	A	109	19.987	−18.375	−40.511	1.00	0.00	C
ATOM	1667	CD1	ILE	A	109	18.536	−20.497	−38.769	1.00	0.00	C
ATOM	1668	H	ILE	A	109	20.554	−22.197	−41.857	1.00	0.00	H
ATOM	1669	HA	ILE	A	109	18.272	−20.326	−41.581	1.00	0.00	H
ATOM	1670	HB	ILE	A	109	21.239	−20.039	−40.949	1.00	0.00	H
ATOM	1671	HG12	ILE	A	109	20.172	−21.691	−39.483	1.00	0.00	H
ATOM	1672	HG13	ILE	A	109	20.664	−20.252	−38.627	1.00	0.00	H
ATOM	1673	HG21	ILE	A	109	20.393	−17.839	−41.368	1.00	0.00	H
ATOM	1674	HG22	ILE	A	109	20.519	−18.039	−39.621	1.00	0.00	H
ATOM	1675	HG23	ILE	A	109	18.928	−18.141	−40.410	1.00	0.00	H
ATOM	1676	HD11	ILE	A	109	18.476	−21.052	−37.834	1.00	0.00	H
ATOM	1677	HD12	ILE	A	109	18.302	−19.453	−38.565	1.00	0.00	H
ATOM	1678	HD13	ILE	A	109	17.805	−20.906	−39.462	1.00	0.00	H
ATOM	1679	N	LEU	A	110	20.673	−19.970	−43.820	1.00	0.00	N
ATOM	1680	CA	LEU	A	110	21.036	−19.400	−45.119	1.00	0.00	C
ATOM	1681	C	LEU	A	110	20.267	−20.019	−46.304	1.00	0.00	C

ATOM 1682 O LEU A 110 20.334 −19.488 −47.415 1.00 0.00 O
ATOM 1683 CB LEU A 110 22.561 −19.507 −45.307 1.00 0.00 C
ATOM 1684 CG LEU A 110 23.409 −18.823 −44.212 1.00 0.00 C
ATOM 1685 CD1 LEU A 110 24.893 −19.021 −44.522 1.00 0.00 C
ATOM 1686 CD2 LEU A 110 23.146 −17.316 −44.123 1.00 0.00 C
ATOM 1687 H LEU A 110 21.330 −20.614 −43.371 1.00 0.00 H
ATOM 1688 HA LEU A 110 20.767 −18.344 −45.112 1.00 0.00 H
ATOM 1689 HB2 LEU A 110 22.823 −19.070 −46.271 1.00 0.00 H
ATOM 1690 HB3 LEU A 110 22.824 −20.564 −45.340 1.00 0.00 H
ATOM 1691 HG LEU A 110 23.195 −19.266 −43.241 1.00 0.00 H
ATOM 1692 HD11 LEU A 110 25.489 −18.585 −43.721 1.00 0.00 H
ATOM 1693 HD12 LEU A 110 25.110 −20.089 −44.552 1.00 0.00 H
ATOM 1694 HD13 LEU A 110 25.151 −18.562 −45.475 1.00 0.00 H
ATOM 1695 HD21 LEU A 110 22.127 −17.140 −43.782 1.00 0.00 H
ATOM 1696 HD22 LEU A 110 23.825 −16.874 −43.394 1.00 0.00 H
ATOM 1697 HD23 LEU A 110 23.299 −16.849 −45.095 1.00 0.00 H
ATOM 1698 N ASN A 111 19.490 −21.088 −46.093 1.00 0.00 N
ATOM 1699 CA ASN A 111 18.670 −21.736 −47.126 1.00 0.00 C
ATOM 1700 C ASN A 111 17.295 −22.229 −46.622 1.00 0.00 C
ATOM 1701 O ASN A 111 16.687 −23.113 −47.224 1.00 0.00 O
ATOM 1702 CB ASN A 111 19.506 −22.812 −47.862 1.00 0.00 C
ATOM 1703 CG ASN A 111 19.685 −22.480 −49.332 1.00 0.00 C
ATOM 1704 OD1 ASN A 111 19.054 −23.053 −50.205 1.00 0.00 O
ATOM 1705 ND2 ASN A 111 20.260 −21.338 −49.630 1.00 0.00 N
ATOM 1706 H ASN A 111 19.628 −21.581 −45.215 1.00 0.00 H
ATOM 1707 HA ASN A 111 18.409 −20.965 −47.852 1.00 0.00 H
ATOM 1708 HB2 ASN A 111 20.498 −22.897 −47.416 1.00 0.00 H
ATOM 1709 HB3 ASN A 111 19.039 −23.791 −47.781 1.00 0.00 H
ATOM 1710 HD21 ASN A 111 20.570 −20.723 −48.884 1.00 0.00 H
ATOM 1711 HD22 ASN A 111 20.264 −21.069 −50.594 1.00 0.00 H

ATOM 1712 N SER A 112 16.679 −21.504 −45.681 1.00 0.00 N
ATOM 1713 CA SER A 112 15.384 −21.867 −45.068 1.00 0.00 C
ATOM 1714 C SER A 112 14.205 −22.026 −46.045 1.00 0.00 C
ATOM 1715 O SER A 112 13.201 −22.648 −45.696 1.00 0.00 O
ATOM 1716 CB SER A 112 15.010 −20.798 −44.037 1.00 0.00 C
ATOM 1717 OG SER A 112 13.794 −21.127 −43.400 1.00 0.00 O
ATOM 1718 H SER A 112 17.258 −20.848 −45.168 1.00 0.00 H
ATOM 1719 HA SER A 112 15.509 −22.813 −44.538 1.00 0.00 H
ATOM 1720 HB2 SER A 112 15.794 −20.708 −43.285 1.00 0.00 H
ATOM 1721 HB3 SER A 112 14.899 −19.837 −44.539 1.00 0.00 H
ATOM 1722 HG SER A 112 13.181 −21.505 −44.044 1.00 0.00 H
ATOM 1723 N THR A 113 14.250 −21.373 −47.212 1.00 0.00 N
ATOM 1724 CA THR A 113 13.223 −21.512 −48.268 1.00 0.00 C
ATOM 1725 C THR A 113 13.362 −22.796 −49.095 1.00 0.00 C
ATOM 1726 O THR A 113 12.437 −23.143 −49.824 1.00 0.00 O
ATOM 1727 CB THR A 113 13.214 −20.278 −49.184 1.00 0.00 C
ATOM 1728 OG1 THR A 113 12.090 −20.308 −50.027 1.00 0.00 O
ATOM 1729 CG2 THR A 113 14.452 −20.149 −50.075 1.00 0.00 C
ATOM 1730 H THR A 113 15.103 −20.884 −47.435 1.00 0.00 H
ATOM 1731 HA THR A 113 12.242 −21.563 −47.795 1.00 0.00 H
ATOM 1732 HB THR A 113 13.144 −19.386 −48.561 1.00 0.00 H
ATOM 1733 HG1 THR A 113 11.995 −21.216 −50.355 1.00 0.00 H
ATOM 1734 HG21 THR A 113 14.380 −19.230 −50.655 1.00 0.00 H
ATOM 1735 HG22 THR A 113 14.520 −20.993 −50.764 1.00 0.00 H
ATOM 1736 HG23 THR A 113 15.354 −20.111 −49.465 1.00 0.00 H
ATOM 1737 N SER A 114 14.512 −23.465 −49.008 1.00 0.00 N
ATOM 1738 CA SER A 114 14.844 −24.720 −49.695 1.00 0.00 C
ATOM 1739 C SER A 114 14.860 −25.911 −48.726 1.00 0.00 C
ATOM 1740 O SER A 114 14.755 −27.060 −49.147 1.00 0.00 O
ATOM 1741 CB SER A 114 16.241 −24.607 −50.323 1.00 0.00 C

ATOM 1742 OG SER A 114 16.469 −23.358 −50.948 1.00 0.00 O

ATOM 1743 H SER A 114 15.252 −23.065 −48.446 1.00 0.00 H

ATOM 1744 HA SER A 114 14.119 −24.921 −50.484 1.00 0.00 H

ATOM 1745 HB2 SER A 114 16.989 −24.726 −49.538 1.00 0.00 H

ATOM 1746 HB3 SER A 114 16.375 −25.406 −51.053 1.00 0.00 H

ATOM 1747 HG SER A 114 17.429 −23.190 −50.864 1.00 0.00 H

ATOM 1748 N TRP A 115 15.109 −25.654 −47.437 1.00 0.00 N

ATOM 1749 CA TRP A 115 15.312 −26.683 −46.417 1.00 0.00 C

ATOM 1750 C TRP A 115 14.010 −27.414 −46.049 1.00 0.00 C

ATOM 1751 O TRP A 115 13.085 −26.836 −45.479 1.00 0.00 O

ATOM 1752 CB TRP A 115 15.995 −26.054 −45.191 1.00 0.00 C

ATOM 1753 CG TRP A 115 16.273 −26.997 −44.059 1.00 0.00 C

ATOM 1754 CD1 TRP A 115 17.028 −28.116 −44.137 1.00 0.00 C

ATOM 1755 CD2 TRP A 115 15.765 −26.965 −42.688 1.00 0.00 C

ATOM 1756 NE1 TRP A 115 17.006 −28.780 −42.927 1.00 0.00 N

ATOM 1757 CE2 TRP A 115 16.231 −28.124 −41.999 1.00 0.00 C

ATOM 1758 CE3 TRP A 115 14.969 −26.065 −41.948 1.00 0.00 C

ATOM 1759 CZ2 TRP A 115 15.877 −28.414 −40.675 1.00 0.00 C

ATOM 1760 CZ3 TRP A 115 14.600 −26.343 −40.618 1.00 0.00 C

ATOM 1761 CH2 TRP A 115 15.039 −27.521 −39.986 1.00 0.00 C

ATOM 1762 H TRP A 115 15.291 −24.696 −47.175 1.00 0.00 H

ATOM 1763 HA TRP A 115 15.998 −27.425 −46.824 1.00 0.00 H

ATOM 1764 HB2 TRP A 115 15.360 −25.250 −44.816 1.00 0.00 H

ATOM 1765 HB3 TRP A 115 16.940 −25.607 −45.502 1.00 0.00 H

ATOM 1766 HD1 TRP A 115 17.539 −28.457 −45.027 1.00 0.00 H

ATOM 1767 HE3 TRP A 115 14.664 −25.144 −42.416 1.00 0.00 H

ATOM 1768 HZ2 TRP A 115 16.220 −29.325 −40.208 1.00 0.00 H

ATOM 1769 HZ3 TRP A 115 13.974 −25.650 −40.071 1.00 0.00 H

ATOM 1770 HH2 TRP A 115 14.726 −27.735 −38.974 1.00 0.00 H

ATOM 1771 HE1 TRP A 115 17.381 −29.718 −42.788 1.00 0.00 H

ATOM 1772 N THR A 116 13.988 −28.730 −46.260 1.00 0.00 N
ATOM 1773 CA THR A 116 13.044 −29.659 −45.621 1.00 0.00 C
ATOM 1774 C THR A 116 13.395 −29.802 −44.138 1.00 0.00 C
ATOM 1775 O THR A 116 14.513 −30.224 −43.849 1.00 0.00 O
ATOM 1776 CB THR A 116 13.144 −31.044 −46.284 1.00 0.00 C
ATOM 1777 OG1 THR A 116 14.486 −31.488 −46.274 1.00 0.00 O
ATOM 1778 CG2 THR A 116 12.672 −31.044 −47.737 1.00 0.00 C
ATOM 1779 H THR A 116 14.766 −29.147 −46.750 1.00 0.00 H
ATOM 1780 HA THR A 116 12.026 −29.282 −45.719 1.00 0.00 H
ATOM 1781 HB THR A 116 12.533 −31.750 −45.720 1.00 0.00 H
ATOM 1782 HG1 THR A 116 14.822 −31.302 −45.383 1.00 0.00 H
ATOM 1783 HG21 THR A 116 12.747 −32.054 −48.139 1.00 0.00 H
ATOM 1784 HG22 THR A 116 11.634 −30.717 −47.789 1.00 0.00 H
ATOM 1785 HG23 THR A 116 13.292 −30.378 −48.338 1.00 0.00 H
ATOM 1786 N GLU A 117 12.437 −29.637 −43.216 1.00 0.00 N
ATOM 1787 CA GLU A 117 12.686 −29.657 −41.755 1.00 0.00 C
ATOM 1788 C GLU A 117 12.916 −31.057 −41.138 1.00 0.00 C
ATOM 1789 O GLU A 117 12.334 −31.429 −40.121 1.00 0.00 O
ATOM 1790 CB GLU A 117 11.648 −28.810 −40.997 1.00 0.00 C
ATOM 1791 CG GLU A 117 10.209 −29.360 −40.956 1.00 0.00 C
ATOM 1792 CD GLU A 117 9.523 −29.123 −39.597 1.00 0.00 C
ATOM 1793 OE1 GLU A 117 8.442 −29.735 −39.428 1.00 0.00 O
ATOM 1794 OE2 GLU A 117 9.649 −27.990 −39.088 1.00 0.00 O
ATOM 1795 H GLU A 117 11.525 −29.334 −43.515 1.00 0.00 H
ATOM 1796 HA GLU A 117 13.629 −29.137 −41.609 1.00 0.00 H
ATOM 1797 HB2 GLU A 117 11.627 −27.825 −41.456 1.00 0.00 H
ATOM 1798 HB3 GLU A 117 12.018 −28.690 −39.977 1.00 0.00 H
ATOM 1799 HG2 GLU A 117 9.628 −28.887 −41.755 1.00 0.00 H
ATOM 1800 HG3 GLU A 117 10.212 −30.430 −41.164 1.00 0.00 H
ATOM 1801 N SER A 118 13.772 −31.860 −41.769 1.00 0.00 N

```
ATOM   1802  CA  SER A 118      14.220 −33.148 −41.248  1.00  0.00           C
ATOM   1803  C   SER A 118      15.060 −32.944 −39.984  1.00  0.00           C
ATOM   1804  O   SER A 118      16.156 −32.380 −40.027  1.00  0.00           O
ATOM   1805  CB  SER A 118      15.004 −33.892 −42.328  1.00  0.00           C
ATOM   1806  OG  SER A 118      15.368 −35.179 −41.872  1.00  0.00           O
ATOM   1807  H   SER A 118      14.290 −31.451 −42.537  1.00  0.00           H
ATOM   1808  HA  SER A 118      13.345 −33.746 −40.994  1.00  0.00           H
ATOM   1809  HB2 SER A 118      14.379 −33.993 −43.217  1.00  0.00           H
ATOM   1810  HB3 SER A 118      15.897 −33.322 −42.589  1.00  0.00           H
ATOM   1811  HG  SER A 118      15.892 −35.609 −42.552  1.00  0.00           H
ATOM   1812  N   LEU A 119      14.518 −33.353 −38.839  1.00  0.00           N
ATOM   1813  CA  LEU A 119      15.201 −33.325 −37.548  1.00  0.00           C
ATOM   1814  C   LEU A 119      15.597 −34.743 −37.130  1.00  0.00           C
ATOM   1815  O   LEU A 119      14.848 −35.703 −37.335  1.00  0.00           O
ATOM   1816  CB  LEU A 119      14.324 −32.623 −36.494  1.00  0.00           C
ATOM   1817  CG  LEU A 119      13.955 −31.160 −36.823  1.00  0.00           C
ATOM   1818  CD1 LEU A 119      13.219 −30.532 −35.637  1.00  0.00           C
ATOM   1819  CD2 LEU A 119      15.189 −30.304 −37.114  1.00  0.00           C
ATOM   1820  H   LEU A 119      13.597 −33.764 −38.875  1.00  0.00           H
ATOM   1821  HA  LEU A 119      16.129 −32.761 −37.641  1.00  0.00           H
ATOM   1822  HB2 LEU A 119      14.862 −32.639 −35.548  1.00  0.00           H
ATOM   1823  HB3 LEU A 119      13.402 −33.191 −36.364  1.00  0.00           H
ATOM   1824  HG  LEU A 119      13.294 −31.134 −37.686  1.00  0.00           H
ATOM   1825 HD21 LEU A 119      14.894 −29.258 −37.186  1.00  0.00           H
ATOM   1826 HD22 LEU A 119      15.931 −30.436 −36.330  1.00  0.00           H
ATOM   1827 HD23 LEU A 119      15.611 −30.593 −38.075  1.00  0.00           H
ATOM   1828 HD11 LEU A 119      12.928 −29.512 −35.890  1.00  0.00           H
ATOM   1829 HD12 LEU A 119      12.313 −31.102 −35.435  1.00  0.00           H
ATOM   1830 HD13 LEU A 119      13.855 −30.525 −34.753  1.00  0.00           H
ATOM   1831  N   SER A 120      16.735 −34.867 −36.449  1.00  0.00           N
```

ATOM 1832 CA SER A 120 17.180 −36.145 −35.891 1.00 0.00 C
ATOM 1833 C SER A 120 17.784 −35.980 −34.505 1.00 0.00 C
ATOM 1834 O SER A 120 18.568 −35.063 −34.266 1.00 0.00 O
ATOM 1835 CB SER A 120 18.134 −36.857 −36.847 1.00 0.00 C
ATOM 1836 OG SER A 120 19.362 −36.163 −37.000 1.00 0.00 O
ATOM 1837 H SER A 120 17.292 −34.038 −36.257 1.00 0.00 H
ATOM 1838 HA SER A 120 16.309 −36.787 −35.782 1.00 0.00 H
ATOM 1839 HB2 SER A 120 18.336 −37.857 −36.459 1.00 0.00 H
ATOM 1840 HB3 SER A 120 17.651 −36.957 −37.820 1.00 0.00 H
ATOM 1841 HG SER A 120 19.170 −35.218 −36.926 1.00 0.00 H
ATOM 1842 N LEU A 121 17.384 −36.852 −33.584 1.00 0.00 N
ATOM 1843 CA LEU A 121 17.939 −36.929 −32.241 1.00 0.00 C
ATOM 1844 C LEU A 121 18.898 −38.115 −32.152 1.00 0.00 C
ATOM 1845 O LEU A 121 18.556 −39.241 −32.514 1.00 0.00 O
ATOM 1846 CB LEU A 121 16.792 −36.955 −31.215 1.00 0.00 C
ATOM 1847 CG LEU A 121 17.273 −36.979 −29.751 1.00 0.00 C
ATOM 1848 CD1 LEU A 121 16.301 −36.197 −28.866 1.00 0.00 C
ATOM 1849 CD2 LEU A 121 17.364 −38.404 −29.198 1.00 0.00 C
ATOM 1850 H LEU A 121 16.810 −37.630 −33.894 1.00 0.00 H
ATOM 1851 HA LEU A 121 18.523 −36.029 −32.046 1.00 0.00 H
ATOM 1852 HB2 LEU A 121 16.207 −36.048 −31.371 1.00 0.00 H
ATOM 1853 HB3 LEU A 121 16.138 −37.808 −31.404 1.00 0.00 H
ATOM 1854 HG LEU A 121 18.250 −36.502 −29.678 1.00 0.00 H
ATOM 1855 HD21 LEU A 121 17.910 −39.049 −29.882 1.00 0.00 H
ATOM 1856 HD22 LEU A 121 17.906 −38.383 −28.255 1.00 0.00 H
ATOM 1857 HD23 LEU A 121 16.366 −38.815 −29.050 1.00 0.00 H
ATOM 1858 HD11 LEU A 121 16.600 −36.268 −27.822 1.00 0.00 H
ATOM 1859 HD12 LEU A 121 15.292 −36.594 −28.977 1.00 0.00 H
ATOM 1860 HD13 LEU A 121 16.302 −35.147 −29.157 1.00 0.00 H
ATOM 1861 N ILE A 122 20.070 −37.873 −31.583 1.00 0.00 N

ATOM 1862 CA ILE A 122 20.992 −38.899 −31.110 1.00 0.00 C

ATOM 1863 C ILE A 122 20.883 −38.946 −29.584 1.00 0.00 C

ATOM 1864 O ILE A 122 20.764 −37.905 −28.940 1.00 0.00 O

ATOM 1865 CB ILE A 122 22.433 −38.619 −31.602 1.00 0.00 C

ATOM 1866 CG1 ILE A 122 22.492 −38.331 −33.123 1.00 0.00 C

ATOM 1867 CG2 ILE A 122 23.350 −39.815 −31.289 1.00 0.00 C

ATOM 1868 CD1 ILE A 122 22.469 −36.837 −33.475 1.00 0.00 C

ATOM 1869 H ILE A 122 20.258 −36.921 −31.279 1.00 0.00 H

ATOM 1870 HA ILE A 122 20.682 −39.862 −31.509 1.00 0.00 H

ATOM 1871 HB ILE A 122 22.827 −37.756 −31.067 1.00 0.00 H

ATOM 1872 HG12 ILE A 122 23.413 −38.741 −33.537 1.00 0.00 H

ATOM 1873 HG13 ILE A 122 21.658 −38.825 −33.618 1.00 0.00 H

ATOM 1874 HG21 ILE A 122 24.365 −39.605 −31.630 1.00 0.00 H

ATOM 1875 HG22 ILE A 122 22.987 −40.711 −31.794 1.00 0.00 H

ATOM 1876 HG23 ILE A 122 23.393 −39.998 −30.217 1.00 0.00 H

ATOM 1877 HD11 ILE A 122 22.604 −36.718 −34.550 1.00 0.00 H

ATOM 1878 HD12 ILE A 122 21.519 −36.385 −33.194 1.00 0.00 H

ATOM 1879 HD13 ILE A 122 23.276 −36.321 −32.957 1.00 0.00 H

ATOM 1880 N ARG A 123 20.995 −40.127 −28.978 1.00 0.00 N

ATOM 1881 CA ARG A 123 21.327 −40.252 −27.551 1.00 0.00 C

ATOM 1882 C ARG A 123 22.633 −41.013 −27.394 1.00 0.00 C

ATOM 1883 O ARG A 123 22.848 −42.007 −28.090 1.00 0.00 O

ATOM 1884 CB ARG A 123 20.135 −40.800 −26.743 1.00 0.00 C

ATOM 1885 CG ARG A 123 19.896 −42.319 −26.839 1.00 0.00 C

ATOM 1886 CD ARG A 123 20.722 −43.108 −25.810 1.00 0.00 C

ATOM 1887 NE ARG A 123 20.310 −44.520 −25.709 1.00 0.00 N

ATOM 1888 CZ ARG A 123 19.320 −44.996 −24.976 1.00 0.00 C

ATOM 1889 NH1 ARG A 123 18.534 −44.232 −24.270 1.00 0.00 N

ATOM 1890 NH2 ARG A 123 19.083 −46.274 −24.973 1.00 0.00 N

ATOM 1891 H ARG A 123 21.082 −40.948 −29.571 1.00 0.00 H

ATOM 1892 HA ARG A 123 21.525 −39.254 −27.156 1.00 0.00 H
ATOM 1893 HB2 ARG A 123 20.263 −40.524 −25.695 1.00 0.00 H
ATOM 1894 HB3 ARG A 123 19.234 −40.291 −27.089 1.00 0.00 H
ATOM 1895 HG2 ARG A 123 20.127 −42.665 −27.845 1.00 0.00 H
ATOM 1896 HG3 ARG A 123 18.838 −42.502 −26.646 1.00 0.00 H
ATOM 1897 HD2 ARG A 123 20.610 −42.626 −24.841 1.00 0.00 H
ATOM 1898 HD3 ARG A 123 21.779 −43.078 −26.070 1.00 0.00 H
ATOM 1899 HE ARG A 123 20.950 −45.212 −26.079 1.00 0.00 H
ATOM 1900 HH11 ARG A 123 18.831 −43.285 −24.107 1.00 0.00 H
ATOM 1901 HH12 ARG A 123 17.925 −44.635 −23.583 1.00 0.00 H
ATOM 1902 HH21 ARG A 123 19.667 −46.871 −25.557 1.00 0.00 H
ATOM 1903 HH22 ARG A 123 18.344 −46.658 −24.419 1.00 0.00 H
ATOM 1904 N LEU A 124 23.464 −40.577 −26.456 1.00 0.00 N
ATOM 1905 CA LEU A 124 24.765 −41.167 −26.145 1.00 0.00 C
ATOM 1906 C LEU A 124 24.841 −41.405 −24.633 1.00 0.00 C
ATOM 1907 O LEU A 124 24.603 −40.486 −23.850 1.00 0.00 O
ATOM 1908 CB LEU A 124 25.889 −40.228 −26.633 1.00 0.00 C
ATOM 1909 CG LEU A 124 25.898 −39.898 −28.140 1.00 0.00 C
ATOM 1910 CD1 LEU A 124 26.962 −38.845 −28.450 1.00 0.00 C
ATOM 1911 CD2 LEU A 124 26.195 −41.125 −29.000 1.00 0.00 C
ATOM 1912 H LEU A 124 23.208 −39.736 −25.943 1.00 0.00 H
ATOM 1913 HA LEU A 124 24.871 −42.129 −26.645 1.00 0.00 H
ATOM 1914 HB2 LEU A 124 25.795 −39.289 −26.088 1.00 0.00 H
ATOM 1915 HB3 LEU A 124 26.849 −40.670 −26.369 1.00 0.00 H
ATOM 1916 HG LEU A 124 24.931 −39.489 −28.431 1.00 0.00 H
ATOM 1917 HD11 LEU A 124 26.989 −38.646 −29.522 1.00 0.00 H
ATOM 1918 HD12 LEU A 124 26.719 −37.918 −27.933 1.00 0.00 H
ATOM 1919 HD13 LEU A 124 27.944 −39.195 −28.128 1.00 0.00 H
ATOM 1920 HD21 LEU A 124 27.173 −41.531 −28.740 1.00 0.00 H
ATOM 1921 HD22 LEU A 124 26.193 −40.846 −30.052 1.00 0.00 H

ATOM 1922 HD23 LEU A 124 25.428 −41.880 −28.847 1.00 0.00 H
ATOM 1923 N ALA A 125 25.099 −42.645 −24.214 1.00 0.00 N
ATOM 1924 CA ALA A 125 25.290 −42.965 −22.801 1.00 0.00 C
ATOM 1925 C ALA A 125 26.560 −42.286 −22.264 1.00 0.00 C
ATOM 1926 O ALA A 125 27.632 −42.451 −22.848 1.00 0.00 O
ATOM 1927 CB ALA A 125 25.332 −44.485 −22.613 1.00 0.00 C
ATOM 1928 H ALA A 125 25.276 −43.368 −24.899 1.00 0.00 H
ATOM 1929 HA ALA A 125 24.434 −42.584 −22.252 1.00 0.00 H
ATOM 1930 HB1 ALA A 125 25.383 −44.716 −21.547 1.00 0.00 H
ATOM 1931 HB2 ALA A 125 24.429 −44.938 −23.020 1.00 0.00 H
ATOM 1932 HB3 ALA A 125 26.209 −44.897 −23.110 1.00 0.00 H
ATOM 1933 N VAL A 126 26.457 −41.559 −21.151 1.00 0.00 N
ATOM 1934 CA VAL A 126 27.574 −40.805 −20.544 1.00 0.00 C
ATOM 1935 C VAL A 126 28.640 −41.715 −19.926 1.00 0.00 C
ATOM 1936 O VAL A 126 29.812 −41.351 −19.890 1.00 0.00 O
ATOM 1937 CB VAL A 126 27.066 −39.795 −19.494 1.00 0.00 C
ATOM 1938 CG1 VAL A 126 26.022 −38.847 −20.098 1.00 0.00 C
ATOM 1939 CG2 VAL A 126 26.477 −40.468 −18.244 1.00 0.00 C
ATOM 1940 H VAL A 126 25.541 −41.497 −20.711 1.00 0.00 H
ATOM 1941 HA VAL A 126 28.072 −40.236 −21.326 1.00 0.00 H
ATOM 1942 HB VAL A 126 27.913 −39.186 −19.179 1.00 0.00 H
ATOM 1943 HG11 VAL A 126 25.795 −38.059 −19.381 1.00 0.00 H
ATOM 1944 HG12 VAL A 126 26.421 −38.392 −21.003 1.00 0.00 H
ATOM 1945 HG13 VAL A 126 25.104 −39.385 −20.333 1.00 0.00 H
ATOM 1946 HG21 VAL A 126 25.731 −41.211 −18.511 1.00 0.00 H
ATOM 1947 HG22 VAL A 126 26.014 −39.720 −17.601 1.00 0.00 H
ATOM 1948 HG23 VAL A 126 27.271 −40.947 −17.675 1.00 0.00 H
ATOM 1949 N ARG A 127 28.264 −42.957 −19.596 1.00 0.00 N
ATOM 1950 CA ARG A 127 29.091 −43.949 −18.894 1.00 0.00 C
ATOM 1951 C ARG A 127 28.913 −45.341 −19.537 1.00 0.00 C

ATOM 1952 O ARG A 127 28.294 −46.226 −18.950 1.00 0.00 O
ATOM 1953 CB ARG A 127 28.709 −43.859 −17.410 1.00 0.00 C
ATOM 1954 CG ARG A 127 29.694 −44.532 −16.445 1.00 0.00 C
ATOM 1955 CD ARG A 127 29.337 −44.218 −14.981 1.00 0.00 C
ATOM 1956 NE ARG A 127 28.027 −44.785 −14.591 1.00 0.00 N
ATOM 1957 CZ ARG A 127 26.842 −44.205 −14.700 1.00 0.00 C
ATOM 1958 NH1 ARG A 127 26.672 −42.965 −15.050 1.00 0.00 N
ATOM 1959 NH2 ARG A 127 25.764 −44.905 −14.536 1.00 0.00 N
ATOM 1960 H ARG A 127 27.278 −43.157 −19.683 1.00 0.00 H
ATOM 1961 HA ARG A 127 30.140 −43.665 −18.979 1.00 0.00 H
ATOM 1962 HB2 ARG A 127 27.711 −44.281 −17.284 1.00 0.00 H
ATOM 1963 HB3 ARG A 127 28.670 −42.803 −17.136 1.00 0.00 H
ATOM 1964 HG2 ARG A 127 29.689 −45.610 −16.603 1.00 0.00 H
ATOM 1965 HG3 ARG A 127 30.698 −44.151 −16.642 1.00 0.00 H
ATOM 1966 HD2 ARG A 127 30.114 −44.641 −14.341 1.00 0.00 H
ATOM 1967 HD3 ARG A 127 29.346 −43.139 −14.828 1.00 0.00 H
ATOM 1968 HE ARG A 127 28.020 −45.746 −14.308 1.00 0.00 H
ATOM 1969 HH11 ARG A 127 27.463 −42.388 −15.274 1.00 0.00 H
ATOM 1970 HH12 ARG A 127 25.712 −42.688 −15.276 1.00 0.00 H
ATOM 1971 HH21 ARG A 127 25.793 −45.900 −14.511 1.00 0.00 H
ATOM 1972 HH22 ARG A 127 24.901 −44.499 −14.924 1.00 0.00 H
ATOM 1973 N PRO A 128 29.234 −45.478 −20.837 1.00 0.00 N
ATOM 1974 CA PRO A 128 28.738 −46.535 −21.723 1.00 0.00 C
ATOM 1975 C PRO A 128 29.149 −47.935 −21.238 1.00 0.00 C
ATOM 1976 O PRO A 128 29.228 −48.879 −22.029 1.00 0.00 O
ATOM 1977 CB PRO A 128 29.303 −46.175 −23.105 1.00 0.00 C
ATOM 1978 CG PRO A 128 30.610 −45.470 −22.761 1.00 0.00 C
ATOM 1979 CD PRO A 128 30.194 −44.647 −21.547 1.00 0.00 C
ATOM 1980 HA PRO A 128 27.650 −46.498 −21.756 1.00 0.00 H
ATOM 1981 HB2 PRO A 128 29.484 −47.038 −23.740 1.00 0.00 H

ATOM	1982	HB3	PRO	A	128	28.632	−45.474	−23.607	1.00	0.00	H
ATOM	1983	HG2	PRO	A	128	31.365	−46.203	−22.473	1.00	0.00	H
ATOM	1984	HG3	PRO	A	128	30.969	−44.856	−23.581	1.00	0.00	H
ATOM	1985	HD2	PRO	A	128	29.721	−43.719	−21.871	1.00	0.00	H
ATOM	1986	HD3	PRO	A	128	31.060	−44.436	−20.922	1.00	0.00	H
ATOM	1987	H	PRO	A	128	29.373	−48.095	−20.184	1.00	0.00	H
ATOM	1988	N	ASP	A	129	22.436	−50.640	−28.124	1.00	0.00	N
ATOM	1989	CA	ASP	A	129	21.606	−49.436	−28.177	1.00	0.00	C
ATOM	1990	C	ASP	A	129	21.352	−48.782	−26.806	1.00	0.00	C
ATOM	1991	O	ASP	A	129	20.647	−47.771	−26.712	1.00	0.00	O
ATOM	1992	CB	ASP	A	129	20.295	−49.717	−28.923	1.00	0.00	C
ATOM	1993	CG	ASP	A	129	20.503	−49.899	−30.428	1.00	0.00	C
ATOM	1994	OD1	ASP	A	129	21.168	−50.881	−30.805	1.00	0.00	O
ATOM	1995	OD2	ASP	A	129	19.705	−49.263	−31.161	1.00	0.00	O
ATOM	1996	HA	ASP	A	129	22.156	−48.706	−28.763	1.00	0.00	H
ATOM	1997	HB2	ASP	A	129	19.829	−50.611	−28.505	1.00	0.00	H
ATOM	1998	HB3	ASP	A	129	19.606	−48.885	−28.765	1.00	0.00	H
ATOM	1999	H	ASP	A	129	22.454	−51.206	−28.976	1.00	0.00	H
ATOM	2000	H	ASP	A	129	22.090	−51.224	−27.369	1.00	0.00	H
ATOM	2001	N	TYR	A	130	21.992	−49.277	−25.741	1.00	0.00	N
ATOM	2002	CA	TYR	A	130	22.263	−48.464	−24.547	1.00	0.00	C
ATOM	2003	C	TYR	A	130	23.263	−47.350	−24.887	1.00	0.00	C
ATOM	2004	O	TYR	A	130	22.895	−46.176	−24.920	1.00	0.00	O
ATOM	2005	CB	TYR	A	130	22.771	−49.347	−23.391	1.00	0.00	C
ATOM	2006	CG	TYR	A	130	23.259	−48.569	−22.172	1.00	0.00	C
ATOM	2007	CD1	TYR	A	130	22.338	−47.886	−21.352	1.00	0.00	C
ATOM	2008	CD2	TYR	A	130	24.634	−48.510	−21.866	1.00	0.00	C
ATOM	2009	CE1	TYR	A	130	22.785	−47.123	−20.250	1.00	0.00	C
ATOM	2010	CE2	TYR	A	130	25.080	−47.762	−20.758	1.00	0.00	C
ATOM	2011	CZ	TYR	A	130	24.162	−47.061	−19.948	1.00	0.00	C

ATOM 2012 OH TYR A 130 24.632 −46.335 −18.897 1.00 0.00 O
ATOM 2013 H TYR A 130 22.543 −50.110 −25.901 1.00 0.00 H
ATOM 2014 HA TYR A 130 21.342 −47.983 −24.217 1.00 0.00 H
ATOM 2015 HB2 TYR A 130 21.965 −50.014 −23.078 1.00 0.00 H
ATOM 2016 HB3 TYR A 130 23.587 −49.975 −23.755 1.00 0.00 H
ATOM 2017 HD1 TYR A 130 21.283 −47.942 −21.570 1.00 0.00 H
ATOM 2018 HD2 TYR A 130 25.352 −49.052 −22.468 1.00 0.00 H
ATOM 2019 HE1 TYR A 130 22.081 −46.579 −19.636 1.00 0.00 H
ATOM 2020 HE2 TYR A 130 26.126 −47.727 −20.497 1.00 0.00 H
ATOM 2021 HH TYR A 130 23.968 −46.063 −18.248 1.00 0.00 H
ATOM 2022 N ASN A 131 24.436 −47.753 −25.384 1.00 0.00 N
ATOM 2023 CA ASN A 131 25.605 −46.901 −25.602 1.00 0.00 C
ATOM 2024 C ASN A 131 25.331 −45.726 −26.552 1.00 0.00 C
ATOM 2025 O ASN A 131 25.723 −44.598 −26.259 1.00 0.00 O
ATOM 2026 CB ASN A 131 26.769 −47.771 −26.131 1.00 0.00 C
ATOM 2027 CG ASN A 131 27.141 −48.939 −25.225 1.00 0.00 C
ATOM 2028 OD1 ASN A 131 26.299 −49.682 −24.752 1.00 0.00 O
ATOM 2029 ND2 ASN A 131 28.402 −49.105 −24.912 1.00 0.00 N
ATOM 2030 H ASN A 131 24.631 −48.747 −25.325 1.00 0.00 H
ATOM 2031 HA ASN A 131 25.902 −46.481 −24.641 1.00 0.00 H
ATOM 2032 HB2 ASN A 131 27.643 −47.132 −26.259 1.00 0.00 H
ATOM 2033 HB3 ASN A 131 26.512 −48.180 −27.108 1.00 0.00 H
ATOM 2034 HD21 ASN A 131 29.108 −48.482 −25.253 1.00 0.00 H
ATOM 2035 HD22 ASN A 131 28.594 −49.730 −24.141 1.00 0.00 H
ATOM 2036 N GLN A 132 24.650 −45.977 −27.672 1.00 0.00 N
ATOM 2037 CA GLN A 132 24.299 −44.979 −28.686 1.00 0.00 C
ATOM 2038 C GLN A 132 22.949 −45.342 −29.317 1.00 0.00 C
ATOM 2039 O GLN A 132 22.684 −46.522 −29.536 1.00 0.00 O
ATOM 2040 CB GLN A 132 25.379 −44.902 −29.787 1.00 0.00 C
ATOM 2041 CG GLN A 132 26.811 −44.675 −29.271 1.00 0.00 C

ATOM 2042 CD GLN A 132 27.807 −44.298 −30.364 1.00 0.00 C
ATOM 2043 OE1 GLN A 132 27.565 −44.420 −31.554 1.00 0.00 O
ATOM 2044 NE2 GLN A 132 28.975 −43.842 −29.978 1.00 0.00 N
ATOM 2045 H GLN A 132 24.312 −46.916 −27.827 1.00 0.00 H
ATOM 2046 HA GLN A 132 24.215 −44.004 −28.212 1.00 0.00 H
ATOM 2047 HB2 GLN A 132 25.115 −44.077 −30.452 1.00 0.00 H
ATOM 2048 HB3 GLN A 132 25.366 −45.824 −30.371 1.00 0.00 H
ATOM 2049 HG2 GLN A 132 27.173 −45.578 −28.781 1.00 0.00 H
ATOM 2050 HG3 GLN A 132 26.801 −43.873 −28.536 1.00 0.00 H
ATOM 2051 HE21 GLN A 132 29.169 −43.718 −28.989 1.00 0.00 H
ATOM 2052 HE22 GLN A 132 29.648 −43.550 −30.675 1.00 0.00 H
ATOM 2053 N LYS A 133 22.153 −44.352 −29.736 1.00 0.00 N
ATOM 2054 CA LYS A 133 21.011 −44.567 −30.645 1.00 0.00 C
ATOM 2055 C LYS A 133 20.738 −43.321 −31.484 1.00 0.00 C
ATOM 2056 O LYS A 133 20.755 −42.215 −30.952 1.00 0.00 O
ATOM 2057 CB LYS A 133 19.764 −45.009 −29.854 1.00 0.00 C
ATOM 2058 CG LYS A 133 18.697 −45.632 −30.765 1.00 0.00 C
ATOM 2059 CD LYS A 133 17.467 −46.066 −29.954 1.00 0.00 C
ATOM 2060 CE LYS A 133 16.652 −47.133 −30.698 1.00 0.00 C
ATOM 2061 NZ LYS A 133 17.302 −48.464 −30.638 1.00 0.00 N
ATOM 2062 H LYS A 133 22.411 −43.397 −29.494 1.00 0.00 H
ATOM 2063 HA LYS A 133 21.283 −45.377 −31.326 1.00 0.00 H
ATOM 2064 HB2 LYS A 133 19.338 −44.155 −29.327 1.00 0.00 H
ATOM 2065 HB3 LYS A 133 20.060 −45.760 −29.119 1.00 0.00 H
ATOM 2066 HG2 LYS A 133 18.376 −44.909 −31.516 1.00 0.00 H
ATOM 2067 HG3 LYS A 133 19.136 −46.490 −31.276 1.00 0.00 H
ATOM 2068 HD2 LYS A 133 16.839 −45.187 −29.795 1.00 0.00 H
ATOM 2069 HD3 LYS A 133 17.773 −46.449 −28.977 1.00 0.00 H
ATOM 2070 HE2 LYS A 133 15.658 −47.188 −30.249 1.00 0.00 H
ATOM 2071 HE3 LYS A 133 16.541 −46.820 −31.741 1.00 0.00 H

ATOM 2072 HZ1 LYS A 133 16.801 −49.178 −31.143 1.00 0.00 H
ATOM 2073 HZ2 LYS A 133 17.484 −48.776 −29.693 1.00 0.00 H
ATOM 2074 HZ3 LYS A 133 18.233 −48.448 −31.073 1.00 0.00 H
ATOM 2075 N TYR A 134 20.411 −43.516 −32.760 1.00 0.00 N
ATOM 2076 CA TYR A 134 19.962 −42.474 −33.692 1.00 0.00 C
ATOM 2077 C TYR A 134 18.449 −42.582 −33.945 1.00 0.00 C
ATOM 2078 O TYR A 134 17.930 −43.688 −34.102 1.00 0.00 O
ATOM 2079 CB TYR A 134 20.763 −42.612 −34.997 1.00 0.00 C
ATOM 2080 CG TYR A 134 20.545 −41.495 −36.000 1.00 0.00 C
ATOM 2081 CD1 TYR A 134 19.536 −41.594 −36.980 1.00 0.00 C
ATOM 2082 CD2 TYR A 134 21.367 −40.354 −35.955 1.00 0.00 C
ATOM 2083 CE1 TYR A 134 19.352 −40.551 −37.911 1.00 0.00 C
ATOM 2084 CE2 TYR A 134 21.163 −39.295 −36.859 1.00 0.00 C
ATOM 2085 CZ TYR A 134 20.167 −39.401 −37.851 1.00 0.00 C
ATOM 2086 OH TYR A 134 19.969 −38.379 −38.723 1.00 0.00 O
ATOM 2087 H TYR A 134 20.363 −44.465 −33.092 1.00 0.00 H
ATOM 2088 HA TYR A 134 20.168 −41.489 −33.270 1.00 0.00 H
ATOM 2089 HB2 TYR A 134 20.511 −43.563 −35.470 1.00 0.00 H
ATOM 2090 HB3 TYR A 134 21.825 −42.646 −34.753 1.00 0.00 H
ATOM 2091 HD1 TYR A 134 18.906 −42.472 −37.022 1.00 0.00 H
ATOM 2092 HD2 TYR A 134 22.161 −40.291 −35.224 1.00 0.00 H
ATOM 2093 HE1 TYR A 134 18.586 −40.614 −38.668 1.00 0.00 H
ATOM 2094 HE2 TYR A 134 21.772 −38.405 −36.805 1.00 0.00 H
ATOM 2095 HH TYR A 134 20.019 −37.528 −38.263 1.00 0.00 H
ATOM 2096 N ILE A 135 17.752 −41.449 −34.045 1.00 0.00 N
ATOM 2097 CA ILE A 135 16.310 −41.346 −34.315 1.00 0.00 C
ATOM 2098 C ILE A 135 16.084 −40.234 −35.346 1.00 0.00 C
ATOM 2099 O ILE A 135 16.319 −39.065 −35.051 1.00 0.00 O
ATOM 2100 CB ILE A 135 15.519 −41.072 −33.005 1.00 0.00 C
ATOM 2101 CG1 ILE A 135 15.700 −42.219 −31.981 1.00 0.00 C

```
ATOM   2102  CG2 ILE A 135      14.023 −40.840 −33.305  1.00  0.00           C
ATOM   2103  CD1 ILE A 135      15.024 −41.976 −30.624  1.00  0.00           C
ATOM   2104  H   ILE A 135      18.227 −40.579 −33.816  1.00  0.00           H
ATOM   2105  HA  ILE A 135      15.952 −42.285 −34.738  1.00  0.00           H
ATOM   2106  HB  ILE A 135      15.911 −40.157 −32.556  1.00  0.00           H
ATOM   2107 HG12 ILE A 135      15.318 −43.148 −32.406  1.00  0.00           H
ATOM   2108 HG13 ILE A 135      16.762 −42.351 −31.778  1.00  0.00           H
ATOM   2109 HD11 ILE A 135      15.334 −42.751 −29.924  1.00  0.00           H
ATOM   2110 HD12 ILE A 135      13.939 −42.017 −30.722  1.00  0.00           H
ATOM   2111 HD13 ILE A 135      15.320 −41.003 −30.230  1.00  0.00           H
ATOM   2112 HG21 ILE A 135      13.889 −39.995 −33.979  1.00  0.00           H
ATOM   2113 HG22 ILE A 135      13.483 −40.591 −32.392  1.00  0.00           H
ATOM   2114 HG23 ILE A 135      13.582 −41.730 −33.755  1.00  0.00           H
ATOM   2115  N   ALA A 136      15.507 −40.564 −36.504  1.00  0.00           N
ATOM   2116  CA  ALA A 136      14.855 −39.575 −37.366  1.00  0.00           C
ATOM   2117  C   ALA A 136      13.444 −39.277 −36.826  1.00  0.00           C
ATOM   2118  O   ALA A 136      12.671 −40.205 −36.577  1.00  0.00           O
ATOM   2119  CB  ALA A 136      14.820 −40.109 −38.802  1.00  0.00           C
ATOM   2120  H   ALA A 136      15.260 −41.529 −36.651  1.00  0.00           H
ATOM   2121  HA  ALA A 136      15.431 −38.647 −37.364  1.00  0.00           H
ATOM   2122  HB1 ALA A 136      14.353 −39.370 −39.454  1.00  0.00           H
ATOM   2123  HB2 ALA A 136      15.836 −40.298 −39.152  1.00  0.00           H
ATOM   2124  HB3 ALA A 136      14.243 −41.034 −38.845  1.00  0.00           H
ATOM   2125  N   ILE A 137      13.132 −38.008 −36.556  1.00  0.00           N
ATOM   2126  CA  ILE A 137      11.854 −37.620 −35.944  1.00  0.00           C
ATOM   2127  C   ILE A 137      10.745 −37.585 −37.000  1.00  0.00           C
ATOM   2128  O   ILE A 137      10.852 −36.910 −38.018  1.00  0.00           O
ATOM   2129  CB  ILE A 137      12.017 −36.292 −35.178  1.00  0.00           C
ATOM   2130  CG1 ILE A 137      12.875 −36.523 −33.909  1.00  0.00           C
ATOM   2131  CG2 ILE A 137      10.665 −35.691 −34.762  1.00  0.00           C
```

ATOM 2132 CD1 ILE A 137 13.977 −35.475 −33.773 1.00 0.00 C
ATOM 2133 H ILE A 137 13.774 −37.268 −36.828 1.00 0.00 H
ATOM 2134 HA ILE A 137 11.579 −38.379 −35.211 1.00 0.00 H
ATOM 2135 HB ILE A 137 12.512 −35.577 −35.839 1.00 0.00 H
ATOM 2136 HG12 ILE A 137 13.355 −37.502 −33.931 1.00 0.00 H
ATOM 2137 HG13 ILE A 137 12.246 −36.494 −33.016 1.00 0.00 H
ATOM 2138 HD11 ILE A 137 14.423 −35.551 −32.784 1.00 0.00 H
ATOM 2139 HD12 ILE A 137 14.739 −35.651 −34.526 1.00 0.00 H
ATOM 2140 HD13 ILE A 137 13.564 −34.483 −33.918 1.00 0.00 H
ATOM 2141 HG21 ILE A 137 10.103 −35.377 −35.642 1.00 0.00 H
ATOM 2142 HG22 ILE A 137 10.829 −34.807 −34.146 1.00 0.00 H
ATOM 2143 HG23 ILE A 137 10.085 −36.411 −34.182 1.00 0.00 H
ATOM 2144 N THR A 138 9.645 −38.290 −36.726 1.00 0.00 N
ATOM 2145 CA THR A 138 8.498 −38.440 −37.644 1.00 0.00 C
ATOM 2146 C THR A 138 7.315 −37.520 −37.324 1.00 0.00 C
ATOM 2147 O THR A 138 6.333 −37.513 −38.065 1.00 0.00 O
ATOM 2148 CB THR A 138 8.022 −39.900 −37.684 1.00 0.00 C
ATOM 2149 OG1 THR A 138 7.709 −40.357 −36.387 1.00 0.00 O
ATOM 2150 CG2 THR A 138 9.081 −40.845 −38.253 1.00 0.00 C
ATOM 2151 H THR A 138 9.632 −38.831 −35.875 1.00 0.00 H
ATOM 2152 HA THR A 138 8.816 −38.175 −38.653 1.00 0.00 H
ATOM 2153 HB THR A 138 7.132 −39.969 −38.309 1.00 0.00 H
ATOM 2154 HG21 THR A 138 8.679 −41.857 −38.302 1.00 0.00 H
ATOM 2155 HG22 THR A 138 9.346 −40.526 −39.261 1.00 0.00 H
ATOM 2156 HG23 THR A 138 9.980 −40.840 −37.635 1.00 0.00 H
ATOM 2157 HG1 THR A 138 8.436 −40.916 −36.102 1.00 0.00 H
ATOM 2158 N LYS A 139 7.394 −36.724 −36.247 1.00 0.00 N
ATOM 2159 CA LYS A 139 6.368 −35.755 −35.831 1.00 0.00 C
ATOM 2160 C LYS A 139 7.001 −34.519 −35.196 1.00 0.00 C
ATOM 2161 O LYS A 139 7.706 −34.636 −34.194 1.00 0.00 O

ATOM	2162	CB	LYS	A	139	5.399	−36.390	−34.824	1.00	0.00	C
ATOM	2163	CG	LYS	A	139	4.450	−37.429	−35.435	1.00	0.00	C
ATOM	2164	CD	LYS	A	139	3.465	−37.888	−34.357	1.00	0.00	C
ATOM	2165	CE	LYS	A	139	2.395	−38.813	−34.930	1.00	0.00	C
ATOM	2166	NZ	LYS	A	139	1.403	−39.149	−33.879	1.00	0.00	N
ATOM	2167	H	LYS	A	139	8.243	−36.764	−35.700	1.00	0.00	H
ATOM	2168	HA	LYS	A	139	5.802	−35.432	−36.707	1.00	0.00	H
ATOM	2169	HB2	LYS	A	139	5.976	−36.851	−34.021	1.00	0.00	H
ATOM	2170	HB3	LYS	A	139	4.792	−35.590	−34.392	1.00	0.00	H
ATOM	2171	HG2	LYS	A	139	3.903	−36.977	−36.263	1.00	0.00	H
ATOM	2172	HG3	LYS	A	139	5.015	−38.289	−35.797	1.00	0.00	H
ATOM	2173	HD2	LYS	A	139	4.016	−38.421	−33.582	1.00	0.00	H
ATOM	2174	HD3	LYS	A	139	2.983	−37.010	−33.920	1.00	0.00	H
ATOM	2175	HE2	LYS	A	139	1.906	−38.303	−35.766	1.00	0.00	H
ATOM	2176	HE3	LYS	A	139	2.879	−39.716	−35.316	1.00	0.00	H
ATOM	2177	HZ1	LYS	A	139	0.676	−39.746	−34.248	1.00	0.00	H
ATOM	2178	HZ2	LYS	A	139	0.983	−38.298	−33.521	1.00	0.00	H
ATOM	2179	HZ3	LYS	A	139	1.858	−39.619	−33.107	1.00	0.00	H
ATOM	2180	N	VAL	A	140	6.590	−33.344	−35.672	1.00	0.00	N
ATOM	2181	CA	VAL	A	140	6.974	−32.042	−35.118	1.00	0.00	C
ATOM	2182	C	VAL	A	140	5.751	−31.125	−35.115	1.00	0.00	C
ATOM	2183	O	VAL	A	140	5.136	−30.895	−36.155	1.00	0.00	O
ATOM	2184	CB	VAL	A	140	8.144	−31.406	−35.897	1.00	0.00	C
ATOM	2185	CG1	VAL	A	140	8.675	−30.189	−35.138	1.00	0.00	C
ATOM	2186	CG2	VAL	A	140	9.329	−32.359	−36.109	1.00	0.00	C
ATOM	2187	H	VAL	A	140	5.960	−33.334	−36.459	1.00	0.00	H
ATOM	2188	HA	VAL	A	140	7.300	−32.185	−34.091	1.00	0.00	H
ATOM	2189	HB	VAL	A	140	7.791	−31.083	−36.876	1.00	0.00	H
ATOM	2190	HG11	VAL	A	140	9.022	−30.476	−34.147	1.00	0.00	H
ATOM	2191	HG12	VAL	A	140	7.900	−29.432	−35.038	1.00	0.00	H

ATOM 2192 HG13 VAL A 140 9.506 −29.769 −35.702 1.00 0.00 H
ATOM 2193 HG21 VAL A 140 9.035 −33.188 −36.753 1.00 0.00 H
ATOM 2194 HG22 VAL A 140 10.142 −31.831 −36.607 1.00 0.00 H
ATOM 2195 HG23 VAL A 140 9.677 −32.746 −35.152 1.00 0.00 H
ATOM 2196 N GLU A 141 5.341 −30.653 −33.942 1.00 0.00 N
ATOM 2197 CA GLU A 141 4.078 −29.939 −33.729 1.00 0.00 C
ATOM 2198 C GLU A 141 4.362 −28.475 −33.356 1.00 0.00 C
ATOM 2199 O GLU A 141 4.981 −28.189 −32.332 1.00 0.00 O
ATOM 2200 CB GLU A 141 3.234 −30.676 −32.673 1.00 0.00 C
ATOM 2201 CG GLU A 141 2.808 −32.075 −33.166 1.00 0.00 C
ATOM 2202 CD GLU A 141 2.043 −32.922 −32.135 1.00 0.00 C
ATOM 2203 OE1 GLU A 141 2.082 −32.580 −30.929 1.00 0.00 O
ATOM 2204 OE2 GLU A 141 1.618 −34.032 −32.535 1.00 0.00 O
ATOM 2205 H GLU A 141 5.866 −30.921 −33.112 1.00 0.00 H
ATOM 2206 HA GLU A 141 3.498 −29.941 −34.652 1.00 0.00 H
ATOM 2207 HB2 GLU A 141 3.821 −30.764 −31.762 1.00 0.00 H
ATOM 2208 HB3 GLU A 141 2.340 −30.087 −32.460 1.00 0.00 H
ATOM 2209 HG2 GLU A 141 2.183 −31.945 −34.053 1.00 0.00 H
ATOM 2210 HG3 GLU A 141 3.694 −32.638 −33.465 1.00 0.00 H
ATOM 2211 N ARG A 142 4.044 −27.543 −34.268 1.00 0.00 N
ATOM 2212 CA ARG A 142 4.571 −26.161 −34.235 1.00 0.00 C
ATOM 2213 C ARG A 142 3.576 −25.054 −34.610 1.00 0.00 C
ATOM 2214 O ARG A 142 3.972 −23.962 −35.010 1.00 0.00 O
ATOM 2215 CB ARG A 142 5.925 −26.076 −34.971 1.00 0.00 C
ATOM 2216 CG ARG A 142 5.940 −26.320 −36.490 1.00 0.00 C
ATOM 2217 CD ARG A 142 5.754 −27.792 −36.899 1.00 0.00 C
ATOM 2218 NE ARG A 142 6.327 −28.106 −38.221 1.00 0.00 N
ATOM 2219 CZ ARG A 142 6.017 −27.592 −39.395 1.00 0.00 C
ATOM 2220 NH1 ARG A 142 5.168 −26.627 −39.572 1.00 0.00 N
ATOM 2221 NH2 ARG A 142 6.628 −27.982 −40.457 1.00 0.00 N

ATOM 2222 H ARG A 142 3.613 −27.868 −35.120 1.00 0.00 H

ATOM 2223 HA ARG A 142 4.797 −25.935 −33.190 1.00 0.00 H

ATOM 2224 HB2 ARG A 142 6.324 −25.079 −34.802 1.00 0.00 H

ATOM 2225 HB3 ARG A 142 6.623 −26.772 −34.506 1.00 0.00 H

ATOM 2226 HG2 ARG A 142 6.910 −25.996 −36.868 1.00 0.00 H

ATOM 2227 HG3 ARG A 142 5.173 −25.709 −36.964 1.00 0.00 H

ATOM 2228 HD2 ARG A 142 6.254 −28.422 −36.168 1.00 0.00 H

ATOM 2229 HD3 ARG A 142 4.697 −28.053 −36.888 1.00 0.00 H

ATOM 2230 HE ARG A 142 7.083 −28.795 −38.268 1.00 0.00 H

ATOM 2231 HH11 ARG A 142 4.839 −26.086 −38.799 1.00 0.00 H

ATOM 2232 HH12 ARG A 142 5.221 −26.165 −40.462 1.00 0.00 H

ATOM 2233 HH21 ARG A 142 7.363 −28.665 −40.273 1.00 0.00 H

ATOM 2234 HH22 ARG A 142 6.716 −27.339 −41.232 1.00 0.00 H

ATOM 2235 N GLY A 143 2.279 −25.299 −34.425 1.00 0.00 N

ATOM 2236 CA GLY A 143 1.241 −24.266 −34.534 1.00 0.00 C

ATOM 2237 C GLY A 143 1.241 −23.542 −35.888 1.00 0.00 C

ATOM 2238 O GLY A 143 1.014 −24.167 −36.921 1.00 0.00 O

ATOM 2239 H GLY A 143 2.026 −26.199 −34.050 1.00 0.00 H

ATOM 2240 HA2 GLY A 143 0.262 −24.725 −34.403 1.00 0.00 H

ATOM 2241 HA3 GLY A 143 1.380 −23.537 −33.736 1.00 0.00 H

ATOM 2242 N LYS A 144 1.447 −22.216 −35.879 1.00 0.00 N

ATOM 2243 CA LYS A 144 1.487 −21.372 −37.091 1.00 0.00 C

ATOM 2244 C LYS A 144 2.871 −21.250 −37.750 1.00 0.00 C

ATOM 2245 O LYS A 144 2.976 −20.586 −38.779 1.00 0.00 O

ATOM 2246 CB LYS A 144 0.877 −19.984 −36.784 1.00 0.00 C

ATOM 2247 CG LYS A 144 −0.319 −19.627 −37.686 1.00 0.00 C

ATOM 2248 CD LYS A 144 0.033 −19.432 −39.171 1.00 0.00 C

ATOM 2249 CE LYS A 144 −1.245 −19.149 −39.970 1.00 0.00 C

ATOM 2250 NZ LYS A 144 −0.968 −18.970 −41.415 1.00 0.00 N

ATOM 2251 H LYS A 144 1.653 −21.781 −34.990 1.00 0.00 H

ATOM	2252	HA	LYS	A	144	0.868	−21.864	−37.838	1.00	0.00	H
ATOM	2253	HB2	LYS	A	144	0.523	−19.957	−35.750	1.00	0.00	H
ATOM	2254	HB3	LYS	A	144	1.640	−19.206	−36.875	1.00	0.00	H
ATOM	2255	HG2	LYS	A	144	−1.067	−20.415	−37.597	1.00	0.00	H
ATOM	2256	HG3	LYS	A	144	−0.761	−18.701	−37.314	1.00	0.00	H
ATOM	2257	HD2	LYS	A	144	0.732	−18.598	−39.273	1.00	0.00	H
ATOM	2258	HD3	LYS	A	144	0.501	−20.337	−39.561	1.00	0.00	H
ATOM	2259	HE2	LYS	A	144	−1.932	−19.987	−39.826	1.00	0.00	H
ATOM	2260	HE3	LYS	A	144	−1.719	−18.246	−39.573	1.00	0.00	H
ATOM	2261	HZ1	LYS	A	144	−0.380	−19.695	−41.794	1.00	0.00	H
ATOM	2262	HZ2	LYS	A	144	−0.503	−18.074	−41.592	1.00	0.00	H
ATOM	2263	HZ3	LYS	A	144	−1.813	−18.886	−41.959	1.00	0.00	H
ATOM	2264	N	TYR	A	145	3.925	−21.834	−37.176	1.00	0.00	N
ATOM	2265	CA	TYR	A	145	5.253	−21.852	−37.800	1.00	0.00	C
ATOM	2266	C	TYR	A	145	5.272	−22.804	−38.999	1.00	0.00	C
ATOM	2267	O	TYR	A	145	4.770	−23.925	−38.927	1.00	0.00	O
ATOM	2268	CB	TYR	A	145	6.342	−22.182	−36.769	1.00	0.00	C
ATOM	2269	CG	TYR	A	145	6.509	−21.056	−35.773	1.00	0.00	C
ATOM	2270	CD1	TYR	A	145	5.715	−21.007	−34.613	1.00	0.00	C
ATOM	2271	CD2	TYR	A	145	7.365	−19.982	−36.079	1.00	0.00	C
ATOM	2272	CE1	TYR	A	145	5.729	−19.858	−33.800	1.00	0.00	C
ATOM	2273	CE2	TYR	A	145	7.389	−18.837	−35.265	1.00	0.00	C
ATOM	2274	CZ	TYR	A	145	6.543	−18.758	−34.145	1.00	0.00	C
ATOM	2275	OH	TYR	A	145	6.492	−17.603	−33.440	1.00	0.00	O
ATOM	2276	H	TYR	A	145	3.782	−22.425	−36.368	1.00	0.00	H
ATOM	2277	HA	TYR	A	145	5.461	−20.851	−38.182	1.00	0.00	H
ATOM	2278	HB2	TYR	A	145	6.094	−23.104	−36.249	1.00	0.00	H
ATOM	2279	HB3	TYR	A	145	7.288	−22.332	−37.288	1.00	0.00	H
ATOM	2280	HD1	TYR	A	145	5.060	−21.831	−34.367	1.00	0.00	H
ATOM	2281	HD2	TYR	A	145	7.955	−20.007	−36.979	1.00	0.00	H

ATOM	2282	HE1	TYR	A	145	5.096	−19.805	−32.928	1.00	0.00	H
ATOM	2283	HE2	TYR	A	145	8.007	−17.991	−35.517	1.00	0.00	H
ATOM	2284	HH	TYR	A	145	5.833	−17.687	−32.700	1.00	0.00	H
ATOM	2285	N	ASP	A	146	5.869	−22.350	−40.102	1.00	0.00	N
ATOM	2286	CA	ASP	A	146	6.030	−23.118	−41.346	1.00	0.00	C
ATOM	2287	C	ASP	A	146	6.996	−24.298	−41.150	1.00	0.00	C
ATOM	2288	O	ASP	A	146	6.691	−25.415	−41.581	1.00	0.00	O
ATOM	2289	CB	ASP	A	146	6.495	−22.127	−42.428	1.00	0.00	C
ATOM	2290	CG	ASP	A	146	6.955	−22.724	−43.769	1.00	0.00	C
ATOM	2291	OD1	ASP	A	146	7.858	−23.583	−43.718	1.00	0.00	O
ATOM	2292	OD2	ASP	A	146	6.922	−21.945	−44.747	1.00	0.00	O
ATOM	2293	H	ASP	A	146	6.360	−21.466	−40.035	1.00	0.00	H
ATOM	2294	HA	ASP	A	146	5.067	−23.530	−41.647	1.00	0.00	H
ATOM	2295	HB2	ASP	A	146	5.683	−21.425	−42.616	1.00	0.00	H
ATOM	2296	HB3	ASP	A	146	7.331	−21.560	−42.018	1.00	0.00	H
ATOM	2297	N	LYS	A	147	8.019	−24.088	−40.310	1.00	0.00	N
ATOM	2298	CA	LYS	A	147	9.024	−25.069	−39.881	1.00	0.00	C
ATOM	2299	C	LYS	A	147	9.731	−24.635	−38.586	1.00	0.00	C
ATOM	2300	O	LYS	A	147	9.635	−23.466	−38.202	1.00	0.00	O
ATOM	2301	CB	LYS	A	147	10.021	−25.317	−41.030	1.00	0.00	C
ATOM	2302	CG	LYS	A	147	10.678	−24.031	−41.555	1.00	0.00	C
ATOM	2303	CD	LYS	A	147	11.762	−24.261	−42.610	1.00	0.00	C
ATOM	2304	CE	LYS	A	147	11.314	−24.966	−43.894	1.00	0.00	C
ATOM	2305	NZ	LYS	A	147	10.284	−24.214	−44.643	1.00	0.00	N
ATOM	2306	H	LYS	A	147	8.125	−23.148	−39.944	1.00	0.00	H
ATOM	2307	HA	LYS	A	147	8.518	−26.008	−39.661	1.00	0.00	H
ATOM	2308	HB2	LYS	A	147	9.499	−25.817	−41.847	1.00	0.00	H
ATOM	2309	HB3	LYS	A	147	10.803	−25.979	−40.663	1.00	0.00	H
ATOM	2310	HG2	LYS	A	147	9.925	−23.356	−41.962	1.00	0.00	H
ATOM	2311	HG3	LYS	A	147	11.161	−23.550	−40.713	1.00	0.00	H

ATOM 2312 HD2 LYS A 147 12.206 −23.300 −42.868 1.00 0.00 H
ATOM 2313 HD3 LYS A 147 12.532 −24.871 −42.149 1.00 0.00 H
ATOM 2314 HE2 LYS A 147 12.195 −25.084 −44.529 1.00 0.00 H
ATOM 2315 HE3 LYS A 147 10.945 −25.964 −43.643 1.00 0.00 H
ATOM 2316 HZ1 LYS A 147 10.019 −24.687 −45.491 1.00 0.00 H
ATOM 2317 HZ2 LYS A 147 10.584 −23.277 −44.886 1.00 0.00 H
ATOM 2318 HZ3 LYS A 147 9.424 −24.097 −44.098 1.00 0.00 H
ATOM 2319 N VAL A 148 10.462 −25.542 −37.933 1.00 0.00 N
ATOM 2320 CA VAL A 148 11.216 −25.325 −36.673 1.00 0.00 C
ATOM 2321 C VAL A 148 12.532 −26.126 −36.630 1.00 0.00 C
ATOM 2322 O VAL A 148 12.715 −27.076 −37.388 1.00 0.00 O
ATOM 2323 CB VAL A 148 10.369 −25.668 −35.428 1.00 0.00 C
ATOM 2324 CG1 VAL A 148 9.206 −24.701 −35.199 1.00 0.00 C
ATOM 2325 CG2 VAL A 148 9.812 −27.091 −35.480 1.00 0.00 C
ATOM 2326 H VAL A 148 10.440 −26.496 −38.303 1.00 0.00 H
ATOM 2327 HA VAL A 148 11.491 −24.275 −36.608 1.00 0.00 H
ATOM 2328 HB VAL A 148 11.008 −25.600 −34.548 1.00 0.00 H
ATOM 2329 HG11 VAL A 148 9.588 −23.726 −34.915 1.00 0.00 H
ATOM 2330 HG12 VAL A 148 8.584 −24.612 −36.085 1.00 0.00 H
ATOM 2331 HG13 VAL A 148 8.597 −25.057 −34.376 1.00 0.00 H
ATOM 2332 HG21 VAL A 148 9.257 −27.310 −34.569 1.00 0.00 H
ATOM 2333 HG22 VAL A 148 10.636 −27.800 −35.566 1.00 0.00 H
ATOM 2334 HG23 VAL A 148 9.150 −27.217 −36.338 1.00 0.00 H
ATOM 2335 N MET A 149 13.474 −25.744 −35.757 1.00 0.00 N
ATOM 2336 CA MET A 149 14.831 −26.317 −35.710 1.00 0.00 C
ATOM 2337 C MET A 149 15.505 −26.168 −34.325 1.00 0.00 C
ATOM 2338 O MET A 149 15.299 −25.136 −33.683 1.00 0.00 O
ATOM 2339 CB MET A 149 15.657 −25.606 −36.796 1.00 0.00 C
ATOM 2340 CG MET A 149 16.999 −26.261 −37.104 1.00 0.00 C
ATOM 2341 SD MET A 149 18.024 −25.355 −38.283 1.00 0.00 S

ATOM	2342	CE	MET	A	149	18.574	−23.966	−37.257	1.00	0.00	C
ATOM	2343	H	MET	A	149	13.302	−24.907	−35.208	1.00	0.00	H
ATOM	2344	HA	MET	A	149	14.758	−27.374	−35.956	1.00	0.00	H
ATOM	2345	HB2	MET	A	149	15.092	−25.600	−37.727	1.00	0.00	H
ATOM	2346	HB3	MET	A	149	15.822	−24.574	−36.492	1.00	0.00	H
ATOM	2347	HG2	MET	A	149	16.790	−27.244	−37.525	1.00	0.00	H
ATOM	2348	HG3	MET	A	149	17.573	−26.388	−36.191	1.00	0.00	H
ATOM	2349	HE1	MET	A	149	19.254	−23.339	−37.832	1.00	0.00	H
ATOM	2350	HE2	MET	A	149	19.097	−24.346	−36.381	1.00	0.00	H
ATOM	2351	HE3	MET	A	149	17.719	−23.370	−36.939	1.00	0.00	H
ATOM	2352	N	PRO	A	150	16.368	−27.107	−33.872	1.00	0.00	N
ATOM	2353	CA	PRO	A	150	17.294	−26.876	−32.751	1.00	0.00	C
ATOM	2354	C	PRO	A	150	18.264	−25.722	−33.045	1.00	0.00	C
ATOM	2355	O	PRO	A	150	18.670	−25.519	−34.189	1.00	0.00	O
ATOM	2356	CB	PRO	A	150	18.069	−28.187	−32.574	1.00	0.00	C
ATOM	2357	CG	PRO	A	150	18.061	−28.772	−33.985	1.00	0.00	C
ATOM	2358	CD	PRO	A	150	16.663	−28.402	−34.473	1.00	0.00	C
ATOM	2359	HA	PRO	A	150	16.737	−26.654	−31.840	1.00	0.00	H
ATOM	2360	HB2	PRO	A	150	19.085	−28.025	−32.216	1.00	0.00	H
ATOM	2361	HB3	PRO	A	150	17.527	−28.841	−31.893	1.00	0.00	H
ATOM	2362	HG2	PRO	A	150	18.223	−29.849	−33.990	1.00	0.00	H
ATOM	2363	HG3	PRO	A	150	18.813	−28.274	−34.600	1.00	0.00	H
ATOM	2364	HD2	PRO	A	150	16.654	−28.371	−35.559	1.00	0.00	H
ATOM	2365	HD3	PRO	A	150	15.941	−29.136	−34.113	1.00	0.00	H
ATOM	2366	N	TYR	A	151	18.590	−24.917	−32.031	1.00	0.00	N
ATOM	2367	CA	TYR	A	151	19.341	−23.674	−32.233	1.00	0.00	C
ATOM	2368	C	TYR	A	151	20.016	−23.186	−30.941	1.00	0.00	C
ATOM	2369	O	TYR	A	151	19.713	−22.114	−30.419	1.00	0.00	O
ATOM	2370	CB	TYR	A	151	18.403	−22.623	−32.868	1.00	0.00	C
ATOM	2371	CG	TYR	A	151	19.048	−21.560	−33.743	1.00	0.00	C

ATOM	2372	CD1	TYR	A	151	18.568	−20.241	−33.676	1.00	0.00	C
ATOM	2373	CD2	TYR	A	151	19.979	−21.910	−34.745	1.00	0.00	C
ATOM	2374	CE1	TYR	A	151	18.960	−19.297	−34.640	1.00	0.00	C
ATOM	2375	CE2	TYR	A	151	20.447	−20.939	−35.653	1.00	0.00	C
ATOM	2376	CZ	TYR	A	151	19.924	−19.630	−35.610	1.00	0.00	C
ATOM	2377	OH	TYR	A	151	20.328	−18.683	−36.497	1.00	0.00	O
ATOM	2378	H	TYR	A	151	18.203	−25.107	−31.111	1.00	0.00	H
ATOM	2379	HA	TYR	A	151	20.143	−23.896	−32.934	1.00	0.00	H
ATOM	2380	HB2	TYR	A	151	17.689	−23.134	−33.513	1.00	0.00	H
ATOM	2381	HB3	TYR	A	151	17.809	−22.150	−32.085	1.00	0.00	H
ATOM	2382	HD1	TYR	A	151	17.850	−19.967	−32.915	1.00	0.00	H
ATOM	2383	HD2	TYR	A	151	20.312	−22.934	−34.848	1.00	0.00	H
ATOM	2384	HE1	TYR	A	151	18.510	−18.323	−34.653	1.00	0.00	H
ATOM	2385	HE2	TYR	A	151	21.162	−21.202	−36.419	1.00	0.00	H
ATOM	2386	HH	TYR	A	151	19.667	−17.995	−36.601	1.00	0.00	H
ATOM	2387	N	GLY	A	152	20.890	−24.009	−30.359	1.00	0.00	N
ATOM	2388	CA	GLY	A	152	21.552	−23.673	−29.097	1.00	0.00	C
ATOM	2389	C	GLY	A	152	22.239	−24.860	−28.426	1.00	0.00	C
ATOM	2390	O	GLY	A	152	21.687	−25.952	−28.480	1.00	0.00	O
ATOM	2391	H	GLY	A	152	21.040	−24.929	−30.754	1.00	0.00	H
ATOM	2392	HA2	GLY	A	152	22.258	−22.859	−29.262	1.00	0.00	H
ATOM	2393	HA3	GLY	A	152	20.781	−23.339	−28.416	1.00	0.00	H
ATOM	2394	N	PRO	A	153	23.405	−24.702	−27.776	1.00	0.00	N
ATOM	2395	CA	PRO	A	153	24.228	−25.843	−27.369	1.00	0.00	C
ATOM	2396	C	PRO	A	153	23.640	−26.672	−26.212	1.00	0.00	C
ATOM	2397	O	PRO	A	153	23.677	−27.905	−26.268	1.00	0.00	O
ATOM	2398	CB	PRO	A	153	25.579	−25.225	−27.000	1.00	0.00	C
ATOM	2399	CG	PRO	A	153	25.225	−23.812	−26.537	1.00	0.00	C
ATOM	2400	CD	PRO	A	153	24.061	−23.444	−27.457	1.00	0.00	C
ATOM	2401	HA	PRO	A	153	24.365	−26.512	−28.219	1.00	0.00	H

ATOM 2402 HB2 PRO A 153 26.205 −25.169 −27.891 1.00 0.00 H
ATOM 2403 HB3 PRO A 153 26.083 −25.789 −26.220 1.00 0.00 H
ATOM 2404 HG2 PRO A 153 24.885 −23.833 −25.499 1.00 0.00 H
ATOM 2405 HG3 PRO A 153 26.064 −23.127 −26.650 1.00 0.00 H
ATOM 2406 HD2 PRO A 153 24.442 −22.986 −28.370 1.00 0.00 H
ATOM 2407 HD3 PRO A 153 23.379 −22.762 −26.948 1.00 0.00 H
ATOM 2408 N SER A 154 23.019 −26.020 −25.223 1.00 0.00 N
ATOM 2409 CA SER A 154 22.569 −26.648 −23.975 1.00 0.00 C
ATOM 2410 C SER A 154 21.151 −26.224 −23.568 1.00 0.00 C
ATOM 2411 O SER A 154 20.751 −25.060 −23.687 1.00 0.00 O
ATOM 2412 CB SER A 154 23.597 −26.414 −22.855 1.00 0.00 C
ATOM 2413 OG SER A 154 23.932 −25.055 −22.654 1.00 0.00 O
ATOM 2414 H SER A 154 23.007 −25.012 −25.238 1.00 0.00 H
ATOM 2415 HA SER A 154 22.538 −27.727 −24.127 1.00 0.00 H
ATOM 2416 HB2 SER A 154 23.223 −26.842 −21.924 1.00 0.00 H
ATOM 2417 HB3 SER A 154 24.514 −26.929 −23.128 1.00 0.00 H
ATOM 2418 HG SER A 154 24.684 −25.048 −22.044 1.00 0.00 H
ATOM 2419 N GLY A 155 20.356 −27.239 −23.228 1.00 0.00 N
ATOM 2420 CA GLY A 155 18.976 −27.155 −22.759 1.00 0.00 C
ATOM 2421 C GLY A 155 18.775 −27.849 −21.409 1.00 0.00 C
ATOM 2422 O GLY A 155 19.743 −28.164 −20.717 1.00 0.00 O
ATOM 2423 H GLY A 155 20.767 −28.166 −23.215 1.00 0.00 H
ATOM 2424 HA2 GLY A 155 18.323 −27.627 −23.491 1.00 0.00 H
ATOM 2425 HA3 GLY A 155 18.692 −26.115 −22.651 1.00 0.00 H
ATOM 2426 N ILE A 156 17.518 −28.117 −21.035 1.00 0.00 N
ATOM 2427 CA ILE A 156 17.182 −28.738 −19.741 1.00 0.00 C
ATOM 2428 C ILE A 156 16.191 −29.894 −19.860 1.00 0.00 C
ATOM 2429 O ILE A 156 15.198 −29.823 −20.575 1.00 0.00 O
ATOM 2430 CB ILE A 156 16.704 −27.705 −18.693 1.00 0.00 C
ATOM 2431 CG1 ILE A 156 15.406 −26.972 −19.102 1.00 0.00 C

ATOM 2432 CG2 ILE A 156 17.847 −26.733 −18.368 1.00 0.00 C
ATOM 2433 CD1 ILE A 156 14.821 −26.095 −17.991 1.00 0.00 C
ATOM 2434 H ILE A 156 16.773 −27.945 −21.701 1.00 0.00 H
ATOM 2435 HA ILE A 156 18.095 −29.181 −19.343 1.00 0.00 H
ATOM 2436 HB ILE A 156 16.488 −28.256 −17.778 1.00 0.00 H
ATOM 2437 HG12 ILE A 156 14.640 −27.700 −19.360 1.00 0.00 H
ATOM 2438 HG13 ILE A 156 15.593 −26.353 −19.980 1.00 0.00 H
ATOM 2439 HD11 ILE A 156 14.602 −26.705 −17.115 1.00 0.00 H
ATOM 2440 HD12 ILE A 156 15.524 −25.312 −17.721 1.00 0.00 H
ATOM 2441 HD13 ILE A 156 13.904 −25.628 −18.349 1.00 0.00 H
ATOM 2442 HG21 ILE A 156 18.759 −27.288 −18.143 1.00 0.00 H
ATOM 2443 HG22 ILE A 156 17.595 −26.106 −17.516 1.00 0.00 H
ATOM 2444 HG23 ILE A 156 18.029 −26.098 −19.230 1.00 0.00 H
ATOM 2445 N LYS A 157 16.353 −30.863 −18.959 1.00 0.00 N
ATOM 2446 CA LYS A 157 15.305 −31.793 −18.525 1.00 0.00 C
ATOM 2447 C LYS A 157 14.466 −31.186 −17.398 1.00 0.00 C
ATOM 2448 O LYS A 157 15.002 −30.494 −16.528 1.00 0.00 O
ATOM 2449 CB LYS A 157 15.896 −33.169 −18.159 1.00 0.00 C
ATOM 2450 CG LYS A 157 16.745 −33.254 −16.876 1.00 0.00 C
ATOM 2451 CD LYS A 157 18.001 −32.367 −16.897 1.00 0.00 C
ATOM 2452 CE LYS A 157 19.039 −32.862 −15.886 1.00 0.00 C
ATOM 2453 NZ LYS A 157 20.409 −32.719 −16.420 1.00 0.00 N
ATOM 2454 H LYS A 157 17.180 −30.805 −18.389 1.00 0.00 H
ATOM 2455 HA LYS A 157 14.626 −31.954 −19.365 1.00 0.00 H
ATOM 2456 HB2 LYS A 157 15.068 −33.871 −18.050 1.00 0.00 H
ATOM 2457 HB3 LYS A 157 16.497 −33.526 −18.997 1.00 0.00 H
ATOM 2458 HG2 LYS A 157 17.052 −34.294 −16.767 1.00 0.00 H
ATOM 2459 HG3 LYS A 157 16.136 −32.996 −16.010 1.00 0.00 H
ATOM 2460 HD2 LYS A 157 18.429 −32.395 −17.900 1.00 0.00 H
ATOM 2461 HD3 LYS A 157 17.737 −31.337 −16.660 1.00 0.00 H

ATOM 2462 HE2 LYS A 157 18.929 −32.313 −14.948 1.00 0.00 H
ATOM 2463 HE3 LYS A 157 18.856 −33.922 −15.690 1.00 0.00 H
ATOM 2464 HZ1 LYS A 157 21.110 −32.990 −15.734 1.00 0.00 H
ATOM 2465 HZ2 LYS A 157 20.655 −31.761 −16.687 1.00 0.00 H
ATOM 2466 HZ3 LYS A 157 20.513 −33.278 −17.262 1.00 0.00 H
ATOM 2467 N GLN A 158 13.197 −31.573 −17.347 1.00 0.00 N
ATOM 2468 CA GLN A 158 12.217 −31.262 −16.303 1.00 0.00 C
ATOM 2469 C GLN A 158 11.470 −32.550 −15.928 1.00 0.00 C
ATOM 2470 O GLN A 158 10.349 −32.794 −16.373 1.00 0.00 O
ATOM 2471 CB GLN A 158 11.275 −30.136 −16.765 1.00 0.00 C
ATOM 2472 CG GLN A 158 12.010 −28.797 −16.962 1.00 0.00 C
ATOM 2473 CD GLN A 158 11.070 −27.595 −17.027 1.00 0.00 C
ATOM 2474 OE1 GLN A 158 9.859 −27.693 −17.077 1.00 0.00 O
ATOM 2475 NE2 GLN A 158 11.590 −26.390 −16.974 1.00 0.00 N
ATOM 2476 H GLN A 158 12.863 −32.133 −18.128 1.00 0.00 H
ATOM 2477 HA GLN A 158 12.734 −30.924 −15.405 1.00 0.00 H
ATOM 2478 HB2 GLN A 158 10.513 −30.006 −15.996 1.00 0.00 H
ATOM 2479 HB3 GLN A 158 10.781 −30.421 −17.696 1.00 0.00 H
ATOM 2480 HG2 GLN A 158 12.691 −28.641 −16.125 1.00 0.00 H
ATOM 2481 HG3 GLN A 158 12.600 −28.835 −17.878 1.00 0.00 H
ATOM 2482 HE21 GLN A 158 12.582 −26.256 −16.929 1.00 0.00 H
ATOM 2483 HE22 GLN A 158 10.924 −25.639 −17.010 1.00 0.00 H
ATOM 2484 N GLY A 159 12.221 −33.490 −15.345 1.00 0.00 N
ATOM 2485 CA GLY A 159 11.855 −34.907 −15.367 1.00 0.00 C
ATOM 2486 C GLY A 159 11.900 −35.471 −16.789 1.00 0.00 C
ATOM 2487 O GLY A 159 12.805 −35.151 −17.557 1.00 0.00 O
ATOM 2488 H GLY A 159 13.179 −33.263 −15.148 1.00 0.00 H
ATOM 2489 HA2 GLY A 159 12.547 −35.481 −14.755 1.00 0.00 H
ATOM 2490 HA3 GLY A 159 10.849 −35.029 −14.962 1.00 0.00 H
ATOM 2491 N ASP A 160 10.856 −36.212 −17.148 1.00 0.00 N

ATOM 2492 CA ASP A 160 10.718 −36.950 −18.411 1.00 0.00 C
ATOM 2493 C ASP A 160 10.544 −36.057 −19.669 1.00 0.00 C
ATOM 2494 O ASP A 160 10.648 −36.533 −20.801 1.00 0.00 O
ATOM 2495 CB ASP A 160 9.501 −37.876 −18.246 1.00 0.00 C
ATOM 2496 CG ASP A 160 9.617 −39.195 −19.005 1.00 0.00 C
ATOM 2497 OD1 ASP A 160 10.722 −39.773 −19.040 1.00 0.00 O
ATOM 2498 OD2 ASP A 160 8.542 −39.697 −19.403 1.00 0.00 O
ATOM 2499 H ASP A 160 10.151 −36.391 −16.452 1.00 0.00 H
ATOM 2500 HA ASP A 160 11.619 −37.554 −18.538 1.00 0.00 H
ATOM 2501 HB2 ASP A 160 9.380 −38.139 −17.193 1.00 0.00 H
ATOM 2502 HB3 ASP A 160 8.599 −37.340 −18.550 1.00 0.00 H
ATOM 2503 N THR A 161 10.279 −34.752 −19.499 1.00 0.00 N
ATOM 2504 CA THR A 161 10.316 −33.761 −20.587 1.00 0.00 C
ATOM 2505 C THR A 161 11.706 −33.151 −20.735 1.00 0.00 C
ATOM 2506 O THR A 161 12.170 −32.458 −19.831 1.00 0.00 O
ATOM 2507 CB THR A 161 9.261 −32.660 −20.394 1.00 0.00 C
ATOM 2508 OG1 THR A 161 7.967 −33.191 −20.555 1.00 0.00 O
ATOM 2509 CG2 THR A 161 9.338 −31.581 −21.477 1.00 0.00 C
ATOM 2510 H THR A 161 10.264 −34.401 −18.550 1.00 0.00 H
ATOM 2511 HA THR A 161 10.092 −34.261 −21.522 1.00 0.00 H
ATOM 2512 HB THR A 161 9.364 −32.202 −19.404 1.00 0.00 H
ATOM 2513 HG1 THR A 161 7.968 −34.123 −20.262 1.00 0.00 H
ATOM 2514 HG21 THR A 161 8.545 −30.852 −21.325 1.00 0.00 H
ATOM 2515 HG22 THR A 161 10.278 −31.046 −21.398 1.00 0.00 H
ATOM 2516 HG23 THR A 161 9.261 −32.014 −22.477 1.00 0.00 H
ATOM 2517 N LEU A 162 12.286 −33.230 −21.938 1.00 0.00 N
ATOM 2518 CA LEU A 162 13.448 −32.434 −22.352 1.00 0.00 C
ATOM 2519 C LEU A 162 12.988 −31.128 −23.029 1.00 0.00 C
ATOM 2520 O LEU A 162 11.986 −31.130 −23.745 1.00 0.00 O
ATOM 2521 CB LEU A 162 14.363 −33.255 −23.287 1.00 0.00 C

ATOM 2522 CG LEU A 162 14.598 −34.727 −22.894 1.00 0.00 C
ATOM 2523 CD1 LEU A 162 15.581 −35.352 −23.883 1.00 0.00 C
ATOM 2524 CD2 LEU A 162 15.179 −34.897 −21.490 1.00 0.00 C
ATOM 2525 H LEU A 162 11.832 −33.794 −22.648 1.00 0.00 H
ATOM 2526 HA LEU A 162 14.035 −32.169 −21.474 1.00 0.00 H
ATOM 2527 HB2 LEU A 162 13.923 −33.245 −24.282 1.00 0.00 H
ATOM 2528 HB3 LEU A 162 15.327 −32.746 −23.358 1.00 0.00 H
ATOM 2529 HG LEU A 162 13.660 −35.276 −22.958 1.00 0.00 H
ATOM 2530 HD11 LEU A 162 15.705 −36.409 −23.648 1.00 0.00 H
ATOM 2531 HD12 LEU A 162 16.545 −34.850 −23.808 1.00 0.00 H
ATOM 2532 HD13 LEU A 162 15.199 −35.254 −24.898 1.00 0.00 H
ATOM 2533 HD21 LEU A 162 15.324 −35.958 −21.287 1.00 0.00 H
ATOM 2534 HD22 LEU A 162 14.485 −34.508 −20.744 1.00 0.00 H
ATOM 2535 HD23 LEU A 162 16.132 −34.380 −21.410 1.00 0.00 H
ATOM 2536 N TYR A 163 13.800 −30.073 −22.944 1.00 0.00 N
ATOM 2537 CA TYR A 163 13.611 −28.778 −23.602 1.00 0.00 C
ATOM 2538 C TYR A 163 14.925 −28.317 −24.241 1.00 0.00 C
ATOM 2539 O TYR A 163 15.799 −27.756 −23.573 1.00 0.00 O
ATOM 2540 CB TYR A 163 13.137 −27.738 −22.581 1.00 0.00 C
ATOM 2541 CG TYR A 163 11.701 −27.871 −22.128 1.00 0.00 C
ATOM 2542 CD1 TYR A 163 10.684 −27.299 −22.913 1.00 0.00 C
ATOM 2543 CD2 TYR A 163 11.393 −28.466 −20.890 1.00 0.00 C
ATOM 2544 CE1 TYR A 163 9.363 −27.265 −22.439 1.00 0.00 C
ATOM 2545 CE2 TYR A 163 10.066 −28.449 −20.419 1.00 0.00 C
ATOM 2546 CZ TYR A 163 9.057 −27.832 −21.188 1.00 0.00 C
ATOM 2547 OH TYR A 163 7.783 −27.790 −20.733 1.00 0.00 O
ATOM 2548 H TYR A 163 14.566 −30.127 −22.282 1.00 0.00 H
ATOM 2549 HA TYR A 163 12.860 −28.859 −24.390 1.00 0.00 H
ATOM 2550 HB2 TYR A 163 13.794 −27.766 −21.709 1.00 0.00 H
ATOM 2551 HB3 TYR A 163 13.248 −26.747 −23.023 1.00 0.00 H

ATOM	2552	HD1	TYR	A	163	10.921	−26.844	−23.861	1.00	0.00	H
ATOM	2553	HD2	TYR	A	163	12.176	−28.920	−20.297	1.00	0.00	H
ATOM	2554	HE1	TYR	A	163	8.586	−26.780	−23.015	1.00	0.00	H
ATOM	2555	HE2	TYR	A	163	9.808	−28.883	−19.465	1.00	0.00	H
ATOM	2556	HH	TYR	A	163	7.313	−27.014	−21.131	1.00	0.00	H
ATOM	2557	N	PHE	A	164	15.043	−28.501	−25.555	1.00	0.00	N
ATOM	2558	CA	PHE	A	164	16.165	−28.004	−26.353	1.00	0.00	C
ATOM	2559	C	PHE	A	164	15.931	−26.532	−26.753	1.00	0.00	C
ATOM	2560	O	PHE	A	164	14.851	−26.237	−27.271	1.00	0.00	O
ATOM	2561	CB	PHE	A	164	16.309	−28.888	−27.602	1.00	0.00	C
ATOM	2562	CG	PHE	A	164	16.519	−30.365	−27.309	1.00	0.00	C
ATOM	2563	CD1	PHE	A	164	17.792	−30.828	−26.931	1.00	0.00	C
ATOM	2564	CD2	PHE	A	164	15.443	−31.272	−27.393	1.00	0.00	C
ATOM	2565	CE1	PHE	A	164	17.994	−32.191	−26.645	1.00	0.00	C
ATOM	2566	CE2	PHE	A	164	15.644	−32.635	−27.105	1.00	0.00	C
ATOM	2567	CZ	PHE	A	164	16.919	−33.093	−26.730	1.00	0.00	C
ATOM	2568	H	PHE	A	164	14.272	−28.939	−26.050	1.00	0.00	H
ATOM	2569	HA	PHE	A	164	17.080	−28.090	−25.768	1.00	0.00	H
ATOM	2570	HB2	PHE	A	164	15.414	−28.777	−28.215	1.00	0.00	H
ATOM	2571	HB3	PHE	A	164	17.149	−28.529	−28.195	1.00	0.00	H
ATOM	2572	HD1	PHE	A	164	18.615	−30.133	−26.855	1.00	0.00	H
ATOM	2573	HD2	PHE	A	164	14.458	−30.922	−27.667	1.00	0.00	H
ATOM	2574	HE1	PHE	A	164	18.973	−32.541	−26.355	1.00	0.00	H
ATOM	2575	HE2	PHE	A	164	14.815	−33.327	−27.162	1.00	0.00	H
ATOM	2576	HZ	PHE	A	164	17.071	−34.136	−26.499	1.00	0.00	H
ATOM	2577	N	PRO	A	165	16.905	−25.607	−26.631	1.00	0.00	N
ATOM	2578	CA	PRO	A	165	16.816	−24.278	−27.248	1.00	0.00	C
ATOM	2579	C	PRO	A	165	16.688	−24.370	−28.777	1.00	0.00	C
ATOM	2580	O	PRO	A	165	17.375	−25.156	−29.434	1.00	0.00	O
ATOM	2581	CB	PRO	A	165	18.091	−23.538	−26.832	1.00	0.00	C

ATOM	2582	CG	PRO	A	165	19.075	−24.682	−26.614	1.00	0.00	C
ATOM	2583	CD	PRO	A	165	18.198	−25.757	−25.982	1.00	0.00	C
ATOM	2584	HA	PRO	A	165	15.949	−23.748	−26.855	1.00	0.00	H
ATOM	2585	HB2	PRO	A	165	17.924	−23.011	−25.891	1.00	0.00	H
ATOM	2586	HB3	PRO	A	165	18.442	−22.853	−27.604	1.00	0.00	H
ATOM	2587	HG2	PRO	A	165	19.432	−25.033	−27.580	1.00	0.00	H
ATOM	2588	HG3	PRO	A	165	19.908	−24.392	−25.979	1.00	0.00	H
ATOM	2589	HD2	PRO	A	165	18.085	−25.562	−24.916	1.00	0.00	H
ATOM	2590	HD3	PRO	A	165	18.635	−26.741	−26.138	1.00	0.00	H
ATOM	2591	N	ALA	A	166	15.809	−23.551	−29.352	1.00	0.00	N
ATOM	2592	CA	ALA	A	166	15.298	−23.750	−30.706	1.00	0.00	C
ATOM	2593	C	ALA	A	166	14.928	−22.440	−31.420	1.00	0.00	C
ATOM	2594	O	ALA	A	166	14.850	−21.373	−30.810	1.00	0.00	O
ATOM	2595	CB	ALA	A	166	14.084	−24.678	−30.595	1.00	0.00	C
ATOM	2596	H	ALA	A	166	15.276	−22.924	−28.757	1.00	0.00	H
ATOM	2597	HA	ALA	A	166	16.053	−24.246	−31.316	1.00	0.00	H
ATOM	2598	HB1	ALA	A	166	13.307	−24.201	−29.999	1.00	0.00	H
ATOM	2599	HB2	ALA	A	166	13.688	−24.895	−31.586	1.00	0.00	H
ATOM	2600	HB3	ALA	A	166	14.379	−25.615	−30.120	1.00	0.00	H
ATOM	2601	N	VAL	A	167	14.563	−22.559	−32.699	1.00	0.00	N
ATOM	2602	CA	VAL	A	167	14.020	−21.481	−33.530	1.00	0.00	C
ATOM	2603	C	VAL	A	167	12.843	−21.965	−34.377	1.00	0.00	C
ATOM	2604	O	VAL	A	167	12.872	−23.062	−34.934	1.00	0.00	O
ATOM	2605	CB	VAL	A	167	15.137	−20.835	−34.373	1.00	0.00	C
ATOM	2606	CG1	VAL	A	167	15.714	−21.752	−35.457	1.00	0.00	C
ATOM	2607	CG2	VAL	A	167	14.710	−19.527	−35.044	1.00	0.00	C
ATOM	2608	H	VAL	A	167	14.650	−23.477	−33.128	1.00	0.00	H
ATOM	2609	HA	VAL	A	167	13.643	−20.715	−32.858	1.00	0.00	H
ATOM	2610	HB	VAL	A	167	15.935	−20.585	−33.682	1.00	0.00	H
ATOM	2611	HG21	VAL	A	167	14.352	−18.832	−34.289	1.00	0.00	H

ATOM 2612 HG22 VAL A 167 13.928 −19.710 −35.781 1.00 0.00 H

ATOM 2613 HG23 VAL A 167 15.573 −19.087 −35.544 1.00 0.00 H

ATOM 2614 HG11 VAL A 167 16.595 −21.289 −35.901 1.00 0.00 H

ATOM 2615 HG12 VAL A 167 16.007 −22.706 −35.021 1.00 0.00 H

ATOM 2616 HG13 VAL A 167 14.975 −21.931 −36.235 1.00 0.00 H

ATOM 2617 N GLY A 168 11.819 −21.123 −34.499 1.00 0.00 N

ATOM 2618 CA GLY A 168 10.718 −21.270 −35.445 1.00 0.00 C

ATOM 2619 C GLY A 168 10.811 −20.262 −36.584 1.00 0.00 C

ATOM 2620 O GLY A 168 11.202 −19.110 −36.383 1.00 0.00 O

ATOM 2621 H GLY A 168 11.868 −20.253 −33.981 1.00 0.00 H

ATOM 2622 HA2 GLY A 168 10.741 −22.264 −35.883 1.00 0.00 H

ATOM 2623 HA3 GLY A 168 9.767 −21.137 −34.930 1.00 0.00 H

ATOM 2624 N PHE A 169 10.417 −20.698 −37.779 1.00 0.00 N

ATOM 2625 CA PHE A 169 10.414 −19.909 −39.007 1.00 0.00 C

ATOM 2626 C PHE A 169 8.964 −19.639 −39.429 1.00 0.00 C

ATOM 2627 O PHE A 169 8.154 −20.566 −39.541 1.00 0.00 O

ATOM 2628 CB PHE A 169 11.165 −20.655 −40.117 1.00 0.00 C

ATOM 2629 CG PHE A 169 12.571 −21.156 −39.803 1.00 0.00 C

ATOM 2630 CD1 PHE A 169 13.682 −20.589 −40.452 1.00 0.00 C

ATOM 2631 CD2 PHE A 169 12.778 −22.253 −38.943 1.00 0.00 C

ATOM 2632 CE1 PHE A 169 14.973 −21.115 −40.257 1.00 0.00 C

ATOM 2633 CE2 PHE A 169 14.065 −22.768 −38.733 1.00 0.00 C

ATOM 2634 CZ PHE A 169 15.166 −22.208 −39.397 1.00 0.00 C

ATOM 2635 H PHE A 169 10.095 −21.659 −37.855 1.00 0.00 H

ATOM 2636 HA PHE A 169 10.919 −18.958 −38.836 1.00 0.00 H

ATOM 2637 HB2 PHE A 169 10.556 −21.505 −40.411 1.00 0.00 H

ATOM 2638 HB3 PHE A 169 11.219 −20.000 −40.985 1.00 0.00 H

ATOM 2639 HD1 PHE A 169 13.542 −19.771 −41.143 1.00 0.00 H

ATOM 2640 HD2 PHE A 169 11.947 −22.725 −38.451 1.00 0.00 H

ATOM 2641 HE1 PHE A 169 15.812 −20.696 −40.794 1.00 0.00 H

ATOM	2642	HE2	PHE	A	169	14.200	−23.606	−38.070	1.00	0.00	H
ATOM	2643	HZ	PHE	A	169	16.153	−22.628	−39.257	1.00	0.00	H
ATOM	2644	N	LEU	A	170	8.610	−18.370	−39.631	1.00	0.00	N
ATOM	2645	CA	LEU	A	170	7.235	−17.944	−39.911	1.00	0.00	C
ATOM	2646	C	LEU	A	170	7.226	−16.895	−41.036	1.00	0.00	C
ATOM	2647	O	LEU	A	170	7.999	−15.938	−40.953	1.00	0.00	O
ATOM	2648	CB	LEU	A	170	6.611	−17.465	−38.587	1.00	0.00	C
ATOM	2649	CG	LEU	A	170	5.220	−16.811	−38.689	1.00	0.00	C
ATOM	2650	CD1	LEU	A	170	4.343	−17.228	−37.507	1.00	0.00	C
ATOM	2651	CD2	LEU	A	170	5.334	−15.287	−38.658	1.00	0.00	C
ATOM	2652	H	LEU	A	170	9.313	−17.648	−39.494	1.00	0.00	H
ATOM	2653	HA	LEU	A	170	6.657	−18.807	−40.236	1.00	0.00	H
ATOM	2654	HB2	LEU	A	170	6.520	−18.348	−37.960	1.00	0.00	H
ATOM	2655	HB3	LEU	A	170	7.298	−16.784	−38.084	1.00	0.00	H
ATOM	2656	HG	LEU	A	170	4.725	−17.125	−39.609	1.00	0.00	H
ATOM	2657	HD21	LEU	A	170	5.849	−14.965	−37.754	1.00	0.00	H
ATOM	2658	HD22	LEU	A	170	5.882	−14.941	−39.532	1.00	0.00	H
ATOM	2659	HD23	LEU	A	170	4.342	−14.844	−38.668	1.00	0.00	H
ATOM	2660	HD11	LEU	A	170	3.366	−16.751	−37.582	1.00	0.00	H
ATOM	2661	HD12	LEU	A	170	4.815	−16.940	−36.567	1.00	0.00	H
ATOM	2662	HD13	LEU	A	170	4.204	−18.310	−37.517	1.00	0.00	H
ATOM	2663	N	PRO	A	171	6.494	−17.104	−42.149	1.00	0.00	N
ATOM	2664	CA	PRO	A	171	6.512	−16.161	−43.262	1.00	0.00	C
ATOM	2665	C	PRO	A	171	6.012	−14.780	−42.819	1.00	0.00	C
ATOM	2666	O	PRO	A	171	5.013	−14.667	−42.111	1.00	0.00	O
ATOM	2667	CB	PRO	A	171	5.670	−16.776	−44.383	1.00	0.00	C
ATOM	2668	CG	PRO	A	171	4.951	−17.976	−43.765	1.00	0.00	C
ATOM	2669	CD	PRO	A	171	5.685	−18.272	−42.458	1.00	0.00	C
ATOM	2670	HA	PRO	A	171	7.535	−16.066	−43.621	1.00	0.00	H
ATOM	2671	HB2	PRO	A	171	4.954	−16.063	−44.792	1.00	0.00	H

ATOM 2672 HB3 PRO A 171 6.330 −17.119 −45.175 1.00 0.00 H
ATOM 2673 HG2 PRO A 171 3.916 −17.719 −43.554 1.00 0.00 H
ATOM 2674 HG3 PRO A 171 4.983 −18.839 −44.432 1.00 0.00 H
ATOM 2675 HD2 PRO A 171 6.336 −19.136 −42.594 1.00 0.00 H
ATOM 2676 HD3 PRO A 171 4.961 −18.470 −41.667 1.00 0.00 H
ATOM 2677 N ARG A 172 6.701 −13.705 −43.221 1.00 0.00 N
ATOM 2678 CA ARG A 172 6.431 −12.338 −42.734 1.00 0.00 C
ATOM 2679 C ARG A 172 5.151 −11.691 −43.289 1.00 0.00 C
ATOM 2680 O ARG A 172 4.867 −10.549 −42.949 1.00 0.00 O
ATOM 2681 CB ARG A 172 7.687 −11.449 −42.829 1.00 0.00 C
ATOM 2682 CG ARG A 172 8.079 −10.972 −44.233 1.00 0.00 C
ATOM 2683 CD ARG A 172 9.132 −9.846 −44.201 1.00 0.00 C
ATOM 2684 NE ARG A 172 10.407 −10.198 −43.527 1.00 0.00 N
ATOM 2685 CZ ARG A 172 10.953 −9.577 −42.489 1.00 0.00 C
ATOM 2686 NH1 ARG A 172 10.243 −8.821 −41.699 1.00 0.00 N
ATOM 2687 NH2 ARG A 172 12.215 −9.714 −42.204 1.00 0.00 N
ATOM 2688 H ARG A 172 7.532 −13.866 −43.784 1.00 0.00 H
ATOM 2689 HA ARG A 172 6.223 −12.440 −41.669 1.00 0.00 H
ATOM 2690 HB2 ARG A 172 7.526 −10.575 −42.195 1.00 0.00 H
ATOM 2691 HB3 ARG A 172 8.530 −12.009 −42.430 1.00 0.00 H
ATOM 2692 HG2 ARG A 172 8.455 −11.814 −44.808 1.00 0.00 H
ATOM 2693 HG3 ARG A 172 7.197 −10.585 −44.742 1.00 0.00 H
ATOM 2694 HD2 ARG A 172 8.684 −8.965 −43.741 1.00 0.00 H
ATOM 2695 HD3 ARG A 172 9.363 −9.581 −45.234 1.00 0.00 H
ATOM 2696 HE ARG A 172 11.009 −10.840 −44.031 1.00 0.00 H
ATOM 2697 HH11 ARG A 172 9.255 −8.798 −41.837 1.00 0.00 H
ATOM 2698 HH12 ARG A 172 10.639 −8.449 −40.841 1.00 0.00 H
ATOM 2699 HH21 ARG A 172 12.810 −10.224 −42.850 1.00 0.00 H
ATOM 2700 HH22 ARG A 172 12.662 −9.093 −41.539 1.00 0.00 H
ATOM 2701 N THR A 173 4.352 −12.439 −44.050 1.00 0.00 N

ATOM 2702 CA THR A 173 2.921 −12.186 −44.302 1.00 0.00 C
ATOM 2703 C THR A 173 2.055 −12.459 −43.067 1.00 0.00 C
ATOM 2704 O THR A 173 1.123 −11.712 −42.792 1.00 0.00 O
ATOM 2705 CB THR A 173 2.430 −13.098 −45.441 1.00 0.00 C
ATOM 2706 OG1 THR A 173 2.840 −14.431 −45.221 1.00 0.00 O
ATOM 2707 CG2 THR A 173 3.014 −12.677 −46.789 1.00 0.00 C
ATOM 2708 H THR A 173 4.659 −13.378 −44.258 1.00 0.00 H
ATOM 2709 HA THR A 173 2.769 −11.146 −44.586 1.00 0.00 H
ATOM 2710 HB THR A 173 1.341 −13.057 −45.503 1.00 0.00 H
ATOM 2711 HG21 THR A 173 2.618 −13.329 −47.568 1.00 0.00 H
ATOM 2712 HG22 THR A 173 2.727 −11.649 −47.009 1.00 0.00 H
ATOM 2713 HG23 THR A 173 4.100 −12.759 −46.774 1.00 0.00 H
ATOM 2714 HG1 THR A 173 2.075 −14.915 −44.813 1.00 0.00 H
ATOM 2715 N GLU A 174 2.429 −13.447 −42.252 1.00 0.00 N
ATOM 2716 CA GLU A 174 1.701 −13.846 −41.041 1.00 0.00 C
ATOM 2717 C GLU A 174 2.125 −13.032 −39.809 1.00 0.00 C
ATOM 2718 O GLU A 174 1.435 −13.051 −38.789 1.00 0.00 O
ATOM 2719 CB GLU A 174 1.941 −15.341 −40.750 1.00 0.00 C
ATOM 2720 CG GLU A 174 1.946 −16.266 −41.972 1.00 0.00 C
ATOM 2721 CD GLU A 174 0.715 −16.072 −42.854 1.00 0.00 C
ATOM 2722 OE1 GLU A 174 −0.327 −16.638 −42.451 1.00 0.00 O
ATOM 2723 OE2 GLU A 174 0.905 −15.677 −44.024 1.00 0.00 O
ATOM 2724 H GLU A 174 3.217 −14.023 −42.529 1.00 0.00 H
ATOM 2725 HA GLU A 174 0.632 −13.687 −41.199 1.00 0.00 H
ATOM 2726 HB2 GLU A 174 1.175 −15.685 −40.053 1.00 0.00 H
ATOM 2727 HB3 GLU A 174 2.906 −15.458 −40.263 1.00 0.00 H
ATOM 2728 HG2 GLU A 174 2.846 −16.071 −42.554 1.00 0.00 H
ATOM 2729 HG3 GLU A 174 1.994 −17.303 −41.628 1.00 0.00 H
ATOM 2730 N PHE A 175 3.310 −12.413 −39.855 1.00 0.00 N
ATOM 2731 CA PHE A 175 3.981 −11.826 −38.696 1.00 0.00 C

ATOM 2732 C PHE A 175 3.301 −10.538 −38.209 1.00 0.00 C

ATOM 2733 O PHE A 175 3.645 −9.430 −38.621 1.00 0.00 O

ATOM 2734 CB PHE A 175 5.469 −11.610 −39.010 1.00 0.00 C

ATOM 2735 CG PHE A 175 6.268 −11.037 −37.851 1.00 0.00 C

ATOM 2736 CD1 PHE A 175 6.228 −11.653 −36.583 1.00 0.00 C

ATOM 2737 CD2 PHE A 175 7.054 −9.884 −38.037 1.00 0.00 C

ATOM 2738 CE1 PHE A 175 6.949 −11.107 −35.507 1.00 0.00 C

ATOM 2739 CE2 PHE A 175 7.789 −9.350 −36.965 1.00 0.00 C

ATOM 2740 CZ PHE A 175 7.728 −9.954 −35.697 1.00 0.00 C

ATOM 2741 H PHE A 175 3.806 −12.439 −40.732 1.00 0.00 H

ATOM 2742 HA PHE A 175 3.919 −12.549 −37.882 1.00 0.00 H

ATOM 2743 HB2 PHE A 175 5.917 −12.562 −39.292 1.00 0.00 H

ATOM 2744 HB3 PHE A 175 5.549 −10.940 −39.867 1.00 0.00 H

ATOM 2745 HD1 PHE A 175 5.634 −12.541 −36.423 1.00 0.00 H

ATOM 2746 HD2 PHE A 175 7.080 −9.401 −39.002 1.00 0.00 H

ATOM 2747 HE1 PHE A 175 6.891 −11.566 −34.530 1.00 0.00 H

ATOM 2748 HE2 PHE A 175 8.382 −8.462 −37.108 1.00 0.00 H

ATOM 2749 HZ PHE A 175 8.261 −9.521 −34.863 1.00 0.00 H

ATOM 2750 N GLN A 176 2.383 −10.694 −37.256 1.00 0.00 N

ATOM 2751 CA GLN A 176 1.738 −9.579 −36.567 1.00 0.00 C

ATOM 2752 C GLN A 176 2.737 −8.891 −35.625 1.00 0.00 C

ATOM 2753 O GLN A 176 3.091 −9.432 −34.579 1.00 0.00 O

ATOM 2754 CB GLN A 176 0.488 −10.068 −35.806 1.00 0.00 C

ATOM 2755 CG GLN A 176 −0.487 −10.906 −36.653 1.00 0.00 C

ATOM 2756 CD GLN A 176 −0.809 −10.270 −38.002 1.00 0.00 C

ATOM 2757 OE1 GLN A 176 −1.278 −9.150 −38.092 1.00 0.00 O

ATOM 2758 NE2 GLN A 176 −0.492 −10.926 −39.095 1.00 0.00 N

ATOM 2759 H GLN A 176 2.120 −11.633 −36.997 1.00 0.00 H

ATOM 2760 HA GLN A 176 1.414 −8.850 −37.313 1.00 0.00 H

ATOM 2761 HB2 GLN A 176 0.797 −10.674 −34.952 1.00 0.00 H

ATOM	2762	HB3	GLN	A	176	−0.042	−9.195	−35.424	1.00	0.00	H
ATOM	2763	HG2	GLN	A	176	−0.061	−11.897	−36.809	1.00	0.00	H
ATOM	2764	HG3	GLN	A	176	−1.416	−11.035	−36.098	1.00	0.00	H
ATOM	2765	HE21	GLN	A	176	0.033	−11.789	−39.025	1.00	0.00	H
ATOM	2766	HE22	GLN	A	176	−0.625	−10.447	−39.967	1.00	0.00	H
ATOM	2767	N	TYR	A	177	3.134	−7.664	−35.963	1.00	0.00	N
ATOM	2768	CA	TYR	A	177	3.960	−6.806	−35.116	1.00	0.00	C
ATOM	2769	C	TYR	A	177	3.527	−5.347	−35.267	1.00	0.00	C
ATOM	2770	O	TYR	A	177	3.466	−4.825	−36.379	1.00	0.00	O
ATOM	2771	CB	TYR	A	177	5.446	−6.992	−35.456	1.00	0.00	C
ATOM	2772	CG	TYR	A	177	6.376	−6.200	−34.552	1.00	0.00	C
ATOM	2773	CD1	TYR	A	177	6.861	−6.783	−33.363	1.00	0.00	C
ATOM	2774	CD2	TYR	A	177	6.705	−4.865	−34.863	1.00	0.00	C
ATOM	2775	CE1	TYR	A	177	7.661	−6.029	−32.482	1.00	0.00	C
ATOM	2776	CE2	TYR	A	177	7.506	−4.112	−33.984	1.00	0.00	C
ATOM	2777	CZ	TYR	A	177	7.970	−4.687	−32.784	1.00	0.00	C
ATOM	2778	OH	TYR	A	177	8.698	−3.943	−31.913	1.00	0.00	O
ATOM	2779	H	TYR	A	177	2.846	−7.297	−36.858	1.00	0.00	H
ATOM	2780	HA	TYR	A	177	3.819	−7.097	−34.074	1.00	0.00	H
ATOM	2781	HB2	TYR	A	177	5.691	−8.051	−35.367	1.00	0.00	H
ATOM	2782	HB3	TYR	A	177	5.621	−6.698	−36.493	1.00	0.00	H
ATOM	2783	HD1	TYR	A	177	6.594	−7.803	−33.115	1.00	0.00	H
ATOM	2784	HD2	TYR	A	177	6.310	−4.399	−35.760	1.00	0.00	H
ATOM	2785	HE1	TYR	A	177	8.015	−6.457	−31.555	1.00	0.00	H
ATOM	2786	HE2	TYR	A	177	7.732	−3.084	−34.212	1.00	0.00	H
ATOM	2787	HH	TYR	A	177	8.548	−3.005	−32.037	1.00	0.00	H
ATOM	2788	N	ASN	A	178	3.233	−4.682	−34.149	1.00	0.00	N
ATOM	2789	CA	ASN	A	178	2.982	−3.245	−34.128	1.00	0.00	C
ATOM	2790	C	ASN	A	178	4.315	−2.486	−34.128	1.00	0.00	C
ATOM	2791	O	ASN	A	178	4.971	−2.394	−33.091	1.00	0.00	O

ATOM	2792	CB	ASN	A	178	2.116	−2.907	−32.903	1.00	0.00	C
ATOM	2793	CG	ASN	A	178	1.811	−1.422	−32.771	1.00	0.00	C
ATOM	2794	OD1	ASN	A	178	1.848	−0.646	−33.716	1.00	0.00	O
ATOM	2795	ND2	ASN	A	178	1.605	−0.944	−31.571	1.00	0.00	N
ATOM	2796	H	ASN	A	178	3.358	−5.150	−33.266	1.00	0.00	H
ATOM	2797	HA	ASN	A	178	2.428	−2.958	−35.024	1.00	0.00	H
ATOM	2798	HB2	ASN	A	178	1.168	−3.440	−32.970	1.00	0.00	H
ATOM	2799	HB3	ASN	A	178	2.627	−3.238	−32.000	1.00	0.00	H
ATOM	2800	HD21	ASN	A	178	1.918	−1.486	−30.769	1.00	0.00	H
ATOM	2801	HD22	ASN	A	178	1.418	0.036	−31.483	1.00	0.00	H
ATOM	2802	N	ASP	A	179	4.616	−1.790	−35.224	1.00	0.00	N
ATOM	2803	CA	ASP	A	179	5.781	−0.901	−35.332	1.00	0.00	C
ATOM	2804	C	ASP	A	179	5.823	0.182	−34.234	1.00	0.00	C
ATOM	2805	O	ASP	A	179	6.901	0.489	−33.732	1.00	0.00	O
ATOM	2806	CB	ASP	A	179	5.811	−0.257	−36.723	1.00	0.00	C
ATOM	2807	CG	ASP	A	179	6.158	−1.207	−37.861	1.00	0.00	C
ATOM	2808	OD1	ASP	A	179	6.438	−2.407	−37.647	1.00	0.00	O
ATOM	2809	OD2	ASP	A	179	6.246	−0.712	−39.003	1.00	0.00	O
ATOM	2810	H	ASP	A	179	4.087	−1.975	−36.064	1.00	0.00	H
ATOM	2811	HA	ASP	A	179	6.691	−1.491	−35.234	1.00	0.00	H
ATOM	2812	HB2	ASP	A	179	4.842	0.196	−36.931	1.00	0.00	H
ATOM	2813	HB3	ASP	A	179	6.553	0.537	−36.728	1.00	0.00	H
ATOM	2814	N	SER	A	180	4.669	0.594	−33.692	1.00	0.00	N
ATOM	2815	CA	SER	A	180	4.578	1.555	−32.575	1.00	0.00	C
ATOM	2816	C	SER	A	180	5.143	1.023	−31.245	1.00	0.00	C
ATOM	2817	O	SER	A	180	5.414	1.814	−30.346	1.00	0.00	O
ATOM	2818	CB	SER	A	180	3.135	2.033	−32.337	1.00	0.00	C
ATOM	2819	OG	SER	A	180	2.353	2.057	−33.519	1.00	0.00	O
ATOM	2820	H	SER	A	180	3.809	0.241	−34.087	1.00	0.00	H
ATOM	2821	HA	SER	A	180	5.170	2.433	−32.838	1.00	0.00	H

ATOM	2822	HB2	SER	A	180	2.651	1.389	−31.608	1.00	0.00	H
ATOM	2823	HB3	SER	A	180	3.166	3.037	−31.912	1.00	0.00	H
ATOM	2824	HG	SER	A	180	2.073	1.147	−33.727	1.00	0.00	H
ATOM	2825	N	ASN	A	181	5.360	−0.294	−31.110	1.00	0.00	N
ATOM	2826	CA	ASN	A	181	6.080	−0.889	−29.977	1.00	0.00	C
ATOM	2827	C	ASN	A	181	7.609	−0.696	−30.074	1.00	0.00	C
ATOM	2828	O	ASN	A	181	8.325	−1.043	−29.136	1.00	0.00	O
ATOM	2829	CB	ASN	A	181	5.755	−2.398	−29.866	1.00	0.00	C
ATOM	2830	CG	ASN	A	181	4.333	−2.748	−29.453	1.00	0.00	C
ATOM	2831	OD1	ASN	A	181	3.362	−2.050	−29.695	1.00	0.00	O
ATOM	2832	ND2	ASN	A	181	4.150	−3.883	−28.819	1.00	0.00	N
ATOM	2833	H	ASN	A	181	5.129	−0.897	−31.892	1.00	0.00	H
ATOM	2834	HA	ASN	A	181	5.759	−0.398	−29.057	1.00	0.00	H
ATOM	2835	HB2	ASN	A	181	5.977	−2.900	−30.806	1.00	0.00	H
ATOM	2836	HB3	ASN	A	181	6.419	−2.825	−29.114	1.00	0.00	H
ATOM	2837	HD21	ASN	A	181	4.931	−4.471	−28.585	1.00	0.00	H
ATOM	2838	HD22	ASN	A	181	3.217	−4.090	−28.514	1.00	0.00	H
ATOM	2839	N	CYS	A	182	8.142	−0.330	−31.246	1.00	0.00	N
ATOM	2840	CA	CYS	A	182	9.580	−0.286	−31.497	1.00	0.00	C
ATOM	2841	C	CYS	A	182	10.253	0.877	−30.720	1.00	0.00	C
ATOM	2842	O	CYS	A	182	9.856	2.022	−30.940	1.00	0.00	O
ATOM	2843	CB	CYS	A	182	9.796	−0.203	−33.014	1.00	0.00	C
ATOM	2844	SG	CYS	A	182	11.476	−0.555	−33.576	1.00	0.00	S
ATOM	2845	H	CYS	A	182	7.524	−0.020	−31.990	1.00	0.00	H
ATOM	2846	HA	CYS	A	182	9.986	−1.241	−31.181	1.00	0.00	H
ATOM	2847	HB2	CYS	A	182	9.129	−0.910	−33.509	1.00	0.00	H
ATOM	2848	HB3	CYS	A	182	9.515	0.795	−33.352	1.00	0.00	H
ATOM	2849	N	PRO	A	183	11.278	0.652	−29.860	1.00	0.00	N
ATOM	2850	CA	PRO	A	183	11.807	1.655	−28.904	1.00	0.00	C
ATOM	2851	C	PRO	A	183	12.537	2.911	−29.444	1.00	0.00	C

ATOM 2852 O PRO A 183 13.381 3.477 −28.752 1.00 0.00 O
ATOM 2853 CB PRO A 183 12.718 0.874 −27.945 1.00 0.00 C
ATOM 2854 CG PRO A 183 12.134 −0.530 −27.966 1.00 0.00 C
ATOM 2855 CD PRO A 183 11.715 −0.676 −29.423 1.00 0.00 C
ATOM 2856 HA PRO A 183 10.952 2.016 −28.331 1.00 0.00 H
ATOM 2857 HB2 PRO A 183 12.705 1.300 −26.940 1.00 0.00 H
ATOM 2858 HB3 PRO A 183 13.737 0.842 −28.334 1.00 0.00 H
ATOM 2859 HG2 PRO A 183 12.869 −1.283 −27.677 1.00 0.00 H
ATOM 2860 HG3 PRO A 183 11.253 −0.576 −27.323 1.00 0.00 H
ATOM 2861 HD2 PRO A 183 10.923 −1.421 −29.462 1.00 0.00 H
ATOM 2862 HD3 PRO A 183 12.563 −1.002 −30.024 1.00 0.00 H
ATOM 2863 N ILE A 184 12.220 3.398 −30.643 1.00 0.00 N
ATOM 2864 CA ILE A 184 12.958 4.432 −31.395 1.00 0.00 C
ATOM 2865 C ILE A 184 12.998 5.847 −30.775 1.00 0.00 C
ATOM 2866 O ILE A 184 13.517 6.759 −31.408 1.00 0.00 O
ATOM 2867 CB ILE A 184 12.403 4.515 −32.837 1.00 0.00 C
ATOM 2868 CG1 ILE A 184 10.954 5.064 −32.857 1.00 0.00 C
ATOM 2869 CG2 ILE A 184 12.518 3.153 −33.545 1.00 0.00 C
ATOM 2870 CD1 ILE A 184 10.458 5.442 −34.253 1.00 0.00 C
ATOM 2871 H ILE A 184 11.435 2.960 −31.114 1.00 0.00 H
ATOM 2872 HA ILE A 184 13.996 4.105 −31.458 1.00 0.00 H
ATOM 2873 HB ILE A 184 13.034 5.212 −33.393 1.00 0.00 H
ATOM 2874 HG12 ILE A 184 10.274 4.326 −32.431 1.00 0.00 H
ATOM 2875 HG13 ILE A 184 10.891 5.966 −32.249 1.00 0.00 H
ATOM 2876 HD11 ILE A 184 9.459 5.873 −34.178 1.00 0.00 H
ATOM 2877 HD12 ILE A 184 11.129 6.174 −34.703 1.00 0.00 H
ATOM 2878 HD13 ILE A 184 10.410 4.556 −34.877 1.00 0.00 H
ATOM 2879 HG21 ILE A 184 13.522 2.749 −33.417 1.00 0.00 H
ATOM 2880 HG22 ILE A 184 12.348 3.284 −34.610 1.00 0.00 H
ATOM 2881 HG23 ILE A 184 11.787 2.448 −33.152 1.00 0.00 H

ATOM 2882 N ILE A 185 12.392 6.090 −29.610 1.00 0.00 N
ATOM 2883 CA ILE A 185 12.001 7.441 −29.146 1.00 0.00 C
ATOM 2884 C ILE A 185 13.188 8.421 −29.028 1.00 0.00 C
ATOM 2885 O ILE A 185 13.058 9.583 −29.403 1.00 0.00 O
ATOM 2886 CB ILE A 185 11.196 7.326 −27.825 1.00 0.00 C
ATOM 2887 CG1 ILE A 185 9.871 6.565 −28.090 1.00 0.00 C
ATOM 2888 CG2 ILE A 185 10.911 8.706 −27.199 1.00 0.00 C
ATOM 2889 CD1 ILE A 185 9.036 6.268 −26.837 1.00 0.00 C
ATOM 2890 H ILE A 185 12.039 5.288 −29.108 1.00 0.00 H
ATOM 2891 HA ILE A 185 11.339 7.873 −29.898 1.00 0.00 H
ATOM 2892 HB ILE A 185 11.790 6.753 −27.111 1.00 0.00 H
ATOM 2893 HG12 ILE A 185 9.260 7.138 −28.789 1.00 0.00 H
ATOM 2894 HG13 ILE A 185 10.092 5.604 −28.554 1.00 0.00 H
ATOM 2895 HG21 ILE A 185 11.843 9.214 −26.946 1.00 0.00 H
ATOM 2896 HG22 ILE A 185 10.346 8.603 −26.275 1.00 0.00 H
ATOM 2897 HG23 ILE A 185 10.349 9.326 −27.899 1.00 0.00 H
ATOM 2898 HD11 ILE A 185 8.212 5.606 −27.106 1.00 0.00 H
ATOM 2899 HD12 ILE A 185 8.616 7.187 −26.429 1.00 0.00 H
ATOM 2900 HD13 ILE A 185 9.654 5.778 −26.084 1.00 0.00 H
ATOM 2901 N HIS A 186 14.368 7.941 −28.626 1.00 0.00 N
ATOM 2902 CA HIS A 186 15.611 8.740 −28.572 1.00 0.00 C
ATOM 2903 C HIS A 186 16.465 8.635 −29.852 1.00 0.00 C
ATOM 2904 O HIS A 186 17.497 9.294 −29.995 1.00 0.00 O
ATOM 2905 CB HIS A 186 16.415 8.312 −27.335 1.00 0.00 C
ATOM 2906 CG HIS A 186 15.611 8.358 −26.059 1.00 0.00 C
ATOM 2907 ND1 HIS A 186 15.133 9.520 −25.452 1.00 0.00 N
ATOM 2908 CD2 HIS A 186 15.178 7.278 −25.345 1.00 0.00 C
ATOM 2909 CE1 HIS A 186 14.431 9.118 −24.384 1.00 0.00 C
ATOM 2910 NE2 HIS A 186 14.440 7.776 −24.293 1.00 0.00 N
ATOM 2911 H HIS A 186 14.411 6.969 −28.363 1.00 0.00 H

ATOM 2912 HA HIS A 186 15.350 9.793 −28.449 1.00 0.00 H
ATOM 2913 HB2 HIS A 186 17.275 8.974 −27.222 1.00 0.00 H
ATOM 2914 HB3 HIS A 186 16.787 7.297 −27.482 1.00 0.00 H
ATOM 2915 HD2 HIS A 186 15.374 6.240 −25.575 1.00 0.00 H
ATOM 2916 HE1 HIS A 186 13.930 9.786 −23.696 1.00 0.00 H
ATOM 2917 HE2 HIS A 186 13.986 7.236 −23.571 1.00 0.00 H
ATOM 2918 N CYS A 187 16.106 7.719 −30.747 1.00 0.00 N
ATOM 2919 CA CYS A 187 16.943 7.215 −31.828 1.00 0.00 C
ATOM 2920 C CYS A 187 16.730 8.024 −33.110 1.00 0.00 C
ATOM 2921 O CYS A 187 15.987 7.623 −33.999 1.00 0.00 O
ATOM 2922 CB CYS A 187 16.640 5.727 −31.996 1.00 0.00 C
ATOM 2923 SG CYS A 187 16.989 4.785 −30.494 1.00 0.00 S
ATOM 2924 H CYS A 187 15.189 7.300 −30.651 1.00 0.00 H
ATOM 2925 HA CYS A 187 17.992 7.304 −31.539 1.00 0.00 H
ATOM 2926 HB2 CYS A 187 15.593 5.603 −32.266 1.00 0.00 H
ATOM 2927 HB3 CYS A 187 17.245 5.326 −32.810 1.00 0.00 H
ATOM 2928 N LYS A 188 17.468 9.137 −33.223 1.00 0.00 N
ATOM 2929 CA LYS A 188 17.288 10.247 −34.190 1.00 0.00 C
ATOM 2930 C LYS A 188 17.003 9.892 −35.669 1.00 0.00 C
ATOM 2931 O LYS A 188 16.627 10.788 −36.419 1.00 0.00 O
ATOM 2932 CB LYS A 188 18.555 11.125 −34.132 1.00 0.00 C
ATOM 2933 CG LYS A 188 18.271 12.622 −34.346 1.00 0.00 C
ATOM 2934 CD LYS A 188 19.472 13.439 −34.854 1.00 0.00 C
ATOM 2935 CE LYS A 188 20.773 13.279 −34.050 1.00 0.00 C
ATOM 2936 NZ LYS A 188 21.578 12.114 −34.501 1.00 0.00 N
ATOM 2937 H LYS A 188 17.979 9.359 −32.377 1.00 0.00 H
ATOM 2938 HA LYS A 188 16.429 10.831 −33.854 1.00 0.00 H
ATOM 2939 HB2 LYS A 188 19.038 11.019 −33.158 1.00 0.00 H
ATOM 2940 HB3 LYS A 188 19.246 10.766 −34.897 1.00 0.00 H
ATOM 2941 HG2 LYS A 188 17.929 13.045 −33.400 1.00 0.00 H

ATOM	2942	HG3	LYS	A	188	17.465	12.750	−35.067	1.00	0.00	H
ATOM	2943	HD2	LYS	A	188	19.654	13.195	−35.900	1.00	0.00	H
ATOM	2944	HD3	LYS	A	188	19.182	14.490	−34.821	1.00	0.00	H
ATOM	2945	HE2	LYS	A	188	21.364	14.190	−34.184	1.00	0.00	H
ATOM	2946	HE3	LYS	A	188	20.525	13.196	−32.987	1.00	0.00	H
ATOM	2947	HZ1	LYS	A	188	22.433	12.032	−33.968	1.00	0.00	H
ATOM	2948	HZ2	LYS	A	188	21.825	12.216	−35.484	1.00	0.00	H
ATOM	2949	HZ3	LYS	A	188	21.054	11.254	−34.408	1.00	0.00	H
ATOM	2950	N	TYR	A	189	17.363	8.693	−36.128	1.00	0.00	N
ATOM	2951	CA	TYR	A	189	17.205	8.242	−37.521	1.00	0.00	C
ATOM	2952	C	TYR	A	189	16.382	6.947	−37.672	1.00	0.00	C
ATOM	2953	O	TYR	A	189	16.008	6.594	−38.790	1.00	0.00	O
ATOM	2954	CB	TYR	A	189	18.598	8.056	−38.152	1.00	0.00	C
ATOM	2955	CG	TYR	A	189	19.576	9.209	−37.974	1.00	0.00	C
ATOM	2956	CD1	TYR	A	189	19.188	10.530	−38.274	1.00	0.00	C
ATOM	2957	CD2	TYR	A	189	20.883	8.952	−37.514	1.00	0.00	C
ATOM	2958	CE1	TYR	A	189	20.093	11.594	−38.094	1.00	0.00	C
ATOM	2959	CE2	TYR	A	189	21.793	10.011	−37.333	1.00	0.00	C
ATOM	2960	CZ	TYR	A	189	21.396	11.331	−37.625	1.00	0.00	C
ATOM	2961	OH	TYR	A	189	22.249	12.355	−37.364	1.00	0.00	O
ATOM	2962	H	TYR	A	189	17.625	8.005	−35.441	1.00	0.00	H
ATOM	2963	HA	TYR	A	189	16.673	9.004	−38.090	1.00	0.00	H
ATOM	2964	HB2	TYR	A	189	18.473	7.878	−39.221	1.00	0.00	H
ATOM	2965	HB3	TYR	A	189	19.041	7.153	−37.726	1.00	0.00	H
ATOM	2966	HD1	TYR	A	189	18.185	10.729	−38.630	1.00	0.00	H
ATOM	2967	HD2	TYR	A	189	21.192	7.932	−37.316	1.00	0.00	H
ATOM	2968	HE1	TYR	A	189	19.784	12.605	−38.316	1.00	0.00	H
ATOM	2969	HE2	TYR	A	189	22.800	9.806	−36.999	1.00	0.00	H
ATOM	2970	HH	TYR	A	189	22.741	12.588	−38.161	1.00	0.00	H
ATOM	2971	N	SER	A	190	16.131	6.226	−36.577	1.00	0.00	N

ATOM 2972 CA SER A 190 15.590 4.867 −36.598 1.00 0.00 C
ATOM 2973 C SER A 190 14.095 4.848 −36.899 1.00 0.00 C
ATOM 2974 O SER A 190 13.304 5.553 −36.271 1.00 0.00 O
ATOM 2975 CB SER A 190 15.873 4.168 −35.269 1.00 0.00 C
ATOM 2976 OG SER A 190 17.270 4.118 −35.054 1.00 0.00 O
ATOM 2977 H SER A 190 16.345 6.610 −35.667 1.00 0.00 H
ATOM 2978 HA SER A 190 16.098 4.310 −37.385 1.00 0.00 H
ATOM 2979 HB2 SER A 190 15.464 3.160 −35.295 1.00 0.00 H
ATOM 2980 HB3 SER A 190 15.396 4.719 −34.458 1.00 0.00 H
ATOM 2981 HG SER A 190 17.654 3.627 −35.827 1.00 0.00 H
ATOM 2982 N LYS A 191 13.677 3.990 −37.833 1.00 0.00 N
ATOM 2983 CA LYS A 191 12.264 3.831 −38.199 1.00 0.00 C
ATOM 2984 C LYS A 191 11.568 2.893 −37.218 1.00 0.00 C
ATOM 2985 O LYS A 191 12.111 1.845 −36.884 1.00 0.00 O
ATOM 2986 CB LYS A 191 12.112 3.321 −39.641 1.00 0.00 C
ATOM 2987 CG LYS A 191 12.939 4.121 −40.664 1.00 0.00 C
ATOM 2988 CD LYS A 191 12.654 3.665 −42.100 1.00 0.00 C
ATOM 2989 CE LYS A 191 11.297 4.189 −42.585 1.00 0.00 C
ATOM 2990 NZ LYS A 191 10.905 3.574 −43.874 1.00 0.00 N
ATOM 2991 H LYS A 191 14.360 3.343 −38.215 1.00 0.00 H
ATOM 2992 HA LYS A 191 11.787 4.810 −38.122 1.00 0.00 H
ATOM 2993 HB2 LYS A 191 12.415 2.276 −39.685 1.00 0.00 H
ATOM 2994 HB3 LYS A 191 11.056 3.371 −39.905 1.00 0.00 H
ATOM 2995 HG2 LYS A 191 12.722 5.186 −40.566 1.00 0.00 H
ATOM 2996 HG3 LYS A 191 14.001 3.964 −40.464 1.00 0.00 H
ATOM 2997 HD2 LYS A 191 13.442 4.051 −42.750 1.00 0.00 H
ATOM 2998 HD3 LYS A 191 12.676 2.573 −42.138 1.00 0.00 H
ATOM 2999 HE2 LYS A 191 11.359 5.277 −42.679 1.00 0.00 H
ATOM 3000 HE3 LYS A 191 10.535 3.954 −41.836 1.00 0.00 H
ATOM 3001 HZ1 LYS A 191 10.055 3.980 −44.235 1.00 0.00 H

ATOM	3002	HZ2	LYS	A	191	11.641	3.610	−44.560	1.00	0.00	H
ATOM	3003	HZ3	LYS	A	191	10.650	2.590	−43.717	1.00	0.00	H
ATOM	3004	N	ALA	A	192	10.265	3.094	−37.037	1.00	0.00	N
ATOM	3005	CA	ALA	A	192	9.406	2.165	−36.295	1.00	0.00	C
ATOM	3006	C	ALA	A	192	9.332	0.763	−36.950	1.00	0.00	C
ATOM	3007	O	ALA	A	192	9.303	−0.252	−36.257	1.00	0.00	O
ATOM	3008	CB	ALA	A	192	8.033	2.837	−36.171	1.00	0.00	C
ATOM	3009	H	ALA	A	192	9.870	3.969	−37.335	1.00	0.00	H
ATOM	3010	HA	ALA	A	192	9.817	2.033	−35.292	1.00	0.00	H
ATOM	3011	HB1	ALA	A	192	7.403	2.260	−35.498	1.00	0.00	H
ATOM	3012	HB2	ALA	A	192	7.551	2.900	−37.148	1.00	0.00	H
ATOM	3013	HB3	ALA	A	192	8.141	3.838	−35.753	1.00	0.00	H
ATOM	3014	N	GLU	A	193	9.560	0.701	−38.268	1.00	0.00	N
ATOM	3015	CA	GLU	A	193	9.720	−0.543	−39.030	1.00	0.00	C
ATOM	3016	C	GLU	A	193	10.927	−1.393	−38.602	1.00	0.00	C
ATOM	3017	O	GLU	A	193	10.941	−2.596	−38.856	1.00	0.00	O
ATOM	3018	CB	GLU	A	193	9.937	−0.226	−40.517	1.00	0.00	C
ATOM	3019	CG	GLU	A	193	8.928	0.727	−41.166	1.00	0.00	C
ATOM	3020	CD	GLU	A	193	8.927	0.524	−42.686	1.00	0.00	C
ATOM	3021	OE1	GLU	A	193	8.488	−0.569	−43.126	1.00	0.00	O
ATOM	3022	OE2	GLU	A	193	9.553	1.363	−43.372	1.00	0.00	O
ATOM	3023	H	GLU	A	193	9.592	1.566	−38.777	1.00	0.00	H
ATOM	3024	HA	GLU	A	193	8.822	−1.152	−38.918	1.00	0.00	H
ATOM	3025	HB2	GLU	A	193	10.934	0.198	−40.655	1.00	0.00	H
ATOM	3026	HB3	GLU	A	193	9.908	−1.178	−41.047	1.00	0.00	H
ATOM	3027	HG2	GLU	A	193	7.929	0.531	−40.779	1.00	0.00	H
ATOM	3028	HG3	GLU	A	193	9.188	1.760	−40.914	1.00	0.00	H
ATOM	3029	N	ASN	A	194	11.999	−0.778	−38.088	1.00	0.00	N
ATOM	3030	CA	ASN	A	194	13.317	−1.412	−37.983	1.00	0.00	C
ATOM	3031	C	ASN	A	194	13.295	−2.685	−37.127	1.00	0.00	C

ATOM 3032 O ASN A 194 13.847 −3.702 −37.548 1.00 0.00 O
ATOM 3033 CB ASN A 194 14.334 −0.401 −37.432 1.00 0.00 C
ATOM 3034 CG ASN A 194 14.736 0.694 −38.409 1.00 0.00 C
ATOM 3035 OD1 ASN A 194 14.377 0.694 −39.578 1.00 0.00 O
ATOM 3036 ND2 ASN A 194 15.578 1.597 −37.972 1.00 0.00 N
ATOM 3037 H ASN A 194 11.936 0.209 −37.867 1.00 0.00 H
ATOM 3038 HA ASN A 194 13.638 −1.712 −38.981 1.00 0.00 H
ATOM 3039 HB2 ASN A 194 13.953 0.057 −36.520 1.00 0.00 H
ATOM 3040 HB3 ASN A 194 15.240 −0.942 −37.171 1.00 0.00 H
ATOM 3041 HD21 ASN A 194 15.926 1.499 −37.019 1.00 0.00 H
ATOM 3042 HD22 ASN A 194 16.283 1.933 −38.624 1.00 0.00 H
ATOM 3043 N CYS A 195 12.455 −2.704 −36.085 1.00 0.00 N
ATOM 3044 CA CYS A 195 12.134 −3.904 −35.319 1.00 0.00 C
ATOM 3045 C CYS A 195 11.551 −5.013 −36.213 1.00 0.00 C
ATOM 3046 O CYS A 195 12.182 −6.054 −36.388 1.00 0.00 O
ATOM 3047 CB CYS A 195 11.164 −3.548 −34.183 1.00 0.00 C
ATOM 3048 SG CYS A 195 11.826 −2.451 −32.905 1.00 0.00 S
ATOM 3049 H CYS A 195 11.987 −1.841 −35.844 1.00 0.00 H
ATOM 3050 HA CYS A 195 13.053 −4.289 −34.877 1.00 0.00 H
ATOM 3051 HB2 CYS A 195 10.872 −4.475 −33.688 1.00 0.00 H
ATOM 3052 HB3 CYS A 195 10.262 −3.095 −34.596 1.00 0.00 H
ATOM 3053 N ARG A 196 10.420 −4.751 −36.883 1.00 0.00 N
ATOM 3054 CA ARG A 196 9.716 −5.702 −37.764 1.00 0.00 C
ATOM 3055 C ARG A 196 10.585 −6.213 −38.916 1.00 0.00 C
ATOM 3056 O ARG A 196 10.500 −7.387 −39.285 1.00 0.00 O
ATOM 3057 CB ARG A 196 8.444 −5.004 −38.282 1.00 0.00 C
ATOM 3058 CG ARG A 196 7.640 −5.814 −39.317 1.00 0.00 C
ATOM 3059 CD ARG A 196 6.301 −5.154 −39.682 1.00 0.00 C
ATOM 3060 NE ARG A 196 6.446 −3.741 −40.066 1.00 0.00 N
ATOM 3061 CZ ARG A 196 6.895 −3.205 −41.180 1.00 0.00 C

ATOM	3062	NH1	ARG	A	196	7.261	−3.903	−42.213	1.00	0.00	N
ATOM	3063	NH2	ARG	A	196	6.977	−1.924	−41.282	1.00	0.00	N
ATOM	3064	H	ARG	A	196	10.031	−3.820	−36.799	1.00	0.00	H
ATOM	3065	HA	ARG	A	196	9.426	−6.570	−37.175	1.00	0.00	H
ATOM	3066	HB2	ARG	A	196	7.798	−4.790	−37.432	1.00	0.00	H
ATOM	3067	HB3	ARG	A	196	8.725	−4.051	−38.733	1.00	0.00	H
ATOM	3068	HG2	ARG	A	196	8.229	−5.930	−40.227	1.00	0.00	H
ATOM	3069	HG3	ARG	A	196	7.429	−6.803	−38.911	1.00	0.00	H
ATOM	3070	HD2	ARG	A	196	5.648	−5.189	−38.807	1.00	0.00	H
ATOM	3071	HD3	ARG	A	196	5.813	−5.714	−40.481	1.00	0.00	H
ATOM	3072	HE	ARG	A	196	6.292	−3.071	−39.305	1.00	0.00	H
ATOM	3073	HH11	ARG	A	196	7.090	−4.884	−42.204	1.00	0.00	H
ATOM	3074	HH12	ARG	A	196	7.563	−3.401	−43.028	1.00	0.00	H
ATOM	3075	HH21	ARG	A	196	6.688	−1.368	−40.472	1.00	0.00	H
ATOM	3076	HH22	ARG	A	196	7.436	−1.455	−42.062	1.00	0.00	H
ATOM	3077	N	LEU	A	197	11.412	−5.347	−39.496	1.00	0.00	N
ATOM	3078	CA	LEU	A	197	12.327	−5.696	−40.582	1.00	0.00	C
ATOM	3079	C	LEU	A	197	13.477	−6.595	−40.096	1.00	0.00	C
ATOM	3080	O	LEU	A	197	13.667	−7.663	−40.682	1.00	0.00	O
ATOM	3081	CB	LEU	A	197	12.843	−4.406	−41.246	1.00	0.00	C
ATOM	3082	CG	LEU	A	197	11.758	−3.560	−41.947	1.00	0.00	C
ATOM	3083	CD1	LEU	A	197	12.386	−2.273	−42.481	1.00	0.00	C
ATOM	3084	CD2	LEU	A	197	11.077	−4.288	−43.109	1.00	0.00	C
ATOM	3085	H	LEU	A	197	11.393	−4.387	−39.159	1.00	0.00	H
ATOM	3086	HA	LEU	A	197	11.783	−6.273	−41.329	1.00	0.00	H
ATOM	3087	HB2	LEU	A	197	13.316	−3.794	−40.478	1.00	0.00	H
ATOM	3088	HB3	LEU	A	197	13.605	−4.671	−41.978	1.00	0.00	H
ATOM	3089	HG	LEU	A	197	10.983	−3.293	−41.236	1.00	0.00	H
ATOM	3090	HD11	LEU	A	197	13.163	−2.500	−43.210	1.00	0.00	H
ATOM	3091	HD12	LEU	A	197	12.819	−1.708	−41.654	1.00	0.00	H

ATOM	3092	HD13	LEU	A	197	11.617	−1.656	−42.948	1.00	0.00	H
ATOM	3093	HD21	LEU	A	197	10.374	−3.608	−43.592	1.00	0.00	H
ATOM	3094	HD22	LEU	A	197	11.810	−4.619	−43.839	1.00	0.00	H
ATOM	3095	HD23	LEU	A	197	10.518	−5.145	−42.739	1.00	0.00	H
ATOM	3096	N	SER	A	198	14.107	−6.260	−38.961	1.00	0.00	N
ATOM	3097	CA	SER	A	198	15.251	−6.986	−38.379	1.00	0.00	C
ATOM	3098	C	SER	A	198	14.941	−8.426	−37.955	1.00	0.00	C
ATOM	3099	O	SER	A	198	15.838	−9.256	−38.021	1.00	0.00	O
ATOM	3100	CB	SER	A	198	15.818	−6.197	−37.186	1.00	0.00	C
ATOM	3101	OG	SER	A	198	16.835	−6.905	−36.490	1.00	0.00	O
ATOM	3102	H	SER	A	198	13.827	−5.400	−38.495	1.00	0.00	H
ATOM	3103	HA	SER	A	198	16.036	−7.048	−39.133	1.00	0.00	H
ATOM	3104	HB2	SER	A	198	16.217	−5.248	−37.544	1.00	0.00	H
ATOM	3105	HB3	SER	A	198	15.008	−5.986	−36.485	1.00	0.00	H
ATOM	3106	HG	SER	A	198	17.565	−7.113	−37.108	1.00	0.00	H
ATOM	3107	N	MET	A	199	13.676	−8.795	−37.708	1.00	0.00	N
ATOM	3108	CA	MET	A	199	13.304	−10.161	−37.283	1.00	0.00	C
ATOM	3109	C	MET	A	199	13.583	−11.284	−38.309	1.00	0.00	C
ATOM	3110	O	MET	A	199	13.324	−12.448	−38.011	1.00	0.00	O
ATOM	3111	CB	MET	A	199	11.833	−10.229	−36.832	1.00	0.00	C
ATOM	3112	CG	MET	A	199	11.420	−9.198	−35.782	1.00	0.00	C
ATOM	3113	SD	MET	A	199	12.495	−9.055	−34.328	1.00	0.00	S
ATOM	3114	CE	MET	A	199	11.635	−7.723	−33.450	1.00	0.00	C
ATOM	3115	H	MET	A	199	12.984	−8.061	−37.633	1.00	0.00	H
ATOM	3116	HA	MET	A	199	13.914	−10.398	−36.413	1.00	0.00	H
ATOM	3117	HB2	MET	A	199	11.193	−10.096	−37.702	1.00	0.00	H
ATOM	3118	HB3	MET	A	199	11.643	−11.219	−36.413	1.00	0.00	H
ATOM	3119	HG2	MET	A	199	11.348	−8.232	−36.269	1.00	0.00	H
ATOM	3120	HG3	MET	A	199	10.423	−9.458	−35.435	1.00	0.00	H
ATOM	3121	HE1	MET	A	199	11.651	−6.816	−34.049	1.00	0.00	H

ATOM	3122	HE2	MET	A	199	12.135	−7.528	−32.504	1.00	0.00	H
ATOM	3123	HE3	MET	A	199	10.602	−8.010	−33.260	1.00	0.00	H
ATOM	3124	N	GLY	A	200	14.090	−10.986	−39.510	1.00	0.00	N
ATOM	3125	CA	GLY	A	200	14.535	−11.989	−40.483	1.00	0.00	C
ATOM	3126	C	GLY	A	200	15.872	−11.627	−41.135	1.00	0.00	C
ATOM	3127	O	GLY	A	200	16.190	−10.452	−41.289	1.00	0.00	O
ATOM	3128	H	GLY	A	200	14.437	−10.043	−39.638	1.00	0.00	H
ATOM	3129	HA2	GLY	A	200	14.650	−12.957	−39.994	1.00	0.00	H
ATOM	3130	HA3	GLY	A	200	13.784	−12.093	−41.264	1.00	0.00	H
ATOM	3131	N	VAL	A	201	16.570	−12.642	−41.661	1.00	0.00	N
ATOM	3132	CA	VAL	A	201	17.931	−12.569	−42.255	1.00	0.00	C
ATOM	3133	C	VAL	A	201	18.082	−11.520	−43.374	1.00	0.00	C
ATOM	3134	O	VAL	A	201	19.172	−11.009	−43.616	1.00	0.00	O
ATOM	3135	CB	VAL	A	201	18.325	−13.981	−42.756	1.00	0.00	C
ATOM	3136	CG1	VAL	A	201	19.585	−14.037	−43.629	1.00	0.00	C
ATOM	3137	CG2	VAL	A	201	18.532	−14.928	−41.567	1.00	0.00	C
ATOM	3138	H	VAL	A	201	16.219	−13.569	−41.474	1.00	0.00	H
ATOM	3139	HA	VAL	A	201	18.637	−12.280	−41.474	1.00	0.00	H
ATOM	3140	HB	VAL	A	201	17.502	−14.371	−43.357	1.00	0.00	H
ATOM	3141	HG11	VAL	A	201	19.409	−13.547	−44.585	1.00	0.00	H
ATOM	3142	HG12	VAL	A	201	19.862	−15.074	−43.823	1.00	0.00	H
ATOM	3143	HG13	VAL	A	201	20.410	−13.540	−43.121	1.00	0.00	H
ATOM	3144	HG21	VAL	A	201	18.754	−15.933	−41.929	1.00	0.00	H
ATOM	3145	HG22	VAL	A	201	17.640	−14.978	−40.946	1.00	0.00	H
ATOM	3146	HG23	VAL	A	201	19.370	−14.585	−40.957	1.00	0.00	H
ATOM	3147	N	ASN	A	202	16.979	−11.096	−43.994	1.00	0.00	N
ATOM	3148	CA	ASN	A	202	16.870	−9.793	−44.650	1.00	0.00	C
ATOM	3149	C	ASN	A	202	15.631	−9.063	−44.101	1.00	0.00	C
ATOM	3150	O	ASN	A	202	14.590	−9.697	−43.926	1.00	0.00	O
ATOM	3151	CB	ASN	A	202	16.735	−9.944	−46.180	1.00	0.00	C

ATOM 3152 CG ASN A 202 17.873 −10.554 −46.983 1.00 0.00 C
ATOM 3153 OD1 ASN A 202 17.722 −10.733 −48.180 1.00 0.00 O
ATOM 3154 ND2 ASN A 202 19.032 −10.814 −46.431 1.00 0.00 N
ATOM 3155 H ASN A 202 16.115 −11.462 −43.624 1.00 0.00 H
ATOM 3156 HA ASN A 202 17.746 −9.182 −44.418 1.00 0.00 H
ATOM 3157 HB2 ASN A 202 16.587 −8.950 −46.595 1.00 0.00 H
ATOM 3158 HB3 ASN A 202 15.851 −10.539 −46.393 1.00 0.00 H
ATOM 3159 HD21 ASN A 202 19.178 −10.720 −45.429 1.00 0.00 H
ATOM 3160 HD22 ASN A 202 19.747 −11.200 −47.020 1.00 0.00 H
ATOM 3161 N SER A 203 15.602 −7.739 −44.270 1.00 0.00 N
ATOM 3162 CA SER A 203 14.370 −6.929 −44.221 1.00 0.00 C
ATOM 3163 C SER A 203 13.289 −7.416 −45.203 1.00 0.00 C
ATOM 3164 O SER A 203 12.124 −7.542 −44.834 1.00 0.00 O
ATOM 3165 CB SER A 203 14.743 −5.479 −44.547 1.00 0.00 C
ATOM 3166 OG SER A 203 15.415 −5.410 −45.796 1.00 0.00 O
ATOM 3167 H SER A 203 16.455 −7.263 −44.525 1.00 0.00 H
ATOM 3168 HA SER A 203 13.953 −6.970 −43.215 1.00 0.00 H
ATOM 3169 HB2 SER A 203 13.845 −4.863 −44.583 1.00 0.00 H
ATOM 3170 HB3 SER A 203 15.398 −5.100 −43.765 1.00 0.00 H
ATOM 3171 HG SER A 203 15.723 −4.504 −45.924 1.00 0.00 H
ATOM 3172 N LYS A 204 13.710 −7.847 −46.401 1.00 0.00 N
ATOM 3173 CA LYS A 204 12.878 −8.440 −47.466 1.00 0.00 C
ATOM 3174 C LYS A 204 12.785 −9.978 −47.449 1.00 0.00 C
ATOM 3175 O LYS A 204 12.532 −10.576 −48.494 1.00 0.00 O
ATOM 3176 CB LYS A 204 13.310 −7.858 −48.830 1.00 0.00 C
ATOM 3177 CG LYS A 204 14.747 −8.213 −49.281 1.00 0.00 C
ATOM 3178 CD LYS A 204 14.869 −9.300 −50.362 1.00 0.00 C
ATOM 3179 CE LYS A 204 14.234 −8.861 −51.689 1.00 0.00 C
ATOM 3180 NZ LYS A 204 14.296 −9.943 −52.702 1.00 0.00 N
ATOM 3181 H LYS A 204 14.678 −7.649 −46.608 1.00 0.00 H

ATOM 3182 HA LYS A 204 11.852 −8.109 −47.292 1.00 0.00 H
ATOM 3183 HB2 LYS A 204 12.588 −8.162 −49.587 1.00 0.00 H
ATOM 3184 HB3 LYS A 204 13.244 −6.770 −48.756 1.00 0.00 H
ATOM 3185 HG2 LYS A 204 15.217 −7.307 −49.666 1.00 0.00 H
ATOM 3186 HG3 LYS A 204 15.330 −8.531 −48.420 1.00 0.00 H
ATOM 3187 HD2 LYS A 204 14.407 −10.223 −50.016 1.00 0.00 H
ATOM 3188 HD3 LYS A 204 15.929 −9.503 −50.522 1.00 0.00 H
ATOM 3189 HE2 LYS A 204 13.191 −8.584 −51.508 1.00 0.00 H
ATOM 3190 HE3 LYS A 204 14.762 −7.971 −52.048 1.00 0.00 H
ATOM 3191 HZ1 LYS A 204 13.897 −9.633 −53.579 1.00 0.00 H
ATOM 3192 HZ2 LYS A 204 13.776 −10.749 −52.378 1.00 0.00 H
ATOM 3193 HZ3 LYS A 204 15.256 −10.223 −52.860 1.00 0.00 H
ATOM 3194 N SER A 205 13.146 −10.635 −46.343 1.00 0.00 N
ATOM 3195 CA SER A 205 13.092 −12.102 −46.226 1.00 0.00 C
ATOM 3196 C SER A 205 11.665 −12.640 −46.381 1.00 0.00 C
ATOM 3197 O SER A 205 10.704 −11.955 −46.042 1.00 0.00 O
ATOM 3198 CB SER A 205 13.662 −12.563 −44.879 1.00 0.00 C
ATOM 3199 OG SER A 205 12.888 −12.063 −43.807 1.00 0.00 O
ATOM 3200 H SER A 205 13.349 −10.104 −45.505 1.00 0.00 H
ATOM 3201 HA SER A 205 13.704 −12.532 −47.018 1.00 0.00 H
ATOM 3202 HB2 SER A 205 13.660 −13.654 −44.840 1.00 0.00 H
ATOM 3203 HB3 SER A 205 14.690 −12.219 −44.782 1.00 0.00 H
ATOM 3204 HG SER A 205 12.640 −12.825 −43.234 1.00 0.00 H
ATOM 3205 N HIS A 206 11.546 −13.915 −46.763 1.00 0.00 N
ATOM 3206 CA HIS A 206 10.296 −14.679 −46.658 1.00 0.00 C
ATOM 3207 C HIS A 206 9.943 −14.948 −45.185 1.00 0.00 C
ATOM 3208 O HIS A 206 9.006 −14.345 −44.673 1.00 0.00 O
ATOM 3209 CB HIS A 206 10.432 −15.959 −47.502 1.00 0.00 C
ATOM 3210 CG HIS A 206 9.279 −16.924 −47.406 1.00 0.00 C
ATOM 3211 ND1 HIS A 206 8.969 −17.668 −46.273 1.00 0.00 N

ATOM 3212 CD2 HIS A 206 8.496 −17.350 −48.440 1.00 0.00 C
ATOM 3213 CE1 HIS A 206 8.020 −18.540 −46.645 1.00 0.00 C
ATOM 3214 NE2 HIS A 206 7.702 −18.357 −47.936 1.00 0.00 N
ATOM 3215 H HIS A 206 12.379 −14.419 −47.020 1.00 0.00 H
ATOM 3216 HA HIS A 206 9.473 −14.088 −47.064 1.00 0.00 H
ATOM 3217 HB2 HIS A 206 11.330 −16.496 −47.193 1.00 0.00 H
ATOM 3218 HB3 HIS A 206 10.561 −15.675 −48.547 1.00 0.00 H
ATOM 3219 HD2 HIS A 206 8.551 −17.017 −49.466 1.00 0.00 H
ATOM 3220 HE1 HIS A 206 7.586 −19.295 −45.999 1.00 0.00 H
ATOM 3221 HE2 HIS A 206 7.050 −18.931 −48.454 1.00 0.00 H
ATOM 3222 N TYR A 207 10.840 −15.602 −44.442 1.00 0.00 N
ATOM 3223 CA TYR A 207 10.692 −15.844 −43.003 1.00 0.00 C
ATOM 3224 C TYR A 207 11.130 −14.660 −42.129 1.00 0.00 C
ATOM 3225 O TYR A 207 12.218 −14.113 −42.339 1.00 0.00 O
ATOM 3226 CB TYR A 207 11.478 −17.109 −42.625 1.00 0.00 C
ATOM 3227 CG TYR A 207 10.986 −18.339 −43.353 1.00 0.00 C
ATOM 3228 CD1 TYR A 207 9.708 −18.833 −43.050 1.00 0.00 C
ATOM 3229 CD2 TYR A 207 11.751 −18.941 −44.370 1.00 0.00 C
ATOM 3230 CE1 TYR A 207 9.187 −19.924 −43.757 1.00 0.00 C
ATOM 3231 CE2 TYR A 207 11.227 −20.040 −45.081 1.00 0.00 C
ATOM 3232 CZ TYR A 207 9.943 −20.535 −44.776 1.00 0.00 C
ATOM 3233 OH TYR A 207 9.435 −21.585 −45.474 1.00 0.00 O
ATOM 3234 H TYR A 207 11.551 −16.125 −44.923 1.00 0.00 H
ATOM 3235 HA TYR A 207 9.640 −16.035 −42.799 1.00 0.00 H
ATOM 3236 HB2 TYR A 207 12.538 −16.955 −42.833 1.00 0.00 H
ATOM 3237 HB3 TYR A 207 11.375 −17.288 −41.555 1.00 0.00 H
ATOM 3238 HD1 TYR A 207 9.112 −18.356 −42.291 1.00 0.00 H
ATOM 3239 HD2 TYR A 207 12.734 −18.564 −44.607 1.00 0.00 H
ATOM 3240 HE1 TYR A 207 8.191 −20.264 −43.550 1.00 0.00 H
ATOM 3241 HE2 TYR A 207 11.780 −20.492 −45.882 1.00 0.00 H

```
ATOM   3242  HH  TYR A 207       8.488 −21.742 −45.241  1.00  0.00           H
ATOM   3243  N   ILE A 208      10.432 −14.453 −41.007  1.00  0.00           N
ATOM   3244  CA  ILE A 208      11.074 −14.082 −39.736  1.00  0.00           C
ATOM   3245  C   ILE A 208      11.494 −15.341 −38.971  1.00  0.00           C
ATOM   3246  O   ILE A 208      10.919 −16.417 −39.155  1.00  0.00           O
ATOM   3247  CB  ILE A 208      10.209 −13.143 −38.860  1.00  0.00           C
ATOM   3248  CG1 ILE A 208       8.884 −13.727 −38.318  1.00  0.00           C
ATOM   3249  CG2 ILE A 208       9.940 −11.835 −39.618  1.00  0.00           C
ATOM   3250  CD1 ILE A 208       9.015 −14.513 −37.005  1.00  0.00           C
ATOM   3251  H   ILE A 208       9.570 −14.983 −40.912  1.00  0.00           H
ATOM   3252  HA  ILE A 208      11.991 −13.539 −39.960  1.00  0.00           H
ATOM   3253  HB  ILE A 208      10.805 −12.866 −37.991  1.00  0.00           H
ATOM   3254 HG12 ILE A 208       8.406 −14.345 −39.072  1.00  0.00           H
ATOM   3255 HG13 ILE A 208       8.212 −12.899 −38.108  1.00  0.00           H
ATOM   3256 HG21 ILE A 208       9.251 −12.027 −40.432  1.00  0.00           H
ATOM   3257 HG22 ILE A 208       9.491 −11.105 −38.945  1.00  0.00           H
ATOM   3258 HG23 ILE A 208      10.874 −11.432 −40.008  1.00  0.00           H
ATOM   3259 HD11 ILE A 208       8.020 −14.728 −36.616  1.00  0.00           H
ATOM   3260 HD12 ILE A 208       9.557 −13.921 −36.266  1.00  0.00           H
ATOM   3261 HD13 ILE A 208       9.530 −15.457 −37.165  1.00  0.00           H
ATOM   3262  N   LEU A 209      12.480 −15.180 −38.091  1.00  0.00           N
ATOM   3263  CA  LEU A 209      12.918 −16.181 −37.124  1.00  0.00           C
ATOM   3264  C   LEU A 209      12.405 −15.803 −35.725  1.00  0.00           C
ATOM   3265  O   LEU A 209      12.503 −14.639 −35.334  1.00  0.00           O
ATOM   3266  CB  LEU A 209      14.457 −16.280 −37.146  1.00  0.00           C
ATOM   3267  CG  LEU A 209      15.110 −16.514 −38.520  1.00  0.00           C
ATOM   3268  CD1 LEU A 209      16.625 −16.636 −38.357  1.00  0.00           C
ATOM   3269  CD2 LEU A 209      14.604 −17.787 −39.190  1.00  0.00           C
ATOM   3270  H   LEU A 209      12.833 −14.237 −37.958  1.00  0.00           H
ATOM   3271  HA  LEU A 209      12.503 −17.155 −37.388  1.00  0.00           H
```

ATOM 3272 HB2 LEU A 209 14.866 −15.355 −36.745 1.00 0.00 H
ATOM 3273 HB3 LEU A 209 14.755 −17.089 −36.483 1.00 0.00 H
ATOM 3274 HG LEU A 209 14.906 −15.665 −39.175 1.00 0.00 H
ATOM 3275 HD11 LEU A 209 17.023 −15.726 −37.907 1.00 0.00 H
ATOM 3276 HD12 LEU A 209 16.863 −17.481 −37.710 1.00 0.00 H
ATOM 3277 HD13 LEU A 209 17.095 −16.786 −39.328 1.00 0.00 H
ATOM 3278 HD21 LEU A 209 15.135 −17.948 −40.127 1.00 0.00 H
ATOM 3279 HD22 LEU A 209 13.540 −17.702 −39.405 1.00 0.00 H
ATOM 3280 HD23 LEU A 209 14.770 −18.644 −38.536 1.00 0.00 H
ATOM 3281 N ARG A 210 12.060 −16.798 −34.904 1.00 0.00 N
ATOM 3282 CA ARG A 210 11.633 −16.638 −33.500 1.00 0.00 C
ATOM 3283 C ARG A 210 12.342 −17.667 −32.619 1.00 0.00 C
ATOM 3284 O ARG A 210 12.143 −18.859 −32.826 1.00 0.00 O
ATOM 3285 CB ARG A 210 10.104 −16.832 −33.448 1.00 0.00 C
ATOM 3286 CG ARG A 210 9.463 −16.556 −32.076 1.00 0.00 C
ATOM 3287 CD ARG A 210 9.222 −15.060 −31.838 1.00 0.00 C
ATOM 3288 NE ARG A 210 8.336 −14.813 −30.683 1.00 0.00 N
ATOM 3289 CZ ARG A 210 7.015 −14.863 −30.657 1.00 0.00 C
ATOM 3290 NH1 ARG A 210 6.290 −15.248 −31.660 1.00 0.00 N
ATOM 3291 NH2 ARG A 210 6.388 −14.676 −29.542 1.00 0.00 N
ATOM 3292 H ARG A 210 11.967 −17.724 −35.319 1.00 0.00 H
ATOM 3293 HA ARG A 210 11.884 −15.636 −33.148 1.00 0.00 H
ATOM 3294 HB2 ARG A 210 9.630 −16.192 −34.195 1.00 0.00 H
ATOM 3295 HB3 ARG A 210 9.878 −17.864 −33.725 1.00 0.00 H
ATOM 3296 HG2 ARG A 210 8.511 −17.078 −32.040 1.00 0.00 H
ATOM 3297 HG3 ARG A 210 10.081 −16.959 −31.276 1.00 0.00 H
ATOM 3298 HD2 ARG A 210 10.185 −14.583 −31.657 1.00 0.00 H
ATOM 3299 HD3 ARG A 210 8.781 −14.613 −32.731 1.00 0.00 H
ATOM 3300 HE ARG A 210 8.762 −14.671 −29.774 1.00 0.00 H
ATOM 3301 HH11 ARG A 210 6.741 −15.705 −32.431 1.00 0.00 H

ATOM 3302 HH12 ARG A 210 5.372 −15.615 −31.394 1.00 0.00 H
ATOM 3303 HH21 ARG A 210 6.952 −14.687 −28.703 1.00 0.00 H
ATOM 3304 HH22 ARG A 210 5.451 −15.085 −29.499 1.00 0.00 H
ATOM 3305 N SER A 211 13.165 −17.241 −31.657 1.00 0.00 N
ATOM 3306 CA SER A 211 13.763 −18.166 −30.675 1.00 0.00 C
ATOM 3307 C SER A 211 12.689 −18.807 −29.785 1.00 0.00 C
ATOM 3308 O SER A 211 11.583 −18.282 −29.644 1.00 0.00 O
ATOM 3309 CB SER A 211 14.818 −17.475 −29.800 1.00 0.00 C
ATOM 3310 OG SER A 211 15.889 −16.997 −30.591 1.00 0.00 O
ATOM 3311 H SER A 211 13.305 −16.253 −31.526 1.00 0.00 H
ATOM 3312 HA SER A 211 14.262 −18.969 −31.217 1.00 0.00 H
ATOM 3313 HB2 SER A 211 14.359 −16.639 −29.271 1.00 0.00 H
ATOM 3314 HB3 SER A 211 15.208 −18.183 −29.066 1.00 0.00 H
ATOM 3315 HG SER A 211 16.656 −17.591 −30.483 1.00 0.00 H
ATOM 3316 N GLY A 212 13.016 −19.934 −29.161 1.00 0.00 N
ATOM 3317 CA GLY A 212 12.067 −20.702 −28.366 1.00 0.00 C
ATOM 3318 C GLY A 212 12.660 −21.996 −27.816 1.00 0.00 C
ATOM 3319 O GLY A 212 13.880 −22.133 −27.699 1.00 0.00 O
ATOM 3320 H GLY A 212 13.903 −20.379 −29.383 1.00 0.00 H
ATOM 3321 HA2 GLY A 212 11.697 −20.105 −27.533 1.00 0.00 H
ATOM 3322 HA3 GLY A 212 11.225 −20.953 −29.009 1.00 0.00 H
ATOM 3323 N LEU A 213 11.789 −22.969 −27.559 1.00 0.00 N
ATOM 3324 CA LEU A 213 12.137 −24.318 −27.114 1.00 0.00 C
ATOM 3325 C LEU A 213 11.505 −25.368 −28.033 1.00 0.00 C
ATOM 3326 O LEU A 213 10.344 −25.238 −28.416 1.00 0.00 O
ATOM 3327 CB LEU A 213 11.678 −24.547 −25.660 1.00 0.00 C
ATOM 3328 CG LEU A 213 12.202 −23.538 −24.625 1.00 0.00 C
ATOM 3329 CD1 LEU A 213 11.518 −23.746 −23.275 1.00 0.00 C
ATOM 3330 CD2 LEU A 213 13.712 −23.665 −24.418 1.00 0.00 C
ATOM 3331 H LEU A 213 10.806 −22.779 −27.742 1.00 0.00 H

ATOM 3332 HA LEU A 213 13.218 −24.440 −27.158 1.00 0.00 H

ATOM 3333 HB2 LEU A 213 10.588 −24.518 −25.642 1.00 0.00 H

ATOM 3334 HB3 LEU A 213 11.989 −25.547 −25.360 1.00 0.00 H

ATOM 3335 HG LEU A 213 11.971 −22.530 −24.959 1.00 0.00 H

ATOM 3336 HD11 LEU A 213 11.852 −22.985 −22.572 1.00 0.00 H

ATOM 3337 HD12 LEU A 213 11.757 −24.728 −22.872 1.00 0.00 H

ATOM 3338 HD13 LEU A 213 10.435 −23.658 −23.387 1.00 0.00 H

ATOM 3339 HD21 LEU A 213 14.231 −23.387 −25.332 1.00 0.00 H

ATOM 3340 HD22 LEU A 213 13.969 −24.692 −24.157 1.00 0.00 H

ATOM 3341 HD23 LEU A 213 14.029 −23.001 −23.616 1.00 0.00 H

ATOM 3342 N LEU A 214 12.201 −26.486 −28.235 1.00 0.00 N

ATOM 3343 CA LEU A 214 11.608 −27.748 −28.674 1.00 0.00 C

ATOM 3344 C LEU A 214 11.495 −28.692 −27.470 1.00 0.00 C

ATOM 3345 O LEU A 214 12.492 −29.215 −26.969 1.00 0.00 O

ATOM 3346 CB LEU A 214 12.415 −28.337 −29.844 1.00 0.00 C

ATOM 3347 CG LEU A 214 12.166 −27.599 −31.172 1.00 0.00 C

ATOM 3348 CD1 LEU A 214 13.267 −27.907 −32.187 1.00 0.00 C

ATOM 3349 CD2 LEU A 214 10.832 −27.982 −31.814 1.00 0.00 C

ATOM 3350 H LEU A 214 13.141 −26.525 −27.851 1.00 0.00 H

ATOM 3351 HA LEU A 214 10.595 −27.567 −29.029 1.00 0.00 H

ATOM 3352 HB2 LEU A 214 12.132 −29.377 −29.972 1.00 0.00 H

ATOM 3353 HB3 LEU A 214 13.476 −28.301 −29.593 1.00 0.00 H

ATOM 3354 HG LEU A 214 12.156 −26.528 −30.990 1.00 0.00 H

ATOM 3355 HD11 LEU A 214 13.274 −28.970 −32.419 1.00 0.00 H

ATOM 3356 HD12 LEU A 214 13.088 −27.340 −33.100 1.00 0.00 H

ATOM 3357 HD13 LEU A 214 14.235 −27.618 −31.776 1.00 0.00 H

ATOM 3358 HD21 LEU A 214 10.012 −27.833 −31.113 1.00 0.00 H

ATOM 3359 HD22 LEU A 214 10.852 −29.023 −32.129 1.00 0.00 H

ATOM 3360 HD23 LEU A 214 10.662 −27.352 −32.684 1.00 0.00 H

ATOM 3361 N LYS A 215 10.266 −28.811 −26.967 1.00 0.00 N

ATOM 3362 CA LYS A 215 9.820 −29.747 −25.930 1.00 0.00 C
ATOM 3363 C LYS A 215 9.801 −31.174 −26.493 1.00 0.00 C
ATOM 3364 O LYS A 215 9.338 −31.381 −27.615 1.00 0.00 O
ATOM 3365 CB LYS A 215 8.419 −29.274 −25.499 1.00 0.00 C
ATOM 3366 CG LYS A 215 7.795 −30.022 −24.315 1.00 0.00 C
ATOM 3367 CD LYS A 215 6.318 −29.619 −24.171 1.00 0.00 C
ATOM 3368 CE LYS A 215 5.649 −30.297 −22.970 1.00 0.00 C
ATOM 3369 NZ LYS A 215 5.777 −29.485 −21.736 1.00 0.00 N
ATOM 3370 H LYS A 215 9.527 −28.344 −27.486 1.00 0.00 H
ATOM 3371 HA LYS A 215 10.500 −29.701 −25.078 1.00 0.00 H
ATOM 3372 HB2 LYS A 215 8.469 −28.215 −25.238 1.00 0.00 H
ATOM 3373 HB3 LYS A 215 7.754 −29.368 −26.356 1.00 0.00 H
ATOM 3374 HG2 LYS A 215 7.851 −31.098 −24.481 1.00 0.00 H
ATOM 3375 HG3 LYS A 215 8.341 −29.765 −23.409 1.00 0.00 H
ATOM 3376 HD2 LYS A 215 6.227 −28.533 −24.085 1.00 0.00 H
ATOM 3377 HD3 LYS A 215 5.788 −29.930 −25.072 1.00 0.00 H
ATOM 3378 HE2 LYS A 215 6.085 −31.290 −22.828 1.00 0.00 H
ATOM 3379 HE3 LYS A 215 4.587 −30.421 −23.203 1.00 0.00 H
ATOM 3380 HZ1 LYS A 215 5.371 −29.936 −20.933 1.00 0.00 H
ATOM 3381 HZ2 LYS A 215 6.738 −29.237 −21.523 1.00 0.00 H
ATOM 3382 HZ3 LYS A 215 5.300 −28.584 −21.849 1.00 0.00 H
ATOM 3383 N TYR A 216 10.211 −32.163 −25.703 1.00 0.00 N
ATOM 3384 CA TYR A 216 10.307 −33.574 −26.106 1.00 0.00 C
ATOM 3385 C TYR A 216 10.047 −34.480 −24.896 1.00 0.00 C
ATOM 3386 O TYR A 216 10.918 −34.632 −24.041 1.00 0.00 O
ATOM 3387 CB TYR A 216 11.706 −33.807 −26.698 1.00 0.00 C
ATOM 3388 CG TYR A 216 11.950 −35.166 −27.331 1.00 0.00 C
ATOM 3389 CD1 TYR A 216 11.496 −35.426 −28.639 1.00 0.00 C
ATOM 3390 CD2 TYR A 216 12.696 −36.143 −26.644 1.00 0.00 C
ATOM 3391 CE1 TYR A 216 11.835 −36.632 −29.285 1.00 0.00 C

ATOM 3392 CE2 TYR A 216 13.027 −37.356 −27.279 1.00 0.00 C
ATOM 3393 CZ TYR A 216 12.609 −37.596 −28.606 1.00 0.00 C
ATOM 3394 OH TYR A 216 12.942 −38.761 −29.220 1.00 0.00 O
ATOM 3395 H TYR A 216 10.663 −31.897 −24.833 1.00 0.00 H
ATOM 3396 HA TYR A 216 9.565 −33.794 −26.877 1.00 0.00 H
ATOM 3397 HB2 TYR A 216 11.902 −33.045 −27.449 1.00 0.00 H
ATOM 3398 HB3 TYR A 216 12.448 −33.645 −25.918 1.00 0.00 H
ATOM 3399 HD1 TYR A 216 10.881 −34.700 −29.150 1.00 0.00 H
ATOM 3400 HD2 TYR A 216 13.019 −35.959 −25.627 1.00 0.00 H
ATOM 3401 HE1 TYR A 216 11.491 −36.834 −30.288 1.00 0.00 H
ATOM 3402 HE2 TYR A 216 13.614 −38.094 −26.756 1.00 0.00 H
ATOM 3403 HH TYR A 216 13.212 −39.420 −28.580 1.00 0.00 H
ATOM 3404 N ASN A 217 8.797 −34.913 −24.708 1.00 0.00 N
ATOM 3405 CA ASN A 217 8.398 −35.717 −23.546 1.00 0.00 C
ATOM 3406 C ASN A 217 8.552 −37.221 −23.814 1.00 0.00 C
ATOM 3407 O ASN A 217 7.847 −37.780 −24.657 1.00 0.00 O
ATOM 3408 CB ASN A 217 6.977 −35.331 −23.115 1.00 0.00 C
ATOM 3409 CG ASN A 217 6.643 −35.996 −21.793 1.00 0.00 C
ATOM 3410 OD1 ASN A 217 7.339 −35.828 −20.808 1.00 0.00 O
ATOM 3411 ND2 ASN A 217 5.697 −36.901 −21.776 1.00 0.00 N
ATOM 3412 H ASN A 217 8.115 −34.752 −25.432 1.00 0.00 H
ATOM 3413 HA ASN A 217 9.056 −35.486 −22.711 1.00 0.00 H
ATOM 3414 HB2 ASN A 217 6.912 −34.252 −22.971 1.00 0.00 H
ATOM 3415 HB3 ASN A 217 6.260 −35.630 −23.878 1.00 0.00 H
ATOM 3416 HD21 ASN A 217 5.228 −37.169 −22.616 1.00 0.00 H
ATOM 3417 HD22 ASN A 217 5.710 −37.505 −20.954 1.00 0.00 H
ATOM 3418 N LEU A 218 9.434 −37.880 −23.063 1.00 0.00 N
ATOM 3419 CA LEU A 218 9.832 −39.273 −23.281 1.00 0.00 C
ATOM 3420 C LEU A 218 8.716 −40.293 −22.977 1.00 0.00 C
ATOM 3421 O LEU A 218 8.602 −41.267 −23.723 1.00 0.00 O

ATOM 3422 CB LEU A 218 11.102 −39.547 −22.456 1.00 0.00 C
ATOM 3423 CG LEU A 218 12.344 −38.735 −22.875 1.00 0.00 C
ATOM 3424 CD1 LEU A 218 13.437 −38.838 −21.816 1.00 0.00 C
ATOM 3425 CD2 LEU A 218 12.925 −39.242 −24.199 1.00 0.00 C
ATOM 3426 H LEU A 218 9.926 −37.370 −22.331 1.00 0.00 H
ATOM 3427 HA LEU A 218 10.072 −39.399 −24.335 1.00 0.00 H
ATOM 3428 HB2 LEU A 218 11.339 −40.610 −22.494 1.00 0.00 H
ATOM 3429 HB3 LEU A 218 10.878 −39.301 −21.424 1.00 0.00 H
ATOM 3430 HG LEU A 218 12.085 −37.682 −22.993 1.00 0.00 H
ATOM 3431 HD21 LEU A 218 12.190 −39.122 −24.992 1.00 0.00 H
ATOM 3432 HD22 LEU A 218 13.816 −38.665 −24.440 1.00 0.00 H
ATOM 3433 HD23 LEU A 218 13.191 −40.294 −24.099 1.00 0.00 H
ATOM 3434 HD11 LEU A 218 14.313 −38.263 −22.113 1.00 0.00 H
ATOM 3435 HD12 LEU A 218 13.717 −39.879 −21.662 1.00 0.00 H
ATOM 3436 HD13 LEU A 218 13.064 −38.438 −20.871 1.00 0.00 H
ATOM 3437 N SER A 219 7.728 −39.936 −22.142 1.00 0.00 N
ATOM 3438 CA SER A 219 6.552 −40.782 −21.839 1.00 0.00 C
ATOM 3439 C SER A 219 5.671 −41.085 −23.058 1.00 0.00 C
ATOM 3440 O SER A 219 4.808 −41.958 −23.002 1.00 0.00 O
ATOM 3441 CB SER A 219 5.637 −40.178 −20.752 1.00 0.00 C
ATOM 3442 OG SER A 219 6.194 −39.092 −20.043 1.00 0.00 O
ATOM 3443 H SER A 219 7.941 −39.201 −21.477 1.00 0.00 H
ATOM 3444 HA SER A 219 6.925 −41.734 −21.462 1.00 0.00 H
ATOM 3445 HB2 SER A 219 5.363 −40.963 −20.044 1.00 0.00 H
ATOM 3446 HB3 SER A 219 4.719 −39.814 −21.215 1.00 0.00 H
ATOM 3447 HG SER A 219 7.087 −39.380 −19.692 1.00 0.00 H
ATOM 3448 N LEU A 220 5.814 −40.321 −24.148 1.00 0.00 N
ATOM 3449 CA LEU A 220 5.107 −40.551 −25.407 1.00 0.00 C
ATOM 3450 C LEU A 220 5.605 −41.847 −26.065 1.00 0.00 C
ATOM 3451 O LEU A 220 5.358 −42.083 −27.251 1.00 0.00 O

ATOM 3452 CB LEU A 220 5.281 −39.321 −26.318 1.00 0.00 C
ATOM 3453 CG LEU A 220 4.695 −38.007 −25.766 1.00 0.00 C
ATOM 3454 CD1 LEU A 220 5.059 −36.857 −26.705 1.00 0.00 C
ATOM 3455 CD2 LEU A 220 3.170 −38.064 −25.653 1.00 0.00 C
ATOM 3456 H LEU A 220 6.579 −39.655 −24.143 1.00 0.00 H
ATOM 3457 HA LEU A 220 4.048 −40.699 −25.193 1.00 0.00 H
ATOM 3458 HB2 LEU A 220 6.347 −39.181 −26.481 1.00 0.00 H
ATOM 3459 HB3 LEU A 220 4.818 −39.526 −27.284 1.00 0.00 H
ATOM 3460 HG LEU A 220 5.110 −37.793 −24.781 1.00 0.00 H
ATOM 3461 HD11 LEU A 220 4.649 −35.922 −26.324 1.00 0.00 H
ATOM 3462 HD12 LEU A 220 4.655 −37.047 −27.698 1.00 0.00 H
ATOM 3463 HD13 LEU A 220 6.143 −36.760 −26.761 1.00 0.00 H
ATOM 3464 HD21 LEU A 220 2.879 −38.806 −24.911 1.00 0.00 H
ATOM 3465 HD22 LEU A 220 2.789 −37.094 −25.333 1.00 0.00 H
ATOM 3466 HD23 LEU A 220 2.732 −38.321 −26.617 1.00 0.00 H
ATOM 3467 H LEU A 220 6.181 −42.563 −25.480 1.00 0.00 H
ATOM 3468 N ILE A 221 9.238 −40.378 −30.930 1.00 0.00 N
ATOM 3469 CA ILE A 221 8.601 −39.460 −29.982 1.00 0.00 C
ATOM 3470 C ILE A 221 8.507 −38.058 −30.614 1.00 0.00 C
ATOM 3471 O ILE A 221 9.183 −37.754 −31.600 1.00 0.00 O
ATOM 3472 CB ILE A 221 9.332 −39.510 −28.611 1.00 0.00 C
ATOM 3473 CG1 ILE A 221 8.897 −40.727 −27.757 1.00 0.00 C
ATOM 3474 CG2 ILE A 221 9.106 −38.265 −27.731 1.00 0.00 C
ATOM 3475 CD1 ILE A 221 9.225 −42.107 −28.341 1.00 0.00 C
ATOM 3476 HA ILE A 221 7.578 −39.802 −29.821 1.00 0.00 H
ATOM 3477 HB ILE A 221 10.404 −39.583 −28.794 1.00 0.00 H
ATOM 3478 HG12 ILE A 221 9.390 −40.667 −26.785 1.00 0.00 H
ATOM 3479 HG13 ILE A 221 7.822 −40.677 −27.586 1.00 0.00 H
ATOM 3480 HG21 ILE A 221 9.468 −37.365 −28.224 1.00 0.00 H
ATOM 3481 HG22 ILE A 221 9.656 −38.365 −26.794 1.00 0.00 H

ATOM 3482 HG23 ILE A 221 8.051 −38.142 −27.500 1.00 0.00 H
ATOM 3483 HD11 ILE A 221 9.016 −42.871 −27.591 1.00 0.00 H
ATOM 3484 HD12 ILE A 221 8.602 −42.307 −29.212 1.00 0.00 H
ATOM 3485 HD13 ILE A 221 10.278 −42.155 −28.618 1.00 0.00 H
ATOM 3486 H ILE A 221 9.789 −39.946 −31.664 1.00 0.00 H
ATOM 3487 H ILE A 221 8.507 −40.925 −31.372 1.00 0.00 H
ATOM 3488 N ILE A 222 7.499 −37.295 −30.186 1.00 0.00 N
ATOM 3489 CA ILE A 222 7.091 −36.014 −30.780 1.00 0.00 C
ATOM 3490 C ILE A 222 7.971 −34.871 −30.258 1.00 0.00 C
ATOM 3491 O ILE A 222 8.118 −34.718 −29.045 1.00 0.00 O
ATOM 3492 CB ILE A 222 5.590 −35.754 −30.493 1.00 0.00 C
ATOM 3493 CG1 ILE A 222 4.731 −36.970 −30.920 1.00 0.00 C
ATOM 3494 CG2 ILE A 222 5.124 −34.465 −31.186 1.00 0.00 C
ATOM 3495 CD1 ILE A 222 3.213 −36.800 −30.784 1.00 0.00 C
ATOM 3496 H ILE A 222 7.020 −37.586 −29.349 1.00 0.00 H
ATOM 3497 HA ILE A 222 7.225 −36.075 −31.862 1.00 0.00 H
ATOM 3498 HB ILE A 222 5.463 −35.611 −29.420 1.00 0.00 H
ATOM 3499 HG12 ILE A 222 4.963 −37.211 −31.953 1.00 0.00 H
ATOM 3500 HG13 ILE A 222 5.002 −37.830 −30.307 1.00 0.00 H
ATOM 3501 HD11 ILE A 222 2.725 −37.760 −30.948 1.00 0.00 H
ATOM 3502 HD12 ILE A 222 2.965 −36.438 −29.786 1.00 0.00 H
ATOM 3503 HD13 ILE A 222 2.844 −36.096 −31.530 1.00 0.00 H
ATOM 3504 HG21 ILE A 222 5.672 −33.608 −30.803 1.00 0.00 H
ATOM 3505 HG22 ILE A 222 4.077 −34.276 −30.958 1.00 0.00 H
ATOM 3506 HG23 ILE A 222 5.256 −34.528 −32.265 1.00 0.00 H
ATOM 3507 N LEU A 223 8.415 −33.980 −31.152 1.00 0.00 N
ATOM 3508 CA LEU A 223 8.852 −32.629 −30.778 1.00 0.00 C
ATOM 3509 C LEU A 223 7.671 −31.651 −30.800 1.00 0.00 C
ATOM 3510 O LEU A 223 6.949 −31.593 −31.793 1.00 0.00 O
ATOM 3511 CB LEU A 223 9.948 −32.132 −31.734 1.00 0.00 C

ATOM 3512 CG LEU A 223 11.311 −32.821 −31.586 1.00 0.00 C

ATOM 3513 CD1 LEU A 223 12.212 −32.365 −32.730 1.00 0.00 C

ATOM 3514 CD2 LEU A 223 12.032 −32.444 −30.292 1.00 0.00 C

ATOM 3515 H LEU A 223 8.192 −34.144 −32.128 1.00 0.00 H

ATOM 3516 HA LEU A 223 9.242 −32.647 −29.762 1.00 0.00 H

ATOM 3517 HB2 LEU A 223 10.087 −31.064 −31.568 1.00 0.00 H

ATOM 3518 HB3 LEU A 223 9.593 −32.270 −32.755 1.00 0.00 H

ATOM 3519 HG LEU A 223 11.188 −33.903 −31.633 1.00 0.00 H

ATOM 3520 HD11 LEU A 223 12.268 −31.278 −32.755 1.00 0.00 H

ATOM 3521 HD12 LEU A 223 13.209 −32.767 −32.573 1.00 0.00 H

ATOM 3522 HD13 LEU A 223 11.818 −32.727 −33.678 1.00 0.00 H

ATOM 3523 HD21 LEU A 223 12.466 −31.453 −30.370 1.00 0.00 H

ATOM 3524 HD22 LEU A 223 11.339 −32.449 −29.457 1.00 0.00 H

ATOM 3525 HD23 LEU A 223 12.832 −33.160 −30.099 1.00 0.00 H

ATOM 3526 N GLN A 224 7.596 −30.752 −29.820 1.00 0.00 N

ATOM 3527 CA GLN A 224 6.581 −29.692 −29.748 1.00 0.00 C

ATOM 3528 C GLN A 224 7.254 −28.324 −29.547 1.00 0.00 C

ATOM 3529 O GLN A 224 8.116 −28.184 −28.680 1.00 0.00 O

ATOM 3530 CB GLN A 224 5.561 −30.008 −28.638 1.00 0.00 C

ATOM 3531 CG GLN A 224 4.902 −31.390 −28.796 1.00 0.00 C

ATOM 3532 CD GLN A 224 3.775 −31.615 −27.796 1.00 0.00 C

ATOM 3533 OE1 GLN A 224 3.917 −31.395 −26.601 1.00 0.00 O

ATOM 3534 NE2 GLN A 224 2.643 −32.127 −28.223 1.00 0.00 N

ATOM 3535 H GLN A 224 8.243 −30.836 −29.041 1.00 0.00 H

ATOM 3536 HA GLN A 224 6.032 −29.657 −30.686 1.00 0.00 H

ATOM 3537 HB2 GLN A 224 4.782 −29.244 −28.660 1.00 0.00 H

ATOM 3538 HB3 GLN A 224 6.052 −29.965 −27.667 1.00 0.00 H

ATOM 3539 HG2 GLN A 224 4.504 −31.483 −29.803 1.00 0.00 H

ATOM 3540 HG3 GLN A 224 5.641 −32.178 −28.650 1.00 0.00 H

ATOM 3541 HE21 GLN A 224 2.484 −32.304 −29.226 1.00 0.00 H

ATOM 3542 HE22 GLN A 224 1.918 −32.267 −27.551 1.00 0.00 H
ATOM 3543 N PHE A 225 6.965 −27.338 −30.401 1.00 0.00 N
ATOM 3544 CA PHE A 225 7.614 −26.020 −30.343 1.00 0.00 C
ATOM 3545 C PHE A 225 6.866 −25.024 −29.446 1.00 0.00 C
ATOM 3546 O PHE A 225 5.677 −24.774 −29.647 1.00 0.00 O
ATOM 3547 CB PHE A 225 7.821 −25.455 −31.751 1.00 0.00 C
ATOM 3548 CG PHE A 225 8.544 −24.116 −31.779 1.00 0.00 C
ATOM 3549 CD1 PHE A 225 9.917 −24.045 −31.475 1.00 0.00 C
ATOM 3550 CD2 PHE A 225 7.844 −22.933 −32.088 1.00 0.00 C
ATOM 3551 CE1 PHE A 225 10.584 −22.806 −31.473 1.00 0.00 C
ATOM 3552 CE2 PHE A 225 8.512 −21.694 −32.093 1.00 0.00 C
ATOM 3553 CZ PHE A 225 9.881 −21.629 −31.782 1.00 0.00 C
ATOM 3554 H PHE A 225 6.244 −27.497 −31.099 1.00 0.00 H
ATOM 3555 HA PHE A 225 8.607 −26.151 −29.916 1.00 0.00 H
ATOM 3556 HB2 PHE A 225 6.849 −25.344 −32.229 1.00 0.00 H
ATOM 3557 HB3 PHE A 225 8.398 −26.175 −32.331 1.00 0.00 H
ATOM 3558 HD1 PHE A 225 10.458 −24.943 −31.217 1.00 0.00 H
ATOM 3559 HD2 PHE A 225 6.787 −22.970 −32.308 1.00 0.00 H
ATOM 3560 HE1 PHE A 225 11.634 −22.756 −31.222 1.00 0.00 H
ATOM 3561 HE2 PHE A 225 7.968 −20.790 −32.325 1.00 0.00 H
ATOM 3562 HZ PHE A 225 10.390 −20.675 −31.772 1.00 0.00 H
ATOM 3563 N ILE A 226 7.625 −24.304 −28.618 1.00 0.00 N
ATOM 3564 CA ILE A 226 7.180 −23.154 −27.828 1.00 0.00 C
ATOM 3565 C ILE A 226 7.976 −21.914 −28.254 1.00 0.00 C
ATOM 3566 O ILE A 226 9.201 −21.909 −28.168 1.00 0.00 O
ATOM 3567 CB ILE A 226 7.353 −23.469 −26.324 1.00 0.00 C
ATOM 3568 CG1 ILE A 226 6.435 −24.610 −25.818 1.00 0.00 C
ATOM 3569 CG2 ILE A 226 7.161 −22.206 −25.477 1.00 0.00 C
ATOM 3570 CD1 ILE A 226 4.926 −24.325 −25.867 1.00 0.00 C
ATOM 3571 H ILE A 226 8.612 −24.539 −28.562 1.00 0.00 H

ATOM 3572 HA ILE A 226 6.127 −22.956 −28.024 1.00 0.00 H
ATOM 3573 HB ILE A 226 8.382 −23.801 −26.171 1.00 0.00 H
ATOM 3574 HG12 ILE A 226 6.634 −25.513 −26.396 1.00 0.00 H
ATOM 3575 HG13 ILE A 226 6.700 −24.830 −24.782 1.00 0.00 H
ATOM 3576 HG21 ILE A 226 6.226 −21.710 −25.735 1.00 0.00 H
ATOM 3577 HG22 ILE A 226 7.161 −22.483 −24.425 1.00 0.00 H
ATOM 3578 HG23 ILE A 226 7.990 −21.516 −25.638 1.00 0.00 H
ATOM 3579 HD11 ILE A 226 4.386 −25.191 −25.486 1.00 0.00 H
ATOM 3580 HD12 ILE A 226 4.608 −24.141 −26.893 1.00 0.00 H
ATOM 3581 HD13 ILE A 226 4.680 −23.463 −25.247 1.00 0.00 H
ATOM 3582 N GLU A 227 7.288 −20.835 −28.626 1.00 0.00 N
ATOM 3583 CA GLU A 227 7.908 −19.535 −28.907 1.00 0.00 C
ATOM 3584 C GLU A 227 8.277 −18.760 −27.628 1.00 0.00 C
ATOM 3585 O GLU A 227 7.578 −18.840 −26.617 1.00 0.00 O
ATOM 3586 CB GLU A 227 6.991 −18.713 −29.825 1.00 0.00 C
ATOM 3587 CG GLU A 227 5.584 −18.406 −29.270 1.00 0.00 C
ATOM 3588 CD GLU A 227 4.750 −17.576 −30.263 1.00 0.00 C
ATOM 3589 OE1 GLU A 227 4.931 −17.794 −31.481 1.00 0.00 O
ATOM 3590 OE2 GLU A 227 4.396 −16.433 −29.902 1.00 0.00 O
ATOM 3591 H GLU A 227 6.282 −20.874 −28.594 1.00 0.00 H
ATOM 3592 HA GLU A 227 8.835 −19.710 −29.456 1.00 0.00 H
ATOM 3593 HB2 GLU A 227 7.484 −17.766 −30.045 1.00 0.00 H
ATOM 3594 HB3 GLU A 227 6.886 −19.263 −30.760 1.00 0.00 H
ATOM 3595 HG2 GLU A 227 5.060 −19.342 −29.061 1.00 0.00 H
ATOM 3596 HG3 GLU A 227 5.681 −17.858 −28.330 1.00 0.00 H
ATOM 3597 N ILE A 228 9.330 −17.936 −27.700 1.00 0.00 N
ATOM 3598 CA ILE A 228 9.667 −16.948 −26.661 1.00 0.00 C
ATOM 3599 C ILE A 228 8.743 −15.723 −26.719 1.00 0.00 C
ATOM 3600 O ILE A 228 8.392 −15.267 −27.809 1.00 0.00 O
ATOM 3601 CB ILE A 228 11.162 −16.557 −26.753 1.00 0.00 C

```
ATOM   3602  CG1 ILE A 228      11.657 −15.952 −25.422  1.00  0.00           C
ATOM   3603  CG2 ILE A 228      11.480 −15.590 −27.913  1.00  0.00           C
ATOM   3604  CD1 ILE A 228      13.176 −16.067 −25.260  1.00  0.00           C
ATOM   3605  H   ILE A 228       9.904 −17.948 −28.535  1.00  0.00           H
ATOM   3606  HA  ILE A 228       9.504 −17.430 −25.697  1.00  0.00           H
ATOM   3607  HB  ILE A 228      11.717 −17.482 −26.922  1.00  0.00           H
ATOM   3608 HG12 ILE A 228      11.200 −16.487 −24.591  1.00  0.00           H
ATOM   3609 HG13 ILE A 228      11.361 −14.905 −25.346  1.00  0.00           H
ATOM   3610 HG21 ILE A 228      11.091 −14.594 −27.697  1.00  0.00           H
ATOM   3611 HG22 ILE A 228      12.557 −15.524 −28.056  1.00  0.00           H
ATOM   3612 HG23 ILE A 228      11.025 −15.946 −28.835  1.00  0.00           H
ATOM   3613 HD11 ILE A 228      13.481 −17.105 −25.380  1.00  0.00           H
ATOM   3614 HD12 ILE A 228      13.688 −15.457 −26.004  1.00  0.00           H
ATOM   3615 HD13 ILE A 228      13.458 −15.730 −24.262  1.00  0.00           H
ATOM   3616  N   ALA A 229       8.451 −15.112 −25.569  1.00  0.00           N
ATOM   3617  CA  ALA A 229       7.614 −13.914 −25.441  1.00  0.00           C
ATOM   3618  C   ALA A 229       8.048 −12.710 −26.315  1.00  0.00           C
ATOM   3619  O   ALA A 229       9.199 −12.581 −26.734  1.00  0.00           O
ATOM   3620  CB  ALA A 229       7.552 −13.532 −23.955  1.00  0.00           C
ATOM   3621  H   ALA A 229       8.679 −15.614 −24.717  1.00  0.00           H
ATOM   3622  HA  ALA A 229       6.605 −14.188 −25.754  1.00  0.00           H
ATOM   3623  HB1 ALA A 229       6.865 −12.696 −23.817  1.00  0.00           H
ATOM   3624  HB2 ALA A 229       8.544 −13.245 −23.605  1.00  0.00           H
ATOM   3625  HB3 ALA A 229       7.192 −14.381 −23.373  1.00  0.00           H
ATOM   3626  N   ASP A 230       7.120 −11.771 −26.517  1.00  0.00           N
ATOM   3627  CA  ASP A 230       7.316 −10.492 −27.218  1.00  0.00           C
ATOM   3628  C   ASP A 230       8.240  −9.513 −26.462  1.00  0.00           C
ATOM   3629  O   ASP A 230       8.782  −8.571 −27.045  1.00  0.00           O
ATOM   3630  CB  ASP A 230       5.942  −9.817 −27.387  1.00  0.00           C
ATOM   3631  CG  ASP A 230       5.325  −9.431 −26.033  1.00  0.00           C
```

ATOM	3632	OD1	ASP	A	230	5.072	−10.388	−25.267	1.00	0.00	O
ATOM	3633	OD2	ASP	A	230	5.645	−8.305	−25.587	1.00	0.00	O
ATOM	3634	H	ASP	A	230	6.227	−11.859	−26.036	1.00	0.00	H
ATOM	3635	HA	ASP	A	230	7.739	−10.686	−28.202	1.00	0.00	H
ATOM	3636	HB2	ASP	A	230	5.267	−10.496	−27.912	1.00	0.00	H
ATOM	3637	HB3	ASP	A	230	6.057	−8.923	−28.003	1.00	0.00	H
ATOM	3638	N	ASN	A	231	8.360	−9.683	−25.142	1.00	0.00	N
ATOM	3639	CA	ASN	A	231	9.047	−8.740	−24.267	1.00	0.00	C
ATOM	3640	C	ASN	A	231	10.544	−8.696	−24.639	1.00	0.00	C
ATOM	3641	O	ASN	A	231	11.232	−9.709	−24.520	1.00	0.00	O
ATOM	3642	CB	ASN	A	231	8.842	−9.156	−22.794	1.00	0.00	C
ATOM	3643	CG	ASN	A	231	7.414	−9.377	−22.302	1.00	0.00	C
ATOM	3644	OD1	ASN	A	231	7.223	−9.968	−21.250	1.00	0.00	O
ATOM	3645	ND2	ASN	A	231	6.375	−8.931	−22.964	1.00	0.00	N
ATOM	3646	H	ASN	A	231	7.796	−10.411	−24.730	1.00	0.00	H
ATOM	3647	HA	ASN	A	231	8.612	−7.751	−24.418	1.00	0.00	H
ATOM	3648	HB2	ASN	A	231	9.383	−10.086	−22.616	1.00	0.00	H
ATOM	3649	HB3	ASN	A	231	9.282	−8.389	−22.157	1.00	0.00	H
ATOM	3650	HD21	ASN	A	231	6.424	−8.600	−23.933	1.00	0.00	H
ATOM	3651	HD22	ASN	A	231	5.475	−9.282	−22.691	1.00	0.00	H
ATOM	3652	N	ARG	A	232	11.042	−7.553	−25.144	1.00	0.00	N
ATOM	3653	CA	ARG	A	232	12.352	−7.442	−25.837	1.00	0.00	C
ATOM	3654	C	ARG	A	232	12.522	−8.508	−26.936	1.00	0.00	C
ATOM	3655	O	ARG	A	232	13.543	−9.192	−27.007	1.00	0.00	O
ATOM	3656	CB	ARG	A	232	13.546	−7.390	−24.850	1.00	0.00	C
ATOM	3657	CG	ARG	A	232	13.652	−6.054	−24.097	1.00	0.00	C
ATOM	3658	CD	ARG	A	232	14.786	−6.038	−23.058	1.00	0.00	C
ATOM	3659	NE	ARG	A	232	16.146	−6.118	−23.637	1.00	0.00	N
ATOM	3660	CZ	ARG	A	232	17.259	−6.313	−22.953	1.00	0.00	C
ATOM	3661	NH1	ARG	A	232	18.393	−6.455	−23.566	1.00	0.00	N

ATOM 3662 NH2 ARG A 232 17.264 −6.371 −21.650 1.00 0.00 N
ATOM 3663 H ARG A 232 10.377 −6.805 −25.281 1.00 0.00 H
ATOM 3664 HA ARG A 232 12.359 −6.502 −26.389 1.00 0.00 H
ATOM 3665 HB2 ARG A 232 14.476 −7.514 −25.408 1.00 0.00 H
ATOM 3666 HB3 ARG A 232 13.469 −8.211 −24.138 1.00 0.00 H
ATOM 3667 HG2 ARG A 232 13.809 −5.245 −24.812 1.00 0.00 H
ATOM 3668 HG3 ARG A 232 12.713 −5.873 −23.573 1.00 0.00 H
ATOM 3669 HD2 ARG A 232 14.639 −6.878 −22.375 1.00 0.00 H
ATOM 3670 HD3 ARG A 232 14.707 −5.115 −22.480 1.00 0.00 H
ATOM 3671 HE ARG A 232 16.274 −6.116 −24.653 1.00 0.00 H
ATOM 3672 HH11 ARG A 232 18.430 −6.224 −24.572 1.00 0.00 H
ATOM 3673 HH12 ARG A 232 19.255 −6.499 −23.067 1.00 0.00 H
ATOM 3674 HH21 ARG A 232 16.464 −6.020 −21.136 1.00 0.00 H
ATOM 3675 HH22 ARG A 232 18.084 −6.677 −21.145 1.00 0.00 H
ATOM 3676 N LEU A 233 11.526 −8.657 −27.811 1.00 0.00 N
ATOM 3677 CA LEU A 233 11.640 −9.473 −29.019 1.00 0.00 C
ATOM 3678 C LEU A 233 12.813 −8.994 −29.889 1.00 0.00 C
ATOM 3679 O LEU A 233 12.936 −7.812 −30.199 1.00 0.00 O
ATOM 3680 CB LEU A 233 10.307 −9.446 −29.792 1.00 0.00 C
ATOM 3681 CG LEU A 233 10.280 −10.286 −31.081 1.00 0.00 C
ATOM 3682 CD1 LEU A 233 10.450 −11.781 −30.806 1.00 0.00 C
ATOM 3683 CD2 LEU A 233 8.944 −10.092 −31.801 1.00 0.00 C
ATOM 3684 H LEU A 233 10.642 −8.197 −27.629 1.00 0.00 H
ATOM 3685 HA LEU A 233 11.834 −10.499 −28.703 1.00 0.00 H
ATOM 3686 HB2 LEU A 233 9.520 −9.812 −29.137 1.00 0.00 H
ATOM 3687 HB3 LEU A 233 10.080 −8.410 −30.047 1.00 0.00 H
ATOM 3688 HG LEU A 233 11.076 −9.963 −31.748 1.00 0.00 H
ATOM 3689 HD11 LEU A 233 10.381 −12.324 −31.747 1.00 0.00 H
ATOM 3690 HD12 LEU A 233 9.674 −12.125 −30.120 1.00 0.00 H
ATOM 3691 HD13 LEU A 233 11.427 −11.980 −30.372 1.00 0.00 H

ATOM 3692 HD21 LEU A 233 8.804 −9.037 −32.036 1.00 0.00 H
ATOM 3693 HD22 LEU A 233 8.124 −10.427 −31.165 1.00 0.00 H
ATOM 3694 HD23 LEU A 233 8.940 −10.665 −32.727 1.00 0.00 H
ATOM 3695 N THR A 234 13.595 −9.950 −30.380 1.00 0.00 N
ATOM 3696 CA THR A 234 14.648 −9.774 −31.390 1.00 0.00 C
ATOM 3697 C THR A 234 14.591 −10.953 −32.357 1.00 0.00 C
ATOM 3698 O THR A 234 13.956 −11.966 −32.048 1.00 0.00 O
ATOM 3699 CB THR A 234 16.049 −9.672 −30.755 1.00 0.00 C
ATOM 3700 OG1 THR A 234 16.340 −10.798 −29.952 1.00 0.00 O
ATOM 3701 CG2 THR A 234 16.215 −8.444 −29.867 1.00 0.00 C
ATOM 3702 H THR A 234 13.367 −10.906 −30.153 1.00 0.00 H
ATOM 3703 HA THR A 234 14.462 −8.862 −31.958 1.00 0.00 H
ATOM 3704 HB THR A 234 16.794 −9.612 −31.549 1.00 0.00 H
ATOM 3705 HG1 THR A 234 16.567 −11.540 −30.532 1.00 0.00 H
ATOM 3706 HG21 THR A 234 16.038 −7.539 −30.451 1.00 0.00 H
ATOM 3707 HG22 THR A 234 15.518 −8.465 −29.030 1.00 0.00 H
ATOM 3708 HG23 THR A 234 17.233 −8.423 −29.485 1.00 0.00 H
ATOM 3709 N ILE A 235 15.323 −10.871 −33.474 1.00 0.00 N
ATOM 3710 CA ILE A 235 15.451 −11.970 −34.441 1.00 0.00 C
ATOM 3711 C ILE A 235 15.783 −13.296 −33.752 1.00 0.00 C
ATOM 3712 O ILE A 235 16.641 −13.347 −32.869 1.00 0.00 O
ATOM 3713 CB ILE A 235 16.486 −11.627 −35.539 1.00 0.00 C
ATOM 3714 CG1 ILE A 235 16.565 −12.770 −36.578 1.00 0.00 C
ATOM 3715 CG2 ILE A 235 17.888 −11.313 −34.978 1.00 0.00 C
ATOM 3716 CD1 ILE A 235 17.174 −12.369 −37.923 1.00 0.00 C
ATOM 3717 H ILE A 235 15.847 −10.028 −33.654 1.00 0.00 H
ATOM 3718 HA ILE A 235 14.480 −12.089 −34.924 1.00 0.00 H
ATOM 3719 HB ILE A 235 16.127 −10.730 −36.038 1.00 0.00 H
ATOM 3720 HG12 ILE A 235 15.559 −13.135 −36.779 1.00 0.00 H
ATOM 3721 HG13 ILE A 235 17.147 −13.596 −36.169 1.00 0.00 H

ATOM 3722 HG21 ILE A 235 18.510 −10.894 −35.766 1.00 0.00 H
ATOM 3723 HG22 ILE A 235 17.834 −10.575 −34.180 1.00 0.00 H
ATOM 3724 HG23 ILE A 235 18.358 −12.217 −34.590 1.00 0.00 H
ATOM 3725 HD11 ILE A 235 18.155 −11.930 −37.781 1.00 0.00 H
ATOM 3726 HD12 ILE A 235 16.539 −11.639 −38.411 1.00 0.00 H
ATOM 3727 HD13 ILE A 235 17.263 −13.247 −38.562 1.00 0.00 H
ATOM 3728 N GLY A 236 15.112 −14.368 −34.178 1.00 0.00 N
ATOM 3729 CA GLY A 236 15.406 −15.726 −33.742 1.00 0.00 C
ATOM 3730 C GLY A 236 16.870 −16.076 −33.984 1.00 0.00 C
ATOM 3731 O GLY A 236 17.353 −16.100 −35.115 1.00 0.00 O
ATOM 3732 H GLY A 236 14.345 −14.232 −34.828 1.00 0.00 H
ATOM 3733 HA2 GLY A 236 14.783 −16.441 −34.274 1.00 0.00 H
ATOM 3734 HA3 GLY A 236 15.188 −15.800 −32.680 1.00 0.00 H
ATOM 3735 N SER A 237 17.580 −16.295 −32.888 1.00 0.00 N
ATOM 3736 CA SER A 237 19.033 −16.396 −32.801 1.00 0.00 C
ATOM 3737 C SER A 237 19.416 −17.476 −31.783 1.00 0.00 C
ATOM 3738 O SER A 237 18.564 −17.853 −30.967 1.00 0.00 O
ATOM 3739 CB SER A 237 19.610 −15.037 −32.419 1.00 0.00 C
ATOM 3740 OG SER A 237 19.157 −14.633 −31.146 1.00 0.00 O
ATOM 3741 H SER A 237 17.090 −16.249 −32.002 1.00 0.00 H
ATOM 3742 HA SER A 237 19.436 −16.655 −33.776 1.00 0.00 H
ATOM 3743 HB2 SER A 237 19.318 −14.298 −33.167 1.00 0.00 H
ATOM 3744 HB3 SER A 237 20.696 −15.110 −32.400 1.00 0.00 H
ATOM 3745 HG SER A 237 19.886 −14.136 −30.719 1.00 0.00 H
ATOM 3746 N PRO A 238 20.635 −18.048 −31.853 1.00 0.00 N
ATOM 3747 CA PRO A 238 20.950 −19.217 −31.049 1.00 0.00 C
ATOM 3748 C PRO A 238 20.926 −18.900 −29.551 1.00 0.00 C
ATOM 3749 O PRO A 238 21.413 −17.858 −29.106 1.00 0.00 O
ATOM 3750 CB PRO A 238 22.297 −19.750 −31.538 1.00 0.00 C
ATOM 3751 CG PRO A 238 22.463 −19.121 −32.921 1.00 0.00 C

ATOM	3752	CD	PRO	A	238	21.701	−17.800	−32.813	1.00	0.00	C
ATOM	3753	HA	PRO	A	238	20.189	−19.960	−31.269	1.00	0.00	H
ATOM	3754	HB2	PRO	A	238	22.300	−20.840	−31.592	1.00	0.00	H
ATOM	3755	HB3	PRO	A	238	23.095	−19.408	−30.888	1.00	0.00	H
ATOM	3756	HG2	PRO	A	238	23.513	−18.963	−33.175	1.00	0.00	H
ATOM	3757	HG3	PRO	A	238	21.986	−19.759	−33.664	1.00	0.00	H
ATOM	3758	HD2	PRO	A	238	21.328	−17.518	−33.798	1.00	0.00	H
ATOM	3759	HD3	PRO	A	238	22.364	−17.025	−32.426	1.00	0.00	H
ATOM	3760	N	SER	A	239	20.348	−19.803	−28.769	1.00	0.00	N
ATOM	3761	CA	SER	A	239	19.942	−19.567	−27.378	1.00	0.00	C
ATOM	3762	C	SER	A	239	20.324	−20.732	−26.459	1.00	0.00	C
ATOM	3763	O	SER	A	239	20.978	−21.674	−26.889	1.00	0.00	O
ATOM	3764	CB	SER	A	239	18.436	−19.250	−27.353	1.00	0.00	C
ATOM	3765	OG	SER	A	239	17.650	−20.206	−28.044	1.00	0.00	O
ATOM	3766	H	SER	A	239	19.967	−20.631	−29.220	1.00	0.00	H
ATOM	3767	HA	SER	A	239	20.461	−18.690	−26.992	1.00	0.00	H
ATOM	3768	HB2	SER	A	239	18.088	−19.188	−26.323	1.00	0.00	H
ATOM	3769	HB3	SER	A	239	18.277	−18.276	−27.822	1.00	0.00	H
ATOM	3770	HG	SER	A	239	17.740	−21.064	−27.624	1.00	0.00	H
ATOM	3771	N	LYS	A	240	20.030	−20.626	−25.164	1.00	0.00	N
ATOM	3772	CA	LYS	A	240	20.440	−21.590	−24.133	1.00	0.00	C
ATOM	3773	C	LYS	A	240	19.489	−21.507	−22.945	1.00	0.00	C
ATOM	3774	O	LYS	A	240	19.059	−20.413	−22.584	1.00	0.00	O
ATOM	3775	CB	LYS	A	240	21.907	−21.288	−23.771	1.00	0.00	C
ATOM	3776	CG	LYS	A	240	22.463	−21.918	−22.488	1.00	0.00	C
ATOM	3777	CD	LYS	A	240	23.974	−21.646	−22.418	1.00	0.00	C
ATOM	3778	CE	LYS	A	240	24.557	−21.807	−21.009	1.00	0.00	C
ATOM	3779	NZ	LYS	A	240	26.030	−21.700	−21.040	1.00	0.00	N
ATOM	3780	H	LYS	A	240	19.565	−19.778	−24.849	1.00	0.00	H
ATOM	3781	HA	LYS	A	240	20.393	−22.605	−24.527	1.00	0.00	H

ATOM 3782 HB2 LYS A 240 22.018 −20.212 −23.652 1.00 0.00 H
ATOM 3783 HB3 LYS A 240 22.535 −21.600 −24.611 1.00 0.00 H
ATOM 3784 HG2 LYS A 240 22.269 −22.989 −22.470 1.00 0.00 H
ATOM 3785 HG3 LYS A 240 21.980 −21.444 −21.640 1.00 0.00 H
ATOM 3786 HD2 LYS A 240 24.486 −22.318 −23.105 1.00 0.00 H
ATOM 3787 HD3 LYS A 240 24.160 −20.619 −22.730 1.00 0.00 H
ATOM 3788 HE2 LYS A 240 24.265 −22.783 −20.599 1.00 0.00 H
ATOM 3789 HE3 LYS A 240 24.130 −21.027 −20.368 1.00 0.00 H
ATOM 3790 HZ1 LYS A 240 26.447 −22.624 −21.122 1.00 0.00 H
ATOM 3791 HZ2 LYS A 240 26.382 −21.071 −21.756 1.00 0.00 H
ATOM 3792 HZ3 LYS A 240 26.420 −21.388 −20.160 1.00 0.00 H
ATOM 3793 N ILE A 241 19.182 −22.642 −22.323 1.00 0.00 N
ATOM 3794 CA ILE A 241 18.385 −22.719 −21.090 1.00 0.00 C
ATOM 3795 C ILE A 241 19.063 −23.682 −20.113 1.00 0.00 C
ATOM 3796 O ILE A 241 19.596 −24.700 −20.544 1.00 0.00 O
ATOM 3797 CB ILE A 241 16.902 −23.058 −21.381 1.00 0.00 C
ATOM 3798 CG1 ILE A 241 16.067 −22.967 −20.081 1.00 0.00 C
ATOM 3799 CG2 ILE A 241 16.719 −24.417 −22.073 1.00 0.00 C
ATOM 3800 CD1 ILE A 241 14.549 −23.078 −20.279 1.00 0.00 C
ATOM 3801 H ILE A 241 19.635 −23.499 −22.633 1.00 0.00 H
ATOM 3802 HA ILE A 241 18.400 −21.736 −20.625 1.00 0.00 H
ATOM 3803 HB ILE A 241 16.535 −22.304 −22.072 1.00 0.00 H
ATOM 3804 HG12 ILE A 241 16.382 −23.749 −19.390 1.00 0.00 H
ATOM 3805 HG13 ILE A 241 16.264 −22.003 −19.610 1.00 0.00 H
ATOM 3806 HG21 ILE A 241 17.289 −24.439 −22.999 1.00 0.00 H
ATOM 3807 HG22 ILE A 241 15.674 −24.584 −22.326 1.00 0.00 H
ATOM 3808 HG23 ILE A 241 17.053 −25.219 −21.418 1.00 0.00 H
ATOM 3809 HD11 ILE A 241 14.208 −22.338 −21.003 1.00 0.00 H
ATOM 3810 HD12 ILE A 241 14.050 −22.899 −19.328 1.00 0.00 H
ATOM 3811 HD13 ILE A 241 14.279 −24.076 −20.624 1.00 0.00 H

ATOM	3812	N	TYR	A	242	19.190	−23.295	−18.840	1.00	0.00	N
ATOM	3813	CA	TYR	A	242	20.089	−23.972	−17.897	1.00	0.00	C
ATOM	3814	C	TYR	A	242	19.654	−23.806	−16.429	1.00	0.00	C
ATOM	3815	O	TYR	A	242	19.313	−22.713	−15.976	1.00	0.00	O
ATOM	3816	CB	TYR	A	242	21.533	−23.505	−18.147	1.00	0.00	C
ATOM	3817	CG	TYR	A	242	21.815	−22.020	−17.985	1.00	0.00	C
ATOM	3818	CD1	TYR	A	242	22.528	−21.574	−16.862	1.00	0.00	C
ATOM	3819	CD2	TYR	A	242	21.433	−21.089	−18.973	1.00	0.00	C
ATOM	3820	CE1	TYR	A	242	22.873	−20.219	−16.732	1.00	0.00	C
ATOM	3821	CE2	TYR	A	242	21.788	−19.730	−18.857	1.00	0.00	C
ATOM	3822	CZ	TYR	A	242	22.519	−19.293	−17.733	1.00	0.00	C
ATOM	3823	OH	TYR	A	242	22.879	−17.990	−17.604	1.00	0.00	O
ATOM	3824	H	TYR	A	242	18.831	−22.380	−18.577	1.00	0.00	H
ATOM	3825	HA	TYR	A	242	20.063	−25.040	−18.120	1.00	0.00	H
ATOM	3826	HB2	TYR	A	242	22.188	−24.065	−17.480	1.00	0.00	H
ATOM	3827	HB3	TYR	A	242	21.816	−23.789	−19.162	1.00	0.00	H
ATOM	3828	HD1	TYR	A	242	22.830	−22.274	−16.096	1.00	0.00	H
ATOM	3829	HD2	TYR	A	242	20.851	−21.414	−19.820	1.00	0.00	H
ATOM	3830	HE1	TYR	A	242	23.421	−19.895	−15.865	1.00	0.00	H
ATOM	3831	HE2	TYR	A	242	21.488	−19.015	−19.608	1.00	0.00	H
ATOM	3832	HH	TYR	A	242	23.598	−17.760	−18.221	1.00	0.00	H
ATOM	3833	N	ASN	A	243	19.658	−24.910	−15.672	1.00	0.00	N
ATOM	3834	CA	ASN	A	243	19.094	−25.023	−14.315	1.00	0.00	C
ATOM	3835	C	ASN	A	243	19.984	−24.412	−13.205	1.00	0.00	C
ATOM	3836	O	ASN	A	243	20.363	−25.095	−12.251	1.00	0.00	O
ATOM	3837	CB	ASN	A	243	18.737	−26.503	−14.042	1.00	0.00	C
ATOM	3838	CG	ASN	A	243	17.537	−27.004	−14.826	1.00	0.00	C
ATOM	3839	OD1	ASN	A	243	16.602	−26.276	−15.103	1.00	0.00	O
ATOM	3840	ND2	ASN	A	243	17.488	−28.277	−15.142	1.00	0.00	N
ATOM	3841	H	ASN	A	243	19.974	−25.762	−16.111	1.00	0.00	H

ATOM 3842 HA ASN A 243 18.165 −24.451 −14.285 1.00 0.00 H
ATOM 3843 HB2 ASN A 243 19.605 −27.129 −14.253 1.00 0.00 H
ATOM 3844 HB3 ASN A 243 18.484 −26.627 −12.989 1.00 0.00 H
ATOM 3845 HD21 ASN A 243 18.243 −28.889 −14.904 1.00 0.00 H
ATOM 3846 HD22 ASN A 243 16.638 −28.603 −15.579 1.00 0.00 H
ATOM 3847 N SER A 244 20.320 −23.122 −13.300 1.00 0.00 N
ATOM 3848 CA SER A 244 21.146 −22.438 −12.292 1.00 0.00 C
ATOM 3849 C SER A 244 20.483 −22.451 −10.914 1.00 0.00 C
ATOM 3850 O SER A 244 19.441 −21.827 −10.715 1.00 0.00 O
ATOM 3851 CB SER A 244 21.423 −20.979 −12.658 1.00 0.00 C
ATOM 3852 OG SER A 244 22.041 −20.887 −13.917 1.00 0.00 O
ATOM 3853 H SER A 244 19.959 −22.596 −14.087 1.00 0.00 H
ATOM 3854 HA SER A 244 22.107 −22.949 −12.227 1.00 0.00 H
ATOM 3855 HB2 SER A 244 20.494 −20.412 −12.658 1.00 0.00 H
ATOM 3856 HB3 SER A 244 22.090 −20.547 −11.909 1.00 0.00 H
ATOM 3857 HG SER A 244 21.383 −20.965 −14.616 1.00 0.00 H
ATOM 3858 N LEU A 245 21.168 −23.003 −9.908 1.00 0.00 N
ATOM 3859 CA LEU A 245 20.837 −22.810 −8.487 1.00 0.00 C
ATOM 3860 C LEU A 245 19.348 −23.088 −8.158 1.00 0.00 C
ATOM 3861 O LEU A 245 18.684 −22.270 −7.524 1.00 0.00 O
ATOM 3862 CB LEU A 245 21.321 −21.415 −8.024 1.00 0.00 C
ATOM 3863 CG LEU A 245 22.781 −21.057 −8.365 1.00 0.00 C
ATOM 3864 CD1 LEU A 245 23.047 −19.604 −7.985 1.00 0.00 C
ATOM 3865 CD2 LEU A 245 23.780 −21.952 −7.631 1.00 0.00 C
ATOM 3866 H LEU A 245 22.026 −23.483 −10.137 1.00 0.00 H
ATOM 3867 HA LEU A 245 21.401 −23.552 −7.921 1.00 0.00 H
ATOM 3868 HB2 LEU A 245 20.684 −20.665 −8.488 1.00 0.00 H
ATOM 3869 HB3 LEU A 245 21.185 −21.338 −6.944 1.00 0.00 H
ATOM 3870 HG LEU A 245 22.946 −21.153 −9.438 1.00 0.00 H
ATOM 3871 HD11 LEU A 245 22.375 −18.949 −8.539 1.00 0.00 H

ATOM	3872	HD12	LEU	A	245	22.911	−19.460	−6.913	1.00	0.00	H
ATOM	3873	HD13	LEU	A	245	24.072	−19.352	−8.256	1.00	0.00	H
ATOM	3874	HD21	LEU	A	245	24.795	−21.631	−7.869	1.00	0.00	H
ATOM	3875	HD22	LEU	A	245	23.663	−22.987	−7.948	1.00	0.00	H
ATOM	3876	HD23	LEU	A	245	23.628	−21.878	−6.554	1.00	0.00	H
ATOM	3877	N	GLY	A	246	18.781	−24.152	−8.742	1.00	0.00	N
ATOM	3878	CA	GLY	A	246	17.403	−24.604	−8.489	1.00	0.00	C
ATOM	3879	C	GLY	A	246	16.291	−23.976	−9.343	1.00	0.00	C
ATOM	3880	O	GLY	A	246	15.135	−24.330	−9.145	1.00	0.00	O
ATOM	3881	H	GLY	A	246	19.359	−24.719	−9.345	1.00	0.00	H
ATOM	3882	HA2	GLY	A	246	17.357	−25.681	−8.640	1.00	0.00	H
ATOM	3883	HA3	GLY	A	246	17.154	−24.406	−7.445	1.00	0.00	H
ATOM	3884	N	GLN	A	247	16.608	−23.114	−10.314	1.00	0.00	N
ATOM	3885	CA	GLN	A	247	15.624	−22.408	−11.156	1.00	0.00	C
ATOM	3886	C	GLN	A	247	16.215	−22.161	−12.562	1.00	0.00	C
ATOM	3887	O	GLN	A	247	17.385	−21.771	−12.643	1.00	0.00	O
ATOM	3888	CB	GLN	A	247	15.210	−21.115	−10.416	1.00	0.00	C
ATOM	3889	CG	GLN	A	247	14.780	−19.906	−11.266	1.00	0.00	C
ATOM	3890	CD	GLN	A	247	13.436	−20.042	−11.966	1.00	0.00	C
ATOM	3891	OE1	GLN	A	247	12.672	−20.968	−11.755	1.00	0.00	O
ATOM	3892	NE2	GLN	A	247	13.119	−19.091	−12.809	1.00	0.00	N
ATOM	3893	H	GLN	A	247	17.581	−22.865	−10.440	1.00	0.00	H
ATOM	3894	HA	GLN	A	247	14.731	−23.023	−11.250	1.00	0.00	H
ATOM	3895	HB2	GLN	A	247	14.413	−21.356	−9.710	1.00	0.00	H
ATOM	3896	HB3	GLN	A	247	16.051	−20.782	−9.814	1.00	0.00	H
ATOM	3897	HG2	GLN	A	247	14.719	−19.035	−10.613	1.00	0.00	H
ATOM	3898	HG3	GLN	A	247	15.536	−19.686	−12.017	1.00	0.00	H
ATOM	3899	HE21	GLN	A	247	13.765	−18.350	−13.000	1.00	0.00	H
ATOM	3900	HE22	GLN	A	247	12.212	−19.109	−13.265	1.00	0.00	H
ATOM	3901	N	PRO	A	248	15.478	−22.358	−13.675	1.00	0.00	N

ATOM 3902 CA PRO A 248 16.040 −22.151 −15.006 1.00 0.00 C
ATOM 3903 C PRO A 248 16.356 −20.681 −15.306 1.00 0.00 C
ATOM 3904 O PRO A 248 15.513 −19.795 −15.154 1.00 0.00 O
ATOM 3905 CB PRO A 248 15.046 −22.736 −16.012 1.00 0.00 C
ATOM 3906 CG PRO A 248 14.166 −23.658 −15.169 1.00 0.00 C
ATOM 3907 CD PRO A 248 14.169 −22.986 −13.798 1.00 0.00 C
ATOM 3908 HA PRO A 248 16.958 −22.731 −15.071 1.00 0.00 H
ATOM 3909 HB2 PRO A 248 15.565 −23.279 −16.800 1.00 0.00 H
ATOM 3910 HB3 PRO A 248 14.441 −21.947 −16.452 1.00 0.00 H
ATOM 3911 HG2 PRO A 248 14.627 −24.641 −15.092 1.00 0.00 H
ATOM 3912 HG3 PRO A 248 13.159 −23.740 −15.577 1.00 0.00 H
ATOM 3913 HD2 PRO A 248 13.394 −22.221 −13.776 1.00 0.00 H
ATOM 3914 HD3 PRO A 248 13.987 −23.734 −13.027 1.00 0.00 H
ATOM 3915 N VAL A 249 17.515 −20.472 −15.924 1.00 0.00 N
ATOM 3916 CA VAL A 249 17.921 −19.226 −16.585 1.00 0.00 C
ATOM 3917 C VAL A 249 17.888 −19.452 −18.093 1.00 0.00 C
ATOM 3918 O VAL A 249 18.285 −20.518 −18.568 1.00 0.00 O
ATOM 3919 CB VAL A 249 19.322 −18.783 −16.121 1.00 0.00 C
ATOM 3920 CG1 VAL A 249 19.739 −17.423 −16.695 1.00 0.00 C
ATOM 3921 CG2 VAL A 249 19.403 −18.657 −14.599 1.00 0.00 C
ATOM 3922 H VAL A 249 18.123 −21.272 −16.060 1.00 0.00 H
ATOM 3923 HA VAL A 249 17.215 −18.434 −16.338 1.00 0.00 H
ATOM 3924 HB VAL A 249 20.043 −19.537 −16.433 1.00 0.00 H
ATOM 3925 HG11 VAL A 249 20.722 −17.148 −16.312 1.00 0.00 H
ATOM 3926 HG12 VAL A 249 19.796 −17.466 −17.781 1.00 0.00 H
ATOM 3927 HG13 VAL A 249 19.028 −16.658 −16.392 1.00 0.00 H
ATOM 3928 HG21 VAL A 249 20.424 −18.423 −14.308 1.00 0.00 H
ATOM 3929 HG22 VAL A 249 18.742 −17.861 −14.261 1.00 0.00 H
ATOM 3930 HG23 VAL A 249 19.108 −19.590 −14.126 1.00 0.00 H
ATOM 3931 N PHE A 250 17.418 −18.464 −18.848 1.00 0.00 N

ATOM 3932 CA PHE A 250 17.481 −18.426 −20.305 1.00 0.00 C
ATOM 3933 C PHE A 250 18.518 −17.397 −20.779 1.00 0.00 C
ATOM 3934 O PHE A 250 18.691 −16.342 −20.166 1.00 0.00 O
ATOM 3935 CB PHE A 250 16.084 −18.132 −20.869 1.00 0.00 C
ATOM 3936 CG PHE A 250 15.937 −18.457 −22.345 1.00 0.00 C
ATOM 3937 CD1 PHE A 250 15.575 −19.760 −22.729 1.00 0.00 C
ATOM 3938 CD2 PHE A 250 16.180 −17.485 −23.334 1.00 0.00 C
ATOM 3939 CE1 PHE A 250 15.489 −20.107 −24.089 1.00 0.00 C
ATOM 3940 CE2 PHE A 250 16.076 −17.827 −24.695 1.00 0.00 C
ATOM 3941 CZ PHE A 250 15.729 −19.136 −25.073 1.00 0.00 C
ATOM 3942 H PHE A 250 17.135 −17.608 −18.380 1.00 0.00 H
ATOM 3943 HA PHE A 250 17.786 −19.405 −20.674 1.00 0.00 H
ATOM 3944 HB2 PHE A 250 15.351 −18.725 −20.320 1.00 0.00 H
ATOM 3945 HB3 PHE A 250 15.848 −17.082 −20.700 1.00 0.00 H
ATOM 3946 HD1 PHE A 250 15.354 −20.492 −21.968 1.00 0.00 H
ATOM 3947 HD2 PHE A 250 16.450 −16.480 −23.047 1.00 0.00 H
ATOM 3948 HE1 PHE A 250 15.211 −21.109 −24.379 1.00 0.00 H
ATOM 3949 HE2 PHE A 250 16.257 −17.083 −25.454 1.00 0.00 H
ATOM 3950 HZ PHE A 250 15.637 −19.396 −26.119 1.00 0.00 H
ATOM 3951 N TYR A 251 19.100 −17.638 −21.951 1.00 0.00 N
ATOM 3952 CA TYR A 251 19.944 −16.706 −22.695 1.00 0.00 C
ATOM 3953 C TYR A 251 19.599 −16.759 −24.190 1.00 0.00 C
ATOM 3954 O TYR A 251 19.381 −17.837 −24.745 1.00 0.00 O
ATOM 3955 CB TYR A 251 21.429 −17.005 −22.416 1.00 0.00 C
ATOM 3956 CG TYR A 251 22.399 −16.590 −23.512 1.00 0.00 C
ATOM 3957 CD1 TYR A 251 23.106 −15.372 −23.450 1.00 0.00 C
ATOM 3958 CD2 TYR A 251 22.575 −17.439 −24.620 1.00 0.00 C
ATOM 3959 CE1 TYR A 251 24.017 −15.034 −24.474 1.00 0.00 C
ATOM 3960 CE2 TYR A 251 23.468 −17.100 −25.644 1.00 0.00 C
ATOM 3961 CZ TYR A 251 24.214 −15.911 −25.563 1.00 0.00 C

ATOM 3962 OH TYR A 251 25.143 −15.666 −26.523 1.00 0.00 O
ATOM 3963 H TYR A 251 18.900 −18.531 −22.395 1.00 0.00 H
ATOM 3964 HA TYR A 251 19.740 −15.694 −22.355 1.00 0.00 H
ATOM 3965 HB2 TYR A 251 21.712 −16.524 −21.477 1.00 0.00 H
ATOM 3966 HB3 TYR A 251 21.548 −18.081 −22.270 1.00 0.00 H
ATOM 3967 HD1 TYR A 251 22.960 −14.702 −22.613 1.00 0.00 H
ATOM 3968 HD2 TYR A 251 22.009 −18.354 −24.696 1.00 0.00 H
ATOM 3969 HE1 TYR A 251 24.593 −14.122 −24.421 1.00 0.00 H
ATOM 3970 HE2 TYR A 251 23.586 −17.756 −26.487 1.00 0.00 H
ATOM 3971 HH TYR A 251 25.031 −14.764 −26.830 1.00 0.00 H
ATOM 3972 N GLN A 252 19.701 −15.618 −24.870 1.00 0.00 N
ATOM 3973 CA GLN A 252 19.637 −15.494 −26.324 1.00 0.00 C
ATOM 3974 C GLN A 252 20.860 −14.718 −26.823 1.00 0.00 C
ATOM 3975 O GLN A 252 21.095 −13.594 −26.387 1.00 0.00 O
ATOM 3976 CB GLN A 252 18.308 −14.816 −26.706 1.00 0.00 C
ATOM 3977 CG GLN A 252 18.171 −14.571 −28.215 1.00 0.00 C
ATOM 3978 CD GLN A 252 16.823 −13.982 −28.631 1.00 0.00 C
ATOM 3979 OE1 GLN A 252 16.031 −13.501 −27.830 1.00 0.00 O
ATOM 3980 NE2 GLN A 252 16.584 −13.846 −29.915 1.00 0.00 N
ATOM 3981 H GLN A 252 19.973 −14.785 −24.353 1.00 0.00 H
ATOM 3982 HA GLN A 252 19.660 −16.484 −26.781 1.00 0.00 H
ATOM 3983 HB2 GLN A 252 17.489 −15.456 −26.385 1.00 0.00 H
ATOM 3984 HB3 GLN A 252 18.229 −13.861 −26.185 1.00 0.00 H
ATOM 3985 HG2 GLN A 252 18.304 −15.520 −28.736 1.00 0.00 H
ATOM 3986 HG3 GLN A 252 18.948 −13.881 −28.541 1.00 0.00 H
ATOM 3987 HE21 GLN A 252 17.261 −14.193 −30.592 1.00 0.00 H
ATOM 3988 HE22 GLN A 252 15.667 −13.558 −30.206 1.00 0.00 H
ATOM 3989 N ALA A 253 21.560 −15.232 −27.836 1.00 0.00 N
ATOM 3990 CA ALA A 253 22.638 −14.482 −28.468 1.00 0.00 C
ATOM 3991 C ALA A 253 22.082 −13.266 −29.226 1.00 0.00 C

ATOM 3992 O ALA A 253 21.237 −13.405 −30.114 1.00 0.00 O
ATOM 3993 CB ALA A 253 23.439 −15.415 −29.375 1.00 0.00 C
ATOM 3994 H ALA A 253 21.343 −16.157 −28.193 1.00 0.00 H
ATOM 3995 HA ALA A 253 23.306 −14.118 −27.685 1.00 0.00 H
ATOM 3996 HB1 ALA A 253 23.830 −16.240 −28.784 1.00 0.00 H
ATOM 3997 HB2 ALA A 253 24.269 −14.862 −29.814 1.00 0.00 H
ATOM 3998 HB3 ALA A 253 22.799 −15.816 −30.161 1.00 0.00 H
ATOM 3999 N SER A 254 22.593 −12.080 −28.905 1.00 0.00 N
ATOM 4000 CA SER A 254 22.084 −10.776 −29.349 1.00 0.00 C
ATOM 4001 C SER A 254 22.418 −10.437 −30.816 1.00 0.00 C
ATOM 4002 O SER A 254 23.145 −9.493 −31.105 1.00 0.00 O
ATOM 4003 CB SER A 254 22.608 −9.713 −28.377 1.00 0.00 C
ATOM 4004 OG SER A 254 22.256 −10.059 −27.050 1.00 0.00 O
ATOM 4005 H SER A 254 23.167 −12.049 −28.074 1.00 0.00 H
ATOM 4006 HA SER A 254 20.998 −10.782 −29.259 1.00 0.00 H
ATOM 4007 HB2 SER A 254 23.691 −9.663 −28.444 1.00 0.00 H
ATOM 4008 HB3 SER A 254 22.191 −8.747 −28.642 1.00 0.00 H
ATOM 4009 HG SER A 254 22.183 −9.229 −26.530 1.00 0.00 H
ATOM 4010 N TYR A 255 21.943 −11.249 −31.763 1.00 0.00 N
ATOM 4011 CA TYR A 255 22.236 −11.165 −33.205 1.00 0.00 C
ATOM 4012 C TYR A 255 21.496 −10.041 −33.969 1.00 0.00 C
ATOM 4013 O TYR A 255 21.383 −10.106 −35.192 1.00 0.00 O
ATOM 4014 CB TYR A 255 22.029 −12.550 −33.848 1.00 0.00 C
ATOM 4015 CG TYR A 255 23.259 −13.434 −33.824 1.00 0.00 C
ATOM 4016 CD1 TYR A 255 24.161 −13.391 −34.905 1.00 0.00 C
ATOM 4017 CD2 TYR A 255 23.505 −14.296 −32.741 1.00 0.00 C
ATOM 4018 CE1 TYR A 255 25.306 −14.211 −34.904 1.00 0.00 C
ATOM 4019 CE2 TYR A 255 24.648 −15.120 −32.737 1.00 0.00 C
ATOM 4020 CZ TYR A 255 25.552 −15.076 −33.818 1.00 0.00 C
ATOM 4021 OH TYR A 255 26.658 −15.866 −33.802 1.00 0.00 O

ATOM 4022 H TYR A 255 21.406 −12.045 −31.441 1.00 0.00 H
ATOM 4023 HA TYR A 255 23.294 −10.924 −33.308 1.00 0.00 H
ATOM 4024 HB2 TYR A 255 21.740 −12.452 −34.891 1.00 0.00 H
ATOM 4025 HB3 TYR A 255 21.203 −13.052 −33.353 1.00 0.00 H
ATOM 4026 HD1 TYR A 255 23.979 −12.723 −35.738 1.00 0.00 H
ATOM 4027 HD2 TYR A 255 22.815 −14.320 −31.913 1.00 0.00 H
ATOM 4028 HE1 TYR A 255 26.004 −14.158 −35.727 1.00 0.00 H
ATOM 4029 HE2 TYR A 255 24.845 −15.789 −31.917 1.00 0.00 H
ATOM 4030 HH TYR A 255 27.157 −15.771 −34.619 1.00 0.00 H
ATOM 4031 N SER A 256 21.010 −8.994 −33.300 1.00 0.00 N
ATOM 4032 CA SER A 256 20.677 −7.727 −33.972 1.00 0.00 C
ATOM 4033 C SER A 256 20.798 −6.541 −33.004 1.00 0.00 C
ATOM 4034 O SER A 256 21.832 −6.417 −32.354 1.00 0.00 O
ATOM 4035 CB SER A 256 19.353 −7.796 −34.762 1.00 0.00 C
ATOM 4036 OG SER A 256 18.171 −7.810 −33.983 1.00 0.00 O
ATOM 4037 H SER A 256 21.242 −8.931 −32.319 1.00 0.00 H
ATOM 4038 HA SER A 256 21.452 −7.552 −34.719 1.00 0.00 H
ATOM 4039 HB2 SER A 256 19.311 −6.919 −35.406 1.00 0.00 H
ATOM 4040 HB3 SER A 256 19.358 −8.668 −35.415 1.00 0.00 H
ATOM 4041 HG SER A 256 17.454 −7.611 −34.607 1.00 0.00 H
ATOM 4042 N TRP A 257 19.880 −5.576 −33.061 1.00 0.00 N
ATOM 4043 CA TRP A 257 20.076 −4.195 −32.608 1.00 0.00 C
ATOM 4044 C TRP A 257 20.078 −3.988 −31.083 1.00 0.00 C
ATOM 4045 O TRP A 257 20.686 −3.028 −30.620 1.00 0.00 O
ATOM 4046 CB TRP A 257 19.001 −3.336 −33.283 1.00 0.00 C
ATOM 4047 CG TRP A 257 17.585 −3.776 −33.052 1.00 0.00 C
ATOM 4048 CD1 TRP A 257 16.830 −4.473 −33.931 1.00 0.00 C
ATOM 4049 CD2 TRP A 257 16.740 −3.570 −31.876 1.00 0.00 C
ATOM 4050 NE1 TRP A 257 15.603 −4.758 −33.364 1.00 0.00 N
ATOM 4051 CE2 TRP A 257 15.489 −4.219 −32.099 1.00 0.00 C

ATOM 4052 CE3 TRP A 257 16.891 −2.866 −30.660 1.00 0.00 C
ATOM 4053 CZ2 TRP A 257 14.461 −4.217 −31.145 1.00 0.00 C
ATOM 4054 CZ3 TRP A 257 15.861 −2.843 −29.702 1.00 0.00 C
ATOM 4055 CH2 TRP A 257 14.653 −3.525 −29.935 1.00 0.00 C
ATOM 4056 H TRP A 257 19.040 −5.767 −33.588 1.00 0.00 H
ATOM 4057 HA TRP A 257 21.049 −3.851 −32.960 1.00 0.00 H
ATOM 4058 HB2 TRP A 257 19.189 −3.326 −34.357 1.00 0.00 H
ATOM 4059 HB3 TRP A 257 19.109 −2.314 −32.930 1.00 0.00 H
ATOM 4060 HD1 TRP A 257 17.157 −4.774 −34.921 1.00 0.00 H
ATOM 4061 HE3 TRP A 257 17.813 −2.336 −30.461 1.00 0.00 H
ATOM 4062 HZ2 TRP A 257 13.537 −4.744 −31.331 1.00 0.00 H
ATOM 4063 HZ3 TRP A 257 16.011 −2.303 −28.776 1.00 0.00 H
ATOM 4064 HH2 TRP A 257 13.875 −3.518 −29.185 1.00 0.00 H
ATOM 4065 HE1 TRP A 257 14.892 −5.302 −33.829 1.00 0.00 H
ATOM 4066 N ASP A 258 19.457 −4.875 −30.303 1.00 0.00 N
ATOM 4067 CA ASP A 258 19.566 −4.928 −28.833 1.00 0.00 C
ATOM 4068 C ASP A 258 20.871 −5.653 −28.450 1.00 0.00 C
ATOM 4069 O ASP A 258 20.862 −6.870 −28.266 1.00 0.00 O
ATOM 4070 CB ASP A 258 18.299 −5.613 −28.266 1.00 0.00 C
ATOM 4071 CG ASP A 258 18.248 −5.793 −26.738 1.00 0.00 C
ATOM 4072 OD1 ASP A 258 19.117 −5.276 −26.005 1.00 0.00 O
ATOM 4073 OD2 ASP A 258 17.266 −6.389 −26.227 1.00 0.00 O
ATOM 4074 H ASP A 258 18.995 −5.644 −30.757 1.00 0.00 H
ATOM 4075 HA ASP A 258 19.613 −3.915 −28.429 1.00 0.00 H
ATOM 4076 HB2 ASP A 258 17.427 −5.031 −28.566 1.00 0.00 H
ATOM 4077 HB3 ASP A 258 18.211 −6.596 −28.723 1.00 0.00 H
ATOM 4078 N THR A 259 22.026 −4.993 −28.636 1.00 0.00 N
ATOM 4079 CA THR A 259 23.347 −5.671 −28.669 1.00 0.00 C
ATOM 4080 C THR A 259 23.848 −6.126 −27.297 1.00 0.00 C
ATOM 4081 O THR A 259 24.853 −6.842 −27.205 1.00 0.00 O

ATOM 4082 CB THR A 259 24.438 −4.816 −29.336 1.00 0.00 C
ATOM 4083 OG1 THR A 259 24.910 −3.772 −28.516 1.00 0.00 O
ATOM 4084 CG2 THR A 259 23.978 −4.224 −30.663 1.00 0.00 C
ATOM 4085 H THR A 259 21.989 −4.007 −28.886 1.00 0.00 H
ATOM 4086 HA THR A 259 23.235 −6.569 −29.275 1.00 0.00 H
ATOM 4087 HB THR A 259 25.286 −5.467 −29.530 1.00 0.00 H
ATOM 4088 HG1 THR A 259 25.388 −4.172 −27.777 1.00 0.00 H
ATOM 4089 HG21 THR A 259 23.209 −3.478 −30.490 1.00 0.00 H
ATOM 4090 HG22 THR A 259 23.583 −5.010 −31.298 1.00 0.00 H
ATOM 4091 HG23 THR A 259 24.817 −3.760 −31.169 1.00 0.00 H
ATOM 4092 N MET A 260 23.191 −5.654 −26.237 1.00 0.00 N
ATOM 4093 CA MET A 260 23.445 −6.013 −24.843 1.00 0.00 C
ATOM 4094 C MET A 260 23.123 −7.489 −24.592 1.00 0.00 C
ATOM 4095 O MET A 260 22.294 −8.077 −25.287 1.00 0.00 O
ATOM 4096 CB MET A 260 22.605 −5.113 −23.918 1.00 0.00 C
ATOM 4097 CG MET A 260 22.806 −3.613 −24.184 1.00 0.00 C
ATOM 4098 SD MET A 260 24.529 −3.038 −24.210 1.00 0.00 S
ATOM 4099 CE MET A 260 24.994 −3.363 −22.491 1.00 0.00 C
ATOM 4100 H MET A 260 22.361 −5.113 −26.430 1.00 0.00 H
ATOM 4101 HA MET A 260 24.500 −5.850 −24.627 1.00 0.00 H
ATOM 4102 HB2 MET A 260 21.548 −5.345 −24.058 1.00 0.00 H
ATOM 4103 HB3 MET A 260 22.862 −5.331 −22.881 1.00 0.00 H
ATOM 4104 HG2 MET A 260 22.357 −3.367 −25.147 1.00 0.00 H
ATOM 4105 HG3 MET A 260 22.265 −3.052 −23.421 1.00 0.00 H
ATOM 4106 HE1 MET A 260 24.352 −2.776 −21.840 1.00 0.00 H
ATOM 4107 HE2 MET A 260 24.873 −4.420 −22.259 1.00 0.00 H
ATOM 4108 HE3 MET A 260 26.031 −3.077 −22.328 1.00 0.00 H
ATOM 4109 N ILE A 261 23.755 −8.113 −23.594 1.00 0.00 N
ATOM 4110 CA ILE A 261 23.518 −9.533 −23.295 1.00 0.00 C
ATOM 4111 C ILE A 261 22.059 −9.792 −22.872 1.00 0.00 C

ATOM 4112 O ILE A 261 21.601 −9.360 −21.813 1.00 0.00 O
ATOM 4113 CB ILE A 261 24.561 −10.088 −22.299 1.00 0.00 C
ATOM 4114 CG1 ILE A 261 24.378 −11.617 −22.165 1.00 0.00 C
ATOM 4115 CG2 ILE A 261 24.552 −9.373 −20.933 1.00 0.00 C
ATOM 4116 CD1 ILE A 261 25.534 −12.326 −21.448 1.00 0.00 C
ATOM 4117 H ILE A 261 24.461 −7.605 −23.070 1.00 0.00 H
ATOM 4118 HA ILE A 261 23.673 −10.072 −24.231 1.00 0.00 H
ATOM 4119 HB ILE A 261 25.540 −9.919 −22.749 1.00 0.00 H
ATOM 4120 HG12 ILE A 261 23.449 −11.839 −21.640 1.00 0.00 H
ATOM 4121 HG13 ILE A 261 24.304 −12.046 −23.165 1.00 0.00 H
ATOM 4122 HG21 ILE A 261 25.359 −9.745 −20.307 1.00 0.00 H
ATOM 4123 HG22 ILE A 261 23.614 −9.531 −20.410 1.00 0.00 H
ATOM 4124 HG23 ILE A 261 24.685 −8.300 −21.073 1.00 0.00 H
ATOM 4125 HD11 ILE A 261 25.372 −13.404 −21.483 1.00 0.00 H
ATOM 4126 HD12 ILE A 261 26.477 −12.095 −21.945 1.00 0.00 H
ATOM 4127 HD13 ILE A 261 25.583 −12.015 −20.407 1.00 0.00 H
ATOM 4128 N LYS A 262 21.324 −10.557 −23.686 1.00 0.00 N
ATOM 4129 CA LYS A 262 19.965 −11.004 −23.363 1.00 0.00 C
ATOM 4130 C LYS A 262 20.021 −12.295 −22.544 1.00 0.00 C
ATOM 4131 O LYS A 262 20.094 −13.389 −23.098 1.00 0.00 O
ATOM 4132 CB LYS A 262 19.143 −11.166 −24.648 1.00 0.00 C
ATOM 4133 CG LYS A 262 18.958 −9.846 −25.415 1.00 0.00 C
ATOM 4134 CD LYS A 262 18.036 −10.048 −26.622 1.00 0.00 C
ATOM 4135 CE LYS A 262 16.576 −10.145 −26.168 1.00 0.00 C
ATOM 4136 NZ LYS A 262 15.732 −10.797 −27.188 1.00 0.00 N
ATOM 4137 H LYS A 262 21.719 −10.837 −24.576 1.00 0.00 H
ATOM 4138 HA LYS A 262 19.473 −10.246 −22.752 1.00 0.00 H
ATOM 4139 HB2 LYS A 262 19.629 −11.887 −25.302 1.00 0.00 H
ATOM 4140 HB3 LYS A 262 18.166 −11.561 −24.377 1.00 0.00 H
ATOM 4141 HG2 LYS A 262 19.925 −9.497 −25.774 1.00 0.00 H

ATOM 4142 HG3 LYS A 262 18.538 −9.085 −24.756 1.00 0.00 H
ATOM 4143 HD2 LYS A 262 18.140 −9.192 −27.291 1.00 0.00 H
ATOM 4144 HD3 LYS A 262 18.337 −10.951 −27.157 1.00 0.00 H
ATOM 4145 HE2 LYS A 262 16.509 −10.706 −25.232 1.00 0.00 H
ATOM 4146 HE3 LYS A 262 16.216 −9.128 −25.979 1.00 0.00 H
ATOM 4147 HZ1 LYS A 262 15.982 −10.487 −28.123 1.00 0.00 H
ATOM 4148 HZ2 LYS A 262 15.824 −11.811 −27.178 1.00 0.00 H
ATOM 4149 HZ3 LYS A 262 14.760 −10.538 −27.038 1.00 0.00 H
ATOM 4150 N LEU A 263 19.926 −12.165 −21.223 1.00 0.00 N
ATOM 4151 CA LEU A 263 19.771 −13.278 −20.276 1.00 0.00 C
ATOM 4152 C LEU A 263 18.744 −12.944 −19.186 1.00 0.00 C
ATOM 4153 O LEU A 263 18.462 −11.769 −18.959 1.00 0.00 O
ATOM 4154 CB LEU A 263 21.144 −13.706 −19.709 1.00 0.00 C
ATOM 4155 CG LEU A 263 21.687 −12.914 −18.499 1.00 0.00 C
ATOM 4156 CD1 LEU A 263 23.035 −13.501 −18.074 1.00 0.00 C
ATOM 4157 CD2 LEU A 263 21.903 −11.426 −18.774 1.00 0.00 C
ATOM 4158 H LEU A 263 19.902 −11.228 −20.846 1.00 0.00 H
ATOM 4159 HA LEU A 263 19.369 −14.124 −20.829 1.00 0.00 H
ATOM 4160 HB2 LEU A 263 21.047 −14.746 −19.394 1.00 0.00 H
ATOM 4161 HB3 LEU A 263 21.881 −13.685 −20.514 1.00 0.00 H
ATOM 4162 HG LEU A 263 20.994 −13.011 −17.665 1.00 0.00 H
ATOM 4163 HD11 LEU A 263 23.756 −13.418 −18.886 1.00 0.00 H
ATOM 4164 HD12 LEU A 263 23.416 −12.972 −17.200 1.00 0.00 H
ATOM 4165 HD13 LEU A 263 22.912 −14.552 −17.808 1.00 0.00 H
ATOM 4166 HD21 LEU A 263 22.342 −10.946 −17.899 1.00 0.00 H
ATOM 4167 HD22 LEU A 263 22.568 −11.309 −19.624 1.00 0.00 H
ATOM 4168 HD23 LEU A 263 20.957 −10.937 −18.991 1.00 0.00 H
ATOM 4169 N GLY A 264 18.201 −13.947 −18.498 1.00 0.00 N
ATOM 4170 CA GLY A 264 17.263 −13.753 −17.386 1.00 0.00 C
ATOM 4171 C GLY A 264 16.701 −15.063 −16.836 1.00 0.00 C

```
ATOM   4172  O   GLY A 264      16.761 −16.093 −17.500  1.00  0.00           O
ATOM   4173  H   GLY A 264      18.392 −14.902 −18.793  1.00  0.00           H
ATOM   4174  HA2 GLY A 264      17.766 −13.220 −16.579  1.00  0.00           H
ATOM   4175  HA3 GLY A 264      16.425 −13.147 −17.725  1.00  0.00           H
ATOM   4176  N   ASP A 265      16.158 −15.038 −15.620  1.00  0.00           N
ATOM   4177  CA  ASP A 265      15.366 −16.158 −15.088  1.00  0.00           C
ATOM   4178  C   ASP A 265      14.077 −16.373 −15.905  1.00  0.00           C
ATOM   4179  O   ASP A 265      13.507 −15.413 −16.431  1.00  0.00           O
ATOM   4180  CB  ASP A 265      15.066 −15.909 −13.599  1.00  0.00           C
ATOM   4181  CG  ASP A 265      16.284 −16.202 −12.721  1.00  0.00           C
ATOM   4182  OD1 ASP A 265      16.769 −17.349 −12.790  1.00  0.00           O
ATOM   4183  OD2 ASP A 265      16.570 −15.445 −11.768  1.00  0.00           O
ATOM   4184  H   ASP A 265      16.061 −14.148 −15.159  1.00  0.00           H
ATOM   4185  HA  ASP A 265      15.950 −17.076 −15.165  1.00  0.00           H
ATOM   4186  HB2 ASP A 265      14.736 −14.879 −13.454  1.00  0.00           H
ATOM   4187  HB3 ASP A 265      14.256 −16.568 −13.285  1.00  0.00           H
ATOM   4188  N   VAL A 266      13.608 −17.621 −16.024  1.00  0.00           N
ATOM   4189  CA  VAL A 266      12.295 −17.905 −16.636  1.00  0.00           C
ATOM   4190  C   VAL A 266      11.166 −17.600 −15.648  1.00  0.00           C
ATOM   4191  O   VAL A 266      11.154 −18.108 −14.529  1.00  0.00           O
ATOM   4192  CB  VAL A 266      12.182 −19.337 −17.208  1.00  0.00           C
ATOM   4193  CG1 VAL A 266      13.321 −19.630 −18.194  1.00  0.00           C
ATOM   4194  CG2 VAL A 266      12.133 −20.456 −16.160  1.00  0.00           C
ATOM   4195  H   VAL A 266      14.120 −18.388 −15.598  1.00  0.00           H
ATOM   4196  HA  VAL A 266      12.171 −17.234 −17.484  1.00  0.00           H
ATOM   4197  HB  VAL A 266      11.250 −19.387 −17.773  1.00  0.00           H
ATOM   4198 HG11 VAL A 266      13.355 −18.847 −18.948  1.00  0.00           H
ATOM   4199 HG12 VAL A 266      13.141 −20.583 −18.690  1.00  0.00           H
ATOM   4200 HG13 VAL A 266      14.278 −19.670 −17.673  1.00  0.00           H
ATOM   4201 HG21 VAL A 266      11.198 −20.408 −15.604  1.00  0.00           H
```

```
ATOM   4202 HG22 VAL A 266      12.964 −20.359 −15.463  1.00  0.00           H
ATOM   4203 HG23 VAL A 266      12.178 −21.427 −16.649  1.00  0.00           H
ATOM   4204  N   ASP A 267      10.173 −16.818 −16.063  1.00  0.00           N
ATOM   4205  CA  ASP A 267       8.910 −16.665 −15.328  1.00  0.00           C
ATOM   4206  C   ASP A 267       7.862 −17.700 −15.777  1.00  0.00           C
ATOM   4207  O   ASP A 267       6.863 −17.916 −15.092  1.00  0.00           O
ATOM   4208  CB  ASP A 267       8.352 −15.234 −15.444  1.00  0.00           C
ATOM   4209  CG  ASP A 267       9.416 −14.160 −15.680  1.00  0.00           C
ATOM   4210  OD1 ASP A 267       9.761 −13.988 −16.869  1.00  0.00           O
ATOM   4211  OD2 ASP A 267       9.451 −13.186 −14.897  1.00  0.00           O
ATOM   4212  H   ASP A 267      10.232 −16.412 −16.993  1.00  0.00           H
ATOM   4213  HA  ASP A 267       9.098 −16.846 −14.268  1.00  0.00           H
ATOM   4214  HB2 ASP A 267       7.648 −15.202 −16.275  1.00  0.00           H
ATOM   4215  HB3 ASP A 267       7.787 −15.005 −14.540  1.00  0.00           H
ATOM   4216  N   THR A 268       8.035 −18.305 −16.959  1.00  0.00           N
ATOM   4217  CA  THR A 268       7.220 −19.432 −17.452  1.00  0.00           C
ATOM   4218  C   THR A 268       7.951 −20.164 −18.589  1.00  0.00           C
ATOM   4219  O   THR A 268       8.837 −19.582 −19.218  1.00  0.00           O
ATOM   4220  CB  THR A 268       5.803 −19.002 −17.913  1.00  0.00           C
ATOM   4221  OG1 THR A 268       5.441 −17.708 −17.480  1.00  0.00           O
ATOM   4222  CG2 THR A 268       4.748 −19.935 −17.320  1.00  0.00           C
ATOM   4223  H   THR A 268       8.834 −18.035 −17.523  1.00  0.00           H
ATOM   4224  HA  THR A 268       7.116 −20.137 −16.629  1.00  0.00           H
ATOM   4225  HB  THR A 268       5.735 −19.029 −18.997  1.00  0.00           H
ATOM   4226 HG21 THR A 268       3.760 −19.623 −17.658  1.00  0.00           H
ATOM   4227 HG22 THR A 268       4.920 −20.959 −17.647  1.00  0.00           H
ATOM   4228 HG23 THR A 268       4.779 −19.887 −16.230  1.00  0.00           H
ATOM   4229  HG1 THR A 268       5.708 −17.665 −16.547  1.00  0.00           H
ATOM   4230  N   VAL A 269       7.686 −21.466 −18.772  1.00  0.00           N
ATOM   4231  CA  VAL A 269       8.402 −22.337 −19.738  1.00  0.00           C
```

ATOM	4232	C	VAL	A	269	7.494	−22.853	−20.860	1.00	0.00	C
ATOM	4233	O	VAL	A	269	7.849	−22.692	−22.025	1.00	0.00	O
ATOM	4234	CB	VAL	A	269	9.124	−23.493	−19.015	1.00	0.00	C
ATOM	4235	CG1	VAL	A	269	9.828	−24.459	−19.977	1.00	0.00	C
ATOM	4236	CG2	VAL	A	269	10.195	−22.941	−18.063	1.00	0.00	C
ATOM	4237	H	VAL	A	269	6.951	−21.878	−18.221	1.00	0.00	H
ATOM	4238	HA	VAL	A	269	9.172	−21.752	−20.241	1.00	0.00	H
ATOM	4239	HB	VAL	A	269	8.396	−24.059	−18.432	1.00	0.00	H
ATOM	4240	HG11	VAL	A	269	9.101	−24.923	−20.645	1.00	0.00	H
ATOM	4241	HG12	VAL	A	269	10.318	−25.257	−19.421	1.00	0.00	H
ATOM	4242	HG13	VAL	A	269	10.567	−23.924	−20.573	1.00	0.00	H
ATOM	4243	HG21	VAL	A	269	9.732	−22.333	−17.286	1.00	0.00	H
ATOM	4244	HG22	VAL	A	269	10.903	−22.327	−18.620	1.00	0.00	H
ATOM	4245	HG23	VAL	A	269	10.730	−23.757	−17.584	1.00	0.00	H
ATOM	4246	N	ASP	A	270	6.255	−23.232	−20.532	1.00	0.00	N
ATOM	4247	CA	ASP	A	270	5.142	−23.327	−21.483	1.00	0.00	C
ATOM	4248	C	ASP	A	270	4.130	−22.195	−21.183	1.00	0.00	C
ATOM	4249	O	ASP	A	270	3.421	−22.291	−20.177	1.00	0.00	O
ATOM	4250	CB	ASP	A	270	4.431	−24.700	−21.403	1.00	0.00	C
ATOM	4251	CG	ASP	A	270	5.237	−25.949	−21.791	1.00	0.00	C
ATOM	4252	OD1	ASP	A	270	6.480	−25.913	−21.897	1.00	0.00	O
ATOM	4253	OD2	ASP	A	270	4.629	−27.043	−21.840	1.00	0.00	O
ATOM	4254	H	ASP	A	270	6.017	−23.307	−19.561	1.00	0.00	H
ATOM	4255	HA	ASP	A	270	5.515	−23.226	−22.500	1.00	0.00	H
ATOM	4256	HB2	ASP	A	270	3.557	−24.658	−22.055	1.00	0.00	H
ATOM	4257	HB3	ASP	A	270	4.072	−24.846	−20.383	1.00	0.00	H
ATOM	4258	N	PRO	A	271	3.998	−21.149	−22.026	1.00	0.00	N
ATOM	4259	CA	PRO	A	271	4.957	−20.696	−23.040	1.00	0.00	C
ATOM	4260	C	PRO	A	271	6.233	−20.106	−22.406	1.00	0.00	C
ATOM	4261	O	PRO	A	271	6.238	−19.761	−21.226	1.00	0.00	O

ATOM 4262 CB PRO A 271 4.200 −19.625 −23.830 1.00 0.00 C
ATOM 4263 CG PRO A 271 3.339 −18.966 −22.753 1.00 0.00 C
ATOM 4264 CD PRO A 271 2.956 −20.143 −21.855 1.00 0.00 C
ATOM 4265 HA PRO A 271 5.223 −21.511 −23.705 1.00 0.00 H
ATOM 4266 HB2 PRO A 271 3.557 −20.103 −24.571 1.00 0.00 H
ATOM 4267 HB3 PRO A 271 4.867 −18.908 −24.312 1.00 0.00 H
ATOM 4268 HG2 PRO A 271 3.937 −18.251 −22.185 1.00 0.00 H
ATOM 4269 HG3 PRO A 271 2.460 −18.479 −23.176 1.00 0.00 H
ATOM 4270 HD2 PRO A 271 2.000 −20.560 −22.174 1.00 0.00 H
ATOM 4271 HD3 PRO A 271 2.892 −19.816 −20.816 1.00 0.00 H
ATOM 4272 N LEU A 272 7.292 −19.889 −23.194 1.00 0.00 N
ATOM 4273 CA LEU A 272 8.548 −19.352 −22.669 1.00 0.00 C
ATOM 4274 C LEU A 272 8.460 −17.831 −22.454 1.00 0.00 C
ATOM 4275 O LEU A 272 8.470 −17.048 −23.410 1.00 0.00 O
ATOM 4276 CB LEU A 272 9.727 −19.773 −23.563 1.00 0.00 C
ATOM 4277 CG LEU A 272 11.075 −19.184 −23.099 1.00 0.00 C
ATOM 4278 CD1 LEU A 272 11.530 −19.709 −21.735 1.00 0.00 C
ATOM 4279 CD2 LEU A 272 12.164 −19.495 −24.118 1.00 0.00 C
ATOM 4280 H LEU A 272 7.242 −20.124 −24.175 1.00 0.00 H
ATOM 4281 HA LEU A 272 8.713 −19.815 −21.695 1.00 0.00 H
ATOM 4282 HB2 LEU A 272 9.794 −20.862 −23.574 1.00 0.00 H
ATOM 4283 HB3 LEU A 272 9.525 −19.437 −24.580 1.00 0.00 H
ATOM 4284 HG LEU A 272 10.997 −18.101 −23.034 1.00 0.00 H
ATOM 4285 HD11 LEU A 272 12.528 −19.334 −21.509 1.00 0.00 H
ATOM 4286 HD12 LEU A 272 10.854 −19.362 −20.954 1.00 0.00 H
ATOM 4287 HD13 LEU A 272 11.542 −20.797 −21.735 1.00 0.00 H
ATOM 4288 HD21 LEU A 272 11.833 −19.220 −25.118 1.00 0.00 H
ATOM 4289 HD22 LEU A 272 13.051 −18.917 −23.868 1.00 0.00 H
ATOM 4290 HD23 LEU A 272 12.409 −20.553 −24.091 1.00 0.00 H
ATOM 4291 N ARG A 273 8.621 −17.405 −21.201 1.00 0.00 N

ATOM	4292	CA	ARG	A	273	8.668	−15.999	−20.776	1.00	0.00	C
ATOM	4293	C	ARG	A	273	9.810	−15.802	−19.776	1.00	0.00	C
ATOM	4294	O	ARG	A	273	10.035	−16.637	−18.903	1.00	0.00	O
ATOM	4295	CB	ARG	A	273	7.262	−15.604	−20.275	1.00	0.00	C
ATOM	4296	CG	ARG	A	273	7.163	−14.381	−19.355	1.00	0.00	C
ATOM	4297	CD	ARG	A	273	7.777	−13.066	−19.871	1.00	0.00	C
ATOM	4298	NE	ARG	A	273	8.600	−12.473	−18.803	1.00	0.00	N
ATOM	4299	CZ	ARG	A	273	8.393	−11.370	−18.118	1.00	0.00	C
ATOM	4300	NH1	ARG	A	273	7.639	−10.399	−18.545	1.00	0.00	N
ATOM	4301	NH2	ARG	A	273	8.920	−11.253	−16.936	1.00	0.00	N
ATOM	4302	H	ARG	A	273	8.672	−18.118	−20.474	1.00	0.00	H
ATOM	4303	HA	ARG	A	273	8.896	−15.384	−21.647	1.00	0.00	H
ATOM	4304	HB2	ARG	A	273	6.620	−15.443	−21.142	1.00	0.00	H
ATOM	4305	HB3	ARG	A	273	6.844	−16.446	−19.718	1.00	0.00	H
ATOM	4306	HG2	ARG	A	273	6.112	−14.202	−19.125	1.00	0.00	H
ATOM	4307	HG3	ARG	A	273	7.644	−14.658	−18.418	1.00	0.00	H
ATOM	4308	HD2	ARG	A	273	8.404	−13.255	−20.741	1.00	0.00	H
ATOM	4309	HD3	ARG	A	273	6.973	−12.393	−20.174	1.00	0.00	H
ATOM	4310	HE	ARG	A	273	9.198	−13.126	−18.294	1.00	0.00	H
ATOM	4311	HH11	ARG	A	273	7.356	−10.416	−19.523	1.00	0.00	H
ATOM	4312	HH12	ARG	A	273	7.552	−9.560	−18.016	1.00	0.00	H
ATOM	4313	HH21	ARG	A	273	9.345	−12.100	−16.530	1.00	0.00	H
ATOM	4314	HH22	ARG	A	273	8.623	−10.545	−16.303	1.00	0.00	H
ATOM	4315	N	VAL	A	274	10.613	−14.763	−20.019	1.00	0.00	N
ATOM	4316	CA	VAL	A	274	11.924	−14.529	−19.395	1.00	0.00	C
ATOM	4317	C	VAL	A	274	11.989	−13.105	−18.848	1.00	0.00	C
ATOM	4318	O	VAL	A	274	11.765	−12.147	−19.593	1.00	0.00	O
ATOM	4319	CB	VAL	A	274	13.061	−14.764	−20.418	1.00	0.00	C
ATOM	4320	CG1	VAL	A	274	14.453	−14.578	−19.799	1.00	0.00	C
ATOM	4321	CG2	VAL	A	274	13.002	−16.166	−21.040	1.00	0.00	C

ATOM 4322 H VAL A 274 10.353 −14.131 −20.758 1.00 0.00 H
ATOM 4323 HA VAL A 274 12.061 −15.219 −18.561 1.00 0.00 H
ATOM 4324 HB VAL A 274 12.955 −14.041 −21.227 1.00 0.00 H
ATOM 4325 HG11 VAL A 274 15.219 −14.748 −20.555 1.00 0.00 H
ATOM 4326 HG12 VAL A 274 14.573 −13.563 −19.427 1.00 0.00 H
ATOM 4327 HG13 VAL A 274 14.592 −15.285 −18.981 1.00 0.00 H
ATOM 4328 HG21 VAL A 274 13.822 −16.290 −21.746 1.00 0.00 H
ATOM 4329 HG22 VAL A 274 13.076 −16.921 −20.259 1.00 0.00 H
ATOM 4330 HG23 VAL A 274 12.071 −16.304 −21.587 1.00 0.00 H
ATOM 4331 N GLN A 275 12.409 −12.939 −17.594 1.00 0.00 N
ATOM 4332 CA GLN A 275 12.701 −11.630 −17.008 1.00 0.00 C
ATOM 4333 C GLN A 275 14.114 −11.186 −17.397 1.00 0.00 C
ATOM 4334 O GLN A 275 15.071 −11.307 −16.628 1.00 0.00 O
ATOM 4335 CB GLN A 275 12.414 −11.589 −15.498 1.00 0.00 C
ATOM 4336 CG GLN A 275 12.926 −12.780 −14.676 1.00 0.00 C
ATOM 4337 CD GLN A 275 12.600 −12.608 −13.202 1.00 0.00 C
ATOM 4338 OE1 GLN A 275 13.440 −12.240 −12.394 1.00 0.00 O
ATOM 4339 NE2 GLN A 275 11.341 −12.699 −12.834 1.00 0.00 N
ATOM 4340 H GLN A 275 12.590 −13.771 −17.037 1.00 0.00 H
ATOM 4341 HA GLN A 275 12.022 −10.901 −17.453 1.00 0.00 H
ATOM 4342 HB2 GLN A 275 12.840 −10.672 −15.089 1.00 0.00 H
ATOM 4343 HB3 GLN A 275 11.336 −11.523 −15.370 1.00 0.00 H
ATOM 4344 HG2 GLN A 275 12.431 −13.688 −15.013 1.00 0.00 H
ATOM 4345 HG3 GLN A 275 14.003 −12.888 −14.798 1.00 0.00 H
ATOM 4346 HE21 GLN A 275 10.622 −12.923 −13.530 1.00 0.00 H
ATOM 4347 HE22 GLN A 275 11.133 −12.578 −11.863 1.00 0.00 H
ATOM 4348 N TRP A 276 14.245 −10.732 −18.647 1.00 0.00 N
ATOM 4349 CA TRP A 276 15.504 −10.239 −19.203 1.00 0.00 C
ATOM 4350 C TRP A 276 16.131 −9.175 −18.297 1.00 0.00 C
ATOM 4351 O TRP A 276 15.516 −8.140 −18.027 1.00 0.00 O

ATOM 4352 CB TRP A 276 15.290 −9.624 −20.590 1.00 0.00 C
ATOM 4353 CG TRP A 276 14.655 −10.480 −21.637 1.00 0.00 C
ATOM 4354 CD1 TRP A 276 13.444 −10.248 −22.186 1.00 0.00 C
ATOM 4355 CD2 TRP A 276 15.197 −11.638 −22.346 1.00 0.00 C
ATOM 4356 NE1 TRP A 276 13.223 −11.134 −23.215 1.00 0.00 N
ATOM 4357 CE2 TRP A 276 14.281 −11.998 −23.380 1.00 0.00 C
ATOM 4358 CE3 TRP A 276 16.379 −12.403 −22.246 1.00 0.00 C
ATOM 4359 CZ2 TRP A 276 14.544 −13.021 −24.298 1.00 0.00 C
ATOM 4360 CZ3 TRP A 276 16.636 −13.464 −23.137 1.00 0.00 C
ATOM 4361 CH2 TRP A 276 15.730 −13.761 −24.172 1.00 0.00 C
ATOM 4362 H TRP A 276 13.437 −10.783 −19.256 1.00 0.00 H
ATOM 4363 HA TRP A 276 16.185 −11.080 −19.297 1.00 0.00 H
ATOM 4364 HB2 TRP A 276 14.682 −8.725 −20.479 1.00 0.00 H
ATOM 4365 HB3 TRP A 276 16.261 −9.307 −20.971 1.00 0.00 H
ATOM 4366 HD1 TRP A 276 12.774 −9.445 −21.907 1.00 0.00 H
ATOM 4367 HE3 TRP A 276 17.082 −12.173 −21.461 1.00 0.00 H
ATOM 4368 HZ2 TRP A 276 13.826 −13.250 −25.071 1.00 0.00 H
ATOM 4369 HZ3 TRP A 276 17.540 −14.049 −23.034 1.00 0.00 H
ATOM 4370 HH2 TRP A 276 15.934 −14.572 −24.857 1.00 0.00 H
ATOM 4371 HE1 TRP A 276 12.414 −11.051 −23.833 1.00 0.00 H
ATOM 4372 N ARG A 277 17.423 −9.323 −17.997 1.00 0.00 N
ATOM 4373 CA ARG A 277 18.202 −8.260 −17.361 1.00 0.00 C
ATOM 4374 C ARG A 277 18.344 −7.103 −18.347 1.00 0.00 C
ATOM 4375 O ARG A 277 18.833 −7.284 −19.465 1.00 0.00 O
ATOM 4376 CB ARG A 277 19.578 −8.760 −16.886 1.00 0.00 C
ATOM 4377 CG ARG A 277 19.602 −10.100 −16.125 1.00 0.00 C
ATOM 4378 CD ARG A 277 18.531 −10.307 −15.045 1.00 0.00 C
ATOM 4379 NE ARG A 277 18.764 −9.476 −13.856 1.00 0.00 N
ATOM 4380 CZ ARG A 277 18.156 −9.603 −12.693 1.00 0.00 C
ATOM 4381 NH1 ARG A 277 17.198 −10.467 −12.496 1.00 0.00 N

ATOM	4382	NH2	ARG	A	277	18.486	−8.830	−11.706	1.00	0.00	N
ATOM	4383	H	ARG	A	277	17.893	−10.170	−18.300	1.00	0.00	H
ATOM	4384	HA	ARG	A	277	17.648	−7.904	−16.490	1.00	0.00	H
ATOM	4385	HB2	ARG	A	277	20.235	−8.866	−17.751	1.00	0.00	H
ATOM	4386	HB3	ARG	A	277	20.006	−7.987	−16.250	1.00	0.00	H
ATOM	4387	HG2	ARG	A	277	19.491	−10.905	−16.846	1.00	0.00	H
ATOM	4388	HG3	ARG	A	277	20.587	−10.215	−15.673	1.00	0.00	H
ATOM	4389	HD2	ARG	A	277	17.540	−10.105	−15.453	1.00	0.00	H
ATOM	4390	HD3	ARG	A	277	18.562	−11.356	−14.749	1.00	0.00	H
ATOM	4391	HE	ARG	A	277	19.579	−8.856	−13.866	1.00	0.00	H
ATOM	4392	HH11	ARG	A	277	16.963	−11.096	−13.240	1.00	0.00	H
ATOM	4393	HH12	ARG	A	277	16.833	−10.625	−11.578	1.00	0.00	H
ATOM	4394	HH21	ARG	A	277	19.139	−8.069	−11.927	1.00	0.00	H
ATOM	4395	HH22	ARG	A	277	18.040	−8.887	−10.818	1.00	0.00	H
ATOM	4396	N	ASN	A	278	18.054	−5.883	−17.907	1.00	0.00	N
ATOM	4397	CA	ASN	A	278	18.405	−4.664	−18.644	1.00	0.00	C
ATOM	4398	C	ASN	A	278	19.880	−4.295	−18.407	1.00	0.00	C
ATOM	4399	O	ASN	A	278	20.185	−3.249	−17.843	1.00	0.00	O
ATOM	4400	CB	ASN	A	278	17.381	−3.558	−18.338	1.00	0.00	C
ATOM	4401	CG	ASN	A	278	15.995	−3.900	−18.867	1.00	0.00	C
ATOM	4402	OD1	ASN	A	278	15.818	−4.607	−19.852	1.00	0.00	O
ATOM	4403	ND2	ASN	A	278	14.959	−3.418	−18.221	1.00	0.00	N
ATOM	4404	H	ASN	A	278	17.696	−5.791	−16.970	1.00	0.00	H
ATOM	4405	HA	ASN	A	278	18.337	−4.861	−19.715	1.00	0.00	H
ATOM	4406	HB2	ASN	A	278	17.339	−3.393	−17.262	1.00	0.00	H
ATOM	4407	HB3	ASN	A	278	17.699	−2.629	−18.810	1.00	0.00	H
ATOM	4408	HD21	ASN	A	278	15.089	−2.844	−17.409	1.00	0.00	H
ATOM	4409	HD22	ASN	A	278	14.053	−3.684	−18.562	1.00	0.00	H
ATOM	4410	N	ASN	A	279	20.765	−5.264	−18.670	1.00	0.00	N
ATOM	4411	CA	ASN	A	279	22.193	−5.176	−18.399	1.00	0.00	C

ATOM 4412 C ASN A 279 22.821 −4.008 −19.176 1.00 0.00 C
ATOM 4413 O ASN A 279 22.593 −3.875 −20.378 1.00 0.00 O
ATOM 4414 CB ASN A 279 22.844 −6.532 −18.740 1.00 0.00 C
ATOM 4415 CG ASN A 279 24.335 −6.559 −18.440 1.00 0.00 C
ATOM 4416 OD1 ASN A 279 25.143 −5.925 −19.101 1.00 0.00 O
ATOM 4417 ND2 ASN A 279 24.755 −7.296 −17.440 1.00 0.00 N
ATOM 4418 H ASN A 279 20.426 −6.101 −19.127 1.00 0.00 H
ATOM 4419 HA ASN A 279 22.305 −4.981 −17.335 1.00 0.00 H
ATOM 4420 HB2 ASN A 279 22.351 −7.322 −18.176 1.00 0.00 H
ATOM 4421 HB3 ASN A 279 22.714 −6.741 −19.802 1.00 0.00 H
ATOM 4422 HD21 ASN A 279 24.094 −7.747 −16.838 1.00 0.00 H
ATOM 4423 HD22 ASN A 279 25.715 −7.166 −17.171 1.00 0.00 H
ATOM 4424 N SER A 280 23.633 −3.194 −18.499 1.00 0.00 N
ATOM 4425 CA SER A 280 24.206 −1.961 −19.055 1.00 0.00 C
ATOM 4426 C SER A 280 25.717 −2.041 −19.328 1.00 0.00 C
ATOM 4427 O SER A 280 26.358 −0.996 −19.424 1.00 0.00 O
ATOM 4428 CB SER A 280 23.839 −0.764 −18.164 1.00 0.00 C
ATOM 4429 OG SER A 280 24.672 −0.685 −17.025 1.00 0.00 O
ATOM 4430 H SER A 280 23.784 −3.386 −17.512 1.00 0.00 H
ATOM 4431 HA SER A 280 23.735 −1.770 −20.017 1.00 0.00 H
ATOM 4432 HB2 SER A 280 22.797 −0.850 −17.851 1.00 0.00 H
ATOM 4433 HB3 SER A 280 23.950 0.154 −18.743 1.00 0.00 H
ATOM 4434 HG SER A 280 25.550 −0.433 −17.341 1.00 0.00 H
ATOM 4435 N VAL A 281 26.322 −3.240 −19.299 1.00 0.00 N
ATOM 4436 CA VAL A 281 27.792 −3.401 −19.235 1.00 0.00 C
ATOM 4437 C VAL A 281 28.392 −4.503 −20.121 1.00 0.00 C
ATOM 4438 O VAL A 281 29.519 −4.331 −20.576 1.00 0.00 O
ATOM 4439 CB VAL A 281 28.276 −3.578 −17.780 1.00 0.00 C
ATOM 4440 CG1 VAL A 281 27.994 −2.347 −16.913 1.00 0.00 C
ATOM 4441 CG2 VAL A 281 27.683 −4.806 −17.074 1.00 0.00 C

ATOM 4442 H VAL A 281 25.741 −4.058 −19.142 1.00 0.00 H
ATOM 4443 HA VAL A 281 28.251 −2.481 −19.599 1.00 0.00 H
ATOM 4444 HB VAL A 281 29.357 −3.692 −17.815 1.00 0.00 H
ATOM 4445 HG11 VAL A 281 28.360 −1.451 −17.414 1.00 0.00 H
ATOM 4446 HG12 VAL A 281 28.502 −2.446 −15.956 1.00 0.00 H
ATOM 4447 HG13 VAL A 281 26.924 −2.249 −16.735 1.00 0.00 H
ATOM 4448 HG21 VAL A 281 26.599 −4.719 −17.009 1.00 0.00 H
ATOM 4449 HG22 VAL A 281 28.083 −4.887 −16.065 1.00 0.00 H
ATOM 4450 HG23 VAL A 281 27.938 −5.710 −17.618 1.00 0.00 H
ATOM 4451 N ILE A 282 27.653 −5.558 −20.483 1.00 0.00 N
ATOM 4452 CA ILE A 282 28.118 −6.590 −21.430 1.00 0.00 C
ATOM 4453 C ILE A 282 27.390 −6.438 −22.773 1.00 0.00 C
ATOM 4454 O ILE A 282 26.171 −6.586 −22.852 1.00 0.00 O
ATOM 4455 CB ILE A 282 27.965 −8.011 −20.837 1.00 0.00 C
ATOM 4456 CG1 ILE A 282 28.742 −8.247 −19.522 1.00 0.00 C
ATOM 4457 CG2 ILE A 282 28.343 −9.100 −21.863 1.00 0.00 C
ATOM 4458 CD1 ILE A 282 30.271 −8.168 −19.618 1.00 0.00 C
ATOM 4459 H ILE A 282 26.713 −5.645 −20.108 1.00 0.00 H
ATOM 4460 HA ILE A 282 29.181 −6.446 −21.624 1.00 0.00 H
ATOM 4461 HB ILE A 282 26.915 −8.137 −20.596 1.00 0.00 H
ATOM 4462 HG12 ILE A 282 28.406 −7.530 −18.777 1.00 0.00 H
ATOM 4463 HG13 ILE A 282 28.481 −9.236 −19.142 1.00 0.00 H
ATOM 4464 HD11 ILE A 282 30.700 −8.300 −18.625 1.00 0.00 H
ATOM 4465 HD12 ILE A 282 30.575 −7.199 −20.009 1.00 0.00 H
ATOM 4466 HD13 ILE A 282 30.652 −8.958 −20.262 1.00 0.00 H
ATOM 4467 HG21 ILE A 282 28.257 −10.086 −21.404 1.00 0.00 H
ATOM 4468 HG22 ILE A 282 27.678 −9.069 −22.724 1.00 0.00 H
ATOM 4469 HG23 ILE A 282 29.367 −8.960 −22.206 1.00 0.00 H
ATOM 4470 N SER A 283 28.164 −6.314 −23.856 1.00 0.00 N
ATOM 4471 CA SER A 283 27.695 −6.274 −25.249 1.00 0.00 C

ATOM 4472 C SER A 283 28.661 −7.033 −26.161 1.00 0.00 C
ATOM 4473 O SER A 283 29.875 −6.905 −25.995 1.00 0.00 O
ATOM 4474 CB SER A 283 27.566 −4.819 −25.716 1.00 0.00 C
ATOM 4475 OG SER A 283 27.142 −4.757 −27.068 1.00 0.00 O
ATOM 4476 H SER A 283 29.161 −6.257 −23.709 1.00 0.00 H
ATOM 4477 HA SER A 283 26.713 −6.743 −25.315 1.00 0.00 H
ATOM 4478 HB2 SER A 283 26.842 −4.304 −25.087 1.00 0.00 H
ATOM 4479 HB3 SER A 283 28.530 −4.315 −25.618 1.00 0.00 H
ATOM 4480 HG SER A 283 27.785 −5.220 −27.620 1.00 0.00 H
ATOM 4481 N ARG A 284 28.136 −7.689 −27.207 1.00 0.00 N
ATOM 4482 CA ARG A 284 28.914 −8.449 −28.215 1.00 0.00 C
ATOM 4483 C ARG A 284 29.420 −7.589 −29.397 1.00 0.00 C
ATOM 4484 O ARG A 284 28.713 −6.634 −29.742 1.00 0.00 O
ATOM 4485 CB ARG A 284 28.085 −9.670 −28.681 1.00 0.00 C
ATOM 4486 CG ARG A 284 27.147 −9.478 −29.889 1.00 0.00 C
ATOM 4487 CD ARG A 284 26.108 −8.354 −29.782 1.00 0.00 C
ATOM 4488 NE ARG A 284 25.513 −8.047 −31.097 1.00 0.00 N
ATOM 4489 CZ ARG A 284 25.760 −7.007 −31.873 1.00 0.00 C
ATOM 4490 NH1 ARG A 284 26.658 −6.110 −31.603 1.00 0.00 N
ATOM 4491 NH2 ARG A 284 25.081 −6.808 −32.956 1.00 0.00 N
ATOM 4492 H ARG A 284 27.124 −7.683 −27.287 1.00 0.00 H
ATOM 4493 HA ARG A 284 29.803 −8.833 −27.707 1.00 0.00 H
ATOM 4494 HB2 ARG A 284 28.788 −10.450 −28.966 1.00 0.00 H
ATOM 4495 HB3 ARG A 284 27.508 −10.060 −27.840 1.00 0.00 H
ATOM 4496 HG2 ARG A 284 26.617 −10.415 −30.066 1.00 0.00 H
ATOM 4497 HG3 ARG A 284 27.763 −9.298 −30.761 1.00 0.00 H
ATOM 4498 HD2 ARG A 284 25.325 −8.667 −29.097 1.00 0.00 H
ATOM 4499 HD3 ARG A 284 26.544 −7.456 −29.357 1.00 0.00 H
ATOM 4500 HE ARG A 284 24.695 −8.588 −31.360 1.00 0.00 H
ATOM 4501 HH11 ARG A 284 27.328 −6.265 −30.862 1.00 0.00 H

ATOM 4502 HH12 ARG A 284 26.705 −5.297 −32.204 1.00 0.00 H
ATOM 4503 HH21 ARG A 284 24.466 −7.528 −33.321 1.00 0.00 H
ATOM 4504 HH22 ARG A 284 25.262 −5.967 −33.488 1.00 0.00 H
ATOM 4505 N PRO A 285 30.507 −7.957 −30.109 1.00 0.00 N
ATOM 4506 CA PRO A 285 30.891 −7.349 −31.393 1.00 0.00 C
ATOM 4507 C PRO A 285 29.763 −7.421 −32.424 1.00 0.00 C
ATOM 4508 O PRO A 285 29.118 −8.457 −32.563 1.00 0.00 O
ATOM 4509 CB PRO A 285 32.114 −8.123 −31.905 1.00 0.00 C
ATOM 4510 CG PRO A 285 32.098 −9.419 −31.101 1.00 0.00 C
ATOM 4511 CD PRO A 285 31.452 −9.011 −29.779 1.00 0.00 C
ATOM 4512 HA PRO A 285 31.175 −6.309 −31.242 1.00 0.00 H
ATOM 4513 HB2 PRO A 285 32.062 −8.325 −32.977 1.00 0.00 H
ATOM 4514 HB3 PRO A 285 33.027 −7.574 −31.692 1.00 0.00 H
ATOM 4515 HG2 PRO A 285 31.467 −10.155 −31.602 1.00 0.00 H
ATOM 4516 HG3 PRO A 285 33.106 −9.808 −30.959 1.00 0.00 H
ATOM 4517 HD2 PRO A 285 32.214 −8.601 −29.113 1.00 0.00 H
ATOM 4518 HD3 PRO A 285 30.975 −9.875 −29.320 1.00 0.00 H
ATOM 4519 N GLY A 286 29.500 −6.314 −33.119 1.00 0.00 N
ATOM 4520 CA GLY A 286 28.614 −6.288 −34.289 1.00 0.00 C
ATOM 4521 C GLY A 286 29.406 −6.298 −35.591 1.00 0.00 C
ATOM 4522 O GLY A 286 30.530 −6.783 −35.618 1.00 0.00 O
ATOM 4523 H GLY A 286 30.129 −5.528 −32.998 1.00 0.00 H
ATOM 4524 HA2 GLY A 286 27.933 −7.140 −34.290 1.00 0.00 H
ATOM 4525 HA3 GLY A 286 28.023 −5.378 −34.263 1.00 0.00 H
ATOM 4526 N GLN A 287 28.922 −5.534 −36.565 1.00 0.00 N
ATOM 4527 CA GLN A 287 29.726 −4.814 −37.553 1.00 0.00 C
ATOM 4528 C GLN A 287 29.271 −3.339 −37.550 1.00 0.00 C
ATOM 4529 O GLN A 287 28.921 −2.813 −36.493 1.00 0.00 O
ATOM 4530 CB GLN A 287 29.648 −5.525 −38.920 1.00 0.00 C
ATOM 4531 CG GLN A 287 30.257 −6.936 −38.841 1.00 0.00 C

ATOM 4532 CD GLN A 287 30.789 −7.450 −40.172 1.00 0.00 C

ATOM 4533 OE1 GLN A 287 30.216 −7.275 −41.234 1.00 0.00 O

ATOM 4534 NE2 GLN A 287 31.918 −8.122 −40.165 1.00 0.00 N

ATOM 4535 H GLN A 287 27.972 −5.177 −36.466 1.00 0.00 H

ATOM 4536 HA GLN A 287 30.770 −4.811 −37.238 1.00 0.00 H

ATOM 4537 HB2 GLN A 287 30.224 −4.948 −39.644 1.00 0.00 H

ATOM 4538 HB3 GLN A 287 28.613 −5.585 −39.261 1.00 0.00 H

ATOM 4539 HG2 GLN A 287 31.097 −6.914 −38.148 1.00 0.00 H

ATOM 4540 HG3 GLN A 287 29.519 −7.639 −38.454 1.00 0.00 H

ATOM 4541 HE21 GLN A 287 32.434 −8.213 −39.305 1.00 0.00 H

ATOM 4542 HE22 GLN A 287 32.272 −8.410 −41.056 1.00 0.00 H

ATOM 4543 N SER A 288 29.408 −2.608 −38.658 1.00 0.00 N

ATOM 4544 CA SER A 288 29.243 −1.145 −38.701 1.00 0.00 C

ATOM 4545 C SER A 288 27.787 −0.633 −38.694 1.00 0.00 C

ATOM 4546 O SER A 288 27.565 0.519 −39.060 1.00 0.00 O

ATOM 4547 CB SER A 288 30.008 −0.592 −39.908 1.00 0.00 C

ATOM 4548 OG SER A 288 29.399 −1.019 −41.112 1.00 0.00 O

ATOM 4549 H SER A 288 29.699 −3.063 −39.511 1.00 0.00 H

ATOM 4550 HA SER A 288 29.711 −0.733 −37.806 1.00 0.00 H

ATOM 4551 HB2 SER A 288 30.015 0.498 −39.871 1.00 0.00 H

ATOM 4552 HB3 SER A 288 31.039 −0.945 −39.874 1.00 0.00 H

ATOM 4553 HG SER A 288 28.564 −0.541 −41.193 1.00 0.00 H

ATOM 4554 N GLN A 289 26.794 −1.485 −38.414 1.00 0.00 N

ATOM 4555 CA GLN A 289 25.361 −1.137 −38.421 1.00 0.00 C

ATOM 4556 C GLN A 289 24.715 −1.346 −37.037 1.00 0.00 C

ATOM 4557 O GLN A 289 24.024 −0.459 −36.543 1.00 0.00 O

ATOM 4558 CB GLN A 289 24.688 −1.946 −39.547 1.00 0.00 C

ATOM 4559 CG GLN A 289 23.149 −1.946 −39.566 1.00 0.00 C

ATOM 4560 CD GLN A 289 22.488 −0.656 −40.041 1.00 0.00 C

ATOM 4561 OE1 GLN A 289 23.092 0.389 −40.212 1.00 0.00 O

ATOM 4562 NE2 GLN A 289 21.204 −0.705 −40.325 1.00 0.00 N
ATOM 4563 H GLN A 289 27.040 −2.430 −38.150 1.00 0.00 H
ATOM 4564 HA GLN A 289 25.237 −0.079 −38.661 1.00 0.00 H
ATOM 4565 HB2 GLN A 289 25.054 −1.589 −40.511 1.00 0.00 H
ATOM 4566 HB3 GLN A 289 25.007 −2.982 −39.450 1.00 0.00 H
ATOM 4567 HG2 GLN A 289 22.769 −2.172 −38.573 1.00 0.00 H
ATOM 4568 HG3 GLN A 289 22.827 −2.748 −40.231 1.00 0.00 H
ATOM 4569 HE21 GLN A 289 20.720 −1.590 −40.283 1.00 0.00 H
ATOM 4570 HE22 GLN A 289 20.749 0.175 −40.494 1.00 0.00 H
ATOM 4571 N CYS A 290 25.108 −2.403 −36.322 1.00 0.00 N
ATOM 4572 CA CYS A 290 24.735 −2.694 −34.935 1.00 0.00 C
ATOM 4573 C CYS A 290 25.978 −3.091 −34.092 1.00 0.00 C
ATOM 4574 O CYS A 290 26.039 −4.225 −33.598 1.00 0.00 O
ATOM 4575 CB CYS A 290 23.617 −3.757 −34.952 1.00 0.00 C
ATOM 4576 SG CYS A 290 22.037 −3.172 −35.631 1.00 0.00 S
ATOM 4577 H CYS A 290 25.664 −3.112 −36.797 1.00 0.00 H
ATOM 4578 HA CYS A 290 24.319 −1.796 −34.477 1.00 0.00 H
ATOM 4579 HB2 CYS A 290 23.957 −4.601 −35.549 1.00 0.00 H
ATOM 4580 HB3 CYS A 290 23.438 −4.109 −33.936 1.00 0.00 H
ATOM 4581 N PRO A 291 26.996 −2.210 −33.939 1.00 0.00 N
ATOM 4582 CA PRO A 291 28.217 −2.450 −33.146 1.00 0.00 C
ATOM 4583 C PRO A 291 27.943 −2.540 −31.630 1.00 0.00 C
ATOM 4584 O PRO A 291 26.814 −2.340 −31.186 1.00 0.00 O
ATOM 4585 CB PRO A 291 29.138 −1.266 −33.472 1.00 0.00 C
ATOM 4586 CG PRO A 291 28.155 −0.132 −33.747 1.00 0.00 C
ATOM 4587 CD PRO A 291 27.021 −0.852 −34.470 1.00 0.00 C
ATOM 4588 HA PRO A 291 28.701 −3.370 −33.471 1.00 0.00 H
ATOM 4589 HB2 PRO A 291 29.820 −1.019 −32.658 1.00 0.00 H
ATOM 4590 HB3 PRO A 291 29.705 −1.479 −34.378 1.00 0.00 H
ATOM 4591 HG2 PRO A 291 28.599 0.648 −34.367 1.00 0.00 H

ATOM 4592 HG3 PRO A 291 27.791 0.281 −32.804 1.00 0.00 H
ATOM 4593 HD2 PRO A 291 26.078 −0.331 −34.299 1.00 0.00 H
ATOM 4594 HD3 PRO A 291 27.242 −0.885 −35.538 1.00 0.00 H
ATOM 4595 N ARG A 292 28.974 −2.818 −30.810 1.00 0.00 N
ATOM 4596 CA ARG A 292 28.834 −2.867 −29.334 1.00 0.00 C
ATOM 4597 C ARG A 292 28.155 −1.619 −28.757 1.00 0.00 C
ATOM 4598 O ARG A 292 28.538 −0.498 −29.079 1.00 0.00 O
ATOM 4599 CB ARG A 292 30.183 −3.090 −28.615 1.00 0.00 C
ATOM 4600 CG ARG A 292 30.550 −4.571 −28.567 1.00 0.00 C
ATOM 4601 CD ARG A 292 31.876 −4.925 −27.891 1.00 0.00 C
ATOM 4602 NE ARG A 292 31.867 −4.848 −26.423 1.00 0.00 N
ATOM 4603 CZ ARG A 292 32.892 −4.379 −25.733 1.00 0.00 C
ATOM 4604 NH1 ARG A 292 33.262 −4.972 −24.638 1.00 0.00 N
ATOM 4605 NH2 ARG A 292 33.638 −3.397 −26.146 1.00 0.00 N
ATOM 4606 H ARG A 292 29.881 −2.976 −31.229 1.00 0.00 H
ATOM 4607 HA ARG A 292 28.168 −3.697 −29.112 1.00 0.00 H
ATOM 4608 HB2 ARG A 292 30.111 −2.740 −27.586 1.00 0.00 H
ATOM 4609 HB3 ARG A 292 30.958 −2.517 −29.118 1.00 0.00 H
ATOM 4610 HG2 ARG A 292 29.759 −5.104 −28.048 1.00 0.00 H
ATOM 4611 HG3 ARG A 292 30.606 −4.923 −29.595 1.00 0.00 H
ATOM 4612 HD2 ARG A 292 32.087 −5.968 −28.133 1.00 0.00 H
ATOM 4613 HD3 ARG A 292 32.678 −4.326 −28.311 1.00 0.00 H
ATOM 4614 HE ARG A 292 31.346 −5.594 −25.980 1.00 0.00 H
ATOM 4615 HH11 ARG A 292 32.906 −5.888 −24.440 1.00 0.00 H
ATOM 4616 HH12 ARG A 292 34.176 −4.727 −24.261 1.00 0.00 H
ATOM 4617 HH21 ARG A 292 33.468 −3.022 −27.073 1.00 0.00 H
ATOM 4618 HH22 ARG A 292 34.607 −3.434 −25.862 1.00 0.00 H
ATOM 4619 N PHE A 293 27.319 −1.858 −27.746 1.00 0.00 N
ATOM 4620 CA PHE A 293 26.547 −0.867 −26.988 1.00 0.00 C
ATOM 4621 C PHE A 293 25.545 −0.047 −27.821 1.00 0.00 C

ATOM 4622 O PHE A 293 25.055 0.979 −27.347 1.00 0.00 O
ATOM 4623 CB PHE A 293 27.466 −0.017 −26.092 1.00 0.00 C
ATOM 4624 CG PHE A 293 28.371 −0.830 −25.183 1.00 0.00 C
ATOM 4625 CD1 PHE A 293 27.847 −1.411 −24.014 1.00 0.00 C
ATOM 4626 CD2 PHE A 293 29.730 −1.010 −25.502 1.00 0.00 C
ATOM 4627 CE1 PHE A 293 28.674 −2.178 −23.174 1.00 0.00 C
ATOM 4628 CE2 PHE A 293 30.557 −1.778 −24.661 1.00 0.00 C
ATOM 4629 CZ PHE A 293 30.027 −2.367 −23.499 1.00 0.00 C
ATOM 4630 H PHE A 293 27.116 −2.826 −27.544 1.00 0.00 H
ATOM 4631 HA PHE A 293 25.914 −1.441 −26.312 1.00 0.00 H
ATOM 4632 HB2 PHE A 293 26.846 0.625 −25.464 1.00 0.00 H
ATOM 4633 HB3 PHE A 293 28.073 0.638 −26.718 1.00 0.00 H
ATOM 4634 HD1 PHE A 293 26.811 −1.252 −23.754 1.00 0.00 H
ATOM 4635 HD2 PHE A 293 30.140 −0.552 −26.391 1.00 0.00 H
ATOM 4636 HE1 PHE A 293 28.276 −2.603 −22.265 1.00 0.00 H
ATOM 4637 HE2 PHE A 293 31.604 −1.898 −24.897 1.00 0.00 H
ATOM 4638 HZ PHE A 293 30.664 −2.941 −22.837 1.00 0.00 H
ATOM 4639 N ASN A 294 25.114 −0.550 −28.986 1.00 0.00 N
ATOM 4640 CA ASN A 294 23.877 −0.066 −29.589 1.00 0.00 C
ATOM 4641 C ASN A 294 22.645 −0.597 −28.831 1.00 0.00 C
ATOM 4642 O ASN A 294 22.563 −1.768 −28.463 1.00 0.00 O
ATOM 4643 CB ASN A 294 23.820 −0.390 −31.089 1.00 0.00 C
ATOM 4644 CG ASN A 294 22.542 0.156 −31.708 1.00 0.00 C
ATOM 4645 OD1 ASN A 294 22.216 1.324 −31.546 1.00 0.00 O
ATOM 4646 ND2 ASN A 294 21.611 −0.706 −32.032 1.00 0.00 N
ATOM 4647 H ASN A 294 25.459 −1.454 −29.284 1.00 0.00 H
ATOM 4648 HA ASN A 294 23.868 1.023 −29.502 1.00 0.00 H
ATOM 4649 HB2 ASN A 294 24.668 0.067 −31.597 1.00 0.00 H
ATOM 4650 HB3 ASN A 294 23.876 −1.464 −31.242 1.00 0.00 H
ATOM 4651 HD21 ASN A 294 21.680 −1.666 −31.723 1.00 0.00 H

ATOM	4652	HD22	ASN	A	294	20.714	−0.331	−32.322	1.00	0.00	H
ATOM	4653	N	VAL	A	295	21.638	0.266	−28.711	1.00	0.00	N
ATOM	4654	CA	VAL	A	295	20.312	−0.046	−28.147	1.00	0.00	C
ATOM	4655	C	VAL	A	295	19.185	0.477	−29.057	1.00	0.00	C
ATOM	4656	O	VAL	A	295	18.021	0.120	−28.887	1.00	0.00	O
ATOM	4657	CB	VAL	A	295	20.211	0.502	−26.700	1.00	0.00	C
ATOM	4658	CG1	VAL	A	295	18.941	0.051	−25.965	1.00	0.00	C
ATOM	4659	CG2	VAL	A	295	21.400	0.070	−25.823	1.00	0.00	C
ATOM	4660	H	VAL	A	295	21.803	1.201	−29.053	1.00	0.00	H
ATOM	4661	HA	VAL	A	295	20.196	−1.130	−28.104	1.00	0.00	H
ATOM	4662	HB	VAL	A	295	20.209	1.592	−26.742	1.00	0.00	H
ATOM	4663	HG21	VAL	A	295	22.329	0.501	−26.197	1.00	0.00	H
ATOM	4664	HG22	VAL	A	295	21.489	−1.018	−25.825	1.00	0.00	H
ATOM	4665	HG23	VAL	A	295	21.265	0.419	−24.799	1.00	0.00	H
ATOM	4666	HG11	VAL	A	295	18.057	0.476	−26.437	1.00	0.00	H
ATOM	4667	HG12	VAL	A	295	18.871	−1.037	−25.979	1.00	0.00	H
ATOM	4668	HG13	VAL	A	295	18.963	0.396	−24.931	1.00	0.00	H
ATOM	4669	N	CYS	A	296	19.512	1.294	−30.066	1.00	0.00	N
ATOM	4670	CA	CYS	A	296	18.532	1.829	−31.004	1.00	0.00	C
ATOM	4671	C	CYS	A	296	18.097	0.776	−32.034	1.00	0.00	C
ATOM	4672	O	CYS	A	296	18.969	0.156	−32.645	1.00	0.00	O
ATOM	4673	CB	CYS	A	296	19.099	3.074	−31.688	1.00	0.00	C
ATOM	4674	SG	CYS	A	296	19.005	4.528	−30.616	1.00	0.00	S
ATOM	4675	H	CYS	A	296	20.487	1.419	−30.300	1.00	0.00	H
ATOM	4676	HA	CYS	A	296	17.668	2.139	−30.426	1.00	0.00	H
ATOM	4677	HB2	CYS	A	296	20.134	2.893	−31.983	1.00	0.00	H
ATOM	4678	HB3	CYS	A	296	18.525	3.278	−32.595	1.00	0.00	H
ATOM	4679	N	PRO	A	297	16.788	0.623	−32.317	1.00	0.00	N
ATOM	4680	CA	PRO	A	297	16.325	−0.359	−33.288	1.00	0.00	C
ATOM	4681	C	PRO	A	297	16.806	−0.068	−34.708	1.00	0.00	C

ATOM	4682	O	PRO	A	297	16.348	0.886	−35.332	1.00	0.00	O
ATOM	4683	CB	PRO	A	297	14.800	−0.382	−33.183	1.00	0.00	C
ATOM	4684	CG	PRO	A	297	14.533	0.135	−31.773	1.00	0.00	C
ATOM	4685	CD	PRO	A	297	15.657	1.141	−31.558	1.00	0.00	C
ATOM	4686	HA	PRO	A	297	16.704	−1.326	−32.984	1.00	0.00	H
ATOM	4687	HB2	PRO	A	297	14.349	0.302	−33.904	1.00	0.00	H
ATOM	4688	HB3	PRO	A	297	14.423	−1.393	−33.325	1.00	0.00	H
ATOM	4689	HG2	PRO	A	297	13.554	0.605	−31.688	1.00	0.00	H
ATOM	4690	HG3	PRO	A	297	14.636	−0.682	−31.060	1.00	0.00	H
ATOM	4691	HD2	PRO	A	297	15.868	1.204	−30.490	1.00	0.00	H
ATOM	4692	HD3	PRO	A	297	15.372	2.117	−31.950	1.00	0.00	H
ATOM	4693	N	GLU	A	298	17.544	−1.007	−35.297	1.00	0.00	N
ATOM	4694	CA	GLU	A	298	18.062	−0.888	−36.661	1.00	0.00	C
ATOM	4695	C	GLU	A	298	17.939	−2.165	−37.487	1.00	0.00	C
ATOM	4696	O	GLU	A	298	17.790	−3.278	−36.973	1.00	0.00	O
ATOM	4697	CB	GLU	A	298	19.510	−0.356	−36.672	1.00	0.00	C
ATOM	4698	CG	GLU	A	298	19.604	1.177	−36.590	1.00	0.00	C
ATOM	4699	CD	GLU	A	298	18.743	1.918	−37.633	1.00	0.00	C
ATOM	4700	OE1	GLU	A	298	18.409	1.311	−38.684	1.00	0.00	O
ATOM	4701	OE2	GLU	A	298	18.120	2.926	−37.224	1.00	0.00	O
ATOM	4702	H	GLU	A	298	17.859	−1.792	−34.747	1.00	0.00	H
ATOM	4703	HA	GLU	A	298	17.433	−0.179	−37.187	1.00	0.00	H
ATOM	4704	HB2	GLU	A	298	20.069	−0.800	−35.847	1.00	0.00	H
ATOM	4705	HB3	GLU	A	298	19.998	−0.664	−37.598	1.00	0.00	H
ATOM	4706	HG2	GLU	A	298	19.304	1.484	−35.585	1.00	0.00	H
ATOM	4707	HG3	GLU	A	298	20.648	1.466	−36.722	1.00	0.00	H
ATOM	4708	N	VAL	A	299	17.913	−1.979	−38.808	1.00	0.00	N
ATOM	4709	CA	VAL	A	299	17.830	−3.079	−39.773	1.00	0.00	C
ATOM	4710	C	VAL	A	299	19.177	−3.800	−39.835	1.00	0.00	C
ATOM	4711	O	VAL	A	299	20.052	−3.424	−40.612	1.00	0.00	O

ATOM	4712	CB	VAL	A	299	17.370	−2.587	−41.161	1.00	0.00	C
ATOM	4713	CG1	VAL	A	299	17.185	−3.761	−42.132	1.00	0.00	C
ATOM	4714	CG2	VAL	A	299	16.025	−1.855	−41.073	1.00	0.00	C
ATOM	4715	H	VAL	A	299	18.055	−1.030	−39.148	1.00	0.00	H
ATOM	4716	HA	VAL	A	299	17.085	−3.790	−39.415	1.00	0.00	H
ATOM	4717	HB	VAL	A	299	18.107	−1.898	−41.574	1.00	0.00	H
ATOM	4718	HG21	VAL	A	299	16.152	−0.932	−40.508	1.00	0.00	H
ATOM	4719	HG22	VAL	A	299	15.287	−2.480	−40.574	1.00	0.00	H
ATOM	4720	HG23	VAL	A	299	15.672	−1.588	−42.068	1.00	0.00	H
ATOM	4721	HG11	VAL	A	299	18.136	−4.269	−42.289	1.00	0.00	H
ATOM	4722	HG12	VAL	A	299	16.843	−3.382	−43.095	1.00	0.00	H
ATOM	4723	HG13	VAL	A	299	16.459	−4.467	−41.728	1.00	0.00	H
ATOM	4724	N	CYS	A	300	19.344	−4.827	−39.004	1.00	0.00	N
ATOM	4725	CA	CYS	A	300	20.543	−5.663	−38.949	1.00	0.00	C
ATOM	4726	C	CYS	A	300	20.222	−7.121	−38.584	1.00	0.00	C
ATOM	4727	O	CYS	A	300	19.201	−7.409	−37.948	1.00	0.00	O
ATOM	4728	CB	CYS	A	300	21.556	−5.042	−37.976	1.00	0.00	C
ATOM	4729	SG	CYS	A	300	21.033	−4.838	−36.252	1.00	0.00	S
ATOM	4730	H	CYS	A	300	18.661	−4.951	−38.269	1.00	0.00	H
ATOM	4731	HA	CYS	A	300	21.008	−5.672	−39.936	1.00	0.00	H
ATOM	4732	HB2	CYS	A	300	22.462	−5.644	−37.980	1.00	0.00	H
ATOM	4733	HB3	CYS	A	300	21.826	−4.063	−38.362	1.00	0.00	H
ATOM	4734	N	TRP	A	301	21.155	−8.009	−38.940	1.00	0.00	N
ATOM	4735	CA	TRP	A	301	21.322	−9.360	−38.400	1.00	0.00	C
ATOM	4736	C	TRP	A	301	22.824	−9.643	−38.283	1.00	0.00	C
ATOM	4737	O	TRP	A	301	23.486	−9.941	−39.274	1.00	0.00	O
ATOM	4738	CB	TRP	A	301	20.620	−10.421	−39.260	1.00	0.00	C
ATOM	4739	CG	TRP	A	301	20.896	−11.841	−38.840	1.00	0.00	C
ATOM	4740	CD1	TRP	A	301	20.707	−12.337	−37.595	1.00	0.00	C
ATOM	4741	CD2	TRP	A	301	21.484	−12.940	−39.606	1.00	0.00	C

ATOM 4742 NE1 TRP A 301 21.059 −13.669 −37.556 1.00 0.00 N
ATOM 4743 CE2 TRP A 301 21.547 −14.098 −38.771 1.00 0.00 C
ATOM 4744 CE3 TRP A 301 21.986 −13.083 −40.921 1.00 0.00 C
ATOM 4745 CZ2 TRP A 301 22.038 −15.331 −39.216 1.00 0.00 C
ATOM 4746 CZ3 TRP A 301 22.491 −14.317 −41.378 1.00 0.00 C
ATOM 4747 CH2 TRP A 301 22.510 −15.443 −40.535 1.00 0.00 C
ATOM 4748 H TRP A 301 21.952 −7.655 −39.452 1.00 0.00 H
ATOM 4749 HA TRP A 301 20.886 −9.402 −37.405 1.00 0.00 H
ATOM 4750 HB2 TRP A 301 19.544 −10.248 −39.221 1.00 0.00 H
ATOM 4751 HB3 TRP A 301 20.940 −10.301 −40.296 1.00 0.00 H
ATOM 4752 HD1 TRP A 301 20.323 −11.764 −36.761 1.00 0.00 H
ATOM 4753 HE3 TRP A 301 21.995 −12.218 −41.569 1.00 0.00 H
ATOM 4754 HZ2 TRP A 301 22.071 −16.182 −38.551 1.00 0.00 H
ATOM 4755 HZ3 TRP A 301 22.877 −14.399 −42.385 1.00 0.00 H
ATOM 4756 HH2 TRP A 301 22.905 −16.384 −40.892 1.00 0.00 H
ATOM 4757 HE1 TRP A 301 21.002 −14.237 −36.724 1.00 0.00 H
ATOM 4758 N GLU A 302 23.391 −9.299 −37.129 1.00 0.00 N
ATOM 4759 CA GLU A 302 24.831 −9.325 −36.871 1.00 0.00 C
ATOM 4760 C GLU A 302 25.118 −9.435 −35.369 1.00 0.00 C
ATOM 4761 O GLU A 302 24.330 −9.000 −34.522 1.00 0.00 O
ATOM 4762 CB GLU A 302 25.526 −8.089 −37.476 1.00 0.00 C
ATOM 4763 CG GLU A 302 25.227 −6.777 −36.738 1.00 0.00 C
ATOM 4764 CD GLU A 302 25.537 −5.563 −37.618 1.00 0.00 C
ATOM 4765 OE1 GLU A 302 24.724 −5.305 −38.528 1.00 0.00 O
ATOM 4766 OE2 GLU A 302 26.351 −4.724 −37.167 1.00 0.00 O
ATOM 4767 H GLU A 302 22.786 −9.091 −36.344 1.00 0.00 H
ATOM 4768 HA GLU A 302 25.246 −10.208 −37.359 1.00 0.00 H
ATOM 4769 HB2 GLU A 302 25.220 −7.997 −38.518 1.00 0.00 H
ATOM 4770 HB3 GLU A 302 26.604 −8.253 −37.464 1.00 0.00 H
ATOM 4771 HG2 GLU A 302 24.171 −6.748 −36.461 1.00 0.00 H

ATOM	4772	HG3	GLU	A	302	25.815	−6.739	−35.817	1.00	0.00	H
ATOM	4773	N	GLY	A	303	26.263	−10.021	−35.039	1.00	0.00	N
ATOM	4774	CA	GLY	A	303	26.666	−10.348	−33.677	1.00	0.00	C
ATOM	4775	C	GLY	A	303	27.616	−11.544	−33.669	1.00	0.00	C
ATOM	4776	O	GLY	A	303	27.719	−12.250	−34.674	1.00	0.00	O
ATOM	4777	H	GLY	A	303	26.848	−10.393	−35.775	1.00	0.00	H
ATOM	4778	HA2	GLY	A	303	27.147	−9.486	−33.229	1.00	0.00	H
ATOM	4779	HA3	GLY	A	303	25.790	−10.601	−33.081	1.00	0.00	H
ATOM	4780	N	THR	A	304	28.075	−11.935	−32.480	1.00	0.00	N
ATOM	4781	CA	THR	A	304	28.517	−13.313	−32.224	1.00	0.00	C
ATOM	4782	C	THR	A	304	27.970	−13.847	−30.900	1.00	0.00	C
ATOM	4783	O	THR	A	304	27.530	−13.095	−30.025	1.00	0.00	O
ATOM	4784	CB	THR	A	304	30.037	−13.532	−32.336	1.00	0.00	C
ATOM	4785	OG1	THR	A	304	30.704	−13.230	−31.140	1.00	0.00	O
ATOM	4786	CG2	THR	A	304	30.745	−12.780	−33.460	1.00	0.00	C
ATOM	4787	H	THR	A	304	27.949	−11.332	−31.683	1.00	0.00	H
ATOM	4788	HA	THR	A	304	28.084	−13.941	−32.998	1.00	0.00	H
ATOM	4789	HB	THR	A	304	30.173	−14.597	−32.521	1.00	0.00	H
ATOM	4790	HG21	THR	A	304	31.800	−13.058	−33.475	1.00	0.00	H
ATOM	4791	HG22	THR	A	304	30.300	−13.047	−34.418	1.00	0.00	H
ATOM	4792	HG23	THR	A	304	30.665	−11.704	−33.309	1.00	0.00	H
ATOM	4793	HG1	THR	A	304	30.636	−12.260	−31.068	1.00	0.00	H
ATOM	4794	N	TYR	A	305	27.905	−15.172	−30.780	1.00	0.00	N
ATOM	4795	CA	TYR	A	305	27.410	−15.864	−29.593	1.00	0.00	C
ATOM	4796	C	TYR	A	305	28.416	−15.743	−28.428	1.00	0.00	C
ATOM	4797	O	TYR	A	305	29.510	−16.304	−28.493	1.00	0.00	O
ATOM	4798	CB	TYR	A	305	27.116	−17.312	−30.015	1.00	0.00	C
ATOM	4799	CG	TYR	A	305	26.492	−18.197	−28.959	1.00	0.00	C
ATOM	4800	CD1	TYR	A	305	25.155	−18.618	−29.070	1.00	0.00	C
ATOM	4801	CD2	TYR	A	305	27.265	−18.636	−27.877	1.00	0.00	C

ATOM 4802 CE1 TYR A 305 24.593 −19.475 −28.100 1.00 0.00 C
ATOM 4803 CE2 TYR A 305 26.712 −19.482 −26.906 1.00 0.00 C
ATOM 4804 CZ TYR A 305 25.368 −19.888 −26.996 1.00 0.00 C
ATOM 4805 OH TYR A 305 24.827 −20.648 −26.016 1.00 0.00 O
ATOM 4806 H TYR A 305 28.273 −15.733 −31.532 1.00 0.00 H
ATOM 4807 HA TYR A 305 26.472 −15.403 −29.282 1.00 0.00 H
ATOM 4808 HB2 TYR A 305 28.045 −17.775 −30.347 1.00 0.00 H
ATOM 4809 HB3 TYR A 305 26.451 −17.289 −30.880 1.00 0.00 H
ATOM 4810 HD1 TYR A 305 24.548 −18.254 −29.885 1.00 0.00 H
ATOM 4811 HD2 TYR A 305 28.289 −18.311 −27.771 1.00 0.00 H
ATOM 4812 HE1 TYR A 305 23.555 −19.776 −28.156 1.00 0.00 H
ATOM 4813 HE2 TYR A 305 27.315 −19.797 −26.068 1.00 0.00 H
ATOM 4814 HH TYR A 305 24.069 −21.178 −26.360 1.00 0.00 H
ATOM 4815 N ASN A 306 28.004 −15.100 −27.330 1.00 0.00 N
ATOM 4816 CA ASN A 306 28.809 −14.855 −26.126 1.00 0.00 C
ATOM 4817 C ASN A 306 27.963 −15.148 −24.872 1.00 0.00 C
ATOM 4818 O ASN A 306 27.151 −14.301 −24.499 1.00 0.00 O
ATOM 4819 CB ASN A 306 29.293 −13.388 −26.116 1.00 0.00 C
ATOM 4820 CG ASN A 306 30.307 −13.048 −27.189 1.00 0.00 C
ATOM 4821 OD1 ASN A 306 31.470 −12.831 −26.911 1.00 0.00 O
ATOM 4822 ND2 ASN A 306 29.876 −12.792 −28.399 1.00 0.00 N
ATOM 4823 H ASN A 306 27.062 −14.731 −27.310 1.00 0.00 H
ATOM 4824 HA ASN A 306 29.679 −15.507 −26.121 1.00 0.00 H
ATOM 4825 HB2 ASN A 306 28.439 −12.718 −26.214 1.00 0.00 H
ATOM 4826 HB3 ASN A 306 29.761 −13.189 −25.151 1.00 0.00 H
ATOM 4827 HD21 ASN A 306 28.911 −12.939 −28.650 1.00 0.00 H
ATOM 4828 HD22 ASN A 306 30.567 −12.678 −29.128 1.00 0.00 H
ATOM 4829 N ASP A 307 28.012 −16.376 −24.334 1.00 0.00 N
ATOM 4830 CA ASP A 307 27.130 −16.788 −23.224 1.00 0.00 C
ATOM 4831 C ASP A 307 27.815 −16.835 −21.845 1.00 0.00 C

ATOM 4832 O ASP A 307 29.041 −16.904 −21.711 1.00 0.00 O
ATOM 4833 CB ASP A 307 26.279 −18.029 −23.577 1.00 0.00 C
ATOM 4834 CG ASP A 307 26.884 −19.413 −23.321 1.00 0.00 C
ATOM 4835 OD1 ASP A 307 27.427 −19.634 −22.221 1.00 0.00 O
ATOM 4836 OD2 ASP A 307 26.378 −20.368 −23.948 1.00 0.00 O
ATOM 4837 H ASP A 307 28.786 −16.979 −24.582 1.00 0.00 H
ATOM 4838 HA ASP A 307 26.382 −16.002 −23.115 1.00 0.00 H
ATOM 4839 HB2 ASP A 307 25.990 −17.961 −24.626 1.00 0.00 H
ATOM 4840 HB3 ASP A 307 25.359 −17.971 −22.993 1.00 0.00 H
ATOM 4841 N ALA A 308 26.976 −16.691 −20.817 1.00 0.00 N
ATOM 4842 CA ALA A 308 27.347 −16.546 −19.416 1.00 0.00 C
ATOM 4843 C ALA A 308 26.333 −17.265 −18.512 1.00 0.00 C
ATOM 4844 O ALA A 308 25.180 −17.486 −18.898 1.00 0.00 O
ATOM 4845 CB ALA A 308 27.440 −15.049 −19.092 1.00 0.00 C
ATOM 4846 H ALA A 308 25.987 −16.711 −21.012 1.00 0.00 H
ATOM 4847 HA ALA A 308 28.323 −17.006 −19.256 1.00 0.00 H
ATOM 4848 HB1 ALA A 308 27.742 −14.912 −18.052 1.00 0.00 H
ATOM 4849 HB2 ALA A 308 28.178 −14.575 −19.741 1.00 0.00 H
ATOM 4850 HB3 ALA A 308 26.469 −14.576 −19.246 1.00 0.00 H
ATOM 4851 N PHE A 309 26.745 −17.555 −17.280 1.00 0.00 N
ATOM 4852 CA PHE A 309 26.056 −18.434 −16.338 1.00 0.00 C
ATOM 4853 C PHE A 309 25.835 −17.743 −14.986 1.00 0.00 C
ATOM 4854 O PHE A 309 26.763 −17.145 −14.444 1.00 0.00 O
ATOM 4855 CB PHE A 309 26.902 −19.701 −16.195 1.00 0.00 C
ATOM 4856 CG PHE A 309 26.203 −20.909 −15.605 1.00 0.00 C
ATOM 4857 CD1 PHE A 309 25.847 −21.983 −16.442 1.00 0.00 C
ATOM 4858 CD2 PHE A 309 25.968 −20.999 −14.220 1.00 0.00 C
ATOM 4859 CE1 PHE A 309 25.321 −23.166 −15.894 1.00 0.00 C
ATOM 4860 CE2 PHE A 309 25.403 −22.166 −13.677 1.00 0.00 C
ATOM 4861 CZ PHE A 309 25.095 −23.255 −14.510 1.00 0.00 C

ATOM	4862	H	PHE	A	309	27.704	−17.317	−17.048	1.00	0.00	H
ATOM	4863	HA	PHE	A	309	25.096	−18.721	−16.751	1.00	0.00	H
ATOM	4864	HB2	PHE	A	309	27.277	−19.980	−17.178	1.00	0.00	H
ATOM	4865	HB3	PHE	A	309	27.773	−19.463	−15.587	1.00	0.00	H
ATOM	4866	HD1	PHE	A	309	26.003	−21.913	−17.510	1.00	0.00	H
ATOM	4867	HD2	PHE	A	309	26.243	−20.185	−13.565	1.00	0.00	H
ATOM	4868	HE1	PHE	A	309	25.084	−24.003	−16.540	1.00	0.00	H
ATOM	4869	HE2	PHE	A	309	25.230	−22.232	−12.612	1.00	0.00	H
ATOM	4870	HZ	PHE	A	309	24.698	−24.166	−14.085	1.00	0.00	H
ATOM	4871	N	LEU	A	310	24.639	−17.861	−14.404	1.00	0.00	N
ATOM	4872	CA	LEU	A	310	24.291	−17.278	−13.103	1.00	0.00	C
ATOM	4873	C	LEU	A	310	24.958	−18.041	−11.939	1.00	0.00	C
ATOM	4874	O	LEU	A	310	24.559	−19.159	−11.616	1.00	0.00	O
ATOM	4875	CB	LEU	A	310	22.757	−17.266	−13.002	1.00	0.00	C
ATOM	4876	CG	LEU	A	310	22.193	−16.566	−11.754	1.00	0.00	C
ATOM	4877	CD1	LEU	A	310	22.444	−15.063	−11.800	1.00	0.00	C
ATOM	4878	CD2	LEU	A	310	20.685	−16.796	−11.695	1.00	0.00	C
ATOM	4879	H	LEU	A	310	23.916	−18.369	−14.889	1.00	0.00	H
ATOM	4880	HA	LEU	A	310	24.640	−16.247	−13.086	1.00	0.00	H
ATOM	4881	HB2	LEU	A	310	22.412	−18.299	−13.002	1.00	0.00	H
ATOM	4882	HB3	LEU	A	310	22.348	−16.777	−13.888	1.00	0.00	H
ATOM	4883	HG	LEU	A	310	22.643	−16.980	−10.851	1.00	0.00	H
ATOM	4884	HD11	LEU	A	310	21.924	−14.577	−10.978	1.00	0.00	H
ATOM	4885	HD12	LEU	A	310	22.079	−14.657	−12.742	1.00	0.00	H
ATOM	4886	HD13	LEU	A	310	23.505	−14.857	−11.706	1.00	0.00	H
ATOM	4887	HD21	LEU	A	310	20.487	−17.860	−11.590	1.00	0.00	H
ATOM	4888	HD22	LEU	A	310	20.257	−16.264	−10.849	1.00	0.00	H
ATOM	4889	HD23	LEU	A	310	20.213	−16.429	−12.607	1.00	0.00	H
ATOM	4890	N	ILE	A	311	25.961	−17.432	−11.296	1.00	0.00	N
ATOM	4891	CA	ILE	A	311	26.731	−18.035	−10.188	1.00	0.00	C

ATOM	4892	C	ILE	A	311	26.261	−17.607	−8.787	1.00	0.00	C
ATOM	4893	O	ILE	A	311	26.547	−18.307	−7.819	1.00	0.00	O
ATOM	4894	CB	ILE	A	311	28.249	−17.820	−10.372	1.00	0.00	C
ATOM	4895	CG1	ILE	A	311	28.657	−16.331	−10.332	1.00	0.00	C
ATOM	4896	CG2	ILE	A	311	28.746	−18.514	−11.653	1.00	0.00	C
ATOM	4897	CD1	ILE	A	311	30.156	−16.137	−10.082	1.00	0.00	C
ATOM	4898	H	ILE	A	311	26.187	−16.482	−11.564	1.00	0.00	H
ATOM	4899	HA	ILE	A	311	26.570	−19.113	−10.218	1.00	0.00	H
ATOM	4900	HB	ILE	A	311	28.735	−18.320	−9.531	1.00	0.00	H
ATOM	4901	HG12	ILE	A	311	28.127	−15.829	−9.523	1.00	0.00	H
ATOM	4902	HG13	ILE	A	311	28.383	−15.847	−11.270	1.00	0.00	H
ATOM	4903	HG21	ILE	A	311	29.834	−18.521	−11.688	1.00	0.00	H
ATOM	4904	HG22	ILE	A	311	28.396	−19.547	−11.675	1.00	0.00	H
ATOM	4905	HG23	ILE	A	311	28.367	−17.999	−12.536	1.00	0.00	H
ATOM	4906	HD11	ILE	A	311	30.372	−15.074	−9.982	1.00	0.00	H
ATOM	4907	HD12	ILE	A	311	30.440	−16.641	−9.161	1.00	0.00	H
ATOM	4908	HD13	ILE	A	311	30.739	−16.544	−10.905	1.00	0.00	H
ATOM	4909	N	ASP	A	312	25.427	−16.568	−8.688	1.00	0.00	N
ATOM	4910	CA	ASP	A	312	24.532	−16.325	−7.549	1.00	0.00	C
ATOM	4911	C	ASP	A	312	23.263	−15.601	−8.019	1.00	0.00	C
ATOM	4912	O	ASP	A	312	23.348	−14.541	−8.635	1.00	0.00	O
ATOM	4913	CB	ASP	A	312	25.229	−15.516	−6.443	1.00	0.00	C
ATOM	4914	CG	ASP	A	312	24.283	−15.203	−5.274	1.00	0.00	C
ATOM	4915	OD1	ASP	A	312	24.347	−14.050	−4.794	1.00	0.00	O
ATOM	4916	OD2	ASP	A	312	23.290	−15.943	−5.090	1.00	0.00	O
ATOM	4917	H	ASP	A	312	25.279	−16.001	−9.516	1.00	0.00	H
ATOM	4918	HA	ASP	A	312	24.240	−17.282	−7.117	1.00	0.00	H
ATOM	4919	HB2	ASP	A	312	26.080	−16.084	−6.063	1.00	0.00	H
ATOM	4920	HB3	ASP	A	312	25.605	−14.582	−6.866	1.00	0.00	H
ATOM	4921	N	ARG	A	313	22.081	−16.113	−7.654	1.00	0.00	N

ATOM	4922	CA	ARG	A	313	20.793	−15.452	−7.915	1.00	0.00	C
ATOM	4923	C	ARG	A	313	20.472	−14.332	−6.919	1.00	0.00	C
ATOM	4924	O	ARG	A	313	19.787	−13.388	−7.299	1.00	0.00	O
ATOM	4925	CB	ARG	A	313	19.680	−16.514	−7.971	1.00	0.00	C
ATOM	4926	CG	ARG	A	313	18.327	−15.915	−8.402	1.00	0.00	C
ATOM	4927	CD	ARG	A	313	17.270	−16.975	−8.732	1.00	0.00	C
ATOM	4928	NE	ARG	A	313	17.539	−17.627	−10.024	1.00	0.00	N
ATOM	4929	CZ	ARG	A	313	18.023	−18.831	−10.255	1.00	0.00	C
ATOM	4930	NH1	ARG	A	313	18.361	−19.655	−9.309	1.00	0.00	N
ATOM	4931	NH2	ARG	A	313	18.138	−19.257	−11.471	1.00	0.00	N
ATOM	4932	H	ARG	A	313	22.112	−16.879	−6.994	1.00	0.00	H
ATOM	4933	HA	ARG	A	313	20.846	−14.968	−8.892	1.00	0.00	H
ATOM	4934	HB2	ARG	A	313	19.976	−17.280	−8.688	1.00	0.00	H
ATOM	4935	HB3	ARG	A	313	19.570	−16.984	−6.992	1.00	0.00	H
ATOM	4936	HG2	ARG	A	313	18.468	−15.281	−9.277	1.00	0.00	H
ATOM	4937	HG3	ARG	A	313	17.940	−15.296	−7.592	1.00	0.00	H
ATOM	4938	HD2	ARG	A	313	17.206	−17.702	−7.922	1.00	0.00	H
ATOM	4939	HD3	ARG	A	313	16.304	−16.472	−8.810	1.00	0.00	H
ATOM	4940	HE	ARG	A	313	17.274	−17.097	−10.861	1.00	0.00	H
ATOM	4941	HH11	ARG	A	313	18.183	−19.434	−8.351	1.00	0.00	H
ATOM	4942	HH12	ARG	A	313	18.696	−20.572	−9.578	1.00	0.00	H
ATOM	4943	HH21	ARG	A	313	17.728	−18.651	−12.190	1.00	0.00	H
ATOM	4944	HH22	ARG	A	313	18.273	−20.242	−11.664	1.00	0.00	H
ATOM	4945	N	LEU	A	314	20.974	−14.388	−5.683	1.00	0.00	N
ATOM	4946	CA	LEU	A	314	20.622	−13.433	−4.622	1.00	0.00	C
ATOM	4947	C	LEU	A	314	21.212	−12.036	−4.872	1.00	0.00	C
ATOM	4948	O	LEU	A	314	20.497	−11.042	−4.797	1.00	0.00	O
ATOM	4949	CB	LEU	A	314	21.071	−13.986	−3.256	1.00	0.00	C
ATOM	4950	CG	LEU	A	314	20.491	−15.365	−2.884	1.00	0.00	C
ATOM	4951	CD1	LEU	A	314	21.051	−15.811	−1.532	1.00	0.00	C

ATOM	4952	CD2	LEU	A	314	18.963	−15.344	−2.779	1.00	0.00	C
ATOM	4953	H	LEU	A	314	21.657	−15.113	−5.465	1.00	0.00	H
ATOM	4954	HA	LEU	A	314	19.539	−13.308	−4.610	1.00	0.00	H
ATOM	4955	HB2	LEU	A	314	20.789	−13.268	−2.485	1.00	0.00	H
ATOM	4956	HB3	LEU	A	314	22.159	−14.063	−3.254	1.00	0.00	H
ATOM	4957	HG	LEU	A	314	20.778	−16.104	−3.631	1.00	0.00	H
ATOM	4958	HD11	LEU	A	314	22.138	−15.869	−1.597	1.00	0.00	H
ATOM	4959	HD12	LEU	A	314	20.664	−16.798	−1.281	1.00	0.00	H
ATOM	4960	HD13	LEU	A	314	20.770	−15.101	−0.755	1.00	0.00	H
ATOM	4961	HD21	LEU	A	314	18.604	−16.319	−2.449	1.00	0.00	H
ATOM	4962	HD22	LEU	A	314	18.647	−14.583	−2.065	1.00	0.00	H
ATOM	4963	HD23	LEU	A	314	18.526	−15.131	−3.753	1.00	0.00	H
ATOM	4964	N	ASN	A	315	22.463	−11.975	−5.331	1.00	0.00	N
ATOM	4965	CA	ASN	A	315	23.101	−10.761	−5.855	1.00	0.00	C
ATOM	4966	C	ASN	A	315	22.906	−10.579	−7.379	1.00	0.00	C
ATOM	4967	O	ASN	A	315	23.472	−9.645	−7.946	1.00	0.00	O
ATOM	4968	CB	ASN	A	315	24.593	−10.786	−5.468	1.00	0.00	C
ATOM	4969	CG	ASN	A	315	24.816	−10.698	−3.971	1.00	0.00	C
ATOM	4970	OD1	ASN	A	315	25.105	−9.644	−3.434	1.00	0.00	O
ATOM	4971	ND2	ASN	A	315	24.794	−11.802	−3.267	1.00	0.00	N
ATOM	4972	H	ASN	A	315	23.014	−12.832	−5.309	1.00	0.00	H
ATOM	4973	HA	ASN	A	315	22.645	−9.889	−5.380	1.00	0.00	H
ATOM	4974	HB2	ASN	A	315	25.059	−11.695	−5.849	1.00	0.00	H
ATOM	4975	HB3	ASN	A	315	25.095	−9.931	−5.915	1.00	0.00	H
ATOM	4976	HD21	ASN	A	315	24.602	−12.690	−3.739	1.00	0.00	H
ATOM	4977	HD22	ASN	A	315	24.938	−11.733	−2.282	1.00	0.00	H
ATOM	4978	N	TRP	A	316	22.248	−11.541	−8.040	1.00	0.00	N
ATOM	4979	CA	TRP	A	316	22.129	−11.727	−9.497	1.00	0.00	C
ATOM	4980	C	TRP	A	316	23.455	−11.548	−10.268	1.00	0.00	C
ATOM	4981	O	TRP	A	316	23.581	−10.745	−11.195	1.00	0.00	O

ATOM	4982	CB	TRP	A	316	20.889	−11.013	−10.071	1.00	0.00	C
ATOM	4983	CG	TRP	A	316	20.049	−11.914	−10.935	1.00	0.00	C
ATOM	4984	CD1	TRP	A	316	18.989	−12.637	−10.500	1.00	0.00	C
ATOM	4985	CD2	TRP	A	316	20.267	−12.337	−12.320	1.00	0.00	C
ATOM	4986	NE1	TRP	A	316	18.524	−13.452	−11.516	1.00	0.00	N
ATOM	4987	CE2	TRP	A	316	19.303	−13.340	−12.645	1.00	0.00	C
ATOM	4988	CE3	TRP	A	316	21.202	−12.009	−13.328	1.00	0.00	C
ATOM	4989	CZ2	TRP	A	316	19.284	−14.005	−13.878	1.00	0.00	C
ATOM	4990	CZ3	TRP	A	316	21.202	−12.680	−14.568	1.00	0.00	C
ATOM	4991	CH2	TRP	A	316	20.251	−13.681	−14.844	1.00	0.00	C
ATOM	4992	H	TRP	A	316	21.792	−12.237	−7.469	1.00	0.00	H
ATOM	4993	HA	TRP	A	316	21.909	−12.786	−9.612	1.00	0.00	H
ATOM	4994	HB2	TRP	A	316	20.263	−10.664	−9.248	1.00	0.00	H
ATOM	4995	HB3	TRP	A	316	21.187	−10.133	−10.642	1.00	0.00	H
ATOM	4996	HD1	TRP	A	316	18.606	−12.621	−9.485	1.00	0.00	H
ATOM	4997	HE3	TRP	A	316	21.938	−11.242	−13.128	1.00	0.00	H
ATOM	4998	HZ2	TRP	A	316	18.562	−14.789	−14.058	1.00	0.00	H
ATOM	4999	HZ3	TRP	A	316	21.949	−12.434	−15.309	1.00	0.00	H
ATOM	5000	HH2	TRP	A	316	20.274	−14.211	−15.786	1.00	0.00	H
ATOM	5001	HE1	TRP	A	316	17.759	−14.133	−11.416	1.00	0.00	H
ATOM	5002	N	VAL	A	317	24.496	−12.241	−9.793	1.00	0.00	N
ATOM	5003	CA	VAL	A	317	25.847	−12.242	−10.370	1.00	0.00	C
ATOM	5004	C	VAL	A	317	26.014	−13.417	−11.335	1.00	0.00	C
ATOM	5005	O	VAL	A	317	25.909	−14.580	−10.944	1.00	0.00	O
ATOM	5006	CB	VAL	A	317	26.925	−12.256	−9.269	1.00	0.00	C
ATOM	5007	CG1	VAL	A	317	28.345	−12.373	−9.836	1.00	0.00	C
ATOM	5008	CG2	VAL	A	317	26.877	−10.958	−8.453	1.00	0.00	C
ATOM	5009	H	VAL	A	317	24.279	−12.953	−9.103	1.00	0.00	H
ATOM	5010	HA	VAL	A	317	25.978	−11.323	−10.937	1.00	0.00	H
ATOM	5011	HB	VAL	A	317	26.748	−13.100	−8.599	1.00	0.00	H

ATOM 5012 HG11 VAL A 317 29.073 −12.322 −9.027 1.00 0.00 H
ATOM 5013 HG12 VAL A 317 28.531 −11.568 −10.547 1.00 0.00 H
ATOM 5014 HG13 VAL A 317 28.471 −13.329 −10.343 1.00 0.00 H
ATOM 5015 HG21 VAL A 317 25.888 −10.835 −8.020 1.00 0.00 H
ATOM 5016 HG22 VAL A 317 27.609 −11.000 −7.646 1.00 0.00 H
ATOM 5017 HG23 VAL A 317 27.087 −10.108 −9.100 1.00 0.00 H
ATOM 5018 N SER A 318 26.391 −13.112 −12.574 1.00 0.00 N
ATOM 5019 CA SER A 318 26.748 −14.075 −13.622 1.00 0.00 C
ATOM 5020 C SER A 318 28.250 −14.068 −13.936 1.00 0.00 C
ATOM 5021 O SER A 318 28.964 −13.116 −13.618 1.00 0.00 O
ATOM 5022 CB SER A 318 25.935 −13.795 −14.889 1.00 0.00 C
ATOM 5023 OG SER A 318 24.554 −13.974 −14.634 1.00 0.00 O
ATOM 5024 H SER A 318 26.569 −12.133 −12.774 1.00 0.00 H
ATOM 5025 HA SER A 318 26.495 −15.074 −13.282 1.00 0.00 H
ATOM 5026 HB2 SER A 318 26.115 −12.772 −15.216 1.00 0.00 H
ATOM 5027 HB3 SER A 318 26.244 −14.477 −15.683 1.00 0.00 H
ATOM 5028 HG SER A 318 24.279 −13.346 −13.956 1.00 0.00 H
ATOM 5029 N ALA A 319 28.724 −15.114 −14.614 1.00 0.00 N
ATOM 5030 CA ALA A 319 30.107 −15.283 −15.054 1.00 0.00 C
ATOM 5031 C ALA A 319 30.175 −15.861 −16.477 1.00 0.00 C
ATOM 5032 O ALA A 319 29.405 −16.760 −16.813 1.00 0.00 O
ATOM 5033 CB ALA A 319 30.824 −16.187 −14.049 1.00 0.00 C
ATOM 5034 H ALA A 319 28.091 −15.888 −14.793 1.00 0.00 H
ATOM 5035 HA ALA A 319 30.595 −14.310 −15.059 1.00 0.00 H
ATOM 5036 HB1 ALA A 319 31.875 −16.269 −14.323 1.00 0.00 H
ATOM 5037 HB2 ALA A 319 30.375 −17.181 −14.055 1.00 0.00 H
ATOM 5038 HB3 ALA A 319 30.744 −15.758 −13.053 1.00 0.00 H
ATOM 5039 N GLY A 320 31.096 −15.380 −17.312 1.00 0.00 N
ATOM 5040 CA GLY A 320 31.263 −15.879 −18.680 1.00 0.00 C
ATOM 5041 C GLY A 320 32.318 −15.132 −19.488 1.00 0.00 C

ATOM 5042 O GLY A 320 32.798 −14.077 −19.073 1.00 0.00 O
ATOM 5043 H GLY A 320 31.707 −14.629 −17.000 1.00 0.00 H
ATOM 5044 HA2 GLY A 320 31.556 −16.925 −18.641 1.00 0.00 H
ATOM 5045 HA3 GLY A 320 30.318 −15.813 −19.218 1.00 0.00 H
ATOM 5046 N VAL A 321 32.692 −15.693 −20.637 1.00 0.00 N
ATOM 5047 CA VAL A 321 33.604 −15.043 −21.593 1.00 0.00 C
ATOM 5048 C VAL A 321 32.809 −14.192 −22.578 1.00 0.00 C
ATOM 5049 O VAL A 321 31.868 −14.695 −23.201 1.00 0.00 O
ATOM 5050 CB VAL A 321 34.511 −16.048 −22.328 1.00 0.00 C
ATOM 5051 CG1 VAL A 321 35.590 −15.364 −23.183 1.00 0.00 C
ATOM 5052 CG2 VAL A 321 35.238 −16.966 −21.348 1.00 0.00 C
ATOM 5053 H VAL A 321 32.219 −16.537 −20.930 1.00 0.00 H
ATOM 5054 HA VAL A 321 34.255 −14.381 −21.035 1.00 0.00 H
ATOM 5055 HB VAL A 321 33.895 −16.673 −22.967 1.00 0.00 H
ATOM 5056 HG11 VAL A 321 36.272 −14.798 −22.549 1.00 0.00 H
ATOM 5057 HG12 VAL A 321 36.163 −16.114 −23.729 1.00 0.00 H
ATOM 5058 HG13 VAL A 321 35.148 −14.684 −23.911 1.00 0.00 H
ATOM 5059 HG21 VAL A 321 34.521 −17.512 −20.740 1.00 0.00 H
ATOM 5060 HG22 VAL A 321 35.879 −16.369 −20.701 1.00 0.00 H
ATOM 5061 HG23 VAL A 321 35.837 −17.688 −21.898 1.00 0.00 H
ATOM 5062 N TYR A 322 33.285 −12.973 −22.831 1.00 0.00 N
ATOM 5063 CA TYR A 322 32.801 −12.090 −23.892 1.00 0.00 C
ATOM 5064 C TYR A 322 33.961 −11.477 −24.684 1.00 0.00 C
ATOM 5065 O TYR A 322 35.053 −11.266 −24.156 1.00 0.00 O
ATOM 5066 CB TYR A 322 31.830 −11.023 −23.343 1.00 0.00 C
ATOM 5067 CG TYR A 322 32.426 −9.796 −22.654 1.00 0.00 C
ATOM 5068 CD1 TYR A 322 32.019 −8.500 −23.043 1.00 0.00 C
ATOM 5069 CD2 TYR A 322 33.328 −9.940 −21.579 1.00 0.00 C
ATOM 5070 CE1 TYR A 322 32.497 −7.366 −22.349 1.00 0.00 C
ATOM 5071 CE2 TYR A 322 33.848 −8.809 −20.924 1.00 0.00 C

ATOM	5072	CZ	TYR	A	322	33.426	−7.519	−21.299	1.00	0.00	C
ATOM	5073	OH	TYR	A	322	33.890	−6.429	−20.637	1.00	0.00	O
ATOM	5074	H	TYR	A	322	34.122	−12.672	−22.337	1.00	0.00	H
ATOM	5075	HA	TYR	A	322	32.235	−12.703	−24.590	1.00	0.00	H
ATOM	5076	HB2	TYR	A	322	31.139	−11.504	−22.648	1.00	0.00	H
ATOM	5077	HB3	TYR	A	322	31.233	−10.676	−24.187	1.00	0.00	H
ATOM	5078	HD1	TYR	A	322	31.322	−8.380	−23.861	1.00	0.00	H
ATOM	5079	HD2	TYR	A	322	33.638	−10.920	−21.256	1.00	0.00	H
ATOM	5080	HE1	TYR	A	322	32.151	−6.374	−22.587	1.00	0.00	H
ATOM	5081	HE2	TYR	A	322	34.568	−8.927	−20.131	1.00	0.00	H
ATOM	5082	HH	TYR	A	322	34.376	−5.834	−21.232	1.00	0.00	H
ATOM	5083	N	LEU	A	323	33.725	−11.244	−25.973	1.00	0.00	N
ATOM	5084	CA	LEU	A	323	34.641	−10.527	−26.856	1.00	0.00	C
ATOM	5085	C	LEU	A	323	34.562	−9.017	−26.600	1.00	0.00	C
ATOM	5086	O	LEU	A	323	33.475	−8.427	−26.584	1.00	0.00	O
ATOM	5087	CB	LEU	A	323	34.310	−10.854	−28.318	1.00	0.00	C
ATOM	5088	CG	LEU	A	323	34.385	−12.346	−28.676	1.00	0.00	C
ATOM	5089	CD1	LEU	A	323	33.882	−12.540	−30.103	1.00	0.00	C
ATOM	5090	CD2	LEU	A	323	35.795	−12.909	−28.553	1.00	0.00	C
ATOM	5091	H	LEU	A	323	32.838	−11.545	−26.359	1.00	0.00	H
ATOM	5092	HA	LEU	A	323	35.662	−10.849	−26.651	1.00	0.00	H
ATOM	5093	HB2	LEU	A	323	34.987	−10.298	−28.969	1.00	0.00	H
ATOM	5094	HB3	LEU	A	323	33.296	−10.515	−28.509	1.00	0.00	H
ATOM	5095	HG	LEU	A	323	33.739	−12.921	−28.019	1.00	0.00	H
ATOM	5096	HD21	LEU	A	323	35.778	−13.958	−28.836	1.00	0.00	H
ATOM	5097	HD22	LEU	A	323	36.472	−12.364	−29.212	1.00	0.00	H
ATOM	5098	HD23	LEU	A	323	36.148	−12.830	−27.527	1.00	0.00	H
ATOM	5099	HD11	LEU	A	323	33.888	−13.599	−30.349	1.00	0.00	H
ATOM	5100	HD12	LEU	A	323	34.535	−12.010	−30.798	1.00	0.00	H
ATOM	5101	HD13	LEU	A	323	32.871	−12.153	−30.190	1.00	0.00	H

ATOM 5102 N ASN A 324 35.718 −8.384 −26.424 1.00 0.00 N
ATOM 5103 CA ASN A 324 35.811 −6.960 −26.108 1.00 0.00 C
ATOM 5104 C ASN A 324 35.888 −6.051 −27.352 1.00 0.00 C
ATOM 5105 O ASN A 324 35.729 −4.837 −27.229 1.00 0.00 O
ATOM 5106 CB ASN A 324 37.016 −6.773 −25.185 1.00 0.00 C
ATOM 5107 CG ASN A 324 37.028 −5.506 −24.360 1.00 0.00 C
ATOM 5108 OD1 ASN A 324 36.033 −4.807 −24.179 1.00 0.00 O
ATOM 5109 ND2 ASN A 324 38.176 −5.208 −23.803 1.00 0.00 N
ATOM 5110 H ASN A 324 36.569 −8.943 −26.393 1.00 0.00 H
ATOM 5111 HA ASN A 324 34.917 −6.684 −25.558 1.00 0.00 H
ATOM 5112 HB2 ASN A 324 37.898 −6.745 −25.810 1.00 0.00 H
ATOM 5113 HB3 ASN A 324 37.100 −7.616 −24.496 1.00 0.00 H
ATOM 5114 HD21 ASN A 324 38.968 −5.823 −23.976 1.00 0.00 H
ATOM 5115 HD22 ASN A 324 38.264 −4.352 −23.294 1.00 0.00 H
ATOM 5116 N SER A 325 36.090 −6.606 −28.549 1.00 0.00 N
ATOM 5117 CA SER A 325 36.057 −5.843 −29.804 1.00 0.00 C
ATOM 5118 C SER A 325 34.652 −5.349 −30.169 1.00 0.00 C
ATOM 5119 O SER A 325 33.654 −6.021 −29.922 1.00 0.00 O
ATOM 5120 CB SER A 325 36.611 −6.699 −30.929 1.00 0.00 C
ATOM 5121 OG SER A 325 36.541 −6.004 −32.157 1.00 0.00 O
ATOM 5122 H SER A 325 36.261 −7.605 −28.599 1.00 0.00 H
ATOM 5123 HA SER A 325 36.709 −4.976 −29.696 1.00 0.00 H
ATOM 5124 HB2 SER A 325 36.047 −7.629 −30.998 1.00 0.00 H
ATOM 5125 HB3 SER A 325 37.654 −6.936 −30.708 1.00 0.00 H
ATOM 5126 HG SER A 325 37.182 −6.512 −32.725 1.00 0.00 H
ATOM 5127 N ASN A 326 34.546 −4.165 −30.784 1.00 0.00 N
ATOM 5128 CA ASN A 326 33.255 −3.555 −31.132 1.00 0.00 C
ATOM 5129 C ASN A 326 32.624 −4.069 −32.442 1.00 0.00 C
ATOM 5130 O ASN A 326 31.402 −3.958 −32.585 1.00 0.00 O
ATOM 5131 CB ASN A 326 33.392 −2.019 −31.127 1.00 0.00 C

ATOM 5132 CG ASN A 326 33.468 −1.402 −29.735 1.00 0.00 C
ATOM 5133 OD1 ASN A 326 33.467 −2.061 −28.707 1.00 0.00 O
ATOM 5134 ND2 ASN A 326 33.310 −0.103 −29.649 1.00 0.00 N
ATOM 5135 H ASN A 326 35.405 −3.686 −31.009 1.00 0.00 H
ATOM 5136 HA ASN A 326 32.536 −3.818 −30.362 1.00 0.00 H
ATOM 5137 HB2 ASN A 326 34.272 −1.722 −31.697 1.00 0.00 H
ATOM 5138 HB3 ASN A 326 32.517 −1.593 −31.619 1.00 0.00 H
ATOM 5139 HD21 ASN A 326 33.211 0.461 −30.473 1.00 0.00 H
ATOM 5140 HD22 ASN A 326 33.266 0.283 −28.722 1.00 0.00 H
ATOM 5141 N GLN A 327 33.425 −4.572 −33.391 1.00 0.00 N
ATOM 5142 CA GLN A 327 33.011 −4.841 −34.786 1.00 0.00 C
ATOM 5143 C GLN A 327 33.590 −6.134 −35.398 1.00 0.00 C
ATOM 5144 O GLN A 327 33.304 −6.448 −36.556 1.00 0.00 O
ATOM 5145 CB GLN A 327 33.422 −3.654 −35.677 1.00 0.00 C
ATOM 5146 CG GLN A 327 32.649 −2.357 −35.397 1.00 0.00 C
ATOM 5147 CD GLN A 327 32.961 −1.259 −36.413 1.00 0.00 C
ATOM 5148 OE1 GLN A 327 32.090 −0.560 −36.898 1.00 0.00 O
ATOM 5149 NE2 GLN A 327 34.198 −1.089 −36.829 1.00 0.00 N
ATOM 5150 H GLN A 327 34.408 −4.677 −33.169 1.00 0.00 H
ATOM 5151 HA GLN A 327 31.929 −4.939 −34.826 1.00 0.00 H
ATOM 5152 HB2 GLN A 327 33.239 −3.917 −36.720 1.00 0.00 H
ATOM 5153 HB3 GLN A 327 34.494 −3.481 −35.557 1.00 0.00 H
ATOM 5154 HG2 GLN A 327 31.580 −2.569 −35.432 1.00 0.00 H
ATOM 5155 HG3 GLN A 327 32.905 −1.988 −34.406 1.00 0.00 H
ATOM 5156 HE21 GLN A 327 34.936 −1.695 −36.514 1.00 0.00 H
ATOM 5157 HE22 GLN A 327 34.333 −0.395 −37.541 1.00 0.00 H
ATOM 5158 N THR A 328 34.497 −6.803 −34.691 1.00 0.00 N
ATOM 5159 CA THR A 328 35.325 −7.900 −35.209 1.00 0.00 C
ATOM 5160 C THR A 328 35.672 −8.890 −34.084 1.00 0.00 C
ATOM 5161 O THR A 328 35.068 −8.845 −33.011 1.00 0.00 O

ATOM 5162 CB THR A 328 36.577 −7.358 −35.941 1.00 0.00 C
ATOM 5163 OG1 THR A 328 37.126 −6.235 −35.297 1.00 0.00 O
ATOM 5164 CG2 THR A 328 36.316 −6.944 −37.387 1.00 0.00 C
ATOM 5165 H THR A 328 34.739 −6.488 −33.763 1.00 0.00 H
ATOM 5166 HA THR A 328 34.744 −8.466 −35.935 1.00 0.00 H
ATOM 5167 HB THR A 328 37.319 −8.150 −35.984 1.00 0.00 H
ATOM 5168 HG1 THR A 328 37.874 −6.609 −34.779 1.00 0.00 H
ATOM 5169 HG21 THR A 328 37.270 −6.697 −37.854 1.00 0.00 H
ATOM 5170 HG22 THR A 328 35.856 −7.768 −37.930 1.00 0.00 H
ATOM 5171 HG23 THR A 328 35.672 −6.067 −37.421 1.00 0.00 H
ATOM 5172 N ALA A 329 36.316 −9.995 −34.461 1.00 0.00 N
ATOM 5173 CA ALA A 329 36.389 −11.229 −33.681 1.00 0.00 C
ATOM 5174 C ALA A 329 37.645 −11.292 −32.785 1.00 0.00 C
ATOM 5175 O ALA A 329 38.484 −12.167 −32.979 1.00 0.00 O
ATOM 5176 CB ALA A 329 36.272 −12.399 −34.675 1.00 0.00 C
ATOM 5177 H ALA A 329 36.842 −9.969 −35.320 1.00 0.00 H
ATOM 5178 HA ALA A 329 35.531 −11.283 −33.010 1.00 0.00 H
ATOM 5179 HB1 ALA A 329 35.338 −12.319 −35.232 1.00 0.00 H
ATOM 5180 HB2 ALA A 329 36.278 −13.345 −34.137 1.00 0.00 H
ATOM 5181 HB3 ALA A 329 37.115 −12.386 −35.367 1.00 0.00 H
ATOM 5182 N GLU A 330 37.824 −10.332 −31.869 1.00 0.00 N
ATOM 5183 CA GLU A 330 39.035 −10.212 −31.032 1.00 0.00 C
ATOM 5184 C GLU A 330 38.749 −10.050 −29.523 1.00 0.00 C
ATOM 5185 O GLU A 330 37.688 −9.559 −29.119 1.00 0.00 O
ATOM 5186 CB GLU A 330 39.955 −9.064 −31.507 1.00 0.00 C
ATOM 5187 CG GLU A 330 40.225 −8.983 −33.022 1.00 0.00 C
ATOM 5188 CD GLU A 330 39.281 −8.040 −33.784 1.00 0.00 C
ATOM 5189 OE1 GLU A 330 39.478 −7.827 −34.997 1.00 0.00 O
ATOM 5190 OE2 GLU A 330 38.243 −7.589 −33.244 1.00 0.00 O
ATOM 5191 H GLU A 330 37.153 −9.578 −31.830 1.00 0.00 H

ATOM	5192	HA	GLU	A	330	39.615	−11.128	−31.137	1.00	0.00	H
ATOM	5193	HB2	GLU	A	330	39.569	−8.110	−31.150	1.00	0.00	H
ATOM	5194	HB3	GLU	A	330	40.919	−9.206	−31.017	1.00	0.00	H
ATOM	5195	HG2	GLU	A	330	41.243	−8.610	−33.155	1.00	0.00	H
ATOM	5196	HG3	GLU	A	330	40.188	−9.983	−33.457	1.00	0.00	H
ATOM	5197	N	ASN	A	331	39.809	−10.259	−28.725	1.00	0.00	N
ATOM	5198	CA	ASN	A	331	39.940	−9.916	−27.299	1.00	0.00	C
ATOM	5199	C	ASN	A	331	38.999	−10.724	−26.363	1.00	0.00	C
ATOM	5200	O	ASN	A	331	37.965	−10.212	−25.917	1.00	0.00	O
ATOM	5201	CB	ASN	A	331	39.849	−8.384	−27.158	1.00	0.00	C
ATOM	5202	CG	ASN	A	331	40.426	−7.858	−25.852	1.00	0.00	C
ATOM	5203	OD1	ASN	A	331	39.807	−7.889	−24.801	1.00	0.00	O
ATOM	5204	ND2	ASN	A	331	41.493	−7.110	−25.945	1.00	0.00	N
ATOM	5205	H	ASN	A	331	40.633	−10.655	−29.168	1.00	0.00	H
ATOM	5206	HA	ASN	A	331	40.961	−10.178	−27.013	1.00	0.00	H
ATOM	5207	HB2	ASN	A	331	38.812	−8.078	−27.247	1.00	0.00	H
ATOM	5208	HB3	ASN	A	331	40.389	−7.924	−27.985	1.00	0.00	H
ATOM	5209	HD21	ASN	A	331	42.014	−7.151	−26.815	1.00	0.00	H
ATOM	5210	HD22	ASN	A	331	42.020	−6.945	−25.080	1.00	0.00	H
ATOM	5211	N	PRO	A	332	39.288	−12.022	−26.120	1.00	0.00	N
ATOM	5212	CA	PRO	A	332	38.471	−12.911	−25.289	1.00	0.00	C
ATOM	5213	C	PRO	A	332	38.709	−12.694	−23.781	1.00	0.00	C
ATOM	5214	O	PRO	A	332	39.639	−13.249	−23.188	1.00	0.00	O
ATOM	5215	CB	PRO	A	332	38.855	−14.324	−25.747	1.00	0.00	C
ATOM	5216	CG	PRO	A	332	40.321	−14.183	−26.163	1.00	0.00	C
ATOM	5217	CD	PRO	A	332	40.397	−12.762	−26.717	1.00	0.00	C
ATOM	5218	HA	PRO	A	332	37.413	−12.747	−25.500	1.00	0.00	H
ATOM	5219	HB2	PRO	A	332	38.724	−15.068	−24.960	1.00	0.00	H
ATOM	5220	HB3	PRO	A	332	38.257	−14.593	−26.618	1.00	0.00	H
ATOM	5221	HG2	PRO	A	332	40.597	−14.913	−26.922	1.00	0.00	H

ATOM	5222	HG3	PRO	A	332	40.971	−14.272	−25.292	1.00	0.00	H
ATOM	5223	HD2	PRO	A	332	40.278	−12.795	−27.801	1.00	0.00	H
ATOM	5224	HD3	PRO	A	332	41.359	−12.316	−26.461	1.00	0.00	H
ATOM	5225	N	VAL	A	333	37.755	−12.043	−23.108	1.00	0.00	N
ATOM	5226	CA	VAL	A	333	37.818	−11.748	−21.663	1.00	0.00	C
ATOM	5227	C	VAL	A	333	36.797	−12.574	−20.884	1.00	0.00	C
ATOM	5228	O	VAL	A	333	35.596	−12.459	−21.122	1.00	0.00	O
ATOM	5229	CB	VAL	A	333	37.630	−10.237	−21.418	1.00	0.00	C
ATOM	5230	CG1	VAL	A	333	37.602	−9.885	−19.926	1.00	0.00	C
ATOM	5231	CG2	VAL	A	333	38.772	−9.436	−22.052	1.00	0.00	C
ATOM	5232	H	VAL	A	333	36.989	−11.634	−23.637	1.00	0.00	H
ATOM	5233	HA	VAL	A	333	38.801	−12.012	−21.284	1.00	0.00	H
ATOM	5234	HB	VAL	A	333	36.688	−9.913	−21.863	1.00	0.00	H
ATOM	5235	HG11	VAL	A	333	37.547	−8.805	−19.817	1.00	0.00	H
ATOM	5236	HG12	VAL	A	333	38.505	−10.253	−19.441	1.00	0.00	H
ATOM	5237	HG13	VAL	A	333	36.724	−10.320	−19.451	1.00	0.00	H
ATOM	5238	HG21	VAL	A	333	39.733	−9.733	−21.631	1.00	0.00	H
ATOM	5239	HG22	VAL	A	333	38.794	−9.608	−23.130	1.00	0.00	H
ATOM	5240	HG23	VAL	A	333	38.623	−8.369	−21.894	1.00	0.00	H
ATOM	5241	N	PHE	A	334	37.233	−13.305	−19.852	1.00	0.00	N
ATOM	5242	CA	PHE	A	334	36.331	−13.835	−18.824	1.00	0.00	C
ATOM	5243	C	PHE	A	334	35.964	−12.719	−17.835	1.00	0.00	C
ATOM	5244	O	PHE	A	334	36.843	−12.129	−17.201	1.00	0.00	O
ATOM	5245	CB	PHE	A	334	36.959	−15.051	−18.122	1.00	0.00	C
ATOM	5246	CG	PHE	A	334	35.997	−15.842	−17.241	1.00	0.00	C
ATOM	5247	CD1	PHE	A	334	35.513	−17.097	−17.663	1.00	0.00	C
ATOM	5248	CD2	PHE	A	334	35.586	−15.337	−15.992	1.00	0.00	C
ATOM	5249	CE1	PHE	A	334	34.633	−17.833	−16.848	1.00	0.00	C
ATOM	5250	CE2	PHE	A	334	34.703	−16.071	−15.179	1.00	0.00	C
ATOM	5251	CZ	PHE	A	334	34.227	−17.322	−15.606	1.00	0.00	C

ATOM 5252 H PHE A 334 38.221 −13.255 −19.622 1.00 0.00 H
ATOM 5253 HA PHE A 334 35.414 −14.176 −19.302 1.00 0.00 H
ATOM 5254 HB2 PHE A 334 37.801 −14.718 −17.518 1.00 0.00 H
ATOM 5255 HB3 PHE A 334 37.358 −15.720 −18.884 1.00 0.00 H
ATOM 5256 HD1 PHE A 334 35.822 −17.512 −18.611 1.00 0.00 H
ATOM 5257 HD2 PHE A 334 35.939 −14.375 −15.658 1.00 0.00 H
ATOM 5258 HE1 PHE A 334 34.270 −18.795 −17.179 1.00 0.00 H
ATOM 5259 HE2 PHE A 334 34.388 −15.673 −14.227 1.00 0.00 H
ATOM 5260 HZ PHE A 334 33.549 −17.891 −14.986 1.00 0.00 H
ATOM 5261 N ALA A 335 34.671 −12.507 −17.602 1.00 0.00 N
ATOM 5262 CA ALA A 335 34.141 −11.521 −16.666 1.00 0.00 C
ATOM 5263 C ALA A 335 33.212 −12.164 −15.628 1.00 0.00 C
ATOM 5264 O ALA A 335 32.470 −13.099 −15.933 1.00 0.00 O
ATOM 5265 CB ALA A 335 33.431 −10.421 −17.464 1.00 0.00 C
ATOM 5266 H ALA A 335 33.994 −13.052 −18.129 1.00 0.00 H
ATOM 5267 HA ALA A 335 34.962 −11.057 −16.120 1.00 0.00 H
ATOM 5268 HB1 ALA A 335 33.030 −9.667 −16.787 1.00 0.00 H
ATOM 5269 HB2 ALA A 335 34.141 −9.946 −18.139 1.00 0.00 H
ATOM 5270 HB3 ALA A 335 32.615 −10.850 −18.047 1.00 0.00 H
ATOM 5271 N VAL A 336 33.139 −11.547 −14.447 1.00 0.00 N
ATOM 5272 CA VAL A 336 32.083 −11.777 −13.449 1.00 0.00 C
ATOM 5273 C VAL A 336 31.351 −10.462 −13.197 1.00 0.00 C
ATOM 5274 O VAL A 336 31.989 −9.424 −13.007 1.00 0.00 O
ATOM 5275 CB VAL A 336 32.650 −12.377 −12.151 1.00 0.00 C
ATOM 5276 CG1 VAL A 336 31.546 −12.621 −11.119 1.00 0.00 C
ATOM 5277 CG2 VAL A 336 33.333 −13.723 −12.418 1.00 0.00 C
ATOM 5278 H VAL A 336 33.774 −10.772 −14.276 1.00 0.00 H
ATOM 5279 HA VAL A 336 31.361 −12.486 −13.849 1.00 0.00 H
ATOM 5280 HB VAL A 336 33.378 −11.687 −11.727 1.00 0.00 H
ATOM 5281 HG11 VAL A 336 31.099 −11.677 −10.822 1.00 0.00 H

ATOM 5282 HG12 VAL A 336 30.778 −13.267 −11.544 1.00 0.00 H
ATOM 5283 HG13 VAL A 336 31.963 −13.094 −10.231 1.00 0.00 H
ATOM 5284 HG21 VAL A 336 32.638 −14.391 −12.921 1.00 0.00 H
ATOM 5285 HG22 VAL A 336 34.201 −13.574 −13.056 1.00 0.00 H
ATOM 5286 HG23 VAL A 336 33.649 −14.177 −11.482 1.00 0.00 H
ATOM 5287 N PHE A 337 30.023 −10.468 −13.299 1.00 0.00 N
ATOM 5288 CA PHE A 337 29.228 −9.250 −13.457 1.00 0.00 C
ATOM 5289 C PHE A 337 27.805 −9.372 −12.899 1.00 0.00 C
ATOM 5290 O PHE A 337 27.212 −10.445 −12.858 1.00 0.00 O
ATOM 5291 CB PHE A 337 29.207 −8.850 −14.944 1.00 0.00 C
ATOM 5292 CG PHE A 337 28.480 −9.814 −15.865 1.00 0.00 C
ATOM 5293 CD1 PHE A 337 27.113 −9.623 −16.147 1.00 0.00 C
ATOM 5294 CD2 PHE A 337 29.167 −10.898 −16.447 1.00 0.00 C
ATOM 5295 CE1 PHE A 337 26.438 −10.507 −17.008 1.00 0.00 C
ATOM 5296 CE2 PHE A 337 28.491 −11.785 −17.303 1.00 0.00 C
ATOM 5297 CZ PHE A 337 27.127 −11.588 −17.585 1.00 0.00 C
ATOM 5298 H PHE A 337 29.551 −11.355 −13.465 1.00 0.00 H
ATOM 5299 HA PHE A 337 29.712 −8.448 −12.901 1.00 0.00 H
ATOM 5300 HB2 PHE A 337 28.742 −7.866 −15.033 1.00 0.00 H
ATOM 5301 HB3 PHE A 337 30.234 −8.747 −15.295 1.00 0.00 H
ATOM 5302 HD1 PHE A 337 26.580 −8.800 −15.693 1.00 0.00 H
ATOM 5303 HD2 PHE A 337 30.216 −11.055 −16.238 1.00 0.00 H
ATOM 5304 HE1 PHE A 337 25.390 −10.360 −17.224 1.00 0.00 H
ATOM 5305 HE2 PHE A 337 29.025 −12.613 −17.751 1.00 0.00 H
ATOM 5306 HZ PHE A 337 26.608 −12.269 −18.244 1.00 0.00 H
ATOM 5307 N LYS A 338 27.240 −8.220 −12.553 1.00 0.00 N
ATOM 5308 CA LYS A 338 25.839 −7.947 −12.214 1.00 0.00 C
ATOM 5309 C LYS A 338 25.263 −7.008 −13.290 1.00 0.00 C
ATOM 5310 O LYS A 338 26.030 −6.417 −14.050 1.00 0.00 O
ATOM 5311 CB LYS A 338 25.860 −7.346 −10.797 1.00 0.00 C

ATOM	5312	CG	LYS	A	338	24.499	−7.194	−10.108	1.00	0.00	C
ATOM	5313	CD	LYS	A	338	24.577	−6.475	−8.746	1.00	0.00	C
ATOM	5314	CE	LYS	A	338	25.443	−7.150	−7.667	1.00	0.00	C
ATOM	5315	NZ	LYS	A	338	26.884	−6.802	−7.778	1.00	0.00	N
ATOM	5316	H	LYS	A	338	27.814	−7.392	−12.688	1.00	0.00	H
ATOM	5317	HA	LYS	A	338	25.260	−8.876	−12.213	1.00	0.00	H
ATOM	5318	HB2	LYS	A	338	26.470	−8.002	−10.176	1.00	0.00	H
ATOM	5319	HB3	LYS	A	338	26.344	−6.372	−10.838	1.00	0.00	H
ATOM	5320	HG2	LYS	A	338	24.059	−8.182	−9.978	1.00	0.00	H
ATOM	5321	HG3	LYS	A	338	23.833	−6.610	−10.740	1.00	0.00	H
ATOM	5322	HD2	LYS	A	338	23.558	−6.428	−8.358	1.00	0.00	H
ATOM	5323	HD3	LYS	A	338	24.917	−5.448	−8.897	1.00	0.00	H
ATOM	5324	HE2	LYS	A	338	25.075	−6.818	−6.693	1.00	0.00	H
ATOM	5325	HE3	LYS	A	338	25.297	−8.233	−7.721	1.00	0.00	H
ATOM	5326	HZ1	LYS	A	338	27.421	−7.108	−6.980	1.00	0.00	H
ATOM	5327	HZ2	LYS	A	338	27.036	−5.788	−7.866	1.00	0.00	H
ATOM	5328	HZ3	LYS	A	338	27.294	−7.172	−8.620	1.00	0.00	H
ATOM	5329	N	ASP	A	339	23.944	−6.818	−13.330	1.00	0.00	N
ATOM	5330	CA	ASP	A	339	23.222	−6.048	−14.366	1.00	0.00	C
ATOM	5331	C	ASP	A	339	23.896	−4.734	−14.809	1.00	0.00	C
ATOM	5332	O	ASP	A	339	24.173	−4.557	−15.994	1.00	0.00	O
ATOM	5333	CB	ASP	A	339	21.796	−5.737	−13.875	1.00	0.00	C
ATOM	5334	CG	ASP	A	339	20.981	−6.974	−13.498	1.00	0.00	C
ATOM	5335	OD1	ASP	A	339	21.248	−8.068	−14.038	1.00	0.00	O
ATOM	5336	OD2	ASP	A	339	20.002	−6.837	−12.738	1.00	0.00	O
ATOM	5337	H	ASP	A	339	23.380	−7.414	−12.740	1.00	0.00	H
ATOM	5338	HA	ASP	A	339	23.130	−6.675	−15.251	1.00	0.00	H
ATOM	5339	HB2	ASP	A	339	21.857	−5.080	−13.006	1.00	0.00	H
ATOM	5340	HB3	ASP	A	339	21.264	−5.205	−14.666	1.00	0.00	H
ATOM	5341	N	ASN	A	340	24.298	−3.885	−13.860	1.00	0.00	N

ATOM 5342 CA ASN A 340 24.885 −2.570 −14.147 1.00 0.00 C
ATOM 5343 C ASN A 340 26.396 −2.501 −13.831 1.00 0.00 C
ATOM 5344 O ASN A 340 26.945 −1.414 −13.664 1.00 0.00 O
ATOM 5345 CB ASN A 340 24.076 −1.470 −13.425 1.00 0.00 C
ATOM 5346 CG ASN A 340 22.606 −1.368 −13.813 1.00 0.00 C
ATOM 5347 OD1 ASN A 340 21.953 −2.294 −14.255 1.00 0.00 O
ATOM 5348 ND2 ASN A 340 21.981 −0.257 −13.505 1.00 0.00 N
ATOM 5349 H ASN A 340 24.028 −4.088 −12.912 1.00 0.00 H
ATOM 5350 HA ASN A 340 24.801 −2.379 −15.216 1.00 0.00 H
ATOM 5351 HB2 ASN A 340 24.140 −1.618 −12.349 1.00 0.00 H
ATOM 5352 HB3 ASN A 340 24.536 −0.510 −13.658 1.00 0.00 H
ATOM 5353 HD21 ASN A 340 22.481 0.518 −13.116 1.00 0.00 H
ATOM 5354 HD22 ASN A 340 21.007 −0.232 −13.745 1.00 0.00 H
ATOM 5355 N GLU A 341 27.044 −3.641 −13.567 1.00 0.00 N
ATOM 5356 CA GLU A 341 28.320 −3.684 −12.845 1.00 0.00 C
ATOM 5357 C GLU A 341 29.136 −4.944 −13.186 1.00 0.00 C
ATOM 5358 O GLU A 341 28.926 −6.011 −12.610 1.00 0.00 O
ATOM 5359 CB GLU A 341 28.009 −3.592 −11.337 1.00 0.00 C
ATOM 5360 CG GLU A 341 29.250 −3.611 −10.429 1.00 0.00 C
ATOM 5361 CD GLU A 341 28.901 −3.792 −8.942 1.00 0.00 C
ATOM 5362 OE1 GLU A 341 27.930 −4.535 −8.646 1.00 0.00 O
ATOM 5363 OE2 GLU A 341 29.795 −3.486 −8.126 1.00 0.00 O
ATOM 5364 H GLU A 341 26.555 −4.518 −13.728 1.00 0.00 H
ATOM 5365 HA GLU A 341 28.917 −2.813 −13.116 1.00 0.00 H
ATOM 5366 HB2 GLU A 341 27.361 −4.427 −11.083 1.00 0.00 H
ATOM 5367 HB3 GLU A 341 27.455 −2.673 −11.139 1.00 0.00 H
ATOM 5368 HG2 GLU A 341 29.915 −4.424 −10.721 1.00 0.00 H
ATOM 5369 HG3 GLU A 341 29.795 −2.675 −10.572 1.00 0.00 H
ATOM 5370 N ILE A 342 30.198 −4.801 −13.985 1.00 0.00 N
ATOM 5371 CA ILE A 342 31.266 −5.814 −14.034 1.00 0.00 C

ATOM 5372 C ILE A 342 32.076 −5.717 −12.738 1.00 0.00 C
ATOM 5373 O ILE A 342 32.664 −4.669 −12.454 1.00 0.00 O
ATOM 5374 CB ILE A 342 32.156 −5.677 −15.287 1.00 0.00 C
ATOM 5375 CG1 ILE A 342 31.302 −5.785 −16.568 1.00 0.00 C
ATOM 5376 CG2 ILE A 342 33.254 −6.755 −15.266 1.00 0.00 C
ATOM 5377 CD1 ILE A 342 32.078 −5.541 −17.866 1.00 0.00 C
ATOM 5378 H ILE A 342 30.361 −3.908 −14.423 1.00 0.00 H
ATOM 5379 HA ILE A 342 30.809 −6.800 −14.070 1.00 0.00 H
ATOM 5380 HB ILE A 342 32.633 −4.695 −15.272 1.00 0.00 H
ATOM 5381 HG12 ILE A 342 30.826 −6.764 −16.617 1.00 0.00 H
ATOM 5382 HG13 ILE A 342 30.519 −5.034 −16.523 1.00 0.00 H
ATOM 5383 HG21 ILE A 342 33.873 −6.669 −16.156 1.00 0.00 H
ATOM 5384 HG22 ILE A 342 33.905 −6.638 −14.401 1.00 0.00 H
ATOM 5385 HG23 ILE A 342 32.806 −7.749 −15.244 1.00 0.00 H
ATOM 5386 HD11 ILE A 342 31.375 −5.399 −18.687 1.00 0.00 H
ATOM 5387 HD12 ILE A 342 32.696 −4.648 −17.776 1.00 0.00 H
ATOM 5388 HD13 ILE A 342 32.704 −6.403 −18.093 1.00 0.00 H
ATOM 5389 N LEU A 343 32.133 −6.804 −11.970 1.00 0.00 N
ATOM 5390 CA LEU A 343 32.840 −6.872 −10.693 1.00 0.00 C
ATOM 5391 C LEU A 343 34.351 −7.012 −10.895 1.00 0.00 C
ATOM 5392 O LEU A 343 35.111 −6.214 −10.352 1.00 0.00 O
ATOM 5393 CB LEU A 343 32.286 −8.039 −9.850 1.00 0.00 C
ATOM 5394 CG LEU A 343 30.801 −7.923 −9.469 1.00 0.00 C
ATOM 5395 CD1 LEU A 343 30.341 −9.201 −8.769 1.00 0.00 C
ATOM 5396 CD2 LEU A 343 30.583 −6.765 −8.503 1.00 0.00 C
ATOM 5397 H LEU A 343 31.714 −7.659 −12.319 1.00 0.00 H
ATOM 5398 HA LEU A 343 32.678 −5.940 −10.152 1.00 0.00 H
ATOM 5399 HB2 LEU A 343 32.436 −8.972 −10.397 1.00 0.00 H
ATOM 5400 HB3 LEU A 343 32.863 −8.099 −8.928 1.00 0.00 H
ATOM 5401 HG LEU A 343 30.190 −7.776 −10.359 1.00 0.00 H

ATOM 5402 HD11 LEU A 343 30.416 −10.040 −9.456 1.00 0.00 H
ATOM 5403 HD12 LEU A 343 30.965 −9.394 −7.896 1.00 0.00 H
ATOM 5404 HD13 LEU A 343 29.306 −9.099 −8.451 1.00 0.00 H
ATOM 5405 HD21 LEU A 343 29.538 −6.697 −8.223 1.00 0.00 H
ATOM 5406 HD22 LEU A 343 30.862 −5.823 −8.967 1.00 0.00 H
ATOM 5407 HD23 LEU A 343 31.173 −6.909 −7.602 1.00 0.00 H
ATOM 5408 N TYR A 344 34.778 −7.973 −11.715 1.00 0.00 N
ATOM 5409 CA TYR A 344 36.188 −8.288 −11.978 1.00 0.00 C
ATOM 5410 C TYR A 344 36.325 −9.150 −13.250 1.00 0.00 C
ATOM 5411 O TYR A 344 35.342 −9.718 −13.734 1.00 0.00 O
ATOM 5412 CB TYR A 344 36.816 −8.955 −10.734 1.00 0.00 C
ATOM 5413 CG TYR A 344 35.981 −10.021 −10.046 1.00 0.00 C
ATOM 5414 CD1 TYR A 344 36.069 −11.354 −10.476 1.00 0.00 C
ATOM 5415 CD2 TYR A 344 35.151 −9.691 −8.956 1.00 0.00 C
ATOM 5416 CE1 TYR A 344 35.326 −12.359 −9.829 1.00 0.00 C
ATOM 5417 CE2 TYR A 344 34.375 −10.683 −8.329 1.00 0.00 C
ATOM 5418 CZ TYR A 344 34.464 −12.022 −8.763 1.00 0.00 C
ATOM 5419 OH TYR A 344 33.758 −12.990 −8.126 1.00 0.00 O
ATOM 5420 H TYR A 344 34.087 −8.579 −12.143 1.00 0.00 H
ATOM 5421 HA TYR A 344 36.730 −7.357 −12.155 1.00 0.00 H
ATOM 5422 HB2 TYR A 344 37.782 −9.384 −11.004 1.00 0.00 H
ATOM 5423 HB3 TYR A 344 37.023 −8.176 −10.001 1.00 0.00 H
ATOM 5424 HD1 TYR A 344 36.719 −11.593 −11.300 1.00 0.00 H
ATOM 5425 HD2 TYR A 344 35.110 −8.673 −8.593 1.00 0.00 H
ATOM 5426 HE1 TYR A 344 35.409 −13.386 −10.144 1.00 0.00 H
ATOM 5427 HE2 TYR A 344 33.731 −10.422 −7.504 1.00 0.00 H
ATOM 5428 HH TYR A 344 33.155 −12.609 −7.463 1.00 0.00 H
ATOM 5429 N GLN A 345 37.522 −9.172 −13.848 1.00 0.00 N
ATOM 5430 CA GLN A 345 37.804 −9.771 −15.164 1.00 0.00 C
ATOM 5431 C GLN A 345 39.182 −10.448 −15.201 1.00 0.00 C

ATOM 5432 O GLN A 345 40.060 −10.084 −14.419 1.00 0.00 O
ATOM 5433 CB GLN A 345 37.770 −8.695 −16.268 1.00 0.00 C
ATOM 5434 CG GLN A 345 36.420 −7.986 −16.448 1.00 0.00 C
ATOM 5435 CD GLN A 345 36.325 −7.177 −17.745 1.00 0.00 C
ATOM 5436 OE1 GLN A 345 35.285 −7.105 −18.378 1.00 0.00 O
ATOM 5437 NE2 GLN A 345 37.386 −6.568 −18.228 1.00 0.00 N
ATOM 5438 H GLN A 345 38.307 −8.741 −13.381 1.00 0.00 H
ATOM 5439 HA GLN A 345 37.051 −10.527 −15.383 1.00 0.00 H
ATOM 5440 HB2 GLN A 345 38.023 −9.175 −17.212 1.00 0.00 H
ATOM 5441 HB3 GLN A 345 38.538 −7.949 −16.058 1.00 0.00 H
ATOM 5442 HG2 GLN A 345 36.263 −7.309 −15.609 1.00 0.00 H
ATOM 5443 HG3 GLN A 345 35.628 −8.732 −16.453 1.00 0.00 H
ATOM 5444 HE21 GLN A 345 38.291 −6.686 −17.807 1.00 0.00 H
ATOM 5445 HE22 GLN A 345 37.279 −6.137 −19.133 1.00 0.00 H
ATOM 5446 N VAL A 346 39.417 −11.281 −16.218 1.00 0.00 N
ATOM 5447 CA VAL A 346 40.752 −11.695 −16.682 1.00 0.00 C
ATOM 5448 C VAL A 346 40.706 −12.018 −18.191 1.00 0.00 C
ATOM 5449 O VAL A 346 39.733 −12.636 −18.636 1.00 0.00 O
ATOM 5450 CB VAL A 346 41.278 −12.878 −15.835 1.00 0.00 C
ATOM 5451 CG1 VAL A 346 40.557 −14.206 −16.109 1.00 0.00 C
ATOM 5452 CG2 VAL A 346 42.782 −13.096 −16.004 1.00 0.00 C
ATOM 5453 H VAL A 346 38.632 −11.555 −16.804 1.00 0.00 H
ATOM 5454 HA VAL A 346 41.422 −10.855 −16.519 1.00 0.00 H
ATOM 5455 HB VAL A 346 41.120 −12.629 −14.786 1.00 0.00 H
ATOM 5456 HG21 VAL A 346 43.319 −12.184 −15.746 1.00 0.00 H
ATOM 5457 HG22 VAL A 346 43.019 −13.377 −17.029 1.00 0.00 H
ATOM 5458 HG23 VAL A 346 43.115 −13.891 −15.336 1.00 0.00 H
ATOM 5459 HG11 VAL A 346 40.898 −14.956 −15.400 1.00 0.00 H
ATOM 5460 HG12 VAL A 346 40.776 −14.555 −17.120 1.00 0.00 H
ATOM 5461 HG13 VAL A 346 39.483 −14.070 −15.998 1.00 0.00 H

ATOM 5462 N PRO A 347 41.672 −11.567 −19.015 1.00 0.00 N
ATOM 5463 CA PRO A 347 41.815 −12.059 −20.389 1.00 0.00 C
ATOM 5464 C PRO A 347 42.199 −13.549 −20.397 1.00 0.00 C
ATOM 5465 O PRO A 347 42.816 −14.042 −19.450 1.00 0.00 O
ATOM 5466 CB PRO A 347 42.892 −11.179 −21.026 1.00 0.00 C
ATOM 5467 CG PRO A 347 43.775 −10.790 −19.843 1.00 0.00 C
ATOM 5468 CD PRO A 347 42.779 −10.674 −18.689 1.00 0.00 C
ATOM 5469 HA PRO A 347 40.886 −11.931 −20.942 1.00 0.00 H
ATOM 5470 HB2 PRO A 347 43.459 −11.707 −21.791 1.00 0.00 H
ATOM 5471 HB3 PRO A 347 42.432 −10.283 −21.448 1.00 0.00 H
ATOM 5472 HG2 PRO A 347 44.486 −11.592 −19.637 1.00 0.00 H
ATOM 5473 HG3 PRO A 347 44.299 −9.850 −20.023 1.00 0.00 H
ATOM 5474 HD2 PRO A 347 42.410 −9.649 −18.628 1.00 0.00 H
ATOM 5475 HD3 PRO A 347 43.267 −10.953 −17.758 1.00 0.00 H
ATOM 5476 N LEU A 348 41.781 −14.292 −21.424 1.00 0.00 N
ATOM 5477 CA LEU A 348 42.153 −15.708 −21.601 1.00 0.00 C
ATOM 5478 C LEU A 348 43.313 −15.930 −22.584 1.00 0.00 C
ATOM 5479 O LEU A 348 43.916 −17.003 −22.567 1.00 0.00 O
ATOM 5480 CB LEU A 348 40.908 −16.515 −21.999 1.00 0.00 C
ATOM 5481 CG LEU A 348 39.815 −16.563 −20.916 1.00 0.00 C
ATOM 5482 CD1 LEU A 348 38.663 −17.422 −21.430 1.00 0.00 C
ATOM 5483 CD2 LEU A 348 40.292 −17.178 −19.595 1.00 0.00 C
ATOM 5484 H LEU A 348 41.218 −13.848 −22.145 1.00 0.00 H
ATOM 5485 HA LEU A 348 42.523 −16.102 −20.657 1.00 0.00 H
ATOM 5486 HB2 LEU A 348 40.490 −16.086 −22.913 1.00 0.00 H
ATOM 5487 HB3 LEU A 348 41.219 −17.535 −22.223 1.00 0.00 H
ATOM 5488 HG LEU A 348 39.435 −15.560 −20.721 1.00 0.00 H
ATOM 5489 HD11 LEU A 348 39.032 −18.402 −21.730 1.00 0.00 H
ATOM 5490 HD12 LEU A 348 38.214 −16.928 −22.291 1.00 0.00 H
ATOM 5491 HD13 LEU A 348 37.916 −17.543 −20.651 1.00 0.00 H

ATOM 5492 HD21 LEU A 348 39.453 −17.289 −18.910 1.00 0.00 H
ATOM 5493 HD22 LEU A 348 41.023 −16.516 −19.130 1.00 0.00 H
ATOM 5494 HD23 LEU A 348 40.746 −18.149 −19.785 1.00 0.00 H
ATOM 5495 N ALA A 349 43.635 −14.908 −23.376 1.00 0.00 N
ATOM 5496 CA ALA A 349 44.818 −14.755 −24.219 1.00 0.00 C
ATOM 5497 C ALA A 349 44.987 −13.252 −24.542 1.00 0.00 C
ATOM 5498 O ALA A 349 44.159 −12.438 −24.120 1.00 0.00 O
ATOM 5499 CB ALA A 349 44.645 −15.607 −25.483 1.00 0.00 C
ATOM 5500 H ALA A 349 43.099 −14.058 −23.283 1.00 0.00 H
ATOM 5501 HA ALA A 349 45.709 −15.090 −23.684 1.00 0.00 H
ATOM 5502 HB1 ALA A 349 44.487 −16.651 −25.214 1.00 0.00 H
ATOM 5503 HB2 ALA A 349 45.547 −15.545 −26.090 1.00 0.00 H
ATOM 5504 HB3 ALA A 349 43.792 −15.247 −26.059 1.00 0.00 H
ATOM 5505 N GLU A 350 46.079 −12.879 −25.204 1.00 0.00 N
ATOM 5506 CA GLU A 350 46.498 −11.494 −25.453 1.00 0.00 C
ATOM 5507 C GLU A 350 45.468 −10.642 −26.233 1.00 0.00 C
ATOM 5508 O GLU A 350 44.559 −11.151 −26.894 1.00 0.00 O
ATOM 5509 CB GLU A 350 47.877 −11.500 −26.147 1.00 0.00 C
ATOM 5510 CG GLU A 350 49.067 −11.778 −25.204 1.00 0.00 C
ATOM 5511 CD GLU A 350 49.010 −13.103 −24.418 1.00 0.00 C
ATOM 5512 OE1 GLU A 350 48.528 −14.117 −24.975 1.00 0.00 O
ATOM 5513 OE2 GLU A 350 49.404 −13.083 −23.229 1.00 0.00 O
ATOM 5514 H GLU A 350 46.787 −13.593 −25.381 1.00 0.00 H
ATOM 5515 HA GLU A 350 46.621 −11.007 −24.485 1.00 0.00 H
ATOM 5516 HB2 GLU A 350 48.047 −10.519 −26.594 1.00 0.00 H
ATOM 5517 HB3 GLU A 350 47.876 −12.231 −26.957 1.00 0.00 H
ATOM 5518 HG2 GLU A 350 49.982 −11.765 −25.800 1.00 0.00 H
ATOM 5519 HG3 GLU A 350 49.132 −10.945 −24.499 1.00 0.00 H
ATOM 5520 N ASP A 351 45.567 −9.315 −26.094 1.00 0.00 N
ATOM 5521 CA ASP A 351 44.497 −8.365 −26.441 1.00 0.00 C

ATOM 5522 C ASP A 351 44.094 −8.301 −27.931 1.00 0.00 C

ATOM 5523 O ASP A 351 42.990 −7.836 −28.226 1.00 0.00 O

ATOM 5524 CB ASP A 351 44.881 −6.958 −25.937 1.00 0.00 C

ATOM 5525 CG ASP A 351 44.639 −6.757 −24.435 1.00 0.00 C

ATOM 5526 OD1 ASP A 351 43.468 −6.950 −24.024 1.00 0.00 O

ATOM 5527 OD2 ASP A 351 45.490 −6.093 −23.805 1.00 0.00 O

ATOM 5528 H ASP A 351 46.339 −8.953 −25.556 1.00 0.00 H

ATOM 5529 HA ASP A 351 43.602 −8.685 −25.906 1.00 0.00 H

ATOM 5530 HB2 ASP A 351 44.280 −6.215 −26.462 1.00 0.00 H

ATOM 5531 HB3 ASP A 351 45.927 −6.759 −26.181 1.00 0.00 H

ATOM 5532 N ASP A 352 44.925 −8.767 −28.866 1.00 0.00 N

ATOM 5533 CA ASP A 352 44.606 −8.863 −30.300 1.00 0.00 C

ATOM 5534 C ASP A 352 44.079 −10.250 −30.726 1.00 0.00 C

ATOM 5535 O ASP A 352 43.694 −10.434 −31.882 1.00 0.00 O

ATOM 5536 CB ASP A 352 45.843 −8.477 −31.136 1.00 0.00 C

ATOM 5537 CG ASP A 352 47.062 −9.401 −30.976 1.00 0.00 C

ATOM 5538 OD1 ASP A 352 47.124 −10.148 −29.971 1.00 0.00 O

ATOM 5539 OD2 ASP A 352 47.972 −9.284 −31.827 1.00 0.00 O

ATOM 5540 H ASP A 352 45.803 −9.192 −28.582 1.00 0.00 H

ATOM 5541 HA ASP A 352 43.818 −8.148 −30.537 1.00 0.00 H

ATOM 5542 HB2 ASP A 352 46.141 −7.463 −30.860 1.00 0.00 H

ATOM 5543 HB3 ASP A 352 45.557 −8.452 −32.189 1.00 0.00 H

ATOM 5544 N THR A 353 44.066 −11.241 −29.828 1.00 0.00 N

ATOM 5545 CA THR A 353 43.853 −12.647 −30.209 1.00 0.00 C

ATOM 5546 C THR A 353 42.453 −12.929 −30.763 1.00 0.00 C

ATOM 5547 O THR A 353 41.426 −12.483 −30.240 1.00 0.00 O

ATOM 5548 CB THR A 353 44.200 −13.636 −29.087 1.00 0.00 C

ATOM 5549 OG1 THR A 353 43.480 −13.376 −27.904 1.00 0.00 O

ATOM 5550 CG2 THR A 353 45.697 −13.631 −28.786 1.00 0.00 C

ATOM 5551 H THR A 353 44.425 −11.039 −28.902 1.00 0.00 H

ATOM 5552 HA THR A 353 44.554 −12.847 −31.020 1.00 0.00 H
ATOM 5553 HB THR A 353 43.935 −14.639 −29.424 1.00 0.00 H
ATOM 5554 HG1 THR A 353 43.870 −12.569 −27.510 1.00 0.00 H
ATOM 5555 HG21 THR A 353 46.249 −13.959 −29.666 1.00 0.00 H
ATOM 5556 HG22 THR A 353 46.034 −12.629 −28.521 1.00 0.00 H
ATOM 5557 HG23 THR A 353 45.915 −14.311 −27.966 1.00 0.00 H
ATOM 5558 N ASN A 354 42.429 −13.715 −31.845 1.00 0.00 N
ATOM 5559 CA ASN A 354 41.236 −14.024 −32.631 1.00 0.00 C
ATOM 5560 C ASN A 354 40.294 −14.957 −31.852 1.00 0.00 C
ATOM 5561 O ASN A 354 40.630 −16.120 −31.626 1.00 0.00 O
ATOM 5562 CB ASN A 354 41.618 −14.676 −33.983 1.00 0.00 C
ATOM 5563 CG ASN A 354 42.868 −14.140 −34.658 1.00 0.00 C
ATOM 5564 OD1 ASN A 354 42.810 −13.580 −35.746 1.00 0.00 O
ATOM 5565 ND2 ASN A 354 44.017 −14.604 −34.223 1.00 0.00 N
ATOM 5566 H ASN A 354 43.313 −14.023 −32.214 1.00 0.00 H
ATOM 5567 HA ASN A 354 40.725 −13.083 −32.837 1.00 0.00 H
ATOM 5568 HB2 ASN A 354 40.779 −14.562 −34.668 1.00 0.00 H
ATOM 5569 HB3 ASN A 354 41.778 −15.743 −33.834 1.00 0.00 H
ATOM 5570 HD21 ASN A 354 44.018 −15.343 −33.525 1.00 0.00 H
ATOM 5571 HD22 ASN A 354 44.874 −14.262 −34.638 1.00 0.00 H
ATOM 5572 N ALA A 355 39.044 −14.557 −31.630 1.00 0.00 N
ATOM 5573 CA ALA A 355 38.017 −15.404 −31.026 1.00 0.00 C
ATOM 5574 C ALA A 355 36.608 −15.069 −31.542 1.00 0.00 C
ATOM 5575 O ALA A 355 36.221 −13.909 −31.643 1.00 0.00 O
ATOM 5576 CB ALA A 355 38.142 −15.320 −29.499 1.00 0.00 C
ATOM 5577 H ALA A 355 38.783 −13.618 −31.920 1.00 0.00 H
ATOM 5578 HA ALA A 355 38.217 −16.432 −31.319 1.00 0.00 H
ATOM 5579 HB1 ALA A 355 37.343 −15.891 −29.029 1.00 0.00 H
ATOM 5580 HB2 ALA A 355 39.102 −15.727 −29.185 1.00 0.00 H
ATOM 5581 HB3 ALA A 355 38.096 −14.278 −29.183 1.00 0.00 H

ATOM	5582	N	GLN	A	356	35.828	−16.103	−31.874	1.00	0.00	N
ATOM	5583	CA	GLN	A	356	34.526	−15.982	−32.544	1.00	0.00	C
ATOM	5584	C	GLN	A	356	33.333	−16.170	−31.594	1.00	0.00	C
ATOM	5585	O	GLN	A	356	32.337	−15.453	−31.702	1.00	0.00	O
ATOM	5586	CB	GLN	A	356	34.480	−16.985	−33.715	1.00	0.00	C
ATOM	5587	CG	GLN	A	356	33.157	−16.936	−34.500	1.00	0.00	C
ATOM	5588	CD	GLN	A	356	33.195	−17.799	−35.760	1.00	0.00	C
ATOM	5589	OE1	GLN	A	356	33.313	−17.314	−36.874	1.00	0.00	O
ATOM	5590	NE2	GLN	A	356	33.064	−19.101	−35.649	1.00	0.00	N
ATOM	5591	H	GLN	A	356	36.215	−17.030	−31.725	1.00	0.00	H
ATOM	5592	HA	GLN	A	356	34.438	−14.979	−32.966	1.00	0.00	H
ATOM	5593	HB2	GLN	A	356	35.296	−16.751	−34.400	1.00	0.00	H
ATOM	5594	HB3	GLN	A	356	34.632	−17.996	−33.333	1.00	0.00	H
ATOM	5595	HG2	GLN	A	356	32.342	−17.284	−33.866	1.00	0.00	H
ATOM	5596	HG3	GLN	A	356	32.955	−15.905	−34.794	1.00	0.00	H
ATOM	5597	HE21	GLN	A	356	33.000	−19.557	−34.732	1.00	0.00	H
ATOM	5598	HE22	GLN	A	356	33.138	−19.652	−36.480	1.00	0.00	H
ATOM	5599	N	LYS	A	357	33.388	−17.173	−30.711	1.00	0.00	N
ATOM	5600	CA	LYS	A	357	32.240	−17.681	−29.939	1.00	0.00	C
ATOM	5601	C	LYS	A	357	32.682	−18.168	−28.565	1.00	0.00	C
ATOM	5602	O	LYS	A	357	33.741	−18.776	−28.439	1.00	0.00	O
ATOM	5603	CB	LYS	A	357	31.547	−18.790	−30.761	1.00	0.00	C
ATOM	5604	CG	LYS	A	357	30.540	−19.664	−29.995	1.00	0.00	C
ATOM	5605	CD	LYS	A	357	29.769	−20.596	−30.942	1.00	0.00	C
ATOM	5606	CE	LYS	A	357	29.078	−21.752	−30.201	1.00	0.00	C
ATOM	5607	NZ	LYS	A	357	28.161	−21.295	−29.126	1.00	0.00	N
ATOM	5608	H	LYS	A	357	34.251	−17.700	−30.664	1.00	0.00	H
ATOM	5609	HA	LYS	A	357	31.531	−16.868	−29.782	1.00	0.00	H
ATOM	5610	HB2	LYS	A	357	31.048	−18.328	−31.615	1.00	0.00	H
ATOM	5611	HB3	LYS	A	357	32.314	−19.458	−31.144	1.00	0.00	H

ATOM 5612 HG2 LYS A 357 31.082 −20.276 −29.271 1.00 0.00 H
ATOM 5613 HG3 LYS A 357 29.835 −19.028 −29.463 1.00 0.00 H
ATOM 5614 HD2 LYS A 357 30.468 −21.027 −31.663 1.00 0.00 H
ATOM 5615 HD3 LYS A 357 29.033 −20.017 −31.501 1.00 0.00 H
ATOM 5616 HE2 LYS A 357 28.535 −22.362 −30.932 1.00 0.00 H
ATOM 5617 HE3 LYS A 357 29.848 −22.398 −29.769 1.00 0.00 H
ATOM 5618 HZ1 LYS A 357 28.234 −20.294 −29.012 1.00 0.00 H
ATOM 5619 HZ2 LYS A 357 27.196 −21.512 −29.355 1.00 0.00 H
ATOM 5620 HZ3 LYS A 357 28.374 −21.740 −28.243 1.00 0.00 H
ATOM 5621 N THR A 358 31.786 −18.091 −27.584 1.00 0.00 N
ATOM 5622 CA THR A 358 32.006 −18.621 −26.227 1.00 0.00 C
ATOM 5623 C THR A 358 30.860 −19.538 −25.788 1.00 0.00 C
ATOM 5624 O THR A 358 29.787 −19.525 −26.394 1.00 0.00 O
ATOM 5625 CB THR A 358 32.270 −17.490 −25.212 1.00 0.00 C
ATOM 5626 OG1 THR A 358 31.074 −16.897 −24.765 1.00 0.00 O
ATOM 5627 CG2 THR A 358 33.141 −16.368 −25.790 1.00 0.00 C
ATOM 5628 H THR A 358 30.932 −17.574 −27.756 1.00 0.00 H
ATOM 5629 HA THR A 358 32.904 −19.234 −26.235 1.00 0.00 H
ATOM 5630 HB THR A 358 32.775 −17.927 −24.352 1.00 0.00 H
ATOM 5631 HG1 THR A 358 30.346 −17.278 −25.293 1.00 0.00 H
ATOM 5632 HG21 THR A 358 32.610 −15.830 −26.577 1.00 0.00 H
ATOM 5633 HG22 THR A 358 34.062 −16.785 −26.192 1.00 0.00 H
ATOM 5634 HG23 THR A 358 33.393 −15.649 −25.018 1.00 0.00 H
ATOM 5635 N ILE A 359 31.105 −20.429 −24.825 1.00 0.00 N
ATOM 5636 CA ILE A 359 30.079 −21.182 −24.077 1.00 0.00 C
ATOM 5637 C ILE A 359 30.595 −21.341 −22.645 1.00 0.00 C
ATOM 5638 O ILE A 359 31.689 −21.875 −22.482 1.00 0.00 O
ATOM 5639 CB ILE A 359 29.778 −22.581 −24.683 1.00 0.00 C
ATOM 5640 CG1 ILE A 359 29.423 −22.537 −26.187 1.00 0.00 C
ATOM 5641 CG2 ILE A 359 28.645 −23.257 −23.884 1.00 0.00 C

ATOM 5642 CD1 ILE A 359 29.248 −23.908 −26.847 1.00 0.00 C

ATOM 5643 H ILE A 359 32.060 −20.505 −24.484 1.00 0.00 H

ATOM 5644 HA ILE A 359 29.155 −20.605 −24.054 1.00 0.00 H

ATOM 5645 HB ILE A 359 30.672 −23.197 −24.578 1.00 0.00 H

ATOM 5646 HG12 ILE A 359 30.232 −22.046 −26.727 1.00 0.00 H

ATOM 5647 HG13 ILE A 359 28.508 −21.959 −26.320 1.00 0.00 H

ATOM 5648 HG21 ILE A 359 28.468 −24.269 −24.240 1.00 0.00 H

ATOM 5649 HG22 ILE A 359 27.722 −22.683 −23.976 1.00 0.00 H

ATOM 5650 HG23 ILE A 359 28.904 −23.343 −22.828 1.00 0.00 H

ATOM 5651 HD11 ILE A 359 29.126 −23.779 −27.920 1.00 0.00 H

ATOM 5652 HD12 ILE A 359 30.133 −24.518 −26.666 1.00 0.00 H

ATOM 5653 HD13 ILE A 359 28.366 −24.413 −26.457 1.00 0.00 H

ATOM 5654 N THR A 360 29.848 −20.909 −21.625 1.00 0.00 N

ATOM 5655 CA THR A 360 30.239 −21.043 −20.209 1.00 0.00 C

ATOM 5656 C THR A 360 29.170 −21.768 −19.397 1.00 0.00 C

ATOM 5657 O THR A 360 28.071 −21.240 −19.221 1.00 0.00 O

ATOM 5658 CB THR A 360 30.554 −19.680 −19.570 1.00 0.00 C

ATOM 5659 OG1 THR A 360 31.510 −18.986 −20.352 1.00 0.00 O

ATOM 5660 CG2 THR A 360 31.101 −19.840 −18.148 1.00 0.00 C

ATOM 5661 H THR A 360 28.953 −20.464 −21.828 1.00 0.00 H

ATOM 5662 HA THR A 360 31.147 −21.635 −20.150 1.00 0.00 H

ATOM 5663 HB THR A 360 29.646 −19.076 −19.532 1.00 0.00 H

ATOM 5664 HG1 THR A 360 31.195 −18.072 −20.349 1.00 0.00 H

ATOM 5665 HG21 THR A 360 31.458 −18.886 −17.767 1.00 0.00 H

ATOM 5666 HG22 THR A 360 31.917 −20.562 −18.137 1.00 0.00 H

ATOM 5667 HG23 THR A 360 30.307 −20.191 −17.488 1.00 0.00 H

ATOM 5668 N ASP A 361 29.554 −22.875 −18.757 1.00 0.00 N

ATOM 5669 CA ASP A 361 28.732 −23.626 −17.797 1.00 0.00 C

ATOM 5670 C ASP A 361 29.445 −23.727 −16.432 1.00 0.00 C

ATOM 5671 O ASP A 361 30.665 −23.899 −16.383 1.00 0.00 O

ATOM	5672	CB	ASP	A	361	28.341	−25.012	−18.352	1.00	0.00	C
ATOM	5673	CG	ASP	A	361	27.499	−25.017	−19.645	1.00	0.00	C
ATOM	5674	OD1	ASP	A	361	26.931	−23.966	−20.023	1.00	0.00	O
ATOM	5675	OD2	ASP	A	361	27.259	−26.122	−20.172	1.00	0.00	O
ATOM	5676	H	ASP	A	361	30.515	−23.185	−18.872	1.00	0.00	H
ATOM	5677	HA	ASP	A	361	27.806	−23.087	−17.636	1.00	0.00	H
ATOM	5678	HB2	ASP	A	361	27.766	−25.528	−17.581	1.00	0.00	H
ATOM	5679	HB3	ASP	A	361	29.253	−25.587	−18.525	1.00	0.00	H
ATOM	5680	N	CYS	A	362	28.711	−23.600	−15.317	1.00	0.00	N
ATOM	5681	CA	CYS	A	362	29.288	−23.544	−13.963	1.00	0.00	C
ATOM	5682	C	CYS	A	362	28.628	−24.526	−12.979	1.00	0.00	C
ATOM	5683	O	CYS	A	362	27.448	−24.856	−13.087	1.00	0.00	O
ATOM	5684	CB	CYS	A	362	29.248	−22.110	−13.412	1.00	0.00	C
ATOM	5685	SG	CYS	A	362	29.929	−20.789	−14.458	1.00	0.00	S
ATOM	5686	H	CYS	A	362	27.710	−23.484	−15.398	1.00	0.00	H
ATOM	5687	HA	CYS	A	362	30.330	−23.843	−14.021	1.00	0.00	H
ATOM	5688	HB2	CYS	A	362	29.773	−22.089	−12.457	1.00	0.00	H
ATOM	5689	HB3	CYS	A	362	28.207	−21.865	−13.210	1.00	0.00	H
ATOM	5690	N	PHE	A	363	29.408	−24.998	−12.005	1.00	0.00	N
ATOM	5691	CA	PHE	A	363	29.070	−26.101	−11.101	1.00	0.00	C
ATOM	5692	C	PHE	A	363	29.947	−26.077	−9.832	1.00	0.00	C
ATOM	5693	O	PHE	A	363	30.757	−25.170	−9.634	1.00	0.00	O
ATOM	5694	CB	PHE	A	363	29.207	−27.430	−11.875	1.00	0.00	C
ATOM	5695	CG	PHE	A	363	30.536	−27.636	−12.581	1.00	0.00	C
ATOM	5696	CD1	PHE	A	363	31.601	−28.282	−11.925	1.00	0.00	C
ATOM	5697	CD2	PHE	A	363	30.700	−27.194	−13.909	1.00	0.00	C
ATOM	5698	CE1	PHE	A	363	32.823	−28.480	−12.591	1.00	0.00	C
ATOM	5699	CE2	PHE	A	363	31.923	−27.385	−14.572	1.00	0.00	C
ATOM	5700	CZ	PHE	A	363	32.984	−28.030	−13.914	1.00	0.00	C
ATOM	5701	H	PHE	A	363	30.362	−24.648	−11.955	1.00	0.00	H

ATOM	5702	HA	PHE	A	363	28.032	−25.997	−10.784	1.00	0.00	H
ATOM	5703	HB2	PHE	A	363	28.416	−27.470	−12.625	1.00	0.00	H
ATOM	5704	HB3	PHE	A	363	29.030	−28.269	−11.200	1.00	0.00	H
ATOM	5705	HD1	PHE	A	363	31.479	−28.650	−10.917	1.00	0.00	H
ATOM	5706	HD2	PHE	A	363	29.882	−26.715	−14.429	1.00	0.00	H
ATOM	5707	HE1	PHE	A	363	33.626	−29.002	−12.092	1.00	0.00	H
ATOM	5708	HE2	PHE	A	363	32.032	−27.051	−15.594	1.00	0.00	H
ATOM	5709	HZ	PHE	A	363	33.915	−28.200	−14.432	1.00	0.00	H
ATOM	5710	N	LEU	A	364	29.728	−27.028	−8.919	1.00	0.00	N
ATOM	5711	CA	LEU	A	364	30.560	−27.230	−7.728	1.00	0.00	C
ATOM	5712	C	LEU	A	364	31.562	−28.372	−7.941	1.00	0.00	C
ATOM	5713	O	LEU	A	364	31.174	−29.437	−8.414	1.00	0.00	O
ATOM	5714	CB	LEU	A	364	29.673	−27.522	−6.502	1.00	0.00	C
ATOM	5715	CG	LEU	A	364	28.669	−26.417	−6.127	1.00	0.00	C
ATOM	5716	CD1	LEU	A	364	27.853	−26.858	−4.912	1.00	0.00	C
ATOM	5717	CD2	LEU	A	364	29.362	−25.101	−5.780	1.00	0.00	C
ATOM	5718	H	LEU	A	364	29.099	−27.778	−9.164	1.00	0.00	H
ATOM	5719	HA	LEU	A	364	31.135	−26.327	−7.531	1.00	0.00	H
ATOM	5720	HB2	LEU	A	364	29.118	−28.442	−6.692	1.00	0.00	H
ATOM	5721	HB3	LEU	A	364	30.325	−27.703	−5.649	1.00	0.00	H
ATOM	5722	HG	LEU	A	364	27.984	−26.247	−6.957	1.00	0.00	H
ATOM	5723	HD11	LEU	A	364	27.325	−27.784	−5.140	1.00	0.00	H
ATOM	5724	HD12	LEU	A	364	27.119	−26.092	−4.662	1.00	0.00	H
ATOM	5725	HD13	LEU	A	364	28.506	−27.020	−4.054	1.00	0.00	H
ATOM	5726	HD21	LEU	A	364	28.621	−24.368	−5.463	1.00	0.00	H
ATOM	5727	HD22	LEU	A	364	29.875	−24.717	−6.658	1.00	0.00	H
ATOM	5728	HD23	LEU	A	364	30.079	−25.254	−4.976	1.00	0.00	H
ATOM	5729	N	LEU	A	365	32.764	−28.232	−7.381	1.00	0.00	N
ATOM	5730	CA	LEU	A	365	33.765	−29.298	−7.254	1.00	0.00	C
ATOM	5731	C	LEU	A	365	34.573	−29.096	−5.958	1.00	0.00	C

ATOM 5732 O LEU A 365 34.895 −27.959 −5.620 1.00 0.00 O
ATOM 5733 CB LEU A 365 34.638 −29.307 −8.520 1.00 0.00 C
ATOM 5734 CG LEU A 365 35.660 −30.462 −8.538 1.00 0.00 C
ATOM 5735 CD1 LEU A 365 35.408 −31.381 −9.734 1.00 0.00 C
ATOM 5736 CD2 LEU A 365 37.081 −29.901 −8.594 1.00 0.00 C
ATOM 5737 H LEU A 365 33.019 −27.317 −7.019 1.00 0.00 H
ATOM 5738 HA LEU A 365 33.250 −30.259 −7.194 1.00 0.00 H
ATOM 5739 HB2 LEU A 365 33.986 −29.380 −9.392 1.00 0.00 H
ATOM 5740 HB3 LEU A 365 35.157 −28.354 −8.592 1.00 0.00 H
ATOM 5741 HG LEU A 365 35.558 −31.060 −7.632 1.00 0.00 H
ATOM 5742 HD11 LEU A 365 34.366 −31.701 −9.747 1.00 0.00 H
ATOM 5743 HD12 LEU A 365 36.023 −32.275 −9.638 1.00 0.00 H
ATOM 5744 HD13 LEU A 365 35.643 −30.880 −10.671 1.00 0.00 H
ATOM 5745 HD21 LEU A 365 37.255 −29.247 −7.741 1.00 0.00 H
ATOM 5746 HD22 LEU A 365 37.794 −30.721 −8.533 1.00 0.00 H
ATOM 5747 HD23 LEU A 365 37.246 −29.353 −9.518 1.00 0.00 H
ATOM 5748 N GLU A 366 34.601 −30.118 −5.091 1.00 0.00 N
ATOM 5749 CA GLU A 366 34.884 −29.983 −3.643 1.00 0.00 C
ATOM 5750 C GLU A 366 34.048 −28.886 −2.936 1.00 0.00 C
ATOM 5751 O GLU A 366 34.477 −28.274 −1.959 1.00 0.00 O
ATOM 5752 CB GLU A 366 36.395 −29.860 −3.355 1.00 0.00 C
ATOM 5753 CG GLU A 366 37.145 −31.198 −3.465 1.00 0.00 C
ATOM 5754 CD GLU A 366 38.190 −31.378 −2.348 1.00 0.00 C
ATOM 5755 OE1 GLU A 366 38.821 −30.375 −1.927 1.00 0.00 O
ATOM 5756 OE2 GLU A 366 38.681 −32.516 −2.173 1.00 0.00 O
ATOM 5757 H GLU A 366 34.370 −31.037 −5.440 1.00 0.00 H
ATOM 5758 HA GLU A 366 34.554 −30.909 −3.170 1.00 0.00 H
ATOM 5759 HB2 GLU A 366 36.510 −29.515 −2.329 1.00 0.00 H
ATOM 5760 HB3 GLU A 366 36.856 −29.118 −4.008 1.00 0.00 H
ATOM 5761 HG2 GLU A 366 37.630 −31.256 −4.443 1.00 0.00 H

ATOM	5762	HG3	GLU	A	366	36.432	−32.022	−3.396	1.00	0.00	H
ATOM	5763	N	ASN	A	367	32.802	−28.675	−3.380	1.00	0.00	N
ATOM	5764	CA	ASN	A	367	31.907	−27.593	−2.925	1.00	0.00	C
ATOM	5765	C	ASN	A	367	32.383	−26.165	−3.288	1.00	0.00	C
ATOM	5766	O	ASN	A	367	31.709	−25.190	−2.958	1.00	0.00	O
ATOM	5767	CB	ASN	A	367	31.563	−27.725	−1.424	1.00	0.00	C
ATOM	5768	CG	ASN	A	367	31.368	−29.150	−0.942	1.00	0.00	C
ATOM	5769	OD1	ASN	A	367	30.305	−29.730	−1.059	1.00	0.00	O
ATOM	5770	ND2	ASN	A	367	32.399	−29.764	−0.407	1.00	0.00	N
ATOM	5771	H	ASN	A	367	32.498	−29.235	−4.161	1.00	0.00	H
ATOM	5772	HA	ASN	A	367	30.969	−27.732	−3.461	1.00	0.00	H
ATOM	5773	HB2	ASN	A	367	32.357	−27.270	−0.832	1.00	0.00	H
ATOM	5774	HB3	ASN	A	367	30.649	−27.170	−1.218	1.00	0.00	H
ATOM	5775	HD21	ASN	A	367	33.303	−29.311	−0.454	1.00	0.00	H
ATOM	5776	HD22	ASN	A	367	32.270	−30.720	−0.134	1.00	0.00	H
ATOM	5777	N	VAL	A	368	33.491	−26.020	−4.022	1.00	0.00	N
ATOM	5778	CA	VAL	A	368	33.969	−24.747	−4.578	1.00	0.00	C
ATOM	5779	C	VAL	A	368	33.324	−24.503	−5.943	1.00	0.00	C
ATOM	5780	O	VAL	A	368	33.290	−25.400	−6.783	1.00	0.00	O
ATOM	5781	CB	VAL	A	368	35.508	−24.732	−4.680	1.00	0.00	C
ATOM	5782	CG1	VAL	A	368	36.033	−23.389	−5.208	1.00	0.00	C
ATOM	5783	CG2	VAL	A	368	36.160	−24.977	−3.311	1.00	0.00	C
ATOM	5784	H	VAL	A	368	33.989	−26.857	−4.310	1.00	0.00	H
ATOM	5785	HA	VAL	A	368	33.669	−23.943	−3.907	1.00	0.00	H
ATOM	5786	HB	VAL	A	368	35.837	−25.513	−5.364	1.00	0.00	H
ATOM	5787	HG21	VAL	A	368	35.901	−25.974	−2.949	1.00	0.00	H
ATOM	5788	HG22	VAL	A	368	37.245	−24.929	−3.397	1.00	0.00	H
ATOM	5789	HG23	VAL	A	368	35.814	−24.238	−2.588	1.00	0.00	H
ATOM	5790	HG11	VAL	A	368	35.705	−23.237	−6.236	1.00	0.00	H
ATOM	5791	HG12	VAL	A	368	37.123	−23.403	−5.202	1.00	0.00	H

ATOM 5792 HG13 VAL A 368 35.676 −22.572 −4.582 1.00 0.00 H
ATOM 5793 N ILE A 369 32.876 −23.270 −6.200 1.00 0.00 N
ATOM 5794 CA ILE A 369 32.316 −22.881 −7.499 1.00 0.00 C
ATOM 5795 C ILE A 369 33.428 −22.871 −8.564 1.00 0.00 C
ATOM 5796 O ILE A 369 34.371 −22.074 −8.495 1.00 0.00 O
ATOM 5797 CB ILE A 369 31.559 −21.530 −7.438 1.00 0.00 C
ATOM 5798 CG1 ILE A 369 30.408 −21.464 −6.404 1.00 0.00 C
ATOM 5799 CG2 ILE A 369 30.949 −21.216 −8.819 1.00 0.00 C
ATOM 5800 CD1 ILE A 369 30.844 −21.176 −4.960 1.00 0.00 C
ATOM 5801 H ILE A 369 32.924 −22.578 −5.473 1.00 0.00 H
ATOM 5802 HA ILE A 369 31.591 −23.642 −7.788 1.00 0.00 H
ATOM 5803 HB ILE A 369 32.268 −20.742 −7.194 1.00 0.00 H
ATOM 5804 HG12 ILE A 369 29.830 −22.386 −6.436 1.00 0.00 H
ATOM 5805 HG13 ILE A 369 29.734 −20.653 −6.681 1.00 0.00 H
ATOM 5806 HG21 ILE A 369 30.446 −20.248 −8.802 1.00 0.00 H
ATOM 5807 HG22 ILE A 369 31.716 −21.179 −9.591 1.00 0.00 H
ATOM 5808 HG23 ILE A 369 30.223 −21.985 −9.091 1.00 0.00 H
ATOM 5809 HD11 ILE A 369 29.959 −20.971 −4.357 1.00 0.00 H
ATOM 5810 HD12 ILE A 369 31.498 −20.304 −4.933 1.00 0.00 H
ATOM 5811 HD13 ILE A 369 31.353 −22.035 −4.527 1.00 0.00 H
ATOM 5812 N TRP A 370 33.221 −23.646 −9.624 1.00 0.00 N
ATOM 5813 CA TRP A 370 34.004 −23.652 −10.859 1.00 0.00 C
ATOM 5814 C TRP A 370 33.111 −23.305 −12.051 1.00 0.00 C
ATOM 5815 O TRP A 370 31.931 −23.647 −12.076 1.00 0.00 O
ATOM 5816 CB TRP A 370 34.646 −25.033 −11.057 1.00 0.00 C
ATOM 5817 CG TRP A 370 35.935 −25.278 −10.333 1.00 0.00 C
ATOM 5818 CD1 TRP A 370 36.062 −25.804 −9.094 1.00 0.00 C
ATOM 5819 CD2 TRP A 370 37.300 −25.058 −10.811 1.00 0.00 C
ATOM 5820 NE1 TRP A 370 37.401 −25.981 −8.797 1.00 0.00 N
ATOM 5821 CE2 TRP A 370 38.209 −25.521 −9.814 1.00 0.00 C

ATOM 5822 CE3 TRP A 370 37.861 −24.560 −12.008 1.00 0.00 C
ATOM 5823 CZ2 TRP A 370 39.600 −25.486 −9.992 1.00 0.00 C
ATOM 5824 CZ3 TRP A 370 39.260 −24.502 −12.189 1.00 0.00 C
ATOM 5825 CH2 TRP A 370 40.127 −24.969 −11.188 1.00 0.00 C
ATOM 5826 H TRP A 370 32.381 −24.220 −9.628 1.00 0.00 H
ATOM 5827 HA TRP A 370 34.795 −22.906 −10.811 1.00 0.00 H
ATOM 5828 HB2 TRP A 370 34.856 −25.166 −12.119 1.00 0.00 H
ATOM 5829 HB3 TRP A 370 33.928 −25.809 −10.785 1.00 0.00 H
ATOM 5830 HD1 TRP A 370 35.228 −26.104 −8.464 1.00 0.00 H
ATOM 5831 HE3 TRP A 370 37.199 −24.243 −12.800 1.00 0.00 H
ATOM 5832 HZ2 TRP A 370 40.251 −25.888 −9.229 1.00 0.00 H
ATOM 5833 HZ3 TRP A 370 39.672 −24.128 −13.115 1.00 0.00 H
ATOM 5834 HH2 TRP A 370 41.194 −24.953 −11.349 1.00 0.00 H
ATOM 5835 HE1 TRP A 370 37.724 −26.535 −8.013 1.00 0.00 H
ATOM 5836 N CYS A 371 33.726 −22.774 −13.102 1.00 0.00 N
ATOM 5837 CA CYS A 371 33.159 −22.695 −14.442 1.00 0.00 C
ATOM 5838 C CYS A 371 34.091 −23.385 −15.442 1.00 0.00 C
ATOM 5839 O CYS A 371 35.315 −23.285 −15.323 1.00 0.00 O
ATOM 5840 CB CYS A 371 32.919 −21.229 −14.810 1.00 0.00 C
ATOM 5841 SG CYS A 371 31.775 −20.338 −13.723 1.00 0.00 S
ATOM 5842 H CYS A 371 34.693 −22.492 −13.002 1.00 0.00 H
ATOM 5843 HA CYS A 371 32.205 −23.213 −14.472 1.00 0.00 H
ATOM 5844 HB2 CYS A 371 33.879 −20.711 −14.791 1.00 0.00 H
ATOM 5845 HB3 CYS A 371 32.533 −21.179 −15.828 1.00 0.00 H
ATOM 5846 N ILE A 372 33.514 −23.989 −16.478 1.00 0.00 N
ATOM 5847 CA ILE A 372 34.205 −24.331 −17.721 1.00 0.00 C
ATOM 5848 C ILE A 372 33.719 −23.359 −18.793 1.00 0.00 C
ATOM 5849 O ILE A 372 32.521 −23.297 −19.076 1.00 0.00 O
ATOM 5850 CB ILE A 372 33.947 −25.805 −18.112 1.00 0.00 C
ATOM 5851 CG1 ILE A 372 34.562 −26.794 −17.094 1.00 0.00 C

ATOM 5852 CG2 ILE A 372 34.447 −26.109 −19.538 1.00 0.00 C
ATOM 5853 CD1 ILE A 372 36.087 −26.936 −17.155 1.00 0.00 C
ATOM 5854 H ILE A 372 32.496 −23.990 −16.515 1.00 0.00 H
ATOM 5855 HA ILE A 372 35.278 −24.189 −17.603 1.00 0.00 H
ATOM 5856 HB ILE A 372 32.866 −25.964 −18.106 1.00 0.00 H
ATOM 5857 HG12 ILE A 372 34.295 −26.483 −16.086 1.00 0.00 H
ATOM 5858 HG13 ILE A 372 34.125 −27.780 −17.260 1.00 0.00 H
ATOM 5859 HG21 ILE A 372 34.319 −27.170 −19.755 1.00 0.00 H
ATOM 5860 HG22 ILE A 372 35.498 −25.838 −19.645 1.00 0.00 H
ATOM 5861 HG23 ILE A 372 33.867 −25.544 −20.267 1.00 0.00 H
ATOM 5862 HD11 ILE A 372 36.560 −25.959 −17.102 1.00 0.00 H
ATOM 5863 HD12 ILE A 372 36.434 −27.544 −16.322 1.00 0.00 H
ATOM 5864 HD13 ILE A 372 36.374 −27.427 −18.082 1.00 0.00 H
ATOM 5865 N SER A 373 34.655 −22.674 −19.445 1.00 0.00 N
ATOM 5866 CA SER A 373 34.400 −21.909 −20.663 1.00 0.00 C
ATOM 5867 C SER A 373 35.076 −22.576 −21.855 1.00 0.00 C
ATOM 5868 O SER A 373 36.304 −22.649 −21.926 1.00 0.00 O
ATOM 5869 CB SER A 373 34.860 −20.458 −20.526 1.00 0.00 C
ATOM 5870 OG SER A 373 34.121 −19.796 −19.517 1.00 0.00 O
ATOM 5871 H SER A 373 35.622 −22.790 −19.160 1.00 0.00 H
ATOM 5872 HA SER A 373 33.332 −21.883 −20.845 1.00 0.00 H
ATOM 5873 HB2 SER A 373 35.921 −20.434 −20.273 1.00 0.00 H
ATOM 5874 HB3 SER A 373 34.711 −19.942 −21.477 1.00 0.00 H
ATOM 5875 HG SER A 373 33.565 −19.107 −19.925 1.00 0.00 H
ATOM 5876 N LEU A 374 34.289 −22.959 −22.858 1.00 0.00 N
ATOM 5877 CA LEU A 374 34.793 −23.130 −24.216 1.00 0.00 C
ATOM 5878 C LEU A 374 34.934 −21.749 −24.865 1.00 0.00 C
ATOM 5879 O LEU A 374 33.976 −20.972 −24.866 1.00 0.00 O
ATOM 5880 CB LEU A 374 33.834 −24.034 −25.013 1.00 0.00 C
ATOM 5881 CG LEU A 374 34.391 −24.484 −26.376 1.00 0.00 C

ATOM 5882 CD1 LEU A 374 35.487 −25.538 −26.208 1.00 0.00 C
ATOM 5883 CD2 LEU A 374 33.282 −25.103 −27.222 1.00 0.00 C
ATOM 5884 H LEU A 374 33.295 −22.780 −22.750 1.00 0.00 H
ATOM 5885 HA LEU A 374 35.777 −23.597 −24.172 1.00 0.00 H
ATOM 5886 HB2 LEU A 374 33.599 −24.918 −24.424 1.00 0.00 H
ATOM 5887 HB3 LEU A 374 32.906 −23.486 −25.175 1.00 0.00 H
ATOM 5888 HG LEU A 374 34.794 −23.630 −26.919 1.00 0.00 H
ATOM 5889 HD11 LEU A 374 36.346 −25.110 −25.694 1.00 0.00 H
ATOM 5890 HD12 LEU A 374 35.810 −25.900 −27.179 1.00 0.00 H
ATOM 5891 HD13 LEU A 374 35.098 −26.384 −25.645 1.00 0.00 H
ATOM 5892 HD21 LEU A 374 33.702 −25.457 −28.163 1.00 0.00 H
ATOM 5893 HD22 LEU A 374 32.520 −24.353 −27.431 1.00 0.00 H
ATOM 5894 HD23 LEU A 374 32.830 −25.945 −26.698 1.00 0.00 H
ATOM 5895 N VAL A 375 36.044 −21.521 −25.565 1.00 0.00 N
ATOM 5896 CA VAL A 375 36.191 −20.403 −26.507 1.00 0.00 C
ATOM 5897 C VAL A 375 36.578 −20.959 −27.873 1.00 0.00 C
ATOM 5898 O VAL A 375 37.483 −21.783 −27.971 1.00 0.00 O
ATOM 5899 CB VAL A 375 37.206 −19.351 −26.014 1.00 0.00 C
ATOM 5900 CG1 VAL A 375 37.118 −18.073 −26.857 1.00 0.00 C
ATOM 5901 CG2 VAL A 375 36.977 −18.946 −24.552 1.00 0.00 C
ATOM 5902 H VAL A 375 36.813 −22.183 −25.505 1.00 0.00 H
ATOM 5903 HA VAL A 375 35.232 −19.898 −26.617 1.00 0.00 H
ATOM 5904 HB VAL A 375 38.214 −19.760 −26.102 1.00 0.00 H
ATOM 5905 HG11 VAL A 375 37.916 −17.387 −26.572 1.00 0.00 H
ATOM 5906 HG12 VAL A 375 37.219 −18.300 −27.917 1.00 0.00 H
ATOM 5907 HG13 VAL A 375 36.157 −17.584 −26.704 1.00 0.00 H
ATOM 5908 HG21 VAL A 375 37.682 −18.162 −24.278 1.00 0.00 H
ATOM 5909 HG22 VAL A 375 35.957 −18.584 −24.419 1.00 0.00 H
ATOM 5910 HG23 VAL A 375 37.147 −19.796 −23.893 1.00 0.00 H
ATOM 5911 N GLU A 376 35.851 −20.579 −28.919 1.00 0.00 N

ATOM 5912 CA GLU A 376 36.264 −20.747 −30.313 1.00 0.00 C
ATOM 5913 C GLU A 376 37.273 −19.642 −30.657 1.00 0.00 C
ATOM 5914 O GLU A 376 36.888 −18.483 −30.801 1.00 0.00 O
ATOM 5915 CB GLU A 376 35.017 −20.720 −31.211 1.00 0.00 C
ATOM 5916 CG GLU A 376 35.308 −21.117 −32.667 1.00 0.00 C
ATOM 5917 CD GLU A 376 34.017 −21.277 −33.488 1.00 0.00 C
ATOM 5918 OE1 GLU A 376 33.056 −20.506 −33.262 1.00 0.00 O
ATOM 5919 OE2 GLU A 376 33.985 −22.125 −34.409 1.00 0.00 O
ATOM 5920 H GLU A 376 35.124 −19.892 −28.747 1.00 0.00 H
ATOM 5921 HA GLU A 376 36.746 −21.714 −30.440 1.00 0.00 H
ATOM 5922 HB2 GLU A 376 34.285 −21.417 −30.802 1.00 0.00 H
ATOM 5923 HB3 GLU A 376 34.585 −19.721 −31.192 1.00 0.00 H
ATOM 5924 HG2 GLU A 376 35.859 −22.060 −32.670 1.00 0.00 H
ATOM 5925 HG3 GLU A 376 35.942 −20.356 −33.128 1.00 0.00 H
ATOM 5926 N ILE A 377 38.560 −19.974 −30.551 1.00 0.00 N
ATOM 5927 CA ILE A 377 39.715 −19.063 −30.511 1.00 0.00 C
ATOM 5928 C ILE A 377 40.825 −19.616 −31.409 1.00 0.00 C
ATOM 5929 O ILE A 377 40.956 −20.835 −31.533 1.00 0.00 O
ATOM 5930 CB ILE A 377 40.193 −18.856 −29.044 1.00 0.00 C
ATOM 5931 CG1 ILE A 377 41.370 −17.861 −28.895 1.00 0.00 C
ATOM 5932 CG2 ILE A 377 40.575 −20.189 −28.381 1.00 0.00 C
ATOM 5933 CD1 ILE A 377 41.760 −17.543 −27.445 1.00 0.00 C
ATOM 5934 H ILE A 377 38.792 −20.946 −30.417 1.00 0.00 H
ATOM 5935 HA ILE A 377 39.417 −18.098 −30.910 1.00 0.00 H
ATOM 5936 HB ILE A 377 39.355 −18.439 −28.493 1.00 0.00 H
ATOM 5937 HG12 ILE A 377 41.090 −16.919 −29.357 1.00 0.00 H
ATOM 5938 HG13 ILE A 377 42.257 −18.239 −29.405 1.00 0.00 H
ATOM 5939 HG21 ILE A 377 39.786 −20.926 −28.493 1.00 0.00 H
ATOM 5940 HG22 ILE A 377 41.480 −20.569 −28.849 1.00 0.00 H
ATOM 5941 HG23 ILE A 377 40.746 −20.042 −27.317 1.00 0.00 H

ATOM 5942 HD11 ILE A 377 42.441 −16.693 −27.441 1.00 0.00 H
ATOM 5943 HD12 ILE A 377 42.272 −18.391 −26.991 1.00 0.00 H
ATOM 5944 HD13 ILE A 377 40.873 −17.294 −26.862 1.00 0.00 H
ATOM 5945 N TYR A 378 41.666 −18.748 −31.963 1.00 0.00 N
ATOM 5946 CA TYR A 378 42.959 −19.143 −32.511 1.00 0.00 C
ATOM 5947 C TYR A 378 44.014 −18.052 −32.337 1.00 0.00 C
ATOM 5948 O TYR A 378 43.752 −16.851 −32.448 1.00 0.00 O
ATOM 5949 CB TYR A 378 42.828 −19.608 −33.963 1.00 0.00 C
ATOM 5950 CG TYR A 378 42.441 −18.546 −34.974 1.00 0.00 C
ATOM 5951 CD1 TYR A 378 43.450 −17.841 −35.654 1.00 0.00 C
ATOM 5952 CD2 TYR A 378 41.086 −18.255 −35.223 1.00 0.00 C
ATOM 5953 CE1 TYR A 378 43.111 −16.807 −36.546 1.00 0.00 C
ATOM 5954 CE2 TYR A 378 40.740 −17.229 −36.127 1.00 0.00 C
ATOM 5955 CZ TYR A 378 41.755 −16.488 −36.770 1.00 0.00 C
ATOM 5956 OH TYR A 378 41.435 −15.394 −37.504 1.00 0.00 O
ATOM 5957 H TYR A 378 41.514 −17.751 −31.822 1.00 0.00 H
ATOM 5958 HA TYR A 378 43.310 −20.003 −31.939 1.00 0.00 H
ATOM 5959 HB2 TYR A 378 43.782 −20.030 −34.263 1.00 0.00 H
ATOM 5960 HB3 TYR A 378 42.114 −20.422 −33.987 1.00 0.00 H
ATOM 5961 HD1 TYR A 378 44.491 −18.061 −35.454 1.00 0.00 H
ATOM 5962 HD2 TYR A 378 40.315 −18.787 −34.685 1.00 0.00 H
ATOM 5963 HE1 TYR A 378 43.883 −16.218 −37.014 1.00 0.00 H
ATOM 5964 HE2 TYR A 378 39.709 −16.955 −36.281 1.00 0.00 H
ATOM 5965 HH TYR A 378 42.146 −14.737 −37.421 1.00 0.00 H
ATOM 5966 N ASP A 379 45.229 −18.505 −32.081 1.00 0.00 N
ATOM 5967 CA ASP A 379 46.409 −17.666 −31.941 1.00 0.00 C
ATOM 5968 C ASP A 379 46.945 −17.392 −33.360 1.00 0.00 C
ATOM 5969 O ASP A 379 46.771 −18.225 −34.255 1.00 0.00 O
ATOM 5970 CB ASP A 379 47.446 −18.347 −31.017 1.00 0.00 C
ATOM 5971 CG ASP A 379 46.906 −18.974 −29.706 1.00 0.00 C

ATOM 5972 OD1 ASP A 379 45.724 −18.749 −29.347 1.00 0.00 O
ATOM 5973 OD2 ASP A 379 47.461 −20.027 −29.313 1.00 0.00 O
ATOM 5974 H ASP A 379 45.380 −19.510 −32.129 1.00 0.00 H
ATOM 5975 HA ASP A 379 46.118 −16.721 −31.479 1.00 0.00 H
ATOM 5976 HB2 ASP A 379 47.936 −19.136 −31.593 1.00 0.00 H
ATOM 5977 HB3 ASP A 379 48.209 −17.610 −30.761 1.00 0.00 H
ATOM 5978 N THR A 380 47.505 −16.206 −33.627 1.00 0.00 N
ATOM 5979 CA THR A 380 47.855 −15.758 −34.996 1.00 0.00 C
ATOM 5980 C THR A 380 49.080 −16.505 −35.558 1.00 0.00 C
ATOM 5981 O THR A 380 50.196 −15.993 −35.596 1.00 0.00 O
ATOM 5982 CB THR A 380 47.994 −14.225 −35.055 1.00 0.00 C
ATOM 5983 OG1 THR A 380 46.804 −13.651 −34.556 1.00 0.00 O
ATOM 5984 CG2 THR A 380 48.147 −13.683 −36.478 1.00 0.00 C
ATOM 5985 H THR A 380 47.552 −15.520 −32.888 1.00 0.00 H
ATOM 5986 HA THR A 380 47.015 −16.009 −35.643 1.00 0.00 H
ATOM 5987 HB THR A 380 48.837 −13.903 −34.442 1.00 0.00 H
ATOM 5988 HG1 THR A 380 47.005 −12.751 −34.267 1.00 0.00 H
ATOM 5989 HG21 THR A 380 48.158 −12.594 −36.455 1.00 0.00 H
ATOM 5990 HG22 THR A 380 47.314 −14.019 −37.097 1.00 0.00 H
ATOM 5991 HG23 THR A 380 49.084 −14.026 −36.915 1.00 0.00 H
ATOM 5992 N GLY A 381 48.872 −17.784 −35.881 1.00 0.00 N
ATOM 5993 CA GLY A 381 49.893 −18.808 −36.122 1.00 0.00 C
ATOM 5994 C GLY A 381 49.376 −20.258 −36.023 1.00 0.00 C
ATOM 5995 O GLY A 381 50.028 −21.162 −36.541 1.00 0.00 O
ATOM 5996 H GLY A 381 47.931 −18.116 −35.707 1.00 0.00 H
ATOM 5997 HA2 GLY A 381 50.690 −18.688 −35.389 1.00 0.00 H
ATOM 5998 HA3 GLY A 381 50.316 −18.663 −37.116 1.00 0.00 H
ATOM 5999 N ASP A 382 48.168 −20.490 −35.488 1.00 0.00 N
ATOM 6000 CA ASP A 382 47.481 −21.792 −35.521 1.00 0.00 C
ATOM 6001 C ASP A 382 47.144 −22.237 −36.963 1.00 0.00 C

ATOM 6002 O ASP A 382 46.095 −21.890 −37.507 1.00 0.00 O
ATOM 6003 CB ASP A 382 46.181 −21.726 −34.690 1.00 0.00 C
ATOM 6004 CG ASP A 382 46.356 −21.843 −33.174 1.00 0.00 C
ATOM 6005 OD1 ASP A 382 47.052 −22.799 −32.764 1.00 0.00 O
ATOM 6006 OD2 ASP A 382 45.466 −21.325 −32.460 1.00 0.00 O
ATOM 6007 H ASP A 382 47.671 −19.725 −35.045 1.00 0.00 H
ATOM 6008 HA ASP A 382 48.130 −22.547 −35.076 1.00 0.00 H
ATOM 6009 HB2 ASP A 382 45.674 −20.793 −34.932 1.00 0.00 H
ATOM 6010 HB3 ASP A 382 45.529 −22.548 −34.994 1.00 0.00 H
ATOM 6011 N SER A 383 47.910 −23.191 −37.507 1.00 0.00 N
ATOM 6012 CA SER A 383 47.548 −23.910 −38.748 1.00 0.00 C
ATOM 6013 C SER A 383 46.277 −24.760 −38.597 1.00 0.00 C
ATOM 6014 O SER A 383 45.556 −24.997 −39.568 1.00 0.00 O
ATOM 6015 CB SER A 383 48.680 −24.854 −39.169 1.00 0.00 C
ATOM 6016 OG SER A 383 49.909 −24.161 −39.274 1.00 0.00 O
ATOM 6017 H SER A 383 48.843 −23.308 −37.136 1.00 0.00 H
ATOM 6018 HA SER A 383 47.380 −23.185 −39.544 1.00 0.00 H
ATOM 6019 HB2 SER A 383 48.782 −25.648 −38.428 1.00 0.00 H
ATOM 6020 HB3 SER A 383 48.434 −25.303 −40.132 1.00 0.00 H
ATOM 6021 HG SER A 383 50.607 −24.788 −39.476 1.00 0.00 H
ATOM 6022 N VAL A 384 46.026 −25.245 −37.375 1.00 0.00 N
ATOM 6023 CA VAL A 384 44.875 −26.073 −36.993 1.00 0.00 C
ATOM 6024 C VAL A 384 44.001 −25.266 −36.031 1.00 0.00 C
ATOM 6025 O VAL A 384 44.065 −25.443 −34.817 1.00 0.00 O
ATOM 6026 CB VAL A 384 45.326 −27.433 −36.412 1.00 0.00 C
ATOM 6027 CG1 VAL A 384 44.135 −28.338 −36.052 1.00 0.00 C
ATOM 6028 CG2 VAL A 384 46.197 −28.214 −37.406 1.00 0.00 C
ATOM 6029 H VAL A 384 46.631 −24.924 −36.633 1.00 0.00 H
ATOM 6030 HA VAL A 384 44.278 −26.280 −37.879 1.00 0.00 H
ATOM 6031 HB VAL A 384 45.916 −27.262 −35.511 1.00 0.00 H

ATOM 6032 HG11 VAL A 384 44.499 −29.296 −35.684 1.00 0.00 H
ATOM 6033 HG12 VAL A 384 43.511 −28.503 −36.929 1.00 0.00 H
ATOM 6034 HG13 VAL A 384 43.531 −27.879 −35.270 1.00 0.00 H
ATOM 6035 HG21 VAL A 384 46.486 −29.174 −36.980 1.00 0.00 H
ATOM 6036 HG22 VAL A 384 47.104 −27.655 −37.632 1.00 0.00 H
ATOM 6037 HG23 VAL A 384 45.650 −28.379 −38.333 1.00 0.00 H
ATOM 6038 N ILE A 385 43.249 −24.310 −36.577 1.00 0.00 N
ATOM 6039 CA ILE A 385 42.337 −23.437 −35.826 1.00 0.00 C
ATOM 6040 C ILE A 385 41.344 −24.305 −35.044 1.00 0.00 C
ATOM 6041 O ILE A 385 40.645 −25.134 −35.635 1.00 0.00 O
ATOM 6042 CB ILE A 385 41.624 −22.470 −36.805 1.00 0.00 C
ATOM 6043 CG1 ILE A 385 42.651 −21.452 −37.353 1.00 0.00 C
ATOM 6044 CG2 ILE A 385 40.401 −21.784 −36.165 1.00 0.00 C
ATOM 6045 CD1 ILE A 385 42.103 −20.467 −38.393 1.00 0.00 C
ATOM 6046 H ILE A 385 43.242 −24.251 −37.589 1.00 0.00 H
ATOM 6047 HA ILE A 385 42.917 −22.850 −35.113 1.00 0.00 H
ATOM 6048 HB ILE A 385 41.254 −23.053 −37.649 1.00 0.00 H
ATOM 6049 HG12 ILE A 385 43.083 −20.886 −36.528 1.00 0.00 H
ATOM 6050 HG13 ILE A 385 43.462 −22.000 −37.833 1.00 0.00 H
ATOM 6051 HG21 ILE A 385 39.947 −21.082 −36.860 1.00 0.00 H
ATOM 6052 HG22 ILE A 385 40.685 −21.247 −35.268 1.00 0.00 H
ATOM 6053 HG23 ILE A 385 39.641 −22.519 −35.901 1.00 0.00 H
ATOM 6054 HD11 ILE A 385 41.596 −21.011 −39.190 1.00 0.00 H
ATOM 6055 HD12 ILE A 385 42.932 −19.902 −38.818 1.00 0.00 H
ATOM 6056 HD13 ILE A 385 41.414 −19.763 −37.927 1.00 0.00 H
ATOM 6057 N ARG A 386 41.248 −24.131 −33.720 1.00 0.00 N
ATOM 6058 CA ARG A 386 40.342 −24.943 −32.893 1.00 0.00 C
ATOM 6059 C ARG A 386 39.898 −24.269 −31.591 1.00 0.00 C
ATOM 6060 O ARG A 386 40.740 −23.708 −30.900 1.00 0.00 O
ATOM 6061 CB ARG A 386 40.968 −26.324 −32.608 1.00 0.00 C

ATOM 6062 CG ARG A 386 42.339 −26.281 −31.895 1.00 0.00 C
ATOM 6063 CD ARG A 386 42.590 −27.542 −31.066 1.00 0.00 C
ATOM 6064 NE ARG A 386 41.672 −27.601 −29.911 1.00 0.00 N
ATOM 6065 CZ ARG A 386 41.283 −28.664 −29.239 1.00 0.00 C
ATOM 6066 NH1 ARG A 386 41.760 −29.854 −29.480 1.00 0.00 N
ATOM 6067 NH2 ARG A 386 40.388 −28.535 −28.308 1.00 0.00 N
ATOM 6068 H ARG A 386 41.840 −23.442 −33.270 1.00 0.00 H
ATOM 6069 HA ARG A 386 39.447 −25.090 −33.492 1.00 0.00 H
ATOM 6070 HB2 ARG A 386 40.255 −26.894 −32.014 1.00 0.00 H
ATOM 6071 HB3 ARG A 386 41.099 −26.858 −33.549 1.00 0.00 H
ATOM 6072 HG2 ARG A 386 43.124 −26.191 −32.640 1.00 0.00 H
ATOM 6073 HG3 ARG A 386 42.420 −25.424 −31.231 1.00 0.00 H
ATOM 6074 HD2 ARG A 386 42.462 −28.413 −31.712 1.00 0.00 H
ATOM 6075 HD3 ARG A 386 43.621 −27.517 −30.707 1.00 0.00 H
ATOM 6076 HE ARG A 386 41.225 −26.729 −29.640 1.00 0.00 H
ATOM 6077 HH11 ARG A 386 42.465 −29.942 −30.189 1.00 0.00 H
ATOM 6078 HH12 ARG A 386 41.450 −30.644 −28.951 1.00 0.00 H
ATOM 6079 HH21 ARG A 386 40.008 −27.600 −28.162 1.00 0.00 H
ATOM 6080 HH22 ARG A 386 40.035 −29.314 −27.791 1.00 0.00 H
ATOM 6081 N PRO A 387 38.706 −24.627 −31.071 1.00 0.00 N
ATOM 6082 CA PRO A 387 38.286 −24.243 −29.731 1.00 0.00 C
ATOM 6083 C PRO A 387 39.254 −24.707 −28.635 1.00 0.00 C
ATOM 6084 O PRO A 387 39.733 −25.847 −28.657 1.00 0.00 O
ATOM 6085 CB PRO A 387 36.888 −24.833 −29.541 1.00 0.00 C
ATOM 6086 CG PRO A 387 36.346 −24.948 −30.961 1.00 0.00 C
ATOM 6087 CD PRO A 387 37.602 −25.271 −31.766 1.00 0.00 C
ATOM 6088 HA PRO A 387 38.233 −23.158 −29.722 1.00 0.00 H
ATOM 6089 HB2 PRO A 387 36.965 −25.835 −29.116 1.00 0.00 H
ATOM 6090 HB3 PRO A 387 36.261 −24.192 −28.921 1.00 0.00 H
ATOM 6091 HG2 PRO A 387 35.601 −25.739 −31.047 1.00 0.00 H

ATOM	6092	HG3	PRO	A	387	35.935	−23.990	−31.280	1.00	0.00	H
ATOM	6093	HD2	PRO	A	387	37.490	−24.875	−32.771	1.00	0.00	H
ATOM	6094	HD3	PRO	A	387	37.763	−26.349	−31.788	1.00	0.00	H
ATOM	6095	N	LYS	A	388	39.444	−23.868	−27.616	1.00	0.00	N
ATOM	6096	CA	LYS	A	388	40.324	−24.111	−26.462	1.00	0.00	C
ATOM	6097	C	LYS	A	388	39.483	−24.012	−25.178	1.00	0.00	C
ATOM	6098	O	LYS	A	388	38.647	−23.117	−25.047	1.00	0.00	O
ATOM	6099	CB	LYS	A	388	41.544	−23.149	−26.504	1.00	0.00	C
ATOM	6100	CG	LYS	A	388	42.324	−23.218	−27.841	1.00	0.00	C
ATOM	6101	CD	LYS	A	388	43.554	−22.292	−27.975	1.00	0.00	C
ATOM	6102	CE	LYS	A	388	43.984	−22.263	−29.457	1.00	0.00	C
ATOM	6103	NZ	LYS	A	388	45.117	−21.350	−29.754	1.00	0.00	N
ATOM	6104	H	LYS	A	388	38.938	−22.985	−27.639	1.00	0.00	H
ATOM	6105	HA	LYS	A	388	40.714	−25.127	−26.516	1.00	0.00	H
ATOM	6106	HB2	LYS	A	388	41.196	−22.129	−26.348	1.00	0.00	H
ATOM	6107	HB3	LYS	A	388	42.221	−23.400	−25.689	1.00	0.00	H
ATOM	6108	HG2	LYS	A	388	42.645	−24.247	−28.010	1.00	0.00	H
ATOM	6109	HG3	LYS	A	388	41.638	−22.950	−28.641	1.00	0.00	H
ATOM	6110	HD2	LYS	A	388	43.300	−21.281	−27.653	1.00	0.00	H
ATOM	6111	HD3	LYS	A	388	44.371	−22.662	−27.354	1.00	0.00	H
ATOM	6112	HE2	LYS	A	388	44.259	−23.272	−29.771	1.00	0.00	H
ATOM	6113	HE3	LYS	A	388	43.127	−21.956	−30.065	1.00	0.00	H
ATOM	6114	HZ1	LYS	A	388	45.295	−21.301	−30.761	1.00	0.00	H
ATOM	6115	HZ2	LYS	A	388	44.985	−20.380	−29.459	1.00	0.00	H
ATOM	6116	HZ3	LYS	A	388	46.018	−21.579	−29.348	1.00	0.00	H
ATOM	6117	N	LEU	A	389	39.511	−25.070	−24.364	1.00	0.00	N
ATOM	6118	CA	LEU	A	389	38.758	−25.163	−23.104	1.00	0.00	C
ATOM	6119	C	LEU	A	389	39.538	−24.504	−21.961	1.00	0.00	C
ATOM	6120	O	LEU	A	389	40.651	−24.934	−21.653	1.00	0.00	O
ATOM	6121	CB	LEU	A	389	38.483	−26.636	−22.747	1.00	0.00	C

ATOM 6122 CG LEU A 389 37.370 −27.324 −23.548 1.00 0.00 C
ATOM 6123 CD1 LEU A 389 37.496 −28.842 −23.408 1.00 0.00 C
ATOM 6124 CD2 LEU A 389 35.992 −26.919 −23.017 1.00 0.00 C
ATOM 6125 H LEU A 389 40.160 −25.814 −24.583 1.00 0.00 H
ATOM 6126 HA LEU A 389 37.807 −24.639 −23.206 1.00 0.00 H
ATOM 6127 HB2 LEU A 389 39.411 −27.191 −22.877 1.00 0.00 H
ATOM 6128 HB3 LEU A 389 38.214 −26.691 −21.692 1.00 0.00 H
ATOM 6129 HG LEU A 389 37.455 −27.066 −24.603 1.00 0.00 H
ATOM 6130 HD11 LEU A 389 38.452 −29.176 −23.810 1.00 0.00 H
ATOM 6131 HD12 LEU A 389 37.426 −29.132 −22.358 1.00 0.00 H
ATOM 6132 HD13 LEU A 389 36.696 −29.332 −23.964 1.00 0.00 H
ATOM 6133 HD21 LEU A 389 35.215 −27.446 −23.570 1.00 0.00 H
ATOM 6134 HD22 LEU A 389 35.903 −27.181 −21.962 1.00 0.00 H
ATOM 6135 HD23 LEU A 389 35.843 −25.846 −23.128 1.00 0.00 H
ATOM 6136 N PHE A 390 38.858 −23.665 −21.183 1.00 0.00 N
ATOM 6137 CA PHE A 390 39.403 −23.004 −19.999 1.00 0.00 C
ATOM 6138 C PHE A 390 38.590 −23.365 −18.750 1.00 0.00 C
ATOM 6139 O PHE A 390 37.382 −23.135 −18.698 1.00 0.00 O
ATOM 6140 CB PHE A 390 39.444 −21.485 −20.223 1.00 0.00 C
ATOM 6141 CG PHE A 390 40.315 −21.040 −21.386 1.00 0.00 C
ATOM 6142 CD1 PHE A 390 41.639 −20.616 −21.159 1.00 0.00 C
ATOM 6143 CD2 PHE A 390 39.789 −21.012 −22.692 1.00 0.00 C
ATOM 6144 CE1 PHE A 390 42.425 −20.152 −22.229 1.00 0.00 C
ATOM 6145 CE2 PHE A 390 40.578 −20.555 −23.763 1.00 0.00 C
ATOM 6146 CZ PHE A 390 41.895 −20.119 −23.531 1.00 0.00 C
ATOM 6147 H PHE A 390 37.923 −23.388 −21.468 1.00 0.00 H
ATOM 6148 HA PHE A 390 40.426 −23.336 −19.846 1.00 0.00 H
ATOM 6149 HB2 PHE A 390 39.809 −21.010 −19.311 1.00 0.00 H
ATOM 6150 HB3 PHE A 390 38.428 −21.123 −20.390 1.00 0.00 H
ATOM 6151 HD1 PHE A 390 42.047 −20.614 −20.160 1.00 0.00 H

```
ATOM   6152  HD2 PHE A 390      38.773 −21.329 −22.875  1.00  0.00           H
ATOM   6153  HE1 PHE A 390      43.426 −19.777 −22.055  1.00  0.00           H
ATOM   6154  HE2 PHE A 390      40.164 −20.516 −24.761  1.00  0.00           H
ATOM   6155  HZ  PHE A 390      42.493 −19.729 −24.344  1.00  0.00           H
ATOM   6156  N   ALA A 391      39.261 −23.851 −17.708  1.00  0.00           N
ATOM   6157  CA  ALA A 391      38.685 −24.025 −16.378  1.00  0.00           C
ATOM   6158  C   ALA A 391      38.947 −22.784 −15.510  1.00  0.00           C
ATOM   6159  O   ALA A 391      40.084 −22.312 −15.401  1.00  0.00           O
ATOM   6160  CB  ALA A 391      39.237 −25.317 −15.764  1.00  0.00           C
ATOM   6161  H   ALA A 391      40.267 −23.955 −17.799  1.00  0.00           H
ATOM   6162  HA  ALA A 391      37.605 −24.140 −16.470  1.00  0.00           H
ATOM   6163  HB1 ALA A 391      39.054 −26.155 −16.437  1.00  0.00           H
ATOM   6164  HB2 ALA A 391      40.310 −25.225 −15.590  1.00  0.00           H
ATOM   6165  HB3 ALA A 391      38.730 −25.526 −14.822  1.00  0.00           H
ATOM   6166  N   VAL A 392      37.916 −22.295 −14.817  1.00  0.00           N
ATOM   6167  CA  VAL A 392      37.956 −21.059 −14.023  1.00  0.00           C
ATOM   6168  C   VAL A 392      37.325 −21.284 −12.649  1.00  0.00           C
ATOM   6169  O   VAL A 392      36.105 −21.292 −12.494  1.00  0.00           O
ATOM   6170  CB  VAL A 392      37.279 −19.888 −14.772  1.00  0.00           C
ATOM   6171  CG1 VAL A 392      37.432 −18.581 −13.980  1.00  0.00           C
ATOM   6172  CG2 VAL A 392      37.892 −19.675 −16.162  1.00  0.00           C
ATOM   6173  H   VAL A 392      37.002 −22.710 −14.988  1.00  0.00           H
ATOM   6174  HA  VAL A 392      38.995 −20.775 −13.864  1.00  0.00           H
ATOM   6175  HB  VAL A 392      36.218 −20.097 −14.897  1.00  0.00           H
ATOM   6176 HG11 VAL A 392      36.990 −17.764 −14.551  1.00  0.00           H
ATOM   6177 HG12 VAL A 392      36.904 −18.648 −13.029  1.00  0.00           H
ATOM   6178 HG13 VAL A 392      38.486 −18.362 −13.806  1.00  0.00           H
ATOM   6179 HG21 VAL A 392      37.499 −18.765 −16.611  1.00  0.00           H
ATOM   6180 HG22 VAL A 392      37.633 −20.508 −16.817  1.00  0.00           H
ATOM   6181 HG23 VAL A 392      38.974 −19.599 −16.088  1.00  0.00           H
```

ATOM	6182	N	LYS	A	393	38.160	−21.395	−11.612	1.00	0.00	N
ATOM	6183	CA	LYS	A	393	37.709	−21.398	−10.214	1.00	0.00	C
ATOM	6184	C	LYS	A	393	37.273	−19.984	−9.827	1.00	0.00	C
ATOM	6185	O	LYS	A	393	38.136	−19.117	−9.663	1.00	0.00	O
ATOM	6186	CB	LYS	A	393	38.837	−21.910	−9.306	1.00	0.00	C
ATOM	6187	CG	LYS	A	393	38.355	−22.085	−7.853	1.00	0.00	C
ATOM	6188	CD	LYS	A	393	39.455	−21.895	−6.795	1.00	0.00	C
ATOM	6189	CE	LYS	A	393	40.248	−20.574	−6.872	1.00	0.00	C
ATOM	6190	NZ	LYS	A	393	39.404	−19.388	−7.178	1.00	0.00	N
ATOM	6191	H	LYS	A	393	39.150	−21.439	−11.805	1.00	0.00	H
ATOM	6192	HA	LYS	A	393	36.852	−22.072	−10.117	1.00	0.00	H
ATOM	6193	HB2	LYS	A	393	39.191	−22.870	−9.669	1.00	0.00	H
ATOM	6194	HB3	LYS	A	393	39.675	−21.216	−9.365	1.00	0.00	H
ATOM	6195	HG2	LYS	A	393	37.931	−23.084	−7.748	1.00	0.00	H
ATOM	6196	HG3	LYS	A	393	37.556	−21.382	−7.628	1.00	0.00	H
ATOM	6197	HD2	LYS	A	393	38.983	−21.957	−5.813	1.00	0.00	H
ATOM	6198	HD3	LYS	A	393	40.157	−22.728	−6.869	1.00	0.00	H
ATOM	6199	HE2	LYS	A	393	40.760	−20.434	−5.915	1.00	0.00	H
ATOM	6200	HE3	LYS	A	393	41.017	−20.676	−7.642	1.00	0.00	H
ATOM	6201	HZ1	LYS	A	393	39.901	−18.520	−7.033	1.00	0.00	H
ATOM	6202	HZ2	LYS	A	393	39.105	−19.392	−8.153	1.00	0.00	H
ATOM	6203	HZ3	LYS	A	393	38.535	−19.393	−6.650	1.00	0.00	H
ATOM	6204	N	ILE	A	394	36.023	−19.817	−9.404	1.00	0.00	N
ATOM	6205	CA	ILE	A	394	35.492	−18.542	−8.896	1.00	0.00	C
ATOM	6206	C	ILE	A	394	36.154	−18.149	−7.549	1.00	0.00	C
ATOM	6207	O	ILE	A	394	36.656	−19.019	−6.823	1.00	0.00	O
ATOM	6208	CB	ILE	A	394	33.949	−18.638	−8.842	1.00	0.00	C
ATOM	6209	CG1	ILE	A	394	33.322	−18.816	−10.249	1.00	0.00	C
ATOM	6210	CG2	ILE	A	394	33.250	−17.485	−8.104	1.00	0.00	C
ATOM	6211	CD1	ILE	A	394	33.636	−17.720	−11.281	1.00	0.00	C

ATOM 6212 H ILE A 394 35.406 −20.624 −9.400 1.00 0.00 H
ATOM 6213 HA ILE A 394 35.755 −17.783 −9.626 1.00 0.00 H
ATOM 6214 HB ILE A 394 33.718 −19.534 −8.272 1.00 0.00 H
ATOM 6215 HG12 ILE A 394 33.643 −19.773 −10.665 1.00 0.00 H
ATOM 6216 HG13 ILE A 394 32.238 −18.867 −10.142 1.00 0.00 H
ATOM 6217 HD11 ILE A 394 33.067 −17.913 −12.191 1.00 0.00 H
ATOM 6218 HD12 ILE A 394 34.696 −17.725 −11.536 1.00 0.00 H
ATOM 6219 HD13 ILE A 394 33.355 −16.739 −10.900 1.00 0.00 H
ATOM 6220 HG21 ILE A 394 32.182 −17.693 −8.058 1.00 0.00 H
ATOM 6221 HG22 ILE A 394 33.612 −17.404 −7.081 1.00 0.00 H
ATOM 6222 HG23 ILE A 394 33.394 −16.540 −8.626 1.00 0.00 H
ATOM 6223 N PRO A 395 36.334 −16.849 −7.234 1.00 0.00 N
ATOM 6224 CA PRO A 395 37.075 −16.429 −6.043 1.00 0.00 C
ATOM 6225 C PRO A 395 36.365 −16.782 −4.730 1.00 0.00 C
ATOM 6226 O PRO A 395 35.196 −16.439 −4.533 1.00 0.00 O
ATOM 6227 CB PRO A 395 37.263 −14.919 −6.169 1.00 0.00 C
ATOM 6228 CG PRO A 395 37.105 −14.661 −7.664 1.00 0.00 C
ATOM 6229 CD PRO A 395 36.057 −15.684 −8.064 1.00 0.00 C
ATOM 6230 HA PRO A 395 38.059 −16.897 −6.062 1.00 0.00 H
ATOM 6231 HB2 PRO A 395 38.236 −14.613 −5.790 1.00 0.00 H
ATOM 6232 HB3 PRO A 395 36.474 −14.398 −5.633 1.00 0.00 H
ATOM 6233 HG2 PRO A 395 36.766 −13.649 −7.868 1.00 0.00 H
ATOM 6234 HG3 PRO A 395 38.038 −14.874 −8.185 1.00 0.00 H
ATOM 6235 HD2 PRO A 395 36.141 −15.881 −9.132 1.00 0.00 H
ATOM 6236 HD3 PRO A 395 35.067 −15.294 −7.826 1.00 0.00 H
ATOM 6237 N ALA A 396 37.124 −17.283 −3.752 1.00 0.00 N
ATOM 6238 CA ALA A 396 36.645 −17.460 −2.380 1.00 0.00 C
ATOM 6239 C ALA A 396 36.486 −16.114 −1.646 1.00 0.00 C
ATOM 6240 O ALA A 396 35.415 −15.854 −1.093 1.00 0.00 O
ATOM 6241 CB ALA A 396 37.609 −18.396 −1.641 1.00 0.00 C

ATOM	6242	H	ALA	A	396	38.093	−17.485	−3.943	1.00	0.00	H
ATOM	6243	HA	ALA	A	396	35.666	−17.941	−2.410	1.00	0.00	H
ATOM	6244	HB1	ALA	A	396	37.249	−18.563	−0.624	1.00	0.00	H
ATOM	6245	HB2	ALA	A	396	37.660	−19.358	−2.153	1.00	0.00	H
ATOM	6246	HB3	ALA	A	396	38.608	−17.958	−1.594	1.00	0.00	H
ATOM	6247	N	GLN	A	397	37.465	−15.219	−1.819	1.00	0.00	N
ATOM	6248	CA	GLN	A	397	37.532	−13.847	−1.299	1.00	0.00	C
ATOM	6249	C	GLN	A	397	37.956	−12.869	−2.408	1.00	0.00	C
ATOM	6250	O	GLN	A	397	38.484	−13.291	−3.435	1.00	0.00	O
ATOM	6251	CB	GLN	A	397	38.544	−13.777	−0.135	1.00	0.00	C
ATOM	6252	CG	GLN	A	397	38.166	−14.568	1.130	1.00	0.00	C
ATOM	6253	CD	GLN	A	397	37.017	−13.948	1.924	1.00	0.00	C
ATOM	6254	OE1	GLN	A	397	36.179	−13.225	1.415	1.00	0.00	O
ATOM	6255	NE2	GLN	A	397	36.926	−14.213	3.207	1.00	0.00	N
ATOM	6256	H	GLN	A	397	38.251	−15.493	−2.391	1.00	0.00	H
ATOM	6257	HA	GLN	A	397	36.547	−13.538	−0.949	1.00	0.00	H
ATOM	6258	HB2	GLN	A	397	39.505	−14.149	−0.494	1.00	0.00	H
ATOM	6259	HB3	GLN	A	397	38.690	−12.735	0.154	1.00	0.00	H
ATOM	6260	HG2	GLN	A	397	37.911	−15.596	0.877	1.00	0.00	H
ATOM	6261	HG3	GLN	A	397	39.044	−14.599	1.776	1.00	0.00	H
ATOM	6262	HE21	GLN	A	397	37.627	−14.756	3.675	1.00	0.00	H
ATOM	6263	HE22	GLN	A	397	36.191	−13.739	3.704	1.00	0.00	H
ATOM	6264	N	CYS	A	398	37.773	−11.566	−2.191	1.00	0.00	N
ATOM	6265	CA	CYS	A	398	38.160	−10.496	−3.125	1.00	0.00	C
ATOM	6266	C	CYS	A	398	39.632	−10.045	−2.981	1.00	0.00	C
ATOM	6267	O	CYS	A	398	39.888	−8.853	−2.844	1.00	0.00	O
ATOM	6268	CB	CYS	A	398	37.188	−9.321	−2.957	1.00	0.00	C
ATOM	6269	SG	CYS	A	398	35.435	−9.738	−3.106	1.00	0.00	S
ATOM	6270	H	CYS	A	398	37.355	−11.281	−1.317	1.00	0.00	H
ATOM	6271	HA	CYS	A	398	38.051	−10.871	−4.143	1.00	0.00	H

ATOM	6272	HB2	CYS	A	398	37.353	−8.867	−1.979	1.00	0.00	H
ATOM	6273	HB3	CYS	A	398	37.423	−8.565	−3.709	1.00	0.00	H
ATOM	6274	N	SER	A	399	40.535	−11.001	−2.749	1.00	0.00	N
ATOM	6275	CA	SER	A	399	41.901	−10.772	−2.258	1.00	0.00	C
ATOM	6276	C	SER	A	399	42.910	−10.475	−3.391	1.00	0.00	C
ATOM	6277	O	SER	A	399	42.968	−9.329	−3.838	1.00	0.00	O
ATOM	6278	CB	SER	A	399	42.249	−11.915	−1.295	1.00	0.00	C
ATOM	6279	OG	SER	A	399	43.590	−11.928	−0.882	1.00	0.00	O
ATOM	6280	H	SER	A	399	40.258	−11.957	−2.913	1.00	0.00	H
ATOM	6281	HA	SER	A	399	41.897	−9.868	−1.649	1.00	0.00	H
ATOM	6282	HB2	SER	A	399	41.618	−11.821	−0.410	1.00	0.00	H
ATOM	6283	HB3	SER	A	399	42.020	−12.870	−1.769	1.00	0.00	H
ATOM	6284	HG	SER	A	399	44.012	−12.649	−1.406	1.00	0.00	H
ATOM	6285	N	GLU	A	400	43.592	−11.479	−3.961	1.00	0.00	N
ATOM	6286	CA	GLU	A	400	44.658	−11.334	−4.988	1.00	0.00	C
ATOM	6287	C	GLU	A	400	44.333	−11.947	−6.357	1.00	0.00	C
ATOM	6288	O	GLU	A	400	44.817	−11.410	−7.383	1.00	0.00	O
ATOM	6289	CB	GLU	A	400	46.039	−11.811	−4.466	1.00	0.00	C
ATOM	6290	CG	GLU	A	400	46.127	−12.750	−3.244	1.00	0.00	C
ATOM	6291	CD	GLU	A	400	45.187	−13.971	−3.281	1.00	0.00	C
ATOM	6292	OE1	GLU	A	400	45.599	−15.037	−3.796	1.00	0.00	O
ATOM	6293	OE2	GLU	A	400	44.158	−13.899	−2.558	1.00	0.00	O
ATOM	6294	OXT	GLU	A	400	43.919	−13.121	−6.402	1.00	0.00	O
ATOM	6295	H	GLU	A	400	43.493	−12.407	−3.555	1.00	0.00	H
ATOM	6296	HA	GLU	A	400	44.775	−10.274	−5.207	1.00	0.00	H
ATOM	6297	HB2	GLU	A	400	46.570	−12.292	−5.288	1.00	0.00	H
ATOM	6298	HB3	GLU	A	400	46.613	−10.916	−4.215	1.00	0.00	H
ATOM	6299	HG2	GLU	A	400	45.912	−12.157	−2.351	1.00	0.00	H
ATOM	6300	HG3	GLU	A	400	47.163	−13.084	−3.144	1.00	0.00	H
ATOM	6301	HXT	GLU	A	400	43.075	−13.195	−5.886	1.00	0.00	H

TER 6302 HXT GLU A 400
HETATM 6303 C1 LIG 1 27.562 −18.664 −34.671 1.00 0.00 C
HETATM 6304 C2 LIG 1 28.067 −17.871 −35.853 1.00 0.00 C
HETATM 6305 N3 LIG 1 28.956 −14.033 −37.234 1.00 0.00 N
HETATM 6306 N4 LIG 1 29.256 −17.133 −35.653 1.00 0.00 N
HETATM 6307 C5 LIG 1 29.884 −14.850 −36.551 1.00 0.00 C
HETATM 6308 C6 LIG 1 28.754 −12.574 −37.028 1.00 0.00 C
HETATM 6309 C7 LIG 1 26.432 −19.658 −34.673 1.00 0.00 C
HETATM 6310 C8 LIG 1 29.897 −16.384 −36.767 1.00 0.00 C
HETATM 6311 C9 LIG 1 27.828 −14.768 −37.828 1.00 0.00 C
HETATM 6312 N10 LIG 1 26.507 −12.708 −38.326 1.00 0.00 N
HETATM 6313 C11 LIG 1 28.225 −18.552 −33.502 1.00 0.00 C
HETATM 6314 N12 LIG 1 26.305 −20.531 −33.621 1.00 0.00 N
HETATM 6315 C13 LIG 1 27.711 −11.909 −38.006 1.00 0.00 C
HETATM 6316 C14 LIG 1 29.872 −17.065 −34.413 1.00 0.00 C
HETATM 6317 O15 LIG 1 27.467 −17.923 −36.927 1.00 0.00 O
HETATM 6318 C16 LIG 1 29.388 −17.720 −33.357 1.00 0.00 C
HETATM 6319 O17 LIG 1 30.695 −14.351 −35.760 1.00 0.00 O
HETATM 6320 C18 LIG 1 30.025 −11.739 −37.164 1.00 0.00 C
HETATM 6321 O19 LIG 1 25.621 −19.677 −35.604 1.00 0.00 O
HETATM 6322 C20 LIG 1 26.985 −14.000 −38.855 1.00 0.00 C
HETATM 6323 C21 LIG 1 25.160 −21.418 −33.470 1.00 0.00 C
HETATM 6324 C22 LIG 1 25.291 −22.413 −32.343 1.00 0.00 C
HETATM 6325 C23 LIG 1 25.664 −12.019 −39.306 1.00 0.00 C
HETATM 6326 C24 LIG 1 30.383 −10.850 −36.135 1.00 0.00 C
HETATM 6327 C25 LIG 1 30.814 −11.777 −38.325 1.00 0.00 C
HETATM 6328 C26 LIG 1 25.012 −22.032 −31.021 1.00 0.00 C
HETATM 6329 C27 LIG 1 25.773 −23.711 −32.582 1.00 0.00 C
HETATM 6330 C28 LIG 1 31.518 −10.047 −36.252 1.00 0.00 C
HETATM 6331 C29 LIG 1 31.956 −10.983 −38.432 1.00 0.00 C

HETATM	6332	C30	LIG	1	25.223	−22.920	−29.962	1.00	0.00	C
HETATM	6333	C31	LIG	1	25.986	−24.599	−31.524	1.00	0.00	C
HETATM	6334	C32	LIG	1	25.704	−24.203	−30.218	1.00	0.00	C
HETATM	6335	C33	LIG	1	32.310	−10.122	−37.395	1.00	0.00	C
HETATM	6336	H1	LIG	1	28.372	−12.489	−36.001	1.00	0.00	H
HETATM	6337	H2	LIG	1	29.371	−16.599	−37.701	1.00	0.00	H
HETATM	6338	H3	LIG	1	30.919	−16.763	−36.836	1.00	0.00	H
HETATM	6339	H4	LIG	1	28.195	−15.686	−38.300	1.00	0.00	H
HETATM	6340	H5	LIG	1	27.187	−15.077	−36.991	1.00	0.00	H
HETATM	6341	H6	LIG	1	27.920	−19.115	−32.622	1.00	0.00	H
HETATM	6342	H7	LIG	1	26.992	−20.479	−32.873	1.00	0.00	H
HETATM	6343	H8	LIG	1	28.218	−11.656	−38.950	1.00	0.00	H
HETATM	6344	H9	LIG	1	27.395	−10.953	−37.566	1.00	0.00	H
HETATM	6345	H10	LIG	1	30.763	−16.448	−34.328	1.00	0.00	H
HETATM	6346	H11	LIG	1	29.873	−17.660	−32.387	1.00	0.00	H
HETATM	6347	H12	LIG	1	26.126	−14.626	−39.126	1.00	0.00	H
HETATM	6348	H13	LIG	1	27.577	−13.848	−39.767	1.00	0.00	H
HETATM	6349	H14	LIG	1	24.290	−20.772	−33.301	1.00	0.00	H
HETATM	6350	H15	LIG	1	24.972	−21.950	−34.410	1.00	0.00	H
HETATM	6351	H16	LIG	1	24.769	−12.613	−39.523	1.00	0.00	H
HETATM	6352	H17	LIG	1	26.189	−11.829	−40.249	1.00	0.00	H
HETATM	6353	H18	LIG	1	25.318	−11.060	−38.904	1.00	0.00	H
HETATM	6354	H19	LIG	1	29.772	−10.768	−35.237	1.00	0.00	H
HETATM	6355	H20	LIG	1	30.555	−12.439	−39.150	1.00	0.00	H
HETATM	6356	H21	LIG	1	24.648	−21.027	−30.813	1.00	0.00	H
HETATM	6357	H22	LIG	1	26.006	−24.028	−33.596	1.00	0.00	H
HETATM	6358	H23	LIG	1	31.783	−9.359	−35.452	1.00	0.00	H
HETATM	6359	H24	LIG	1	32.576	−11.043	−39.324	1.00	0.00	H
HETATM	6360	H25	LIG	1	25.032	−22.610	−28.937	1.00	0.00	H
HETATM	6361	H26	LIG	1	26.391	−25.593	−31.706	1.00	0.00	H

```
HETATM 6362  H27 LIG     1      25.879 −24.891 −29.393  1.00  0.00           H
HETATM 6363  H28 LIG     1      33.203  −9.509 −37.481  1.00  0.00           H
TER    6364  H28 LIG     1
CONECT   27 6269
CONECT  335  693
CONECT  693  335
CONECT 1346 1552
CONECT 1552 1346
CONECT 2844 3048
CONECT 2923 4674
CONECT 3048 2844
CONECT 4576 4729
CONECT 4674 2923
CONECT 4729 4576
CONECT 5685 5841
CONECT 5841 5685
CONECT 6269   27
CONECT 6303 6304 6309 6313
CONECT 6304 6303 6306 6317
CONECT 6305 6307 6308 6311
CONECT 6306 6304 6310 6316
CONECT 6307 6305 6310 6319
CONECT 6308 6305 6315 6320 6336
CONECT 6309 6303 6314 6321
CONECT 6310 6306 6307 6337 6338
CONECT 6311 6305 6322 6339 6340
CONECT 6312 6315 6322 6325
CONECT 6313 6303 6318 6341
CONECT 6314 6309 6323 6342
CONECT 6315 6308 6312 6343 6344
```

CONECT 6316 6306 6318 6345

CONECT 6317 6304

CONECT 6318 6313 6316 6346

CONECT 6319 6307

CONECT 6320 6308 6326 6327

CONECT 6321 6309

CONECT 6322 6311 6312 6347 6348

CONECT 6323 6314 6324 6349 6350

CONECT 6324 6323 6328 6329

CONECT 6325 6312 6351 6352 6353

CONECT 6326 6320 6330 6354

CONECT 6327 6320 6331 6355

CONECT 6328 6324 6332 6356

CONECT 6329 6324 6333 6357

CONECT 6330 6326 6335 6358

CONECT 6331 6327 6335 6359

CONECT 6332 6328 6334 6360

CONECT 6333 6329 6334 6361

CONECT 6334 6332 6333 6362

CONECT 6335 6330 6331 6363

CONECT 6336 6308

CONECT 6337 6310

CONECT 6338 6310

CONECT 6339 6311

CONECT 6340 6311

CONECT 6341 6313

CONECT 6342 6314

CONECT 6343 6315

CONECT 6344 6315

CONECT 6345 6316

CONECT 6346 6318

CONECT 6347 6322

CONECT 6348 6322

CONECT 6349 6323

CONECT 6350 6323

CONECT 6351 6325

CONECT 6352 6325

CONECT 6353 6325

CONECT 6354 6326

CONECT 6355 6327

CONECT 6356 6328

CONECT 6357 6329

CONECT 6358 6330

CONECT 6359 6331

CONECT 6360 6332

CONECT 6361 6333

CONECT 6362 6334

CONECT 6363 6335

MASTER 0 0 0 0 0 0 0 3 6362 2 75 0

END

Top2−coordinates

HEADER CSD ENTRY

CRYST1 1.0000 1.0000 1.0000 90.00 90.00 90.00

SCALE1 1.000000 0.000000 0.000000 0.000000

SCALE2 0.000000 1.000000 0.000000 0.000000

SCALE3 0.000000 0.000000 1.000000 0.000000

ATOM 1 N ILE A 1 32.653 −16.268 −1.319 1.00 0.00 N

ATOM 2 CA ILE A 1 32.633 −15.941 −2.763 1.00 0.00 C

ATOM 3 C ILE A 1 32.837 −14.441 −2.909 1.00 0.00 C

ATOM	4	O	ILE	A	1	32.290	−13.705	−2.093	1.00	0.00	O
ATOM	5	CB	ILE	A	1	31.347	−16.438	−3.468	1.00	0.00	C
ATOM	6	CG1	ILE	A	1	31.341	−16.009	−4.953	1.00	0.00	C
ATOM	7	CG2	ILE	A	1	30.059	−15.978	−2.757	1.00	0.00	C
ATOM	8	CD1	ILE	A	1	30.325	−16.768	−5.817	1.00	0.00	C
ATOM	9	HA	ILE	A	1	33.476	−16.435	−3.236	1.00	0.00	H
ATOM	10	HB	ILE	A	1	31.370	−17.529	−3.437	1.00	0.00	H
ATOM	11	HG12	ILE	A	1	31.130	−14.941	−5.030	1.00	0.00	H
ATOM	12	HG13	ILE	A	1	32.330	−16.191	−5.375	1.00	0.00	H
ATOM	13	HD11	ILE	A	1	30.420	−16.435	−6.849	1.00	0.00	H
ATOM	14	HD12	ILE	A	1	30.514	−17.841	−5.770	1.00	0.00	H
ATOM	15	HD13	ILE	A	1	29.306	−16.561	−5.490	1.00	0.00	H
ATOM	16	H1	ILE	A	1	32.449	−17.244	−1.163	1.00	0.00	H
ATOM	17	H2	ILE	A	1	31.970	−15.687	−0.848	1.00	0.00	H
ATOM	18	H3	ILE	A	1	33.568	−16.041	−0.940	1.00	0.00	H
ATOM	19	HG21	ILE	A	1	29.185	−16.396	−3.259	1.00	0.00	H
ATOM	20	HG22	ILE	A	1	29.968	−14.891	−2.784	1.00	0.00	H
ATOM	21	HG23	ILE	A	1	30.021	−16.320	−1.724	1.00	0.00	H
ATOM	22	N	CYS	A	2	33.675	−13.977	−3.840	1.00	0.00	N
ATOM	23	CA	CYS	A	2	33.809	−12.535	−4.055	1.00	0.00	C
ATOM	24	C	CYS	A	2	32.574	−11.948	−4.761	1.00	0.00	C
ATOM	25	O	CYS	A	2	32.343	−12.226	−5.937	1.00	0.00	O
ATOM	26	CB	CYS	A	2	35.092	−12.191	−4.815	1.00	0.00	C
ATOM	27	SG	CYS	A	2	35.277	−10.404	−5.017	1.00	0.00	S
ATOM	28	H	CYS	A	2	34.138	−14.619	−4.473	1.00	0.00	H
ATOM	29	HA	CYS	A	2	33.888	−12.061	−3.076	1.00	0.00	H
ATOM	30	HB2	CYS	A	2	35.060	−12.645	−5.806	1.00	0.00	H
ATOM	31	HB3	CYS	A	2	35.956	−12.576	−4.276	1.00	0.00	H
ATOM	32	N	LEU	A	3	31.841	−11.084	−4.056	1.00	0.00	N
ATOM	33	CA	LEU	A	3	30.699	−10.317	−4.576	1.00	0.00	C

ATOM 34 C LEU A 3 31.032 −8.834 −4.838 1.00 0.00 C
ATOM 35 O LEU A 3 30.234 −8.131 −5.451 1.00 0.00 O
ATOM 36 CB LEU A 3 29.536 −10.434 −3.572 1.00 0.00 C
ATOM 37 CG LEU A 3 29.029 −11.868 −3.324 1.00 0.00 C
ATOM 38 CD1 LEU A 3 28.068 −11.866 −2.138 1.00 0.00 C
ATOM 39 CD2 LEU A 3 28.323 −12.447 −4.551 1.00 0.00 C
ATOM 40 H LEU A 3 32.062 −10.971 −3.078 1.00 0.00 H
ATOM 41 HA LEU A 3 30.378 −10.733 −5.533 1.00 0.00 H
ATOM 42 HB2 LEU A 3 29.865 −10.009 −2.622 1.00 0.00 H
ATOM 43 HB3 LEU A 3 28.699 −9.829 −3.927 1.00 0.00 H
ATOM 44 HG LEU A 3 29.866 −12.517 −3.068 1.00 0.00 H
ATOM 45 HD11 LEU A 3 27.641 −12.859 −1.999 1.00 0.00 H
ATOM 46 HD12 LEU A 3 27.266 −11.150 −2.317 1.00 0.00 H
ATOM 47 HD13 LEU A 3 28.600 −11.576 −1.232 1.00 0.00 H
ATOM 48 HD21 LEU A 3 29.023 −12.547 −5.379 1.00 0.00 H
ATOM 49 HD22 LEU A 3 27.501 −11.795 −4.847 1.00 0.00 H
ATOM 50 HD23 LEU A 3 27.917 −13.432 −4.318 1.00 0.00 H
ATOM 51 N GLN A 4 32.174 −8.356 −4.332 1.00 0.00 N
ATOM 52 CA GLN A 4 32.625 −6.966 −4.438 1.00 0.00 C
ATOM 53 C GLN A 4 33.318 −6.704 −5.783 1.00 0.00 C
ATOM 54 O GLN A 4 34.098 −7.532 −6.258 1.00 0.00 O
ATOM 55 CB GLN A 4 33.568 −6.659 −3.256 1.00 0.00 C
ATOM 56 CG GLN A 4 33.907 −5.174 −3.041 1.00 0.00 C
ATOM 57 CD GLN A 4 32.715 −4.319 −2.625 1.00 0.00 C
ATOM 58 OE1 GLN A 4 31.658 −4.797 −2.257 1.00 0.00 O
ATOM 59 NE2 GLN A 4 32.833 −3.013 −2.682 1.00 0.00 N
ATOM 60 H GLN A 4 32.809 −9.008 −3.905 1.00 0.00 H
ATOM 61 HA GLN A 4 31.749 −6.318 −4.366 1.00 0.00 H
ATOM 62 HB2 GLN A 4 33.132 −7.049 −2.334 1.00 0.00 H
ATOM 63 HB3 GLN A 4 34.509 −7.178 −3.425 1.00 0.00 H

ATOM 64 HG2 GLN A 4 34.659 −5.101 −2.255 1.00 0.00 H

ATOM 65 HG3 GLN A 4 34.345 −4.761 −3.945 1.00 0.00 H

ATOM 66 HE21 GLN A 4 33.685 −2.587 −2.999 1.00 0.00 H

ATOM 67 HE22 GLN A 4 32.019 −2.489 −2.414 1.00 0.00 H

ATOM 68 N LYS A 5 33.179 −5.485 −6.311 1.00 0.00 N

ATOM 69 CA LYS A 5 33.993 −4.990 −7.427 1.00 0.00 C

ATOM 70 C LYS A 5 35.463 −4.805 −7.021 1.00 0.00 C

ATOM 71 O LYS A 5 35.744 −4.157 −6.016 1.00 0.00 O

ATOM 72 CB LYS A 5 33.343 −3.703 −7.956 1.00 0.00 C

ATOM 73 CG LYS A 5 34.110 −3.120 −9.144 1.00 0.00 C

ATOM 74 CD LYS A 5 33.252 −2.138 −9.952 1.00 0.00 C

ATOM 75 CE LYS A 5 34.025 −1.589 −11.158 1.00 0.00 C

ATOM 76 NZ LYS A 5 34.494 −2.678 −12.052 1.00 0.00 N

ATOM 77 H LYS A 5 32.506 −4.854 −5.897 1.00 0.00 H

ATOM 78 HA LYS A 5 33.964 −5.741 −8.213 1.00 0.00 H

ATOM 79 HB2 LYS A 5 32.329 −3.946 −8.275 1.00 0.00 H

ATOM 80 HB3 LYS A 5 33.285 −2.956 −7.162 1.00 0.00 H

ATOM 81 HG2 LYS A 5 35.010 −2.616 −8.788 1.00 0.00 H

ATOM 82 HG3 LYS A 5 34.402 −3.937 −9.795 1.00 0.00 H

ATOM 83 HD2 LYS A 5 32.350 −2.647 −10.299 1.00 0.00 H

ATOM 84 HD3 LYS A 5 32.950 −1.310 −9.308 1.00 0.00 H

ATOM 85 HE2 LYS A 5 34.877 −1.007 −10.794 1.00 0.00 H

ATOM 86 HE3 LYS A 5 33.363 −0.913 −11.708 1.00 0.00 H

ATOM 87 HZ1 LYS A 5 34.932 −2.323 −12.889 1.00 0.00 H

ATOM 88 HZ2 LYS A 5 35.137 −3.292 −11.566 1.00 0.00 H

ATOM 89 HZ3 LYS A 5 33.708 −3.272 −12.317 1.00 0.00 H

ATOM 90 N THR A 6 36.403 −5.279 −7.844 1.00 0.00 N

ATOM 91 CA THR A 6 37.856 −5.126 −7.630 1.00 0.00 C

ATOM 92 C THR A 6 38.647 −5.237 −8.944 1.00 0.00 C

ATOM 93 O THR A 6 38.111 −5.652 −9.971 1.00 0.00 O

ATOM 94 CB THR A 6 38.376 −6.122 −6.569 1.00 0.00 C
ATOM 95 OG1 THR A 6 39.739 −5.865 −6.316 1.00 0.00 O
ATOM 96 CG2 THR A 6 38.270 −7.592 −6.974 1.00 0.00 C
ATOM 97 H THR A 6 36.108 −5.742 −8.700 1.00 0.00 H
ATOM 98 HA THR A 6 38.038 −4.126 −7.236 1.00 0.00 H
ATOM 99 HB THR A 6 37.819 −5.975 −5.643 1.00 0.00 H
ATOM 100 HG1 THR A 6 40.069 −6.475 −5.641 1.00 0.00 H
ATOM 101 HG21 THR A 6 38.657 −8.210 −6.165 1.00 0.00 H
ATOM 102 HG22 THR A 6 37.224 −7.854 −7.143 1.00 0.00 H
ATOM 103 HG23 THR A 6 38.843 −7.785 −7.881 1.00 0.00 H
ATOM 104 N THR A 7 39.919 −4.835 −8.924 1.00 0.00 N
ATOM 105 CA THR A 7 40.904 −5.065 −9.996 1.00 0.00 C
ATOM 106 C THR A 7 41.967 −6.108 −9.622 1.00 0.00 C
ATOM 107 O THR A 7 42.910 −6.306 −10.383 1.00 0.00 O
ATOM 108 CB THR A 7 41.566 −3.750 −10.439 1.00 0.00 C
ATOM 109 OG1 THR A 7 42.236 −3.148 −9.357 1.00 0.00 O
ATOM 110 CG2 THR A 7 40.544 −2.746 −10.975 1.00 0.00 C
ATOM 111 H THR A 7 40.283 −4.513 −8.035 1.00 0.00 H
ATOM 112 HA THR A 7 40.392 −5.474 −10.866 1.00 0.00 H
ATOM 113 HB THR A 7 42.285 −3.957 −11.233 1.00 0.00 H
ATOM 114 HG21 THR A 7 41.069 −1.869 −11.353 1.00 0.00 H
ATOM 115 HG22 THR A 7 39.862 −2.438 −10.183 1.00 0.00 H
ATOM 116 HG23 THR A 7 39.978 −3.200 −11.789 1.00 0.00 H
ATOM 117 HG1 THR A 7 43.081 −3.597 −9.254 1.00 0.00 H
ATOM 118 N SER A 8 41.831 −6.792 −8.479 1.00 0.00 N
ATOM 119 CA SER A 8 42.648 −7.967 −8.139 1.00 0.00 C
ATOM 120 C SER A 8 42.492 −9.069 −9.196 1.00 0.00 C
ATOM 121 O SER A 8 41.383 −9.338 −9.659 1.00 0.00 O
ATOM 122 CB SER A 8 42.242 −8.541 −6.780 1.00 0.00 C
ATOM 123 OG SER A 8 42.255 −7.570 −5.754 1.00 0.00 O

ATOM	124	H	SER	A	8	41.087	−6.545	−7.842	1.00	0.00	H
ATOM	125	HA	SER	A	8	43.694	−7.668	−8.092	1.00	0.00	H
ATOM	126	HB2	SER	A	8	41.236	−8.955	−6.849	1.00	0.00	H
ATOM	127	HB3	SER	A	8	42.938	−9.339	−6.520	1.00	0.00	H
ATOM	128	HG	SER	A	8	42.544	−8.039	−4.942	1.00	0.00	H
ATOM	129	N	THR	A	9	43.545	−9.851	−9.436	1.00	0.00	N
ATOM	130	CA	THR	A	9	43.583	−10.945	−10.433	1.00	0.00	C
ATOM	131	C	THR	A	9	42.946	−12.254	−9.931	1.00	0.00	C
ATOM	132	O	THR	A	9	43.398	−13.354	−10.246	1.00	0.00	O
ATOM	133	CB	THR	A	9	45.011	−11.166	−10.966	1.00	0.00	C
ATOM	134	OG1	THR	A	9	45.889	−11.607	−9.943	1.00	0.00	O
ATOM	135	CG2	THR	A	9	45.595	−9.902	−11.599	1.00	0.00	C
ATOM	136	H	THR	A	9	44.359	−9.740	−8.843	1.00	0.00	H
ATOM	137	HA	THR	A	9	42.977	−10.631	−11.285	1.00	0.00	H
ATOM	138	HB	THR	A	9	44.970	−11.928	−11.748	1.00	0.00	H
ATOM	139	HG21	THR	A	9	46.543	−10.143	−12.081	1.00	0.00	H
ATOM	140	HG22	THR	A	9	45.764	−9.131	−10.848	1.00	0.00	H
ATOM	141	HG23	THR	A	9	44.910	−9.517	−12.355	1.00	0.00	H
ATOM	142	HG1	THR	A	9	45.581	−11.264	−9.081	1.00	0.00	H
ATOM	143	N	ILE	A	10	41.898	−12.159	−9.110	1.00	0.00	N
ATOM	144	CA	ILE	A	10	41.276	−13.273	−8.367	1.00	0.00	C
ATOM	145	C	ILE	A	10	40.673	−14.378	−9.256	1.00	0.00	C
ATOM	146	O	ILE	A	10	40.501	−15.519	−8.812	1.00	0.00	O
ATOM	147	CB	ILE	A	10	40.227	−12.716	−7.379	1.00	0.00	C
ATOM	148	CG1	ILE	A	10	39.160	−11.831	−8.065	1.00	0.00	C
ATOM	149	CG2	ILE	A	10	40.916	−12.004	−6.207	1.00	0.00	C
ATOM	150	CD1	ILE	A	10	38.103	−11.279	−7.101	1.00	0.00	C
ATOM	151	H	ILE	A	10	41.584	−11.220	−8.901	1.00	0.00	H
ATOM	152	HA	ILE	A	10	42.056	−13.761	−7.783	1.00	0.00	H
ATOM	153	HB	ILE	A	10	39.721	−13.573	−6.948	1.00	0.00	H

ATOM 154 HG12 ILE A 10 39.630 −10.980 −8.555 1.00 0.00 H
ATOM 155 HG13 ILE A 10 38.654 −12.417 −8.832 1.00 0.00 H
ATOM 156 HG21 ILE A 10 41.531 −11.184 −6.567 1.00 0.00 H
ATOM 157 HG22 ILE A 10 41.560 −12.711 −5.682 1.00 0.00 H
ATOM 158 HG23 ILE A 10 40.194 −11.632 −5.487 1.00 0.00 H
ATOM 159 HD11 ILE A 10 37.733 −12.067 −6.449 1.00 0.00 H
ATOM 160 HD12 ILE A 10 37.276 −10.863 −7.670 1.00 0.00 H
ATOM 161 HD13 ILE A 10 38.533 −10.487 −6.492 1.00 0.00 H
ATOM 162 N LEU A 11 40.408 −14.076 −10.529 1.00 0.00 N
ATOM 163 CA LEU A 11 40.169 −15.065 −11.579 1.00 0.00 C
ATOM 164 C LEU A 11 41.513 −15.603 −12.095 1.00 0.00 C
ATOM 165 O LEU A 11 42.148 −14.989 −12.945 1.00 0.00 O
ATOM 166 CB LEU A 11 39.368 −14.411 −12.718 1.00 0.00 C
ATOM 167 CG LEU A 11 37.929 −14.021 −12.357 1.00 0.00 C
ATOM 168 CD1 LEU A 11 37.406 −13.062 −13.424 1.00 0.00 C
ATOM 169 CD2 LEU A 11 37.013 −15.243 −12.273 1.00 0.00 C
ATOM 170 H LEU A 11 40.670 −13.148 −10.830 1.00 0.00 H
ATOM 171 HA LEU A 11 39.597 −15.901 −11.178 1.00 0.00 H
ATOM 172 HB2 LEU A 11 39.904 −13.514 −13.031 1.00 0.00 H
ATOM 173 HB3 LEU A 11 39.339 −15.094 −13.567 1.00 0.00 H
ATOM 174 HG LEU A 11 37.916 −13.511 −11.394 1.00 0.00 H
ATOM 175 HD21 LEU A 11 37.370 −15.933 −11.511 1.00 0.00 H
ATOM 176 HD22 LEU A 11 36.005 −14.927 −12.014 1.00 0.00 H
ATOM 177 HD23 LEU A 11 36.986 −15.747 −13.238 1.00 0.00 H
ATOM 178 HD11 LEU A 11 37.965 −12.130 −13.364 1.00 0.00 H
ATOM 179 HD12 LEU A 11 37.551 −13.487 −14.415 1.00 0.00 H
ATOM 180 HD13 LEU A 11 36.350 −12.847 −13.270 1.00 0.00 H
ATOM 181 N LYS A 12 41.922 −16.783 −11.613 1.00 0.00 N
ATOM 182 CA LYS A 12 43.185 −17.445 −11.998 1.00 0.00 C
ATOM 183 C LYS A 12 42.898 −18.715 −12.827 1.00 0.00 C

ATOM	184	O	LYS	A	12	42.657	−19.768	−12.220	1.00	0.00	O
ATOM	185	CB	LYS	A	12	44.061	−17.686	−10.749	1.00	0.00	C
ATOM	186	CG	LYS	A	12	44.321	−16.380	−9.971	1.00	0.00	C
ATOM	187	CD	LYS	A	12	45.404	−16.508	−8.886	1.00	0.00	C
ATOM	188	CE	LYS	A	12	45.336	−15.373	−7.849	1.00	0.00	C
ATOM	189	NZ	LYS	A	12	45.434	−14.022	−8.445	1.00	0.00	N
ATOM	190	H	LYS	A	12	41.368	−17.191	−10.876	1.00	0.00	H
ATOM	191	HA	LYS	A	12	43.757	−16.763	−12.628	1.00	0.00	H
ATOM	192	HB2	LYS	A	12	43.575	−18.404	−10.086	1.00	0.00	H
ATOM	193	HB3	LYS	A	12	45.016	−18.102	−11.074	1.00	0.00	H
ATOM	194	HG2	LYS	A	12	44.634	−15.610	−10.677	1.00	0.00	H
ATOM	195	HG3	LYS	A	12	43.389	−16.064	−9.498	1.00	0.00	H
ATOM	196	HD2	LYS	A	12	45.268	−17.449	−8.353	1.00	0.00	H
ATOM	197	HD3	LYS	A	12	46.388	−16.518	−9.356	1.00	0.00	H
ATOM	198	HE2	LYS	A	12	44.382	−15.433	−7.315	1.00	0.00	H
ATOM	199	HE3	LYS	A	12	46.121	−15.499	−7.097	1.00	0.00	H
ATOM	200	HZ1	LYS	A	12	45.123	−13.338	−7.743	1.00	0.00	H
ATOM	201	HZ2	LYS	A	12	46.354	−13.750	−8.749	1.00	0.00	H
ATOM	202	HZ3	LYS	A	12	44.778	−13.890	−9.207	1.00	0.00	H
ATOM	203	N	PRO	A	13	42.721	−18.603	−14.161	1.00	0.00	N
ATOM	204	CA	PRO	A	13	42.212	−19.673	−15.031	1.00	0.00	C
ATOM	205	C	PRO	A	13	43.206	−20.839	−15.209	1.00	0.00	C
ATOM	206	O	PRO	A	13	44.265	−20.883	−14.575	1.00	0.00	O
ATOM	207	CB	PRO	A	13	41.911	−18.964	−16.367	1.00	0.00	C
ATOM	208	CG	PRO	A	13	42.965	−17.865	−16.425	1.00	0.00	C
ATOM	209	CD	PRO	A	13	43.020	−17.421	−14.967	1.00	0.00	C
ATOM	210	HA	PRO	A	13	41.282	−20.076	−14.631	1.00	0.00	H
ATOM	211	HB2	PRO	A	13	40.928	−18.497	−16.321	1.00	0.00	H
ATOM	212	HB3	PRO	A	13	41.968	−19.622	−17.235	1.00	0.00	H
ATOM	213	HG2	PRO	A	13	43.928	−18.283	−16.726	1.00	0.00	H

ATOM 214 HG3 PRO A 13 42.672 −17.049 −17.088 1.00 0.00 H
ATOM 215 HD2 PRO A 13 42.255 −16.665 −14.798 1.00 0.00 H
ATOM 216 HD3 PRO A 13 44.008 −17.015 −14.745 1.00 0.00 H
ATOM 217 N ARG A 14 42.865 −21.803 −16.072 1.00 0.00 N
ATOM 218 CA ARG A 14 43.782 −22.766 −16.709 1.00 0.00 C
ATOM 219 C ARG A 14 43.227 −23.218 −18.056 1.00 0.00 C
ATOM 220 O ARG A 14 42.066 −23.608 −18.128 1.00 0.00 O
ATOM 221 CB ARG A 14 44.005 −23.992 −15.797 1.00 0.00 C
ATOM 222 CG ARG A 14 45.386 −23.988 −15.114 1.00 0.00 C
ATOM 223 CD ARG A 14 45.287 −24.303 −13.619 1.00 0.00 C
ATOM 224 NE ARG A 14 44.662 −23.182 −12.895 1.00 0.00 N
ATOM 225 CZ ARG A 14 44.369 −23.138 −11.612 1.00 0.00 C
ATOM 226 NH1 ARG A 14 44.609 −24.144 −10.816 1.00 0.00 N
ATOM 227 NH2 ARG A 14 43.821 −22.076 −11.098 1.00 0.00 N
ATOM 228 H ARG A 14 41.901 −21.808 −16.395 1.00 0.00 H
ATOM 229 HA ARG A 14 44.734 −22.269 −16.907 1.00 0.00 H
ATOM 230 HB2 ARG A 14 43.937 −24.908 −16.388 1.00 0.00 H
ATOM 231 HB3 ARG A 14 43.203 −24.044 −15.057 1.00 0.00 H
ATOM 232 HG2 ARG A 14 46.014 −24.742 −15.592 1.00 0.00 H
ATOM 233 HG3 ARG A 14 45.879 −23.022 −15.230 1.00 0.00 H
ATOM 234 HD2 ARG A 14 44.706 −25.217 −13.484 1.00 0.00 H
ATOM 235 HD3 ARG A 14 46.296 −24.465 −13.233 1.00 0.00 H
ATOM 236 HE ARG A 14 44.494 −22.336 −13.437 1.00 0.00 H
ATOM 237 HH11 ARG A 14 45.013 −24.978 −11.206 1.00 0.00 H
ATOM 238 HH12 ARG A 14 44.382 −24.111 −9.841 1.00 0.00 H
ATOM 239 HH21 ARG A 14 43.557 −21.300 −11.706 1.00 0.00 H
ATOM 240 HH22 ARG A 14 43.639 −22.022 −10.120 1.00 0.00 H
ATOM 241 N LEU A 15 44.096 −23.335 −19.060 1.00 0.00 N
ATOM 242 CA LEU A 15 43.834 −24.121 −20.267 1.00 0.00 C
ATOM 243 C LEU A 15 43.809 −25.615 −19.898 1.00 0.00 C

ATOM 244 O LEU A 15 44.801 −26.138 −19.398 1.00 0.00 O
ATOM 245 CB LEU A 15 44.931 −23.783 −21.301 1.00 0.00 C
ATOM 246 CG LEU A 15 44.927 −24.630 −22.587 1.00 0.00 C
ATOM 247 CD1 LEU A 15 43.663 −24.422 −23.421 1.00 0.00 C
ATOM 248 CD2 LEU A 15 46.128 −24.262 −23.456 1.00 0.00 C
ATOM 249 H LEU A 15 45.048 −23.052 −18.898 1.00 0.00 H
ATOM 250 HA LEU A 15 42.862 −23.838 −20.677 1.00 0.00 H
ATOM 251 HB2 LEU A 15 45.902 −23.920 −20.820 1.00 0.00 H
ATOM 252 HB3 LEU A 15 44.840 −22.730 −21.573 1.00 0.00 H
ATOM 253 HG LEU A 15 45.005 −25.687 −22.333 1.00 0.00 H
ATOM 254 HD11 LEU A 15 43.740 −24.969 −24.360 1.00 0.00 H
ATOM 255 HD12 LEU A 15 43.535 −23.359 −23.634 1.00 0.00 H
ATOM 256 HD13 LEU A 15 42.792 −24.780 −22.880 1.00 0.00 H
ATOM 257 HD21 LEU A 15 47.052 −24.432 −22.903 1.00 0.00 H
ATOM 258 HD22 LEU A 15 46.077 −23.211 −23.744 1.00 0.00 H
ATOM 259 HD23 LEU A 15 46.145 −24.876 −24.356 1.00 0.00 H
ATOM 260 N ILE A 16 42.721 −26.314 −20.227 1.00 0.00 N
ATOM 261 CA ILE A 16 42.559 −27.771 −20.037 1.00 0.00 C
ATOM 262 C ILE A 16 42.486 −28.521 −21.383 1.00 0.00 C
ATOM 263 O ILE A 16 41.862 −29.576 −21.500 1.00 0.00 O
ATOM 264 CB ILE A 16 41.385 −28.092 −19.078 1.00 0.00 C
ATOM 265 CG1 ILE A 16 40.023 −27.655 −19.659 1.00 0.00 C
ATOM 266 CG2 ILE A 16 41.615 −27.485 −17.683 1.00 0.00 C
ATOM 267 CD1 ILE A 16 38.852 −28.479 −19.122 1.00 0.00 C
ATOM 268 H ILE A 16 41.935 −25.803 −20.625 1.00 0.00 H
ATOM 269 HA ILE A 16 43.458 −28.152 −19.551 1.00 0.00 H
ATOM 270 HB ILE A 16 41.368 −29.177 −18.952 1.00 0.00 H
ATOM 271 HG12 ILE A 16 39.852 −26.598 −19.454 1.00 0.00 H
ATOM 272 HG13 ILE A 16 40.038 −27.793 −20.735 1.00 0.00 H
ATOM 273 HG21 ILE A 16 42.589 −27.793 −17.302 1.00 0.00 H

ATOM 274 HG22 ILE A 16 41.572 −26.397 −17.726 1.00 0.00 H
ATOM 275 HG23 ILE A 16 40.849 −27.847 −16.995 1.00 0.00 H
ATOM 276 HD11 ILE A 16 39.016 −29.536 −19.331 1.00 0.00 H
ATOM 277 HD12 ILE A 16 37.935 −28.164 −19.622 1.00 0.00 H
ATOM 278 HD13 ILE A 16 38.746 −28.328 −18.049 1.00 0.00 H
ATOM 279 N SER A 17 43.075 −27.945 −22.436 1.00 0.00 N
ATOM 280 CA SER A 17 42.952 −28.358 −23.846 1.00 0.00 C
ATOM 281 C SER A 17 41.504 −28.349 −24.343 1.00 0.00 C
ATOM 282 O SER A 17 41.102 −27.443 −25.080 1.00 0.00 O
ATOM 283 CB SER A 17 43.609 −29.723 −24.097 1.00 0.00 C
ATOM 284 OG SER A 17 42.722 −30.772 −23.763 1.00 0.00 O
ATOM 285 H SER A 17 43.557 −27.078 −22.252 1.00 0.00 H
ATOM 286 HA SER A 17 43.496 −27.629 −24.446 1.00 0.00 H
ATOM 287 HB2 SER A 17 44.527 −29.810 −23.512 1.00 0.00 H
ATOM 288 HB3 SER A 17 43.861 −29.808 −25.155 1.00 0.00 H
ATOM 289 HG SER A 17 42.459 −30.659 −22.829 1.00 0.00 H
ATOM 290 H SER A 17 40.824 −29.147 −24.045 1.00 0.00 H
ATOM 291 N GLU A 18 47.852 −30.099 −40.413 1.00 0.00 N
ATOM 292 CA GLU A 18 47.877 −29.395 −41.699 1.00 0.00 C
ATOM 293 C GLU A 18 46.935 −28.166 −41.646 1.00 0.00 C
ATOM 294 O GLU A 18 46.825 −27.560 −40.581 1.00 0.00 O
ATOM 295 CB GLU A 18 47.624 −30.413 −42.829 1.00 0.00 C
ATOM 296 CG GLU A 18 48.078 −29.927 −44.224 1.00 0.00 C
ATOM 297 CD GLU A 18 46.946 −29.884 −45.262 1.00 0.00 C
ATOM 298 OE1 GLU A 18 45.827 −29.477 −44.875 1.00 0.00 O
ATOM 299 OE2 GLU A 18 47.248 −30.083 −46.458 1.00 0.00 O
ATOM 300 HA GLU A 18 48.884 −29.003 −41.835 1.00 0.00 H
ATOM 301 HB2 GLU A 18 46.574 −30.706 −42.827 1.00 0.00 H
ATOM 302 HB3 GLU A 18 48.200 −31.314 −42.608 1.00 0.00 H
ATOM 303 HG2 GLU A 18 48.520 −28.932 −44.139 1.00 0.00 H

ATOM	304	HG3	GLU	A	18	48.869	−30.595	−44.576	1.00	0.00	H
ATOM	305	H	GLU	A	18	47.471	−29.570	−39.635	1.00	0.00	H
ATOM	306	H	GLU	A	18	48.805	−30.358	−40.179	1.00	0.00	H
ATOM	307	N	GLY	A	19	46.356	−27.704	−42.760	1.00	0.00	N
ATOM	308	CA	GLY	A	19	45.510	−26.507	−42.816	1.00	0.00	C
ATOM	309	C	GLY	A	19	44.054	−26.823	−42.475	1.00	0.00	C
ATOM	310	O	GLY	A	19	43.267	−27.200	−43.348	1.00	0.00	O
ATOM	311	H	GLY	A	19	46.347	−28.301	−43.587	1.00	0.00	H
ATOM	312	HA2	GLY	A	19	45.886	−25.752	−42.125	1.00	0.00	H
ATOM	313	HA3	GLY	A	19	45.543	−26.100	−43.825	1.00	0.00	H
ATOM	314	N	VAL	A	20	43.654	−26.625	−41.218	1.00	0.00	N
ATOM	315	CA	VAL	A	20	42.374	−27.122	−40.679	1.00	0.00	C
ATOM	316	C	VAL	A	20	41.660	−26.060	−39.843	1.00	0.00	C
ATOM	317	O	VAL	A	20	42.298	−25.317	−39.105	1.00	0.00	O
ATOM	318	CB	VAL	A	20	42.614	−28.437	−39.897	1.00	0.00	C
ATOM	319	CG1	VAL	A	20	41.449	−28.895	−39.005	1.00	0.00	C
ATOM	320	CG2	VAL	A	20	42.910	−29.593	−40.867	1.00	0.00	C
ATOM	321	H	VAL	A	20	44.333	−26.237	−40.562	1.00	0.00	H
ATOM	322	HA	VAL	A	20	41.711	−27.349	−41.509	1.00	0.00	H
ATOM	323	HB	VAL	A	20	43.481	−28.303	−39.255	1.00	0.00	H
ATOM	324	HG21	VAL	A	20	43.075	−30.512	−40.306	1.00	0.00	H
ATOM	325	HG22	VAL	A	20	42.080	−29.712	−41.562	1.00	0.00	H
ATOM	326	HG23	VAL	A	20	43.818	−29.378	−41.433	1.00	0.00	H
ATOM	327	HG11	VAL	A	20	41.688	−29.855	−38.543	1.00	0.00	H
ATOM	328	HG12	VAL	A	20	40.539	−29.003	−39.591	1.00	0.00	H
ATOM	329	HG13	VAL	A	20	41.285	−28.176	−38.203	1.00	0.00	H
ATOM	330	N	CYS	A	21	40.324	−26.107	−39.823	1.00	0.00	N
ATOM	331	CA	CYS	A	21	39.523	−25.474	−38.775	1.00	0.00	C
ATOM	332	C	CYS	A	21	38.522	−26.450	−38.134	1.00	0.00	C
ATOM	333	O	CYS	A	21	37.795	−27.162	−38.826	1.00	0.00	O

ATOM 334 CB CYS A 21 38.843 −24.203 −39.296 1.00 0.00 C
ATOM 335 SG CYS A 21 37.836 −23.395 −38.020 1.00 0.00 S
ATOM 336 H CYS A 21 39.865 −26.774 −40.434 1.00 0.00 H
ATOM 337 HA CYS A 21 40.194 −25.155 −37.982 1.00 0.00 H
ATOM 338 HB2 CYS A 21 39.624 −23.512 −39.620 1.00 0.00 H
ATOM 339 HB3 CYS A 21 38.218 −24.450 −40.154 1.00 0.00 H
ATOM 340 N ILE A 22 38.426 −26.415 −36.806 1.00 0.00 N
ATOM 341 CA ILE A 22 37.385 −27.046 −35.989 1.00 0.00 C
ATOM 342 C ILE A 22 36.337 −25.982 −35.619 1.00 0.00 C
ATOM 343 O ILE A 22 36.694 −24.978 −35.011 1.00 0.00 O
ATOM 344 CB ILE A 22 38.038 −27.659 −34.727 1.00 0.00 C
ATOM 345 CG1 ILE A 22 39.206 −28.632 −35.012 1.00 0.00 C
ATOM 346 CG2 ILE A 22 36.981 −28.338 −33.852 1.00 0.00 C
ATOM 347 CD1 ILE A 22 38.840 −29.866 −35.843 1.00 0.00 C
ATOM 348 H ILE A 22 39.069 −25.793 −36.321 1.00 0.00 H
ATOM 349 HA ILE A 22 36.892 −27.834 −36.557 1.00 0.00 H
ATOM 350 HB ILE A 22 38.456 −26.845 −34.143 1.00 0.00 H
ATOM 351 HG12 ILE A 22 39.621 −28.972 −34.062 1.00 0.00 H
ATOM 352 HG13 ILE A 22 40.004 −28.096 −35.530 1.00 0.00 H
ATOM 353 HG21 ILE A 22 36.462 −29.086 −34.444 1.00 0.00 H
ATOM 354 HG22 ILE A 22 36.261 −27.607 −33.482 1.00 0.00 H
ATOM 355 HG23 ILE A 22 37.452 −28.819 −32.994 1.00 0.00 H
ATOM 356 HD11 ILE A 22 39.740 −30.451 −36.037 1.00 0.00 H
ATOM 357 HD12 ILE A 22 38.416 −29.547 −36.790 1.00 0.00 H
ATOM 358 HD13 ILE A 22 38.124 −30.488 −35.308 1.00 0.00 H
ATOM 359 N THR A 23 35.061 −26.173 −35.973 1.00 0.00 N
ATOM 360 CA THR A 23 34.011 −25.139 −35.810 1.00 0.00 C
ATOM 361 C THR A 23 32.593 −25.736 −35.677 1.00 0.00 C
ATOM 362 O THR A 23 32.461 −26.906 −35.326 1.00 0.00 O
ATOM 363 CB THR A 23 34.154 −24.107 −36.947 1.00 0.00 C

ATOM 364 OG1 THR A 23 33.341 −22.973 −36.758 1.00 0.00 O
ATOM 365 CG2 THR A 23 33.810 −24.689 −38.317 1.00 0.00 C
ATOM 366 H THR A 23 34.825 −26.999 −36.512 1.00 0.00 H
ATOM 367 HA THR A 23 34.204 −24.604 −34.880 1.00 0.00 H
ATOM 368 HB THR A 23 35.187 −23.761 −36.969 1.00 0.00 H
ATOM 369 HG1 THR A 23 32.510 −23.136 −37.279 1.00 0.00 H
ATOM 370 HG21 THR A 23 33.941 −23.913 −39.065 1.00 0.00 H
ATOM 371 HG22 THR A 23 32.777 −25.034 −38.338 1.00 0.00 H
ATOM 372 HG23 THR A 23 34.480 −25.518 −38.544 1.00 0.00 H
ATOM 373 N ASP A 24 31.544 −24.905 −35.745 1.00 0.00 N
ATOM 374 CA ASP A 24 30.172 −25.150 −35.241 1.00 0.00 C
ATOM 375 C ASP A 24 30.130 −25.782 −33.823 1.00 0.00 C
ATOM 376 O ASP A 24 29.601 −26.890 −33.655 1.00 0.00 O
ATOM 377 CB ASP A 24 29.320 −25.911 −36.277 1.00 0.00 C
ATOM 378 CG ASP A 24 27.824 −25.942 −35.912 1.00 0.00 C
ATOM 379 OD1 ASP A 24 27.267 −24.883 −35.555 1.00 0.00 O
ATOM 380 OD2 ASP A 24 27.179 −26.974 −36.224 1.00 0.00 O
ATOM 381 H ASP A 24 31.771 −23.951 −36.009 1.00 0.00 H
ATOM 382 HA ASP A 24 29.708 −24.169 −35.132 1.00 0.00 H
ATOM 383 HB2 ASP A 24 29.698 −26.930 −36.377 1.00 0.00 H
ATOM 384 HB3 ASP A 24 29.425 −25.420 −37.246 1.00 0.00 H
ATOM 385 N PRO A 25 30.851 −25.203 −32.835 1.00 0.00 N
ATOM 386 CA PRO A 25 31.111 −25.870 −31.568 1.00 0.00 C
ATOM 387 C PRO A 25 29.889 −25.889 −30.640 1.00 0.00 C
ATOM 388 O PRO A 25 29.296 −24.848 −30.335 1.00 0.00 O
ATOM 389 CB PRO A 25 32.292 −25.131 −30.942 1.00 0.00 C
ATOM 390 CG PRO A 25 32.151 −23.713 −31.483 1.00 0.00 C
ATOM 391 CD PRO A 25 31.562 −23.927 −32.874 1.00 0.00 C
ATOM 392 HA PRO A 25 31.420 −26.894 −31.768 1.00 0.00 H
ATOM 393 HB2 PRO A 25 32.249 −25.151 −29.856 1.00 0.00 H

ATOM 394 HB3 PRO A 25 33.223 −25.568 −31.302 1.00 0.00 H
ATOM 395 HG2 PRO A 25 31.458 −23.149 −30.863 1.00 0.00 H
ATOM 396 HG3 PRO A 25 33.116 −23.213 −31.533 1.00 0.00 H
ATOM 397 HD2 PRO A 25 32.390 −23.982 −33.577 1.00 0.00 H
ATOM 398 HD3 PRO A 25 30.898 −23.104 −33.142 1.00 0.00 H
ATOM 399 N LEU A 26 29.713 −27.034 −29.981 1.00 0.00 N
ATOM 400 CA LEU A 26 28.813 −27.256 −28.849 1.00 0.00 C
ATOM 401 C LEU A 26 29.625 −27.688 −27.624 1.00 0.00 C
ATOM 402 O LEU A 26 30.477 −28.568 −27.731 1.00 0.00 O
ATOM 403 CB LEU A 26 27.745 −28.286 −29.272 1.00 0.00 C
ATOM 404 CG LEU A 26 26.740 −28.757 −28.193 1.00 0.00 C
ATOM 405 CD1 LEU A 26 25.454 −29.220 −28.876 1.00 0.00 C
ATOM 406 CD2 LEU A 26 27.221 −29.953 −27.368 1.00 0.00 C
ATOM 407 H LEU A 26 30.322 −27.806 −30.239 1.00 0.00 H
ATOM 408 HA LEU A 26 28.304 −26.323 −28.604 1.00 0.00 H
ATOM 409 HB2 LEU A 26 27.186 −27.834 −30.093 1.00 0.00 H
ATOM 410 HB3 LEU A 26 28.252 −29.158 −29.670 1.00 0.00 H
ATOM 411 HG LEU A 26 26.504 −27.928 −27.528 1.00 0.00 H
ATOM 412 HD21 LEU A 26 26.458 −30.227 −26.639 1.00 0.00 H
ATOM 413 HD22 LEU A 26 27.418 −30.808 −28.013 1.00 0.00 H
ATOM 414 HD23 LEU A 26 28.118 −29.704 −26.811 1.00 0.00 H
ATOM 415 HD11 LEU A 26 24.730 −29.544 −28.129 1.00 0.00 H
ATOM 416 HD12 LEU A 26 25.658 −30.041 −29.563 1.00 0.00 H
ATOM 417 HD13 LEU A 26 25.025 −28.386 −29.425 1.00 0.00 H
ATOM 418 N LEU A 27 29.206 −27.209 −26.454 1.00 0.00 N
ATOM 419 CA LEU A 27 29.513 −27.755 −25.133 1.00 0.00 C
ATOM 420 C LEU A 27 28.234 −27.672 −24.288 1.00 0.00 C
ATOM 421 O LEU A 27 27.495 −26.694 −24.406 1.00 0.00 O
ATOM 422 CB LEU A 27 30.689 −26.983 −24.496 1.00 0.00 C
ATOM 423 CG LEU A 27 31.011 −27.381 −23.038 1.00 0.00 C

ATOM 424 CD1 LEU A 27 31.580 −28.798 −22.938 1.00 0.00 C
ATOM 425 CD2 LEU A 27 32.040 −26.429 −22.435 1.00 0.00 C
ATOM 426 H LEU A 27 28.448 −26.543 −26.461 1.00 0.00 H
ATOM 427 HA LEU A 27 29.796 −28.802 −25.235 1.00 0.00 H
ATOM 428 HB2 LEU A 27 31.579 −27.130 −25.106 1.00 0.00 H
ATOM 429 HB3 LEU A 27 30.441 −25.921 −24.508 1.00 0.00 H
ATOM 430 HG LEU A 27 30.113 −27.312 −22.427 1.00 0.00 H
ATOM 431 HD11 LEU A 27 30.841 −29.523 −23.270 1.00 0.00 H
ATOM 432 HD12 LEU A 27 32.477 −28.893 −23.549 1.00 0.00 H
ATOM 433 HD13 LEU A 27 31.822 −29.024 −21.898 1.00 0.00 H
ATOM 434 HD21 LEU A 27 32.996 −26.535 −22.941 1.00 0.00 H
ATOM 435 HD22 LEU A 27 32.160 −26.669 −21.380 1.00 0.00 H
ATOM 436 HD23 LEU A 27 31.686 −25.400 −22.512 1.00 0.00 H
ATOM 437 N ALA A 28 28.009 −28.657 −23.427 1.00 0.00 N
ATOM 438 CA ALA A 28 26.996 −28.628 −22.378 1.00 0.00 C
ATOM 439 C ALA A 28 27.507 −29.389 −21.151 1.00 0.00 C
ATOM 440 O ALA A 28 28.128 −30.441 −21.322 1.00 0.00 O
ATOM 441 CB ALA A 28 25.713 −29.267 −22.919 1.00 0.00 C
ATOM 442 H ALA A 28 28.619 −29.466 −23.444 1.00 0.00 H
ATOM 443 HA ALA A 28 26.790 −27.596 −22.093 1.00 0.00 H
ATOM 444 HB1 ALA A 28 24.916 −29.174 −22.180 1.00 0.00 H
ATOM 445 HB2 ALA A 28 25.417 −28.773 −23.843 1.00 0.00 H
ATOM 446 HB3 ALA A 28 25.883 −30.325 −23.121 1.00 0.00 H
ATOM 447 N VAL A 29 27.206 −28.915 −19.942 1.00 0.00 N
ATOM 448 CA VAL A 29 27.513 −29.596 −18.675 1.00 0.00 C
ATOM 449 C VAL A 29 26.244 −29.750 −17.836 1.00 0.00 C
ATOM 450 O VAL A 29 25.599 −28.771 −17.465 1.00 0.00 O
ATOM 451 CB VAL A 29 28.634 −28.874 −17.896 1.00 0.00 C
ATOM 452 CG1 VAL A 29 28.977 −29.598 −16.585 1.00 0.00 C
ATOM 453 CG2 VAL A 29 29.927 −28.768 −18.719 1.00 0.00 C

```
ATOM    454  H   VAL A  29      26.836 −27.965 −19.892  1.00  0.00           H
ATOM    455  HA  VAL A  29      27.875 −30.598 −18.899  1.00  0.00           H
ATOM    456  HB  VAL A  29      28.304 −27.867 −17.648  1.00  0.00           H
ATOM    457 HG11 VAL A  29      28.121 −29.578 −15.911  1.00  0.00           H
ATOM    458 HG12 VAL A  29      29.809 −29.097 −16.090  1.00  0.00           H
ATOM    459 HG13 VAL A  29      29.251 −30.633 −16.790  1.00  0.00           H
ATOM    460 HG21 VAL A  29      30.706 −28.288 −18.128  1.00  0.00           H
ATOM    461 HG22 VAL A  29      30.257 −29.762 −19.023  1.00  0.00           H
ATOM    462 HG23 VAL A  29      29.751 −28.160 −19.608  1.00  0.00           H
ATOM    463  N   ASP A  30      25.979 −30.976 −17.386  1.00  0.00           N
ATOM    464  CA  ASP A  30      24.855 −31.337 −16.523  1.00  0.00           C
ATOM    465  C   ASP A  30      25.327 −32.279 −15.413  1.00  0.00           C
ATOM    466  O   ASP A  30      25.739 −33.407 −15.671  1.00  0.00           O
ATOM    467  CB  ASP A  30      23.759 −32.039 −17.332  1.00  0.00           C
ATOM    468  CG  ASP A  30      22.716 −31.107 −17.950  1.00  0.00           C
ATOM    469  OD1 ASP A  30      21.921 −30.639 −17.098  1.00  0.00           O
ATOM    470  OD2 ASP A  30      22.293 −31.477 −19.072  1.00  0.00           O
ATOM    471  H   ASP A  30      26.607 −31.723 −17.667  1.00  0.00           H
ATOM    472  HA  ASP A  30      24.427 −30.444 −16.065  1.00  0.00           H
ATOM    473  HB2 ASP A  30      23.226 −32.714 −16.659  1.00  0.00           H
ATOM    474  HB3 ASP A  30      24.223 −32.663 −18.098  1.00  0.00           H
ATOM    475  N   ASN A  31      25.123 −31.875 −14.157  1.00  0.00           N
ATOM    476  CA  ASN A  31      25.235 −32.733 −12.966  1.00  0.00           C
ATOM    477  C   ASN A  31      26.608 −33.416 −12.719  1.00  0.00           C
ATOM    478  O   ASN A  31      26.746 −34.096 −11.708  1.00  0.00           O
ATOM    479  CB  ASN A  31      24.060 −33.742 −12.963  1.00  0.00           C
ATOM    480  CG  ASN A  31      22.694 −33.090 −13.103  1.00  0.00           C
ATOM    481  OD1 ASN A  31      22.000 −33.203 −14.105  1.00  0.00           O
ATOM    482  ND2 ASN A  31      22.285 −32.321 −12.120  1.00  0.00           N
ATOM    483  H   ASN A  31      24.757 −30.944 −14.033  1.00  0.00           H
```

ATOM 484 HA ASN A 31 25.105 −32.083 −12.102 1.00 0.00 H
ATOM 485 HB2 ASN A 31 24.189 −34.450 −13.781 1.00 0.00 H
ATOM 486 HB3 ASN A 31 24.078 −34.307 −12.031 1.00 0.00 H
ATOM 487 HD21 ASN A 31 22.831 −32.248 −11.281 1.00 0.00 H
ATOM 488 HD22 ASN A 31 21.389 −31.884 −12.224 1.00 0.00 H
ATOM 489 N GLY A 32 27.624 −33.166 −13.555 1.00 0.00 N
ATOM 490 CA GLY A 32 28.937 −33.834 −13.541 1.00 0.00 C
ATOM 491 C GLY A 32 29.290 −34.541 −14.861 1.00 0.00 C
ATOM 492 O GLY A 32 30.462 −34.810 −15.122 1.00 0.00 O
ATOM 493 H GLY A 32 27.401 −32.630 −14.381 1.00 0.00 H
ATOM 494 HA2 GLY A 32 28.961 −34.586 −12.751 1.00 0.00 H
ATOM 495 HA3 GLY A 32 29.713 −33.096 −13.333 1.00 0.00 H
ATOM 496 N PHE A 33 28.309 −34.709 −15.750 1.00 0.00 N
ATOM 497 CA PHE A 33 28.478 −35.166 −17.130 1.00 0.00 C
ATOM 498 C PHE A 33 28.590 −33.987 −18.105 1.00 0.00 C
ATOM 499 O PHE A 33 28.134 −32.882 −17.805 1.00 0.00 O
ATOM 500 CB PHE A 33 27.300 −36.078 −17.500 1.00 0.00 C
ATOM 501 CG PHE A 33 27.084 −37.199 −16.501 1.00 0.00 C
ATOM 502 CD1 PHE A 33 28.071 −38.188 −16.349 1.00 0.00 C
ATOM 503 CD2 PHE A 33 25.950 −37.211 −15.669 1.00 0.00 C
ATOM 504 CE1 PHE A 33 27.928 −39.190 −15.375 1.00 0.00 C
ATOM 505 CE2 PHE A 33 25.804 −38.216 −14.695 1.00 0.00 C
ATOM 506 CZ PHE A 33 26.791 −39.208 −14.550 1.00 0.00 C
ATOM 507 H PHE A 33 27.377 −34.401 −15.500 1.00 0.00 H
ATOM 508 HA PHE A 33 29.396 −35.748 −17.200 1.00 0.00 H
ATOM 509 HB2 PHE A 33 26.392 −35.478 −17.572 1.00 0.00 H
ATOM 510 HB3 PHE A 33 27.488 −36.518 −18.481 1.00 0.00 H
ATOM 511 HD1 PHE A 33 28.945 −38.169 −16.980 1.00 0.00 H
ATOM 512 HD2 PHE A 33 25.198 −36.440 −15.770 1.00 0.00 H
ATOM 513 HE1 PHE A 33 28.701 −39.938 −15.258 1.00 0.00 H

ATOM 514 HE2 PHE A 33 24.941 −38.221 −14.049 1.00 0.00 H
ATOM 515 HZ PHE A 33 26.683 −39.976 −13.797 1.00 0.00 H
ATOM 516 N PHE A 34 29.056 −34.251 −19.328 1.00 0.00 N
ATOM 517 CA PHE A 34 29.075 −33.267 −20.415 1.00 0.00 C
ATOM 518 C PHE A 34 28.679 −33.861 −21.773 1.00 0.00 C
ATOM 519 O PHE A 34 28.847 −35.060 −22.012 1.00 0.00 O
ATOM 520 CB PHE A 34 30.447 −32.569 −20.477 1.00 0.00 C
ATOM 521 CG PHE A 34 31.565 −33.383 −21.107 1.00 0.00 C
ATOM 522 CD1 PHE A 34 31.845 −33.249 −22.480 1.00 0.00 C
ATOM 523 CD2 PHE A 34 32.323 −34.275 −20.326 1.00 0.00 C
ATOM 524 CE1 PHE A 34 32.858 −34.022 −23.075 1.00 0.00 C
ATOM 525 CE2 PHE A 34 33.350 −35.031 −20.916 1.00 0.00 C
ATOM 526 CZ PHE A 34 33.609 −34.914 −22.291 1.00 0.00 C
ATOM 527 H PHE A 34 29.396 −35.187 −19.526 1.00 0.00 H
ATOM 528 HA PHE A 34 28.330 −32.510 −20.183 1.00 0.00 H
ATOM 529 HB2 PHE A 34 30.343 −31.653 −21.055 1.00 0.00 H
ATOM 530 HB3 PHE A 34 30.736 −32.267 −19.474 1.00 0.00 H
ATOM 531 HD1 PHE A 34 31.271 −32.560 −23.084 1.00 0.00 H
ATOM 532 HD2 PHE A 34 32.106 −34.395 −19.276 1.00 0.00 H
ATOM 533 HE1 PHE A 34 33.056 −33.932 −24.133 1.00 0.00 H
ATOM 534 HE2 PHE A 34 33.921 −35.719 −20.313 1.00 0.00 H
ATOM 535 HZ PHE A 34 34.383 −35.513 −22.744 1.00 0.00 H
ATOM 536 N ALA A 35 28.321 −32.971 −22.701 1.00 0.00 N
ATOM 537 CA ALA A 35 28.228 −33.227 −24.137 1.00 0.00 C
ATOM 538 C ALA A 35 29.031 −32.200 −24.947 1.00 0.00 C
ATOM 539 O ALA A 35 29.260 −31.073 −24.511 1.00 0.00 O
ATOM 540 CB ALA A 35 26.762 −33.245 −24.574 1.00 0.00 C
ATOM 541 H ALA A 35 28.244 −32.004 −22.401 1.00 0.00 H
ATOM 542 HA ALA A 35 28.654 −34.206 −24.346 1.00 0.00 H
ATOM 543 HB1 ALA A 35 26.694 −33.401 −25.651 1.00 0.00 H

ATOM	544	HB2	ALA	A	35	26.259	−34.063	−24.073	1.00	0.00	H
ATOM	545	HB3	ALA	A	35	26.278	−32.303	−24.316	1.00	0.00	H
ATOM	546	N	TYR	A	36	29.423	−32.590	−26.158	1.00	0.00	N
ATOM	547	CA	TYR	A	36	30.275	−31.819	−27.057	1.00	0.00	C
ATOM	548	C	TYR	A	36	29.952	−32.124	−28.529	1.00	0.00	C
ATOM	549	O	TYR	A	36	29.590	−33.249	−28.875	1.00	0.00	O
ATOM	550	CB	TYR	A	36	31.739	−32.124	−26.712	1.00	0.00	C
ATOM	551	CG	TYR	A	36	32.755	−31.482	−27.635	1.00	0.00	C
ATOM	552	CD1	TYR	A	36	33.206	−30.172	−27.384	1.00	0.00	C
ATOM	553	CD2	TYR	A	36	33.223	−32.184	−28.763	1.00	0.00	C
ATOM	554	CE1	TYR	A	36	34.138	−29.567	−28.250	1.00	0.00	C
ATOM	555	CE2	TYR	A	36	34.147	−31.578	−29.633	1.00	0.00	C
ATOM	556	CZ	TYR	A	36	34.613	−30.270	−29.376	1.00	0.00	C
ATOM	557	OH	TYR	A	36	35.520	−29.692	−30.206	1.00	0.00	O
ATOM	558	H	TYR	A	36	29.238	−33.554	−26.420	1.00	0.00	H
ATOM	559	HA	TYR	A	36	30.104	−30.761	−26.882	1.00	0.00	H
ATOM	560	HB2	TYR	A	36	31.879	−33.203	−26.732	1.00	0.00	H
ATOM	561	HB3	TYR	A	36	31.935	−31.791	−25.691	1.00	0.00	H
ATOM	562	HD1	TYR	A	36	32.819	−29.622	−26.536	1.00	0.00	H
ATOM	563	HD2	TYR	A	36	32.859	−33.179	−28.970	1.00	0.00	H
ATOM	564	HE1	TYR	A	36	34.481	−28.560	−28.069	1.00	0.00	H
ATOM	565	HE2	TYR	A	36	34.499	−32.115	−30.498	1.00	0.00	H
ATOM	566	HH	TYR	A	36	35.090	−29.358	−31.010	1.00	0.00	H
ATOM	567	N	SER	A	37	30.176	−31.160	−29.424	1.00	0.00	N
ATOM	568	CA	SER	A	37	30.195	−31.384	−30.879	1.00	0.00	C
ATOM	569	C	SER	A	37	31.152	−30.421	−31.571	1.00	0.00	C
ATOM	570	O	SER	A	37	31.192	−29.246	−31.206	1.00	0.00	O
ATOM	571	CB	SER	A	37	28.771	−31.323	−31.479	1.00	0.00	C
ATOM	572	OG	SER	A	37	28.649	−30.497	−32.636	1.00	0.00	O
ATOM	573	H	SER	A	37	30.533	−30.273	−29.080	1.00	0.00	H

ATOM 574 HA SER A 37 30.581 −32.388 −31.051 1.00 0.00 H
ATOM 575 HB2 SER A 37 28.481 −32.337 −31.747 1.00 0.00 H
ATOM 576 HB3 SER A 37 28.061 −30.985 −30.726 1.00 0.00 H
ATOM 577 HG SER A 37 28.840 −29.586 −32.367 1.00 0.00 H
ATOM 578 N HIS A 38 31.698 −30.845 −32.714 1.00 0.00 N
ATOM 579 CA HIS A 38 32.311 −29.950 −33.701 1.00 0.00 C
ATOM 580 C HIS A 38 32.132 −30.462 −35.130 1.00 0.00 C
ATOM 581 O HIS A 38 31.985 −31.663 −35.359 1.00 0.00 O
ATOM 582 CB HIS A 38 33.795 −29.706 −33.368 1.00 0.00 C
ATOM 583 CG HIS A 38 34.754 −30.875 −33.523 1.00 0.00 C
ATOM 584 ND1 HIS A 38 35.715 −31.238 −32.574 1.00 0.00 N
ATOM 585 CD2 HIS A 38 35.013 −31.576 −34.670 1.00 0.00 C
ATOM 586 CE1 HIS A 38 36.486 −32.172 −33.150 1.00 0.00 C
ATOM 587 NE2 HIS A 38 36.083 −32.401 −34.408 1.00 0.00 N
ATOM 588 H HIS A 38 31.507 −31.799 −33.004 1.00 0.00 H
ATOM 589 HA HIS A 38 31.805 −28.980 −33.651 1.00 0.00 H
ATOM 590 HB2 HIS A 38 34.147 −28.900 −34.011 1.00 0.00 H
ATOM 591 HB3 HIS A 38 33.860 −29.337 −32.345 1.00 0.00 H
ATOM 592 HD2 HIS A 38 34.513 −31.467 −35.620 1.00 0.00 H
ATOM 593 HE1 HIS A 38 37.324 −32.661 −32.669 1.00 0.00 H
ATOM 594 HE2 HIS A 38 36.519 −33.044 −35.057 1.00 0.00 H
ATOM 595 N LEU A 39 32.341 −29.570 −36.093 1.00 0.00 N
ATOM 596 CA LEU A 39 32.568 −29.848 −37.506 1.00 0.00 C
ATOM 597 C LEU A 39 34.018 −29.492 −37.876 1.00 0.00 C
ATOM 598 O LEU A 39 34.403 −28.324 −37.860 1.00 0.00 O
ATOM 599 CB LEU A 39 31.531 −29.038 −38.307 1.00 0.00 C
ATOM 600 CG LEU A 39 31.610 −29.229 −39.830 1.00 0.00 C
ATOM 601 CD1 LEU A 39 31.315 −30.676 −40.228 1.00 0.00 C
ATOM 602 CD2 LEU A 39 30.582 −28.326 −40.513 1.00 0.00 C
ATOM 603 H LEU A 39 32.463 −28.604 −35.802 1.00 0.00 H

ATOM 604 HA LEU A 39 32.412 −30.907 −37.697 1.00 0.00 H
ATOM 605 HB2 LEU A 39 30.532 −29.316 −37.968 1.00 0.00 H
ATOM 606 HB3 LEU A 39 31.674 −27.979 −38.083 1.00 0.00 H
ATOM 607 HG LEU A 39 32.601 −28.953 −40.192 1.00 0.00 H
ATOM 608 HD11 LEU A 39 32.150 −31.314 −39.946 1.00 0.00 H
ATOM 609 HD12 LEU A 39 31.171 −30.743 −41.303 1.00 0.00 H
ATOM 610 HD13 LEU A 39 30.404 −31.020 −39.741 1.00 0.00 H
ATOM 611 HD21 LEU A 39 30.661 −28.427 −41.594 1.00 0.00 H
ATOM 612 HD22 LEU A 39 29.577 −28.611 −40.200 1.00 0.00 H
ATOM 613 HD23 LEU A 39 30.761 −27.289 −40.230 1.00 0.00 H
ATOM 614 N GLU A 40 34.829 −30.497 −38.201 1.00 0.00 N
ATOM 615 CA GLU A 40 36.172 −30.314 −38.761 1.00 0.00 C
ATOM 616 C GLU A 40 36.102 −29.971 −40.254 1.00 0.00 C
ATOM 617 O GLU A 40 35.412 −30.654 −41.019 1.00 0.00 O
ATOM 618 CB GLU A 40 37.008 −31.587 −38.537 1.00 0.00 C
ATOM 619 CG GLU A 40 38.426 −31.465 −39.123 1.00 0.00 C
ATOM 620 CD GLU A 40 39.310 −32.676 −38.824 1.00 0.00 C
ATOM 621 OE1 GLU A 40 38.843 −33.782 −39.175 1.00 0.00 O
ATOM 622 OE2 GLU A 40 40.538 −32.459 −38.884 1.00 0.00 O
ATOM 623 H GLU A 40 34.438 −31.433 −38.231 1.00 0.00 H
ATOM 624 HA GLU A 40 36.660 −29.484 −38.247 1.00 0.00 H
ATOM 625 HB2 GLU A 40 36.499 −32.422 −39.013 1.00 0.00 H
ATOM 626 HB3 GLU A 40 37.080 −31.782 −37.467 1.00 0.00 H
ATOM 627 HG2 GLU A 40 38.895 −30.563 −38.733 1.00 0.00 H
ATOM 628 HG3 GLU A 40 38.365 −31.355 −40.208 1.00 0.00 H
ATOM 629 N LYS A 41 36.969 −29.057 −40.697 1.00 0.00 N
ATOM 630 CA LYS A 41 37.115 −28.605 −42.084 1.00 0.00 C
ATOM 631 C LYS A 41 38.571 −28.599 −42.541 1.00 0.00 C
ATOM 632 O LYS A 41 39.459 −28.232 −41.779 1.00 0.00 O
ATOM 633 CB LYS A 41 36.532 −27.192 −42.215 1.00 0.00 C

ATOM 634 CG LYS A 41 35.000 −27.188 −42.132 1.00 0.00 C
ATOM 635 CD LYS A 41 34.505 −25.755 −41.942 1.00 0.00 C
ATOM 636 CE LYS A 41 32.984 −25.682 −42.087 1.00 0.00 C
ATOM 637 NZ LYS A 41 32.604 −25.277 −43.460 1.00 0.00 N
ATOM 638 H LYS A 41 37.493 −28.535 −39.997 1.00 0.00 H
ATOM 639 HA LYS A 41 36.563 −29.279 −42.730 1.00 0.00 H
ATOM 640 HB2 LYS A 41 36.950 −26.561 −41.427 1.00 0.00 H
ATOM 641 HB3 LYS A 41 36.825 −26.766 −43.175 1.00 0.00 H
ATOM 642 HG2 LYS A 41 34.590 −27.616 −43.047 1.00 0.00 H
ATOM 643 HG3 LYS A 41 34.661 −27.781 −41.283 1.00 0.00 H
ATOM 644 HD2 LYS A 41 34.988 −25.087 −42.657 1.00 0.00 H
ATOM 645 HD3 LYS A 41 34.781 −25.432 −40.939 1.00 0.00 H
ATOM 646 HE2 LYS A 41 32.556 −26.654 −41.826 1.00 0.00 H
ATOM 647 HE3 LYS A 41 32.605 −24.942 −41.374 1.00 0.00 H
ATOM 648 HZ1 LYS A 41 31.619 −25.409 −43.624 1.00 0.00 H
ATOM 649 HZ2 LYS A 41 32.785 −24.283 −43.584 1.00 0.00 H
ATOM 650 HZ3 LYS A 41 33.148 −25.784 −44.157 1.00 0.00 H
ATOM 651 N ILE A 42 38.782 −28.846 −43.832 1.00 0.00 N
ATOM 652 CA ILE A 42 39.983 −28.435 −44.572 1.00 0.00 C
ATOM 653 C ILE A 42 39.874 −26.930 −44.849 1.00 0.00 C
ATOM 654 O ILE A 42 38.910 −26.489 −45.483 1.00 0.00 O
ATOM 655 CB ILE A 42 40.129 −29.225 −45.896 1.00 0.00 C
ATOM 656 CG1 ILE A 42 40.051 −30.752 −45.664 1.00 0.00 C
ATOM 657 CG2 ILE A 42 41.447 −28.838 −46.594 1.00 0.00 C
ATOM 658 CD1 ILE A 42 40.092 −31.599 −46.942 1.00 0.00 C
ATOM 659 H ILE A 42 37.971 −29.107 −44.381 1.00 0.00 H
ATOM 660 HA ILE A 42 40.868 −28.617 −43.958 1.00 0.00 H
ATOM 661 HB ILE A 42 39.304 −28.947 −46.553 1.00 0.00 H
ATOM 662 HG12 ILE A 42 40.865 −31.058 −45.007 1.00 0.00 H
ATOM 663 HG13 ILE A 42 39.105 −30.985 −45.176 1.00 0.00 H

```
ATOM    664 HG21 ILE A  42      41.492 −27.765 −46.778  1.00  0.00           H
ATOM    665 HG22 ILE A  42      41.532 −29.335 −47.560  1.00  0.00           H
ATOM    666 HG23 ILE A  42      42.301 −29.122 −45.976  1.00  0.00           H
ATOM    667 HD11 ILE A  42      39.877 −32.639 −46.691  1.00  0.00           H
ATOM    668 HD12 ILE A  42      39.340 −31.244 −47.649  1.00  0.00           H
ATOM    669 HD13 ILE A  42      41.078 −31.552 −47.401  1.00  0.00           H
ATOM    670  N   GLY A  43      40.853 −26.149 −44.400  1.00  0.00           N
ATOM    671  CA  GLY A  43      40.871 −24.693 −44.530  1.00  0.00           C
ATOM    672  C   GLY A  43      39.778 −23.973 −43.726  1.00  0.00           C
ATOM    673  O   GLY A  43      39.364 −24.418 −42.658  1.00  0.00           O
ATOM    674  H   GLY A  43      41.642 −26.589 −43.932  1.00  0.00           H
ATOM    675  HA2 GLY A  43      41.840 −24.322 −44.191  1.00  0.00           H
ATOM    676  HA3 GLY A  43      40.764 −24.438 −45.584  1.00  0.00           H
ATOM    677  N   SER A  44      39.377 −22.792 −44.214  1.00  0.00           N
ATOM    678  CA  SER A  44      38.618 −21.785 −43.448  1.00  0.00           C
ATOM    679  C   SER A  44      37.361 −22.322 −42.740  1.00  0.00           C
ATOM    680  O   SER A  44      36.545 −23.020 −43.338  1.00  0.00           O
ATOM    681  CB  SER A  44      38.240 −20.607 −44.361  1.00  0.00           C
ATOM    682  OG  SER A  44      37.289 −20.990 −45.342  1.00  0.00           O
ATOM    683  H   SER A  44      39.825 −22.488 −45.062  1.00  0.00           H
ATOM    684  HA  SER A  44      39.286 −21.399 −42.677  1.00  0.00           H
ATOM    685  HB2 SER A  44      39.138 −20.229 −44.853  1.00  0.00           H
ATOM    686  HB3 SER A  44      37.816 −19.804 −43.756  1.00  0.00           H
ATOM    687  HG  SER A  44      37.612 −21.795 −45.763  1.00  0.00           H
ATOM    688  N   CYS A  45      37.075 −21.793 −41.549  1.00  0.00           N
ATOM    689  CA  CYS A  45      35.934 −22.219 −40.729  1.00  0.00           C
ATOM    690  C   CYS A  45      34.570 −22.018 −41.415  1.00  0.00           C
ATOM    691  O   CYS A  45      33.711 −22.895 −41.369  1.00  0.00           O
ATOM    692  CB  CYS A  45      35.988 −21.445 −39.408  1.00  0.00           C
ATOM    693  SG  CYS A  45      37.608 −21.463 −38.602  1.00  0.00           S
```

ATOM	694	H	CYS	A	45	37.786	−21.248	−41.082	1.00	0.00	H
ATOM	695	HA	CYS	A	45	36.045	−23.281	−40.508	1.00	0.00	H
ATOM	696	HB2	CYS	A	45	35.253	−21.860	−38.715	1.00	0.00	H
ATOM	697	HB3	CYS	A	45	35.720	−20.404	−39.589	1.00	0.00	H
ATOM	698	N	THR	A	46	34.412	−20.958	−42.212	1.00	0.00	N
ATOM	699	CA	THR	A	46	33.191	−20.738	−43.001	1.00	0.00	C
ATOM	700	C	THR	A	46	33.122	−21.675	−44.214	1.00	0.00	C
ATOM	701	O	THR	A	46	32.301	−22.593	−44.245	1.00	0.00	O
ATOM	702	CB	THR	A	46	33.042	−19.262	−43.407	1.00	0.00	C
ATOM	703	OG1	THR	A	46	34.166	−18.816	−44.133	1.00	0.00	O
ATOM	704	CG2	THR	A	46	32.906	−18.338	−42.199	1.00	0.00	C
ATOM	705	H	THR	A	46	35.167	−20.294	−42.291	1.00	0.00	H
ATOM	706	HA	THR	A	46	32.331	−20.983	−42.375	1.00	0.00	H
ATOM	707	HB	THR	A	46	32.152	−19.153	−44.028	1.00	0.00	H
ATOM	708	HG1	THR	A	46	34.385	−17.941	−43.792	1.00	0.00	H
ATOM	709	HG21	THR	A	46	32.720	−17.316	−42.530	1.00	0.00	H
ATOM	710	HG22	THR	A	46	32.063	−18.658	−41.586	1.00	0.00	H
ATOM	711	HG23	THR	A	46	33.810	−18.359	−41.587	1.00	0.00	H
ATOM	712	N	ARG	A	47	34.016	−21.517	−45.198	1.00	0.00	N
ATOM	713	CA	ARG	A	47	33.886	−22.128	−46.540	1.00	0.00	C
ATOM	714	C	ARG	A	47	34.731	−23.388	−46.768	1.00	0.00	C
ATOM	715	O	ARG	A	47	34.648	−23.979	−47.837	1.00	0.00	O
ATOM	716	CB	ARG	A	47	34.132	−21.052	−47.614	1.00	0.00	C
ATOM	717	CG	ARG	A	47	33.023	−19.987	−47.591	1.00	0.00	C
ATOM	718	CD	ARG	A	47	33.265	−18.883	−48.625	1.00	0.00	C
ATOM	719	NE	ARG	A	47	32.190	−17.870	−48.565	1.00	0.00	N
ATOM	720	CZ	ARG	A	47	32.310	−16.559	−48.697	1.00	0.00	C
ATOM	721	NH1	ARG	A	47	33.448	−15.983	−48.965	1.00	0.00	N
ATOM	722	NH2	ARG	A	47	31.267	−15.790	−48.557	1.00	0.00	N
ATOM	723	H	ARG	A	47	34.702	−20.778	−45.071	1.00	0.00	H

ATOM	724	HA	ARG	A	47	32.861	−22.482	−46.665	1.00	0.00	H
ATOM	725	HB2	ARG	A	47	35.104	−20.584	−47.445	1.00	0.00	H
ATOM	726	HB3	ARG	A	47	34.140	−21.519	−48.601	1.00	0.00	H
ATOM	727	HG2	ARG	A	47	32.065	−20.467	−47.798	1.00	0.00	H
ATOM	728	HG3	ARG	A	47	32.975	−19.526	−46.604	1.00	0.00	H
ATOM	729	HD2	ARG	A	47	34.236	−18.430	−48.416	1.00	0.00	H
ATOM	730	HD3	ARG	A	47	33.293	−19.328	−49.622	1.00	0.00	H
ATOM	731	HE	ARG	A	47	31.265	−18.219	−48.377	1.00	0.00	H
ATOM	732	HH11	ARG	A	47	34.266	−16.554	−49.081	1.00	0.00	H
ATOM	733	HH12	ARG	A	47	33.516	−14.985	−49.046	1.00	0.00	H
ATOM	734	HH21	ARG	A	47	30.372	−16.184	−48.327	1.00	0.00	H
ATOM	735	HH22	ARG	A	47	31.361	−14.793	−48.633	1.00	0.00	H
ATOM	736	N	GLY	A	48	35.482	−23.836	−45.765	1.00	0.00	N
ATOM	737	CA	GLY	A	48	36.289	−25.056	−45.809	1.00	0.00	C
ATOM	738	C	GLY	A	48	35.466	−26.342	−45.930	1.00	0.00	C
ATOM	739	O	GLY	A	48	34.333	−26.424	−45.436	1.00	0.00	O
ATOM	740	H	GLY	A	48	35.528	−23.289	−44.912	1.00	0.00	H
ATOM	741	HA2	GLY	A	48	36.974	−24.997	−46.656	1.00	0.00	H
ATOM	742	HA3	GLY	A	48	36.888	−25.119	−44.900	1.00	0.00	H
ATOM	743	N	ILE	A	49	36.051	−27.347	−46.588	1.00	0.00	N
ATOM	744	CA	ILE	A	49	35.428	−28.644	−46.886	1.00	0.00	C
ATOM	745	C	ILE	A	49	35.351	−29.481	−45.608	1.00	0.00	C
ATOM	746	O	ILE	A	49	36.379	−29.747	−44.992	1.00	0.00	O
ATOM	747	CB	ILE	A	49	36.209	−29.404	−47.990	1.00	0.00	C
ATOM	748	CG1	ILE	A	49	36.279	−28.617	−49.321	1.00	0.00	C
ATOM	749	CG2	ILE	A	49	35.559	−30.776	−48.263	1.00	0.00	C
ATOM	750	CD1	ILE	A	49	37.525	−27.728	−49.447	1.00	0.00	C
ATOM	751	H	ILE	A	49	37.019	−27.217	−46.857	1.00	0.00	H
ATOM	752	HA	ILE	A	49	34.414	−28.467	−47.246	1.00	0.00	H
ATOM	753	HB	ILE	A	49	37.224	−29.595	−47.639	1.00	0.00	H

ATOM	754	HG12	ILE	A	49	35.380	−28.011	−49.445	1.00	0.00	H
ATOM	755	HG13	ILE	A	49	36.316	−29.319	−50.156	1.00	0.00	H
ATOM	756	HG21	ILE	A	49	35.588	−31.407	−47.373	1.00	0.00	H
ATOM	757	HG22	ILE	A	49	34.526	−30.654	−48.587	1.00	0.00	H
ATOM	758	HG23	ILE	A	49	36.115	−31.301	−49.042	1.00	0.00	H
ATOM	759	HD11	ILE	A	49	37.547	−27.284	−50.443	1.00	0.00	H
ATOM	760	HD12	ILE	A	49	37.508	−26.926	−48.712	1.00	0.00	H
ATOM	761	HD13	ILE	A	49	38.426	−28.328	−49.310	1.00	0.00	H
ATOM	762	N	ALA	A	50	34.159	−29.948	−45.237	1.00	0.00	N
ATOM	763	CA	ALA	A	50	33.963	−30.791	−44.057	1.00	0.00	C
ATOM	764	C	ALA	A	50	34.711	−32.136	−44.171	1.00	0.00	C
ATOM	765	O	ALA	A	50	34.429	−32.908	−45.085	1.00	0.00	O
ATOM	766	CB	ALA	A	50	32.458	−31.011	−43.871	1.00	0.00	C
ATOM	767	H	ALA	A	50	33.364	−29.765	−45.825	1.00	0.00	H
ATOM	768	HA	ALA	A	50	34.340	−30.256	−43.185	1.00	0.00	H
ATOM	769	HB1	ALA	A	50	32.300	−31.663	−43.015	1.00	0.00	H
ATOM	770	HB2	ALA	A	50	32.039	−31.494	−44.754	1.00	0.00	H
ATOM	771	HB3	ALA	A	50	31.955	−30.058	−43.697	1.00	0.00	H
ATOM	772	N	LYS	A	51	35.543	−32.472	−43.175	1.00	0.00	N
ATOM	773	CA	LYS	A	51	36.100	−33.828	−42.993	1.00	0.00	C
ATOM	774	C	LYS	A	51	35.198	−34.708	−42.127	1.00	0.00	C
ATOM	775	O	LYS	A	51	34.843	−35.806	−42.535	1.00	0.00	O
ATOM	776	CB	LYS	A	51	37.523	−33.785	−42.404	1.00	0.00	C
ATOM	777	CG	LYS	A	51	38.551	−33.093	−43.320	1.00	0.00	C
ATOM	778	CD	LYS	A	51	39.938	−33.764	−43.288	1.00	0.00	C
ATOM	779	CE	LYS	A	51	40.584	−33.827	−41.897	1.00	0.00	C
ATOM	780	NZ	LYS	A	51	41.147	−32.528	−41.462	1.00	0.00	N
ATOM	781	H	LYS	A	51	35.676	−31.791	−42.433	1.00	0.00	H
ATOM	782	HA	LYS	A	51	36.151	−34.327	−43.963	1.00	0.00	H
ATOM	783	HB2	LYS	A	51	37.837	−34.818	−42.235	1.00	0.00	H

ATOM 784 HB3 LYS A 51 37.507 −33.281 −41.438 1.00 0.00 H
ATOM 785 HG2 LYS A 51 38.194 −33.133 −44.350 1.00 0.00 H
ATOM 786 HG3 LYS A 51 38.639 −32.045 −43.031 1.00 0.00 H
ATOM 787 HD2 LYS A 51 39.821 −34.788 −43.650 1.00 0.00 H
ATOM 788 HD3 LYS A 51 40.608 −33.255 −43.981 1.00 0.00 H
ATOM 789 HE2 LYS A 51 39.834 −34.160 −41.172 1.00 0.00 H
ATOM 790 HE3 LYS A 51 41.379 −34.578 −41.904 1.00 0.00 H
ATOM 791 HZ1 LYS A 51 42.028 −32.307 −41.901 1.00 0.00 H
ATOM 792 HZ2 LYS A 51 40.487 −31.776 −41.593 1.00 0.00 H
ATOM 793 HZ3 LYS A 51 41.281 −32.563 −40.443 1.00 0.00 H
ATOM 794 N GLN A 52 34.765 −34.202 −40.970 1.00 0.00 N
ATOM 795 CA GLN A 52 33.913 −34.935 −40.026 1.00 0.00 C
ATOM 796 C GLN A 52 33.124 −33.981 −39.118 1.00 0.00 C
ATOM 797 O GLN A 52 33.687 −33.060 −38.532 1.00 0.00 O
ATOM 798 CB GLN A 52 34.737 −35.946 −39.201 1.00 0.00 C
ATOM 799 CG GLN A 52 35.890 −35.327 −38.396 1.00 0.00 C
ATOM 800 CD GLN A 52 36.834 −36.376 −37.825 1.00 0.00 C
ATOM 801 OE1 GLN A 52 36.428 −37.393 −37.287 1.00 0.00 O
ATOM 802 NE2 GLN A 52 38.126 −36.138 −37.865 1.00 0.00 N
ATOM 803 H GLN A 52 35.093 −33.285 −40.698 1.00 0.00 H
ATOM 804 HA GLN A 52 33.203 −35.526 −40.600 1.00 0.00 H
ATOM 805 HB2 GLN A 52 35.154 −36.686 −39.886 1.00 0.00 H
ATOM 806 HB3 GLN A 52 34.071 −36.470 −38.515 1.00 0.00 H
ATOM 807 HG2 GLN A 52 36.468 −34.685 −39.054 1.00 0.00 H
ATOM 808 HG3 GLN A 52 35.503 −34.722 −37.577 1.00 0.00 H
ATOM 809 HE21 GLN A 52 38.475 −35.301 −38.343 1.00 0.00 H
ATOM 810 HE22 GLN A 52 38.736 −36.845 −37.504 1.00 0.00 H
ATOM 811 N ARG A 53 31.837 −34.276 −38.908 1.00 0.00 N
ATOM 812 CA ARG A 53 31.103 −33.891 −37.696 1.00 0.00 C
ATOM 813 C ARG A 53 31.421 −34.950 −36.645 1.00 0.00 C

```
ATOM    814  O   ARG A  53      31.179 −36.118 −36.926  1.00  0.00           O
ATOM    815  CB  ARG A  53      29.593 −33.831 −38.012  1.00  0.00           C
ATOM    816  CG  ARG A  53      28.643 −33.751 −36.794  1.00  0.00           C
ATOM    817  CD  ARG A  53      28.634 −32.400 −36.071  1.00  0.00           C
ATOM    818  NE  ARG A  53      28.074 −31.349 −36.937  1.00  0.00           N
ATOM    819  CZ  ARG A  53      27.935 −30.070 −36.651  1.00  0.00           C
ATOM    820  NH1 ARG A  53      27.457 −29.278 −37.555  1.00  0.00           N
ATOM    821  NH2 ARG A  53      28.225 −29.537 −35.500  1.00  0.00           N
ATOM    822  H   ARG A  53      31.490 −35.099 −39.380  1.00  0.00           H
ATOM    823  HA  ARG A  53      31.443 −32.922 −37.336  1.00  0.00           H
ATOM    824  HB2 ARG A  53      29.333 −34.730 −38.567  1.00  0.00           H
ATOM    825  HB3 ARG A  53      29.404 −32.989 −38.678  1.00  0.00           H
ATOM    826  HG2 ARG A  53      27.629 −33.950 −37.139  1.00  0.00           H
ATOM    827  HG3 ARG A  53      28.891 −34.532 −36.077  1.00  0.00           H
ATOM    828  HD2 ARG A  53      29.647 −32.139 −35.769  1.00  0.00           H
ATOM    829  HD3 ARG A  53      28.023 −32.487 −35.170  1.00  0.00           H
ATOM    830  HE  ARG A  53      27.794 −31.610 −37.868  1.00  0.00           H
ATOM    831 HH11 ARG A  53      27.018 −29.651 −38.389  1.00  0.00           H
ATOM    832 HH12 ARG A  53      27.295 −28.313 −37.259  1.00  0.00           H
ATOM    833 HH21 ARG A  53      28.509 −30.102 −34.712  1.00  0.00           H
ATOM    834 HH22 ARG A  53      28.038 −28.542 −35.384  1.00  0.00           H
ATOM    835  N   ILE A  54      31.771 −34.554 −35.426  1.00  0.00           N
ATOM    836  CA  ILE A  54      31.750 −35.456 −34.264  1.00  0.00           C
ATOM    837  C   ILE A  54      30.752 −34.940 −33.232  1.00  0.00           C
ATOM    838  O   ILE A  54      30.565 −33.728 −33.090  1.00  0.00           O
ATOM    839  CB  ILE A  54      33.154 −35.736 −33.671  1.00  0.00           C
ATOM    840  CG1 ILE A  54      33.655 −34.642 −32.699  1.00  0.00           C
ATOM    841  CG2 ILE A  54      34.173 −36.033 −34.788  1.00  0.00           C
ATOM    842  CD1 ILE A  54      34.960 −35.011 −31.982  1.00  0.00           C
ATOM    843  H   ILE A  54      31.884 −33.560 −35.253  1.00  0.00           H
```

ATOM	844	HA	ILE	A	54	31.375	−36.428	−34.589	1.00	0.00	H
ATOM	845	HB	ILE	A	54	33.054	−36.652	−33.084	1.00	0.00	H
ATOM	846	HG12	ILE	A	54	32.912	−34.477	−31.918	1.00	0.00	H
ATOM	847	HG13	ILE	A	54	33.786	−33.706	−33.240	1.00	0.00	H
ATOM	848	HG21	ILE	A	54	35.075	−36.482	−34.372	1.00	0.00	H
ATOM	849	HG22	ILE	A	54	34.437	−35.118	−35.316	1.00	0.00	H
ATOM	850	HG23	ILE	A	54	33.748	−36.747	−35.495	1.00	0.00	H
ATOM	851	HD11	ILE	A	54	34.847	−35.971	−31.477	1.00	0.00	H
ATOM	852	HD12	ILE	A	54	35.193	−34.251	−31.239	1.00	0.00	H
ATOM	853	HD13	ILE	A	54	35.784	−35.068	−32.692	1.00	0.00	H
ATOM	854	N	ILE	A	55	30.161	−35.864	−32.481	1.00	0.00	N
ATOM	855	CA	ILE	A	55	29.270	−35.602	−31.349	1.00	0.00	C
ATOM	856	C	ILE	A	55	29.651	−36.583	−30.237	1.00	0.00	C
ATOM	857	O	ILE	A	55	29.774	−37.775	−30.503	1.00	0.00	O
ATOM	858	CB	ILE	A	55	27.786	−35.752	−31.762	1.00	0.00	C
ATOM	859	CG1	ILE	A	55	27.451	−35.005	−33.078	1.00	0.00	C
ATOM	860	CG2	ILE	A	55	26.899	−35.273	−30.600	1.00	0.00	C
ATOM	861	CD1	ILE	A	55	25.974	−35.032	−33.482	1.00	0.00	C
ATOM	862	H	ILE	A	55	30.328	−36.843	−32.708	1.00	0.00	H
ATOM	863	HA	ILE	A	55	29.433	−34.586	−30.993	1.00	0.00	H
ATOM	864	HB	ILE	A	55	27.583	−36.813	−31.926	1.00	0.00	H
ATOM	865	HG12	ILE	A	55	28.007	−35.468	−33.894	1.00	0.00	H
ATOM	866	HG13	ILE	A	55	27.764	−33.965	−33.001	1.00	0.00	H
ATOM	867	HD11	ILE	A	55	25.867	−34.639	−34.494	1.00	0.00	H
ATOM	868	HD12	ILE	A	55	25.603	−36.054	−33.453	1.00	0.00	H
ATOM	869	HD13	ILE	A	55	25.387	−34.411	−32.805	1.00	0.00	H
ATOM	870	HG21	ILE	A	55	27.131	−35.822	−29.687	1.00	0.00	H
ATOM	871	HG22	ILE	A	55	25.855	−35.461	−30.840	1.00	0.00	H
ATOM	872	HG23	ILE	A	55	27.050	−34.208	−30.425	1.00	0.00	H
ATOM	873	N	GLY	A	56	29.941	−36.108	−29.029	1.00	0.00	N

```
ATOM    874  CA  GLY A  56      30.489 −36.947 −27.959  1.00  0.00           C
ATOM    875  C   GLY A  56      30.115 −36.481 −26.558  1.00  0.00           C
ATOM    876  O   GLY A  56      29.592 −35.380 −26.386  1.00  0.00           O
ATOM    877  H   GLY A  56      29.799 −35.120 −28.836  1.00  0.00           H
ATOM    878  HA2 GLY A  56      31.574 −36.976 −28.033  1.00  0.00           H
ATOM    879  HA3 GLY A  56      30.113 −37.963 −28.070  1.00  0.00           H
ATOM    880  N   VAL A  57      30.293 −37.367 −25.581  1.00  0.00           N
ATOM    881  CA  VAL A  57      29.871 −37.190 −24.183  1.00  0.00           C
ATOM    882  C   VAL A  57      30.884 −37.811 −23.218  1.00  0.00           C
ATOM    883  O   VAL A  57      31.718 −38.636 −23.611  1.00  0.00           O
ATOM    884  CB  VAL A  57      28.455 −37.762 −23.925  1.00  0.00           C
ATOM    885  CG1 VAL A  57      27.394 −37.155 −24.847  1.00  0.00           C
ATOM    886  CG2 VAL A  57      28.369 −39.280 −24.089  1.00  0.00           C
ATOM    887  H   VAL A  57      30.743 −38.249 −25.813  1.00  0.00           H
ATOM    888  HA  VAL A  57      29.846 −36.124 −23.967  1.00  0.00           H
ATOM    889  HB  VAL A  57      28.169 −37.529 −22.898  1.00  0.00           H
ATOM    890 HG21 VAL A  57      29.077 −39.771 −23.422  1.00  0.00           H
ATOM    891 HG22 VAL A  57      27.368 −39.625 −23.831  1.00  0.00           H
ATOM    892 HG23 VAL A  57      28.588 −39.545 −25.122  1.00  0.00           H
ATOM    893 HG11 VAL A  57      27.337 −36.083 −24.677  1.00  0.00           H
ATOM    894 HG12 VAL A  57      27.634 −37.345 −25.889  1.00  0.00           H
ATOM    895 HG13 VAL A  57      26.430 −37.606 −24.636  1.00  0.00           H
ATOM    896  N   GLY A  58      30.778 −37.455 −21.939  1.00  0.00           N
ATOM    897  CA  GLY A  58      31.604 −38.039 −20.889  1.00  0.00           C
ATOM    898  C   GLY A  58      31.428 −37.387 −19.522  1.00  0.00           C
ATOM    899  O   GLY A  58      30.374 −36.828 −19.220  1.00  0.00           O
ATOM    900  H   GLY A  58      30.100 −36.746 −21.679  1.00  0.00           H
ATOM    901  HA2 GLY A  58      31.372 −39.098 −20.788  1.00  0.00           H
ATOM    902  HA3 GLY A  58      32.642 −37.944 −21.189  1.00  0.00           H
ATOM    903  N   GLU A  59      32.484 −37.431 −18.709  1.00  0.00           N
```

ATOM 904 CA GLU A 59 32.478 −37.059 −17.286 1.00 0.00 C
ATOM 905 C GLU A 59 33.516 −35.947 −16.996 1.00 0.00 C
ATOM 906 O GLU A 59 34.571 −35.895 −17.633 1.00 0.00 O
ATOM 907 CB GLU A 59 32.691 −38.324 −16.420 1.00 0.00 C
ATOM 908 CG GLU A 59 31.831 −39.527 −16.879 1.00 0.00 C
ATOM 909 CD GLU A 59 31.691 −40.658 −15.847 1.00 0.00 C
ATOM 910 OE1 GLU A 59 32.668 −40.913 −15.109 1.00 0.00 O
ATOM 911 OE2 GLU A 59 30.652 −41.363 −15.901 1.00 0.00 O
ATOM 912 H GLU A 59 33.330 −37.866 −19.064 1.00 0.00 H
ATOM 913 HA GLU A 59 31.497 −36.661 −17.025 1.00 0.00 H
ATOM 914 HB2 GLU A 59 33.742 −38.614 −16.457 1.00 0.00 H
ATOM 915 HB3 GLU A 59 32.436 −38.072 −15.390 1.00 0.00 H
ATOM 916 HG2 GLU A 59 30.836 −39.163 −17.128 1.00 0.00 H
ATOM 917 HG3 GLU A 59 32.264 −39.943 −17.792 1.00 0.00 H
ATOM 918 N VAL A 60 33.189 −34.980 −16.124 1.00 0.00 N
ATOM 919 CA VAL A 60 33.978 −33.742 −15.898 1.00 0.00 C
ATOM 920 C VAL A 60 34.874 −33.844 −14.647 1.00 0.00 C
ATOM 921 O VAL A 60 34.716 −33.103 −13.677 1.00 0.00 O
ATOM 922 CB VAL A 60 33.088 −32.474 −15.905 1.00 0.00 C
ATOM 923 CG1 VAL A 60 33.923 −31.183 −15.995 1.00 0.00 C
ATOM 924 CG2 VAL A 60 32.144 −32.432 −17.114 1.00 0.00 C
ATOM 925 H VAL A 60 32.287 −35.044 −15.658 1.00 0.00 H
ATOM 926 HA VAL A 60 34.658 −33.630 −16.740 1.00 0.00 H
ATOM 927 HB VAL A 60 32.481 −32.447 −14.999 1.00 0.00 H
ATOM 928 HG11 VAL A 60 33.262 −30.317 −16.001 1.00 0.00 H
ATOM 929 HG12 VAL A 60 34.526 −31.188 −16.903 1.00 0.00 H
ATOM 930 HG13 VAL A 60 34.583 −31.098 −15.135 1.00 0.00 H
ATOM 931 HG21 VAL A 60 31.554 −31.515 −17.093 1.00 0.00 H
ATOM 932 HG22 VAL A 60 32.724 −32.465 −18.036 1.00 0.00 H
ATOM 933 HG23 VAL A 60 31.452 −33.273 −17.087 1.00 0.00 H

ATOM 934 N LEU A 61 35.668 −34.914 −14.577 1.00 0.00 N

ATOM 935 CA LEU A 61 36.376 −35.344 −13.362 1.00 0.00 C

ATOM 936 C LEU A 61 37.560 −34.429 −12.982 1.00 0.00 C

ATOM 937 O LEU A 61 38.222 −33.833 −13.839 1.00 0.00 O

ATOM 938 CB LEU A 61 36.828 −36.815 −13.502 1.00 0.00 C

ATOM 939 CG LEU A 61 35.766 −37.806 −14.024 1.00 0.00 C

ATOM 940 CD1 LEU A 61 36.388 −39.192 −14.190 1.00 0.00 C

ATOM 941 CD2 LEU A 61 34.568 −37.915 −13.081 1.00 0.00 C

ATOM 942 H LEU A 61 35.735 −35.486 −15.405 1.00 0.00 H

ATOM 943 HA LEU A 61 35.664 −35.294 −12.536 1.00 0.00 H

ATOM 944 HB2 LEU A 61 37.679 −36.846 −14.176 1.00 0.00 H

ATOM 945 HB3 LEU A 61 37.179 −37.160 −12.528 1.00 0.00 H

ATOM 946 HG LEU A 61 35.416 −37.483 −15.004 1.00 0.00 H

ATOM 947 HD11 LEU A 61 35.634 −39.879 −14.583 1.00 0.00 H

ATOM 948 HD12 LEU A 61 36.743 −39.563 −13.230 1.00 0.00 H

ATOM 949 HD13 LEU A 61 37.217 −39.142 −14.896 1.00 0.00 H

ATOM 950 HD21 LEU A 61 34.899 −38.199 −12.083 1.00 0.00 H

ATOM 951 HD22 LEU A 61 34.039 −36.964 −13.037 1.00 0.00 H

ATOM 952 HD23 LEU A 61 33.879 −38.676 −13.455 1.00 0.00 H

ATOM 953 N ASP A 62 37.947 −34.452 −11.706 1.00 0.00 N

ATOM 954 CA ASP A 62 39.197 −33.879 −11.194 1.00 0.00 C

ATOM 955 C ASP A 62 40.246 −34.977 −10.893 1.00 0.00 C

ATOM 956 O ASP A 62 40.281 −36.019 −11.554 1.00 0.00 O

ATOM 957 CB ASP A 62 38.898 −32.980 −9.981 1.00 0.00 C

ATOM 958 CG ASP A 62 38.368 −33.705 −8.737 1.00 0.00 C

ATOM 959 OD1 ASP A 62 38.344 −33.038 −7.682 1.00 0.00 O

ATOM 960 OD2 ASP A 62 38.142 −34.932 −8.817 1.00 0.00 O

ATOM 961 H ASP A 62 37.426 −35.013 −11.041 1.00 0.00 H

ATOM 962 HA ASP A 62 39.624 −33.219 −11.941 1.00 0.00 H

ATOM 963 HB2 ASP A 62 39.807 −32.441 −9.713 1.00 0.00 H

ATOM 964 HB3 ASP A 62 38.176 −32.227 −10.297 1.00 0.00 H
ATOM 965 N ARG A 63 41.282 −34.592 −10.139 1.00 0.00 N
ATOM 966 CA ARG A 63 42.199 −35.449 −9.365 1.00 0.00 C
ATOM 967 C ARG A 63 42.535 −34.676 −8.076 1.00 0.00 C
ATOM 968 O ARG A 63 42.035 −33.574 −7.877 1.00 0.00 O
ATOM 969 CB ARG A 63 43.478 −35.822 −10.155 1.00 0.00 C
ATOM 970 CG ARG A 63 43.325 −36.720 −11.398 1.00 0.00 C
ATOM 971 CD ARG A 63 43.109 −36.074 −12.781 1.00 0.00 C
ATOM 972 NE ARG A 63 43.115 −34.596 −12.795 1.00 0.00 N
ATOM 973 CZ ARG A 63 42.185 −33.804 −13.287 1.00 0.00 C
ATOM 974 NH1 ARG A 63 41.069 −34.260 −13.779 1.00 0.00 N
ATOM 975 NH2 ARG A 63 42.342 −32.523 −13.233 1.00 0.00 N
ATOM 976 H ARG A 63 41.182 −33.660 −9.752 1.00 0.00 H
ATOM 977 HA ARG A 63 41.664 −36.357 −9.078 1.00 0.00 H
ATOM 978 HB2 ARG A 63 44.113 −36.398 −9.481 1.00 0.00 H
ATOM 979 HB3 ARG A 63 44.040 −34.925 −10.398 1.00 0.00 H
ATOM 980 HG2 ARG A 63 44.245 −37.302 −11.479 1.00 0.00 H
ATOM 981 HG3 ARG A 63 42.528 −37.440 −11.216 1.00 0.00 H
ATOM 982 HD2 ARG A 63 42.175 −36.461 −13.186 1.00 0.00 H
ATOM 983 HD3 ARG A 63 43.903 −36.420 −13.445 1.00 0.00 H
ATOM 984 HE ARG A 63 43.900 −34.109 −12.353 1.00 0.00 H
ATOM 985 HH11 ARG A 63 40.810 −35.185 −13.468 1.00 0.00 H
ATOM 986 HH12 ARG A 63 40.301 −33.623 −13.952 1.00 0.00 H
ATOM 987 HH21 ARG A 63 43.032 −32.184 −12.557 1.00 0.00 H
ATOM 988 HH22 ARG A 63 41.523 −31.932 −13.333 1.00 0.00 H
ATOM 989 N GLY A 64 43.596 −35.063 −7.362 1.00 0.00 N
ATOM 990 CA GLY A 64 44.177 −34.264 −6.269 1.00 0.00 C
ATOM 991 C GLY A 64 44.691 −32.858 −6.646 1.00 0.00 C
ATOM 992 O GLY A 64 44.932 −32.060 −5.744 1.00 0.00 O
ATOM 993 H GLY A 64 44.027 −35.941 −7.599 1.00 0.00 H

ATOM	994	HA2	GLY	A	64	43.419	−34.136	−5.494	1.00	0.00	H
ATOM	995	HA3	GLY	A	64	45.013	−34.813	−5.838	1.00	0.00	H
ATOM	996	N	ASP	A	65	44.792	−32.517	−7.939	1.00	0.00	N
ATOM	997	CA	ASP	A	65	45.024	−31.142	−8.429	1.00	0.00	C
ATOM	998	C	ASP	A	65	43.776	−30.227	−8.348	1.00	0.00	C
ATOM	999	O	ASP	A	65	43.903	−28.999	−8.368	1.00	0.00	O
ATOM	1000	CB	ASP	A	65	45.556	−31.212	−9.873	1.00	0.00	C
ATOM	1001	CG	ASP	A	65	44.576	−31.843	−10.870	1.00	0.00	C
ATOM	1002	OD1	ASP	A	65	44.908	−32.874	−11.493	1.00	0.00	O
ATOM	1003	OD2	ASP	A	65	43.435	−31.355	−11.002	1.00	0.00	O
ATOM	1004	H	ASP	A	65	44.545	−33.209	−8.628	1.00	0.00	H
ATOM	1005	HA	ASP	A	65	45.793	−30.680	−7.810	1.00	0.00	H
ATOM	1006	HB2	ASP	A	65	45.792	−30.201	−10.209	1.00	0.00	H
ATOM	1007	HB3	ASP	A	65	46.486	−31.785	−9.872	1.00	0.00	H
ATOM	1008	N	LYS	A	66	42.592	−30.831	−8.185	1.00	0.00	N
ATOM	1009	CA	LYS	A	66	41.267	−30.221	−7.978	1.00	0.00	C
ATOM	1010	C	LYS	A	66	40.803	−29.293	−9.107	1.00	0.00	C
ATOM	1011	O	LYS	A	66	40.002	−28.384	−8.881	1.00	0.00	O
ATOM	1012	CB	LYS	A	66	41.139	−29.639	−6.559	1.00	0.00	C
ATOM	1013	CG	LYS	A	66	41.758	−30.592	−5.520	1.00	0.00	C
ATOM	1014	CD	LYS	A	66	41.206	−30.392	−4.115	1.00	0.00	C
ATOM	1015	CE	LYS	A	66	41.977	−31.308	−3.158	1.00	0.00	C
ATOM	1016	NZ	LYS	A	66	41.169	−31.616	−1.960	1.00	0.00	N
ATOM	1017	H	LYS	A	66	42.608	−31.843	−8.160	1.00	0.00	H
ATOM	1018	HA	LYS	A	66	40.554	−31.049	−8.011	1.00	0.00	H
ATOM	1019	HB2	LYS	A	66	41.645	−28.672	−6.506	1.00	0.00	H
ATOM	1020	HB3	LYS	A	66	40.081	−29.492	−6.341	1.00	0.00	H
ATOM	1021	HG2	LYS	A	66	42.837	−30.439	−5.503	1.00	0.00	H
ATOM	1022	HG3	LYS	A	66	41.551	−31.625	−5.808	1.00	0.00	H
ATOM	1023	HD2	LYS	A	66	41.310	−29.351	−3.806	1.00	0.00	H

ATOM 1024 HD3 LYS A 66 40.148 −30.660 −4.137 1.00 0.00 H
ATOM 1025 HE2 LYS A 66 42.910 −30.813 −2.883 1.00 0.00 H
ATOM 1026 HE3 LYS A 66 42.221 −32.236 −3.679 1.00 0.00 H
ATOM 1027 HZ1 LYS A 66 41.682 −31.991 −1.183 1.00 0.00 H
ATOM 1028 HZ2 LYS A 66 40.404 −32.260 −2.193 1.00 0.00 H
ATOM 1029 HZ3 LYS A 66 40.584 −30.823 −1.686 1.00 0.00 H
ATOM 1030 N VAL A 67 41.310 −29.508 −10.324 1.00 0.00 N
ATOM 1031 CA VAL A 67 40.852 −28.860 −11.563 1.00 0.00 C
ATOM 1032 C VAL A 67 39.850 −29.779 −12.285 1.00 0.00 C
ATOM 1033 O VAL A 67 40.228 −30.898 −12.642 1.00 0.00 O
ATOM 1034 CB VAL A 67 42.044 −28.531 −12.488 1.00 0.00 C
ATOM 1035 CG1 VAL A 67 41.593 −27.774 −13.744 1.00 0.00 C
ATOM 1036 CG2 VAL A 67 43.100 −27.665 −11.783 1.00 0.00 C
ATOM 1037 H VAL A 67 42.002 −30.252 −10.416 1.00 0.00 H
ATOM 1038 HA VAL A 67 40.371 −27.920 −11.307 1.00 0.00 H
ATOM 1039 HB VAL A 67 42.526 −29.456 −12.803 1.00 0.00 H
ATOM 1040 HG11 VAL A 67 42.458 −27.519 −14.357 1.00 0.00 H
ATOM 1041 HG12 VAL A 67 41.057 −26.866 −13.470 1.00 0.00 H
ATOM 1042 HG13 VAL A 67 40.941 −28.408 −14.345 1.00 0.00 H
ATOM 1043 HG21 VAL A 67 43.904 −27.427 −12.479 1.00 0.00 H
ATOM 1044 HG22 VAL A 67 42.645 −26.752 −11.404 1.00 0.00 H
ATOM 1045 HG23 VAL A 67 43.532 −28.223 −10.949 1.00 0.00 H
ATOM 1046 N PRO A 68 38.633 −29.325 −12.636 1.00 0.00 N
ATOM 1047 CA PRO A 68 37.737 −30.098 −13.493 1.00 0.00 C
ATOM 1048 C PRO A 68 38.318 −30.227 −14.910 1.00 0.00 C
ATOM 1049 O PRO A 68 38.679 −29.220 −15.524 1.00 0.00 O
ATOM 1050 CB PRO A 68 36.395 −29.355 −13.495 1.00 0.00 C
ATOM 1051 CG PRO A 68 36.513 −28.293 −12.401 1.00 0.00 C
ATOM 1052 CD PRO A 68 38.013 −28.077 −12.239 1.00 0.00 C
ATOM 1053 HA PRO A 68 37.596 −31.090 −13.062 1.00 0.00 H

ATOM 1054 HB2 PRO A 68 36.233 −28.867 −14.455 1.00 0.00 H
ATOM 1055 HB3 PRO A 68 35.575 −30.041 −13.282 1.00 0.00 H
ATOM 1056 HG2 PRO A 68 36.099 −28.678 −11.470 1.00 0.00 H
ATOM 1057 HG3 PRO A 68 36.007 −27.370 −12.685 1.00 0.00 H
ATOM 1058 HD2 PRO A 68 38.237 −27.834 −11.201 1.00 0.00 H
ATOM 1059 HD3 PRO A 68 38.350 −27.280 −12.904 1.00 0.00 H
ATOM 1060 N SER A 69 38.291 −31.436 −15.468 1.00 0.00 N
ATOM 1061 CA SER A 69 38.810 −31.764 −16.805 1.00 0.00 C
ATOM 1062 C SER A 69 37.825 −32.657 −17.558 1.00 0.00 C
ATOM 1063 O SER A 69 37.126 −33.456 −16.944 1.00 0.00 O
ATOM 1064 CB SER A 69 40.164 −32.477 −16.705 1.00 0.00 C
ATOM 1065 OG SER A 69 41.124 −31.676 −16.042 1.00 0.00 O
ATOM 1066 H SER A 69 37.953 −32.215 −14.909 1.00 0.00 H
ATOM 1067 HA SER A 69 38.953 −30.846 −17.375 1.00 0.00 H
ATOM 1068 HB2 SER A 69 40.033 −33.410 −16.159 1.00 0.00 H
ATOM 1069 HB3 SER A 69 40.525 −32.711 −17.708 1.00 0.00 H
ATOM 1070 HG SER A 69 41.895 −32.215 −15.835 1.00 0.00 H
ATOM 1071 N MET A 70 37.794 −32.584 −18.893 1.00 0.00 N
ATOM 1072 CA MET A 70 36.953 −33.487 −19.689 1.00 0.00 C
ATOM 1073 C MET A 70 37.548 −34.907 −19.730 1.00 0.00 C
ATOM 1074 O MET A 70 38.711 −35.080 −20.102 1.00 0.00 O
ATOM 1075 CB MET A 70 36.684 −32.908 −21.096 1.00 0.00 C
ATOM 1076 CG MET A 70 37.801 −33.133 −22.127 1.00 0.00 C
ATOM 1077 SD MET A 70 37.475 −32.468 −23.780 1.00 0.00 S
ATOM 1078 CE MET A 70 38.727 −33.401 −24.703 1.00 0.00 C
ATOM 1079 H MET A 70 38.431 −31.965 −19.371 1.00 0.00 H
ATOM 1080 HA MET A 70 35.984 −33.556 −19.189 1.00 0.00 H
ATOM 1081 HB2 MET A 70 35.782 −33.380 −21.485 1.00 0.00 H
ATOM 1082 HB3 MET A 70 36.486 −31.840 −21.012 1.00 0.00 H
ATOM 1083 HG2 MET A 70 37.941 −34.206 −22.247 1.00 0.00 H

ATOM	1084	HG3	MET	A	70	38.733	−32.706	−21.750	1.00	0.00	H
ATOM	1085	HE1	MET	A	70	38.688	−33.121	−25.755	1.00	0.00	H
ATOM	1086	HE2	MET	A	70	38.528	−34.469	−24.609	1.00	0.00	H
ATOM	1087	HE3	MET	A	70	39.718	−33.184	−24.303	1.00	0.00	H
ATOM	1088	N	PHE	A	71	36.694	−35.919	−19.593	1.00	0.00	N
ATOM	1089	CA	PHE	A	71	36.996	−37.318	−19.900	1.00	0.00	C
ATOM	1090	C	PHE	A	71	35.923	−37.860	−20.845	1.00	0.00	C
ATOM	1091	O	PHE	A	71	34.844	−38.256	−20.410	1.00	0.00	O
ATOM	1092	CB	PHE	A	71	37.115	−38.135	−18.601	1.00	0.00	C
ATOM	1093	CG	PHE	A	71	38.319	−37.751	−17.761	1.00	0.00	C
ATOM	1094	CD1	PHE	A	71	39.526	−38.465	−17.878	1.00	0.00	C
ATOM	1095	CD2	PHE	A	71	38.246	−36.649	−16.892	1.00	0.00	C
ATOM	1096	CE1	PHE	A	71	40.654	−38.077	−17.131	1.00	0.00	C
ATOM	1097	CE2	PHE	A	71	39.374	−36.256	−16.155	1.00	0.00	C
ATOM	1098	CZ	PHE	A	71	40.578	−36.968	−16.270	1.00	0.00	C
ATOM	1099	H	PHE	A	71	35.799	−35.736	−19.144	1.00	0.00	H
ATOM	1100	HA	PHE	A	71	37.956	−37.389	−20.412	1.00	0.00	H
ATOM	1101	HB2	PHE	A	71	37.194	−39.193	−18.858	1.00	0.00	H
ATOM	1102	HB3	PHE	A	71	36.209	−38.014	−18.006	1.00	0.00	H
ATOM	1103	HD1	PHE	A	71	39.590	−39.311	−18.546	1.00	0.00	H
ATOM	1104	HD2	PHE	A	71	37.322	−36.093	−16.800	1.00	0.00	H
ATOM	1105	HE1	PHE	A	71	41.578	−38.629	−17.223	1.00	0.00	H
ATOM	1106	HE2	PHE	A	71	39.297	−35.410	−15.492	1.00	0.00	H
ATOM	1107	HZ	PHE	A	71	41.444	−36.666	−15.698	1.00	0.00	H
ATOM	1108	N	MET	A	72	36.182	−37.772	−22.156	1.00	0.00	N
ATOM	1109	CA	MET	A	72	35.320	−38.366	−23.189	1.00	0.00	C
ATOM	1110	C	MET	A	72	35.211	−39.877	−22.971	1.00	0.00	C
ATOM	1111	O	MET	A	72	36.225	−40.572	−22.988	1.00	0.00	O
ATOM	1112	CB	MET	A	72	35.903	−38.118	−24.591	1.00	0.00	C
ATOM	1113	CG	MET	A	72	35.610	−36.727	−25.155	1.00	0.00	C

ATOM 1114 SD MET A 72 33.900 −36.526 −25.726 1.00 0.00 S
ATOM 1115 CE MET A 72 34.105 −35.118 −26.845 1.00 0.00 C
ATOM 1116 H MET A 72 37.090 −37.441 −22.432 1.00 0.00 H
ATOM 1117 HA MET A 72 34.319 −37.937 −23.128 1.00 0.00 H
ATOM 1118 HB2 MET A 72 35.486 −38.850 −25.286 1.00 0.00 H
ATOM 1119 HB3 MET A 72 36.983 −38.271 −24.559 1.00 0.00 H
ATOM 1120 HG2 MET A 72 35.848 −35.962 −24.415 1.00 0.00 H
ATOM 1121 HG3 MET A 72 36.267 −36.581 −26.012 1.00 0.00 H
ATOM 1122 HE1 MET A 72 33.158 −34.925 −27.345 1.00 0.00 H
ATOM 1123 HE2 MET A 72 34.410 −34.236 −26.281 1.00 0.00 H
ATOM 1124 HE3 MET A 72 34.861 −35.352 −27.596 1.00 0.00 H
ATOM 1125 N THR A 73 33.985 −40.388 −22.892 1.00 0.00 N
ATOM 1126 CA THR A 73 33.703 −41.828 −22.770 1.00 0.00 C
ATOM 1127 C THR A 73 33.118 −42.423 −24.053 1.00 0.00 C
ATOM 1128 O THR A 73 33.195 −43.634 −24.259 1.00 0.00 O
ATOM 1129 CB THR A 73 32.724 −42.089 −21.619 1.00 0.00 C
ATOM 1130 OG1 THR A 73 31.494 −41.478 −21.922 1.00 0.00 O
ATOM 1131 CG2 THR A 73 33.207 −41.549 −20.275 1.00 0.00 C
ATOM 1132 H THR A 73 33.187 −39.756 −22.898 1.00 0.00 H
ATOM 1133 HA THR A 73 34.624 −42.369 −22.551 1.00 0.00 H
ATOM 1134 HB THR A 73 32.587 −43.168 −21.532 1.00 0.00 H
ATOM 1135 HG1 THR A 73 30.965 −41.424 −21.101 1.00 0.00 H
ATOM 1136 HG21 THR A 73 34.183 −41.972 −20.046 1.00 0.00 H
ATOM 1137 HG22 THR A 73 32.517 −41.855 −19.491 1.00 0.00 H
ATOM 1138 HG23 THR A 73 33.283 −40.460 −20.285 1.00 0.00 H
ATOM 1139 N ASN A 74 32.483 −41.597 −24.893 1.00 0.00 N
ATOM 1140 CA ASN A 74 31.578 −42.048 −25.945 1.00 0.00 C
ATOM 1141 C ASN A 74 31.463 −40.993 −27.060 1.00 0.00 C
ATOM 1142 O ASN A 74 31.212 −39.822 −26.778 1.00 0.00 O
ATOM 1143 CB ASN A 74 30.227 −42.298 −25.260 1.00 0.00 C

ATOM 1144 CG ASN A 74 29.172 −42.951 −26.120 1.00 0.00 C

ATOM 1145 OD1 ASN A 74 29.389 −43.366 −27.247 1.00 0.00 O

ATOM 1146 ND2 ASN A 74 28.011 −43.150 −25.556 1.00 0.00 N

ATOM 1147 H ASN A 74 32.390 −40.631 −24.598 1.00 0.00 H

ATOM 1148 HA ASN A 74 31.949 −42.983 −26.369 1.00 0.00 H

ATOM 1149 HB2 ASN A 74 30.367 −42.925 −24.384 1.00 0.00 H

ATOM 1150 HB3 ASN A 74 29.841 −41.352 −24.901 1.00 0.00 H

ATOM 1151 HD21 ASN A 74 27.888 −42.863 −24.588 1.00 0.00 H

ATOM 1152 HD22 ASN A 74 27.297 −43.655 −26.062 1.00 0.00 H

ATOM 1153 N VAL A 75 31.651 −41.390 −28.325 1.00 0.00 N

ATOM 1154 CA VAL A 75 31.692 −40.484 −29.489 1.00 0.00 C

ATOM 1155 C VAL A 75 31.021 −41.130 −30.705 1.00 0.00 C

ATOM 1156 O VAL A 75 31.187 −42.324 −30.954 1.00 0.00 O

ATOM 1157 CB VAL A 75 33.138 −40.041 −29.824 1.00 0.00 C

ATOM 1158 CG1 VAL A 75 33.187 −39.020 −30.972 1.00 0.00 C

ATOM 1159 CG2 VAL A 75 33.861 −39.399 −28.628 1.00 0.00 C

ATOM 1160 H VAL A 75 31.775 −42.376 −28.507 1.00 0.00 H

ATOM 1161 HA VAL A 75 31.122 −39.589 −29.247 1.00 0.00 H

ATOM 1162 HB VAL A 75 33.709 −40.920 −30.126 1.00 0.00 H

ATOM 1163 HG11 VAL A 75 34.213 −38.694 −31.137 1.00 0.00 H

ATOM 1164 HG12 VAL A 75 32.835 −39.471 −31.899 1.00 0.00 H

ATOM 1165 HG13 VAL A 75 32.569 −38.154 −30.731 1.00 0.00 H

ATOM 1166 HG21 VAL A 75 34.853 −39.060 −28.926 1.00 0.00 H

ATOM 1167 HG22 VAL A 75 33.289 −38.551 −28.251 1.00 0.00 H

ATOM 1168 HG23 VAL A 75 33.989 −40.127 −27.828 1.00 0.00 H

ATOM 1169 N TRP A 76 30.305 −40.329 −31.493 1.00 0.00 N

ATOM 1170 CA TRP A 76 29.620 −40.710 −32.727 1.00 0.00 C

ATOM 1171 C TRP A 76 29.911 −39.713 −33.860 1.00 0.00 C

ATOM 1172 O TRP A 76 30.058 −38.510 −33.629 1.00 0.00 O

ATOM 1173 CB TRP A 76 28.120 −40.845 −32.440 1.00 0.00 C

ATOM 1174 CG TRP A 76 27.261 −41.178 −33.622 1.00 0.00 C
ATOM 1175 CD1 TRP A 76 27.079 −42.417 −34.126 1.00 0.00 C
ATOM 1176 CD2 TRP A 76 26.498 −40.280 −34.488 1.00 0.00 C
ATOM 1177 NE1 TRP A 76 26.255 −42.355 −35.232 1.00 0.00 N
ATOM 1178 CE2 TRP A 76 25.853 −41.063 −35.492 1.00 0.00 C
ATOM 1179 CE3 TRP A 76 26.269 −38.888 −34.515 1.00 0.00 C
ATOM 1180 CZ2 TRP A 76 25.015 −40.499 −36.466 1.00 0.00 C
ATOM 1181 CZ3 TRP A 76 25.418 −38.312 −35.478 1.00 0.00 C
ATOM 1182 CH2 TRP A 76 24.795 −39.112 −36.454 1.00 0.00 C
ATOM 1183 H TRP A 76 30.218 −39.356 −31.219 1.00 0.00 H
ATOM 1184 HA TRP A 76 29.987 −41.684 −33.050 1.00 0.00 H
ATOM 1185 HB2 TRP A 76 27.979 −41.617 −31.683 1.00 0.00 H
ATOM 1186 HB3 TRP A 76 27.763 −39.905 −32.017 1.00 0.00 H
ATOM 1187 HD1 TRP A 76 27.517 −43.322 −33.716 1.00 0.00 H
ATOM 1188 HE3 TRP A 76 26.745 −38.267 −33.771 1.00 0.00 H
ATOM 1189 HZ2 TRP A 76 24.538 −41.127 −37.203 1.00 0.00 H
ATOM 1190 HZ3 TRP A 76 25.234 −37.247 −35.465 1.00 0.00 H
ATOM 1191 HH2 TRP A 76 24.147 −38.660 −37.192 1.00 0.00 H
ATOM 1192 HE1 TRP A 76 26.002 −43.167 −35.775 1.00 0.00 H
ATOM 1193 N THR A 77 29.888 −40.215 −35.096 1.00 0.00 N
ATOM 1194 CA THR A 77 30.163 −39.469 −36.333 1.00 0.00 C
ATOM 1195 C THR A 77 29.091 −39.823 −37.373 1.00 0.00 C
ATOM 1196 O THR A 77 28.882 −41.013 −37.620 1.00 0.00 O
ATOM 1197 CB THR A 77 31.572 −39.809 −36.853 1.00 0.00 C
ATOM 1198 OG1 THR A 77 32.533 −39.288 −35.964 1.00 0.00 O
ATOM 1199 CG2 THR A 77 31.900 −39.246 −38.236 1.00 0.00 C
ATOM 1200 H THR A 77 29.656 −41.191 −35.202 1.00 0.00 H
ATOM 1201 HA THR A 77 30.150 −38.404 −36.123 1.00 0.00 H
ATOM 1202 HB THR A 77 31.685 −40.894 −36.892 1.00 0.00 H
ATOM 1203 HG1 THR A 77 33.399 −39.394 −36.366 1.00 0.00 H

ATOM 1204 HG21 THR A 77 32.943 −39.449 −38.479 1.00 0.00 H
ATOM 1205 HG22 THR A 77 31.275 −39.723 −38.991 1.00 0.00 H
ATOM 1206 HG23 THR A 77 31.731 −38.172 −38.257 1.00 0.00 H
ATOM 1207 N PRO A 78 28.410 −38.850 −38.013 1.00 0.00 N
ATOM 1208 CA PRO A 78 27.413 −39.143 −39.041 1.00 0.00 C
ATOM 1209 C PRO A 78 28.034 −39.780 −40.298 1.00 0.00 C
ATOM 1210 O PRO A 78 29.108 −39.345 −40.715 1.00 0.00 O
ATOM 1211 CB PRO A 78 26.752 −37.807 −39.395 1.00 0.00 C
ATOM 1212 CG PRO A 78 27.151 −36.851 −38.277 1.00 0.00 C
ATOM 1213 CD PRO A 78 28.472 −37.420 −37.769 1.00 0.00 C
ATOM 1214 HA PRO A 78 26.674 −39.813 −38.604 1.00 0.00 H
ATOM 1215 HB2 PRO A 78 25.669 −37.904 −39.471 1.00 0.00 H
ATOM 1216 HB3 PRO A 78 27.160 −37.431 −40.330 1.00 0.00 H
ATOM 1217 HG2 PRO A 78 27.265 −35.834 −38.650 1.00 0.00 H
ATOM 1218 HG3 PRO A 78 26.407 −36.877 −37.485 1.00 0.00 H
ATOM 1219 HD2 PRO A 78 28.585 −37.186 −36.710 1.00 0.00 H
ATOM 1220 HD3 PRO A 78 29.297 −37.007 −38.348 1.00 0.00 H
ATOM 1221 N PRO A 79 27.249 −40.545 −41.085 1.00 0.00 N
ATOM 1222 CA PRO A 79 27.665 −41.049 −42.401 1.00 0.00 C
ATOM 1223 C PRO A 79 27.792 −39.964 −43.491 1.00 0.00 C
ATOM 1224 O PRO A 79 28.243 −40.258 −44.595 1.00 0.00 O
ATOM 1225 CB PRO A 79 26.621 −42.110 −42.768 1.00 0.00 C
ATOM 1226 CG PRO A 79 25.361 −41.621 −42.057 1.00 0.00 C
ATOM 1227 CD PRO A 79 25.912 −41.025 −40.763 1.00 0.00 C
ATOM 1228 HA PRO A 79 28.640 −41.531 −42.310 1.00 0.00 H
ATOM 1229 HB2 PRO A 79 26.923 −43.072 −42.351 1.00 0.00 H
ATOM 1230 HB3 PRO A 79 26.472 −42.197 −43.845 1.00 0.00 H
ATOM 1231 HG2 PRO A 79 24.884 −40.839 −42.649 1.00 0.00 H
ATOM 1232 HG3 PRO A 79 24.663 −42.437 −41.861 1.00 0.00 H
ATOM 1233 HD2 PRO A 79 25.984 −41.802 −39.999 1.00 0.00 H

ATOM 1234 HD3 PRO A 79 25.264 −40.218 −40.419 1.00 0.00 H
ATOM 1235 N ASN A 80 27.436 −38.707 −43.199 1.00 0.00 N
ATOM 1236 CA ASN A 80 27.847 −37.546 −43.987 1.00 0.00 C
ATOM 1237 C ASN A 80 28.017 −36.308 −43.075 1.00 0.00 C
ATOM 1238 O ASN A 80 27.078 −35.952 −42.360 1.00 0.00 O
ATOM 1239 CB ASN A 80 26.834 −37.295 −45.115 1.00 0.00 C
ATOM 1240 CG ASN A 80 27.359 −36.266 −46.097 1.00 0.00 C
ATOM 1241 OD1 ASN A 80 27.651 −35.133 −45.750 1.00 0.00 O
ATOM 1242 ND2 ASN A 80 27.756 −36.689 −47.273 1.00 0.00 N
ATOM 1243 H ASN A 80 27.096 −38.523 −42.269 1.00 0.00 H
ATOM 1244 HA ASN A 80 28.803 −37.788 −44.451 1.00 0.00 H
ATOM 1245 HB2 ASN A 80 26.638 −38.228 −45.643 1.00 0.00 H
ATOM 1246 HB3 ASN A 80 25.893 −36.935 −44.700 1.00 0.00 H
ATOM 1247 HD21 ASN A 80 27.652 −37.655 −47.524 1.00 0.00 H
ATOM 1248 HD22 ASN A 80 28.212 −36.014 −47.861 1.00 0.00 H
ATOM 1249 N PRO A 81 29.185 −35.636 −43.073 1.00 0.00 N
ATOM 1250 CA PRO A 81 29.442 −34.505 −42.182 1.00 0.00 C
ATOM 1251 C PRO A 81 28.842 −33.176 −42.669 1.00 0.00 C
ATOM 1252 O PRO A 81 28.701 −32.248 −41.878 1.00 0.00 O
ATOM 1253 CB PRO A 81 30.966 −34.435 −42.125 1.00 0.00 C
ATOM 1254 CG PRO A 81 31.396 −34.856 −43.528 1.00 0.00 C
ATOM 1255 CD PRO A 81 30.382 −35.955 −43.846 1.00 0.00 C
ATOM 1256 HA PRO A 81 29.050 −34.711 −41.185 1.00 0.00 H
ATOM 1257 HB2 PRO A 81 31.305 −35.187 −41.416 1.00 0.00 H
ATOM 1258 HB3 PRO A 81 31.335 −33.447 −41.851 1.00 0.00 H
ATOM 1259 HG2 PRO A 81 31.279 −34.026 −44.224 1.00 0.00 H
ATOM 1260 HG3 PRO A 81 32.418 −35.233 −43.547 1.00 0.00 H
ATOM 1261 HD2 PRO A 81 30.777 −36.920 −43.522 1.00 0.00 H
ATOM 1262 HD3 PRO A 81 30.174 −35.972 −44.917 1.00 0.00 H
ATOM 1263 N SER A 82 28.540 −33.051 −43.965 1.00 0.00 N

ATOM 1264 CA SER A 82 28.065 −31.816 −44.599 1.00 0.00 C
ATOM 1265 C SER A 82 26.572 −31.547 −44.395 1.00 0.00 C
ATOM 1266 O SER A 82 26.124 −30.445 −44.696 1.00 0.00 O
ATOM 1267 CB SER A 82 28.344 −31.869 −46.106 1.00 0.00 C
ATOM 1268 OG SER A 82 29.722 −32.086 −46.353 1.00 0.00 O
ATOM 1269 H SER A 82 28.644 −33.861 −44.569 1.00 0.00 H
ATOM 1270 HA SER A 82 28.606 −30.970 −44.175 1.00 0.00 H
ATOM 1271 HB2 SER A 82 28.038 −30.929 −46.568 1.00 0.00 H
ATOM 1272 HB3 SER A 82 27.768 −32.679 −46.554 1.00 0.00 H
ATOM 1273 HG SER A 82 30.202 −31.285 −46.139 1.00 0.00 H
ATOM 1274 N THR A 83 25.807 −32.544 −43.940 1.00 0.00 N
ATOM 1275 CA THR A 83 24.331 −32.536 −43.904 1.00 0.00 C
ATOM 1276 C THR A 83 23.748 −32.495 −42.483 1.00 0.00 C
ATOM 1277 O THR A 83 22.591 −32.871 −42.299 1.00 0.00 O
ATOM 1278 CB THR A 83 23.777 −33.749 −44.679 1.00 0.00 C
ATOM 1279 OG1 THR A 83 24.192 −34.950 −44.067 1.00 0.00 O
ATOM 1280 CG2 THR A 83 24.235 −33.792 −46.137 1.00 0.00 C
ATOM 1281 H THR A 83 26.250 −33.443 −43.801 1.00 0.00 H
ATOM 1282 HA THR A 83 23.966 −31.639 −44.405 1.00 0.00 H
ATOM 1283 HB THR A 83 22.688 −33.719 −44.667 1.00 0.00 H
ATOM 1284 HG21 THR A 83 23.740 −34.620 −46.644 1.00 0.00 H
ATOM 1285 HG22 THR A 83 25.313 −33.932 −46.196 1.00 0.00 H
ATOM 1286 HG23 THR A 83 23.966 −32.859 −46.633 1.00 0.00 H
ATOM 1287 HG1 THR A 83 23.721 −35.017 −43.231 1.00 0.00 H
ATOM 1288 N ILE A 84 24.568 −32.200 −41.464 1.00 0.00 N
ATOM 1289 CA ILE A 84 24.195 −32.190 −40.036 1.00 0.00 C
ATOM 1290 C ILE A 84 24.608 −30.855 −39.401 1.00 0.00 C
ATOM 1291 O ILE A 84 25.792 −30.503 −39.420 1.00 0.00 O
ATOM 1292 CB ILE A 84 24.820 −33.401 −39.299 1.00 0.00 C
ATOM 1293 CG1 ILE A 84 24.274 −34.758 −39.803 1.00 0.00 C

ATOM 1294 CG2 ILE A 84 24.653 −33.303 −37.770 1.00 0.00 C
ATOM 1295 CD1 ILE A 84 22.788 −35.029 −39.526 1.00 0.00 C
ATOM 1296 H ILE A 84 25.499 −31.887 −41.700 1.00 0.00 H
ATOM 1297 HA ILE A 84 23.113 −32.261 −39.952 1.00 0.00 H
ATOM 1298 HB ILE A 84 25.892 −33.394 −39.503 1.00 0.00 H
ATOM 1299 HG12 ILE A 84 24.846 −35.554 −39.329 1.00 0.00 H
ATOM 1300 HG13 ILE A 84 24.449 −34.840 −40.876 1.00 0.00 H
ATOM 1301 HG21 ILE A 84 25.232 −32.469 −37.371 1.00 0.00 H
ATOM 1302 HG22 ILE A 84 23.604 −33.154 −37.515 1.00 0.00 H
ATOM 1303 HG23 ILE A 84 25.006 −34.218 −37.294 1.00 0.00 H
ATOM 1304 HD11 ILE A 84 22.513 −35.993 −39.954 1.00 0.00 H
ATOM 1305 HD12 ILE A 84 22.171 −34.255 −39.981 1.00 0.00 H
ATOM 1306 HD13 ILE A 84 22.598 −35.060 −38.453 1.00 0.00 H
ATOM 1307 N HIS A 85 23.664 −30.163 −38.755 1.00 0.00 N
ATOM 1308 CA HIS A 85 23.795 −28.751 −38.366 1.00 0.00 C
ATOM 1309 C HIS A 85 23.184 −28.450 −36.986 1.00 0.00 C
ATOM 1310 O HIS A 85 22.183 −29.059 −36.604 1.00 0.00 O
ATOM 1311 CB HIS A 85 23.124 −27.860 −39.432 1.00 0.00 C
ATOM 1312 CG HIS A 85 23.448 −28.223 −40.858 1.00 0.00 C
ATOM 1313 ND1 HIS A 85 24.583 −27.875 −41.551 1.00 0.00 N
ATOM 1314 CD2 HIS A 85 22.744 −29.094 −41.645 1.00 0.00 C
ATOM 1315 CE1 HIS A 85 24.558 −28.511 −42.733 1.00 0.00 C
ATOM 1316 NE2 HIS A 85 23.461 −29.275 −42.830 1.00 0.00 N
ATOM 1317 H HIS A 85 22.721 −30.544 −38.758 1.00 0.00 H
ATOM 1318 HA HIS A 85 24.851 −28.486 −38.326 1.00 0.00 H
ATOM 1319 HB2 HIS A 85 22.043 −27.908 −39.311 1.00 0.00 H
ATOM 1320 HB3 HIS A 85 23.430 −26.828 −39.265 1.00 0.00 H
ATOM 1321 HD2 HIS A 85 21.832 −29.605 −41.366 1.00 0.00 H
ATOM 1322 HE1 HIS A 85 25.318 −28.433 −43.500 1.00 0.00 H
ATOM 1323 HD1 HIS A 85 25.298 −27.238 −41.235 1.00 0.00 H

ATOM	1324	N	HIS	A	86	23.709	−27.410	−36.324	1.00	0.00		N
ATOM	1325	CA	HIS	A	86	23.065	−26.677	−35.219	1.00	0.00		C
ATOM	1326	C	HIS	A	86	22.537	−27.561	−34.072	1.00	0.00		C
ATOM	1327	O	HIS	A	86	21.420	−27.382	−33.588	1.00	0.00		O
ATOM	1328	CB	HIS	A	86	22.010	−25.694	−35.756	1.00	0.00		C
ATOM	1329	CG	HIS	A	86	22.453	−24.893	−36.956	1.00	0.00		C
ATOM	1330	ND1	HIS	A	86	23.614	−24.157	−37.090	1.00	0.00		N
ATOM	1331	CD2	HIS	A	86	21.812	−24.856	−38.164	1.00	0.00		C
ATOM	1332	CE1	HIS	A	86	23.649	−23.667	−38.343	1.00	0.00		C
ATOM	1333	NE2	HIS	A	86	22.577	−24.080	−39.036	1.00	0.00		N
ATOM	1334	H	HIS	A	86	24.543	−26.980	−36.704	1.00	0.00		H
ATOM	1335	HA	HIS	A	86	23.850	−26.068	−34.769	1.00	0.00		H
ATOM	1336	HB2	HIS	A	86	21.117	−26.258	−36.032	1.00	0.00		H
ATOM	1337	HB3	HIS	A	86	21.729	−25.004	−34.960	1.00	0.00		H
ATOM	1338	HD2	HIS	A	86	20.892	−25.367	−38.409	1.00	0.00		H
ATOM	1339	HE1	HIS	A	86	24.437	−23.046	−38.753	1.00	0.00		H
ATOM	1340	HD1	HIS	A	86	24.350	−24.049	−36.403	1.00	0.00		H
ATOM	1341	N	CYS	A	87	23.316	−28.577	−33.692	1.00	0.00		N
ATOM	1342	CA	CYS	A	87	22.938	−29.526	−32.649	1.00	0.00		C
ATOM	1343	C	CYS	A	87	22.659	−28.844	−31.295	1.00	0.00		C
ATOM	1344	O	CYS	A	87	23.218	−27.791	−30.989	1.00	0.00		O
ATOM	1345	CB	CYS	A	87	24.023	−30.599	−32.526	1.00	0.00		C
ATOM	1346	SG	CYS	A	87	24.293	−31.608	−34.012	1.00	0.00		S
ATOM	1347	H	CYS	A	87	24.207	−28.690	−34.146	1.00	0.00		H
ATOM	1348	HA	CYS	A	87	22.022	−30.012	−32.969	1.00	0.00		H
ATOM	1349	HB2	CYS	A	87	23.763	−31.270	−31.705	1.00	0.00		H
ATOM	1350	HB3	CYS	A	87	24.961	−30.107	−32.267	1.00	0.00		H
ATOM	1351	N	SER	A	88	21.868	−29.506	−30.449	1.00	0.00		N
ATOM	1352	CA	SER	A	88	21.390	−28.989	−29.164	1.00	0.00		C
ATOM	1353	C	SER	A	88	21.182	−30.116	−28.155	1.00	0.00		C

ATOM 1354 O SER A 88 20.615 −31.137 −28.526 1.00 0.00 O
ATOM 1355 CB SER A 88 20.058 −28.282 −29.403 1.00 0.00 C
ATOM 1356 OG SER A 88 19.662 −27.658 −28.205 1.00 0.00 O
ATOM 1357 H SER A 88 21.394 −30.334 −30.803 1.00 0.00 H
ATOM 1358 HA SER A 88 22.121 −28.294 −28.760 1.00 0.00 H
ATOM 1359 HB2 SER A 88 20.176 −27.522 −30.177 1.00 0.00 H
ATOM 1360 HB3 SER A 88 19.301 −29.000 −29.711 1.00 0.00 H
ATOM 1361 HG SER A 88 20.285 −26.913 −28.103 1.00 0.00 H
ATOM 1362 N SER A 89 21.634 −29.979 −26.904 1.00 0.00 N
ATOM 1363 CA SER A 89 21.833 −31.136 −26.010 1.00 0.00 C
ATOM 1364 C SER A 89 21.270 −30.982 −24.589 1.00 0.00 C
ATOM 1365 O SER A 89 20.991 −29.875 −24.131 1.00 0.00 O
ATOM 1366 CB SER A 89 23.321 −31.493 −25.993 1.00 0.00 C
ATOM 1367 OG SER A 89 24.068 −30.503 −25.323 1.00 0.00 O
ATOM 1368 H SER A 89 22.062 −29.099 −26.636 1.00 0.00 H
ATOM 1369 HA SER A 89 21.318 −31.991 −26.438 1.00 0.00 H
ATOM 1370 HB2 SER A 89 23.459 −32.446 −25.482 1.00 0.00 H
ATOM 1371 HB3 SER A 89 23.684 −31.593 −27.016 1.00 0.00 H
ATOM 1372 HG SER A 89 23.939 −29.631 −25.743 1.00 0.00 H
ATOM 1373 N THR A 90 20.986 −32.108 −23.922 1.00 0.00 N
ATOM 1374 CA THR A 90 20.414 −32.200 −22.558 1.00 0.00 C
ATOM 1375 C THR A 90 20.697 −33.580 −21.939 1.00 0.00 C
ATOM 1376 O THR A 90 20.427 −34.594 −22.583 1.00 0.00 O
ATOM 1377 CB THR A 90 18.891 −31.980 −22.630 1.00 0.00 C
ATOM 1378 OG1 THR A 90 18.615 −30.636 −22.942 1.00 0.00 O
ATOM 1379 CG2 THR A 90 18.153 −32.261 −21.325 1.00 0.00 C
ATOM 1380 H THR A 90 21.140 −32.989 −24.406 1.00 0.00 H
ATOM 1381 HA THR A 90 20.850 −31.433 −21.915 1.00 0.00 H
ATOM 1382 HB THR A 90 18.473 −32.618 −23.410 1.00 0.00 H
ATOM 1383 HG1 THR A 90 19.369 −30.297 −23.452 1.00 0.00 H

ATOM 1384 HG21 THR A 90 18.583 −31.657 −20.526 1.00 0.00 H
ATOM 1385 HG22 THR A 90 18.222 −33.316 −21.064 1.00 0.00 H
ATOM 1386 HG23 THR A 90 17.100 −32.011 −21.456 1.00 0.00 H
ATOM 1387 N TYR A 91 21.145 −33.650 −20.677 1.00 0.00 N
ATOM 1388 CA TYR A 91 21.287 −34.919 −19.944 1.00 0.00 C
ATOM 1389 C TYR A 91 19.981 −35.370 −19.275 1.00 0.00 C
ATOM 1390 O TYR A 91 19.447 −34.636 −18.437 1.00 0.00 O
ATOM 1391 CB TYR A 91 22.385 −34.801 −18.881 1.00 0.00 C
ATOM 1392 CG TYR A 91 22.529 −36.011 −17.974 1.00 0.00 C
ATOM 1393 CD1 TYR A 91 23.097 −37.195 −18.477 1.00 0.00 C
ATOM 1394 CD2 TYR A 91 22.058 −35.969 −16.646 1.00 0.00 C
ATOM 1395 CE1 TYR A 91 23.179 −38.337 −17.660 1.00 0.00 C
ATOM 1396 CE2 TYR A 91 22.133 −37.110 −15.827 1.00 0.00 C
ATOM 1397 CZ TYR A 91 22.692 −38.299 −16.337 1.00 0.00 C
ATOM 1398 OH TYR A 91 22.755 −39.405 −15.553 1.00 0.00 O
ATOM 1399 H TYR A 91 21.417 −32.791 −20.195 1.00 0.00 H
ATOM 1400 HA TYR A 91 21.592 −35.697 −20.640 1.00 0.00 H
ATOM 1401 HB2 TYR A 91 22.147 −33.946 −18.261 1.00 0.00 H
ATOM 1402 HB3 TYR A 91 23.336 −34.614 −19.371 1.00 0.00 H
ATOM 1403 HD1 TYR A 91 23.459 −37.228 −19.496 1.00 0.00 H
ATOM 1404 HD2 TYR A 91 21.632 −35.066 −16.248 1.00 0.00 H
ATOM 1405 HE1 TYR A 91 23.618 −39.245 −18.038 1.00 0.00 H
ATOM 1406 HE2 TYR A 91 21.757 −37.080 −14.816 1.00 0.00 H
ATOM 1407 HH TYR A 91 22.729 −39.202 −14.618 1.00 0.00 H
ATOM 1408 N HIS A 92 19.656 −36.657 −19.398 1.00 0.00 N
ATOM 1409 CA HIS A 92 18.632 −37.365 −18.623 1.00 0.00 C
ATOM 1410 C HIS A 92 19.028 −38.845 −18.440 1.00 0.00 C
ATOM 1411 O HIS A 92 19.458 −39.490 −19.390 1.00 0.00 O
ATOM 1412 CB HIS A 92 17.262 −37.163 −19.304 1.00 0.00 C
ATOM 1413 CG HIS A 92 16.217 −38.180 −18.927 1.00 0.00 C

ATOM 1414 ND1 HIS A 92 16.001 −39.401 −19.576 1.00 0.00 N
ATOM 1415 CD2 HIS A 92 15.449 −38.127 −17.804 1.00 0.00 C
ATOM 1416 CE1 HIS A 92 15.092 −40.046 −18.822 1.00 0.00 C
ATOM 1417 NE2 HIS A 92 14.749 −39.308 −17.754 1.00 0.00 N
ATOM 1418 H HIS A 92 20.220 −37.236 −20.017 1.00 0.00 H
ATOM 1419 HA HIS A 92 18.572 −36.925 −17.630 1.00 0.00 H
ATOM 1420 HB2 HIS A 92 16.889 −36.171 −19.051 1.00 0.00 H
ATOM 1421 HB3 HIS A 92 17.380 −37.188 −20.384 1.00 0.00 H
ATOM 1422 HD2 HIS A 92 15.440 −37.346 −17.059 1.00 0.00 H
ATOM 1423 HE1 HIS A 92 14.743 −41.056 −18.995 1.00 0.00 H
ATOM 1424 HE2 HIS A 92 14.274 −39.680 −16.923 1.00 0.00 H
ATOM 1425 N GLU A 93 19.054 −39.300 −17.181 1.00 0.00 N
ATOM 1426 CA GLU A 93 19.219 −40.703 −16.737 1.00 0.00 C
ATOM 1427 C GLU A 93 20.207 −41.561 −17.562 1.00 0.00 C
ATOM 1428 O GLU A 93 19.822 −42.365 −18.404 1.00 0.00 O
ATOM 1429 CB GLU A 93 17.835 −41.356 −16.555 1.00 0.00 C
ATOM 1430 CG GLU A 93 17.053 −40.651 −15.429 1.00 0.00 C
ATOM 1431 CD GLU A 93 15.624 −41.183 −15.266 1.00 0.00 C
ATOM 1432 OE1 GLU A 93 15.458 −42.420 −15.209 1.00 0.00 O
ATOM 1433 OE2 GLU A 93 14.707 −40.332 −15.183 1.00 0.00 O
ATOM 1434 H GLU A 93 18.656 −38.687 −16.491 1.00 0.00 H
ATOM 1435 HA GLU A 93 19.656 −40.670 −15.740 1.00 0.00 H
ATOM 1436 HB2 GLU A 93 17.277 −41.300 −17.490 1.00 0.00 H
ATOM 1437 HB3 GLU A 93 17.970 −42.404 −16.287 1.00 0.00 H
ATOM 1438 HG2 GLU A 93 17.010 −39.577 −15.630 1.00 0.00 H
ATOM 1439 HG3 GLU A 93 17.596 −40.782 −14.490 1.00 0.00 H
ATOM 1440 N ASP A 94 21.507 −41.383 −17.299 1.00 0.00 N
ATOM 1441 CA ASP A 94 22.657 −41.976 −18.005 1.00 0.00 C
ATOM 1442 C ASP A 94 22.823 −41.631 −19.496 1.00 0.00 C
ATOM 1443 O ASP A 94 23.880 −41.936 −20.052 1.00 0.00 O

ATOM 1444 CB ASP A 94 22.789 −43.492 −17.757 1.00 0.00 C
ATOM 1445 CG ASP A 94 23.340 −43.884 −16.386 1.00 0.00 C
ATOM 1446 OD1 ASP A 94 24.017 −43.046 −15.742 1.00 0.00 O
ATOM 1447 OD2 ASP A 94 23.561 −45.104 −16.227 1.00 0.00 O
ATOM 1448 H ASP A 94 21.744 −40.704 −16.588 1.00 0.00 H
ATOM 1449 HA ASP A 94 23.536 −41.525 −17.548 1.00 0.00 H
ATOM 1450 HB2 ASP A 94 23.474 −43.906 −18.496 1.00 0.00 H
ATOM 1451 HB3 ASP A 94 21.826 −43.980 −17.911 1.00 0.00 H
ATOM 1452 N PHE A 95 21.958 −40.813 −20.098 1.00 0.00 N
ATOM 1453 CA PHE A 95 22.097 −40.379 −21.491 1.00 0.00 C
ATOM 1454 C PHE A 95 22.221 −38.860 −21.615 1.00 0.00 C
ATOM 1455 O PHE A 95 21.340 −38.113 −21.195 1.00 0.00 O
ATOM 1456 CB PHE A 95 20.932 −40.910 −22.336 1.00 0.00 C
ATOM 1457 CG PHE A 95 20.602 −42.374 −22.111 1.00 0.00 C
ATOM 1458 CD1 PHE A 95 21.484 −43.379 −22.547 1.00 0.00 C
ATOM 1459 CD2 PHE A 95 19.443 −42.725 −21.395 1.00 0.00 C
ATOM 1460 CE1 PHE A 95 21.209 −44.728 −22.267 1.00 0.00 C
ATOM 1461 CE2 PHE A 95 19.175 −44.073 −21.102 1.00 0.00 C
ATOM 1462 CZ PHE A 95 20.058 −45.076 −21.538 1.00 0.00 C
ATOM 1463 H PHE A 95 21.096 −40.573 −19.613 1.00 0.00 H
ATOM 1464 HA PHE A 95 23.004 −40.810 −21.909 1.00 0.00 H
ATOM 1465 HB2 PHE A 95 20.048 −40.322 −22.102 1.00 0.00 H
ATOM 1466 HB3 PHE A 95 21.163 −40.751 −23.390 1.00 0.00 H
ATOM 1467 HD1 PHE A 95 22.389 −43.114 −23.070 1.00 0.00 H
ATOM 1468 HD2 PHE A 95 18.802 −41.953 −20.986 1.00 0.00 H
ATOM 1469 HE1 PHE A 95 21.910 −45.489 −22.576 1.00 0.00 H
ATOM 1470 HE2 PHE A 95 18.329 −44.319 −20.474 1.00 0.00 H
ATOM 1471 HZ PHE A 95 19.870 −46.104 −21.269 1.00 0.00 H
ATOM 1472 N TYR A 96 23.187 −38.399 −22.409 1.00 0.00 N
ATOM 1473 CA TYR A 96 23.070 −37.093 −23.052 1.00 0.00 C

ATOM	1474	C	TYR	A	96	22.317	−37.261	−24.378	1.00	0.00	C
ATOM	1475	O	TYR	A	96	22.768	−37.948	−25.301	1.00	0.00	O
ATOM	1476	CB	TYR	A	96	24.439	−36.415	−23.192	1.00	0.00	C
ATOM	1477	CG	TYR	A	96	24.534	−35.090	−22.456	1.00	0.00	C
ATOM	1478	CD1	TYR	A	96	25.375	−34.950	−21.333	1.00	0.00	C
ATOM	1479	CD2	TYR	A	96	23.805	−33.983	−22.933	1.00	0.00	C
ATOM	1480	CE1	TYR	A	96	25.460	−33.709	−20.669	1.00	0.00	C
ATOM	1481	CE2	TYR	A	96	23.900	−32.742	−22.276	1.00	0.00	C
ATOM	1482	CZ	TYR	A	96	24.709	−32.609	−21.131	1.00	0.00	C
ATOM	1483	OH	TYR	A	96	24.703	−31.442	−20.444	1.00	0.00	O
ATOM	1484	H	TYR	A	96	23.824	−39.070	−22.826	1.00	0.00	H
ATOM	1485	HA	TYR	A	96	22.469	−36.437	−22.424	1.00	0.00	H
ATOM	1486	HB2	TYR	A	96	25.216	−37.080	−22.816	1.00	0.00	H
ATOM	1487	HB3	TYR	A	96	24.639	−36.225	−24.248	1.00	0.00	H
ATOM	1488	HD1	TYR	A	96	25.954	−35.788	−20.974	1.00	0.00	H
ATOM	1489	HD2	TYR	A	96	23.179	−34.087	−23.807	1.00	0.00	H
ATOM	1490	HE1	TYR	A	96	26.068	−33.594	−19.784	1.00	0.00	H
ATOM	1491	HE2	TYR	A	96	23.364	−31.877	−22.634	1.00	0.00	H
ATOM	1492	HH	TYR	A	96	23.810	−31.267	−20.096	1.00	0.00	H
ATOM	1493	N	TYR	A	97	21.108	−36.709	−24.428	1.00	0.00	N
ATOM	1494	CA	TYR	A	97	20.309	−36.566	−25.640	1.00	0.00	C
ATOM	1495	C	TYR	A	97	20.795	−35.330	−26.407	1.00	0.00	C
ATOM	1496	O	TYR	A	97	21.166	−34.322	−25.800	1.00	0.00	O
ATOM	1497	CB	TYR	A	97	18.822	−36.437	−25.275	1.00	0.00	C
ATOM	1498	CG	TYR	A	97	18.215	−37.618	−24.529	1.00	0.00	C
ATOM	1499	CD1	TYR	A	97	17.333	−38.505	−25.182	1.00	0.00	C
ATOM	1500	CD2	TYR	A	97	18.480	−37.796	−23.156	1.00	0.00	C
ATOM	1501	CE1	TYR	A	97	16.731	−39.566	−24.473	1.00	0.00	C
ATOM	1502	CE2	TYR	A	97	17.884	−38.857	−22.451	1.00	0.00	C
ATOM	1503	CZ	TYR	A	97	17.000	−39.736	−23.099	1.00	0.00	C

ATOM 1504 OH TYR A 97 16.403 −40.728 −22.390 1.00 0.00 O
ATOM 1505 H TYR A 97 20.815 −36.155 −23.629 1.00 0.00 H
ATOM 1506 HA TYR A 97 20.436 −37.446 −26.270 1.00 0.00 H
ATOM 1507 HB2 TYR A 97 18.692 −35.542 −24.663 1.00 0.00 H
ATOM 1508 HB3 TYR A 97 18.261 −36.281 −26.194 1.00 0.00 H
ATOM 1509 HD1 TYR A 97 17.082 −38.350 −26.218 1.00 0.00 H
ATOM 1510 HD2 TYR A 97 19.132 −37.114 −22.625 1.00 0.00 H
ATOM 1511 HE1 TYR A 97 16.030 −40.230 −24.953 1.00 0.00 H
ATOM 1512 HE2 TYR A 97 18.101 −39.007 −21.404 1.00 0.00 H
ATOM 1513 HH TYR A 97 16.318 −40.466 −21.459 1.00 0.00 H
ATOM 1514 N THR A 98 20.795 −35.383 −27.739 1.00 0.00 N
ATOM 1515 CA THR A 98 21.269 −34.285 −28.588 1.00 0.00 C
ATOM 1516 C THR A 98 20.570 −34.264 −29.951 1.00 0.00 C
ATOM 1517 O THR A 98 20.570 −35.240 −30.698 1.00 0.00 O
ATOM 1518 CB THR A 98 22.803 −34.281 −28.676 1.00 0.00 C
ATOM 1519 OG1 THR A 98 23.254 −33.191 −29.446 1.00 0.00 O
ATOM 1520 CG2 THR A 98 23.419 −35.541 −29.275 1.00 0.00 C
ATOM 1521 H THR A 98 20.556 −36.259 −28.196 1.00 0.00 H
ATOM 1522 HA THR A 98 21.000 −33.368 −28.083 1.00 0.00 H
ATOM 1523 HB THR A 98 23.195 −34.164 −27.665 1.00 0.00 H
ATOM 1524 HG1 THR A 98 24.213 −33.218 −29.435 1.00 0.00 H
ATOM 1525 HG21 THR A 98 24.487 −35.544 −29.073 1.00 0.00 H
ATOM 1526 HG22 THR A 98 23.238 −35.572 −30.349 1.00 0.00 H
ATOM 1527 HG23 THR A 98 22.994 −36.428 −28.807 1.00 0.00 H
ATOM 1528 N LEU A 99 19.791 −33.206 −30.167 1.00 0.00 N
ATOM 1529 CA LEU A 99 18.923 −32.956 −31.312 1.00 0.00 C
ATOM 1530 C LEU A 99 19.660 −32.108 −32.361 1.00 0.00 C
ATOM 1531 O LEU A 99 20.055 −30.983 −32.065 1.00 0.00 O
ATOM 1532 CB LEU A 99 17.666 −32.258 −30.755 1.00 0.00 C
ATOM 1533 CG LEU A 99 16.650 −31.785 −31.805 1.00 0.00 C

ATOM	1534	CD1	LEU	A	99	16.073	−32.946	−32.608	1.00	0.00	C
ATOM	1535	CD2	LEU	A	99	15.499	−31.051	−31.120	1.00	0.00	C
ATOM	1536	H	LEU	A	99	19.866	−32.450	−29.494	1.00	0.00	H
ATOM	1537	HA	LEU	A	99	18.635	−33.903	−31.766	1.00	0.00	H
ATOM	1538	HB2	LEU	A	99	17.166	−32.935	−30.061	1.00	0.00	H
ATOM	1539	HB3	LEU	A	99	17.985	−31.383	−30.184	1.00	0.00	H
ATOM	1540	HG	LEU	A	99	17.130	−31.099	−32.495	1.00	0.00	H
ATOM	1541	HD11	LEU	A	99	16.861	−33.447	−33.164	1.00	0.00	H
ATOM	1542	HD12	LEU	A	99	15.591	−33.654	−31.935	1.00	0.00	H
ATOM	1543	HD13	LEU	A	99	15.348	−32.563	−33.320	1.00	0.00	H
ATOM	1544	HD21	LEU	A	99	15.886	−30.192	−30.570	1.00	0.00	H
ATOM	1545	HD22	LEU	A	99	14.788	−30.701	−31.865	1.00	0.00	H
ATOM	1546	HD23	LEU	A	99	14.996	−31.718	−30.420	1.00	0.00	H
ATOM	1547	N	CYS	A	100	19.743	−32.584	−33.603	1.00	0.00	N
ATOM	1548	CA	CYS	A	100	20.414	−31.916	−34.724	1.00	0.00	C
ATOM	1549	C	CYS	A	100	19.472	−31.741	−35.927	1.00	0.00	C
ATOM	1550	O	CYS	A	100	18.683	−32.636	−36.255	1.00	0.00	O
ATOM	1551	CB	CYS	A	100	21.642	−32.731	−35.147	1.00	0.00	C
ATOM	1552	SG	CYS	A	100	22.896	−33.093	−33.884	1.00	0.00	S
ATOM	1553	H	CYS	A	100	19.356	−33.504	−33.785	1.00	0.00	H
ATOM	1554	HA	CYS	A	100	20.748	−30.924	−34.419	1.00	0.00	H
ATOM	1555	HB2	CYS	A	100	21.282	−33.691	−35.523	1.00	0.00	H
ATOM	1556	HB3	CYS	A	100	22.131	−32.215	−35.973	1.00	0.00	H
ATOM	1557	N	ALA	A	101	19.672	−30.665	−36.687	1.00	0.00	N
ATOM	1558	CA	ALA	A	101	19.045	−30.474	−37.993	1.00	0.00	C
ATOM	1559	C	ALA	A	101	19.756	−31.302	−39.077	1.00	0.00	C
ATOM	1560	O	ALA	A	101	20.987	−31.284	−39.165	1.00	0.00	O
ATOM	1561	CB	ALA	A	101	19.064	−28.979	−38.318	1.00	0.00	C
ATOM	1562	H	ALA	A	101	20.419	−30.022	−36.439	1.00	0.00	H
ATOM	1563	HA	ALA	A	101	18.005	−30.799	−37.937	1.00	0.00	H

ATOM 1564 HB1 ALA A 101 20.088 −28.641 −38.460 1.00 0.00 H
ATOM 1565 HB2 ALA A 101 18.632 −28.425 −37.488 1.00 0.00 H
ATOM 1566 HB3 ALA A 101 18.491 −28.784 −39.225 1.00 0.00 H
ATOM 1567 N VAL A 102 18.984 −31.951 −39.952 1.00 0.00 N
ATOM 1568 CA VAL A 102 19.468 −32.679 −41.135 1.00 0.00 C
ATOM 1569 C VAL A 102 19.127 −31.875 −42.390 1.00 0.00 C
ATOM 1570 O VAL A 102 18.004 −31.390 −42.515 1.00 0.00 O
ATOM 1571 CB VAL A 102 18.851 −34.090 −41.228 1.00 0.00 C
ATOM 1572 CG1 VAL A 102 19.548 −34.932 −42.305 1.00 0.00 C
ATOM 1573 CG2 VAL A 102 18.906 −34.861 −39.904 1.00 0.00 C
ATOM 1574 H VAL A 102 17.974 −31.892 −39.849 1.00 0.00 H
ATOM 1575 HA VAL A 102 20.547 −32.785 −41.066 1.00 0.00 H
ATOM 1576 HB VAL A 102 17.804 −33.996 −41.505 1.00 0.00 H
ATOM 1577 HG11 VAL A 102 19.419 −34.475 −43.285 1.00 0.00 H
ATOM 1578 HG12 VAL A 102 20.613 −35.014 −42.082 1.00 0.00 H
ATOM 1579 HG13 VAL A 102 19.112 −35.929 −42.333 1.00 0.00 H
ATOM 1580 HG21 VAL A 102 18.294 −34.358 −39.155 1.00 0.00 H
ATOM 1581 HG22 VAL A 102 18.506 −35.865 −40.042 1.00 0.00 H
ATOM 1582 HG23 VAL A 102 19.933 −34.928 −39.548 1.00 0.00 H
ATOM 1583 N SER A 103 20.038 −31.756 −43.361 1.00 0.00 N
ATOM 1584 CA SER A 103 19.801 −30.930 −44.556 1.00 0.00 C
ATOM 1585 C SER A 103 20.377 −31.517 −45.842 1.00 0.00 C
ATOM 1586 O SER A 103 21.553 −31.866 −45.909 1.00 0.00 O
ATOM 1587 CB SER A 103 20.364 −29.529 −44.337 1.00 0.00 C
ATOM 1588 OG SER A 103 20.047 −28.697 −45.429 1.00 0.00 O
ATOM 1589 H SER A 103 20.962 −32.151 −43.212 1.00 0.00 H
ATOM 1590 HA SER A 103 18.727 −30.823 −44.707 1.00 0.00 H
ATOM 1591 HB2 SER A 103 19.955 −29.104 −43.421 1.00 0.00 H
ATOM 1592 HB3 SER A 103 21.444 −29.596 −44.254 1.00 0.00 H
ATOM 1593 HG SER A 103 20.532 −27.860 −45.253 1.00 0.00 H

ATOM 1594 N HIS A 104 19.592 −31.448 −46.923 1.00 0.00 N
ATOM 1595 CA HIS A 104 20.047 −31.680 −48.303 1.00 0.00 C
ATOM 1596 C HIS A 104 20.580 −30.412 −48.995 1.00 0.00 C
ATOM 1597 O HIS A 104 21.123 −30.503 −50.091 1.00 0.00 O
ATOM 1598 CB HIS A 104 18.896 −32.315 −49.116 1.00 0.00 C
ATOM 1599 CG HIS A 104 19.210 −33.639 −49.769 1.00 0.00 C
ATOM 1600 ND1 HIS A 104 18.514 −34.157 −50.861 1.00 0.00 N
ATOM 1601 CD2 HIS A 104 20.090 −34.589 −49.328 1.00 0.00 C
ATOM 1602 CE1 HIS A 104 19.014 −35.380 −51.082 1.00 0.00 C
ATOM 1603 NE2 HIS A 104 19.964 −35.666 −50.177 1.00 0.00 N
ATOM 1604 H HIS A 104 18.648 −31.116 −46.794 1.00 0.00 H
ATOM 1605 HA HIS A 104 20.896 −32.362 −48.281 1.00 0.00 H
ATOM 1606 HB2 HIS A 104 18.031 −32.474 −48.472 1.00 0.00 H
ATOM 1607 HB3 HIS A 104 18.579 −31.622 −49.895 1.00 0.00 H
ATOM 1608 HD2 HIS A 104 20.733 −34.519 −48.463 1.00 0.00 H
ATOM 1609 HE1 HIS A 104 18.682 −36.042 −51.872 1.00 0.00 H
ATOM 1610 HE2 HIS A 104 20.477 −36.535 −50.124 1.00 0.00 H
ATOM 1611 N VAL A 105 20.430 −29.234 −48.374 1.00 0.00 N
ATOM 1612 CA VAL A 105 20.701 −27.912 −48.983 1.00 0.00 C
ATOM 1613 C VAL A 105 21.814 −27.133 −48.266 1.00 0.00 C
ATOM 1614 O VAL A 105 22.018 −25.951 −48.527 1.00 0.00 O
ATOM 1615 CB VAL A 105 19.407 −27.081 −49.114 1.00 0.00 C
ATOM 1616 CG1 VAL A 105 18.363 −27.818 −49.964 1.00 0.00 C
ATOM 1617 CG2 VAL A 105 18.773 −26.731 −47.761 1.00 0.00 C
ATOM 1618 H VAL A 105 20.024 −29.237 −47.447 1.00 0.00 H
ATOM 1619 HA VAL A 105 21.073 −28.069 −49.995 1.00 0.00 H
ATOM 1620 HB VAL A 105 19.642 −26.149 −49.626 1.00 0.00 H
ATOM 1621 HG11 VAL A 105 18.790 −28.068 −50.935 1.00 0.00 H
ATOM 1622 HG12 VAL A 105 17.494 −27.184 −50.118 1.00 0.00 H
ATOM 1623 HG13 VAL A 105 18.036 −28.734 −49.472 1.00 0.00 H

ATOM 1624 HG21 VAL A 105 19.497 −26.221 −47.124 1.00 0.00 H
ATOM 1625 HG22 VAL A 105 18.422 −27.632 −47.261 1.00 0.00 H
ATOM 1626 HG23 VAL A 105 17.929 −26.063 −47.917 1.00 0.00 H
ATOM 1627 N GLY A 106 22.529 −27.778 −47.341 1.00 0.00 N
ATOM 1628 CA GLY A 106 23.466 −27.114 −46.437 1.00 0.00 C
ATOM 1629 C GLY A 106 22.743 −26.305 −45.354 1.00 0.00 C
ATOM 1630 O GLY A 106 21.701 −26.724 −44.861 1.00 0.00 O
ATOM 1631 H GLY A 106 22.290 −28.738 −47.150 1.00 0.00 H
ATOM 1632 HA2 GLY A 106 24.106 −26.445 −47.009 1.00 0.00 H
ATOM 1633 HA3 GLY A 106 24.092 −27.856 −45.945 1.00 0.00 H
ATOM 1634 N ASP A 107 23.335 −25.183 −44.949 1.00 0.00 N
ATOM 1635 CA ASP A 107 22.908 −24.343 −43.821 1.00 0.00 C
ATOM 1636 C ASP A 107 21.398 −23.977 −43.870 1.00 0.00 C
ATOM 1637 O ASP A 107 20.968 −23.246 −44.770 1.00 0.00 O
ATOM 1638 CB ASP A 107 23.832 −23.116 −43.789 1.00 0.00 C
ATOM 1639 CG ASP A 107 23.573 −22.135 −42.639 1.00 0.00 C
ATOM 1640 OD1 ASP A 107 22.392 −21.792 −42.418 1.00 0.00 O
ATOM 1641 OD2 ASP A 107 24.564 −21.501 −42.229 1.00 0.00 O
ATOM 1642 H ASP A 107 24.144 −24.864 −45.456 1.00 0.00 H
ATOM 1643 HA ASP A 107 23.109 −24.885 −42.898 1.00 0.00 H
ATOM 1644 HB2 ASP A 107 24.861 −23.470 −43.713 1.00 0.00 H
ATOM 1645 HB3 ASP A 107 23.740 −22.579 −44.733 1.00 0.00 H
ATOM 1646 N PRO A 108 20.582 −24.454 −42.905 1.00 0.00 N
ATOM 1647 CA PRO A 108 19.149 −24.176 −42.858 1.00 0.00 C
ATOM 1648 C PRO A 108 18.764 −22.710 −42.664 1.00 0.00 C
ATOM 1649 O PRO A 108 17.672 −22.324 −43.072 1.00 0.00 O
ATOM 1650 CB PRO A 108 18.629 −24.989 −41.675 1.00 0.00 C
ATOM 1651 CG PRO A 108 19.568 −26.185 −41.627 1.00 0.00 C
ATOM 1652 CD PRO A 108 20.900 −25.519 −41.956 1.00 0.00 C
ATOM 1653 HA PRO A 108 18.693 −24.544 −43.778 1.00 0.00 H

ATOM 1654 HB2 PRO A 108 17.594 −25.279 −41.810 1.00 0.00 H
ATOM 1655 HB3 PRO A 108 18.742 −24.404 −40.765 1.00 0.00 H
ATOM 1656 HG2 PRO A 108 19.581 −26.661 −40.648 1.00 0.00 H
ATOM 1657 HG3 PRO A 108 19.306 −26.898 −42.409 1.00 0.00 H
ATOM 1658 HD2 PRO A 108 21.581 −26.261 −42.372 1.00 0.00 H
ATOM 1659 HD3 PRO A 108 21.327 −25.081 −41.055 1.00 0.00 H
ATOM 1660 N ILE A 109 19.603 −21.880 −42.039 1.00 0.00 N
ATOM 1661 CA ILE A 109 19.321 −20.449 −41.843 1.00 0.00 C
ATOM 1662 C ILE A 109 19.552 −19.687 −43.150 1.00 0.00 C
ATOM 1663 O ILE A 109 18.659 −18.987 −43.635 1.00 0.00 O
ATOM 1664 CB ILE A 109 20.190 −19.891 −40.690 1.00 0.00 C
ATOM 1665 CG1 ILE A 109 19.950 −20.631 −39.355 1.00 0.00 C
ATOM 1666 CG2 ILE A 109 19.987 −18.375 −40.511 1.00 0.00 C
ATOM 1667 CD1 ILE A 109 18.536 −20.497 −38.769 1.00 0.00 C
ATOM 1668 H ILE A 109 20.554 −22.197 −41.857 1.00 0.00 H
ATOM 1669 HA ILE A 109 18.272 −20.326 −41.581 1.00 0.00 H
ATOM 1670 HB ILE A 109 21.239 −20.039 −40.949 1.00 0.00 H
ATOM 1671 HG12 ILE A 109 20.172 −21.691 −39.483 1.00 0.00 H
ATOM 1672 HG13 ILE A 109 20.664 −20.252 −38.627 1.00 0.00 H
ATOM 1673 HG21 ILE A 109 20.393 −17.839 −41.368 1.00 0.00 H
ATOM 1674 HG22 ILE A 109 20.519 −18.039 −39.621 1.00 0.00 H
ATOM 1675 HG23 ILE A 109 18.928 −18.141 −40.410 1.00 0.00 H
ATOM 1676 HD11 ILE A 109 18.476 −21.052 −37.834 1.00 0.00 H
ATOM 1677 HD12 ILE A 109 18.302 −19.453 −38.565 1.00 0.00 H
ATOM 1678 HD13 ILE A 109 17.805 −20.906 −39.462 1.00 0.00 H
ATOM 1679 N LEU A 110 20.673 −19.970 −43.820 1.00 0.00 N
ATOM 1680 CA LEU A 110 21.036 −19.400 −45.119 1.00 0.00 C
ATOM 1681 C LEU A 110 20.267 −20.019 −46.304 1.00 0.00 C
ATOM 1682 O LEU A 110 20.334 −19.488 −47.415 1.00 0.00 O
ATOM 1683 CB LEU A 110 22.561 −19.507 −45.307 1.00 0.00 C

ATOM 1684 CG LEU A 110 23.409 −18.823 −44.212 1.00 0.00 C
ATOM 1685 CD1 LEU A 110 24.893 −19.021 −44.522 1.00 0.00 C
ATOM 1686 CD2 LEU A 110 23.146 −17.316 −44.123 1.00 0.00 C
ATOM 1687 H LEU A 110 21.330 −20.614 −43.371 1.00 0.00 H
ATOM 1688 HA LEU A 110 20.767 −18.344 −45.112 1.00 0.00 H
ATOM 1689 HB2 LEU A 110 22.823 −19.070 −46.271 1.00 0.00 H
ATOM 1690 HB3 LEU A 110 22.824 −20.564 −45.340 1.00 0.00 H
ATOM 1691 HG LEU A 110 23.195 −19.266 −43.241 1.00 0.00 H
ATOM 1692 HD11 LEU A 110 25.489 −18.585 −43.721 1.00 0.00 H
ATOM 1693 HD12 LEU A 110 25.110 −20.089 −44.552 1.00 0.00 H
ATOM 1694 HD13 LEU A 110 25.151 −18.562 −45.475 1.00 0.00 H
ATOM 1695 HD21 LEU A 110 22.127 −17.140 −43.782 1.00 0.00 H
ATOM 1696 HD22 LEU A 110 23.825 −16.874 −43.394 1.00 0.00 H
ATOM 1697 HD23 LEU A 110 23.299 −16.849 −45.095 1.00 0.00 H
ATOM 1698 N ASN A 111 19.490 −21.088 −46.093 1.00 0.00 N
ATOM 1699 CA ASN A 111 18.670 −21.736 −47.126 1.00 0.00 C
ATOM 1700 C ASN A 111 17.295 −22.229 −46.622 1.00 0.00 C
ATOM 1701 O ASN A 111 16.687 −23.113 −47.224 1.00 0.00 O
ATOM 1702 CB ASN A 111 19.506 −22.812 −47.862 1.00 0.00 C
ATOM 1703 CG ASN A 111 19.685 −22.480 −49.332 1.00 0.00 C
ATOM 1704 OD1 ASN A 111 19.054 −23.053 −50.205 1.00 0.00 O
ATOM 1705 ND2 ASN A 111 20.260 −21.338 −49.630 1.00 0.00 N
ATOM 1706 H ASN A 111 19.628 −21.581 −45.215 1.00 0.00 H
ATOM 1707 HA ASN A 111 18.409 −20.965 −47.852 1.00 0.00 H
ATOM 1708 HB2 ASN A 111 20.498 −22.897 −47.416 1.00 0.00 H
ATOM 1709 HB3 ASN A 111 19.039 −23.791 −47.781 1.00 0.00 H
ATOM 1710 HD21 ASN A 111 20.570 −20.723 −48.884 1.00 0.00 H
ATOM 1711 HD22 ASN A 111 20.264 −21.069 −50.594 1.00 0.00 H
ATOM 1712 N SER A 112 16.679 −21.504 −45.681 1.00 0.00 N
ATOM 1713 CA SER A 112 15.384 −21.867 −45.068 1.00 0.00 C

ATOM 1714 C SER A 112 14.205 −22.026 −46.045 1.00 0.00 C
ATOM 1715 O SER A 112 13.201 −22.648 −45.696 1.00 0.00 O
ATOM 1716 CB SER A 112 15.010 −20.798 −44.037 1.00 0.00 C
ATOM 1717 OG SER A 112 13.794 −21.127 −43.400 1.00 0.00 O
ATOM 1718 H SER A 112 17.258 −20.848 −45.168 1.00 0.00 H
ATOM 1719 HA SER A 112 15.509 −22.813 −44.538 1.00 0.00 H
ATOM 1720 HB2 SER A 112 15.794 −20.708 −43.285 1.00 0.00 H
ATOM 1721 HB3 SER A 112 14.899 −19.837 −44.539 1.00 0.00 H
ATOM 1722 HG SER A 112 13.181 −21.505 −44.044 1.00 0.00 H
ATOM 1723 N THR A 113 14.250 −21.373 −47.212 1.00 0.00 N
ATOM 1724 CA THR A 113 13.223 −21.512 −48.268 1.00 0.00 C
ATOM 1725 C THR A 113 13.362 −22.796 −49.095 1.00 0.00 C
ATOM 1726 O THR A 113 12.437 −23.143 −49.824 1.00 0.00 O
ATOM 1727 CB THR A 113 13.214 −20.278 −49.184 1.00 0.00 C
ATOM 1728 OG1 THR A 113 12.090 −20.308 −50.027 1.00 0.00 O
ATOM 1729 CG2 THR A 113 14.452 −20.149 −50.075 1.00 0.00 C
ATOM 1730 H THR A 113 15.103 −20.884 −47.435 1.00 0.00 H
ATOM 1731 HA THR A 113 12.242 −21.563 −47.795 1.00 0.00 H
ATOM 1732 HB THR A 113 13.144 −19.386 −48.561 1.00 0.00 H
ATOM 1733 HG1 THR A 113 11.995 −21.216 −50.355 1.00 0.00 H
ATOM 1734 HG21 THR A 113 14.380 −19.230 −50.655 1.00 0.00 H
ATOM 1735 HG22 THR A 113 14.520 −20.993 −50.764 1.00 0.00 H
ATOM 1736 HG23 THR A 113 15.354 −20.111 −49.465 1.00 0.00 H
ATOM 1737 N SER A 114 14.512 −23.465 −49.008 1.00 0.00 N
ATOM 1738 CA SER A 114 14.844 −24.720 −49.695 1.00 0.00 C
ATOM 1739 C SER A 114 14.860 −25.911 −48.726 1.00 0.00 C
ATOM 1740 O SER A 114 14.755 −27.060 −49.147 1.00 0.00 O
ATOM 1741 CB SER A 114 16.241 −24.607 −50.323 1.00 0.00 C
ATOM 1742 OG SER A 114 16.469 −23.358 −50.948 1.00 0.00 O
ATOM 1743 H SER A 114 15.252 −23.065 −48.446 1.00 0.00 H

ATOM 1744 HA SER A 114 14.119 −24.921 −50.484 1.00 0.00 H
ATOM 1745 HB2 SER A 114 16.989 −24.726 −49.538 1.00 0.00 H
ATOM 1746 HB3 SER A 114 16.375 −25.406 −51.053 1.00 0.00 H
ATOM 1747 HG SER A 114 17.429 −23.190 −50.864 1.00 0.00 H
ATOM 1748 N TRP A 115 15.109 −25.654 −47.437 1.00 0.00 N
ATOM 1749 CA TRP A 115 15.312 −26.683 −46.417 1.00 0.00 C
ATOM 1750 C TRP A 115 14.010 −27.414 −46.049 1.00 0.00 C
ATOM 1751 O TRP A 115 13.085 −26.836 −45.479 1.00 0.00 O
ATOM 1752 CB TRP A 115 15.995 −26.054 −45.191 1.00 0.00 C
ATOM 1753 CG TRP A 115 16.273 −26.997 −44.059 1.00 0.00 C
ATOM 1754 CD1 TRP A 115 17.028 −28.116 −44.137 1.00 0.00 C
ATOM 1755 CD2 TRP A 115 15.765 −26.965 −42.688 1.00 0.00 C
ATOM 1756 NE1 TRP A 115 17.006 −28.780 −42.927 1.00 0.00 N
ATOM 1757 CE2 TRP A 115 16.231 −28.124 −41.999 1.00 0.00 C
ATOM 1758 CE3 TRP A 115 14.969 −26.065 −41.948 1.00 0.00 C
ATOM 1759 CZ2 TRP A 115 15.877 −28.414 −40.675 1.00 0.00 C
ATOM 1760 CZ3 TRP A 115 14.600 −26.343 −40.618 1.00 0.00 C
ATOM 1761 CH2 TRP A 115 15.039 −27.521 −39.986 1.00 0.00 C
ATOM 1762 H TRP A 115 15.291 −24.696 −47.175 1.00 0.00 H
ATOM 1763 HA TRP A 115 15.998 −27.425 −46.824 1.00 0.00 H
ATOM 1764 HB2 TRP A 115 15.360 −25.250 −44.816 1.00 0.00 H
ATOM 1765 HB3 TRP A 115 16.940 −25.607 −45.502 1.00 0.00 H
ATOM 1766 HD1 TRP A 115 17.539 −28.457 −45.027 1.00 0.00 H
ATOM 1767 HE3 TRP A 115 14.664 −25.144 −42.416 1.00 0.00 H
ATOM 1768 HZ2 TRP A 115 16.220 −29.325 −40.208 1.00 0.00 H
ATOM 1769 HZ3 TRP A 115 13.974 −25.650 −40.071 1.00 0.00 H
ATOM 1770 HH2 TRP A 115 14.726 −27.735 −38.974 1.00 0.00 H
ATOM 1771 HE1 TRP A 115 17.381 −29.718 −42.788 1.00 0.00 H
ATOM 1772 N THR A 116 13.988 −28.730 −46.260 1.00 0.00 N
ATOM 1773 CA THR A 116 13.044 −29.659 −45.621 1.00 0.00 C

ATOM 1774 C THR A 116 13.395 −29.802 −44.138 1.00 0.00 C
ATOM 1775 O THR A 116 14.513 −30.224 −43.849 1.00 0.00 O
ATOM 1776 CB THR A 116 13.144 −31.044 −46.284 1.00 0.00 C
ATOM 1777 OG1 THR A 116 14.486 −31.488 −46.274 1.00 0.00 O
ATOM 1778 CG2 THR A 116 12.672 −31.044 −47.737 1.00 0.00 C
ATOM 1779 H THR A 116 14.766 −29.147 −46.750 1.00 0.00 H
ATOM 1780 HA THR A 116 12.026 −29.282 −45.719 1.00 0.00 H
ATOM 1781 HB THR A 116 12.533 −31.750 −45.720 1.00 0.00 H
ATOM 1782 HG1 THR A 116 14.822 −31.302 −45.383 1.00 0.00 H
ATOM 1783 HG21 THR A 116 12.747 −32.054 −48.139 1.00 0.00 H
ATOM 1784 HG22 THR A 116 11.634 −30.717 −47.789 1.00 0.00 H
ATOM 1785 HG23 THR A 116 13.292 −30.378 −48.338 1.00 0.00 H
ATOM 1786 N GLU A 117 12.437 −29.637 −43.216 1.00 0.00 N
ATOM 1787 CA GLU A 117 12.686 −29.657 −41.755 1.00 0.00 C
ATOM 1788 C GLU A 117 12.916 −31.057 −41.138 1.00 0.00 C
ATOM 1789 O GLU A 117 12.334 −31.429 −40.121 1.00 0.00 O
ATOM 1790 CB GLU A 117 11.648 −28.810 −40.997 1.00 0.00 C
ATOM 1791 CG GLU A 117 10.209 −29.360 −40.956 1.00 0.00 C
ATOM 1792 CD GLU A 117 9.523 −29.123 −39.597 1.00 0.00 C
ATOM 1793 OE1 GLU A 117 8.442 −29.735 −39.428 1.00 0.00 O
ATOM 1794 OE2 GLU A 117 9.649 −27.990 −39.088 1.00 0.00 O
ATOM 1795 H GLU A 117 11.525 −29.334 −43.515 1.00 0.00 H
ATOM 1796 HA GLU A 117 13.629 −29.137 −41.609 1.00 0.00 H
ATOM 1797 HB2 GLU A 117 11.627 −27.825 −41.456 1.00 0.00 H
ATOM 1798 HB3 GLU A 117 12.018 −28.690 −39.977 1.00 0.00 H
ATOM 1799 HG2 GLU A 117 9.628 −28.887 −41.755 1.00 0.00 H
ATOM 1800 HG3 GLU A 117 10.212 −30.430 −41.164 1.00 0.00 H
ATOM 1801 N SER A 118 13.772 −31.860 −41.769 1.00 0.00 N
ATOM 1802 CA SER A 118 14.220 −33.148 −41.248 1.00 0.00 C
ATOM 1803 C SER A 118 15.060 −32.944 −39.984 1.00 0.00 C

ATOM 1804 O SER A 118 16.156 −32.380 −40.027 1.00 0.00 O
ATOM 1805 CB SER A 118 15.004 −33.892 −42.328 1.00 0.00 C
ATOM 1806 OG SER A 118 15.368 −35.179 −41.872 1.00 0.00 O
ATOM 1807 H SER A 118 14.290 −31.451 −42.537 1.00 0.00 H
ATOM 1808 HA SER A 118 13.345 −33.746 −40.994 1.00 0.00 H
ATOM 1809 HB2 SER A 118 14.379 −33.993 −43.217 1.00 0.00 H
ATOM 1810 HB3 SER A 118 15.897 −33.322 −42.589 1.00 0.00 H
ATOM 1811 HG SER A 118 15.892 −35.609 −42.552 1.00 0.00 H
ATOM 1812 N LEU A 119 14.518 −33.353 −38.839 1.00 0.00 N
ATOM 1813 CA LEU A 119 15.201 −33.325 −37.548 1.00 0.00 C
ATOM 1814 C LEU A 119 15.597 −34.743 −37.130 1.00 0.00 C
ATOM 1815 O LEU A 119 14.848 −35.703 −37.335 1.00 0.00 O
ATOM 1816 CB LEU A 119 14.324 −32.623 −36.494 1.00 0.00 C
ATOM 1817 CG LEU A 119 13.955 −31.160 −36.823 1.00 0.00 C
ATOM 1818 CD1 LEU A 119 13.219 −30.532 −35.637 1.00 0.00 C
ATOM 1819 CD2 LEU A 119 15.189 −30.304 −37.114 1.00 0.00 C
ATOM 1820 H LEU A 119 13.597 −33.764 −38.875 1.00 0.00 H
ATOM 1821 HA LEU A 119 16.129 −32.761 −37.641 1.00 0.00 H
ATOM 1822 HB2 LEU A 119 14.862 −32.639 −35.548 1.00 0.00 H
ATOM 1823 HB3 LEU A 119 13.402 −33.191 −36.364 1.00 0.00 H
ATOM 1824 HG LEU A 119 13.294 −31.134 −37.686 1.00 0.00 H
ATOM 1825 HD21 LEU A 119 14.894 −29.258 −37.186 1.00 0.00 H
ATOM 1826 HD22 LEU A 119 15.931 −30.436 −36.330 1.00 0.00 H
ATOM 1827 HD23 LEU A 119 15.611 −30.593 −38.075 1.00 0.00 H
ATOM 1828 HD11 LEU A 119 12.928 −29.512 −35.890 1.00 0.00 H
ATOM 1829 HD12 LEU A 119 12.313 −31.102 −35.435 1.00 0.00 H
ATOM 1830 HD13 LEU A 119 13.855 −30.525 −34.753 1.00 0.00 H
ATOM 1831 N SER A 120 16.735 −34.867 −36.449 1.00 0.00 N
ATOM 1832 CA SER A 120 17.180 −36.145 −35.891 1.00 0.00 C
ATOM 1833 C SER A 120 17.784 −35.980 −34.505 1.00 0.00 C

ATOM 1834 O SER A 120 18.568 −35.063 −34.266 1.00 0.00 O
ATOM 1835 CB SER A 120 18.134 −36.857 −36.847 1.00 0.00 C
ATOM 1836 OG SER A 120 19.362 −36.163 −37.000 1.00 0.00 O
ATOM 1837 H SER A 120 17.292 −34.038 −36.257 1.00 0.00 H
ATOM 1838 HA SER A 120 16.309 −36.787 −35.782 1.00 0.00 H
ATOM 1839 HB2 SER A 120 18.336 −37.857 −36.459 1.00 0.00 H
ATOM 1840 HB3 SER A 120 17.651 −36.957 −37.820 1.00 0.00 H
ATOM 1841 HG SER A 120 19.170 −35.218 −36.926 1.00 0.00 H
ATOM 1842 N LEU A 121 17.384 −36.852 −33.584 1.00 0.00 N
ATOM 1843 CA LEU A 121 17.939 −36.929 −32.241 1.00 0.00 C
ATOM 1844 C LEU A 121 18.898 −38.115 −32.152 1.00 0.00 C
ATOM 1845 O LEU A 121 18.556 −39.241 −32.514 1.00 0.00 O
ATOM 1846 CB LEU A 121 16.792 −36.955 −31.215 1.00 0.00 C
ATOM 1847 CG LEU A 121 17.273 −36.979 −29.751 1.00 0.00 C
ATOM 1848 CD1 LEU A 121 16.301 −36.197 −28.866 1.00 0.00 C
ATOM 1849 CD2 LEU A 121 17.364 −38.404 −29.198 1.00 0.00 C
ATOM 1850 H LEU A 121 16.810 −37.630 −33.894 1.00 0.00 H
ATOM 1851 HA LEU A 121 18.523 −36.029 −32.046 1.00 0.00 H
ATOM 1852 HB2 LEU A 121 16.207 −36.048 −31.371 1.00 0.00 H
ATOM 1853 HB3 LEU A 121 16.138 −37.808 −31.404 1.00 0.00 H
ATOM 1854 HG LEU A 121 18.250 −36.502 −29.678 1.00 0.00 H
ATOM 1855 HD21 LEU A 121 17.910 −39.049 −29.882 1.00 0.00 H
ATOM 1856 HD22 LEU A 121 17.906 −38.383 −28.255 1.00 0.00 H
ATOM 1857 HD23 LEU A 121 16.366 −38.815 −29.050 1.00 0.00 H
ATOM 1858 HD11 LEU A 121 16.600 −36.268 −27.822 1.00 0.00 H
ATOM 1859 HD12 LEU A 121 15.292 −36.594 −28.977 1.00 0.00 H
ATOM 1860 HD13 LEU A 121 16.302 −35.147 −29.157 1.00 0.00 H
ATOM 1861 N ILE A 122 20.070 −37.873 −31.583 1.00 0.00 N
ATOM 1862 CA ILE A 122 20.992 −38.899 −31.110 1.00 0.00 C
ATOM 1863 C ILE A 122 20.883 −38.946 −29.584 1.00 0.00 C

```
ATOM   1864  O   ILE A 122      20.764 −37.905 −28.940  1.00  0.00           O
ATOM   1865  CB  ILE A 122      22.433 −38.619 −31.602  1.00  0.00           C
ATOM   1866  CG1 ILE A 122      22.492 −38.331 −33.123  1.00  0.00           C
ATOM   1867  CG2 ILE A 122      23.350 −39.815 −31.289  1.00  0.00           C
ATOM   1868  CD1 ILE A 122      22.469 −36.837 −33.475  1.00  0.00           C
ATOM   1869  H   ILE A 122      20.258 −36.921 −31.279  1.00  0.00           H
ATOM   1870  HA  ILE A 122      20.682 −39.862 −31.509  1.00  0.00           H
ATOM   1871  HB  ILE A 122      22.827 −37.756 −31.067  1.00  0.00           H
ATOM   1872 HG12 ILE A 122      23.413 −38.741 −33.537  1.00  0.00           H
ATOM   1873 HG13 ILE A 122      21.658 −38.825 −33.618  1.00  0.00           H
ATOM   1874 HG21 ILE A 122      24.365 −39.605 −31.630  1.00  0.00           H
ATOM   1875 HG22 ILE A 122      22.987 −40.711 −31.794  1.00  0.00           H
ATOM   1876 HG23 ILE A 122      23.393 −39.998 −30.217  1.00  0.00           H
ATOM   1877 HD11 ILE A 122      22.604 −36.718 −34.550  1.00  0.00           H
ATOM   1878 HD12 ILE A 122      21.519 −36.385 −33.194  1.00  0.00           H
ATOM   1879 HD13 ILE A 122      23.276 −36.321 −32.957  1.00  0.00           H
ATOM   1880  N   ARG A 123      20.995 −40.127 −28.978  1.00  0.00           N
ATOM   1881  CA  ARG A 123      21.327 −40.252 −27.551  1.00  0.00           C
ATOM   1882  C   ARG A 123      22.633 −41.013 −27.394  1.00  0.00           C
ATOM   1883  O   ARG A 123      22.848 −42.007 −28.090  1.00  0.00           O
ATOM   1884  CB  ARG A 123      20.135 −40.800 −26.743  1.00  0.00           C
ATOM   1885  CG  ARG A 123      19.896 −42.319 −26.839  1.00  0.00           C
ATOM   1886  CD  ARG A 123      20.722 −43.108 −25.810  1.00  0.00           C
ATOM   1887  NE  ARG A 123      20.310 −44.520 −25.709  1.00  0.00           N
ATOM   1888  CZ  ARG A 123      19.320 −44.996 −24.976  1.00  0.00           C
ATOM   1889  NH1 ARG A 123      18.534 −44.232 −24.270  1.00  0.00           N
ATOM   1890  NH2 ARG A 123      19.083 −46.274 −24.973  1.00  0.00           N
ATOM   1891  H   ARG A 123      21.082 −40.948 −29.571  1.00  0.00           H
ATOM   1892  HA  ARG A 123      21.525 −39.254 −27.156  1.00  0.00           H
ATOM   1893  HB2 ARG A 123      20.263 −40.524 −25.695  1.00  0.00           H
```

ATOM 1894 HB3 ARG A 123 19.234 −40.291 −27.089 1.00 0.00 H
ATOM 1895 HG2 ARG A 123 20.127 −42.665 −27.845 1.00 0.00 H
ATOM 1896 HG3 ARG A 123 18.838 −42.502 −26.646 1.00 0.00 H
ATOM 1897 HD2 ARG A 123 20.610 −42.626 −24.841 1.00 0.00 H
ATOM 1898 HD3 ARG A 123 21.779 −43.078 −26.070 1.00 0.00 H
ATOM 1899 HE ARG A 123 20.950 −45.212 −26.079 1.00 0.00 H
ATOM 1900 HH11 ARG A 123 18.831 −43.285 −24.107 1.00 0.00 H
ATOM 1901 HH12 ARG A 123 17.925 −44.635 −23.583 1.00 0.00 H
ATOM 1902 HH21 ARG A 123 19.667 −46.871 −25.557 1.00 0.00 H
ATOM 1903 HH22 ARG A 123 18.344 −46.658 −24.419 1.00 0.00 H
ATOM 1904 N LEU A 124 23.464 −40.577 −26.456 1.00 0.00 N
ATOM 1905 CA LEU A 124 24.765 −41.167 −26.145 1.00 0.00 C
ATOM 1906 C LEU A 124 24.841 −41.405 −24.633 1.00 0.00 C
ATOM 1907 O LEU A 124 24.603 −40.486 −23.850 1.00 0.00 O
ATOM 1908 CB LEU A 124 25.889 −40.228 −26.633 1.00 0.00 C
ATOM 1909 CG LEU A 124 25.898 −39.898 −28.140 1.00 0.00 C
ATOM 1910 CD1 LEU A 124 26.962 −38.845 −28.450 1.00 0.00 C
ATOM 1911 CD2 LEU A 124 26.195 −41.125 −29.000 1.00 0.00 C
ATOM 1912 H LEU A 124 23.208 −39.736 −25.943 1.00 0.00 H
ATOM 1913 HA LEU A 124 24.871 −42.129 −26.645 1.00 0.00 H
ATOM 1914 HB2 LEU A 124 25.795 −39.289 −26.088 1.00 0.00 H
ATOM 1915 HB3 LEU A 124 26.849 −40.670 −26.369 1.00 0.00 H
ATOM 1916 HG LEU A 124 24.931 −39.489 −28.431 1.00 0.00 H
ATOM 1917 HD11 LEU A 124 26.989 −38.646 −29.522 1.00 0.00 H
ATOM 1918 HD12 LEU A 124 26.719 −37.918 −27.933 1.00 0.00 H
ATOM 1919 HD13 LEU A 124 27.944 −39.195 −28.128 1.00 0.00 H
ATOM 1920 HD21 LEU A 124 27.173 −41.531 −28.740 1.00 0.00 H
ATOM 1921 HD22 LEU A 124 26.193 −40.846 −30.052 1.00 0.00 H
ATOM 1922 HD23 LEU A 124 25.428 −41.880 −28.847 1.00 0.00 H
ATOM 1923 N ALA A 125 25.099 −42.645 −24.214 1.00 0.00 N

ATOM 1924 CA ALA A 125 25.290 −42.965 −22.801 1.00 0.00 C
ATOM 1925 C ALA A 125 26.560 −42.286 −22.264 1.00 0.00 C
ATOM 1926 O ALA A 125 27.632 −42.451 −22.848 1.00 0.00 O
ATOM 1927 CB ALA A 125 25.332 −44.485 −22.613 1.00 0.00 C
ATOM 1928 H ALA A 125 25.276 −43.368 −24.899 1.00 0.00 H
ATOM 1929 HA ALA A 125 24.434 −42.584 −22.252 1.00 0.00 H
ATOM 1930 HB1 ALA A 125 25.383 −44.716 −21.547 1.00 0.00 H
ATOM 1931 HB2 ALA A 125 24.429 −44.938 −23.020 1.00 0.00 H
ATOM 1932 HB3 ALA A 125 26.209 −44.897 −23.110 1.00 0.00 H
ATOM 1933 N VAL A 126 26.457 −41.559 −21.151 1.00 0.00 N
ATOM 1934 CA VAL A 126 27.574 −40.805 −20.544 1.00 0.00 C
ATOM 1935 C VAL A 126 28.640 −41.715 −19.926 1.00 0.00 C
ATOM 1936 O VAL A 126 29.812 −41.351 −19.890 1.00 0.00 O
ATOM 1937 CB VAL A 126 27.066 −39.795 −19.494 1.00 0.00 C
ATOM 1938 CG1 VAL A 126 26.022 −38.847 −20.098 1.00 0.00 C
ATOM 1939 CG2 VAL A 126 26.477 −40.468 −18.244 1.00 0.00 C
ATOM 1940 H VAL A 126 25.541 −41.497 −20.711 1.00 0.00 H
ATOM 1941 HA VAL A 126 28.072 −40.236 −21.326 1.00 0.00 H
ATOM 1942 HB VAL A 126 27.913 −39.186 −19.179 1.00 0.00 H
ATOM 1943 HG11 VAL A 126 25.795 −38.059 −19.381 1.00 0.00 H
ATOM 1944 HG12 VAL A 126 26.421 −38.392 −21.003 1.00 0.00 H
ATOM 1945 HG13 VAL A 126 25.104 −39.385 −20.333 1.00 0.00 H
ATOM 1946 HG21 VAL A 126 25.731 −41.211 −18.511 1.00 0.00 H
ATOM 1947 HG22 VAL A 126 26.014 −39.720 −17.601 1.00 0.00 H
ATOM 1948 HG23 VAL A 126 27.271 −40.947 −17.675 1.00 0.00 H
ATOM 1949 N ARG A 127 28.264 −42.957 −19.596 1.00 0.00 N
ATOM 1950 CA ARG A 127 29.091 −43.949 −18.894 1.00 0.00 C
ATOM 1951 C ARG A 127 28.913 −45.341 −19.537 1.00 0.00 C
ATOM 1952 O ARG A 127 28.294 −46.226 −18.950 1.00 0.00 O
ATOM 1953 CB ARG A 127 28.709 −43.859 −17.410 1.00 0.00 C

ATOM 1954 CG ARG A 127 29.694 −44.532 −16.445 1.00 0.00 C
ATOM 1955 CD ARG A 127 29.337 −44.218 −14.981 1.00 0.00 C
ATOM 1956 NE ARG A 127 28.027 −44.785 −14.591 1.00 0.00 N
ATOM 1957 CZ ARG A 127 26.842 −44.205 −14.700 1.00 0.00 C
ATOM 1958 NH1 ARG A 127 26.672 −42.965 −15.050 1.00 0.00 N
ATOM 1959 NH2 ARG A 127 25.764 −44.905 −14.536 1.00 0.00 N
ATOM 1960 H ARG A 127 27.278 −43.157 −19.683 1.00 0.00 H
ATOM 1961 HA ARG A 127 30.140 −43.665 −18.979 1.00 0.00 H
ATOM 1962 HB2 ARG A 127 27.711 −44.281 −17.284 1.00 0.00 H
ATOM 1963 HB3 ARG A 127 28.670 −42.803 −17.136 1.00 0.00 H
ATOM 1964 HG2 ARG A 127 29.689 −45.610 −16.603 1.00 0.00 H
ATOM 1965 HG3 ARG A 127 30.698 −44.151 −16.642 1.00 0.00 H
ATOM 1966 HD2 ARG A 127 30.114 −44.641 −14.341 1.00 0.00 H
ATOM 1967 HD3 ARG A 127 29.346 −43.139 −14.828 1.00 0.00 H
ATOM 1968 HE ARG A 127 28.020 −45.746 −14.308 1.00 0.00 H
ATOM 1969 HH11 ARG A 127 27.463 −42.388 −15.274 1.00 0.00 H
ATOM 1970 HH12 ARG A 127 25.712 −42.688 −15.276 1.00 0.00 H
ATOM 1971 HH21 ARG A 127 25.793 −45.900 −14.511 1.00 0.00 H
ATOM 1972 HH22 ARG A 127 24.901 −44.499 −14.924 1.00 0.00 H
ATOM 1973 N PRO A 128 29.234 −45.478 −20.837 1.00 0.00 N
ATOM 1974 CA PRO A 128 28.738 −46.535 −21.723 1.00 0.00 C
ATOM 1975 C PRO A 128 29.149 −47.935 −21.238 1.00 0.00 C
ATOM 1976 O PRO A 128 29.228 −48.879 −22.029 1.00 0.00 O
ATOM 1977 CB PRO A 128 29.303 −46.175 −23.105 1.00 0.00 C
ATOM 1978 CG PRO A 128 30.610 −45.470 −22.761 1.00 0.00 C
ATOM 1979 CD PRO A 128 30.194 −44.647 −21.547 1.00 0.00 C
ATOM 1980 HA PRO A 128 27.650 −46.498 −21.756 1.00 0.00 H
ATOM 1981 HB2 PRO A 128 29.484 −47.038 −23.740 1.00 0.00 H
ATOM 1982 HB3 PRO A 128 28.632 −45.474 −23.607 1.00 0.00 H
ATOM 1983 HG2 PRO A 128 31.365 −46.203 −22.473 1.00 0.00 H

ATOM	1984	HG3	PRO	A	128	30.969	−44.856	−23.581	1.00	0.00	H
ATOM	1985	HD2	PRO	A	128	29.721	−43.719	−21.871	1.00	0.00	H
ATOM	1986	HD3	PRO	A	128	31.060	−44.436	−20.922	1.00	0.00	H
ATOM	1987	H	PRO	A	128	29.373	−48.095	−20.184	1.00	0.00	H
ATOM	1988	N	ASP	A	129	22.436	−50.640	−28.124	1.00	0.00	N
ATOM	1989	CA	ASP	A	129	21.606	−49.436	−28.177	1.00	0.00	C
ATOM	1990	C	ASP	A	129	21.352	−48.782	−26.806	1.00	0.00	C
ATOM	1991	O	ASP	A	129	20.647	−47.771	−26.712	1.00	0.00	O
ATOM	1992	CB	ASP	A	129	20.295	−49.717	−28.923	1.00	0.00	C
ATOM	1993	CG	ASP	A	129	20.503	−49.899	−30.428	1.00	0.00	C
ATOM	1994	OD1	ASP	A	129	21.168	−50.881	−30.805	1.00	0.00	O
ATOM	1995	OD2	ASP	A	129	19.705	−49.263	−31.161	1.00	0.00	O
ATOM	1996	HA	ASP	A	129	22.156	−48.706	−28.763	1.00	0.00	H
ATOM	1997	HB2	ASP	A	129	19.829	−50.611	−28.505	1.00	0.00	H
ATOM	1998	HB3	ASP	A	129	19.606	−48.885	−28.765	1.00	0.00	H
ATOM	1999	H	ASP	A	129	22.454	−51.206	−28.976	1.00	0.00	H
ATOM	2000	H	ASP	A	129	22.090	−51.224	−27.369	1.00	0.00	H
ATOM	2001	N	TYR	A	130	21.992	−49.277	−25.741	1.00	0.00	N
ATOM	2002	CA	TYR	A	130	22.263	−48.464	−24.547	1.00	0.00	C
ATOM	2003	C	TYR	A	130	23.263	−47.350	−24.887	1.00	0.00	C
ATOM	2004	O	TYR	A	130	22.895	−46.176	−24.920	1.00	0.00	O
ATOM	2005	CB	TYR	A	130	22.771	−49.347	−23.391	1.00	0.00	C
ATOM	2006	CG	TYR	A	130	23.259	−48.569	−22.172	1.00	0.00	C
ATOM	2007	CD1	TYR	A	130	22.338	−47.886	−21.352	1.00	0.00	C
ATOM	2008	CD2	TYR	A	130	24.634	−48.510	−21.866	1.00	0.00	C
ATOM	2009	CE1	TYR	A	130	22.785	−47.123	−20.250	1.00	0.00	C
ATOM	2010	CE2	TYR	A	130	25.080	−47.762	−20.758	1.00	0.00	C
ATOM	2011	CZ	TYR	A	130	24.162	−47.061	−19.948	1.00	0.00	C
ATOM	2012	OH	TYR	A	130	24.632	−46.335	−18.897	1.00	0.00	O
ATOM	2013	H	TYR	A	130	22.543	−50.110	−25.901	1.00	0.00	H

ATOM 2014 HA TYR A 130 21.342 −47.983 −24.217 1.00 0.00 H
ATOM 2015 HB2 TYR A 130 21.965 −50.014 −23.078 1.00 0.00 H
ATOM 2016 HB3 TYR A 130 23.587 −49.975 −23.755 1.00 0.00 H
ATOM 2017 HD1 TYR A 130 21.283 −47.942 −21.570 1.00 0.00 H
ATOM 2018 HD2 TYR A 130 25.352 −49.052 −22.468 1.00 0.00 H
ATOM 2019 HE1 TYR A 130 22.081 −46.579 −19.636 1.00 0.00 H
ATOM 2020 HE2 TYR A 130 26.126 −47.727 −20.497 1.00 0.00 H
ATOM 2021 HH TYR A 130 23.968 −46.063 −18.248 1.00 0.00 H
ATOM 2022 N ASN A 131 24.436 −47.753 −25.384 1.00 0.00 N
ATOM 2023 CA ASN A 131 25.605 −46.901 −25.602 1.00 0.00 C
ATOM 2024 C ASN A 131 25.331 −45.726 −26.552 1.00 0.00 C
ATOM 2025 O ASN A 131 25.723 −44.598 −26.259 1.00 0.00 O
ATOM 2026 CB ASN A 131 26.769 −47.771 −26.131 1.00 0.00 C
ATOM 2027 CG ASN A 131 27.141 −48.939 −25.225 1.00 0.00 C
ATOM 2028 OD1 ASN A 131 26.299 −49.682 −24.752 1.00 0.00 O
ATOM 2029 ND2 ASN A 131 28.402 −49.105 −24.912 1.00 0.00 N
ATOM 2030 H ASN A 131 24.631 −48.747 −25.325 1.00 0.00 H
ATOM 2031 HA ASN A 131 25.902 −46.481 −24.641 1.00 0.00 H
ATOM 2032 HB2 ASN A 131 27.643 −47.132 −26.259 1.00 0.00 H
ATOM 2033 HB3 ASN A 131 26.512 −48.180 −27.108 1.00 0.00 H
ATOM 2034 HD21 ASN A 131 29.108 −48.482 −25.253 1.00 0.00 H
ATOM 2035 HD22 ASN A 131 28.594 −49.730 −24.141 1.00 0.00 H
ATOM 2036 N GLN A 132 24.650 −45.977 −27.672 1.00 0.00 N
ATOM 2037 CA GLN A 132 24.299 −44.979 −28.686 1.00 0.00 C
ATOM 2038 C GLN A 132 22.949 −45.342 −29.317 1.00 0.00 C
ATOM 2039 O GLN A 132 22.684 −46.522 −29.536 1.00 0.00 O
ATOM 2040 CB GLN A 132 25.379 −44.902 −29.787 1.00 0.00 C
ATOM 2041 CG GLN A 132 26.811 −44.675 −29.271 1.00 0.00 C
ATOM 2042 CD GLN A 132 27.807 −44.298 −30.364 1.00 0.00 C
ATOM 2043 OE1 GLN A 132 27.565 −44.420 −31.554 1.00 0.00 O

ATOM	2044	NE2	GLN	A	132	28.975	−43.842	−29.978	1.00	0.00	N
ATOM	2045	H	GLN	A	132	24.312	−46.916	−27.827	1.00	0.00	H
ATOM	2046	HA	GLN	A	132	24.215	−44.004	−28.212	1.00	0.00	H
ATOM	2047	HB2	GLN	A	132	25.115	−44.077	−30.452	1.00	0.00	H
ATOM	2048	HB3	GLN	A	132	25.366	−45.824	−30.371	1.00	0.00	H
ATOM	2049	HG2	GLN	A	132	27.173	−45.578	−28.781	1.00	0.00	H
ATOM	2050	HG3	GLN	A	132	26.801	−43.873	−28.536	1.00	0.00	H
ATOM	2051	HE21	GLN	A	132	29.169	−43.718	−28.989	1.00	0.00	H
ATOM	2052	HE22	GLN	A	132	29.648	−43.550	−30.675	1.00	0.00	H
ATOM	2053	N	LYS	A	133	22.153	−44.352	−29.736	1.00	0.00	N
ATOM	2054	CA	LYS	A	133	21.011	−44.567	−30.645	1.00	0.00	C
ATOM	2055	C	LYS	A	133	20.738	−43.321	−31.484	1.00	0.00	C
ATOM	2056	O	LYS	A	133	20.755	−42.215	−30.952	1.00	0.00	O
ATOM	2057	CB	LYS	A	133	19.764	−45.009	−29.854	1.00	0.00	C
ATOM	2058	CG	LYS	A	133	18.697	−45.632	−30.765	1.00	0.00	C
ATOM	2059	CD	LYS	A	133	17.467	−46.066	−29.954	1.00	0.00	C
ATOM	2060	CE	LYS	A	133	16.652	−47.133	−30.698	1.00	0.00	C
ATOM	2061	NZ	LYS	A	133	17.302	−48.464	−30.638	1.00	0.00	N
ATOM	2062	H	LYS	A	133	22.411	−43.397	−29.494	1.00	0.00	H
ATOM	2063	HA	LYS	A	133	21.283	−45.377	−31.326	1.00	0.00	H
ATOM	2064	HB2	LYS	A	133	19.338	−44.155	−29.327	1.00	0.00	H
ATOM	2065	HB3	LYS	A	133	20.060	−45.760	−29.119	1.00	0.00	H
ATOM	2066	HG2	LYS	A	133	18.376	−44.909	−31.516	1.00	0.00	H
ATOM	2067	HG3	LYS	A	133	19.136	−46.490	−31.276	1.00	0.00	H
ATOM	2068	HD2	LYS	A	133	16.839	−45.187	−29.795	1.00	0.00	H
ATOM	2069	HD3	LYS	A	133	17.773	−46.449	−28.977	1.00	0.00	H
ATOM	2070	HE2	LYS	A	133	15.658	−47.188	−30.249	1.00	0.00	H
ATOM	2071	HE3	LYS	A	133	16.541	−46.820	−31.741	1.00	0.00	H
ATOM	2072	HZ1	LYS	A	133	16.801	−49.178	−31.143	1.00	0.00	H
ATOM	2073	HZ2	LYS	A	133	17.484	−48.776	−29.693	1.00	0.00	H

ATOM	2074	HZ3	LYS	A	133	18.233	−48.448	−31.073	1.00	0.00	H
ATOM	2075	N	TYR	A	134	20.411	−43.516	−32.760	1.00	0.00	N
ATOM	2076	CA	TYR	A	134	19.962	−42.474	−33.692	1.00	0.00	C
ATOM	2077	C	TYR	A	134	18.449	−42.582	−33.945	1.00	0.00	C
ATOM	2078	O	TYR	A	134	17.930	−43.688	−34.102	1.00	0.00	O
ATOM	2079	CB	TYR	A	134	20.763	−42.612	−34.997	1.00	0.00	C
ATOM	2080	CG	TYR	A	134	20.545	−41.495	−36.000	1.00	0.00	C
ATOM	2081	CD1	TYR	A	134	19.536	−41.594	−36.980	1.00	0.00	C
ATOM	2082	CD2	TYR	A	134	21.367	−40.354	−35.955	1.00	0.00	C
ATOM	2083	CE1	TYR	A	134	19.352	−40.551	−37.911	1.00	0.00	C
ATOM	2084	CE2	TYR	A	134	21.163	−39.295	−36.859	1.00	0.00	C
ATOM	2085	CZ	TYR	A	134	20.167	−39.401	−37.851	1.00	0.00	C
ATOM	2086	OH	TYR	A	134	19.969	−38.379	−38.723	1.00	0.00	O
ATOM	2087	H	TYR	A	134	20.363	−44.465	−33.092	1.00	0.00	H
ATOM	2088	HA	TYR	A	134	20.168	−41.489	−33.270	1.00	0.00	H
ATOM	2089	HB2	TYR	A	134	20.511	−43.563	−35.470	1.00	0.00	H
ATOM	2090	HB3	TYR	A	134	21.825	−42.646	−34.753	1.00	0.00	H
ATOM	2091	HD1	TYR	A	134	18.906	−42.472	−37.022	1.00	0.00	H
ATOM	2092	HD2	TYR	A	134	22.161	−40.291	−35.224	1.00	0.00	H
ATOM	2093	HE1	TYR	A	134	18.586	−40.614	−38.668	1.00	0.00	H
ATOM	2094	HE2	TYR	A	134	21.772	−38.405	−36.805	1.00	0.00	H
ATOM	2095	HH	TYR	A	134	20.019	−37.528	−38.263	1.00	0.00	H
ATOM	2096	N	ILE	A	135	17.752	−41.449	−34.045	1.00	0.00	N
ATOM	2097	CA	ILE	A	135	16.310	−41.346	−34.315	1.00	0.00	C
ATOM	2098	C	ILE	A	135	16.084	−40.234	−35.346	1.00	0.00	C
ATOM	2099	O	ILE	A	135	16.319	−39.065	−35.051	1.00	0.00	O
ATOM	2100	CB	ILE	A	135	15.519	−41.072	−33.005	1.00	0.00	C
ATOM	2101	CG1	ILE	A	135	15.700	−42.219	−31.981	1.00	0.00	C
ATOM	2102	CG2	ILE	A	135	14.023	−40.840	−33.305	1.00	0.00	C
ATOM	2103	CD1	ILE	A	135	15.024	−41.976	−30.624	1.00	0.00	C

ATOM 2104 H ILE A 135 18.227 −40.579 −33.816 1.00 0.00 H
ATOM 2105 HA ILE A 135 15.952 −42.285 −34.738 1.00 0.00 H
ATOM 2106 HB ILE A 135 15.911 −40.157 −32.556 1.00 0.00 H
ATOM 2107 HG12 ILE A 135 15.318 −43.148 −32.406 1.00 0.00 H
ATOM 2108 HG13 ILE A 135 16.762 −42.351 −31.778 1.00 0.00 H
ATOM 2109 HD11 ILE A 135 15.334 −42.751 −29.924 1.00 0.00 H
ATOM 2110 HD12 ILE A 135 13.939 −42.017 −30.722 1.00 0.00 H
ATOM 2111 HD13 ILE A 135 15.320 −41.003 −30.230 1.00 0.00 H
ATOM 2112 HG21 ILE A 135 13.889 −39.995 −33.979 1.00 0.00 H
ATOM 2113 HG22 ILE A 135 13.483 −40.591 −32.392 1.00 0.00 H
ATOM 2114 HG23 ILE A 135 13.582 −41.730 −33.755 1.00 0.00 H
ATOM 2115 N ALA A 136 15.507 −40.564 −36.504 1.00 0.00 N
ATOM 2116 CA ALA A 136 14.855 −39.575 −37.366 1.00 0.00 C
ATOM 2117 C ALA A 136 13.444 −39.277 −36.826 1.00 0.00 C
ATOM 2118 O ALA A 136 12.671 −40.205 −36.577 1.00 0.00 O
ATOM 2119 CB ALA A 136 14.820 −40.109 −38.802 1.00 0.00 C
ATOM 2120 H ALA A 136 15.260 −41.529 −36.651 1.00 0.00 H
ATOM 2121 HA ALA A 136 15.431 −38.647 −37.364 1.00 0.00 H
ATOM 2122 HB1 ALA A 136 14.353 −39.370 −39.454 1.00 0.00 H
ATOM 2123 HB2 ALA A 136 15.836 −40.298 −39.152 1.00 0.00 H
ATOM 2124 HB3 ALA A 136 14.243 −41.034 −38.845 1.00 0.00 H
ATOM 2125 N ILE A 137 13.132 −38.008 −36.556 1.00 0.00 N
ATOM 2126 CA ILE A 137 11.854 −37.620 −35.944 1.00 0.00 C
ATOM 2127 C ILE A 137 10.745 −37.585 −37.000 1.00 0.00 C
ATOM 2128 O ILE A 137 10.852 −36.910 −38.018 1.00 0.00 O
ATOM 2129 CB ILE A 137 12.017 −36.292 −35.178 1.00 0.00 C
ATOM 2130 CG1 ILE A 137 12.875 −36.523 −33.909 1.00 0.00 C
ATOM 2131 CG2 ILE A 137 10.665 −35.691 −34.762 1.00 0.00 C
ATOM 2132 CD1 ILE A 137 13.977 −35.475 −33.773 1.00 0.00 C
ATOM 2133 H ILE A 137 13.774 −37.268 −36.828 1.00 0.00 H

ATOM 2134 HA ILE A 137 11.579 −38.379 −35.211 1.00 0.00 H
ATOM 2135 HB ILE A 137 12.512 −35.577 −35.839 1.00 0.00 H
ATOM 2136 HG12 ILE A 137 13.355 −37.502 −33.931 1.00 0.00 H
ATOM 2137 HG13 ILE A 137 12.246 −36.494 −33.016 1.00 0.00 H
ATOM 2138 HD11 ILE A 137 14.423 −35.551 −32.784 1.00 0.00 H
ATOM 2139 HD12 ILE A 137 14.739 −35.651 −34.526 1.00 0.00 H
ATOM 2140 HD13 ILE A 137 13.564 −34.483 −33.918 1.00 0.00 H
ATOM 2141 HG21 ILE A 137 10.103 −35.377 −35.642 1.00 0.00 H
ATOM 2142 HG22 ILE A 137 10.829 −34.807 −34.146 1.00 0.00 H
ATOM 2143 HG23 ILE A 137 10.085 −36.411 −34.182 1.00 0.00 H
ATOM 2144 N THR A 138 9.645 −38.290 −36.726 1.00 0.00 N
ATOM 2145 CA THR A 138 8.498 −38.440 −37.644 1.00 0.00 C
ATOM 2146 C THR A 138 7.315 −37.520 −37.324 1.00 0.00 C
ATOM 2147 O THR A 138 6.333 −37.513 −38.065 1.00 0.00 O
ATOM 2148 CB THR A 138 8.022 −39.900 −37.684 1.00 0.00 C
ATOM 2149 OG1 THR A 138 7.709 −40.357 −36.387 1.00 0.00 O
ATOM 2150 CG2 THR A 138 9.081 −40.845 −38.253 1.00 0.00 C
ATOM 2151 H THR A 138 9.632 −38.831 −35.875 1.00 0.00 H
ATOM 2152 HA THR A 138 8.816 −38.175 −38.653 1.00 0.00 H
ATOM 2153 HB THR A 138 7.132 −39.969 −38.309 1.00 0.00 H
ATOM 2154 HG21 THR A 138 8.679 −41.857 −38.302 1.00 0.00 H
ATOM 2155 HG22 THR A 138 9.346 −40.526 −39.261 1.00 0.00 H
ATOM 2156 HG23 THR A 138 9.980 −40.840 −37.635 1.00 0.00 H
ATOM 2157 HG1 THR A 138 8.436 −40.916 −36.102 1.00 0.00 H
ATOM 2158 N LYS A 139 7.394 −36.724 −36.247 1.00 0.00 N
ATOM 2159 CA LYS A 139 6.368 −35.755 −35.831 1.00 0.00 C
ATOM 2160 C LYS A 139 7.001 −34.519 −35.196 1.00 0.00 C
ATOM 2161 O LYS A 139 7.706 −34.636 −34.194 1.00 0.00 O
ATOM 2162 CB LYS A 139 5.399 −36.390 −34.824 1.00 0.00 C
ATOM 2163 CG LYS A 139 4.450 −37.429 −35.435 1.00 0.00 C

ATOM 2164 CD LYS A 139 3.465 −37.888 −34.357 1.00 0.00 C
ATOM 2165 CE LYS A 139 2.395 −38.813 −34.930 1.00 0.00 C
ATOM 2166 NZ LYS A 139 1.403 −39.149 −33.879 1.00 0.00 N
ATOM 2167 H LYS A 139 8.243 −36.764 −35.700 1.00 0.00 H
ATOM 2168 HA LYS A 139 5.802 −35.432 −36.707 1.00 0.00 H
ATOM 2169 HB2 LYS A 139 5.976 −36.851 −34.021 1.00 0.00 H
ATOM 2170 HB3 LYS A 139 4.792 −35.590 −34.392 1.00 0.00 H
ATOM 2171 HG2 LYS A 139 3.903 −36.977 −36.263 1.00 0.00 H
ATOM 2172 HG3 LYS A 139 5.015 −38.289 −35.797 1.00 0.00 H
ATOM 2173 HD2 LYS A 139 4.016 −38.421 −33.582 1.00 0.00 H
ATOM 2174 HD3 LYS A 139 2.983 −37.010 −33.920 1.00 0.00 H
ATOM 2175 HE2 LYS A 139 1.906 −38.303 −35.766 1.00 0.00 H
ATOM 2176 HE3 LYS A 139 2.879 −39.716 −35.316 1.00 0.00 H
ATOM 2177 HZ1 LYS A 139 0.676 −39.746 −34.248 1.00 0.00 H
ATOM 2178 HZ2 LYS A 139 0.983 −38.298 −33.521 1.00 0.00 H
ATOM 2179 HZ3 LYS A 139 1.858 −39.619 −33.107 1.00 0.00 H
ATOM 2180 N VAL A 140 6.590 −33.344 −35.672 1.00 0.00 N
ATOM 2181 CA VAL A 140 6.974 −32.042 −35.118 1.00 0.00 C
ATOM 2182 C VAL A 140 5.751 −31.125 −35.115 1.00 0.00 C
ATOM 2183 O VAL A 140 5.136 −30.895 −36.155 1.00 0.00 O
ATOM 2184 CB VAL A 140 8.144 −31.406 −35.897 1.00 0.00 C
ATOM 2185 CG1 VAL A 140 8.675 −30.189 −35.138 1.00 0.00 C
ATOM 2186 CG2 VAL A 140 9.329 −32.359 −36.109 1.00 0.00 C
ATOM 2187 H VAL A 140 5.960 −33.334 −36.459 1.00 0.00 H
ATOM 2188 HA VAL A 140 7.300 −32.185 −34.091 1.00 0.00 H
ATOM 2189 HB VAL A 140 7.791 −31.083 −36.876 1.00 0.00 H
ATOM 2190 HG11 VAL A 140 9.022 −30.476 −34.147 1.00 0.00 H
ATOM 2191 HG12 VAL A 140 7.900 −29.432 −35.038 1.00 0.00 H
ATOM 2192 HG13 VAL A 140 9.506 −29.769 −35.702 1.00 0.00 H
ATOM 2193 HG21 VAL A 140 9.035 −33.188 −36.753 1.00 0.00 H

ATOM 2194 HG22 VAL A 140 10.142 −31.831 −36.607 1.00 0.00 H
ATOM 2195 HG23 VAL A 140 9.677 −32.746 −35.152 1.00 0.00 H
ATOM 2196 N GLU A 141 5.341 −30.653 −33.942 1.00 0.00 N
ATOM 2197 CA GLU A 141 4.078 −29.939 −33.729 1.00 0.00 C
ATOM 2198 C GLU A 141 4.362 −28.475 −33.356 1.00 0.00 C
ATOM 2199 O GLU A 141 4.981 −28.189 −32.332 1.00 0.00 O
ATOM 2200 CB GLU A 141 3.234 −30.676 −32.673 1.00 0.00 C
ATOM 2201 CG GLU A 141 2.808 −32.075 −33.166 1.00 0.00 C
ATOM 2202 CD GLU A 141 2.043 −32.922 −32.135 1.00 0.00 C
ATOM 2203 OE1 GLU A 141 2.082 −32.580 −30.929 1.00 0.00 O
ATOM 2204 OE2 GLU A 141 1.618 −34.032 −32.535 1.00 0.00 O
ATOM 2205 H GLU A 141 5.866 −30.921 −33.112 1.00 0.00 H
ATOM 2206 HA GLU A 141 3.498 −29.941 −34.652 1.00 0.00 H
ATOM 2207 HB2 GLU A 141 3.821 −30.764 −31.762 1.00 0.00 H
ATOM 2208 HB3 GLU A 141 2.340 −30.087 −32.460 1.00 0.00 H
ATOM 2209 HG2 GLU A 141 2.183 −31.945 −34.053 1.00 0.00 H
ATOM 2210 HG3 GLU A 141 3.694 −32.638 −33.465 1.00 0.00 H
ATOM 2211 N ARG A 142 4.044 −27.543 −34.268 1.00 0.00 N
ATOM 2212 CA ARG A 142 4.571 −26.161 −34.235 1.00 0.00 C
ATOM 2213 C ARG A 142 3.576 −25.054 −34.610 1.00 0.00 C
ATOM 2214 O ARG A 142 3.972 −23.962 −35.010 1.00 0.00 O
ATOM 2215 CB ARG A 142 5.925 −26.076 −34.971 1.00 0.00 C
ATOM 2216 CG ARG A 142 5.940 −26.320 −36.490 1.00 0.00 C
ATOM 2217 CD ARG A 142 5.754 −27.792 −36.899 1.00 0.00 C
ATOM 2218 NE ARG A 142 6.327 −28.106 −38.221 1.00 0.00 N
ATOM 2219 CZ ARG A 142 6.017 −27.592 −39.395 1.00 0.00 C
ATOM 2220 NH1 ARG A 142 5.168 −26.627 −39.572 1.00 0.00 N
ATOM 2221 NH2 ARG A 142 6.628 −27.982 −40.457 1.00 0.00 N
ATOM 2222 H ARG A 142 3.613 −27.868 −35.120 1.00 0.00 H
ATOM 2223 HA ARG A 142 4.797 −25.935 −33.190 1.00 0.00 H

ATOM 2224 HB2 ARG A 142 6.324 −25.079 −34.802 1.00 0.00 H
ATOM 2225 HB3 ARG A 142 6.623 −26.772 −34.506 1.00 0.00 H
ATOM 2226 HG2 ARG A 142 6.910 −25.996 −36.868 1.00 0.00 H
ATOM 2227 HG3 ARG A 142 5.173 −25.709 −36.964 1.00 0.00 H
ATOM 2228 HD2 ARG A 142 6.254 −28.422 −36.168 1.00 0.00 H
ATOM 2229 HD3 ARG A 142 4.697 −28.053 −36.888 1.00 0.00 H
ATOM 2230 HE ARG A 142 7.083 −28.795 −38.268 1.00 0.00 H
ATOM 2231 HH11 ARG A 142 4.839 −26.086 −38.799 1.00 0.00 H
ATOM 2232 HH12 ARG A 142 5.221 −26.165 −40.462 1.00 0.00 H
ATOM 2233 HH21 ARG A 142 7.363 −28.665 −40.273 1.00 0.00 H
ATOM 2234 HH22 ARG A 142 6.716 −27.339 −41.232 1.00 0.00 H
ATOM 2235 N GLY A 143 2.279 −25.299 −34.425 1.00 0.00 N
ATOM 2236 CA GLY A 143 1.241 −24.266 −34.534 1.00 0.00 C
ATOM 2237 C GLY A 143 1.241 −23.542 −35.888 1.00 0.00 C
ATOM 2238 O GLY A 143 1.014 −24.167 −36.921 1.00 0.00 O
ATOM 2239 H GLY A 143 2.026 −26.199 −34.050 1.00 0.00 H
ATOM 2240 HA2 GLY A 143 0.262 −24.725 −34.403 1.00 0.00 H
ATOM 2241 HA3 GLY A 143 1.380 −23.537 −33.736 1.00 0.00 H
ATOM 2242 N LYS A 144 1.447 −22.216 −35.879 1.00 0.00 N
ATOM 2243 CA LYS A 144 1.487 −21.372 −37.091 1.00 0.00 C
ATOM 2244 C LYS A 144 2.871 −21.250 −37.750 1.00 0.00 C
ATOM 2245 O LYS A 144 2.976 −20.586 −38.779 1.00 0.00 O
ATOM 2246 CB LYS A 144 0.877 −19.984 −36.784 1.00 0.00 C
ATOM 2247 CG LYS A 144 −0.319 −19.627 −37.686 1.00 0.00 C
ATOM 2248 CD LYS A 144 0.033 −19.432 −39.171 1.00 0.00 C
ATOM 2249 CE LYS A 144 −1.245 −19.149 −39.970 1.00 0.00 C
ATOM 2250 NZ LYS A 144 −0.968 −18.970 −41.415 1.00 0.00 N
ATOM 2251 H LYS A 144 1.653 −21.781 −34.990 1.00 0.00 H
ATOM 2252 HA LYS A 144 0.868 −21.864 −37.838 1.00 0.00 H
ATOM 2253 HB2 LYS A 144 0.523 −19.957 −35.750 1.00 0.00 H

ATOM 2254 HB3 LYS A 144 1.640 −19.206 −36.875 1.00 0.00 H
ATOM 2255 HG2 LYS A 144 −1.067 −20.415 −37.597 1.00 0.00 H
ATOM 2256 HG3 LYS A 144 −0.761 −18.701 −37.314 1.00 0.00 H
ATOM 2257 HD2 LYS A 144 0.732 −18.598 −39.273 1.00 0.00 H
ATOM 2258 HD3 LYS A 144 0.501 −20.337 −39.561 1.00 0.00 H
ATOM 2259 HE2 LYS A 144 −1.932 −19.987 −39.826 1.00 0.00 H
ATOM 2260 HE3 LYS A 144 −1.719 −18.246 −39.573 1.00 0.00 H
ATOM 2261 HZ1 LYS A 144 −0.380 −19.695 −41.794 1.00 0.00 H
ATOM 2262 HZ2 LYS A 144 −0.503 −18.074 −41.592 1.00 0.00 H
ATOM 2263 HZ3 LYS A 144 −1.813 −18.886 −41.959 1.00 0.00 H
ATOM 2264 N TYR A 145 3.925 −21.834 −37.176 1.00 0.00 N
ATOM 2265 CA TYR A 145 5.253 −21.852 −37.800 1.00 0.00 C
ATOM 2266 C TYR A 145 5.272 −22.804 −38.999 1.00 0.00 C
ATOM 2267 O TYR A 145 4.770 −23.925 −38.927 1.00 0.00 O
ATOM 2268 CB TYR A 145 6.342 −22.182 −36.769 1.00 0.00 C
ATOM 2269 CG TYR A 145 6.509 −21.056 −35.773 1.00 0.00 C
ATOM 2270 CD1 TYR A 145 5.715 −21.007 −34.613 1.00 0.00 C
ATOM 2271 CD2 TYR A 145 7.365 −19.982 −36.079 1.00 0.00 C
ATOM 2272 CE1 TYR A 145 5.729 −19.858 −33.800 1.00 0.00 C
ATOM 2273 CE2 TYR A 145 7.389 −18.837 −35.265 1.00 0.00 C
ATOM 2274 CZ TYR A 145 6.543 −18.758 −34.145 1.00 0.00 C
ATOM 2275 OH TYR A 145 6.492 −17.603 −33.440 1.00 0.00 O
ATOM 2276 H TYR A 145 3.782 −22.425 −36.368 1.00 0.00 H
ATOM 2277 HA TYR A 145 5.461 −20.851 −38.182 1.00 0.00 H
ATOM 2278 HB2 TYR A 145 6.094 −23.104 −36.249 1.00 0.00 H
ATOM 2279 HB3 TYR A 145 7.288 −22.332 −37.288 1.00 0.00 H
ATOM 2280 HD1 TYR A 145 5.060 −21.831 −34.367 1.00 0.00 H
ATOM 2281 HD2 TYR A 145 7.955 −20.007 −36.979 1.00 0.00 H
ATOM 2282 HE1 TYR A 145 5.096 −19.805 −32.928 1.00 0.00 H
ATOM 2283 HE2 TYR A 145 8.007 −17.991 −35.517 1.00 0.00 H

ATOM 2284 HH TYR A 145 5.833 −17.687 −32.700 1.00 0.00 H
ATOM 2285 N ASP A 146 5.869 −22.350 −40.102 1.00 0.00 N
ATOM 2286 CA ASP A 146 6.030 −23.118 −41.346 1.00 0.00 C
ATOM 2287 C ASP A 146 6.996 −24.298 −41.150 1.00 0.00 C
ATOM 2288 O ASP A 146 6.691 −25.415 −41.581 1.00 0.00 O
ATOM 2289 CB ASP A 146 6.495 −22.127 −42.428 1.00 0.00 C
ATOM 2290 CG ASP A 146 6.955 −22.724 −43.769 1.00 0.00 C
ATOM 2291 OD1 ASP A 146 7.858 −23.583 −43.718 1.00 0.00 O
ATOM 2292 OD2 ASP A 146 6.922 −21.945 −44.747 1.00 0.00 O
ATOM 2293 H ASP A 146 6.360 −21.466 −40.035 1.00 0.00 H
ATOM 2294 HA ASP A 146 5.067 −23.530 −41.647 1.00 0.00 H
ATOM 2295 HB2 ASP A 146 5.683 −21.425 −42.616 1.00 0.00 H
ATOM 2296 HB3 ASP A 146 7.331 −21.560 −42.018 1.00 0.00 H
ATOM 2297 N LYS A 147 8.019 −24.088 −40.310 1.00 0.00 N
ATOM 2298 CA LYS A 147 9.024 −25.069 −39.881 1.00 0.00 C
ATOM 2299 C LYS A 147 9.731 −24.635 −38.586 1.00 0.00 C
ATOM 2300 O LYS A 147 9.635 −23.466 −38.202 1.00 0.00 O
ATOM 2301 CB LYS A 147 10.021 −25.317 −41.030 1.00 0.00 C
ATOM 2302 CG LYS A 147 10.678 −24.031 −41.555 1.00 0.00 C
ATOM 2303 CD LYS A 147 11.762 −24.261 −42.610 1.00 0.00 C
ATOM 2304 CE LYS A 147 11.314 −24.966 −43.894 1.00 0.00 C
ATOM 2305 NZ LYS A 147 10.284 −24.214 −44.643 1.00 0.00 N
ATOM 2306 H LYS A 147 8.125 −23.148 −39.944 1.00 0.00 H
ATOM 2307 HA LYS A 147 8.518 −26.008 −39.661 1.00 0.00 H
ATOM 2308 HB2 LYS A 147 9.499 −25.817 −41.847 1.00 0.00 H
ATOM 2309 HB3 LYS A 147 10.803 −25.979 −40.663 1.00 0.00 H
ATOM 2310 HG2 LYS A 147 9.925 −23.356 −41.962 1.00 0.00 H
ATOM 2311 HG3 LYS A 147 11.161 −23.550 −40.713 1.00 0.00 H
ATOM 2312 HD2 LYS A 147 12.206 −23.300 −42.868 1.00 0.00 H
ATOM 2313 HD3 LYS A 147 12.532 −24.871 −42.149 1.00 0.00 H

ATOM	2314	HE2	LYS	A	147	12.195	−25.084	−44.529	1.00	0.00	H
ATOM	2315	HE3	LYS	A	147	10.945	−25.964	−43.643	1.00	0.00	H
ATOM	2316	HZ1	LYS	A	147	10.019	−24.687	−45.491	1.00	0.00	H
ATOM	2317	HZ2	LYS	A	147	10.584	−23.277	−44.886	1.00	0.00	H
ATOM	2318	HZ3	LYS	A	147	9.424	−24.097	−44.098	1.00	0.00	H
ATOM	2319	N	VAL	A	148	10.462	−25.542	−37.933	1.00	0.00	N
ATOM	2320	CA	VAL	A	148	11.216	−25.325	−36.673	1.00	0.00	C
ATOM	2321	C	VAL	A	148	12.532	−26.126	−36.630	1.00	0.00	C
ATOM	2322	O	VAL	A	148	12.715	−27.076	−37.388	1.00	0.00	O
ATOM	2323	CB	VAL	A	148	10.369	−25.668	−35.428	1.00	0.00	C
ATOM	2324	CG1	VAL	A	148	9.206	−24.701	−35.199	1.00	0.00	C
ATOM	2325	CG2	VAL	A	148	9.812	−27.091	−35.480	1.00	0.00	C
ATOM	2326	H	VAL	A	148	10.440	−26.496	−38.303	1.00	0.00	H
ATOM	2327	HA	VAL	A	148	11.491	−24.275	−36.608	1.00	0.00	H
ATOM	2328	HB	VAL	A	148	11.008	−25.600	−34.548	1.00	0.00	H
ATOM	2329	HG11	VAL	A	148	9.588	−23.726	−34.915	1.00	0.00	H
ATOM	2330	HG12	VAL	A	148	8.584	−24.612	−36.085	1.00	0.00	H
ATOM	2331	HG13	VAL	A	148	8.597	−25.057	−34.376	1.00	0.00	H
ATOM	2332	HG21	VAL	A	148	9.257	−27.310	−34.569	1.00	0.00	H
ATOM	2333	HG22	VAL	A	148	10.636	−27.800	−35.566	1.00	0.00	H
ATOM	2334	HG23	VAL	A	148	9.150	−27.217	−36.338	1.00	0.00	H
ATOM	2335	N	MET	A	149	13.474	−25.744	−35.757	1.00	0.00	N
ATOM	2336	CA	MET	A	149	14.831	−26.317	−35.710	1.00	0.00	C
ATOM	2337	C	MET	A	149	15.505	−26.168	−34.325	1.00	0.00	C
ATOM	2338	O	MET	A	149	15.299	−25.136	−33.683	1.00	0.00	O
ATOM	2339	CB	MET	A	149	15.657	−25.606	−36.796	1.00	0.00	C
ATOM	2340	CG	MET	A	149	16.999	−26.261	−37.104	1.00	0.00	C
ATOM	2341	SD	MET	A	149	18.024	−25.355	−38.283	1.00	0.00	S
ATOM	2342	CE	MET	A	149	18.574	−23.966	−37.257	1.00	0.00	C
ATOM	2343	H	MET	A	149	13.302	−24.907	−35.208	1.00	0.00	H

ATOM 2344 HA MET A 149 14.758 −27.374 −35.956 1.00 0.00 H
ATOM 2345 HB2 MET A 149 15.092 −25.600 −37.727 1.00 0.00 H
ATOM 2346 HB3 MET A 149 15.822 −24.574 −36.492 1.00 0.00 H
ATOM 2347 HG2 MET A 149 16.790 −27.244 −37.525 1.00 0.00 H
ATOM 2348 HG3 MET A 149 17.573 −26.388 −36.191 1.00 0.00 H
ATOM 2349 HE1 MET A 149 19.254 −23.339 −37.832 1.00 0.00 H
ATOM 2350 HE2 MET A 149 19.097 −24.346 −36.381 1.00 0.00 H
ATOM 2351 HE3 MET A 149 17.719 −23.370 −36.939 1.00 0.00 H
ATOM 2352 N PRO A 150 16.368 −27.107 −33.872 1.00 0.00 N
ATOM 2353 CA PRO A 150 17.294 −26.876 −32.751 1.00 0.00 C
ATOM 2354 C PRO A 150 18.264 −25.722 −33.045 1.00 0.00 C
ATOM 2355 O PRO A 150 18.670 −25.519 −34.189 1.00 0.00 O
ATOM 2356 CB PRO A 150 18.069 −28.187 −32.574 1.00 0.00 C
ATOM 2357 CG PRO A 150 18.061 −28.772 −33.985 1.00 0.00 C
ATOM 2358 CD PRO A 150 16.663 −28.402 −34.473 1.00 0.00 C
ATOM 2359 HA PRO A 150 16.737 −26.654 −31.840 1.00 0.00 H
ATOM 2360 HB2 PRO A 150 19.085 −28.025 −32.216 1.00 0.00 H
ATOM 2361 HB3 PRO A 150 17.527 −28.841 −31.893 1.00 0.00 H
ATOM 2362 HG2 PRO A 150 18.223 −29.849 −33.990 1.00 0.00 H
ATOM 2363 HG3 PRO A 150 18.813 −28.274 −34.600 1.00 0.00 H
ATOM 2364 HD2 PRO A 150 16.654 −28.371 −35.559 1.00 0.00 H
ATOM 2365 HD3 PRO A 150 15.941 −29.136 −34.113 1.00 0.00 H
ATOM 2366 N TYR A 151 18.590 −24.917 −32.031 1.00 0.00 N
ATOM 2367 CA TYR A 151 19.341 −23.674 −32.233 1.00 0.00 C
ATOM 2368 C TYR A 151 20.016 −23.186 −30.941 1.00 0.00 C
ATOM 2369 O TYR A 151 19.713 −22.114 −30.419 1.00 0.00 O
ATOM 2370 CB TYR A 151 18.403 −22.623 −32.868 1.00 0.00 C
ATOM 2371 CG TYR A 151 19.048 −21.560 −33.743 1.00 0.00 C
ATOM 2372 CD1 TYR A 151 18.568 −20.241 −33.676 1.00 0.00 C
ATOM 2373 CD2 TYR A 151 19.979 −21.910 −34.745 1.00 0.00 C

ATOM 2374 CE1 TYR A 151 18.960 −19.297 −34.640 1.00 0.00 C
ATOM 2375 CE2 TYR A 151 20.447 −20.939 −35.653 1.00 0.00 C
ATOM 2376 CZ TYR A 151 19.924 −19.630 −35.610 1.00 0.00 C
ATOM 2377 OH TYR A 151 20.328 −18.683 −36.497 1.00 0.00 O
ATOM 2378 H TYR A 151 18.203 −25.107 −31.111 1.00 0.00 H
ATOM 2379 HA TYR A 151 20.143 −23.896 −32.934 1.00 0.00 H
ATOM 2380 HB2 TYR A 151 17.689 −23.134 −33.513 1.00 0.00 H
ATOM 2381 HB3 TYR A 151 17.809 −22.150 −32.085 1.00 0.00 H
ATOM 2382 HD1 TYR A 151 17.850 −19.967 −32.915 1.00 0.00 H
ATOM 2383 HD2 TYR A 151 20.312 −22.934 −34.848 1.00 0.00 H
ATOM 2384 HE1 TYR A 151 18.510 −18.323 −34.653 1.00 0.00 H
ATOM 2385 HE2 TYR A 151 21.162 −21.202 −36.419 1.00 0.00 H
ATOM 2386 HH TYR A 151 19.667 −17.995 −36.601 1.00 0.00 H
ATOM 2387 N GLY A 152 20.890 −24.009 −30.359 1.00 0.00 N
ATOM 2388 CA GLY A 152 21.552 −23.673 −29.097 1.00 0.00 C
ATOM 2389 C GLY A 152 22.239 −24.860 −28.426 1.00 0.00 C
ATOM 2390 O GLY A 152 21.687 −25.952 −28.480 1.00 0.00 O
ATOM 2391 H GLY A 152 21.040 −24.929 −30.754 1.00 0.00 H
ATOM 2392 HA2 GLY A 152 22.258 −22.859 −29.262 1.00 0.00 H
ATOM 2393 HA3 GLY A 152 20.781 −23.339 −28.416 1.00 0.00 H
ATOM 2394 N PRO A 153 23.405 −24.702 −27.776 1.00 0.00 N
ATOM 2395 CA PRO A 153 24.228 −25.843 −27.369 1.00 0.00 C
ATOM 2396 C PRO A 153 23.640 −26.672 −26.212 1.00 0.00 C
ATOM 2397 O PRO A 153 23.677 −27.905 −26.268 1.00 0.00 O
ATOM 2398 CB PRO A 153 25.579 −25.225 −27.000 1.00 0.00 C
ATOM 2399 CG PRO A 153 25.225 −23.812 −26.537 1.00 0.00 C
ATOM 2400 CD PRO A 153 24.061 −23.444 −27.457 1.00 0.00 C
ATOM 2401 HA PRO A 153 24.365 −26.512 −28.219 1.00 0.00 H
ATOM 2402 HB2 PRO A 153 26.205 −25.169 −27.891 1.00 0.00 H
ATOM 2403 HB3 PRO A 153 26.083 −25.789 −26.220 1.00 0.00 H

ATOM 2404 HG2 PRO A 153 24.885 −23.833 −25.499 1.00 0.00 H
ATOM 2405 HG3 PRO A 153 26.064 −23.127 −26.650 1.00 0.00 H
ATOM 2406 HD2 PRO A 153 24.442 −22.986 −28.370 1.00 0.00 H
ATOM 2407 HD3 PRO A 153 23.379 −22.762 −26.948 1.00 0.00 H
ATOM 2408 N SER A 154 23.019 −26.020 −25.223 1.00 0.00 N
ATOM 2409 CA SER A 154 22.569 −26.648 −23.975 1.00 0.00 C
ATOM 2410 C SER A 154 21.151 −26.224 −23.568 1.00 0.00 C
ATOM 2411 O SER A 154 20.751 −25.060 −23.687 1.00 0.00 O
ATOM 2412 CB SER A 154 23.597 −26.414 −22.855 1.00 0.00 C
ATOM 2413 OG SER A 154 23.932 −25.055 −22.654 1.00 0.00 O
ATOM 2414 H SER A 154 23.007 −25.012 −25.238 1.00 0.00 H
ATOM 2415 HA SER A 154 22.538 −27.727 −24.127 1.00 0.00 H
ATOM 2416 HB2 SER A 154 23.223 −26.842 −21.924 1.00 0.00 H
ATOM 2417 HB3 SER A 154 24.514 −26.929 −23.128 1.00 0.00 H
ATOM 2418 HG SER A 154 24.684 −25.048 −22.044 1.00 0.00 H
ATOM 2419 N GLY A 155 20.356 −27.239 −23.228 1.00 0.00 N
ATOM 2420 CA GLY A 155 18.976 −27.155 −22.759 1.00 0.00 C
ATOM 2421 C GLY A 155 18.775 −27.849 −21.409 1.00 0.00 C
ATOM 2422 O GLY A 155 19.743 −28.164 −20.717 1.00 0.00 O
ATOM 2423 H GLY A 155 20.767 −28.166 −23.215 1.00 0.00 H
ATOM 2424 HA2 GLY A 155 18.323 −27.627 −23.491 1.00 0.00 H
ATOM 2425 HA3 GLY A 155 18.692 −26.115 −22.651 1.00 0.00 H
ATOM 2426 N ILE A 156 17.518 −28.117 −21.035 1.00 0.00 N
ATOM 2427 CA ILE A 156 17.182 −28.738 −19.741 1.00 0.00 C
ATOM 2428 C ILE A 156 16.191 −29.894 −19.860 1.00 0.00 C
ATOM 2429 O ILE A 156 15.198 −29.823 −20.575 1.00 0.00 O
ATOM 2430 CB ILE A 156 16.704 −27.705 −18.693 1.00 0.00 C
ATOM 2431 CG1 ILE A 156 15.406 −26.972 −19.102 1.00 0.00 C
ATOM 2432 CG2 ILE A 156 17.847 −26.733 −18.368 1.00 0.00 C
ATOM 2433 CD1 ILE A 156 14.821 −26.095 −17.991 1.00 0.00 C

ATOM 2434 H ILE A 156 16.773 −27.945 −21.701 1.00 0.00 H

ATOM 2435 HA ILE A 156 18.095 −29.181 −19.343 1.00 0.00 H

ATOM 2436 HB ILE A 156 16.488 −28.256 −17.778 1.00 0.00 H

ATOM 2437 HG12 ILE A 156 14.640 −27.700 −19.360 1.00 0.00 H

ATOM 2438 HG13 ILE A 156 15.593 −26.353 −19.980 1.00 0.00 H

ATOM 2439 HD11 ILE A 156 14.602 −26.705 −17.115 1.00 0.00 H

ATOM 2440 HD12 ILE A 156 15.524 −25.312 −17.721 1.00 0.00 H

ATOM 2441 HD13 ILE A 156 13.904 −25.628 −18.349 1.00 0.00 H

ATOM 2442 HG21 ILE A 156 18.759 −27.288 −18.143 1.00 0.00 H

ATOM 2443 HG22 ILE A 156 17.595 −26.106 −17.516 1.00 0.00 H

ATOM 2444 HG23 ILE A 156 18.029 −26.098 −19.230 1.00 0.00 H

ATOM 2445 N LYS A 157 16.353 −30.863 −18.959 1.00 0.00 N

ATOM 2446 CA LYS A 157 15.305 −31.793 −18.525 1.00 0.00 C

ATOM 2447 C LYS A 157 14.466 −31.186 −17.398 1.00 0.00 C

ATOM 2448 O LYS A 157 15.002 −30.494 −16.528 1.00 0.00 O

ATOM 2449 CB LYS A 157 15.896 −33.169 −18.159 1.00 0.00 C

ATOM 2450 CG LYS A 157 16.745 −33.254 −16.876 1.00 0.00 C

ATOM 2451 CD LYS A 157 18.001 −32.367 −16.897 1.00 0.00 C

ATOM 2452 CE LYS A 157 19.039 −32.862 −15.886 1.00 0.00 C

ATOM 2453 NZ LYS A 157 20.409 −32.719 −16.420 1.00 0.00 N

ATOM 2454 H LYS A 157 17.180 −30.805 −18.389 1.00 0.00 H

ATOM 2455 HA LYS A 157 14.626 −31.954 −19.365 1.00 0.00 H

ATOM 2456 HB2 LYS A 157 15.068 −33.871 −18.050 1.00 0.00 H

ATOM 2457 HB3 LYS A 157 16.497 −33.526 −18.997 1.00 0.00 H

ATOM 2458 HG2 LYS A 157 17.052 −34.294 −16.767 1.00 0.00 H

ATOM 2459 HG3 LYS A 157 16.136 −32.996 −16.010 1.00 0.00 H

ATOM 2460 HD2 LYS A 157 18.429 −32.395 −17.900 1.00 0.00 H

ATOM 2461 HD3 LYS A 157 17.737 −31.337 −16.660 1.00 0.00 H

ATOM 2462 HE2 LYS A 157 18.929 −32.313 −14.948 1.00 0.00 H

ATOM 2463 HE3 LYS A 157 18.856 −33.922 −15.690 1.00 0.00 H

ATOM 2464 HZ1 LYS A 157 21.110 −32.990 −15.734 1.00 0.00 H
ATOM 2465 HZ2 LYS A 157 20.655 −31.761 −16.687 1.00 0.00 H
ATOM 2466 HZ3 LYS A 157 20.513 −33.278 −17.262 1.00 0.00 H
ATOM 2467 N GLN A 158 13.197 −31.573 −17.347 1.00 0.00 N
ATOM 2468 CA GLN A 158 12.217 −31.262 −16.303 1.00 0.00 C
ATOM 2469 C GLN A 158 11.470 −32.550 −15.928 1.00 0.00 C
ATOM 2470 O GLN A 158 10.349 −32.794 −16.373 1.00 0.00 O
ATOM 2471 CB GLN A 158 11.275 −30.136 −16.765 1.00 0.00 C
ATOM 2472 CG GLN A 158 12.010 −28.797 −16.962 1.00 0.00 C
ATOM 2473 CD GLN A 158 11.070 −27.595 −17.027 1.00 0.00 C
ATOM 2474 OE1 GLN A 158 9.859 −27.693 −17.077 1.00 0.00 O
ATOM 2475 NE2 GLN A 158 11.590 −26.390 −16.974 1.00 0.00 N
ATOM 2476 H GLN A 158 12.863 −32.133 −18.128 1.00 0.00 H
ATOM 2477 HA GLN A 158 12.734 −30.924 −15.405 1.00 0.00 H
ATOM 2478 HB2 GLN A 158 10.513 −30.006 −15.996 1.00 0.00 H
ATOM 2479 HB3 GLN A 158 10.781 −30.421 −17.696 1.00 0.00 H
ATOM 2480 HG2 GLN A 158 12.691 −28.641 −16.125 1.00 0.00 H
ATOM 2481 HG3 GLN A 158 12.600 −28.835 −17.878 1.00 0.00 H
ATOM 2482 HE21 GLN A 158 12.582 −26.256 −16.929 1.00 0.00 H
ATOM 2483 HE22 GLN A 158 10.924 −25.639 −17.010 1.00 0.00 H
ATOM 2484 N GLY A 159 12.221 −33.490 −15.345 1.00 0.00 N
ATOM 2485 CA GLY A 159 11.855 −34.907 −15.367 1.00 0.00 C
ATOM 2486 C GLY A 159 11.900 −35.471 −16.789 1.00 0.00 C
ATOM 2487 O GLY A 159 12.805 −35.151 −17.557 1.00 0.00 O
ATOM 2488 H GLY A 159 13.179 −33.263 −15.148 1.00 0.00 H
ATOM 2489 HA2 GLY A 159 12.547 −35.481 −14.755 1.00 0.00 H
ATOM 2490 HA3 GLY A 159 10.849 −35.029 −14.962 1.00 0.00 H
ATOM 2491 N ASP A 160 10.856 −36.212 −17.148 1.00 0.00 N
ATOM 2492 CA ASP A 160 10.718 −36.950 −18.411 1.00 0.00 C
ATOM 2493 C ASP A 160 10.544 −36.057 −19.669 1.00 0.00 C

ATOM	2494	O	ASP	A	160	10.648	−36.533	−20.801	1.00	0.00	O
ATOM	2495	CB	ASP	A	160	9.501	−37.876	−18.246	1.00	0.00	C
ATOM	2496	CG	ASP	A	160	9.617	−39.195	−19.005	1.00	0.00	C
ATOM	2497	OD1	ASP	A	160	10.722	−39.773	−19.040	1.00	0.00	O
ATOM	2498	OD2	ASP	A	160	8.542	−39.697	−19.403	1.00	0.00	O
ATOM	2499	H	ASP	A	160	10.151	−36.391	−16.452	1.00	0.00	H
ATOM	2500	HA	ASP	A	160	11.619	−37.554	−18.538	1.00	0.00	H
ATOM	2501	HB2	ASP	A	160	9.380	−38.139	−17.193	1.00	0.00	H
ATOM	2502	HB3	ASP	A	160	8.599	−37.340	−18.550	1.00	0.00	H
ATOM	2503	N	THR	A	161	10.279	−34.752	−19.499	1.00	0.00	N
ATOM	2504	CA	THR	A	161	10.316	−33.761	−20.587	1.00	0.00	C
ATOM	2505	C	THR	A	161	11.706	−33.151	−20.735	1.00	0.00	C
ATOM	2506	O	THR	A	161	12.170	−32.458	−19.831	1.00	0.00	O
ATOM	2507	CB	THR	A	161	9.261	−32.660	−20.394	1.00	0.00	C
ATOM	2508	OG1	THR	A	161	7.967	−33.191	−20.555	1.00	0.00	O
ATOM	2509	CG2	THR	A	161	9.338	−31.581	−21.477	1.00	0.00	C
ATOM	2510	H	THR	A	161	10.264	−34.401	−18.550	1.00	0.00	H
ATOM	2511	HA	THR	A	161	10.092	−34.261	−21.522	1.00	0.00	H
ATOM	2512	HB	THR	A	161	9.364	−32.202	−19.404	1.00	0.00	H
ATOM	2513	HG1	THR	A	161	7.968	−34.123	−20.262	1.00	0.00	H
ATOM	2514	HG21	THR	A	161	8.545	−30.852	−21.325	1.00	0.00	H
ATOM	2515	HG22	THR	A	161	10.278	−31.046	−21.398	1.00	0.00	H
ATOM	2516	HG23	THR	A	161	9.261	−32.014	−22.477	1.00	0.00	H
ATOM	2517	N	LEU	A	162	12.286	−33.230	−21.938	1.00	0.00	N
ATOM	2518	CA	LEU	A	162	13.448	−32.434	−22.352	1.00	0.00	C
ATOM	2519	C	LEU	A	162	12.988	−31.128	−23.029	1.00	0.00	C
ATOM	2520	O	LEU	A	162	11.986	−31.130	−23.745	1.00	0.00	O
ATOM	2521	CB	LEU	A	162	14.363	−33.255	−23.287	1.00	0.00	C
ATOM	2522	CG	LEU	A	162	14.598	−34.727	−22.894	1.00	0.00	C
ATOM	2523	CD1	LEU	A	162	15.581	−35.352	−23.883	1.00	0.00	C

ATOM 2524 CD2 LEU A 162 15.179 −34.897 −21.490 1.00 0.00 C
ATOM 2525 H LEU A 162 11.832 −33.794 −22.648 1.00 0.00 H
ATOM 2526 HA LEU A 162 14.035 −32.169 −21.474 1.00 0.00 H
ATOM 2527 HB2 LEU A 162 13.923 −33.245 −24.282 1.00 0.00 H
ATOM 2528 HB3 LEU A 162 15.327 −32.746 −23.358 1.00 0.00 H
ATOM 2529 HG LEU A 162 13.660 −35.276 −22.958 1.00 0.00 H
ATOM 2530 HD11 LEU A 162 15.705 −36.409 −23.648 1.00 0.00 H
ATOM 2531 HD12 LEU A 162 16.545 −34.850 −23.808 1.00 0.00 H
ATOM 2532 HD13 LEU A 162 15.199 −35.254 −24.898 1.00 0.00 H
ATOM 2533 HD21 LEU A 162 15.324 −35.958 −21.287 1.00 0.00 H
ATOM 2534 HD22 LEU A 162 14.485 −34.508 −20.744 1.00 0.00 H
ATOM 2535 HD23 LEU A 162 16.132 −34.380 −21.410 1.00 0.00 H
ATOM 2536 N TYR A 163 13.800 −30.073 −22.944 1.00 0.00 N
ATOM 2537 CA TYR A 163 13.611 −28.778 −23.602 1.00 0.00 C
ATOM 2538 C TYR A 163 14.925 −28.317 −24.241 1.00 0.00 C
ATOM 2539 O TYR A 163 15.799 −27.756 −23.573 1.00 0.00 O
ATOM 2540 CB TYR A 163 13.137 −27.738 −22.581 1.00 0.00 C
ATOM 2541 CG TYR A 163 11.701 −27.871 −22.128 1.00 0.00 C
ATOM 2542 CD1 TYR A 163 10.684 −27.299 −22.913 1.00 0.00 C
ATOM 2543 CD2 TYR A 163 11.393 −28.466 −20.890 1.00 0.00 C
ATOM 2544 CE1 TYR A 163 9.363 −27.265 −22.439 1.00 0.00 C
ATOM 2545 CE2 TYR A 163 10.066 −28.449 −20.419 1.00 0.00 C
ATOM 2546 CZ TYR A 163 9.057 −27.832 −21.188 1.00 0.00 C
ATOM 2547 OH TYR A 163 7.783 −27.790 −20.733 1.00 0.00 O
ATOM 2548 H TYR A 163 14.566 −30.127 −22.282 1.00 0.00 H
ATOM 2549 HA TYR A 163 12.860 −28.859 −24.390 1.00 0.00 H
ATOM 2550 HB2 TYR A 163 13.794 −27.766 −21.709 1.00 0.00 H
ATOM 2551 HB3 TYR A 163 13.248 −26.747 −23.023 1.00 0.00 H
ATOM 2552 HD1 TYR A 163 10.921 −26.844 −23.861 1.00 0.00 H
ATOM 2553 HD2 TYR A 163 12.176 −28.920 −20.297 1.00 0.00 H

ATOM	2554	HE1	TYR	A	163	8.586	−26.780	−23.015	1.00	0.00	H
ATOM	2555	HE2	TYR	A	163	9.808	−28.883	−19.465	1.00	0.00	H
ATOM	2556	HH	TYR	A	163	7.313	−27.014	−21.131	1.00	0.00	H
ATOM	2557	N	PHE	A	164	15.043	−28.501	−25.555	1.00	0.00	N
ATOM	2558	CA	PHE	A	164	16.165	−28.004	−26.353	1.00	0.00	C
ATOM	2559	C	PHE	A	164	15.931	−26.532	−26.753	1.00	0.00	C
ATOM	2560	O	PHE	A	164	14.851	−26.237	−27.271	1.00	0.00	O
ATOM	2561	CB	PHE	A	164	16.309	−28.888	−27.602	1.00	0.00	C
ATOM	2562	CG	PHE	A	164	16.519	−30.365	−27.309	1.00	0.00	C
ATOM	2563	CD1	PHE	A	164	17.792	−30.828	−26.931	1.00	0.00	C
ATOM	2564	CD2	PHE	A	164	15.443	−31.272	−27.393	1.00	0.00	C
ATOM	2565	CE1	PHE	A	164	17.994	−32.191	−26.645	1.00	0.00	C
ATOM	2566	CE2	PHE	A	164	15.644	−32.635	−27.105	1.00	0.00	C
ATOM	2567	CZ	PHE	A	164	16.919	−33.093	−26.730	1.00	0.00	C
ATOM	2568	H	PHE	A	164	14.272	−28.939	−26.050	1.00	0.00	H
ATOM	2569	HA	PHE	A	164	17.080	−28.090	−25.768	1.00	0.00	H
ATOM	2570	HB2	PHE	A	164	15.414	−28.777	−28.215	1.00	0.00	H
ATOM	2571	HB3	PHE	A	164	17.149	−28.529	−28.195	1.00	0.00	H
ATOM	2572	HD1	PHE	A	164	18.615	−30.133	−26.855	1.00	0.00	H
ATOM	2573	HD2	PHE	A	164	14.458	−30.922	−27.667	1.00	0.00	H
ATOM	2574	HE1	PHE	A	164	18.973	−32.541	−26.355	1.00	0.00	H
ATOM	2575	HE2	PHE	A	164	14.815	−33.327	−27.162	1.00	0.00	H
ATOM	2576	HZ	PHE	A	164	17.071	−34.136	−26.499	1.00	0.00	H
ATOM	2577	N	PRO	A	165	16.905	−25.607	−26.631	1.00	0.00	N
ATOM	2578	CA	PRO	A	165	16.816	−24.278	−27.248	1.00	0.00	C
ATOM	2579	C	PRO	A	165	16.688	−24.370	−28.777	1.00	0.00	C
ATOM	2580	O	PRO	A	165	17.375	−25.156	−29.434	1.00	0.00	O
ATOM	2581	CB	PRO	A	165	18.091	−23.538	−26.832	1.00	0.00	C
ATOM	2582	CG	PRO	A	165	19.075	−24.682	−26.614	1.00	0.00	C
ATOM	2583	CD	PRO	A	165	18.198	−25.757	−25.982	1.00	0.00	C

ATOM 2584 HA PRO A 165 15.949 −23.748 −26.855 1.00 0.00 H

ATOM 2585 HB2 PRO A 165 17.924 −23.011 −25.891 1.00 0.00 H

ATOM 2586 HB3 PRO A 165 18.442 −22.853 −27.604 1.00 0.00 H

ATOM 2587 HG2 PRO A 165 19.432 −25.033 −27.580 1.00 0.00 H

ATOM 2588 HG3 PRO A 165 19.908 −24.392 −25.979 1.00 0.00 H

ATOM 2589 HD2 PRO A 165 18.085 −25.562 −24.916 1.00 0.00 H

ATOM 2590 HD3 PRO A 165 18.635 −26.741 −26.138 1.00 0.00 H

ATOM 2591 N ALA A 166 15.809 −23.551 −29.352 1.00 0.00 N

ATOM 2592 CA ALA A 166 15.298 −23.750 −30.706 1.00 0.00 C

ATOM 2593 C ALA A 166 14.928 −22.440 −31.420 1.00 0.00 C

ATOM 2594 O ALA A 166 14.850 −21.373 −30.810 1.00 0.00 O

ATOM 2595 CB ALA A 166 14.084 −24.678 −30.595 1.00 0.00 C

ATOM 2596 H ALA A 166 15.276 −22.924 −28.757 1.00 0.00 H

ATOM 2597 HA ALA A 166 16.053 −24.246 −31.316 1.00 0.00 H

ATOM 2598 HB1 ALA A 166 13.307 −24.201 −29.999 1.00 0.00 H

ATOM 2599 HB2 ALA A 166 13.688 −24.895 −31.586 1.00 0.00 H

ATOM 2600 HB3 ALA A 166 14.379 −25.615 −30.120 1.00 0.00 H

ATOM 2601 N VAL A 167 14.563 −22.559 −32.699 1.00 0.00 N

ATOM 2602 CA VAL A 167 14.020 −21.481 −33.530 1.00 0.00 C

ATOM 2603 C VAL A 167 12.843 −21.965 −34.377 1.00 0.00 C

ATOM 2604 O VAL A 167 12.872 −23.062 −34.934 1.00 0.00 O

ATOM 2605 CB VAL A 167 15.137 −20.835 −34.373 1.00 0.00 C

ATOM 2606 CG1 VAL A 167 15.714 −21.752 −35.457 1.00 0.00 C

ATOM 2607 CG2 VAL A 167 14.710 −19.527 −35.044 1.00 0.00 C

ATOM 2608 H VAL A 167 14.650 −23.477 −33.128 1.00 0.00 H

ATOM 2609 HA VAL A 167 13.643 −20.715 −32.858 1.00 0.00 H

ATOM 2610 HB VAL A 167 15.935 −20.585 −33.682 1.00 0.00 H

ATOM 2611 HG21 VAL A 167 14.352 −18.832 −34.289 1.00 0.00 H

ATOM 2612 HG22 VAL A 167 13.928 −19.710 −35.781 1.00 0.00 H

ATOM 2613 HG23 VAL A 167 15.573 −19.087 −35.544 1.00 0.00 H

```
ATOM   2614 HG11 VAL A 167      16.595 −21.289 −35.901  1.00  0.00           H
ATOM   2615 HG12 VAL A 167      16.007 −22.706 −35.021  1.00  0.00           H
ATOM   2616 HG13 VAL A 167      14.975 −21.931 −36.235  1.00  0.00           H
ATOM   2617  N   GLY A 168      11.819 −21.123 −34.499  1.00  0.00           N
ATOM   2618  CA  GLY A 168      10.718 −21.270 −35.445  1.00  0.00           C
ATOM   2619  C   GLY A 168      10.811 −20.262 −36.584  1.00  0.00           C
ATOM   2620  O   GLY A 168      11.202 −19.110 −36.383  1.00  0.00           O
ATOM   2621  H   GLY A 168      11.868 −20.253 −33.981  1.00  0.00           H
ATOM   2622  HA2 GLY A 168      10.741 −22.264 −35.883  1.00  0.00           H
ATOM   2623  HA3 GLY A 168       9.767 −21.137 −34.930  1.00  0.00           H
ATOM   2624  N   PHE A 169      10.417 −20.698 −37.779  1.00  0.00           N
ATOM   2625  CA  PHE A 169      10.414 −19.909 −39.007  1.00  0.00           C
ATOM   2626  C   PHE A 169       8.964 −19.639 −39.429  1.00  0.00           C
ATOM   2627  O   PHE A 169       8.154 −20.566 −39.541  1.00  0.00           O
ATOM   2628  CB  PHE A 169      11.165 −20.655 −40.117  1.00  0.00           C
ATOM   2629  CG  PHE A 169      12.571 −21.156 −39.803  1.00  0.00           C
ATOM   2630  CD1 PHE A 169      13.682 −20.589 −40.452  1.00  0.00           C
ATOM   2631  CD2 PHE A 169      12.778 −22.253 −38.943  1.00  0.00           C
ATOM   2632  CE1 PHE A 169      14.973 −21.115 −40.257  1.00  0.00           C
ATOM   2633  CE2 PHE A 169      14.065 −22.768 −38.733  1.00  0.00           C
ATOM   2634  CZ  PHE A 169      15.166 −22.208 −39.397  1.00  0.00           C
ATOM   2635  H   PHE A 169      10.095 −21.659 −37.855  1.00  0.00           H
ATOM   2636  HA  PHE A 169      10.919 −18.958 −38.836  1.00  0.00           H
ATOM   2637  HB2 PHE A 169      10.556 −21.505 −40.411  1.00  0.00           H
ATOM   2638  HB3 PHE A 169      11.219 −20.000 −40.985  1.00  0.00           H
ATOM   2639  HD1 PHE A 169      13.542 −19.771 −41.143  1.00  0.00           H
ATOM   2640  HD2 PHE A 169      11.947 −22.725 −38.451  1.00  0.00           H
ATOM   2641  HE1 PHE A 169      15.812 −20.696 −40.794  1.00  0.00           H
ATOM   2642  HE2 PHE A 169      14.200 −23.606 −38.070  1.00  0.00           H
ATOM   2643  HZ  PHE A 169      16.153 −22.628 −39.257  1.00  0.00           H
```

ATOM	2644	N	LEU	A	170	8.610	−18.370	−39.631	1.00	0.00	N
ATOM	2645	CA	LEU	A	170	7.235	−17.944	−39.911	1.00	0.00	C
ATOM	2646	C	LEU	A	170	7.226	−16.895	−41.036	1.00	0.00	C
ATOM	2647	O	LEU	A	170	7.999	−15.938	−40.953	1.00	0.00	O
ATOM	2648	CB	LEU	A	170	6.611	−17.465	−38.587	1.00	0.00	C
ATOM	2649	CG	LEU	A	170	5.220	−16.811	−38.689	1.00	0.00	C
ATOM	2650	CD1	LEU	A	170	4.343	−17.228	−37.507	1.00	0.00	C
ATOM	2651	CD2	LEU	A	170	5.334	−15.287	−38.658	1.00	0.00	C
ATOM	2652	H	LEU	A	170	9.313	−17.648	−39.494	1.00	0.00	H
ATOM	2653	HA	LEU	A	170	6.657	−18.807	−40.236	1.00	0.00	H
ATOM	2654	HB2	LEU	A	170	6.520	−18.348	−37.960	1.00	0.00	H
ATOM	2655	HB3	LEU	A	170	7.298	−16.784	−38.084	1.00	0.00	H
ATOM	2656	HG	LEU	A	170	4.725	−17.125	−39.609	1.00	0.00	H
ATOM	2657	HD21	LEU	A	170	5.849	−14.965	−37.754	1.00	0.00	H
ATOM	2658	HD22	LEU	A	170	5.882	−14.941	−39.532	1.00	0.00	H
ATOM	2659	HD23	LEU	A	170	4.342	−14.844	−38.668	1.00	0.00	H
ATOM	2660	HD11	LEU	A	170	3.366	−16.751	−37.582	1.00	0.00	H
ATOM	2661	HD12	LEU	A	170	4.815	−16.940	−36.567	1.00	0.00	H
ATOM	2662	HD13	LEU	A	170	4.204	−18.310	−37.517	1.00	0.00	H
ATOM	2663	N	PRO	A	171	6.494	−17.104	−42.149	1.00	0.00	N
ATOM	2664	CA	PRO	A	171	6.512	−16.161	−43.262	1.00	0.00	C
ATOM	2665	C	PRO	A	171	6.012	−14.780	−42.819	1.00	0.00	C
ATOM	2666	O	PRO	A	171	5.013	−14.667	−42.111	1.00	0.00	O
ATOM	2667	CB	PRO	A	171	5.670	−16.776	−44.383	1.00	0.00	C
ATOM	2668	CG	PRO	A	171	4.951	−17.976	−43.765	1.00	0.00	C
ATOM	2669	CD	PRO	A	171	5.685	−18.272	−42.458	1.00	0.00	C
ATOM	2670	HA	PRO	A	171	7.535	−16.066	−43.621	1.00	0.00	H
ATOM	2671	HB2	PRO	A	171	4.954	−16.063	−44.792	1.00	0.00	H
ATOM	2672	HB3	PRO	A	171	6.330	−17.119	−45.175	1.00	0.00	H
ATOM	2673	HG2	PRO	A	171	3.916	−17.719	−43.554	1.00	0.00	H

ATOM 2674 HG3 PRO A 171 4.983 −18.839 −44.432 1.00 0.00 H
ATOM 2675 HD2 PRO A 171 6.336 −19.136 −42.594 1.00 0.00 H
ATOM 2676 HD3 PRO A 171 4.961 −18.470 −41.667 1.00 0.00 H
ATOM 2677 N ARG A 172 6.701 −13.705 −43.221 1.00 0.00 N
ATOM 2678 CA ARG A 172 6.431 −12.338 −42.734 1.00 0.00 C
ATOM 2679 C ARG A 172 5.151 −11.691 −43.289 1.00 0.00 C
ATOM 2680 O ARG A 172 4.867 −10.549 −42.949 1.00 0.00 O
ATOM 2681 CB ARG A 172 7.687 −11.449 −42.829 1.00 0.00 C
ATOM 2682 CG ARG A 172 8.079 −10.972 −44.233 1.00 0.00 C
ATOM 2683 CD ARG A 172 9.132 −9.846 −44.201 1.00 0.00 C
ATOM 2684 NE ARG A 172 10.407 −10.198 −43.527 1.00 0.00 N
ATOM 2685 CZ ARG A 172 10.953 −9.577 −42.489 1.00 0.00 C
ATOM 2686 NH1 ARG A 172 10.243 −8.821 −41.699 1.00 0.00 N
ATOM 2687 NH2 ARG A 172 12.215 −9.714 −42.204 1.00 0.00 N
ATOM 2688 H ARG A 172 7.532 −13.866 −43.784 1.00 0.00 H
ATOM 2689 HA ARG A 172 6.223 −12.440 −41.669 1.00 0.00 H
ATOM 2690 HB2 ARG A 172 7.526 −10.575 −42.195 1.00 0.00 H
ATOM 2691 HB3 ARG A 172 8.530 −12.009 −42.430 1.00 0.00 H
ATOM 2692 HG2 ARG A 172 8.455 −11.814 −44.808 1.00 0.00 H
ATOM 2693 HG3 ARG A 172 7.197 −10.585 −44.742 1.00 0.00 H
ATOM 2694 HD2 ARG A 172 8.684 −8.965 −43.741 1.00 0.00 H
ATOM 2695 HD3 ARG A 172 9.363 −9.581 −45.234 1.00 0.00 H
ATOM 2696 HE ARG A 172 11.009 −10.840 −44.031 1.00 0.00 H
ATOM 2697 HH11 ARG A 172 9.255 −8.798 −41.837 1.00 0.00 H
ATOM 2698 HH12 ARG A 172 10.639 −8.449 −40.841 1.00 0.00 H
ATOM 2699 HH21 ARG A 172 12.810 −10.224 −42.850 1.00 0.00 H
ATOM 2700 HH22 ARG A 172 12.662 −9.093 −41.539 1.00 0.00 H
ATOM 2701 N THR A 173 4.352 −12.439 −44.050 1.00 0.00 N
ATOM 2702 CA THR A 173 2.921 −12.186 −44.302 1.00 0.00 C
ATOM 2703 C THR A 173 2.055 −12.459 −43.067 1.00 0.00 C

```
ATOM   2704  O   THR A 173       1.123 −11.712 −42.792  1.00  0.00           O
ATOM   2705  CB  THR A 173       2.430 −13.098 −45.441  1.00  0.00           C
ATOM   2706  OG1 THR A 173       2.840 −14.431 −45.221  1.00  0.00           O
ATOM   2707  CG2 THR A 173       3.014 −12.677 −46.789  1.00  0.00           C
ATOM   2708  H   THR A 173       4.659 −13.378 −44.258  1.00  0.00           H
ATOM   2709  HA  THR A 173       2.769 −11.146 −44.586  1.00  0.00           H
ATOM   2710  HB  THR A 173       1.341 −13.057 −45.503  1.00  0.00           H
ATOM   2711 HG21 THR A 173       2.618 −13.329 −47.568  1.00  0.00           H
ATOM   2712 HG22 THR A 173       2.727 −11.649 −47.009  1.00  0.00           H
ATOM   2713 HG23 THR A 173       4.100 −12.759 −46.774  1.00  0.00           H
ATOM   2714  HG1 THR A 173       2.075 −14.915 −44.813  1.00  0.00           H
ATOM   2715  N   GLU A 174       2.429 −13.447 −42.252  1.00  0.00           N
ATOM   2716  CA  GLU A 174       1.701 −13.846 −41.041  1.00  0.00           C
ATOM   2717  C   GLU A 174       2.125 −13.032 −39.809  1.00  0.00           C
ATOM   2718  O   GLU A 174       1.435 −13.051 −38.789  1.00  0.00           O
ATOM   2719  CB  GLU A 174       1.941 −15.341 −40.750  1.00  0.00           C
ATOM   2720  CG  GLU A 174       1.946 −16.266 −41.972  1.00  0.00           C
ATOM   2721  CD  GLU A 174       0.715 −16.072 −42.854  1.00  0.00           C
ATOM   2722  OE1 GLU A 174      −0.327 −16.638 −42.451  1.00  0.00           O
ATOM   2723  OE2 GLU A 174       0.905 −15.677 −44.024  1.00  0.00           O
ATOM   2724  H   GLU A 174       3.217 −14.023 −42.529  1.00  0.00           H
ATOM   2725  HA  GLU A 174       0.632 −13.687 −41.199  1.00  0.00           H
ATOM   2726  HB2 GLU A 174       1.175 −15.685 −40.053  1.00  0.00           H
ATOM   2727  HB3 GLU A 174       2.906 −15.458 −40.263  1.00  0.00           H
ATOM   2728  HG2 GLU A 174       2.846 −16.071 −42.554  1.00  0.00           H
ATOM   2729  HG3 GLU A 174       1.994 −17.303 −41.628  1.00  0.00           H
ATOM   2730  N   PHE A 175       3.310 −12.413 −39.855  1.00  0.00           N
ATOM   2731  CA  PHE A 175       3.981 −11.826 −38.696  1.00  0.00           C
ATOM   2732  C   PHE A 175       3.301 −10.538 −38.209  1.00  0.00           C
ATOM   2733  O   PHE A 175       3.645  −9.430 −38.621  1.00  0.00           O
```

ATOM 2734 CB PHE A 175 5.469 −11.610 −39.010 1.00 0.00 C
ATOM 2735 CG PHE A 175 6.268 −11.037 −37.851 1.00 0.00 C
ATOM 2736 CD1 PHE A 175 6.228 −11.653 −36.583 1.00 0.00 C
ATOM 2737 CD2 PHE A 175 7.054 −9.884 −38.037 1.00 0.00 C
ATOM 2738 CE1 PHE A 175 6.949 −11.107 −35.507 1.00 0.00 C
ATOM 2739 CE2 PHE A 175 7.789 −9.350 −36.965 1.00 0.00 C
ATOM 2740 CZ PHE A 175 7.728 −9.954 −35.697 1.00 0.00 C
ATOM 2741 H PHE A 175 3.806 −12.439 −40.732 1.00 0.00 H
ATOM 2742 HA PHE A 175 3.919 −12.549 −37.882 1.00 0.00 H
ATOM 2743 HB2 PHE A 175 5.917 −12.562 −39.292 1.00 0.00 H
ATOM 2744 HB3 PHE A 175 5.549 −10.940 −39.867 1.00 0.00 H
ATOM 2745 HD1 PHE A 175 5.634 −12.541 −36.423 1.00 0.00 H
ATOM 2746 HD2 PHE A 175 7.080 −9.401 −39.002 1.00 0.00 H
ATOM 2747 HE1 PHE A 175 6.891 −11.566 −34.530 1.00 0.00 H
ATOM 2748 HE2 PHE A 175 8.382 −8.462 −37.108 1.00 0.00 H
ATOM 2749 HZ PHE A 175 8.261 −9.521 −34.863 1.00 0.00 H
ATOM 2750 N GLN A 176 2.383 −10.694 −37.256 1.00 0.00 N
ATOM 2751 CA GLN A 176 1.738 −9.579 −36.567 1.00 0.00 C
ATOM 2752 C GLN A 176 2.737 −8.891 −35.625 1.00 0.00 C
ATOM 2753 O GLN A 176 3.091 −9.432 −34.579 1.00 0.00 O
ATOM 2754 CB GLN A 176 0.488 −10.068 −35.806 1.00 0.00 C
ATOM 2755 CG GLN A 176 −0.487 −10.906 −36.653 1.00 0.00 C
ATOM 2756 CD GLN A 176 −0.809 −10.270 −38.002 1.00 0.00 C
ATOM 2757 OE1 GLN A 176 −1.278 −9.150 −38.092 1.00 0.00 O
ATOM 2758 NE2 GLN A 176 −0.492 −10.926 −39.095 1.00 0.00 N
ATOM 2759 H GLN A 176 2.120 −11.633 −36.997 1.00 0.00 H
ATOM 2760 HA GLN A 176 1.414 −8.850 −37.313 1.00 0.00 H
ATOM 2761 HB2 GLN A 176 0.797 −10.674 −34.952 1.00 0.00 H
ATOM 2762 HB3 GLN A 176 −0.042 −9.195 −35.424 1.00 0.00 H
ATOM 2763 HG2 GLN A 176 −0.061 −11.897 −36.809 1.00 0.00 H

ATOM 2764 HG3 GLN A 176 −1.416 −11.035 −36.098 1.00 0.00 H
ATOM 2765 HE21 GLN A 176 0.033 −11.789 −39.025 1.00 0.00 H
ATOM 2766 HE22 GLN A 176 −0.625 −10.447 −39.967 1.00 0.00 H
ATOM 2767 N TYR A 177 3.134 −7.664 −35.963 1.00 0.00 N
ATOM 2768 CA TYR A 177 3.960 −6.806 −35.116 1.00 0.00 C
ATOM 2769 C TYR A 177 3.527 −5.347 −35.267 1.00 0.00 C
ATOM 2770 O TYR A 177 3.466 −4.825 −36.379 1.00 0.00 O
ATOM 2771 CB TYR A 177 5.446 −6.992 −35.456 1.00 0.00 C
ATOM 2772 CG TYR A 177 6.376 −6.200 −34.552 1.00 0.00 C
ATOM 2773 CD1 TYR A 177 6.861 −6.783 −33.363 1.00 0.00 C
ATOM 2774 CD2 TYR A 177 6.705 −4.865 −34.863 1.00 0.00 C
ATOM 2775 CE1 TYR A 177 7.661 −6.029 −32.482 1.00 0.00 C
ATOM 2776 CE2 TYR A 177 7.506 −4.112 −33.984 1.00 0.00 C
ATOM 2777 CZ TYR A 177 7.970 −4.687 −32.784 1.00 0.00 C
ATOM 2778 OH TYR A 177 8.698 −3.943 −31.913 1.00 0.00 O
ATOM 2779 H TYR A 177 2.846 −7.297 −36.858 1.00 0.00 H
ATOM 2780 HA TYR A 177 3.819 −7.097 −34.074 1.00 0.00 H
ATOM 2781 HB2 TYR A 177 5.691 −8.051 −35.367 1.00 0.00 H
ATOM 2782 HB3 TYR A 177 5.621 −6.698 −36.493 1.00 0.00 H
ATOM 2783 HD1 TYR A 177 6.594 −7.803 −33.115 1.00 0.00 H
ATOM 2784 HD2 TYR A 177 6.310 −4.399 −35.760 1.00 0.00 H
ATOM 2785 HE1 TYR A 177 8.015 −6.457 −31.555 1.00 0.00 H
ATOM 2786 HE2 TYR A 177 7.732 −3.084 −34.212 1.00 0.00 H
ATOM 2787 HH TYR A 177 8.548 −3.005 −32.037 1.00 0.00 H
ATOM 2788 N ASN A 178 3.233 −4.682 −34.149 1.00 0.00 N
ATOM 2789 CA ASN A 178 2.982 −3.245 −34.128 1.00 0.00 C
ATOM 2790 C ASN A 178 4.315 −2.486 −34.128 1.00 0.00 C
ATOM 2791 O ASN A 178 4.971 −2.394 −33.091 1.00 0.00 O
ATOM 2792 CB ASN A 178 2.116 −2.907 −32.903 1.00 0.00 C
ATOM 2793 CG ASN A 178 1.811 −1.422 −32.771 1.00 0.00 C

ATOM	2794	OD1	ASN	A	178	1.848	−0.646	−33.716	1.00	0.00	O
ATOM	2795	ND2	ASN	A	178	1.605	−0.944	−31.571	1.00	0.00	N
ATOM	2796	H	ASN	A	178	3.358	−5.150	−33.266	1.00	0.00	H
ATOM	2797	HA	ASN	A	178	2.428	−2.958	−35.024	1.00	0.00	H
ATOM	2798	HB2	ASN	A	178	1.168	−3.440	−32.970	1.00	0.00	H
ATOM	2799	HB3	ASN	A	178	2.627	−3.238	−32.000	1.00	0.00	H
ATOM	2800	HD21	ASN	A	178	1.918	−1.486	−30.769	1.00	0.00	H
ATOM	2801	HD22	ASN	A	178	1.418	0.036	−31.483	1.00	0.00	H
ATOM	2802	N	ASP	A	179	4.616	−1.790	−35.224	1.00	0.00	N
ATOM	2803	CA	ASP	A	179	5.781	−0.901	−35.332	1.00	0.00	C
ATOM	2804	C	ASP	A	179	5.823	0.182	−34.234	1.00	0.00	C
ATOM	2805	O	ASP	A	179	6.901	0.489	−33.732	1.00	0.00	O
ATOM	2806	CB	ASP	A	179	5.811	−0.257	−36.723	1.00	0.00	C
ATOM	2807	CG	ASP	A	179	6.158	−1.207	−37.861	1.00	0.00	C
ATOM	2808	OD1	ASP	A	179	6.438	−2.407	−37.647	1.00	0.00	O
ATOM	2809	OD2	ASP	A	179	6.246	−0.712	−39.003	1.00	0.00	O
ATOM	2810	H	ASP	A	179	4.087	−1.975	−36.064	1.00	0.00	H
ATOM	2811	HA	ASP	A	179	6.691	−1.491	−35.234	1.00	0.00	H
ATOM	2812	HB2	ASP	A	179	4.842	0.196	−36.931	1.00	0.00	H
ATOM	2813	HB3	ASP	A	179	6.553	0.537	−36.728	1.00	0.00	H
ATOM	2814	N	SER	A	180	4.669	0.594	−33.692	1.00	0.00	N
ATOM	2815	CA	SER	A	180	4.578	1.555	−32.575	1.00	0.00	C
ATOM	2816	C	SER	A	180	5.143	1.023	−31.245	1.00	0.00	C
ATOM	2817	O	SER	A	180	5.414	1.814	−30.346	1.00	0.00	O
ATOM	2818	CB	SER	A	180	3.135	2.033	−32.337	1.00	0.00	C
ATOM	2819	OG	SER	A	180	2.353	2.057	−33.519	1.00	0.00	O
ATOM	2820	H	SER	A	180	3.809	0.241	−34.087	1.00	0.00	H
ATOM	2821	HA	SER	A	180	5.170	2.433	−32.838	1.00	0.00	H
ATOM	2822	HB2	SER	A	180	2.651	1.389	−31.608	1.00	0.00	H
ATOM	2823	HB3	SER	A	180	3.166	3.037	−31.912	1.00	0.00	H

ATOM 2824 HG SER A 180 2.073 1.147 −33.727 1.00 0.00 H
ATOM 2825 N ASN A 181 5.360 −0.294 −31.110 1.00 0.00 N
ATOM 2826 CA ASN A 181 6.080 −0.889 −29.977 1.00 0.00 C
ATOM 2827 C ASN A 181 7.609 −0.696 −30.074 1.00 0.00 C
ATOM 2828 O ASN A 181 8.325 −1.043 −29.136 1.00 0.00 O
ATOM 2829 CB ASN A 181 5.755 −2.398 −29.866 1.00 0.00 C
ATOM 2830 CG ASN A 181 4.333 −2.748 −29.453 1.00 0.00 C
ATOM 2831 OD1 ASN A 181 3.362 −2.050 −29.695 1.00 0.00 O
ATOM 2832 ND2 ASN A 181 4.150 −3.883 −28.819 1.00 0.00 N
ATOM 2833 H ASN A 181 5.129 −0.897 −31.892 1.00 0.00 H
ATOM 2834 HA ASN A 181 5.759 −0.398 −29.057 1.00 0.00 H
ATOM 2835 HB2 ASN A 181 5.977 −2.900 −30.806 1.00 0.00 H
ATOM 2836 HB3 ASN A 181 6.419 −2.825 −29.114 1.00 0.00 H
ATOM 2837 HD21 ASN A 181 4.931 −4.471 −28.585 1.00 0.00 H
ATOM 2838 HD22 ASN A 181 3.217 −4.090 −28.514 1.00 0.00 H
ATOM 2839 N CYS A 182 8.142 −0.330 −31.246 1.00 0.00 N
ATOM 2840 CA CYS A 182 9.580 −0.286 −31.497 1.00 0.00 C
ATOM 2841 C CYS A 182 10.253 0.877 −30.720 1.00 0.00 C
ATOM 2842 O CYS A 182 9.856 2.022 −30.940 1.00 0.00 O
ATOM 2843 CB CYS A 182 9.796 −0.203 −33.014 1.00 0.00 C
ATOM 2844 SG CYS A 182 11.476 −0.555 −33.576 1.00 0.00 S
ATOM 2845 H CYS A 182 7.524 −0.020 −31.990 1.00 0.00 H
ATOM 2846 HA CYS A 182 9.986 −1.241 −31.181 1.00 0.00 H
ATOM 2847 HB2 CYS A 182 9.129 −0.910 −33.509 1.00 0.00 H
ATOM 2848 HB3 CYS A 182 9.515 0.795 −33.352 1.00 0.00 H
ATOM 2849 N PRO A 183 11.278 0.652 −29.860 1.00 0.00 N
ATOM 2850 CA PRO A 183 11.807 1.655 −28.904 1.00 0.00 C
ATOM 2851 C PRO A 183 12.537 2.911 −29.444 1.00 0.00 C
ATOM 2852 O PRO A 183 13.381 3.477 −28.752 1.00 0.00 O
ATOM 2853 CB PRO A 183 12.718 0.874 −27.945 1.00 0.00 C

ATOM 2854 CG PRO A 183 12.134 −0.530 −27.966 1.00 0.00 C
ATOM 2855 CD PRO A 183 11.715 −0.676 −29.423 1.00 0.00 C
ATOM 2856 HA PRO A 183 10.952 2.016 −28.331 1.00 0.00 H
ATOM 2857 HB2 PRO A 183 12.705 1.300 −26.940 1.00 0.00 H
ATOM 2858 HB3 PRO A 183 13.737 0.842 −28.334 1.00 0.00 H
ATOM 2859 HG2 PRO A 183 12.869 −1.283 −27.677 1.00 0.00 H
ATOM 2860 HG3 PRO A 183 11.253 −0.576 −27.323 1.00 0.00 H
ATOM 2861 HD2 PRO A 183 10.923 −1.421 −29.462 1.00 0.00 H
ATOM 2862 HD3 PRO A 183 12.563 −1.002 −30.024 1.00 0.00 H
ATOM 2863 N ILE A 184 12.220 3.398 −30.643 1.00 0.00 N
ATOM 2864 CA ILE A 184 12.958 4.432 −31.395 1.00 0.00 C
ATOM 2865 C ILE A 184 12.998 5.847 −30.775 1.00 0.00 C
ATOM 2866 O ILE A 184 13.517 6.759 −31.408 1.00 0.00 O
ATOM 2867 CB ILE A 184 12.403 4.515 −32.837 1.00 0.00 C
ATOM 2868 CG1 ILE A 184 10.954 5.064 −32.857 1.00 0.00 C
ATOM 2869 CG2 ILE A 184 12.518 3.153 −33.545 1.00 0.00 C
ATOM 2870 CD1 ILE A 184 10.458 5.442 −34.253 1.00 0.00 C
ATOM 2871 H ILE A 184 11.435 2.960 −31.114 1.00 0.00 H
ATOM 2872 HA ILE A 184 13.996 4.105 −31.458 1.00 0.00 H
ATOM 2873 HB ILE A 184 13.034 5.212 −33.393 1.00 0.00 H
ATOM 2874 HG12 ILE A 184 10.274 4.326 −32.431 1.00 0.00 H
ATOM 2875 HG13 ILE A 184 10.891 5.966 −32.249 1.00 0.00 H
ATOM 2876 HD11 ILE A 184 9.459 5.873 −34.178 1.00 0.00 H
ATOM 2877 HD12 ILE A 184 11.129 6.174 −34.703 1.00 0.00 H
ATOM 2878 HD13 ILE A 184 10.410 4.556 −34.877 1.00 0.00 H
ATOM 2879 HG21 ILE A 184 13.522 2.749 −33.417 1.00 0.00 H
ATOM 2880 HG22 ILE A 184 12.348 3.284 −34.610 1.00 0.00 H
ATOM 2881 HG23 ILE A 184 11.787 2.448 −33.152 1.00 0.00 H
ATOM 2882 N ILE A 185 12.392 6.090 −29.610 1.00 0.00 N
ATOM 2883 CA ILE A 185 12.001 7.441 −29.146 1.00 0.00 C

ATOM	2884	C	ILE	A	185	13.188	8.421	−29.028	1.00	0.00	C
ATOM	2885	O	ILE	A	185	13.058	9.583	−29.403	1.00	0.00	O
ATOM	2886	CB	ILE	A	185	11.196	7.326	−27.825	1.00	0.00	C
ATOM	2887	CG1	ILE	A	185	9.871	6.565	−28.090	1.00	0.00	C
ATOM	2888	CG2	ILE	A	185	10.911	8.706	−27.199	1.00	0.00	C
ATOM	2889	CD1	ILE	A	185	9.036	6.268	−26.837	1.00	0.00	C
ATOM	2890	H	ILE	A	185	12.039	5.288	−29.108	1.00	0.00	H
ATOM	2891	HA	ILE	A	185	11.339	7.873	−29.898	1.00	0.00	H
ATOM	2892	HB	ILE	A	185	11.790	6.753	−27.111	1.00	0.00	H
ATOM	2893	HG12	ILE	A	185	9.260	7.138	−28.789	1.00	0.00	H
ATOM	2894	HG13	ILE	A	185	10.092	5.604	−28.554	1.00	0.00	H
ATOM	2895	HG21	ILE	A	185	11.843	9.214	−26.946	1.00	0.00	H
ATOM	2896	HG22	ILE	A	185	10.346	8.603	−26.275	1.00	0.00	H
ATOM	2897	HG23	ILE	A	185	10.349	9.326	−27.899	1.00	0.00	H
ATOM	2898	HD11	ILE	A	185	8.212	5.606	−27.106	1.00	0.00	H
ATOM	2899	HD12	ILE	A	185	8.616	7.187	−26.429	1.00	0.00	H
ATOM	2900	HD13	ILE	A	185	9.654	5.778	−26.084	1.00	0.00	H
ATOM	2901	N	HIS	A	186	14.368	7.941	−28.626	1.00	0.00	N
ATOM	2902	CA	HIS	A	186	15.611	8.740	−28.572	1.00	0.00	C
ATOM	2903	C	HIS	A	186	16.465	8.635	−29.852	1.00	0.00	C
ATOM	2904	O	HIS	A	186	17.497	9.294	−29.995	1.00	0.00	O
ATOM	2905	CB	HIS	A	186	16.415	8.312	−27.335	1.00	0.00	C
ATOM	2906	CG	HIS	A	186	15.611	8.358	−26.059	1.00	0.00	C
ATOM	2907	ND1	HIS	A	186	15.133	9.520	−25.452	1.00	0.00	N
ATOM	2908	CD2	HIS	A	186	15.178	7.278	−25.345	1.00	0.00	C
ATOM	2909	CE1	HIS	A	186	14.431	9.118	−24.384	1.00	0.00	C
ATOM	2910	NE2	HIS	A	186	14.440	7.776	−24.293	1.00	0.00	N
ATOM	2911	H	HIS	A	186	14.411	6.969	−28.363	1.00	0.00	H
ATOM	2912	HA	HIS	A	186	15.350	9.793	−28.449	1.00	0.00	H
ATOM	2913	HB2	HIS	A	186	17.275	8.974	−27.222	1.00	0.00	H

ATOM	2914	HB3	HIS	A	186	16.787	7.297	−27.482	1.00	0.00	H
ATOM	2915	HD2	HIS	A	186	15.374	6.240	−25.575	1.00	0.00	H
ATOM	2916	HE1	HIS	A	186	13.930	9.786	−23.696	1.00	0.00	H
ATOM	2917	HE2	HIS	A	186	13.986	7.236	−23.571	1.00	0.00	H
ATOM	2918	N	CYS	A	187	16.106	7.719	−30.747	1.00	0.00	N
ATOM	2919	CA	CYS	A	187	16.943	7.215	−31.828	1.00	0.00	C
ATOM	2920	C	CYS	A	187	16.730	8.024	−33.110	1.00	0.00	C
ATOM	2921	O	CYS	A	187	15.987	7.623	−33.999	1.00	0.00	O
ATOM	2922	CB	CYS	A	187	16.640	5.727	−31.996	1.00	0.00	C
ATOM	2923	SG	CYS	A	187	16.989	4.785	−30.494	1.00	0.00	S
ATOM	2924	H	CYS	A	187	15.189	7.300	−30.651	1.00	0.00	H
ATOM	2925	HA	CYS	A	187	17.992	7.304	−31.539	1.00	0.00	H
ATOM	2926	HB2	CYS	A	187	15.593	5.603	−32.266	1.00	0.00	H
ATOM	2927	HB3	CYS	A	187	17.245	5.326	−32.810	1.00	0.00	H
ATOM	2928	N	LYS	A	188	17.468	9.137	−33.223	1.00	0.00	N
ATOM	2929	CA	LYS	A	188	17.288	10.247	−34.190	1.00	0.00	C
ATOM	2930	C	LYS	A	188	17.003	9.892	−35.669	1.00	0.00	C
ATOM	2931	O	LYS	A	188	16.627	10.788	−36.419	1.00	0.00	O
ATOM	2932	CB	LYS	A	188	18.555	11.125	−34.132	1.00	0.00	C
ATOM	2933	CG	LYS	A	188	18.271	12.622	−34.346	1.00	0.00	C
ATOM	2934	CD	LYS	A	188	19.472	13.439	−34.854	1.00	0.00	C
ATOM	2935	CE	LYS	A	188	20.773	13.279	−34.050	1.00	0.00	C
ATOM	2936	NZ	LYS	A	188	21.578	12.114	−34.501	1.00	0.00	N
ATOM	2937	H	LYS	A	188	17.979	9.359	−32.377	1.00	0.00	H
ATOM	2938	HA	LYS	A	188	16.429	10.831	−33.854	1.00	0.00	H
ATOM	2939	HB2	LYS	A	188	19.038	11.019	−33.158	1.00	0.00	H
ATOM	2940	HB3	LYS	A	188	19.246	10.766	−34.897	1.00	0.00	H
ATOM	2941	HG2	LYS	A	188	17.929	13.045	−33.400	1.00	0.00	H
ATOM	2942	HG3	LYS	A	188	17.465	12.750	−35.067	1.00	0.00	H
ATOM	2943	HD2	LYS	A	188	19.654	13.195	−35.900	1.00	0.00	H

ATOM 2944 HD3 LYS A 188 19.182 14.490 −34.821 1.00 0.00 H
ATOM 2945 HE2 LYS A 188 21.364 14.190 −34.184 1.00 0.00 H
ATOM 2946 HE3 LYS A 188 20.525 13.196 −32.987 1.00 0.00 H
ATOM 2947 HZ1 LYS A 188 22.433 12.032 −33.968 1.00 0.00 H
ATOM 2948 HZ2 LYS A 188 21.825 12.216 −35.484 1.00 0.00 H
ATOM 2949 HZ3 LYS A 188 21.054 11.254 −34.408 1.00 0.00 H
ATOM 2950 N TYR A 189 17.363 8.693 −36.128 1.00 0.00 N
ATOM 2951 CA TYR A 189 17.205 8.242 −37.521 1.00 0.00 C
ATOM 2952 C TYR A 189 16.382 6.947 −37.672 1.00 0.00 C
ATOM 2953 O TYR A 189 16.008 6.594 −38.790 1.00 0.00 O
ATOM 2954 CB TYR A 189 18.598 8.056 −38.152 1.00 0.00 C
ATOM 2955 CG TYR A 189 19.576 9.209 −37.974 1.00 0.00 C
ATOM 2956 CD1 TYR A 189 19.188 10.530 −38.274 1.00 0.00 C
ATOM 2957 CD2 TYR A 189 20.883 8.952 −37.514 1.00 0.00 C
ATOM 2958 CE1 TYR A 189 20.093 11.594 −38.094 1.00 0.00 C
ATOM 2959 CE2 TYR A 189 21.793 10.011 −37.333 1.00 0.00 C
ATOM 2960 CZ TYR A 189 21.396 11.331 −37.625 1.00 0.00 C
ATOM 2961 OH TYR A 189 22.249 12.355 −37.364 1.00 0.00 O
ATOM 2962 H TYR A 189 17.625 8.005 −35.441 1.00 0.00 H
ATOM 2963 HA TYR A 189 16.673 9.004 −38.090 1.00 0.00 H
ATOM 2964 HB2 TYR A 189 18.473 7.878 −39.221 1.00 0.00 H
ATOM 2965 HB3 TYR A 189 19.041 7.153 −37.726 1.00 0.00 H
ATOM 2966 HD1 TYR A 189 18.185 10.729 −38.630 1.00 0.00 H
ATOM 2967 HD2 TYR A 189 21.192 7.932 −37.316 1.00 0.00 H
ATOM 2968 HE1 TYR A 189 19.784 12.605 −38.316 1.00 0.00 H
ATOM 2969 HE2 TYR A 189 22.800 9.806 −36.999 1.00 0.00 H
ATOM 2970 HH TYR A 189 22.741 12.588 −38.161 1.00 0.00 H
ATOM 2971 N SER A 190 16.131 6.226 −36.577 1.00 0.00 N
ATOM 2972 CA SER A 190 15.590 4.867 −36.598 1.00 0.00 C
ATOM 2973 C SER A 190 14.095 4.848 −36.899 1.00 0.00 C

ATOM	2974	O	SER	A	190	13.304	5.553	−36.271	1.00	0.00	O
ATOM	2975	CB	SER	A	190	15.873	4.168	−35.269	1.00	0.00	C
ATOM	2976	OG	SER	A	190	17.270	4.118	−35.054	1.00	0.00	O
ATOM	2977	H	SER	A	190	16.345	6.610	−35.667	1.00	0.00	H
ATOM	2978	HA	SER	A	190	16.098	4.310	−37.385	1.00	0.00	H
ATOM	2979	HB2	SER	A	190	15.464	3.160	−35.295	1.00	0.00	H
ATOM	2980	HB3	SER	A	190	15.396	4.719	−34.458	1.00	0.00	H
ATOM	2981	HG	SER	A	190	17.654	3.627	−35.827	1.00	0.00	H
ATOM	2982	N	LYS	A	191	13.677	3.990	−37.833	1.00	0.00	N
ATOM	2983	CA	LYS	A	191	12.264	3.831	−38.199	1.00	0.00	C
ATOM	2984	C	LYS	A	191	11.568	2.893	−37.218	1.00	0.00	C
ATOM	2985	O	LYS	A	191	12.111	1.845	−36.884	1.00	0.00	O
ATOM	2986	CB	LYS	A	191	12.112	3.321	−39.641	1.00	0.00	C
ATOM	2987	CG	LYS	A	191	12.939	4.121	−40.664	1.00	0.00	C
ATOM	2988	CD	LYS	A	191	12.654	3.665	−42.100	1.00	0.00	C
ATOM	2989	CE	LYS	A	191	11.297	4.189	−42.585	1.00	0.00	C
ATOM	2990	NZ	LYS	A	191	10.905	3.574	−43.874	1.00	0.00	N
ATOM	2991	H	LYS	A	191	14.360	3.343	−38.215	1.00	0.00	H
ATOM	2992	HA	LYS	A	191	11.787	4.810	−38.122	1.00	0.00	H
ATOM	2993	HB2	LYS	A	191	12.415	2.276	−39.685	1.00	0.00	H
ATOM	2994	HB3	LYS	A	191	11.056	3.371	−39.905	1.00	0.00	H
ATOM	2995	HG2	LYS	A	191	12.722	5.186	−40.566	1.00	0.00	H
ATOM	2996	HG3	LYS	A	191	14.001	3.964	−40.464	1.00	0.00	H
ATOM	2997	HD2	LYS	A	191	13.442	4.051	−42.750	1.00	0.00	H
ATOM	2998	HD3	LYS	A	191	12.676	2.573	−42.138	1.00	0.00	H
ATOM	2999	HE2	LYS	A	191	11.359	5.277	−42.679	1.00	0.00	H
ATOM	3000	HE3	LYS	A	191	10.535	3.954	−41.836	1.00	0.00	H
ATOM	3001	HZ1	LYS	A	191	10.055	3.980	−44.235	1.00	0.00	H
ATOM	3002	HZ2	LYS	A	191	11.641	3.610	−44.560	1.00	0.00	H
ATOM	3003	HZ3	LYS	A	191	10.650	2.590	−43.717	1.00	0.00	H

ATOM	3004	N	ALA	A	192	10.265	3.094	−37.037	1.00	0.00	N
ATOM	3005	CA	ALA	A	192	9.406	2.165	−36.295	1.00	0.00	C
ATOM	3006	C	ALA	A	192	9.332	0.763	−36.950	1.00	0.00	C
ATOM	3007	O	ALA	A	192	9.303	−0.252	−36.257	1.00	0.00	O
ATOM	3008	CB	ALA	A	192	8.033	2.837	−36.171	1.00	0.00	C
ATOM	3009	H	ALA	A	192	9.870	3.969	−37.335	1.00	0.00	H
ATOM	3010	HA	ALA	A	192	9.817	2.033	−35.292	1.00	0.00	H
ATOM	3011	HB1	ALA	A	192	7.403	2.260	−35.498	1.00	0.00	H
ATOM	3012	HB2	ALA	A	192	7.551	2.900	−37.148	1.00	0.00	H
ATOM	3013	HB3	ALA	A	192	8.141	3.838	−35.753	1.00	0.00	H
ATOM	3014	N	GLU	A	193	9.560	0.701	−38.268	1.00	0.00	N
ATOM	3015	CA	GLU	A	193	9.720	−0.543	−39.030	1.00	0.00	C
ATOM	3016	C	GLU	A	193	10.927	−1.393	−38.602	1.00	0.00	C
ATOM	3017	O	GLU	A	193	10.941	−2.596	−38.856	1.00	0.00	O
ATOM	3018	CB	GLU	A	193	9.937	−0.226	−40.517	1.00	0.00	C
ATOM	3019	CG	GLU	A	193	8.928	0.727	−41.166	1.00	0.00	C
ATOM	3020	CD	GLU	A	193	8.927	0.524	−42.686	1.00	0.00	C
ATOM	3021	OE1	GLU	A	193	8.488	−0.569	−43.126	1.00	0.00	O
ATOM	3022	OE2	GLU	A	193	9.553	1.363	−43.372	1.00	0.00	O
ATOM	3023	H	GLU	A	193	9.592	1.566	−38.777	1.00	0.00	H
ATOM	3024	HA	GLU	A	193	8.822	−1.152	−38.918	1.00	0.00	H
ATOM	3025	HB2	GLU	A	193	10.934	0.198	−40.655	1.00	0.00	H
ATOM	3026	HB3	GLU	A	193	9.908	−1.178	−41.047	1.00	0.00	H
ATOM	3027	HG2	GLU	A	193	7.929	0.531	−40.779	1.00	0.00	H
ATOM	3028	HG3	GLU	A	193	9.188	1.760	−40.914	1.00	0.00	H
ATOM	3029	N	ASN	A	194	11.999	−0.778	−38.088	1.00	0.00	N
ATOM	3030	CA	ASN	A	194	13.317	−1.412	−37.983	1.00	0.00	C
ATOM	3031	C	ASN	A	194	13.295	−2.685	−37.127	1.00	0.00	C
ATOM	3032	O	ASN	A	194	13.847	−3.702	−37.548	1.00	0.00	O
ATOM	3033	CB	ASN	A	194	14.334	−0.401	−37.432	1.00	0.00	C

ATOM	3034	CG	ASN	A	194	14.736	0.694	−38.409	1.00	0.00		C
ATOM	3035	OD1	ASN	A	194	14.377	0.694	−39.578	1.00	0.00		O
ATOM	3036	ND2	ASN	A	194	15.578	1.597	−37.972	1.00	0.00		N
ATOM	3037	H	ASN	A	194	11.936	0.209	−37.867	1.00	0.00		H
ATOM	3038	HA	ASN	A	194	13.638	−1.712	−38.981	1.00	0.00		H
ATOM	3039	HB2	ASN	A	194	13.953	0.057	−36.520	1.00	0.00		H
ATOM	3040	HB3	ASN	A	194	15.240	−0.942	−37.171	1.00	0.00		H
ATOM	3041	HD21	ASN	A	194	15.926	1.499	−37.019	1.00	0.00		H
ATOM	3042	HD22	ASN	A	194	16.283	1.933	−38.624	1.00	0.00		H
ATOM	3043	N	CYS	A	195	12.455	−2.704	−36.085	1.00	0.00		N
ATOM	3044	CA	CYS	A	195	12.134	−3.904	−35.319	1.00	0.00		C
ATOM	3045	C	CYS	A	195	11.551	−5.013	−36.213	1.00	0.00		C
ATOM	3046	O	CYS	A	195	12.182	−6.054	−36.388	1.00	0.00		O
ATOM	3047	CB	CYS	A	195	11.164	−3.548	−34.183	1.00	0.00		C
ATOM	3048	SG	CYS	A	195	11.826	−2.451	−32.905	1.00	0.00		S
ATOM	3049	H	CYS	A	195	11.987	−1.841	−35.844	1.00	0.00		H
ATOM	3050	HA	CYS	A	195	13.053	−4.289	−34.877	1.00	0.00		H
ATOM	3051	HB2	CYS	A	195	10.872	−4.475	−33.688	1.00	0.00		H
ATOM	3052	HB3	CYS	A	195	10.262	−3.095	−34.596	1.00	0.00		H
ATOM	3053	N	ARG	A	196	10.420	−4.751	−36.883	1.00	0.00		N
ATOM	3054	CA	ARG	A	196	9.716	−5.702	−37.764	1.00	0.00		C
ATOM	3055	C	ARG	A	196	10.585	−6.213	−38.916	1.00	0.00		C
ATOM	3056	O	ARG	A	196	10.500	−7.387	−39.285	1.00	0.00		O
ATOM	3057	CB	ARG	A	196	8.444	−5.004	−38.282	1.00	0.00		C
ATOM	3058	CG	ARG	A	196	7.640	−5.814	−39.317	1.00	0.00		C
ATOM	3059	CD	ARG	A	196	6.301	−5.154	−39.682	1.00	0.00		C
ATOM	3060	NE	ARG	A	196	6.446	−3.741	−40.066	1.00	0.00		N
ATOM	3061	CZ	ARG	A	196	6.895	−3.205	−41.180	1.00	0.00		C
ATOM	3062	NH1	ARG	A	196	7.261	−3.903	−42.213	1.00	0.00		N
ATOM	3063	NH2	ARG	A	196	6.977	−1.924	−41.282	1.00	0.00		N

ATOM	3064	H	ARG	A	196	10.031	−3.820	−36.799	1.00	0.00	H
ATOM	3065	HA	ARG	A	196	9.426	−6.570	−37.175	1.00	0.00	H
ATOM	3066	HB2	ARG	A	196	7.798	−4.790	−37.432	1.00	0.00	H
ATOM	3067	HB3	ARG	A	196	8.725	−4.051	−38.733	1.00	0.00	H
ATOM	3068	HG2	ARG	A	196	8.229	−5.930	−40.227	1.00	0.00	H
ATOM	3069	HG3	ARG	A	196	7.429	−6.803	−38.911	1.00	0.00	H
ATOM	3070	HD2	ARG	A	196	5.648	−5.189	−38.807	1.00	0.00	H
ATOM	3071	HD3	ARG	A	196	5.813	−5.714	−40.481	1.00	0.00	H
ATOM	3072	HE	ARG	A	196	6.292	−3.071	−39.305	1.00	0.00	H
ATOM	3073	HH11	ARG	A	196	7.090	−4.884	−42.204	1.00	0.00	H
ATOM	3074	HH12	ARG	A	196	7.563	−3.401	−43.028	1.00	0.00	H
ATOM	3075	HH21	ARG	A	196	6.688	−1.368	−40.472	1.00	0.00	H
ATOM	3076	HH22	ARG	A	196	7.436	−1.455	−42.062	1.00	0.00	H
ATOM	3077	N	LEU	A	197	11.412	−5.347	−39.496	1.00	0.00	N
ATOM	3078	CA	LEU	A	197	12.327	−5.696	−40.582	1.00	0.00	C
ATOM	3079	C	LEU	A	197	13.477	−6.595	−40.096	1.00	0.00	C
ATOM	3080	O	LEU	A	197	13.667	−7.663	−40.682	1.00	0.00	O
ATOM	3081	CB	LEU	A	197	12.843	−4.406	−41.246	1.00	0.00	C
ATOM	3082	CG	LEU	A	197	11.758	−3.560	−41.947	1.00	0.00	C
ATOM	3083	CD1	LEU	A	197	12.386	−2.273	−42.481	1.00	0.00	C
ATOM	3084	CD2	LEU	A	197	11.077	−4.288	−43.109	1.00	0.00	C
ATOM	3085	H	LEU	A	197	11.393	−4.387	−39.159	1.00	0.00	H
ATOM	3086	HA	LEU	A	197	11.783	−6.273	−41.329	1.00	0.00	H
ATOM	3087	HB2	LEU	A	197	13.316	−3.794	−40.478	1.00	0.00	H
ATOM	3088	HB3	LEU	A	197	13.605	−4.671	−41.978	1.00	0.00	H
ATOM	3089	HG	LEU	A	197	10.983	−3.293	−41.236	1.00	0.00	H
ATOM	3090	HD11	LEU	A	197	13.163	−2.500	−43.210	1.00	0.00	H
ATOM	3091	HD12	LEU	A	197	12.819	−1.708	−41.654	1.00	0.00	H
ATOM	3092	HD13	LEU	A	197	11.617	−1.656	−42.948	1.00	0.00	H
ATOM	3093	HD21	LEU	A	197	10.374	−3.608	−43.592	1.00	0.00	H

ATOM 3094 HD22 LEU A 197 11.810 −4.619 −43.839 1.00 0.00 H
ATOM 3095 HD23 LEU A 197 10.518 −5.145 −42.739 1.00 0.00 H
ATOM 3096 N SER A 198 14.107 −6.260 −38.961 1.00 0.00 N
ATOM 3097 CA SER A 198 15.251 −6.986 −38.379 1.00 0.00 C
ATOM 3098 C SER A 198 14.941 −8.426 −37.955 1.00 0.00 C
ATOM 3099 O SER A 198 15.838 −9.256 −38.021 1.00 0.00 O
ATOM 3100 CB SER A 198 15.818 −6.197 −37.186 1.00 0.00 C
ATOM 3101 OG SER A 198 16.835 −6.905 −36.490 1.00 0.00 O
ATOM 3102 H SER A 198 13.827 −5.400 −38.495 1.00 0.00 H
ATOM 3103 HA SER A 198 16.036 −7.048 −39.133 1.00 0.00 H
ATOM 3104 HB2 SER A 198 16.217 −5.248 −37.544 1.00 0.00 H
ATOM 3105 HB3 SER A 198 15.008 −5.986 −36.485 1.00 0.00 H
ATOM 3106 HG SER A 198 17.565 −7.113 −37.108 1.00 0.00 H
ATOM 3107 N MET A 199 13.676 −8.795 −37.708 1.00 0.00 N
ATOM 3108 CA MET A 199 13.304 −10.161 −37.283 1.00 0.00 C
ATOM 3109 C MET A 199 13.583 −11.284 −38.309 1.00 0.00 C
ATOM 3110 O MET A 199 13.324 −12.448 −38.011 1.00 0.00 O
ATOM 3111 CB MET A 199 11.833 −10.229 −36.832 1.00 0.00 C
ATOM 3112 CG MET A 199 11.420 −9.198 −35.782 1.00 0.00 C
ATOM 3113 SD MET A 199 12.495 −9.055 −34.328 1.00 0.00 S
ATOM 3114 CE MET A 199 11.635 −7.723 −33.450 1.00 0.00 C
ATOM 3115 H MET A 199 12.984 −8.061 −37.633 1.00 0.00 H
ATOM 3116 HA MET A 199 13.914 −10.398 −36.413 1.00 0.00 H
ATOM 3117 HB2 MET A 199 11.193 −10.096 −37.702 1.00 0.00 H
ATOM 3118 HB3 MET A 199 11.643 −11.219 −36.413 1.00 0.00 H
ATOM 3119 HG2 MET A 199 11.348 −8.232 −36.269 1.00 0.00 H
ATOM 3120 HG3 MET A 199 10.423 −9.458 −35.435 1.00 0.00 H
ATOM 3121 HE1 MET A 199 11.651 −6.816 −34.049 1.00 0.00 H
ATOM 3122 HE2 MET A 199 12.135 −7.528 −32.504 1.00 0.00 H
ATOM 3123 HE3 MET A 199 10.602 −8.010 −33.260 1.00 0.00 H

ATOM 3124 N GLY A 200 14.090 −10.986 −39.510 1.00 0.00 N
ATOM 3125 CA GLY A 200 14.535 −11.989 −40.483 1.00 0.00 C
ATOM 3126 C GLY A 200 15.872 −11.627 −41.135 1.00 0.00 C
ATOM 3127 O GLY A 200 16.190 −10.452 −41.289 1.00 0.00 O
ATOM 3128 H GLY A 200 14.437 −10.043 −39.638 1.00 0.00 H
ATOM 3129 HA2 GLY A 200 14.650 −12.957 −39.994 1.00 0.00 H
ATOM 3130 HA3 GLY A 200 13.784 −12.093 −41.264 1.00 0.00 H
ATOM 3131 N VAL A 201 16.570 −12.642 −41.661 1.00 0.00 N
ATOM 3132 CA VAL A 201 17.931 −12.569 −42.255 1.00 0.00 C
ATOM 3133 C VAL A 201 18.082 −11.520 −43.374 1.00 0.00 C
ATOM 3134 O VAL A 201 19.172 −11.009 −43.616 1.00 0.00 O
ATOM 3135 CB VAL A 201 18.325 −13.981 −42.756 1.00 0.00 C
ATOM 3136 CG1 VAL A 201 19.585 −14.037 −43.629 1.00 0.00 C
ATOM 3137 CG2 VAL A 201 18.532 −14.928 −41.567 1.00 0.00 C
ATOM 3138 H VAL A 201 16.219 −13.569 −41.474 1.00 0.00 H
ATOM 3139 HA VAL A 201 18.637 −12.280 −41.474 1.00 0.00 H
ATOM 3140 HB VAL A 201 17.502 −14.371 −43.357 1.00 0.00 H
ATOM 3141 HG11 VAL A 201 19.409 −13.547 −44.585 1.00 0.00 H
ATOM 3142 HG12 VAL A 201 19.862 −15.074 −43.823 1.00 0.00 H
ATOM 3143 HG13 VAL A 201 20.410 −13.540 −43.121 1.00 0.00 H
ATOM 3144 HG21 VAL A 201 18.754 −15.933 −41.929 1.00 0.00 H
ATOM 3145 HG22 VAL A 201 17.640 −14.978 −40.946 1.00 0.00 H
ATOM 3146 HG23 VAL A 201 19.370 −14.585 −40.957 1.00 0.00 H
ATOM 3147 N ASN A 202 16.979 −11.096 −43.994 1.00 0.00 N
ATOM 3148 CA ASN A 202 16.870 −9.793 −44.650 1.00 0.00 C
ATOM 3149 C ASN A 202 15.631 −9.063 −44.101 1.00 0.00 C
ATOM 3150 O ASN A 202 14.590 −9.697 −43.926 1.00 0.00 O
ATOM 3151 CB ASN A 202 16.735 −9.944 −46.180 1.00 0.00 C
ATOM 3152 CG ASN A 202 17.873 −10.554 −46.983 1.00 0.00 C
ATOM 3153 OD1 ASN A 202 17.722 −10.733 −48.180 1.00 0.00 O

ATOM	3154	ND2	ASN	A	202	19.032	−10.814	−46.431	1.00	0.00	N
ATOM	3155	H	ASN	A	202	16.115	−11.462	−43.624	1.00	0.00	H
ATOM	3156	HA	ASN	A	202	17.746	−9.182	−44.418	1.00	0.00	H
ATOM	3157	HB2	ASN	A	202	16.587	−8.950	−46.595	1.00	0.00	H
ATOM	3158	HB3	ASN	A	202	15.851	−10.539	−46.393	1.00	0.00	H
ATOM	3159	HD21	ASN	A	202	19.178	−10.720	−45.429	1.00	0.00	H
ATOM	3160	HD22	ASN	A	202	19.747	−11.200	−47.020	1.00	0.00	H
ATOM	3161	N	SER	A	203	15.602	−7.739	−44.270	1.00	0.00	N
ATOM	3162	CA	SER	A	203	14.370	−6.929	−44.221	1.00	0.00	C
ATOM	3163	C	SER	A	203	13.289	−7.416	−45.203	1.00	0.00	C
ATOM	3164	O	SER	A	203	12.124	−7.542	−44.834	1.00	0.00	O
ATOM	3165	CB	SER	A	203	14.743	−5.479	−44.547	1.00	0.00	C
ATOM	3166	OG	SER	A	203	15.415	−5.410	−45.796	1.00	0.00	O
ATOM	3167	H	SER	A	203	16.455	−7.263	−44.525	1.00	0.00	H
ATOM	3168	HA	SER	A	203	13.953	−6.970	−43.215	1.00	0.00	H
ATOM	3169	HB2	SER	A	203	13.845	−4.863	−44.583	1.00	0.00	H
ATOM	3170	HB3	SER	A	203	15.398	−5.100	−43.765	1.00	0.00	H
ATOM	3171	HG	SER	A	203	15.723	−4.504	−45.924	1.00	0.00	H
ATOM	3172	N	LYS	A	204	13.710	−7.847	−46.401	1.00	0.00	N
ATOM	3173	CA	LYS	A	204	12.878	−8.440	−47.466	1.00	0.00	C
ATOM	3174	C	LYS	A	204	12.785	−9.978	−47.449	1.00	0.00	C
ATOM	3175	O	LYS	A	204	12.532	−10.576	−48.494	1.00	0.00	O
ATOM	3176	CB	LYS	A	204	13.310	−7.858	−48.830	1.00	0.00	C
ATOM	3177	CG	LYS	A	204	14.747	−8.213	−49.281	1.00	0.00	C
ATOM	3178	CD	LYS	A	204	14.869	−9.300	−50.362	1.00	0.00	C
ATOM	3179	CE	LYS	A	204	14.234	−8.861	−51.689	1.00	0.00	C
ATOM	3180	NZ	LYS	A	204	14.296	−9.943	−52.702	1.00	0.00	N
ATOM	3181	H	LYS	A	204	14.678	−7.649	−46.608	1.00	0.00	H
ATOM	3182	HA	LYS	A	204	11.852	−8.109	−47.292	1.00	0.00	H
ATOM	3183	HB2	LYS	A	204	12.588	−8.162	−49.587	1.00	0.00	H

ATOM	3184	HB3	LYS	A	204	13.244	−6.770	−48.756	1.00	0.00	H
ATOM	3185	HG2	LYS	A	204	15.217	−7.307	−49.666	1.00	0.00	H
ATOM	3186	HG3	LYS	A	204	15.330	−8.531	−48.420	1.00	0.00	H
ATOM	3187	HD2	LYS	A	204	14.407	−10.223	−50.016	1.00	0.00	H
ATOM	3188	HD3	LYS	A	204	15.929	−9.503	−50.522	1.00	0.00	H
ATOM	3189	HE2	LYS	A	204	13.191	−8.584	−51.508	1.00	0.00	H
ATOM	3190	HE3	LYS	A	204	14.762	−7.971	−52.048	1.00	0.00	H
ATOM	3191	HZ1	LYS	A	204	13.897	−9.633	−53.579	1.00	0.00	H
ATOM	3192	HZ2	LYS	A	204	13.776	−10.749	−52.378	1.00	0.00	H
ATOM	3193	HZ3	LYS	A	204	15.256	−10.223	−52.860	1.00	0.00	H
ATOM	3194	N	SER	A	205	13.146	−10.635	−46.343	1.00	0.00	N
ATOM	3195	CA	SER	A	205	13.092	−12.102	−46.226	1.00	0.00	C
ATOM	3196	C	SER	A	205	11.665	−12.640	−46.381	1.00	0.00	C
ATOM	3197	O	SER	A	205	10.704	−11.955	−46.042	1.00	0.00	O
ATOM	3198	CB	SER	A	205	13.662	−12.563	−44.879	1.00	0.00	C
ATOM	3199	OG	SER	A	205	12.888	−12.063	−43.807	1.00	0.00	O
ATOM	3200	H	SER	A	205	13.349	−10.104	−45.505	1.00	0.00	H
ATOM	3201	HA	SER	A	205	13.704	−12.532	−47.018	1.00	0.00	H
ATOM	3202	HB2	SER	A	205	13.660	−13.654	−44.840	1.00	0.00	H
ATOM	3203	HB3	SER	A	205	14.690	−12.219	−44.782	1.00	0.00	H
ATOM	3204	HG	SER	A	205	12.640	−12.825	−43.234	1.00	0.00	H
ATOM	3205	N	HIS	A	206	11.546	−13.915	−46.763	1.00	0.00	N
ATOM	3206	CA	HIS	A	206	10.296	−14.679	−46.658	1.00	0.00	C
ATOM	3207	C	HIS	A	206	9.943	−14.948	−45.185	1.00	0.00	C
ATOM	3208	O	HIS	A	206	9.006	−14.345	−44.673	1.00	0.00	O
ATOM	3209	CB	HIS	A	206	10.432	−15.959	−47.502	1.00	0.00	C
ATOM	3210	CG	HIS	A	206	9.279	−16.924	−47.406	1.00	0.00	C
ATOM	3211	ND1	HIS	A	206	8.969	−17.668	−46.273	1.00	0.00	N
ATOM	3212	CD2	HIS	A	206	8.496	−17.350	−48.440	1.00	0.00	C
ATOM	3213	CE1	HIS	A	206	8.020	−18.540	−46.645	1.00	0.00	C

ATOM 3214 NE2 HIS A 206 7.702 −18.357 −47.936 1.00 0.00 N
ATOM 3215 H HIS A 206 12.379 −14.419 −47.020 1.00 0.00 H
ATOM 3216 HA HIS A 206 9.473 −14.088 −47.064 1.00 0.00 H
ATOM 3217 HB2 HIS A 206 11.330 −16.496 −47.193 1.00 0.00 H
ATOM 3218 HB3 HIS A 206 10.561 −15.675 −48.547 1.00 0.00 H
ATOM 3219 HD2 HIS A 206 8.551 −17.017 −49.466 1.00 0.00 H
ATOM 3220 HE1 HIS A 206 7.586 −19.295 −45.999 1.00 0.00 H
ATOM 3221 HE2 HIS A 206 7.050 −18.931 −48.454 1.00 0.00 H
ATOM 3222 N TYR A 207 10.840 −15.602 −44.442 1.00 0.00 N
ATOM 3223 CA TYR A 207 10.692 −15.844 −43.003 1.00 0.00 C
ATOM 3224 C TYR A 207 11.130 −14.660 −42.129 1.00 0.00 C
ATOM 3225 O TYR A 207 12.218 −14.113 −42.339 1.00 0.00 O
ATOM 3226 CB TYR A 207 11.478 −17.109 −42.625 1.00 0.00 C
ATOM 3227 CG TYR A 207 10.986 −18.339 −43.353 1.00 0.00 C
ATOM 3228 CD1 TYR A 207 9.708 −18.833 −43.050 1.00 0.00 C
ATOM 3229 CD2 TYR A 207 11.751 −18.941 −44.370 1.00 0.00 C
ATOM 3230 CE1 TYR A 207 9.187 −19.924 −43.757 1.00 0.00 C
ATOM 3231 CE2 TYR A 207 11.227 −20.040 −45.081 1.00 0.00 C
ATOM 3232 CZ TYR A 207 9.943 −20.535 −44.776 1.00 0.00 C
ATOM 3233 OH TYR A 207 9.435 −21.585 −45.474 1.00 0.00 O
ATOM 3234 H TYR A 207 11.551 −16.125 −44.923 1.00 0.00 H
ATOM 3235 HA TYR A 207 9.640 −16.035 −42.799 1.00 0.00 H
ATOM 3236 HB2 TYR A 207 12.538 −16.955 −42.833 1.00 0.00 H
ATOM 3237 HB3 TYR A 207 11.375 −17.288 −41.555 1.00 0.00 H
ATOM 3238 HD1 TYR A 207 9.112 −18.356 −42.291 1.00 0.00 H
ATOM 3239 HD2 TYR A 207 12.734 −18.564 −44.607 1.00 0.00 H
ATOM 3240 HE1 TYR A 207 8.191 −20.264 −43.550 1.00 0.00 H
ATOM 3241 HE2 TYR A 207 11.780 −20.492 −45.882 1.00 0.00 H
ATOM 3242 HH TYR A 207 8.488 −21.742 −45.241 1.00 0.00 H
ATOM 3243 N ILE A 208 10.432 −14.453 −41.007 1.00 0.00 N

ATOM 3244 CA ILE A 208 11.074 −14.082 −39.736 1.00 0.00 C
ATOM 3245 C ILE A 208 11.494 −15.341 −38.971 1.00 0.00 C
ATOM 3246 O ILE A 208 10.919 −16.417 −39.155 1.00 0.00 O
ATOM 3247 CB ILE A 208 10.209 −13.143 −38.860 1.00 0.00 C
ATOM 3248 CG1 ILE A 208 8.884 −13.727 −38.318 1.00 0.00 C
ATOM 3249 CG2 ILE A 208 9.940 −11.835 −39.618 1.00 0.00 C
ATOM 3250 CD1 ILE A 208 9.015 −14.513 −37.005 1.00 0.00 C
ATOM 3251 H ILE A 208 9.570 −14.983 −40.912 1.00 0.00 H
ATOM 3252 HA ILE A 208 11.991 −13.539 −39.960 1.00 0.00 H
ATOM 3253 HB ILE A 208 10.805 −12.866 −37.991 1.00 0.00 H
ATOM 3254 HG12 ILE A 208 8.406 −14.345 −39.072 1.00 0.00 H
ATOM 3255 HG13 ILE A 208 8.212 −12.899 −38.108 1.00 0.00 H
ATOM 3256 HG21 ILE A 208 9.251 −12.027 −40.432 1.00 0.00 H
ATOM 3257 HG22 ILE A 208 9.491 −11.105 −38.945 1.00 0.00 H
ATOM 3258 HG23 ILE A 208 10.874 −11.432 −40.008 1.00 0.00 H
ATOM 3259 HD11 ILE A 208 8.020 −14.728 −36.616 1.00 0.00 H
ATOM 3260 HD12 ILE A 208 9.557 −13.921 −36.266 1.00 0.00 H
ATOM 3261 HD13 ILE A 208 9.530 −15.457 −37.165 1.00 0.00 H
ATOM 3262 N LEU A 209 12.480 −15.180 −38.091 1.00 0.00 N
ATOM 3263 CA LEU A 209 12.918 −16.181 −37.124 1.00 0.00 C
ATOM 3264 C LEU A 209 12.405 −15.803 −35.725 1.00 0.00 C
ATOM 3265 O LEU A 209 12.503 −14.639 −35.334 1.00 0.00 O
ATOM 3266 CB LEU A 209 14.457 −16.280 −37.146 1.00 0.00 C
ATOM 3267 CG LEU A 209 15.110 −16.514 −38.520 1.00 0.00 C
ATOM 3268 CD1 LEU A 209 16.625 −16.636 −38.357 1.00 0.00 C
ATOM 3269 CD2 LEU A 209 14.604 −17.787 −39.190 1.00 0.00 C
ATOM 3270 H LEU A 209 12.833 −14.237 −37.958 1.00 0.00 H
ATOM 3271 HA LEU A 209 12.503 −17.155 −37.388 1.00 0.00 H
ATOM 3272 HB2 LEU A 209 14.866 −15.355 −36.745 1.00 0.00 H
ATOM 3273 HB3 LEU A 209 14.755 −17.089 −36.483 1.00 0.00 H

ATOM 3274 HG LEU A 209 14.906 −15.665 −39.175 1.00 0.00 H
ATOM 3275 HD11 LEU A 209 17.023 −15.726 −37.907 1.00 0.00 H
ATOM 3276 HD12 LEU A 209 16.863 −17.481 −37.710 1.00 0.00 H
ATOM 3277 HD13 LEU A 209 17.095 −16.786 −39.328 1.00 0.00 H
ATOM 3278 HD21 LEU A 209 15.135 −17.948 −40.127 1.00 0.00 H
ATOM 3279 HD22 LEU A 209 13.540 −17.702 −39.405 1.00 0.00 H
ATOM 3280 HD23 LEU A 209 14.770 −18.644 −38.536 1.00 0.00 H
ATOM 3281 N ARG A 210 12.060 −16.798 −34.904 1.00 0.00 N
ATOM 3282 CA ARG A 210 11.633 −16.638 −33.500 1.00 0.00 C
ATOM 3283 C ARG A 210 12.342 −17.667 −32.619 1.00 0.00 C
ATOM 3284 O ARG A 210 12.143 −18.859 −32.826 1.00 0.00 O
ATOM 3285 CB ARG A 210 10.104 −16.832 −33.448 1.00 0.00 C
ATOM 3286 CG ARG A 210 9.463 −16.556 −32.076 1.00 0.00 C
ATOM 3287 CD ARG A 210 9.222 −15.060 −31.838 1.00 0.00 C
ATOM 3288 NE ARG A 210 8.336 −14.813 −30.683 1.00 0.00 N
ATOM 3289 CZ ARG A 210 7.015 −14.863 −30.657 1.00 0.00 C
ATOM 3290 NH1 ARG A 210 6.290 −15.248 −31.660 1.00 0.00 N
ATOM 3291 NH2 ARG A 210 6.388 −14.676 −29.542 1.00 0.00 N
ATOM 3292 H ARG A 210 11.967 −17.724 −35.319 1.00 0.00 H
ATOM 3293 HA ARG A 210 11.884 −15.636 −33.148 1.00 0.00 H
ATOM 3294 HB2 ARG A 210 9.630 −16.192 −34.195 1.00 0.00 H
ATOM 3295 HB3 ARG A 210 9.878 −17.864 −33.725 1.00 0.00 H
ATOM 3296 HG2 ARG A 210 8.511 −17.078 −32.040 1.00 0.00 H
ATOM 3297 HG3 ARG A 210 10.081 −16.959 −31.276 1.00 0.00 H
ATOM 3298 HD2 ARG A 210 10.185 −14.583 −31.657 1.00 0.00 H
ATOM 3299 HD3 ARG A 210 8.781 −14.613 −32.731 1.00 0.00 H
ATOM 3300 HE ARG A 210 8.762 −14.671 −29.774 1.00 0.00 H
ATOM 3301 HH11 ARG A 210 6.741 −15.705 −32.431 1.00 0.00 H
ATOM 3302 HH12 ARG A 210 5.372 −15.615 −31.394 1.00 0.00 H
ATOM 3303 HH21 ARG A 210 6.952 −14.687 −28.703 1.00 0.00 H

ATOM 3304 HH22 ARG A 210 5.451 −15.085 −29.499 1.00 0.00 H
ATOM 3305 N SER A 211 13.165 −17.241 −31.657 1.00 0.00 N
ATOM 3306 CA SER A 211 13.763 −18.166 −30.675 1.00 0.00 C
ATOM 3307 C SER A 211 12.689 −18.807 −29.785 1.00 0.00 C
ATOM 3308 O SER A 211 11.583 −18.282 −29.644 1.00 0.00 O
ATOM 3309 CB SER A 211 14.818 −17.475 −29.800 1.00 0.00 C
ATOM 3310 OG SER A 211 15.889 −16.997 −30.591 1.00 0.00 O
ATOM 3311 H SER A 211 13.305 −16.253 −31.526 1.00 0.00 H
ATOM 3312 HA SER A 211 14.262 −18.969 −31.217 1.00 0.00 H
ATOM 3313 HB2 SER A 211 14.359 −16.639 −29.271 1.00 0.00 H
ATOM 3314 HB3 SER A 211 15.208 −18.183 −29.066 1.00 0.00 H
ATOM 3315 HG SER A 211 16.656 −17.591 −30.483 1.00 0.00 H
ATOM 3316 N GLY A 212 13.016 −19.934 −29.161 1.00 0.00 N
ATOM 3317 CA GLY A 212 12.067 −20.702 −28.366 1.00 0.00 C
ATOM 3318 C GLY A 212 12.660 −21.996 −27.816 1.00 0.00 C
ATOM 3319 O GLY A 212 13.880 −22.133 −27.699 1.00 0.00 O
ATOM 3320 H GLY A 212 13.903 −20.379 −29.383 1.00 0.00 H
ATOM 3321 HA2 GLY A 212 11.697 −20.105 −27.533 1.00 0.00 H
ATOM 3322 HA3 GLY A 212 11.225 −20.953 −29.009 1.00 0.00 H
ATOM 3323 N LEU A 213 11.789 −22.969 −27.559 1.00 0.00 N
ATOM 3324 CA LEU A 213 12.137 −24.318 −27.114 1.00 0.00 C
ATOM 3325 C LEU A 213 11.505 −25.368 −28.033 1.00 0.00 C
ATOM 3326 O LEU A 213 10.344 −25.238 −28.416 1.00 0.00 O
ATOM 3327 CB LEU A 213 11.678 −24.547 −25.660 1.00 0.00 C
ATOM 3328 CG LEU A 213 12.202 −23.538 −24.625 1.00 0.00 C
ATOM 3329 CD1 LEU A 213 11.518 −23.746 −23.275 1.00 0.00 C
ATOM 3330 CD2 LEU A 213 13.712 −23.665 −24.418 1.00 0.00 C
ATOM 3331 H LEU A 213 10.806 −22.779 −27.742 1.00 0.00 H
ATOM 3332 HA LEU A 213 13.218 −24.440 −27.158 1.00 0.00 H
ATOM 3333 HB2 LEU A 213 10.588 −24.518 −25.642 1.00 0.00 H

ATOM 3334 HB3 LEU A 213 11.989 −25.547 −25.360 1.00 0.00 H
ATOM 3335 HG LEU A 213 11.971 −22.530 −24.959 1.00 0.00 H
ATOM 3336 HD11 LEU A 213 11.852 −22.985 −22.572 1.00 0.00 H
ATOM 3337 HD12 LEU A 213 11.757 −24.728 −22.872 1.00 0.00 H
ATOM 3338 HD13 LEU A 213 10.435 −23.658 −23.387 1.00 0.00 H
ATOM 3339 HD21 LEU A 213 14.231 −23.387 −25.332 1.00 0.00 H
ATOM 3340 HD22 LEU A 213 13.969 −24.692 −24.157 1.00 0.00 H
ATOM 3341 HD23 LEU A 213 14.029 −23.001 −23.616 1.00 0.00 H
ATOM 3342 N LEU A 214 12.201 −26.486 −28.235 1.00 0.00 N
ATOM 3343 CA LEU A 214 11.608 −27.748 −28.674 1.00 0.00 C
ATOM 3344 C LEU A 214 11.495 −28.692 −27.470 1.00 0.00 C
ATOM 3345 O LEU A 214 12.492 −29.215 −26.969 1.00 0.00 O
ATOM 3346 CB LEU A 214 12.415 −28.337 −29.844 1.00 0.00 C
ATOM 3347 CG LEU A 214 12.166 −27.599 −31.172 1.00 0.00 C
ATOM 3348 CD1 LEU A 214 13.267 −27.907 −32.187 1.00 0.00 C
ATOM 3349 CD2 LEU A 214 10.832 −27.982 −31.814 1.00 0.00 C
ATOM 3350 H LEU A 214 13.141 −26.525 −27.851 1.00 0.00 H
ATOM 3351 HA LEU A 214 10.595 −27.567 −29.029 1.00 0.00 H
ATOM 3352 HB2 LEU A 214 12.132 −29.377 −29.972 1.00 0.00 H
ATOM 3353 HB3 LEU A 214 13.476 −28.301 −29.593 1.00 0.00 H
ATOM 3354 HG LEU A 214 12.156 −26.528 −30.990 1.00 0.00 H
ATOM 3355 HD11 LEU A 214 13.274 −28.970 −32.419 1.00 0.00 H
ATOM 3356 HD12 LEU A 214 13.088 −27.340 −33.100 1.00 0.00 H
ATOM 3357 HD13 LEU A 214 14.235 −27.618 −31.776 1.00 0.00 H
ATOM 3358 HD21 LEU A 214 10.012 −27.833 −31.113 1.00 0.00 H
ATOM 3359 HD22 LEU A 214 10.852 −29.023 −32.129 1.00 0.00 H
ATOM 3360 HD23 LEU A 214 10.662 −27.352 −32.684 1.00 0.00 H
ATOM 3361 N LYS A 215 10.266 −28.811 −26.967 1.00 0.00 N
ATOM 3362 CA LYS A 215 9.820 −29.747 −25.930 1.00 0.00 C
ATOM 3363 C LYS A 215 9.801 −31.174 −26.493 1.00 0.00 C

ATOM	3364	O	LYS	A	215	9.338	−31.381	−27.615	1.00	0.00	O
ATOM	3365	CB	LYS	A	215	8.419	−29.274	−25.499	1.00	0.00	C
ATOM	3366	CG	LYS	A	215	7.795	−30.022	−24.315	1.00	0.00	C
ATOM	3367	CD	LYS	A	215	6.318	−29.619	−24.171	1.00	0.00	C
ATOM	3368	CE	LYS	A	215	5.649	−30.297	−22.970	1.00	0.00	C
ATOM	3369	NZ	LYS	A	215	5.777	−29.485	−21.736	1.00	0.00	N
ATOM	3370	H	LYS	A	215	9.527	−28.344	−27.486	1.00	0.00	H
ATOM	3371	HA	LYS	A	215	10.500	−29.701	−25.078	1.00	0.00	H
ATOM	3372	HB2	LYS	A	215	8.469	−28.215	−25.238	1.00	0.00	H
ATOM	3373	HB3	LYS	A	215	7.754	−29.368	−26.356	1.00	0.00	H
ATOM	3374	HG2	LYS	A	215	7.851	−31.098	−24.481	1.00	0.00	H
ATOM	3375	HG3	LYS	A	215	8.341	−29.765	−23.409	1.00	0.00	H
ATOM	3376	HD2	LYS	A	215	6.227	−28.533	−24.085	1.00	0.00	H
ATOM	3377	HD3	LYS	A	215	5.788	−29.930	−25.072	1.00	0.00	H
ATOM	3378	HE2	LYS	A	215	6.085	−31.290	−22.828	1.00	0.00	H
ATOM	3379	HE3	LYS	A	215	4.587	−30.421	−23.203	1.00	0.00	H
ATOM	3380	HZ1	LYS	A	215	5.371	−29.936	−20.933	1.00	0.00	H
ATOM	3381	HZ2	LYS	A	215	6.738	−29.237	−21.523	1.00	0.00	H
ATOM	3382	HZ3	LYS	A	215	5.300	−28.584	−21.849	1.00	0.00	H
ATOM	3383	N	TYR	A	216	10.211	−32.163	−25.703	1.00	0.00	N
ATOM	3384	CA	TYR	A	216	10.307	−33.574	−26.106	1.00	0.00	C
ATOM	3385	C	TYR	A	216	10.047	−34.480	−24.896	1.00	0.00	C
ATOM	3386	O	TYR	A	216	10.918	−34.632	−24.041	1.00	0.00	O
ATOM	3387	CB	TYR	A	216	11.706	−33.807	−26.698	1.00	0.00	C
ATOM	3388	CG	TYR	A	216	11.950	−35.166	−27.331	1.00	0.00	C
ATOM	3389	CD1	TYR	A	216	11.496	−35.426	−28.639	1.00	0.00	C
ATOM	3390	CD2	TYR	A	216	12.696	−36.143	−26.644	1.00	0.00	C
ATOM	3391	CE1	TYR	A	216	11.835	−36.632	−29.285	1.00	0.00	C
ATOM	3392	CE2	TYR	A	216	13.027	−37.356	−27.279	1.00	0.00	C
ATOM	3393	CZ	TYR	A	216	12.609	−37.596	−28.606	1.00	0.00	C

ATOM 3394 OH TYR A 216 12.942 −38.761 −29.220 1.00 0.00 O
ATOM 3395 H TYR A 216 10.663 −31.897 −24.833 1.00 0.00 H
ATOM 3396 HA TYR A 216 9.565 −33.794 −26.877 1.00 0.00 H
ATOM 3397 HB2 TYR A 216 11.902 −33.045 −27.449 1.00 0.00 H
ATOM 3398 HB3 TYR A 216 12.448 −33.645 −25.918 1.00 0.00 H
ATOM 3399 HD1 TYR A 216 10.881 −34.700 −29.150 1.00 0.00 H
ATOM 3400 HD2 TYR A 216 13.019 −35.959 −25.627 1.00 0.00 H
ATOM 3401 HE1 TYR A 216 11.491 −36.834 −30.288 1.00 0.00 H
ATOM 3402 HE2 TYR A 216 13.614 −38.094 −26.756 1.00 0.00 H
ATOM 3403 HH TYR A 216 13.212 −39.420 −28.580 1.00 0.00 H
ATOM 3404 N ASN A 217 8.797 −34.913 −24.708 1.00 0.00 N
ATOM 3405 CA ASN A 217 8.398 −35.717 −23.546 1.00 0.00 C
ATOM 3406 C ASN A 217 8.552 −37.221 −23.814 1.00 0.00 C
ATOM 3407 O ASN A 217 7.847 −37.780 −24.657 1.00 0.00 O
ATOM 3408 CB ASN A 217 6.977 −35.331 −23.115 1.00 0.00 C
ATOM 3409 CG ASN A 217 6.643 −35.996 −21.793 1.00 0.00 C
ATOM 3410 OD1 ASN A 217 7.339 −35.828 −20.808 1.00 0.00 O
ATOM 3411 ND2 ASN A 217 5.697 −36.901 −21.776 1.00 0.00 N
ATOM 3412 H ASN A 217 8.115 −34.752 −25.432 1.00 0.00 H
ATOM 3413 HA ASN A 217 9.056 −35.486 −22.711 1.00 0.00 H
ATOM 3414 HB2 ASN A 217 6.912 −34.252 −22.971 1.00 0.00 H
ATOM 3415 HB3 ASN A 217 6.260 −35.630 −23.878 1.00 0.00 H
ATOM 3416 HD21 ASN A 217 5.228 −37.169 −22.616 1.00 0.00 H
ATOM 3417 HD22 ASN A 217 5.710 −37.505 −20.954 1.00 0.00 H
ATOM 3418 N LEU A 218 9.434 −37.880 −23.063 1.00 0.00 N
ATOM 3419 CA LEU A 218 9.832 −39.273 −23.281 1.00 0.00 C
ATOM 3420 C LEU A 218 8.716 −40.293 −22.977 1.00 0.00 C
ATOM 3421 O LEU A 218 8.602 −41.267 −23.723 1.00 0.00 O
ATOM 3422 CB LEU A 218 11.102 −39.547 −22.456 1.00 0.00 C
ATOM 3423 CG LEU A 218 12.344 −38.735 −22.875 1.00 0.00 C

ATOM 3424 CD1 LEU A 218 13.437 −38.838 −21.816 1.00 0.00 C
ATOM 3425 CD2 LEU A 218 12.925 −39.242 −24.199 1.00 0.00 C
ATOM 3426 H LEU A 218 9.926 −37.370 −22.331 1.00 0.00 H
ATOM 3427 HA LEU A 218 10.072 −39.399 −24.335 1.00 0.00 H
ATOM 3428 HB2 LEU A 218 11.339 −40.610 −22.494 1.00 0.00 H
ATOM 3429 HB3 LEU A 218 10.878 −39.301 −21.424 1.00 0.00 H
ATOM 3430 HG LEU A 218 12.085 −37.682 −22.993 1.00 0.00 H
ATOM 3431 HD21 LEU A 218 12.190 −39.122 −24.992 1.00 0.00 H
ATOM 3432 HD22 LEU A 218 13.816 −38.665 −24.440 1.00 0.00 H
ATOM 3433 HD23 LEU A 218 13.191 −40.294 −24.099 1.00 0.00 H
ATOM 3434 HD11 LEU A 218 14.313 −38.263 −22.113 1.00 0.00 H
ATOM 3435 HD12 LEU A 218 13.717 −39.879 −21.662 1.00 0.00 H
ATOM 3436 HD13 LEU A 218 13.064 −38.438 −20.871 1.00 0.00 H
ATOM 3437 N SER A 219 7.728 −39.936 −22.142 1.00 0.00 N
ATOM 3438 CA SER A 219 6.552 −40.782 −21.839 1.00 0.00 C
ATOM 3439 C SER A 219 5.671 −41.085 −23.058 1.00 0.00 C
ATOM 3440 O SER A 219 4.808 −41.958 −23.002 1.00 0.00 O
ATOM 3441 CB SER A 219 5.637 −40.178 −20.752 1.00 0.00 C
ATOM 3442 OG SER A 219 6.194 −39.092 −20.043 1.00 0.00 O
ATOM 3443 H SER A 219 7.941 −39.201 −21.477 1.00 0.00 H
ATOM 3444 HA SER A 219 6.925 −41.734 −21.462 1.00 0.00 H
ATOM 3445 HB2 SER A 219 5.363 −40.963 −20.044 1.00 0.00 H
ATOM 3446 HB3 SER A 219 4.719 −39.814 −21.215 1.00 0.00 H
ATOM 3447 HG SER A 219 7.087 −39.380 −19.692 1.00 0.00 H
ATOM 3448 N LEU A 220 5.814 −40.321 −24.148 1.00 0.00 N
ATOM 3449 CA LEU A 220 5.107 −40.551 −25.407 1.00 0.00 C
ATOM 3450 C LEU A 220 5.605 −41.847 −26.065 1.00 0.00 C
ATOM 3451 O LEU A 220 5.358 −42.083 −27.251 1.00 0.00 O
ATOM 3452 CB LEU A 220 5.281 −39.321 −26.318 1.00 0.00 C
ATOM 3453 CG LEU A 220 4.695 −38.007 −25.766 1.00 0.00 C

ATOM	3454	CD1	LEU	A	220	5.059	−36.857	−26.705	1.00	0.00	C
ATOM	3455	CD2	LEU	A	220	3.170	−38.064	−25.653	1.00	0.00	C
ATOM	3456	H	LEU	A	220	6.579	−39.655	−24.143	1.00	0.00	H
ATOM	3457	HA	LEU	A	220	4.048	−40.699	−25.193	1.00	0.00	H
ATOM	3458	HB2	LEU	A	220	6.347	−39.181	−26.481	1.00	0.00	H
ATOM	3459	HB3	LEU	A	220	4.818	−39.526	−27.284	1.00	0.00	H
ATOM	3460	HG	LEU	A	220	5.110	−37.793	−24.781	1.00	0.00	H
ATOM	3461	HD11	LEU	A	220	4.649	−35.922	−26.324	1.00	0.00	H
ATOM	3462	HD12	LEU	A	220	4.655	−37.047	−27.698	1.00	0.00	H
ATOM	3463	HD13	LEU	A	220	6.143	−36.760	−26.761	1.00	0.00	H
ATOM	3464	HD21	LEU	A	220	2.879	−38.806	−24.911	1.00	0.00	H
ATOM	3465	HD22	LEU	A	220	2.789	−37.094	−25.333	1.00	0.00	H
ATOM	3466	HD23	LEU	A	220	2.732	−38.321	−26.617	1.00	0.00	H
ATOM	3467	H	LEU	A	220	6.181	−42.563	−25.480	1.00	0.00	H
ATOM	3468	N	ILE	A	221	9.238	−40.378	−30.930	1.00	0.00	N
ATOM	3469	CA	ILE	A	221	8.601	−39.460	−29.982	1.00	0.00	C
ATOM	3470	C	ILE	A	221	8.507	−38.058	−30.614	1.00	0.00	C
ATOM	3471	O	ILE	A	221	9.183	−37.754	−31.600	1.00	0.00	O
ATOM	3472	CB	ILE	A	221	9.332	−39.510	−28.611	1.00	0.00	C
ATOM	3473	CG1	ILE	A	221	8.897	−40.727	−27.757	1.00	0.00	C
ATOM	3474	CG2	ILE	A	221	9.106	−38.265	−27.731	1.00	0.00	C
ATOM	3475	CD1	ILE	A	221	9.225	−42.107	−28.341	1.00	0.00	C
ATOM	3476	HA	ILE	A	221	7.578	−39.802	−29.821	1.00	0.00	H
ATOM	3477	HB	ILE	A	221	10.404	−39.583	−28.794	1.00	0.00	H
ATOM	3478	HG12	ILE	A	221	9.390	−40.667	−26.785	1.00	0.00	H
ATOM	3479	HG13	ILE	A	221	7.822	−40.677	−27.586	1.00	0.00	H
ATOM	3480	HG21	ILE	A	221	9.468	−37.365	−28.224	1.00	0.00	H
ATOM	3481	HG22	ILE	A	221	9.656	−38.365	−26.794	1.00	0.00	H
ATOM	3482	HG23	ILE	A	221	8.051	−38.142	−27.500	1.00	0.00	H
ATOM	3483	HD11	ILE	A	221	9.016	−42.871	−27.591	1.00	0.00	H

ATOM 3484 HD12 ILE A 221 8.602 −42.307 −29.212 1.00 0.00 H
ATOM 3485 HD13 ILE A 221 10.278 −42.155 −28.618 1.00 0.00 H
ATOM 3486 H ILE A 221 9.789 −39.946 −31.664 1.00 0.00 H
ATOM 3487 H ILE A 221 8.507 −40.925 −31.372 1.00 0.00 H
ATOM 3488 N ILE A 222 7.499 −37.295 −30.186 1.00 0.00 N
ATOM 3489 CA ILE A 222 7.091 −36.014 −30.780 1.00 0.00 C
ATOM 3490 C ILE A 222 7.971 −34.871 −30.258 1.00 0.00 C
ATOM 3491 O ILE A 222 8.118 −34.718 −29.045 1.00 0.00 O
ATOM 3492 CB ILE A 222 5.590 −35.754 −30.493 1.00 0.00 C
ATOM 3493 CG1 ILE A 222 4.731 −36.970 −30.920 1.00 0.00 C
ATOM 3494 CG2 ILE A 222 5.124 −34.465 −31.186 1.00 0.00 C
ATOM 3495 CD1 ILE A 222 3.213 −36.800 −30.784 1.00 0.00 C
ATOM 3496 H ILE A 222 7.020 −37.586 −29.349 1.00 0.00 H
ATOM 3497 HA ILE A 222 7.225 −36.075 −31.862 1.00 0.00 H
ATOM 3498 HB ILE A 222 5.463 −35.611 −29.420 1.00 0.00 H
ATOM 3499 HG12 ILE A 222 4.963 −37.211 −31.953 1.00 0.00 H
ATOM 3500 HG13 ILE A 222 5.002 −37.830 −30.307 1.00 0.00 H
ATOM 3501 HD11 ILE A 222 2.725 −37.760 −30.948 1.00 0.00 H
ATOM 3502 HD12 ILE A 222 2.965 −36.438 −29.786 1.00 0.00 H
ATOM 3503 HD13 ILE A 222 2.844 −36.096 −31.530 1.00 0.00 H
ATOM 3504 HG21 ILE A 222 5.672 −33.608 −30.803 1.00 0.00 H
ATOM 3505 HG22 ILE A 222 4.077 −34.276 −30.958 1.00 0.00 H
ATOM 3506 HG23 ILE A 222 5.256 −34.528 −32.265 1.00 0.00 H
ATOM 3507 N LEU A 223 8.415 −33.980 −31.152 1.00 0.00 N
ATOM 3508 CA LEU A 223 8.852 −32.629 −30.778 1.00 0.00 C
ATOM 3509 C LEU A 223 7.671 −31.651 −30.800 1.00 0.00 C
ATOM 3510 O LEU A 223 6.949 −31.593 −31.793 1.00 0.00 O
ATOM 3511 CB LEU A 223 9.948 −32.132 −31.734 1.00 0.00 C
ATOM 3512 CG LEU A 223 11.311 −32.821 −31.586 1.00 0.00 C
ATOM 3513 CD1 LEU A 223 12.212 −32.365 −32.730 1.00 0.00 C

ATOM 3514 CD2 LEU A 223 12.032 −32.444 −30.292 1.00 0.00 C
ATOM 3515 H LEU A 223 8.192 −34.144 −32.128 1.00 0.00 H
ATOM 3516 HA LEU A 223 9.242 −32.647 −29.762 1.00 0.00 H
ATOM 3517 HB2 LEU A 223 10.087 −31.064 −31.568 1.00 0.00 H
ATOM 3518 HB3 LEU A 223 9.593 −32.270 −32.755 1.00 0.00 H
ATOM 3519 HG LEU A 223 11.188 −33.903 −31.633 1.00 0.00 H
ATOM 3520 HD11 LEU A 223 12.268 −31.278 −32.755 1.00 0.00 H
ATOM 3521 HD12 LEU A 223 13.209 −32.767 −32.573 1.00 0.00 H
ATOM 3522 HD13 LEU A 223 11.818 −32.727 −33.678 1.00 0.00 H
ATOM 3523 HD21 LEU A 223 12.466 −31.453 −30.370 1.00 0.00 H
ATOM 3524 HD22 LEU A 223 11.339 −32.449 −29.457 1.00 0.00 H
ATOM 3525 HD23 LEU A 223 12.832 −33.160 −30.099 1.00 0.00 H
ATOM 3526 N GLN A 224 7.596 −30.752 −29.820 1.00 0.00 N
ATOM 3527 CA GLN A 224 6.581 −29.692 −29.748 1.00 0.00 C
ATOM 3528 C GLN A 224 7.254 −28.324 −29.547 1.00 0.00 C
ATOM 3529 O GLN A 224 8.116 −28.184 −28.680 1.00 0.00 O
ATOM 3530 CB GLN A 224 5.561 −30.008 −28.638 1.00 0.00 C
ATOM 3531 CG GLN A 224 4.902 −31.390 −28.796 1.00 0.00 C
ATOM 3532 CD GLN A 224 3.775 −31.615 −27.796 1.00 0.00 C
ATOM 3533 OE1 GLN A 224 3.917 −31.395 −26.601 1.00 0.00 O
ATOM 3534 NE2 GLN A 224 2.643 −32.127 −28.223 1.00 0.00 N
ATOM 3535 H GLN A 224 8.243 −30.836 −29.041 1.00 0.00 H
ATOM 3536 HA GLN A 224 6.032 −29.657 −30.686 1.00 0.00 H
ATOM 3537 HB2 GLN A 224 4.782 −29.244 −28.660 1.00 0.00 H
ATOM 3538 HB3 GLN A 224 6.052 −29.965 −27.667 1.00 0.00 H
ATOM 3539 HG2 GLN A 224 4.504 −31.483 −29.803 1.00 0.00 H
ATOM 3540 HG3 GLN A 224 5.641 −32.178 −28.650 1.00 0.00 H
ATOM 3541 HE21 GLN A 224 2.484 −32.304 −29.226 1.00 0.00 H
ATOM 3542 HE22 GLN A 224 1.918 −32.267 −27.551 1.00 0.00 H
ATOM 3543 N PHE A 225 6.965 −27.338 −30.401 1.00 0.00 N

ATOM 3544 CA PHE A 225 7.614 −26.020 −30.343 1.00 0.00 C
ATOM 3545 C PHE A 225 6.866 −25.024 −29.446 1.00 0.00 C
ATOM 3546 O PHE A 225 5.677 −24.774 −29.647 1.00 0.00 O
ATOM 3547 CB PHE A 225 7.821 −25.455 −31.751 1.00 0.00 C
ATOM 3548 CG PHE A 225 8.544 −24.116 −31.779 1.00 0.00 C
ATOM 3549 CD1 PHE A 225 9.917 −24.045 −31.475 1.00 0.00 C
ATOM 3550 CD2 PHE A 225 7.844 −22.933 −32.088 1.00 0.00 C
ATOM 3551 CE1 PHE A 225 10.584 −22.806 −31.473 1.00 0.00 C
ATOM 3552 CE2 PHE A 225 8.512 −21.694 −32.093 1.00 0.00 C
ATOM 3553 CZ PHE A 225 9.881 −21.629 −31.782 1.00 0.00 C
ATOM 3554 H PHE A 225 6.244 −27.497 −31.099 1.00 0.00 H
ATOM 3555 HA PHE A 225 8.607 −26.151 −29.916 1.00 0.00 H
ATOM 3556 HB2 PHE A 225 6.849 −25.344 −32.229 1.00 0.00 H
ATOM 3557 HB3 PHE A 225 8.398 −26.175 −32.331 1.00 0.00 H
ATOM 3558 HD1 PHE A 225 10.458 −24.943 −31.217 1.00 0.00 H
ATOM 3559 HD2 PHE A 225 6.787 −22.970 −32.308 1.00 0.00 H
ATOM 3560 HE1 PHE A 225 11.634 −22.756 −31.222 1.00 0.00 H
ATOM 3561 HE2 PHE A 225 7.968 −20.790 −32.325 1.00 0.00 H
ATOM 3562 HZ PHE A 225 10.390 −20.675 −31.772 1.00 0.00 H
ATOM 3563 N ILE A 226 7.625 −24.304 −28.618 1.00 0.00 N
ATOM 3564 CA ILE A 226 7.180 −23.154 −27.828 1.00 0.00 C
ATOM 3565 C ILE A 226 7.976 −21.914 −28.254 1.00 0.00 C
ATOM 3566 O ILE A 226 9.201 −21.909 −28.168 1.00 0.00 O
ATOM 3567 CB ILE A 226 7.353 −23.469 −26.324 1.00 0.00 C
ATOM 3568 CG1 ILE A 226 6.435 −24.610 −25.818 1.00 0.00 C
ATOM 3569 CG2 ILE A 226 7.161 −22.206 −25.477 1.00 0.00 C
ATOM 3570 CD1 ILE A 226 4.926 −24.325 −25.867 1.00 0.00 C
ATOM 3571 H ILE A 226 8.612 −24.539 −28.562 1.00 0.00 H
ATOM 3572 HA ILE A 226 6.127 −22.956 −28.024 1.00 0.00 H
ATOM 3573 HB ILE A 226 8.382 −23.801 −26.171 1.00 0.00 H

ATOM 3574 HG12 ILE A 226 6.634 −25.513 −26.396 1.00 0.00 H
ATOM 3575 HG13 ILE A 226 6.700 −24.830 −24.782 1.00 0.00 H
ATOM 3576 HG21 ILE A 226 6.226 −21.710 −25.735 1.00 0.00 H
ATOM 3577 HG22 ILE A 226 7.161 −22.483 −24.425 1.00 0.00 H
ATOM 3578 HG23 ILE A 226 7.990 −21.516 −25.638 1.00 0.00 H
ATOM 3579 HD11 ILE A 226 4.386 −25.191 −25.486 1.00 0.00 H
ATOM 3580 HD12 ILE A 226 4.608 −24.141 −26.893 1.00 0.00 H
ATOM 3581 HD13 ILE A 226 4.680 −23.463 −25.247 1.00 0.00 H
ATOM 3582 N GLU A 227 7.288 −20.835 −28.626 1.00 0.00 N
ATOM 3583 CA GLU A 227 7.908 −19.535 −28.907 1.00 0.00 C
ATOM 3584 C GLU A 227 8.277 −18.760 −27.628 1.00 0.00 C
ATOM 3585 O GLU A 227 7.578 −18.840 −26.617 1.00 0.00 O
ATOM 3586 CB GLU A 227 6.991 −18.713 −29.825 1.00 0.00 C
ATOM 3587 CG GLU A 227 5.584 −18.406 −29.270 1.00 0.00 C
ATOM 3588 CD GLU A 227 4.750 −17.576 −30.263 1.00 0.00 C
ATOM 3589 OE1 GLU A 227 4.931 −17.794 −31.481 1.00 0.00 O
ATOM 3590 OE2 GLU A 227 4.396 −16.433 −29.902 1.00 0.00 O
ATOM 3591 H GLU A 227 6.282 −20.874 −28.594 1.00 0.00 H
ATOM 3592 HA GLU A 227 8.835 −19.710 −29.456 1.00 0.00 H
ATOM 3593 HB2 GLU A 227 7.484 −17.766 −30.045 1.00 0.00 H
ATOM 3594 HB3 GLU A 227 6.886 −19.263 −30.760 1.00 0.00 H
ATOM 3595 HG2 GLU A 227 5.060 −19.342 −29.061 1.00 0.00 H
ATOM 3596 HG3 GLU A 227 5.681 −17.858 −28.330 1.00 0.00 H
ATOM 3597 N ILE A 228 9.330 −17.936 −27.700 1.00 0.00 N
ATOM 3598 CA ILE A 228 9.667 −16.948 −26.661 1.00 0.00 C
ATOM 3599 C ILE A 228 8.743 −15.723 −26.719 1.00 0.00 C
ATOM 3600 O ILE A 228 8.392 −15.267 −27.809 1.00 0.00 O
ATOM 3601 CB ILE A 228 11.162 −16.557 −26.753 1.00 0.00 C
ATOM 3602 CG1 ILE A 228 11.657 −15.952 −25.422 1.00 0.00 C
ATOM 3603 CG2 ILE A 228 11.480 −15.590 −27.913 1.00 0.00 C

```
ATOM   3604  CD1 ILE A 228      13.176 −16.067 −25.260  1.00  0.00           C
ATOM   3605  H   ILE A 228       9.904 −17.948 −28.535  1.00  0.00           H
ATOM   3606  HA  ILE A 228       9.504 −17.430 −25.697  1.00  0.00           H
ATOM   3607  HB  ILE A 228      11.717 −17.482 −26.922  1.00  0.00           H
ATOM   3608 HG12 ILE A 228      11.200 −16.487 −24.591  1.00  0.00           H
ATOM   3609 HG13 ILE A 228      11.361 −14.905 −25.346  1.00  0.00           H
ATOM   3610 HG21 ILE A 228      11.091 −14.594 −27.697  1.00  0.00           H
ATOM   3611 HG22 ILE A 228      12.557 −15.524 −28.056  1.00  0.00           H
ATOM   3612 HG23 ILE A 228      11.025 −15.946 −28.835  1.00  0.00           H
ATOM   3613 HD11 ILE A 228      13.481 −17.105 −25.380  1.00  0.00           H
ATOM   3614 HD12 ILE A 228      13.688 −15.457 −26.004  1.00  0.00           H
ATOM   3615 HD13 ILE A 228      13.458 −15.730 −24.262  1.00  0.00           H
ATOM   3616  N   ALA A 229       8.451 −15.112 −25.569  1.00  0.00           N
ATOM   3617  CA  ALA A 229       7.614 −13.914 −25.441  1.00  0.00           C
ATOM   3618  C   ALA A 229       8.048 −12.710 −26.315  1.00  0.00           C
ATOM   3619  O   ALA A 229       9.199 −12.581 −26.734  1.00  0.00           O
ATOM   3620  CB  ALA A 229       7.552 −13.532 −23.955  1.00  0.00           C
ATOM   3621  H   ALA A 229       8.679 −15.614 −24.717  1.00  0.00           H
ATOM   3622  HA  ALA A 229       6.605 −14.188 −25.754  1.00  0.00           H
ATOM   3623  HB1 ALA A 229       6.865 −12.696 −23.817  1.00  0.00           H
ATOM   3624  HB2 ALA A 229       8.544 −13.245 −23.605  1.00  0.00           H
ATOM   3625  HB3 ALA A 229       7.192 −14.381 −23.373  1.00  0.00           H
ATOM   3626  N   ASP A 230       7.120 −11.771 −26.517  1.00  0.00           N
ATOM   3627  CA  ASP A 230       7.316 −10.492 −27.218  1.00  0.00           C
ATOM   3628  C   ASP A 230       8.240  −9.513 −26.462  1.00  0.00           C
ATOM   3629  O   ASP A 230       8.782  −8.571 −27.045  1.00  0.00           O
ATOM   3630  CB  ASP A 230       5.942  −9.817 −27.387  1.00  0.00           C
ATOM   3631  CG  ASP A 230       5.325  −9.431 −26.033  1.00  0.00           C
ATOM   3632  OD1 ASP A 230       5.072 −10.388 −25.267  1.00  0.00           O
ATOM   3633  OD2 ASP A 230       5.645  −8.305 −25.587  1.00  0.00           O
```

ATOM	3634	H	ASP	A	230	6.227	−11.859	−26.036	1.00	0.00	H
ATOM	3635	HA	ASP	A	230	7.739	−10.686	−28.202	1.00	0.00	H
ATOM	3636	HB2	ASP	A	230	5.267	−10.496	−27.912	1.00	0.00	H
ATOM	3637	HB3	ASP	A	230	6.057	−8.923	−28.003	1.00	0.00	H
ATOM	3638	N	ASN	A	231	8.360	−9.683	−25.142	1.00	0.00	N
ATOM	3639	CA	ASN	A	231	9.047	−8.740	−24.267	1.00	0.00	C
ATOM	3640	C	ASN	A	231	10.544	−8.696	−24.639	1.00	0.00	C
ATOM	3641	O	ASN	A	231	11.232	−9.709	−24.520	1.00	0.00	O
ATOM	3642	CB	ASN	A	231	8.842	−9.156	−22.794	1.00	0.00	C
ATOM	3643	CG	ASN	A	231	7.414	−9.377	−22.302	1.00	0.00	C
ATOM	3644	OD1	ASN	A	231	7.223	−9.968	−21.250	1.00	0.00	O
ATOM	3645	ND2	ASN	A	231	6.375	−8.931	−22.964	1.00	0.00	N
ATOM	3646	H	ASN	A	231	7.796	−10.411	−24.730	1.00	0.00	H
ATOM	3647	HA	ASN	A	231	8.612	−7.751	−24.418	1.00	0.00	H
ATOM	3648	HB2	ASN	A	231	9.383	−10.086	−22.616	1.00	0.00	H
ATOM	3649	HB3	ASN	A	231	9.282	−8.389	−22.157	1.00	0.00	H
ATOM	3650	HD21	ASN	A	231	6.424	−8.600	−23.933	1.00	0.00	H
ATOM	3651	HD22	ASN	A	231	5.475	−9.282	−22.691	1.00	0.00	H
ATOM	3652	N	ARG	A	232	11.042	−7.553	−25.144	1.00	0.00	N
ATOM	3653	CA	ARG	A	232	12.352	−7.442	−25.837	1.00	0.00	C
ATOM	3654	C	ARG	A	232	12.522	−8.508	−26.936	1.00	0.00	C
ATOM	3655	O	ARG	A	232	13.543	−9.192	−27.007	1.00	0.00	O
ATOM	3656	CB	ARG	A	232	13.546	−7.390	−24.850	1.00	0.00	C
ATOM	3657	CG	ARG	A	232	13.652	−6.054	−24.097	1.00	0.00	C
ATOM	3658	CD	ARG	A	232	14.786	−6.038	−23.058	1.00	0.00	C
ATOM	3659	NE	ARG	A	232	16.146	−6.118	−23.637	1.00	0.00	N
ATOM	3660	CZ	ARG	A	232	17.259	−6.313	−22.953	1.00	0.00	C
ATOM	3661	NH1	ARG	A	232	18.393	−6.455	−23.566	1.00	0.00	N
ATOM	3662	NH2	ARG	A	232	17.264	−6.371	−21.650	1.00	0.00	N
ATOM	3663	H	ARG	A	232	10.377	−6.805	−25.281	1.00	0.00	H

ATOM 3664 HA ARG A 232 12.359 −6.502 −26.389 1.00 0.00 H
ATOM 3665 HB2 ARG A 232 14.476 −7.514 −25.408 1.00 0.00 H
ATOM 3666 HB3 ARG A 232 13.469 −8.211 −24.138 1.00 0.00 H
ATOM 3667 HG2 ARG A 232 13.809 −5.245 −24.812 1.00 0.00 H
ATOM 3668 HG3 ARG A 232 12.713 −5.873 −23.573 1.00 0.00 H
ATOM 3669 HD2 ARG A 232 14.639 −6.878 −22.375 1.00 0.00 H
ATOM 3670 HD3 ARG A 232 14.707 −5.115 −22.480 1.00 0.00 H
ATOM 3671 HE ARG A 232 16.274 −6.116 −24.653 1.00 0.00 H
ATOM 3672 HH11 ARG A 232 18.430 −6.224 −24.572 1.00 0.00 H
ATOM 3673 HH12 ARG A 232 19.255 −6.499 −23.067 1.00 0.00 H
ATOM 3674 HH21 ARG A 232 16.464 −6.020 −21.136 1.00 0.00 H
ATOM 3675 HH22 ARG A 232 18.084 −6.677 −21.145 1.00 0.00 H
ATOM 3676 N LEU A 233 11.526 −8.657 −27.811 1.00 0.00 N
ATOM 3677 CA LEU A 233 11.640 −9.473 −29.019 1.00 0.00 C
ATOM 3678 C LEU A 233 12.813 −8.994 −29.889 1.00 0.00 C
ATOM 3679 O LEU A 233 12.936 −7.812 −30.199 1.00 0.00 O
ATOM 3680 CB LEU A 233 10.307 −9.446 −29.792 1.00 0.00 C
ATOM 3681 CG LEU A 233 10.280 −10.286 −31.081 1.00 0.00 C
ATOM 3682 CD1 LEU A 233 10.450 −11.781 −30.806 1.00 0.00 C
ATOM 3683 CD2 LEU A 233 8.944 −10.092 −31.801 1.00 0.00 C
ATOM 3684 H LEU A 233 10.642 −8.197 −27.629 1.00 0.00 H
ATOM 3685 HA LEU A 233 11.834 −10.499 −28.703 1.00 0.00 H
ATOM 3686 HB2 LEU A 233 9.520 −9.812 −29.137 1.00 0.00 H
ATOM 3687 HB3 LEU A 233 10.080 −8.410 −30.047 1.00 0.00 H
ATOM 3688 HG LEU A 233 11.076 −9.963 −31.748 1.00 0.00 H
ATOM 3689 HD11 LEU A 233 10.381 −12.324 −31.747 1.00 0.00 H
ATOM 3690 HD12 LEU A 233 9.674 −12.125 −30.120 1.00 0.00 H
ATOM 3691 HD13 LEU A 233 11.427 −11.980 −30.372 1.00 0.00 H
ATOM 3692 HD21 LEU A 233 8.804 −9.037 −32.036 1.00 0.00 H
ATOM 3693 HD22 LEU A 233 8.124 −10.427 −31.165 1.00 0.00 H

```
ATOM   3694 HD23 LEU A 233       8.940 −10.665 −32.727  1.00  0.00           H
ATOM   3695  N   THR A 234      13.595  −9.950 −30.380  1.00  0.00           N
ATOM   3696  CA  THR A 234      14.648  −9.774 −31.390  1.00  0.00           C
ATOM   3697  C   THR A 234      14.591 −10.953 −32.357  1.00  0.00           C
ATOM   3698  O   THR A 234      13.956 −11.966 −32.048  1.00  0.00           O
ATOM   3699  CB  THR A 234      16.049  −9.672 −30.755  1.00  0.00           C
ATOM   3700  OG1 THR A 234      16.340 −10.798 −29.952  1.00  0.00           O
ATOM   3701  CG2 THR A 234      16.215  −8.444 −29.867  1.00  0.00           C
ATOM   3702  H   THR A 234      13.367 −10.906 −30.153  1.00  0.00           H
ATOM   3703  HA  THR A 234      14.462  −8.862 −31.958  1.00  0.00           H
ATOM   3704  HB  THR A 234      16.794  −9.612 −31.549  1.00  0.00           H
ATOM   3705  HG1 THR A 234      16.567 −11.540 −30.532  1.00  0.00           H
ATOM   3706 HG21 THR A 234      16.038  −7.539 −30.451  1.00  0.00           H
ATOM   3707 HG22 THR A 234      15.518  −8.465 −29.030  1.00  0.00           H
ATOM   3708 HG23 THR A 234      17.233  −8.423 −29.485  1.00  0.00           H
ATOM   3709  N   ILE A 235      15.323 −10.871 −33.474  1.00  0.00           N
ATOM   3710  CA  ILE A 235      15.451 −11.970 −34.441  1.00  0.00           C
ATOM   3711  C   ILE A 235      15.783 −13.296 −33.752  1.00  0.00           C
ATOM   3712  O   ILE A 235      16.641 −13.347 −32.869  1.00  0.00           O
ATOM   3713  CB  ILE A 235      16.486 −11.627 −35.539  1.00  0.00           C
ATOM   3714  CG1 ILE A 235      16.565 −12.770 −36.578  1.00  0.00           C
ATOM   3715  CG2 ILE A 235      17.888 −11.313 −34.978  1.00  0.00           C
ATOM   3716  CD1 ILE A 235      17.174 −12.369 −37.923  1.00  0.00           C
ATOM   3717  H   ILE A 235      15.847 −10.028 −33.654  1.00  0.00           H
ATOM   3718  HA  ILE A 235      14.480 −12.089 −34.924  1.00  0.00           H
ATOM   3719  HB  ILE A 235      16.127 −10.730 −36.038  1.00  0.00           H
ATOM   3720 HG12 ILE A 235      15.559 −13.135 −36.779  1.00  0.00           H
ATOM   3721 HG13 ILE A 235      17.147 −13.596 −36.169  1.00  0.00           H
ATOM   3722 HG21 ILE A 235      18.510 −10.894 −35.766  1.00  0.00           H
ATOM   3723 HG22 ILE A 235      17.834 −10.575 −34.180  1.00  0.00           H
```

ATOM 3724 HG23 ILE A 235 18.358 −12.217 −34.590 1.00 0.00 H
ATOM 3725 HD11 ILE A 235 18.155 −11.930 −37.781 1.00 0.00 H
ATOM 3726 HD12 ILE A 235 16.539 −11.639 −38.411 1.00 0.00 H
ATOM 3727 HD13 ILE A 235 17.263 −13.247 −38.562 1.00 0.00 H
ATOM 3728 N GLY A 236 15.112 −14.368 −34.178 1.00 0.00 N
ATOM 3729 CA GLY A 236 15.406 −15.726 −33.742 1.00 0.00 C
ATOM 3730 C GLY A 236 16.870 −16.076 −33.984 1.00 0.00 C
ATOM 3731 O GLY A 236 17.353 −16.100 −35.115 1.00 0.00 O
ATOM 3732 H GLY A 236 14.345 −14.232 −34.828 1.00 0.00 H
ATOM 3733 HA2 GLY A 236 14.783 −16.441 −34.274 1.00 0.00 H
ATOM 3734 HA3 GLY A 236 15.188 −15.800 −32.680 1.00 0.00 H
ATOM 3735 N SER A 237 17.580 −16.295 −32.888 1.00 0.00 N
ATOM 3736 CA SER A 237 19.033 −16.396 −32.801 1.00 0.00 C
ATOM 3737 C SER A 237 19.416 −17.476 −31.783 1.00 0.00 C
ATOM 3738 O SER A 237 18.564 −17.853 −30.967 1.00 0.00 O
ATOM 3739 CB SER A 237 19.610 −15.037 −32.419 1.00 0.00 C
ATOM 3740 OG SER A 237 19.157 −14.633 −31.146 1.00 0.00 O
ATOM 3741 H SER A 237 17.090 −16.249 −32.002 1.00 0.00 H
ATOM 3742 HA SER A 237 19.436 −16.655 −33.776 1.00 0.00 H
ATOM 3743 HB2 SER A 237 19.318 −14.298 −33.167 1.00 0.00 H
ATOM 3744 HB3 SER A 237 20.696 −15.110 −32.400 1.00 0.00 H
ATOM 3745 HG SER A 237 19.886 −14.136 −30.719 1.00 0.00 H
ATOM 3746 N PRO A 238 20.635 −18.048 −31.853 1.00 0.00 N
ATOM 3747 CA PRO A 238 20.950 −19.217 −31.049 1.00 0.00 C
ATOM 3748 C PRO A 238 20.926 −18.900 −29.551 1.00 0.00 C
ATOM 3749 O PRO A 238 21.413 −17.858 −29.106 1.00 0.00 O
ATOM 3750 CB PRO A 238 22.297 −19.750 −31.538 1.00 0.00 C
ATOM 3751 CG PRO A 238 22.463 −19.121 −32.921 1.00 0.00 C
ATOM 3752 CD PRO A 238 21.701 −17.800 −32.813 1.00 0.00 C
ATOM 3753 HA PRO A 238 20.189 −19.960 −31.269 1.00 0.00 H

ATOM	3754	HB2	PRO	A	238	22.300	−20.840	−31.592	1.00	0.00	H
ATOM	3755	HB3	PRO	A	238	23.095	−19.408	−30.888	1.00	0.00	H
ATOM	3756	HG2	PRO	A	238	23.513	−18.963	−33.175	1.00	0.00	H
ATOM	3757	HG3	PRO	A	238	21.986	−19.759	−33.664	1.00	0.00	H
ATOM	3758	HD2	PRO	A	238	21.328	−17.518	−33.798	1.00	0.00	H
ATOM	3759	HD3	PRO	A	238	22.364	−17.025	−32.426	1.00	0.00	H
ATOM	3760	N	SER	A	239	20.348	−19.803	−28.769	1.00	0.00	N
ATOM	3761	CA	SER	A	239	19.942	−19.567	−27.378	1.00	0.00	C
ATOM	3762	C	SER	A	239	20.324	−20.732	−26.459	1.00	0.00	C
ATOM	3763	O	SER	A	239	20.978	−21.674	−26.889	1.00	0.00	O
ATOM	3764	CB	SER	A	239	18.436	−19.250	−27.353	1.00	0.00	C
ATOM	3765	OG	SER	A	239	17.650	−20.206	−28.044	1.00	0.00	O
ATOM	3766	H	SER	A	239	19.967	−20.631	−29.220	1.00	0.00	H
ATOM	3767	HA	SER	A	239	20.461	−18.690	−26.992	1.00	0.00	H
ATOM	3768	HB2	SER	A	239	18.088	−19.188	−26.323	1.00	0.00	H
ATOM	3769	HB3	SER	A	239	18.277	−18.276	−27.822	1.00	0.00	H
ATOM	3770	HG	SER	A	239	17.740	−21.064	−27.624	1.00	0.00	H
ATOM	3771	N	LYS	A	240	20.030	−20.626	−25.164	1.00	0.00	N
ATOM	3772	CA	LYS	A	240	20.440	−21.590	−24.133	1.00	0.00	C
ATOM	3773	C	LYS	A	240	19.489	−21.507	−22.945	1.00	0.00	C
ATOM	3774	O	LYS	A	240	19.059	−20.413	−22.584	1.00	0.00	O
ATOM	3775	CB	LYS	A	240	21.907	−21.288	−23.771	1.00	0.00	C
ATOM	3776	CG	LYS	A	240	22.463	−21.918	−22.488	1.00	0.00	C
ATOM	3777	CD	LYS	A	240	23.974	−21.646	−22.418	1.00	0.00	C
ATOM	3778	CE	LYS	A	240	24.557	−21.807	−21.009	1.00	0.00	C
ATOM	3779	NZ	LYS	A	240	26.030	−21.700	−21.040	1.00	0.00	N
ATOM	3780	H	LYS	A	240	19.565	−19.778	−24.849	1.00	0.00	H
ATOM	3781	HA	LYS	A	240	20.393	−22.605	−24.527	1.00	0.00	H
ATOM	3782	HB2	LYS	A	240	22.018	−20.212	−23.652	1.00	0.00	H
ATOM	3783	HB3	LYS	A	240	22.535	−21.600	−24.611	1.00	0.00	H

ATOM 3784 HG2 LYS A 240 22.269 −22.989 −22.470 1.00 0.00 H
ATOM 3785 HG3 LYS A 240 21.980 −21.444 −21.640 1.00 0.00 H
ATOM 3786 HD2 LYS A 240 24.486 −22.318 −23.105 1.00 0.00 H
ATOM 3787 HD3 LYS A 240 24.160 −20.619 −22.730 1.00 0.00 H
ATOM 3788 HE2 LYS A 240 24.265 −22.783 −20.599 1.00 0.00 H
ATOM 3789 HE3 LYS A 240 24.130 −21.027 −20.368 1.00 0.00 H
ATOM 3790 HZ1 LYS A 240 26.447 −22.624 −21.122 1.00 0.00 H
ATOM 3791 HZ2 LYS A 240 26.382 −21.071 −21.756 1.00 0.00 H
ATOM 3792 HZ3 LYS A 240 26.420 −21.388 −20.160 1.00 0.00 H
ATOM 3793 N ILE A 241 19.182 −22.642 −22.323 1.00 0.00 N
ATOM 3794 CA ILE A 241 18.385 −22.719 −21.090 1.00 0.00 C
ATOM 3795 C ILE A 241 19.063 −23.682 −20.113 1.00 0.00 C
ATOM 3796 O ILE A 241 19.596 −24.700 −20.544 1.00 0.00 O
ATOM 3797 CB ILE A 241 16.902 −23.058 −21.381 1.00 0.00 C
ATOM 3798 CG1 ILE A 241 16.067 −22.967 −20.081 1.00 0.00 C
ATOM 3799 CG2 ILE A 241 16.719 −24.417 −22.073 1.00 0.00 C
ATOM 3800 CD1 ILE A 241 14.549 −23.078 −20.279 1.00 0.00 C
ATOM 3801 H ILE A 241 19.635 −23.499 −22.633 1.00 0.00 H
ATOM 3802 HA ILE A 241 18.400 −21.736 −20.625 1.00 0.00 H
ATOM 3803 HB ILE A 241 16.535 −22.304 −22.072 1.00 0.00 H
ATOM 3804 HG12 ILE A 241 16.382 −23.749 −19.390 1.00 0.00 H
ATOM 3805 HG13 ILE A 241 16.264 −22.003 −19.610 1.00 0.00 H
ATOM 3806 HG21 ILE A 241 17.289 −24.439 −22.999 1.00 0.00 H
ATOM 3807 HG22 ILE A 241 15.674 −24.584 −22.326 1.00 0.00 H
ATOM 3808 HG23 ILE A 241 17.053 −25.219 −21.418 1.00 0.00 H
ATOM 3809 HD11 ILE A 241 14.208 −22.338 −21.003 1.00 0.00 H
ATOM 3810 HD12 ILE A 241 14.050 −22.899 −19.328 1.00 0.00 H
ATOM 3811 HD13 ILE A 241 14.279 −24.076 −20.624 1.00 0.00 H
ATOM 3812 N TYR A 242 19.190 −23.295 −18.840 1.00 0.00 N
ATOM 3813 CA TYR A 242 20.089 −23.972 −17.897 1.00 0.00 C

ATOM	3814	C	TYR	A	242	19.654	−23.806	−16.429	1.00	0.00	C
ATOM	3815	O	TYR	A	242	19.313	−22.713	−15.976	1.00	0.00	O
ATOM	3816	CB	TYR	A	242	21.533	−23.505	−18.147	1.00	0.00	C
ATOM	3817	CG	TYR	A	242	21.815	−22.020	−17.985	1.00	0.00	C
ATOM	3818	CD1	TYR	A	242	22.528	−21.574	−16.862	1.00	0.00	C
ATOM	3819	CD2	TYR	A	242	21.433	−21.089	−18.973	1.00	0.00	C
ATOM	3820	CE1	TYR	A	242	22.873	−20.219	−16.732	1.00	0.00	C
ATOM	3821	CE2	TYR	A	242	21.788	−19.730	−18.857	1.00	0.00	C
ATOM	3822	CZ	TYR	A	242	22.519	−19.293	−17.733	1.00	0.00	C
ATOM	3823	OH	TYR	A	242	22.879	−17.990	−17.604	1.00	0.00	O
ATOM	3824	H	TYR	A	242	18.831	−22.380	−18.577	1.00	0.00	H
ATOM	3825	HA	TYR	A	242	20.063	−25.040	−18.120	1.00	0.00	H
ATOM	3826	HB2	TYR	A	242	22.188	−24.065	−17.480	1.00	0.00	H
ATOM	3827	HB3	TYR	A	242	21.816	−23.789	−19.162	1.00	0.00	H
ATOM	3828	HD1	TYR	A	242	22.830	−22.274	−16.096	1.00	0.00	H
ATOM	3829	HD2	TYR	A	242	20.851	−21.414	−19.820	1.00	0.00	H
ATOM	3830	HE1	TYR	A	242	23.421	−19.895	−15.865	1.00	0.00	H
ATOM	3831	HE2	TYR	A	242	21.488	−19.015	−19.608	1.00	0.00	H
ATOM	3832	HH	TYR	A	242	23.598	−17.760	−18.221	1.00	0.00	H
ATOM	3833	N	ASN	A	243	19.658	−24.910	−15.672	1.00	0.00	N
ATOM	3834	CA	ASN	A	243	19.094	−25.023	−14.315	1.00	0.00	C
ATOM	3835	C	ASN	A	243	19.984	−24.412	−13.205	1.00	0.00	C
ATOM	3836	O	ASN	A	243	20.363	−25.095	−12.251	1.00	0.00	O
ATOM	3837	CB	ASN	A	243	18.737	−26.503	−14.042	1.00	0.00	C
ATOM	3838	CG	ASN	A	243	17.537	−27.004	−14.826	1.00	0.00	C
ATOM	3839	OD1	ASN	A	243	16.602	−26.276	−15.103	1.00	0.00	O
ATOM	3840	ND2	ASN	A	243	17.488	−28.277	−15.142	1.00	0.00	N
ATOM	3841	H	ASN	A	243	19.974	−25.762	−16.111	1.00	0.00	H
ATOM	3842	HA	ASN	A	243	18.165	−24.451	−14.285	1.00	0.00	H
ATOM	3843	HB2	ASN	A	243	19.605	−27.129	−14.253	1.00	0.00	H

```
ATOM   3844  HB3 ASN A 243      18.484 −26.627 −12.989  1.00  0.00           H
ATOM   3845 HD21 ASN A 243      18.243 −28.889 −14.904  1.00  0.00           H
ATOM   3846 HD22 ASN A 243      16.638 −28.603 −15.579  1.00  0.00           H
ATOM   3847  N   SER A 244      20.320 −23.122 −13.300  1.00  0.00           N
ATOM   3848  CA  SER A 244      21.146 −22.438 −12.292  1.00  0.00           C
ATOM   3849  C   SER A 244      20.483 −22.451 −10.914  1.00  0.00           C
ATOM   3850  O   SER A 244      19.441 −21.827 −10.715  1.00  0.00           O
ATOM   3851  CB  SER A 244      21.423 −20.979 −12.658  1.00  0.00           C
ATOM   3852  OG  SER A 244      22.041 −20.887 −13.917  1.00  0.00           O
ATOM   3853  H   SER A 244      19.959 −22.596 −14.087  1.00  0.00           H
ATOM   3854  HA  SER A 244      22.107 −22.949 −12.227  1.00  0.00           H
ATOM   3855  HB2 SER A 244      20.494 −20.412 −12.658  1.00  0.00           H
ATOM   3856  HB3 SER A 244      22.090 −20.547 −11.909  1.00  0.00           H
ATOM   3857  HG  SER A 244      21.383 −20.965 −14.616  1.00  0.00           H
ATOM   3858  N   LEU A 245      21.168 −23.003  −9.908  1.00  0.00           N
ATOM   3859  CA  LEU A 245      20.837 −22.810  −8.487  1.00  0.00           C
ATOM   3860  C   LEU A 245      19.348 −23.088  −8.158  1.00  0.00           C
ATOM   3861  O   LEU A 245      18.684 −22.270  −7.524  1.00  0.00           O
ATOM   3862  CB  LEU A 245      21.321 −21.415  −8.024  1.00  0.00           C
ATOM   3863  CG  LEU A 245      22.781 −21.057  −8.365  1.00  0.00           C
ATOM   3864  CD1 LEU A 245      23.047 −19.604  −7.985  1.00  0.00           C
ATOM   3865  CD2 LEU A 245      23.780 −21.952  −7.631  1.00  0.00           C
ATOM   3866  H   LEU A 245      22.026 −23.483 −10.137  1.00  0.00           H
ATOM   3867  HA  LEU A 245      21.401 −23.552  −7.921  1.00  0.00           H
ATOM   3868  HB2 LEU A 245      20.684 −20.665  −8.488  1.00  0.00           H
ATOM   3869  HB3 LEU A 245      21.185 −21.338  −6.944  1.00  0.00           H
ATOM   3870  HG  LEU A 245      22.946 −21.153  −9.438  1.00  0.00           H
ATOM   3871 HD11 LEU A 245      22.375 −18.949  −8.539  1.00  0.00           H
ATOM   3872 HD12 LEU A 245      22.911 −19.460  −6.913  1.00  0.00           H
ATOM   3873 HD13 LEU A 245      24.072 −19.352  −8.256  1.00  0.00           H
```

ATOM 3874 HD21 LEU A 245 24.795 −21.631 −7.869 1.00 0.00 H
ATOM 3875 HD22 LEU A 245 23.663 −22.987 −7.948 1.00 0.00 H
ATOM 3876 HD23 LEU A 245 23.628 −21.878 −6.554 1.00 0.00 H
ATOM 3877 N GLY A 246 18.781 −24.152 −8.742 1.00 0.00 N
ATOM 3878 CA GLY A 246 17.403 −24.604 −8.489 1.00 0.00 C
ATOM 3879 C GLY A 246 16.291 −23.976 −9.343 1.00 0.00 C
ATOM 3880 O GLY A 246 15.135 −24.330 −9.145 1.00 0.00 O
ATOM 3881 H GLY A 246 19.359 −24.719 −9.345 1.00 0.00 H
ATOM 3882 HA2 GLY A 246 17.357 −25.681 −8.640 1.00 0.00 H
ATOM 3883 HA3 GLY A 246 17.154 −24.406 −7.445 1.00 0.00 H
ATOM 3884 N GLN A 247 16.608 −23.114 −10.314 1.00 0.00 N
ATOM 3885 CA GLN A 247 15.624 −22.408 −11.156 1.00 0.00 C
ATOM 3886 C GLN A 247 16.215 −22.161 −12.562 1.00 0.00 C
ATOM 3887 O GLN A 247 17.385 −21.771 −12.643 1.00 0.00 O
ATOM 3888 CB GLN A 247 15.210 −21.115 −10.416 1.00 0.00 C
ATOM 3889 CG GLN A 247 14.780 −19.906 −11.266 1.00 0.00 C
ATOM 3890 CD GLN A 247 13.436 −20.042 −11.966 1.00 0.00 C
ATOM 3891 OE1 GLN A 247 12.672 −20.968 −11.755 1.00 0.00 O
ATOM 3892 NE2 GLN A 247 13.119 −19.091 −12.809 1.00 0.00 N
ATOM 3893 H GLN A 247 17.581 −22.865 −10.440 1.00 0.00 H
ATOM 3894 HA GLN A 247 14.731 −23.023 −11.250 1.00 0.00 H
ATOM 3895 HB2 GLN A 247 14.413 −21.356 −9.710 1.00 0.00 H
ATOM 3896 HB3 GLN A 247 16.051 −20.782 −9.814 1.00 0.00 H
ATOM 3897 HG2 GLN A 247 14.719 −19.035 −10.613 1.00 0.00 H
ATOM 3898 HG3 GLN A 247 15.536 −19.686 −12.017 1.00 0.00 H
ATOM 3899 HE21 GLN A 247 13.765 −18.350 −13.000 1.00 0.00 H
ATOM 3900 HE22 GLN A 247 12.212 −19.109 −13.265 1.00 0.00 H
ATOM 3901 N PRO A 248 15.478 −22.358 −13.675 1.00 0.00 N
ATOM 3902 CA PRO A 248 16.040 −22.151 −15.006 1.00 0.00 C
ATOM 3903 C PRO A 248 16.356 −20.681 −15.306 1.00 0.00 C

ATOM 3904 O PRO A 248 15.513 −19.795 −15.154 1.00 0.00 O
ATOM 3905 CB PRO A 248 15.046 −22.736 −16.012 1.00 0.00 C
ATOM 3906 CG PRO A 248 14.166 −23.658 −15.169 1.00 0.00 C
ATOM 3907 CD PRO A 248 14.169 −22.986 −13.798 1.00 0.00 C
ATOM 3908 HA PRO A 248 16.958 −22.731 −15.071 1.00 0.00 H
ATOM 3909 HB2 PRO A 248 15.565 −23.279 −16.800 1.00 0.00 H
ATOM 3910 HB3 PRO A 248 14.441 −21.947 −16.452 1.00 0.00 H
ATOM 3911 HG2 PRO A 248 14.627 −24.641 −15.092 1.00 0.00 H
ATOM 3912 HG3 PRO A 248 13.159 −23.740 −15.577 1.00 0.00 H
ATOM 3913 HD2 PRO A 248 13.394 −22.221 −13.776 1.00 0.00 H
ATOM 3914 HD3 PRO A 248 13.987 −23.734 −13.027 1.00 0.00 H
ATOM 3915 N VAL A 249 17.515 −20.472 −15.924 1.00 0.00 N
ATOM 3916 CA VAL A 249 17.921 −19.226 −16.585 1.00 0.00 C
ATOM 3917 C VAL A 249 17.888 −19.452 −18.093 1.00 0.00 C
ATOM 3918 O VAL A 249 18.285 −20.518 −18.568 1.00 0.00 O
ATOM 3919 CB VAL A 249 19.322 −18.783 −16.121 1.00 0.00 C
ATOM 3920 CG1 VAL A 249 19.739 −17.423 −16.695 1.00 0.00 C
ATOM 3921 CG2 VAL A 249 19.403 −18.657 −14.599 1.00 0.00 C
ATOM 3922 H VAL A 249 18.123 −21.272 −16.060 1.00 0.00 H
ATOM 3923 HA VAL A 249 17.215 −18.434 −16.338 1.00 0.00 H
ATOM 3924 HB VAL A 249 20.043 −19.537 −16.433 1.00 0.00 H
ATOM 3925 HG11 VAL A 249 20.722 −17.148 −16.312 1.00 0.00 H
ATOM 3926 HG12 VAL A 249 19.796 −17.466 −17.781 1.00 0.00 H
ATOM 3927 HG13 VAL A 249 19.028 −16.658 −16.392 1.00 0.00 H
ATOM 3928 HG21 VAL A 249 20.424 −18.423 −14.308 1.00 0.00 H
ATOM 3929 HG22 VAL A 249 18.742 −17.861 −14.261 1.00 0.00 H
ATOM 3930 HG23 VAL A 249 19.108 −19.590 −14.126 1.00 0.00 H
ATOM 3931 N PHE A 250 17.418 −18.464 −18.848 1.00 0.00 N
ATOM 3932 CA PHE A 250 17.481 −18.426 −20.305 1.00 0.00 C
ATOM 3933 C PHE A 250 18.518 −17.397 −20.779 1.00 0.00 C

ATOM 3934 O PHE A 250 18.691 −16.342 −20.166 1.00 0.00 O
ATOM 3935 CB PHE A 250 16.084 −18.132 −20.869 1.00 0.00 C
ATOM 3936 CG PHE A 250 15.937 −18.457 −22.345 1.00 0.00 C
ATOM 3937 CD1 PHE A 250 15.575 −19.760 −22.729 1.00 0.00 C
ATOM 3938 CD2 PHE A 250 16.180 −17.485 −23.334 1.00 0.00 C
ATOM 3939 CE1 PHE A 250 15.489 −20.107 −24.089 1.00 0.00 C
ATOM 3940 CE2 PHE A 250 16.076 −17.827 −24.695 1.00 0.00 C
ATOM 3941 CZ PHE A 250 15.729 −19.136 −25.073 1.00 0.00 C
ATOM 3942 H PHE A 250 17.135 −17.608 −18.380 1.00 0.00 H
ATOM 3943 HA PHE A 250 17.786 −19.405 −20.674 1.00 0.00 H
ATOM 3944 HB2 PHE A 250 15.351 −18.725 −20.320 1.00 0.00 H
ATOM 3945 HB3 PHE A 250 15.848 −17.082 −20.700 1.00 0.00 H
ATOM 3946 HD1 PHE A 250 15.354 −20.492 −21.968 1.00 0.00 H
ATOM 3947 HD2 PHE A 250 16.450 −16.480 −23.047 1.00 0.00 H
ATOM 3948 HE1 PHE A 250 15.211 −21.109 −24.379 1.00 0.00 H
ATOM 3949 HE2 PHE A 250 16.257 −17.083 −25.454 1.00 0.00 H
ATOM 3950 HZ PHE A 250 15.637 −19.396 −26.119 1.00 0.00 H
ATOM 3951 N TYR A 251 19.100 −17.638 −21.951 1.00 0.00 N
ATOM 3952 CA TYR A 251 19.944 −16.706 −22.695 1.00 0.00 C
ATOM 3953 C TYR A 251 19.599 −16.759 −24.190 1.00 0.00 C
ATOM 3954 O TYR A 251 19.381 −17.837 −24.745 1.00 0.00 O
ATOM 3955 CB TYR A 251 21.429 −17.005 −22.416 1.00 0.00 C
ATOM 3956 CG TYR A 251 22.399 −16.590 −23.512 1.00 0.00 C
ATOM 3957 CD1 TYR A 251 23.106 −15.372 −23.450 1.00 0.00 C
ATOM 3958 CD2 TYR A 251 22.575 −17.439 −24.620 1.00 0.00 C
ATOM 3959 CE1 TYR A 251 24.017 −15.034 −24.474 1.00 0.00 C
ATOM 3960 CE2 TYR A 251 23.468 −17.100 −25.644 1.00 0.00 C
ATOM 3961 CZ TYR A 251 24.214 −15.911 −25.563 1.00 0.00 C
ATOM 3962 OH TYR A 251 25.143 −15.666 −26.523 1.00 0.00 O
ATOM 3963 H TYR A 251 18.900 −18.531 −22.395 1.00 0.00 H

```
ATOM   3964  HA  TYR A 251      19.740 −15.694 −22.355  1.00  0.00           H
ATOM   3965  HB2 TYR A 251      21.712 −16.524 −21.477  1.00  0.00           H
ATOM   3966  HB3 TYR A 251      21.548 −18.081 −22.270  1.00  0.00           H
ATOM   3967  HD1 TYR A 251      22.960 −14.702 −22.613  1.00  0.00           H
ATOM   3968  HD2 TYR A 251      22.009 −18.354 −24.696  1.00  0.00           H
ATOM   3969  HE1 TYR A 251      24.593 −14.122 −24.421  1.00  0.00           H
ATOM   3970  HE2 TYR A 251      23.586 −17.756 −26.487  1.00  0.00           H
ATOM   3971  HH  TYR A 251      24.914 −16.176 −27.303  1.00  0.00           H
ATOM   3972  N   GLN A 252      19.701 −15.618 −24.870  1.00  0.00           N
ATOM   3973  CA  GLN A 252      19.637 −15.494 −26.324  1.00  0.00           C
ATOM   3974  C   GLN A 252      20.860 −14.718 −26.823  1.00  0.00           C
ATOM   3975  O   GLN A 252      21.095 −13.594 −26.387  1.00  0.00           O
ATOM   3976  CB  GLN A 252      18.308 −14.816 −26.706  1.00  0.00           C
ATOM   3977  CG  GLN A 252      18.171 −14.571 −28.215  1.00  0.00           C
ATOM   3978  CD  GLN A 252      16.823 −13.982 −28.631  1.00  0.00           C
ATOM   3979  OE1 GLN A 252      16.031 −13.501 −27.830  1.00  0.00           O
ATOM   3980  NE2 GLN A 252      16.584 −13.846 −29.915  1.00  0.00           N
ATOM   3981  H   GLN A 252      19.973 −14.785 −24.353  1.00  0.00           H
ATOM   3982  HA  GLN A 252      19.660 −16.484 −26.781  1.00  0.00           H
ATOM   3983  HB2 GLN A 252      17.489 −15.456 −26.385  1.00  0.00           H
ATOM   3984  HB3 GLN A 252      18.229 −13.861 −26.185  1.00  0.00           H
ATOM   3985  HG2 GLN A 252      18.304 −15.520 −28.736  1.00  0.00           H
ATOM   3986  HG3 GLN A 252      18.948 −13.881 −28.541  1.00  0.00           H
ATOM   3987 HE21 GLN A 252      17.261 −14.193 −30.592  1.00  0.00           H
ATOM   3988 HE22 GLN A 252      15.667 −13.558 −30.206  1.00  0.00           H
ATOM   3989  N   ALA A 253      21.560 −15.232 −27.836  1.00  0.00           N
ATOM   3990  CA  ALA A 253      22.638 −14.482 −28.468  1.00  0.00           C
ATOM   3991  C   ALA A 253      22.082 −13.266 −29.226  1.00  0.00           C
ATOM   3992  O   ALA A 253      21.237 −13.405 −30.114  1.00  0.00           O
ATOM   3993  CB  ALA A 253      23.439 −15.415 −29.375  1.00  0.00           C
```

ATOM	3994	H	ALA	A	253	21.343	−16.157	−28.193	1.00	0.00	H
ATOM	3995	HA	ALA	A	253	23.306	−14.118	−27.685	1.00	0.00	H
ATOM	3996	HB1	ALA	A	253	23.830	−16.240	−28.784	1.00	0.00	H
ATOM	3997	HB2	ALA	A	253	24.269	−14.862	−29.814	1.00	0.00	H
ATOM	3998	HB3	ALA	A	253	22.799	−15.816	−30.161	1.00	0.00	H
ATOM	3999	N	SER	A	254	22.593	−12.080	−28.905	1.00	0.00	N
ATOM	4000	CA	SER	A	254	22.084	−10.776	−29.349	1.00	0.00	C
ATOM	4001	C	SER	A	254	22.418	−10.437	−30.816	1.00	0.00	C
ATOM	4002	O	SER	A	254	23.145	−9.493	−31.105	1.00	0.00	O
ATOM	4003	CB	SER	A	254	22.608	−9.713	−28.377	1.00	0.00	C
ATOM	4004	OG	SER	A	254	22.256	−10.059	−27.050	1.00	0.00	O
ATOM	4005	H	SER	A	254	23.167	−12.049	−28.074	1.00	0.00	H
ATOM	4006	HA	SER	A	254	20.998	−10.782	−29.259	1.00	0.00	H
ATOM	4007	HB2	SER	A	254	23.691	−9.663	−28.444	1.00	0.00	H
ATOM	4008	HB3	SER	A	254	22.191	−8.747	−28.642	1.00	0.00	H
ATOM	4009	HG	SER	A	254	22.183	−9.229	−26.530	1.00	0.00	H
ATOM	4010	N	TYR	A	255	21.943	−11.249	−31.763	1.00	0.00	N
ATOM	4011	CA	TYR	A	255	22.236	−11.165	−33.205	1.00	0.00	C
ATOM	4012	C	TYR	A	255	21.496	−10.041	−33.969	1.00	0.00	C
ATOM	4013	O	TYR	A	255	21.383	−10.106	−35.192	1.00	0.00	O
ATOM	4014	CB	TYR	A	255	22.029	−12.550	−33.848	1.00	0.00	C
ATOM	4015	CG	TYR	A	255	23.259	−13.434	−33.824	1.00	0.00	C
ATOM	4016	CD1	TYR	A	255	24.161	−13.391	−34.905	1.00	0.00	C
ATOM	4017	CD2	TYR	A	255	23.505	−14.296	−32.741	1.00	0.00	C
ATOM	4018	CE1	TYR	A	255	25.306	−14.211	−34.904	1.00	0.00	C
ATOM	4019	CE2	TYR	A	255	24.648	−15.120	−32.737	1.00	0.00	C
ATOM	4020	CZ	TYR	A	255	25.552	−15.076	−33.818	1.00	0.00	C
ATOM	4021	OH	TYR	A	255	26.658	−15.866	−33.802	1.00	0.00	O
ATOM	4022	H	TYR	A	255	21.406	−12.045	−31.441	1.00	0.00	H
ATOM	4023	HA	TYR	A	255	23.294	−10.924	−33.308	1.00	0.00	H

ATOM 4024 HB2 TYR A 255 21.740 −12.452 −34.891 1.00 0.00 H
ATOM 4025 HB3 TYR A 255 21.203 −13.052 −33.353 1.00 0.00 H
ATOM 4026 HD1 TYR A 255 23.979 −12.723 −35.738 1.00 0.00 H
ATOM 4027 HD2 TYR A 255 22.815 −14.320 −31.913 1.00 0.00 H
ATOM 4028 HE1 TYR A 255 26.004 −14.158 −35.727 1.00 0.00 H
ATOM 4029 HE2 TYR A 255 24.845 −15.789 −31.917 1.00 0.00 H
ATOM 4030 HH TYR A 255 26.718 −16.384 −34.609 1.00 0.00 H
ATOM 4031 N SER A 256 21.010 −8.994 −33.300 1.00 0.00 N
ATOM 4032 CA SER A 256 20.677 −7.727 −33.972 1.00 0.00 C
ATOM 4033 C SER A 256 20.798 −6.541 −33.004 1.00 0.00 C
ATOM 4034 O SER A 256 21.832 −6.417 −32.354 1.00 0.00 O
ATOM 4035 CB SER A 256 19.353 −7.796 −34.762 1.00 0.00 C
ATOM 4036 OG SER A 256 18.171 −7.810 −33.983 1.00 0.00 O
ATOM 4037 H SER A 256 21.242 −8.931 −32.319 1.00 0.00 H
ATOM 4038 HA SER A 256 21.452 −7.552 −34.719 1.00 0.00 H
ATOM 4039 HB2 SER A 256 19.311 −6.919 −35.406 1.00 0.00 H
ATOM 4040 HB3 SER A 256 19.358 −8.668 −35.415 1.00 0.00 H
ATOM 4041 HG SER A 256 17.454 −7.611 −34.607 1.00 0.00 H
ATOM 4042 N TRP A 257 19.880 −5.576 −33.061 1.00 0.00 N
ATOM 4043 CA TRP A 257 20.076 −4.195 −32.608 1.00 0.00 C
ATOM 4044 C TRP A 257 20.078 −3.988 −31.083 1.00 0.00 C
ATOM 4045 O TRP A 257 20.686 −3.028 −30.620 1.00 0.00 O
ATOM 4046 CB TRP A 257 19.001 −3.336 −33.283 1.00 0.00 C
ATOM 4047 CG TRP A 257 17.585 −3.776 −33.052 1.00 0.00 C
ATOM 4048 CD1 TRP A 257 16.830 −4.473 −33.931 1.00 0.00 C
ATOM 4049 CD2 TRP A 257 16.740 −3.570 −31.876 1.00 0.00 C
ATOM 4050 NE1 TRP A 257 15.603 −4.758 −33.364 1.00 0.00 N
ATOM 4051 CE2 TRP A 257 15.489 −4.219 −32.099 1.00 0.00 C
ATOM 4052 CE3 TRP A 257 16.891 −2.866 −30.660 1.00 0.00 C
ATOM 4053 CZ2 TRP A 257 14.461 −4.217 −31.145 1.00 0.00 C

ATOM 4054 CZ3 TRP A 257 15.861 −2.843 −29.702 1.00 0.00 C
ATOM 4055 CH2 TRP A 257 14.653 −3.525 −29.935 1.00 0.00 C
ATOM 4056 H TRP A 257 19.040 −5.767 −33.588 1.00 0.00 H
ATOM 4057 HA TRP A 257 21.049 −3.851 −32.960 1.00 0.00 H
ATOM 4058 HB2 TRP A 257 19.189 −3.326 −34.357 1.00 0.00 H
ATOM 4059 HB3 TRP A 257 19.109 −2.314 −32.930 1.00 0.00 H
ATOM 4060 HD1 TRP A 257 17.157 −4.774 −34.921 1.00 0.00 H
ATOM 4061 HE3 TRP A 257 17.813 −2.336 −30.461 1.00 0.00 H
ATOM 4062 HZ2 TRP A 257 13.537 −4.744 −31.331 1.00 0.00 H
ATOM 4063 HZ3 TRP A 257 16.011 −2.303 −28.776 1.00 0.00 H
ATOM 4064 HH2 TRP A 257 13.875 −3.518 −29.185 1.00 0.00 H
ATOM 4065 HE1 TRP A 257 14.892 −5.302 −33.829 1.00 0.00 H
ATOM 4066 N ASP A 258 19.457 −4.875 −30.303 1.00 0.00 N
ATOM 4067 CA ASP A 258 19.566 −4.928 −28.833 1.00 0.00 C
ATOM 4068 C ASP A 258 20.871 −5.653 −28.450 1.00 0.00 C
ATOM 4069 O ASP A 258 20.862 −6.870 −28.266 1.00 0.00 O
ATOM 4070 CB ASP A 258 18.299 −5.613 −28.266 1.00 0.00 C
ATOM 4071 CG ASP A 258 18.248 −5.793 −26.738 1.00 0.00 C
ATOM 4072 OD1 ASP A 258 19.117 −5.276 −26.005 1.00 0.00 O
ATOM 4073 OD2 ASP A 258 17.266 −6.389 −26.227 1.00 0.00 O
ATOM 4074 H ASP A 258 18.995 −5.644 −30.757 1.00 0.00 H
ATOM 4075 HA ASP A 258 19.613 −3.915 −28.429 1.00 0.00 H
ATOM 4076 HB2 ASP A 258 17.427 −5.031 −28.566 1.00 0.00 H
ATOM 4077 HB3 ASP A 258 18.211 −6.596 −28.723 1.00 0.00 H
ATOM 4078 N THR A 259 22.026 −4.993 −28.636 1.00 0.00 N
ATOM 4079 CA THR A 259 23.347 −5.671 −28.669 1.00 0.00 C
ATOM 4080 C THR A 259 23.848 −6.126 −27.297 1.00 0.00 C
ATOM 4081 O THR A 259 24.853 −6.842 −27.205 1.00 0.00 O
ATOM 4082 CB THR A 259 24.438 −4.816 −29.336 1.00 0.00 C
ATOM 4083 OG1 THR A 259 24.910 −3.772 −28.516 1.00 0.00 O

ATOM 4084 CG2 THR A 259 23.978 −4.224 −30.663 1.00 0.00 C
ATOM 4085 H THR A 259 21.989 −4.007 −28.886 1.00 0.00 H
ATOM 4086 HA THR A 259 23.235 −6.569 −29.275 1.00 0.00 H
ATOM 4087 HB THR A 259 25.286 −5.467 −29.530 1.00 0.00 H
ATOM 4088 HG1 THR A 259 25.388 −4.172 −27.777 1.00 0.00 H
ATOM 4089 HG21 THR A 259 23.209 −3.478 −30.490 1.00 0.00 H
ATOM 4090 HG22 THR A 259 23.583 −5.010 −31.298 1.00 0.00 H
ATOM 4091 HG23 THR A 259 24.817 −3.760 −31.169 1.00 0.00 H
ATOM 4092 N MET A 260 23.191 −5.654 −26.237 1.00 0.00 N
ATOM 4093 CA MET A 260 23.445 −6.013 −24.843 1.00 0.00 C
ATOM 4094 C MET A 260 23.123 −7.489 −24.592 1.00 0.00 C
ATOM 4095 O MET A 260 22.294 −8.077 −25.287 1.00 0.00 O
ATOM 4096 CB MET A 260 22.605 −5.113 −23.918 1.00 0.00 C
ATOM 4097 CG MET A 260 22.806 −3.613 −24.184 1.00 0.00 C
ATOM 4098 SD MET A 260 24.529 −3.038 −24.210 1.00 0.00 S
ATOM 4099 CE MET A 260 24.994 −3.363 −22.491 1.00 0.00 C
ATOM 4100 H MET A 260 22.361 −5.113 −26.430 1.00 0.00 H
ATOM 4101 HA MET A 260 24.500 −5.850 −24.627 1.00 0.00 H
ATOM 4102 HB2 MET A 260 21.548 −5.345 −24.058 1.00 0.00 H
ATOM 4103 HB3 MET A 260 22.862 −5.331 −22.881 1.00 0.00 H
ATOM 4104 HG2 MET A 260 22.357 −3.367 −25.147 1.00 0.00 H
ATOM 4105 HG3 MET A 260 22.265 −3.052 −23.421 1.00 0.00 H
ATOM 4106 HE1 MET A 260 24.352 −2.776 −21.840 1.00 0.00 H
ATOM 4107 HE2 MET A 260 24.873 −4.420 −22.259 1.00 0.00 H
ATOM 4108 HE3 MET A 260 26.031 −3.077 −22.328 1.00 0.00 H
ATOM 4109 N ILE A 261 23.755 −8.113 −23.594 1.00 0.00 N
ATOM 4110 CA ILE A 261 23.518 −9.533 −23.295 1.00 0.00 C
ATOM 4111 C ILE A 261 22.059 −9.792 −22.872 1.00 0.00 C
ATOM 4112 O ILE A 261 21.601 −9.360 −21.813 1.00 0.00 O
ATOM 4113 CB ILE A 261 24.561 −10.088 −22.299 1.00 0.00 C

ATOM	4114	CG1	ILE	A	261	24.378	−11.617	−22.165	1.00	0.00	C
ATOM	4115	CG2	ILE	A	261	24.552	−9.373	−20.933	1.00	0.00	C
ATOM	4116	CD1	ILE	A	261	25.534	−12.326	−21.448	1.00	0.00	C
ATOM	4117	H	ILE	A	261	24.461	−7.605	−23.070	1.00	0.00	H
ATOM	4118	HA	ILE	A	261	23.673	−10.072	−24.231	1.00	0.00	H
ATOM	4119	HB	ILE	A	261	25.540	−9.919	−22.749	1.00	0.00	H
ATOM	4120	HG12	ILE	A	261	23.449	−11.839	−21.640	1.00	0.00	H
ATOM	4121	HG13	ILE	A	261	24.304	−12.046	−23.165	1.00	0.00	H
ATOM	4122	HG21	ILE	A	261	25.359	−9.745	−20.307	1.00	0.00	H
ATOM	4123	HG22	ILE	A	261	23.614	−9.531	−20.410	1.00	0.00	H
ATOM	4124	HG23	ILE	A	261	24.685	−8.300	−21.073	1.00	0.00	H
ATOM	4125	HD11	ILE	A	261	25.372	−13.404	−21.483	1.00	0.00	H
ATOM	4126	HD12	ILE	A	261	26.477	−12.095	−21.945	1.00	0.00	H
ATOM	4127	HD13	ILE	A	261	25.583	−12.015	−20.407	1.00	0.00	H
ATOM	4128	N	LYS	A	262	21.324	−10.557	−23.686	1.00	0.00	N
ATOM	4129	CA	LYS	A	262	19.965	−11.004	−23.363	1.00	0.00	C
ATOM	4130	C	LYS	A	262	20.021	−12.295	−22.544	1.00	0.00	C
ATOM	4131	O	LYS	A	262	20.094	−13.389	−23.098	1.00	0.00	O
ATOM	4132	CB	LYS	A	262	19.143	−11.166	−24.648	1.00	0.00	C
ATOM	4133	CG	LYS	A	262	18.958	−9.846	−25.415	1.00	0.00	C
ATOM	4134	CD	LYS	A	262	18.036	−10.048	−26.622	1.00	0.00	C
ATOM	4135	CE	LYS	A	262	16.576	−10.145	−26.168	1.00	0.00	C
ATOM	4136	NZ	LYS	A	262	15.732	−10.797	−27.188	1.00	0.00	N
ATOM	4137	H	LYS	A	262	21.719	−10.837	−24.576	1.00	0.00	H
ATOM	4138	HA	LYS	A	262	19.473	−10.246	−22.752	1.00	0.00	H
ATOM	4139	HB2	LYS	A	262	19.629	−11.887	−25.302	1.00	0.00	H
ATOM	4140	HB3	LYS	A	262	18.166	−11.561	−24.377	1.00	0.00	H
ATOM	4141	HG2	LYS	A	262	19.925	−9.497	−25.774	1.00	0.00	H
ATOM	4142	HG3	LYS	A	262	18.538	−9.085	−24.756	1.00	0.00	H
ATOM	4143	HD2	LYS	A	262	18.140	−9.192	−27.291	1.00	0.00	H

ATOM	4144	HD3	LYS	A	262	18.337	−10.951	−27.157	1.00	0.00	H
ATOM	4145	HE2	LYS	A	262	16.509	−10.706	−25.232	1.00	0.00	H
ATOM	4146	HE3	LYS	A	262	16.216	−9.128	−25.979	1.00	0.00	H
ATOM	4147	HZ1	LYS	A	262	15.982	−10.487	−28.123	1.00	0.00	H
ATOM	4148	HZ2	LYS	A	262	15.824	−11.811	−27.178	1.00	0.00	H
ATOM	4149	HZ3	LYS	A	262	14.760	−10.538	−27.038	1.00	0.00	H
ATOM	4150	N	LEU	A	263	19.926	−12.165	−21.223	1.00	0.00	N
ATOM	4151	CA	LEU	A	263	19.771	−13.278	−20.276	1.00	0.00	C
ATOM	4152	C	LEU	A	263	18.744	−12.944	−19.186	1.00	0.00	C
ATOM	4153	O	LEU	A	263	18.462	−11.769	−18.959	1.00	0.00	O
ATOM	4154	CB	LEU	A	263	21.144	−13.706	−19.709	1.00	0.00	C
ATOM	4155	CG	LEU	A	263	21.687	−12.914	−18.499	1.00	0.00	C
ATOM	4156	CD1	LEU	A	263	23.035	−13.501	−18.074	1.00	0.00	C
ATOM	4157	CD2	LEU	A	263	21.903	−11.426	−18.774	1.00	0.00	C
ATOM	4158	H	LEU	A	263	19.902	−11.228	−20.846	1.00	0.00	H
ATOM	4159	HA	LEU	A	263	19.369	−14.124	−20.829	1.00	0.00	H
ATOM	4160	HB2	LEU	A	263	21.047	−14.746	−19.394	1.00	0.00	H
ATOM	4161	HB3	LEU	A	263	21.881	−13.685	−20.514	1.00	0.00	H
ATOM	4162	HG	LEU	A	263	20.994	−13.011	−17.665	1.00	0.00	H
ATOM	4163	HD11	LEU	A	263	23.756	−13.418	−18.886	1.00	0.00	H
ATOM	4164	HD12	LEU	A	263	23.416	−12.972	−17.200	1.00	0.00	H
ATOM	4165	HD13	LEU	A	263	22.912	−14.552	−17.808	1.00	0.00	H
ATOM	4166	HD21	LEU	A	263	22.342	−10.946	−17.899	1.00	0.00	H
ATOM	4167	HD22	LEU	A	263	22.568	−11.309	−19.624	1.00	0.00	H
ATOM	4168	HD23	LEU	A	263	20.957	−10.937	−18.991	1.00	0.00	H
ATOM	4169	N	GLY	A	264	18.201	−13.947	−18.498	1.00	0.00	N
ATOM	4170	CA	GLY	A	264	17.263	−13.753	−17.386	1.00	0.00	C
ATOM	4171	C	GLY	A	264	16.701	−15.063	−16.836	1.00	0.00	C
ATOM	4172	O	GLY	A	264	16.761	−16.093	−17.500	1.00	0.00	O
ATOM	4173	H	GLY	A	264	18.392	−14.902	−18.793	1.00	0.00	H

ATOM 4174 HA2 GLY A 264 17.766 −13.220 −16.579 1.00 0.00 H
ATOM 4175 HA3 GLY A 264 16.425 −13.147 −17.725 1.00 0.00 H
ATOM 4176 N ASP A 265 16.158 −15.038 −15.620 1.00 0.00 N
ATOM 4177 CA ASP A 265 15.366 −16.158 −15.088 1.00 0.00 C
ATOM 4178 C ASP A 265 14.077 −16.373 −15.905 1.00 0.00 C
ATOM 4179 O ASP A 265 13.507 −15.413 −16.431 1.00 0.00 O
ATOM 4180 CB ASP A 265 15.066 −15.909 −13.599 1.00 0.00 C
ATOM 4181 CG ASP A 265 16.284 −16.202 −12.721 1.00 0.00 C
ATOM 4182 OD1 ASP A 265 16.769 −17.349 −12.790 1.00 0.00 O
ATOM 4183 OD2 ASP A 265 16.570 −15.445 −11.768 1.00 0.00 O
ATOM 4184 H ASP A 265 16.061 −14.148 −15.159 1.00 0.00 H
ATOM 4185 HA ASP A 265 15.950 −17.076 −15.165 1.00 0.00 H
ATOM 4186 HB2 ASP A 265 14.736 −14.879 −13.454 1.00 0.00 H
ATOM 4187 HB3 ASP A 265 14.256 −16.568 −13.285 1.00 0.00 H
ATOM 4188 N VAL A 266 13.608 −17.621 −16.024 1.00 0.00 N
ATOM 4189 CA VAL A 266 12.295 −17.905 −16.636 1.00 0.00 C
ATOM 4190 C VAL A 266 11.166 −17.600 −15.648 1.00 0.00 C
ATOM 4191 O VAL A 266 11.154 −18.108 −14.529 1.00 0.00 O
ATOM 4192 CB VAL A 266 12.182 −19.337 −17.208 1.00 0.00 C
ATOM 4193 CG1 VAL A 266 13.321 −19.630 −18.194 1.00 0.00 C
ATOM 4194 CG2 VAL A 266 12.133 −20.456 −16.160 1.00 0.00 C
ATOM 4195 H VAL A 266 14.120 −18.388 −15.598 1.00 0.00 H
ATOM 4196 HA VAL A 266 12.171 −17.234 −17.484 1.00 0.00 H
ATOM 4197 HB VAL A 266 11.250 −19.387 −17.773 1.00 0.00 H
ATOM 4198 HG11 VAL A 266 13.355 −18.847 −18.948 1.00 0.00 H
ATOM 4199 HG12 VAL A 266 13.141 −20.583 −18.690 1.00 0.00 H
ATOM 4200 HG13 VAL A 266 14.278 −19.670 −17.673 1.00 0.00 H
ATOM 4201 HG21 VAL A 266 11.198 −20.408 −15.604 1.00 0.00 H
ATOM 4202 HG22 VAL A 266 12.964 −20.359 −15.463 1.00 0.00 H
ATOM 4203 HG23 VAL A 266 12.178 −21.427 −16.649 1.00 0.00 H

ATOM 4204 N ASP A 267 10.173 −16.818 −16.063 1.00 0.00 N
ATOM 4205 CA ASP A 267 8.910 −16.665 −15.328 1.00 0.00 C
ATOM 4206 C ASP A 267 7.862 −17.700 −15.777 1.00 0.00 C
ATOM 4207 O ASP A 267 6.863 −17.916 −15.092 1.00 0.00 O
ATOM 4208 CB ASP A 267 8.352 −15.234 −15.444 1.00 0.00 C
ATOM 4209 CG ASP A 267 9.416 −14.160 −15.680 1.00 0.00 C
ATOM 4210 OD1 ASP A 267 9.761 −13.988 −16.869 1.00 0.00 O
ATOM 4211 OD2 ASP A 267 9.451 −13.186 −14.897 1.00 0.00 O
ATOM 4212 H ASP A 267 10.232 −16.412 −16.993 1.00 0.00 H
ATOM 4213 HA ASP A 267 9.098 −16.846 −14.268 1.00 0.00 H
ATOM 4214 HB2 ASP A 267 7.648 −15.202 −16.275 1.00 0.00 H
ATOM 4215 HB3 ASP A 267 7.787 −15.005 −14.540 1.00 0.00 H
ATOM 4216 N THR A 268 8.035 −18.305 −16.959 1.00 0.00 N
ATOM 4217 CA THR A 268 7.220 −19.432 −17.452 1.00 0.00 C
ATOM 4218 C THR A 268 7.951 −20.164 −18.589 1.00 0.00 C
ATOM 4219 O THR A 268 8.837 −19.582 −19.218 1.00 0.00 O
ATOM 4220 CB THR A 268 5.803 −19.002 −17.913 1.00 0.00 C
ATOM 4221 OG1 THR A 268 5.441 −17.708 −17.480 1.00 0.00 O
ATOM 4222 CG2 THR A 268 4.748 −19.935 −17.320 1.00 0.00 C
ATOM 4223 H THR A 268 8.834 −18.035 −17.523 1.00 0.00 H
ATOM 4224 HA THR A 268 7.116 −20.137 −16.629 1.00 0.00 H
ATOM 4225 HB THR A 268 5.735 −19.029 −18.997 1.00 0.00 H
ATOM 4226 HG21 THR A 268 3.760 −19.623 −17.658 1.00 0.00 H
ATOM 4227 HG22 THR A 268 4.920 −20.959 −17.647 1.00 0.00 H
ATOM 4228 HG23 THR A 268 4.779 −19.887 −16.230 1.00 0.00 H
ATOM 4229 HG1 THR A 268 5.708 −17.665 −16.547 1.00 0.00 H
ATOM 4230 N VAL A 269 7.686 −21.466 −18.772 1.00 0.00 N
ATOM 4231 CA VAL A 269 8.402 −22.337 −19.738 1.00 0.00 C
ATOM 4232 C VAL A 269 7.494 −22.853 −20.860 1.00 0.00 C
ATOM 4233 O VAL A 269 7.849 −22.692 −22.025 1.00 0.00 O

ATOM 4234 CB VAL A 269 9.124 −23.493 −19.015 1.00 0.00 C
ATOM 4235 CG1 VAL A 269 9.828 −24.459 −19.977 1.00 0.00 C
ATOM 4236 CG2 VAL A 269 10.195 −22.941 −18.063 1.00 0.00 C
ATOM 4237 H VAL A 269 6.951 −21.878 −18.221 1.00 0.00 H
ATOM 4238 HA VAL A 269 9.172 −21.752 −20.241 1.00 0.00 H
ATOM 4239 HB VAL A 269 8.396 −24.059 −18.432 1.00 0.00 H
ATOM 4240 HG11 VAL A 269 9.101 −24.923 −20.645 1.00 0.00 H
ATOM 4241 HG12 VAL A 269 10.318 −25.257 −19.421 1.00 0.00 H
ATOM 4242 HG13 VAL A 269 10.567 −23.924 −20.573 1.00 0.00 H
ATOM 4243 HG21 VAL A 269 9.732 −22.333 −17.286 1.00 0.00 H
ATOM 4244 HG22 VAL A 269 10.903 −22.327 −18.620 1.00 0.00 H
ATOM 4245 HG23 VAL A 269 10.730 −23.757 −17.584 1.00 0.00 H
ATOM 4246 N ASP A 270 6.255 −23.232 −20.532 1.00 0.00 N
ATOM 4247 CA ASP A 270 5.142 −23.327 −21.483 1.00 0.00 C
ATOM 4248 C ASP A 270 4.130 −22.195 −21.183 1.00 0.00 C
ATOM 4249 O ASP A 270 3.421 −22.291 −20.177 1.00 0.00 O
ATOM 4250 CB ASP A 270 4.431 −24.700 −21.403 1.00 0.00 C
ATOM 4251 CG ASP A 270 5.237 −25.949 −21.791 1.00 0.00 C
ATOM 4252 OD1 ASP A 270 6.480 −25.913 −21.897 1.00 0.00 O
ATOM 4253 OD2 ASP A 270 4.629 −27.043 −21.840 1.00 0.00 O
ATOM 4254 H ASP A 270 6.017 −23.307 −19.561 1.00 0.00 H
ATOM 4255 HA ASP A 270 5.515 −23.226 −22.500 1.00 0.00 H
ATOM 4256 HB2 ASP A 270 3.557 −24.658 −22.055 1.00 0.00 H
ATOM 4257 HB3 ASP A 270 4.072 −24.846 −20.383 1.00 0.00 H
ATOM 4258 N PRO A 271 3.998 −21.149 −22.026 1.00 0.00 N
ATOM 4259 CA PRO A 271 4.957 −20.696 −23.040 1.00 0.00 C
ATOM 4260 C PRO A 271 6.233 −20.106 −22.406 1.00 0.00 C
ATOM 4261 O PRO A 271 6.238 −19.761 −21.226 1.00 0.00 O
ATOM 4262 CB PRO A 271 4.200 −19.625 −23.830 1.00 0.00 C
ATOM 4263 CG PRO A 271 3.339 −18.966 −22.753 1.00 0.00 C

ATOM 4264 CD PRO A 271 2.956 −20.143 −21.855 1.00 0.00 C

ATOM 4265 HA PRO A 271 5.223 −21.511 −23.705 1.00 0.00 H

ATOM 4266 HB2 PRO A 271 3.557 −20.103 −24.571 1.00 0.00 H

ATOM 4267 HB3 PRO A 271 4.867 −18.908 −24.312 1.00 0.00 H

ATOM 4268 HG2 PRO A 271 3.937 −18.251 −22.185 1.00 0.00 H

ATOM 4269 HG3 PRO A 271 2.460 −18.479 −23.176 1.00 0.00 H

ATOM 4270 HD2 PRO A 271 2.000 −20.560 −22.174 1.00 0.00 H

ATOM 4271 HD3 PRO A 271 2.892 −19.816 −20.816 1.00 0.00 H

ATOM 4272 N LEU A 272 7.292 −19.889 −23.194 1.00 0.00 N

ATOM 4273 CA LEU A 272 8.548 −19.352 −22.669 1.00 0.00 C

ATOM 4274 C LEU A 272 8.460 −17.831 −22.454 1.00 0.00 C

ATOM 4275 O LEU A 272 8.470 −17.048 −23.410 1.00 0.00 O

ATOM 4276 CB LEU A 272 9.727 −19.773 −23.563 1.00 0.00 C

ATOM 4277 CG LEU A 272 11.075 −19.184 −23.099 1.00 0.00 C

ATOM 4278 CD1 LEU A 272 11.530 −19.709 −21.735 1.00 0.00 C

ATOM 4279 CD2 LEU A 272 12.164 −19.495 −24.118 1.00 0.00 C

ATOM 4280 H LEU A 272 7.242 −20.124 −24.175 1.00 0.00 H

ATOM 4281 HA LEU A 272 8.713 −19.815 −21.695 1.00 0.00 H

ATOM 4282 HB2 LEU A 272 9.794 −20.862 −23.574 1.00 0.00 H

ATOM 4283 HB3 LEU A 272 9.525 −19.437 −24.580 1.00 0.00 H

ATOM 4284 HG LEU A 272 10.997 −18.101 −23.034 1.00 0.00 H

ATOM 4285 HD11 LEU A 272 12.528 −19.334 −21.509 1.00 0.00 H

ATOM 4286 HD12 LEU A 272 10.854 −19.362 −20.954 1.00 0.00 H

ATOM 4287 HD13 LEU A 272 11.542 −20.797 −21.735 1.00 0.00 H

ATOM 4288 HD21 LEU A 272 11.833 −19.220 −25.118 1.00 0.00 H

ATOM 4289 HD22 LEU A 272 13.051 −18.917 −23.868 1.00 0.00 H

ATOM 4290 HD23 LEU A 272 12.409 −20.553 −24.091 1.00 0.00 H

ATOM 4291 N ARG A 273 8.621 −17.405 −21.201 1.00 0.00 N

ATOM 4292 CA ARG A 273 8.668 −15.999 −20.776 1.00 0.00 C

ATOM 4293 C ARG A 273 9.810 −15.802 −19.776 1.00 0.00 C

ATOM 4294 O ARG A 273 10.035 −16.637 −18.903 1.00 0.00 O
ATOM 4295 CB ARG A 273 7.262 −15.604 −20.275 1.00 0.00 C
ATOM 4296 CG ARG A 273 7.163 −14.381 −19.355 1.00 0.00 C
ATOM 4297 CD ARG A 273 7.777 −13.066 −19.871 1.00 0.00 C
ATOM 4298 NE ARG A 273 8.600 −12.473 −18.803 1.00 0.00 N
ATOM 4299 CZ ARG A 273 8.393 −11.370 −18.118 1.00 0.00 C
ATOM 4300 NH1 ARG A 273 7.639 −10.399 −18.545 1.00 0.00 N
ATOM 4301 NH2 ARG A 273 8.920 −11.253 −16.936 1.00 0.00 N
ATOM 4302 H ARG A 273 8.672 −18.118 −20.474 1.00 0.00 H
ATOM 4303 HA ARG A 273 8.896 −15.384 −21.647 1.00 0.00 H
ATOM 4304 HB2 ARG A 273 6.620 −15.443 −21.142 1.00 0.00 H
ATOM 4305 HB3 ARG A 273 6.844 −16.446 −19.718 1.00 0.00 H
ATOM 4306 HG2 ARG A 273 6.112 −14.202 −19.125 1.00 0.00 H
ATOM 4307 HG3 ARG A 273 7.644 −14.658 −18.418 1.00 0.00 H
ATOM 4308 HD2 ARG A 273 8.404 −13.255 −20.741 1.00 0.00 H
ATOM 4309 HD3 ARG A 273 6.973 −12.393 −20.174 1.00 0.00 H
ATOM 4310 HE ARG A 273 9.198 −13.126 −18.294 1.00 0.00 H
ATOM 4311 HH11 ARG A 273 7.356 −10.416 −19.523 1.00 0.00 H
ATOM 4312 HH12 ARG A 273 7.552 −9.560 −18.016 1.00 0.00 H
ATOM 4313 HH21 ARG A 273 9.345 −12.100 −16.530 1.00 0.00 H
ATOM 4314 HH22 ARG A 273 8.623 −10.545 −16.303 1.00 0.00 H
ATOM 4315 N VAL A 274 10.613 −14.763 −20.019 1.00 0.00 N
ATOM 4316 CA VAL A 274 11.924 −14.529 −19.395 1.00 0.00 C
ATOM 4317 C VAL A 274 11.989 −13.105 −18.848 1.00 0.00 C
ATOM 4318 O VAL A 274 11.765 −12.147 −19.593 1.00 0.00 O
ATOM 4319 CB VAL A 274 13.061 −14.764 −20.418 1.00 0.00 C
ATOM 4320 CG1 VAL A 274 14.453 −14.578 −19.799 1.00 0.00 C
ATOM 4321 CG2 VAL A 274 13.002 −16.166 −21.040 1.00 0.00 C
ATOM 4322 H VAL A 274 10.353 −14.131 −20.758 1.00 0.00 H
ATOM 4323 HA VAL A 274 12.061 −15.219 −18.561 1.00 0.00 H

ATOM 4324 HB VAL A 274 12.955 −14.041 −21.227 1.00 0.00 H
ATOM 4325 HG11 VAL A 274 15.219 −14.748 −20.555 1.00 0.00 H
ATOM 4326 HG12 VAL A 274 14.573 −13.563 −19.427 1.00 0.00 H
ATOM 4327 HG13 VAL A 274 14.592 −15.285 −18.981 1.00 0.00 H
ATOM 4328 HG21 VAL A 274 13.822 −16.290 −21.746 1.00 0.00 H
ATOM 4329 HG22 VAL A 274 13.076 −16.921 −20.259 1.00 0.00 H
ATOM 4330 HG23 VAL A 274 12.071 −16.304 −21.587 1.00 0.00 H
ATOM 4331 N GLN A 275 12.409 −12.939 −17.594 1.00 0.00 N
ATOM 4332 CA GLN A 275 12.701 −11.630 −17.008 1.00 0.00 C
ATOM 4333 C GLN A 275 14.114 −11.186 −17.397 1.00 0.00 C
ATOM 4334 O GLN A 275 15.071 −11.307 −16.628 1.00 0.00 O
ATOM 4335 CB GLN A 275 12.414 −11.589 −15.498 1.00 0.00 C
ATOM 4336 CG GLN A 275 12.926 −12.780 −14.676 1.00 0.00 C
ATOM 4337 CD GLN A 275 12.600 −12.608 −13.202 1.00 0.00 C
ATOM 4338 OE1 GLN A 275 13.440 −12.240 −12.394 1.00 0.00 O
ATOM 4339 NE2 GLN A 275 11.341 −12.699 −12.834 1.00 0.00 N
ATOM 4340 H GLN A 275 12.590 −13.771 −17.037 1.00 0.00 H
ATOM 4341 HA GLN A 275 12.022 −10.901 −17.453 1.00 0.00 H
ATOM 4342 HB2 GLN A 275 12.840 −10.672 −15.089 1.00 0.00 H
ATOM 4343 HB3 GLN A 275 11.336 −11.523 −15.370 1.00 0.00 H
ATOM 4344 HG2 GLN A 275 12.431 −13.688 −15.013 1.00 0.00 H
ATOM 4345 HG3 GLN A 275 14.003 −12.888 −14.798 1.00 0.00 H
ATOM 4346 HE21 GLN A 275 10.622 −12.923 −13.530 1.00 0.00 H
ATOM 4347 HE22 GLN A 275 11.133 −12.578 −11.863 1.00 0.00 H
ATOM 4348 N TRP A 276 14.245 −10.732 −18.647 1.00 0.00 N
ATOM 4349 CA TRP A 276 15.504 −10.239 −19.203 1.00 0.00 C
ATOM 4350 C TRP A 276 16.131 −9.175 −18.297 1.00 0.00 C
ATOM 4351 O TRP A 276 15.516 −8.140 −18.027 1.00 0.00 O
ATOM 4352 CB TRP A 276 15.290 −9.624 −20.590 1.00 0.00 C
ATOM 4353 CG TRP A 276 14.655 −10.480 −21.637 1.00 0.00 C

ATOM	4354	CD1	TRP	A	276	13.444	−10.248	−22.186	1.00	0.00	C
ATOM	4355	CD2	TRP	A	276	15.197	−11.638	−22.346	1.00	0.00	C
ATOM	4356	NE1	TRP	A	276	13.223	−11.134	−23.215	1.00	0.00	N
ATOM	4357	CE2	TRP	A	276	14.281	−11.998	−23.380	1.00	0.00	C
ATOM	4358	CE3	TRP	A	276	16.379	−12.403	−22.246	1.00	0.00	C
ATOM	4359	CZ2	TRP	A	276	14.544	−13.021	−24.298	1.00	0.00	C
ATOM	4360	CZ3	TRP	A	276	16.636	−13.464	−23.137	1.00	0.00	C
ATOM	4361	CH2	TRP	A	276	15.730	−13.761	−24.172	1.00	0.00	C
ATOM	4362	H	TRP	A	276	13.437	−10.783	−19.256	1.00	0.00	H
ATOM	4363	HA	TRP	A	276	16.185	−11.080	−19.297	1.00	0.00	H
ATOM	4364	HB2	TRP	A	276	14.682	−8.725	−20.479	1.00	0.00	H
ATOM	4365	HB3	TRP	A	276	16.261	−9.307	−20.971	1.00	0.00	H
ATOM	4366	HD1	TRP	A	276	12.774	−9.445	−21.907	1.00	0.00	H
ATOM	4367	HE3	TRP	A	276	17.082	−12.173	−21.461	1.00	0.00	H
ATOM	4368	HZ2	TRP	A	276	13.826	−13.250	−25.071	1.00	0.00	H
ATOM	4369	HZ3	TRP	A	276	17.540	−14.049	−23.034	1.00	0.00	H
ATOM	4370	HH2	TRP	A	276	15.934	−14.572	−24.857	1.00	0.00	H
ATOM	4371	HE1	TRP	A	276	12.414	−11.051	−23.833	1.00	0.00	H
ATOM	4372	N	ARG	A	277	17.423	−9.323	−17.997	1.00	0.00	N
ATOM	4373	CA	ARG	A	277	18.202	−8.260	−17.361	1.00	0.00	C
ATOM	4374	C	ARG	A	277	18.344	−7.103	−18.347	1.00	0.00	C
ATOM	4375	O	ARG	A	277	18.833	−7.284	−19.465	1.00	0.00	O
ATOM	4376	CB	ARG	A	277	19.578	−8.760	−16.886	1.00	0.00	C
ATOM	4377	CG	ARG	A	277	19.602	−10.100	−16.125	1.00	0.00	C
ATOM	4378	CD	ARG	A	277	18.531	−10.307	−15.045	1.00	0.00	C
ATOM	4379	NE	ARG	A	277	18.764	−9.476	−13.856	1.00	0.00	N
ATOM	4380	CZ	ARG	A	277	18.156	−9.603	−12.693	1.00	0.00	C
ATOM	4381	NH1	ARG	A	277	17.198	−10.467	−12.496	1.00	0.00	N
ATOM	4382	NH2	ARG	A	277	18.486	−8.830	−11.706	1.00	0.00	N
ATOM	4383	H	ARG	A	277	17.893	−10.170	−18.300	1.00	0.00	H

ATOM 4384 HA ARG A 277 17.648 −7.904 −16.490 1.00 0.00 H
ATOM 4385 HB2 ARG A 277 20.235 −8.866 −17.751 1.00 0.00 H
ATOM 4386 HB3 ARG A 277 20.006 −7.987 −16.250 1.00 0.00 H
ATOM 4387 HG2 ARG A 277 19.491 −10.905 −16.846 1.00 0.00 H
ATOM 4388 HG3 ARG A 277 20.587 −10.215 −15.673 1.00 0.00 H
ATOM 4389 HD2 ARG A 277 17.540 −10.105 −15.453 1.00 0.00 H
ATOM 4390 HD3 ARG A 277 18.562 −11.356 −14.749 1.00 0.00 H
ATOM 4391 HE ARG A 277 19.579 −8.856 −13.866 1.00 0.00 H
ATOM 4392 HH11 ARG A 277 16.963 −11.096 −13.240 1.00 0.00 H
ATOM 4393 HH12 ARG A 277 16.833 −10.625 −11.578 1.00 0.00 H
ATOM 4394 HH21 ARG A 277 19.139 −8.069 −11.927 1.00 0.00 H
ATOM 4395 HH22 ARG A 277 18.040 −8.887 −10.818 1.00 0.00 H
ATOM 4396 N ASN A 278 18.054 −5.883 −17.907 1.00 0.00 N
ATOM 4397 CA ASN A 278 18.405 −4.664 −18.644 1.00 0.00 C
ATOM 4398 C ASN A 278 19.880 −4.295 −18.407 1.00 0.00 C
ATOM 4399 O ASN A 278 20.185 −3.249 −17.843 1.00 0.00 O
ATOM 4400 CB ASN A 278 17.381 −3.558 −18.338 1.00 0.00 C
ATOM 4401 CG ASN A 278 15.995 −3.900 −18.867 1.00 0.00 C
ATOM 4402 OD1 ASN A 278 15.818 −4.607 −19.852 1.00 0.00 O
ATOM 4403 ND2 ASN A 278 14.959 −3.418 −18.221 1.00 0.00 N
ATOM 4404 H ASN A 278 17.696 −5.791 −16.970 1.00 0.00 H
ATOM 4405 HA ASN A 278 18.337 −4.861 −19.715 1.00 0.00 H
ATOM 4406 HB2 ASN A 278 17.339 −3.393 −17.262 1.00 0.00 H
ATOM 4407 HB3 ASN A 278 17.699 −2.629 −18.810 1.00 0.00 H
ATOM 4408 HD21 ASN A 278 15.089 −2.844 −17.409 1.00 0.00 H
ATOM 4409 HD22 ASN A 278 14.053 −3.684 −18.562 1.00 0.00 H
ATOM 4410 N ASN A 279 20.765 −5.264 −18.670 1.00 0.00 N
ATOM 4411 CA ASN A 279 22.193 −5.176 −18.399 1.00 0.00 C
ATOM 4412 C ASN A 279 22.821 −4.008 −19.176 1.00 0.00 C
ATOM 4413 O ASN A 279 22.593 −3.875 −20.378 1.00 0.00 O

ATOM 4414 CB ASN A 279 22.844 −6.532 −18.740 1.00 0.00 C
ATOM 4415 CG ASN A 279 24.335 −6.559 −18.440 1.00 0.00 C
ATOM 4416 OD1 ASN A 279 25.143 −5.925 −19.101 1.00 0.00 O
ATOM 4417 ND2 ASN A 279 24.755 −7.296 −17.440 1.00 0.00 N
ATOM 4418 H ASN A 279 20.426 −6.101 −19.127 1.00 0.00 H
ATOM 4419 HA ASN A 279 22.305 −4.981 −17.335 1.00 0.00 H
ATOM 4420 HB2 ASN A 279 22.351 −7.322 −18.176 1.00 0.00 H
ATOM 4421 HB3 ASN A 279 22.714 −6.741 −19.802 1.00 0.00 H
ATOM 4422 HD21 ASN A 279 24.094 −7.747 −16.838 1.00 0.00 H
ATOM 4423 HD22 ASN A 279 25.715 −7.166 −17.171 1.00 0.00 H
ATOM 4424 N SER A 280 23.633 −3.194 −18.499 1.00 0.00 N
ATOM 4425 CA SER A 280 24.206 −1.961 −19.055 1.00 0.00 C
ATOM 4426 C SER A 280 25.717 −2.041 −19.328 1.00 0.00 C
ATOM 4427 O SER A 280 26.358 −0.996 −19.424 1.00 0.00 O
ATOM 4428 CB SER A 280 23.839 −0.764 −18.164 1.00 0.00 C
ATOM 4429 OG SER A 280 24.672 −0.685 −17.025 1.00 0.00 O
ATOM 4430 H SER A 280 23.784 −3.386 −17.512 1.00 0.00 H
ATOM 4431 HA SER A 280 23.735 −1.770 −20.017 1.00 0.00 H
ATOM 4432 HB2 SER A 280 22.797 −0.850 −17.851 1.00 0.00 H
ATOM 4433 HB3 SER A 280 23.950 0.154 −18.743 1.00 0.00 H
ATOM 4434 HG SER A 280 25.550 −0.433 −17.341 1.00 0.00 H
ATOM 4435 N VAL A 281 26.322 −3.240 −19.299 1.00 0.00 N
ATOM 4436 CA VAL A 281 27.792 −3.401 −19.235 1.00 0.00 C
ATOM 4437 C VAL A 281 28.392 −4.503 −20.121 1.00 0.00 C
ATOM 4438 O VAL A 281 29.519 −4.331 −20.576 1.00 0.00 O
ATOM 4439 CB VAL A 281 28.276 −3.578 −17.780 1.00 0.00 C
ATOM 4440 CG1 VAL A 281 27.994 −2.347 −16.913 1.00 0.00 C
ATOM 4441 CG2 VAL A 281 27.683 −4.806 −17.074 1.00 0.00 C
ATOM 4442 H VAL A 281 25.741 −4.058 −19.142 1.00 0.00 H
ATOM 4443 HA VAL A 281 28.251 −2.481 −19.599 1.00 0.00 H

ATOM 4444 HB VAL A 281 29.357 −3.692 −17.815 1.00 0.00 H
ATOM 4445 HG11 VAL A 281 28.360 −1.451 −17.414 1.00 0.00 H
ATOM 4446 HG12 VAL A 281 28.502 −2.446 −15.956 1.00 0.00 H
ATOM 4447 HG13 VAL A 281 26.924 −2.249 −16.735 1.00 0.00 H
ATOM 4448 HG21 VAL A 281 26.599 −4.719 −17.009 1.00 0.00 H
ATOM 4449 HG22 VAL A 281 28.083 −4.887 −16.065 1.00 0.00 H
ATOM 4450 HG23 VAL A 281 27.938 −5.710 −17.618 1.00 0.00 H
ATOM 4451 N ILE A 282 27.653 −5.558 −20.483 1.00 0.00 N
ATOM 4452 CA ILE A 282 28.118 −6.590 −21.430 1.00 0.00 C
ATOM 4453 C ILE A 282 27.390 −6.438 −22.773 1.00 0.00 C
ATOM 4454 O ILE A 282 26.171 −6.586 −22.852 1.00 0.00 O
ATOM 4455 CB ILE A 282 27.965 −8.011 −20.837 1.00 0.00 C
ATOM 4456 CG1 ILE A 282 28.742 −8.247 −19.522 1.00 0.00 C
ATOM 4457 CG2 ILE A 282 28.343 −9.100 −21.863 1.00 0.00 C
ATOM 4458 CD1 ILE A 282 30.271 −8.168 −19.618 1.00 0.00 C
ATOM 4459 H ILE A 282 26.713 −5.645 −20.108 1.00 0.00 H
ATOM 4460 HA ILE A 282 29.181 −6.446 −21.624 1.00 0.00 H
ATOM 4461 HB ILE A 282 26.915 −8.137 −20.596 1.00 0.00 H
ATOM 4462 HG12 ILE A 282 28.406 −7.530 −18.777 1.00 0.00 H
ATOM 4463 HG13 ILE A 282 28.481 −9.236 −19.142 1.00 0.00 H
ATOM 4464 HD11 ILE A 282 30.700 −8.300 −18.625 1.00 0.00 H
ATOM 4465 HD12 ILE A 282 30.575 −7.199 −20.009 1.00 0.00 H
ATOM 4466 HD13 ILE A 282 30.652 −8.958 −20.262 1.00 0.00 H
ATOM 4467 HG21 ILE A 282 28.257 −10.086 −21.404 1.00 0.00 H
ATOM 4468 HG22 ILE A 282 27.678 −9.069 −22.724 1.00 0.00 H
ATOM 4469 HG23 ILE A 282 29.367 −8.960 −22.206 1.00 0.00 H
ATOM 4470 N SER A 283 28.164 −6.314 −23.856 1.00 0.00 N
ATOM 4471 CA SER A 283 27.695 −6.274 −25.249 1.00 0.00 C
ATOM 4472 C SER A 283 28.661 −7.033 −26.161 1.00 0.00 C
ATOM 4473 O SER A 283 29.875 −6.905 −25.995 1.00 0.00 O

ATOM 4474 CB SER A 283 27.566 −4.819 −25.716 1.00 0.00 C
ATOM 4475 OG SER A 283 27.142 −4.757 −27.068 1.00 0.00 O
ATOM 4476 H SER A 283 29.161 −6.257 −23.709 1.00 0.00 H
ATOM 4477 HA SER A 283 26.713 −6.743 −25.315 1.00 0.00 H
ATOM 4478 HB2 SER A 283 26.842 −4.304 −25.087 1.00 0.00 H
ATOM 4479 HB3 SER A 283 28.530 −4.315 −25.618 1.00 0.00 H
ATOM 4480 HG SER A 283 27.785 −5.220 −27.620 1.00 0.00 H
ATOM 4481 N ARG A 284 28.136 −7.689 −27.207 1.00 0.00 N
ATOM 4482 CA ARG A 284 28.914 −8.449 −28.215 1.00 0.00 C
ATOM 4483 C ARG A 284 29.420 −7.589 −29.397 1.00 0.00 C
ATOM 4484 O ARG A 284 28.713 −6.634 −29.742 1.00 0.00 O
ATOM 4485 CB ARG A 284 28.085 −9.670 −28.681 1.00 0.00 C
ATOM 4486 CG ARG A 284 27.147 −9.478 −29.889 1.00 0.00 C
ATOM 4487 CD ARG A 284 26.108 −8.354 −29.782 1.00 0.00 C
ATOM 4488 NE ARG A 284 25.513 −8.047 −31.097 1.00 0.00 N
ATOM 4489 CZ ARG A 284 25.760 −7.007 −31.873 1.00 0.00 C
ATOM 4490 NH1 ARG A 284 26.658 −6.110 −31.603 1.00 0.00 N
ATOM 4491 NH2 ARG A 284 25.081 −6.808 −32.956 1.00 0.00 N
ATOM 4492 H ARG A 284 27.124 −7.683 −27.287 1.00 0.00 H
ATOM 4493 HA ARG A 284 29.803 −8.833 −27.707 1.00 0.00 H
ATOM 4494 HB2 ARG A 284 28.788 −10.450 −28.966 1.00 0.00 H
ATOM 4495 HB3 ARG A 284 27.508 −10.060 −27.840 1.00 0.00 H
ATOM 4496 HG2 ARG A 284 26.617 −10.415 −30.066 1.00 0.00 H
ATOM 4497 HG3 ARG A 284 27.763 −9.298 −30.761 1.00 0.00 H
ATOM 4498 HD2 ARG A 284 25.325 −8.667 −29.097 1.00 0.00 H
ATOM 4499 HD3 ARG A 284 26.544 −7.456 −29.357 1.00 0.00 H
ATOM 4500 HE ARG A 284 24.695 −8.588 −31.360 1.00 0.00 H
ATOM 4501 HH11 ARG A 284 27.328 −6.265 −30.862 1.00 0.00 H
ATOM 4502 HH12 ARG A 284 26.705 −5.297 −32.204 1.00 0.00 H
ATOM 4503 HH21 ARG A 284 24.466 −7.528 −33.321 1.00 0.00 H

ATOM 4504 HH22 ARG A 284 25.262 −5.967 −33.488 1.00 0.00 H
ATOM 4505 N PRO A 285 30.507 −7.957 −30.109 1.00 0.00 N
ATOM 4506 CA PRO A 285 30.891 −7.349 −31.393 1.00 0.00 C
ATOM 4507 C PRO A 285 29.763 −7.421 −32.424 1.00 0.00 C
ATOM 4508 O PRO A 285 29.118 −8.457 −32.563 1.00 0.00 O
ATOM 4509 CB PRO A 285 32.114 −8.123 −31.905 1.00 0.00 C
ATOM 4510 CG PRO A 285 32.098 −9.419 −31.101 1.00 0.00 C
ATOM 4511 CD PRO A 285 31.452 −9.011 −29.779 1.00 0.00 C
ATOM 4512 HA PRO A 285 31.175 −6.309 −31.242 1.00 0.00 H
ATOM 4513 HB2 PRO A 285 32.062 −8.325 −32.977 1.00 0.00 H
ATOM 4514 HB3 PRO A 285 33.027 −7.574 −31.692 1.00 0.00 H
ATOM 4515 HG2 PRO A 285 31.467 −10.155 −31.602 1.00 0.00 H
ATOM 4516 HG3 PRO A 285 33.106 −9.808 −30.959 1.00 0.00 H
ATOM 4517 HD2 PRO A 285 32.214 −8.601 −29.113 1.00 0.00 H
ATOM 4518 HD3 PRO A 285 30.975 −9.875 −29.320 1.00 0.00 H
ATOM 4519 N GLY A 286 29.500 −6.314 −33.119 1.00 0.00 N
ATOM 4520 CA GLY A 286 28.614 −6.288 −34.289 1.00 0.00 C
ATOM 4521 C GLY A 286 29.406 −6.298 −35.591 1.00 0.00 C
ATOM 4522 O GLY A 286 30.530 −6.783 −35.618 1.00 0.00 O
ATOM 4523 H GLY A 286 30.129 −5.528 −32.998 1.00 0.00 H
ATOM 4524 HA2 GLY A 286 27.933 −7.140 −34.290 1.00 0.00 H
ATOM 4525 HA3 GLY A 286 28.023 −5.378 −34.263 1.00 0.00 H
ATOM 4526 N GLN A 287 28.922 −5.534 −36.565 1.00 0.00 N
ATOM 4527 CA GLN A 287 29.726 −4.814 −37.553 1.00 0.00 C
ATOM 4528 C GLN A 287 29.271 −3.339 −37.550 1.00 0.00 C
ATOM 4529 O GLN A 287 28.921 −2.813 −36.493 1.00 0.00 O
ATOM 4530 CB GLN A 287 29.648 −5.525 −38.920 1.00 0.00 C
ATOM 4531 CG GLN A 287 30.257 −6.936 −38.841 1.00 0.00 C
ATOM 4532 CD GLN A 287 30.789 −7.450 −40.172 1.00 0.00 C
ATOM 4533 OE1 GLN A 287 30.216 −7.275 −41.234 1.00 0.00 O

ATOM	4534	NE2	GLN	A	287	31.918	−8.122	−40.165	1.00	0.00	N
ATOM	4535	H	GLN	A	287	27.972	−5.177	−36.466	1.00	0.00	H
ATOM	4536	HA	GLN	A	287	30.770	−4.811	−37.238	1.00	0.00	H
ATOM	4537	HB2	GLN	A	287	30.224	−4.948	−39.644	1.00	0.00	H
ATOM	4538	HB3	GLN	A	287	28.613	−5.585	−39.261	1.00	0.00	H
ATOM	4539	HG2	GLN	A	287	31.097	−6.914	−38.148	1.00	0.00	H
ATOM	4540	HG3	GLN	A	287	29.519	−7.639	−38.454	1.00	0.00	H
ATOM	4541	HE21	GLN	A	287	32.434	−8.213	−39.305	1.00	0.00	H
ATOM	4542	HE22	GLN	A	287	32.272	−8.410	−41.056	1.00	0.00	H
ATOM	4543	N	SER	A	288	29.408	−2.608	−38.658	1.00	0.00	N
ATOM	4544	CA	SER	A	288	29.243	−1.145	−38.701	1.00	0.00	C
ATOM	4545	C	SER	A	288	27.787	−0.633	−38.694	1.00	0.00	C
ATOM	4546	O	SER	A	288	27.565	0.519	−39.060	1.00	0.00	O
ATOM	4547	CB	SER	A	288	30.008	−0.592	−39.908	1.00	0.00	C
ATOM	4548	OG	SER	A	288	29.399	−1.019	−41.112	1.00	0.00	O
ATOM	4549	H	SER	A	288	29.699	−3.063	−39.511	1.00	0.00	H
ATOM	4550	HA	SER	A	288	29.711	−0.733	−37.806	1.00	0.00	H
ATOM	4551	HB2	SER	A	288	30.015	0.498	−39.871	1.00	0.00	H
ATOM	4552	HB3	SER	A	288	31.039	−0.945	−39.874	1.00	0.00	H
ATOM	4553	HG	SER	A	288	28.564	−0.541	−41.193	1.00	0.00	H
ATOM	4554	N	GLN	A	289	26.794	−1.485	−38.414	1.00	0.00	N
ATOM	4555	CA	GLN	A	289	25.361	−1.137	−38.421	1.00	0.00	C
ATOM	4556	C	GLN	A	289	24.715	−1.346	−37.037	1.00	0.00	C
ATOM	4557	O	GLN	A	289	24.024	−0.459	−36.543	1.00	0.00	O
ATOM	4558	CB	GLN	A	289	24.688	−1.946	−39.547	1.00	0.00	C
ATOM	4559	CG	GLN	A	289	23.149	−1.946	−39.566	1.00	0.00	C
ATOM	4560	CD	GLN	A	289	22.488	−0.656	−40.041	1.00	0.00	C
ATOM	4561	OE1	GLN	A	289	23.092	0.389	−40.212	1.00	0.00	O
ATOM	4562	NE2	GLN	A	289	21.204	−0.705	−40.325	1.00	0.00	N
ATOM	4563	H	GLN	A	289	27.040	−2.430	−38.150	1.00	0.00	H

ATOM	4564	HA	GLN	A	289	25.237	−0.079	−38.661	1.00	0.00	H
ATOM	4565	HB2	GLN	A	289	25.054	−1.589	−40.511	1.00	0.00	H
ATOM	4566	HB3	GLN	A	289	25.007	−2.982	−39.450	1.00	0.00	H
ATOM	4567	HG2	GLN	A	289	22.769	−2.172	−38.573	1.00	0.00	H
ATOM	4568	HG3	GLN	A	289	22.827	−2.748	−40.231	1.00	0.00	H
ATOM	4569	HE21	GLN	A	289	20.720	−1.590	−40.283	1.00	0.00	H
ATOM	4570	HE22	GLN	A	289	20.749	0.175	−40.494	1.00	0.00	H
ATOM	4571	N	CYS	A	290	25.108	−2.403	−36.322	1.00	0.00	N
ATOM	4572	CA	CYS	A	290	24.735	−2.694	−34.935	1.00	0.00	C
ATOM	4573	C	CYS	A	290	25.978	−3.091	−34.092	1.00	0.00	C
ATOM	4574	O	CYS	A	290	26.039	−4.225	−33.598	1.00	0.00	O
ATOM	4575	CB	CYS	A	290	23.617	−3.757	−34.952	1.00	0.00	C
ATOM	4576	SG	CYS	A	290	22.037	−3.172	−35.631	1.00	0.00	S
ATOM	4577	H	CYS	A	290	25.664	−3.112	−36.797	1.00	0.00	H
ATOM	4578	HA	CYS	A	290	24.319	−1.796	−34.477	1.00	0.00	H
ATOM	4579	HB2	CYS	A	290	23.957	−4.601	−35.549	1.00	0.00	H
ATOM	4580	HB3	CYS	A	290	23.438	−4.109	−33.936	1.00	0.00	H
ATOM	4581	N	PRO	A	291	26.996	−2.210	−33.939	1.00	0.00	N
ATOM	4582	CA	PRO	A	291	28.217	−2.450	−33.146	1.00	0.00	C
ATOM	4583	C	PRO	A	291	27.943	−2.540	−31.630	1.00	0.00	C
ATOM	4584	O	PRO	A	291	26.814	−2.340	−31.186	1.00	0.00	O
ATOM	4585	CB	PRO	A	291	29.138	−1.266	−33.472	1.00	0.00	C
ATOM	4586	CG	PRO	A	291	28.155	−0.132	−33.747	1.00	0.00	C
ATOM	4587	CD	PRO	A	291	27.021	−0.852	−34.470	1.00	0.00	C
ATOM	4588	HA	PRO	A	291	28.701	−3.370	−33.471	1.00	0.00	H
ATOM	4589	HB2	PRO	A	291	29.820	−1.019	−32.658	1.00	0.00	H
ATOM	4590	HB3	PRO	A	291	29.705	−1.479	−34.378	1.00	0.00	H
ATOM	4591	HG2	PRO	A	291	28.599	0.648	−34.367	1.00	0.00	H
ATOM	4592	HG3	PRO	A	291	27.791	0.281	−32.804	1.00	0.00	H
ATOM	4593	HD2	PRO	A	291	26.078	−0.331	−34.299	1.00	0.00	H

ATOM 4594 HD3 PRO A 291 27.242 −0.885 −35.538 1.00 0.00 H
ATOM 4595 N ARG A 292 28.974 −2.818 −30.810 1.00 0.00 N
ATOM 4596 CA ARG A 292 28.834 −2.867 −29.334 1.00 0.00 C
ATOM 4597 C ARG A 292 28.155 −1.619 −28.757 1.00 0.00 C
ATOM 4598 O ARG A 292 28.538 −0.498 −29.079 1.00 0.00 O
ATOM 4599 CB ARG A 292 30.183 −3.090 −28.615 1.00 0.00 C
ATOM 4600 CG ARG A 292 30.550 −4.571 −28.567 1.00 0.00 C
ATOM 4601 CD ARG A 292 31.876 −4.925 −27.891 1.00 0.00 C
ATOM 4602 NE ARG A 292 31.867 −4.848 −26.423 1.00 0.00 N
ATOM 4603 CZ ARG A 292 32.892 −4.379 −25.733 1.00 0.00 C
ATOM 4604 NH1 ARG A 292 33.262 −4.972 −24.638 1.00 0.00 N
ATOM 4605 NH2 ARG A 292 33.638 −3.397 −26.146 1.00 0.00 N
ATOM 4606 H ARG A 292 29.881 −2.976 −31.229 1.00 0.00 H
ATOM 4607 HA ARG A 292 28.168 −3.697 −29.112 1.00 0.00 H
ATOM 4608 HB2 ARG A 292 30.111 −2.740 −27.586 1.00 0.00 H
ATOM 4609 HB3 ARG A 292 30.958 −2.517 −29.118 1.00 0.00 H
ATOM 4610 HG2 ARG A 292 29.759 −5.104 −28.048 1.00 0.00 H
ATOM 4611 HG3 ARG A 292 30.606 −4.923 −29.595 1.00 0.00 H
ATOM 4612 HD2 ARG A 292 32.087 −5.968 −28.133 1.00 0.00 H
ATOM 4613 HD3 ARG A 292 32.678 −4.326 −28.311 1.00 0.00 H
ATOM 4614 HE ARG A 292 31.346 −5.594 −25.980 1.00 0.00 H
ATOM 4615 HH11 ARG A 292 32.906 −5.888 −24.440 1.00 0.00 H
ATOM 4616 HH12 ARG A 292 34.176 −4.727 −24.261 1.00 0.00 H
ATOM 4617 HH21 ARG A 292 33.468 −3.022 −27.073 1.00 0.00 H
ATOM 4618 HH22 ARG A 292 34.607 −3.434 −25.862 1.00 0.00 H
ATOM 4619 N PHE A 293 27.319 −1.858 −27.746 1.00 0.00 N
ATOM 4620 CA PHE A 293 26.547 −0.867 −26.988 1.00 0.00 C
ATOM 4621 C PHE A 293 25.545 −0.047 −27.821 1.00 0.00 C
ATOM 4622 O PHE A 293 25.055 0.979 −27.347 1.00 0.00 O
ATOM 4623 CB PHE A 293 27.466 −0.017 −26.092 1.00 0.00 C

ATOM	4624	CG	PHE	A	293	28.371	−0.830	−25.183	1.00	0.00	C
ATOM	4625	CD1	PHE	A	293	27.847	−1.411	−24.014	1.00	0.00	C
ATOM	4626	CD2	PHE	A	293	29.730	−1.010	−25.502	1.00	0.00	C
ATOM	4627	CE1	PHE	A	293	28.674	−2.178	−23.174	1.00	0.00	C
ATOM	4628	CE2	PHE	A	293	30.557	−1.778	−24.661	1.00	0.00	C
ATOM	4629	CZ	PHE	A	293	30.027	−2.367	−23.499	1.00	0.00	C
ATOM	4630	H	PHE	A	293	27.116	−2.826	−27.544	1.00	0.00	H
ATOM	4631	HA	PHE	A	293	25.914	−1.441	−26.312	1.00	0.00	H
ATOM	4632	HB2	PHE	A	293	26.846	0.625	−25.464	1.00	0.00	H
ATOM	4633	HB3	PHE	A	293	28.073	0.638	−26.718	1.00	0.00	H
ATOM	4634	HD1	PHE	A	293	26.811	−1.252	−23.754	1.00	0.00	H
ATOM	4635	HD2	PHE	A	293	30.140	−0.552	−26.391	1.00	0.00	H
ATOM	4636	HE1	PHE	A	293	28.276	−2.603	−22.265	1.00	0.00	H
ATOM	4637	HE2	PHE	A	293	31.604	−1.898	−24.897	1.00	0.00	H
ATOM	4638	HZ	PHE	A	293	30.664	−2.941	−22.837	1.00	0.00	H
ATOM	4639	N	ASN	A	294	25.114	−0.550	−28.986	1.00	0.00	N
ATOM	4640	CA	ASN	A	294	23.877	−0.066	−29.589	1.00	0.00	C
ATOM	4641	C	ASN	A	294	22.645	−0.597	−28.831	1.00	0.00	C
ATOM	4642	O	ASN	A	294	22.563	−1.768	−28.463	1.00	0.00	O
ATOM	4643	CB	ASN	A	294	23.820	−0.390	−31.089	1.00	0.00	C
ATOM	4644	CG	ASN	A	294	22.542	0.156	−31.708	1.00	0.00	C
ATOM	4645	OD1	ASN	A	294	22.216	1.324	−31.546	1.00	0.00	O
ATOM	4646	ND2	ASN	A	294	21.611	−0.706	−32.032	1.00	0.00	N
ATOM	4647	H	ASN	A	294	25.459	−1.454	−29.284	1.00	0.00	H
ATOM	4648	HA	ASN	A	294	23.868	1.023	−29.502	1.00	0.00	H
ATOM	4649	HB2	ASN	A	294	24.668	0.067	−31.597	1.00	0.00	H
ATOM	4650	HB3	ASN	A	294	23.876	−1.464	−31.242	1.00	0.00	H
ATOM	4651	HD21	ASN	A	294	21.680	−1.666	−31.723	1.00	0.00	H
ATOM	4652	HD22	ASN	A	294	20.714	−0.331	−32.322	1.00	0.00	H
ATOM	4653	N	VAL	A	295	21.638	0.266	−28.711	1.00	0.00	N

ATOM	4654	CA	VAL	A	295	20.312	−0.046	−28.147	1.00	0.00	C
ATOM	4655	C	VAL	A	295	19.185	0.477	−29.057	1.00	0.00	C
ATOM	4656	O	VAL	A	295	18.021	0.120	−28.887	1.00	0.00	O
ATOM	4657	CB	VAL	A	295	20.211	0.502	−26.700	1.00	0.00	C
ATOM	4658	CG1	VAL	A	295	18.941	0.051	−25.965	1.00	0.00	C
ATOM	4659	CG2	VAL	A	295	21.400	0.070	−25.823	1.00	0.00	C
ATOM	4660	H	VAL	A	295	21.803	1.201	−29.053	1.00	0.00	H
ATOM	4661	HA	VAL	A	295	20.196	−1.130	−28.104	1.00	0.00	H
ATOM	4662	HB	VAL	A	295	20.209	1.592	−26.742	1.00	0.00	H
ATOM	4663	HG21	VAL	A	295	22.329	0.501	−26.197	1.00	0.00	H
ATOM	4664	HG22	VAL	A	295	21.489	−1.018	−25.825	1.00	0.00	H
ATOM	4665	HG23	VAL	A	295	21.265	0.419	−24.799	1.00	0.00	H
ATOM	4666	HG11	VAL	A	295	18.057	0.476	−26.437	1.00	0.00	H
ATOM	4667	HG12	VAL	A	295	18.871	−1.037	−25.979	1.00	0.00	H
ATOM	4668	HG13	VAL	A	295	18.963	0.396	−24.931	1.00	0.00	H
ATOM	4669	N	CYS	A	296	19.512	1.294	−30.066	1.00	0.00	N
ATOM	4670	CA	CYS	A	296	18.532	1.829	−31.004	1.00	0.00	C
ATOM	4671	C	CYS	A	296	18.097	0.776	−32.034	1.00	0.00	C
ATOM	4672	O	CYS	A	296	18.969	0.156	−32.645	1.00	0.00	O
ATOM	4673	CB	CYS	A	296	19.099	3.074	−31.688	1.00	0.00	C
ATOM	4674	SG	CYS	A	296	19.005	4.528	−30.616	1.00	0.00	S
ATOM	4675	H	CYS	A	296	20.487	1.419	−30.300	1.00	0.00	H
ATOM	4676	HA	CYS	A	296	17.668	2.139	−30.426	1.00	0.00	H
ATOM	4677	HB2	CYS	A	296	20.134	2.893	−31.983	1.00	0.00	H
ATOM	4678	HB3	CYS	A	296	18.525	3.278	−32.595	1.00	0.00	H
ATOM	4679	N	PRO	A	297	16.788	0.623	−32.317	1.00	0.00	N
ATOM	4680	CA	PRO	A	297	16.325	−0.359	−33.288	1.00	0.00	C
ATOM	4681	C	PRO	A	297	16.806	−0.068	−34.708	1.00	0.00	C
ATOM	4682	O	PRO	A	297	16.348	0.886	−35.332	1.00	0.00	O
ATOM	4683	CB	PRO	A	297	14.800	−0.382	−33.183	1.00	0.00	C

ATOM	4684	CG	PRO	A	297	14.533	0.135	−31.773	1.00	0.00	C
ATOM	4685	CD	PRO	A	297	15.657	1.141	−31.558	1.00	0.00	C
ATOM	4686	HA	PRO	A	297	16.704	−1.326	−32.984	1.00	0.00	H
ATOM	4687	HB2	PRO	A	297	14.349	0.302	−33.904	1.00	0.00	H
ATOM	4688	HB3	PRO	A	297	14.423	−1.393	−33.325	1.00	0.00	H
ATOM	4689	HG2	PRO	A	297	13.554	0.605	−31.688	1.00	0.00	H
ATOM	4690	HG3	PRO	A	297	14.636	−0.682	−31.060	1.00	0.00	H
ATOM	4691	HD2	PRO	A	297	15.868	1.204	−30.490	1.00	0.00	H
ATOM	4692	HD3	PRO	A	297	15.372	2.117	−31.950	1.00	0.00	H
ATOM	4693	N	GLU	A	298	17.544	−1.007	−35.297	1.00	0.00	N
ATOM	4694	CA	GLU	A	298	18.062	−0.888	−36.661	1.00	0.00	C
ATOM	4695	C	GLU	A	298	17.939	−2.165	−37.487	1.00	0.00	C
ATOM	4696	O	GLU	A	298	17.790	−3.278	−36.973	1.00	0.00	O
ATOM	4697	CB	GLU	A	298	19.510	−0.356	−36.672	1.00	0.00	C
ATOM	4698	CG	GLU	A	298	19.604	1.177	−36.590	1.00	0.00	C
ATOM	4699	CD	GLU	A	298	18.743	1.918	−37.633	1.00	0.00	C
ATOM	4700	OE1	GLU	A	298	18.409	1.311	−38.684	1.00	0.00	O
ATOM	4701	OE2	GLU	A	298	18.120	2.926	−37.224	1.00	0.00	O
ATOM	4702	H	GLU	A	298	17.859	−1.792	−34.747	1.00	0.00	H
ATOM	4703	HA	GLU	A	298	17.433	−0.179	−37.187	1.00	0.00	H
ATOM	4704	HB2	GLU	A	298	20.069	−0.800	−35.847	1.00	0.00	H
ATOM	4705	HB3	GLU	A	298	19.998	−0.664	−37.598	1.00	0.00	H
ATOM	4706	HG2	GLU	A	298	19.304	1.484	−35.585	1.00	0.00	H
ATOM	4707	HG3	GLU	A	298	20.648	1.466	−36.722	1.00	0.00	H
ATOM	4708	N	VAL	A	299	17.913	−1.979	−38.808	1.00	0.00	N
ATOM	4709	CA	VAL	A	299	17.830	−3.079	−39.773	1.00	0.00	C
ATOM	4710	C	VAL	A	299	19.177	−3.800	−39.835	1.00	0.00	C
ATOM	4711	O	VAL	A	299	20.052	−3.424	−40.612	1.00	0.00	O
ATOM	4712	CB	VAL	A	299	17.370	−2.587	−41.161	1.00	0.00	C
ATOM	4713	CG1	VAL	A	299	17.185	−3.761	−42.132	1.00	0.00	C

ATOM	4714	CG2	VAL	A	299	16.025	−1.855	−41.073	1.00	0.00	C
ATOM	4715	H	VAL	A	299	18.055	−1.030	−39.148	1.00	0.00	H
ATOM	4716	HA	VAL	A	299	17.085	−3.790	−39.415	1.00	0.00	H
ATOM	4717	HB	VAL	A	299	18.107	−1.898	−41.574	1.00	0.00	H
ATOM	4718	HG21	VAL	A	299	16.152	−0.932	−40.508	1.00	0.00	H
ATOM	4719	HG22	VAL	A	299	15.287	−2.480	−40.574	1.00	0.00	H
ATOM	4720	HG23	VAL	A	299	15.672	−1.588	−42.068	1.00	0.00	H
ATOM	4721	HG11	VAL	A	299	18.136	−4.269	−42.289	1.00	0.00	H
ATOM	4722	HG12	VAL	A	299	16.843	−3.382	−43.095	1.00	0.00	H
ATOM	4723	HG13	VAL	A	299	16.459	−4.467	−41.728	1.00	0.00	H
ATOM	4724	N	CYS	A	300	19.344	−4.827	−39.004	1.00	0.00	N
ATOM	4725	CA	CYS	A	300	20.543	−5.663	−38.949	1.00	0.00	C
ATOM	4726	C	CYS	A	300	20.222	−7.121	−38.584	1.00	0.00	C
ATOM	4727	O	CYS	A	300	19.201	−7.409	−37.948	1.00	0.00	O
ATOM	4728	CB	CYS	A	300	21.556	−5.042	−37.976	1.00	0.00	C
ATOM	4729	SG	CYS	A	300	21.033	−4.838	−36.252	1.00	0.00	S
ATOM	4730	H	CYS	A	300	18.661	−4.951	−38.269	1.00	0.00	H
ATOM	4731	HA	CYS	A	300	21.008	−5.672	−39.936	1.00	0.00	H
ATOM	4732	HB2	CYS	A	300	22.462	−5.644	−37.980	1.00	0.00	H
ATOM	4733	HB3	CYS	A	300	21.826	−4.063	−38.362	1.00	0.00	H
ATOM	4734	N	TRP	A	301	21.155	−8.009	−38.940	1.00	0.00	N
ATOM	4735	CA	TRP	A	301	21.322	−9.360	−38.400	1.00	0.00	C
ATOM	4736	C	TRP	A	301	22.824	−9.643	−38.283	1.00	0.00	C
ATOM	4737	O	TRP	A	301	23.486	−9.941	−39.274	1.00	0.00	O
ATOM	4738	CB	TRP	A	301	20.620	−10.421	−39.260	1.00	0.00	C
ATOM	4739	CG	TRP	A	301	20.896	−11.841	−38.840	1.00	0.00	C
ATOM	4740	CD1	TRP	A	301	20.707	−12.337	−37.595	1.00	0.00	C
ATOM	4741	CD2	TRP	A	301	21.484	−12.940	−39.606	1.00	0.00	C
ATOM	4742	NE1	TRP	A	301	21.059	−13.669	−37.556	1.00	0.00	N
ATOM	4743	CE2	TRP	A	301	21.547	−14.098	−38.771	1.00	0.00	C

ATOM 4744 CE3 TRP A 301 21.986 −13.083 −40.921 1.00 0.00 C
ATOM 4745 CZ2 TRP A 301 22.038 −15.331 −39.216 1.00 0.00 C
ATOM 4746 CZ3 TRP A 301 22.491 −14.317 −41.378 1.00 0.00 C
ATOM 4747 CH2 TRP A 301 22.510 −15.443 −40.535 1.00 0.00 C
ATOM 4748 H TRP A 301 21.952 −7.655 −39.452 1.00 0.00 H
ATOM 4749 HA TRP A 301 20.886 −9.402 −37.405 1.00 0.00 H
ATOM 4750 HB2 TRP A 301 19.544 −10.248 −39.221 1.00 0.00 H
ATOM 4751 HB3 TRP A 301 20.940 −10.301 −40.296 1.00 0.00 H
ATOM 4752 HD1 TRP A 301 20.323 −11.764 −36.761 1.00 0.00 H
ATOM 4753 HE3 TRP A 301 21.995 −12.218 −41.569 1.00 0.00 H
ATOM 4754 HZ2 TRP A 301 22.071 −16.182 −38.551 1.00 0.00 H
ATOM 4755 HZ3 TRP A 301 22.877 −14.399 −42.385 1.00 0.00 H
ATOM 4756 HH2 TRP A 301 22.905 −16.384 −40.892 1.00 0.00 H
ATOM 4757 HE1 TRP A 301 21.002 −14.237 −36.724 1.00 0.00 H
ATOM 4758 N GLU A 302 23.391 −9.299 −37.129 1.00 0.00 N
ATOM 4759 CA GLU A 302 24.831 −9.325 −36.871 1.00 0.00 C
ATOM 4760 C GLU A 302 25.118 −9.435 −35.369 1.00 0.00 C
ATOM 4761 O GLU A 302 24.330 −9.000 −34.522 1.00 0.00 O
ATOM 4762 CB GLU A 302 25.526 −8.089 −37.476 1.00 0.00 C
ATOM 4763 CG GLU A 302 25.227 −6.777 −36.738 1.00 0.00 C
ATOM 4764 CD GLU A 302 25.537 −5.563 −37.618 1.00 0.00 C
ATOM 4765 OE1 GLU A 302 24.724 −5.305 −38.528 1.00 0.00 O
ATOM 4766 OE2 GLU A 302 26.351 −4.724 −37.167 1.00 0.00 O
ATOM 4767 H GLU A 302 22.786 −9.091 −36.344 1.00 0.00 H
ATOM 4768 HA GLU A 302 25.246 −10.208 −37.359 1.00 0.00 H
ATOM 4769 HB2 GLU A 302 25.220 −7.997 −38.518 1.00 0.00 H
ATOM 4770 HB3 GLU A 302 26.604 −8.253 −37.464 1.00 0.00 H
ATOM 4771 HG2 GLU A 302 24.171 −6.748 −36.461 1.00 0.00 H
ATOM 4772 HG3 GLU A 302 25.815 −6.739 −35.817 1.00 0.00 H
ATOM 4773 N GLY A 303 26.263 −10.021 −35.039 1.00 0.00 N

ATOM 4774 CA GLY A 303 26.666 −10.348 −33.677 1.00 0.00 C
ATOM 4775 C GLY A 303 27.616 −11.544 −33.669 1.00 0.00 C
ATOM 4776 O GLY A 303 27.719 −12.250 −34.674 1.00 0.00 O
ATOM 4777 H GLY A 303 26.848 −10.393 −35.775 1.00 0.00 H
ATOM 4778 HA2 GLY A 303 27.147 −9.486 −33.229 1.00 0.00 H
ATOM 4779 HA3 GLY A 303 25.790 −10.601 −33.081 1.00 0.00 H
ATOM 4780 N THR A 304 28.075 −11.935 −32.480 1.00 0.00 N
ATOM 4781 CA THR A 304 28.517 −13.313 −32.224 1.00 0.00 C
ATOM 4782 C THR A 304 27.970 −13.847 −30.900 1.00 0.00 C
ATOM 4783 O THR A 304 27.530 −13.095 −30.025 1.00 0.00 O
ATOM 4784 CB THR A 304 30.037 −13.532 −32.336 1.00 0.00 C
ATOM 4785 OG1 THR A 304 30.704 −13.230 −31.140 1.00 0.00 O
ATOM 4786 CG2 THR A 304 30.745 −12.780 −33.460 1.00 0.00 C
ATOM 4787 H THR A 304 27.949 −11.332 −31.683 1.00 0.00 H
ATOM 4788 HA THR A 304 28.084 −13.941 −32.998 1.00 0.00 H
ATOM 4789 HB THR A 304 30.173 −14.597 −32.521 1.00 0.00 H
ATOM 4790 HG21 THR A 304 31.800 −13.058 −33.475 1.00 0.00 H
ATOM 4791 HG22 THR A 304 30.300 −13.047 −34.418 1.00 0.00 H
ATOM 4792 HG23 THR A 304 30.665 −11.704 −33.309 1.00 0.00 H
ATOM 4793 HG1 THR A 304 30.425 −12.319 −30.935 1.00 0.00 H
ATOM 4794 N TYR A 305 27.905 −15.172 −30.780 1.00 0.00 N
ATOM 4795 CA TYR A 305 27.410 −15.864 −29.593 1.00 0.00 C
ATOM 4796 C TYR A 305 28.416 −15.743 −28.428 1.00 0.00 C
ATOM 4797 O TYR A 305 29.510 −16.304 −28.493 1.00 0.00 O
ATOM 4798 CB TYR A 305 27.116 −17.312 −30.015 1.00 0.00 C
ATOM 4799 CG TYR A 305 26.492 −18.197 −28.959 1.00 0.00 C
ATOM 4800 CD1 TYR A 305 25.155 −18.618 −29.070 1.00 0.00 C
ATOM 4801 CD2 TYR A 305 27.265 −18.636 −27.877 1.00 0.00 C
ATOM 4802 CE1 TYR A 305 24.593 −19.475 −28.100 1.00 0.00 C
ATOM 4803 CE2 TYR A 305 26.712 −19.482 −26.906 1.00 0.00 C

ATOM 4804 CZ TYR A 305 25.368 −19.888 −26.996 1.00 0.00 C
ATOM 4805 OH TYR A 305 24.827 −20.648 −26.016 1.00 0.00 O
ATOM 4806 H TYR A 305 28.273 −15.733 −31.532 1.00 0.00 H
ATOM 4807 HA TYR A 305 26.472 −15.403 −29.282 1.00 0.00 H
ATOM 4808 HB2 TYR A 305 28.045 −17.775 −30.347 1.00 0.00 H
ATOM 4809 HB3 TYR A 305 26.451 −17.289 −30.880 1.00 0.00 H
ATOM 4810 HD1 TYR A 305 24.548 −18.254 −29.885 1.00 0.00 H
ATOM 4811 HD2 TYR A 305 28.289 −18.311 −27.771 1.00 0.00 H
ATOM 4812 HE1 TYR A 305 23.555 −19.776 −28.156 1.00 0.00 H
ATOM 4813 HE2 TYR A 305 27.315 −19.797 −26.068 1.00 0.00 H
ATOM 4814 HH TYR A 305 25.398 −20.641 −25.210 1.00 0.00 H
ATOM 4815 N ASN A 306 28.004 −15.100 −27.330 1.00 0.00 N
ATOM 4816 CA ASN A 306 28.809 −14.855 −26.126 1.00 0.00 C
ATOM 4817 C ASN A 306 27.963 −15.148 −24.872 1.00 0.00 C
ATOM 4818 O ASN A 306 27.151 −14.301 −24.499 1.00 0.00 O
ATOM 4819 CB ASN A 306 29.293 −13.388 −26.116 1.00 0.00 C
ATOM 4820 CG ASN A 306 30.307 −13.048 −27.189 1.00 0.00 C
ATOM 4821 OD1 ASN A 306 31.470 −12.831 −26.911 1.00 0.00 O
ATOM 4822 ND2 ASN A 306 29.876 −12.792 −28.399 1.00 0.00 N
ATOM 4823 H ASN A 306 27.062 −14.731 −27.310 1.00 0.00 H
ATOM 4824 HA ASN A 306 29.679 −15.507 −26.121 1.00 0.00 H
ATOM 4825 HB2 ASN A 306 28.439 −12.718 −26.214 1.00 0.00 H
ATOM 4826 HB3 ASN A 306 29.761 −13.189 −25.151 1.00 0.00 H
ATOM 4827 HD21 ASN A 306 28.911 −12.939 −28.650 1.00 0.00 H
ATOM 4828 HD22 ASN A 306 30.567 −12.678 −29.128 1.00 0.00 H
ATOM 4829 N ASP A 307 28.012 −16.376 −24.334 1.00 0.00 N
ATOM 4830 CA ASP A 307 27.130 −16.788 −23.224 1.00 0.00 C
ATOM 4831 C ASP A 307 27.815 −16.835 −21.845 1.00 0.00 C
ATOM 4832 O ASP A 307 29.041 −16.904 −21.711 1.00 0.00 O
ATOM 4833 CB ASP A 307 26.279 −18.029 −23.577 1.00 0.00 C

```
ATOM   4834  CG  ASP A 307      26.884 −19.413 −23.321  1.00  0.00           C
ATOM   4835  OD1 ASP A 307      27.427 −19.634 −22.221  1.00  0.00           O
ATOM   4836  OD2 ASP A 307      26.378 −20.368 −23.948  1.00  0.00           O
ATOM   4837  H   ASP A 307      28.786 −16.979 −24.582  1.00  0.00           H
ATOM   4838  HA  ASP A 307      26.382 −16.002 −23.115  1.00  0.00           H
ATOM   4839  HB2 ASP A 307      25.990 −17.961 −24.626  1.00  0.00           H
ATOM   4840  HB3 ASP A 307      25.359 −17.971 −22.993  1.00  0.00           H
ATOM   4841  N   ALA A 308      26.976 −16.691 −20.817  1.00  0.00           N
ATOM   4842  CA  ALA A 308      27.347 −16.546 −19.416  1.00  0.00           C
ATOM   4843  C   ALA A 308      26.333 −17.265 −18.512  1.00  0.00           C
ATOM   4844  O   ALA A 308      25.180 −17.486 −18.898  1.00  0.00           O
ATOM   4845  CB  ALA A 308      27.440 −15.049 −19.092  1.00  0.00           C
ATOM   4846  H   ALA A 308      25.987 −16.711 −21.012  1.00  0.00           H
ATOM   4847  HA  ALA A 308      28.323 −17.006 −19.256  1.00  0.00           H
ATOM   4848  HB1 ALA A 308      27.742 −14.912 −18.052  1.00  0.00           H
ATOM   4849  HB2 ALA A 308      28.178 −14.575 −19.741  1.00  0.00           H
ATOM   4850  HB3 ALA A 308      26.469 −14.576 −19.246  1.00  0.00           H
ATOM   4851  N   PHE A 309      26.745 −17.555 −17.280  1.00  0.00           N
ATOM   4852  CA  PHE A 309      26.056 −18.434 −16.338  1.00  0.00           C
ATOM   4853  C   PHE A 309      25.835 −17.743 −14.986  1.00  0.00           C
ATOM   4854  O   PHE A 309      26.763 −17.145 −14.444  1.00  0.00           O
ATOM   4855  CB  PHE A 309      26.902 −19.701 −16.195  1.00  0.00           C
ATOM   4856  CG  PHE A 309      26.203 −20.909 −15.605  1.00  0.00           C
ATOM   4857  CD1 PHE A 309      25.847 −21.983 −16.442  1.00  0.00           C
ATOM   4858  CD2 PHE A 309      25.968 −20.999 −14.220  1.00  0.00           C
ATOM   4859  CE1 PHE A 309      25.321 −23.166 −15.894  1.00  0.00           C
ATOM   4860  CE2 PHE A 309      25.403 −22.166 −13.677  1.00  0.00           C
ATOM   4861  CZ  PHE A 309      25.095 −23.255 −14.510  1.00  0.00           C
ATOM   4862  H   PHE A 309      27.704 −17.317 −17.048  1.00  0.00           H
ATOM   4863  HA  PHE A 309      25.096 −18.721 −16.751  1.00  0.00           H
```

ATOM 4864 HB2 PHE A 309 27.277 −19.980 −17.178 1.00 0.00 H
ATOM 4865 HB3 PHE A 309 27.773 −19.463 −15.587 1.00 0.00 H
ATOM 4866 HD1 PHE A 309 26.003 −21.913 −17.510 1.00 0.00 H
ATOM 4867 HD2 PHE A 309 26.243 −20.185 −13.565 1.00 0.00 H
ATOM 4868 HE1 PHE A 309 25.084 −24.003 −16.540 1.00 0.00 H
ATOM 4869 HE2 PHE A 309 25.230 −22.232 −12.612 1.00 0.00 H
ATOM 4870 HZ PHE A 309 24.698 −24.166 −14.085 1.00 0.00 H
ATOM 4871 N LEU A 310 24.639 −17.861 −14.404 1.00 0.00 N
ATOM 4872 CA LEU A 310 24.291 −17.278 −13.103 1.00 0.00 C
ATOM 4873 C LEU A 310 24.958 −18.041 −11.939 1.00 0.00 C
ATOM 4874 O LEU A 310 24.559 −19.159 −11.616 1.00 0.00 O
ATOM 4875 CB LEU A 310 22.757 −17.266 −13.002 1.00 0.00 C
ATOM 4876 CG LEU A 310 22.193 −16.566 −11.754 1.00 0.00 C
ATOM 4877 CD1 LEU A 310 22.444 −15.063 −11.800 1.00 0.00 C
ATOM 4878 CD2 LEU A 310 20.685 −16.796 −11.695 1.00 0.00 C
ATOM 4879 H LEU A 310 23.916 −18.369 −14.889 1.00 0.00 H
ATOM 4880 HA LEU A 310 24.640 −16.247 −13.086 1.00 0.00 H
ATOM 4881 HB2 LEU A 310 22.412 −18.299 −13.002 1.00 0.00 H
ATOM 4882 HB3 LEU A 310 22.348 −16.777 −13.888 1.00 0.00 H
ATOM 4883 HG LEU A 310 22.643 −16.980 −10.851 1.00 0.00 H
ATOM 4884 HD11 LEU A 310 21.924 −14.577 −10.978 1.00 0.00 H
ATOM 4885 HD12 LEU A 310 22.079 −14.657 −12.742 1.00 0.00 H
ATOM 4886 HD13 LEU A 310 23.505 −14.857 −11.706 1.00 0.00 H
ATOM 4887 HD21 LEU A 310 20.487 −17.860 −11.590 1.00 0.00 H
ATOM 4888 HD22 LEU A 310 20.257 −16.264 −10.849 1.00 0.00 H
ATOM 4889 HD23 LEU A 310 20.213 −16.429 −12.607 1.00 0.00 H
ATOM 4890 N ILE A 311 25.961 −17.432 −11.296 1.00 0.00 N
ATOM 4891 CA ILE A 311 26.731 −18.035 −10.188 1.00 0.00 C
ATOM 4892 C ILE A 311 26.261 −17.607 −8.787 1.00 0.00 C
ATOM 4893 O ILE A 311 26.547 −18.307 −7.819 1.00 0.00 O

ATOM 4894 CB ILE A 311 28.249 −17.820 −10.372 1.00 0.00 C
ATOM 4895 CG1 ILE A 311 28.657 −16.331 −10.332 1.00 0.00 C
ATOM 4896 CG2 ILE A 311 28.746 −18.514 −11.653 1.00 0.00 C
ATOM 4897 CD1 ILE A 311 30.156 −16.137 −10.082 1.00 0.00 C
ATOM 4898 H ILE A 311 26.187 −16.482 −11.564 1.00 0.00 H
ATOM 4899 HA ILE A 311 26.570 −19.113 −10.218 1.00 0.00 H
ATOM 4900 HB ILE A 311 28.735 −18.320 −9.531 1.00 0.00 H
ATOM 4901 HG12 ILE A 311 28.127 −15.829 −9.523 1.00 0.00 H
ATOM 4902 HG13 ILE A 311 28.383 −15.847 −11.270 1.00 0.00 H
ATOM 4903 HG21 ILE A 311 29.834 −18.521 −11.688 1.00 0.00 H
ATOM 4904 HG22 ILE A 311 28.396 −19.547 −11.675 1.00 0.00 H
ATOM 4905 HG23 ILE A 311 28.367 −17.999 −12.536 1.00 0.00 H
ATOM 4906 HD11 ILE A 311 30.372 −15.074 −9.982 1.00 0.00 H
ATOM 4907 HD12 ILE A 311 30.440 −16.641 −9.161 1.00 0.00 H
ATOM 4908 HD13 ILE A 311 30.739 −16.544 −10.905 1.00 0.00 H
ATOM 4909 N ASP A 312 25.427 −16.568 −8.688 1.00 0.00 N
ATOM 4910 CA ASP A 312 24.532 −16.325 −7.549 1.00 0.00 C
ATOM 4911 C ASP A 312 23.263 −15.601 −8.019 1.00 0.00 C
ATOM 4912 O ASP A 312 23.348 −14.541 −8.635 1.00 0.00 O
ATOM 4913 CB ASP A 312 25.229 −15.516 −6.443 1.00 0.00 C
ATOM 4914 CG ASP A 312 24.283 −15.203 −5.274 1.00 0.00 C
ATOM 4915 OD1 ASP A 312 24.347 −14.050 −4.794 1.00 0.00 O
ATOM 4916 OD2 ASP A 312 23.290 −15.943 −5.090 1.00 0.00 O
ATOM 4917 H ASP A 312 25.279 −16.001 −9.516 1.00 0.00 H
ATOM 4918 HA ASP A 312 24.240 −17.282 −7.117 1.00 0.00 H
ATOM 4919 HB2 ASP A 312 26.080 −16.084 −6.063 1.00 0.00 H
ATOM 4920 HB3 ASP A 312 25.605 −14.582 −6.866 1.00 0.00 H
ATOM 4921 N ARG A 313 22.081 −16.113 −7.654 1.00 0.00 N
ATOM 4922 CA ARG A 313 20.793 −15.452 −7.915 1.00 0.00 C
ATOM 4923 C ARG A 313 20.472 −14.332 −6.919 1.00 0.00 C

```
ATOM   4924  O   ARG A 313      19.787 −13.388  −7.299  1.00  0.00           O
ATOM   4925  CB  ARG A 313      19.680 −16.514  −7.971  1.00  0.00           C
ATOM   4926  CG  ARG A 313      18.327 −15.915  −8.402  1.00  0.00           C
ATOM   4927  CD  ARG A 313      17.270 −16.975  −8.732  1.00  0.00           C
ATOM   4928  NE  ARG A 313      17.539 −17.627 −10.024  1.00  0.00           N
ATOM   4929  CZ  ARG A 313      18.023 −18.831 −10.255  1.00  0.00           C
ATOM   4930  NH1 ARG A 313      18.361 −19.655  −9.309  1.00  0.00           N
ATOM   4931  NH2 ARG A 313      18.138 −19.257 −11.471  1.00  0.00           N
ATOM   4932  H   ARG A 313      22.112 −16.879  −6.994  1.00  0.00           H
ATOM   4933  HA  ARG A 313      20.846 −14.968  −8.892  1.00  0.00           H
ATOM   4934  HB2 ARG A 313      19.976 −17.280  −8.688  1.00  0.00           H
ATOM   4935  HB3 ARG A 313      19.570 −16.984  −6.992  1.00  0.00           H
ATOM   4936  HG2 ARG A 313      18.468 −15.281  −9.277  1.00  0.00           H
ATOM   4937  HG3 ARG A 313      17.940 −15.296  −7.592  1.00  0.00           H
ATOM   4938  HD2 ARG A 313      17.206 −17.702  −7.922  1.00  0.00           H
ATOM   4939  HD3 ARG A 313      16.304 −16.472  −8.810  1.00  0.00           H
ATOM   4940  HE  ARG A 313      17.274 −17.097 −10.861  1.00  0.00           H
ATOM   4941 HH11 ARG A 313      18.183 −19.434  −8.351  1.00  0.00           H
ATOM   4942 HH12 ARG A 313      18.696 −20.572  −9.578  1.00  0.00           H
ATOM   4943 HH21 ARG A 313      17.728 −18.651 −12.190  1.00  0.00           H
ATOM   4944 HH22 ARG A 313      18.273 −20.242 −11.664  1.00  0.00           H
ATOM   4945  N   LEU A 314      20.974 −14.388  −5.683  1.00  0.00           N
ATOM   4946  CA  LEU A 314      20.622 −13.433  −4.622  1.00  0.00           C
ATOM   4947  C   LEU A 314      21.212 −12.036  −4.872  1.00  0.00           C
ATOM   4948  O   LEU A 314      20.497 −11.042  −4.797  1.00  0.00           O
ATOM   4949  CB  LEU A 314      21.071 −13.986  −3.256  1.00  0.00           C
ATOM   4950  CG  LEU A 314      20.491 −15.365  −2.884  1.00  0.00           C
ATOM   4951  CD1 LEU A 314      21.051 −15.811  −1.532  1.00  0.00           C
ATOM   4952  CD2 LEU A 314      18.963 −15.344  −2.779  1.00  0.00           C
ATOM   4953  H   LEU A 314      21.657 −15.113  −5.465  1.00  0.00           H
```

ATOM 4954 HA LEU A 314 19.539 −13.308 −4.610 1.00 0.00 H
ATOM 4955 HB2 LEU A 314 20.789 −13.268 −2.485 1.00 0.00 H
ATOM 4956 HB3 LEU A 314 22.159 −14.063 −3.254 1.00 0.00 H
ATOM 4957 HG LEU A 314 20.778 −16.104 −3.631 1.00 0.00 H
ATOM 4958 HD11 LEU A 314 22.138 −15.869 −1.597 1.00 0.00 H
ATOM 4959 HD12 LEU A 314 20.664 −16.798 −1.281 1.00 0.00 H
ATOM 4960 HD13 LEU A 314 20.770 −15.101 −0.755 1.00 0.00 H
ATOM 4961 HD21 LEU A 314 18.604 −16.319 −2.449 1.00 0.00 H
ATOM 4962 HD22 LEU A 314 18.647 −14.583 −2.065 1.00 0.00 H
ATOM 4963 HD23 LEU A 314 18.526 −15.131 −3.753 1.00 0.00 H
ATOM 4964 N ASN A 315 22.463 −11.975 −5.331 1.00 0.00 N
ATOM 4965 CA ASN A 315 23.101 −10.761 −5.855 1.00 0.00 C
ATOM 4966 C ASN A 315 22.906 −10.579 −7.379 1.00 0.00 C
ATOM 4967 O ASN A 315 23.472 −9.645 −7.946 1.00 0.00 O
ATOM 4968 CB ASN A 315 24.593 −10.786 −5.468 1.00 0.00 C
ATOM 4969 CG ASN A 315 24.816 −10.698 −3.971 1.00 0.00 C
ATOM 4970 OD1 ASN A 315 25.105 −9.644 −3.434 1.00 0.00 O
ATOM 4971 ND2 ASN A 315 24.794 −11.802 −3.267 1.00 0.00 N
ATOM 4972 H ASN A 315 23.014 −12.832 −5.309 1.00 0.00 H
ATOM 4973 HA ASN A 315 22.645 −9.889 −5.380 1.00 0.00 H
ATOM 4974 HB2 ASN A 315 25.059 −11.695 −5.849 1.00 0.00 H
ATOM 4975 HB3 ASN A 315 25.095 −9.931 −5.915 1.00 0.00 H
ATOM 4976 HD21 ASN A 315 24.602 −12.690 −3.739 1.00 0.00 H
ATOM 4977 HD22 ASN A 315 24.938 −11.733 −2.282 1.00 0.00 H
ATOM 4978 N TRP A 316 22.248 −11.541 −8.040 1.00 0.00 N
ATOM 4979 CA TRP A 316 22.129 −11.727 −9.497 1.00 0.00 C
ATOM 4980 C TRP A 316 23.455 −11.548 −10.268 1.00 0.00 C
ATOM 4981 O TRP A 316 23.581 −10.745 −11.195 1.00 0.00 O
ATOM 4982 CB TRP A 316 20.889 −11.013 −10.071 1.00 0.00 C
ATOM 4983 CG TRP A 316 20.049 −11.914 −10.935 1.00 0.00 C

ATOM	4984	CD1	TRP	A	316	18.989	−12.637	−10.500	1.00	0.00	C
ATOM	4985	CD2	TRP	A	316	20.267	−12.337	−12.320	1.00	0.00	C
ATOM	4986	NE1	TRP	A	316	18.524	−13.452	−11.516	1.00	0.00	N
ATOM	4987	CE2	TRP	A	316	19.303	−13.340	−12.645	1.00	0.00	C
ATOM	4988	CE3	TRP	A	316	21.202	−12.009	−13.328	1.00	0.00	C
ATOM	4989	CZ2	TRP	A	316	19.284	−14.005	−13.878	1.00	0.00	C
ATOM	4990	CZ3	TRP	A	316	21.202	−12.680	−14.568	1.00	0.00	C
ATOM	4991	CH2	TRP	A	316	20.251	−13.681	−14.844	1.00	0.00	C
ATOM	4992	H	TRP	A	316	21.792	−12.237	−7.469	1.00	0.00	H
ATOM	4993	HA	TRP	A	316	21.909	−12.786	−9.612	1.00	0.00	H
ATOM	4994	HB2	TRP	A	316	20.263	−10.664	−9.248	1.00	0.00	H
ATOM	4995	HB3	TRP	A	316	21.187	−10.133	−10.642	1.00	0.00	H
ATOM	4996	HD1	TRP	A	316	18.606	−12.621	−9.485	1.00	0.00	H
ATOM	4997	HE3	TRP	A	316	21.938	−11.242	−13.128	1.00	0.00	H
ATOM	4998	HZ2	TRP	A	316	18.562	−14.789	−14.058	1.00	0.00	H
ATOM	4999	HZ3	TRP	A	316	21.949	−12.434	−15.309	1.00	0.00	H
ATOM	5000	HH2	TRP	A	316	20.274	−14.211	−15.786	1.00	0.00	H
ATOM	5001	HE1	TRP	A	316	17.759	−14.133	−11.416	1.00	0.00	H
ATOM	5002	N	VAL	A	317	24.496	−12.241	−9.793	1.00	0.00	N
ATOM	5003	CA	VAL	A	317	25.847	−12.242	−10.370	1.00	0.00	C
ATOM	5004	C	VAL	A	317	26.014	−13.417	−11.335	1.00	0.00	C
ATOM	5005	O	VAL	A	317	25.909	−14.580	−10.944	1.00	0.00	O
ATOM	5006	CB	VAL	A	317	26.925	−12.256	−9.269	1.00	0.00	C
ATOM	5007	CG1	VAL	A	317	28.345	−12.373	−9.836	1.00	0.00	C
ATOM	5008	CG2	VAL	A	317	26.877	−10.958	−8.453	1.00	0.00	C
ATOM	5009	H	VAL	A	317	24.279	−12.953	−9.103	1.00	0.00	H
ATOM	5010	HA	VAL	A	317	25.978	−11.323	−10.937	1.00	0.00	H
ATOM	5011	HB	VAL	A	317	26.748	−13.100	−8.599	1.00	0.00	H
ATOM	5012	HG11	VAL	A	317	29.073	−12.322	−9.027	1.00	0.00	H
ATOM	5013	HG12	VAL	A	317	28.531	−11.568	−10.547	1.00	0.00	H

ATOM 5014 HG13 VAL A 317 28.471 −13.329 −10.343 1.00 0.00 H
ATOM 5015 HG21 VAL A 317 25.888 −10.835 −8.020 1.00 0.00 H
ATOM 5016 HG22 VAL A 317 27.609 −11.000 −7.646 1.00 0.00 H
ATOM 5017 HG23 VAL A 317 27.087 −10.108 −9.100 1.00 0.00 H
ATOM 5018 N SER A 318 26.391 −13.112 −12.574 1.00 0.00 N
ATOM 5019 CA SER A 318 26.748 −14.075 −13.622 1.00 0.00 C
ATOM 5020 C SER A 318 28.250 −14.068 −13.936 1.00 0.00 C
ATOM 5021 O SER A 318 28.964 −13.116 −13.618 1.00 0.00 O
ATOM 5022 CB SER A 318 25.935 −13.795 −14.889 1.00 0.00 C
ATOM 5023 OG SER A 318 24.554 −13.974 −14.634 1.00 0.00 O
ATOM 5024 H SER A 318 26.569 −12.133 −12.774 1.00 0.00 H
ATOM 5025 HA SER A 318 26.495 −15.074 −13.282 1.00 0.00 H
ATOM 5026 HB2 SER A 318 26.115 −12.772 −15.216 1.00 0.00 H
ATOM 5027 HB3 SER A 318 26.244 −14.477 −15.683 1.00 0.00 H
ATOM 5028 HG SER A 318 24.279 −13.346 −13.956 1.00 0.00 H
ATOM 5029 N ALA A 319 28.724 −15.114 −14.614 1.00 0.00 N
ATOM 5030 CA ALA A 319 30.107 −15.283 −15.054 1.00 0.00 C
ATOM 5031 C ALA A 319 30.175 −15.861 −16.477 1.00 0.00 C
ATOM 5032 O ALA A 319 29.405 −16.760 −16.813 1.00 0.00 O
ATOM 5033 CB ALA A 319 30.824 −16.187 −14.049 1.00 0.00 C
ATOM 5034 H ALA A 319 28.091 −15.888 −14.793 1.00 0.00 H
ATOM 5035 HA ALA A 319 30.595 −14.310 −15.059 1.00 0.00 H
ATOM 5036 HB1 ALA A 319 31.875 −16.269 −14.323 1.00 0.00 H
ATOM 5037 HB2 ALA A 319 30.375 −17.181 −14.055 1.00 0.00 H
ATOM 5038 HB3 ALA A 319 30.744 −15.758 −13.053 1.00 0.00 H
ATOM 5039 N GLY A 320 31.096 −15.380 −17.312 1.00 0.00 N
ATOM 5040 CA GLY A 320 31.263 −15.879 −18.680 1.00 0.00 C
ATOM 5041 C GLY A 320 32.318 −15.132 −19.488 1.00 0.00 C
ATOM 5042 O GLY A 320 32.798 −14.077 −19.073 1.00 0.00 O
ATOM 5043 H GLY A 320 31.707 −14.629 −17.000 1.00 0.00 H

ATOM	5044	HA2	GLY	A	320	31.556	−16.925	−18.641	1.00	0.00	H
ATOM	5045	HA3	GLY	A	320	30.318	−15.813	−19.218	1.00	0.00	H
ATOM	5046	N	VAL	A	321	32.692	−15.693	−20.637	1.00	0.00	N
ATOM	5047	CA	VAL	A	321	33.604	−15.043	−21.593	1.00	0.00	C
ATOM	5048	C	VAL	A	321	32.809	−14.192	−22.578	1.00	0.00	C
ATOM	5049	O	VAL	A	321	31.868	−14.695	−23.201	1.00	0.00	O
ATOM	5050	CB	VAL	A	321	34.511	−16.048	−22.328	1.00	0.00	C
ATOM	5051	CG1	VAL	A	321	35.590	−15.364	−23.183	1.00	0.00	C
ATOM	5052	CG2	VAL	A	321	35.238	−16.966	−21.348	1.00	0.00	C
ATOM	5053	H	VAL	A	321	32.219	−16.537	−20.930	1.00	0.00	H
ATOM	5054	HA	VAL	A	321	34.255	−14.381	−21.035	1.00	0.00	H
ATOM	5055	HB	VAL	A	321	33.895	−16.673	−22.967	1.00	0.00	H
ATOM	5056	HG11	VAL	A	321	36.272	−14.798	−22.549	1.00	0.00	H
ATOM	5057	HG12	VAL	A	321	36.163	−16.114	−23.729	1.00	0.00	H
ATOM	5058	HG13	VAL	A	321	35.148	−14.684	−23.911	1.00	0.00	H
ATOM	5059	HG21	VAL	A	321	34.521	−17.512	−20.740	1.00	0.00	H
ATOM	5060	HG22	VAL	A	321	35.879	−16.369	−20.701	1.00	0.00	H
ATOM	5061	HG23	VAL	A	321	35.837	−17.688	−21.898	1.00	0.00	H
ATOM	5062	N	TYR	A	322	33.285	−12.973	−22.831	1.00	0.00	N
ATOM	5063	CA	TYR	A	322	32.801	−12.090	−23.892	1.00	0.00	C
ATOM	5064	C	TYR	A	322	33.961	−11.477	−24.684	1.00	0.00	C
ATOM	5065	O	TYR	A	322	35.053	−11.266	−24.156	1.00	0.00	O
ATOM	5066	CB	TYR	A	322	31.830	−11.023	−23.343	1.00	0.00	C
ATOM	5067	CG	TYR	A	322	32.426	−9.796	−22.654	1.00	0.00	C
ATOM	5068	CD1	TYR	A	322	32.019	−8.500	−23.043	1.00	0.00	C
ATOM	5069	CD2	TYR	A	322	33.328	−9.940	−21.579	1.00	0.00	C
ATOM	5070	CE1	TYR	A	322	32.497	−7.366	−22.349	1.00	0.00	C
ATOM	5071	CE2	TYR	A	322	33.848	−8.809	−20.924	1.00	0.00	C
ATOM	5072	CZ	TYR	A	322	33.426	−7.519	−21.299	1.00	0.00	C
ATOM	5073	OH	TYR	A	322	33.890	−6.429	−20.637	1.00	0.00	O

ATOM 5074 H TYR A 322 34.122 −12.672 −22.337 1.00 0.00 H
ATOM 5075 HA TYR A 322 32.235 −12.703 −24.590 1.00 0.00 H
ATOM 5076 HB2 TYR A 322 31.139 −11.504 −22.648 1.00 0.00 H
ATOM 5077 HB3 TYR A 322 31.233 −10.676 −24.187 1.00 0.00 H
ATOM 5078 HD1 TYR A 322 31.322 −8.380 −23.861 1.00 0.00 H
ATOM 5079 HD2 TYR A 322 33.638 −10.920 −21.256 1.00 0.00 H
ATOM 5080 HE1 TYR A 322 32.151 −6.374 −22.587 1.00 0.00 H
ATOM 5081 HE2 TYR A 322 34.568 −8.927 −20.131 1.00 0.00 H
ATOM 5082 HH TYR A 322 33.796 −6.534 −19.674 1.00 0.00 H
ATOM 5083 N LEU A 323 33.725 −11.244 −25.973 1.00 0.00 N
ATOM 5084 CA LEU A 323 34.641 −10.527 −26.856 1.00 0.00 C
ATOM 5085 C LEU A 323 34.562 −9.017 −26.600 1.00 0.00 C
ATOM 5086 O LEU A 323 33.475 −8.427 −26.584 1.00 0.00 O
ATOM 5087 CB LEU A 323 34.310 −10.854 −28.318 1.00 0.00 C
ATOM 5088 CG LEU A 323 34.385 −12.346 −28.676 1.00 0.00 C
ATOM 5089 CD1 LEU A 323 33.882 −12.540 −30.103 1.00 0.00 C
ATOM 5090 CD2 LEU A 323 35.795 −12.909 −28.553 1.00 0.00 C
ATOM 5091 H LEU A 323 32.838 −11.545 −26.359 1.00 0.00 H
ATOM 5092 HA LEU A 323 35.662 −10.849 −26.651 1.00 0.00 H
ATOM 5093 HB2 LEU A 323 34.987 −10.298 −28.969 1.00 0.00 H
ATOM 5094 HB3 LEU A 323 33.296 −10.515 −28.509 1.00 0.00 H
ATOM 5095 HG LEU A 323 33.739 −12.921 −28.019 1.00 0.00 H
ATOM 5096 HD21 LEU A 323 35.778 −13.958 −28.836 1.00 0.00 H
ATOM 5097 HD22 LEU A 323 36.472 −12.364 −29.212 1.00 0.00 H
ATOM 5098 HD23 LEU A 323 36.148 −12.830 −27.527 1.00 0.00 H
ATOM 5099 HD11 LEU A 323 33.888 −13.599 −30.349 1.00 0.00 H
ATOM 5100 HD12 LEU A 323 34.535 −12.010 −30.798 1.00 0.00 H
ATOM 5101 HD13 LEU A 323 32.871 −12.153 −30.190 1.00 0.00 H
ATOM 5102 N ASN A 324 35.718 −8.384 −26.424 1.00 0.00 N
ATOM 5103 CA ASN A 324 35.811 −6.960 −26.108 1.00 0.00 C

ATOM 5104 C ASN A 324 35.888 −6.051 −27.352 1.00 0.00 C
ATOM 5105 O ASN A 324 35.729 −4.837 −27.229 1.00 0.00 O
ATOM 5106 CB ASN A 324 37.016 −6.773 −25.185 1.00 0.00 C
ATOM 5107 CG ASN A 324 37.028 −5.506 −24.360 1.00 0.00 C
ATOM 5108 OD1 ASN A 324 36.033 −4.807 −24.179 1.00 0.00 O
ATOM 5109 ND2 ASN A 324 38.176 −5.208 −23.803 1.00 0.00 N
ATOM 5110 H ASN A 324 36.569 −8.943 −26.393 1.00 0.00 H
ATOM 5111 HA ASN A 324 34.917 −6.684 −25.558 1.00 0.00 H
ATOM 5112 HB2 ASN A 324 37.898 −6.745 −25.810 1.00 0.00 H
ATOM 5113 HB3 ASN A 324 37.100 −7.616 −24.496 1.00 0.00 H
ATOM 5114 HD21 ASN A 324 38.968 −5.823 −23.976 1.00 0.00 H
ATOM 5115 HD22 ASN A 324 38.264 −4.352 −23.294 1.00 0.00 H
ATOM 5116 N SER A 325 36.090 −6.606 −28.549 1.00 0.00 N
ATOM 5117 CA SER A 325 36.057 −5.843 −29.804 1.00 0.00 C
ATOM 5118 C SER A 325 34.652 −5.349 −30.169 1.00 0.00 C
ATOM 5119 O SER A 325 33.654 −6.021 −29.922 1.00 0.00 O
ATOM 5120 CB SER A 325 36.611 −6.699 −30.929 1.00 0.00 C
ATOM 5121 OG SER A 325 36.541 −6.004 −32.157 1.00 0.00 O
ATOM 5122 H SER A 325 36.261 −7.605 −28.599 1.00 0.00 H
ATOM 5123 HA SER A 325 36.709 −4.976 −29.696 1.00 0.00 H
ATOM 5124 HB2 SER A 325 36.047 −7.629 −30.998 1.00 0.00 H
ATOM 5125 HB3 SER A 325 37.654 −6.936 −30.708 1.00 0.00 H
ATOM 5126 HG SER A 325 37.182 −6.512 −32.725 1.00 0.00 H
ATOM 5127 N ASN A 326 34.546 −4.165 −30.784 1.00 0.00 N
ATOM 5128 CA ASN A 326 33.255 −3.555 −31.132 1.00 0.00 C
ATOM 5129 C ASN A 326 32.624 −4.069 −32.442 1.00 0.00 C
ATOM 5130 O ASN A 326 31.402 −3.958 −32.585 1.00 0.00 O
ATOM 5131 CB ASN A 326 33.392 −2.019 −31.127 1.00 0.00 C
ATOM 5132 CG ASN A 326 33.468 −1.402 −29.735 1.00 0.00 C
ATOM 5133 OD1 ASN A 326 33.467 −2.061 −28.707 1.00 0.00 O

ATOM	5134	ND2	ASN	A	326	33.310	−0.103	−29.649	1.00	0.00	N
ATOM	5135	H	ASN	A	326	35.405	−3.686	−31.009	1.00	0.00	H
ATOM	5136	HA	ASN	A	326	32.536	−3.818	−30.362	1.00	0.00	H
ATOM	5137	HB2	ASN	A	326	34.272	−1.722	−31.697	1.00	0.00	H
ATOM	5138	HB3	ASN	A	326	32.517	−1.593	−31.619	1.00	0.00	H
ATOM	5139	HD21	ASN	A	326	33.211	0.461	−30.473	1.00	0.00	H
ATOM	5140	HD22	ASN	A	326	33.266	0.283	−28.722	1.00	0.00	H
ATOM	5141	N	GLN	A	327	33.425	−4.572	−33.391	1.00	0.00	N
ATOM	5142	CA	GLN	A	327	33.011	−4.841	−34.786	1.00	0.00	C
ATOM	5143	C	GLN	A	327	33.590	−6.134	−35.398	1.00	0.00	C
ATOM	5144	O	GLN	A	327	33.304	−6.448	−36.556	1.00	0.00	O
ATOM	5145	CB	GLN	A	327	33.422	−3.654	−35.677	1.00	0.00	C
ATOM	5146	CG	GLN	A	327	32.649	−2.357	−35.397	1.00	0.00	C
ATOM	5147	CD	GLN	A	327	32.961	−1.259	−36.413	1.00	0.00	C
ATOM	5148	OE1	GLN	A	327	32.090	−0.560	−36.898	1.00	0.00	O
ATOM	5149	NE2	GLN	A	327	34.198	−1.089	−36.829	1.00	0.00	N
ATOM	5150	H	GLN	A	327	34.408	−4.677	−33.169	1.00	0.00	H
ATOM	5151	HA	GLN	A	327	31.929	−4.939	−34.826	1.00	0.00	H
ATOM	5152	HB2	GLN	A	327	33.239	−3.917	−36.720	1.00	0.00	H
ATOM	5153	HB3	GLN	A	327	34.494	−3.481	−35.557	1.00	0.00	H
ATOM	5154	HG2	GLN	A	327	31.580	−2.569	−35.432	1.00	0.00	H
ATOM	5155	HG3	GLN	A	327	32.905	−1.988	−34.406	1.00	0.00	H
ATOM	5156	HE21	GLN	A	327	34.936	−1.695	−36.514	1.00	0.00	H
ATOM	5157	HE22	GLN	A	327	34.333	−0.395	−37.541	1.00	0.00	H
ATOM	5158	N	THR	A	328	34.497	−6.803	−34.691	1.00	0.00	N
ATOM	5159	CA	THR	A	328	35.325	−7.900	−35.209	1.00	0.00	C
ATOM	5160	C	THR	A	328	35.672	−8.890	−34.084	1.00	0.00	C
ATOM	5161	O	THR	A	328	35.068	−8.845	−33.011	1.00	0.00	O
ATOM	5162	CB	THR	A	328	36.577	−7.358	−35.941	1.00	0.00	C
ATOM	5163	OG1	THR	A	328	37.126	−6.235	−35.297	1.00	0.00	O

ATOM 5164 CG2 THR A 328 36.316 −6.944 −37.387 1.00 0.00 C
ATOM 5165 H THR A 328 34.739 −6.488 −33.763 1.00 0.00 H
ATOM 5166 HA THR A 328 34.744 −8.466 −35.935 1.00 0.00 H
ATOM 5167 HB THR A 328 37.319 −8.150 −35.984 1.00 0.00 H
ATOM 5168 HG1 THR A 328 37.874 −6.609 −34.779 1.00 0.00 H
ATOM 5169 HG21 THR A 328 37.270 −6.697 −37.854 1.00 0.00 H
ATOM 5170 HG22 THR A 328 35.856 −7.768 −37.930 1.00 0.00 H
ATOM 5171 HG23 THR A 328 35.672 −6.067 −37.421 1.00 0.00 H
ATOM 5172 N ALA A 329 36.316 −9.995 −34.461 1.00 0.00 N
ATOM 5173 CA ALA A 329 36.389 −11.229 −33.681 1.00 0.00 C
ATOM 5174 C ALA A 329 37.645 −11.292 −32.785 1.00 0.00 C
ATOM 5175 O ALA A 329 38.484 −12.167 −32.979 1.00 0.00 O
ATOM 5176 CB ALA A 329 36.272 −12.399 −34.675 1.00 0.00 C
ATOM 5177 H ALA A 329 36.842 −9.969 −35.320 1.00 0.00 H
ATOM 5178 HA ALA A 329 35.531 −11.283 −33.010 1.00 0.00 H
ATOM 5179 HB1 ALA A 329 35.338 −12.319 −35.232 1.00 0.00 H
ATOM 5180 HB2 ALA A 329 36.278 −13.345 −34.137 1.00 0.00 H
ATOM 5181 HB3 ALA A 329 37.115 −12.386 −35.367 1.00 0.00 H
ATOM 5182 N GLU A 330 37.824 −10.332 −31.869 1.00 0.00 N
ATOM 5183 CA GLU A 330 39.035 −10.212 −31.032 1.00 0.00 C
ATOM 5184 C GLU A 330 38.749 −10.050 −29.523 1.00 0.00 C
ATOM 5185 O GLU A 330 37.688 −9.559 −29.119 1.00 0.00 O
ATOM 5186 CB GLU A 330 39.955 −9.064 −31.507 1.00 0.00 C
ATOM 5187 CG GLU A 330 40.225 −8.983 −33.022 1.00 0.00 C
ATOM 5188 CD GLU A 330 39.281 −8.040 −33.784 1.00 0.00 C
ATOM 5189 OE1 GLU A 330 39.478 −7.827 −34.997 1.00 0.00 O
ATOM 5190 OE2 GLU A 330 38.243 −7.589 −33.244 1.00 0.00 O
ATOM 5191 H GLU A 330 37.153 −9.578 −31.830 1.00 0.00 H
ATOM 5192 HA GLU A 330 39.615 −11.128 −31.137 1.00 0.00 H
ATOM 5193 HB2 GLU A 330 39.569 −8.110 −31.150 1.00 0.00 H

ATOM	5194	HB3	GLU	A	330	40.919	−9.206	−31.017	1.00	0.00	H
ATOM	5195	HG2	GLU	A	330	41.243	−8.610	−33.155	1.00	0.00	H
ATOM	5196	HG3	GLU	A	330	40.188	−9.983	−33.457	1.00	0.00	H
ATOM	5197	N	ASN	A	331	39.809	−10.259	−28.725	1.00	0.00	N
ATOM	5198	CA	ASN	A	331	39.940	−9.916	−27.299	1.00	0.00	C
ATOM	5199	C	ASN	A	331	38.999	−10.724	−26.363	1.00	0.00	C
ATOM	5200	O	ASN	A	331	37.965	−10.212	−25.917	1.00	0.00	O
ATOM	5201	CB	ASN	A	331	39.849	−8.384	−27.158	1.00	0.00	C
ATOM	5202	CG	ASN	A	331	40.426	−7.858	−25.852	1.00	0.00	C
ATOM	5203	OD1	ASN	A	331	39.807	−7.889	−24.801	1.00	0.00	O
ATOM	5204	ND2	ASN	A	331	41.493	−7.110	−25.945	1.00	0.00	N
ATOM	5205	H	ASN	A	331	40.633	−10.655	−29.168	1.00	0.00	H
ATOM	5206	HA	ASN	A	331	40.961	−10.178	−27.013	1.00	0.00	H
ATOM	5207	HB2	ASN	A	331	38.812	−8.078	−27.247	1.00	0.00	H
ATOM	5208	HB3	ASN	A	331	40.389	−7.924	−27.985	1.00	0.00	H
ATOM	5209	HD21	ASN	A	331	42.014	−7.151	−26.815	1.00	0.00	H
ATOM	5210	HD22	ASN	A	331	42.020	−6.945	−25.080	1.00	0.00	H
ATOM	5211	N	PRO	A	332	39.288	−12.022	−26.120	1.00	0.00	N
ATOM	5212	CA	PRO	A	332	38.471	−12.911	−25.289	1.00	0.00	C
ATOM	5213	C	PRO	A	332	38.709	−12.694	−23.781	1.00	0.00	C
ATOM	5214	O	PRO	A	332	39.639	−13.249	−23.188	1.00	0.00	O
ATOM	5215	CB	PRO	A	332	38.855	−14.324	−25.747	1.00	0.00	C
ATOM	5216	CG	PRO	A	332	40.321	−14.183	−26.163	1.00	0.00	C
ATOM	5217	CD	PRO	A	332	40.397	−12.762	−26.717	1.00	0.00	C
ATOM	5218	HA	PRO	A	332	37.413	−12.747	−25.500	1.00	0.00	H
ATOM	5219	HB2	PRO	A	332	38.724	−15.068	−24.960	1.00	0.00	H
ATOM	5220	HB3	PRO	A	332	38.257	−14.593	−26.618	1.00	0.00	H
ATOM	5221	HG2	PRO	A	332	40.597	−14.913	−26.922	1.00	0.00	H
ATOM	5222	HG3	PRO	A	332	40.971	−14.272	−25.292	1.00	0.00	H
ATOM	5223	HD2	PRO	A	332	40.278	−12.795	−27.801	1.00	0.00	H

ATOM	5224	HD3	PRO	A	332	41.359	−12.316	−26.461	1.00	0.00	H
ATOM	5225	N	VAL	A	333	37.755	−12.043	−23.108	1.00	0.00	N
ATOM	5226	CA	VAL	A	333	37.818	−11.748	−21.663	1.00	0.00	C
ATOM	5227	C	VAL	A	333	36.797	−12.574	−20.884	1.00	0.00	C
ATOM	5228	O	VAL	A	333	35.596	−12.459	−21.122	1.00	0.00	O
ATOM	5229	CB	VAL	A	333	37.630	−10.237	−21.418	1.00	0.00	C
ATOM	5230	CG1	VAL	A	333	37.602	−9.885	−19.926	1.00	0.00	C
ATOM	5231	CG2	VAL	A	333	38.772	−9.436	−22.052	1.00	0.00	C
ATOM	5232	H	VAL	A	333	36.989	−11.634	−23.637	1.00	0.00	H
ATOM	5233	HA	VAL	A	333	38.801	−12.012	−21.284	1.00	0.00	H
ATOM	5234	HB	VAL	A	333	36.688	−9.913	−21.863	1.00	0.00	H
ATOM	5235	HG11	VAL	A	333	37.547	−8.805	−19.817	1.00	0.00	H
ATOM	5236	HG12	VAL	A	333	38.505	−10.253	−19.441	1.00	0.00	H
ATOM	5237	HG13	VAL	A	333	36.724	−10.320	−19.451	1.00	0.00	H
ATOM	5238	HG21	VAL	A	333	39.733	−9.733	−21.631	1.00	0.00	H
ATOM	5239	HG22	VAL	A	333	38.794	−9.608	−23.130	1.00	0.00	H
ATOM	5240	HG23	VAL	A	333	38.623	−8.369	−21.894	1.00	0.00	H
ATOM	5241	N	PHE	A	334	37.233	−13.305	−19.852	1.00	0.00	N
ATOM	5242	CA	PHE	A	334	36.331	−13.835	−18.824	1.00	0.00	C
ATOM	5243	C	PHE	A	334	35.964	−12.719	−17.835	1.00	0.00	C
ATOM	5244	O	PHE	A	334	36.843	−12.129	−17.201	1.00	0.00	O
ATOM	5245	CB	PHE	A	334	36.959	−15.051	−18.122	1.00	0.00	C
ATOM	5246	CG	PHE	A	334	35.997	−15.842	−17.241	1.00	0.00	C
ATOM	5247	CD1	PHE	A	334	35.513	−17.097	−17.663	1.00	0.00	C
ATOM	5248	CD2	PHE	A	334	35.586	−15.337	−15.992	1.00	0.00	C
ATOM	5249	CE1	PHE	A	334	34.633	−17.833	−16.848	1.00	0.00	C
ATOM	5250	CE2	PHE	A	334	34.703	−16.071	−15.179	1.00	0.00	C
ATOM	5251	CZ	PHE	A	334	34.227	−17.322	−15.606	1.00	0.00	C
ATOM	5252	H	PHE	A	334	38.221	−13.255	−19.622	1.00	0.00	H
ATOM	5253	HA	PHE	A	334	35.414	−14.176	−19.302	1.00	0.00	H

ATOM	5254	HB2	PHE	A	334	37.801	−14.718	−17.518	1.00	0.00	H
ATOM	5255	HB3	PHE	A	334	37.358	−15.720	−18.884	1.00	0.00	H
ATOM	5256	HD1	PHE	A	334	35.822	−17.512	−18.611	1.00	0.00	H
ATOM	5257	HD2	PHE	A	334	35.939	−14.375	−15.658	1.00	0.00	H
ATOM	5258	HE1	PHE	A	334	34.270	−18.795	−17.179	1.00	0.00	H
ATOM	5259	HE2	PHE	A	334	34.388	−15.673	−14.227	1.00	0.00	H
ATOM	5260	HZ	PHE	A	334	33.549	−17.891	−14.986	1.00	0.00	H
ATOM	5261	N	ALA	A	335	34.671	−12.507	−17.602	1.00	0.00	N
ATOM	5262	CA	ALA	A	335	34.141	−11.521	−16.666	1.00	0.00	C
ATOM	5263	C	ALA	A	335	33.212	−12.164	−15.628	1.00	0.00	C
ATOM	5264	O	ALA	A	335	32.470	−13.099	−15.933	1.00	0.00	O
ATOM	5265	CB	ALA	A	335	33.431	−10.421	−17.464	1.00	0.00	C
ATOM	5266	H	ALA	A	335	33.994	−13.052	−18.129	1.00	0.00	H
ATOM	5267	HA	ALA	A	335	34.962	−11.057	−16.120	1.00	0.00	H
ATOM	5268	HB1	ALA	A	335	33.030	−9.667	−16.787	1.00	0.00	H
ATOM	5269	HB2	ALA	A	335	34.141	−9.946	−18.139	1.00	0.00	H
ATOM	5270	HB3	ALA	A	335	32.615	−10.850	−18.047	1.00	0.00	H
ATOM	5271	N	VAL	A	336	33.139	−11.547	−14.447	1.00	0.00	N
ATOM	5272	CA	VAL	A	336	32.083	−11.777	−13.449	1.00	0.00	C
ATOM	5273	C	VAL	A	336	31.351	−10.462	−13.197	1.00	0.00	C
ATOM	5274	O	VAL	A	336	31.989	−9.424	−13.007	1.00	0.00	O
ATOM	5275	CB	VAL	A	336	32.650	−12.377	−12.151	1.00	0.00	C
ATOM	5276	CG1	VAL	A	336	31.546	−12.621	−11.119	1.00	0.00	C
ATOM	5277	CG2	VAL	A	336	33.333	−13.723	−12.418	1.00	0.00	C
ATOM	5278	H	VAL	A	336	33.774	−10.772	−14.276	1.00	0.00	H
ATOM	5279	HA	VAL	A	336	31.361	−12.486	−13.849	1.00	0.00	H
ATOM	5280	HB	VAL	A	336	33.378	−11.687	−11.727	1.00	0.00	H
ATOM	5281	HG11	VAL	A	336	31.099	−11.677	−10.822	1.00	0.00	H
ATOM	5282	HG12	VAL	A	336	30.778	−13.267	−11.544	1.00	0.00	H
ATOM	5283	HG13	VAL	A	336	31.963	−13.094	−10.231	1.00	0.00	H

ATOM 5284 HG21 VAL A 336 32.638 −14.391 −12.921 1.00 0.00 H
ATOM 5285 HG22 VAL A 336 34.201 −13.574 −13.056 1.00 0.00 H
ATOM 5286 HG23 VAL A 336 33.649 −14.177 −11.482 1.00 0.00 H
ATOM 5287 N PHE A 337 30.023 −10.468 −13.299 1.00 0.00 N
ATOM 5288 CA PHE A 337 29.228 −9.250 −13.457 1.00 0.00 C
ATOM 5289 C PHE A 337 27.805 −9.372 −12.899 1.00 0.00 C
ATOM 5290 O PHE A 337 27.212 −10.445 −12.858 1.00 0.00 O
ATOM 5291 CB PHE A 337 29.207 −8.850 −14.944 1.00 0.00 C
ATOM 5292 CG PHE A 337 28.480 −9.814 −15.865 1.00 0.00 C
ATOM 5293 CD1 PHE A 337 27.113 −9.623 −16.147 1.00 0.00 C
ATOM 5294 CD2 PHE A 337 29.167 −10.898 −16.447 1.00 0.00 C
ATOM 5295 CE1 PHE A 337 26.438 −10.507 −17.008 1.00 0.00 C
ATOM 5296 CE2 PHE A 337 28.491 −11.785 −17.303 1.00 0.00 C
ATOM 5297 CZ PHE A 337 27.127 −11.588 −17.585 1.00 0.00 C
ATOM 5298 H PHE A 337 29.551 −11.355 −13.465 1.00 0.00 H
ATOM 5299 HA PHE A 337 29.712 −8.448 −12.901 1.00 0.00 H
ATOM 5300 HB2 PHE A 337 28.742 −7.866 −15.033 1.00 0.00 H
ATOM 5301 HB3 PHE A 337 30.234 −8.747 −15.295 1.00 0.00 H
ATOM 5302 HD1 PHE A 337 26.580 −8.800 −15.693 1.00 0.00 H
ATOM 5303 HD2 PHE A 337 30.216 −11.055 −16.238 1.00 0.00 H
ATOM 5304 HE1 PHE A 337 25.390 −10.360 −17.224 1.00 0.00 H
ATOM 5305 HE2 PHE A 337 29.025 −12.613 −17.751 1.00 0.00 H
ATOM 5306 HZ PHE A 337 26.608 −12.269 −18.244 1.00 0.00 H
ATOM 5307 N LYS A 338 27.240 −8.220 −12.553 1.00 0.00 N
ATOM 5308 CA LYS A 338 25.839 −7.947 −12.214 1.00 0.00 C
ATOM 5309 C LYS A 338 25.263 −7.008 −13.290 1.00 0.00 C
ATOM 5310 O LYS A 338 26.030 −6.417 −14.050 1.00 0.00 O
ATOM 5311 CB LYS A 338 25.860 −7.346 −10.797 1.00 0.00 C
ATOM 5312 CG LYS A 338 24.499 −7.194 −10.108 1.00 0.00 C
ATOM 5313 CD LYS A 338 24.577 −6.475 −8.746 1.00 0.00 C

ATOM	5314	CE	LYS	A	338	25.443	−7.150	−7.667	1.00	0.00	C
ATOM	5315	NZ	LYS	A	338	26.884	−6.802	−7.778	1.00	0.00	N
ATOM	5316	H	LYS	A	338	27.814	−7.392	−12.688	1.00	0.00	H
ATOM	5317	HA	LYS	A	338	25.260	−8.876	−12.213	1.00	0.00	H
ATOM	5318	HB2	LYS	A	338	26.470	−8.002	−10.176	1.00	0.00	H
ATOM	5319	HB3	LYS	A	338	26.344	−6.372	−10.838	1.00	0.00	H
ATOM	5320	HG2	LYS	A	338	24.059	−8.182	−9.978	1.00	0.00	H
ATOM	5321	HG3	LYS	A	338	23.833	−6.610	−10.740	1.00	0.00	H
ATOM	5322	HD2	LYS	A	338	23.558	−6.428	−8.358	1.00	0.00	H
ATOM	5323	HD3	LYS	A	338	24.917	−5.448	−8.897	1.00	0.00	H
ATOM	5324	HE2	LYS	A	338	25.075	−6.818	−6.693	1.00	0.00	H
ATOM	5325	HE3	LYS	A	338	25.297	−8.233	−7.721	1.00	0.00	H
ATOM	5326	HZ1	LYS	A	338	27.421	−7.108	−6.980	1.00	0.00	H
ATOM	5327	HZ2	LYS	A	338	27.036	−5.788	−7.866	1.00	0.00	H
ATOM	5328	HZ3	LYS	A	338	27.294	−7.172	−8.620	1.00	0.00	H
ATOM	5329	N	ASP	A	339	23.944	−6.818	−13.330	1.00	0.00	N
ATOM	5330	CA	ASP	A	339	23.222	−6.048	−14.366	1.00	0.00	C
ATOM	5331	C	ASP	A	339	23.896	−4.734	−14.809	1.00	0.00	C
ATOM	5332	O	ASP	A	339	24.173	−4.557	−15.994	1.00	0.00	O
ATOM	5333	CB	ASP	A	339	21.796	−5.737	−13.875	1.00	0.00	C
ATOM	5334	CG	ASP	A	339	20.981	−6.974	−13.498	1.00	0.00	C
ATOM	5335	OD1	ASP	A	339	21.248	−8.068	−14.038	1.00	0.00	O
ATOM	5336	OD2	ASP	A	339	20.002	−6.837	−12.738	1.00	0.00	O
ATOM	5337	H	ASP	A	339	23.380	−7.414	−12.740	1.00	0.00	H
ATOM	5338	HA	ASP	A	339	23.130	−6.675	−15.251	1.00	0.00	H
ATOM	5339	HB2	ASP	A	339	21.857	−5.080	−13.006	1.00	0.00	H
ATOM	5340	HB3	ASP	A	339	21.264	−5.205	−14.666	1.00	0.00	H
ATOM	5341	N	ASN	A	340	24.298	−3.885	−13.860	1.00	0.00	N
ATOM	5342	CA	ASN	A	340	24.885	−2.570	−14.147	1.00	0.00	C
ATOM	5343	C	ASN	A	340	26.396	−2.501	−13.831	1.00	0.00	C

ATOM	5344	O	ASN	A	340	26.945	−1.414	−13.664	1.00	0.00	O
ATOM	5345	CB	ASN	A	340	24.076	−1.470	−13.425	1.00	0.00	C
ATOM	5346	CG	ASN	A	340	22.606	−1.368	−13.813	1.00	0.00	C
ATOM	5347	OD1	ASN	A	340	21.953	−2.294	−14.255	1.00	0.00	O
ATOM	5348	ND2	ASN	A	340	21.981	−0.257	−13.505	1.00	0.00	N
ATOM	5349	H	ASN	A	340	24.028	−4.088	−12.912	1.00	0.00	H
ATOM	5350	HA	ASN	A	340	24.801	−2.379	−15.216	1.00	0.00	H
ATOM	5351	HB2	ASN	A	340	24.140	−1.618	−12.349	1.00	0.00	H
ATOM	5352	HB3	ASN	A	340	24.536	−0.510	−13.658	1.00	0.00	H
ATOM	5353	HD21	ASN	A	340	22.481	0.518	−13.116	1.00	0.00	H
ATOM	5354	HD22	ASN	A	340	21.007	−0.232	−13.745	1.00	0.00	H
ATOM	5355	N	GLU	A	341	27.044	−3.641	−13.567	1.00	0.00	N
ATOM	5356	CA	GLU	A	341	28.320	−3.684	−12.845	1.00	0.00	C
ATOM	5357	C	GLU	A	341	29.136	−4.944	−13.186	1.00	0.00	C
ATOM	5358	O	GLU	A	341	28.926	−6.011	−12.610	1.00	0.00	O
ATOM	5359	CB	GLU	A	341	28.009	−3.592	−11.337	1.00	0.00	C
ATOM	5360	CG	GLU	A	341	29.250	−3.611	−10.429	1.00	0.00	C
ATOM	5361	CD	GLU	A	341	28.901	−3.792	−8.942	1.00	0.00	C
ATOM	5362	OE1	GLU	A	341	27.930	−4.535	−8.646	1.00	0.00	O
ATOM	5363	OE2	GLU	A	341	29.795	−3.486	−8.126	1.00	0.00	O
ATOM	5364	H	GLU	A	341	26.555	−4.518	−13.728	1.00	0.00	H
ATOM	5365	HA	GLU	A	341	28.917	−2.813	−13.116	1.00	0.00	H
ATOM	5366	HB2	GLU	A	341	27.361	−4.427	−11.083	1.00	0.00	H
ATOM	5367	HB3	GLU	A	341	27.455	−2.673	−11.139	1.00	0.00	H
ATOM	5368	HG2	GLU	A	341	29.915	−4.424	−10.721	1.00	0.00	H
ATOM	5369	HG3	GLU	A	341	29.795	−2.675	−10.572	1.00	0.00	H
ATOM	5370	N	ILE	A	342	30.198	−4.801	−13.985	1.00	0.00	N
ATOM	5371	CA	ILE	A	342	31.266	−5.814	−14.034	1.00	0.00	C
ATOM	5372	C	ILE	A	342	32.076	−5.717	−12.738	1.00	0.00	C
ATOM	5373	O	ILE	A	342	32.664	−4.669	−12.454	1.00	0.00	O

ATOM 5374 CB ILE A 342 32.156 −5.677 −15.287 1.00 0.00 C
ATOM 5375 CG1 ILE A 342 31.302 −5.785 −16.568 1.00 0.00 C
ATOM 5376 CG2 ILE A 342 33.254 −6.755 −15.266 1.00 0.00 C
ATOM 5377 CD1 ILE A 342 32.078 −5.541 −17.866 1.00 0.00 C
ATOM 5378 H ILE A 342 30.361 −3.908 −14.423 1.00 0.00 H
ATOM 5379 HA ILE A 342 30.809 −6.800 −14.070 1.00 0.00 H
ATOM 5380 HB ILE A 342 32.633 −4.695 −15.272 1.00 0.00 H
ATOM 5381 HG12 ILE A 342 30.826 −6.764 −16.617 1.00 0.00 H
ATOM 5382 HG13 ILE A 342 30.519 −5.034 −16.523 1.00 0.00 H
ATOM 5383 HG21 ILE A 342 33.873 −6.669 −16.156 1.00 0.00 H
ATOM 5384 HG22 ILE A 342 33.905 −6.638 −14.401 1.00 0.00 H
ATOM 5385 HG23 ILE A 342 32.806 −7.749 −15.244 1.00 0.00 H
ATOM 5386 HD11 ILE A 342 31.375 −5.399 −18.687 1.00 0.00 H
ATOM 5387 HD12 ILE A 342 32.696 −4.648 −17.776 1.00 0.00 H
ATOM 5388 HD13 ILE A 342 32.704 −6.403 −18.093 1.00 0.00 H
ATOM 5389 N LEU A 343 32.133 −6.804 −11.970 1.00 0.00 N
ATOM 5390 CA LEU A 343 32.840 −6.872 −10.693 1.00 0.00 C
ATOM 5391 C LEU A 343 34.351 −7.012 −10.895 1.00 0.00 C
ATOM 5392 O LEU A 343 35.111 −6.214 −10.352 1.00 0.00 O
ATOM 5393 CB LEU A 343 32.286 −8.039 −9.850 1.00 0.00 C
ATOM 5394 CG LEU A 343 30.801 −7.923 −9.469 1.00 0.00 C
ATOM 5395 CD1 LEU A 343 30.341 −9.201 −8.769 1.00 0.00 C
ATOM 5396 CD2 LEU A 343 30.583 −6.765 −8.503 1.00 0.00 C
ATOM 5397 H LEU A 343 31.714 −7.659 −12.319 1.00 0.00 H
ATOM 5398 HA LEU A 343 32.678 −5.940 −10.152 1.00 0.00 H
ATOM 5399 HB2 LEU A 343 32.436 −8.972 −10.397 1.00 0.00 H
ATOM 5400 HB3 LEU A 343 32.863 −8.099 −8.928 1.00 0.00 H
ATOM 5401 HG LEU A 343 30.190 −7.776 −10.359 1.00 0.00 H
ATOM 5402 HD11 LEU A 343 30.416 −10.040 −9.456 1.00 0.00 H
ATOM 5403 HD12 LEU A 343 30.965 −9.394 −7.896 1.00 0.00 H

ATOM 5404 HD13 LEU A 343 29.306 −9.099 −8.451 1.00 0.00 H
ATOM 5405 HD21 LEU A 343 29.538 −6.697 −8.223 1.00 0.00 H
ATOM 5406 HD22 LEU A 343 30.862 −5.823 −8.967 1.00 0.00 H
ATOM 5407 HD23 LEU A 343 31.173 −6.909 −7.602 1.00 0.00 H
ATOM 5408 N TYR A 344 34.778 −7.973 −11.715 1.00 0.00 N
ATOM 5409 CA TYR A 344 36.188 −8.288 −11.978 1.00 0.00 C
ATOM 5410 C TYR A 344 36.325 −9.150 −13.250 1.00 0.00 C
ATOM 5411 O TYR A 344 35.342 −9.718 −13.734 1.00 0.00 O
ATOM 5412 CB TYR A 344 36.816 −8.955 −10.734 1.00 0.00 C
ATOM 5413 CG TYR A 344 35.981 −10.021 −10.046 1.00 0.00 C
ATOM 5414 CD1 TYR A 344 36.069 −11.354 −10.476 1.00 0.00 C
ATOM 5415 CD2 TYR A 344 35.151 −9.691 −8.956 1.00 0.00 C
ATOM 5416 CE1 TYR A 344 35.326 −12.359 −9.829 1.00 0.00 C
ATOM 5417 CE2 TYR A 344 34.375 −10.683 −8.329 1.00 0.00 C
ATOM 5418 CZ TYR A 344 34.464 −12.022 −8.763 1.00 0.00 C
ATOM 5419 OH TYR A 344 33.758 −12.990 −8.126 1.00 0.00 O
ATOM 5420 H TYR A 344 34.087 −8.579 −12.143 1.00 0.00 H
ATOM 5421 HA TYR A 344 36.730 −7.357 −12.155 1.00 0.00 H
ATOM 5422 HB2 TYR A 344 37.782 −9.384 −11.004 1.00 0.00 H
ATOM 5423 HB3 TYR A 344 37.023 −8.176 −10.001 1.00 0.00 H
ATOM 5424 HD1 TYR A 344 36.719 −11.593 −11.300 1.00 0.00 H
ATOM 5425 HD2 TYR A 344 35.110 −8.673 −8.593 1.00 0.00 H
ATOM 5426 HE1 TYR A 344 35.409 −13.386 −10.144 1.00 0.00 H
ATOM 5427 HE2 TYR A 344 33.731 −10.422 −7.504 1.00 0.00 H
ATOM 5428 HH TYR A 344 33.155 −12.609 −7.463 1.00 0.00 H
ATOM 5429 N GLN A 345 37.522 −9.172 −13.848 1.00 0.00 N
ATOM 5430 CA GLN A 345 37.804 −9.771 −15.164 1.00 0.00 C
ATOM 5431 C GLN A 345 39.182 −10.448 −15.201 1.00 0.00 C
ATOM 5432 O GLN A 345 40.060 −10.084 −14.419 1.00 0.00 O
ATOM 5433 CB GLN A 345 37.770 −8.695 −16.268 1.00 0.00 C

ATOM	5434	CG	GLN	A	345	36.420	−7.986	−16.448	1.00	0.00	C
ATOM	5435	CD	GLN	A	345	36.325	−7.177	−17.745	1.00	0.00	C
ATOM	5436	OE1	GLN	A	345	35.285	−7.105	−18.378	1.00	0.00	O
ATOM	5437	NE2	GLN	A	345	37.386	−6.568	−18.228	1.00	0.00	N
ATOM	5438	H	GLN	A	345	38.307	−8.741	−13.381	1.00	0.00	H
ATOM	5439	HA	GLN	A	345	37.051	−10.527	−15.383	1.00	0.00	H
ATOM	5440	HB2	GLN	A	345	38.023	−9.175	−17.212	1.00	0.00	H
ATOM	5441	HB3	GLN	A	345	38.538	−7.949	−16.058	1.00	0.00	H
ATOM	5442	HG2	GLN	A	345	36.263	−7.309	−15.609	1.00	0.00	H
ATOM	5443	HG3	GLN	A	345	35.628	−8.732	−16.453	1.00	0.00	H
ATOM	5444	HE21	GLN	A	345	38.291	−6.686	−17.807	1.00	0.00	H
ATOM	5445	HE22	GLN	A	345	37.279	−6.137	−19.133	1.00	0.00	H
ATOM	5446	N	VAL	A	346	39.417	−11.281	−16.218	1.00	0.00	N
ATOM	5447	CA	VAL	A	346	40.752	−11.695	−16.682	1.00	0.00	C
ATOM	5448	C	VAL	A	346	40.706	−12.018	−18.191	1.00	0.00	C
ATOM	5449	O	VAL	A	346	39.733	−12.636	−18.636	1.00	0.00	O
ATOM	5450	CB	VAL	A	346	41.278	−12.878	−15.835	1.00	0.00	C
ATOM	5451	CG1	VAL	A	346	40.557	−14.206	−16.109	1.00	0.00	C
ATOM	5452	CG2	VAL	A	346	42.782	−13.096	−16.004	1.00	0.00	C
ATOM	5453	H	VAL	A	346	38.632	−11.555	−16.804	1.00	0.00	H
ATOM	5454	HA	VAL	A	346	41.422	−10.855	−16.519	1.00	0.00	H
ATOM	5455	HB	VAL	A	346	41.120	−12.629	−14.786	1.00	0.00	H
ATOM	5456	HG21	VAL	A	346	43.319	−12.184	−15.746	1.00	0.00	H
ATOM	5457	HG22	VAL	A	346	43.019	−13.377	−17.029	1.00	0.00	H
ATOM	5458	HG23	VAL	A	346	43.115	−13.891	−15.336	1.00	0.00	H
ATOM	5459	HG11	VAL	A	346	40.898	−14.956	−15.400	1.00	0.00	H
ATOM	5460	HG12	VAL	A	346	40.776	−14.555	−17.120	1.00	0.00	H
ATOM	5461	HG13	VAL	A	346	39.483	−14.070	−15.998	1.00	0.00	H
ATOM	5462	N	PRO	A	347	41.672	−11.567	−19.015	1.00	0.00	N
ATOM	5463	CA	PRO	A	347	41.815	−12.059	−20.389	1.00	0.00	C

ATOM	5464	C	PRO	A	347	42.199	−13.549	−20.397	1.00	0.00	C
ATOM	5465	O	PRO	A	347	42.816	−14.042	−19.450	1.00	0.00	O
ATOM	5466	CB	PRO	A	347	42.892	−11.179	−21.026	1.00	0.00	C
ATOM	5467	CG	PRO	A	347	43.775	−10.790	−19.843	1.00	0.00	C
ATOM	5468	CD	PRO	A	347	42.779	−10.674	−18.689	1.00	0.00	C
ATOM	5469	HA	PRO	A	347	40.886	−11.931	−20.942	1.00	0.00	H
ATOM	5470	HB2	PRO	A	347	43.459	−11.707	−21.791	1.00	0.00	H
ATOM	5471	HB3	PRO	A	347	42.432	−10.283	−21.448	1.00	0.00	H
ATOM	5472	HG2	PRO	A	347	44.486	−11.592	−19.637	1.00	0.00	H
ATOM	5473	HG3	PRO	A	347	44.299	−9.850	−20.023	1.00	0.00	H
ATOM	5474	HD2	PRO	A	347	42.410	−9.649	−18.628	1.00	0.00	H
ATOM	5475	HD3	PRO	A	347	43.267	−10.953	−17.758	1.00	0.00	H
ATOM	5476	N	LEU	A	348	41.781	−14.292	−21.424	1.00	0.00	N
ATOM	5477	CA	LEU	A	348	42.153	−15.708	−21.601	1.00	0.00	C
ATOM	5478	C	LEU	A	348	43.313	−15.930	−22.584	1.00	0.00	C
ATOM	5479	O	LEU	A	348	43.916	−17.003	−22.567	1.00	0.00	O
ATOM	5480	CB	LEU	A	348	40.908	−16.515	−21.999	1.00	0.00	C
ATOM	5481	CG	LEU	A	348	39.815	−16.563	−20.916	1.00	0.00	C
ATOM	5482	CD1	LEU	A	348	38.663	−17.422	−21.430	1.00	0.00	C
ATOM	5483	CD2	LEU	A	348	40.292	−17.178	−19.595	1.00	0.00	C
ATOM	5484	H	LEU	A	348	41.218	−13.848	−22.145	1.00	0.00	H
ATOM	5485	HA	LEU	A	348	42.523	−16.102	−20.657	1.00	0.00	H
ATOM	5486	HB2	LEU	A	348	40.490	−16.086	−22.913	1.00	0.00	H
ATOM	5487	HB3	LEU	A	348	41.219	−17.535	−22.223	1.00	0.00	H
ATOM	5488	HG	LEU	A	348	39.435	−15.560	−20.721	1.00	0.00	H
ATOM	5489	HD11	LEU	A	348	39.032	−18.402	−21.730	1.00	0.00	H
ATOM	5490	HD12	LEU	A	348	38.214	−16.928	−22.291	1.00	0.00	H
ATOM	5491	HD13	LEU	A	348	37.916	−17.543	−20.651	1.00	0.00	H
ATOM	5492	HD21	LEU	A	348	39.453	−17.289	−18.910	1.00	0.00	H
ATOM	5493	HD22	LEU	A	348	41.023	−16.516	−19.130	1.00	0.00	H

ATOM	5494	HD23	LEU	A	348	40.746	−18.149	−19.785	1.00	0.00	H
ATOM	5495	N	ALA	A	349	43.635	−14.908	−23.376	1.00	0.00	N
ATOM	5496	CA	ALA	A	349	44.818	−14.755	−24.219	1.00	0.00	C
ATOM	5497	C	ALA	A	349	44.987	−13.252	−24.542	1.00	0.00	C
ATOM	5498	O	ALA	A	349	44.159	−12.438	−24.120	1.00	0.00	O
ATOM	5499	CB	ALA	A	349	44.645	−15.607	−25.483	1.00	0.00	C
ATOM	5500	H	ALA	A	349	43.099	−14.058	−23.283	1.00	0.00	H
ATOM	5501	HA	ALA	A	349	45.709	−15.090	−23.684	1.00	0.00	H
ATOM	5502	HB1	ALA	A	349	44.487	−16.651	−25.214	1.00	0.00	H
ATOM	5503	HB2	ALA	A	349	45.547	−15.545	−26.090	1.00	0.00	H
ATOM	5504	HB3	ALA	A	349	43.792	−15.247	−26.059	1.00	0.00	H
ATOM	5505	N	GLU	A	350	46.079	−12.879	−25.204	1.00	0.00	N
ATOM	5506	CA	GLU	A	350	46.498	−11.494	−25.453	1.00	0.00	C
ATOM	5507	C	GLU	A	350	45.468	−10.642	−26.233	1.00	0.00	C
ATOM	5508	O	GLU	A	350	44.559	−11.151	−26.894	1.00	0.00	O
ATOM	5509	CB	GLU	A	350	47.877	−11.500	−26.147	1.00	0.00	C
ATOM	5510	CG	GLU	A	350	49.067	−11.778	−25.204	1.00	0.00	C
ATOM	5511	CD	GLU	A	350	49.010	−13.103	−24.418	1.00	0.00	C
ATOM	5512	OE1	GLU	A	350	48.528	−14.117	−24.975	1.00	0.00	O
ATOM	5513	OE2	GLU	A	350	49.404	−13.083	−23.229	1.00	0.00	O
ATOM	5514	H	GLU	A	350	46.787	−13.593	−25.381	1.00	0.00	H
ATOM	5515	HA	GLU	A	350	46.621	−11.007	−24.485	1.00	0.00	H
ATOM	5516	HB2	GLU	A	350	48.047	−10.519	−26.594	1.00	0.00	H
ATOM	5517	HB3	GLU	A	350	47.876	−12.231	−26.957	1.00	0.00	H
ATOM	5518	HG2	GLU	A	350	49.982	−11.765	−25.800	1.00	0.00	H
ATOM	5519	HG3	GLU	A	350	49.132	−10.945	−24.499	1.00	0.00	H
ATOM	5520	N	ASP	A	351	45.567	−9.315	−26.094	1.00	0.00	N
ATOM	5521	CA	ASP	A	351	44.497	−8.365	−26.441	1.00	0.00	C
ATOM	5522	C	ASP	A	351	44.094	−8.301	−27.931	1.00	0.00	C
ATOM	5523	O	ASP	A	351	42.990	−7.836	−28.226	1.00	0.00	O

ATOM 5524 CB ASP A 351 44.881 −6.958 −25.937 1.00 0.00 C
ATOM 5525 CG ASP A 351 44.639 −6.757 −24.435 1.00 0.00 C
ATOM 5526 OD1 ASP A 351 43.468 −6.950 −24.024 1.00 0.00 O
ATOM 5527 OD2 ASP A 351 45.490 −6.093 −23.805 1.00 0.00 O
ATOM 5528 H ASP A 351 46.339 −8.953 −25.556 1.00 0.00 H
ATOM 5529 HA ASP A 351 43.602 −8.685 −25.906 1.00 0.00 H
ATOM 5530 HB2 ASP A 351 44.280 −6.215 −26.462 1.00 0.00 H
ATOM 5531 HB3 ASP A 351 45.927 −6.759 −26.181 1.00 0.00 H
ATOM 5532 N ASP A 352 44.925 −8.767 −28.866 1.00 0.00 N
ATOM 5533 CA ASP A 352 44.606 −8.863 −30.300 1.00 0.00 C
ATOM 5534 C ASP A 352 44.079 −10.250 −30.726 1.00 0.00 C
ATOM 5535 O ASP A 352 43.694 −10.434 −31.882 1.00 0.00 O
ATOM 5536 CB ASP A 352 45.843 −8.477 −31.136 1.00 0.00 C
ATOM 5537 CG ASP A 352 47.062 −9.401 −30.976 1.00 0.00 C
ATOM 5538 OD1 ASP A 352 47.124 −10.148 −29.971 1.00 0.00 O
ATOM 5539 OD2 ASP A 352 47.972 −9.284 −31.827 1.00 0.00 O
ATOM 5540 H ASP A 352 45.803 −9.192 −28.582 1.00 0.00 H
ATOM 5541 HA ASP A 352 43.818 −8.148 −30.537 1.00 0.00 H
ATOM 5542 HB2 ASP A 352 46.141 −7.463 −30.860 1.00 0.00 H
ATOM 5543 HB3 ASP A 352 45.557 −8.452 −32.189 1.00 0.00 H
ATOM 5544 N THR A 353 44.066 −11.241 −29.828 1.00 0.00 N
ATOM 5545 CA THR A 353 43.853 −12.647 −30.209 1.00 0.00 C
ATOM 5546 C THR A 353 42.453 −12.929 −30.763 1.00 0.00 C
ATOM 5547 O THR A 353 41.426 −12.483 −30.240 1.00 0.00 O
ATOM 5548 CB THR A 353 44.200 −13.636 −29.087 1.00 0.00 C
ATOM 5549 OG1 THR A 353 43.480 −13.376 −27.904 1.00 0.00 O
ATOM 5550 CG2 THR A 353 45.697 −13.631 −28.786 1.00 0.00 C
ATOM 5551 H THR A 353 44.425 −11.039 −28.902 1.00 0.00 H
ATOM 5552 HA THR A 353 44.554 −12.847 −31.020 1.00 0.00 H
ATOM 5553 HB THR A 353 43.935 −14.639 −29.424 1.00 0.00 H

ATOM 5554 HG1 THR A 353 43.870 −12.569 −27.510 1.00 0.00 H
ATOM 5555 HG21 THR A 353 46.249 −13.959 −29.666 1.00 0.00 H
ATOM 5556 HG22 THR A 353 46.034 −12.629 −28.521 1.00 0.00 H
ATOM 5557 HG23 THR A 353 45.915 −14.311 −27.966 1.00 0.00 H
ATOM 5558 N ASN A 354 42.429 −13.715 −31.845 1.00 0.00 N
ATOM 5559 CA ASN A 354 41.236 −14.024 −32.631 1.00 0.00 C
ATOM 5560 C ASN A 354 40.294 −14.957 −31.852 1.00 0.00 C
ATOM 5561 O ASN A 354 40.630 −16.120 −31.626 1.00 0.00 O
ATOM 5562 CB ASN A 354 41.618 −14.676 −33.983 1.00 0.00 C
ATOM 5563 CG ASN A 354 42.868 −14.140 −34.658 1.00 0.00 C
ATOM 5564 OD1 ASN A 354 42.810 −13.580 −35.746 1.00 0.00 O
ATOM 5565 ND2 ASN A 354 44.017 −14.604 −34.223 1.00 0.00 N
ATOM 5566 H ASN A 354 43.313 −14.023 −32.214 1.00 0.00 H
ATOM 5567 HA ASN A 354 40.725 −13.083 −32.837 1.00 0.00 H
ATOM 5568 HB2 ASN A 354 40.779 −14.562 −34.668 1.00 0.00 H
ATOM 5569 HB3 ASN A 354 41.778 −15.743 −33.834 1.00 0.00 H
ATOM 5570 HD21 ASN A 354 44.018 −15.343 −33.525 1.00 0.00 H
ATOM 5571 HD22 ASN A 354 44.874 −14.262 −34.638 1.00 0.00 H
ATOM 5572 N ALA A 355 39.044 −14.557 −31.630 1.00 0.00 N
ATOM 5573 CA ALA A 355 38.017 −15.404 −31.026 1.00 0.00 C
ATOM 5574 C ALA A 355 36.608 −15.069 −31.542 1.00 0.00 C
ATOM 5575 O ALA A 355 36.221 −13.909 −31.643 1.00 0.00 O
ATOM 5576 CB ALA A 355 38.142 −15.320 −29.499 1.00 0.00 C
ATOM 5577 H ALA A 355 38.783 −13.618 −31.920 1.00 0.00 H
ATOM 5578 HA ALA A 355 38.217 −16.432 −31.319 1.00 0.00 H
ATOM 5579 HB1 ALA A 355 37.343 −15.891 −29.029 1.00 0.00 H
ATOM 5580 HB2 ALA A 355 39.102 −15.727 −29.185 1.00 0.00 H
ATOM 5581 HB3 ALA A 355 38.096 −14.278 −29.183 1.00 0.00 H
ATOM 5582 N GLN A 356 35.828 −16.103 −31.874 1.00 0.00 N
ATOM 5583 CA GLN A 356 34.526 −15.982 −32.544 1.00 0.00 C

ATOM 5584 C GLN A 356 33.333 −16.170 −31.594 1.00 0.00 C

ATOM 5585 O GLN A 356 32.337 −15.453 −31.702 1.00 0.00 O

ATOM 5586 CB GLN A 356 34.480 −16.985 −33.715 1.00 0.00 C

ATOM 5587 CG GLN A 356 33.157 −16.936 −34.500 1.00 0.00 C

ATOM 5588 CD GLN A 356 33.195 −17.799 −35.760 1.00 0.00 C

ATOM 5589 OE1 GLN A 356 33.313 −17.314 −36.874 1.00 0.00 O

ATOM 5590 NE2 GLN A 356 33.064 −19.101 −35.649 1.00 0.00 N

ATOM 5591 H GLN A 356 36.215 −17.030 −31.725 1.00 0.00 H

ATOM 5592 HA GLN A 356 34.438 −14.979 −32.966 1.00 0.00 H

ATOM 5593 HB2 GLN A 356 35.296 −16.751 −34.400 1.00 0.00 H

ATOM 5594 HB3 GLN A 356 34.632 −17.996 −33.333 1.00 0.00 H

ATOM 5595 HG2 GLN A 356 32.342 −17.284 −33.866 1.00 0.00 H

ATOM 5596 HG3 GLN A 356 32.955 −15.905 −34.794 1.00 0.00 H

ATOM 5597 HE21 GLN A 356 33.000 −19.557 −34.732 1.00 0.00 H

ATOM 5598 HE22 GLN A 356 33.138 −19.652 −36.480 1.00 0.00 H

ATOM 5599 N LYS A 357 33.388 −17.173 −30.711 1.00 0.00 N

ATOM 5600 CA LYS A 357 32.240 −17.681 −29.939 1.00 0.00 C

ATOM 5601 C LYS A 357 32.682 −18.168 −28.565 1.00 0.00 C

ATOM 5602 O LYS A 357 33.741 −18.776 −28.439 1.00 0.00 O

ATOM 5603 CB LYS A 357 31.547 −18.790 −30.761 1.00 0.00 C

ATOM 5604 CG LYS A 357 30.540 −19.664 −29.995 1.00 0.00 C

ATOM 5605 CD LYS A 357 29.769 −20.596 −30.942 1.00 0.00 C

ATOM 5606 CE LYS A 357 29.078 −21.752 −30.201 1.00 0.00 C

ATOM 5607 NZ LYS A 357 28.161 −21.295 −29.126 1.00 0.00 N

ATOM 5608 H LYS A 357 34.251 −17.700 −30.664 1.00 0.00 H

ATOM 5609 HA LYS A 357 31.531 −16.868 −29.782 1.00 0.00 H

ATOM 5610 HB2 LYS A 357 31.048 −18.328 −31.615 1.00 0.00 H

ATOM 5611 HB3 LYS A 357 32.314 −19.458 −31.144 1.00 0.00 H

ATOM 5612 HG2 LYS A 357 31.082 −20.276 −29.271 1.00 0.00 H

ATOM 5613 HG3 LYS A 357 29.835 −19.028 −29.463 1.00 0.00 H

ATOM 5614 HD2 LYS A 357 30.468 −21.027 −31.663 1.00 0.00 H
ATOM 5615 HD3 LYS A 357 29.033 −20.017 −31.501 1.00 0.00 H
ATOM 5616 HE2 LYS A 357 28.535 −22.362 −30.932 1.00 0.00 H
ATOM 5617 HE3 LYS A 357 29.848 −22.398 −29.769 1.00 0.00 H
ATOM 5618 HZ1 LYS A 357 28.395 −20.352 −28.850 1.00 0.00 H
ATOM 5619 HZ2 LYS A 357 27.197 −21.295 −29.446 1.00 0.00 H
ATOM 5620 HZ3 LYS A 357 28.214 −21.895 −28.314 1.00 0.00 H
ATOM 5621 N THR A 358 31.786 −18.091 −27.584 1.00 0.00 N
ATOM 5622 CA THR A 358 32.006 −18.621 −26.227 1.00 0.00 C
ATOM 5623 C THR A 358 30.860 −19.538 −25.788 1.00 0.00 C
ATOM 5624 O THR A 358 29.787 −19.525 −26.394 1.00 0.00 O
ATOM 5625 CB THR A 358 32.270 −17.490 −25.212 1.00 0.00 C
ATOM 5626 OG1 THR A 358 31.074 −16.897 −24.765 1.00 0.00 O
ATOM 5627 CG2 THR A 358 33.141 −16.368 −25.790 1.00 0.00 C
ATOM 5628 H THR A 358 30.932 −17.574 −27.756 1.00 0.00 H
ATOM 5629 HA THR A 358 32.904 −19.234 −26.235 1.00 0.00 H
ATOM 5630 HB THR A 358 32.775 −17.927 −24.352 1.00 0.00 H
ATOM 5631 HG1 THR A 358 30.441 −16.923 −25.509 1.00 0.00 H
ATOM 5632 HG21 THR A 358 32.610 −15.830 −26.577 1.00 0.00 H
ATOM 5633 HG22 THR A 358 34.062 −16.785 −26.192 1.00 0.00 H
ATOM 5634 HG23 THR A 358 33.393 −15.649 −25.018 1.00 0.00 H
ATOM 5635 N ILE A 359 31.105 −20.429 −24.825 1.00 0.00 N
ATOM 5636 CA ILE A 359 30.079 −21.182 −24.077 1.00 0.00 C
ATOM 5637 C ILE A 359 30.595 −21.341 −22.645 1.00 0.00 C
ATOM 5638 O ILE A 359 31.689 −21.875 −22.482 1.00 0.00 O
ATOM 5639 CB ILE A 359 29.778 −22.581 −24.683 1.00 0.00 C
ATOM 5640 CG1 ILE A 359 29.423 −22.537 −26.187 1.00 0.00 C
ATOM 5641 CG2 ILE A 359 28.645 −23.257 −23.884 1.00 0.00 C
ATOM 5642 CD1 ILE A 359 29.248 −23.908 −26.847 1.00 0.00 C
ATOM 5643 H ILE A 359 32.060 −20.505 −24.484 1.00 0.00 H

ATOM 5644 HA ILE A 359 29.155 −20.605 −24.054 1.00 0.00 H

ATOM 5645 HB ILE A 359 30.672 −23.197 −24.578 1.00 0.00 H

ATOM 5646 HG12 ILE A 359 30.232 −22.046 −26.727 1.00 0.00 H

ATOM 5647 HG13 ILE A 359 28.508 −21.959 −26.320 1.00 0.00 H

ATOM 5648 HG21 ILE A 359 28.468 −24.269 −24.240 1.00 0.00 H

ATOM 5649 HG22 ILE A 359 27.722 −22.683 −23.976 1.00 0.00 H

ATOM 5650 HG23 ILE A 359 28.904 −23.343 −22.828 1.00 0.00 H

ATOM 5651 HD11 ILE A 359 29.126 −23.779 −27.920 1.00 0.00 H

ATOM 5652 HD12 ILE A 359 30.133 −24.518 −26.666 1.00 0.00 H

ATOM 5653 HD13 ILE A 359 28.366 −24.413 −26.457 1.00 0.00 H

ATOM 5654 N THR A 360 29.848 −20.909 −21.625 1.00 0.00 N

ATOM 5655 CA THR A 360 30.239 −21.043 −20.209 1.00 0.00 C

ATOM 5656 C THR A 360 29.170 −21.768 −19.397 1.00 0.00 C

ATOM 5657 O THR A 360 28.071 −21.240 −19.221 1.00 0.00 O

ATOM 5658 CB THR A 360 30.554 −19.680 −19.570 1.00 0.00 C

ATOM 5659 OG1 THR A 360 31.510 −18.986 −20.352 1.00 0.00 O

ATOM 5660 CG2 THR A 360 31.101 −19.840 −18.148 1.00 0.00 C

ATOM 5661 H THR A 360 28.953 −20.464 −21.828 1.00 0.00 H

ATOM 5662 HA THR A 360 31.147 −21.635 −20.150 1.00 0.00 H

ATOM 5663 HB THR A 360 29.646 −19.076 −19.532 1.00 0.00 H

ATOM 5664 HG1 THR A 360 30.986 −18.336 −20.839 1.00 0.00 H

ATOM 5665 HG21 THR A 360 31.458 −18.886 −17.767 1.00 0.00 H

ATOM 5666 HG22 THR A 360 31.917 −20.562 −18.137 1.00 0.00 H

ATOM 5667 HG23 THR A 360 30.307 −20.191 −17.488 1.00 0.00 H

ATOM 5668 N ASP A 361 29.554 −22.875 −18.757 1.00 0.00 N

ATOM 5669 CA ASP A 361 28.732 −23.626 −17.797 1.00 0.00 C

ATOM 5670 C ASP A 361 29.445 −23.727 −16.432 1.00 0.00 C

ATOM 5671 O ASP A 361 30.665 −23.899 −16.383 1.00 0.00 O

ATOM 5672 CB ASP A 361 28.341 −25.012 −18.352 1.00 0.00 C

ATOM 5673 CG ASP A 361 27.499 −25.017 −19.645 1.00 0.00 C

ATOM	5674	OD1	ASP	A	361	26.931	−23.966	−20.023	1.00	0.00	O
ATOM	5675	OD2	ASP	A	361	27.259	−26.122	−20.172	1.00	0.00	O
ATOM	5676	H	ASP	A	361	30.515	−23.185	−18.872	1.00	0.00	H
ATOM	5677	HA	ASP	A	361	27.806	−23.087	−17.636	1.00	0.00	H
ATOM	5678	HB2	ASP	A	361	27.766	−25.528	−17.581	1.00	0.00	H
ATOM	5679	HB3	ASP	A	361	29.253	−25.587	−18.525	1.00	0.00	H
ATOM	5680	N	CYS	A	362	28.711	−23.600	−15.317	1.00	0.00	N
ATOM	5681	CA	CYS	A	362	29.288	−23.544	−13.963	1.00	0.00	C
ATOM	5682	C	CYS	A	362	28.628	−24.526	−12.979	1.00	0.00	C
ATOM	5683	O	CYS	A	362	27.448	−24.856	−13.087	1.00	0.00	O
ATOM	5684	CB	CYS	A	362	29.248	−22.110	−13.412	1.00	0.00	C
ATOM	5685	SG	CYS	A	362	29.929	−20.789	−14.458	1.00	0.00	S
ATOM	5686	H	CYS	A	362	27.710	−23.484	−15.398	1.00	0.00	H
ATOM	5687	HA	CYS	A	362	30.330	−23.843	−14.021	1.00	0.00	H
ATOM	5688	HB2	CYS	A	362	29.773	−22.089	−12.457	1.00	0.00	H
ATOM	5689	HB3	CYS	A	362	28.207	−21.865	−13.210	1.00	0.00	H
ATOM	5690	N	PHE	A	363	29.408	−24.998	−12.005	1.00	0.00	N
ATOM	5691	CA	PHE	A	363	29.070	−26.101	−11.101	1.00	0.00	C
ATOM	5692	C	PHE	A	363	29.947	−26.077	−9.832	1.00	0.00	C
ATOM	5693	O	PHE	A	363	30.757	−25.170	−9.634	1.00	0.00	O
ATOM	5694	CB	PHE	A	363	29.207	−27.430	−11.875	1.00	0.00	C
ATOM	5695	CG	PHE	A	363	30.536	−27.636	−12.581	1.00	0.00	C
ATOM	5696	CD1	PHE	A	363	31.601	−28.282	−11.925	1.00	0.00	C
ATOM	5697	CD2	PHE	A	363	30.700	−27.194	−13.909	1.00	0.00	C
ATOM	5698	CE1	PHE	A	363	32.823	−28.480	−12.591	1.00	0.00	C
ATOM	5699	CE2	PHE	A	363	31.923	−27.385	−14.572	1.00	0.00	C
ATOM	5700	CZ	PHE	A	363	32.984	−28.030	−13.914	1.00	0.00	C
ATOM	5701	H	PHE	A	363	30.362	−24.648	−11.955	1.00	0.00	H
ATOM	5702	HA	PHE	A	363	28.032	−25.997	−10.784	1.00	0.00	H
ATOM	5703	HB2	PHE	A	363	28.416	−27.470	−12.625	1.00	0.00	H

ATOM 5704 HB3 PHE A 363 29.030 −28.269 −11.200 1.00 0.00 H
ATOM 5705 HD1 PHE A 363 31.479 −28.650 −10.917 1.00 0.00 H
ATOM 5706 HD2 PHE A 363 29.882 −26.715 −14.429 1.00 0.00 H
ATOM 5707 HE1 PHE A 363 33.626 −29.002 −12.092 1.00 0.00 H
ATOM 5708 HE2 PHE A 363 32.032 −27.051 −15.594 1.00 0.00 H
ATOM 5709 HZ PHE A 363 33.915 −28.200 −14.432 1.00 0.00 H
ATOM 5710 N LEU A 364 29.728 −27.028 −8.919 1.00 0.00 N
ATOM 5711 CA LEU A 364 30.560 −27.230 −7.728 1.00 0.00 C
ATOM 5712 C LEU A 364 31.562 −28.372 −7.941 1.00 0.00 C
ATOM 5713 O LEU A 364 31.174 −29.437 −8.414 1.00 0.00 O
ATOM 5714 CB LEU A 364 29.673 −27.522 −6.502 1.00 0.00 C
ATOM 5715 CG LEU A 364 28.669 −26.417 −6.127 1.00 0.00 C
ATOM 5716 CD1 LEU A 364 27.853 −26.858 −4.912 1.00 0.00 C
ATOM 5717 CD2 LEU A 364 29.362 −25.101 −5.780 1.00 0.00 C
ATOM 5718 H LEU A 364 29.099 −27.778 −9.164 1.00 0.00 H
ATOM 5719 HA LEU A 364 31.135 −26.327 −7.531 1.00 0.00 H
ATOM 5720 HB2 LEU A 364 29.118 −28.442 −6.692 1.00 0.00 H
ATOM 5721 HB3 LEU A 364 30.325 −27.703 −5.649 1.00 0.00 H
ATOM 5722 HG LEU A 364 27.984 −26.247 −6.957 1.00 0.00 H
ATOM 5723 HD11 LEU A 364 27.325 −27.784 −5.140 1.00 0.00 H
ATOM 5724 HD12 LEU A 364 27.119 −26.092 −4.662 1.00 0.00 H
ATOM 5725 HD13 LEU A 364 28.506 −27.020 −4.054 1.00 0.00 H
ATOM 5726 HD21 LEU A 364 28.621 −24.368 −5.463 1.00 0.00 H
ATOM 5727 HD22 LEU A 364 29.875 −24.717 −6.658 1.00 0.00 H
ATOM 5728 HD23 LEU A 364 30.079 −25.254 −4.976 1.00 0.00 H
ATOM 5729 N LEU A 365 32.764 −28.232 −7.381 1.00 0.00 N
ATOM 5730 CA LEU A 365 33.765 −29.298 −7.254 1.00 0.00 C
ATOM 5731 C LEU A 365 34.573 −29.096 −5.958 1.00 0.00 C
ATOM 5732 O LEU A 365 34.895 −27.959 −5.620 1.00 0.00 O
ATOM 5733 CB LEU A 365 34.638 −29.307 −8.520 1.00 0.00 C

ATOM 5734 CG LEU A 365 35.660 −30.462 −8.538 1.00 0.00 C
ATOM 5735 CD1 LEU A 365 35.408 −31.381 −9.734 1.00 0.00 C
ATOM 5736 CD2 LEU A 365 37.081 −29.901 −8.594 1.00 0.00 C
ATOM 5737 H LEU A 365 33.019 −27.317 −7.019 1.00 0.00 H
ATOM 5738 HA LEU A 365 33.250 −30.259 −7.194 1.00 0.00 H
ATOM 5739 HB2 LEU A 365 33.986 −29.380 −9.392 1.00 0.00 H
ATOM 5740 HB3 LEU A 365 35.157 −28.354 −8.592 1.00 0.00 H
ATOM 5741 HG LEU A 365 35.558 −31.060 −7.632 1.00 0.00 H
ATOM 5742 HD11 LEU A 365 34.366 −31.701 −9.747 1.00 0.00 H
ATOM 5743 HD12 LEU A 365 36.023 −32.275 −9.638 1.00 0.00 H
ATOM 5744 HD13 LEU A 365 35.643 −30.880 −10.671 1.00 0.00 H
ATOM 5745 HD21 LEU A 365 37.255 −29.247 −7.741 1.00 0.00 H
ATOM 5746 HD22 LEU A 365 37.794 −30.721 −8.533 1.00 0.00 H
ATOM 5747 HD23 LEU A 365 37.246 −29.353 −9.518 1.00 0.00 H
ATOM 5748 N GLU A 366 34.601 −30.118 −5.091 1.00 0.00 N
ATOM 5749 CA GLU A 366 34.884 −29.983 −3.643 1.00 0.00 C
ATOM 5750 C GLU A 366 34.048 −28.886 −2.936 1.00 0.00 C
ATOM 5751 O GLU A 366 34.477 −28.274 −1.959 1.00 0.00 O
ATOM 5752 CB GLU A 366 36.395 −29.860 −3.355 1.00 0.00 C
ATOM 5753 CG GLU A 366 37.145 −31.198 −3.465 1.00 0.00 C
ATOM 5754 CD GLU A 366 38.190 −31.378 −2.348 1.00 0.00 C
ATOM 5755 OE1 GLU A 366 38.821 −30.375 −1.927 1.00 0.00 O
ATOM 5756 OE2 GLU A 366 38.681 −32.516 −2.173 1.00 0.00 O
ATOM 5757 H GLU A 366 34.370 −31.037 −5.440 1.00 0.00 H
ATOM 5758 HA GLU A 366 34.554 −30.909 −3.170 1.00 0.00 H
ATOM 5759 HB2 GLU A 366 36.510 −29.515 −2.329 1.00 0.00 H
ATOM 5760 HB3 GLU A 366 36.856 −29.118 −4.008 1.00 0.00 H
ATOM 5761 HG2 GLU A 366 37.630 −31.256 −4.443 1.00 0.00 H
ATOM 5762 HG3 GLU A 366 36.432 −32.022 −3.396 1.00 0.00 H
ATOM 5763 N ASN A 367 32.802 −28.675 −3.380 1.00 0.00 N

ATOM 5764 CA ASN A 367 31.907 −27.593 −2.925 1.00 0.00 C
ATOM 5765 C ASN A 367 32.383 −26.165 −3.288 1.00 0.00 C
ATOM 5766 O ASN A 367 31.709 −25.190 −2.958 1.00 0.00 O
ATOM 5767 CB ASN A 367 31.563 −27.725 −1.424 1.00 0.00 C
ATOM 5768 CG ASN A 367 31.368 −29.150 −0.942 1.00 0.00 C
ATOM 5769 OD1 ASN A 367 30.305 −29.730 −1.059 1.00 0.00 O
ATOM 5770 ND2 ASN A 367 32.399 −29.764 −0.407 1.00 0.00 N
ATOM 5771 H ASN A 367 32.498 −29.235 −4.161 1.00 0.00 H
ATOM 5772 HA ASN A 367 30.969 −27.732 −3.461 1.00 0.00 H
ATOM 5773 HB2 ASN A 367 32.357 −27.270 −0.832 1.00 0.00 H
ATOM 5774 HB3 ASN A 367 30.649 −27.170 −1.218 1.00 0.00 H
ATOM 5775 HD21 ASN A 367 33.303 −29.311 −0.454 1.00 0.00 H
ATOM 5776 HD22 ASN A 367 32.270 −30.720 −0.134 1.00 0.00 H
ATOM 5777 N VAL A 368 33.491 −26.020 −4.022 1.00 0.00 N
ATOM 5778 CA VAL A 368 33.969 −24.747 −4.578 1.00 0.00 C
ATOM 5779 C VAL A 368 33.324 −24.503 −5.943 1.00 0.00 C
ATOM 5780 O VAL A 368 33.290 −25.400 −6.783 1.00 0.00 O
ATOM 5781 CB VAL A 368 35.508 −24.732 −4.680 1.00 0.00 C
ATOM 5782 CG1 VAL A 368 36.033 −23.389 −5.208 1.00 0.00 C
ATOM 5783 CG2 VAL A 368 36.160 −24.977 −3.311 1.00 0.00 C
ATOM 5784 H VAL A 368 33.989 −26.857 −4.310 1.00 0.00 H
ATOM 5785 HA VAL A 368 33.669 −23.943 −3.907 1.00 0.00 H
ATOM 5786 HB VAL A 368 35.837 −25.513 −5.364 1.00 0.00 H
ATOM 5787 HG21 VAL A 368 35.901 −25.974 −2.949 1.00 0.00 H
ATOM 5788 HG22 VAL A 368 37.245 −24.929 −3.397 1.00 0.00 H
ATOM 5789 HG23 VAL A 368 35.814 −24.238 −2.588 1.00 0.00 H
ATOM 5790 HG11 VAL A 368 35.705 −23.237 −6.236 1.00 0.00 H
ATOM 5791 HG12 VAL A 368 37.123 −23.403 −5.202 1.00 0.00 H
ATOM 5792 HG13 VAL A 368 35.676 −22.572 −4.582 1.00 0.00 H
ATOM 5793 N ILE A 369 32.876 −23.270 −6.200 1.00 0.00 N

ATOM	5794	CA	ILE	A	369	32.316	−22.881	−7.499	1.00	0.00	C
ATOM	5795	C	ILE	A	369	33.428	−22.871	−8.564	1.00	0.00	C
ATOM	5796	O	ILE	A	369	34.371	−22.074	−8.495	1.00	0.00	O
ATOM	5797	CB	ILE	A	369	31.559	−21.530	−7.438	1.00	0.00	C
ATOM	5798	CG1	ILE	A	369	30.408	−21.464	−6.404	1.00	0.00	C
ATOM	5799	CG2	ILE	A	369	30.949	−21.216	−8.819	1.00	0.00	C
ATOM	5800	CD1	ILE	A	369	30.844	−21.176	−4.960	1.00	0.00	C
ATOM	5801	H	ILE	A	369	32.924	−22.578	−5.473	1.00	0.00	H
ATOM	5802	HA	ILE	A	369	31.591	−23.642	−7.788	1.00	0.00	H
ATOM	5803	HB	ILE	A	369	32.268	−20.742	−7.194	1.00	0.00	H
ATOM	5804	HG12	ILE	A	369	29.830	−22.386	−6.436	1.00	0.00	H
ATOM	5805	HG13	ILE	A	369	29.734	−20.653	−6.681	1.00	0.00	H
ATOM	5806	HG21	ILE	A	369	30.446	−20.248	−8.802	1.00	0.00	H
ATOM	5807	HG22	ILE	A	369	31.716	−21.179	−9.591	1.00	0.00	H
ATOM	5808	HG23	ILE	A	369	30.223	−21.985	−9.091	1.00	0.00	H
ATOM	5809	HD11	ILE	A	369	29.959	−20.971	−4.357	1.00	0.00	H
ATOM	5810	HD12	ILE	A	369	31.498	−20.304	−4.933	1.00	0.00	H
ATOM	5811	HD13	ILE	A	369	31.353	−22.035	−4.527	1.00	0.00	H
ATOM	5812	N	TRP	A	370	33.221	−23.646	−9.624	1.00	0.00	N
ATOM	5813	CA	TRP	A	370	34.004	−23.652	−10.859	1.00	0.00	C
ATOM	5814	C	TRP	A	370	33.111	−23.305	−12.051	1.00	0.00	C
ATOM	5815	O	TRP	A	370	31.931	−23.647	−12.076	1.00	0.00	O
ATOM	5816	CB	TRP	A	370	34.646	−25.033	−11.057	1.00	0.00	C
ATOM	5817	CG	TRP	A	370	35.935	−25.278	−10.333	1.00	0.00	C
ATOM	5818	CD1	TRP	A	370	36.062	−25.804	−9.094	1.00	0.00	C
ATOM	5819	CD2	TRP	A	370	37.300	−25.058	−10.811	1.00	0.00	C
ATOM	5820	NE1	TRP	A	370	37.401	−25.981	−8.797	1.00	0.00	N
ATOM	5821	CE2	TRP	A	370	38.209	−25.521	−9.814	1.00	0.00	C
ATOM	5822	CE3	TRP	A	370	37.861	−24.560	−12.008	1.00	0.00	C
ATOM	5823	CZ2	TRP	A	370	39.600	−25.486	−9.992	1.00	0.00	C

ATOM 5824 CZ3 TRP A 370 39.260 −24.502 −12.189 1.00 0.00 C
ATOM 5825 CH2 TRP A 370 40.127 −24.969 −11.188 1.00 0.00 C
ATOM 5826 H TRP A 370 32.381 −24.220 −9.628 1.00 0.00 H
ATOM 5827 HA TRP A 370 34.795 −22.906 −10.811 1.00 0.00 H
ATOM 5828 HB2 TRP A 370 34.856 −25.166 −12.119 1.00 0.00 H
ATOM 5829 HB3 TRP A 370 33.928 −25.809 −10.785 1.00 0.00 H
ATOM 5830 HD1 TRP A 370 35.228 −26.104 −8.464 1.00 0.00 H
ATOM 5831 HE3 TRP A 370 37.199 −24.243 −12.800 1.00 0.00 H
ATOM 5832 HZ2 TRP A 370 40.251 −25.888 −9.229 1.00 0.00 H
ATOM 5833 HZ3 TRP A 370 39.672 −24.128 −13.115 1.00 0.00 H
ATOM 5834 HH2 TRP A 370 41.194 −24.953 −11.349 1.00 0.00 H
ATOM 5835 HE1 TRP A 370 37.724 −26.535 −8.013 1.00 0.00 H
ATOM 5836 N CYS A 371 33.726 −22.774 −13.102 1.00 0.00 N
ATOM 5837 CA CYS A 371 33.159 −22.695 −14.442 1.00 0.00 C
ATOM 5838 C CYS A 371 34.091 −23.385 −15.442 1.00 0.00 C
ATOM 5839 O CYS A 371 35.315 −23.285 −15.323 1.00 0.00 O
ATOM 5840 CB CYS A 371 32.919 −21.229 −14.810 1.00 0.00 C
ATOM 5841 SG CYS A 371 31.775 −20.338 −13.723 1.00 0.00 S
ATOM 5842 H CYS A 371 34.693 −22.492 −13.002 1.00 0.00 H
ATOM 5843 HA CYS A 371 32.205 −23.213 −14.472 1.00 0.00 H
ATOM 5844 HB2 CYS A 371 33.879 −20.711 −14.791 1.00 0.00 H
ATOM 5845 HB3 CYS A 371 32.533 −21.179 −15.828 1.00 0.00 H
ATOM 5846 N ILE A 372 33.514 −23.989 −16.478 1.00 0.00 N
ATOM 5847 CA ILE A 372 34.205 −24.331 −17.721 1.00 0.00 C
ATOM 5848 C ILE A 372 33.719 −23.359 −18.793 1.00 0.00 C
ATOM 5849 O ILE A 372 32.521 −23.297 −19.076 1.00 0.00 O
ATOM 5850 CB ILE A 372 33.947 −25.805 −18.112 1.00 0.00 C
ATOM 5851 CG1 ILE A 372 34.562 −26.794 −17.094 1.00 0.00 C
ATOM 5852 CG2 ILE A 372 34.447 −26.109 −19.538 1.00 0.00 C
ATOM 5853 CD1 ILE A 372 36.087 −26.936 −17.155 1.00 0.00 C

ATOM 5854 H ILE A 372 32.496 −23.990 −16.515 1.00 0.00 H
ATOM 5855 HA ILE A 372 35.278 −24.189 −17.603 1.00 0.00 H
ATOM 5856 HB ILE A 372 32.866 −25.964 −18.106 1.00 0.00 H
ATOM 5857 HG12 ILE A 372 34.295 −26.483 −16.086 1.00 0.00 H
ATOM 5858 HG13 ILE A 372 34.125 −27.780 −17.260 1.00 0.00 H
ATOM 5859 HG21 ILE A 372 34.319 −27.170 −19.755 1.00 0.00 H
ATOM 5860 HG22 ILE A 372 35.498 −25.838 −19.645 1.00 0.00 H
ATOM 5861 HG23 ILE A 372 33.867 −25.544 −20.267 1.00 0.00 H
ATOM 5862 HD11 ILE A 372 36.560 −25.959 −17.102 1.00 0.00 H
ATOM 5863 HD12 ILE A 372 36.434 −27.544 −16.322 1.00 0.00 H
ATOM 5864 HD13 ILE A 372 36.374 −27.427 −18.082 1.00 0.00 H
ATOM 5865 N SER A 373 34.655 −22.674 −19.445 1.00 0.00 N
ATOM 5866 CA SER A 373 34.400 −21.909 −20.663 1.00 0.00 C
ATOM 5867 C SER A 373 35.076 −22.576 −21.855 1.00 0.00 C
ATOM 5868 O SER A 373 36.304 −22.649 −21.926 1.00 0.00 O
ATOM 5869 CB SER A 373 34.860 −20.458 −20.526 1.00 0.00 C
ATOM 5870 OG SER A 373 34.121 −19.796 −19.517 1.00 0.00 O
ATOM 5871 H SER A 373 35.622 −22.790 −19.160 1.00 0.00 H
ATOM 5872 HA SER A 373 33.332 −21.883 −20.845 1.00 0.00 H
ATOM 5873 HB2 SER A 373 35.921 −20.434 −20.273 1.00 0.00 H
ATOM 5874 HB3 SER A 373 34.711 −19.942 −21.477 1.00 0.00 H
ATOM 5875 HG SER A 373 33.176 −19.799 −19.757 1.00 0.00 H
ATOM 5876 N LEU A 374 34.289 −22.959 −22.858 1.00 0.00 N
ATOM 5877 CA LEU A 374 34.793 −23.130 −24.216 1.00 0.00 C
ATOM 5878 C LEU A 374 34.934 −21.749 −24.865 1.00 0.00 C
ATOM 5879 O LEU A 374 33.976 −20.972 −24.866 1.00 0.00 O
ATOM 5880 CB LEU A 374 33.834 −24.034 −25.013 1.00 0.00 C
ATOM 5881 CG LEU A 374 34.391 −24.484 −26.376 1.00 0.00 C
ATOM 5882 CD1 LEU A 374 35.487 −25.538 −26.208 1.00 0.00 C
ATOM 5883 CD2 LEU A 374 33.282 −25.103 −27.222 1.00 0.00 C

ATOM 5884 H LEU A 374 33.295 −22.780 −22.750 1.00 0.00 H

ATOM 5885 HA LEU A 374 35.777 −23.597 −24.172 1.00 0.00 H

ATOM 5886 HB2 LEU A 374 33.599 −24.918 −24.424 1.00 0.00 H

ATOM 5887 HB3 LEU A 374 32.906 −23.486 −25.175 1.00 0.00 H

ATOM 5888 HG LEU A 374 34.794 −23.630 −26.919 1.00 0.00 H

ATOM 5889 HD11 LEU A 374 36.346 −25.110 −25.694 1.00 0.00 H

ATOM 5890 HD12 LEU A 374 35.810 −25.900 −27.179 1.00 0.00 H

ATOM 5891 HD13 LEU A 374 35.098 −26.384 −25.645 1.00 0.00 H

ATOM 5892 HD21 LEU A 374 33.702 −25.457 −28.163 1.00 0.00 H

ATOM 5893 HD22 LEU A 374 32.520 −24.353 −27.431 1.00 0.00 H

ATOM 5894 HD23 LEU A 374 32.830 −25.945 −26.698 1.00 0.00 H

ATOM 5895 N VAL A 375 36.044 −21.521 −25.565 1.00 0.00 N

ATOM 5896 CA VAL A 375 36.191 −20.403 −26.507 1.00 0.00 C

ATOM 5897 C VAL A 375 36.578 −20.959 −27.873 1.00 0.00 C

ATOM 5898 O VAL A 375 37.483 −21.783 −27.971 1.00 0.00 O

ATOM 5899 CB VAL A 375 37.206 −19.351 −26.014 1.00 0.00 C

ATOM 5900 CG1 VAL A 375 37.118 −18.073 −26.857 1.00 0.00 C

ATOM 5901 CG2 VAL A 375 36.977 −18.946 −24.552 1.00 0.00 C

ATOM 5902 H VAL A 375 36.813 −22.183 −25.505 1.00 0.00 H

ATOM 5903 HA VAL A 375 35.232 −19.898 −26.617 1.00 0.00 H

ATOM 5904 HB VAL A 375 38.214 −19.760 −26.102 1.00 0.00 H

ATOM 5905 HG11 VAL A 375 37.916 −17.387 −26.572 1.00 0.00 H

ATOM 5906 HG12 VAL A 375 37.219 −18.300 −27.917 1.00 0.00 H

ATOM 5907 HG13 VAL A 375 36.157 −17.584 −26.704 1.00 0.00 H

ATOM 5908 HG21 VAL A 375 37.682 −18.162 −24.278 1.00 0.00 H

ATOM 5909 HG22 VAL A 375 35.957 −18.584 −24.419 1.00 0.00 H

ATOM 5910 HG23 VAL A 375 37.147 −19.796 −23.893 1.00 0.00 H

ATOM 5911 N GLU A 376 35.851 −20.579 −28.919 1.00 0.00 N

ATOM 5912 CA GLU A 376 36.264 −20.747 −30.313 1.00 0.00 C

ATOM 5913 C GLU A 376 37.273 −19.642 −30.657 1.00 0.00 C

ATOM 5914 O GLU A 376 36.888 −18.483 −30.801 1.00 0.00 O

ATOM 5915 CB GLU A 376 35.017 −20.720 −31.211 1.00 0.00 C

ATOM 5916 CG GLU A 376 35.308 −21.117 −32.667 1.00 0.00 C

ATOM 5917 CD GLU A 376 34.017 −21.277 −33.488 1.00 0.00 C

ATOM 5918 OE1 GLU A 376 33.056 −20.506 −33.262 1.00 0.00 O

ATOM 5919 OE2 GLU A 376 33.985 −22.125 −34.409 1.00 0.00 O

ATOM 5920 H GLU A 376 35.124 −19.892 −28.747 1.00 0.00 H

ATOM 5921 HA GLU A 376 36.746 −21.714 −30.440 1.00 0.00 H

ATOM 5922 HB2 GLU A 376 34.285 −21.417 −30.802 1.00 0.00 H

ATOM 5923 HB3 GLU A 376 34.585 −19.721 −31.192 1.00 0.00 H

ATOM 5924 HG2 GLU A 376 35.859 −22.060 −32.670 1.00 0.00 H

ATOM 5925 HG3 GLU A 376 35.942 −20.356 −33.128 1.00 0.00 H

ATOM 5926 N ILE A 377 38.560 −19.974 −30.551 1.00 0.00 N

ATOM 5927 CA ILE A 377 39.715 −19.063 −30.511 1.00 0.00 C

ATOM 5928 C ILE A 377 40.825 −19.616 −31.409 1.00 0.00 C

ATOM 5929 O ILE A 377 40.956 −20.835 −31.533 1.00 0.00 O

ATOM 5930 CB ILE A 377 40.193 −18.856 −29.044 1.00 0.00 C

ATOM 5931 CG1 ILE A 377 41.370 −17.861 −28.895 1.00 0.00 C

ATOM 5932 CG2 ILE A 377 40.575 −20.189 −28.381 1.00 0.00 C

ATOM 5933 CD1 ILE A 377 41.760 −17.543 −27.445 1.00 0.00 C

ATOM 5934 H ILE A 377 38.792 −20.946 −30.417 1.00 0.00 H

ATOM 5935 HA ILE A 377 39.417 −18.098 −30.910 1.00 0.00 H

ATOM 5936 HB ILE A 377 39.355 −18.439 −28.493 1.00 0.00 H

ATOM 5937 HG12 ILE A 377 41.090 −16.919 −29.357 1.00 0.00 H

ATOM 5938 HG13 ILE A 377 42.257 −18.239 −29.405 1.00 0.00 H

ATOM 5939 HG21 ILE A 377 39.786 −20.926 −28.493 1.00 0.00 H

ATOM 5940 HG22 ILE A 377 41.480 −20.569 −28.849 1.00 0.00 H

ATOM 5941 HG23 ILE A 377 40.746 −20.042 −27.317 1.00 0.00 H

ATOM 5942 HD11 ILE A 377 42.441 −16.693 −27.441 1.00 0.00 H

ATOM 5943 HD12 ILE A 377 42.272 −18.391 −26.991 1.00 0.00 H

ATOM	5944	HD13	ILE	A	377	40.873	−17.294	−26.862	1.00	0.00	H
ATOM	5945	N	TYR	A	378	41.666	−18.748	−31.963	1.00	0.00	N
ATOM	5946	CA	TYR	A	378	42.959	−19.143	−32.511	1.00	0.00	C
ATOM	5947	C	TYR	A	378	44.014	−18.052	−32.337	1.00	0.00	C
ATOM	5948	O	TYR	A	378	43.752	−16.851	−32.448	1.00	0.00	O
ATOM	5949	CB	TYR	A	378	42.828	−19.608	−33.963	1.00	0.00	C
ATOM	5950	CG	TYR	A	378	42.441	−18.546	−34.974	1.00	0.00	C
ATOM	5951	CD1	TYR	A	378	43.450	−17.841	−35.654	1.00	0.00	C
ATOM	5952	CD2	TYR	A	378	41.086	−18.255	−35.223	1.00	0.00	C
ATOM	5953	CE1	TYR	A	378	43.111	−16.807	−36.546	1.00	0.00	C
ATOM	5954	CE2	TYR	A	378	40.740	−17.229	−36.127	1.00	0.00	C
ATOM	5955	CZ	TYR	A	378	41.755	−16.488	−36.770	1.00	0.00	C
ATOM	5956	OH	TYR	A	378	41.435	−15.394	−37.504	1.00	0.00	O
ATOM	5957	H	TYR	A	378	41.514	−17.751	−31.822	1.00	0.00	H
ATOM	5958	HA	TYR	A	378	43.310	−20.003	−31.939	1.00	0.00	H
ATOM	5959	HB2	TYR	A	378	43.782	−20.030	−34.263	1.00	0.00	H
ATOM	5960	HB3	TYR	A	378	42.114	−20.422	−33.987	1.00	0.00	H
ATOM	5961	HD1	TYR	A	378	44.491	−18.061	−35.454	1.00	0.00	H
ATOM	5962	HD2	TYR	A	378	40.315	−18.787	−34.685	1.00	0.00	H
ATOM	5963	HE1	TYR	A	378	43.883	−16.218	−37.014	1.00	0.00	H
ATOM	5964	HE2	TYR	A	378	39.709	−16.955	−36.281	1.00	0.00	H
ATOM	5965	HH	TYR	A	378	40.980	−14.751	−36.934	1.00	0.00	H
ATOM	5966	N	ASP	A	379	45.229	−18.505	−32.081	1.00	0.00	N
ATOM	5967	CA	ASP	A	379	46.409	−17.666	−31.941	1.00	0.00	C
ATOM	5968	C	ASP	A	379	46.945	−17.392	−33.360	1.00	0.00	C
ATOM	5969	O	ASP	A	379	46.771	−18.225	−34.255	1.00	0.00	O
ATOM	5970	CB	ASP	A	379	47.446	−18.347	−31.017	1.00	0.00	C
ATOM	5971	CG	ASP	A	379	46.906	−18.974	−29.706	1.00	0.00	C
ATOM	5972	OD1	ASP	A	379	45.724	−18.749	−29.347	1.00	0.00	O
ATOM	5973	OD2	ASP	A	379	47.461	−20.027	−29.313	1.00	0.00	O

ATOM	5974	H	ASP	A	379	45.380	−19.510	−32.129	1.00	0.00	H
ATOM	5975	HA	ASP	A	379	46.118	−16.721	−31.479	1.00	0.00	H
ATOM	5976	HB2	ASP	A	379	47.936	−19.136	−31.593	1.00	0.00	H
ATOM	5977	HB3	ASP	A	379	48.209	−17.610	−30.761	1.00	0.00	H
ATOM	5978	N	THR	A	380	47.505	−16.206	−33.627	1.00	0.00	N
ATOM	5979	CA	THR	A	380	47.855	−15.758	−34.996	1.00	0.00	C
ATOM	5980	C	THR	A	380	49.080	−16.505	−35.558	1.00	0.00	C
ATOM	5981	O	THR	A	380	50.196	−15.993	−35.596	1.00	0.00	O
ATOM	5982	CB	THR	A	380	47.994	−14.225	−35.055	1.00	0.00	C
ATOM	5983	OG1	THR	A	380	46.804	−13.651	−34.556	1.00	0.00	O
ATOM	5984	CG2	THR	A	380	48.147	−13.683	−36.478	1.00	0.00	C
ATOM	5985	H	THR	A	380	47.552	−15.520	−32.888	1.00	0.00	H
ATOM	5986	HA	THR	A	380	47.015	−16.009	−35.643	1.00	0.00	H
ATOM	5987	HB	THR	A	380	48.837	−13.903	−34.442	1.00	0.00	H
ATOM	5988	HG1	THR	A	380	47.005	−12.751	−34.267	1.00	0.00	H
ATOM	5989	HG21	THR	A	380	48.158	−12.594	−36.455	1.00	0.00	H
ATOM	5990	HG22	THR	A	380	47.314	−14.019	−37.097	1.00	0.00	H
ATOM	5991	HG23	THR	A	380	49.084	−14.026	−36.915	1.00	0.00	H
ATOM	5992	N	GLY	A	381	48.872	−17.784	−35.881	1.00	0.00	N
ATOM	5993	CA	GLY	A	381	49.893	−18.808	−36.122	1.00	0.00	C
ATOM	5994	C	GLY	A	381	49.376	−20.258	−36.023	1.00	0.00	C
ATOM	5995	O	GLY	A	381	50.028	−21.162	−36.541	1.00	0.00	O
ATOM	5996	H	GLY	A	381	47.931	−18.116	−35.707	1.00	0.00	H
ATOM	5997	HA2	GLY	A	381	50.690	−18.688	−35.389	1.00	0.00	H
ATOM	5998	HA3	GLY	A	381	50.316	−18.663	−37.116	1.00	0.00	H
ATOM	5999	N	ASP	A	382	48.168	−20.490	−35.488	1.00	0.00	N
ATOM	6000	CA	ASP	A	382	47.481	−21.792	−35.521	1.00	0.00	C
ATOM	6001	C	ASP	A	382	47.144	−22.237	−36.963	1.00	0.00	C
ATOM	6002	O	ASP	A	382	46.095	−21.890	−37.507	1.00	0.00	O
ATOM	6003	CB	ASP	A	382	46.181	−21.726	−34.690	1.00	0.00	C

ATOM 6004 CG ASP A 382 46.356 −21.843 −33.174 1.00 0.00 C
ATOM 6005 OD1 ASP A 382 47.052 −22.799 −32.764 1.00 0.00 O
ATOM 6006 OD2 ASP A 382 45.466 −21.325 −32.460 1.00 0.00 O
ATOM 6007 H ASP A 382 47.671 −19.725 −35.045 1.00 0.00 H
ATOM 6008 HA ASP A 382 48.130 −22.547 −35.076 1.00 0.00 H
ATOM 6009 HB2 ASP A 382 45.674 −20.793 −34.932 1.00 0.00 H
ATOM 6010 HB3 ASP A 382 45.529 −22.548 −34.994 1.00 0.00 H
ATOM 6011 N SER A 383 47.910 −23.191 −37.507 1.00 0.00 N
ATOM 6012 CA SER A 383 47.548 −23.910 −38.748 1.00 0.00 C
ATOM 6013 C SER A 383 46.277 −24.760 −38.597 1.00 0.00 C
ATOM 6014 O SER A 383 45.556 −24.997 −39.568 1.00 0.00 O
ATOM 6015 CB SER A 383 48.680 −24.854 −39.169 1.00 0.00 C
ATOM 6016 OG SER A 383 49.909 −24.161 −39.274 1.00 0.00 O
ATOM 6017 H SER A 383 48.843 −23.308 −37.136 1.00 0.00 H
ATOM 6018 HA SER A 383 47.380 −23.185 −39.544 1.00 0.00 H
ATOM 6019 HB2 SER A 383 48.782 −25.648 −38.428 1.00 0.00 H
ATOM 6020 HB3 SER A 383 48.434 −25.303 −40.132 1.00 0.00 H
ATOM 6021 HG SER A 383 50.607 −24.788 −39.476 1.00 0.00 H
ATOM 6022 N VAL A 384 46.026 −25.245 −37.375 1.00 0.00 N
ATOM 6023 CA VAL A 384 44.875 −26.073 −36.993 1.00 0.00 C
ATOM 6024 C VAL A 384 44.001 −25.266 −36.031 1.00 0.00 C
ATOM 6025 O VAL A 384 44.065 −25.443 −34.817 1.00 0.00 O
ATOM 6026 CB VAL A 384 45.326 −27.433 −36.412 1.00 0.00 C
ATOM 6027 CG1 VAL A 384 44.135 −28.338 −36.052 1.00 0.00 C
ATOM 6028 CG2 VAL A 384 46.197 −28.214 −37.406 1.00 0.00 C
ATOM 6029 H VAL A 384 46.631 −24.924 −36.633 1.00 0.00 H
ATOM 6030 HA VAL A 384 44.278 −26.280 −37.879 1.00 0.00 H
ATOM 6031 HB VAL A 384 45.916 −27.262 −35.511 1.00 0.00 H
ATOM 6032 HG11 VAL A 384 44.499 −29.296 −35.684 1.00 0.00 H
ATOM 6033 HG12 VAL A 384 43.511 −28.503 −36.929 1.00 0.00 H

ATOM	6034	HG13	VAL	A	384	43.531	−27.879	−35.270	1.00	0.00	H
ATOM	6035	HG21	VAL	A	384	46.486	−29.174	−36.980	1.00	0.00	H
ATOM	6036	HG22	VAL	A	384	47.104	−27.655	−37.632	1.00	0.00	H
ATOM	6037	HG23	VAL	A	384	45.650	−28.379	−38.333	1.00	0.00	H
ATOM	6038	N	ILE	A	385	43.249	−24.310	−36.577	1.00	0.00	N
ATOM	6039	CA	ILE	A	385	42.337	−23.437	−35.826	1.00	0.00	C
ATOM	6040	C	ILE	A	385	41.344	−24.305	−35.044	1.00	0.00	C
ATOM	6041	O	ILE	A	385	40.645	−25.134	−35.635	1.00	0.00	O
ATOM	6042	CB	ILE	A	385	41.624	−22.470	−36.805	1.00	0.00	C
ATOM	6043	CG1	ILE	A	385	42.651	−21.452	−37.353	1.00	0.00	C
ATOM	6044	CG2	ILE	A	385	40.401	−21.784	−36.165	1.00	0.00	C
ATOM	6045	CD1	ILE	A	385	42.103	−20.467	−38.393	1.00	0.00	C
ATOM	6046	H	ILE	A	385	43.242	−24.251	−37.589	1.00	0.00	H
ATOM	6047	HA	ILE	A	385	42.917	−22.850	−35.113	1.00	0.00	H
ATOM	6048	HB	ILE	A	385	41.254	−23.053	−37.649	1.00	0.00	H
ATOM	6049	HG12	ILE	A	385	43.083	−20.886	−36.528	1.00	0.00	H
ATOM	6050	HG13	ILE	A	385	43.462	−22.000	−37.833	1.00	0.00	H
ATOM	6051	HG21	ILE	A	385	39.947	−21.082	−36.860	1.00	0.00	H
ATOM	6052	HG22	ILE	A	385	40.685	−21.247	−35.268	1.00	0.00	H
ATOM	6053	HG23	ILE	A	385	39.641	−22.519	−35.901	1.00	0.00	H
ATOM	6054	HD11	ILE	A	385	41.596	−21.011	−39.190	1.00	0.00	H
ATOM	6055	HD12	ILE	A	385	42.932	−19.902	−38.818	1.00	0.00	H
ATOM	6056	HD13	ILE	A	385	41.414	−19.763	−37.927	1.00	0.00	H
ATOM	6057	N	ARG	A	386	41.248	−24.131	−33.720	1.00	0.00	N
ATOM	6058	CA	ARG	A	386	40.342	−24.943	−32.893	1.00	0.00	C
ATOM	6059	C	ARG	A	386	39.898	−24.269	−31.591	1.00	0.00	C
ATOM	6060	O	ARG	A	386	40.740	−23.708	−30.900	1.00	0.00	O
ATOM	6061	CB	ARG	A	386	40.968	−26.324	−32.608	1.00	0.00	C
ATOM	6062	CG	ARG	A	386	42.339	−26.281	−31.895	1.00	0.00	C
ATOM	6063	CD	ARG	A	386	42.590	−27.542	−31.066	1.00	0.00	C

ATOM 6064 NE ARG A 386 41.672 −27.601 −29.911 1.00 0.00 N
ATOM 6065 CZ ARG A 386 41.283 −28.664 −29.239 1.00 0.00 C
ATOM 6066 NH1 ARG A 386 41.760 −29.854 −29.480 1.00 0.00 N
ATOM 6067 NH2 ARG A 386 40.388 −28.535 −28.308 1.00 0.00 N
ATOM 6068 H ARG A 386 41.840 −23.442 −33.270 1.00 0.00 H
ATOM 6069 HA ARG A 386 39.447 −25.090 −33.492 1.00 0.00 H
ATOM 6070 HB2 ARG A 386 40.255 −26.894 −32.014 1.00 0.00 H
ATOM 6071 HB3 ARG A 386 41.099 −26.858 −33.549 1.00 0.00 H
ATOM 6072 HG2 ARG A 386 43.124 −26.191 −32.640 1.00 0.00 H
ATOM 6073 HG3 ARG A 386 42.420 −25.424 −31.231 1.00 0.00 H
ATOM 6074 HD2 ARG A 386 42.462 −28.413 −31.712 1.00 0.00 H
ATOM 6075 HD3 ARG A 386 43.621 −27.517 −30.707 1.00 0.00 H
ATOM 6076 HE ARG A 386 41.225 −26.729 −29.640 1.00 0.00 H
ATOM 6077 HH11 ARG A 386 42.465 −29.942 −30.189 1.00 0.00 H
ATOM 6078 HH12 ARG A 386 41.450 −30.644 −28.951 1.00 0.00 H
ATOM 6079 HH21 ARG A 386 40.008 −27.600 −28.162 1.00 0.00 H
ATOM 6080 HH22 ARG A 386 40.035 −29.314 −27.791 1.00 0.00 H
ATOM 6081 N PRO A 387 38.706 −24.627 −31.071 1.00 0.00 N
ATOM 6082 CA PRO A 387 38.286 −24.243 −29.731 1.00 0.00 C
ATOM 6083 C PRO A 387 39.254 −24.707 −28.635 1.00 0.00 C
ATOM 6084 O PRO A 387 39.733 −25.847 −28.657 1.00 0.00 O
ATOM 6085 CB PRO A 387 36.888 −24.833 −29.541 1.00 0.00 C
ATOM 6086 CG PRO A 387 36.346 −24.948 −30.961 1.00 0.00 C
ATOM 6087 CD PRO A 387 37.602 −25.271 −31.766 1.00 0.00 C
ATOM 6088 HA PRO A 387 38.233 −23.158 −29.722 1.00 0.00 H
ATOM 6089 HB2 PRO A 387 36.965 −25.835 −29.116 1.00 0.00 H
ATOM 6090 HB3 PRO A 387 36.261 −24.192 −28.921 1.00 0.00 H
ATOM 6091 HG2 PRO A 387 35.601 −25.739 −31.047 1.00 0.00 H
ATOM 6092 HG3 PRO A 387 35.935 −23.990 −31.280 1.00 0.00 H
ATOM 6093 HD2 PRO A 387 37.490 −24.875 −32.771 1.00 0.00 H

ATOM	6094	HD3	PRO	A	387	37.763	−26.349	−31.788	1.00	0.00	H
ATOM	6095	N	LYS	A	388	39.444	−23.868	−27.616	1.00	0.00	N
ATOM	6096	CA	LYS	A	388	40.324	−24.111	−26.462	1.00	0.00	C
ATOM	6097	C	LYS	A	388	39.483	−24.012	−25.178	1.00	0.00	C
ATOM	6098	O	LYS	A	388	38.647	−23.117	−25.047	1.00	0.00	O
ATOM	6099	CB	LYS	A	388	41.544	−23.149	−26.504	1.00	0.00	C
ATOM	6100	CG	LYS	A	388	42.324	−23.218	−27.841	1.00	0.00	C
ATOM	6101	CD	LYS	A	388	43.554	−22.292	−27.975	1.00	0.00	C
ATOM	6102	CE	LYS	A	388	43.984	−22.263	−29.457	1.00	0.00	C
ATOM	6103	NZ	LYS	A	388	45.117	−21.350	−29.754	1.00	0.00	N
ATOM	6104	H	LYS	A	388	38.938	−22.985	−27.639	1.00	0.00	H
ATOM	6105	HA	LYS	A	388	40.714	−25.127	−26.516	1.00	0.00	H
ATOM	6106	HB2	LYS	A	388	41.196	−22.129	−26.348	1.00	0.00	H
ATOM	6107	HB3	LYS	A	388	42.221	−23.400	−25.689	1.00	0.00	H
ATOM	6108	HG2	LYS	A	388	42.645	−24.247	−28.010	1.00	0.00	H
ATOM	6109	HG3	LYS	A	388	41.638	−22.950	−28.641	1.00	0.00	H
ATOM	6110	HD2	LYS	A	388	43.300	−21.281	−27.653	1.00	0.00	H
ATOM	6111	HD3	LYS	A	388	44.371	−22.662	−27.354	1.00	0.00	H
ATOM	6112	HE2	LYS	A	388	44.259	−23.272	−29.771	1.00	0.00	H
ATOM	6113	HE3	LYS	A	388	43.127	−21.956	−30.065	1.00	0.00	H
ATOM	6114	HZ1	LYS	A	388	45.295	−21.301	−30.761	1.00	0.00	H
ATOM	6115	HZ2	LYS	A	388	44.985	−20.380	−29.459	1.00	0.00	H
ATOM	6116	HZ3	LYS	A	388	46.018	−21.579	−29.348	1.00	0.00	H
ATOM	6117	N	LEU	A	389	39.511	−25.070	−24.364	1.00	0.00	N
ATOM	6118	CA	LEU	A	389	38.758	−25.163	−23.104	1.00	0.00	C
ATOM	6119	C	LEU	A	389	39.538	−24.504	−21.961	1.00	0.00	C
ATOM	6120	O	LEU	A	389	40.651	−24.934	−21.653	1.00	0.00	O
ATOM	6121	CB	LEU	A	389	38.483	−26.636	−22.747	1.00	0.00	C
ATOM	6122	CG	LEU	A	389	37.370	−27.324	−23.548	1.00	0.00	C
ATOM	6123	CD1	LEU	A	389	37.496	−28.842	−23.408	1.00	0.00	C

ATOM	6124	CD2	LEU	A	389	35.992	−26.919	−23.017	1.00	0.00	C
ATOM	6125	H	LEU	A	389	40.160	−25.814	−24.583	1.00	0.00	H
ATOM	6126	HA	LEU	A	389	37.807	−24.639	−23.206	1.00	0.00	H
ATOM	6127	HB2	LEU	A	389	39.411	−27.191	−22.877	1.00	0.00	H
ATOM	6128	HB3	LEU	A	389	38.214	−26.691	−21.692	1.00	0.00	H
ATOM	6129	HG	LEU	A	389	37.455	−27.066	−24.603	1.00	0.00	H
ATOM	6130	HD11	LEU	A	389	38.452	−29.176	−23.810	1.00	0.00	H
ATOM	6131	HD12	LEU	A	389	37.426	−29.132	−22.358	1.00	0.00	H
ATOM	6132	HD13	LEU	A	389	36.696	−29.332	−23.964	1.00	0.00	H
ATOM	6133	HD21	LEU	A	389	35.215	−27.446	−23.570	1.00	0.00	H
ATOM	6134	HD22	LEU	A	389	35.903	−27.181	−21.962	1.00	0.00	H
ATOM	6135	HD23	LEU	A	389	35.843	−25.846	−23.128	1.00	0.00	H
ATOM	6136	N	PHE	A	390	38.858	−23.665	−21.183	1.00	0.00	N
ATOM	6137	CA	PHE	A	390	39.403	−23.004	−19.999	1.00	0.00	C
ATOM	6138	C	PHE	A	390	38.590	−23.365	−18.750	1.00	0.00	C
ATOM	6139	O	PHE	A	390	37.382	−23.135	−18.698	1.00	0.00	O
ATOM	6140	CB	PHE	A	390	39.444	−21.485	−20.223	1.00	0.00	C
ATOM	6141	CG	PHE	A	390	40.315	−21.040	−21.386	1.00	0.00	C
ATOM	6142	CD1	PHE	A	390	41.639	−20.616	−21.159	1.00	0.00	C
ATOM	6143	CD2	PHE	A	390	39.789	−21.012	−22.692	1.00	0.00	C
ATOM	6144	CE1	PHE	A	390	42.425	−20.152	−22.229	1.00	0.00	C
ATOM	6145	CE2	PHE	A	390	40.578	−20.555	−23.763	1.00	0.00	C
ATOM	6146	CZ	PHE	A	390	41.895	−20.119	−23.531	1.00	0.00	C
ATOM	6147	H	PHE	A	390	37.923	−23.388	−21.468	1.00	0.00	H
ATOM	6148	HA	PHE	A	390	40.426	−23.336	−19.846	1.00	0.00	H
ATOM	6149	HB2	PHE	A	390	39.809	−21.010	−19.311	1.00	0.00	H
ATOM	6150	HB3	PHE	A	390	38.428	−21.123	−20.390	1.00	0.00	H
ATOM	6151	HD1	PHE	A	390	42.047	−20.614	−20.160	1.00	0.00	H
ATOM	6152	HD2	PHE	A	390	38.773	−21.329	−22.875	1.00	0.00	H
ATOM	6153	HE1	PHE	A	390	43.426	−19.777	−22.055	1.00	0.00	H

ATOM 6154 HE2 PHE A 390 40.164 −20.516 −24.761 1.00 0.00 H
ATOM 6155 HZ PHE A 390 42.493 −19.729 −24.344 1.00 0.00 H
ATOM 6156 N ALA A 391 39.261 −23.851 −17.708 1.00 0.00 N
ATOM 6157 CA ALA A 391 38.685 −24.025 −16.378 1.00 0.00 C
ATOM 6158 C ALA A 391 38.947 −22.784 −15.510 1.00 0.00 C
ATOM 6159 O ALA A 391 40.084 −22.312 −15.401 1.00 0.00 O
ATOM 6160 CB ALA A 391 39.237 −25.317 −15.764 1.00 0.00 C
ATOM 6161 H ALA A 391 40.267 −23.955 −17.799 1.00 0.00 H
ATOM 6162 HA ALA A 391 37.605 −24.140 −16.470 1.00 0.00 H
ATOM 6163 HB1 ALA A 391 39.054 −26.155 −16.437 1.00 0.00 H
ATOM 6164 HB2 ALA A 391 40.310 −25.225 −15.590 1.00 0.00 H
ATOM 6165 HB3 ALA A 391 38.730 −25.526 −14.822 1.00 0.00 H
ATOM 6166 N VAL A 392 37.916 −22.295 −14.817 1.00 0.00 N
ATOM 6167 CA VAL A 392 37.956 −21.059 −14.023 1.00 0.00 C
ATOM 6168 C VAL A 392 37.325 −21.284 −12.649 1.00 0.00 C
ATOM 6169 O VAL A 392 36.105 −21.292 −12.494 1.00 0.00 O
ATOM 6170 CB VAL A 392 37.279 −19.888 −14.772 1.00 0.00 C
ATOM 6171 CG1 VAL A 392 37.432 −18.581 −13.980 1.00 0.00 C
ATOM 6172 CG2 VAL A 392 37.892 −19.675 −16.162 1.00 0.00 C
ATOM 6173 H VAL A 392 37.002 −22.710 −14.988 1.00 0.00 H
ATOM 6174 HA VAL A 392 38.995 −20.775 −13.864 1.00 0.00 H
ATOM 6175 HB VAL A 392 36.218 −20.097 −14.897 1.00 0.00 H
ATOM 6176 HG11 VAL A 392 36.990 −17.764 −14.551 1.00 0.00 H
ATOM 6177 HG12 VAL A 392 36.904 −18.648 −13.029 1.00 0.00 H
ATOM 6178 HG13 VAL A 392 38.486 −18.362 −13.806 1.00 0.00 H
ATOM 6179 HG21 VAL A 392 37.499 −18.765 −16.611 1.00 0.00 H
ATOM 6180 HG22 VAL A 392 37.633 −20.508 −16.817 1.00 0.00 H
ATOM 6181 HG23 VAL A 392 38.974 −19.599 −16.088 1.00 0.00 H
ATOM 6182 N LYS A 393 38.160 −21.395 −11.612 1.00 0.00 N
ATOM 6183 CA LYS A 393 37.709 −21.398 −10.214 1.00 0.00 C

ATOM	6184	C	LYS	A	393	37.273	−19.984	−9.827	1.00	0.00	C
ATOM	6185	O	LYS	A	393	38.136	−19.117	−9.663	1.00	0.00	O
ATOM	6186	CB	LYS	A	393	38.837	−21.910	−9.306	1.00	0.00	C
ATOM	6187	CG	LYS	A	393	38.355	−22.085	−7.853	1.00	0.00	C
ATOM	6188	CD	LYS	A	393	39.455	−21.895	−6.795	1.00	0.00	C
ATOM	6189	CE	LYS	A	393	40.248	−20.574	−6.872	1.00	0.00	C
ATOM	6190	NZ	LYS	A	393	39.404	−19.388	−7.178	1.00	0.00	N
ATOM	6191	H	LYS	A	393	39.150	−21.439	−11.805	1.00	0.00	H
ATOM	6192	HA	LYS	A	393	36.852	−22.072	−10.117	1.00	0.00	H
ATOM	6193	HB2	LYS	A	393	39.191	−22.870	−9.669	1.00	0.00	H
ATOM	6194	HB3	LYS	A	393	39.675	−21.216	−9.365	1.00	0.00	H
ATOM	6195	HG2	LYS	A	393	37.931	−23.084	−7.748	1.00	0.00	H
ATOM	6196	HG3	LYS	A	393	37.556	−21.382	−7.628	1.00	0.00	H
ATOM	6197	HD2	LYS	A	393	38.983	−21.957	−5.813	1.00	0.00	H
ATOM	6198	HD3	LYS	A	393	40.157	−22.728	−6.869	1.00	0.00	H
ATOM	6199	HE2	LYS	A	393	40.760	−20.434	−5.915	1.00	0.00	H
ATOM	6200	HE3	LYS	A	393	41.017	−20.676	−7.642	1.00	0.00	H
ATOM	6201	HZ1	LYS	A	393	39.901	−18.520	−7.033	1.00	0.00	H
ATOM	6202	HZ2	LYS	A	393	39.105	−19.392	−8.153	1.00	0.00	H
ATOM	6203	HZ3	LYS	A	393	38.535	−19.393	−6.650	1.00	0.00	H
ATOM	6204	N	ILE	A	394	36.023	−19.817	−9.404	1.00	0.00	N
ATOM	6205	CA	ILE	A	394	35.492	−18.542	−8.896	1.00	0.00	C
ATOM	6206	C	ILE	A	394	36.154	−18.149	−7.549	1.00	0.00	C
ATOM	6207	O	ILE	A	394	36.656	−19.019	−6.823	1.00	0.00	O
ATOM	6208	CB	ILE	A	394	33.949	−18.638	−8.842	1.00	0.00	C
ATOM	6209	CG1	ILE	A	394	33.322	−18.816	−10.249	1.00	0.00	C
ATOM	6210	CG2	ILE	A	394	33.250	−17.485	−8.104	1.00	0.00	C
ATOM	6211	CD1	ILE	A	394	33.636	−17.720	−11.281	1.00	0.00	C
ATOM	6212	H	ILE	A	394	35.406	−20.624	−9.400	1.00	0.00	H
ATOM	6213	HA	ILE	A	394	35.755	−17.783	−9.626	1.00	0.00	H

ATOM 6214 HB ILE A 394 33.718 −19.534 −8.272 1.00 0.00 H
ATOM 6215 HG12 ILE A 394 33.643 −19.773 −10.665 1.00 0.00 H
ATOM 6216 HG13 ILE A 394 32.238 −18.867 −10.142 1.00 0.00 H
ATOM 6217 HD11 ILE A 394 33.067 −17.913 −12.191 1.00 0.00 H
ATOM 6218 HD12 ILE A 394 34.696 −17.725 −11.536 1.00 0.00 H
ATOM 6219 HD13 ILE A 394 33.355 −16.739 −10.900 1.00 0.00 H
ATOM 6220 HG21 ILE A 394 32.182 −17.693 −8.058 1.00 0.00 H
ATOM 6221 HG22 ILE A 394 33.612 −17.404 −7.081 1.00 0.00 H
ATOM 6222 HG23 ILE A 394 33.394 −16.540 −8.626 1.00 0.00 H
ATOM 6223 N PRO A 395 36.334 −16.849 −7.234 1.00 0.00 N
ATOM 6224 CA PRO A 395 37.075 −16.429 −6.043 1.00 0.00 C
ATOM 6225 C PRO A 395 36.365 −16.782 −4.730 1.00 0.00 C
ATOM 6226 O PRO A 395 35.196 −16.439 −4.533 1.00 0.00 O
ATOM 6227 CB PRO A 395 37.263 −14.919 −6.169 1.00 0.00 C
ATOM 6228 CG PRO A 395 37.105 −14.661 −7.664 1.00 0.00 C
ATOM 6229 CD PRO A 395 36.057 −15.684 −8.064 1.00 0.00 C
ATOM 6230 HA PRO A 395 38.059 −16.897 −6.062 1.00 0.00 H
ATOM 6231 HB2 PRO A 395 38.236 −14.613 −5.790 1.00 0.00 H
ATOM 6232 HB3 PRO A 395 36.474 −14.398 −5.633 1.00 0.00 H
ATOM 6233 HG2 PRO A 395 36.766 −13.649 −7.868 1.00 0.00 H
ATOM 6234 HG3 PRO A 395 38.038 −14.874 −8.185 1.00 0.00 H
ATOM 6235 HD2 PRO A 395 36.141 −15.881 −9.132 1.00 0.00 H
ATOM 6236 HD3 PRO A 395 35.067 −15.294 −7.826 1.00 0.00 H
ATOM 6237 N ALA A 396 37.124 −17.283 −3.752 1.00 0.00 N
ATOM 6238 CA ALA A 396 36.645 −17.460 −2.380 1.00 0.00 C
ATOM 6239 C ALA A 396 36.486 −16.114 −1.646 1.00 0.00 C
ATOM 6240 O ALA A 396 35.415 −15.854 −1.093 1.00 0.00 O
ATOM 6241 CB ALA A 396 37.609 −18.396 −1.641 1.00 0.00 C
ATOM 6242 H ALA A 396 38.093 −17.485 −3.943 1.00 0.00 H
ATOM 6243 HA ALA A 396 35.666 −17.941 −2.410 1.00 0.00 H

ATOM	6244	HB1	ALA	A	396	37.249	−18.563	−0.624	1.00	0.00	H
ATOM	6245	HB2	ALA	A	396	37.660	−19.358	−2.153	1.00	0.00	H
ATOM	6246	HB3	ALA	A	396	38.608	−17.958	−1.594	1.00	0.00	H
ATOM	6247	N	GLN	A	397	37.465	−15.219	−1.819	1.00	0.00	N
ATOM	6248	CA	GLN	A	397	37.532	−13.847	−1.299	1.00	0.00	C
ATOM	6249	C	GLN	A	397	37.956	−12.869	−2.408	1.00	0.00	C
ATOM	6250	O	GLN	A	397	38.484	−13.291	−3.435	1.00	0.00	O
ATOM	6251	CB	GLN	A	397	38.544	−13.777	−0.135	1.00	0.00	C
ATOM	6252	CG	GLN	A	397	38.166	−14.568	1.130	1.00	0.00	C
ATOM	6253	CD	GLN	A	397	37.017	−13.948	1.924	1.00	0.00	C
ATOM	6254	OE1	GLN	A	397	36.179	−13.225	1.415	1.00	0.00	O
ATOM	6255	NE2	GLN	A	397	36.926	−14.213	3.207	1.00	0.00	N
ATOM	6256	H	GLN	A	397	38.251	−15.493	−2.391	1.00	0.00	H
ATOM	6257	HA	GLN	A	397	36.547	−13.538	−0.949	1.00	0.00	H
ATOM	6258	HB2	GLN	A	397	39.505	−14.149	−0.494	1.00	0.00	H
ATOM	6259	HB3	GLN	A	397	38.690	−12.735	0.154	1.00	0.00	H
ATOM	6260	HG2	GLN	A	397	37.911	−15.596	0.877	1.00	0.00	H
ATOM	6261	HG3	GLN	A	397	39.044	−14.599	1.776	1.00	0.00	H
ATOM	6262	HE21	GLN	A	397	37.627	−14.756	3.675	1.00	0.00	H
ATOM	6263	HE22	GLN	A	397	36.191	−13.739	3.704	1.00	0.00	H
ATOM	6264	N	CYS	A	398	37.773	−11.566	−2.191	1.00	0.00	N
ATOM	6265	CA	CYS	A	398	38.160	−10.496	−3.125	1.00	0.00	C
ATOM	6266	C	CYS	A	398	39.632	−10.045	−2.981	1.00	0.00	C
ATOM	6267	O	CYS	A	398	39.888	−8.853	−2.844	1.00	0.00	O
ATOM	6268	CB	CYS	A	398	37.188	−9.321	−2.957	1.00	0.00	C
ATOM	6269	SG	CYS	A	398	35.435	−9.738	−3.106	1.00	0.00	S
ATOM	6270	H	CYS	A	398	37.355	−11.281	−1.317	1.00	0.00	H
ATOM	6271	HA	CYS	A	398	38.051	−10.871	−4.143	1.00	0.00	H
ATOM	6272	HB2	CYS	A	398	37.353	−8.867	−1.979	1.00	0.00	H
ATOM	6273	HB3	CYS	A	398	37.423	−8.565	−3.709	1.00	0.00	H

ATOM 6274 N SER A 399 40.535 −11.001 −2.749 1.00 0.00 N
ATOM 6275 CA SER A 399 41.901 −10.772 −2.258 1.00 0.00 C
ATOM 6276 C SER A 399 42.910 −10.475 −3.391 1.00 0.00 C
ATOM 6277 O SER A 399 42.968 −9.329 −3.838 1.00 0.00 O
ATOM 6278 CB SER A 399 42.249 −11.915 −1.295 1.00 0.00 C
ATOM 6279 OG SER A 399 43.590 −11.928 −0.882 1.00 0.00 O
ATOM 6280 H SER A 399 40.258 −11.957 −2.913 1.00 0.00 H
ATOM 6281 HA SER A 399 41.897 −9.868 −1.649 1.00 0.00 H
ATOM 6282 HB2 SER A 399 41.618 −11.821 −0.410 1.00 0.00 H
ATOM 6283 HB3 SER A 399 42.020 −12.870 −1.769 1.00 0.00 H
ATOM 6284 HG SER A 399 44.012 −12.649 −1.406 1.00 0.00 H
ATOM 6285 N GLU A 400 43.592 −11.479 −3.961 1.00 0.00 N
ATOM 6286 CA GLU A 400 44.658 −11.334 −4.988 1.00 0.00 C
ATOM 6287 C GLU A 400 44.333 −11.947 −6.357 1.00 0.00 C
ATOM 6288 O GLU A 400 44.817 −11.410 −7.383 1.00 0.00 O
ATOM 6289 CB GLU A 400 46.039 −11.811 −4.466 1.00 0.00 C
ATOM 6290 CG GLU A 400 46.127 −12.750 −3.244 1.00 0.00 C
ATOM 6291 CD GLU A 400 45.187 −13.971 −3.281 1.00 0.00 C
ATOM 6292 OE1 GLU A 400 45.599 −15.037 −3.796 1.00 0.00 O
ATOM 6293 OE2 GLU A 400 44.158 −13.899 −2.558 1.00 0.00 O
ATOM 6294 OXT GLU A 400 43.919 −13.121 −6.402 1.00 0.00 O
ATOM 6295 H GLU A 400 43.493 −12.407 −3.555 1.00 0.00 H
ATOM 6296 HA GLU A 400 44.775 −10.274 −5.207 1.00 0.00 H
ATOM 6297 HB2 GLU A 400 46.570 −12.292 −5.288 1.00 0.00 H
ATOM 6298 HB3 GLU A 400 46.613 −10.916 −4.215 1.00 0.00 H
ATOM 6299 HG2 GLU A 400 45.912 −12.157 −2.351 1.00 0.00 H
ATOM 6300 HG3 GLU A 400 47.163 −13.084 −3.144 1.00 0.00 H
ATOM 6301 HXT GLU A 400 43.075 −13.195 −5.886 1.00 0.00 H
TER 6302 HXT GLU A 400
HETATM 6303 C1 UNK 1 27.871 −20.347 −33.669 1.00 0.00 C

HETATM	6304	C2	UNK	1	28.417	−18.954	−33.794	1.00	0.00	C
HETATM	6305	N3	UNK	1	29.580	−18.802	−34.558	1.00	0.00	N
HETATM	6306	C4	UNK	1	26.589	−20.698	−32.982	1.00	0.00	C
HETATM	6307	N5	UNK	1	26.056	−21.926	−33.283	1.00	0.00	N
HETATM	6308	C6	UNK	1	28.553	−21.356	−34.242	1.00	0.00	C
HETATM	6309	C7	UNK	1	30.136	−17.459	−34.755	1.00	0.00	C
HETATM	6310	C8	UNK	1	29.163	−16.503	−35.457	1.00	0.00	C
HETATM	6311	C9	UNK	1	30.218	−19.882	−35.146	1.00	0.00	C
HETATM	6312	C10	UNK	1	29.405	−13.402	−37.312	1.00	0.00	C
HETATM	6313	C11	UNK	1	28.446	−12.420	−38.033	1.00	0.00	C
HETATM	6314	O12	UNK	1	27.839	−18.008	−33.255	1.00	0.00	O
HETATM	6315	C13	UNK	1	29.756	−21.125	−34.995	1.00	0.00	C
HETATM	6316	O14	UNK	1	26.069	−19.941	−32.164	1.00	0.00	O
HETATM	6317	N15	UNK	1	29.544	−15.177	−35.475	1.00	0.00	N
HETATM	6318	C16	UNK	1	28.729	−14.148	−36.111	1.00	0.00	C
HETATM	6319	C17	UNK	1	27.277	−12.900	−38.661	1.00	0.00	C
HETATM	6320	O18	UNK	1	28.126	−16.901	−35.988	1.00	0.00	O
HETATM	6321	C19	UNK	1	26.407	−12.034	−39.323	1.00	0.00	C
HETATM	6322	C20	UNK	1	24.866	−22.454	−32.635	1.00	0.00	C
HETATM	6323	F21	UNK	1	25.304	−12.522	−39.911	1.00	0.00	F
HETATM	6324	C22	UNK	1	28.694	−11.036	−38.120	1.00	0.00	C
HETATM	6325	C23	UNK	1	27.815	−10.174	−38.786	1.00	0.00	C
HETATM	6326	C24	UNK	1	29.894	−14.470	−38.321	1.00	0.00	C
HETATM	6327	C25	UNK	1	30.621	−12.600	−36.799	1.00	0.00	C
HETATM	6328	C26	UNK	1	26.667	−10.675	−39.388	1.00	0.00	C
HETATM	6329	C27	UNK	1	25.153	−23.835	−32.030	1.00	0.00	C
HETATM	6330	C28	UNK	1	23.703	−22.522	−33.630	1.00	0.00	C
HETATM	6331	C29	UNK	1	23.904	−24.447	−31.403	1.00	0.00	C
HETATM	6332	C30	UNK	1	22.455	−23.134	−32.999	1.00	0.00	C
HETATM	6333	C31	UNK	1	22.746	−24.504	−32.395	1.00	0.00	C

HETATM	6334	H1	UNK	1	26.542	−22.503	−33.970	1.00	0.00	H
HETATM	6335	H2	UNK	1	28.202	−22.383	−34.184	1.00	0.00	H
HETATM	6336	H3	UNK	1	30.373	−17.038	−33.771	1.00	0.00	H
HETATM	6337	H4	UNK	1	31.076	−17.504	−35.314	1.00	0.00	H
HETATM	6338	H5	UNK	1	31.117	−19.685	−35.726	1.00	0.00	H
HETATM	6339	H6	UNK	1	30.261	−21.973	−35.447	1.00	0.00	H
HETATM	6340	H7	UNK	1	30.448	−14.915	−35.098	1.00	0.00	H
HETATM	6341	H8	UNK	1	28.432	−13.429	−35.338	1.00	0.00	H
HETATM	6342	H9	UNK	1	27.818	−14.657	−36.450	1.00	0.00	H
HETATM	6343	H10	UNK	1	27.021	−13.955	−38.658	1.00	0.00	H
HETATM	6344	H11	UNK	1	24.592	−21.768	−31.823	1.00	0.00	H
HETATM	6345	H12	UNK	1	29.579	−10.585	−37.678	1.00	0.00	H
HETATM	6346	H13	UNK	1	28.029	−9.108	−38.843	1.00	0.00	H
HETATM	6347	H14	UNK	1	30.438	−14.030	−39.163	1.00	0.00	H
HETATM	6348	H15	UNK	1	29.049	−15.035	−38.734	1.00	0.00	H
HETATM	6349	H16	UNK	1	30.578	−15.185	−37.852	1.00	0.00	H
HETATM	6350	H17	UNK	1	31.135	−12.067	−37.606	1.00	0.00	H
HETATM	6351	H18	UNK	1	31.369	−13.251	−36.334	1.00	0.00	H
HETATM	6352	H19	UNK	1	30.315	−11.852	−36.058	1.00	0.00	H
HETATM	6353	H20	UNK	1	25.980	−10.018	−39.909	1.00	0.00	H
HETATM	6354	H21	UNK	1	25.538	−24.508	−32.807	1.00	0.00	H
HETATM	6355	H22	UNK	1	25.941	−23.759	−31.270	1.00	0.00	H
HETATM	6356	H23	UNK	1	23.470	−21.517	−34.002	1.00	0.00	H
HETATM	6357	H24	UNK	1	23.994	−23.116	−34.507	1.00	0.00	H
HETATM	6358	H25	UNK	1	24.130	−25.457	−31.042	1.00	0.00	H
HETATM	6359	H26	UNK	1	23.607	−23.854	−30.529	1.00	0.00	H
HETATM	6360	H27	UNK	1	22.075	−22.464	−32.218	1.00	0.00	H
HETATM	6361	H28	UNK	1	21.666	−23.224	−33.754	1.00	0.00	H
HETATM	6362	H29	UNK	1	22.987	−25.213	−33.197	1.00	0.00	H
HETATM	6363	H30	UNK	1	21.849	−24.884	−31.892	1.00	0.00	H

TER 6364 H30 UNK 1

CONECT 27 6269

CONECT 335 693

CONECT 693 335

CONECT 1346 1552

CONECT 1552 1346

CONECT 2844 3048

CONECT 2923 4674

CONECT 3048 2844

CONECT 4576 4729

CONECT 4674 2923

CONECT 4729 4576

CONECT 5685 5841

CONECT 5841 5685

CONECT 6269 27

CONECT 6303 6304 6306 6308

CONECT 6304 6303 6305 6314

CONECT 6305 6304 6309 6311

CONECT 6306 6303 6307 6316

CONECT 6307 6306 6322 6334

CONECT 6308 6303 6315 6335

CONECT 6309 6305 6310 6336 6337

CONECT 6310 6309 6317 6320

CONECT 6311 6305 6315 6338

CONECT 6312 6313 6318 6326 6327

CONECT 6313 6312 6319 6324

CONECT 6314 6304

CONECT 6315 6308 6311 6339

CONECT 6316 6306

CONECT 6317 6310 6318 6340

CONECT 6318 6312 6317 6341 6342

CONECT 6319 6313 6321 6343

CONECT 6320 6310

CONECT 6321 6319 6323 6328

CONECT 6322 6307 6329 6330 6344

CONECT 6323 6321

CONECT 6324 6313 6325 6345

CONECT 6325 6324 6328 6346

CONECT 6326 6312 6347 6348 6349

CONECT 6327 6312 6350 6351 6352

CONECT 6328 6321 6325 6353

CONECT 6329 6322 6331 6354 6355

CONECT 6330 6322 6332 6356 6357

CONECT 6331 6329 6333 6358 6359

CONECT 6332 6330 6333 6360 6361

CONECT 6333 6331 6332 6362 6363

CONECT 6334 6307

CONECT 6335 6308

CONECT 6336 6309

CONECT 6337 6309

CONECT 6338 6311

CONECT 6339 6315

CONECT 6340 6317

CONECT 6341 6318

CONECT 6342 6318

CONECT 6343 6319

CONECT 6344 6322

CONECT 6345 6324

CONECT 6346 6325

CONECT 6347 6326

CONECT 6348 6326

CONECT 6349 6326

CONECT 6350 6327

CONECT 6351 6327

CONECT 6352 6327

CONECT 6353 6328

CONECT 6354 6329

CONECT 6355 6329

CONECT 6356 6330

CONECT 6357 6330

CONECT 6358 6331

CONECT 6359 6331

CONECT 6360 6332

CONECT 6361 6332

CONECT 6362 6333

CONECT 6363 6333

MASTER 0 0 0 0 0 0 0 3 6362 2 75 0

END

Top1−combine−coordinates

ATOM 1 N ILE A 188 32.653 −16.268 −1.319 1.00 51.04 N1+

ATOM 2 CA ILE A 188 32.633 −15.941 −2.763 1.00 51.24 C

ATOM 3 C ILE A 188 32.837 −14.441 −2.909 1.00 51.38 C

ATOM 4 O ILE A 188 32.290 −13.705 −2.093 1.00 51.55 O

ATOM 5 CB ILE A 188 31.347 −16.438 −3.468 1.00 51.49 C

ATOM 6 CG1 ILE A 188 31.341 −16.009 −4.953 1.00 51.69 C

ATOM 7 CG2 ILE A 188 30.059 −15.978 −2.757 1.00 51.70 C

ATOM 8 CD1 ILE A 188 30.325 −16.768 −5.817 1.00 51.91 C

ATOM 9 1HD1 ILE A 188 30.382 −16.410 −6.845 1.00 0.00 H

ATOM 10 2HD1 ILE A 188 29.320 −16.600 −5.430 1.00 0.00 H

ATOM 11 HT1 ILE A 188 32.449 −17.244 −1.163 1.00 0.00 H

ATOM 12 HT2 ILE A 188 31.970 −15.687 −0.848 1.00 0.00 H

ATOM 13 HT3 ILE A 188 33.568 −16.041 −0.940 1.00 0.00 H

ATOM 14 HA ILE A 188 33.483 −16.440 −3.229 1.00 51.50 H

ATOM 15 HB ILE A 188 31.362 −17.528 −3.443 1.00 51.50 H

ATOM 16 1HG1 ILE A 188 31.106 −14.946 −5.002 1.00 51.50 H

ATOM 17 2HG1 ILE A 188 32.336 −16.181 −5.363 1.00 51.50 H

ATOM 18 1HG2 ILE A 188 29.191 −16.356 −3.296 1.00 51.50 H

ATOM 19 2HG2 ILE A 188 30.025 −14.889 −2.734 1.00 51.50 H

ATOM 20 3HG2 ILE A 188 30.049 −16.363 −1.737 1.00 51.50 H

ATOM 21 3HD1 ILE A 188 30.550 −17.834 −5.791 1.00 51.50 H

ATOM 22 N CYS A 189 33.675 −13.977 −3.840 1.00 23.07 N

ATOM 23 CA CYS A 189 33.809 −12.535 −4.055 1.00 23.21 C

ATOM 24 C CYS A 189 32.574 −11.948 −4.761 1.00 23.54 C

ATOM 25 O CYS A 189 32.343 −12.226 −5.937 1.00 23.69 O

ATOM 26 CB CYS A 189 35.092 −12.191 −4.815 1.00 23.15 C

ATOM 27 SG CYS A 189 35.277 −10.404 −5.017 1.00 23.21 S

ATOM 28 HN CYS A 189 34.138 −14.619 −4.473 1.00 0.00 H

ATOM 29 HA CYS A 189 33.877 −12.065 −3.074 1.00 23.31 H

ATOM 30 HB1 CYS A 189 35.947 −12.576 −4.260 1.00 23.31 H

ATOM 31 HB2 CYS A 189 35.057 −12.658 −5.799 1.00 23.31 H

ATOM 32 N LEU A 190 31.841 −11.084 −4.056 1.00 50.65 N

ATOM 33 CA LEU A 190 30.699 −10.317 −4.576 1.00 50.98 C

ATOM 34 C LEU A 190 31.032 −8.834 −4.838 1.00 51.13 C

ATOM 35 O LEU A 190 30.234 −8.131 −5.451 1.00 51.41 O

ATOM 36 CB LEU A 190 29.536 −10.434 −3.572 1.00 51.07 C

ATOM	37	CG	LEU	A	190	29.029	−11.868	−3.324	1.00	51.02	C
ATOM	38	CD1	LEU	A	190	28.068	−11.866	−2.138	1.00	51.16	C
ATOM	39	CD2	LEU	A	190	28.323	−12.447	−4.551	1.00	51.19	C
ATOM	40	HN	LEU	A	190	32.062	−10.971	−3.078	1.00	0.00	H
ATOM	41	HA	LEU	A	190	30.385	−10.768	−5.517	1.00	51.08	H
ATOM	42	HB1	LEU	A	190	29.870	−10.024	−2.619	1.00	51.08	H
ATOM	43	HB2	LEU	A	190	28.703	−9.842	−3.950	1.00	51.08	H
ATOM	44	HG	LEU	A	190	29.882	−12.502	−3.081	1.00	51.08	H
ATOM	45	1HD1	LEU	A	190	27.708	−12.879	−1.960	1.00	51.08	H
ATOM	46	2HD1	LEU	A	190	27.223	−11.213	−2.356	1.00	51.08	H
ATOM	47	3HD1	LEU	A	190	28.587	−11.504	−1.250	1.00	51.08	H
ATOM	48	1HD2	LEU	A	190	27.982	−13.459	−4.331	1.00	51.08	H
ATOM	49	2HD2	LEU	A	190	29.017	−12.472	−5.392	1.00	51.08	H
ATOM	50	3HD2	LEU	A	190	27.466	−11.823	−4.806	1.00	51.08	H
ATOM	51	N	GLN	A	191	32.174	−8.356	−4.332	1.00	23.97	N
ATOM	52	CA	GLN	A	191	32.625	−6.966	−4.438	1.00	24.11	C
ATOM	53	C	GLN	A	191	33.318	−6.704	−5.783	1.00	24.14	C
ATOM	54	O	GLN	A	191	34.098	−7.532	−6.258	1.00	23.95	O
ATOM	55	CB	GLN	A	191	33.568	−6.659	−3.256	1.00	23.92	C
ATOM	56	CG	GLN	A	191	33.907	−5.174	−3.041	1.00	24.14	C
ATOM	57	CD	GLN	A	191	32.715	−4.319	−2.625	1.00	24.38	C
ATOM	58	OE1	GLN	A	191	31.658	−4.797	−2.257	1.00	24.39	O
ATOM	59	NE2	GLN	A	191	32.833	−3.013	−2.682	1.00	24.59	N
ATOM	60	HN	GLN	A	191	32.809	−9.008	−3.905	1.00	0.00	H
ATOM	61	1HE2	GLN	A	191	33.685	−2.587	−2.999	1.00	0.00	H
ATOM	62	2HE2	GLN	A	191	32.019	−2.489	−2.414	1.00	0.00	H
ATOM	63	HA	GLN	A	191	31.755	−6.314	−4.365	1.00	24.18	H

ATOM 64 HB1 GLN A 191 33.096 −7.030 −2.346 1.00 24.18 H

ATOM 65 HB2 GLN A 191 34.502 −7.194 −3.425 1.00 24.18 H

ATOM 66 HG1 GLN A 191 34.666 −5.105 −2.262 1.00 24.18 H

ATOM 67 HG2 GLN A 191 34.306 −4.775 −3.974 1.00 24.18 H

ATOM 68 N LYS A 192 33.179 −5.485 −6.311 1.00 24.41 N

ATOM 69 CA LYS A 192 33.993 −4.990 −7.427 1.00 24.45 C

ATOM 70 C LYS A 192 35.463 −4.805 −7.021 1.00 24.22 C

ATOM 71 O LYS A 192 35.744 −4.157 −6.016 1.00 24.15 O

ATOM 72 CB LYS A 192 33.343 −3.703 −7.956 1.00 24.77 C

ATOM 73 CG LYS A 192 34.110 −3.120 −9.144 1.00 24.86 C

ATOM 74 CD LYS A 192 33.252 −2.138 −9.952 1.00 25.21 C

ATOM 75 CE LYS A 192 34.025 −1.589 −11.158 1.00 25.32 C

ATOM 76 NZ LYS A 192 34.494 −2.678 −12.052 1.00 25.19 N1+

ATOM 77 HN LYS A 192 32.506 −4.854 −5.897 1.00 0.00 H

ATOM 78 HZ1 LYS A 192 34.932 −2.323 −12.889 1.00 0.00 H

ATOM 79 HZ2 LYS A 192 35.137 −3.292 −11.566 1.00 0.00 H

ATOM 80 HZ3 LYS A 192 33.708 −3.272 −12.317 1.00 0.00 H

ATOM 81 HA LYS A 192 33.957 −5.734 −8.223 1.00 24.73 H

ATOM 82 HB1 LYS A 192 32.324 −3.927 −8.271 1.00 24.73 H

ATOM 83 HB2 LYS A 192 33.322 −2.965 −7.155 1.00 24.73 H

ATOM 84 HG1 LYS A 192 34.990 −2.595 −8.772 1.00 24.73 H

ATOM 85 HG2 LYS A 192 34.421 −3.935 −9.797 1.00 24.73 H

ATOM 86 HD1 LYS A 192 32.360 −2.654 −10.307 1.00 24.73 H

ATOM 87 HD2 LYS A 192 32.961 −1.308 −9.309 1.00 24.73 H

ATOM 88 HE1 LYS A 192 33.372 −0.925 −11.724 1.00 24.73 H

ATOM 89 HE2 LYS A 192 34.889 −1.030 −10.799 1.00 24.73 H

ATOM 90 N THR A 193 36.402 −5.279 −7.844 1.00 27.90 N

```
ATOM     91  CA  THR A 193      37.856  −5.126  −7.630  1.00 27.72           C
ATOM     92  C   THR A 193      38.646  −5.237  −8.944  1.00 27.67           C
ATOM     93  O   THR A 193      38.110  −5.652  −9.971  1.00 27.64           O
ATOM     94  CB  THR A 193      38.375  −6.122  −6.569  1.00 27.43           C
ATOM     95  OG1 THR A 193      39.738  −5.865  −6.316  1.00 27.25           O
ATOM     96  CG2 THR A 193      38.269  −7.592  −6.974  1.00 27.28           C
ATOM     97  HN  THR A 193      36.107  −5.742  −8.700  1.00  0.00           H
ATOM     98  HG1 THR A 193      40.069  −6.475  −5.641  1.00  0.00           H
ATOM     99  HA  THR A 193      38.021  −4.122  −7.238  1.00 27.56           H
ATOM    100  HB  THR A 193      37.814  −5.973  −5.646  1.00 27.56           H
ATOM    101 1HG2 THR A 193      38.656  −8.219  −6.171  1.00 27.56           H
ATOM    102 2HG2 THR A 193      38.851  −7.763  −7.880  1.00 27.56           H
ATOM    103 3HG2 THR A 193      37.225  −7.844  −7.160  1.00 27.56           H
ATOM    104  N   THR A 194      39.918  −4.835  −8.924  1.00 23.90           N
ATOM    105  CA  THR A 194      40.904  −5.065  −9.996  1.00 23.87           C
ATOM    106  C   THR A 194      41.966  −6.108  −9.622  1.00 23.58           C
ATOM    107  O   THR A 194      42.909  −6.306 −10.383  1.00 23.54           O
ATOM    108  CB  THR A 194      41.565  −3.750 −10.439  1.00 24.10           C
ATOM    109  OG1 THR A 194      42.236  −3.148  −9.357  1.00 24.17           O
ATOM    110  CG2 THR A 194      40.543  −2.746 −10.975  1.00 24.37           C
ATOM    111  HN  THR A 194      40.283  −4.513  −8.035  1.00  0.00           H
ATOM    112  HG1 THR A 194      43.081  −3.597  −9.254  1.00  0.00           H
ATOM    113  HA  THR A 194      40.359  −5.456 −10.855  1.00 23.93           H
ATOM    114  HB  THR A 194      42.289  −3.968 −11.224  1.00 23.93           H
ATOM    115 1HG2 THR A 194      41.054  −1.832 −11.276  1.00 23.93           H
ATOM    116 2HG2 THR A 194      39.816  −2.515 −10.196  1.00 23.93           H
ATOM    117 3HG2 THR A 194      40.029  −3.174 −11.835  1.00 23.93           H
```

ATOM 118 N SER A 195 41.831 −6.792 −8.479 1.00 23.39 N

ATOM 119 CA SER A 195 42.647 −7.967 −8.139 1.00 23.10 C

ATOM 120 C SER A 195 42.491 −9.069 −9.196 1.00 23.09 C

ATOM 121 O SER A 195 41.382 −9.338 −9.659 1.00 23.24 O

ATOM 122 CB SER A 195 42.241 −8.541 −6.780 1.00 22.92 C

ATOM 123 OG SER A 195 42.255 −7.570 −5.754 1.00 22.92 O

ATOM 124 HN SER A 195 41.086 −6.545 −7.842 1.00 0.00 H

ATOM 125 HG SER A 195 42.543 −8.039 −4.942 1.00 0.00 H

ATOM 126 HA SER A 195 43.694 −7.667 −8.095 1.00 23.11 H

ATOM 127 HB1 SER A 195 41.234 −8.949 −6.860 1.00 23.11 H

ATOM 128 HB2 SER A 195 42.936 −9.338 −6.516 1.00 23.11 H

ATOM 129 N THR A 196 43.545 −9.851 −9.436 1.00 41.71 N

ATOM 130 CA THR A 196 43.582 −10.945 −10.433 1.00 41.69 C

ATOM 131 C THR A 196 42.945 −12.254 −9.931 1.00 41.49 C

ATOM 132 O THR A 196 43.398 −13.354 −10.246 1.00 41.34 O

ATOM 133 CB THR A 196 45.010 −11.166 −10.966 1.00 41.65 C

ATOM 134 OG1 THR A 196 45.888 −11.607 −9.943 1.00 41.41 O

ATOM 135 CG2 THR A 196 45.594 −9.902 −11.599 1.00 41.87 C

ATOM 136 HN THR A 196 44.358 −9.740 −8.843 1.00 0.00 H

ATOM 137 HG1 THR A 196 45.580 −11.264 −9.081 1.00 0.00 H

ATOM 138 HA THR A 196 42.982 −10.613 −11.280 1.00 41.59 H

ATOM 139 HB THR A 196 44.969 −11.940 −11.733 1.00 41.59 H

ATOM 140 1HG2 THR A 196 46.602 −10.108 −11.960 1.00 41.59 H

ATOM 141 2HG2 THR A 196 45.631 −9.106 −10.855 1.00 41.59 H

ATOM 142 3HG2 THR A 196 44.966 −9.590 −12.434 1.00 41.59 H

ATOM 143 N ILE A 197 41.897 −12.159 −9.110 1.00 23.24 N

ATOM 144 CA ILE A 197 41.276 −13.273 −8.367 1.00 23.08 C

ATOM	145	C	ILE	A	197	40.673	−14.378	−9.256	1.00	23.12	C
ATOM	146	O	ILE	A	197	40.500	−15.519	−8.812	1.00	22.96	O
ATOM	147	CB	ILE	A	197	40.227	−12.716	−7.379	1.00	23.15	C
ATOM	148	CG1	ILE	A	197	39.159	−11.831	−8.065	1.00	23.43	C
ATOM	149	CG2	ILE	A	197	40.916	−12.004	−6.207	1.00	23.15	C
ATOM	150	CD1	ILE	A	197	38.103	−11.279	−7.101	1.00	23.57	C
ATOM	151	HN	ILE	A	197	41.583	−11.220	−8.901	1.00	0.00	H
ATOM	152	1HG2	ILE	A	197	40.162	−11.618	−5.521	1.00	0.00	H
ATOM	153	2HG2	ILE	A	197	41.518	−11.178	−6.586	1.00	0.00	H
ATOM	154	HA	ILE	A	197	42.062	−13.736	−7.771	1.00	23.21	H
ATOM	155	HB	ILE	A	197	39.702	−13.575	−6.960	1.00	23.21	H
ATOM	156	1HG1	ILE	A	197	39.663	−10.990	−8.541	1.00	23.21	H
ATOM	157	2HG1	ILE	A	197	38.653	−12.428	−8.824	1.00	23.21	H
ATOM	158	3HG2	ILE	A	197	41.559	−12.709	−5.680	1.00	23.21	H
ATOM	159	1HD1	ILE	A	197	37.389	−10.669	−7.654	1.00	23.21	H
ATOM	160	2HD1	ILE	A	197	38.589	−10.669	−6.340	1.00	23.21	H
ATOM	161	3HD1	ILE	A	197	37.579	−12.107	−6.623	1.00	23.21	H
ATOM	162	N	LEU	A	198	40.408	−14.076	−10.529	1.00	22.85	N
ATOM	163	CA	LEU	A	198	40.169	−15.065	−11.579	1.00	22.95	C
ATOM	164	C	LEU	A	198	41.512	−15.603	−12.095	1.00	22.89	C
ATOM	165	O	LEU	A	198	42.148	−14.989	−12.945	1.00	22.95	O
ATOM	166	CB	LEU	A	198	39.368	−14.411	−12.718	1.00	23.21	C
ATOM	167	CG	LEU	A	198	37.928	−14.021	−12.357	1.00	23.35	C
ATOM	168	CD1	LEU	A	198	37.405	−13.062	−13.424	1.00	23.59	C
ATOM	169	CD2	LEU	A	198	37.012	−15.243	−12.273	1.00	23.40	C
ATOM	170	HN	LEU	A	198	40.670	−13.148	−10.830	1.00	0.00	H
ATOM	171	HA	LEU	A	198	39.590	−15.891	−11.165	1.00	23.15	H

ATOM	172	HB1	LEU	A	198	39.895	−13.509	−13.027	1.00	23.15	H
ATOM	173	HB2	LEU	A	198	39.329	−15.113	−13.551	1.00	23.15	H
ATOM	174	HG	LEU	A	198	37.930	−13.512	−11.393	1.00	23.15	H
ATOM	175	1HD1	LEU	A	198	36.382	−12.774	−13.184	1.00	23.15	H
ATOM	176	2HD1	LEU	A	198	37.425	−13.554	−14.396	1.00	23.15	H
ATOM	177	3HD1	LEU	A	198	38.035	−12.173	−13.454	1.00	23.15	H
ATOM	178	1HD2	LEU	A	198	36.002	−14.924	−12.015	1.00	23.15	H
ATOM	179	2HD2	LEU	A	198	37.384	−15.924	−11.508	1.00	23.15	H
ATOM	180	3HD2	LEU	A	198	36.997	−15.753	−13.236	1.00	23.15	H
ATOM	181	N	LYS	A	199	41.921	−16.783	−11.613	1.00	22.81	N
ATOM	182	CA	LYS	A	199	43.184	−17.445	−11.998	1.00	22.73	C
ATOM	183	C	LYS	A	199	42.898	−18.715	−12.827	1.00	22.86	C
ATOM	184	O	LYS	A	199	42.656	−19.768	−12.220	1.00	22.78	O
ATOM	185	CB	LYS	A	199	44.060	−17.686	−10.749	1.00	22.47	C
ATOM	186	CG	LYS	A	199	44.320	−16.380	−9.971	1.00	22.32	C
ATOM	187	CD	LYS	A	199	45.403	−16.508	−8.886	1.00	22.05	C
ATOM	188	CE	LYS	A	199	45.335	−15.373	−7.849	1.00	21.96	C
ATOM	189	NZ	LYS	A	199	45.433	−14.022	−8.445	1.00	22.09	N1+
ATOM	190	HN	LYS	A	199	41.367	−17.191	−10.876	1.00	0.00	H
ATOM	191	HZ1	LYS	A	199	45.122	−13.338	−7.743	1.00	0.00	H
ATOM	192	HZ2	LYS	A	199	46.353	−13.750	−8.749	1.00	0.00	H
ATOM	193	HZ3	LYS	A	199	44.777	−13.890	−9.207	1.00	0.00	H
ATOM	194	HA	LYS	A	199	43.726	−16.754	−12.644	1.00	22.45	H
ATOM	195	HB1	LYS	A	199	43.552	−18.392	−10.093	1.00	22.45	H
ATOM	196	HB2	LYS	A	199	45.016	−18.105	−11.064	1.00	22.45	H
ATOM	197	HG1	LYS	A	199	44.634	−15.614	−10.681	1.00	22.45	H
ATOM	198	HG2	LYS	A	199	43.389	−16.074	−9.493	1.00	22.45	H

ATOM	199	HD1	LYS	A	199	45.271	−17.460	−8.371	1.00	22.45	H
ATOM	200	HD2	LYS	A	199	46.382	−16.486	−9.365	1.00	22.45	H
ATOM	201	HE1	LYS	A	199	44.386	−15.448	−7.318	1.00	22.45	H
ATOM	202	HE2	LYS	A	199	46.157	−15.500	−7.145	1.00	22.45	H
ATOM	203	N	PRO	A	200	42.721	−18.603	−14.161	1.00	46.45	N
ATOM	204	CA	PRO	A	200	42.211	−19.673	−15.031	1.00	46.62	C
ATOM	205	C	PRO	A	200	43.206	−20.839	−15.209	1.00	46.57	C
ATOM	206	O	PRO	A	200	44.264	−20.883	−14.575	1.00	46.50	O
ATOM	207	CB	PRO	A	200	41.911	−18.964	−16.367	1.00	46.83	C
ATOM	208	CG	PRO	A	200	42.965	−17.865	−16.425	1.00	46.78	C
ATOM	209	CD	PRO	A	200	43.019	−17.421	−14.967	1.00	46.57	C
ATOM	210	HA	PRO	A	200	41.280	−20.058	−14.615	1.00	46.62	H
ATOM	211	HB1	PRO	A	200	42.017	−19.651	−17.206	1.00	46.62	H
ATOM	212	HB2	PRO	A	200	40.906	−18.542	−16.368	1.00	46.62	H
ATOM	213	HG1	PRO	A	200	43.927	−18.253	−16.759	1.00	46.62	H
ATOM	214	HG2	PRO	A	200	42.655	−17.050	−17.078	1.00	46.62	H
ATOM	215	HD1	PRO	A	200	44.012	−17.042	−14.726	1.00	46.62	H
ATOM	216	HD2	PRO	A	200	42.278	−16.643	−14.783	1.00	46.62	H
ATOM	217	N	ARG	A	201	42.864	−21.803	−16.072	1.00	32.66	N
ATOM	218	CA	ARG	A	201	43.782	−22.766	−16.709	1.00	32.66	C
ATOM	219	C	ARG	A	201	43.226	−23.218	−18.056	1.00	32.93	C
ATOM	220	O	ARG	A	201	42.065	−23.608	−18.128	1.00	33.05	O
ATOM	221	CB	ARG	A	201	44.004	−23.992	−15.797	1.00	32.48	C
ATOM	222	CG	ARG	A	201	45.385	−23.988	−15.114	1.00	32.20	C
ATOM	223	CD	ARG	A	201	45.286	−24.303	−13.619	1.00	32.00	C
ATOM	224	NE	ARG	A	201	44.661	−23.182	−12.895	1.00	31.86	N
ATOM	225	CZ	ARG	A	201	44.368	−23.138	−11.612	1.00	31.60	C

ATOM 226 NH1 ARG A 201 44.608 −24.144 −10.816 1.00 31.41 N
ATOM 227 NH2 ARG A 201 43.820 −22.076 −11.098 1.00 31.54 N
ATOM 228 HN ARG A 201 41.900 −21.808 −16.395 1.00 0.00 H
ATOM 229 HE ARG A 201 44.493 −22.336 −13.437 1.00 0.00 H
ATOM 230 1HH1 ARG A 201 45.012 −24.978 −11.206 1.00 0.00 H
ATOM 231 2HH1 ARG A 201 44.381 −24.111 −9.841 1.00 0.00 H
ATOM 232 1HH2 ARG A 201 43.557 −21.300 −11.706 1.00 0.00 H
ATOM 233 2HH2 ARG A 201 43.639 −22.022 −10.120 1.00 0.00 H
ATOM 234 HA ARG A 201 44.742 −22.276 −16.874 1.00 32.22 H
ATOM 235 HB1 ARG A 201 43.234 −23.995 −15.025 1.00 32.22 H
ATOM 236 HB2 ARG A 201 43.917 −24.895 −16.401 1.00 32.22 H
ATOM 237 HG1 ARG A 201 46.015 −24.739 −15.591 1.00 32.22 H
ATOM 238 HG2 ARG A 201 45.835 −23.003 −15.237 1.00 32.22 H
ATOM 239 HD1 ARG A 201 44.682 −25.200 −13.481 1.00 32.22 H
ATOM 240 HD2 ARG A 201 46.287 −24.473 −13.221 1.00 32.22 H
ATOM 241 N LEU A 202 44.095 −23.335 −19.059 1.00 23.67 N
ATOM 242 CA LEU A 202 43.834 −24.121 −20.266 1.00 23.95 C
ATOM 243 C LEU A 202 43.808 −25.615 −19.898 1.00 24.04 C
ATOM 244 O LEU A 202 44.800 −26.138 −19.397 1.00 23.97 O
ATOM 245 CB LEU A 202 44.930 −23.783 −21.300 1.00 24.03 C
ATOM 246 CG LEU A 202 44.926 −24.630 −22.587 1.00 24.32 C
ATOM 247 CD1 LEU A 202 43.662 −24.422 −23.420 1.00 24.44 C
ATOM 248 CD2 LEU A 202 46.127 −24.262 −23.455 1.00 24.39 C
ATOM 249 HN LEU A 202 45.047 −23.052 −18.898 1.00 0.00 H
ATOM 250 HA LEU A 202 42.863 −23.838 −20.672 1.00 24.10 H
ATOM 251 HB1 LEU A 202 44.807 −22.739 −21.587 1.00 24.10 H
ATOM 252 HB2 LEU A 202 45.898 −23.916 −20.816 1.00 24.10 H

ATOM 253 HG LEU A 202 44.998 −25.683 −22.315 1.00 24.10 H

ATOM 254 1HD1 LEU A 202 43.710 −25.042 −24.315 1.00 24.10 H

ATOM 255 2HD1 LEU A 202 43.585 −23.374 −23.708 1.00 24.10 H

ATOM 256 3HD1 LEU A 202 42.788 −24.702 −22.832 1.00 24.10 H

ATOM 257 1HD2 LEU A 202 46.118 −24.865 −24.363 1.00 24.10 H

ATOM 258 2HD2 LEU A 202 47.047 −24.452 −22.903 1.00 24.10 H

ATOM 259 3HD2 LEU A 202 46.075 −23.206 −23.720 1.00 24.10 H

ATOM 260 N ILE A 203 42.720 −26.314 −20.227 1.00 41.12 N

ATOM 261 CA ILE A 203 42.558 −27.771 −20.036 1.00 41.21 C

ATOM 262 C ILE A 203 42.485 −28.521 −21.382 1.00 41.54 C

ATOM 263 O ILE A 203 41.862 −29.575 −21.499 1.00 41.69 O

ATOM 264 CB ILE A 203 41.384 −28.092 −19.078 1.00 41.12 C

ATOM 265 CG1 ILE A 203 40.023 −27.655 −19.658 1.00 41.28 C

ATOM 266 CG2 ILE A 203 41.614 −27.485 −17.683 1.00 40.79 C

ATOM 267 CD1 ILE A 203 38.851 −28.479 −19.122 1.00 41.28 C

ATOM 268 HN ILE A 203 41.935 −25.803 −20.624 1.00 0.00 H

ATOM 269 HA ILE A 203 43.464 −28.118 −19.539 1.00 41.25 H

ATOM 270 HB ILE A 203 41.352 −29.175 −18.958 1.00 41.25 H

ATOM 271 1HG1 ILE A 203 39.857 −26.608 −19.403 1.00 41.25 H

ATOM 272 2HG1 ILE A 203 40.057 −27.764 −20.742 1.00 41.25 H

ATOM 273 1HG2 ILE A 203 40.771 −27.730 −17.036 1.00 41.25 H

ATOM 274 2HG2 ILE A 203 41.704 −26.402 −17.768 1.00 41.25 H

ATOM 275 3HG2 ILE A 203 42.530 −27.893 −17.255 1.00 41.25 H

ATOM 276 1HD1 ILE A 203 37.922 −28.123 −19.568 1.00 41.25 H

ATOM 277 2HD1 ILE A 203 38.797 −28.373 −18.038 1.00 41.25 H

ATOM 278 3HD1 ILE A 203 38.997 −29.529 −19.378 1.00 41.25 H

ATOM 279 N SER A 204 43.075 −27.945 −22.436 1.00 45.79 N

ATOM	280	CA	SER A	204	42.951	−28.358	−23.845	1.00	46.10	C
ATOM	281	C	SER A	204	41.503	−28.349	−24.342	1.00	46.35	C
ATOM	282	O	SER A	204	41.101	−27.443	−25.079	1.00	46.59	O
ATOM	283	CB	SER A	204	43.609	−29.722	−24.096	1.00	46.13	C
ATOM	284	OG	SER A	204	42.722	−30.771	−23.762	1.00	46.07	O
ATOM	285	HN	SER A	204	43.557	−27.078	−22.251	1.00	0.00	H
ATOM	286	HG	SER A	204	42.459	−30.658	−22.829	1.00	0.00	H
ATOM	287	HA	SER A	204	43.497	−27.626	−24.441	1.00	46.17	H
ATOM	288	HC	SER A	204	40.823	−29.147	−24.044	1.00	46.17	H
ATOM	289	HB1	SER A	204	43.877	−29.802	−25.149	1.00	46.17	H
ATOM	290	HB2	SER A	204	44.507	−29.804	−23.484	1.00	46.17	H
ATOM	291	N	GLU A	213	47.851	−30.099	−40.412	1.00	28.22	N
ATOM	292	CA	GLU A	213	47.876	−29.395	−41.698	1.00	28.20	C
ATOM	293	C	GLU A	213	46.934	−28.166	−41.645	1.00	28.01	C
ATOM	294	O	GLU A	213	46.824	−27.560	−40.580	1.00	27.70	O
ATOM	295	CB	GLU A	213	47.623	−30.413	−42.828	1.00	28.58	C
ATOM	296	CG	GLU A	213	48.077	−29.927	−44.223	1.00	28.63	C
ATOM	297	CD	GLU A	213	46.945	−29.883	−45.261	1.00	28.79	C
ATOM	298	OE1	GLU A	213	45.826	−29.477	−44.874	1.00	29.02	O
ATOM	299	OE2	GLU A	213	47.247	−30.082	−46.457	1.00	28.69	O
ATOM	300	HN1	GLU A	213	46.854	−30.428	−40.204	1.00	0.00	H
ATOM	301	HN2	GLU A	213	48.177	−29.438	−39.636	1.00	28.43	H
ATOM	302	HA	GLU A	213	48.888	−29.015	−41.833	1.00	28.43	H
ATOM	303	HB1	GLU A	213	48.164	−31.329	−42.591	1.00	28.43	H
ATOM	304	HB2	GLU A	213	46.554	−30.622	−42.869	1.00	28.43	H
ATOM	305	HG1	GLU A	213	48.489	−28.923	−44.120	1.00	28.43	H
ATOM	306	HG2	GLU A	213	48.851	−30.602	−44.587	1.00	28.43	H

ATOM 307 N GLY A 214 46.355 −27.704 −42.759 1.00 28.20 N

ATOM 308 CA GLY A 214 45.509 −26.507 −42.815 1.00 28.08 C

ATOM 309 C GLY A 214 44.053 −26.823 −42.474 1.00 28.09 C

ATOM 310 O GLY A 214 43.267 −27.200 −43.347 1.00 28.25 O

ATOM 311 HN GLY A 214 46.346 −28.301 −43.586 1.00 0.00 H

ATOM 312 HA1 GLY A 214 45.887 −25.775 −42.102 1.00 28.15 H

ATOM 313 HA2 GLY A 214 45.553 −26.091 −43.822 1.00 28.15 H

ATOM 314 N VAL A 215 43.654 −26.625 −41.217 1.00 27.92 N

ATOM 315 CA VAL A 215 42.374 −27.122 −40.678 1.00 27.94 C

ATOM 316 C VAL A 215 41.659 −26.060 −39.842 1.00 27.61 C

ATOM 317 O VAL A 215 42.298 −25.317 −39.104 1.00 27.38 O

ATOM 318 CB VAL A 215 42.613 −28.437 −39.896 1.00 28.10 C

ATOM 319 CG1 VAL A 215 41.448 −28.895 −39.004 1.00 27.94 C

ATOM 320 CG2 VAL A 215 42.909 −29.592 −40.866 1.00 28.50 C

ATOM 321 HN VAL A 215 44.332 −26.237 −40.561 1.00 0.00 H

ATOM 322 HA VAL A 215 41.731 −27.358 −41.526 1.00 27.91 H

ATOM 323 HB VAL A 215 43.488 −28.297 −39.262 1.00 27.91 H

ATOM 324 1HG1 VAL A 215 41.718 −29.824 −38.502 1.00 27.91 H

ATOM 325 2HG1 VAL A 215 40.562 −29.058 −39.618 1.00 27.91 H

ATOM 326 3HG1 VAL A 215 41.237 −28.128 −38.259 1.00 27.91 H

ATOM 327 1HG2 VAL A 215 43.075 −30.509 −40.300 1.00 27.91 H

ATOM 328 2HG2 VAL A 215 43.801 −29.359 −41.448 1.00 27.91 H

ATOM 329 3HG2 VAL A 215 42.062 −29.729 −41.538 1.00 27.91 H

ATOM 330 N CYS A 216 40.323 −26.107 −39.822 1.00 35.13 N

ATOM 331 CA CYS A 216 39.522 −25.474 −38.774 1.00 34.87 C

ATOM 332 C CYS A 216 38.521 −26.450 −38.133 1.00 34.94 C

ATOM 333 O CYS A 216 37.795 −27.162 −38.825 1.00 35.18 O

ATOM 334 CB CYS A 216 38.843 −24.203 −39.295 1.00 34.80 C

ATOM 335 SG CYS A 216 37.835 −23.395 −38.019 1.00 34.59 S

ATOM 336 HN CYS A 216 39.865 −26.774 −40.433 1.00 0.00 H

ATOM 337 HA CYS A 216 40.213 −25.166 −37.989 1.00 34.92 H

ATOM 338 HB1 CYS A 216 39.611 −23.506 −39.629 1.00 34.92 H

ATOM 339 HB2 CYS A 216 38.201 −24.467 −40.135 1.00 34.92 H

ATOM 340 N ILE A 217 38.426 −26.415 −36.805 1.00 27.23 N

ATOM 341 CA ILE A 217 37.384 −27.046 −35.988 1.00 27.24 C

ATOM 342 C ILE A 217 36.337 −25.982 −35.618 1.00 27.06 C

ATOM 343 O ILE A 217 36.694 −24.978 −35.010 1.00 26.81 O

ATOM 344 CB ILE A 217 38.037 −27.659 −34.726 1.00 27.10 C

ATOM 345 CG1 ILE A 217 39.206 −28.632 −35.011 1.00 27.29 C

ATOM 346 CG2 ILE A 217 36.981 −28.338 −33.851 1.00 27.10 C

ATOM 347 CD1 ILE A 217 38.840 −29.866 −35.842 1.00 27.63 C

ATOM 348 HN ILE A 217 39.069 −25.793 −36.320 1.00 0.00 H

ATOM 349 HA ILE A 217 36.905 −27.837 −36.565 1.00 27.18 H

ATOM 350 HB ILE A 217 38.446 −26.832 −34.146 1.00 27.18 H

ATOM 351 1HG1 ILE A 217 39.979 −28.082 −35.547 1.00 27.18 H

ATOM 352 2HG1 ILE A 217 39.600 −28.975 −34.054 1.00 27.18 H

ATOM 353 1HG2 ILE A 217 37.459 −28.763 −32.968 1.00 27.18 H

ATOM 354 2HG2 ILE A 217 36.494 −29.132 −34.418 1.00 27.18 H

ATOM 355 3HG2 ILE A 217 36.237 −27.603 −33.543 1.00 27.18 H

ATOM 356 1HD1 ILE A 217 39.726 −30.484 −35.986 1.00 27.18 H

ATOM 357 2HD1 ILE A 217 38.456 −29.551 −36.812 1.00 27.18 H

ATOM 358 3HD1 ILE A 217 38.077 −30.443 −35.320 1.00 27.18 H

ATOM 359 N THR A 218 35.061 −26.173 −35.972 1.00 27.21 N

ATOM 360 CA THR A 218 34.011 −25.139 −35.809 1.00 27.08 C

ATOM 361 C THR A 218 32.593 −25.736 −35.676 1.00 27.25 C

ATOM 362 O THR A 218 32.461 −26.906 −35.325 1.00 27.47 O

ATOM 363 CB THR A 218 34.154 −24.107 −36.946 1.00 27.09 C

ATOM 364 OG1 THR A 218 33.341 −22.973 −36.757 1.00 26.93 O

ATOM 365 CG2 THR A 218 33.810 −24.689 −38.316 1.00 27.38 C

ATOM 366 HN THR A 218 34.825 −26.999 −36.511 1.00 0.00 H

ATOM 367 HG1 THR A 218 33.615 −22.575 −35.888 1.00 0.00 H

ATOM 368 HA THR A 218 34.223 −24.615 −34.877 1.00 27.20 H

ATOM 369 HB THR A 218 35.192 −23.776 −36.972 1.00 27.20 H

ATOM 370 1HG2 THR A 218 33.928 −23.919 −39.078 1.00 27.20 H

ATOM 371 2HG2 THR A 218 32.779 −25.042 −38.313 1.00 27.20 H

ATOM 372 3HG2 THR A 218 34.478 −25.522 −38.535 1.00 27.20 H

ATOM 373 N ASP A 219 31.544 −24.905 −35.744 1.00 39.43 N

ATOM 374 CA ASP A 219 30.172 −25.150 −35.240 1.00 39.58 C

ATOM 375 C ASP A 219 30.130 −25.782 −33.822 1.00 39.54 C

ATOM 376 O ASP A 219 29.601 −26.890 −33.654 1.00 39.76 O

ATOM 377 CB ASP A 219 29.320 −25.911 −36.276 1.00 39.91 C

ATOM 378 CG ASP A 219 27.824 −25.942 −35.911 1.00 40.10 C

ATOM 379 OD1 ASP A 219 27.267 −24.883 −35.554 1.00 40.00 O

ATOM 380 OD2 ASP A 219 27.179 −26.974 −36.223 1.00 40.36 O

ATOM 381 HN ASP A 219 31.771 −23.951 −36.008 1.00 0.00 H

ATOM 382 HA ASP A 219 29.712 −24.167 −35.141 1.00 39.83 H

ATOM 383 HB1 ASP A 219 29.432 −25.424 −37.244 1.00 39.83 H

ATOM 384 HB2 ASP A 219 29.684 −26.936 −36.340 1.00 39.83 H

ATOM 385 N PRO A 220 30.851 −25.203 −32.834 1.00 27.00 N

ATOM 386 CA PRO A 220 31.111 −25.870 −31.567 1.00 26.94 C

ATOM 387 C PRO A 220 29.889 −25.889 −30.639 1.00 26.98 C

ATOM 388 O PRO A 220 29.296 −24.848 −30.334 1.00 26.93 O

ATOM 389 CB PRO A 220 32.292 −25.131 −30.941 1.00 26.63 C

ATOM 390 CG PRO A 220 32.151 −23.713 −31.482 1.00 26.51 C

ATOM 391 CD PRO A 220 31.562 −23.927 −32.873 1.00 26.74 C

ATOM 392 HA PRO A 220 31.411 −26.898 −31.769 1.00 26.82 H

ATOM 393 HB1 PRO A 220 32.226 −25.141 −29.853 1.00 26.82 H

ATOM 394 HB2 PRO A 220 33.238 −25.574 −31.252 1.00 26.82 H

ATOM 395 HG1 PRO A 220 31.479 −23.123 −30.860 1.00 26.82 H

ATOM 396 HG2 PRO A 220 33.121 −23.219 −31.540 1.00 26.82 H

ATOM 397 HD1 PRO A 220 30.872 −23.119 −33.117 1.00 26.82 H

ATOM 398 HD2 PRO A 220 32.358 −23.963 −33.617 1.00 26.82 H

ATOM 399 N LEU A 221 29.713 −27.034 −29.980 1.00 27.07 N

ATOM 400 CA LEU A 221 28.813 −27.256 −28.848 1.00 27.10 C

ATOM 401 C LEU A 221 29.625 −27.688 −27.623 1.00 26.91 C

ATOM 402 O LEU A 221 30.477 −28.568 −27.730 1.00 26.93 O

ATOM 403 CB LEU A 221 27.745 −28.286 −29.271 1.00 27.48 C

ATOM 404 CG LEU A 221 26.740 −28.757 −28.192 1.00 27.62 C

ATOM 405 CD1 LEU A 221 25.454 −29.220 −28.875 1.00 28.03 C

ATOM 406 CD2 LEU A 221 27.221 −29.953 −27.367 1.00 27.55 C

ATOM 407 HN LEU A 221 30.322 −27.806 −30.238 1.00 0.00 H

ATOM 408 HA LEU A 221 28.313 −26.316 −28.616 1.00 27.34 H

ATOM 409 HB1 LEU A 221 27.168 −27.843 −30.083 1.00 27.34 H

ATOM 410 HB2 LEU A 221 28.270 −29.169 −29.636 1.00 27.34 H

ATOM 411 HG LEU A 221 26.514 −27.926 −27.524 1.00 27.34 H

ATOM 412 1HD1 LEU A 221 24.741 −29.553 −28.121 1.00 27.34 H

ATOM 413 2HD1 LEU A 221 25.678 −30.044 −29.552 1.00 27.34 H

ATOM 414 3HD1 LEU A 221 25.024 −28.393 −29.440 1.00 27.34 H

ATOM 415 1HD2 LEU A 221 26.459 −30.220 −26.634 1.00 27.34 H
ATOM 416 2HD2 LEU A 221 28.145 −29.691 −26.851 1.00 27.34 H
ATOM 417 3HD2 LEU A 221 27.402 −30.801 −28.027 1.00 27.34 H
ATOM 418 N LEU A 222 29.206 −27.209 −26.453 1.00 26.73 N
ATOM 419 CA LEU A 222 29.513 −27.755 −25.132 1.00 26.61 C
ATOM 420 C LEU A 222 28.234 −27.672 −24.287 1.00 26.66 C
ATOM 421 O LEU A 222 27.495 −26.694 −24.405 1.00 26.58 O
ATOM 422 CB LEU A 222 30.689 −26.983 −24.495 1.00 26.27 C
ATOM 423 CG LEU A 222 31.011 −27.381 −23.037 1.00 26.07 C
ATOM 424 CD1 LEU A 222 31.580 −28.798 −22.937 1.00 26.22 C
ATOM 425 CD2 LEU A 222 32.040 −26.429 −22.434 1.00 25.72 C
ATOM 426 HN LEU A 222 28.448 −26.543 −26.460 1.00 0.00 H
ATOM 427 HA LEU A 222 29.796 −28.802 −25.241 1.00 26.36 H
ATOM 428 HB1 LEU A 222 31.578 −27.161 −25.099 1.00 26.36 H
ATOM 429 HB2 LEU A 222 30.445 −25.921 −24.511 1.00 26.36 H
ATOM 430 HG LEU A 222 30.095 −27.328 −22.448 1.00 26.36 H
ATOM 431 1HD1 LEU A 222 31.791 −29.033 −21.894 1.00 26.36 H
ATOM 432 2HD1 LEU A 222 32.501 −28.862 −23.516 1.00 26.36 H
ATOM 433 3HD1 LEU A 222 30.854 −29.510 −23.330 1.00 26.36 H
ATOM 434 1HD2 LEU A 222 32.253 −26.726 −21.407 1.00 26.36 H
ATOM 435 2HD2 LEU A 222 31.645 −25.413 −22.443 1.00 26.36 H
ATOM 436 3HD2 LEU A 222 32.958 −26.467 −23.020 1.00 26.36 H
ATOM 437 N ALA A 223 28.009 −28.657 −23.426 1.00 26.80 N
ATOM 438 CA ALA A 223 26.996 −28.628 −22.378 1.00 26.85 C
ATOM 439 C ALA A 223 27.507 −29.389 −21.151 1.00 26.69 C
ATOM 440 O ALA A 223 28.128 −30.440 −21.321 1.00 26.66 O
ATOM 441 CB ALA A 223 25.713 −29.267 −22.918 1.00 27.24 C

ATOM 442 HN ALA A 223 28.619 −29.466 −23.443 1.00 0.00 H

ATOM 443 HA ALA A 223 26.794 −27.593 −22.101 1.00 26.85 H

ATOM 444 HB1 ALA A 223 24.946 −29.252 −22.144 1.00 26.85 H

ATOM 445 HB2 ALA A 223 25.915 −30.298 −23.209 1.00 26.85 H

ATOM 446 HB3 ALA A 223 25.364 −28.706 −23.785 1.00 26.85 H

ATOM 447 N VAL A 224 27.206 −28.915 −19.942 1.00 26.59 N

ATOM 448 CA VAL A 224 27.513 −29.596 −18.675 1.00 26.40 C

ATOM 449 C VAL A 224 26.244 −29.750 −17.836 1.00 26.52 C

ATOM 450 O VAL A 224 25.599 −28.771 −17.465 1.00 26.41 O

ATOM 451 CB VAL A 224 28.634 −28.874 −17.896 1.00 26.00 C

ATOM 452 CG1 VAL A 224 28.977 −29.597 −16.585 1.00 25.79 C

ATOM 453 CG2 VAL A 224 29.927 −28.768 −18.719 1.00 25.90 C

ATOM 454 HN VAL A 224 26.836 −27.965 −19.892 1.00 0.00 H

ATOM 455 HA VAL A 224 27.872 −30.596 −18.917 1.00 26.23 H

ATOM 456 HB VAL A 224 28.294 −27.867 −17.656 1.00 26.23 H

ATOM 457 1HG1 VAL A 224 29.770 −29.056 −16.068 1.00 26.23 H

ATOM 458 2HG1 VAL A 224 29.313 −30.610 −16.805 1.00 26.23 H

ATOM 459 3HG1 VAL A 224 28.092 −29.638 −15.950 1.00 26.23 H

ATOM 460 1HG2 VAL A 224 30.689 −28.254 −18.134 1.00 26.23 H

ATOM 461 2HG2 VAL A 224 29.732 −28.207 −19.633 1.00 26.23 H

ATOM 462 3HG2 VAL A 224 30.279 −29.768 −18.974 1.00 26.23 H

ATOM 463 N ASP A 225 25.979 −30.975 −17.386 1.00 26.75 N

ATOM 464 CA ASP A 225 24.855 −31.337 −16.523 1.00 26.90 C

ATOM 465 C ASP A 225 25.327 −32.278 −15.413 1.00 26.77 C

ATOM 466 O ASP A 225 25.739 −33.407 −15.671 1.00 26.89 O

ATOM 467 CB ASP A 225 23.759 −32.039 −17.332 1.00 27.35 C

ATOM 468 CG ASP A 225 22.716 −31.106 −17.950 1.00 27.50 C

ATOM 469 OD1 ASP A 225 21.921 −30.638 −17.098 1.00 27.50 O
ATOM 470 OD2 ASP A 225 22.293 −31.477 −19.071 1.00 27.63 O
ATOM 471 HN ASP A 225 26.607 −31.722 −17.667 1.00 0.00 H
ATOM 472 HA ASP A 225 24.444 −30.433 −16.074 1.00 27.16 H
ATOM 473 HB1 ASP A 225 24.237 −32.594 −18.139 1.00 27.16 H
ATOM 474 HB2 ASP A 225 23.242 −32.733 −16.670 1.00 27.16 H
ATOM 475 N ASN A 226 25.123 −31.875 −14.157 1.00 28.10 N
ATOM 476 CA ASN A 226 25.235 −32.733 −12.966 1.00 28.01 C
ATOM 477 C ASN A 226 26.608 −33.416 −12.719 1.00 27.81 C
ATOM 478 O ASN A 226 26.746 −34.096 −11.708 1.00 27.68 O
ATOM 479 CB ASN A 226 24.060 −33.741 −12.963 1.00 28.41 C
ATOM 480 CG ASN A 226 22.694 −33.090 −13.103 1.00 28.65 C
ATOM 481 OD1 ASN A 226 22.000 −33.202 −14.105 1.00 29.05 O
ATOM 482 ND2 ASN A 226 22.285 −32.320 −12.120 1.00 28.43 N
ATOM 483 HN ASN A 226 24.757 −30.943 −14.033 1.00 0.00 H
ATOM 484 1HD2 ASN A 226 22.831 −32.247 −11.281 1.00 0.00 H
ATOM 485 2HD2 ASN A 226 21.389 −31.884 −12.224 1.00 0.00 H
ATOM 486 HA ASN A 226 25.069 −32.079 −12.110 1.00 28.27 H
ATOM 487 HB1 ASN A 226 24.198 −34.432 −13.794 1.00 28.27 H
ATOM 488 HB2 ASN A 226 24.083 −34.293 −12.023 1.00 28.27 H
ATOM 489 N GLY A 227 27.624 −33.165 −13.555 1.00 34.27 N
ATOM 490 CA GLY A 227 28.937 −33.834 −13.541 1.00 34.17 C
ATOM 491 C GLY A 227 29.290 −34.541 −14.861 1.00 34.37 C
ATOM 492 O GLY A 227 30.462 −34.809 −15.122 1.00 34.28 O
ATOM 493 HN GLY A 227 27.401 −32.629 −14.381 1.00 0.00 H
ATOM 494 HA1 GLY A 227 29.702 −33.084 −13.338 1.00 34.27 H
ATOM 495 HA2 GLY A 227 28.935 −34.577 −12.744 1.00 34.27 H

```
ATOM    496  N   PHE A 228      28.309 −34.709 −15.750  1.00 26.62           N
ATOM    497  CA  PHE A 228      28.478 −35.165 −17.130  1.00 26.86           C
ATOM    498  C   PHE A 228      28.590 −33.987 −18.105  1.00 26.81           C
ATOM    499  O   PHE A 228      28.134 −32.881 −17.805  1.00 26.69           O
ATOM    500  CB  PHE A 228      27.300 −36.078 −17.500  1.00 27.32           C
ATOM    501  CG  PHE A 228      27.084 −37.198 −16.501  1.00 27.40           C
ATOM    502  CD1 PHE A 228      28.071 −38.187 −16.349  1.00 27.62           C
ATOM    503  CD2 PHE A 228      25.950 −37.210 −15.669  1.00 27.27           C
ATOM    504  CE1 PHE A 228      27.928 −39.189 −15.375  1.00 27.72           C
ATOM    505  CE2 PHE A 228      25.804 −38.215 −14.695  1.00 27.36           C
ATOM    506  CZ  PHE A 228      26.791 −39.207 −14.550  1.00 27.58           C
ATOM    507  HN  PHE A 228      27.377 −34.401 −15.500  1.00  0.00           H
ATOM    508  HA  PHE A 228      29.397 −35.749 −17.189  1.00 27.20           H
ATOM    509  HB1 PHE A 228      26.394 −35.474 −17.547  1.00 27.20           H
ATOM    510  HB2 PHE A 228      27.495 −36.518 −18.478  1.00 27.20           H
ATOM    511  HD1 PHE A 228      28.952 −38.176 −16.990  1.00 27.20           H
ATOM    512  HD2 PHE A 228      25.185 −36.441 −15.779  1.00 27.20           H
ATOM    513  HE1 PHE A 228      28.698 −39.952 −15.259  1.00 27.20           H
ATOM    514  HE2 PHE A 228      24.924 −38.225 −14.051  1.00 27.20           H
ATOM    515  HZ  PHE A 228      26.674 −39.988 −13.799  1.00 27.20           H
ATOM    516  N   PHE A 229      29.056 −34.250 −19.327  1.00 26.92           N
ATOM    517  CA  PHE A 229      29.075 −33.267 −20.414  1.00 26.87           C
ATOM    518  C   PHE A 229      28.679 −33.860 −21.772  1.00 27.24           C
ATOM    519  O   PHE A 229      28.847 −35.059 −22.012  1.00 27.48           O
ATOM    520  CB  PHE A 229      30.447 −32.569 −20.477  1.00 26.52           C
ATOM    521  CG  PHE A 229      31.565 −33.382 −21.106  1.00 26.56           C
ATOM    522  CD1 PHE A 229      31.845 −33.249 −22.480  1.00 26.79           C
```

ATOM	523	CD2	PHE	A	229	32.323	−34.274	−20.326	1.00	26.39	C
ATOM	524	CE1	PHE	A	229	32.858	−34.021	−23.074	1.00	26.87	C
ATOM	525	CE2	PHE	A	229	33.350	−35.030	−20.916	1.00	26.46	C
ATOM	526	CZ	PHE	A	229	33.609	−34.914	−22.291	1.00	26.71	C
ATOM	527	HN	PHE	A	229	29.396	−35.186	−19.525	1.00	0.00	H
ATOM	528	HA	PHE	A	229	28.337	−32.503	−20.169	1.00	26.80	H
ATOM	529	HB1	PHE	A	229	30.331	−31.653	−21.056	1.00	26.80	H
ATOM	530	HB2	PHE	A	229	30.745	−32.321	−19.458	1.00	26.80	H
ATOM	531	HD1	PHE	A	229	31.274	−32.545	−23.085	1.00	26.80	H
ATOM	532	HD2	PHE	A	229	32.113	−34.379	−19.262	1.00	26.80	H
ATOM	533	HE1	PHE	A	229	33.061	−33.927	−24.141	1.00	26.80	H
ATOM	534	HE2	PHE	A	229	33.947	−35.708	−20.305	1.00	26.80	H
ATOM	535	HZ	PHE	A	229	34.392	−35.516	−22.751	1.00	26.80	H
ATOM	536	N	ALA	A	230	28.321	−32.971	−22.701	1.00	27.28	N
ATOM	537	CA	ALA	A	230	28.228	−33.226	−24.136	1.00	27.59	C
ATOM	538	C	ALA	A	230	29.031	−32.200	−24.946	1.00	27.40	C
ATOM	539	O	ALA	A	230	29.260	−31.073	−24.510	1.00	27.06	O
ATOM	540	CB	ALA	A	230	26.762	−33.244	−24.573	1.00	27.81	C
ATOM	541	HN	ALA	A	230	28.244	−32.003	−22.401	1.00	0.00	H
ATOM	542	HA	ALA	A	230	28.649	−34.212	−24.330	1.00	27.43	H
ATOM	543	HB1	ALA	A	230	26.704	−33.435	−25.645	1.00	27.43	H
ATOM	544	HB2	ALA	A	230	26.304	−32.280	−24.351	1.00	27.43	H
ATOM	545	HB3	ALA	A	230	26.232	−34.030	−24.035	1.00	27.43	H
ATOM	546	N	TYR	A	231	29.423	−32.589	−26.157	1.00	27.63	N
ATOM	547	CA	TYR	A	231	30.275	−31.818	−27.056	1.00	27.48	C
ATOM	548	C	TYR	A	231	29.952	−32.123	−28.528	1.00	27.78	C
ATOM	549	O	TYR	A	231	29.590	−33.249	−28.874	1.00	28.10	O

ATOM 550 CB TYR A 231 31.739 −32.123 −26.711 1.00 27.22 C

ATOM 551 CG TYR A 231 32.755 −31.481 −27.634 1.00 27.17 C

ATOM 552 CD1 TYR A 231 33.206 −30.171 −27.383 1.00 26.87 C

ATOM 553 CD2 TYR A 231 33.223 −32.183 −28.762 1.00 27.44 C

ATOM 554 CE1 TYR A 231 34.138 −29.566 −28.249 1.00 26.83 C

ATOM 555 CE2 TYR A 231 34.147 −31.578 −29.632 1.00 27.40 C

ATOM 556 CZ TYR A 231 34.613 −30.270 −29.375 1.00 27.10 C

ATOM 557 OH TYR A 231 35.520 −29.692 −30.205 1.00 27.08 O

ATOM 558 HN TYR A 231 29.238 −33.553 −26.419 1.00 0.00 H

ATOM 559 HH TYR A 231 35.609 −30.200 −31.028 1.00 0.00 H

ATOM 560 HA TYR A 231 30.093 −30.758 −26.877 1.00 27.34 H

ATOM 561 HB1 TYR A 231 31.930 −31.769 −25.698 1.00 27.34 H

ATOM 562 HB2 TYR A 231 31.879 −33.203 −26.751 1.00 27.34 H

ATOM 563 HD1 TYR A 231 32.833 −29.624 −26.517 1.00 27.34 H

ATOM 564 HD2 TYR A 231 32.869 −33.195 −28.960 1.00 27.34 H

ATOM 565 HE1 TYR A 231 34.492 −28.555 −28.049 1.00 27.34 H

ATOM 566 HE2 TYR A 231 34.505 −32.120 −30.507 1.00 27.34 H

ATOM 567 N SER A 232 30.176 −31.160 −29.423 1.00 27.69 N

ATOM 568 CA SER A 232 30.195 −31.384 −30.878 1.00 27.91 C

ATOM 569 C SER A 232 31.152 −30.420 −31.570 1.00 27.68 C

ATOM 570 O SER A 232 31.192 −29.246 −31.205 1.00 27.38 O

ATOM 571 CB SER A 232 28.771 −31.323 −31.478 1.00 28.17 C

ATOM 572 OG SER A 232 28.649 −30.496 −32.635 1.00 28.36 O

ATOM 573 HN SER A 232 30.533 −30.272 −29.079 1.00 0.00 H

ATOM 574 HG SER A 232 28.840 −29.585 −32.366 1.00 0.00 H

ATOM 575 HA SER A 232 30.574 −32.392 −31.045 1.00 27.86 H

ATOM 576 HB1 SER A 232 28.473 −32.335 −31.751 1.00 27.87 H

ATOM	577	HB2	SER	A	232	28.096	−30.939	−30.713	1.00	27.87	H
ATOM	578	N	HIS	A	233	31.698	−30.844	−32.713	1.00	27.87	N
ATOM	579	CA	HIS	A	233	32.311	−29.949	−33.700	1.00	27.74	C
ATOM	580	C	HIS	A	233	32.132	−30.462	−35.129	1.00	28.06	C
ATOM	581	O	HIS	A	233	31.985	−31.663	−35.358	1.00	28.37	O
ATOM	582	CB	HIS	A	233	33.795	−29.706	−33.367	1.00	27.50	C
ATOM	583	CG	HIS	A	233	34.754	−30.874	−33.522	1.00	27.68	C
ATOM	584	ND1	HIS	A	233	35.715	−31.238	−32.573	1.00	27.55	N
ATOM	585	CD2	HIS	A	233	35.013	−31.575	−34.669	1.00	27.99	C
ATOM	586	CE1	HIS	A	233	36.486	−32.172	−33.149	1.00	27.78	C
ATOM	587	NE2	HIS	A	233	36.082	−32.401	−34.407	1.00	28.06	N
ATOM	588	HN	HIS	A	233	31.507	−31.798	−33.003	1.00	0.00	H
ATOM	589	HE2	HIS	A	233	36.518	−33.043	−35.056	1.00	0.00	H
ATOM	590	HA	HIS	A	233	31.800	−28.989	−33.632	1.00	27.86	H
ATOM	591	HB1	HIS	A	233	34.148	−28.906	−34.018	1.00	27.86	H
ATOM	592	HB2	HIS	A	233	33.846	−29.379	−32.329	1.00	27.86	H
ATOM	593	HD2	HIS	A	233	34.474	−31.494	−35.613	1.00	27.86	H
ATOM	594	HE1	HIS	A	233	37.323	−32.674	−32.663	1.00	27.86	H
ATOM	595	N	LEU	A	234	32.341	−29.570	−36.092	1.00	27.98	N
ATOM	596	CA	LEU	A	234	32.568	−29.847	−37.505	1.00	28.25	C
ATOM	597	C	LEU	A	234	34.018	−29.492	−37.875	1.00	28.15	C
ATOM	598	O	LEU	A	234	34.403	−28.324	−37.859	1.00	27.88	O
ATOM	599	CB	LEU	A	234	31.531	−29.038	−38.306	1.00	28.33	C
ATOM	600	CG	LEU	A	234	31.610	−29.229	−39.829	1.00	28.61	C
ATOM	601	CD1	LEU	A	234	31.315	−30.675	−40.227	1.00	28.96	C
ATOM	602	CD2	LEU	A	234	30.582	−28.326	−40.512	1.00	28.65	C
ATOM	603	HN	LEU	A	234	32.463	−28.604	−35.801	1.00	0.00	H

ATOM 604 HA LEU A 234 32.406 −30.909 −37.688 1.00 28.35 H

ATOM 605 HB1 LEU A 234 30.537 −29.339 −37.977 1.00 28.35 H

ATOM 606 HB2 LEU A 234 31.682 −27.981 −38.088 1.00 28.35 H

ATOM 607 HG LEU A 234 32.609 −28.959 −40.173 1.00 28.35 H

ATOM 608 1HD1 LEU A 234 31.379 −30.775 −41.310 1.00 28.35 H

ATOM 609 2HD1 LEU A 234 30.312 −30.946 −39.897 1.00 28.35 H

ATOM 610 3HD1 LEU A 234 32.043 −31.337 −39.758 1.00 28.35 H

ATOM 611 1HD2 LEU A 234 30.639 −28.462 −41.592 1.00 28.35 H

ATOM 612 2HD2 LEU A 234 30.792 −27.285 −40.266 1.00 28.35 H

ATOM 613 3HD2 LEU A 234 29.582 −28.586 −40.166 1.00 28.35 H

ATOM 614 N GLU A 235 34.829 −30.497 −38.200 1.00 28.42 N

ATOM 615 CA GLU A 235 36.171 −30.314 −38.760 1.00 28.40 C

ATOM 616 C GLU A 235 36.102 −29.971 −40.253 1.00 28.55 C

ATOM 617 O GLU A 235 35.412 −30.653 −41.018 1.00 28.84 O

ATOM 618 CB GLU A 235 37.008 −31.586 −38.536 1.00 28.63 C

ATOM 619 CG GLU A 235 38.425 −31.464 −39.122 1.00 28.53 C

ATOM 620 CD GLU A 235 39.310 −32.675 −38.823 1.00 28.76 C

ATOM 621 OE1 GLU A 235 38.842 −33.781 −39.174 1.00 28.81 O

ATOM 622 OE2 GLU A 235 40.538 −32.459 −38.883 1.00 28.89 O

ATOM 623 HN GLU A 235 34.438 −31.433 −38.230 1.00 0.00 H

ATOM 624 HA GLU A 235 36.653 −29.487 −38.238 1.00 28.65 H

ATOM 625 HB1 GLU A 235 37.087 −31.769 −37.464 1.00 28.65 H

ATOM 626 HB2 GLU A 235 36.503 −32.426 −39.013 1.00 28.65 H

ATOM 627 HG1 GLU A 235 38.344 −31.354 −40.203 1.00 28.65 H

ATOM 628 HG2 GLU A 235 38.898 −30.577 −38.700 1.00 28.65 H

ATOM 629 N LYS A 236 36.969 −29.057 −40.696 1.00 28.34 N

ATOM 630 CA LYS A 236 37.114 −28.605 −42.083 1.00 28.45 C

ATOM	631	C	LYS	A	236	38.570	−28.599	−42.540	1.00	28.50	C
ATOM	632	O	LYS	A	236	39.458	−28.232	−41.778	1.00	28.31	O
ATOM	633	CB	LYS	A	236	36.531	−27.192	−42.214	1.00	28.19	C
ATOM	634	CG	LYS	A	236	35.000	−27.188	−42.131	1.00	28.17	C
ATOM	635	CD	LYS	A	236	34.505	−25.755	−41.941	1.00	27.94	C
ATOM	636	CE	LYS	A	236	32.984	−25.682	−42.086	1.00	28.02	C
ATOM	637	NZ	LYS	A	236	32.604	−25.277	−43.459	1.00	28.25	N1+
ATOM	638	HN	LYS	A	236	37.493	−28.535	−39.996	1.00	0.00	H
ATOM	639	HZ1	LYS	A	236	31.619	−25.409	−43.623	1.00	0.00	H
ATOM	640	HZ2	LYS	A	236	32.785	−24.283	−43.583	1.00	0.00	H
ATOM	641	HZ3	LYS	A	236	33.148	−25.784	−44.156	1.00	0.00	H
ATOM	642	HA	LYS	A	236	36.549	−29.276	−42.729	1.00	28.24	H
ATOM	643	HB1	LYS	A	236	36.928	−26.573	−41.410	1.00	28.24	H
ATOM	644	HB2	LYS	A	236	36.832	−26.776	−43.176	1.00	28.24	H
ATOM	645	HG1	LYS	A	236	34.585	−27.597	−43.052	1.00	28.24	H
ATOM	646	HG2	LYS	A	236	34.680	−27.798	−41.286	1.00	28.24	H
ATOM	647	HD1	LYS	A	236	34.786	−25.409	−40.946	1.00	28.24	H
ATOM	648	HD2	LYS	A	236	34.967	−25.115	−42.693	1.00	28.24	H
ATOM	649	HE1	LYS	A	236	32.558	−26.662	−41.872	1.00	28.24	H
ATOM	650	HE2	LYS	A	236	32.592	−24.952	−41.377	1.00	28.24	H
ATOM	651	N	ILE	A	237	38.781	−28.846	−43.831	1.00	35.45	N
ATOM	652	CA	ILE	A	237	39.982	−28.435	−44.571	1.00	35.50	C
ATOM	653	C	ILE	A	237	39.874	−26.930	−44.848	1.00	35.28	C
ATOM	654	O	ILE	A	237	38.910	−26.489	−45.482	1.00	35.36	O
ATOM	655	CB	ILE	A	237	40.128	−29.225	−45.895	1.00	35.90	C
ATOM	656	CG1	ILE	A	237	40.050	−30.751	−45.663	1.00	36.20	C
ATOM	657	CG2	ILE	A	237	41.446	−28.838	−46.593	1.00	35.95	C

ATOM 658 CD1 ILE A 237 40.091 −31.598 −46.941 1.00 36.62 C

ATOM 659 HN ILE A 237 37.971 −29.107 −44.380 1.00 0.00 H

ATOM 660 1HD1 ILE A 237 40.031 −32.654 −46.679 1.00 0.00 H

ATOM 661 2HD1 ILE A 237 41.023 −31.408 −47.473 1.00 0.00 H

ATOM 662 HA ILE A 237 40.860 −28.618 −43.951 1.00 35.78 H

ATOM 663 HB ILE A 237 39.304 −28.941 −46.550 1.00 35.78 H

ATOM 664 1HG1 ILE A 237 40.893 −31.042 −45.036 1.00 35.78 H

ATOM 665 2HG1 ILE A 237 39.117 −30.968 −45.142 1.00 35.78 H

ATOM 666 1HG2 ILE A 237 41.543 −29.397 −47.524 1.00 35.78 H

ATOM 667 2HG2 ILE A 237 42.286 −29.073 −45.940 1.00 35.78 H

ATOM 668 3HG2 ILE A 237 41.443 −27.770 −46.810 1.00 35.78 H

ATOM 669 3HD1 ILE A 237 39.248 −31.334 −47.580 1.00 35.78 H

ATOM 670 N GLY A 238 40.853 −26.149 −44.399 1.00 28.33 N

ATOM 671 CA GLY A 238 40.871 −24.693 −44.529 1.00 28.13 C

ATOM 672 C GLY A 238 39.777 −23.973 −43.725 1.00 27.87 C

ATOM 673 O GLY A 238 39.363 −24.418 −42.657 1.00 27.74 O

ATOM 674 HN GLY A 238 41.642 −26.589 −43.931 1.00 0.00 H

ATOM 675 HA1 GLY A 238 41.840 −24.332 −44.186 1.00 28.02 H

ATOM 676 HA2 GLY A 238 40.741 −24.443 −45.582 1.00 28.02 H

ATOM 677 N SER A 239 39.376 −22.792 −44.213 1.00 28.81 N

ATOM 678 CA SER A 239 38.617 −21.785 −43.447 1.00 28.58 C

ATOM 679 C SER A 239 37.361 −22.322 −42.739 1.00 28.53 C

ATOM 680 O SER A 239 36.545 −23.020 −43.337 1.00 28.72 O

ATOM 681 CB SER A 239 38.240 −20.607 −44.360 1.00 28.62 C

ATOM 682 OG SER A 239 37.289 −20.990 −45.341 1.00 28.85 O

ATOM 683 HN SER A 239 39.825 −22.488 −45.061 1.00 0.00 H

ATOM 684 HG SER A 239 37.612 −21.795 −45.762 1.00 0.00 H

ATOM 685 HA SER A 239 39.282 −21.397 −42.676 1.00 28.68 H

ATOM 686 HB1 SER A 239 37.817 −19.808 −43.750 1.00 28.68 H

ATOM 687 HB2 SER A 239 39.138 −20.246 −44.860 1.00 28.68 H

ATOM 688 N CYS A 240 37.074 −21.793 −41.548 1.00 27.28 N

ATOM 689 CA CYS A 240 35.934 −22.219 −40.728 1.00 27.23 C

ATOM 690 C CYS A 240 34.570 −22.018 −41.414 1.00 27.33 C

ATOM 691 O CYS A 240 33.711 −22.895 −41.368 1.00 27.37 O

ATOM 692 CB CYS A 240 35.988 −21.445 −39.407 1.00 26.95 C

ATOM 693 SG CYS A 240 37.607 −21.463 −38.601 1.00 26.84 S

ATOM 694 HN CYS A 240 37.786 −21.248 −41.081 1.00 0.00 H

ATOM 695 HA CYS A 240 36.051 −23.280 −40.506 1.00 27.17 H

ATOM 696 HB1 CYS A 240 35.718 −20.408 −39.607 1.00 27.17 H

ATOM 697 HB2 CYS A 240 35.261 −21.885 −38.724 1.00 27.17 H

ATOM 698 N THR A 241 34.412 −20.958 −42.211 1.00 27.37 N

ATOM 699 CA THR A 241 33.191 −20.738 −43.000 1.00 27.45 C

ATOM 700 C THR A 241 33.122 −21.675 −44.213 1.00 27.73 C

ATOM 701 O THR A 241 32.301 −22.593 −44.244 1.00 27.88 O

ATOM 702 CB THR A 241 33.042 −19.262 −43.406 1.00 27.30 C

ATOM 703 OG1 THR A 241 34.166 −18.816 −44.132 1.00 27.40 O

ATOM 704 CG2 THR A 241 32.906 −18.338 −42.198 1.00 27.06 C

ATOM 705 HN THR A 241 35.167 −20.294 −42.290 1.00 0.00 H

ATOM 706 HG1 THR A 241 34.385 −17.941 −43.791 1.00 0.00 H

ATOM 707 HA THR A 241 32.345 −20.981 −42.357 1.00 27.46 H

ATOM 708 HB THR A 241 32.155 −19.157 −44.031 1.00 27.46 H

ATOM 709 1HG2 THR A 241 32.804 −17.307 −42.538 1.00 27.46 H

ATOM 710 2HG2 THR A 241 33.793 −18.427 −41.570 1.00 27.46 H

ATOM 711 3HG2 THR A 241 32.024 −18.619 −41.622 1.00 27.46 H

ATOM 712 N ARG A 242 34.016 −21.517 −45.197 1.00 27.82 N

ATOM 713 CA ARG A 242 33.886 −22.128 −46.539 1.00 28.08 C

ATOM 714 C ARG A 242 34.731 −23.388 −46.767 1.00 28.31 C

ATOM 715 O ARG A 242 34.648 −23.979 −47.836 1.00 28.57 O

ATOM 716 CB ARG A 242 34.132 −21.052 −47.613 1.00 28.06 C

ATOM 717 CG ARG A 242 33.023 −19.987 −47.590 1.00 28.01 C

ATOM 718 CD ARG A 242 33.265 −18.883 −48.624 1.00 27.82 C

ATOM 719 NE ARG A 242 32.190 −17.870 −48.564 1.00 27.72 N

ATOM 720 CZ ARG A 242 32.310 −16.559 −48.696 1.00 27.59 C

ATOM 721 NH1 ARG A 242 33.448 −15.983 −48.964 1.00 27.55 N

ATOM 722 NH2 ARG A 242 31.267 −15.790 −48.556 1.00 27.52 N

ATOM 723 HN ARG A 242 34.702 −20.778 −45.070 1.00 0.00 H

ATOM 724 HE ARG A 242 31.265 −18.219 −48.376 1.00 0.00 H

ATOM 725 1HH1 ARG A 242 34.266 −16.554 −49.080 1.00 0.00 H

ATOM 726 2HH1 ARG A 242 33.516 −14.985 −49.045 1.00 0.00 H

ATOM 727 1HH2 ARG A 242 30.372 −16.184 −48.326 1.00 0.00 H

ATOM 728 2HH2 ARG A 242 31.361 −14.793 −48.632 1.00 0.00 H

ATOM 729 HA ARG A 242 32.844 −22.432 −46.640 1.00 27.91 H

ATOM 730 HB1 ARG A 242 35.092 −20.571 −47.423 1.00 27.91 H

ATOM 731 HB2 ARG A 242 34.151 −21.526 −48.594 1.00 27.91 H

ATOM 732 HG1 ARG A 242 32.069 −20.468 −47.806 1.00 27.91 H

ATOM 733 HG2 ARG A 242 32.988 −19.538 −46.598 1.00 27.91 H

ATOM 734 HD1 ARG A 242 34.222 −18.403 −48.418 1.00 27.91 H

ATOM 735 HD2 ARG A 242 33.286 −19.324 −49.621 1.00 27.91 H

ATOM 736 N GLY A 243 35.482 −23.836 −45.764 1.00 28.24 N

ATOM 737 CA GLY A 243 36.289 −25.056 −45.808 1.00 28.47 C

ATOM 738 C GLY A 243 35.466 −26.342 −45.929 1.00 28.74 C

ATOM 739 O GLY A 243 34.333 −26.424 −45.435 1.00 28.70 O

ATOM 740 HN GLY A 243 35.528 −23.289 −44.911 1.00 0.00 H

ATOM 741 HA1 GLY A 243 36.957 −24.994 −46.667 1.00 28.54 H

ATOM 742 HA2 GLY A 243 36.878 −25.111 −44.892 1.00 28.54 H

ATOM 743 N ILE A 244 36.051 −27.347 −46.587 1.00 29.04 N

ATOM 744 CA ILE A 244 35.428 −28.644 −46.885 1.00 29.36 C

ATOM 745 C ILE A 244 35.351 −29.481 −45.607 1.00 29.31 C

ATOM 746 O ILE A 244 36.378 −29.747 −44.991 1.00 29.18 O

ATOM 747 CB ILE A 244 36.208 −29.404 −47.989 1.00 29.73 C

ATOM 748 CG1 ILE A 244 36.279 −28.617 −49.320 1.00 29.85 C

ATOM 749 CG2 ILE A 244 35.559 −30.776 −48.262 1.00 30.08 C

ATOM 750 CD1 ILE A 244 37.524 −27.728 −49.446 1.00 29.71 C

ATOM 751 HN ILE A 244 37.018 −27.217 −46.856 1.00 0.00 H

ATOM 752 HA ILE A 244 34.413 −28.465 −47.241 1.00 29.53 H

ATOM 753 HB ILE A 244 37.226 −29.571 −47.636 1.00 29.53 H

ATOM 754 1HG1 ILE A 244 36.282 −29.332 −50.143 1.00 29.53 H

ATOM 755 2HG1 ILE A 244 35.395 −27.983 −49.392 1.00 29.53 H

ATOM 756 1HG2 ILE A 244 36.119 −31.295 −49.039 1.00 29.53 H

ATOM 757 2HG2 ILE A 244 34.530 −30.633 −48.591 1.00 29.53 H

ATOM 758 3HG2 ILE A 244 35.568 −31.371 −47.349 1.00 29.53 H

ATOM 759 1HD1 ILE A 244 37.504 −27.208 −50.404 1.00 29.53 H

ATOM 760 2HD1 ILE A 244 38.420 −28.346 −49.387 1.00 29.53 H

ATOM 761 3HD1 ILE A 244 37.533 −26.998 −48.637 1.00 29.53 H

ATOM 762 N ALA A 245 34.159 −29.948 −45.236 1.00 29.42 N

ATOM 763 CA ALA A 245 33.963 −30.790 −44.056 1.00 29.35 C

ATOM 764 C ALA A 245 34.711 −32.135 −44.170 1.00 29.63 C

ATOM 765 O ALA A 245 34.429 −32.908 −45.084 1.00 30.00 O

ATOM	766	CB	ALA	A	245	32.458	−31.010	−43.870	1.00	29.41	C
ATOM	767	HN	ALA	A	245	33.364	−29.765	−45.824	1.00	0.00	H
ATOM	768	HA	ALA	A	245	34.344	−30.257	−43.185	1.00	29.56	H
ATOM	769	HB1	ALA	A	245	32.287	−31.636	−42.994	1.00	29.56	H
ATOM	770	HB2	ALA	A	245	32.050	−31.502	−44.753	1.00	29.56	H
ATOM	771	HB3	ALA	A	245	31.964	−30.048	−43.730	1.00	29.56	H
ATOM	772	N	LYS	A	246	35.543	−32.472	−43.174	1.00	29.46	N
ATOM	773	CA	LYS	A	246	36.100	−33.827	−42.992	1.00	29.72	C
ATOM	774	C	LYS	A	246	35.198	−34.707	−42.126	1.00	29.91	C
ATOM	775	O	LYS	A	246	34.843	−35.805	−42.534	1.00	30.30	O
ATOM	776	CB	LYS	A	246	37.522	−33.785	−42.403	1.00	29.50	C
ATOM	777	CG	LYS	A	246	38.550	−33.093	−43.319	1.00	29.57	C
ATOM	778	CD	LYS	A	246	39.937	−33.764	−43.287	1.00	29.86	C
ATOM	779	CE	LYS	A	246	40.583	−33.826	−41.896	1.00	29.66	C
ATOM	780	NZ	LYS	A	246	41.146	−32.527	−41.461	1.00	29.22	N1+
ATOM	781	HN	LYS	A	246	35.676	−31.791	−42.432	1.00	0.00	H
ATOM	782	HA	LYS	A	246	36.151	−34.326	−43.962	1.00	0.00	H
ATOM	783	HZ1	LYS	A	246	42.027	−32.307	−41.900	1.00	0.00	H
ATOM	784	HZ2	LYS	A	246	40.487	−31.776	−41.592	1.00	0.00	H
ATOM	785	HZ3	LYS	A	246	41.280	−32.562	−40.442	1.00	0.00	H
ATOM	786	HB1	LYS	A	246	37.487	−33.246	−41.456	1.00	29.69	H
ATOM	787	HB2	LYS	A	246	37.852	−34.809	−42.227	1.00	29.69	H
ATOM	788	HG1	LYS	A	246	38.176	−33.119	−44.342	1.00	29.69	H
ATOM	789	HG2	LYS	A	246	38.659	−32.057	−42.998	1.00	29.69	H
ATOM	790	HD1	LYS	A	246	39.830	−34.783	−43.658	1.00	29.69	H
ATOM	791	HD2	LYS	A	246	40.601	−33.204	−43.946	1.00	29.69	H
ATOM	792	HE1	LYS	A	246	39.826	−34.135	−41.175	1.00	29.69	H

ATOM	793	HE2	LYS	A	246	41.386	−34.562	−41.919	1.00	29.69	H
ATOM	794	N	GLN	A	247	34.765	−34.201	−40.969	1.00	29.65	N
ATOM	795	CA	GLN	A	247	33.913	−34.935	−40.025	1.00	29.82	C
ATOM	796	C	GLN	A	247	33.124	−33.981	−39.117	1.00	29.51	C
ATOM	797	O	GLN	A	247	33.687	−33.059	−38.531	1.00	29.17	O
ATOM	798	CB	GLN	A	247	34.737	−35.945	−39.200	1.00	29.98	C
ATOM	799	CG	GLN	A	247	35.890	−35.326	−38.395	1.00	29.64	C
ATOM	800	CD	GLN	A	247	36.834	−36.375	−37.824	1.00	29.83	C
ATOM	801	OE1	GLN	A	247	36.427	−37.392	−37.286	1.00	29.67	O
ATOM	802	NE2	GLN	A	247	38.125	−36.137	−37.864	1.00	30.19	N
ATOM	803	HN	GLN	A	247	35.093	−33.285	−40.697	1.00	0.00	H
ATOM	804	1HE2	GLN	A	247	38.475	−35.300	−38.342	1.00	0.00	H
ATOM	805	2HE2	GLN	A	247	38.735	−36.844	−37.503	1.00	0.00	H
ATOM	806	HA	GLN	A	247	33.190	−35.503	−40.610	1.00	29.72	H
ATOM	807	HB1	GLN	A	247	34.064	−36.442	−38.502	1.00	29.72	H
ATOM	808	HB2	GLN	A	247	35.158	−36.680	−39.886	1.00	29.72	H
ATOM	809	HG1	GLN	A	247	36.458	−34.665	−39.050	1.00	29.72	H
ATOM	810	HG2	GLN	A	247	35.470	−34.749	−37.571	1.00	29.72	H
ATOM	811	N	ARG	A	248	31.837	−34.276	−38.907	1.00	32.41	N
ATOM	812	CA	ARG	A	248	31.103	−33.890	−37.695	1.00	32.19	C
ATOM	813	C	ARG	A	248	31.421	−34.949	−36.644	1.00	32.21	C
ATOM	814	O	ARG	A	248	31.179	−36.117	−36.925	1.00	32.54	O
ATOM	815	CB	ARG	A	248	29.593	−33.830	−38.011	1.00	32.42	C
ATOM	816	CG	ARG	A	248	28.643	−33.750	−36.793	1.00	32.30	C
ATOM	817	CD	ARG	A	248	28.634	−32.399	−36.070	1.00	31.87	C
ATOM	818	NE	ARG	A	248	28.074	−31.348	−36.936	1.00	31.85	N
ATOM	819	CZ	ARG	A	248	27.935	−30.070	−36.650	1.00	31.55	C

ATOM 820 NH1 ARG A 248 27.457 −29.278 −37.554 1.00 31.55 N

ATOM 821 NH2 ARG A 248 28.225 −29.537 −35.499 1.00 31.24 N

ATOM 822 HN ARG A 248 31.490 −35.099 −39.379 1.00 0.00 H

ATOM 823 HE ARG A 248 27.794 −31.610 −37.867 1.00 0.00 H

ATOM 824 1HH1 ARG A 248 27.018 −29.651 −38.388 1.00 0.00 H

ATOM 825 2HH1 ARG A 248 27.295 −28.313 −37.258 1.00 0.00 H

ATOM 826 1HH2 ARG A 248 28.509 −30.101 −34.711 1.00 0.00 H

ATOM 827 2HH2 ARG A 248 28.038 −28.542 −35.383 1.00 0.00 H

ATOM 828 HA ARG A 248 31.447 −32.914 −37.351 1.00 32.01 H

ATOM 829 HB1 ARG A 248 29.417 −32.949 −38.628 1.00 32.01 H

ATOM 830 HB2 ARG A 248 29.336 −34.726 −38.575 1.00 32.01 H

ATOM 831 HG1 ARG A 248 27.630 −33.955 −37.140 1.00 32.01 H

ATOM 832 HG2 ARG A 248 28.945 −34.515 −36.077 1.00 32.01 H

ATOM 833 HD1 ARG A 248 28.027 −32.480 −35.168 1.00 32.01 H

ATOM 834 HD2 ARG A 248 29.655 −32.132 −35.798 1.00 32.01 H

ATOM 835 N ILE A 249 31.771 −34.553 −35.425 1.00 29.12 N

ATOM 836 CA ILE A 249 31.750 −35.455 −34.263 1.00 29.14 C

ATOM 837 C ILE A 249 30.752 −34.939 −33.231 1.00 29.00 C

ATOM 838 O ILE A 249 30.565 −33.728 −33.089 1.00 28.78 O

ATOM 839 CB ILE A 249 33.154 −35.736 −33.670 1.00 28.90 C

ATOM 840 CG1 ILE A 249 33.655 −34.642 −32.698 1.00 28.47 C

ATOM 841 CG2 ILE A 249 34.173 −36.033 −34.787 1.00 28.89 C

ATOM 842 CD1 ILE A 249 34.960 −35.011 −31.981 1.00 28.27 C

ATOM 843 HN ILE A 249 31.884 −33.559 −35.252 1.00 0.00 H

ATOM 844 HA ILE A 249 31.365 −36.411 −34.618 1.00 28.82 H

ATOM 845 HB ILE A 249 33.064 −36.651 −33.084 1.00 28.82 H

ATOM 846 1HG1 ILE A 249 33.820 −33.726 −33.265 1.00 28.82 H

ATOM 847 2HG1 ILE A 249 32.885 −34.470 −31.946 1.00 28.82 H
ATOM 848 1HG2 ILE A 249 35.151 −36.227 −34.346 1.00 28.82 H
ATOM 849 2HG2 ILE A 249 34.241 −35.175 −35.455 1.00 28.82 H
ATOM 850 3HG2 ILE A 249 33.850 −36.908 −35.351 1.00 28.82 H
ATOM 851 1HD1 ILE A 249 35.252 −34.198 −31.316 1.00 28.82 H
ATOM 852 2HD1 ILE A 249 35.746 −35.177 −32.718 1.00 28.82 H
ATOM 853 3HD1 ILE A 249 34.811 −35.920 −31.399 1.00 28.82 H
ATOM 854 N ILE A 250 30.161 −35.863 −32.480 1.00 34.08 N
ATOM 855 CA ILE A 250 29.270 −35.601 −31.348 1.00 33.97 C
ATOM 856 C ILE A 250 29.651 −36.582 −30.236 1.00 33.98 C
ATOM 857 O ILE A 250 29.774 −37.774 −30.502 1.00 34.32 O
ATOM 858 CB ILE A 250 27.786 −35.752 −31.761 1.00 34.29 C
ATOM 859 CG1 ILE A 250 27.451 −35.004 −33.077 1.00 34.21 C
ATOM 860 CG2 ILE A 250 26.899 −35.273 −30.599 1.00 34.23 C
ATOM 861 CD1 ILE A 250 25.974 −35.031 −33.481 1.00 34.57 C
ATOM 862 HN ILE A 250 30.328 −36.842 −32.707 1.00 0.00 H
ATOM 863 HA ILE A 250 29.437 −34.584 −30.995 1.00 34.21 H
ATOM 864 HB ILE A 250 27.588 −36.812 −31.919 1.00 34.21 H
ATOM 865 1HG1 ILE A 250 27.750 −33.962 −32.959 1.00 34.21 H
ATOM 866 2HG1 ILE A 250 28.029 −35.459 −33.881 1.00 34.21 H
ATOM 867 1HG2 ILE A 250 25.850 −35.374 −30.877 1.00 34.21 H
ATOM 868 2HG2 ILE A 250 27.118 −34.228 −30.381 1.00 34.21 H
ATOM 869 3HG2 ILE A 250 27.100 −35.878 −29.715 1.00 34.21 H
ATOM 870 1HD1 ILE A 250 25.842 −34.482 −34.413 1.00 34.21 H
ATOM 871 2HD1 ILE A 250 25.374 −34.567 −32.698 1.00 34.21 H
ATOM 872 3HD1 ILE A 250 25.654 −36.064 −33.620 1.00 34.21 H
ATOM 873 N GLY A 251 29.941 −36.107 −29.028 1.00 28.69 N

ATOM	874	CA	GLY	A	251	30.489	−36.946	−27.958	1.00	28.65	C
ATOM	875	C	GLY	A	251	30.115	−36.480	−26.557	1.00	28.38	C
ATOM	876	O	GLY	A	251	29.592	−35.380	−26.385	1.00	28.18	O
ATOM	877	HN	GLY	A	251	29.799	−35.119	−28.835	1.00	0.00	H
ATOM	878	HA1	GLY	A	251	30.116	−37.961	−28.093	1.00	28.48	H
ATOM	879	HA2	GLY	A	251	31.576	−36.943	−28.042	1.00	28.48	H
ATOM	880	N	VAL	A	252	30.293	−37.366	−25.580	1.00	28.40	N
ATOM	881	CA	VAL	A	252	29.871	−37.189	−24.182	1.00	28.19	C
ATOM	882	C	VAL	A	252	30.884	−37.810	−23.218	1.00	28.01	C
ATOM	883	O	VAL	A	252	31.718	−38.635	−23.610	1.00	28.16	O
ATOM	884	CB	VAL	A	252	28.455	−37.761	−23.924	1.00	28.54	C
ATOM	885	CG1	VAL	A	252	27.394	−37.154	−24.846	1.00	28.82	C
ATOM	886	CG2	VAL	A	252	28.369	−39.279	−24.088	1.00	28.88	C
ATOM	887	HN	VAL	A	252	30.743	−38.248	−25.812	1.00	0.00	H
ATOM	888	HA	VAL	A	252	29.837	−36.118	−23.980	1.00	28.43	H
ATOM	889	HB	VAL	A	252	28.181	−37.521	−22.897	1.00	28.43	H
ATOM	890	1HG1	VAL	A	252	26.422	−37.592	−24.620	1.00	28.43	H
ATOM	891	2HG1	VAL	A	252	27.653	−37.361	−25.884	1.00	28.43	H
ATOM	892	3HG1	VAL	A	252	27.351	−36.076	−24.691	1.00	28.43	H
ATOM	893	1HG2	VAL	A	252	27.349	−39.609	−23.892	1.00	28.43	H
ATOM	894	2HG2	VAL	A	252	29.047	−39.760	−23.383	1.00	28.43	H
ATOM	895	3HG2	VAL	A	252	28.649	−39.551	−25.105	1.00	28.43	H
ATOM	896	N	GLY	A	253	30.778	−37.454	−21.939	1.00	27.71	N
ATOM	897	CA	GLY	A	253	31.604	−38.038	−20.888	1.00	27.52	C
ATOM	898	C	GLY	A	253	31.428	−37.386	−19.521	1.00	27.15	C
ATOM	899	O	GLY	A	253	30.374	−36.827	−19.219	1.00	27.12	O
ATOM	900	HN	GLY	A	253	30.100	−36.745	−21.678	1.00	0.00	H

ATOM	901	HA1	GLY	A	253	31.348	−39.094	−20.799	1.00	27.38	H
ATOM	902	HA2	GLY	A	253	32.649	−37.941	−21.181	1.00	27.38	H
ATOM	903	N	GLU	A	254	32.484	−37.430	−18.709	1.00	34.09	N
ATOM	904	CA	GLU	A	254	32.478	−37.058	−17.286	1.00	33.75	C
ATOM	905	C	GLU	A	254	33.516	−35.946	−16.996	1.00	33.31	C
ATOM	906	O	GLU	A	254	34.571	−35.894	−17.633	1.00	33.21	O
ATOM	907	CB	GLU	A	254	32.691	−38.323	−16.420	1.00	33.76	C
ATOM	908	CG	GLU	A	254	31.831	−39.526	−16.879	1.00	34.15	C
ATOM	909	CD	GLU	A	254	31.691	−40.657	−15.847	1.00	34.03	C
ATOM	910	OE1	GLU	A	254	32.668	−40.912	−15.109	1.00	33.63	O
ATOM	911	OE2	GLU	A	254	30.652	−41.362	−15.901	1.00	34.34	O
ATOM	912	HN	GLU	A	254	33.330	−37.865	−19.064	1.00	0.00	H
ATOM	913	HA	GLU	A	254	31.491	−36.659	−17.052	1.00	33.81	H
ATOM	914	HB1	GLU	A	254	33.742	−38.607	−16.474	1.00	33.81	H
ATOM	915	HB2	GLU	A	254	32.431	−38.085	−15.389	1.00	33.81	H
ATOM	916	HG1	GLU	A	254	30.832	−39.157	−17.114	1.00	33.81	H
ATOM	917	HG2	GLU	A	254	32.286	−39.943	−17.778	1.00	33.81	H
ATOM	918	N	VAL	A	255	33.189	−34.980	−16.124	1.00	25.87	N
ATOM	919	CA	VAL	A	255	33.978	−33.741	−15.898	1.00	25.47	C
ATOM	920	C	VAL	A	255	34.874	−33.844	−14.647	1.00	25.20	C
ATOM	921	O	VAL	A	255	34.716	−33.103	−13.677	1.00	25.14	O
ATOM	922	CB	VAL	A	255	33.088	−32.473	−15.905	1.00	25.38	C
ATOM	923	CG1	VAL	A	255	33.923	−31.183	−15.995	1.00	24.99	C
ATOM	924	CG2	VAL	A	255	32.144	−32.431	−17.114	1.00	25.63	C
ATOM	925	HN	VAL	A	255	32.287	−35.043	−15.658	1.00	0.00	H
ATOM	926	HA	VAL	A	255	34.651	−33.647	−16.750	1.00	25.38	H
ATOM	927	HB	VAL	A	255	32.495	−32.450	−14.990	1.00	25.38	H

ATOM	928	1HG1	VAL	A	255	33.259	−30.319	−15.997	1.00	25.38	H
ATOM	929	2HG1	VAL	A	255	34.509	−31.192	−16.914	1.00	25.38	H
ATOM	930	3HG1	VAL	A	255	34.594	−31.123	−15.138	1.00	25.38	H
ATOM	931	1HG2	VAL	A	255	31.541	−31.524	−17.074	1.00	25.38	H
ATOM	932	2HG2	VAL	A	255	31.489	−33.302	−17.095	1.00	25.38	H
ATOM	933	3HG2	VAL	A	255	32.730	−32.437	−18.033	1.00	25.38	H
ATOM	934	N	LEU	A	256	35.668	−34.914	−14.577	1.00	25.04	N
ATOM	935	CA	LEU	A	256	36.375	−35.344	−13.362	1.00	24.83	C
ATOM	936	C	LEU	A	256	37.560	−34.429	−12.982	1.00	24.43	C
ATOM	937	O	LEU	A	256	38.222	−33.832	−13.839	1.00	24.39	O
ATOM	938	CB	LEU	A	256	36.827	−36.814	−13.502	1.00	25.04	C
ATOM	939	CG	LEU	A	256	35.766	−37.805	−14.024	1.00	25.50	C
ATOM	940	CD1	LEU	A	256	36.387	−39.191	−14.190	1.00	25.67	C
ATOM	941	CD2	LEU	A	256	34.568	−37.914	−13.081	1.00	25.62	C
ATOM	942	HN	LEU	A	256	35.735	−35.486	−15.405	1.00	0.00	H
ATOM	943	HA	LEU	A	256	35.659	−35.303	−12.541	1.00	25.07	H
ATOM	944	HB1	LEU	A	256	37.672	−36.838	−14.190	1.00	25.07	H
ATOM	945	HB2	LEU	A	256	37.149	−37.158	−12.519	1.00	25.07	H
ATOM	946	HG	LEU	A	256	35.415	−37.461	−14.997	1.00	25.07	H
ATOM	947	1HD1	LEU	A	256	35.633	−39.886	−14.559	1.00	25.07	H
ATOM	948	2HD1	LEU	A	256	36.762	−39.539	−13.228	1.00	25.07	H
ATOM	949	3HD1	LEU	A	256	37.210	−39.138	−14.902	1.00	25.07	H
ATOM	950	1HD2	LEU	A	256	33.847	−38.622	−13.489	1.00	25.07	H
ATOM	951	2HD2	LEU	A	256	34.097	−36.937	−12.977	1.00	25.07	H
ATOM	952	3HD2	LEU	A	256	34.904	−38.261	−12.104	1.00	25.07	H
ATOM	953	N	ASP	A	257	37.946	−34.451	−11.706	1.00	24.14	N
ATOM	954	CA	ASP	A	257	39.196	−33.878	−11.194	1.00	23.75	C

ATOM	955	C	ASP	A	257	40.246	−34.976	−10.893	1.00	23.70	C
ATOM	956	O	ASP	A	257	40.280	−36.018	−11.554	1.00	23.88	O
ATOM	957	CB	ASP	A	257	38.898	−32.979	−9.981	1.00	23.51	C
ATOM	958	CG	ASP	A	257	38.367	−33.704	−8.737	1.00	23.67	C
ATOM	959	OD1	ASP	A	257	38.344	−33.037	−7.682	1.00	23.58	O
ATOM	960	OD2	ASP	A	257	38.141	−34.932	−8.817	1.00	23.93	O
ATOM	961	HN	ASP	A	257	37.426	−35.012	−11.041	1.00	0.00	H
ATOM	962	HA	ASP	A	257	39.607	−33.241	−11.978	1.00	23.77	H
ATOM	963	HB1	ASP	A	257	39.822	−32.471	−9.705	1.00	23.77	H
ATOM	964	HB2	ASP	A	257	38.154	−32.242	−10.284	1.00	23.77	H
ATOM	965	N	ARG	A	258	41.282	−34.591	−10.139	1.00	31.69	N
ATOM	966	CA	ARG	A	258	42.198	−35.448	−9.365	1.00	31.57	C
ATOM	967	C	ARG	A	258	42.535	−34.675	−8.076	1.00	31.15	C
ATOM	968	O	ARG	A	258	42.035	−33.573	−7.877	1.00	31.05	O
ATOM	969	CB	ARG	A	258	43.478	−35.822	−10.155	1.00	31.63	C
ATOM	970	CG	ARG	A	258	43.325	−36.719	−11.398	1.00	32.06	C
ATOM	971	CD	ARG	A	258	43.109	−36.073	−12.781	1.00	32.29	C
ATOM	972	NE	ARG	A	258	43.115	−34.595	−12.795	1.00	32.04	N
ATOM	973	CZ	ARG	A	258	42.185	−33.803	−13.287	1.00	32.01	C
ATOM	974	NH1	ARG	A	258	41.069	−34.259	−13.779	1.00	32.22	N
ATOM	975	NH2	ARG	A	258	42.341	−32.522	−13.233	1.00	31.79	N
ATOM	976	HN	ARG	A	258	41.182	−33.660	−9.752	1.00	0.00	H
ATOM	977	HE	ARG	A	258	43.899	−34.109	−12.353	1.00	0.00	H
ATOM	978	1HH1	ARG	A	258	40.810	−35.185	−13.468	1.00	0.00	H
ATOM	979	2HH1	ARG	A	258	40.301	−33.622	−13.952	1.00	0.00	H
ATOM	980	1HH2	ARG	A	258	43.031	−32.183	−12.557	1.00	0.00	H
ATOM	981	2HH2	ARG	A	258	41.523	−31.931	−13.333	1.00	0.00	H

ATOM	982	HA	ARG	A	258	41.674	−36.365	−9.095	1.00	31.77	H
ATOM	983	HB1	ARG	A	258	43.940	−34.891	−10.482	1.00	31.77	H
ATOM	984	HB2	ARG	A	258	44.146	−36.337	−9.464	1.00	31.77	H
ATOM	985	HG1	ARG	A	258	44.231	−37.321	−11.468	1.00	31.77	H
ATOM	986	HG2	ARG	A	258	42.470	−37.370	−11.215	1.00	31.77	H
ATOM	987	HD1	ARG	A	258	43.903	−36.419	−13.442	1.00	31.77	H
ATOM	988	HD2	ARG	A	258	42.145	−36.409	−13.163	1.00	31.77	H
ATOM	989	N	GLY	A	259	43.596	−35.062	−7.362	1.00	41.93	N
ATOM	990	CA	GLY	A	259	44.176	−34.263	−6.269	1.00	41.54	C
ATOM	991	C	GLY	A	259	44.690	−32.857	−6.646	1.00	41.30	C
ATOM	992	O	GLY	A	259	44.931	−32.060	−5.744	1.00	41.02	O
ATOM	993	HN	GLY	A	259	44.026	−35.941	−7.599	1.00	0.00	H
ATOM	994	HA1	GLY	A	259	43.409	−34.142	−5.504	1.00	41.45	H
ATOM	995	HA2	GLY	A	259	45.015	−34.824	−5.857	1.00	41.45	H
ATOM	996	N	ASP	A	260	44.791	−32.516	−7.939	1.00	50.86	N
ATOM	997	CA	ASP	A	260	45.023	−31.142	−8.429	1.00	50.70	C
ATOM	998	C	ASP	A	260	43.776	−30.226	−8.348	1.00	50.79	C
ATOM	999	O	ASP	A	260	43.902	−28.999	−8.368	1.00	50.71	O
ATOM	1000	CB	ASP	A	260	45.555	−31.212	−9.873	1.00	50.84	C
ATOM	1001	CG	ASP	A	260	44.575	−31.843	−10.870	1.00	50.90	C
ATOM	1002	OD1	ASP	A	260	44.907	−32.873	−11.493	1.00	50.67	O
ATOM	1003	OD2	ASP	A	260	43.434	−31.354	−11.002	1.00	51.19	O
ATOM	1004	HN	ASP	A	260	44.544	−33.209	−8.628	1.00	0.00	H
ATOM	1005	HA	ASP	A	260	45.802	−30.697	−7.810	1.00	50.83	H
ATOM	1006	HB1	ASP	A	260	45.777	−30.198	−10.206	1.00	50.83	H
ATOM	1007	HB2	ASP	A	260	46.471	−31.803	−9.872	1.00	50.83	H
ATOM	1008	N	LYS	A	261	42.591	−30.830	−8.185	1.00	22.34	N

ATOM 1009 CA LYS A 261 41.266 −30.221 −7.978 1.00 22.44 C

ATOM 1010 C LYS A 261 40.802 −29.293 −9.107 1.00 22.62 C

ATOM 1011 O LYS A 261 40.002 −28.384 −8.881 1.00 22.63 O

ATOM 1012 CB LYS A 261 41.139 −29.638 −6.559 1.00 22.15 C

ATOM 1013 CG LYS A 261 41.757 −30.591 −5.520 1.00 21.90 C

ATOM 1014 CD LYS A 261 41.205 −30.392 −4.115 1.00 21.66 C

ATOM 1015 CE LYS A 261 41.977 −31.307 −3.158 1.00 21.44 C

ATOM 1016 NZ LYS A 261 41.169 −31.616 −1.960 1.00 21.17 N1+

ATOM 1017 HN LYS A 261 42.608 −31.843 −8.160 1.00 0.00 H

ATOM 1018 HZ1 LYS A 261 41.681 −31.991 −1.183 1.00 0.00 H

ATOM 1019 HZ2 LYS A 261 40.403 −32.259 −2.193 1.00 0.00 H

ATOM 1020 HZ3 LYS A 261 40.583 −30.822 −1.686 1.00 0.00 H

ATOM 1021 HA LYS A 261 40.568 −31.058 −8.000 1.00 22.04 H

ATOM 1022 HB1 LYS A 261 41.658 −28.680 −6.518 1.00 22.04 H

ATOM 1023 HB2 LYS A 261 40.084 −29.491 −6.326 1.00 22.04 H

ATOM 1024 HG1 LYS A 261 41.555 −31.617 −5.829 1.00 22.04 H

ATOM 1025 HG2 LYS A 261 42.834 −30.424 −5.495 1.00 22.04 H

ATOM 1026 HD1 LYS A 261 41.333 −29.352 −3.813 1.00 22.04 H

ATOM 1027 HD2 LYS A 261 40.146 −30.649 −4.096 1.00 22.04 H

ATOM 1028 HE1 LYS A 261 42.223 −32.236 −3.672 1.00 22.04 H

ATOM 1029 HE2 LYS A 261 42.896 −30.808 −2.850 1.00 22.04 H

ATOM 1030 N VAL A 262 41.310 −29.508 −10.324 1.00 46.68 N

ATOM 1031 CA VAL A 262 40.851 −28.860 −11.563 1.00 46.91 C

ATOM 1032 C VAL A 262 39.850 −29.779 −12.285 1.00 47.22 C

ATOM 1033 O VAL A 262 40.228 −30.897 −12.642 1.00 47.27 O

ATOM 1034 CB VAL A 262 42.044 −28.531 −12.488 1.00 46.93 C

ATOM 1035 CG1 VAL A 262 41.592 −27.774 −13.744 1.00 47.26 C

ATOM 1036 CG2 VAL A 262 43.100 −27.665 −11.783 1.00 46.71 C
ATOM 1037 HN VAL A 262 42.001 −30.251 −10.416 1.00 0.00 H
ATOM 1038 HA VAL A 262 40.345 −27.930 −11.303 1.00 47.00 H
ATOM 1039 HB VAL A 262 42.512 −29.466 −12.796 1.00 47.00 H
ATOM 1040 1HG1 VAL A 262 42.458 −27.560 −14.371 1.00 47.00 H
ATOM 1041 2HG1 VAL A 262 41.114 −26.838 −13.453 1.00 47.00 H
ATOM 1042 3HG1 VAL A 262 40.883 −28.385 −14.302 1.00 47.00 H
ATOM 1043 1HG2 VAL A 262 43.920 −27.459 −12.471 1.00 47.00 H
ATOM 1044 2HG2 VAL A 262 43.483 −28.196 −10.911 1.00 47.00 H
ATOM 1045 3HG2 VAL A 262 42.647 −26.725 −11.466 1.00 47.00 H
ATOM 1046 N PRO A 263 38.633 −29.325 −12.636 1.00 23.53 N
ATOM 1047 CA PRO A 263 37.737 −30.097 −13.493 1.00 23.88 C
ATOM 1048 C PRO A 263 38.317 −30.226 −14.910 1.00 24.06 C
ATOM 1049 O PRO A 263 38.679 −29.220 −15.524 1.00 23.93 O
ATOM 1050 CB PRO A 263 36.395 −29.355 −13.495 1.00 24.04 C
ATOM 1051 CG PRO A 263 36.512 −28.293 −12.401 1.00 23.78 C
ATOM 1052 CD PRO A 263 38.012 −28.077 −12.239 1.00 23.49 C
ATOM 1053 HA PRO A 263 37.599 −31.091 −13.068 1.00 23.82 H
ATOM 1054 HB1 PRO A 263 36.221 −28.886 −14.463 1.00 23.82 H
ATOM 1055 HB2 PRO A 263 35.579 −30.043 −13.272 1.00 23.82 H
ATOM 1056 HG1 PRO A 263 36.021 −27.369 −12.707 1.00 23.82 H
ATOM 1057 HG2 PRO A 263 36.071 −28.649 −11.470 1.00 23.82 H
ATOM 1058 HD1 PRO A 263 38.350 −27.263 −12.881 1.00 23.82 H
ATOM 1059 HD2 PRO A 263 38.253 −27.846 −11.201 1.00 23.82 H
ATOM 1060 N SER A 264 38.291 −31.436 −15.468 1.00 24.37 N
ATOM 1061 CA SER A 264 38.810 −31.763 −16.805 1.00 24.59 C
ATOM 1062 C SER A 264 37.825 −32.657 −17.558 1.00 24.99 C

ATOM 1063 O SER A 264 37.125 −33.456 −16.944 1.00 25.12 O

ATOM 1064 CB SER A 264 40.164 −32.477 −16.705 1.00 24.49 C

ATOM 1065 OG SER A 264 41.124 −31.675 −16.042 1.00 24.25 O

ATOM 1066 HN SER A 264 37.953 −32.215 −14.909 1.00 0.00 H

ATOM 1067 HG SER A 264 41.895 −32.214 −15.835 1.00 0.00 H

ATOM 1068 HA SER A 264 38.942 −30.838 −17.366 1.00 24.64 H

ATOM 1069 HB1 SER A 264 40.034 −33.406 −16.150 1.00 24.63 H

ATOM 1070 HB2 SER A 264 40.522 −32.700 −17.710 1.00 24.63 H

ATOM 1071 N MET A 265 37.794 −32.583 −18.893 1.00 25.19 N

ATOM 1072 CA MET A 265 36.953 −33.487 −19.689 1.00 25.58 C

ATOM 1073 C MET A 265 37.548 −34.907 −19.730 1.00 25.81 C

ATOM 1074 O MET A 265 38.710 −35.079 −20.101 1.00 25.75 O

ATOM 1075 CB MET A 265 36.683 −32.908 −21.096 1.00 25.71 C

ATOM 1076 CG MET A 265 37.801 −33.132 −22.126 1.00 26.00 C

ATOM 1077 SD MET A 265 37.475 −32.467 −23.779 1.00 26.23 S

ATOM 1078 CE MET A 265 38.726 −33.401 −24.702 1.00 26.60 C

ATOM 1079 HN MET A 265 38.430 −31.965 −19.370 1.00 0.00 H

ATOM 1080 HA MET A 265 35.991 −33.558 −19.182 1.00 25.86 H

ATOM 1081 HB1 MET A 265 35.774 −33.370 −21.481 1.00 25.86 H

ATOM 1082 HB2 MET A 265 36.530 −31.834 −20.994 1.00 25.86 H

ATOM 1083 HG1 MET A 265 38.708 −32.661 −21.746 1.00 25.86 H

ATOM 1084 HG2 MET A 265 37.961 −34.206 −22.221 1.00 25.86 H

ATOM 1085 HE1 MET A 265 38.681 −33.126 −25.756 1.00 25.86 H

ATOM 1086 HE2 MET A 265 38.533 −34.469 −24.597 1.00 25.86 H

ATOM 1087 HE3 MET A 265 39.716 −33.171 −24.308 1.00 25.86 H

ATOM 1088 N PHE A 266 36.693 −35.919 −19.593 1.00 34.60 N

ATOM 1089 CA PHE A 266 36.995 −37.317 −19.899 1.00 34.90 C

ATOM 1090 C PHE A 266 35.923 −37.859 −20.844 1.00 35.34 C

ATOM 1091 O PHE A 266 34.844 −38.255 −20.409 1.00 35.52 O

ATOM 1092 CB PHE A 266 37.115 −38.134 −18.601 1.00 34.77 C

ATOM 1093 CG PHE A 266 38.318 −37.750 −17.761 1.00 34.36 C

ATOM 1094 CD1 PHE A 266 39.526 −38.464 −17.878 1.00 34.35 C

ATOM 1095 CD2 PHE A 266 38.245 −36.648 −16.892 1.00 33.99 C

ATOM 1096 CE1 PHE A 266 40.654 −38.076 −17.131 1.00 33.96 C

ATOM 1097 CE2 PHE A 266 39.373 −36.255 −16.155 1.00 33.61 C

ATOM 1098 CZ PHE A 266 40.578 −36.967 −16.270 1.00 33.59 C

ATOM 1099 HN PHE A 266 35.799 −35.736 −19.143 1.00 0.00 H

ATOM 1100 HA PHE A 266 37.954 −37.354 −20.415 1.00 34.45 H

ATOM 1101 HB1 PHE A 266 36.214 −37.976 −18.008 1.00 34.45 H

ATOM 1102 HB2 PHE A 266 37.198 −39.189 −18.862 1.00 34.45 H

ATOM 1103 HD1 PHE A 266 39.588 −39.320 −18.550 1.00 34.45 H

ATOM 1104 HD2 PHE A 266 37.310 −36.097 −16.790 1.00 34.45 H

ATOM 1105 HE1 PHE A 266 41.586 −38.635 −17.220 1.00 34.45 H

ATOM 1106 HE2 PHE A 266 39.313 −35.393 −15.490 1.00 34.45 H

ATOM 1107 HZ PHE A 266 41.451 −36.661 −15.694 1.00 34.45 H

ATOM 1108 N MET A 267 36.181 −37.771 −22.155 1.00 32.46 N

ATOM 1109 CA MET A 267 35.320 −38.365 −23.188 1.00 32.90 C

ATOM 1110 C MET A 267 35.211 −39.876 −22.971 1.00 33.24 C

ATOM 1111 O MET A 267 36.225 −40.571 −22.988 1.00 33.24 O

ATOM 1112 CB MET A 267 35.903 −38.117 −24.590 1.00 33.04 C

ATOM 1113 CG MET A 267 35.610 −36.726 −25.154 1.00 32.94 C

ATOM 1114 SD MET A 267 33.900 −36.525 −25.725 1.00 33.35 S

ATOM 1115 CE MET A 267 34.105 −35.118 −26.844 1.00 33.21 C

ATOM 1116 HN MET A 267 37.089 −37.440 −22.431 1.00 0.00 H

ATOM 1117 HA MET A 267 34.327 −37.920 −23.128 1.00 33.05 H
ATOM 1118 HB1 MET A 267 36.984 −38.244 −24.538 1.00 33.05 H
ATOM 1119 HB2 MET A 267 35.483 −38.857 −25.271 1.00 33.05 H
ATOM 1120 HG1 MET A 267 35.802 −35.990 −24.374 1.00 33.05 H
ATOM 1121 HG2 MET A 267 36.279 −36.547 −25.996 1.00 33.05 H
ATOM 1122 HE1 MET A 267 33.142 −34.861 −27.285 1.00 33.05 H
ATOM 1123 HE2 MET A 267 34.808 −35.381 −27.635 1.00 33.05 H
ATOM 1124 HE3 MET A 267 34.489 −34.263 −26.287 1.00 33.05 H
ATOM 1125 N THR A 268 33.985 −40.387 −22.891 1.00 28.08 N
ATOM 1126 CA THR A 268 33.703 −41.827 −22.769 1.00 28.46 C
ATOM 1127 C THR A 268 33.118 −42.422 −24.052 1.00 28.95 C
ATOM 1128 O THR A 268 33.195 −43.633 −24.258 1.00 29.26 O
ATOM 1129 CB THR A 268 32.724 −42.088 −21.619 1.00 28.53 C
ATOM 1130 OG1 THR A 268 31.494 −41.477 −21.922 1.00 28.68 O
ATOM 1131 CG2 THR A 268 33.207 −41.548 −20.274 1.00 28.08 C
ATOM 1132 HN THR A 268 33.187 −39.755 −22.898 1.00 0.00 H
ATOM 1133 HG1 THR A 268 30.965 −41.423 −21.100 1.00 0.00 H
ATOM 1134 HA THR A 268 34.638 −42.342 −22.547 1.00 28.58 H
ATOM 1135 HB THR A 268 32.568 −43.163 −21.529 1.00 28.58 H
ATOM 1136 1HG2 THR A 268 32.465 −41.768 −19.506 1.00 28.58 H
ATOM 1137 2HG2 THR A 268 33.348 −40.469 −20.344 1.00 28.58 H
ATOM 1138 3HG2 THR A 268 34.153 −42.021 −20.011 1.00 28.58 H
ATOM 1139 N ASN A 269 32.483 −41.596 −24.892 1.00 29.03 N
ATOM 1140 CA ASN A 269 31.578 −42.047 −25.944 1.00 29.52 C
ATOM 1141 C ASN A 269 31.463 −40.992 −27.059 1.00 29.43 C
ATOM 1142 O ASN A 269 31.212 −39.821 −26.777 1.00 29.07 O
ATOM 1143 CB ASN A 269 30.227 −42.297 −25.259 1.00 29.73 C

ATOM 1144 CG ASN A 269 29.172 −42.950 −26.119 1.00 30.31 C
ATOM 1145 OD1 ASN A 269 29.389 −43.365 −27.246 1.00 30.61 O
ATOM 1146 ND2 ASN A 269 28.011 −43.149 −25.555 1.00 30.49 N
ATOM 1147 HN ASN A 269 32.390 −40.630 −24.597 1.00 0.00 H
ATOM 1148 1HD2 ASN A 269 27.888 −42.862 −24.587 1.00 0.00 H
ATOM 1149 2HD2 ASN A 269 27.297 −43.654 −26.061 1.00 0.00 H
ATOM 1150 HA ASN A 269 31.949 −42.981 −26.366 1.00 29.77 H
ATOM 1151 HB1 ASN A 269 30.402 −42.940 −24.397 1.00 29.77 H
ATOM 1152 HB2 ASN A 269 29.838 −41.336 −24.923 1.00 29.77 H
ATOM 1153 N VAL A 270 31.651 −41.389 −28.324 1.00 29.75 N
ATOM 1154 CA VAL A 270 31.692 −40.483 −29.488 1.00 29.65 C
ATOM 1155 C VAL A 270 31.021 −41.129 −30.704 1.00 30.11 C
ATOM 1156 O VAL A 270 31.187 −42.323 −30.953 1.00 30.49 O
ATOM 1157 CB VAL A 270 33.138 −40.040 −29.823 1.00 29.46 C
ATOM 1158 CG1 VAL A 270 33.187 −39.019 −30.971 1.00 29.48 C
ATOM 1159 CG2 VAL A 270 33.861 −39.398 −28.627 1.00 28.96 C
ATOM 1160 HN VAL A 270 31.775 −42.375 −28.506 1.00 0.00 H
ATOM 1161 HA VAL A 270 31.123 −39.589 −29.232 1.00 29.70 H
ATOM 1162 HB VAL A 270 33.702 −40.922 −30.128 1.00 29.70 H
ATOM 1163 1HG1 VAL A 270 34.223 −38.741 −31.167 1.00 29.70 H
ATOM 1164 2HG1 VAL A 270 32.619 −38.131 −30.693 1.00 29.70 H
ATOM 1165 3HG1 VAL A 270 32.754 −39.460 −31.869 1.00 29.70 H
ATOM 1166 1HG2 VAL A 270 34.869 −39.109 −28.924 1.00 29.70 H
ATOM 1167 2HG2 VAL A 270 33.916 −40.115 −27.808 1.00 29.70 H
ATOM 1168 3HG2 VAL A 270 33.311 −38.515 −28.301 1.00 29.70 H
ATOM 1169 N TRP A 271 30.305 −40.328 −31.492 1.00 30.07 N
ATOM 1170 CA TRP A 271 29.620 −40.709 −32.726 1.00 30.51 C

ATOM	1171	C	TRP	A	271	29.911	−39.712	−33.859	1.00	30.47	C
ATOM	1172	O	TRP	A	271	30.058	−38.509	−33.628	1.00	30.13	O
ATOM	1173	CB	TRP	A	271	28.120	−40.844	−32.439	1.00	30.64	C
ATOM	1174	CG	TRP	A	271	27.261	−41.177	−33.621	1.00	31.10	C
ATOM	1175	CD1	TRP	A	271	27.079	−42.416	−34.125	1.00	31.61	C
ATOM	1176	CD2	TRP	A	271	26.498	−40.279	−34.487	1.00	31.10	C
ATOM	1177	NE1	TRP	A	271	26.255	−42.354	−35.231	1.00	31.93	N
ATOM	1178	CE2	TRP	A	271	25.853	−41.062	−35.491	1.00	31.62	C
ATOM	1179	CE3	TRP	A	271	26.269	−38.887	−34.514	1.00	30.73	C
ATOM	1180	CZ2	TRP	A	271	25.015	−40.498	−36.465	1.00	31.75	C
ATOM	1181	CZ3	TRP	A	271	25.418	−38.311	−35.477	1.00	30.87	C
ATOM	1182	CH2	TRP	A	271	24.795	−39.111	−36.453	1.00	31.36	C
ATOM	1183	HN	TRP	A	271	30.218	−39.355	−31.218	1.00	0.00	H
ATOM	1184	HE1	TRP	A	271	26.002	−43.166	−35.774	1.00	0.00	H
ATOM	1185	HA	TRP	A	271	29.994	−41.686	−33.032	1.00	30.99	H
ATOM	1186	HB1	TRP	A	271	27.990	−41.633	−31.698	1.00	30.99	H
ATOM	1187	HB2	TRP	A	271	27.771	−39.897	−32.027	1.00	30.99	H
ATOM	1188	HD1	TRP	A	271	27.517	−43.328	−33.720	1.00	30.99	H
ATOM	1189	HE3	TRP	A	271	26.758	−38.248	−33.779	1.00	30.99	H
ATOM	1190	HZ2	TRP	A	271	24.542	−41.127	−37.219	1.00	30.99	H
ATOM	1191	HZ3	TRP	A	271	25.240	−37.236	−35.466	1.00	30.99	H
ATOM	1192	HH2	TRP	A	271	24.143	−38.655	−37.198	1.00	30.99	H
ATOM	1193	N	THR	A	272	29.888	−40.214	−35.095	1.00	30.85	N
ATOM	1194	CA	THR	A	272	30.163	−39.468	−36.332	1.00	30.86	C
ATOM	1195	C	THR	A	272	29.091	−39.822	−37.372	1.00	31.24	C
ATOM	1196	O	THR	A	272	28.882	−41.012	−37.619	1.00	31.69	O
ATOM	1197	CB	THR	A	272	31.572	−39.808	−36.852	1.00	31.00	C

ATOM 1198 OG1 THR A 272 32.533 −39.287 −35.963 1.00 30.68 O
ATOM 1199 CG2 THR A 272 31.900 −39.245 −38.235 1.00 30.94 C
ATOM 1200 HN THR A 272 29.656 −41.190 −35.201 1.00 0.00 H
ATOM 1201 HG1 THR A 272 33.399 −39.393 −36.365 1.00 0.00 H
ATOM 1202 HA THR A 272 30.112 −38.400 −36.120 1.00 31.04 H
ATOM 1203 HB THR A 272 31.679 −40.892 −36.883 1.00 31.04 H
ATOM 1204 1HG2 THR A 272 32.912 −39.536 −38.516 1.00 31.04 H
ATOM 1205 2HG2 THR A 272 31.829 −38.158 −38.211 1.00 31.04 H
ATOM 1206 3HG2 THR A 272 31.193 −39.639 −38.965 1.00 31.04 H
ATOM 1207 N PRO A 273 28.410 −38.849 −38.012 1.00 31.08 N
ATOM 1208 CA PRO A 273 27.413 −39.142 −39.040 1.00 31.46 C
ATOM 1209 C PRO A 273 28.034 −39.779 −40.297 1.00 31.70 C
ATOM 1210 O PRO A 273 29.108 −39.344 −40.714 1.00 31.47 O
ATOM 1211 CB PRO A 273 26.752 −37.806 −39.394 1.00 31.19 C
ATOM 1212 CG PRO A 273 27.151 −36.850 −38.276 1.00 30.68 C
ATOM 1213 CD PRO A 273 28.472 −37.419 −37.768 1.00 30.60 C
ATOM 1214 HA PRO A 273 26.662 −39.817 −38.630 1.00 31.17 H
ATOM 1215 HB1 PRO A 273 27.117 −37.443 −40.355 1.00 31.17 H
ATOM 1216 HB2 PRO A 273 25.668 −37.915 −39.434 1.00 31.17 H
ATOM 1217 HG1 PRO A 273 27.285 −35.838 −38.659 1.00 31.17 H
ATOM 1218 HG2 PRO A 273 26.401 −36.843 −37.485 1.00 31.17 H
ATOM 1219 HD1 PRO A 273 29.308 −36.978 −38.311 1.00 31.17 H
ATOM 1220 HD2 PRO A 273 28.585 −37.221 −36.702 1.00 31.17 H
ATOM 1221 N PRO A 274 27.249 −40.544 −41.084 1.00 32.17 N
ATOM 1222 CA PRO A 274 27.665 −41.048 −42.400 1.00 32.50 C
ATOM 1223 C PRO A 274 27.792 −39.963 −43.490 1.00 32.32 C
ATOM 1224 O PRO A 274 28.243 −40.257 −44.594 1.00 32.50 O

ATOM 1225 CB PRO A 274 26.621 −42.109 −42.767 1.00 33.03 C

ATOM 1226 CG PRO A 274 25.361 −41.620 −42.056 1.00 32.87 C

ATOM 1227 CD PRO A 274 25.912 −41.024 −40.762 1.00 32.44 C

ATOM 1228 HA PRO A 274 28.631 −41.540 −42.290 1.00 32.55 H

ATOM 1229 HB1 PRO A 274 26.469 −42.151 −43.846 1.00 32.55 H

ATOM 1230 HB2 PRO A 274 26.919 −43.092 −42.403 1.00 32.55 H

ATOM 1231 HG1 PRO A 274 24.845 −40.864 −42.648 1.00 32.55 H

ATOM 1232 HG2 PRO A 274 24.681 −42.447 −41.849 1.00 32.55 H

ATOM 1233 HD1 PRO A 274 25.283 −40.199 −40.426 1.00 32.55 H

ATOM 1234 HD2 PRO A 274 25.959 −41.786 −39.984 1.00 32.55 H

ATOM 1235 N ASN A 275 27.436 −38.706 −43.198 1.00 32.01 N

ATOM 1236 CA ASN A 275 27.847 −37.545 −43.986 1.00 31.73 C

ATOM 1237 C ASN A 275 28.017 −36.307 −43.074 1.00 31.21 C

ATOM 1238 O ASN A 275 27.078 −35.951 −42.359 1.00 31.14 O

ATOM 1239 CB ASN A 275 26.834 −37.294 −45.114 1.00 31.96 C

ATOM 1240 CG ASN A 275 27.359 −36.265 −46.096 1.00 31.71 C

ATOM 1241 OD1 ASN A 275 27.651 −35.133 −45.749 1.00 31.31 O

ATOM 1242 ND2 ASN A 275 27.756 −36.688 −47.272 1.00 31.95 N

ATOM 1243 HN ASN A 275 27.096 −38.522 −42.268 1.00 0.00 H

ATOM 1244 1HD2 ASN A 275 27.652 −37.654 −47.523 1.00 0.00 H

ATOM 1245 2HD2 ASN A 275 28.212 −36.014 −47.860 1.00 0.00 H

ATOM 1246 HA ASN A 275 28.813 −37.767 −44.440 1.00 31.63 H

ATOM 1247 HB1 ASN A 275 26.650 −38.229 −45.642 1.00 31.63 H

ATOM 1248 HB2 ASN A 275 25.902 −36.931 −44.682 1.00 31.63 H

ATOM 1249 N PRO A 276 29.185 −35.635 −43.072 1.00 30.86 N

ATOM 1250 CA PRO A 276 29.442 −34.504 −42.181 1.00 30.41 C

ATOM 1251 C PRO A 276 28.842 −33.176 −42.668 1.00 30.19 C

ATOM 1252 O PRO A 276 28.701 −32.247 −41.877 1.00 29.83 O

ATOM 1253 CB PRO A 276 30.966 −34.435 −42.124 1.00 30.19 C

ATOM 1254 CG PRO A 276 31.396 −34.856 −43.527 1.00 30.41 C

ATOM 1255 CD PRO A 276 30.382 −35.954 −43.845 1.00 30.88 C

ATOM 1256 HA PRO A 276 29.054 −34.732 −41.188 1.00 30.40 H

ATOM 1257 HB1 PRO A 276 31.302 −33.422 −41.902 1.00 30.40 H

ATOM 1258 HB2 PRO A 276 31.359 −35.122 −41.374 1.00 30.40 H

ATOM 1259 HG1 PRO A 276 31.319 −34.028 −44.231 1.00 30.40 H

ATOM 1260 HG2 PRO A 276 32.415 −35.243 −43.531 1.00 30.40 H

ATOM 1261 HD1 PRO A 276 30.153 −35.961 −44.911 1.00 30.40 H

ATOM 1262 HD2 PRO A 276 30.774 −36.928 −43.551 1.00 30.40 H

ATOM 1263 N SER A 277 28.540 −33.051 −43.964 1.00 30.43 N

ATOM 1264 CA SER A 277 28.065 −31.815 −44.598 1.00 30.27 C

ATOM 1265 C SER A 277 26.572 −31.546 −44.394 1.00 30.24 C

ATOM 1266 O SER A 277 26.124 −30.444 −44.695 1.00 29.99 O

ATOM 1267 CB SER A 277 28.344 −31.868 −46.105 1.00 30.54 C

ATOM 1268 OG SER A 277 29.722 −32.085 −46.352 1.00 30.71 O

ATOM 1269 HN SER A 277 28.644 −33.861 −44.568 1.00 0.00 H

ATOM 1270 HG SER A 277 30.202 −31.284 −46.138 1.00 0.00 H

ATOM 1271 HA SER A 277 28.623 −30.979 −44.177 1.00 30.36 H

ATOM 1272 HB1 SER A 277 27.769 −32.682 −46.547 1.00 30.36 H

ATOM 1273 HB2 SER A 277 28.044 −30.923 −46.558 1.00 30.36 H

ATOM 1274 N THR A 278 25.807 −32.544 −43.939 1.00 30.51 N

ATOM 1275 CA THR A 278 24.331 −32.536 −43.903 1.00 30.62 C

ATOM 1276 C THR A 278 23.748 −32.494 −42.482 1.00 30.50 C

ATOM 1277 O THR A 278 22.591 −32.870 −42.298 1.00 30.61 O

ATOM 1278 CB THR A 278 23.777 −33.748 −44.678 1.00 31.11 C

ATOM 1279 OG1 THR A 278 24.192 −34.949 −44.066 1.00 31.31 O

ATOM 1280 CG2 THR A 278 24.235 −33.792 −46.136 1.00 31.28 C

ATOM 1281 HN THR A 278 26.250 −33.442 −43.800 1.00 0.00 H

ATOM 1282 HG1 THR A 278 23.721 −35.016 −43.230 1.00 0.00 H

ATOM 1283 HA THR A 278 23.996 −31.637 −44.420 1.00 30.85 H

ATOM 1284 HB THR A 278 22.688 −33.706 −44.657 1.00 30.85 H

ATOM 1285 1HG2 THR A 278 23.810 −34.669 −46.625 1.00 30.85 H

ATOM 1286 2HG2 THR A 278 25.323 −33.847 −46.174 1.00 30.85 H

ATOM 1287 3HG2 THR A 278 23.899 −32.892 −46.650 1.00 30.85 H

ATOM 1288 N ILE A 279 24.568 −32.199 −41.463 1.00 30.29 N

ATOM 1289 CA ILE A 279 24.195 −32.189 −40.035 1.00 30.12 C

ATOM 1290 C ILE A 279 24.608 −30.855 −39.400 1.00 29.68 C

ATOM 1291 O ILE A 279 25.792 −30.502 −39.419 1.00 29.47 O

ATOM 1292 CB ILE A 279 24.820 −33.400 −39.298 1.00 30.15 C

ATOM 1293 CG1 ILE A 279 24.274 −34.758 −39.802 1.00 30.62 C

ATOM 1294 CG2 ILE A 279 24.653 −33.302 −37.769 1.00 29.89 C

ATOM 1295 CD1 ILE A 279 22.788 −35.028 −39.525 1.00 30.76 C

ATOM 1296 HN ILE A 279 25.499 −31.886 −41.699 1.00 0.00 H

ATOM 1297 HA ILE A 279 23.110 −32.275 −39.968 1.00 30.12 H

ATOM 1298 HB ILE A 279 25.890 −33.386 −39.506 1.00 30.12 H

ATOM 1299 1HG1 ILE A 279 24.425 −34.799 −40.881 1.00 30.12 H

ATOM 1300 2HG1 ILE A 279 24.852 −35.549 −39.323 1.00 30.12 H

ATOM 1301 1HG2 ILE A 279 25.107 −34.173 −37.297 1.00 30.12 H

ATOM 1302 2HG2 ILE A 279 23.592 −33.266 −37.520 1.00 30.12 H

ATOM 1303 3HG2 ILE A 279 25.142 −32.397 −37.407 1.00 30.12 H

ATOM 1304 1HD1 ILE A 279 22.516 −36.006 −39.921 1.00 30.12 H

ATOM 1305 2HD1 ILE A 279 22.183 −34.260 −40.007 1.00 30.12 H

ATOM 1306 3HD1 ILE A 279 22.609 −35.009 −38.450 1.00 30.12 H
ATOM 1307 N HIS A 280 23.664 −30.163 −38.754 1.00 29.56 N
ATOM 1308 CA HIS A 280 23.795 −28.751 −38.365 1.00 29.19 C
ATOM 1309 C HIS A 280 23.184 −28.450 −36.985 1.00 29.06 C
ATOM 1310 O HIS A 280 22.183 −29.059 −36.603 1.00 29.28 O
ATOM 1311 CB HIS A 280 23.124 −27.860 −39.431 1.00 29.17 C
ATOM 1312 CG HIS A 280 23.448 −28.223 −40.857 1.00 29.42 C
ATOM 1313 ND1 HIS A 280 24.583 −27.875 −41.550 1.00 29.30 N
ATOM 1314 CD2 HIS A 280 22.744 −29.094 −41.644 1.00 29.80 C
ATOM 1315 CE1 HIS A 280 24.558 −28.511 −42.732 1.00 29.59 C
ATOM 1316 NE2 HIS A 280 23.461 −29.275 −42.829 1.00 29.90 N
ATOM 1317 HN HIS A 280 22.721 −30.544 −38.757 1.00 0.00 H
ATOM 1318 HD1 HIS A 280 25.298 −27.238 −41.234 1.00 0.00 H
ATOM 1319 HA HIS A 280 24.856 −28.503 −38.331 1.00 29.43 H
ATOM 1320 HB1 HIS A 280 22.044 −27.931 −39.301 1.00 29.43 H
ATOM 1321 HB2 HIS A 280 23.444 −26.832 −39.263 1.00 29.43 H
ATOM 1322 HD2 HIS A 280 21.793 −29.563 −41.391 1.00 29.43 H
ATOM 1323 HE1 HIS A 280 25.322 −28.419 −43.504 1.00 29.43 H
ATOM 1324 N HIS A 281 23.709 −27.410 −36.323 1.00 28.71 N
ATOM 1325 CA HIS A 281 23.065 −26.677 −35.218 1.00 28.56 C
ATOM 1326 C HIS A 281 22.537 −27.561 −34.071 1.00 28.69 C
ATOM 1327 O HIS A 281 21.420 −27.382 −33.587 1.00 28.78 O
ATOM 1328 CB HIS A 281 22.010 −25.694 −35.755 1.00 28.58 C
ATOM 1329 CG HIS A 281 22.453 −24.893 −36.955 1.00 28.41 C
ATOM 1330 ND1 HIS A 281 23.614 −24.157 −37.089 1.00 28.11 N
ATOM 1331 CD2 HIS A 281 21.812 −24.856 −38.163 1.00 28.50 C
ATOM 1332 CE1 HIS A 281 23.649 −23.667 −38.342 1.00 28.04 C

ATOM 1333 NE2 HIS A 281 22.577 −24.080 −39.035 1.00 28.27 N
ATOM 1334 HN HIS A 281 24.543 −26.980 −36.703 1.00 0.00 H
ATOM 1335 HD1 HIS A 281 24.350 −24.049 −36.402 1.00 0.00 H
ATOM 1336 HA HIS A 281 23.848 −26.059 −34.778 1.00 28.47 H
ATOM 1337 HB1 HIS A 281 21.125 −26.265 −36.035 1.00 28.46 H
ATOM 1338 HB2 HIS A 281 21.756 −24.997 −34.956 1.00 28.46 H
ATOM 1339 HD2 HIS A 281 20.869 −25.347 −38.403 1.00 28.46 H
ATOM 1340 HE1 HIS A 281 24.437 −23.026 −38.738 1.00 28.46 H
ATOM 1341 N CYS A 282 23.316 −28.577 −33.691 1.00 28.70 N
ATOM 1342 CA CYS A 282 22.938 −29.526 −32.648 1.00 28.83 C
ATOM 1343 C CYS A 282 22.659 −28.844 −31.294 1.00 28.57 C
ATOM 1344 O CYS A 282 23.218 −27.791 −30.988 1.00 28.26 O
ATOM 1345 CB CYS A 282 24.023 −30.599 −32.525 1.00 28.90 C
ATOM 1346 SG CYS A 282 24.293 −31.607 −34.011 1.00 29.22 S
ATOM 1347 HN CYS A 282 24.207 −28.690 −34.145 1.00 0.00 H
ATOM 1348 HA CYS A 282 22.019 −30.019 −32.966 1.00 28.75 H
ATOM 1349 HB1 CYS A 282 24.962 −30.102 −32.281 1.00 28.75 H
ATOM 1350 HB2 CYS A 282 23.744 −31.268 −31.711 1.00 28.75 H
ATOM 1351 N SER A 283 21.868 −29.506 −30.448 1.00 28.73 N
ATOM 1352 CA SER A 283 21.390 −28.989 −29.163 1.00 28.52 C
ATOM 1353 C SER A 283 21.182 −30.115 −28.154 1.00 28.71 C
ATOM 1354 O SER A 283 20.615 −31.136 −28.525 1.00 29.07 O
ATOM 1355 CB SER A 283 20.058 −28.282 −29.402 1.00 28.54 C
ATOM 1356 OG SER A 283 19.662 −27.658 −28.204 1.00 28.27 O
ATOM 1357 HN SER A 283 21.394 −30.333 −30.802 1.00 0.00 H
ATOM 1358 HG SER A 283 20.285 −26.913 −28.102 1.00 0.00 H
ATOM 1359 HA SER A 283 22.113 −28.276 −28.767 1.00 28.64 H

ATOM 1360 HB1 SER A 283 20.175 −27.533 −30.185 1.00 28.64 H
ATOM 1361 HB2 SER A 283 19.305 −29.010 −29.704 1.00 28.64 H
ATOM 1362 N SER A 284 21.634 −29.978 −26.903 1.00 28.47 N
ATOM 1363 CA SER A 284 21.833 −31.135 −26.009 1.00 28.60 C
ATOM 1364 C SER A 284 21.270 −30.981 −24.588 1.00 28.50 C
ATOM 1365 O SER A 284 20.991 −29.874 −24.130 1.00 28.17 O
ATOM 1366 CB SER A 284 23.321 −31.492 −25.992 1.00 28.41 C
ATOM 1367 OG SER A 284 24.068 −30.502 −25.322 1.00 28.02 O
ATOM 1368 HN SER A 284 22.062 −29.099 −26.635 1.00 0.00 H
ATOM 1369 HG SER A 284 23.939 −29.631 −25.742 1.00 0.00 H
ATOM 1370 HA SER A 284 21.312 −31.978 −26.464 1.00 28.36 H
ATOM 1371 HB1 SER A 284 23.453 −32.446 −25.481 1.00 28.36 H
ATOM 1372 HB2 SER A 284 23.679 −31.576 −27.018 1.00 28.36 H
ATOM 1373 N THR A 285 20.986 −32.107 −23.921 1.00 28.82 N
ATOM 1374 CA THR A 285 20.414 −32.199 −22.558 1.00 28.79 C
ATOM 1375 C THR A 285 20.697 −33.579 −21.938 1.00 28.95 C
ATOM 1376 O THR A 285 20.427 −34.594 −22.582 1.00 29.31 O
ATOM 1377 CB THR A 285 18.891 −31.979 −22.629 1.00 29.06 C
ATOM 1378 OG1 THR A 285 18.615 −30.635 −22.942 1.00 28.88 O
ATOM 1379 CG2 THR A 285 18.153 −32.260 −21.324 1.00 29.12 C
ATOM 1380 HN THR A 285 21.140 −32.988 −24.405 1.00 0.00 H
ATOM 1381 HG1 THR A 285 19.369 −30.296 −23.451 1.00 0.00 H
ATOM 1382 HA THR A 285 20.858 −31.427 −21.930 1.00 28.99 H
ATOM 1383 HB THR A 285 18.479 −32.616 −23.412 1.00 28.99 H
ATOM 1384 1HG2 THR A 285 17.087 −32.080 −21.463 1.00 28.99 H
ATOM 1385 2HG2 THR A 285 18.533 −31.602 −20.542 1.00 28.99 H
ATOM 1386 3HG2 THR A 285 18.311 −33.299 −21.033 1.00 28.99 H

ATOM 1387 N TYR A 286 21.145 −33.649 −20.677 1.00 28.68 N
ATOM 1388 CA TYR A 286 21.287 −34.919 −19.943 1.00 28.79 C
ATOM 1389 C TYR A 286 19.981 −35.369 −19.274 1.00 29.13 C
ATOM 1390 O TYR A 286 19.447 −34.636 −18.437 1.00 29.15 O
ATOM 1391 CB TYR A 286 22.385 −34.801 −18.881 1.00 28.37 C
ATOM 1392 CG TYR A 286 22.529 −36.010 −17.974 1.00 28.43 C
ATOM 1393 CD1 TYR A 286 23.097 −37.194 −18.477 1.00 28.48 C
ATOM 1394 CD2 TYR A 286 22.058 −35.969 −16.646 1.00 28.45 C
ATOM 1395 CE1 TYR A 286 23.179 −38.336 −17.660 1.00 28.55 C
ATOM 1396 CE2 TYR A 286 22.133 −37.109 −15.827 1.00 28.52 C
ATOM 1397 CZ TYR A 286 22.692 −38.298 −16.337 1.00 28.56 C
ATOM 1398 OH TYR A 286 22.755 −39.404 −15.553 1.00 28.63 O
ATOM 1399 HN TYR A 286 21.417 −32.791 −20.195 1.00 0.00 H
ATOM 1400 HH TYR A 286 22.729 −39.201 −14.618 1.00 0.00 H
ATOM 1401 HA TYR A 286 21.586 −35.689 −20.654 1.00 28.64 H
ATOM 1402 HB1 TYR A 286 23.335 −34.646 −19.392 1.00 28.64 H
ATOM 1403 HB2 TYR A 286 22.162 −33.935 −18.258 1.00 28.64 H
ATOM 1404 HD1 TYR A 286 23.473 −37.227 −19.499 1.00 28.64 H
ATOM 1405 HD2 TYR A 286 21.632 −35.046 −16.251 1.00 28.64 H
ATOM 1406 HE1 TYR A 286 23.620 −39.253 −18.051 1.00 28.64 H
ATOM 1407 HE2 TYR A 286 21.761 −37.074 −14.803 1.00 28.64 H
ATOM 1408 N HIS A 287 19.656 −36.656 −19.397 1.00 29.43 N
ATOM 1409 CA HIS A 287 18.632 −37.364 −18.623 1.00 29.74 C
ATOM 1410 C HIS A 287 19.028 −38.844 −18.440 1.00 29.98 C
ATOM 1411 O HIS A 287 19.458 −39.489 −19.389 1.00 30.22 O
ATOM 1412 CB HIS A 287 17.262 −37.162 −19.303 1.00 30.15 C
ATOM 1413 CG HIS A 287 16.217 −38.179 −18.927 1.00 30.49 C

ATOM 1414 ND1 HIS A 287 16.001 −39.400 −19.576 1.00 31.01 N

ATOM 1415 CD2 HIS A 287 15.449 −38.126 −17.804 1.00 30.40 C

ATOM 1416 CE1 HIS A 287 15.092 −40.045 −18.822 1.00 31.24 C

ATOM 1417 NE2 HIS A 287 14.749 −39.307 −17.754 1.00 30.87 N

ATOM 1418 HN HIS A 287 20.220 −37.235 −20.017 1.00 0.00 H

ATOM 1419 HE2 HIS A 287 14.274 −39.679 −16.923 1.00 0.00 H

ATOM 1420 HA HIS A 287 18.585 −36.906 −17.635 1.00 30.35 H

ATOM 1421 HB1 HIS A 287 16.889 −36.175 −19.029 1.00 30.35 H

ATOM 1422 HB2 HIS A 287 17.408 −37.209 −20.382 1.00 30.35 H

ATOM 1423 HD2 HIS A 287 15.399 −37.308 −17.086 1.00 30.35 H

ATOM 1424 HE1 HIS A 287 14.689 −41.032 −19.048 1.00 30.35 H

ATOM 1425 N GLU A 288 19.054 −39.299 −17.181 1.00 49.70 N

ATOM 1426 CA GLU A 288 19.219 −40.702 −16.737 1.00 49.93 C

ATOM 1427 C GLU A 288 20.207 −41.560 −17.562 1.00 49.97 C

ATOM 1428 O GLU A 288 19.822 −42.364 −18.404 1.00 50.41 O

ATOM 1429 CB GLU A 288 17.835 −41.355 −16.555 1.00 50.49 C

ATOM 1430 CG GLU A 288 17.053 −40.650 −15.429 1.00 50.53 C

ATOM 1431 CD GLU A 288 15.624 −41.182 −15.266 1.00 51.14 C

ATOM 1432 OE1 GLU A 288 15.458 −42.419 −15.209 1.00 51.54 O

ATOM 1433 OE2 GLU A 288 14.707 −40.331 −15.183 1.00 51.23 O

ATOM 1434 HN GLU A 288 18.656 −38.686 −16.491 1.00 0.00 H

ATOM 1435 HA GLU A 288 19.648 −40.643 −15.737 1.00 50.55 H

ATOM 1436 HB1 GLU A 288 17.275 −41.274 −17.486 1.00 50.55 H

ATOM 1437 HB2 GLU A 288 17.965 −42.406 −16.298 1.00 50.55 H

ATOM 1438 HG1 GLU A 288 17.587 −40.798 −14.490 1.00 50.55 H

ATOM 1439 HG2 GLU A 288 17.003 −39.585 −15.656 1.00 50.55 H

ATOM 1440 N ASP A 289 21.507 −41.382 −17.299 1.00 33.41 N

ATOM 1441 CA ASP A 289 22.657 −41.975 −18.005 1.00 33.44 C

ATOM 1442 C ASP A 289 22.823 −41.630 −19.495 1.00 33.66 C

ATOM 1443 O ASP A 289 23.880 −41.935 −20.052 1.00 33.88 O

ATOM 1444 CB ASP A 289 22.789 −43.491 −17.757 1.00 33.75 C

ATOM 1445 CG ASP A 289 23.340 −43.883 −16.386 1.00 33.52 C

ATOM 1446 OD1 ASP A 289 24.017 −43.045 −15.742 1.00 33.39 O

ATOM 1447 OD2 ASP A 289 23.561 −45.103 −16.227 1.00 33.49 O

ATOM 1448 HN ASP A 289 21.744 −40.703 −16.588 1.00 0.00 H

ATOM 1449 HA ASP A 289 23.532 −41.539 −17.522 1.00 33.57 H

ATOM 1450 HB1 ASP A 289 21.800 −43.937 −17.862 1.00 33.57 H

ATOM 1451 HB2 ASP A 289 23.455 −43.900 −18.517 1.00 33.57 H

ATOM 1452 N PHE A 290 21.958 −40.812 −20.097 1.00 29.92 N

ATOM 1453 CA PHE A 290 22.097 −40.378 −21.490 1.00 29.96 C

ATOM 1454 C PHE A 290 22.221 −38.859 −21.614 1.00 29.56 C

ATOM 1455 O PHE A 290 21.340 −38.112 −21.194 1.00 29.39 O

ATOM 1456 CB PHE A 290 20.932 −40.909 −22.335 1.00 30.46 C

ATOM 1457 CG PHE A 290 20.602 −42.373 −22.111 1.00 30.94 C

ATOM 1458 CD1 PHE A 290 21.484 −43.378 −22.546 1.00 31.25 C

ATOM 1459 CD2 PHE A 290 19.443 −42.724 −21.395 1.00 31.12 C

ATOM 1460 CE1 PHE A 290 21.209 −44.727 −22.267 1.00 31.74 C

ATOM 1461 CE2 PHE A 290 19.175 −44.072 −21.102 1.00 31.60 C

ATOM 1462 CZ PHE A 290 20.058 −45.075 −21.537 1.00 31.92 C

ATOM 1463 HN PHE A 290 21.096 −40.572 −19.612 1.00 0.00 H

ATOM 1464 HZ PHE A 290 19.870 −46.103 −21.268 1.00 0.00 H

ATOM 1465 HA PHE A 290 23.015 −40.817 −21.882 1.00 30.71 H

ATOM 1466 HB1 PHE A 290 20.045 −40.321 −22.098 1.00 30.71 H

ATOM 1467 HB2 PHE A 290 21.187 −40.776 −23.386 1.00 30.71 H

ATOM 1468 HD1 PHE A 290 22.383 −43.110 −23.101 1.00 30.71 H

ATOM 1469 HD2 PHE A 290 18.751 −41.948 −21.067 1.00 30.71 H

ATOM 1470 HE1 PHE A 290 21.888 −45.505 −22.616 1.00 30.71 H

ATOM 1471 HE2 PHE A 290 18.282 −44.340 −20.537 1.00 30.71 H

ATOM 1472 N TYR A 291 23.187 −38.398 −22.408 1.00 29.46 N

ATOM 1473 CA TYR A 291 23.070 −37.092 −23.052 1.00 29.29 C

ATOM 1474 C TYR A 291 22.317 −37.260 −24.377 1.00 29.70 C

ATOM 1475 O TYR A 291 22.768 −37.947 −25.300 1.00 29.98 O

ATOM 1476 CB TYR A 291 24.439 −36.414 −23.192 1.00 28.88 C

ATOM 1477 CG TYR A 291 24.534 −35.090 −22.456 1.00 28.44 C

ATOM 1478 CD1 TYR A 291 25.375 −34.949 −21.332 1.00 28.12 C

ATOM 1479 CD2 TYR A 291 23.805 −33.983 −22.932 1.00 28.34 C

ATOM 1480 CE1 TYR A 291 25.460 −33.708 −20.668 1.00 27.73 C

ATOM 1481 CE2 TYR A 291 23.900 −32.741 −22.275 1.00 27.97 C

ATOM 1482 CZ TYR A 291 24.709 −32.609 −21.131 1.00 27.66 C

ATOM 1483 OH TYR A 291 24.703 −31.442 −20.443 1.00 27.31 O

ATOM 1484 HN TYR A 291 23.824 −39.069 −22.825 1.00 0.00 H

ATOM 1485 HH TYR A 291 23.810 −31.266 −20.096 1.00 0.00 H

ATOM 1486 HA TYR A 291 22.458 −36.463 −22.406 1.00 28.57 H

ATOM 1487 HB1 TYR A 291 25.199 −37.086 −22.794 1.00 28.57 H

ATOM 1488 HB2 TYR A 291 24.629 −36.235 −24.250 1.00 28.57 H

ATOM 1489 HD1 TYR A 291 25.958 −35.799 −20.977 1.00 28.57 H

ATOM 1490 HD2 TYR A 291 23.167 −34.088 −23.809 1.00 28.57 H

ATOM 1491 HE1 TYR A 291 26.106 −33.599 −19.797 1.00 28.57 H

ATOM 1492 HE2 TYR A 291 23.347 −31.881 −22.653 1.00 28.57 H

ATOM 1493 N TYR A 292 21.108 −36.708 −24.427 1.00 37.87 N

ATOM 1494 CA TYR A 292 20.309 −36.565 −25.639 1.00 38.21 C

ATOM 1495 C TYR A 292 20.795 −35.329 −26.406 1.00 37.92 C
ATOM 1496 O TYR A 292 21.166 −34.321 −25.799 1.00 37.57 O
ATOM 1497 CB TYR A 292 18.822 −36.436 −25.274 1.00 38.48 C
ATOM 1498 CG TYR A 292 18.215 −37.617 −24.528 1.00 38.79 C
ATOM 1499 CD1 TYR A 292 17.333 −38.504 −25.181 1.00 39.29 C
ATOM 1500 CD2 TYR A 292 18.480 −37.795 −23.155 1.00 38.61 C
ATOM 1501 CE1 TYR A 292 16.731 −39.565 −24.472 1.00 39.62 C
ATOM 1502 CE2 TYR A 292 17.884 −38.856 −22.451 1.00 38.91 C
ATOM 1503 CZ TYR A 292 17.000 −39.735 −23.098 1.00 39.42 C
ATOM 1504 OH TYR A 292 16.403 −40.727 −22.390 1.00 39.75 O
ATOM 1505 HN TYR A 292 20.815 −36.154 −23.628 1.00 0.00 H
ATOM 1506 HH TYR A 292 16.318 −40.465 −21.458 1.00 0.00 H
ATOM 1507 HA TYR A 292 20.447 −37.448 −26.263 1.00 38.70 H
ATOM 1508 HB1 TYR A 292 18.708 −35.551 −24.648 1.00 38.70 H
ATOM 1509 HB2 TYR A 292 18.262 −36.302 −26.199 1.00 38.70 H
ATOM 1510 HD1 TYR A 292 17.115 −38.369 −26.240 1.00 38.70 H
ATOM 1511 HD2 TYR A 292 19.149 −37.108 −22.637 1.00 38.70 H
ATOM 1512 HE1 TYR A 292 16.059 −40.252 −24.986 1.00 38.70 H
ATOM 1513 HE2 TYR A 292 18.110 −38.998 −21.394 1.00 38.70 H
ATOM 1514 N THR A 293 20.795 −35.383 −27.738 1.00 29.96 N
ATOM 1515 CA THR A 293 21.269 −34.285 −28.587 1.00 29.69 C
ATOM 1516 C THR A 293 20.570 −34.263 −29.950 1.00 29.95 C
ATOM 1517 O THR A 293 20.570 −35.239 −30.697 1.00 30.26 O
ATOM 1518 CB THR A 293 22.803 −34.280 −28.675 1.00 29.41 C
ATOM 1519 OG1 THR A 293 23.254 −33.191 −29.445 1.00 29.45 O
ATOM 1520 CG2 THR A 293 23.419 −35.541 −29.274 1.00 29.54 C
ATOM 1521 HN THR A 293 20.556 −36.258 −28.195 1.00 0.00 H

ATOM 1522 HG1 THR A 293 24.213 −33.217 −29.434 1.00 0.00 H

ATOM 1523 HA THR A 293 20.987 −33.361 −28.082 1.00 29.75 H

ATOM 1524 HB THR A 293 23.196 −34.162 −27.665 1.00 29.75 H

ATOM 1525 1HG2 THR A 293 24.504 −35.442 −29.294 1.00 29.75 H

ATOM 1526 2HG2 THR A 293 23.049 −35.679 −30.290 1.00 29.75 H

ATOM 1527 3HG2 THR A 293 23.144 −36.404 −28.667 1.00 29.75 H

ATOM 1528 N LEU A 294 19.791 −33.206 −30.166 1.00 29.82 N

ATOM 1529 CA LEU A 294 18.923 −32.956 −31.311 1.00 30.04 C

ATOM 1530 C LEU A 294 19.660 −32.108 −32.360 1.00 29.83 C

ATOM 1531 O LEU A 294 20.055 −30.983 −32.064 1.00 29.50 O

ATOM 1532 CB LEU A 294 17.666 −32.257 −30.754 1.00 30.03 C

ATOM 1533 CG LEU A 294 16.650 −31.785 −31.804 1.00 30.29 C

ATOM 1534 CD1 LEU A 294 16.073 −32.945 −32.607 1.00 30.80 C

ATOM 1535 CD2 LEU A 294 15.499 −31.050 −31.119 1.00 30.22 C

ATOM 1536 HN LEU A 294 19.866 −32.450 −29.493 1.00 0.00 H

ATOM 1537 HA LEU A 294 18.636 −33.908 −31.757 1.00 30.07 H

ATOM 1538 HB1 LEU A 294 17.160 −32.957 −30.089 1.00 30.07 H

ATOM 1539 HB2 LEU A 294 17.991 −31.385 −30.186 1.00 30.07 H

ATOM 1540 HG LEU A 294 17.147 −31.098 −32.489 1.00 30.07 H

ATOM 1541 1HD1 LEU A 294 15.360 −32.563 −33.337 1.00 30.07 H

ATOM 1542 2HD1 LEU A 294 15.568 −33.638 −31.934 1.00 30.07 H

ATOM 1543 3HD1 LEU A 294 16.879 −33.465 −33.125 1.00 30.07 H

ATOM 1544 1HD2 LEU A 294 14.782 −30.718 −31.869 1.00 30.07 H

ATOM 1545 2HD2 LEU A 294 15.888 −30.185 −30.581 1.00 30.07 H

ATOM 1546 3HD2 LEU A 294 15.005 −31.722 −30.417 1.00 30.07 H

ATOM 1547 N CYS A 295 19.743 −32.583 −33.602 1.00 30.05 N

ATOM 1548 CA CYS A 295 20.414 −31.915 −34.723 1.00 29.93 C

ATOM 1549 C CYS A 295 19.472 −31.741 −35.926 1.00 30.19 C

ATOM 1550 O CYS A 295 18.683 −32.635 −36.254 1.00 30.50 O

ATOM 1551 CB CYS A 295 21.642 −32.731 −35.146 1.00 30.05 C

ATOM 1552 SG CYS A 295 22.896 −33.093 −33.883 1.00 29.80 S

ATOM 1553 HN CYS A 295 19.356 −33.503 −33.784 1.00 0.00 H

ATOM 1554 HA CYS A 295 20.746 −30.930 −34.394 1.00 30.09 H

ATOM 1555 HB1 CYS A 295 21.281 −33.686 −35.529 1.00 30.09 H

ATOM 1556 HB2 CYS A 295 22.137 −32.182 −35.947 1.00 30.09 H

ATOM 1557 N ALA A 296 19.672 −30.664 −36.686 1.00 30.06 N

ATOM 1558 CA ALA A 296 19.045 −30.474 −37.992 1.00 30.32 C

ATOM 1559 C ALA A 296 19.756 −31.302 −39.076 1.00 30.46 C

ATOM 1560 O ALA A 296 20.987 −31.283 −39.164 1.00 30.28 O

ATOM 1561 CB ALA A 296 19.064 −28.979 −38.317 1.00 30.10 C

ATOM 1562 HN ALA A 296 20.419 −30.021 −36.438 1.00 0.00 H

ATOM 1563 HA ALA A 296 18.007 −30.802 −37.930 1.00 30.24 H

ATOM 1564 HB1 ALA A 296 18.600 −28.812 −39.289 1.00 30.24 H

ATOM 1565 HB2 ALA A 296 20.095 −28.625 −38.341 1.00 30.24 H

ATOM 1566 HB3 ALA A 296 18.511 −28.433 −37.553 1.00 30.24 H

ATOM 1567 N VAL A 297 18.984 −31.950 −39.951 1.00 30.78 N

ATOM 1568 CA VAL A 297 19.468 −32.678 −41.134 1.00 30.98 C

ATOM 1569 C VAL A 297 19.127 −31.875 −42.389 1.00 30.87 C

ATOM 1570 O VAL A 297 18.004 −31.389 −42.514 1.00 30.84 O

ATOM 1571 CB VAL A 297 18.851 −34.089 −41.227 1.00 31.52 C

ATOM 1572 CG1 VAL A 297 19.548 −34.931 −42.304 1.00 31.76 C

ATOM 1573 CG2 VAL A 297 18.906 −34.861 −39.903 1.00 31.70 C

ATOM 1574 HN VAL A 297 17.974 −31.892 −39.848 1.00 0.00 H

ATOM 1575 HA VAL A 297 20.552 −32.775 −41.065 1.00 31.21 H

ATOM 1576 HB VAL A 297 17.804 −33.980 −41.510 1.00 31.21 H

ATOM 1577 1HG1 VAL A 297 19.091 −35.920 −42.345 1.00 31.21 H

ATOM 1578 2HG1 VAL A 297 20.606 −35.030 −42.060 1.00 31.21 H

ATOM 1579 3HG1 VAL A 297 19.443 −34.442 −43.273 1.00 31.21 H

ATOM 1580 1HG2 VAL A 297 18.456 −35.845 −40.037 1.00 31.21 H

ATOM 1581 2HG2 VAL A 297 18.357 −34.312 −39.138 1.00 31.21 H

ATOM 1582 3HG2 VAL A 297 19.944 −34.976 −39.592 1.00 31.21 H

ATOM 1583 N SER A 298 20.038 −31.756 −43.360 1.00 30.82 N

ATOM 1584 CA SER A 298 19.801 −30.929 −44.555 1.00 30.79 C

ATOM 1585 C SER A 298 20.377 −31.516 −45.841 1.00 30.94 C

ATOM 1586 O SER A 298 21.553 −31.866 −45.908 1.00 30.88 O

ATOM 1587 CB SER A 298 20.364 −29.529 −44.336 1.00 30.35 C

ATOM 1588 OG SER A 298 20.047 −28.697 −45.428 1.00 30.29 O

ATOM 1589 HN SER A 298 20.962 −32.151 −43.211 1.00 0.00 H

ATOM 1590 HG SER A 298 20.532 −27.860 −45.252 1.00 0.00 H

ATOM 1591 HA SER A 298 18.723 −30.836 −44.686 1.00 30.68 H

ATOM 1592 HB1 SER A 298 19.935 −29.108 −43.427 1.00 30.68 H

ATOM 1593 HB2 SER A 298 21.447 −29.590 −44.233 1.00 30.68 H

ATOM 1594 N HIS A 299 19.592 −31.447 −46.922 1.00 31.13 N

ATOM 1595 CA HIS A 299 20.047 −31.680 −48.302 1.00 31.31 C

ATOM 1596 C HIS A 299 20.580 −30.411 −48.994 1.00 30.95 C

ATOM 1597 O HIS A 299 21.123 −30.503 −50.090 1.00 30.98 O

ATOM 1598 CB HIS A 299 18.896 −32.314 −49.115 1.00 31.69 C

ATOM 1599 CG HIS A 299 19.210 −33.639 −49.768 1.00 32.16 C

ATOM 1600 ND1 HIS A 299 18.514 −34.157 −50.860 1.00 32.47 N

ATOM 1601 CD2 HIS A 299 20.090 −34.588 −49.327 1.00 32.40 C

ATOM 1602 CE1 HIS A 299 19.014 −35.379 −51.081 1.00 32.89 C

```
ATOM   1603  NE2 HIS A 299      19.964 −35.666 −50.176  1.00 32.86           N
ATOM   1604  HN  HIS A 299      18.648 −31.115 −46.793  1.00  0.00           H
ATOM   1605  HE2 HIS A 299      20.477 −36.534 −50.123  1.00  0.00           H
ATOM   1606  HA  HIS A 299      20.862 −32.403 −48.264  1.00 31.88           H
ATOM   1607  HB1 HIS A 299      18.053 −32.464 −48.441  1.00 31.88           H
ATOM   1608  HB2 HIS A 299      18.614 −31.612 −49.900  1.00 31.88           H
ATOM   1609  HD2 HIS A 299      20.760 −34.508 −48.471  1.00 31.88           H
ATOM   1610  HE1 HIS A 299      18.695 −36.046 −51.882  1.00 31.88           H
ATOM   1611  N   VAL A 300      20.430 −29.234 −48.373  1.00 37.47           N
ATOM   1612  CA  VAL A 300      20.701 −27.912 −48.982  1.00 37.19           C
ATOM   1613  C   VAL A 300      21.814 −27.133 −48.265  1.00 36.77           C
ATOM   1614  O   VAL A 300      22.018 −25.951 −48.526  1.00 36.52           O
ATOM   1615  CB  VAL A 300      19.407 −27.081 −49.113  1.00 37.21           C
ATOM   1616  CG1 VAL A 300      18.363 −27.818 −49.963  1.00 37.63           C
ATOM   1617  CG2 VAL A 300      18.773 −26.731 −47.760  1.00 37.10           C
ATOM   1618  HN  VAL A 300      20.024 −29.237 −47.446  1.00  0.00           H
ATOM   1619  HA  VAL A 300      21.055 −28.101 −49.995  1.00 37.13           H
ATOM   1620  HB  VAL A 300      19.656 −26.148 −49.619  1.00 37.13           H
ATOM   1621 1HG1 VAL A 300      17.461 −27.210 −50.039  1.00 37.13           H
ATOM   1622 2HG1 VAL A 300      18.119 −28.771 −49.494  1.00 37.13           H
ATOM   1623 3HG1 VAL A 300      18.766 −27.996 −50.960  1.00 37.13           H
ATOM   1624 1HG2 VAL A 300      17.867 −26.147 −47.923  1.00 37.13           H
ATOM   1625 2HG2 VAL A 300      19.479 −26.149 −47.167  1.00 37.13           H
ATOM   1626 3HG2 VAL A 300      18.522 −27.648 −47.228  1.00 37.13           H
ATOM   1627  N   GLY A 301      22.529 −27.778 −47.340  1.00 38.24           N
ATOM   1628  CA  GLY A 301      23.466 −27.114 −46.436  1.00 37.87           C
ATOM   1629  C   GLY A 301      22.743 −26.305 −45.353  1.00 37.71           C
```

ATOM 1630 O GLY A 301 21.701 −26.724 −44.860 1.00 37.88 O
ATOM 1631 HN GLY A 301 22.290 −28.738 −47.149 1.00 0.00 H
ATOM 1632 HA1 GLY A 301 24.087 −27.870 −45.955 1.00 37.92 H
ATOM 1633 HA2 GLY A 301 24.097 −26.440 −47.016 1.00 37.92 H
ATOM 1634 N ASP A 302 23.335 −25.183 −44.948 1.00 29.01 N
ATOM 1635 CA ASP A 302 22.908 −24.343 −43.820 1.00 28.85 C
ATOM 1636 C ASP A 302 21.398 −23.977 −43.869 1.00 28.88 C
ATOM 1637 O ASP A 302 20.968 −23.246 −44.769 1.00 28.76 O
ATOM 1638 CB ASP A 302 23.832 −23.116 −43.788 1.00 28.51 C
ATOM 1639 CG ASP A 302 23.573 −22.135 −42.638 1.00 28.35 C
ATOM 1640 OD1 ASP A 302 22.392 −21.792 −42.417 1.00 28.51 O
ATOM 1641 OD2 ASP A 302 24.564 −21.501 −42.228 1.00 28.09 O
ATOM 1642 HN ASP A 302 24.144 −24.864 −45.455 1.00 0.00 H
ATOM 1643 HA ASP A 302 23.080 −24.909 −42.904 1.00 28.62 H
ATOM 1644 HB1 ASP A 302 24.860 −23.470 −43.705 1.00 28.62 H
ATOM 1645 HB2 ASP A 302 23.708 −22.576 −44.727 1.00 28.62 H
ATOM 1646 N PRO A 303 20.582 −24.454 −42.904 1.00 29.07 N
ATOM 1647 CA PRO A 303 19.149 −24.176 −42.857 1.00 29.18 C
ATOM 1648 C PRO A 303 18.764 −22.710 −42.663 1.00 28.93 C
ATOM 1649 O PRO A 303 17.672 −22.324 −43.071 1.00 28.97 O
ATOM 1650 CB PRO A 303 18.629 −24.989 −41.674 1.00 29.41 C
ATOM 1651 CG PRO A 303 19.568 −26.185 −41.626 1.00 29.52 C
ATOM 1652 CD PRO A 303 20.900 −25.519 −41.955 1.00 29.21 C
ATOM 1653 HA PRO A 303 18.686 −24.546 −43.772 1.00 29.18 H
ATOM 1654 HB1 PRO A 303 18.685 −24.413 −40.751 1.00 29.18 H
ATOM 1655 HB2 PRO A 303 17.600 −25.308 −41.842 1.00 29.18 H
ATOM 1656 HG1 PRO A 303 19.583 −26.643 −40.637 1.00 29.18 H

ATOM 1657 HG2 PRO A 303 19.300 −26.935 −42.371 1.00 29.18 H

ATOM 1658 HD1 PRO A 303 21.351 −25.103 −41.054 1.00 29.18 H

ATOM 1659 HD2 PRO A 303 21.584 −26.238 −42.405 1.00 29.18 H

ATOM 1660 N ILE A 304 19.603 −21.880 −42.038 1.00 28.67 N

ATOM 1661 CA ILE A 304 19.321 −20.449 −41.842 1.00 28.45 C

ATOM 1662 C ILE A 304 19.552 −19.687 −43.149 1.00 28.38 C

ATOM 1663 O ILE A 304 18.659 −18.987 −43.634 1.00 28.33 O

ATOM 1664 CB ILE A 304 20.190 −19.891 −40.689 1.00 28.19 C

ATOM 1665 CG1 ILE A 304 19.950 −20.631 −39.354 1.00 28.25 C

ATOM 1666 CG2 ILE A 304 19.987 −18.375 −40.510 1.00 27.99 C

ATOM 1667 CD1 ILE A 304 18.536 −20.497 −38.768 1.00 28.30 C

ATOM 1668 HN ILE A 304 20.554 −22.197 −41.856 1.00 0.00 H

ATOM 1669 HA ILE A 304 18.273 −20.342 −41.564 1.00 28.32 H

ATOM 1670 HB ILE A 304 21.233 −20.048 −40.964 1.00 28.32 H

ATOM 1671 1HG1 ILE A 304 20.147 −21.691 −39.518 1.00 28.32 H

ATOM 1672 2HG1 ILE A 304 20.655 −20.239 −38.621 1.00 28.32 H

ATOM 1673 1HG2 ILE A 304 20.613 −18.018 −39.692 1.00 28.32 H

ATOM 1674 2HG2 ILE A 304 18.941 −18.173 −40.281 1.00 28.32 H

ATOM 1675 3HG2 ILE A 304 20.264 −17.860 −41.430 1.00 28.32 H

ATOM 1676 1HD1 ILE A 304 18.476 −21.053 −37.833 1.00 28.32 H

ATOM 1677 2HD1 ILE A 304 17.810 −20.897 −39.475 1.00 28.32 H

ATOM 1678 3HD1 ILE A 304 18.318 −19.446 −38.580 1.00 28.32 H

ATOM 1679 N LEU A 305 20.673 −19.970 −43.819 1.00 28.40 N

ATOM 1680 CA LEU A 305 21.036 −19.400 −45.118 1.00 28.32 C

ATOM 1681 C LEU A 305 20.267 −20.019 −46.303 1.00 28.53 C

ATOM 1682 O LEU A 305 20.334 −19.488 −47.414 1.00 28.48 O

ATOM 1683 CB LEU A 305 22.561 −19.507 −45.306 1.00 28.17 C

ATOM 1684 CG LEU A 305 23.409 −18.823 −44.211 1.00 27.94 C

ATOM 1685 CD1 LEU A 305 24.893 −19.021 −44.521 1.00 27.86 C

ATOM 1686 CD2 LEU A 305 23.146 −17.316 −44.122 1.00 27.76 C

ATOM 1687 HN LEU A 305 21.330 −20.614 −43.370 1.00 0.00 H

ATOM 1688 HA LEU A 305 20.787 −18.339 −45.088 1.00 28.18 H

ATOM 1689 HB1 LEU A 305 22.825 −20.564 −45.325 1.00 28.18 H

ATOM 1690 HB2 LEU A 305 22.815 −19.051 −46.263 1.00 28.18 H

ATOM 1691 HG LEU A 305 23.183 −19.281 −43.248 1.00 28.18 H

ATOM 1692 1HD1 LEU A 305 25.493 −18.539 −43.749 1.00 28.18 H

ATOM 1693 2HD1 LEU A 305 25.124 −18.579 −45.490 1.00 28.18 H

ATOM 1694 3HD1 LEU A 305 25.121 −20.087 −44.545 1.00 28.18 H

ATOM 1695 1HD2 LEU A 305 23.767 −16.884 −43.337 1.00 28.18 H

ATOM 1696 2HD2 LEU A 305 22.095 −17.143 −43.889 1.00 28.18 H

ATOM 1697 3HD2 LEU A 305 23.388 −16.847 −45.076 1.00 28.18 H

ATOM 1698 N ASN A 306 19.490 −21.088 −46.092 1.00 28.79 N

ATOM 1699 CA ASN A 306 18.670 −21.736 −47.125 1.00 29.02 C

ATOM 1700 C ASN A 306 17.295 −22.229 −46.621 1.00 29.27 C

ATOM 1701 O ASN A 306 16.687 −23.113 −47.223 1.00 29.56 O

ATOM 1702 CB ASN A 306 19.506 −22.812 −47.861 1.00 29.14 C

ATOM 1703 CG ASN A 306 19.685 −22.480 −49.331 1.00 29.16 C

ATOM 1704 OD1 ASN A 306 19.054 −23.053 −50.204 1.00 29.42 O

ATOM 1705 ND2 ASN A 306 20.260 −21.338 −49.629 1.00 28.91 N

ATOM 1706 HN ASN A 306 19.628 −21.581 −45.214 1.00 0.00 H

ATOM 1707 1HD2 ASN A 306 20.570 −20.723 −48.883 1.00 0.00 H

ATOM 1708 2HD2 ASN A 306 20.264 −21.069 −50.593 1.00 0.00 H

ATOM 1709 HA ASN A 306 18.458 −20.965 −47.865 1.00 29.16 H

ATOM 1710 HB1 ASN A 306 20.488 −22.877 −47.392 1.00 29.16 H

ATOM	1711	HB2	ASN	A	306	18.998	−23.772	−47.776	1.00	29.16	H
ATOM	1712	N	SER	A	307	16.679	−21.504	−45.680	1.00	37.31	N
ATOM	1713	CA	SER	A	307	15.384	−21.867	−45.067	1.00	37.51	C
ATOM	1714	C	SER	A	307	14.205	−22.026	−46.044	1.00	37.80	C
ATOM	1715	O	SER	A	307	13.201	−22.648	−45.695	1.00	38.09	O
ATOM	1716	CB	SER	A	307	15.010	−20.798	−44.036	1.00	37.32	C
ATOM	1717	OG	SER	A	307	13.794	−21.127	−43.399	1.00	37.52	O
ATOM	1718	HN	SER	A	307	17.258	−20.848	−45.167	1.00	0.00	H
ATOM	1719	HG	SER	A	307	13.181	−21.505	−44.043	1.00	0.00	H
ATOM	1720	HA	SER	A	307	15.514	−22.812	−44.540	1.00	37.59	H
ATOM	1721	HB1	SER	A	307	15.800	−20.730	−43.288	1.00	37.59	H
ATOM	1722	HB2	SER	A	307	14.902	−19.837	−44.539	1.00	37.59	H
ATOM	1723	N	THR	A	308	14.250	−21.373	−47.211	1.00	47.88	N
ATOM	1724	CA	THR	A	308	13.223	−21.512	−48.267	1.00	48.11	C
ATOM	1725	C	THR	A	308	13.362	−22.796	−49.094	1.00	48.41	C
ATOM	1726	O	THR	A	308	12.437	−23.143	−49.823	1.00	48.70	O
ATOM	1727	CB	THR	A	308	13.214	−20.278	−49.183	1.00	47.94	C
ATOM	1728	OG1	THR	A	308	12.090	−20.308	−50.026	1.00	48.12	O
ATOM	1729	CG2	THR	A	308	14.452	−20.149	−50.074	1.00	47.91	C
ATOM	1730	HN	THR	A	308	15.103	−20.884	−47.434	1.00	0.00	H
ATOM	1731	HG1	THR	A	308	11.995	−21.216	−50.354	1.00	0.00	H
ATOM	1732	HA	THR	A	308	12.253	−21.552	−47.770	1.00	48.15	H
ATOM	1733	HB	THR	A	308	13.149	−19.388	−48.557	1.00	48.15	H
ATOM	1734	1HG2	THR	A	308	14.366	−19.253	−50.688	1.00	48.15	H
ATOM	1735	2HG2	THR	A	308	14.529	−21.025	−50.718	1.00	48.15	H
ATOM	1736	3HG2	THR	A	308	15.343	−20.078	−49.450	1.00	48.15	H
ATOM	1737	N	SER	A	309	14.512	−23.465	−49.007	1.00	30.05	N

ATOM 1738 CA SER A 309 14.844 −24.720 −49.694 1.00 30.35 C
ATOM 1739 C SER A 309 14.860 −25.911 −48.725 1.00 30.55 C
ATOM 1740 O SER A 309 14.755 −27.060 −49.146 1.00 30.83 O
ATOM 1741 CB SER A 309 16.241 −24.607 −50.322 1.00 30.22 C
ATOM 1742 OG SER A 309 16.469 −23.358 −50.947 1.00 30.03 O
ATOM 1743 HN SER A 309 15.252 −23.065 −48.445 1.00 0.00 H
ATOM 1744 HG SER A 309 17.429 −23.190 −50.863 1.00 0.00 H
ATOM 1745 HA SER A 309 14.111 −24.905 −50.479 1.00 30.34 H
ATOM 1746 HB1 SER A 309 16.986 −24.744 −49.538 1.00 30.34 H
ATOM 1747 HB2 SER A 309 16.350 −25.394 −51.069 1.00 30.34 H
ATOM 1748 N TRP A 310 15.109 −25.654 −47.436 1.00 30.42 N
ATOM 1749 CA TRP A 310 15.312 −26.683 −46.416 1.00 30.56 C
ATOM 1750 C TRP A 310 14.010 −27.414 −46.048 1.00 30.94 C
ATOM 1751 O TRP A 310 13.085 −26.836 −45.478 1.00 30.94 O
ATOM 1752 CB TRP A 310 15.995 −26.054 −45.190 1.00 30.25 C
ATOM 1753 CG TRP A 310 16.273 −26.997 −44.058 1.00 30.32 C
ATOM 1754 CD1 TRP A 310 17.028 −28.116 −44.136 1.00 30.36 C
ATOM 1755 CD2 TRP A 310 15.765 −26.965 −42.687 1.00 30.34 C
ATOM 1756 NE1 TRP A 310 17.006 −28.780 −42.926 1.00 30.40 N
ATOM 1757 CE2 TRP A 310 16.231 −28.124 −41.998 1.00 30.38 C
ATOM 1758 CE3 TRP A 310 14.969 −26.065 −41.947 1.00 30.34 C
ATOM 1759 CZ2 TRP A 310 15.877 −28.414 −40.674 1.00 30.41 C
ATOM 1760 CZ3 TRP A 310 14.600 −26.343 −40.617 1.00 30.39 C
ATOM 1761 CH2 TRP A 310 15.039 −27.521 −39.985 1.00 30.42 C
ATOM 1762 HN TRP A 310 15.291 −24.696 −47.174 1.00 0.00 H
ATOM 1763 HE1 TRP A 310 17.381 −29.717 −42.787 1.00 0.00 H
ATOM 1764 HA TRP A 310 15.998 −27.423 −46.829 1.00 30.46 H

ATOM	1765	HB1	TRP	A	310	16.945	−25.629	−45.515	1.00	30.46	H
ATOM	1766	HB2	TRP	A	310	15.349	−25.259	−44.816	1.00	30.46	H
ATOM	1767	HD1	TRP	A	310	17.572	−28.444	−45.021	1.00	30.46	H
ATOM	1768	HE3	TRP	A	310	14.634	−25.138	−42.413	1.00	30.46	H
ATOM	1769	HZ2	TRP	A	310	16.245	−29.317	−40.187	1.00	30.46	H
ATOM	1770	HZ3	TRP	A	310	13.969	−25.640	−40.073	1.00	30.46	H
ATOM	1771	HH2	TRP	A	310	14.730	−27.740	−38.963	1.00	30.46	H
ATOM	1772	N	THR	A	311	13.988	−28.730	−46.259	1.00	63.68	N
ATOM	1773	CA	THR	A	311	13.044	−29.658	−45.620	1.00	64.05	C
ATOM	1774	C	THR	A	311	13.395	−29.802	−44.137	1.00	63.88	C
ATOM	1775	O	THR	A	311	14.513	−30.223	−43.848	1.00	63.62	O
ATOM	1776	CB	THR	A	311	13.144	−31.044	−46.283	1.00	64.38	C
ATOM	1777	OG1	THR	A	311	14.486	−31.487	−46.273	1.00	64.15	O
ATOM	1778	CG2	THR	A	311	12.672	−31.043	−47.736	1.00	64.60	C
ATOM	1779	HN	THR	A	311	14.766	−29.147	−46.749	1.00	0.00	H
ATOM	1780	HG1	THR	A	311	14.822	−31.301	−45.382	1.00	0.00	H
ATOM	1781	HA	THR	A	311	12.028	−29.275	−45.721	1.00	64.05	H
ATOM	1782	HB	THR	A	311	12.536	−31.750	−45.716	1.00	64.05	H
ATOM	1783	1HG2	THR	A	311	12.766	−32.047	−48.150	1.00	64.05	H
ATOM	1784	2HG2	THR	A	311	13.284	−30.352	−48.317	1.00	64.05	H
ATOM	1785	3HG2	THR	A	311	11.629	−30.729	−47.780	1.00	64.05	H
ATOM	1786	N	GLU	A	312	12.437	−29.637	−43.215	1.00	31.64	N
ATOM	1787	CA	GLU	A	312	12.686	−29.657	−41.754	1.00	31.46	C
ATOM	1788	C	GLU	A	312	12.916	−31.056	−41.137	1.00	31.70	C
ATOM	1789	O	GLU	A	312	12.334	−31.428	−40.120	1.00	31.93	O
ATOM	1790	CB	GLU	A	312	11.648	−28.810	−40.996	1.00	31.48	C
ATOM	1791	CG	GLU	A	312	10.209	−29.360	−40.955	1.00	31.91	C

ATOM	1792	CD	GLU	A	312	9.523	−29.123	−39.596	1.00	31.86	C
ATOM	1793	OE1	GLU	A	312	8.442	−29.734	−39.427	1.00	32.21	O
ATOM	1794	OE2	GLU	A	312	9.649	−27.990	−39.087	1.00	31.49	O
ATOM	1795	HN	GLU	A	312	11.525	−29.334	−43.514	1.00	0.00	H
ATOM	1796	HA	GLU	A	312	13.631	−29.131	−41.621	1.00	31.74	H
ATOM	1797	HB1	GLU	A	312	11.992	−28.707	−39.967	1.00	31.74	H
ATOM	1798	HB2	GLU	A	312	11.614	−27.828	−41.468	1.00	31.74	H
ATOM	1799	HG1	GLU	A	312	9.625	−28.867	−41.732	1.00	31.74	H
ATOM	1800	HG2	GLU	A	312	10.240	−30.432	−41.148	1.00	31.74	H
ATOM	1801	N	SER	A	313	13.772	−31.859	−41.768	1.00	31.66	N
ATOM	1802	CA	SER	A	313	14.220	−33.147	−41.247	1.00	31.95	C
ATOM	1803	C	SER	A	313	15.060	−32.943	−39.983	1.00	31.68	C
ATOM	1804	O	SER	A	313	16.156	−32.379	−40.026	1.00	31.28	O
ATOM	1805	CB	SER	A	313	15.004	−33.891	−42.327	1.00	32.07	C
ATOM	1806	OG	SER	A	313	15.368	−35.179	−41.871	1.00	32.36	O
ATOM	1807	HN	SER	A	313	14.290	−31.450	−42.536	1.00	0.00	H
ATOM	1808	HG	SER	A	313	15.892	−35.609	−42.551	1.00	0.00	H
ATOM	1809	HA	SER	A	313	13.342	−33.739	−40.987	1.00	31.83	H
ATOM	1810	HB1	SER	A	313	14.384	−33.987	−43.219	1.00	31.83	H
ATOM	1811	HB2	SER	A	313	15.905	−33.328	−42.569	1.00	31.83	H
ATOM	1812	N	LEU	A	314	14.518	−33.353	−38.838	1.00	31.91	N
ATOM	1813	CA	LEU	A	314	15.201	−33.325	−37.547	1.00	31.66	C
ATOM	1814	C	LEU	A	314	15.597	−34.742	−37.129	1.00	31.94	C
ATOM	1815	O	LEU	A	314	14.848	−35.702	−37.334	1.00	32.37	O
ATOM	1816	CB	LEU	A	314	14.324	−32.622	−36.493	1.00	31.53	C
ATOM	1817	CG	LEU	A	314	13.955	−31.159	−36.822	1.00	31.20	C
ATOM	1818	CD1	LEU	A	314	13.219	−30.532	−35.636	1.00	31.20	C

ATOM 1819 CD2 LEU A 314 15.189 −30.303 −37.113 1.00 30.76 C
ATOM 1820 HN LEU A 314 13.597 −33.764 −38.874 1.00 0.00 H
ATOM 1821 HA LEU A 314 16.115 −32.743 −37.664 1.00 31.57 H
ATOM 1822 HB1 LEU A 314 13.399 −33.189 −36.390 1.00 31.57 H
ATOM 1823 HB2 LEU A 314 14.863 −32.630 −35.545 1.00 31.57 H
ATOM 1824 HG LEU A 314 13.300 −31.146 −37.693 1.00 31.57 H
ATOM 1825 1HD1 LEU A 314 12.961 −29.500 −35.873 1.00 31.57 H
ATOM 1826 2HD1 LEU A 314 13.863 −30.552 −34.756 1.00 31.57 H
ATOM 1827 3HD1 LEU A 314 12.309 −31.096 −35.433 1.00 31.57 H
ATOM 1828 1HD2 LEU A 314 14.879 −29.283 −37.339 1.00 31.57 H
ATOM 1829 2HD2 LEU A 314 15.726 −30.717 −37.967 1.00 31.57 H
ATOM 1830 3HD2 LEU A 314 15.843 −30.299 −36.241 1.00 31.57 H
ATOM 1831 N SER A 315 16.735 −34.866 −36.448 1.00 31.68 N
ATOM 1832 CA SER A 315 17.180 −36.144 −35.890 1.00 31.89 C
ATOM 1833 C SER A 315 17.784 −35.980 −34.504 1.00 31.54 C
ATOM 1834 O SER A 315 18.568 −35.062 −34.265 1.00 31.11 O
ATOM 1835 CB SER A 315 18.134 −36.856 −36.846 1.00 32.00 C
ATOM 1836 OG SER A 315 19.362 −36.162 −36.999 1.00 31.65 O
ATOM 1837 HN SER A 315 17.292 −34.038 −36.256 1.00 0.00 H
ATOM 1838 HG SER A 315 19.170 −35.217 −36.925 1.00 0.00 H
ATOM 1839 HA SER A 315 16.298 −36.775 −35.785 1.00 31.64 H
ATOM 1840 HB1 SER A 315 18.341 −37.853 −36.456 1.00 31.65 H
ATOM 1841 HB2 SER A 315 17.655 −36.939 −37.821 1.00 31.65 H
ATOM 1842 N LEU A 316 17.384 −36.851 −33.583 1.00 31.74 N
ATOM 1843 CA LEU A 316 17.939 −36.928 −32.240 1.00 31.44 C
ATOM 1844 C LEU A 316 18.898 −38.114 −32.151 1.00 31.58 C
ATOM 1845 O LEU A 316 18.556 −39.240 −32.513 1.00 32.03 O

```
ATOM   1846  CB  LEU A 316      16.792 −36.954 −31.214  1.00 31.53           C
ATOM   1847  CG  LEU A 316      17.273 −36.978 −29.750  1.00 31.25           C
ATOM   1848  CD1 LEU A 316      16.301 −36.196 −28.865  1.00 31.16           C
ATOM   1849  CD2 LEU A 316      17.364 −38.403 −29.197  1.00 31.53           C
ATOM   1850  HN  LEU A 316      16.810 −37.629 −33.893  1.00  0.00           H
ATOM   1851  HA  LEU A 316      18.517 −36.020 −32.068  1.00 31.53           H
ATOM   1852  HB1 LEU A 316      16.179 −36.065 −31.360  1.00 31.53           H
ATOM   1853  HB2 LEU A 316      16.190 −37.845 −31.393  1.00 31.53           H
ATOM   1854  HG  LEU A 316      18.257 −36.512 −29.693  1.00 31.53           H
ATOM   1855 1HD1 LEU A 316      16.650 −36.219 −27.833  1.00 31.53           H
ATOM   1856 2HD1 LEU A 316      15.311 −36.648 −28.924  1.00 31.53           H
ATOM   1857 3HD1 LEU A 316      16.248 −35.162 −29.207  1.00 31.53           H
ATOM   1858 1HD2 LEU A 316      17.707 −38.371 −28.163  1.00 31.53           H
ATOM   1859 2HD2 LEU A 316      18.069 −38.981 −29.795  1.00 31.53           H
ATOM   1860 3HD2 LEU A 316      16.381 −38.873 −29.239  1.00 31.53           H
ATOM   1861  N   ILE A 317      20.070 −37.872 −31.582  1.00 31.20           N
ATOM   1862  CA  ILE A 317      20.992 −38.898 −31.109  1.00 31.26           C
ATOM   1863  C   ILE A 317      20.883 −38.945 −29.583  1.00 31.02           C
ATOM   1864  O   ILE A 317      20.764 −37.904 −28.939  1.00 30.58           O
ATOM   1865  CB  ILE A 317      22.433 −38.618 −31.601  1.00 31.04           C
ATOM   1866  CG1 ILE A 317      22.492 −38.330 −33.122  1.00 31.28           C
ATOM   1867  CG2 ILE A 317      23.350 −39.814 −31.288  1.00 31.13           C
ATOM   1868  CD1 ILE A 317      22.469 −36.836 −33.474  1.00 31.03           C
ATOM   1869  HN  ILE A 317      20.258 −36.920 −31.278  1.00  0.00           H
ATOM   1870  HA  ILE A 317      20.672 −39.861 −31.508  1.00 31.07           H
ATOM   1871  HB  ILE A 317      22.814 −37.745 −31.071  1.00 31.07           H
ATOM   1872 1HG1 ILE A 317      23.412 −38.761 −33.517  1.00 31.07           H
```

ATOM 1873 2HG1 ILE A 317 21.634 −38.807 −33.595 1.00 31.07 H

ATOM 1874 1HG2 ILE A 317 24.359 −39.601 −31.640 1.00 31.07 H

ATOM 1875 2HG2 ILE A 317 22.971 −40.704 −31.790 1.00 31.07 H

ATOM 1876 3HG2 ILE A 317 23.370 −39.985 −30.212 1.00 31.07 H

ATOM 1877 1HD1 ILE A 317 22.513 −36.717 −34.556 1.00 31.07 H

ATOM 1878 2HD1 ILE A 317 23.327 −36.342 −33.019 1.00 31.07 H

ATOM 1879 3HD1 ILE A 317 21.550 −36.387 −33.097 1.00 31.07 H

ATOM 1880 N ARG A 318 20.995 −40.126 −28.977 1.00 31.34 N

ATOM 1881 CA ARG A 318 21.327 −40.251 −27.550 1.00 31.16 C

ATOM 1882 C ARG A 318 22.633 −41.012 −27.393 1.00 31.10 C

ATOM 1883 O ARG A 318 22.848 −42.006 −28.089 1.00 31.47 O

ATOM 1884 CB ARG A 318 20.135 −40.799 −26.742 1.00 31.56 C

ATOM 1885 CG ARG A 318 19.896 −42.318 −26.838 1.00 32.13 C

ATOM 1886 CD ARG A 318 20.722 −43.107 −25.809 1.00 32.27 C

ATOM 1887 NE ARG A 318 20.310 −44.519 −25.708 1.00 32.85 N

ATOM 1888 CZ ARG A 318 19.320 −44.995 −24.975 1.00 33.29 C

ATOM 1889 NH1 ARG A 318 18.534 −44.231 −24.269 1.00 33.24 N

ATOM 1890 NH2 ARG A 318 19.083 −46.273 −24.972 1.00 33.82 N

ATOM 1891 HN ARG A 318 21.082 −40.947 −29.570 1.00 0.00 H

ATOM 1892 HE ARG A 318 20.950 −45.211 −26.078 1.00 0.00 H

ATOM 1893 1HH1 ARG A 318 18.831 −43.284 −24.106 1.00 0.00 H

ATOM 1894 2HH1 ARG A 318 17.925 −44.634 −23.582 1.00 0.00 H

ATOM 1895 1HH2 ARG A 318 19.667 −46.870 −25.556 1.00 0.00 H

ATOM 1896 2HH2 ARG A 318 18.344 −46.657 −24.418 1.00 0.00 H

ATOM 1897 HA ARG A 318 21.511 −39.241 −27.185 1.00 32.20 H

ATOM 1898 HB1 ARG A 318 20.302 −40.553 −25.693 1.00 32.20 H

ATOM 1899 HB2 ARG A 318 19.234 −40.297 −27.094 1.00 32.20 H

ATOM 1900 HG1 ARG A 318 18.838 −42.518 −26.665 1.00 32.20 H
ATOM 1901 HG2 ARG A 318 20.171 −42.653 −27.838 1.00 32.20 H
ATOM 1902 HD1 ARG A 318 21.771 −43.071 −26.103 1.00 32.20 H
ATOM 1903 HD2 ARG A 318 20.601 −42.638 −24.832 1.00 32.20 H
ATOM 1904 N LEU A 319 23.464 −40.576 −26.455 1.00 30.64 N
ATOM 1905 CA LEU A 319 24.765 −41.166 −26.144 1.00 30.54 C
ATOM 1906 C LEU A 319 24.841 −41.404 −24.632 1.00 30.35 C
ATOM 1907 O LEU A 319 24.603 −40.485 −23.849 1.00 29.99 O
ATOM 1908 CB LEU A 319 25.889 −40.227 −26.632 1.00 30.17 C
ATOM 1909 CG LEU A 319 25.898 −39.897 −28.139 1.00 30.26 C
ATOM 1910 CD1 LEU A 319 26.962 −38.844 −28.449 1.00 29.80 C
ATOM 1911 CD2 LEU A 319 26.195 −41.124 −28.999 1.00 30.72 C
ATOM 1912 HN LEU A 319 23.208 −39.735 −25.942 1.00 0.00 H
ATOM 1913 HA LEU A 319 24.853 −42.123 −26.659 1.00 30.31 H
ATOM 1914 HB1 LEU A 319 25.797 −39.288 −26.087 1.00 30.31 H
ATOM 1915 HB2 LEU A 319 26.842 −40.696 −26.389 1.00 30.31 H
ATOM 1916 HG LEU A 319 24.921 −39.501 −28.417 1.00 30.31 H
ATOM 1917 1HD1 LEU A 319 26.955 −38.623 −29.516 1.00 30.31 H
ATOM 1918 2HD1 LEU A 319 27.943 −39.223 −28.164 1.00 30.31 H
ATOM 1919 3HD1 LEU A 319 26.748 −37.934 −27.888 1.00 30.31 H
ATOM 1920 1HD2 LEU A 319 26.190 −40.840 −30.051 1.00 30.31 H
ATOM 1921 2HD2 LEU A 319 25.433 −41.884 −28.825 1.00 30.31 H
ATOM 1922 3HD2 LEU A 319 27.174 −41.525 −28.736 1.00 30.31 H
ATOM 1923 N ALA A 320 25.099 −42.644 −24.213 1.00 30.61 N
ATOM 1924 CA ALA A 320 25.290 −42.964 −22.800 1.00 30.44 C
ATOM 1925 C ALA A 320 26.560 −42.285 −22.263 1.00 29.90 C
ATOM 1926 O ALA A 320 27.632 −42.450 −22.847 1.00 29.81 O

```
ATOM   1927  CB  ALA A 320      25.332 −44.484 −22.612  1.00 30.84           C
ATOM   1928  HN  ALA A 320      25.276 −43.367 −24.898  1.00  0.00           H
ATOM   1929  HA  ALA A 320      24.436 −42.576 −22.245  1.00 30.32           H
ATOM   1930  HB1 ALA A 320      25.475 −44.716 −21.557  1.00 30.32           H
ATOM   1931  HB2 ALA A 320      26.158 −44.899 −23.190  1.00 30.32           H
ATOM   1932  HB3 ALA A 320      24.394 −44.919 −22.956  1.00 30.32           H
ATOM   1933  N   VAL A 321      26.457 −41.558 −21.150  1.00 55.87           N
ATOM   1934  CA  VAL A 321      27.574 −40.804 −20.543  1.00 55.36           C
ATOM   1935  C   VAL A 321      28.640 −41.714 −19.925  1.00 55.36           C
ATOM   1936  O   VAL A 321      29.812 −41.350 −19.889  1.00 55.04           O
ATOM   1937  CB  VAL A 321      27.066 −39.794 −19.493  1.00 54.95           C
ATOM   1938  CG1 VAL A 321      26.022 −38.846 −20.097  1.00 54.90           C
ATOM   1939  CG2 VAL A 321      26.477 −40.467 −18.244  1.00 55.05           C
ATOM   1940  HN  VAL A 321      25.541 −41.496 −20.710  1.00  0.00           H
ATOM   1941  HA  VAL A 321      28.053 −40.234 −21.339  1.00 55.22           H
ATOM   1942  HB  VAL A 321      27.916 −39.191 −19.175  1.00 55.22           H
ATOM   1943 1HG1 VAL A 321      25.682 −38.146 −19.334  1.00 55.22           H
ATOM   1944 2HG1 VAL A 321      25.173 −39.424 −20.462  1.00 55.22           H
ATOM   1945 3HG1 VAL A 321      26.468 −38.293 −20.924  1.00 55.22           H
ATOM   1946 1HG2 VAL A 321      26.138 −39.703 −17.545  1.00 55.22           H
ATOM   1947 2HG2 VAL A 321      27.241 −41.082 −17.768  1.00 55.22           H
ATOM   1948 3HG2 VAL A 321      25.634 −41.095 −18.532  1.00 55.22           H
ATOM   1949  N   ARG A 322      28.264 −42.956 −19.596  1.00 37.18           N
ATOM   1950  CA  ARG A 322      29.091 −43.948 −18.894  1.00 37.18           C
ATOM   1951  C   ARG A 322      28.913 −45.340 −19.536  1.00 37.74           C
ATOM   1952  O   ARG A 322      28.294 −46.225 −18.950  1.00 38.00           O
ATOM   1953  CB  ARG A 322      28.709 −43.858 −17.410  1.00 37.05           C
```

ATOM 1954 CG ARG A 322 29.694 −44.531 −16.445 1.00 36.97 C

ATOM 1955 CD ARG A 322 29.337 −44.217 −14.981 1.00 36.47 C

ATOM 1956 NE ARG A 322 28.027 −44.784 −14.591 1.00 36.59 N

ATOM 1957 CZ ARG A 322 26.842 −44.204 −14.700 1.00 36.64 C

ATOM 1958 NH1 ARG A 322 26.672 −42.964 −15.050 1.00 36.58 N

ATOM 1959 NH2 ARG A 322 25.764 −44.904 −14.536 1.00 36.77 N

ATOM 1960 HN ARG A 322 27.278 −43.156 −19.682 1.00 0.00 H

ATOM 1961 HE ARG A 322 28.020 −45.745 −14.308 1.00 0.00 H

ATOM 1962 1HH1 ARG A 322 27.463 −42.387 −15.274 1.00 0.00 H

ATOM 1963 2HH1 ARG A 322 25.712 −42.687 −15.276 1.00 0.00 H

ATOM 1964 1HH2 ARG A 322 25.793 −45.899 −14.511 1.00 0.00 H

ATOM 1965 2HH2 ARG A 322 24.901 −44.498 −14.924 1.00 0.00 H

ATOM 1966 HA ARG A 322 30.136 −43.656 −18.996 1.00 37.02 H

ATOM 1967 HB1 ARG A 322 28.640 −42.804 −17.142 1.00 37.02 H

ATOM 1968 HB2 ARG A 322 27.735 −44.330 −17.282 1.00 37.02 H

ATOM 1969 HG1 ARG A 322 29.659 −45.610 −16.596 1.00 37.02 H

ATOM 1970 HG2 ARG A 322 30.700 −44.167 −16.652 1.00 37.02 H

ATOM 1971 HD1 ARG A 322 30.108 −44.637 −14.334 1.00 37.02 H

ATOM 1972 HD2 ARG A 322 29.303 −43.135 −14.852 1.00 37.02 H

ATOM 1973 N PRO A 323 29.234 −45.477 −20.837 1.00 30.14 N

ATOM 1974 CA PRO A 323 28.738 −46.534 −21.722 1.00 30.74 C

ATOM 1975 C PRO A 323 29.149 −47.934 −21.237 1.00 31.12 C

ATOM 1976 O PRO A 323 29.228 −48.878 −22.028 1.00 31.05 O

ATOM 1977 CB PRO A 323 29.303 −46.174 −23.105 1.00 30.74 C

ATOM 1978 CG PRO A 323 30.610 −45.469 −22.760 1.00 30.17 C

ATOM 1979 CD PRO A 323 30.194 −44.646 −21.547 1.00 29.79 C

ATOM 1980 HA PRO A 323 27.650 −46.480 −21.761 1.00 30.54 H

```
ATOM   1981  HC  PRO A 323      29.373 −48.095 −20.182  1.00 30.54           H
ATOM   1982  HB1 PRO A 323      29.485 −47.069 −23.700  1.00 30.54           H
ATOM   1983  HB2 PRO A 323      28.628 −45.509 −23.644  1.00 30.54           H
ATOM   1984  HG1 PRO A 323      31.394 −46.183 −22.508  1.00 30.54           H
ATOM   1985  HG2 PRO A 323      30.947 −44.832 −23.578  1.00 30.54           H
ATOM   1986  HD1 PRO A 323      31.056 −44.433 −20.915  1.00 30.54           H
ATOM   1987  HD2 PRO A 323      29.732 −43.710 −21.860  1.00 30.54           H
ATOM   1988  N   ASP A 329      22.436 −50.639 −28.123  1.00 34.57           N
ATOM   1989  CA  ASP A 329      21.606 −49.435 −28.176  1.00 34.22           C
ATOM   1990  C   ASP A 329      21.352 −48.781 −26.805  1.00 33.80           C
ATOM   1991  O   ASP A 329      20.647 −47.770 −26.711  1.00 33.55           O
ATOM   1992  CB  ASP A 329      20.295 −49.716 −28.922  1.00 34.68           C
ATOM   1993  CG  ASP A 329      20.503 −49.898 −30.427  1.00 34.80           C
ATOM   1994  OD1 ASP A 329      21.168 −50.880 −30.804  1.00 34.33           O
ATOM   1995  OD2 ASP A 329      19.705 −49.262 −31.160  1.00 35.36           O
ATOM   1996  HN1 ASP A 329      21.960 −51.374 −27.509  1.00  0.00           H
ATOM   1997  HN2 ASP A 329      23.393 −50.395 −27.710  1.00 34.41           H
ATOM   1998  HA  ASP A 329      22.154 −48.705 −28.773  1.00 34.41           H
ATOM   1999  HB1 ASP A 329      19.852 −50.626 −28.518  1.00 34.41           H
ATOM   2000  HB2 ASP A 329      19.616 −48.879 −28.762  1.00 34.41           H
ATOM   2001  N   TYR A 330      21.992 −49.276 −25.740  1.00 33.72           N
ATOM   2002  CA  TYR A 330      22.263 −48.463 −24.546  1.00 33.20           C
ATOM   2003  C   TYR A 330      23.263 −47.349 −24.886  1.00 32.60           C
ATOM   2004  O   TYR A 330      22.895 −46.175 −24.919  1.00 32.21           O
ATOM   2005  CB  TYR A 330      22.771 −49.346 −23.390  1.00 33.19           C
ATOM   2006  CG  TYR A 330      23.259 −48.568 −22.172  1.00 32.61           C
ATOM   2007  CD1 TYR A 330      22.338 −47.885 −21.351  1.00 32.26           C
```

ATOM 2008 CD2 TYR A 330 24.634 −48.509 −21.866 1.00 32.45 C

ATOM 2009 CE1 TYR A 330 22.785 −47.122 −20.250 1.00 31.77 C

ATOM 2010 CE2 TYR A 330 25.080 −47.761 −20.757 1.00 31.93 C

ATOM 2011 CZ TYR A 330 24.162 −47.060 −19.947 1.00 31.61 C

ATOM 2012 OH TYR A 330 24.632 −46.334 −18.897 1.00 31.12 O

ATOM 2013 HN TYR A 330 22.543 −50.109 −25.900 1.00 0.00 H

ATOM 2014 HH TYR A 330 23.968 −46.062 −18.248 1.00 0.00 H

ATOM 2015 HA TYR A 330 21.329 −47.997 −24.233 1.00 32.39 H

ATOM 2016 HB1 TYR A 330 21.956 −49.997 −23.075 1.00 32.39 H

ATOM 2017 HB2 TYR A 330 23.598 −49.952 −23.761 1.00 32.39 H

ATOM 2018 HD1 TYR A 330 21.272 −47.947 −21.568 1.00 32.39 H

ATOM 2019 HD2 TYR A 330 25.353 −49.042 −22.488 1.00 32.39 H

ATOM 2020 HE1 TYR A 330 22.067 −46.581 −19.634 1.00 32.39 H

ATOM 2021 HE2 TYR A 330 26.144 −47.724 −20.523 1.00 32.39 H

ATOM 2022 N ASN A 331 24.436 −47.752 −25.383 1.00 32.56 N

ATOM 2023 CA ASN A 331 25.605 −46.900 −25.601 1.00 32.02 C

ATOM 2024 C ASN A 331 25.331 −45.725 −26.551 1.00 31.95 C

ATOM 2025 O ASN A 331 25.723 −44.597 −26.258 1.00 31.51 O

ATOM 2026 CB ASN A 331 26.769 −47.770 −26.130 1.00 32.05 C

ATOM 2027 CG ASN A 331 27.141 −48.938 −25.224 1.00 32.09 C

ATOM 2028 OD1 ASN A 331 26.299 −49.681 −24.751 1.00 31.93 O

ATOM 2029 ND2 ASN A 331 28.402 −49.104 −24.911 1.00 32.30 N

ATOM 2030 HN ASN A 331 24.631 −48.746 −25.324 1.00 0.00 H

ATOM 2031 1HD2 ASN A 331 29.108 −48.481 −25.252 1.00 0.00 H

ATOM 2032 2HD2 ASN A 331 28.594 −49.729 −24.140 1.00 0.00 H

ATOM 2033 HA ASN A 331 25.905 −46.489 −24.637 1.00 32.05 H

ATOM 2034 HB1 ASN A 331 26.481 −48.170 −27.102 1.00 32.05 H

ATOM 2035 HB2 ASN A 331 27.646 −47.133 −26.244 1.00 32.05 H
ATOM 2036 N GLN A 332 24.650 −45.976 −27.671 1.00 32.39 N
ATOM 2037 CA GLN A 332 24.299 −44.978 −28.685 1.00 32.30 C
ATOM 2038 C GLN A 332 22.949 −45.341 −29.316 1.00 32.80 C
ATOM 2039 O GLN A 332 22.684 −46.521 −29.535 1.00 33.32 O
ATOM 2040 CB GLN A 332 25.379 −44.901 −29.786 1.00 32.24 C
ATOM 2041 CG GLN A 332 26.811 −44.674 −29.270 1.00 31.71 C
ATOM 2042 CD GLN A 332 27.807 −44.297 −30.363 1.00 31.54 C
ATOM 2043 OE1 GLN A 332 27.565 −44.419 −31.553 1.00 31.87 O
ATOM 2044 NE2 GLN A 332 28.975 −43.841 −29.977 1.00 31.04 N
ATOM 2045 HN GLN A 332 24.312 −46.915 −27.826 1.00 0.00 H
ATOM 2046 1HE2 GLN A 332 29.169 −43.717 −28.988 1.00 0.00 H
ATOM 2047 2HE2 GLN A 332 29.648 −43.549 −30.674 1.00 0.00 H
ATOM 2048 HA GLN A 332 24.214 −44.003 −28.206 1.00 32.13 H
ATOM 2049 HB1 GLN A 332 25.364 −45.838 −30.342 1.00 32.13 H
ATOM 2050 HB2 GLN A 332 25.125 −44.078 −30.453 1.00 32.13 H
ATOM 2051 HG1 GLN A 332 26.788 −43.870 −28.534 1.00 32.13 H
ATOM 2052 HG2 GLN A 332 27.154 −45.593 −28.794 1.00 32.13 H
ATOM 2053 N LYS A 333 22.153 −44.351 −29.735 1.00 32.66 N
ATOM 2054 CA LYS A 333 21.011 −44.566 −30.644 1.00 33.07 C
ATOM 2055 C LYS A 333 20.738 −43.320 −31.483 1.00 32.84 C
ATOM 2056 O LYS A 333 20.755 −42.214 −30.951 1.00 32.33 O
ATOM 2057 CB LYS A 333 19.764 −45.008 −29.853 1.00 33.24 C
ATOM 2058 CG LYS A 333 18.697 −45.631 −30.764 1.00 33.61 C
ATOM 2059 CD LYS A 333 17.467 −46.065 −29.953 1.00 33.92 C
ATOM 2060 CE LYS A 333 16.652 −47.132 −30.697 1.00 34.58 C
ATOM 2061 NZ LYS A 333 17.302 −48.463 −30.637 1.00 34.67 N1+

ATOM 2062 HN LYS A 333 22.411 −43.396 −29.493 1.00 0.00 H

ATOM 2063 HZ1 LYS A 333 16.801 −49.177 −31.142 1.00 0.00 H

ATOM 2064 HZ2 LYS A 333 17.484 −48.775 −29.692 1.00 0.00 H

ATOM 2065 HZ3 LYS A 333 18.233 −48.447 −31.072 1.00 0.00 H

ATOM 2066 HA LYS A 333 21.276 −45.374 −31.326 1.00 33.44 H

ATOM 2067 HB1 LYS A 333 20.063 −45.744 −29.107 1.00 33.44 H

ATOM 2068 HB2 LYS A 333 19.337 −44.138 −29.355 1.00 33.44 H

ATOM 2069 HG1 LYS A 333 18.392 −44.896 −31.509 1.00 33.44 H

ATOM 2070 HG2 LYS A 333 19.120 −46.502 −31.264 1.00 33.44 H

ATOM 2071 HD1 LYS A 333 17.798 −46.474 −28.998 1.00 33.44 H

ATOM 2072 HD2 LYS A 333 16.834 −45.195 −29.776 1.00 33.44 H

ATOM 2073 HE1 LYS A 333 15.664 −47.201 −30.242 1.00 33.44 H

ATOM 2074 HE2 LYS A 333 16.552 −46.835 −31.741 1.00 33.44 H

ATOM 2075 N TYR A 334 20.411 −43.515 −32.759 1.00 33.24 N

ATOM 2076 CA TYR A 334 19.962 −42.473 −33.691 1.00 33.11 C

ATOM 2077 C TYR A 334 18.449 −42.581 −33.944 1.00 33.44 C

ATOM 2078 O TYR A 334 17.930 −43.687 −34.101 1.00 33.93 O

ATOM 2079 CB TYR A 334 20.763 −42.611 −34.996 1.00 33.28 C

ATOM 2080 CG TYR A 334 20.545 −41.494 −35.999 1.00 32.90 C

ATOM 2081 CD1 TYR A 334 19.536 −41.593 −36.979 1.00 33.04 C

ATOM 2082 CD2 TYR A 334 21.367 −40.353 −35.954 1.00 32.40 C

ATOM 2083 CE1 TYR A 334 19.352 −40.550 −37.910 1.00 32.68 C

ATOM 2084 CE2 TYR A 334 21.163 −39.294 −36.858 1.00 32.06 C

ATOM 2085 CZ TYR A 334 20.167 −39.400 −37.850 1.00 32.19 C

ATOM 2086 OH TYR A 334 19.969 −38.378 −38.722 1.00 31.85 O

ATOM 2087 HN TYR A 334 20.363 −44.464 −33.091 1.00 0.00 H

ATOM 2088 HH TYR A 334 20.019 −37.527 −38.262 1.00 0.00 H

ATOM 2089 HA TYR A 334 20.174 −41.498 −33.253 1.00 32.84 H

ATOM 2090 HB1 TYR A 334 21.823 −42.636 −34.742 1.00 32.84 H

ATOM 2091 HB2 TYR A 334 20.480 −43.551 −35.469 1.00 32.84 H

ATOM 2092 HD1 TYR A 334 18.898 −42.476 −37.017 1.00 32.84 H

ATOM 2093 HD2 TYR A 334 22.166 −40.289 −35.215 1.00 32.84 H

ATOM 2094 HE1 TYR A 334 18.581 −40.632 −38.676 1.00 32.84 H

ATOM 2095 HE2 TYR A 334 21.774 −38.394 −36.791 1.00 32.84 H

ATOM 2096 N ILE A 335 17.752 −41.448 −34.044 1.00 33.18 N

ATOM 2097 CA ILE A 335 16.310 −41.345 −34.314 1.00 33.48 C

ATOM 2098 C ILE A 335 16.084 −40.233 −35.345 1.00 33.30 C

ATOM 2099 O ILE A 335 16.319 −39.064 −35.050 1.00 32.82 O

ATOM 2100 CB ILE A 335 15.519 −41.071 −33.004 1.00 33.41 C

ATOM 2101 CG1 ILE A 335 15.700 −42.218 −31.980 1.00 33.82 C

ATOM 2102 CG2 ILE A 335 14.023 −40.839 −33.304 1.00 33.54 C

ATOM 2103 CD1 ILE A 335 15.024 −41.975 −30.623 1.00 33.89 C

ATOM 2104 HN ILE A 335 18.227 −40.578 −33.815 1.00 0.00 H

ATOM 2105 HA ILE A 335 15.966 −42.289 −34.738 1.00 33.43 H

ATOM 2106 HB ILE A 335 15.916 −40.159 −32.558 1.00 33.43 H

ATOM 2107 1HG1 ILE A 335 15.281 −43.127 −32.411 1.00 33.43 H

ATOM 2108 2HG1 ILE A 335 16.768 −42.355 −31.808 1.00 33.43 H

ATOM 2109 1HG2 ILE A 335 13.490 −40.649 −32.372 1.00 33.43 H

ATOM 2110 2HG2 ILE A 335 13.607 −41.724 −33.786 1.00 33.43 H

ATOM 2111 3HG2 ILE A 335 13.914 −39.980 −33.966 1.00 33.43 H

ATOM 2112 1HD1 ILE A 335 15.203 −42.829 −29.969 1.00 33.43 H

ATOM 2113 2HD1 ILE A 335 13.951 −41.848 −30.769 1.00 33.43 H

ATOM 2114 3HD1 ILE A 335 15.437 −41.076 −30.166 1.00 33.43 H

ATOM 2115 N ALA A 336 15.507 −40.563 −36.503 1.00 48.91 N

ATOM	2116	CA	ALA	A	336	14.855	−39.574	−37.365	1.00	48.81	C
ATOM	2117	C	ALA	A	336	13.444	−39.276	−36.825	1.00	48.88	C
ATOM	2118	O	ALA	A	336	12.671	−40.204	−36.576	1.00	49.36	O
ATOM	2119	CB	ALA	A	336	14.820	−40.108	−38.801	1.00	49.23	C
ATOM	2120	HN	ALA	A	336	15.260	−41.528	−36.650	1.00	0.00	H
ATOM	2121	HA	ALA	A	336	15.439	−38.654	−37.347	1.00	49.04	H
ATOM	2122	HB1	ALA	A	336	14.336	−39.377	−39.449	1.00	49.04	H
ATOM	2123	HB2	ALA	A	336	14.261	−41.043	−38.828	1.00	49.04	H
ATOM	2124	HB3	ALA	A	336	15.838	−40.284	−39.149	1.00	49.04	H
ATOM	2125	N	ILE	A	337	13.132	−38.007	−36.555	1.00	33.24	N
ATOM	2126	CA	ILE	A	337	11.854	−37.619	−35.943	1.00	33.27	C
ATOM	2127	C	ILE	A	337	10.745	−37.584	−36.999	1.00	33.57	C
ATOM	2128	O	ILE	A	337	10.852	−36.909	−38.017	1.00	33.45	O
ATOM	2129	CB	ILE	A	337	12.017	−36.291	−35.177	1.00	32.73	C
ATOM	2130	CG1	ILE	A	337	12.875	−36.522	−33.908	1.00	32.41	C
ATOM	2131	CG2	ILE	A	337	10.665	−35.690	−34.761	1.00	32.81	C
ATOM	2132	CD1	ILE	A	337	13.977	−35.475	−33.772	1.00	31.85	C
ATOM	2133	HN	ILE	A	337	13.774	−37.267	−36.827	1.00	0.00	H
ATOM	2134	HA	ILE	A	337	11.591	−38.387	−35.216	1.00	32.92	H
ATOM	2135	HB	ILE	A	337	12.532	−35.578	−35.821	1.00	32.92	H
ATOM	2136	1HG1	ILE	A	337	12.229	−36.471	−33.031	1.00	32.92	H
ATOM	2137	2HG1	ILE	A	337	13.332	−37.510	−33.966	1.00	32.92	H
ATOM	2138	1HG2	ILE	A	337	10.832	−34.756	−34.225	1.00	32.92	H
ATOM	2139	2HG2	ILE	A	337	10.139	−36.391	−34.113	1.00	32.92	H
ATOM	2140	3HG2	ILE	A	337	10.064	−35.496	−35.650	1.00	32.92	H
ATOM	2141	1HD1	ILE	A	337	14.556	−35.672	−32.870	1.00	32.92	H
ATOM	2142	2HD1	ILE	A	337	13.530	−34.483	−33.707	1.00	32.92	H

ATOM 2143 3HD1 ILE A 337 14.633 −35.522 −34.642 1.00 32.92 H
ATOM 2144 N THR A 338 9.645 −38.289 −36.725 1.00 33.99 N
ATOM 2145 CA THR A 338 8.498 −38.439 −37.643 1.00 34.40 C
ATOM 2146 C THR A 338 7.315 −37.519 −37.323 1.00 34.37 C
ATOM 2147 O THR A 338 6.333 −37.512 −38.064 1.00 34.43 O
ATOM 2148 CB THR A 338 8.022 −39.899 −37.683 1.00 35.02 C
ATOM 2149 OG1 THR A 338 7.709 −40.356 −36.386 1.00 35.10 O
ATOM 2150 CG2 THR A 338 9.081 −40.844 −38.252 1.00 35.14 C
ATOM 2151 HN THR A 338 9.632 −38.830 −35.874 1.00 0.00 H
ATOM 2152 HG1 THR A 338 8.436 −40.915 −36.101 1.00 0.00 H
ATOM 2153 HA THR A 338 8.847 −38.183 −38.644 1.00 34.64 H
ATOM 2154 HB THR A 338 7.127 −39.960 −38.303 1.00 34.64 H
ATOM 2155 1HG2 THR A 338 8.694 −41.863 −38.258 1.00 34.64 H
ATOM 2156 2HG2 THR A 338 9.978 −40.800 −37.634 1.00 34.64 H
ATOM 2157 3HG2 THR A 338 9.327 −40.543 −39.270 1.00 34.64 H
ATOM 2158 N LYS A 339 7.394 −36.723 −36.246 1.00 44.67 N
ATOM 2159 CA LYS A 339 6.368 −35.754 −35.830 1.00 44.64 C
ATOM 2160 C LYS A 339 7.001 −34.518 −35.195 1.00 44.06 C
ATOM 2161 O LYS A 339 7.706 −34.636 −34.193 1.00 43.81 O
ATOM 2162 CB LYS A 339 5.399 −36.389 −34.823 1.00 45.08 C
ATOM 2163 CG LYS A 339 4.450 −37.428 −35.434 1.00 45.68 C
ATOM 2164 CD LYS A 339 3.465 −37.887 −34.356 1.00 46.21 C
ATOM 2165 CE LYS A 339 2.395 −38.812 −34.929 1.00 46.84 C
ATOM 2166 NZ LYS A 339 1.403 −39.148 −33.878 1.00 47.34 N1+
ATOM 2167 HN LYS A 339 8.243 −36.763 −35.699 1.00 0.00 H
ATOM 2168 HZ1 LYS A 339 0.676 −39.745 −34.247 1.00 0.00 H
ATOM 2169 HZ2 LYS A 339 0.983 −38.297 −33.520 1.00 0.00 H

ATOM	2170	HZ3	LYS	A	339	1.858	−39.618	−33.106	1.00	0.00	H
ATOM	2171	HA	LYS	A	339	5.804	−35.444	−36.710	1.00	45.37	H
ATOM	2172	HB1	LYS	A	339	5.986	−36.878	−34.045	1.00	45.37	H
ATOM	2173	HB2	LYS	A	339	4.797	−35.595	−34.380	1.00	45.37	H
ATOM	2174	HG1	LYS	A	339	3.903	−36.981	−36.264	1.00	45.37	H
ATOM	2175	HG2	LYS	A	339	5.024	−38.282	−35.794	1.00	45.37	H
ATOM	2176	HD1	LYS	A	339	4.014	−38.420	−33.579	1.00	45.37	H
ATOM	2177	HD2	LYS	A	339	2.981	−37.011	−33.924	1.00	45.37	H
ATOM	2178	HE1	LYS	A	339	1.890	−38.312	−35.756	1.00	45.37	H
ATOM	2179	HE2	LYS	A	339	2.864	−39.728	−35.289	1.00	45.37	H
ATOM	2180	N	VAL	A	340	6.590	−33.344	−35.671	1.00	33.47	N
ATOM	2181	CA	VAL	A	340	6.974	−32.042	−35.117	1.00	32.97	C
ATOM	2182	C	VAL	A	340	5.751	−31.124	−35.114	1.00	33.00	C
ATOM	2183	O	VAL	A	340	5.136	−30.895	−36.154	1.00	33.08	O
ATOM	2184	CB	VAL	A	340	8.144	−31.405	−35.896	1.00	32.55	C
ATOM	2185	CG1	VAL	A	340	8.675	−30.188	−35.137	1.00	32.09	C
ATOM	2186	CG2	VAL	A	340	9.329	−32.359	−36.108	1.00	32.46	C
ATOM	2187	HN	VAL	A	340	5.960	−33.333	−36.458	1.00	0.00	H
ATOM	2188	HA	VAL	A	340	7.292	−32.191	−34.085	1.00	32.80	H
ATOM	2189	HB	VAL	A	340	7.779	−31.078	−36.870	1.00	32.80	H
ATOM	2190	1HG1	VAL	A	340	9.501	−29.744	−35.693	1.00	32.80	H
ATOM	2191	2HG1	VAL	A	340	9.026	−30.498	−34.153	1.00	32.80	H
ATOM	2192	3HG1	VAL	A	340	7.878	−29.454	−35.023	1.00	32.80	H
ATOM	2193	1HG2	VAL	A	340	10.115	−31.846	−36.662	1.00	32.80	H
ATOM	2194	2HG2	VAL	A	340	8.998	−33.231	−36.672	1.00	32.80	H
ATOM	2195	3HG2	VAL	A	340	9.717	−32.678	−35.141	1.00	32.80	H
ATOM	2196	N	GLU	A	341	5.341	−30.652	−33.941	1.00	49.50	N

ATOM	2197	CA	GLU	A	341	4.078	−29.938	−33.728	1.00	49.55	C
ATOM	2198	C	GLU	A	341	4.362	−28.475	−33.355	1.00	49.06	C
ATOM	2199	O	GLU	A	341	4.981	−28.189	−32.331	1.00	48.77	O
ATOM	2200	CB	GLU	A	341	3.234	−30.675	−32.672	1.00	49.79	C
ATOM	2201	CG	GLU	A	341	2.808	−32.075	−33.165	1.00	50.32	C
ATOM	2202	CD	GLU	A	341	2.043	−32.921	−32.134	1.00	50.60	C
ATOM	2203	OE1	GLU	A	341	2.082	−32.579	−30.928	1.00	50.33	O
ATOM	2204	OE2	GLU	A	341	1.618	−34.032	−32.534	1.00	51.10	O
ATOM	2205	HN	GLU	A	341	5.866	−30.920	−33.111	1.00	0.00	H
ATOM	2206	HA	GLU	A	341	3.524	−29.945	−34.667	1.00	49.89	H
ATOM	2207	HB1	GLU	A	341	3.823	−30.784	−31.761	1.00	49.89	H
ATOM	2208	HB2	GLU	A	341	2.341	−30.087	−32.459	1.00	49.89	H
ATOM	2209	HG1	GLU	A	341	2.168	−31.946	−34.038	1.00	49.89	H
ATOM	2210	HG2	GLU	A	341	3.707	−32.621	−33.450	1.00	49.89	H
ATOM	2211	N	ARG	A	342	4.044	−27.543	−34.267	1.00	32.42	N
ATOM	2212	CA	ARG	A	342	4.571	−26.161	−34.234	1.00	31.99	C
ATOM	2213	C	ARG	A	342	3.576	−25.054	−34.609	1.00	32.01	C
ATOM	2214	O	ARG	A	342	3.972	−23.962	−35.009	1.00	31.69	O
ATOM	2215	CB	ARG	A	342	5.925	−26.076	−34.970	1.00	31.67	C
ATOM	2216	CG	ARG	A	342	5.940	−26.320	−36.489	1.00	31.83	C
ATOM	2217	CD	ARG	A	342	5.754	−27.792	−36.898	1.00	32.12	C
ATOM	2218	NE	ARG	A	342	6.327	−28.106	−38.220	1.00	32.19	N
ATOM	2219	CZ	ARG	A	342	6.017	−27.592	−39.394	1.00	32.47	C
ATOM	2220	NH1	ARG	A	342	5.168	−26.627	−39.571	1.00	32.71	N
ATOM	2221	NH2	ARG	A	342	6.628	−27.982	−40.456	1.00	32.50	N
ATOM	2222	HN	ARG	A	342	3.613	−27.868	−35.119	1.00	0.00	H
ATOM	2223	HE	ARG	A	342	7.083	−28.795	−38.267	1.00	0.00	H

ATOM	2224	1HH1	ARG	A	342	4.839	−26.086	−38.798	1.00	0.00	H
ATOM	2225	2HH1	ARG	A	342	5.221	−26.165	−40.461	1.00	0.00	H
ATOM	2226	1HH2	ARG	A	342	7.363	−28.665	−40.272	1.00	0.00	H
ATOM	2227	2HH2	ARG	A	342	6.716	−27.339	−41.231	1.00	0.00	H
ATOM	2228	HA	ARG	A	342	4.810	−25.978	−33.186	1.00	32.15	H
ATOM	2229	HB1	ARG	A	342	6.324	−25.076	−34.799	1.00	32.15	H
ATOM	2230	HB2	ARG	A	342	6.587	−26.814	−34.517	1.00	32.15	H
ATOM	2231	HG1	ARG	A	342	5.134	−25.738	−36.936	1.00	32.15	H
ATOM	2232	HG2	ARG	A	342	6.898	−25.976	−36.880	1.00	32.15	H
ATOM	2233	HD1	ARG	A	342	6.239	−28.422	−36.152	1.00	32.15	H
ATOM	2234	HD2	ARG	A	342	4.687	−28.012	−36.922	1.00	32.15	H
ATOM	2235	N	GLY	A	343	2.279	−25.299	−34.424	1.00	32.41	N
ATOM	2236	CA	GLY	A	343	1.241	−24.266	−34.533	1.00	32.51	C
ATOM	2237	C	GLY	A	343	1.241	−23.542	−35.887	1.00	32.44	C
ATOM	2238	O	GLY	A	343	1.014	−24.167	−36.920	1.00	32.74	O
ATOM	2239	HN	GLY	A	343	2.026	−26.199	−34.049	1.00	0.00	H
ATOM	2240	HA1	GLY	A	343	0.268	−24.738	−34.394	1.00	32.53	H
ATOM	2241	HA2	GLY	A	343	1.404	−23.529	−33.747	1.00	32.53	H
ATOM	2242	N	LYS	A	344	1.447	−22.216	−35.878	1.00	32.05	N
ATOM	2243	CA	LYS	A	344	1.487	−21.372	−37.090	1.00	31.95	C
ATOM	2244	C	LYS	A	344	2.871	−21.250	−37.749	1.00	31.64	C
ATOM	2245	O	LYS	A	344	2.976	−20.586	−38.778	1.00	31.67	O
ATOM	2246	CB	LYS	A	344	0.877	−19.984	−36.783	1.00	31.81	C
ATOM	2247	CG	LYS	A	344	−0.319	−19.627	−37.685	1.00	32.03	C
ATOM	2248	CD	LYS	A	344	0.033	−19.432	−39.170	1.00	31.88	C
ATOM	2249	CE	LYS	A	344	−1.245	−19.149	−39.969	1.00	32.20	C
ATOM	2250	NZ	LYS	A	344	−0.968	−18.970	−41.414	1.00	32.04	N1+

ATOM 2251 HN LYS A 344 1.653 −21.781 −34.989 1.00 0.00 H
ATOM 2252 HZ1 LYS A 344 −0.380 −19.695 −41.793 1.00 0.00 H
ATOM 2253 HZ2 LYS A 344 −0.503 −18.074 −41.591 1.00 0.00 H
ATOM 2254 HZ3 LYS A 344 −1.813 −18.886 −41.958 1.00 0.00 H
ATOM 2255 HA LYS A 344 0.833 −21.846 −37.822 1.00 31.92 H
ATOM 2256 HB1 LYS A 344 0.542 −19.976 −35.746 1.00 31.92 H
ATOM 2257 HB2 LYS A 344 1.651 −19.229 −36.921 1.00 31.92 H
ATOM 2258 HG1 LYS A 344 −1.051 −20.431 −37.612 1.00 31.92 H
ATOM 2259 HG2 LYS A 344 −0.758 −18.700 −37.316 1.00 31.92 H
ATOM 2260 HD1 LYS A 344 0.719 −18.591 −39.273 1.00 31.92 H
ATOM 2261 HD2 LYS A 344 0.507 −20.336 −39.552 1.00 31.92 H
ATOM 2262 HE1 LYS A 344 −1.931 −19.987 −39.843 1.00 31.92 H
ATOM 2263 HE2 LYS A 344 −1.707 −18.240 −39.584 1.00 31.92 H
ATOM 2264 N TYR A 345 3.925 −21.834 −37.175 1.00 31.63 N
ATOM 2265 CA TYR A 345 5.253 −21.852 −37.799 1.00 31.32 C
ATOM 2266 C TYR A 345 5.272 −22.804 −38.998 1.00 31.52 C
ATOM 2267 O TYR A 345 4.770 −23.925 −38.926 1.00 31.84 O
ATOM 2268 CB TYR A 345 6.342 −22.182 −36.768 1.00 31.08 C
ATOM 2269 CG TYR A 345 6.509 −21.056 −35.772 1.00 30.90 C
ATOM 2270 CD1 TYR A 345 5.715 −21.007 −34.612 1.00 31.05 C
ATOM 2271 CD2 TYR A 345 7.365 −19.982 −36.078 1.00 30.61 C
ATOM 2272 CE1 TYR A 345 5.729 −19.858 −33.799 1.00 30.91 C
ATOM 2273 CE2 TYR A 345 7.389 −18.837 −35.264 1.00 30.48 C
ATOM 2274 CZ TYR A 345 6.543 −18.758 −34.144 1.00 30.63 C
ATOM 2275 OH TYR A 345 6.492 −17.603 −33.439 1.00 30.51 O
ATOM 2276 HN TYR A 345 3.782 −22.425 −36.367 1.00 0.00 H
ATOM 2277 HH TYR A 345 5.833 −17.687 −32.699 1.00 0.00 H

ATOM 2278 HA TYR A 345 5.452 −20.848 −38.175 1.00 31.04 H
ATOM 2279 HB1 TYR A 345 6.063 −23.091 −36.234 1.00 31.04 H
ATOM 2280 HB2 TYR A 345 7.287 −22.341 −37.287 1.00 31.04 H
ATOM 2281 HD1 TYR A 345 5.089 −21.858 −34.343 1.00 31.04 H
ATOM 2282 HD2 TYR A 345 8.014 −20.039 −36.952 1.00 31.04 H
ATOM 2283 HE1 TYR A 345 5.111 −19.818 −32.902 1.00 31.04 H
ATOM 2284 HE2 TYR A 345 8.062 −18.012 −35.500 1.00 31.04 H
ATOM 2285 N ASP A 346 5.869 −22.350 −40.101 1.00 31.10 N
ATOM 2286 CA ASP A 346 6.030 −23.118 −41.345 1.00 31.28 C
ATOM 2287 C ASP A 346 6.996 −24.298 −41.149 1.00 31.32 C
ATOM 2288 O ASP A 346 6.691 −25.415 −41.580 1.00 31.66 O
ATOM 2289 CB ASP A 346 6.495 −22.127 −42.427 1.00 31.04 C
ATOM 2290 CG ASP A 346 6.955 −22.724 −43.768 1.00 31.26 C
ATOM 2291 OD1 ASP A 346 7.858 −23.583 −43.717 1.00 31.53 O
ATOM 2292 OD2 ASP A 346 6.922 −21.945 −44.746 1.00 31.17 O
ATOM 2293 HN ASP A 346 6.360 −21.466 −40.034 1.00 0.00 H
ATOM 2294 HB1 ASP A 346 5.683 −21.425 −42.615 1.00 0.00 H
ATOM 2295 HB2 ASP A 346 7.331 −21.560 −42.017 1.00 0.00 H
ATOM 2296 HA ASP A 346 5.056 −23.513 −41.635 1.00 31.29 H
ATOM 2297 N LYS A 347 8.019 −24.088 −40.309 1.00 31.00 N
ATOM 2298 CA LYS A 347 9.024 −25.069 −39.880 1.00 31.01 C
ATOM 2299 C LYS A 347 9.731 −24.635 −38.585 1.00 30.69 C
ATOM 2300 O LYS A 347 9.635 −23.466 −38.201 1.00 30.42 O
ATOM 2301 CB LYS A 347 10.021 −25.317 −41.029 1.00 30.92 C
ATOM 2302 CG LYS A 347 10.678 −24.031 −41.554 1.00 30.70 C
ATOM 2303 CD LYS A 347 11.762 −24.261 −42.609 1.00 30.51 C
ATOM 2304 CE LYS A 347 11.314 −24.966 −43.893 1.00 30.82 C

ATOM 2305 NZ LYS A 347 10.284 −24.214 −44.642 1.00 31.19 N1+
ATOM 2306 HN LYS A 347 8.125 −23.148 −39.943 1.00 0.00 H
ATOM 2307 HZ1 LYS A 347 10.019 −24.687 −45.490 1.00 0.00 H
ATOM 2308 HZ2 LYS A 347 10.584 −23.277 −44.885 1.00 0.00 H
ATOM 2309 HZ3 LYS A 347 9.424 −24.097 −44.097 1.00 0.00 H
ATOM 2310 HA LYS A 347 8.508 −26.008 −39.679 1.00 30.81 H
ATOM 2311 HB1 LYS A 347 10.805 −25.983 −40.669 1.00 30.81 H
ATOM 2312 HB2 LYS A 347 9.488 −25.794 −41.852 1.00 30.81 H
ATOM 2313 HG1 LYS A 347 9.901 −23.406 −41.994 1.00 30.81 H
ATOM 2314 HG2 LYS A 347 11.129 −23.510 −40.710 1.00 30.81 H
ATOM 2315 HD1 LYS A 347 12.167 −23.288 −42.887 1.00 30.81 H
ATOM 2316 HD2 LYS A 347 12.547 −24.865 −42.154 1.00 30.81 H
ATOM 2317 HE1 LYS A 347 12.183 −25.098 −44.537 1.00 30.81 H
ATOM 2318 HE2 LYS A 347 10.906 −25.942 −43.628 1.00 30.81 H
ATOM 2319 N VAL A 348 10.462 −25.542 −37.932 1.00 30.74 N
ATOM 2320 CA VAL A 348 11.216 −25.325 −36.672 1.00 30.45 C
ATOM 2321 C VAL A 348 12.532 −26.126 −36.629 1.00 30.37 C
ATOM 2322 O VAL A 348 12.715 −27.076 −37.387 1.00 30.60 O
ATOM 2323 CB VAL A 348 10.369 −25.668 −35.427 1.00 30.60 C
ATOM 2324 CG1 VAL A 348 9.206 −24.701 −35.198 1.00 30.99 C
ATOM 2325 CG2 VAL A 348 9.812 −27.091 −35.479 1.00 30.67 C
ATOM 2326 HN VAL A 348 10.440 −26.496 −38.302 1.00 0.00 H
ATOM 2327 HA VAL A 348 11.471 −24.267 −36.617 1.00 30.63 H
ATOM 2328 HB VAL A 348 11.024 −25.604 −34.558 1.00 30.63 H
ATOM 2329 1HG1 VAL A 348 8.653 −25.001 −34.308 1.00 30.63 H
ATOM 2330 2HG1 VAL A 348 8.541 −24.721 −36.061 1.00 30.63 H
ATOM 2331 3HG1 VAL A 348 9.594 −23.691 −35.062 1.00 30.63 H

ATOM 2332 1HG2 VAL A 348 9.224 −27.285 −34.582 1.00 30.63 H

ATOM 2333 2HG2 VAL A 348 10.636 −27.803 −35.533 1.00 30.63 H

ATOM 2334 3HG2 VAL A 348 9.179 −27.202 −36.359 1.00 30.63 H

ATOM 2335 N MET A 349 13.474 −25.744 −35.756 1.00 30.05 N

ATOM 2336 CA MET A 349 14.831 −26.317 −35.709 1.00 29.89 C

ATOM 2337 C MET A 349 15.505 −26.168 −34.324 1.00 29.58 C

ATOM 2338 O MET A 349 15.299 −25.136 −33.682 1.00 29.38 O

ATOM 2339 CB MET A 349 15.657 −25.606 −36.795 1.00 29.75 C

ATOM 2340 CG MET A 349 16.999 −26.261 −37.103 1.00 29.69 C

ATOM 2341 SD MET A 349 18.024 −25.355 −38.282 1.00 29.34 S

ATOM 2342 CE MET A 349 18.574 −23.966 −37.256 1.00 28.98 C

ATOM 2343 HN MET A 349 13.302 −24.907 −35.207 1.00 0.00 H

ATOM 2344 HB1 MET A 349 15.092 −25.600 −37.726 1.00 0.00 H

ATOM 2345 HB2 MET A 349 15.822 −24.574 −36.491 1.00 0.00 H

ATOM 2346 HA MET A 349 14.771 −27.377 −35.955 1.00 29.58 H

ATOM 2347 HG1 MET A 349 17.555 −26.353 −36.170 1.00 29.58 H

ATOM 2348 HG2 MET A 349 16.807 −27.253 −37.511 1.00 29.58 H

ATOM 2349 HE1 MET A 349 19.219 −23.312 −37.844 1.00 29.58 H

ATOM 2350 HE2 MET A 349 19.128 −24.345 −36.397 1.00 29.58 H

ATOM 2351 HE3 MET A 349 17.707 −23.404 −36.909 1.00 29.58 H

ATOM 2352 N PRO A 350 16.368 −27.107 −33.871 1.00 29.54 N

ATOM 2353 CA PRO A 350 17.294 −26.876 −32.750 1.00 29.23 C

ATOM 2354 C PRO A 350 18.264 −25.722 −33.044 1.00 28.89 C

ATOM 2355 O PRO A 350 18.670 −25.519 −34.188 1.00 28.87 O

ATOM 2356 CB PRO A 350 18.069 −28.187 −32.573 1.00 29.24 C

ATOM 2357 CG PRO A 350 18.061 −28.772 −33.984 1.00 29.53 C

ATOM 2358 CD PRO A 350 16.663 −28.402 −34.472 1.00 29.80 C

ATOM	2359	HA	PRO	A	350	16.728	−26.659	−31.844	1.00	29.30	H
ATOM	2360	HB1	PRO	A	350	19.087	−27.999	−32.231	1.00	29.30	H
ATOM	2361	HB2	PRO	A	350	17.565	−28.850	−31.870	1.00	29.30	H
ATOM	2362	HG1	PRO	A	350	18.831	−28.316	−34.606	1.00	29.30	H
ATOM	2363	HG2	PRO	A	350	18.201	−29.853	−33.965	1.00	29.30	H
ATOM	2364	HD1	PRO	A	350	16.647	−28.329	−35.559	1.00	29.30	H
ATOM	2365	HD2	PRO	A	350	15.937	−29.147	−34.146	1.00	29.30	H
ATOM	2366	N	TYR	A	351	18.590	−24.917	−32.030	1.00	28.63	N
ATOM	2367	CA	TYR	A	351	19.341	−23.674	−32.232	1.00	28.32	C
ATOM	2368	C	TYR	A	351	20.016	−23.186	−30.940	1.00	28.05	C
ATOM	2369	O	TYR	A	351	19.713	−22.114	−30.418	1.00	27.95	O
ATOM	2370	CB	TYR	A	351	18.403	−22.623	−32.867	1.00	28.34	C
ATOM	2371	CG	TYR	A	351	19.048	−21.560	−33.742	1.00	28.15	C
ATOM	2372	CD1	TYR	A	351	18.568	−20.241	−33.675	1.00	28.01	C
ATOM	2373	CD2	TYR	A	351	19.979	−21.910	−34.744	1.00	28.14	C
ATOM	2374	CE1	TYR	A	351	18.960	−19.297	−34.639	1.00	27.86	C
ATOM	2375	CE2	TYR	A	351	20.447	−20.939	−35.652	1.00	27.98	C
ATOM	2376	CZ	TYR	A	351	19.924	−19.630	−35.609	1.00	27.84	C
ATOM	2377	OH	TYR	A	351	20.328	−18.683	−36.496	1.00	27.70	O
ATOM	2378	HN	TYR	A	351	18.203	−25.107	−31.110	1.00	0.00	H
ATOM	2379	HH	TYR	A	351	19.667	−17.995	−36.600	1.00	0.00	H
ATOM	2380	HA	TYR	A	351	20.131	−23.884	−32.953	1.00	28.08	H
ATOM	2381	HB1	TYR	A	351	17.678	−23.158	−33.480	1.00	28.08	H
ATOM	2382	HB2	TYR	A	351	17.886	−22.111	−32.055	1.00	28.08	H
ATOM	2383	HD1	TYR	A	351	17.890	−19.949	−32.873	1.00	28.08	H
ATOM	2384	HD2	TYR	A	351	20.338	−22.937	−34.816	1.00	28.08	H
ATOM	2385	HE1	TYR	A	351	18.515	−18.302	−34.636	1.00	28.08	H

ATOM 2386 HE2 TYR A 351 21.211 −21.199 −36.385 1.00 28.08 H

ATOM 2387 N GLY A 352 20.890 −24.009 −30.358 1.00 61.59 N

ATOM 2388 CA GLY A 352 21.552 −23.673 −29.096 1.00 61.31 C

ATOM 2389 C GLY A 352 22.239 −24.860 −28.425 1.00 61.32 C

ATOM 2390 O GLY A 352 21.687 −25.952 −28.479 1.00 61.57 O

ATOM 2391 HN GLY A 352 21.040 −24.929 −30.753 1.00 0.00 H

ATOM 2392 HA1 GLY A 352 22.303 −22.909 −29.295 1.00 61.45 H

ATOM 2393 HA2 GLY A 352 20.803 −23.277 −28.410 1.00 61.45 H

ATOM 2394 N PRO A 353 23.405 −24.702 −27.775 1.00 27.41 N

ATOM 2395 CA PRO A 353 24.228 −25.843 −27.368 1.00 27.40 C

ATOM 2396 C PRO A 353 23.640 −26.672 −26.211 1.00 27.59 C

ATOM 2397 O PRO A 353 23.677 −27.905 −26.267 1.00 27.81 O

ATOM 2398 CB PRO A 353 25.579 −25.225 −26.999 1.00 27.05 C

ATOM 2399 CG PRO A 353 25.225 −23.812 −26.536 1.00 26.92 C

ATOM 2400 CD PRO A 353 24.061 −23.444 −27.456 1.00 27.12 C

ATOM 2401 HA PRO A 353 24.367 −26.497 −28.229 1.00 27.33 H

ATOM 2402 HB1 PRO A 353 26.056 −25.787 −26.196 1.00 27.33 H

ATOM 2403 HB2 PRO A 353 26.240 −25.195 −27.865 1.00 27.33 H

ATOM 2404 HG1 PRO A 353 24.918 −23.806 −25.490 1.00 27.33 H

ATOM 2405 HG2 PRO A 353 26.065 −23.131 −26.672 1.00 27.33 H

ATOM 2406 HD1 PRO A 353 23.370 −22.773 −26.946 1.00 27.33 H

ATOM 2407 HD2 PRO A 353 24.429 −22.966 −28.364 1.00 27.33 H

ATOM 2408 N SER A 354 23.019 −26.020 −25.222 1.00 27.51 N

ATOM 2409 CA SER A 354 22.569 −26.648 −23.974 1.00 27.64 C

ATOM 2410 C SER A 354 21.151 −26.224 −23.567 1.00 27.79 C

ATOM 2411 O SER A 354 20.751 −25.060 −23.686 1.00 27.72 O

ATOM 2412 CB SER A 354 23.597 −26.414 −22.855 1.00 27.36 C

ATOM	2413	OG	SER	A	354	23.932	−25.055	−22.653	1.00	27.06	O
ATOM	2414	HN	SER	A	354	23.007	−25.012	−25.237	1.00	0.00	H
ATOM	2415	HG	SER	A	354	24.684	−25.048	−22.043	1.00	0.00	H
ATOM	2416	HA	SER	A	354	22.538	−27.722	−24.155	1.00	27.51	H
ATOM	2417	HB1	SER	A	354	23.186	−26.807	−21.925	1.00	27.51	H
ATOM	2418	HB2	SER	A	354	24.508	−26.957	−23.108	1.00	27.51	H
ATOM	2419	N	GLY	A	355	20.356	−27.239	−23.227	1.00	28.03	N
ATOM	2420	CA	GLY	A	355	18.976	−27.155	−22.758	1.00	28.21	C
ATOM	2421	C	GLY	A	355	18.775	−27.849	−21.408	1.00	28.15	C
ATOM	2422	O	GLY	A	355	19.743	−28.164	−20.716	1.00	27.85	O
ATOM	2423	HN	GLY	A	355	20.767	−28.166	−23.214	1.00	0.00	H
ATOM	2424	HA1	GLY	A	355	18.705	−26.104	−22.655	1.00	28.06	H
ATOM	2425	HA2	GLY	A	355	18.327	−27.628	−23.495	1.00	28.06	H
ATOM	2426	N	ILE	A	356	17.518	−28.117	−21.034	1.00	28.45	N
ATOM	2427	CA	ILE	A	356	17.182	−28.738	−19.741	1.00	28.46	C
ATOM	2428	C	ILE	A	356	16.191	−29.893	−19.859	1.00	28.89	C
ATOM	2429	O	ILE	A	356	15.198	−29.823	−20.574	1.00	29.19	O
ATOM	2430	CB	ILE	A	356	16.704	−27.705	−18.693	1.00	28.26	C
ATOM	2431	CG1	ILE	A	356	15.406	−26.972	−19.102	1.00	28.43	C
ATOM	2432	CG2	ILE	A	356	17.847	−26.733	−18.368	1.00	27.82	C
ATOM	2433	CD1	ILE	A	356	14.821	−26.095	−17.991	1.00	28.26	C
ATOM	2434	HN	ILE	A	356	16.773	−27.945	−21.701	1.00	0.00	H
ATOM	2435	HA	ILE	A	356	18.108	−29.164	−19.354	1.00	28.47	H
ATOM	2436	HB	ILE	A	356	16.484	−28.256	−17.779	1.00	28.47	H
ATOM	2437	1HG1	ILE	A	356	15.624	−26.339	−19.962	1.00	28.47	H
ATOM	2438	2HG1	ILE	A	356	14.662	−27.718	−19.380	1.00	28.47	H
ATOM	2439	1HG2	ILE	A	356	17.508	−26.006	−17.630	1.00	28.47	H

ATOM 2440 2HG2 ILE A 356 18.151 −26.212 −19.276 1.00 28.47 H

ATOM 2441 3HG2 ILE A 356 18.695 −27.289 −17.967 1.00 28.47 H

ATOM 2442 1HD1 ILE A 356 13.912 −25.612 −18.349 1.00 28.47 H

ATOM 2443 2HD1 ILE A 356 15.548 −25.334 −17.707 1.00 28.47 H

ATOM 2444 3HD1 ILE A 356 14.586 −26.714 −17.125 1.00 28.47 H

ATOM 2445 N LYS A 357 16.353 −30.862 −18.959 1.00 42.34 N

ATOM 2446 CA LYS A 357 15.305 −31.792 −18.525 1.00 42.77 C

ATOM 2447 C LYS A 357 14.466 −31.186 −17.398 1.00 42.88 C

ATOM 2448 O LYS A 357 15.002 −30.493 −16.528 1.00 42.60 O

ATOM 2449 CB LYS A 357 15.896 −33.169 −18.159 1.00 42.82 C

ATOM 2450 CG LYS A 357 16.745 −33.253 −16.876 1.00 42.57 C

ATOM 2451 CD LYS A 357 18.001 −32.366 −16.897 1.00 42.06 C

ATOM 2452 CE LYS A 357 19.039 −32.862 −15.886 1.00 41.78 C

ATOM 2453 NZ LYS A 357 20.409 −32.719 −16.420 1.00 41.37 N1+

ATOM 2454 HN LYS A 357 17.180 −30.804 −18.389 1.00 0.00 H

ATOM 2455 HZ1 LYS A 357 21.110 −32.990 −15.734 1.00 0.00 H

ATOM 2456 HZ2 LYS A 357 20.655 −31.760 −16.687 1.00 0.00 H

ATOM 2457 HZ3 LYS A 357 20.513 −33.277 −17.262 1.00 0.00 H

ATOM 2458 HA LYS A 357 14.639 −31.944 −19.374 1.00 42.35 H

ATOM 2459 HB1 LYS A 357 15.063 −33.864 −18.047 1.00 42.35 H

ATOM 2460 HB2 LYS A 357 16.526 −33.487 −18.990 1.00 42.35 H

ATOM 2461 HG1 LYS A 357 16.124 −32.948 −16.034 1.00 42.35 H

ATOM 2462 HG2 LYS A 357 17.059 −34.288 −16.740 1.00 42.35 H

ATOM 2463 HD1 LYS A 357 18.437 −32.389 −17.896 1.00 42.35 H

ATOM 2464 HD2 LYS A 357 17.719 −31.343 −16.646 1.00 42.35 H

ATOM 2465 HE1 LYS A 357 18.951 −32.278 −14.970 1.00 42.35 H

ATOM 2466 HE2 LYS A 357 18.850 −33.913 −15.668 1.00 42.35 H

ATOM 2467 N GLN A 358 13.197 −31.573 −17.347 1.00 29.91 N

ATOM 2468 CA GLN A 358 12.217 −31.262 −16.303 1.00 30.11 C

ATOM 2469 C GLN A 358 11.470 −32.549 −15.928 1.00 30.55 C

ATOM 2470 O GLN A 358 10.349 −32.794 −16.373 1.00 30.95 O

ATOM 2471 CB GLN A 358 11.275 −30.135 −16.765 1.00 30.25 C

ATOM 2472 CG GLN A 358 12.010 −28.797 −16.962 1.00 29.87 C

ATOM 2473 CD GLN A 358 11.070 −27.595 −17.027 1.00 30.05 C

ATOM 2474 OE1 GLN A 358 9.859 −27.693 −17.077 1.00 30.45 O

ATOM 2475 NE2 GLN A 358 11.590 −26.390 −16.974 1.00 29.77 N

ATOM 2476 HN GLN A 358 12.863 −32.132 −18.128 1.00 0.00 H

ATOM 2477 1HE2 GLN A 358 12.582 −26.256 −16.929 1.00 0.00 H

ATOM 2478 2HE2 GLN A 358 10.924 −25.639 −17.010 1.00 0.00 H

ATOM 2479 HA GLN A 358 12.757 −30.914 −15.423 1.00 30.21 H

ATOM 2480 HB1 GLN A 358 10.819 −30.426 −17.711 1.00 30.21 H

ATOM 2481 HB2 GLN A 358 10.497 −29.999 −16.013 1.00 30.21 H

ATOM 2482 HG1 GLN A 358 12.697 −28.654 −16.128 1.00 30.21 H

ATOM 2483 HG2 GLN A 358 12.574 −28.846 −17.894 1.00 30.21 H

ATOM 2484 N GLY A 359 12.221 −33.489 −15.345 1.00 30.49 N

ATOM 2485 CA GLY A 359 11.855 −34.906 −15.367 1.00 30.89 C

ATOM 2486 C GLY A 359 11.900 −35.470 −16.789 1.00 31.08 C

ATOM 2487 O GLY A 359 12.805 −35.150 −17.557 1.00 30.84 O

ATOM 2488 HN GLY A 359 13.179 −33.262 −15.148 1.00 0.00 H

ATOM 2489 HA1 GLY A 359 12.553 −35.463 −14.742 1.00 30.83 H

ATOM 2490 HA2 GLY A 359 10.845 −35.018 −14.973 1.00 30.83 H

ATOM 2491 N ASP A 360 10.856 −36.211 −17.148 1.00 31.53 N

ATOM 2492 CA ASP A 360 10.718 −36.949 −18.411 1.00 31.77 C

ATOM 2493 C ASP A 360 10.544 −36.056 −19.668 1.00 31.71 C

ATOM 2494 O ASP A 360 10.648 −36.532 −20.800 1.00 32.07 O
ATOM 2495 CB ASP A 360 9.501 −37.875 −18.246 1.00 32.39 C
ATOM 2496 CG ASP A 360 9.617 −39.194 −19.004 1.00 32.55 C
ATOM 2497 OD1 ASP A 360 10.722 −39.772 −19.039 1.00 32.33 O
ATOM 2498 OD2 ASP A 360 8.542 −39.696 −19.402 1.00 32.94 O
ATOM 2499 HN ASP A 360 10.151 −36.390 −16.452 1.00 0.00 H
ATOM 2500 HA ASP A 360 11.605 −37.567 −18.548 1.00 32.16 H
ATOM 2501 HB1 ASP A 360 9.382 −38.099 −17.186 1.00 32.16 H
ATOM 2502 HB2 ASP A 360 8.618 −37.348 −18.608 1.00 32.16 H
ATOM 2503 N THR A 361 10.279 −34.751 −19.498 1.00 31.27 N
ATOM 2504 CA THR A 361 10.316 −33.761 −20.586 1.00 31.23 C
ATOM 2505 C THR A 361 11.706 −33.151 −20.735 1.00 30.74 C
ATOM 2506 O THR A 361 12.170 −32.457 −19.830 1.00 30.38 O
ATOM 2507 CB THR A 361 9.261 −32.660 −20.393 1.00 31.43 C
ATOM 2508 OG1 THR A 361 7.967 −33.191 −20.554 1.00 31.83 O
ATOM 2509 CG2 THR A 361 9.338 −31.580 −21.477 1.00 31.61 C
ATOM 2510 HN THR A 361 10.264 −34.400 −18.550 1.00 0.00 H
ATOM 2511 HG1 THR A 361 7.968 −34.122 −20.262 1.00 0.00 H
ATOM 2512 HA THR A 361 10.083 −34.282 −21.515 1.00 31.21 H
ATOM 2513 HB THR A 361 9.363 −32.210 −19.405 1.00 31.21 H
ATOM 2514 1HG2 THR A 361 8.572 −30.826 −21.295 1.00 31.21 H
ATOM 2515 2HG2 THR A 361 9.175 −32.034 −22.455 1.00 31.21 H
ATOM 2516 3HG2 THR A 361 10.322 −31.111 −21.454 1.00 31.21 H
ATOM 2517 N LEU A 362 12.286 −33.229 −21.937 1.00 30.73 N
ATOM 2518 CA LEU A 362 13.448 −32.433 −22.352 1.00 30.30 C
ATOM 2519 C LEU A 362 12.988 −31.128 −23.028 1.00 30.21 C
ATOM 2520 O LEU A 362 11.986 −31.129 −23.744 1.00 30.52 O

ATOM	2521	CB	LEU	A	362	14.363	−33.254	−23.286	1.00	30.34	C
ATOM	2522	CG	LEU	A	362	14.598	−34.727	−22.893	1.00	30.50	C
ATOM	2523	CD1	LEU	A	362	15.581	−35.351	−23.882	1.00	30.55	C
ATOM	2524	CD2	LEU	A	362	15.179	−34.896	−21.489	1.00	30.16	C
ATOM	2525	HN	LEU	A	362	11.832	−33.794	−22.648	1.00	0.00	H
ATOM	2526	HA	LEU	A	362	14.018	−32.172	−21.460	1.00	30.41	H
ATOM	2527	HB1	LEU	A	362	13.917	−33.243	−24.281	1.00	30.41	H
ATOM	2528	HB2	LEU	A	362	15.334	−32.760	−23.317	1.00	30.41	H
ATOM	2529	HG	LEU	A	362	13.651	−35.263	−22.950	1.00	30.41	H
ATOM	2530	1HD1	LEU	A	362	15.755	−36.393	−23.614	1.00	30.41	H
ATOM	2531	2HD1	LEU	A	362	16.524	−34.806	−23.850	1.00	30.41	H
ATOM	2532	3HD1	LEU	A	362	15.166	−35.300	−24.889	1.00	30.41	H
ATOM	2533	1HD2	LEU	A	362	15.319	−35.956	−21.278	1.00	30.41	H
ATOM	2534	2HD2	LEU	A	362	14.493	−34.468	−20.758	1.00	30.41	H
ATOM	2535	3HD2	LEU	A	362	16.140	−34.384	−21.429	1.00	30.41	H
ATOM	2536	N	TYR	A	363	13.800	−30.073	−22.943	1.00	29.80	N
ATOM	2537	CA	TYR	A	363	13.611	−28.778	−23.601	1.00	29.69	C
ATOM	2538	C	TYR	A	363	14.925	−28.317	−24.240	1.00	29.35	C
ATOM	2539	O	TYR	A	363	15.799	−27.756	−23.572	1.00	29.03	O
ATOM	2540	CB	TYR	A	363	13.137	−27.738	−22.580	1.00	29.57	C
ATOM	2541	CG	TYR	A	363	11.701	−27.871	−22.127	1.00	29.95	C
ATOM	2542	CD1	TYR	A	363	10.684	−27.299	−22.913	1.00	30.19	C
ATOM	2543	CD2	TYR	A	363	11.393	−28.466	−20.889	1.00	30.09	C
ATOM	2544	CE1	TYR	A	363	9.363	−27.265	−22.439	1.00	30.57	C
ATOM	2545	CE2	TYR	A	363	10.066	−28.449	−20.419	1.00	30.48	C
ATOM	2546	CZ	TYR	A	363	9.057	−27.832	−21.187	1.00	30.72	C
ATOM	2547	OH	TYR	A	363	7.783	−27.790	−20.732	1.00	31.13	O

```
ATOM   2548  HN  TYR A 363      14.566 −30.126 −22.281  1.00  0.00           H
ATOM   2549  HH  TYR A 363       7.313 −27.014 −21.130  1.00  0.00           H
ATOM   2550  HA  TYR A 363      12.855 −28.882 −24.379  1.00 30.05           H
ATOM   2551  HB1 TYR A 363      13.775 −27.818 −21.700  1.00 30.05           H
ATOM   2552  HB2 TYR A 363      13.257 −26.751 −23.027  1.00 30.05           H
ATOM   2553  HD1 TYR A 363      10.922 −26.882 −23.891  1.00 30.05           H
ATOM   2554  HD2 TYR A 363      12.178 −28.937 −20.297  1.00 30.05           H
ATOM   2555  HE1 TYR A 363       8.578 −26.802 −23.037  1.00 30.05           H
ATOM   2556  HE2 TYR A 363       9.819 −28.912 −19.464  1.00 30.05           H
ATOM   2557  N   PHE A 364      15.043 −28.501 −25.554  1.00 29.44           N
ATOM   2558  CA  PHE A 364      16.165 −28.004 −26.352  1.00 29.16           C
ATOM   2559  C   PHE A 364      15.931 −26.532 −26.752  1.00 29.02           C
ATOM   2560  O   PHE A 364      14.851 −26.237 −27.270  1.00 29.22           O
ATOM   2561  CB  PHE A 364      16.309 −28.888 −27.601  1.00 29.37           C
ATOM   2562  CG  PHE A 364      16.519 −30.364 −27.308  1.00 29.56           C
ATOM   2563  CD1 PHE A 364      17.792 −30.828 −26.930  1.00 29.38           C
ATOM   2564  CD2 PHE A 364      15.443 −31.271 −27.392  1.00 29.95           C
ATOM   2565  CE1 PHE A 364      17.994 −32.190 −26.644  1.00 29.58           C
ATOM   2566  CE2 PHE A 364      15.644 −32.634 −27.104  1.00 30.15           C
ATOM   2567  CZ  PHE A 364      16.919 −33.092 −26.729  1.00 29.96           C
ATOM   2568  HN  PHE A 364      14.272 −28.939 −26.049  1.00  0.00           H
ATOM   2569  HA  PHE A 364      17.079 −28.071 −25.761  1.00 29.53           H
ATOM   2570  HB1 PHE A 364      15.402 −28.785 −28.197  1.00 29.53           H
ATOM   2571  HB2 PHE A 364      17.164 −28.530 −28.175  1.00 29.53           H
ATOM   2572  HD1 PHE A 364      18.625 −30.129 −26.858  1.00 29.53           H
ATOM   2573  HD2 PHE A 364      14.454 −30.916 −27.681  1.00 29.53           H
ATOM   2574  HE1 PHE A 364      18.983 −32.546 −26.357  1.00 29.53           H
```

ATOM 2575 HE2 PHE A 364 14.811 −33.334 −27.172 1.00 29.53 H

ATOM 2576 HZ PHE A 364 17.074 −34.147 −26.504 1.00 29.53 H

ATOM 2577 N PRO A 365 16.905 −25.607 −26.630 1.00 28.68 N

ATOM 2578 CA PRO A 365 16.816 −24.278 −27.247 1.00 28.60 C

ATOM 2579 C PRO A 365 16.688 −24.370 −28.776 1.00 28.76 C

ATOM 2580 O PRO A 365 17.375 −25.156 −29.433 1.00 28.80 O

ATOM 2581 CB PRO A 365 18.091 −23.538 −26.831 1.00 28.21 C

ATOM 2582 CG PRO A 365 19.075 −24.682 −26.613 1.00 28.10 C

ATOM 2583 CD PRO A 365 18.198 −25.757 −25.981 1.00 28.38 C

ATOM 2584 HA PRO A 365 15.949 −23.753 −26.845 1.00 28.50 H

ATOM 2585 HB1 PRO A 365 18.433 −22.868 −27.620 1.00 28.50 H

ATOM 2586 HB2 PRO A 365 17.936 −22.971 −25.913 1.00 28.50 H

ATOM 2587 HG1 PRO A 365 19.497 −25.024 −27.558 1.00 28.50 H

ATOM 2588 HG2 PRO A 365 19.880 −24.386 −25.941 1.00 28.50 H

ATOM 2589 HD1 PRO A 365 18.610 −26.749 −26.169 1.00 28.50 H

ATOM 2590 HD2 PRO A 365 18.109 −25.598 −24.906 1.00 28.50 H

ATOM 2591 N ALA A 366 15.809 −23.551 −29.351 1.00 28.87 N

ATOM 2592 CA ALA A 366 15.298 −23.750 −30.705 1.00 29.07 C

ATOM 2593 C ALA A 366 14.928 −22.440 −31.419 1.00 29.01 C

ATOM 2594 O ALA A 366 14.850 −21.373 −30.809 1.00 28.83 O

ATOM 2595 CB ALA A 366 14.084 −24.678 −30.594 1.00 29.47 C

ATOM 2596 HN ALA A 366 15.276 −22.924 −28.756 1.00 0.00 H

ATOM 2597 HA ALA A 366 16.065 −24.256 −31.291 1.00 29.05 H

ATOM 2598 HB1 ALA A 366 13.669 −24.854 −31.586 1.00 29.05 H

ATOM 2599 HB2 ALA A 366 13.327 −24.213 −29.962 1.00 29.05 H

ATOM 2600 HB3 ALA A 366 14.391 −25.627 −30.155 1.00 29.05 H

ATOM 2601 N VAL A 367 14.563 −22.559 −32.698 1.00 29.17 N

ATOM	2602	CA	VAL	A	367	14.020	−21.481	−33.529	1.00	29.11	C
ATOM	2603	C	VAL	A	367	12.843	−21.965	−34.376	1.00	29.41	C
ATOM	2604	O	VAL	A	367	12.872	−23.062	−34.933	1.00	29.61	O
ATOM	2605	CB	VAL	A	367	15.137	−20.835	−34.372	1.00	28.84	C
ATOM	2606	CG1	VAL	A	367	15.714	−21.752	−35.456	1.00	28.96	C
ATOM	2607	CG2	VAL	A	367	14.710	−19.527	−35.043	1.00	28.75	C
ATOM	2608	HN	VAL	A	367	14.650	−23.477	−33.127	1.00	0.00	H
ATOM	2609	HA	VAL	A	367	13.639	−20.714	−32.855	1.00	29.12	H
ATOM	2610	HB	VAL	A	367	15.953	−20.593	−33.690	1.00	29.12	H
ATOM	2611	1HG1	VAL	A	367	16.494	−21.222	−36.003	1.00	29.12	H
ATOM	2612	2HG1	VAL	A	367	14.922	−22.044	−36.145	1.00	29.12	H
ATOM	2613	3HG1	VAL	A	367	16.138	−22.642	−34.992	1.00	29.12	H
ATOM	2614	1HG2	VAL	A	367	15.543	−19.125	−35.620	1.00	29.12	H
ATOM	2615	2HG2	VAL	A	367	14.416	−18.806	−34.280	1.00	29.12	H
ATOM	2616	3HG2	VAL	A	367	13.867	−19.717	−35.707	1.00	29.12	H
ATOM	2617	N	GLY	A	368	11.819	−21.123	−34.498	1.00	29.46	N
ATOM	2618	CA	GLY	A	368	10.718	−21.270	−35.444	1.00	29.75	C
ATOM	2619	C	GLY	A	368	10.811	−20.262	−36.583	1.00	29.61	C
ATOM	2620	O	GLY	A	368	11.202	−19.110	−36.382	1.00	29.36	O
ATOM	2621	HN	GLY	A	368	11.868	−20.253	−33.980	1.00	0.00	H
ATOM	2622	HA1	GLY	A	368	10.744	−22.277	−35.861	1.00	29.55	H
ATOM	2623	HA2	GLY	A	368	9.777	−21.119	−34.916	1.00	29.55	H
ATOM	2624	N	PHE	A	369	10.417	−20.698	−37.778	1.00	29.79	N
ATOM	2625	CA	PHE	A	369	10.414	−19.909	−39.006	1.00	29.68	C
ATOM	2626	C	PHE	A	369	8.964	−19.639	−39.428	1.00	29.92	C
ATOM	2627	O	PHE	A	369	8.154	−20.566	−39.540	1.00	30.18	O
ATOM	2628	CB	PHE	A	369	11.165	−20.655	−40.116	1.00	29.68	C

ATOM 2629 CG PHE A 369 12.571 −21.156 −39.802 1.00 29.51 C

ATOM 2630 CD1 PHE A 369 13.682 −20.589 −40.451 1.00 29.32 C

ATOM 2631 CD2 PHE A 369 12.778 −22.253 −38.942 1.00 29.56 C

ATOM 2632 CE1 PHE A 369 14.973 −21.115 −40.256 1.00 29.18 C

ATOM 2633 CE2 PHE A 369 14.065 −22.768 −38.732 1.00 29.41 C

ATOM 2634 CZ PHE A 369 15.166 −22.208 −39.396 1.00 29.23 C

ATOM 2635 HN PHE A 369 10.095 −21.659 −37.854 1.00 0.00 H

ATOM 2636 HA PHE A 369 10.912 −18.958 −38.818 1.00 29.59 H

ATOM 2637 HB1 PHE A 369 10.564 −21.522 −40.391 1.00 29.59 H

ATOM 2638 HB2 PHE A 369 11.242 −19.980 −40.968 1.00 29.59 H

ATOM 2639 HD1 PHE A 369 13.542 −19.733 −41.112 1.00 29.59 H

ATOM 2640 HD2 PHE A 369 11.927 −22.707 −38.434 1.00 29.59 H

ATOM 2641 HE1 PHE A 369 15.824 −20.673 −40.774 1.00 29.59 H

ATOM 2642 HE2 PHE A 369 14.210 −23.606 −38.051 1.00 29.59 H

ATOM 2643 HZ PHE A 369 16.165 −22.618 −39.246 1.00 29.59 H

ATOM 2644 N LEU A 370 8.610 −18.370 −39.630 1.00 29.85 N

ATOM 2645 CA LEU A 370 7.235 −17.944 −39.910 1.00 30.06 C

ATOM 2646 C LEU A 370 7.226 −16.895 −41.035 1.00 29.90 C

ATOM 2647 O LEU A 370 7.999 −15.938 −40.952 1.00 29.61 O

ATOM 2648 CB LEU A 370 6.611 −17.465 −38.586 1.00 30.07 C

ATOM 2649 CG LEU A 370 5.220 −16.811 −38.688 1.00 30.32 C

ATOM 2650 CD1 LEU A 370 4.343 −17.228 −37.506 1.00 30.46 C

ATOM 2651 CD2 LEU A 370 5.334 −15.287 −38.657 1.00 30.17 C

ATOM 2652 HN LEU A 370 9.313 −17.648 −39.493 1.00 0.00 H

ATOM 2653 HA LEU A 370 6.674 −18.813 −40.253 1.00 30.05 H

ATOM 2654 HB1 LEU A 370 6.525 −18.329 −37.927 1.00 30.06 H

ATOM 2655 HB2 LEU A 370 7.290 −16.736 −38.143 1.00 30.06 H

ATOM 2656 HG LEU A 370 4.743 −17.119 −39.618 1.00 30.05 H

ATOM 2657 1HD1 LEU A 370 3.364 −16.756 −37.595 1.00 30.05 H

ATOM 2658 2HD1 LEU A 370 4.814 −16.913 −36.575 1.00 30.05 H

ATOM 2659 3HD1 LEU A 370 4.225 −18.312 −37.505 1.00 30.05 H

ATOM 2660 1HD2 LEU A 370 4.340 −14.846 −38.730 1.00 30.05 H

ATOM 2661 2HD2 LEU A 370 5.943 −14.951 −39.496 1.00 30.05 H

ATOM 2662 3HD2 LEU A 370 5.801 −14.976 −37.722 1.00 30.05 H

ATOM 2663 N PRO A 371 6.494 −17.104 −42.148 1.00 30.08 N

ATOM 2664 CA PRO A 371 6.512 −16.161 −43.261 1.00 29.90 C

ATOM 2665 C PRO A 371 6.012 −14.780 −42.818 1.00 29.87 C

ATOM 2666 O PRO A 371 5.013 −14.667 −42.110 1.00 30.10 O

ATOM 2667 CB PRO A 371 5.670 −16.776 −44.382 1.00 30.11 C

ATOM 2668 CG PRO A 371 4.951 −17.976 −43.764 1.00 30.46 C

ATOM 2669 CD PRO A 371 5.685 −18.272 −42.457 1.00 30.45 C

ATOM 2670 HA PRO A 371 7.538 −16.061 −43.614 1.00 30.14 H

ATOM 2671 HB1 PRO A 371 4.945 −16.051 −44.752 1.00 30.14 H

ATOM 2672 HB2 PRO A 371 6.311 −17.100 −45.202 1.00 30.14 H

ATOM 2673 HG1 PRO A 371 3.907 −17.733 −43.566 1.00 30.14 H

ATOM 2674 HG2 PRO A 371 5.004 −18.836 −44.432 1.00 30.14 H

ATOM 2675 HD1 PRO A 371 4.967 −18.452 −41.657 1.00 30.14 H

ATOM 2676 HD2 PRO A 371 6.323 −19.148 −42.576 1.00 30.14 H

ATOM 2677 N ARG A 372 6.701 −13.705 −43.220 1.00 39.74 N

ATOM 2678 CA ARG A 372 6.431 −12.338 −42.733 1.00 39.73 C

ATOM 2679 C ARG A 372 5.151 −11.691 −43.288 1.00 39.94 C

ATOM 2680 O ARG A 372 4.867 −10.549 −42.948 1.00 40.06 O

ATOM 2681 CB ARG A 372 7.687 −11.449 −42.828 1.00 39.44 C

ATOM 2682 CG ARG A 372 8.079 −10.972 −44.232 1.00 39.24 C

ATOM	2683	CD	ARG	A	372	9.132	−9.846	−44.200	1.00	39.00	C
ATOM	2684	NE	ARG	A	372	10.407	−10.198	−43.526	1.00	38.78	N
ATOM	2685	CZ	ARG	A	372	10.953	−9.577	−42.488	1.00	38.60	C
ATOM	2686	NH1	ARG	A	372	10.243	−8.821	−41.698	1.00	38.61	N
ATOM	2687	NH2	ARG	A	372	12.215	−9.714	−42.203	1.00	38.43	N
ATOM	2688	HN	ARG	A	372	7.532	−13.866	−43.783	1.00	0.00	H
ATOM	2689	HE	ARG	A	372	11.009	−10.840	−44.030	1.00	0.00	H
ATOM	2690	1HH1	ARG	A	372	9.255	−8.798	−41.836	1.00	0.00	H
ATOM	2691	2HH1	ARG	A	372	10.639	−8.449	−40.840	1.00	0.00	H
ATOM	2692	1HH2	ARG	A	372	12.810	−10.224	−42.849	1.00	0.00	H
ATOM	2693	2HH2	ARG	A	372	12.662	−9.093	−41.538	1.00	0.00	H
ATOM	2694	HA	ARG	A	372	6.248	−12.452	−41.665	1.00	39.23	H
ATOM	2695	HB1	ARG	A	372	7.515	−10.565	−42.213	1.00	39.23	H
ATOM	2696	HB2	ARG	A	372	8.526	−12.016	−42.424	1.00	39.23	H
ATOM	2697	HG1	ARG	A	372	8.487	−11.817	−44.787	1.00	39.23	H
ATOM	2698	HG2	ARG	A	372	7.187	−10.602	−44.737	1.00	39.23	H
ATOM	2699	HD1	ARG	A	372	9.359	−9.567	−45.229	1.00	39.23	H
ATOM	2700	HD2	ARG	A	372	8.698	−8.993	−43.679	1.00	39.23	H
ATOM	2701	N	THR	A	373	4.352	−12.439	−44.049	1.00	29.86	N
ATOM	2702	CA	THR	A	373	2.921	−12.186	−44.301	1.00	30.10	C
ATOM	2703	C	THR	A	373	2.055	−12.459	−43.066	1.00	30.40	C
ATOM	2704	O	THR	A	373	1.123	−11.712	−42.791	1.00	30.61	O
ATOM	2705	CB	THR	A	373	2.430	−13.098	−45.440	1.00	30.20	C
ATOM	2706	OG1	THR	A	373	2.840	−14.431	−45.220	1.00	30.34	O
ATOM	2707	CG2	THR	A	373	3.014	−12.677	−46.788	1.00	29.92	C
ATOM	2708	HN	THR	A	373	4.659	−13.378	−44.257	1.00	0.00	H
ATOM	2709	HG1	THR	A	373	2.075	−14.915	−44.812	1.00	0.00	H

ATOM 2710 HA THR A 373 2.793 −11.146 −44.600 1.00 30.20 H

ATOM 2711 HB THR A 373 1.342 −13.059 −45.490 1.00 30.20 H

ATOM 2712 1HG2 THR A 373 2.646 −13.343 −47.568 1.00 30.20 H

ATOM 2713 2HG2 THR A 373 4.102 −12.733 −46.747 1.00 30.20 H

ATOM 2714 3HG2 THR A 373 2.712 −11.654 −47.012 1.00 30.20 H

ATOM 2715 N GLU A 374 2.429 −13.447 −42.251 1.00 30.43 N

ATOM 2716 CA GLU A 374 1.701 −13.846 −41.040 1.00 30.71 C

ATOM 2717 C GLU A 374 2.125 −13.032 −39.808 1.00 30.59 C

ATOM 2718 O GLU A 374 1.435 −13.051 −38.788 1.00 30.80 O

ATOM 2719 CB GLU A 374 1.941 −15.341 −40.749 1.00 30.92 C

ATOM 2720 CG GLU A 374 1.946 −16.266 −41.971 1.00 31.03 C

ATOM 2721 CD GLU A 374 0.715 −16.072 −42.853 1.00 31.30 C

ATOM 2722 OE1 GLU A 374 −0.327 −16.638 −42.450 1.00 31.63 O

ATOM 2723 OE2 GLU A 374 0.905 −15.677 −44.023 1.00 31.19 O

ATOM 2724 HN GLU A 374 3.217 −14.023 −42.528 1.00 0.00 H

ATOM 2725 HA GLU A 374 0.635 −13.692 −41.209 1.00 30.96 H

ATOM 2726 HB1 GLU A 374 2.908 −15.434 −40.255 1.00 30.96 H

ATOM 2727 HB2 GLU A 374 1.154 −15.681 −40.076 1.00 30.96 H

ATOM 2728 HG1 GLU A 374 2.837 −16.058 −42.563 1.00 30.96 H

ATOM 2729 HG2 GLU A 374 1.971 −17.300 −41.627 1.00 30.96 H

ATOM 2730 N PHE A 375 3.310 −12.413 −39.854 1.00 30.27 N

ATOM 2731 CA PHE A 375 3.981 −11.826 −38.695 1.00 30.14 C

ATOM 2732 C PHE A 375 3.301 −10.538 −38.208 1.00 30.19 C

ATOM 2733 O PHE A 375 3.645 −9.430 −38.620 1.00 30.11 O

ATOM 2734 CB PHE A 375 5.469 −11.610 −39.009 1.00 29.79 C

ATOM 2735 CG PHE A 375 6.268 −11.037 −37.850 1.00 29.62 C

ATOM 2736 CD1 PHE A 375 6.228 −11.653 −36.582 1.00 29.76 C

ATOM 2737 CD2 PHE A 375 7.054 −9.884 −38.036 1.00 29.34 C

ATOM 2738 CE1 PHE A 375 6.949 −11.107 −35.506 1.00 29.61 C

ATOM 2739 CE2 PHE A 375 7.789 −9.350 −36.964 1.00 29.19 C

ATOM 2740 CZ PHE A 375 7.728 −9.954 −35.696 1.00 29.33 C

ATOM 2741 HN PHE A 375 3.806 −12.439 −40.731 1.00 0.00 H

ATOM 2742 HA PHE A 375 3.922 −12.551 −37.884 1.00 29.76 H

ATOM 2743 HB1 PHE A 375 5.904 −12.571 −39.284 1.00 29.76 H

ATOM 2744 HB2 PHE A 375 5.545 −10.922 −39.851 1.00 29.76 H

ATOM 2745 HD1 PHE A 375 5.636 −12.556 −36.436 1.00 29.76 H

ATOM 2746 HD2 PHE A 375 7.093 −9.404 −39.014 1.00 29.76 H

ATOM 2747 HE1 PHE A 375 6.904 −11.578 −34.524 1.00 29.76 H

ATOM 2748 HE2 PHE A 375 8.408 −8.466 −37.116 1.00 29.76 H

ATOM 2749 HZ PHE A 375 8.285 −9.528 −34.861 1.00 29.76 H

ATOM 2750 N GLN A 376 2.383 −10.694 −37.255 1.00 30.34 N

ATOM 2751 CA GLN A 376 1.738 −9.579 −36.566 1.00 30.47 C

ATOM 2752 C GLN A 376 2.737 −8.891 −35.624 1.00 30.27 C

ATOM 2753 O GLN A 376 3.091 −9.432 −34.578 1.00 30.24 O

ATOM 2754 CB GLN A 376 0.488 −10.068 −35.805 1.00 30.84 C

ATOM 2755 CG GLN A 376 −0.487 −10.906 −36.652 1.00 31.14 C

ATOM 2756 CD GLN A 376 −0.809 −10.270 −38.001 1.00 31.05 C

ATOM 2757 OE1 GLN A 376 −1.278 −9.150 −38.091 1.00 31.11 O

ATOM 2758 NE2 GLN A 376 −0.492 −10.926 −39.094 1.00 30.92 N

ATOM 2759 HN GLN A 376 2.120 −11.633 −36.996 1.00 0.00 H

ATOM 2760 1HE2 GLN A 376 0.033 −11.789 −39.024 1.00 0.00 H

ATOM 2761 2HE2 GLN A 376 −0.625 −10.447 −39.966 1.00 0.00 H

ATOM 2762 HA GLN A 376 1.420 −8.854 −37.315 1.00 30.71 H

ATOM 2763 HB1 GLN A 376 0.818 −10.677 −34.964 1.00 30.71 H

ATOM 2764 HB2 GLN A 376 −0.048 −9.194 −35.434 1.00 30.71 H
ATOM 2765 HG1 GLN A 376 −0.040 −11.885 −36.828 1.00 30.71 H
ATOM 2766 HG2 GLN A 376 −1.416 −11.025 −36.095 1.00 30.71 H
ATOM 2767 N TYR A 377 3.134 −7.664 −35.962 1.00 50.03 N
ATOM 2768 CA TYR A 377 3.960 −6.806 −35.115 1.00 49.87 C
ATOM 2769 C TYR A 377 3.527 −5.347 −35.266 1.00 49.97 C
ATOM 2770 O TYR A 377 3.466 −4.825 −36.378 1.00 49.92 O
ATOM 2771 CB TYR A 377 5.446 −6.992 −35.455 1.00 49.53 C
ATOM 2772 CG TYR A 377 6.376 −6.200 −34.551 1.00 49.40 C
ATOM 2773 CD1 TYR A 377 6.861 −6.783 −33.362 1.00 49.50 C
ATOM 2774 CD2 TYR A 377 6.705 −4.865 −34.862 1.00 49.20 C
ATOM 2775 CE1 TYR A 377 7.661 −6.029 −32.481 1.00 49.40 C
ATOM 2776 CE2 TYR A 377 7.506 −4.112 −33.983 1.00 49.12 C
ATOM 2777 CZ TYR A 377 7.970 −4.687 −32.783 1.00 49.21 C
ATOM 2778 OH TYR A 377 8.698 −3.943 −31.912 1.00 49.15 O
ATOM 2779 HN TYR A 377 2.846 −7.297 −36.857 1.00 0.00 H
ATOM 2780 HH TYR A 377 8.548 −3.005 −32.036 1.00 0.00 H
ATOM 2781 HA TYR A 377 3.807 −7.102 −34.077 1.00 49.53 H
ATOM 2782 HB1 TYR A 377 5.692 −8.050 −35.362 1.00 49.53 H
ATOM 2783 HB2 TYR A 377 5.608 −6.670 −36.484 1.00 49.53 H
ATOM 2784 HD1 TYR A 377 6.617 −7.818 −33.124 1.00 49.53 H
ATOM 2785 HD2 TYR A 377 6.338 −4.415 −35.785 1.00 49.53 H
ATOM 2786 HE1 TYR A 377 8.041 −6.483 −31.566 1.00 49.53 H
ATOM 2787 HE2 TYR A 377 7.768 −3.083 −34.231 1.00 49.53 H
ATOM 2788 N ASN A 378 3.233 −4.682 −34.148 1.00 34.98 N
ATOM 2789 CA ASN A 378 2.982 −3.245 −34.127 1.00 35.08 C
ATOM 2790 C ASN A 378 4.315 −2.486 −34.127 1.00 34.82 C

ATOM 2791 O ASN A 378 4.971 −2.394 −33.090 1.00 34.72 O

ATOM 2792 CB ASN A 378 2.116 −2.907 −32.902 1.00 35.37 C

ATOM 2793 CG ASN A 378 1.811 −1.422 −32.770 1.00 35.45 C

ATOM 2794 OD1 ASN A 378 1.848 −0.646 −33.715 1.00 35.27 O

ATOM 2795 ND2 ASN A 378 1.605 −0.944 −31.570 1.00 35.74 N

ATOM 2796 HN ASN A 378 3.358 −5.150 −33.265 1.00 0.00 H

ATOM 2797 1HD2 ASN A 378 1.918 −1.486 −30.768 1.00 0.00 H

ATOM 2798 2HD2 ASN A 378 1.418 0.036 −31.482 1.00 0.00 H

ATOM 2799 HA ASN A 378 2.430 −2.976 −35.027 1.00 35.18 H

ATOM 2800 HB1 ASN A 378 1.173 −3.447 −32.986 1.00 35.18 H

ATOM 2801 HB2 ASN A 378 2.644 −3.232 −32.005 1.00 35.18 H

ATOM 2802 N ASP A 379 4.616 −1.790 −35.223 1.00 29.95 N

ATOM 2803 CA ASP A 379 5.781 −0.901 −35.331 1.00 29.73 C

ATOM 2804 C ASP A 379 5.823 0.182 −34.233 1.00 29.83 C

ATOM 2805 O ASP A 379 6.901 0.489 −33.731 1.00 29.65 O

ATOM 2806 CB ASP A 379 5.811 −0.257 −36.722 1.00 29.67 C

ATOM 2807 CG ASP A 379 6.158 −1.207 −37.860 1.00 29.51 C

ATOM 2808 OD1 ASP A 379 6.438 −2.407 −37.646 1.00 29.27 O

ATOM 2809 OD2 ASP A 379 6.246 −0.712 −39.002 1.00 29.66 O

ATOM 2810 HN ASP A 379 4.087 −1.975 −36.063 1.00 0.00 H

ATOM 2811 HA ASP A 379 6.677 −1.513 −35.229 1.00 29.66 H

ATOM 2812 HB1 ASP A 379 4.826 0.165 −36.921 1.00 29.66 H

ATOM 2813 HB2 ASP A 379 6.553 0.542 −36.709 1.00 29.66 H

ATOM 2814 N SER A 380 4.669 0.594 −33.691 1.00 30.51 N

ATOM 2815 CA SER A 380 4.578 1.555 −32.574 1.00 30.65 C

ATOM 2816 C SER A 380 5.143 1.023 −31.244 1.00 30.54 C

ATOM 2817 O SER A 380 5.414 1.814 −30.345 1.00 30.55 O

ATOM	2818	CB	SER	A	380	3.135	2.033	−32.336	1.00	31.03	C
ATOM	2819	OG	SER	A	380	2.353	2.057	−33.518	1.00	31.14	O
ATOM	2820	HN	SER	A	380	3.809	0.241	−34.086	1.00	0.00	H
ATOM	2821	HG	SER	A	380	2.073	1.147	−33.726	1.00	0.00	H
ATOM	2822	HA	SER	A	380	5.168	2.429	−32.850	1.00	30.74	H
ATOM	2823	HB1	SER	A	380	2.661	1.361	−31.621	1.00	30.74	H
ATOM	2824	HB2	SER	A	380	3.168	3.041	−31.922	1.00	30.74	H
ATOM	2825	N	ASN	A	381	5.360	−0.294	−31.109	1.00	30.08	N
ATOM	2826	CA	ASN	A	381	6.080	−0.889	−29.976	1.00	29.95	C
ATOM	2827	C	ASN	A	381	7.609	−0.696	−30.073	1.00	29.61	C
ATOM	2828	O	ASN	A	381	8.325	−1.043	−29.135	1.00	29.48	O
ATOM	2829	CB	ASN	A	381	5.755	−2.398	−29.865	1.00	29.99	C
ATOM	2830	CG	ASN	A	381	4.333	−2.748	−29.452	1.00	30.34	C
ATOM	2831	OD1	ASN	A	381	3.362	−2.050	−29.694	1.00	30.56	O
ATOM	2832	ND2	ASN	A	381	4.150	−3.883	−28.818	1.00	30.43	N
ATOM	2833	HN	ASN	A	381	5.129	−0.897	−31.891	1.00	0.00	H
ATOM	2834	1HD2	ASN	A	381	4.931	−4.471	−28.584	1.00	0.00	H
ATOM	2835	2HD2	ASN	A	381	3.217	−4.090	−28.513	1.00	0.00	H
ATOM	2836	HA	ASN	A	381	5.736	−0.402	−29.064	1.00	30.06	H
ATOM	2837	HB1	ASN	A	381	5.940	−2.851	−30.839	1.00	30.06	H
ATOM	2838	HB2	ASN	A	381	6.432	−2.831	−29.128	1.00	30.06	H
ATOM	2839	N	CYS	A	382	8.142	−0.330	−31.245	1.00	29.47	N
ATOM	2840	CA	CYS	A	382	9.580	−0.286	−31.496	1.00	29.18	C
ATOM	2841	C	CYS	A	382	10.253	0.877	−30.719	1.00	29.19	C
ATOM	2842	O	CYS	A	382	9.856	2.022	−30.939	1.00	29.32	O
ATOM	2843	CB	CYS	A	382	9.796	−0.203	−33.013	1.00	29.06	C
ATOM	2844	SG	CYS	A	382	11.476	−0.555	−33.575	1.00	28.72	S

ATOM	2845	HN	CYS	A	382	7.524	−0.020	−31.989	1.00	0.00	H
ATOM	2846	HA	CYS	A	382	10.013	−1.222	−31.144	1.00	29.16	H
ATOM	2847	HB1	CYS	A	382	9.125	−0.919	−33.488	1.00	29.16	H
ATOM	2848	HB2	CYS	A	382	9.538	0.806	−33.335	1.00	29.16	H
ATOM	2849	N	PRO	A	383	11.278	0.652	−29.859	1.00	54.27	N
ATOM	2850	CA	PRO	A	383	11.807	1.655	−28.903	1.00	54.34	C
ATOM	2851	C	PRO	A	383	12.537	2.911	−29.443	1.00	54.19	C
ATOM	2852	O	PRO	A	383	13.381	3.477	−28.751	1.00	53.96	O
ATOM	2853	CB	PRO	A	383	12.718	0.874	−27.944	1.00	54.27	C
ATOM	2854	CG	PRO	A	383	12.134	−0.530	−27.965	1.00	54.10	C
ATOM	2855	CD	PRO	A	383	11.715	−0.676	−29.422	1.00	54.07	C
ATOM	2856	HA	PRO	A	383	10.960	2.010	−28.316	1.00	54.17	H
ATOM	2857	HB1	PRO	A	383	13.748	0.873	−28.300	1.00	54.17	H
ATOM	2858	HB2	PRO	A	383	12.680	1.297	−26.940	1.00	54.17	H
ATOM	2859	HG1	PRO	A	383	12.882	−1.274	−27.692	1.00	54.17	H
ATOM	2860	HG2	PRO	A	383	11.277	−0.613	−27.296	1.00	54.17	H
ATOM	2861	HD1	PRO	A	383	12.558	−1.013	−30.025	1.00	54.17	H
ATOM	2862	HD2	PRO	A	383	10.896	−1.390	−29.509	1.00	54.17	H
ATOM	2863	N	ILE	A	384	12.220	3.398	−30.642	1.00	33.61	N
ATOM	2864	CA	ILE	A	384	12.958	4.432	−31.394	1.00	33.52	C
ATOM	2865	C	ILE	A	384	12.998	5.847	−30.774	1.00	33.74	C
ATOM	2866	O	ILE	A	384	13.517	6.759	−31.407	1.00	33.73	O
ATOM	2867	CB	ILE	A	384	12.403	4.515	−32.836	1.00	33.48	C
ATOM	2868	CG1	ILE	A	384	10.954	5.064	−32.856	1.00	33.76	C
ATOM	2869	CG2	ILE	A	384	12.518	3.153	−33.544	1.00	33.33	C
ATOM	2870	CD1	ILE	A	384	10.458	5.442	−34.252	1.00	33.73	C
ATOM	2871	HN	ILE	A	384	11.435	2.960	−31.113	1.00	0.00	H

ATOM 2872 HA ILE A 384 13.996 4.105 −31.457 1.00 0.00 H
ATOM 2873 HB ILE A 384 13.026 5.221 −33.385 1.00 33.61 H
ATOM 2874 1HG1 ILE A 384 10.291 4.299 −32.451 1.00 33.61 H
ATOM 2875 2HG1 ILE A 384 10.914 5.952 −32.225 1.00 33.61 H
ATOM 2876 1HG2 ILE A 384 12.122 3.235 −34.556 1.00 33.61 H
ATOM 2877 2HG2 ILE A 384 11.949 2.406 −32.991 1.00 33.61 H
ATOM 2878 3HG2 ILE A 384 13.565 2.853 −33.587 1.00 33.61 H
ATOM 2879 1HD1 ILE A 384 9.437 5.818 −34.186 1.00 33.61 H
ATOM 2880 2HD1 ILE A 384 10.480 4.563 −34.896 1.00 33.61 H
ATOM 2881 3HD1 ILE A 384 11.103 6.215 −34.670 1.00 33.61 H
ATOM 2882 N ILE A 385 12.392 6.090 −29.609 1.00 41.53 N
ATOM 2883 CA ILE A 385 12.001 7.441 −29.145 1.00 41.81 C
ATOM 2884 C ILE A 385 13.188 8.421 −29.027 1.00 41.76 C
ATOM 2885 O ILE A 385 13.058 9.583 −29.402 1.00 41.95 O
ATOM 2886 CB ILE A 385 11.196 7.326 −27.824 1.00 42.09 C
ATOM 2887 CG1 ILE A 385 9.871 6.565 −28.089 1.00 42.20 C
ATOM 2888 CG2 ILE A 385 10.911 8.706 −27.198 1.00 42.42 C
ATOM 2889 CD1 ILE A 385 9.036 6.268 −26.836 1.00 42.55 C
ATOM 2890 HN ILE A 385 12.039 5.288 −29.107 1.00 0.00 H
ATOM 2891 HA ILE A 385 11.324 7.851 −29.895 1.00 42.04 H
ATOM 2892 HB ILE A 385 11.786 6.746 −27.115 1.00 42.04 H
ATOM 2893 1HG1 ILE A 385 9.264 7.167 −28.766 1.00 42.04 H
ATOM 2894 2HG1 ILE A 385 10.115 5.616 −28.566 1.00 42.04 H
ATOM 2895 1HG2 ILE A 385 10.345 8.577 −26.275 1.00 42.04 H
ATOM 2896 2HG2 ILE A 385 10.332 9.310 −27.897 1.00 42.04 H
ATOM 2897 3HG2 ILE A 385 11.854 9.208 −26.979 1.00 42.04 H
ATOM 2898 1HD1 ILE A 385 8.130 5.734 −27.121 1.00 42.04 H

ATOM	2899	2HD1	ILE	A	385	8.767	7.205	−26.348	1.00	42.04	H
ATOM	2900	3HD1	ILE	A	385	9.618	5.654	−26.148	1.00	42.04	H
ATOM	2901	N	HIS	A	386	14.368	7.941	−28.625	1.00	29.46	N
ATOM	2902	CA	HIS	A	386	15.611	8.740	−28.571	1.00	29.42	C
ATOM	2903	C	HIS	A	386	16.465	8.635	−29.851	1.00	29.17	C
ATOM	2904	O	HIS	A	386	17.497	9.294	−29.994	1.00	29.07	O
ATOM	2905	CB	HIS	A	386	16.415	8.312	−27.334	1.00	29.35	C
ATOM	2906	CG	HIS	A	386	15.611	8.358	−26.058	1.00	29.60	C
ATOM	2907	ND1	HIS	A	386	15.133	9.520	−25.451	1.00	29.94	N
ATOM	2908	CD2	HIS	A	386	15.178	7.278	−25.344	1.00	29.60	C
ATOM	2909	CE1	HIS	A	386	14.431	9.118	−24.383	1.00	30.13	C
ATOM	2910	NE2	HIS	A	386	14.440	7.776	−24.292	1.00	29.92	N
ATOM	2911	HN	HIS	A	386	14.411	6.969	−28.362	1.00	0.00	H
ATOM	2912	HE2	HIS	A	386	13.986	7.236	−23.570	1.00	0.00	H
ATOM	2913	HA	HIS	A	386	15.331	9.785	−28.442	1.00	29.57	H
ATOM	2914	HB1	HIS	A	386	16.766	7.291	−27.485	1.00	29.57	H
ATOM	2915	HB2	HIS	A	386	17.270	8.979	−27.229	1.00	29.57	H
ATOM	2916	HD2	HIS	A	386	15.377	6.229	−25.563	1.00	29.57	H
ATOM	2917	HE1	HIS	A	386	13.924	9.785	−23.686	1.00	29.57	H
ATOM	2918	N	CYS	A	387	16.106	7.719	−30.746	1.00	35.54	N
ATOM	2919	CA	CYS	A	387	16.943	7.215	−31.827	1.00	35.30	C
ATOM	2920	C	CYS	A	387	16.730	8.024	−33.109	1.00	35.39	C
ATOM	2921	O	CYS	A	387	15.987	7.623	−33.998	1.00	35.38	O
ATOM	2922	CB	CYS	A	387	16.640	5.727	−31.995	1.00	35.10	C
ATOM	2923	SG	CYS	A	387	16.989	4.785	−30.493	1.00	34.90	S
ATOM	2924	HN	CYS	A	387	15.189	7.300	−30.650	1.00	0.00	H
ATOM	2925	HA	CYS	A	387	17.986	7.319	−31.528	1.00	35.27	H

ATOM 2926 HB1 CYS A 387 15.586 5.610 −32.246 1.00 35.27 H
ATOM 2927 HB2 CYS A 387 17.253 5.334 −32.806 1.00 35.27 H
ATOM 2928 N LYS A 388 17.468 9.137 −33.222 1.00 29.04 N
ATOM 2929 CA LYS A 388 17.288 10.247 −34.189 1.00 29.18 C
ATOM 2930 C LYS A 388 17.003 9.892 −35.668 1.00 29.02 C
ATOM 2931 O LYS A 388 16.627 10.788 −36.418 1.00 29.16 O
ATOM 2932 CB LYS A 388 18.555 11.125 −34.131 1.00 29.19 C
ATOM 2933 CG LYS A 388 18.271 12.622 −34.345 1.00 29.50 C
ATOM 2934 CD LYS A 388 19.472 13.439 −34.853 1.00 29.52 C
ATOM 2935 CE LYS A 388 20.773 13.279 −34.049 1.00 29.35 C
ATOM 2936 NZ LYS A 388 21.578 12.114 −34.500 1.00 29.01 N1+
ATOM 2937 HN LYS A 388 17.979 9.359 −32.376 1.00 0.00 H
ATOM 2938 HZ1 LYS A 388 22.433 12.032 −33.967 1.00 0.00 H
ATOM 2939 HZ2 LYS A 388 21.825 12.216 −35.483 1.00 0.00 H
ATOM 2940 HZ3 LYS A 388 21.054 11.254 −34.407 1.00 0.00 H
ATOM 2941 HA LYS A 388 16.455 10.855 −33.835 1.00 29.22 H
ATOM 2942 HB1 LYS A 388 19.019 10.998 −33.153 1.00 29.22 H
ATOM 2943 HB2 LYS A 388 19.243 10.790 −34.907 1.00 29.22 H
ATOM 2944 HG1 LYS A 388 17.466 12.714 −35.074 1.00 29.22 H
ATOM 2945 HG2 LYS A 388 17.951 13.046 −33.393 1.00 29.22 H
ATOM 2946 HD1 LYS A 388 19.673 13.135 −35.880 1.00 29.22 H
ATOM 2947 HD2 LYS A 388 19.193 14.493 −34.833 1.00 29.22 H
ATOM 2948 HE1 LYS A 388 21.370 14.183 −34.166 1.00 29.22 H
ATOM 2949 HE2 LYS A 388 20.520 13.144 −32.997 1.00 29.22 H
ATOM 2950 N TYR A 389 17.363 8.693 −36.127 1.00 28.75 N
ATOM 2951 CA TYR A 389 17.205 8.242 −37.520 1.00 28.60 C
ATOM 2952 C TYR A 389 16.382 6.947 −37.671 1.00 28.49 C

ATOM	2953	O	TYR	A	389	16.008	6.594	−38.789	1.00	28.40	O
ATOM	2954	CB	TYR	A	389	18.598	8.056	−38.151	1.00	28.39	C
ATOM	2955	CG	TYR	A	389	19.576	9.209	−37.973	1.00	28.48	C
ATOM	2956	CD1	TYR	A	389	19.188	10.530	−38.273	1.00	28.48	C
ATOM	2957	CD2	TYR	A	389	20.883	8.952	−37.513	1.00	28.60	C
ATOM	2958	CE1	TYR	A	389	20.093	11.594	−38.093	1.00	28.59	C
ATOM	2959	CE2	TYR	A	389	21.793	10.011	−37.332	1.00	28.72	C
ATOM	2960	CZ	TYR	A	389	21.396	11.331	−37.624	1.00	28.72	C
ATOM	2961	OH	TYR	A	389	22.249	12.355	−37.363	1.00	28.86	O
ATOM	2962	HN	TYR	A	389	17.625	8.005	−35.440	1.00	0.00	H
ATOM	2963	HH	TYR	A	389	22.741	12.588	−38.160	1.00	0.00	H
ATOM	2964	HA	TYR	A	389	16.691	9.030	−38.070	1.00	28.59	H
ATOM	2965	HB1	TYR	A	389	19.048	7.167	−37.708	1.00	28.59	H
ATOM	2966	HB2	TYR	A	389	18.460	7.899	−39.221	1.00	28.59	H
ATOM	2967	HD1	TYR	A	389	18.183	10.729	−38.646	1.00	28.59	H
ATOM	2968	HD2	TYR	A	389	21.191	7.929	−37.296	1.00	28.59	H
ATOM	2969	HE1	TYR	A	389	19.787	12.616	−38.315	1.00	28.59	H
ATOM	2970	HE2	TYR	A	389	22.800	9.811	−36.967	1.00	28.59	H
ATOM	2971	N	SER	A	390	16.131	6.226	−36.576	1.00	28.50	N
ATOM	2972	CA	SER	A	390	15.590	4.867	−36.597	1.00	28.40	C
ATOM	2973	C	SER	A	390	14.095	4.848	−36.898	1.00	28.59	C
ATOM	2974	O	SER	A	390	13.304	5.553	−36.270	1.00	28.81	O
ATOM	2975	CB	SER	A	390	15.873	4.168	−35.268	1.00	28.34	C
ATOM	2976	OG	SER	A	390	17.270	4.118	−35.053	1.00	28.20	O
ATOM	2977	HN	SER	A	390	16.345	6.610	−35.666	1.00	0.00	H
ATOM	2978	HG	SER	A	390	17.654	3.627	−35.826	1.00	0.00	H
ATOM	2979	HA	SER	A	390	16.100	4.313	−37.385	1.00	28.47	H

ATOM	2980	HB1	SER	A	390	15.400	4.722	−34.457	1.00	28.47	H
ATOM	2981	HB2	SER	A	390	15.473	3.154	−35.298	1.00	28.47	H
ATOM	2982	N	LYS	A	391	13.677	3.990	−37.832	1.00	28.50	N
ATOM	2983	CA	LYS	A	391	12.264	3.831	−38.198	1.00	28.66	C
ATOM	2984	C	LYS	A	391	11.568	2.893	−37.217	1.00	28.73	C
ATOM	2985	O	LYS	A	391	12.111	1.845	−36.883	1.00	28.63	O
ATOM	2986	CB	LYS	A	391	12.112	3.321	−39.640	1.00	28.55	C
ATOM	2987	CG	LYS	A	391	12.939	4.121	−40.663	1.00	28.49	C
ATOM	2988	CD	LYS	A	391	12.654	3.665	−42.099	1.00	28.44	C
ATOM	2989	CE	LYS	A	391	11.297	4.189	−42.584	1.00	28.65	C
ATOM	2990	NZ	LYS	A	391	10.905	3.574	−43.873	1.00	28.62	N1+
ATOM	2991	HN	LYS	A	391	14.360	3.343	−38.214	1.00	0.00	H
ATOM	2992	HZ1	LYS	A	391	10.055	3.980	−44.234	1.00	0.00	H
ATOM	2993	HZ2	LYS	A	391	11.641	3.610	−44.559	1.00	0.00	H
ATOM	2994	HZ3	LYS	A	391	10.650	2.590	−43.716	1.00	0.00	H
ATOM	2995	HA	LYS	A	391	11.785	4.808	−38.131	1.00	28.59	H
ATOM	2996	HB1	LYS	A	391	12.434	2.280	−39.674	1.00	28.59	H
ATOM	2997	HB2	LYS	A	391	11.061	3.386	−39.920	1.00	28.59	H
ATOM	2998	HG1	LYS	A	391	12.689	5.178	−40.571	1.00	28.59	H
ATOM	2999	HG2	LYS	A	391	13.998	3.978	−40.450	1.00	28.59	H
ATOM	3000	HD1	LYS	A	391	13.437	4.044	−42.755	1.00	28.59	H
ATOM	3001	HD2	LYS	A	391	12.648	2.576	−42.132	1.00	28.59	H
ATOM	3002	HE1	LYS	A	391	10.540	3.955	−41.836	1.00	28.59	H
ATOM	3003	HE2	LYS	A	391	11.360	5.270	−42.712	1.00	28.59	H
ATOM	3004	N	ALA	A	392	10.265	3.094	−37.036	1.00	38.67	N
ATOM	3005	CA	ALA	A	392	9.406	2.165	−36.294	1.00	38.80	C
ATOM	3006	C	ALA	A	392	9.332	0.763	−36.949	1.00	38.67	C

ATOM	3007	O	ALA	A	392	9.303	−0.252	−36.256	1.00	38.68	O
ATOM	3008	CB	ALA	A	392	8.033	2.837	−36.170	1.00	39.08	C
ATOM	3009	HN	ALA	A	392	9.870	3.969	−37.334	1.00	0.00	H
ATOM	3010	HA	ALA	A	392	9.818	2.049	−35.292	1.00	38.78	H
ATOM	3011	HB1	ALA	A	392	7.355	2.182	−35.623	1.00	38.78	H
ATOM	3012	HB2	ALA	A	392	7.630	3.027	−37.165	1.00	38.78	H
ATOM	3013	HB3	ALA	A	392	8.137	3.781	−35.634	1.00	38.78	H
ATOM	3014	N	GLU	A	393	9.560	0.701	−38.267	1.00	32.27	N
ATOM	3015	CA	GLU	A	393	9.720	−0.543	−39.029	1.00	32.19	C
ATOM	3016	C	GLU	A	393	10.927	−1.393	−38.601	1.00	31.99	C
ATOM	3017	O	GLU	A	393	10.941	−2.596	−38.855	1.00	31.95	O
ATOM	3018	CB	GLU	A	393	9.937	−0.226	−40.516	1.00	32.12	C
ATOM	3019	CG	GLU	A	393	8.928	0.727	−41.165	1.00	32.18	C
ATOM	3020	CD	GLU	A	393	8.927	0.524	−42.685	1.00	31.98	C
ATOM	3021	OE1	GLU	A	393	8.488	−0.569	−43.125	1.00	31.89	O
ATOM	3022	OE2	GLU	A	393	9.553	1.363	−43.371	1.00	31.92	O
ATOM	3023	HN	GLU	A	393	9.592	1.566	−38.776	1.00	0.00	H
ATOM	3024	HA	GLU	A	393	8.814	−1.140	−38.924	1.00	32.05	H
ATOM	3025	HB1	GLU	A	393	10.927	0.219	−40.619	1.00	32.05	H
ATOM	3026	HB2	GLU	A	393	9.903	−1.168	−41.064	1.00	32.05	H
ATOM	3027	HG1	GLU	A	393	7.932	0.522	−40.772	1.00	32.05	H
ATOM	3028	HG2	GLU	A	393	9.204	1.757	−40.938	1.00	32.05	H
ATOM	3029	N	ASN	A	394	11.999	−0.778	−38.087	1.00	32.64	N
ATOM	3030	CA	ASN	A	394	13.317	−1.412	−37.982	1.00	32.44	C
ATOM	3031	C	ASN	A	394	13.295	−2.685	−37.126	1.00	32.45	C
ATOM	3032	O	ASN	A	394	13.847	−3.702	−37.547	1.00	32.34	O
ATOM	3033	CB	ASN	A	394	14.334	−0.401	−37.431	1.00	32.41	C

ATOM 3034 CG ASN A 394 14.736 0.694 −38.408 1.00 32.39 C
ATOM 3035 OD1 ASN A 394 14.377 0.694 −39.577 1.00 32.47 O
ATOM 3036 ND2 ASN A 394 15.578 1.597 −37.971 1.00 32.30 N
ATOM 3037 HN ASN A 394 11.936 0.209 −37.866 1.00 0.00 H
ATOM 3038 1HD2 ASN A 394 15.926 1.499 −37.018 1.00 0.00 H
ATOM 3039 2HD2 ASN A 394 16.283 1.933 −38.623 1.00 0.00 H
ATOM 3040 HA ASN A 394 13.634 −1.691 −38.987 1.00 32.43 H
ATOM 3041 HB1 ASN A 394 13.899 0.073 −36.551 1.00 32.43 H
ATOM 3042 HB2 ASN A 394 15.233 −0.946 −37.144 1.00 32.43 H
ATOM 3043 N CYS A 395 12.455 −2.704 −36.084 1.00 31.44 N
ATOM 3044 CA CYS A 395 12.134 −3.904 −35.318 1.00 31.44 C
ATOM 3045 C CYS A 395 11.551 −5.013 −36.212 1.00 31.49 C
ATOM 3046 O CYS A 395 12.182 −6.054 −36.387 1.00 31.38 O
ATOM 3047 CB CYS A 395 11.164 −3.548 −34.182 1.00 31.64 C
ATOM 3048 SG CYS A 395 11.826 −2.451 −32.904 1.00 31.62 S
ATOM 3049 HN CYS A 395 11.987 −1.841 −35.843 1.00 0.00 H
ATOM 3050 HA CYS A 395 13.056 −4.278 −34.872 1.00 31.50 H
ATOM 3051 HB1 CYS A 395 10.294 −3.062 −34.624 1.00 31.50 H
ATOM 3052 HB2 CYS A 395 10.856 −4.476 −33.701 1.00 31.50 H
ATOM 3053 N ARG A 396 10.420 −4.751 −36.882 1.00 28.58 N
ATOM 3054 CA ARG A 396 9.716 −5.702 −37.763 1.00 28.67 C
ATOM 3055 C ARG A 396 10.585 −6.213 −38.915 1.00 28.50 C
ATOM 3056 O ARG A 396 10.500 −7.387 −39.284 1.00 28.50 O
ATOM 3057 CB ARG A 396 8.444 −5.004 −38.281 1.00 28.89 C
ATOM 3058 CG ARG A 396 7.640 −5.814 −39.316 1.00 29.00 C
ATOM 3059 CD ARG A 396 6.301 −5.154 −39.681 1.00 29.22 C
ATOM 3060 NE ARG A 396 6.446 −3.741 −40.065 1.00 29.17 N

ATOM	3061	CZ	ARG	A	396	6.895	−3.205	−41.179	1.00	29.02	C
ATOM	3062	NH1	ARG	A	396	7.261	−3.903	−42.212	1.00	28.90	N
ATOM	3063	NH2	ARG	A	396	6.977	−1.924	−41.281	1.00	29.01	N
ATOM	3064	HN	ARG	A	396	10.031	−3.820	−36.798	1.00	0.00	H
ATOM	3065	HE	ARG	A	396	6.292	−3.071	−39.304	1.00	0.00	H
ATOM	3066	1HH1	ARG	A	396	7.090	−4.884	−42.203	1.00	0.00	H
ATOM	3067	2HH1	ARG	A	396	7.563	−3.401	−43.027	1.00	0.00	H
ATOM	3068	1HH2	ARG	A	396	6.688	−1.368	−40.471	1.00	0.00	H
ATOM	3069	2HH2	ARG	A	396	7.436	−1.455	−42.061	1.00	0.00	H
ATOM	3070	HA	ARG	A	396	9.412	−6.559	−37.162	1.00	28.86	H
ATOM	3071	HB1	ARG	A	396	7.795	−4.805	−37.428	1.00	28.86	H
ATOM	3072	HB2	ARG	A	396	8.738	−4.061	−38.742	1.00	28.86	H
ATOM	3073	HG1	ARG	A	396	8.238	−5.912	−40.222	1.00	28.86	H
ATOM	3074	HG2	ARG	A	396	7.439	−6.803	−38.904	1.00	28.86	H
ATOM	3075	HD1	ARG	A	396	5.861	−5.698	−40.517	1.00	28.86	H
ATOM	3076	HD2	ARG	A	396	5.637	−5.214	−38.818	1.00	28.86	H
ATOM	3077	N	LEU	A	397	11.412	−5.347	−39.495	1.00	28.38	N
ATOM	3078	CA	LEU	A	397	12.327	−5.696	−40.581	1.00	28.20	C
ATOM	3079	C	LEU	A	397	13.477	−6.595	−40.095	1.00	28.06	C
ATOM	3080	O	LEU	A	397	13.667	−7.663	−40.681	1.00	28.01	O
ATOM	3081	CB	LEU	A	397	12.843	−4.406	−41.245	1.00	28.09	C
ATOM	3082	CG	LEU	A	397	11.758	−3.560	−41.946	1.00	28.20	C
ATOM	3083	CD1	LEU	A	397	12.386	−2.273	−42.480	1.00	28.15	C
ATOM	3084	CD2	LEU	A	397	11.077	−4.288	−43.108	1.00	28.17	C
ATOM	3085	HN	LEU	A	397	11.393	−4.387	−39.158	1.00	0.00	H
ATOM	3086	HA	LEU	A	397	11.761	−6.253	−41.328	1.00	28.16	H
ATOM	3087	HB1	LEU	A	397	13.308	−3.791	−40.475	1.00	28.16	H

ATOM 3088 HB2 LEU A 397 13.590 −4.683 −41.989 1.00 28.16 H

ATOM 3089 HG LEU A 397 10.998 −3.295 −41.211 1.00 28.16 H

ATOM 3090 1HD1 LEU A 397 11.622 −1.674 −42.975 1.00 28.16 H

ATOM 3091 2HD1 LEU A 397 13.172 −2.521 −43.193 1.00 28.16 H

ATOM 3092 3HD1 LEU A 397 12.812 −1.706 −41.653 1.00 28.16 H

ATOM 3093 1HD2 LEU A 397 10.326 −3.637 −43.555 1.00 28.16 H

ATOM 3094 2HD2 LEU A 397 10.597 −5.194 −42.738 1.00 28.16 H

ATOM 3095 3HD2 LEU A 397 11.822 −4.552 −43.859 1.00 28.16 H

ATOM 3096 N SER A 398 14.107 −6.260 −38.960 1.00 29.91 N

ATOM 3097 CA SER A 398 15.251 −6.986 −38.378 1.00 29.76 C

ATOM 3098 C SER A 398 14.941 −8.426 −37.954 1.00 29.83 C

ATOM 3099 O SER A 398 15.838 −9.256 −38.020 1.00 29.71 O

ATOM 3100 CB SER A 398 15.818 −6.197 −37.185 1.00 29.68 C

ATOM 3101 OG SER A 398 16.835 −6.905 −36.489 1.00 29.55 O

ATOM 3102 HN SER A 398 13.827 −5.400 −38.494 1.00 0.00 H

ATOM 3103 HG SER A 398 17.565 −7.113 −37.107 1.00 0.00 H

ATOM 3104 HA SER A 398 16.030 −7.031 −39.139 1.00 29.74 H

ATOM 3105 HB1 SER A 398 16.236 −5.261 −37.554 1.00 29.74 H

ATOM 3106 HB2 SER A 398 15.005 −5.984 −36.491 1.00 29.74 H

ATOM 3107 N MET A 399 13.676 −8.795 −37.707 1.00 28.13 N

ATOM 3108 CA MET A 399 13.304 −10.161 −37.282 1.00 28.23 C

ATOM 3109 C MET A 399 13.583 −11.284 −38.308 1.00 28.22 C

ATOM 3110 O MET A 399 13.324 −12.448 −38.010 1.00 28.30 O

ATOM 3111 CB MET A 399 11.833 −10.229 −36.831 1.00 28.49 C

ATOM 3112 CG MET A 399 11.420 −9.198 −35.781 1.00 28.56 C

ATOM 3113 SD MET A 399 12.495 −9.055 −34.327 1.00 28.44 S

ATOM 3114 CE MET A 399 11.635 −7.723 −33.449 1.00 28.56 C

ATOM 3115 HN MET A 399 12.984 −8.061 −37.632 1.00 0.00 H

ATOM 3116 HA MET A 399 13.908 −10.387 −36.403 1.00 28.37 H

ATOM 3117 HB1 MET A 399 11.205 −10.083 −37.710 1.00 28.37 H

ATOM 3118 HB2 MET A 399 11.654 −11.221 −36.416 1.00 28.37 H

ATOM 3119 HG1 MET A 399 11.389 −8.223 −36.268 1.00 28.37 H

ATOM 3120 HG2 MET A 399 10.422 −9.462 −35.431 1.00 28.37 H

ATOM 3121 HE1 MET A 399 12.161 −7.500 −32.521 1.00 28.37 H

ATOM 3122 HE2 MET A 399 10.616 −8.036 −33.222 1.00 28.37 H

ATOM 3123 HE3 MET A 399 11.610 −6.831 −34.075 1.00 28.37 H

ATOM 3124 N GLY A 400 14.090 −10.986 −39.509 1.00 28.13 N

ATOM 3125 CA GLY A 400 14.535 −11.989 −40.482 1.00 28.11 C

ATOM 3126 C GLY A 400 15.872 −11.627 −41.134 1.00 27.89 C

ATOM 3127 O GLY A 400 16.190 −10.452 −41.288 1.00 27.75 O

ATOM 3128 HN GLY A 400 14.437 −10.043 −39.637 1.00 0.00 H

ATOM 3129 HA1 GLY A 400 14.644 −12.946 −39.971 1.00 27.97 H

ATOM 3130 HA2 GLY A 400 13.779 −12.077 −41.262 1.00 27.97 H

ATOM 3131 N VAL A 401 16.570 −12.642 −41.660 1.00 27.88 N

ATOM 3132 CA VAL A 401 17.931 −12.569 −42.254 1.00 27.71 C

ATOM 3133 C VAL A 401 18.082 −11.520 −43.373 1.00 27.63 C

ATOM 3134 O VAL A 401 19.172 −11.009 −43.615 1.00 27.49 O

ATOM 3135 CB VAL A 401 18.325 −13.981 −42.755 1.00 27.78 C

ATOM 3136 CG1 VAL A 401 19.585 −14.037 −43.628 1.00 27.64 C

ATOM 3137 CG2 VAL A 401 18.532 −14.928 −41.566 1.00 27.85 C

ATOM 3138 HN VAL A 401 16.219 −13.569 −41.473 1.00 0.00 H

ATOM 3139 HA VAL A 401 18.623 −12.299 −41.456 1.00 27.71 H

ATOM 3140 HB VAL A 401 17.494 −14.367 −43.346 1.00 27.71 H

ATOM 3141 1HG1 VAL A 401 19.775 −15.068 −43.927 1.00 27.71 H

ATOM 3142 2HG1 VAL A 401 20.437 −13.660 −43.062 1.00 27.71 H

ATOM 3143 3HG1 VAL A 401 19.440 −13.423 −44.517 1.00 27.71 H

ATOM 3144 1HG2 VAL A 401 18.808 −15.917 −41.932 1.00 27.71 H

ATOM 3145 2HG2 VAL A 401 17.608 −14.999 −40.992 1.00 27.71 H

ATOM 3146 3HG2 VAL A 401 19.327 −14.542 −40.928 1.00 27.71 H

ATOM 3147 N ASN A 402 16.979 −11.096 −43.993 1.00 27.75 N

ATOM 3148 CA ASN A 402 16.870 −9.793 −44.649 1.00 27.71 C

ATOM 3149 C ASN A 402 15.631 −9.063 −44.100 1.00 27.82 C

ATOM 3150 O ASN A 402 14.590 −9.697 −43.925 1.00 27.97 O

ATOM 3151 CB ASN A 402 16.735 −9.944 −46.179 1.00 27.78 C

ATOM 3152 CG ASN A 402 17.873 −10.554 −46.982 1.00 27.74 C

ATOM 3153 OD1 ASN A 402 17.722 −10.733 −48.179 1.00 27.60 O

ATOM 3154 ND2 ASN A 402 19.032 −10.814 −46.430 1.00 27.89 N

ATOM 3155 HN ASN A 402 16.115 −11.462 −43.623 1.00 0.00 H

ATOM 3156 HB1 ASN A 402 15.855 −10.562 −46.359 1.00 0.00 H

ATOM 3157 1HD2 ASN A 402 19.178 −10.720 −45.428 1.00 0.00 H

ATOM 3158 2HD2 ASN A 402 19.747 −11.200 −47.019 1.00 0.00 H

ATOM 3159 HA ASN A 402 17.760 −9.205 −44.425 1.00 27.78 H

ATOM 3160 HB2 ASN A 402 16.566 −8.945 −46.581 1.00 27.78 H

ATOM 3161 N SER A 403 15.602 −7.739 −44.269 1.00 27.76 N

ATOM 3162 CA SER A 403 14.370 −6.929 −44.220 1.00 27.88 C

ATOM 3163 C SER A 403 13.289 −7.416 −45.202 1.00 28.04 C

ATOM 3164 O SER A 403 12.124 −7.542 −44.833 1.00 28.19 O

ATOM 3165 CB SER A 403 14.743 −5.479 −44.546 1.00 27.82 C

ATOM 3166 OG SER A 403 15.415 −5.410 −45.795 1.00 27.78 O

ATOM 3167 HN SER A 403 16.455 −7.263 −44.524 1.00 0.00 H

ATOM 3168 HG SER A 403 15.723 −4.504 −45.923 1.00 0.00 H

ATOM	3169	HA	SER	A	403	13.966	−6.967	−43.208	1.00	27.91	H
ATOM	3170	HB1	SER	A	403	13.836	−4.877	−44.593	1.00	27.91	H
ATOM	3171	HB2	SER	A	403	15.397	−5.092	−43.765	1.00	27.91	H
ATOM	3172	N	LYS	A	404	13.710	−7.847	−46.400	1.00	28.00	N
ATOM	3173	CA	LYS	A	404	12.878	−8.440	−47.465	1.00	28.14	C
ATOM	3174	C	LYS	A	404	12.785	−9.978	−47.448	1.00	28.29	C
ATOM	3175	O	LYS	A	404	12.532	−10.576	−48.493	1.00	28.45	O
ATOM	3176	CB	LYS	A	404	13.310	−7.858	−48.829	1.00	28.05	C
ATOM	3177	CG	LYS	A	404	14.747	−8.213	−49.280	1.00	27.95	C
ATOM	3178	CD	LYS	A	404	14.869	−9.300	−50.361	1.00	28.03	C
ATOM	3179	CE	LYS	A	404	14.234	−8.861	−51.688	1.00	27.98	C
ATOM	3180	NZ	LYS	A	404	14.296	−9.943	−52.701	1.00	27.94	N1+
ATOM	3181	HN	LYS	A	404	14.678	−7.649	−46.607	1.00	0.00	H
ATOM	3182	HZ1	LYS	A	404	13.897	−9.633	−53.578	1.00	0.00	H
ATOM	3183	HZ2	LYS	A	404	13.776	−10.749	−52.377	1.00	0.00	H
ATOM	3184	HZ3	LYS	A	404	15.256	−10.223	−52.859	1.00	0.00	H
ATOM	3185	HA	LYS	A	404	11.865	−8.077	−47.291	1.00	28.09	H
ATOM	3186	HB1	LYS	A	404	12.619	−8.229	−49.586	1.00	28.09	H
ATOM	3187	HB2	LYS	A	404	13.236	−6.772	−48.769	1.00	28.09	H
ATOM	3188	HG1	LYS	A	404	15.208	−7.305	−49.668	1.00	28.09	H
ATOM	3189	HG2	LYS	A	404	15.297	−8.553	−48.402	1.00	28.09	H
ATOM	3190	HD1	LYS	A	404	15.925	−9.513	−50.529	1.00	28.09	H
ATOM	3191	HD2	LYS	A	404	14.367	−10.202	−50.012	1.00	28.09	H
ATOM	3192	HE1	LYS	A	404	13.191	−8.599	−51.512	1.00	28.09	H
ATOM	3193	HE2	LYS	A	404	14.769	−7.990	−52.066	1.00	28.09	H
ATOM	3194	N	SER	A	405	13.146	−10.635	−46.342	1.00	28.23	N
ATOM	3195	CA	SER	A	405	13.092	−12.102	−46.225	1.00	28.36	C

ATOM	3196	C	SER	A	405	11.665	−12.640	−46.380	1.00	28.62	C
ATOM	3197	O	SER	A	405	10.704	−11.955	−46.041	1.00	28.71	O
ATOM	3198	CB	SER	A	405	13.662	−12.563	−44.878	1.00	28.30	C
ATOM	3199	OG	SER	A	405	12.888	−12.063	−43.806	1.00	28.39	O
ATOM	3200	HN	SER	A	405	13.349	−10.104	−45.504	1.00	0.00	H
ATOM	3201	HG	SER	A	405	12.640	−12.825	−43.233	1.00	0.00	H
ATOM	3202	HA	SER	A	405	13.705	−12.530	−47.018	1.00	28.43	H
ATOM	3203	HB1	SER	A	405	13.659	−13.652	−44.843	1.00	28.43	H
ATOM	3204	HB2	SER	A	405	14.685	−12.199	−44.780	1.00	28.43	H
ATOM	3205	N	HIS	A	406	11.546	−13.915	−46.762	1.00	28.77	N
ATOM	3206	CA	HIS	A	406	10.296	−14.679	−46.657	1.00	29.05	C
ATOM	3207	C	HIS	A	406	9.943	−14.948	−45.184	1.00	29.17	C
ATOM	3208	O	HIS	A	406	9.006	−14.345	−44.672	1.00	29.36	O
ATOM	3209	CB	HIS	A	406	10.432	−15.959	−47.501	1.00	29.22	C
ATOM	3210	CG	HIS	A	406	9.279	−16.924	−47.405	1.00	29.54	C
ATOM	3211	ND1	HIS	A	406	8.969	−17.668	−46.272	1.00	29.74	N
ATOM	3212	CD2	HIS	A	406	8.496	−17.350	−48.439	1.00	29.72	C
ATOM	3213	CE1	HIS	A	406	8.020	−18.540	−46.644	1.00	30.04	C
ATOM	3214	NE2	HIS	A	406	7.702	−18.357	−47.935	1.00	30.03	N
ATOM	3215	HN	HIS	A	406	12.379	−14.419	−47.019	1.00	0.00	H
ATOM	3216	HE2	HIS	A	406	7.050	−18.931	−48.453	1.00	0.00	H
ATOM	3217	HA	HIS	A	406	9.497	−14.076	−47.089	1.00	29.46	H
ATOM	3218	HB1	HIS	A	406	10.536	−15.663	−48.545	1.00	29.46	H
ATOM	3219	HB2	HIS	A	406	11.334	−16.480	−47.178	1.00	29.46	H
ATOM	3220	HD2	HIS	A	406	8.498	−16.969	−49.460	1.00	29.46	H
ATOM	3221	HE1	HIS	A	406	7.572	−19.289	−45.992	1.00	29.46	H
ATOM	3222	N	TYR	A	407	10.840	−15.602	−44.441	1.00	29.08	N

ATOM	3223	CA	TYR	A	407	10.692	−15.844	−43.002	1.00	29.17	C
ATOM	3224	C	TYR	A	407	11.130	−14.660	−42.128	1.00	28.96	C
ATOM	3225	O	TYR	A	407	12.218	−14.113	−42.338	1.00	28.74	O
ATOM	3226	CB	TYR	A	407	11.478	−17.109	−42.624	1.00	29.25	C
ATOM	3227	CG	TYR	A	407	10.986	−18.339	−43.352	1.00	29.54	C
ATOM	3228	CD1	TYR	A	407	9.708	−18.833	−43.049	1.00	29.82	C
ATOM	3229	CD2	TYR	A	407	11.751	−18.941	−44.369	1.00	29.57	C
ATOM	3230	CE1	TYR	A	407	9.187	−19.924	−43.756	1.00	30.10	C
ATOM	3231	CE2	TYR	A	407	11.227	−20.040	−45.080	1.00	29.87	C
ATOM	3232	CZ	TYR	A	407	9.943	−20.535	−44.775	1.00	30.13	C
ATOM	3233	OH	TYR	A	407	9.435	−21.585	−45.473	1.00	30.46	O
ATOM	3234	HN	TYR	A	407	11.551	−16.125	−44.922	1.00	0.00	H
ATOM	3235	HH	TYR	A	407	8.488	−21.742	−45.240	1.00	0.00	H
ATOM	3236	HA	TYR	A	407	9.637	−16.034	−42.802	1.00	29.56	H
ATOM	3237	HB1	TYR	A	407	12.528	−16.954	−42.871	1.00	29.56	H
ATOM	3238	HB2	TYR	A	407	11.377	−17.275	−41.551	1.00	29.56	H
ATOM	3239	HD1	TYR	A	407	9.118	−18.366	−42.260	1.00	29.56	H
ATOM	3240	HD2	TYR	A	407	12.744	−18.559	−44.606	1.00	29.56	H
ATOM	3241	HE1	TYR	A	407	8.193	−20.302	−43.517	1.00	29.56	H
ATOM	3242	HE2	TYR	A	407	11.817	−20.508	−45.868	1.00	29.56	H
ATOM	3243	N	ILE	A	408	10.432	−14.453	−41.006	1.00	29.04	N
ATOM	3244	CA	ILE	A	408	11.074	−14.082	−39.735	1.00	28.85	C
ATOM	3245	C	ILE	A	408	11.494	−15.341	−38.970	1.00	28.90	C
ATOM	3246	O	ILE	A	408	10.919	−16.417	−39.154	1.00	29.12	O
ATOM	3247	CB	ILE	A	408	10.209	−13.143	−38.859	1.00	28.90	C
ATOM	3248	CG1	ILE	A	408	8.884	−13.727	−38.317	1.00	29.19	C
ATOM	3249	CG2	ILE	A	408	9.940	−11.835	−39.617	1.00	28.83	C

ATOM 3250 CD1 ILE A 408 9.015 −14.513 −37.004 1.00 29.25 C

ATOM 3251 HN ILE A 408 9.570 −14.983 −40.911 1.00 0.00 H

ATOM 3252 HA ILE A 408 11.986 −13.539 −39.983 1.00 29.01 H

ATOM 3253 HB ILE A 408 10.813 −12.882 −37.990 1.00 29.01 H

ATOM 3254 1HG1 ILE A 408 8.193 −12.901 −38.150 1.00 29.01 H

ATOM 3255 2HG1 ILE A 408 8.474 −14.397 −39.073 1.00 29.01 H

ATOM 3256 1HG2 ILE A 408 9.331 −11.175 −38.999 1.00 29.01 H

ATOM 3257 2HG2 ILE A 408 9.411 −12.054 −40.544 1.00 29.01 H

ATOM 3258 3HG2 ILE A 408 10.887 −11.346 −39.846 1.00 29.01 H

ATOM 3259 1HD1 ILE A 408 8.035 −14.884 −36.702 1.00 29.01 H

ATOM 3260 2HD1 ILE A 408 9.411 −13.859 −36.227 1.00 29.01 H

ATOM 3261 3HD1 ILE A 408 9.692 −15.355 −37.150 1.00 29.01 H

ATOM 3262 N LEU A 409 12.480 −15.180 −38.090 1.00 28.70 N

ATOM 3263 CA LEU A 409 12.918 −16.181 −37.123 1.00 28.69 C

ATOM 3264 C LEU A 409 12.405 −15.803 −35.724 1.00 28.72 C

ATOM 3265 O LEU A 409 12.503 −14.639 −35.333 1.00 28.54 O

ATOM 3266 CB LEU A 409 14.457 −16.280 −37.145 1.00 28.44 C

ATOM 3267 CG LEU A 409 15.110 −16.514 −38.519 1.00 28.40 C

ATOM 3268 CD1 LEU A 409 16.625 −16.636 −38.356 1.00 28.20 C

ATOM 3269 CD2 LEU A 409 14.604 −17.787 −39.189 1.00 28.67 C

ATOM 3270 HN LEU A 409 12.833 −14.237 −37.957 1.00 0.00 H

ATOM 3271 HA LEU A 409 12.499 −17.148 −37.402 1.00 28.54 H

ATOM 3272 HB1 LEU A 409 14.855 −15.348 −36.744 1.00 28.54 H

ATOM 3273 HB2 LEU A 409 14.745 −17.107 −36.496 1.00 28.54 H

ATOM 3274 HG LEU A 409 14.891 −15.663 −39.164 1.00 28.54 H

ATOM 3275 1HD1 LEU A 409 17.084 −16.802 −39.331 1.00 28.54 H

ATOM 3276 2HD1 LEU A 409 16.854 −17.476 −37.700 1.00 28.54 H

ATOM	3277	3HD1	LEU	A	409	17.019	−15.718	−37.921	1.00	28.54	H
ATOM	3278	1HD2	LEU	A	409	15.094	−17.909	−40.155	1.00	28.54	H
ATOM	3279	2HD2	LEU	A	409	13.526	−17.718	−39.335	1.00	28.54	H
ATOM	3280	3HD2	LEU	A	409	14.830	−18.646	−38.557	1.00	28.54	H
ATOM	3281	N	ARG	A	410	12.060	−16.798	−34.903	1.00	28.95	N
ATOM	3282	CA	ARG	A	410	11.633	−16.638	−33.499	1.00	29.00	C
ATOM	3283	C	ARG	A	410	12.342	−17.667	−32.618	1.00	28.96	C
ATOM	3284	O	ARG	A	410	12.143	−18.859	−32.825	1.00	29.08	O
ATOM	3285	CB	ARG	A	410	10.104	−16.832	−33.447	1.00	29.32	C
ATOM	3286	CG	ARG	A	410	9.463	−16.556	−32.075	1.00	29.43	C
ATOM	3287	CD	ARG	A	410	9.222	−15.060	−31.837	1.00	29.22	C
ATOM	3288	NE	ARG	A	410	8.336	−14.813	−30.682	1.00	29.41	N
ATOM	3289	CZ	ARG	A	410	7.015	−14.863	−30.656	1.00	29.65	C
ATOM	3290	NH1	ARG	A	410	6.290	−15.248	−31.659	1.00	29.72	N
ATOM	3291	NH2	ARG	A	410	6.388	−14.676	−29.541	1.00	29.82	N
ATOM	3292	HN	ARG	A	410	11.967	−17.724	−35.318	1.00	0.00	H
ATOM	3293	HE	ARG	A	410	8.762	−14.671	−29.773	1.00	0.00	H
ATOM	3294	1HH1	ARG	A	410	6.741	−15.705	−32.430	1.00	0.00	H
ATOM	3295	2HH1	ARG	A	410	5.372	−15.615	−31.393	1.00	0.00	H
ATOM	3296	1HH2	ARG	A	410	6.952	−14.687	−28.702	1.00	0.00	H
ATOM	3297	2HH2	ARG	A	410	5.451	−15.085	−29.498	1.00	0.00	H
ATOM	3298	HA	ARG	A	410	11.883	−15.634	−33.156	1.00	29.32	H
ATOM	3299	HB1	ARG	A	410	9.652	−16.157	−34.174	1.00	29.32	H
ATOM	3300	HB2	ARG	A	410	9.884	−17.863	−33.722	1.00	29.32	H
ATOM	3301	HG1	ARG	A	410	8.507	−17.078	−32.023	1.00	29.32	H
ATOM	3302	HG2	ARG	A	410	10.126	−16.933	−31.296	1.00	29.32	H
ATOM	3303	HD1	ARG	A	410	10.181	−14.575	−31.653	1.00	29.32	H

ATOM 3304 HD2 ARG A 410 8.763 −14.633 −32.729 1.00 29.32 H

ATOM 3305 N SER A 411 13.165 −17.241 −31.656 1.00 28.78 N

ATOM 3306 CA SER A 411 13.763 −18.166 −30.674 1.00 28.71 C

ATOM 3307 C SER A 411 12.689 −18.807 −29.784 1.00 28.96 C

ATOM 3308 O SER A 411 11.583 −18.282 −29.643 1.00 29.14 O

ATOM 3309 CB SER A 411 14.818 −17.475 −29.799 1.00 28.40 C

ATOM 3310 OG SER A 411 15.889 −16.997 −30.590 1.00 28.18 O

ATOM 3311 HN SER A 411 13.305 −16.253 −31.525 1.00 0.00 H

ATOM 3312 HG SER A 411 16.656 −17.591 −30.482 1.00 0.00 H

ATOM 3313 HA SER A 411 14.259 −18.964 −31.226 1.00 28.69 H

ATOM 3314 HB1 SER A 411 14.357 −16.636 −29.278 1.00 28.69 H

ATOM 3315 HB2 SER A 411 15.202 −18.189 −29.071 1.00 28.69 H

ATOM 3316 N GLY A 412 13.016 −19.934 −29.160 1.00 28.98 N

ATOM 3317 CA GLY A 412 12.067 −20.702 −28.365 1.00 29.24 C

ATOM 3318 C GLY A 412 12.660 −21.996 −27.815 1.00 29.23 C

ATOM 3319 O GLY A 412 13.880 −22.133 −27.698 1.00 28.98 O

ATOM 3320 HN GLY A 412 13.903 −20.379 −29.382 1.00 0.00 H

ATOM 3321 HA1 GLY A 412 11.738 −20.087 −27.527 1.00 29.11 H

ATOM 3322 HA2 GLY A 412 11.211 −20.951 −28.992 1.00 29.11 H

ATOM 3323 N LEU A 413 11.789 −22.969 −27.558 1.00 29.53 N

ATOM 3324 CA LEU A 413 12.137 −24.318 −27.113 1.00 29.59 C

ATOM 3325 C LEU A 413 11.505 −25.368 −28.032 1.00 29.95 C

ATOM 3326 O LEU A 413 10.344 −25.238 −28.415 1.00 30.22 O

ATOM 3327 CB LEU A 413 11.678 −24.547 −25.659 1.00 29.66 C

ATOM 3328 CG LEU A 413 12.202 −23.538 −24.624 1.00 29.39 C

ATOM 3329 CD1 LEU A 413 11.518 −23.746 −23.274 1.00 29.64 C

ATOM 3330 CD2 LEU A 413 13.712 −23.665 −24.417 1.00 29.11 C

ATOM 3331 HN LEU A 413 10.806 −22.779 −27.741 1.00 0.00 H

ATOM 3332 HA LEU A 413 13.221 −24.428 −27.155 1.00 29.64 H

ATOM 3333 HB1 LEU A 413 10.589 −24.508 −25.642 1.00 29.64 H

ATOM 3334 HB2 LEU A 413 12.011 −25.540 −25.356 1.00 29.64 H

ATOM 3335 HG LEU A 413 11.981 −22.530 −24.976 1.00 29.64 H

ATOM 3336 1HD1 LEU A 413 11.903 −23.022 −22.556 1.00 29.64 H

ATOM 3337 2HD1 LEU A 413 11.720 −24.755 −22.915 1.00 29.64 H

ATOM 3338 3HD1 LEU A 413 10.442 −23.609 −23.386 1.00 29.64 H

ATOM 3339 1HD2 LEU A 413 14.041 −22.934 −23.678 1.00 29.64 H

ATOM 3340 2HD2 LEU A 413 14.225 −23.483 −25.361 1.00 29.64 H

ATOM 3341 3HD2 LEU A 413 13.948 −24.669 −24.064 1.00 29.64 H

ATOM 3342 N LEU A 414 12.201 −26.486 −28.234 1.00 29.98 N

ATOM 3343 CA LEU A 414 11.608 −27.748 −28.673 1.00 30.37 C

ATOM 3344 C LEU A 414 11.495 −28.692 −27.469 1.00 30.54 C

ATOM 3345 O LEU A 414 12.492 −29.215 −26.968 1.00 30.32 O

ATOM 3346 CB LEU A 414 12.415 −28.337 −29.843 1.00 30.34 C

ATOM 3347 CG LEU A 414 12.166 −27.599 −31.171 1.00 30.36 C

ATOM 3348 CD1 LEU A 414 13.267 −27.907 −32.186 1.00 30.22 C

ATOM 3349 CD2 LEU A 414 10.832 −27.982 −31.813 1.00 30.80 C

ATOM 3350 HN LEU A 414 13.141 −26.525 −27.850 1.00 0.00 H

ATOM 3351 HA LEU A 414 10.600 −27.539 −29.032 1.00 30.37 H

ATOM 3352 HB1 LEU A 414 13.476 −28.271 −29.602 1.00 30.37 H

ATOM 3353 HB2 LEU A 414 12.134 −29.383 −29.968 1.00 30.37 H

ATOM 3354 HG LEU A 414 12.163 −26.526 −30.977 1.00 30.37 H

ATOM 3355 1HD1 LEU A 414 13.066 −27.373 −33.114 1.00 30.37 H

ATOM 3356 2HD1 LEU A 414 13.291 −28.979 −32.382 1.00 30.37 H

ATOM 3357 3HD1 LEU A 414 14.230 −27.589 −31.786 1.00 30.37 H

```
ATOM   3358 1HD2 LEU A 414      10.707 −27.433 −32.746  1.00 30.37           H
ATOM   3359 2HD2 LEU A 414      10.017 −27.734 −31.134  1.00 30.37           H
ATOM   3360 3HD2 LEU A 414      10.821 −29.053 −32.017  1.00 30.37           H
ATOM   3361  N   LYS A 415      10.266 −28.811 −26.966  1.00 30.93           N
ATOM   3362  CA  LYS A 415       9.820 −29.746 −25.929  1.00 31.15           C
ATOM   3363  C   LYS A 415       9.801 −31.174 −26.492  1.00 31.47           C
ATOM   3364  O   LYS A 415       9.338 −31.381 −27.614  1.00 31.77           O
ATOM   3365  CB  LYS A 415       8.419 −29.274 −25.498  1.00 31.44           C
ATOM   3366  CG  LYS A 415       7.795 −30.021 −24.314  1.00 31.88           C
ATOM   3367  CD  LYS A 415       6.318 −29.618 −24.170  1.00 32.14           C
ATOM   3368  CE  LYS A 415       5.649 −30.297 −22.969  1.00 32.69           C
ATOM   3369  NZ  LYS A 415       5.777 −29.485 −21.736  1.00 32.84           N1+
ATOM   3370  HN  LYS A 415       9.527 −28.344 −27.485  1.00  0.00           H
ATOM   3371  HZ1 LYS A 415       5.371 −29.935 −20.932  1.00  0.00           H
ATOM   3372  HZ2 LYS A 415       6.738 −29.237 −21.523  1.00  0.00           H
ATOM   3373  HZ3 LYS A 415       5.300 −28.584 −21.849  1.00  0.00           H
ATOM   3374  HA  LYS A 415      10.499 −29.698 −25.078  1.00 31.81           H
ATOM   3375  HB1 LYS A 415       8.490 −28.220 −25.228  1.00 31.81           H
ATOM   3376  HB2 LYS A 415       7.752 −29.386 −26.352  1.00 31.81           H
ATOM   3377  HG1 LYS A 415       7.863 −31.095 −24.487  1.00 31.81           H
ATOM   3378  HG2 LYS A 415       8.331 −29.765 −23.400  1.00 31.81           H
ATOM   3379  HD1 LYS A 415       6.260 −28.537 −24.040  1.00 31.81           H
ATOM   3380  HD2 LYS A 415       5.786 −29.905 −25.077  1.00 31.81           H
ATOM   3381  HE1 LYS A 415       4.591 −30.440 −23.189  1.00 31.81           H
ATOM   3382  HE2 LYS A 415       6.120 −31.266 −22.804  1.00 31.81           H
ATOM   3383  N   TYR A 416      10.211 −32.163 −25.702  1.00 31.42           N
ATOM   3384  CA  TYR A 416      10.307 −33.573 −26.105  1.00 31.73           C
```

ATOM 3385 C TYR A 416 10.047 −34.480 −24.895 1.00 31.90 C

ATOM 3386 O TYR A 416 10.918 −34.631 −24.040 1.00 31.62 O

ATOM 3387 CB TYR A 416 11.706 −33.806 −26.697 1.00 31.48 C

ATOM 3388 CG TYR A 416 11.950 −35.166 −27.330 1.00 31.86 C

ATOM 3389 CD1 TYR A 416 11.496 −35.426 −28.638 1.00 32.12 C

ATOM 3390 CD2 TYR A 416 12.696 −36.142 −26.643 1.00 31.95 C

ATOM 3391 CE1 TYR A 416 11.835 −36.631 −29.284 1.00 32.49 C

ATOM 3392 CE2 TYR A 416 13.027 −37.355 −27.278 1.00 32.33 C

ATOM 3393 CZ TYR A 416 12.609 −37.595 −28.605 1.00 32.61 C

ATOM 3394 OH TYR A 416 12.942 −38.760 −29.219 1.00 33.01 O

ATOM 3395 HN TYR A 416 10.663 −31.896 −24.832 1.00 0.00 H

ATOM 3396 HH TYR A 416 13.212 −39.419 −28.579 1.00 0.00 H

ATOM 3397 HA TYR A 416 9.558 −33.777 −26.870 1.00 32.04 H

ATOM 3398 HB1 TYR A 416 11.873 −33.048 −27.463 1.00 32.04 H

ATOM 3399 HB2 TYR A 416 12.432 −33.678 −25.894 1.00 32.04 H

ATOM 3400 HD1 TYR A 416 10.879 −34.690 −29.153 1.00 32.04 H

ATOM 3401 HD2 TYR A 416 13.018 −35.959 −25.618 1.00 32.04 H

ATOM 3402 HE1 TYR A 416 11.501 −36.818 −30.304 1.00 32.04 H

ATOM 3403 HE2 TYR A 416 13.606 −38.109 −26.745 1.00 32.04 H

ATOM 3404 N ASN A 417 8.797 −34.913 −24.707 1.00 32.37 N

ATOM 3405 CA ASN A 417 8.398 −35.716 −23.545 1.00 32.60 C

ATOM 3406 C ASN A 417 8.552 −37.220 −23.813 1.00 32.91 C

ATOM 3407 O ASN A 417 7.847 −37.779 −24.656 1.00 33.38 O

ATOM 3408 CB ASN A 417 6.977 −35.330 −23.114 1.00 32.98 C

ATOM 3409 CG ASN A 417 6.643 −35.996 −21.792 1.00 33.03 C

ATOM 3410 OD1 ASN A 417 7.339 −35.828 −20.807 1.00 32.78 O

ATOM 3411 ND2 ASN A 417 5.697 −36.900 −21.775 1.00 33.34 N

ATOM 3412 HN ASN A 417 8.115 −34.751 −25.431 1.00 0.00 H

ATOM 3413 1HD2 ASN A 417 5.228 −37.168 −22.615 1.00 0.00 H

ATOM 3414 2HD2 ASN A 417 5.710 −37.504 −20.954 1.00 0.00 H

ATOM 3415 HA ASN A 417 9.069 −35.460 −22.725 1.00 32.92 H

ATOM 3416 HB1 ASN A 417 6.915 −34.248 −23.000 1.00 32.92 H

ATOM 3417 HB2 ASN A 417 6.267 −35.657 −23.874 1.00 32.92 H

ATOM 3418 N LEU A 418 9.434 −37.879 −23.062 1.00 32.67 N

ATOM 3419 CA LEU A 418 9.832 −39.272 −23.280 1.00 32.94 C

ATOM 3420 C LEU A 418 8.716 −40.292 −22.976 1.00 33.45 C

ATOM 3421 O LEU A 418 8.602 −41.266 −23.722 1.00 33.83 O

ATOM 3422 CB LEU A 418 11.102 −39.546 −22.455 1.00 32.53 C

ATOM 3423 CG LEU A 418 12.344 −38.734 −22.875 1.00 32.07 C

ATOM 3424 CD1 LEU A 418 13.437 −38.837 −21.815 1.00 31.67 C

ATOM 3425 CD2 LEU A 418 12.925 −39.241 −24.198 1.00 32.31 C

ATOM 3426 HN LEU A 418 9.926 −37.369 −22.330 1.00 0.00 H

ATOM 3427 HA LEU A 418 10.095 −39.377 −24.332 1.00 32.68 H

ATOM 3428 HB1 LEU A 418 10.884 −39.314 −21.413 1.00 32.68 H

ATOM 3429 HB2 LEU A 418 11.343 −40.605 −22.549 1.00 32.68 H

ATOM 3430 HG LEU A 418 12.061 −37.688 −22.991 1.00 32.68 H

ATOM 3431 1HD1 LEU A 418 14.305 −38.257 −22.130 1.00 32.68 H

ATOM 3432 2HD1 LEU A 418 13.724 −39.881 −21.689 1.00 32.68 H

ATOM 3433 3HD1 LEU A 418 13.064 −38.446 −20.868 1.00 32.68 H

ATOM 3434 1HD2 LEU A 418 13.799 −38.646 −24.463 1.00 32.68 H

ATOM 3435 2HD2 LEU A 418 12.174 −39.153 −24.983 1.00 32.68 H

ATOM 3436 3HD2 LEU A 418 13.216 −40.286 −24.091 1.00 32.68 H

ATOM 3437 N SER A 419 7.728 −39.935 −22.141 1.00 33.50 N

ATOM 3438 CA SER A 419 6.552 −40.781 −21.838 1.00 34.04 C

ATOM	3439	C	SER	A	419	5.671	−41.084	−23.058	1.00	34.59	C
ATOM	3440	O	SER	A	419	4.808	−41.957	−23.001	1.00	35.09	O
ATOM	3441	CB	SER	A	419	5.637	−40.177	−20.751	1.00	33.95	C
ATOM	3442	OG	SER	A	419	6.194	−39.091	−20.042	1.00	33.55	O
ATOM	3443	HN	SER	A	419	7.941	−39.200	−21.476	1.00	0.00	H
ATOM	3444	HG	SER	A	419	7.087	−39.379	−19.691	1.00	0.00	H
ATOM	3445	HA	SER	A	419	6.924	−41.733	−21.459	1.00	34.12	H
ATOM	3446	HB1	SER	A	419	4.721	−39.833	−21.232	1.00	34.12	H
ATOM	3447	HB2	SER	A	419	5.399	−40.963	−20.034	1.00	34.12	H
ATOM	3448	N	LEU	A	420	5.814	−40.320	−24.147	1.00	34.51	N
ATOM	3449	CA	LEU	A	420	5.107	−40.550	−25.406	1.00	35.00	C
ATOM	3450	C	LEU	A	420	5.605	−41.846	−26.064	1.00	35.44	C
ATOM	3451	O	LEU	A	420	5.358	−42.082	−27.250	1.00	35.95	O
ATOM	3452	CB	LEU	A	420	5.281	−39.320	−26.317	1.00	34.74	C
ATOM	3453	CG	LEU	A	420	4.695	−38.006	−25.765	1.00	34.51	C
ATOM	3454	CD1	LEU	A	420	5.059	−36.856	−26.704	1.00	33.93	C
ATOM	3455	CD2	LEU	A	420	3.170	−38.063	−25.652	1.00	34.98	C
ATOM	3456	HN	LEU	A	420	6.579	−39.654	−24.142	1.00	0.00	H
ATOM	3457	HA	LEU	A	420	4.046	−40.665	−25.186	1.00	34.88	H
ATOM	3458	HC	LEU	A	420	6.181	−42.563	−25.479	1.00	34.88	H
ATOM	3459	HB1	LEU	A	420	6.348	−39.170	−26.481	1.00	34.88	H
ATOM	3460	HB2	LEU	A	420	4.793	−39.533	−27.268	1.00	34.88	H
ATOM	3461	HG	LEU	A	420	5.119	−37.811	−24.780	1.00	34.88	H
ATOM	3462	1HD1	LEU	A	420	4.646	−35.925	−26.316	1.00	34.88	H
ATOM	3463	2HD1	LEU	A	420	4.647	−37.051	−27.694	1.00	34.88	H
ATOM	3464	3HD1	LEU	A	420	6.144	−36.771	−26.772	1.00	34.88	H
ATOM	3465	1HD2	LEU	A	420	2.799	−37.117	−25.259	1.00	34.88	H

ATOM 3466 2HD2 LEU A 420 2.885 −38.872 −24.979 1.00 34.88 H
ATOM 3467 3HD2 LEU A 420 2.739 −38.241 −26.637 1.00 34.88 H
ATOM 3468 N ILE A 424 9.238 −40.377 −30.929 1.00 34.45 N
ATOM 3469 CA ILE A 424 8.601 −39.459 −29.981 1.00 34.27 C
ATOM 3470 C ILE A 424 8.507 −38.057 −30.613 1.00 33.88 C
ATOM 3471 O ILE A 424 9.183 −37.753 −31.599 1.00 33.54 O
ATOM 3472 CB ILE A 424 9.332 −39.509 −28.610 1.00 34.01 C
ATOM 3473 CG1 ILE A 424 8.897 −40.726 −27.756 1.00 34.49 C
ATOM 3474 CG2 ILE A 424 9.106 −38.264 −27.730 1.00 33.66 C
ATOM 3475 CD1 ILE A 424 9.225 −42.106 −28.340 1.00 35.04 C
ATOM 3476 HN1 ILE A 424 10.221 −40.024 −31.163 1.00 0.00 H
ATOM 3477 HN2 ILE A 424 8.655 −40.427 −31.825 1.00 34.17 H
ATOM 3478 HA ILE A 424 7.583 −39.812 −29.820 1.00 34.17 H
ATOM 3479 HB ILE A 424 10.401 −39.597 −28.801 1.00 34.17 H
ATOM 3480 1HG1 ILE A 424 9.391 −40.648 −26.787 1.00 34.17 H
ATOM 3481 2HG1 ILE A 424 7.817 −40.669 −27.620 1.00 34.17 H
ATOM 3482 1HG2 ILE A 424 9.649 −38.378 −26.792 1.00 34.17 H
ATOM 3483 2HG2 ILE A 424 8.042 −38.154 −27.522 1.00 34.17 H
ATOM 3484 3HG2 ILE A 424 9.467 −37.378 −28.253 1.00 34.17 H
ATOM 3485 1HD1 ILE A 424 8.875 −42.882 −27.659 1.00 34.17 H
ATOM 3486 2HD1 ILE A 424 10.303 −42.198 −28.472 1.00 34.17 H
ATOM 3487 3HD1 ILE A 424 8.730 −42.220 −29.304 1.00 34.17 H
ATOM 3488 N ILE A 425 7.499 −37.294 −30.185 1.00 33.98 N
ATOM 3489 CA ILE A 425 7.091 −36.014 −30.779 1.00 33.71 C
ATOM 3490 C ILE A 425 7.971 −34.870 −30.257 1.00 33.14 C
ATOM 3491 O ILE A 425 8.118 −34.718 −29.044 1.00 33.01 O
ATOM 3492 CB ILE A 425 5.590 −35.753 −30.492 1.00 33.99 C

```
ATOM   3493  CG1 ILE A 425       4.731 −36.969 −30.919  1.00 34.60           C
ATOM   3494  CG2 ILE A 425       5.124 −34.464 −31.185  1.00 33.68           C
ATOM   3495  CD1 ILE A 425       3.213 −36.799 −30.783  1.00 34.81           C
ATOM   3496  HN  ILE A 425       7.020 −37.585 −29.348  1.00  0.00           H
ATOM   3497  HA  ILE A 425       7.225 −36.081 −31.859  1.00 33.86           H
ATOM   3498  HB  ILE A 425       5.472 −35.619 −29.417  1.00 33.86           H
ATOM   3499 1HG1 ILE A 425       4.950 −37.182 −31.965  1.00 33.86           H
ATOM   3500 2HG1 ILE A 425       5.028 −37.819 −30.305  1.00 33.86           H
ATOM   3501 1HG2 ILE A 425       4.068 −34.298 −30.972  1.00 33.86           H
ATOM   3502 2HG2 ILE A 425       5.266 −34.558 −32.262  1.00 33.86           H
ATOM   3503 3HG2 ILE A 425       5.706 −33.621 −30.814  1.00 33.86           H
ATOM   3504 1HD1 ILE A 425       2.714 −37.711 −31.110  1.00 33.86           H
ATOM   3505 2HD1 ILE A 425       2.884 −35.964 −31.401  1.00 33.86           H
ATOM   3506 3HD1 ILE A 425       2.961 −36.601 −29.741  1.00 33.86           H
ATOM   3507  N   LEU A 426       8.415 −33.980 −31.151  1.00 32.81           N
ATOM   3508  CA  LEU A 426       8.852 −32.628 −30.777  1.00 32.29           C
ATOM   3509  C   LEU A 426       7.671 −31.650 −30.799  1.00 32.29           C
ATOM   3510  O   LEU A 426       6.949 −31.592 −31.792  1.00 32.54           O
ATOM   3511  CB  LEU A 426       9.948 −32.131 −31.733  1.00 31.95           C
ATOM   3512  CG  LEU A 426      11.311 −32.820 −31.585  1.00 31.92           C
ATOM   3513  CD1 LEU A 426      12.212 −32.365 −32.729  1.00 31.80           C
ATOM   3514  CD2 LEU A 426      12.032 −32.443 −30.291  1.00 31.56           C
ATOM   3515  HN  LEU A 426       8.192 −34.143 −32.127  1.00  0.00           H
ATOM   3516  HA  LEU A 426       9.259 −32.660 −29.766  1.00 32.15           H
ATOM   3517  HB1 LEU A 426       9.601 −32.289 −32.754  1.00 32.15           H
ATOM   3518  HB2 LEU A 426      10.089 −31.065 −31.557  1.00 32.15           H
ATOM   3519  HG  LEU A 426      11.181 −33.901 −31.630  1.00 32.14           H
```

ATOM 3520 1HD1 LEU A 426 13.186 −32.846 −32.639 1.00 32.14 H

ATOM 3521 2HD1 LEU A 426 12.336 −31.283 −32.686 1.00 32.14 H

ATOM 3522 3HD1 LEU A 426 11.758 −32.640 −33.681 1.00 32.14 H

ATOM 3523 1HD2 LEU A 426 12.990 −32.962 −30.244 1.00 32.14 H

ATOM 3524 2HD2 LEU A 426 11.420 −32.732 −29.436 1.00 32.14 H

ATOM 3525 3HD2 LEU A 426 12.201 −31.366 −30.269 1.00 32.14 H

ATOM 3526 N GLN A 427 7.596 −30.751 −29.819 1.00 32.01 N

ATOM 3527 CA GLN A 427 6.581 −29.691 −29.747 1.00 32.05 C

ATOM 3528 C GLN A 427 7.254 −28.324 −29.546 1.00 31.59 C

ATOM 3529 O GLN A 427 8.116 −28.184 −28.679 1.00 31.34 O

ATOM 3530 CB GLN A 427 5.561 −30.007 −28.637 1.00 32.34 C

ATOM 3531 CG GLN A 427 4.902 −31.389 −28.795 1.00 32.77 C

ATOM 3532 CD GLN A 427 3.775 −31.614 −27.795 1.00 33.14 C

ATOM 3533 OE1 GLN A 427 3.917 −31.395 −26.600 1.00 33.09 O

ATOM 3534 NE2 GLN A 427 2.643 −32.127 −28.222 1.00 33.55 N

ATOM 3535 HN GLN A 427 8.243 −30.835 −29.040 1.00 0.00 H

ATOM 3536 1HE2 GLN A 427 2.484 −32.304 −29.225 1.00 0.00 H

ATOM 3537 2HE2 GLN A 427 1.918 −32.267 −27.550 1.00 0.00 H

ATOM 3538 HA GLN A 427 6.049 −29.668 −30.698 1.00 32.43 H

ATOM 3539 HB1 GLN A 427 6.074 −29.976 −27.676 1.00 32.43 H

ATOM 3540 HB2 GLN A 427 4.780 −29.247 −28.657 1.00 32.43 H

ATOM 3541 HG1 GLN A 427 4.496 −31.470 −29.803 1.00 32.43 H

ATOM 3542 HG2 GLN A 427 5.661 −32.157 −28.646 1.00 32.43 H

ATOM 3543 N PHE A 428 6.965 −27.338 −30.400 1.00 31.50 N

ATOM 3544 CA PHE A 428 7.614 −26.020 −30.342 1.00 31.09 C

ATOM 3545 C PHE A 428 6.866 −25.024 −29.445 1.00 31.08 C

ATOM 3546 O PHE A 428 5.677 −24.774 −29.646 1.00 31.41 O

ATOM 3547 CB PHE A 428 7.821 −25.455 −31.750 1.00 31.02 C

ATOM 3548 CG PHE A 428 8.544 −24.116 −31.778 1.00 30.60 C

ATOM 3549 CD1 PHE A 428 9.917 −24.045 −31.474 1.00 30.28 C

ATOM 3550 CD2 PHE A 428 7.844 −22.933 −32.087 1.00 30.54 C

ATOM 3551 CE1 PHE A 428 10.584 −22.806 −31.472 1.00 29.91 C

ATOM 3552 CE2 PHE A 428 8.512 −21.694 −32.092 1.00 30.18 C

ATOM 3553 CZ PHE A 428 9.881 −21.629 −31.781 1.00 29.87 C

ATOM 3554 HN PHE A 428 6.244 −27.497 −31.098 1.00 0.00 H

ATOM 3555 HA PHE A 428 8.602 −26.167 −29.906 1.00 30.68 H

ATOM 3556 HB1 PHE A 428 8.406 −26.173 −32.325 1.00 30.68 H

ATOM 3557 HB2 PHE A 428 6.844 −25.327 −32.215 1.00 30.68 H

ATOM 3558 HD1 PHE A 428 10.467 −24.956 −31.239 1.00 30.68 H

ATOM 3559 HD2 PHE A 428 6.781 −22.977 −32.323 1.00 30.68 H

ATOM 3560 HE1 PHE A 428 11.646 −22.759 −31.231 1.00 30.68 H

ATOM 3561 HE2 PHE A 428 7.966 −20.783 −32.338 1.00 30.68 H

ATOM 3562 HZ PHE A 428 10.396 −20.668 −31.779 1.00 30.68 H

ATOM 3563 N ILE A 429 7.625 −24.304 −28.617 1.00 30.74 N

ATOM 3564 CA ILE A 429 7.180 −23.154 −27.827 1.00 30.70 C

ATOM 3565 C ILE A 429 7.976 −21.914 −28.253 1.00 30.33 C

ATOM 3566 O ILE A 429 9.201 −21.909 −28.167 1.00 30.02 O

ATOM 3567 CB ILE A 429 7.353 −23.469 −26.323 1.00 30.69 C

ATOM 3568 CG1 ILE A 429 6.435 −24.610 −25.817 1.00 31.13 C

ATOM 3569 CG2 ILE A 429 7.161 −22.206 −25.476 1.00 30.51 C

ATOM 3570 CD1 ILE A 429 4.926 −24.325 −25.866 1.00 31.38 C

ATOM 3571 HN ILE A 429 8.612 −24.539 −28.561 1.00 0.00 H

ATOM 3572 HA ILE A 429 6.123 −22.978 −28.029 1.00 30.69 H

ATOM 3573 HB ILE A 429 8.382 −23.798 −26.180 1.00 30.69 H

ATOM 3574 1HG1 ILE A 429 6.629 −25.491 −26.428 1.00 30.69 H

ATOM 3575 2HG1 ILE A 429 6.702 −24.820 −24.781 1.00 30.69 H

ATOM 3576 1HG2 ILE A 429 7.287 −22.452 −24.422 1.00 30.69 H

ATOM 3577 2HG2 ILE A 429 6.159 −21.808 −25.638 1.00 30.69 H

ATOM 3578 3HG2 ILE A 429 7.900 −21.458 −25.765 1.00 30.69 H

ATOM 3579 1HD1 ILE A 429 4.380 −25.190 −25.489 1.00 30.69 H

ATOM 3580 2HD1 ILE A 429 4.626 −24.127 −26.895 1.00 30.69 H

ATOM 3581 3HD1 ILE A 429 4.700 −23.456 −25.248 1.00 30.69 H

ATOM 3582 N GLU A 430 7.288 −20.835 −28.625 1.00 30.39 N

ATOM 3583 CA GLU A 430 7.908 −19.535 −28.906 1.00 30.08 C

ATOM 3584 C GLU A 430 8.277 −18.760 −27.627 1.00 29.90 C

ATOM 3585 O GLU A 430 7.578 −18.840 −26.616 1.00 30.07 O

ATOM 3586 CB GLU A 430 6.991 −18.713 −29.824 1.00 30.21 C

ATOM 3587 CG GLU A 430 5.584 −18.406 −29.269 1.00 30.38 C

ATOM 3588 CD GLU A 430 4.750 −17.576 −30.262 1.00 30.63 C

ATOM 3589 OE1 GLU A 430 4.931 −17.794 −31.480 1.00 30.68 O

ATOM 3590 OE2 GLU A 430 4.396 −16.433 −29.901 1.00 30.79 O

ATOM 3591 HN GLU A 430 6.282 −20.874 −28.593 1.00 0.00 H

ATOM 3592 HA GLU A 430 8.832 −19.725 −29.452 1.00 30.35 H

ATOM 3593 HB1 GLU A 430 7.485 −17.763 −30.026 1.00 30.35 H

ATOM 3594 HB2 GLU A 430 6.869 −19.265 −30.756 1.00 30.35 H

ATOM 3595 HG1 GLU A 430 5.068 −19.346 −29.074 1.00 30.35 H

ATOM 3596 HG2 GLU A 430 5.685 −17.847 −28.339 1.00 30.35 H

ATOM 3597 N ILE A 431 9.330 −17.936 −27.699 1.00 29.56 N

ATOM 3598 CA ILE A 431 9.667 −16.948 −26.660 1.00 29.38 C

ATOM 3599 C ILE A 431 8.743 −15.723 −26.718 1.00 29.53 C

ATOM 3600 O ILE A 431 8.392 −15.267 −27.808 1.00 29.62 O

ATOM 3601 CB ILE A 431 11.162 −16.557 −26.752 1.00 29.00 C

ATOM 3602 CG1 ILE A 431 11.657 −15.952 −25.421 1.00 28.83 C

ATOM 3603 CG2 ILE A 431 11.480 −15.590 −27.912 1.00 28.92 C

ATOM 3604 CD1 ILE A 431 13.176 −16.067 −25.259 1.00 28.52 C

ATOM 3605 HN ILE A 431 9.904 −17.948 −28.534 1.00 0.00 H

ATOM 3606 HA ILE A 431 9.514 −17.427 −25.693 1.00 29.17 H

ATOM 3607 HB ILE A 431 11.729 −17.472 −26.925 1.00 29.17 H

ATOM 3608 1HG1 ILE A 431 11.380 −14.898 −25.391 1.00 29.17 H

ATOM 3609 2HG1 ILE A 431 11.176 −16.478 −24.597 1.00 29.17 H

ATOM 3610 1HG2 ILE A 431 12.546 −15.361 −27.913 1.00 29.17 H

ATOM 3611 2HG2 ILE A 431 10.911 −14.669 −27.784 1.00 29.17 H

ATOM 3612 3HG2 ILE A 431 11.208 −16.056 −28.859 1.00 29.17 H

ATOM 3613 1HD1 ILE A 431 13.475 −15.627 −24.307 1.00 29.17 H

ATOM 3614 2HD1 ILE A 431 13.670 −15.538 −26.074 1.00 29.17 H

ATOM 3615 3HD1 ILE A 431 13.466 −17.118 −25.280 1.00 29.17 H

ATOM 3616 N ALA A 432 8.451 −15.112 −25.568 1.00 29.57 N

ATOM 3617 CA ALA A 432 7.614 −13.914 −25.440 1.00 29.73 C

ATOM 3618 C ALA A 432 8.048 −12.710 −26.314 1.00 29.55 C

ATOM 3619 O ALA A 432 9.199 −12.581 −26.733 1.00 29.25 O

ATOM 3620 CB ALA A 432 7.552 −13.532 −23.954 1.00 29.72 C

ATOM 3621 HN ALA A 432 8.679 −15.614 −24.716 1.00 0.00 H

ATOM 3622 HA ALA A 432 6.605 −14.190 −25.746 1.00 29.56 H

ATOM 3623 HB1 ALA A 432 6.934 −12.642 −23.833 1.00 29.56 H

ATOM 3624 HB2 ALA A 432 8.558 −13.327 −23.589 1.00 29.56 H

ATOM 3625 HB3 ALA A 432 7.119 −14.355 −23.385 1.00 29.56 H

ATOM 3626 N ASP A 433 7.120 −11.771 −26.516 1.00 29.73 N

ATOM 3627 CA ASP A 433 7.316 −10.492 −27.217 1.00 29.58 C

ATOM	3628	C	ASP	A	433	8.240	−9.513	−26.461	1.00	29.37	C
ATOM	3629	O	ASP	A	433	8.782	−8.571	−27.044	1.00	29.27	O
ATOM	3630	CB	ASP	A	433	5.942	−9.817	−27.386	1.00	29.85	C
ATOM	3631	CG	ASP	A	433	5.325	−9.431	−26.032	1.00	30.11	C
ATOM	3632	OD1	ASP	A	433	5.072	−10.388	−25.266	1.00	30.09	O
ATOM	3633	OD2	ASP	A	433	5.645	−8.305	−25.586	1.00	30.34	O
ATOM	3634	HN	ASP	A	433	6.227	−11.859	−26.035	1.00	0.00	H
ATOM	3635	HA	ASP	A	433	7.738	−10.689	−28.202	1.00	29.79	H
ATOM	3636	HB1	ASP	A	433	6.063	−8.916	−27.988	1.00	29.79	H
ATOM	3637	HB2	ASP	A	433	5.270	−10.508	−27.896	1.00	29.79	H
ATOM	3638	N	ASN	A	434	8.360	−9.683	−25.141	1.00	29.30	N
ATOM	3639	CA	ASN	A	434	9.047	−8.740	−24.266	1.00	29.19	C
ATOM	3640	C	ASN	A	434	10.544	−8.696	−24.638	1.00	28.85	C
ATOM	3641	O	ASN	A	434	11.232	−9.709	−24.519	1.00	28.68	O
ATOM	3642	CB	ASN	A	434	8.842	−9.156	−22.793	1.00	29.31	C
ATOM	3643	CG	ASN	A	434	7.414	−9.377	−22.302	1.00	29.69	C
ATOM	3644	OD1	ASN	A	434	7.223	−9.968	−21.249	1.00	29.84	O
ATOM	3645	ND2	ASN	A	434	6.375	−8.931	−22.963	1.00	29.87	N
ATOM	3646	HN	ASN	A	434	7.796	−10.411	−24.729	1.00	0.00	H
ATOM	3647	1HD2	ASN	A	434	6.424	−8.600	−23.932	1.00	0.00	H
ATOM	3648	2HD2	ASN	A	434	5.475	−9.282	−22.690	1.00	0.00	H
ATOM	3649	HA	ASN	A	434	8.619	−7.748	−24.414	1.00	29.34	H
ATOM	3650	HB1	ASN	A	434	9.385	−10.089	−22.642	1.00	29.34	H
ATOM	3651	HB2	ASN	A	434	9.281	−8.375	−22.172	1.00	29.34	H
ATOM	3652	N	ARG	A	435	11.042	−7.553	−25.143	1.00	30.70	N
ATOM	3653	CA	ARG	A	435	12.352	−7.442	−25.836	1.00	30.41	C
ATOM	3654	C	ARG	A	435	12.522	−8.508	−26.935	1.00	30.33	C

ATOM 3655 O ARG A 435 13.543 −9.192 −27.006 1.00 30.15 O
ATOM 3656 CB ARG A 435 13.546 −7.390 −24.849 1.00 30.20 C
ATOM 3657 CG ARG A 435 13.652 −6.054 −24.096 1.00 30.27 C
ATOM 3658 CD ARG A 435 14.786 −6.038 −23.057 1.00 30.09 C
ATOM 3659 NE ARG A 435 16.146 −6.118 −23.636 1.00 29.80 N
ATOM 3660 CZ ARG A 435 17.259 −6.313 −22.952 1.00 29.70 C
ATOM 3661 NH1 ARG A 435 18.393 −6.455 −23.565 1.00 29.45 N
ATOM 3662 NH2 ARG A 435 17.264 −6.371 −21.649 1.00 29.88 N
ATOM 3663 HN ARG A 435 10.377 −6.805 −25.280 1.00 0.00 H
ATOM 3664 HE ARG A 435 16.274 −6.116 −24.652 1.00 0.00 H
ATOM 3665 1HH1 ARG A 435 18.430 −6.224 −24.571 1.00 0.00 H
ATOM 3666 2HH1 ARG A 435 19.255 −6.499 −23.066 1.00 0.00 H
ATOM 3667 1HH2 ARG A 435 16.464 −6.020 −21.135 1.00 0.00 H
ATOM 3668 2HH2 ARG A 435 18.084 −6.677 −21.145 1.00 0.00 H
ATOM 3669 HA ARG A 435 12.342 −6.480 −26.348 1.00 30.09 H
ATOM 3670 HB1 ARG A 435 13.427 −8.190 −24.119 1.00 30.09 H
ATOM 3671 HB2 ARG A 435 14.467 −7.545 −25.411 1.00 30.09 H
ATOM 3672 HG1 ARG A 435 13.834 −5.260 −24.821 1.00 30.09 H
ATOM 3673 HG2 ARG A 435 12.708 −5.869 −23.583 1.00 30.09 H
ATOM 3674 HD1 ARG A 435 14.712 −5.112 −22.486 1.00 30.09 H
ATOM 3675 HD2 ARG A 435 14.649 −6.889 −22.390 1.00 30.09 H
ATOM 3676 N LEU A 436 11.526 −8.657 −27.810 1.00 28.55 N
ATOM 3677 CA LEU A 436 11.640 −9.473 −29.018 1.00 28.54 C
ATOM 3678 C LEU A 436 12.813 −8.994 −29.888 1.00 28.30 C
ATOM 3679 O LEU A 436 12.936 −7.812 −30.198 1.00 28.26 O
ATOM 3680 CB LEU A 436 10.307 −9.446 −29.791 1.00 28.79 C
ATOM 3681 CG LEU A 436 10.280 −10.286 −31.080 1.00 28.94 C

ATOM 3682 CD1 LEU A 436 10.450 −11.781 −30.805 1.00 28.93 C
ATOM 3683 CD2 LEU A 436 8.944 −10.092 −31.800 1.00 29.25 C
ATOM 3684 HN LEU A 436 10.642 −8.197 −27.628 1.00 0.00 H
ATOM 3685 HA LEU A 436 11.838 −10.502 −28.716 1.00 28.69 H
ATOM 3686 HB1 LEU A 436 9.526 −9.819 −29.129 1.00 28.69 H
ATOM 3687 HB2 LEU A 436 10.093 −8.411 −30.058 1.00 28.69 H
ATOM 3688 HG LEU A 436 11.085 −9.954 −31.735 1.00 28.70 H
ATOM 3689 1HD1 LEU A 436 10.424 −12.329 −31.747 1.00 28.70 H
ATOM 3690 2HD1 LEU A 436 9.641 −12.127 −30.161 1.00 28.70 H
ATOM 3691 3HD1 LEU A 436 11.406 −11.954 −30.311 1.00 28.70 H
ATOM 3692 1HD2 LEU A 436 8.932 −10.689 −32.712 1.00 28.70 H
ATOM 3693 2HD2 LEU A 436 8.817 −9.040 −32.054 1.00 28.70 H
ATOM 3694 3HD2 LEU A 436 8.130 −10.408 −31.148 1.00 28.70 H
ATOM 3695 N THR A 437 13.595 −9.950 −30.379 1.00 28.80 N
ATOM 3696 CA THR A 437 14.648 −9.774 −31.389 1.00 28.61 C
ATOM 3697 C THR A 437 14.591 −10.953 −32.356 1.00 28.65 C
ATOM 3698 O THR A 437 13.956 −11.966 −32.047 1.00 28.76 O
ATOM 3699 CB THR A 437 16.049 −9.672 −30.754 1.00 28.36 C
ATOM 3700 OG1 THR A 437 16.340 −10.798 −29.951 1.00 28.32 O
ATOM 3701 CG2 THR A 437 16.215 −8.444 −29.866 1.00 28.33 C
ATOM 3702 HN THR A 437 13.367 −10.906 −30.152 1.00 0.00 H
ATOM 3703 HG1 THR A 437 16.567 −11.540 −30.531 1.00 0.00 H
ATOM 3704 HA THR A 437 14.449 −8.857 −31.944 1.00 28.55 H
ATOM 3705 HB THR A 437 16.787 −9.620 −31.555 1.00 28.55 H
ATOM 3706 1HG2 THR A 437 17.222 −8.430 −29.449 1.00 28.55 H
ATOM 3707 2HG2 THR A 437 15.487 −8.480 −29.055 1.00 28.55 H
ATOM 3708 3HG2 THR A 437 16.055 −7.543 −30.458 1.00 28.55 H

ATOM	3709	N	ILE	A	438	15.323	−10.871	−33.473	1.00	27.92	N
ATOM	3710	CA	ILE	A	438	15.451	−11.970	−34.440	1.00	27.96	C
ATOM	3711	C	ILE	A	438	15.783	−13.296	−33.751	1.00	27.93	C
ATOM	3712	O	ILE	A	438	16.641	−13.347	−32.868	1.00	27.78	O
ATOM	3713	CB	ILE	A	438	16.486	−11.627	−35.538	1.00	27.81	C
ATOM	3714	CG1	ILE	A	438	16.565	−12.770	−36.577	1.00	27.92	C
ATOM	3715	CG2	ILE	A	438	17.888	−11.313	−34.977	1.00	27.59	C
ATOM	3716	CD1	ILE	A	438	17.174	−12.369	−37.922	1.00	27.82	C
ATOM	3717	HN	ILE	A	438	15.847	−10.028	−33.653	1.00	0.00	H
ATOM	3718	HA	ILE	A	438	14.484	−12.089	−34.929	1.00	27.84	H
ATOM	3719	HB	ILE	A	438	16.132	−10.736	−36.056	1.00	27.84	H
ATOM	3720	1HG1	ILE	A	438	17.172	−13.571	−36.155	1.00	27.84	H
ATOM	3721	2HG1	ILE	A	438	15.554	−13.135	−36.757	1.00	27.84	H
ATOM	3722	1HG2	ILE	A	438	18.565	−11.081	−35.799	1.00	27.84	H
ATOM	3723	2HG2	ILE	A	438	18.264	−12.178	−34.431	1.00	27.84	H
ATOM	3724	3HG2	ILE	A	438	17.827	−10.457	−34.304	1.00	27.84	H
ATOM	3725	1HD1	ILE	A	438	17.189	−13.233	−38.587	1.00	27.84	H
ATOM	3726	2HD1	ILE	A	438	18.192	−12.012	−37.768	1.00	27.84	H
ATOM	3727	3HD1	ILE	A	438	16.575	−11.576	−38.371	1.00	27.84	H
ATOM	3728	N	GLY	A	439	15.112	−14.368	−34.177	1.00	28.10	N
ATOM	3729	CA	GLY	A	439	15.406	−15.726	−33.741	1.00	28.11	C
ATOM	3730	C	GLY	A	439	16.870	−16.076	−33.983	1.00	27.85	C
ATOM	3731	O	GLY	A	439	17.353	−16.100	−35.114	1.00	27.73	O
ATOM	3732	HN	GLY	A	439	14.345	−14.232	−34.827	1.00	0.00	H
ATOM	3733	HA1	GLY	A	439	15.191	−15.812	−32.676	1.00	27.95	H
ATOM	3734	HA2	GLY	A	439	14.777	−16.422	−34.296	1.00	27.95	H
ATOM	3735	N	SER	A	440	17.580	−16.295	−32.887	1.00	42.44	N

ATOM	3736	CA	SER	A	440	19.033	−16.396	−32.800	1.00	42.21	C
ATOM	3737	C	SER	A	440	19.416	−17.476	−31.782	1.00	42.22	C
ATOM	3738	O	SER	A	440	18.564	−17.853	−30.966	1.00	42.34	O
ATOM	3739	CB	SER	A	440	19.610	−15.037	−32.418	1.00	42.01	C
ATOM	3740	OG	SER	A	440	19.157	−14.633	−31.145	1.00	42.03	O
ATOM	3741	HN	SER	A	440	17.090	−16.249	−32.001	1.00	0.00	H
ATOM	3742	HG	SER	A	440	19.886	−14.136	−30.718	1.00	0.00	H
ATOM	3743	HA	SER	A	440	19.424	−16.681	−33.777	1.00	42.21	H
ATOM	3744	HB1	SER	A	440	20.698	−15.103	−32.404	1.00	42.21	H
ATOM	3745	HB2	SER	A	440	19.300	−14.298	−33.157	1.00	42.21	H
ATOM	3746	N	PRO	A	441	20.635	−18.048	−31.852	1.00	27.43	N
ATOM	3747	CA	PRO	A	441	20.950	−19.217	−31.048	1.00	27.40	C
ATOM	3748	C	PRO	A	441	20.926	−18.900	−29.550	1.00	27.28	C
ATOM	3749	O	PRO	A	441	21.413	−17.858	−29.105	1.00	27.10	O
ATOM	3750	CB	PRO	A	441	22.297	−19.750	−31.537	1.00	27.22	C
ATOM	3751	CG	PRO	A	441	22.463	−19.121	−32.920	1.00	27.30	C
ATOM	3752	CD	PRO	A	441	21.701	−17.800	−32.812	1.00	27.32	C
ATOM	3753	HA	PRO	A	441	20.194	−19.977	−31.245	1.00	27.29	H
ATOM	3754	HB1	PRO	A	441	23.102	−19.439	−30.871	1.00	27.29	H
ATOM	3755	HB2	PRO	A	441	22.281	−20.838	−31.605	1.00	27.29	H
ATOM	3756	HG1	PRO	A	441	23.515	−18.945	−33.143	1.00	27.29	H
ATOM	3757	HG2	PRO	A	441	22.029	−19.758	−33.691	1.00	27.29	H
ATOM	3758	HD1	PRO	A	441	22.359	−17.008	−32.455	1.00	27.29	H
ATOM	3759	HD2	PRO	A	441	21.285	−17.519	−33.780	1.00	27.29	H
ATOM	3760	N	SER	A	442	20.348	−19.803	−28.768	1.00	27.41	N
ATOM	3761	CA	SER	A	442	19.942	−19.567	−27.377	1.00	27.34	C
ATOM	3762	C	SER	A	442	20.324	−20.732	−26.458	1.00	27.40	C

ATOM 3763 O SER A 442 20.978 −21.674 −26.888 1.00 27.55 O
ATOM 3764 CB SER A 442 18.436 −19.250 −27.352 1.00 27.52 C
ATOM 3765 OG SER A 442 17.650 −20.206 −28.043 1.00 27.81 O
ATOM 3766 HN SER A 442 19.967 −20.631 −29.219 1.00 0.00 H
ATOM 3767 HG SER A 442 17.740 −21.064 −27.623 1.00 0.00 H
ATOM 3768 HA SER A 442 20.471 −18.682 −27.025 1.00 27.50 H
ATOM 3769 HB1 SER A 442 18.107 −19.217 −26.313 1.00 27.51 H
ATOM 3770 HB2 SER A 442 18.280 −18.275 −27.814 1.00 27.51 H
ATOM 3771 N LYS A 443 20.030 −20.626 −25.163 1.00 27.29 N
ATOM 3772 CA LYS A 443 20.440 −21.590 −24.132 1.00 27.31 C
ATOM 3773 C LYS A 443 19.489 −21.507 −22.944 1.00 27.35 C
ATOM 3774 O LYS A 443 19.059 −20.413 −22.584 1.00 27.20 O
ATOM 3775 CB LYS A 443 21.907 −21.288 −23.770 1.00 27.03 C
ATOM 3776 CG LYS A 443 22.463 −21.918 −22.488 1.00 26.89 C
ATOM 3777 CD LYS A 443 23.974 −21.646 −22.417 1.00 26.60 C
ATOM 3778 CE LYS A 443 24.557 −21.807 −21.009 1.00 26.37 C
ATOM 3779 NZ LYS A 443 26.030 −21.700 −21.039 1.00 26.08 N1+
ATOM 3780 HN LYS A 443 19.565 −19.778 −24.848 1.00 0.00 H
ATOM 3781 HZ1 LYS A 443 26.447 −22.624 −21.121 1.00 0.00 H
ATOM 3782 HZ2 LYS A 443 26.382 −21.071 −21.756 1.00 0.00 H
ATOM 3783 HZ3 LYS A 443 26.420 −21.388 −20.160 1.00 0.00 H
ATOM 3784 HA LYS A 443 20.385 −22.594 −24.553 1.00 26.90 H
ATOM 3785 HB1 LYS A 443 22.525 −21.633 −24.598 1.00 26.90 H
ATOM 3786 HB2 LYS A 443 22.003 −20.207 −23.671 1.00 26.90 H
ATOM 3787 HG1 LYS A 443 21.971 −21.478 −21.621 1.00 26.90 H
ATOM 3788 HG2 LYS A 443 22.285 −22.993 −22.501 1.00 26.90 H
ATOM 3789 HD1 LYS A 443 24.482 −22.344 −23.083 1.00 26.90 H

ATOM	3790	HD2	LYS	A	443	24.157	−20.625	−22.752	1.00	26.90	H
ATOM	3791	HE1	LYS	A	443	24.155	−21.026	−20.364	1.00	26.90	H
ATOM	3792	HE2	LYS	A	443	24.277	−22.784	−20.615	1.00	26.90	H
ATOM	3793	N	ILE	A	444	19.182	−22.642	−22.323	1.00	27.58	N
ATOM	3794	CA	ILE	A	444	18.385	−22.719	−21.089	1.00	27.62	C
ATOM	3795	C	ILE	A	444	19.063	−23.682	−20.113	1.00	27.48	C
ATOM	3796	O	ILE	A	444	19.596	−24.700	−20.544	1.00	27.53	O
ATOM	3797	CB	ILE	A	444	16.902	−23.058	−21.380	1.00	28.00	C
ATOM	3798	CG1	ILE	A	444	16.067	−22.967	−20.080	1.00	28.08	C
ATOM	3799	CG2	ILE	A	444	16.719	−24.417	−22.072	1.00	28.23	C
ATOM	3800	CD1	ILE	A	444	14.549	−23.078	−20.279	1.00	28.47	C
ATOM	3801	HN	ILE	A	444	19.635	−23.499	−22.632	1.00	0.00	H
ATOM	3802	HA	ILE	A	444	18.405	−21.730	−20.631	1.00	27.87	H
ATOM	3803	HB	ILE	A	444	16.524	−22.295	−22.061	1.00	27.87	H
ATOM	3804	1HG1	ILE	A	444	16.382	−23.774	−19.418	1.00	27.87	H
ATOM	3805	2HG1	ILE	A	444	16.277	−22.007	−19.609	1.00	27.87	H
ATOM	3806	1HG2	ILE	A	444	15.658	−24.596	−22.248	1.00	27.87	H
ATOM	3807	2HG2	ILE	A	444	17.119	−25.206	−21.435	1.00	27.87	H
ATOM	3808	3HG2	ILE	A	444	17.250	−24.415	−23.024	1.00	27.87	H
ATOM	3809	1HD1	ILE	A	444	14.049	−23.003	−19.313	1.00	27.87	H
ATOM	3810	2HD1	ILE	A	444	14.311	−24.038	−20.737	1.00	27.87	H
ATOM	3811	3HD1	ILE	A	444	14.207	−22.272	−20.928	1.00	27.87	H
ATOM	3812	N	TYR	A	445	19.190	−23.295	−18.840	1.00	27.31	N
ATOM	3813	CA	TYR	A	445	20.089	−23.972	−17.897	1.00	27.14	C
ATOM	3814	C	TYR	A	445	19.654	−23.806	−16.429	1.00	27.08	C
ATOM	3815	O	TYR	A	445	19.313	−22.713	−15.976	1.00	27.00	O
ATOM	3816	CB	TYR	A	445	21.533	−23.505	−18.147	1.00	26.82	C

```
ATOM   3817  CG  TYR A 445      21.815 −22.020 −17.985  1.00 26.58           C
ATOM   3818  CD1 TYR A 445      22.528 −21.574 −16.862  1.00 26.32           C
ATOM   3819  CD2 TYR A 445      21.433 −21.089 −18.973  1.00 26.63           C
ATOM   3820  CE1 TYR A 445      22.873 −20.219 −16.732  1.00 26.13           C
ATOM   3821  CE2 TYR A 445      21.788 −19.730 −18.857  1.00 26.45           C
ATOM   3822  CZ  TYR A 445      22.519 −19.293 −17.733  1.00 26.21           C
ATOM   3823  OH  TYR A 445      22.879 −17.990 −17.604  1.00 26.05           O
ATOM   3824  HN  TYR A 445      18.831 −22.380 −18.577  1.00  0.00           H
ATOM   3825  HH  TYR A 445      23.598 −17.760 −18.221  1.00  0.00           H
ATOM   3826  HA  TYR A 445      20.051 −25.037 −18.125  1.00 26.64           H
ATOM   3827  HB1 TYR A 445      22.177 −24.040 −17.449  1.00 26.64           H
ATOM   3828  HB2 TYR A 445      21.795 −23.780 −19.169  1.00 26.64           H
ATOM   3829  HD1 TYR A 445      22.816 −22.283 −16.086  1.00 26.64           H
ATOM   3830  HD2 TYR A 445      20.857 −21.424 −19.836  1.00 26.64           H
ATOM   3831  HE1 TYR A 445      23.418 −19.881 −15.851  1.00 26.64           H
ATOM   3832  HE2 TYR A 445      21.499 −19.020 −19.632  1.00 26.64           H
ATOM   3833  N   ASN A 446      19.658 −24.910 −15.672  1.00 48.90           N
ATOM   3834  CA  ASN A 446      19.094 −25.023 −14.315  1.00 48.86           C
ATOM   3835  C   ASN A 446      19.984 −24.412 −13.205  1.00 48.47           C
ATOM   3836  O   ASN A 446      20.363 −25.095 −12.251  1.00 48.24           O
ATOM   3837  CB  ASN A 446      18.737 −26.503 −14.042  1.00 49.01           C
ATOM   3838  CG  ASN A 446      17.537 −27.004 −14.826  1.00 49.43           C
ATOM   3839  OD1 ASN A 446      16.602 −26.276 −15.103  1.00 49.56           O
ATOM   3840  ND2 ASN A 446      17.488 −28.277 −15.142  1.00 49.67           N
ATOM   3841  HN  ASN A 446      19.974 −25.762 −16.111  1.00  0.00           H
ATOM   3842 1HD2 ASN A 446      18.243 −28.889 −14.904  1.00  0.00           H
ATOM   3843 2HD2 ASN A 446      16.638 −28.603 −15.579  1.00  0.00           H
```

ATOM 3844 HA ASN A 446 18.157 −24.465 −14.313 1.00 49.02 H

ATOM 3845 HB1 ASN A 446 19.598 −27.117 −14.304 1.00 49.02 H

ATOM 3846 HB2 ASN A 446 18.522 −26.613 −12.979 1.00 49.02 H

ATOM 3847 N SER A 447 20.320 −23.122 −13.300 1.00 26.66 N

ATOM 3848 CA SER A 447 21.146 −22.438 −12.292 1.00 26.31 C

ATOM 3849 C SER A 447 20.483 −22.451 −10.914 1.00 26.35 C

ATOM 3850 O SER A 447 19.441 −21.827 −10.715 1.00 26.61 O

ATOM 3851 CB SER A 447 21.423 −20.979 −12.658 1.00 26.16 C

ATOM 3852 OG SER A 447 22.041 −20.887 −13.917 1.00 26.11 O

ATOM 3853 HN SER A 447 19.959 −22.596 −14.087 1.00 0.00 H

ATOM 3854 HG SER A 447 21.383 −20.965 −14.616 1.00 0.00 H

ATOM 3855 HA SER A 447 22.100 −22.960 −12.219 1.00 26.37 H

ATOM 3856 HB1 SER A 447 20.480 −20.432 −12.682 1.00 26.37 H

ATOM 3857 HB2 SER A 447 22.079 −20.541 −11.906 1.00 26.37 H

ATOM 3858 N LEU A 448 21.168 −23.003 −9.908 1.00 26.11 N

ATOM 3859 CA LEU A 448 20.837 −22.810 −8.487 1.00 26.08 C

ATOM 3860 C LEU A 448 19.348 −23.088 −8.158 1.00 26.46 C

ATOM 3861 O LEU A 448 18.684 −22.270 −7.524 1.00 26.55 O

ATOM 3862 CB LEU A 448 21.321 −21.415 −8.024 1.00 25.87 C

ATOM 3863 CG LEU A 448 22.781 −21.057 −8.365 1.00 25.61 C

ATOM 3864 CD1 LEU A 448 23.047 −19.604 −7.985 1.00 25.35 C

ATOM 3865 CD2 LEU A 448 23.780 −21.952 −7.631 1.00 25.44 C

ATOM 3866 HN LEU A 448 22.026 −23.483 −10.137 1.00 0.00 H

ATOM 3867 HA LEU A 448 21.421 −23.543 −7.929 1.00 25.93 H

ATOM 3868 HB1 LEU A 448 20.678 −20.668 −8.489 1.00 25.93 H

ATOM 3869 HB2 LEU A 448 21.211 −21.367 −6.941 1.00 25.93 H

ATOM 3870 HG LEU A 448 22.929 −21.172 −9.439 1.00 25.93 H

```
ATOM   3871 1HD1 LEU A 448      24.079 −19.348  −8.225  1.00 25.93           H
ATOM   3872 2HD1 LEU A 448      22.880 −19.471  −6.916  1.00 25.93           H
ATOM   3873 3HD1 LEU A 448      22.373 −18.953  −8.542  1.00 25.93           H
ATOM   3874 1HD2 LEU A 448      24.795 −21.663  −7.902  1.00 25.93           H
ATOM   3875 2HD2 LEU A 448      23.612 −22.992  −7.912  1.00 25.93           H
ATOM   3876 3HD2 LEU A 448      23.646 −21.840  −6.555  1.00 25.93           H
ATOM   3877  N   GLY A 449      18.781 −24.152  −8.742  1.00 26.72           N
ATOM   3878  CA  GLY A 449      17.403 −24.604  −8.489  1.00 27.11           C
ATOM   3879  C   GLY A 449      16.291 −23.976  −9.343  1.00 27.46           C
ATOM   3880  O   GLY A 449      15.135 −24.330  −9.145  1.00 27.80           O
ATOM   3881  HN  GLY A 449      19.359 −24.719  −9.345  1.00  0.00           H
ATOM   3882  HA1 GLY A 449      17.375 −25.681  −8.655  1.00 27.27           H
ATOM   3883  HA2 GLY A 449      17.174 −24.391  −7.445  1.00 27.27           H
ATOM   3884  N   GLN A 450      16.608 −23.114 −10.314  1.00 27.37           N
ATOM   3885  CA  GLN A 450      15.624 −22.408 −11.156  1.00 27.66           C
ATOM   3886  C   GLN A 450      16.215 −22.161 −12.562  1.00 27.50           C
ATOM   3887  O   GLN A 450      17.385 −21.771 −12.643  1.00 27.16           O
ATOM   3888  CB  GLN A 450      15.210 −21.115 −10.416  1.00 27.78           C
ATOM   3889  CG  GLN A 450      14.780 −19.906 −11.266  1.00 27.81           C
ATOM   3890  CD  GLN A 450      13.436 −20.042 −11.966  1.00 28.20           C
ATOM   3891  OE1 GLN A 450      12.672 −20.968 −11.755  1.00 28.43           O
ATOM   3892  NE2 GLN A 450      13.119 −19.091 −12.809  1.00 28.29           N
ATOM   3893  HN  GLN A 450      17.581 −22.865 −10.440  1.00  0.00           H
ATOM   3894 1HE2 GLN A 450      13.765 −18.350 −13.000  1.00  0.00           H
ATOM   3895 2HE2 GLN A 450      12.212 −19.109 −13.265  1.00  0.00           H
ATOM   3896  HA  GLN A 450      14.743 −23.042 −11.260  1.00 27.80           H
ATOM   3897  HB1 GLN A 450      14.374 −21.368  −9.764  1.00 27.80           H
```

ATOM 3898 HB2 GLN A 450 16.061 −20.801 −9.811 1.00 27.80 H
ATOM 3899 HG1 GLN A 450 14.731 −19.036 −10.611 1.00 27.80 H
ATOM 3900 HG2 GLN A 450 15.541 −19.745 −12.029 1.00 27.80 H
ATOM 3901 N PRO A 451 15.478 −22.358 −13.675 1.00 27.73 N
ATOM 3902 CA PRO A 451 16.040 −22.151 −15.006 1.00 27.60 C
ATOM 3903 C PRO A 451 16.356 −20.681 −15.306 1.00 27.50 C
ATOM 3904 O PRO A 451 15.513 −19.795 −15.154 1.00 27.64 O
ATOM 3905 CB PRO A 451 15.046 −22.736 −16.012 1.00 27.91 C
ATOM 3906 CG PRO A 451 14.166 −23.658 −15.169 1.00 28.25 C
ATOM 3907 CD PRO A 451 14.169 −22.986 −13.798 1.00 28.15 C
ATOM 3908 HA PRO A 451 16.968 −22.719 −15.075 1.00 27.83 H
ATOM 3909 HB1 PRO A 451 14.451 −21.947 −16.472 1.00 27.83 H
ATOM 3910 HB2 PRO A 451 15.566 −23.299 −16.787 1.00 27.83 H
ATOM 3911 HG1 PRO A 451 13.156 −23.713 −15.575 1.00 27.83 H
ATOM 3912 HG2 PRO A 451 14.591 −24.660 −15.114 1.00 27.83 H
ATOM 3913 HD1 PRO A 451 13.380 −22.236 −13.740 1.00 27.83 H
ATOM 3914 HD2 PRO A 451 14.026 −23.726 −13.011 1.00 27.83 H
ATOM 3915 N VAL A 452 17.515 −20.472 −15.924 1.00 27.28 N
ATOM 3916 CA VAL A 452 17.921 −19.226 −16.585 1.00 27.17 C
ATOM 3917 C VAL A 452 17.888 −19.452 −18.093 1.00 27.20 C
ATOM 3918 O VAL A 452 18.285 −20.518 −18.568 1.00 27.13 O
ATOM 3919 CB VAL A 452 19.322 −18.783 −16.121 1.00 26.83 C
ATOM 3920 CG1 VAL A 452 19.739 −17.423 −16.695 1.00 26.82 C
ATOM 3921 CG2 VAL A 452 19.403 −18.657 −14.599 1.00 26.76 C
ATOM 3922 HN VAL A 452 18.123 −21.272 −16.060 1.00 0.00 H
ATOM 3923 HA VAL A 452 17.204 −18.445 −16.332 1.00 27.03 H
ATOM 3924 HB VAL A 452 20.046 −19.531 −16.445 1.00 27.03 H

ATOM 3925 1HG1 VAL A 452 20.734 −17.165 −16.333 1.00 27.03 H

ATOM 3926 2HG1 VAL A 452 19.028 −16.660 −16.377 1.00 27.03 H

ATOM 3927 3HG1 VAL A 452 19.752 −17.476 −17.784 1.00 27.03 H

ATOM 3928 1HG2 VAL A 452 20.407 −18.343 −14.313 1.00 27.03 H

ATOM 3929 2HG2 VAL A 452 19.181 −19.621 −14.142 1.00 27.03 H

ATOM 3930 3HG2 VAL A 452 18.680 −17.917 −14.256 1.00 27.03 H

ATOM 3931 N PHE A 453 17.418 −18.464 −18.848 1.00 27.32 N

ATOM 3932 CA PHE A 453 17.481 −18.426 −20.305 1.00 27.39 C

ATOM 3933 C PHE A 453 18.518 −17.397 −20.778 1.00 27.15 C

ATOM 3934 O PHE A 453 18.691 −16.342 −20.165 1.00 27.02 O

ATOM 3935 CB PHE A 453 16.084 −18.132 −20.869 1.00 27.70 C

ATOM 3936 CG PHE A 453 15.937 −18.457 −22.344 1.00 27.81 C

ATOM 3937 CD1 PHE A 453 15.575 −19.760 −22.728 1.00 28.03 C

ATOM 3938 CD2 PHE A 453 16.180 −17.485 −23.333 1.00 27.70 C

ATOM 3939 CE1 PHE A 453 15.489 −20.107 −24.088 1.00 28.15 C

ATOM 3940 CE2 PHE A 453 16.076 −17.827 −24.694 1.00 27.80 C

ATOM 3941 CZ PHE A 453 15.729 −19.136 −25.072 1.00 28.02 C

ATOM 3942 HN PHE A 453 17.135 −17.608 −18.380 1.00 0.00 H

ATOM 3943 HA PHE A 453 17.791 −19.409 −20.658 1.00 27.64 H

ATOM 3944 HB1 PHE A 453 15.358 −18.724 −20.313 1.00 27.64 H

ATOM 3945 HB2 PHE A 453 15.873 −17.072 −20.728 1.00 27.64 H

ATOM 3946 HD1 PHE A 453 15.359 −20.508 −21.965 1.00 27.64 H

ATOM 3947 HD2 PHE A 453 16.448 −16.469 −23.045 1.00 27.64 H

ATOM 3948 HE1 PHE A 453 15.237 −21.127 −24.377 1.00 27.64 H

ATOM 3949 HE2 PHE A 453 16.265 −17.074 −25.459 1.00 27.64 H

ATOM 3950 HZ PHE A 453 15.647 −19.396 −26.127 1.00 27.64 H

ATOM 3951 N TYR A 454 19.100 −17.638 −21.950 1.00 27.11 N

ATOM 3952 CA TYR A 454 19.944 −16.706 −22.695 1.00 26.94 C

ATOM 3953 C TYR A 454 19.599 −16.759 −24.189 1.00 27.06 C

ATOM 3954 O TYR A 454 19.381 −17.837 −24.744 1.00 27.13 O

ATOM 3955 CB TYR A 454 21.429 −17.005 −22.415 1.00 26.67 C

ATOM 3956 CG TYR A 454 22.399 −16.590 −23.511 1.00 26.56 C

ATOM 3957 CD1 TYR A 454 23.106 −15.372 −23.449 1.00 26.46 C

ATOM 3958 CD2 TYR A 454 22.575 −17.439 −24.619 1.00 26.58 C

ATOM 3959 CE1 TYR A 454 24.017 −15.034 −24.473 1.00 26.38 C

ATOM 3960 CE2 TYR A 454 23.468 −17.100 −25.643 1.00 26.49 C

ATOM 3961 CZ TYR A 454 24.214 −15.911 −25.562 1.00 26.39 C

ATOM 3962 OH TYR A 454 25.143 −15.666 −26.522 1.00 26.32 O

ATOM 3963 HN TYR A 454 18.900 −18.531 −22.395 1.00 0.00 H

ATOM 3964 HH TYR A 454 25.482 −16.507 −26.837 1.00 0.00 H

ATOM 3965 HA TYR A 454 19.731 −15.699 −22.336 1.00 26.67 H

ATOM 3966 HB1 TYR A 454 21.710 −16.481 −21.502 1.00 26.67 H

ATOM 3967 HB2 TYR A 454 21.532 −18.080 −22.265 1.00 26.67 H

ATOM 3968 HD1 TYR A 454 22.949 −14.692 −22.612 1.00 26.67 H

ATOM 3969 HD2 TYR A 454 22.011 −18.370 −24.681 1.00 26.67 H

ATOM 3970 HE1 TYR A 454 24.569 −14.095 −24.423 1.00 26.67 H

ATOM 3971 HE2 TYR A 454 23.585 −17.759 −26.504 1.00 26.67 H

ATOM 3972 N GLN A 455 19.701 −15.618 −24.869 1.00 27.06 N

ATOM 3973 CA GLN A 455 19.637 −15.494 −26.323 1.00 27.15 C

ATOM 3974 C GLN A 455 20.860 −14.718 −26.822 1.00 26.94 C

ATOM 3975 O GLN A 455 21.095 −13.594 −26.386 1.00 26.82 O

ATOM 3976 CB GLN A 455 18.308 −14.816 −26.705 1.00 27.36 C

ATOM 3977 CG GLN A 455 18.171 −14.571 −28.214 1.00 27.48 C

ATOM 3978 CD GLN A 455 16.823 −13.982 −28.630 1.00 27.72 C

ATOM 3979 OE1 GLN A 455 16.031 −13.501 −27.829 1.00 27.83 O
ATOM 3980 NE2 GLN A 455 16.584 −13.846 −29.914 1.00 27.80 N
ATOM 3981 HN GLN A 455 19.973 −14.785 −24.352 1.00 0.00 H
ATOM 3982 1HE2 GLN A 455 17.261 −14.193 −30.591 1.00 0.00 H
ATOM 3983 2HE2 GLN A 455 15.667 −13.558 −30.205 1.00 0.00 H
ATOM 3984 HA GLN A 455 19.656 −16.493 −26.759 1.00 27.35 H
ATOM 3985 HB1 GLN A 455 17.487 −15.455 −26.381 1.00 27.35 H
ATOM 3986 HB2 GLN A 455 18.247 −13.857 −26.191 1.00 27.35 H
ATOM 3987 HG1 GLN A 455 18.956 −13.880 −28.521 1.00 27.35 H
ATOM 3988 HG2 GLN A 455 18.302 −15.523 −28.729 1.00 27.35 H
ATOM 3989 N ALA A 456 21.560 −15.232 −27.835 1.00 26.92 N
ATOM 3990 CA ALA A 456 22.638 −14.482 −28.467 1.00 26.76 C
ATOM 3991 C ALA A 456 22.082 −13.266 −29.225 1.00 26.83 C
ATOM 3992 O ALA A 456 21.237 −13.405 −30.113 1.00 27.02 O
ATOM 3993 CB ALA A 456 23.439 −15.415 −29.374 1.00 26.74 C
ATOM 3994 HN ALA A 456 21.343 −16.157 −28.192 1.00 0.00 H
ATOM 3995 HA ALA A 456 23.303 −14.118 −27.683 1.00 26.85 H
ATOM 3996 HB1 ALA A 456 24.246 −14.857 −29.848 1.00 26.85 H
ATOM 3997 HB2 ALA A 456 22.784 −15.828 −30.141 1.00 26.85 H
ATOM 3998 HB3 ALA A 456 23.859 −16.227 −28.781 1.00 26.85 H
ATOM 3999 N SER A 457 22.593 −12.080 −28.904 1.00 57.19 N
ATOM 4000 CA SER A 457 22.084 −10.776 −29.348 1.00 57.25 C
ATOM 4001 C SER A 457 22.418 −10.437 −30.815 1.00 57.25 C
ATOM 4002 O SER A 457 23.145 −9.493 −31.104 1.00 57.14 O
ATOM 4003 CB SER A 457 22.608 −9.713 −28.376 1.00 57.13 C
ATOM 4004 OG SER A 457 22.256 −10.059 −27.049 1.00 57.13 O
ATOM 4005 HN SER A 457 23.167 −12.049 −28.073 1.00 0.00 H

ATOM 4006 HG SER A 457 22.183 −9.229 −26.529 1.00 0.00 H

ATOM 4007 HA SER A 457 20.998 −10.799 −29.255 1.00 57.18 H

ATOM 4008 HB1 SER A 457 23.693 −9.652 −28.458 1.00 57.18 H

ATOM 4009 HB2 SER A 457 22.169 −8.747 −28.626 1.00 57.18 H

ATOM 4010 N TYR A 458 21.943 −11.249 −31.762 1.00 26.89 N

ATOM 4011 CA TYR A 458 22.236 −11.165 −33.204 1.00 26.89 C

ATOM 4012 C TYR A 458 21.496 −10.041 −33.968 1.00 26.95 C

ATOM 4013 O TYR A 458 21.383 −10.106 −35.191 1.00 26.95 O

ATOM 4014 CB TYR A 458 22.029 −12.550 −33.847 1.00 27.01 C

ATOM 4015 CG TYR A 458 23.259 −13.434 −33.823 1.00 26.91 C

ATOM 4016 CD1 TYR A 458 24.161 −13.391 −34.904 1.00 26.84 C

ATOM 4017 CD2 TYR A 458 23.505 −14.296 −32.740 1.00 26.88 C

ATOM 4018 CE1 TYR A 458 25.306 −14.211 −34.903 1.00 26.77 C

ATOM 4019 CE2 TYR A 458 24.648 −15.120 −32.736 1.00 26.80 C

ATOM 4020 CZ TYR A 458 25.552 −15.076 −33.817 1.00 26.75 C

ATOM 4021 OH TYR A 458 26.658 −15.866 −33.801 1.00 26.68 O

ATOM 4022 HN TYR A 458 21.406 −12.045 −31.440 1.00 0.00 H

ATOM 4023 HH TYR A 458 27.137 −15.798 −34.632 1.00 0.00 H

ATOM 4024 HA TYR A 458 23.300 −10.944 −33.288 1.00 26.86 H

ATOM 4025 HB1 TYR A 458 21.230 −13.061 −33.309 1.00 26.86 H

ATOM 4026 HB2 TYR A 458 21.733 −12.404 −34.886 1.00 26.86 H

ATOM 4027 HD1 TYR A 458 23.972 −12.722 −35.743 1.00 26.86 H

ATOM 4028 HD2 TYR A 458 22.810 −14.327 −31.901 1.00 26.86 H

ATOM 4029 HE1 TYR A 458 26.002 −14.177 −35.741 1.00 26.86 H

ATOM 4030 HE2 TYR A 458 24.833 −15.791 −31.897 1.00 26.86 H

ATOM 4031 N SER A 459 21.010 −8.994 −33.299 1.00 36.02 N

ATOM 4032 CA SER A 459 20.677 −7.727 −33.971 1.00 36.09 C

ATOM 4033 C SER A 459 20.798 −6.541 −33.003 1.00 36.08 C

ATOM 4034 O SER A 459 21.832 −6.417 −32.353 1.00 35.99 O

ATOM 4035 CB SER A 459 19.353 −7.796 −34.761 1.00 36.27 C

ATOM 4036 OG SER A 459 18.171 −7.810 −33.982 1.00 36.38 O

ATOM 4037 HN SER A 459 21.242 −8.931 −32.318 1.00 0.00 H

ATOM 4038 HG SER A 459 17.454 −7.611 −34.606 1.00 0.00 H

ATOM 4039 HA SER A 459 21.457 −7.577 −34.718 1.00 36.14 H

ATOM 4040 HB1 SER A 459 19.310 −6.927 −35.418 1.00 36.14 H

ATOM 4041 HB2 SER A 459 19.369 −8.706 −35.361 1.00 36.14 H

ATOM 4042 N TRP A 460 19.880 −5.576 −33.060 1.00 27.19 N

ATOM 4043 CA TRP A 460 20.076 −4.195 −32.607 1.00 27.20 C

ATOM 4044 C TRP A 460 20.078 −3.988 −31.082 1.00 27.19 C

ATOM 4045 O TRP A 460 20.686 −3.028 −30.619 1.00 27.14 O

ATOM 4046 CB TRP A 460 19.001 −3.336 −33.282 1.00 27.38 C

ATOM 4047 CG TRP A 460 17.585 −3.776 −33.051 1.00 27.55 C

ATOM 4048 CD1 TRP A 460 16.830 −4.473 −33.930 1.00 27.66 C

ATOM 4049 CD2 TRP A 460 16.740 −3.570 −31.875 1.00 27.66 C

ATOM 4050 NE1 TRP A 460 15.603 −4.758 −33.363 1.00 27.84 N

ATOM 4051 CE2 TRP A 460 15.489 −4.219 −32.098 1.00 27.85 C

ATOM 4052 CE3 TRP A 460 16.891 −2.866 −30.659 1.00 27.63 C

ATOM 4053 CZ2 TRP A 460 14.461 −4.217 −31.144 1.00 28.01 C

ATOM 4054 CZ3 TRP A 460 15.861 −2.843 −29.701 1.00 27.79 C

ATOM 4055 CH2 TRP A 460 14.653 −3.525 −29.934 1.00 27.98 C

ATOM 4056 HN TRP A 460 19.040 −5.767 −33.587 1.00 0.00 H

ATOM 4057 HE1 TRP A 460 14.892 −5.302 −33.828 1.00 0.00 H

ATOM 4058 HA TRP A 460 21.043 −3.862 −32.983 1.00 27.58 H

ATOM 4059 HB1 TRP A 460 19.101 −2.317 −32.908 1.00 27.58 H

ATOM 4060 HB2 TRP A 460 19.186 −3.349 −34.356 1.00 27.58 H
ATOM 4061 HD1 TRP A 460 17.142 −4.765 −34.933 1.00 27.58 H
ATOM 4062 HE3 TRP A 460 17.820 −2.332 −30.460 1.00 27.58 H
ATOM 4063 HZ2 TRP A 460 13.527 −4.744 −31.337 1.00 27.58 H
ATOM 4064 HZ3 TRP A 460 16.000 −2.292 −28.771 1.00 27.58 H
ATOM 4065 HH2 TRP A 460 13.867 −3.517 −29.179 1.00 27.58 H
ATOM 4066 N ASP A 461 19.457 −4.875 −30.302 1.00 27.26 N
ATOM 4067 CA ASP A 461 19.566 −4.928 −28.832 1.00 27.24 C
ATOM 4068 C ASP A 461 20.871 −5.653 −28.449 1.00 27.02 C
ATOM 4069 O ASP A 461 20.862 −6.870 −28.265 1.00 26.96 O
ATOM 4070 CB ASP A 461 18.299 −5.613 −28.265 1.00 27.40 C
ATOM 4071 CG ASP A 461 18.248 −5.793 −26.737 1.00 27.43 C
ATOM 4072 OD1 ASP A 461 19.117 −5.276 −26.004 1.00 27.29 O
ATOM 4073 OD2 ASP A 461 17.266 −6.389 −26.226 1.00 27.62 O
ATOM 4074 HN ASP A 461 18.995 −5.644 −30.756 1.00 0.00 H
ATOM 4075 HA ASP A 461 19.610 −3.908 −28.450 1.00 27.28 H
ATOM 4076 HB1 ASP A 461 17.438 −5.013 −28.558 1.00 27.28 H
ATOM 4077 HB2 ASP A 461 18.225 −6.601 −28.719 1.00 27.28 H
ATOM 4078 N THR A 462 22.026 −4.993 −28.635 1.00 26.91 N
ATOM 4079 CA THR A 462 23.347 −5.671 −28.668 1.00 26.71 C
ATOM 4080 C THR A 462 23.848 −6.126 −27.296 1.00 26.62 C
ATOM 4081 O THR A 462 24.853 −6.842 −27.204 1.00 26.44 O
ATOM 4082 CB THR A 462 24.438 −4.816 −29.335 1.00 26.65 C
ATOM 4083 OG1 THR A 462 24.910 −3.772 −28.515 1.00 26.72 O
ATOM 4084 CG2 THR A 462 23.978 −4.224 −30.662 1.00 26.73 C
ATOM 4085 HN THR A 462 21.989 −4.007 −28.885 1.00 0.00 H
ATOM 4086 HG1 THR A 462 25.388 −4.172 −27.776 1.00 0.00 H

ATOM 4087 HA THR A 462 23.229 −6.567 −29.277 1.00 26.68 H
ATOM 4088 HB THR A 462 25.283 −5.472 −29.546 1.00 26.68 H
ATOM 4089 1HG2 THR A 462 24.783 −3.629 −31.094 1.00 26.68 H
ATOM 4090 2HG2 THR A 462 23.107 −3.590 −30.495 1.00 26.68 H
ATOM 4091 3HG2 THR A 462 23.714 −5.029 −31.348 1.00 26.68 H
ATOM 4092 N MET A 463 23.191 −5.654 −26.236 1.00 26.74 N
ATOM 4093 CA MET A 463 23.445 −6.013 −24.842 1.00 26.67 C
ATOM 4094 C MET A 463 23.123 −7.489 −24.591 1.00 26.61 C
ATOM 4095 O MET A 463 22.294 −8.077 −25.286 1.00 26.70 O
ATOM 4096 CB MET A 463 22.605 −5.113 −23.917 1.00 26.86 C
ATOM 4097 CG MET A 463 22.806 −3.613 −24.183 1.00 26.94 C
ATOM 4098 SD MET A 463 24.529 −3.038 −24.209 1.00 26.74 S
ATOM 4099 CE MET A 463 24.994 −3.363 −22.491 1.00 26.56 C
ATOM 4100 HN MET A 463 22.361 −5.113 −26.429 1.00 0.00 H
ATOM 4101 HA MET A 463 24.500 −5.845 −24.626 1.00 26.73 H
ATOM 4102 HB1 MET A 463 21.552 −5.352 −24.064 1.00 26.73 H
ATOM 4103 HB2 MET A 463 22.884 −5.322 −22.884 1.00 26.73 H
ATOM 4104 HG1 MET A 463 22.362 −3.383 −25.152 1.00 26.73 H
ATOM 4105 HG2 MET A 463 22.282 −3.062 −23.402 1.00 26.73 H
ATOM 4106 HE1 MET A 463 26.031 −3.066 −22.332 1.00 26.73 H
ATOM 4107 HE2 MET A 463 24.885 −4.427 −22.280 1.00 26.73 H
ATOM 4108 HE3 MET A 463 24.347 −2.793 −21.825 1.00 26.73 H
ATOM 4109 N ILE A 464 23.755 −8.113 −23.593 1.00 54.73 N
ATOM 4110 CA ILE A 464 23.518 −9.533 −23.294 1.00 54.68 C
ATOM 4111 C ILE A 464 22.059 −9.792 −22.871 1.00 54.89 C
ATOM 4112 O ILE A 464 21.601 −9.360 −21.812 1.00 55.00 O
ATOM 4113 CB ILE A 464 24.561 −10.088 −22.299 1.00 54.48 C

ATOM 4114 CG1 ILE A 464 24.378 −11.617 −22.164 1.00 54.44 C
ATOM 4115 CG2 ILE A 464 24.552 −9.373 −20.933 1.00 54.53 C
ATOM 4116 CD1 ILE A 464 25.534 −12.326 −21.448 1.00 54.22 C
ATOM 4117 HN ILE A 464 24.461 −7.605 −23.069 1.00 0.00 H
ATOM 4118 HA ILE A 464 23.667 −10.073 −24.229 1.00 54.62 H
ATOM 4119 HB ILE A 464 25.544 −9.922 −22.740 1.00 54.62 H
ATOM 4120 1HG1 ILE A 464 23.462 −11.804 −21.603 1.00 54.62 H
ATOM 4121 2HG1 ILE A 464 24.284 −12.040 −23.164 1.00 54.62 H
ATOM 4122 1HG2 ILE A 464 25.310 −9.815 −20.286 1.00 54.62 H
ATOM 4123 2HG2 ILE A 464 23.571 −9.483 −20.471 1.00 54.62 H
ATOM 4124 3HG2 ILE A 464 24.769 −8.314 −21.075 1.00 54.62 H
ATOM 4125 1HD1 ILE A 464 25.328 −13.395 −21.395 1.00 54.62 H
ATOM 4126 2HD1 ILE A 464 25.637 −11.926 −20.439 1.00 54.62 H
ATOM 4127 3HD1 ILE A 464 26.459 −12.162 −22.000 1.00 54.62 H
ATOM 4128 N LYS A 465 21.324 −10.557 −23.685 1.00 26.70 N
ATOM 4129 CA LYS A 465 19.965 −11.004 −23.362 1.00 26.91 C
ATOM 4130 C LYS A 465 20.021 −12.295 −22.544 1.00 26.86 C
ATOM 4131 O LYS A 465 20.094 −13.389 −23.097 1.00 26.83 O
ATOM 4132 CB LYS A 465 19.143 −11.166 −24.647 1.00 27.06 C
ATOM 4133 CG LYS A 465 18.958 −9.846 −25.414 1.00 27.19 C
ATOM 4134 CD LYS A 465 18.036 −10.048 −26.621 1.00 27.39 C
ATOM 4135 CE LYS A 465 16.576 −10.145 −26.167 1.00 27.64 C
ATOM 4136 NZ LYS A 465 15.732 −10.797 −27.187 1.00 27.83 N1+
ATOM 4137 HN LYS A 465 21.719 −10.837 −24.575 1.00 0.00 H
ATOM 4138 HZ1 LYS A 465 15.982 −10.487 −28.122 1.00 0.00 H
ATOM 4139 HZ2 LYS A 465 15.824 −11.811 −27.177 1.00 0.00 H
ATOM 4140 HZ3 LYS A 465 14.760 −10.538 −27.037 1.00 0.00 H

ATOM 4141 HA LYS A 465 19.491 −10.236 −22.751 1.00 27.16 H
ATOM 4142 HB1 LYS A 465 19.653 −11.877 −25.298 1.00 27.16 H
ATOM 4143 HB2 LYS A 465 18.159 −11.555 −24.383 1.00 27.16 H
ATOM 4144 HG1 LYS A 465 18.518 −9.103 −24.748 1.00 27.16 H
ATOM 4145 HG2 LYS A 465 19.930 −9.494 −25.761 1.00 27.16 H
ATOM 4146 HD1 LYS A 465 18.145 −9.204 −27.302 1.00 27.16 H
ATOM 4147 HD2 LYS A 465 18.314 −10.968 −27.135 1.00 27.16 H
ATOM 4148 HE1 LYS A 465 16.530 −10.726 −25.246 1.00 27.16 H
ATOM 4149 HE2 LYS A 465 16.196 −9.140 −25.983 1.00 27.16 H
ATOM 4150 N LEU A 466 19.926 −12.165 −21.222 1.00 26.86 N
ATOM 4151 CA LEU A 466 19.771 −13.278 −20.276 1.00 26.84 C
ATOM 4152 C LEU A 466 18.744 −12.944 −19.185 1.00 27.00 C
ATOM 4153 O LEU A 466 18.462 −11.769 −18.959 1.00 27.10 O
ATOM 4154 CB LEU A 466 21.144 −13.706 −19.709 1.00 26.55 C
ATOM 4155 CG LEU A 466 21.687 −12.914 −18.499 1.00 26.47 C
ATOM 4156 CD1 LEU A 466 23.035 −13.501 −18.074 1.00 26.18 C
ATOM 4157 CD2 LEU A 466 21.903 −11.426 −18.774 1.00 26.53 C
ATOM 4158 HN LEU A 466 19.902 −11.228 −20.845 1.00 0.00 H
ATOM 4159 HA LEU A 466 19.378 −14.125 −20.838 1.00 26.69 H
ATOM 4160 HB1 LEU A 466 21.063 −14.750 −19.407 1.00 26.69 H
ATOM 4161 HB2 LEU A 466 21.873 −13.615 −20.514 1.00 26.69 H
ATOM 4162 HG LEU A 466 20.985 −13.017 −17.671 1.00 26.69 H
ATOM 4163 1HD1 LEU A 466 23.421 −12.944 −17.220 1.00 26.69 H
ATOM 4164 2HD1 LEU A 466 23.740 −13.430 −18.902 1.00 26.69 H
ATOM 4165 3HD1 LEU A 466 22.905 −14.547 −17.796 1.00 26.69 H
ATOM 4166 1HD2 LEU A 466 22.285 −10.942 −17.875 1.00 26.69 H
ATOM 4167 2HD2 LEU A 466 20.956 −10.967 −19.059 1.00 26.69 H

ATOM 4168 3HD2 LEU A 466 22.622 −11.306 −19.584 1.00 26.69 H

ATOM 4169 N GLY A 467 18.201 −13.947 −18.498 1.00 27.06 N

ATOM 4170 CA GLY A 467 17.263 −13.753 −17.386 1.00 27.20 C

ATOM 4171 C GLY A 467 16.701 −15.063 −16.836 1.00 27.36 C

ATOM 4172 O GLY A 467 16.761 −16.093 −17.500 1.00 27.44 O

ATOM 4173 HN GLY A 467 18.392 −14.902 −18.793 1.00 0.00 H

ATOM 4174 HA1 GLY A 467 17.783 −13.234 −16.581 1.00 27.27 H

ATOM 4175 HA2 GLY A 467 16.433 −13.140 −17.737 1.00 27.27 H

ATOM 4176 N ASP A 468 16.158 −15.038 −15.620 1.00 27.42 N

ATOM 4177 CA ASP A 468 15.366 −16.158 −15.088 1.00 27.61 C

ATOM 4178 C ASP A 468 14.077 −16.373 −15.905 1.00 27.96 C

ATOM 4179 O ASP A 468 13.507 −15.413 −16.431 1.00 28.13 O

ATOM 4180 CB ASP A 468 15.066 −15.909 −13.599 1.00 27.64 C

ATOM 4181 CG ASP A 468 16.284 −16.202 −12.721 1.00 27.32 C

ATOM 4182 OD1 ASP A 468 16.769 −17.349 −12.790 1.00 27.07 O

ATOM 4183 OD2 ASP A 468 16.570 −15.445 −11.768 1.00 27.33 O

ATOM 4184 HN ASP A 468 16.061 −14.148 −15.159 1.00 0.00 H

ATOM 4185 HA ASP A 468 15.968 −17.063 −15.164 1.00 27.56 H

ATOM 4186 HB1 ASP A 468 14.777 −14.866 −13.466 1.00 27.56 H

ATOM 4187 HB2 ASP A 468 14.245 −16.556 −13.291 1.00 27.56 H

ATOM 4188 N VAL A 469 13.608 −17.621 −16.024 1.00 28.08 N

ATOM 4189 CA VAL A 469 12.295 −17.905 −16.636 1.00 28.43 C

ATOM 4190 C VAL A 469 11.166 −17.600 −15.648 1.00 28.70 C

ATOM 4191 O VAL A 469 11.154 −18.108 −14.529 1.00 28.67 O

ATOM 4192 CB VAL A 469 12.182 −19.337 −17.208 1.00 28.48 C

ATOM 4193 CG1 VAL A 469 13.321 −19.630 −18.194 1.00 28.26 C

ATOM 4194 CG2 VAL A 469 12.133 −20.456 −16.160 1.00 28.42 C

ATOM 4195 HN VAL A 469 14.120 −18.388 −15.598 1.00 0.00 H

ATOM 4196 HA VAL A 469 12.179 −17.218 −17.474 1.00 28.43 H

ATOM 4197 HB VAL A 469 11.250 −19.384 −17.771 1.00 28.43 H

ATOM 4198 1HG1 VAL A 469 13.216 −20.644 −18.580 1.00 28.43 H

ATOM 4199 2HG1 VAL A 469 14.279 −19.534 −17.682 1.00 28.43 H

ATOM 4200 3HG1 VAL A 469 13.278 −18.921 −19.020 1.00 28.43 H

ATOM 4201 1HG2 VAL A 469 12.054 −21.421 −16.661 1.00 28.43 H

ATOM 4202 2HG2 VAL A 469 11.268 −20.311 −15.513 1.00 28.43 H

ATOM 4203 3HG2 VAL A 469 13.043 −20.433 −15.560 1.00 28.43 H

ATOM 4204 N ASP A 470 10.173 −16.818 −16.063 1.00 39.49 N

ATOM 4205 CA ASP A 470 8.910 −16.665 −15.328 1.00 39.78 C

ATOM 4206 C ASP A 470 7.862 −17.700 −15.777 1.00 40.13 C

ATOM 4207 O ASP A 470 6.863 −17.916 −15.092 1.00 40.29 O

ATOM 4208 CB ASP A 470 8.352 −15.234 −15.444 1.00 39.91 C

ATOM 4209 CG ASP A 470 9.416 −14.160 −15.680 1.00 39.59 C

ATOM 4210 OD1 ASP A 470 9.761 −13.988 −16.869 1.00 39.27 O

ATOM 4211 OD2 ASP A 470 9.451 −13.186 −14.897 1.00 39.68 O

ATOM 4212 HN ASP A 470 10.232 −16.412 −16.993 1.00 0.00 H

ATOM 4213 HA ASP A 470 9.121 −16.849 −14.275 1.00 39.77 H

ATOM 4214 HB1 ASP A 470 7.650 −15.208 −16.278 1.00 39.77 H

ATOM 4215 HB2 ASP A 470 7.827 −14.997 −14.519 1.00 39.77 H

ATOM 4216 N THR A 471 8.035 −18.305 −16.959 1.00 29.72 N

ATOM 4217 CA THR A 471 7.220 −19.432 −17.452 1.00 30.01 C

ATOM 4218 C THR A 471 7.951 −20.164 −18.589 1.00 29.86 C

ATOM 4219 O THR A 471 8.837 −19.582 −19.218 1.00 29.67 O

ATOM 4220 CB THR A 471 5.803 −19.002 −17.913 1.00 30.44 C

ATOM 4221 OG1 THR A 471 5.441 −17.708 −17.480 1.00 30.39 O

ATOM 4222 CG2 THR A 471 4.748 −19.935 −17.320 1.00 30.66 C
ATOM 4223 HN THR A 471 8.834 −18.035 −17.523 1.00 0.00 H
ATOM 4224 HG1 THR A 471 5.708 −17.665 −16.547 1.00 0.00 H
ATOM 4225 HA THR A 471 7.100 −20.137 −16.629 1.00 30.11 H
ATOM 4226 HB THR A 471 5.750 −19.045 −19.001 1.00 30.11 H
ATOM 4227 1HG2 THR A 471 3.758 −19.622 −17.651 1.00 30.11 H
ATOM 4228 2HG2 THR A 471 4.796 −19.894 −16.232 1.00 30.11 H
ATOM 4229 3HG2 THR A 471 4.936 −20.956 −17.653 1.00 30.11 H
ATOM 4230 N VAL A 472 7.686 −21.466 −18.772 1.00 29.99 N
ATOM 4231 CA VAL A 472 8.402 −22.337 −19.738 1.00 29.91 C
ATOM 4232 C VAL A 472 7.494 −22.853 −20.860 1.00 30.22 C
ATOM 4233 O VAL A 472 7.849 −22.692 −22.024 1.00 30.11 O
ATOM 4234 CB VAL A 472 9.124 −23.493 −19.014 1.00 29.92 C
ATOM 4235 CG1 VAL A 472 9.828 −24.459 −19.977 1.00 29.85 C
ATOM 4236 CG2 VAL A 472 10.195 −22.941 −18.063 1.00 29.58 C
ATOM 4237 HN VAL A 472 6.951 −21.878 −18.221 1.00 0.00 H
ATOM 4238 HA VAL A 472 9.172 −21.725 −20.208 1.00 29.94 H
ATOM 4239 HB VAL A 472 8.392 −24.052 −18.431 1.00 29.94 H
ATOM 4240 1HG1 VAL A 472 10.317 −25.249 −19.407 1.00 29.94 H
ATOM 4241 2HG1 VAL A 472 10.574 −23.915 −20.557 1.00 29.94 H
ATOM 4242 3HG1 VAL A 472 9.094 −24.899 −20.652 1.00 29.94 H
ATOM 4243 1HG2 VAL A 472 10.696 −23.768 −17.560 1.00 29.94 H
ATOM 4244 2HG2 VAL A 472 9.725 −22.296 −17.321 1.00 29.94 H
ATOM 4245 3HG2 VAL A 472 10.926 −22.367 −18.632 1.00 29.94 H
ATOM 4246 N ASP A 473 6.255 −23.232 −20.532 1.00 51.41 N
ATOM 4247 CA ASP A 473 5.142 −23.327 −21.482 1.00 51.73 C
ATOM 4248 C ASP A 473 4.130 −22.195 −21.182 1.00 52.06 C

ATOM 4249 O ASP A 473 3.421 −22.291 −20.176 1.00 52.38 O

ATOM 4250 CB ASP A 473 4.431 −24.700 −21.402 1.00 51.95 C

ATOM 4251 CG ASP A 473 5.237 −25.949 −21.790 1.00 51.66 C

ATOM 4252 OD1 ASP A 473 6.480 −25.913 −21.896 1.00 51.33 O

ATOM 4253 OD2 ASP A 473 4.629 −27.043 −21.839 1.00 51.80 O

ATOM 4254 HN ASP A 473 6.017 −23.307 −19.560 1.00 0.00 H

ATOM 4255 HA ASP A 473 5.530 −23.192 −22.491 1.00 51.79 H

ATOM 4256 HB1 ASP A 473 4.099 −24.834 −20.372 1.00 51.79 H

ATOM 4257 HB2 ASP A 473 3.564 −24.656 −22.061 1.00 51.79 H

ATOM 4258 N PRO A 474 3.998 −21.149 −22.025 1.00 31.22 N

ATOM 4259 CA PRO A 474 4.957 −20.696 −23.039 1.00 30.90 C

ATOM 4260 C PRO A 474 6.233 −20.106 −22.405 1.00 30.47 C

ATOM 4261 O PRO A 474 6.238 −19.761 −21.225 1.00 30.41 O

ATOM 4262 CB PRO A 474 4.200 −19.625 −23.829 1.00 31.07 C

ATOM 4263 CG PRO A 474 3.339 −18.966 −22.752 1.00 31.32 C

ATOM 4264 CD PRO A 474 2.956 −20.143 −21.855 1.00 31.52 C

ATOM 4265 HA PRO A 474 5.220 −21.525 −23.697 1.00 30.99 H

ATOM 4266 HB1 PRO A 474 4.887 −18.907 −24.278 1.00 30.99 H

ATOM 4267 HB2 PRO A 474 3.582 −20.073 −24.607 1.00 30.99 H

ATOM 4268 HG1 PRO A 474 3.908 −18.219 −22.198 1.00 30.99 H

ATOM 4269 HG2 PRO A 474 2.455 −18.501 −23.187 1.00 30.99 H

ATOM 4270 HD1 PRO A 474 2.908 −19.823 −20.814 1.00 30.99 H

ATOM 4271 HD2 PRO A 474 1.990 −20.547 −22.156 1.00 30.99 H

ATOM 4272 N LEU A 475 7.292 −19.889 −23.193 1.00 30.19 N

ATOM 4273 CA LEU A 475 8.548 −19.352 −22.668 1.00 29.79 C

ATOM 4274 C LEU A 475 8.460 −17.831 −22.454 1.00 29.62 C

ATOM 4275 O LEU A 475 8.470 −17.048 −23.409 1.00 29.51 O

ATOM 4276 CB LEU A 475 9.727 −19.773 −23.562 1.00 29.57 C

ATOM 4277 CG LEU A 475 11.075 −19.184 −23.098 1.00 29.17 C

ATOM 4278 CD1 LEU A 475 11.530 −19.709 −21.734 1.00 29.09 C

ATOM 4279 CD2 LEU A 475 12.164 −19.495 −24.117 1.00 29.00 C

ATOM 4280 HN LEU A 475 7.242 −20.124 −24.174 1.00 0.00 H

ATOM 4281 HA LEU A 475 8.708 −19.806 −21.690 1.00 29.49 H

ATOM 4282 HB1 LEU A 475 9.801 −20.860 −23.550 1.00 29.49 H

ATOM 4283 HB2 LEU A 475 9.531 −19.433 −24.579 1.00 29.49 H

ATOM 4284 HG LEU A 475 10.970 −18.101 −23.030 1.00 29.49 H

ATOM 4285 1HD1 LEU A 475 12.484 −19.254 −21.469 1.00 29.49 H

ATOM 4286 2HD1 LEU A 475 11.645 −20.792 −21.781 1.00 29.49 H

ATOM 4287 3HD1 LEU A 475 10.785 −19.455 −20.980 1.00 29.49 H

ATOM 4288 1HD2 LEU A 475 13.110 −19.074 −23.778 1.00 29.49 H

ATOM 4289 2HD2 LEU A 475 11.897 −19.059 −25.080 1.00 29.49 H

ATOM 4290 3HD2 LEU A 475 12.265 −20.575 −24.224 1.00 29.49 H

ATOM 4291 N ARG A 476 8.621 −17.405 −21.201 1.00 29.61 N

ATOM 4292 CA ARG A 476 8.668 −15.999 −20.776 1.00 29.46 C

ATOM 4293 C ARG A 476 9.810 −15.802 −19.775 1.00 29.09 C

ATOM 4294 O ARG A 476 10.035 −16.637 −18.903 1.00 29.03 O

ATOM 4295 CB ARG A 476 7.262 −15.604 −20.274 1.00 29.73 C

ATOM 4296 CG ARG A 476 7.163 −14.381 −19.354 1.00 29.60 C

ATOM 4297 CD ARG A 476 7.777 −13.066 −19.870 1.00 29.61 C

ATOM 4298 NE ARG A 476 8.600 −12.473 −18.803 1.00 29.28 N

ATOM 4299 CZ ARG A 476 8.393 −11.370 −18.118 1.00 29.29 C

ATOM 4300 NH1 ARG A 476 7.639 −10.399 −18.545 1.00 29.59 N

ATOM 4301 NH2 ARG A 476 8.920 −11.253 −16.936 1.00 29.00 N

ATOM 4302 HN ARG A 476 8.672 −18.118 −20.474 1.00 0.00 H

ATOM 4303 HE ARG A 476 9.198 −13.126 −18.294 1.00 0.00 H
ATOM 4304 1HH1 ARG A 476 7.356 −10.416 −19.523 1.00 0.00 H
ATOM 4305 2HH1 ARG A 476 7.552 −9.560 −18.016 1.00 0.00 H
ATOM 4306 1HH2 ARG A 476 9.345 −12.100 −16.530 1.00 0.00 H
ATOM 4307 2HH2 ARG A 476 8.623 −10.545 −16.303 1.00 0.00 H
ATOM 4308 HA ARG A 476 8.889 −15.394 −21.655 1.00 29.39 H
ATOM 4309 HB1 ARG A 476 6.645 −15.405 −21.150 1.00 29.39 H
ATOM 4310 HB2 ARG A 476 6.857 −16.457 −19.730 1.00 29.39 H
ATOM 4311 HG1 ARG A 476 6.106 −14.198 −19.164 1.00 29.39 H
ATOM 4312 HG2 ARG A 476 7.663 −14.632 −18.418 1.00 29.39 H
ATOM 4313 HD1 ARG A 476 8.399 −13.270 −20.741 1.00 29.39 H
ATOM 4314 HD2 ARG A 476 6.981 −12.374 −20.145 1.00 29.39 H
ATOM 4315 N VAL A 477 10.613 −14.763 −20.018 1.00 28.85 N
ATOM 4316 CA VAL A 477 11.924 −14.529 −19.394 1.00 28.51 C
ATOM 4317 C VAL A 477 11.989 −13.105 −18.848 1.00 28.47 C
ATOM 4318 O VAL A 477 11.765 −12.147 −19.593 1.00 28.48 O
ATOM 4319 CB VAL A 477 13.061 −14.764 −20.417 1.00 28.28 C
ATOM 4320 CG1 VAL A 477 14.453 −14.578 −19.799 1.00 27.96 C
ATOM 4321 CG2 VAL A 477 13.002 −16.166 −21.039 1.00 28.42 C
ATOM 4322 HN VAL A 477 10.353 −14.131 −20.757 1.00 0.00 H
ATOM 4323 HA VAL A 477 12.048 −15.227 −18.566 1.00 28.42 H
ATOM 4324 HB VAL A 477 12.948 −14.035 −21.219 1.00 28.42 H
ATOM 4325 1HG1 VAL A 477 15.215 −14.754 −20.558 1.00 28.42 H
ATOM 4326 2HG1 VAL A 477 14.586 −15.286 −18.981 1.00 28.42 H
ATOM 4327 3HG1 VAL A 477 14.549 −13.561 −19.418 1.00 28.42 H
ATOM 4328 1HG2 VAL A 477 13.820 −16.283 −21.750 1.00 28.42 H
ATOM 4329 2HG2 VAL A 477 12.051 −16.295 −21.555 1.00 28.42 H

ATOM 4330 3HG2 VAL A 477 13.094 −16.917 −20.254 1.00 28.42 H
ATOM 4331 N GLN A 478 12.409 −12.939 −17.594 1.00 28.42 N
ATOM 4332 CA GLN A 478 12.701 −11.630 −17.008 1.00 28.38 C
ATOM 4333 C GLN A 478 14.114 −11.186 −17.397 1.00 28.05 C
ATOM 4334 O GLN A 478 15.071 −11.307 −16.628 1.00 27.83 O
ATOM 4335 CB GLN A 478 12.414 −11.589 −15.498 1.00 28.45 C
ATOM 4336 CG GLN A 478 12.926 −12.780 −14.676 1.00 28.17 C
ATOM 4337 CD GLN A 478 12.600 −12.608 −13.202 1.00 28.23 C
ATOM 4338 OE1 GLN A 478 13.440 −12.240 −12.394 1.00 28.04 O
ATOM 4339 NE2 GLN A 478 11.341 −12.699 −12.834 1.00 28.52 N
ATOM 4340 HN GLN A 478 12.590 −13.771 −17.037 1.00 0.00 H
ATOM 4341 1HE2 GLN A 478 10.622 −12.923 −13.530 1.00 0.00 H
ATOM 4342 2HE2 GLN A 478 11.133 −12.578 −11.863 1.00 0.00 H
ATOM 4343 HA GLN A 478 12.015 −10.922 −17.473 1.00 28.23 H
ATOM 4344 HB1 GLN A 478 12.875 −10.686 −15.097 1.00 28.23 H
ATOM 4345 HB2 GLN A 478 11.333 −11.535 −15.367 1.00 28.23 H
ATOM 4346 HG1 GLN A 478 12.455 −13.693 −15.041 1.00 28.23 H
ATOM 4347 HG2 GLN A 478 14.007 −12.856 −14.795 1.00 28.23 H
ATOM 4348 N TRP A 479 14.245 −10.732 −18.647 1.00 28.03 N
ATOM 4349 CA TRP A 479 15.504 −10.239 −19.202 1.00 27.76 C
ATOM 4350 C TRP A 479 16.131 −9.175 −18.297 1.00 27.66 C
ATOM 4351 O TRP A 479 15.516 −8.140 −18.027 1.00 27.86 O
ATOM 4352 CB TRP A 479 15.290 −9.624 −20.589 1.00 27.82 C
ATOM 4353 CG TRP A 479 14.655 −10.480 −21.637 1.00 27.93 C
ATOM 4354 CD1 TRP A 479 13.444 −10.248 −22.186 1.00 28.21 C
ATOM 4355 CD2 TRP A 479 15.197 −11.638 −22.346 1.00 27.79 C
ATOM 4356 NE1 TRP A 479 13.223 −11.134 −23.215 1.00 28.25 N

ATOM	4357	CE2	TRP	A	479	14.281	−11.998	−23.379	1.00	28.00	C
ATOM	4358	CE3	TRP	A	479	16.379	−12.403	−22.245	1.00	27.52	C
ATOM	4359	CZ2	TRP	A	479	14.544	−13.021	−24.297	1.00	27.97	C
ATOM	4360	CZ3	TRP	A	479	16.636	−13.464	−23.137	1.00	27.49	C
ATOM	4361	CH2	TRP	A	479	15.730	−13.761	−24.171	1.00	27.72	C
ATOM	4362	HN	TRP	A	479	13.437	−10.783	−19.256	1.00	0.00	H
ATOM	4363	HE1	TRP	A	479	12.414	−11.051	−23.832	1.00	0.00	H
ATOM	4364	HA	TRP	A	479	16.198	−11.075	−19.293	1.00	27.86	H
ATOM	4365	HB1	TRP	A	479	14.660	−8.744	−20.461	1.00	27.86	H
ATOM	4366	HB2	TRP	A	479	16.267	−9.320	−20.965	1.00	27.86	H
ATOM	4367	HD1	TRP	A	479	12.746	−9.475	−21.863	1.00	27.86	H
ATOM	4368	HE3	TRP	A	479	17.104	−12.170	−21.465	1.00	27.86	H
ATOM	4369	HZ2	TRP	A	479	13.838	−13.240	−25.098	1.00	27.86	H
ATOM	4370	HZ3	TRP	A	479	17.543	−14.058	−23.025	1.00	27.86	H
ATOM	4371	HH2	TRP	A	479	15.949	−14.565	−24.874	1.00	27.86	H
ATOM	4372	N	ARG	A	480	17.423	−9.323	−17.997	1.00	27.38	N
ATOM	4373	CA	ARG	A	480	18.202	−8.260	−17.361	1.00	27.28	C
ATOM	4374	C	ARG	A	480	18.344	−7.103	−18.347	1.00	27.35	C
ATOM	4375	O	ARG	A	480	18.833	−7.284	−19.465	1.00	27.32	O
ATOM	4376	CB	ARG	A	480	19.578	−8.760	−16.886	1.00	26.96	C
ATOM	4377	CG	ARG	A	480	19.602	−10.100	−16.125	1.00	26.85	C
ATOM	4378	CD	ARG	A	480	18.531	−10.307	−15.045	1.00	26.99	C
ATOM	4379	NE	ARG	A	480	18.764	−9.476	−13.856	1.00	26.84	N
ATOM	4380	CZ	ARG	A	480	18.156	−9.603	−12.693	1.00	26.92	C
ATOM	4381	NH1	ARG	A	480	17.198	−10.467	−12.496	1.00	27.17	N
ATOM	4382	NH2	ARG	A	480	18.486	−8.830	−11.706	1.00	26.78	N
ATOM	4383	HN	ARG	A	480	17.893	−10.170	−18.300	1.00	0.00	H

ATOM 4384 HE ARG A 480 19.579 −8.856 −13.866 1.00 0.00 H

ATOM 4385 1HH1 ARG A 480 16.963 −11.096 −13.240 1.00 0.00 H

ATOM 4386 2HH1 ARG A 480 16.833 −10.625 −11.578 1.00 0.00 H

ATOM 4387 1HH2 ARG A 480 19.139 −8.069 −11.927 1.00 0.00 H

ATOM 4388 2HH2 ARG A 480 18.040 −8.887 −10.818 1.00 0.00 H

ATOM 4389 HA ARG A 480 17.649 −7.903 −16.492 1.00 27.08 H

ATOM 4390 HB1 ARG A 480 20.212 −8.869 −17.766 1.00 27.08 H

ATOM 4391 HB2 ARG A 480 19.997 −7.999 −16.228 1.00 27.08 H

ATOM 4392 HG1 ARG A 480 19.487 −10.897 −16.859 1.00 27.08 H

ATOM 4393 HG2 ARG A 480 20.576 −10.187 −15.643 1.00 27.08 H

ATOM 4394 HD1 ARG A 480 17.558 −10.052 −15.464 1.00 27.08 H

ATOM 4395 HD2 ARG A 480 18.535 −11.355 −14.745 1.00 27.08 H

ATOM 4396 N ASN A 481 18.054 −5.883 −17.907 1.00 39.58 N

ATOM 4397 CA ASN A 481 18.405 −4.664 −18.644 1.00 39.63 C

ATOM 4398 C ASN A 481 19.880 −4.295 −18.407 1.00 39.36 C

ATOM 4399 O ASN A 481 20.185 −3.249 −17.843 1.00 39.37 O

ATOM 4400 CB ASN A 481 17.381 −3.558 −18.338 1.00 39.93 C

ATOM 4401 CG ASN A 481 15.995 −3.900 −18.867 1.00 40.20 C

ATOM 4402 OD1 ASN A 481 15.818 −4.607 −19.851 1.00 40.19 O

ATOM 4403 ND2 ASN A 481 14.959 −3.418 −18.221 1.00 40.44 N

ATOM 4404 HN ASN A 481 17.696 −5.791 −16.970 1.00 0.00 H

ATOM 4405 1HD2 ASN A 481 15.089 −2.844 −17.409 1.00 0.00 H

ATOM 4406 2HD2 ASN A 481 14.053 −3.684 −18.562 1.00 0.00 H

ATOM 4407 HA ASN A 481 18.306 −4.899 −19.704 1.00 39.84 H

ATOM 4408 HB1 ASN A 481 17.322 −3.423 −17.258 1.00 39.84 H

ATOM 4409 HB2 ASN A 481 17.716 −2.631 −18.803 1.00 39.84 H

ATOM 4410 N ASN A 482 20.765 −5.264 −18.670 1.00 27.02 N

ATOM	4411	CA	ASN	A	482	22.193	−5.176	−18.399	1.00	26.76	C
ATOM	4412	C	ASN	A	482	22.821	−4.008	−19.175	1.00	26.76	C
ATOM	4413	O	ASN	A	482	22.593	−3.875	−20.378	1.00	26.82	O
ATOM	4414	CB	ASN	A	482	22.844	−6.532	−18.740	1.00	26.54	C
ATOM	4415	CG	ASN	A	482	24.335	−6.559	−18.440	1.00	26.27	C
ATOM	4416	OD1	ASN	A	482	25.143	−5.925	−19.100	1.00	26.24	O
ATOM	4417	ND2	ASN	A	482	24.755	−7.296	−17.440	1.00	26.09	N
ATOM	4418	HN	ASN	A	482	20.426	−6.101	−19.126	1.00	0.00	H
ATOM	4419	1HD2	ASN	A	482	24.094	−7.747	−16.838	1.00	0.00	H
ATOM	4420	2HD2	ASN	A	482	25.715	−7.166	−17.171	1.00	0.00	H
ATOM	4421	HA	ASN	A	482	22.328	−4.990	−17.333	1.00	26.56	H
ATOM	4422	HB1	ASN	A	482	22.356	−7.310	−18.153	1.00	26.56	H
ATOM	4423	HB2	ASN	A	482	22.698	−6.732	−19.802	1.00	26.56	H
ATOM	4424	N	SER	A	483	23.633	−3.194	−18.499	1.00	30.82	N
ATOM	4425	CA	SER	A	483	24.206	−1.961	−19.054	1.00	30.88	C
ATOM	4426	C	SER	A	483	25.717	−2.041	−19.327	1.00	30.65	C
ATOM	4427	O	SER	A	483	26.358	−0.996	−19.424	1.00	30.70	O
ATOM	4428	CB	SER	A	483	23.839	−0.764	−18.164	1.00	31.07	C
ATOM	4429	OG	SER	A	483	24.672	−0.685	−17.025	1.00	30.95	O
ATOM	4430	HN	SER	A	483	23.784	−3.386	−17.512	1.00	0.00	H
ATOM	4431	HG	SER	A	483	25.550	−0.433	−17.341	1.00	0.00	H
ATOM	4432	HA	SER	A	483	23.722	−1.796	−20.017	1.00	30.84	H
ATOM	4433	HB1	SER	A	483	23.946	0.153	−18.744	1.00	30.84	H
ATOM	4434	HB2	SER	A	483	22.804	−0.870	−17.838	1.00	30.84	H
ATOM	4435	N	VAL	A	484	26.322	−3.240	−19.298	1.00	26.29	N
ATOM	4436	CA	VAL	A	484	27.792	−3.401	−19.234	1.00	26.09	C
ATOM	4437	C	VAL	A	484	28.392	−4.503	−20.120	1.00	25.96	C

ATOM 4438 O VAL A 484 29.519 −4.331 −20.575 1.00 25.91 O

ATOM 4439 CB VAL A 484 28.276 −3.578 −17.780 1.00 25.92 C

ATOM 4440 CG1 VAL A 484 27.994 −2.347 −16.913 1.00 26.08 C

ATOM 4441 CG2 VAL A 484 27.683 −4.806 −17.074 1.00 25.72 C

ATOM 4442 HN VAL A 484 25.741 −4.058 −19.142 1.00 0.00 H

ATOM 4443 HA VAL A 484 28.218 −2.461 −19.585 1.00 26.00 H

ATOM 4444 HB VAL A 484 29.357 −3.712 −17.812 1.00 26.00 H

ATOM 4445 1HG1 VAL A 484 28.355 −2.525 −15.900 1.00 26.00 H

ATOM 4446 2HG1 VAL A 484 26.921 −2.158 −16.888 1.00 26.00 H

ATOM 4447 3HG1 VAL A 484 28.506 −1.481 −17.333 1.00 26.00 H

ATOM 4448 1HG2 VAL A 484 28.070 −4.863 −16.057 1.00 26.00 H

ATOM 4449 2HG2 VAL A 484 27.961 −5.708 −17.619 1.00 26.00 H

ATOM 4450 3HG2 VAL A 484 26.597 −4.720 −17.045 1.00 26.00 H

ATOM 4451 N ILE A 485 27.653 −5.558 −20.482 1.00 25.93 N

ATOM 4452 CA ILE A 485 28.118 −6.590 −21.429 1.00 25.83 C

ATOM 4453 C ILE A 485 27.390 −6.438 −22.773 1.00 25.99 C

ATOM 4454 O ILE A 485 26.171 −6.586 −22.852 1.00 26.18 O

ATOM 4455 CB ILE A 485 27.965 −8.011 −20.837 1.00 25.74 C

ATOM 4456 CG1 ILE A 485 28.742 −8.247 −19.521 1.00 25.55 C

ATOM 4457 CG2 ILE A 485 28.343 −9.100 −21.862 1.00 25.69 C

ATOM 4458 CD1 ILE A 485 30.271 −8.168 −19.617 1.00 25.41 C

ATOM 4459 HN ILE A 485 26.713 −5.645 −20.108 1.00 0.00 H

ATOM 4460 HA ILE A 485 29.180 −6.420 −21.608 1.00 25.79 H

ATOM 4461 HB ILE A 485 26.908 −8.146 −20.608 1.00 25.79 H

ATOM 4462 1HG1 ILE A 485 28.414 −7.496 −18.802 1.00 25.79 H

ATOM 4463 2HG1 ILE A 485 28.482 −9.240 −19.154 1.00 25.79 H

ATOM 4464 1HG2 ILE A 485 28.223 −10.084 −21.409 1.00 25.79 H

ATOM	4465	2HG2	ILE	A	485	29.380	−8.966	−22.169	1.00	25.79	H
ATOM	4466	3HG2	ILE	A	485	27.694	−9.020	−22.734	1.00	25.79	H
ATOM	4467	1HD1	ILE	A	485	30.707	−8.350	−18.635	1.00	25.79	H
ATOM	4468	2HD1	ILE	A	485	30.563	−7.177	−19.966	1.00	25.79	H
ATOM	4469	3HD1	ILE	A	485	30.631	−8.921	−20.319	1.00	25.79	H
ATOM	4470	N	SER	A	486	28.164	−6.314	−23.855	1.00	25.92	N
ATOM	4471	CA	SER	A	486	27.695	−6.274	−25.248	1.00	26.04	C
ATOM	4472	C	SER	A	486	28.661	−7.033	−26.160	1.00	25.93	C
ATOM	4473	O	SER	A	486	29.875	−6.905	−25.994	1.00	25.79	O
ATOM	4474	CB	SER	A	486	27.566	−4.819	−25.715	1.00	26.14	C
ATOM	4475	OG	SER	A	486	27.142	−4.757	−27.067	1.00	26.27	O
ATOM	4476	HN	SER	A	486	29.161	−6.257	−23.708	1.00	0.00	H
ATOM	4477	HG	SER	A	486	27.785	−5.220	−27.619	1.00	0.00	H
ATOM	4478	HA	SER	A	486	26.715	−6.748	−25.302	1.00	26.02	H
ATOM	4479	HB1	SER	A	486	26.836	−4.307	−25.088	1.00	26.02	H
ATOM	4480	HB2	SER	A	486	28.534	−4.327	−25.622	1.00	26.02	H
ATOM	4481	N	ARG	A	487	28.136	−7.689	−27.206	1.00	26.00	N
ATOM	4482	CA	ARG	A	487	28.914	−8.449	−28.214	1.00	25.93	C
ATOM	4483	C	ARG	A	487	29.420	−7.589	−29.396	1.00	25.98	C
ATOM	4484	O	ARG	A	487	28.713	−6.634	−29.741	1.00	26.08	O
ATOM	4485	CB	ARG	A	487	28.085	−9.670	−28.680	1.00	26.02	C
ATOM	4486	CG	ARG	A	487	27.147	−9.478	−29.888	1.00	26.18	C
ATOM	4487	CD	ARG	A	487	26.108	−8.354	−29.781	1.00	26.31	C
ATOM	4488	NE	ARG	A	487	25.513	−8.047	−31.096	1.00	26.45	N
ATOM	4489	CZ	ARG	A	487	25.760	−7.007	−31.872	1.00	26.49	C
ATOM	4490	NH1	ARG	A	487	26.658	−6.110	−31.602	1.00	26.40	N
ATOM	4491	NH2	ARG	A	487	25.081	−6.808	−32.955	1.00	26.62	N

ATOM 4492 HN ARG A 487 27.124 −7.683 −27.286 1.00 0.00 H
ATOM 4493 HE ARG A 487 24.695 −8.588 −31.359 1.00 0.00 H
ATOM 4494 1HH1 ARG A 487 27.328 −6.265 −30.861 1.00 0.00 H
ATOM 4495 2HH1 ARG A 487 26.705 −5.297 −32.203 1.00 0.00 H
ATOM 4496 1HH2 ARG A 487 24.466 −7.528 −33.320 1.00 0.00 H
ATOM 4497 2HH2 ARG A 487 25.262 −5.967 −33.487 1.00 0.00 H
ATOM 4498 HA ARG A 487 29.796 −8.840 −27.706 1.00 26.22 H
ATOM 4499 HB1 ARG A 487 28.788 −10.463 −28.936 1.00 26.22 H
ATOM 4500 HB2 ARG A 487 27.470 −9.987 −27.838 1.00 26.22 H
ATOM 4501 HG1 ARG A 487 27.769 −9.271 −30.759 1.00 26.22 H
ATOM 4502 HG2 ARG A 487 26.608 −10.413 −30.039 1.00 26.22 H
ATOM 4503 HD1 ARG A 487 25.318 −8.665 −29.098 1.00 26.22 H
ATOM 4504 HD2 ARG A 487 26.593 −7.458 −29.393 1.00 26.22 H
ATOM 4505 N PRO A 488 30.507 −7.957 −30.108 1.00 25.91 N
ATOM 4506 CA PRO A 488 30.891 −7.349 −31.392 1.00 25.97 C
ATOM 4507 C PRO A 488 29.763 −7.421 −32.423 1.00 26.10 C
ATOM 4508 O PRO A 488 29.118 −8.457 −32.562 1.00 26.12 O
ATOM 4509 CB PRO A 488 32.114 −8.123 −31.904 1.00 25.88 C
ATOM 4510 CG PRO A 488 32.098 −9.419 −31.100 1.00 25.78 C
ATOM 4511 CD PRO A 488 31.452 −9.011 −29.778 1.00 25.79 C
ATOM 4512 HA PRO A 488 31.166 −6.307 −31.230 1.00 25.94 H
ATOM 4513 HB1 PRO A 488 32.023 −8.328 −32.971 1.00 25.94 H
ATOM 4514 HB2 PRO A 488 33.031 −7.564 −31.718 1.00 25.94 H
ATOM 4515 HG1 PRO A 488 31.505 −10.182 −31.604 1.00 25.94 H
ATOM 4516 HG2 PRO A 488 33.110 −9.791 −30.940 1.00 25.94 H
ATOM 4517 HD1 PRO A 488 30.934 −9.859 −29.331 1.00 25.94 H
ATOM 4518 HD2 PRO A 488 32.207 −8.640 −29.085 1.00 25.94 H

ATOM 4519 N GLY A 489 29.500 −6.314 −33.118 1.00 26.20 N

ATOM 4520 CA GLY A 489 28.614 −6.288 −34.288 1.00 26.31 C

ATOM 4521 C GLY A 489 29.406 −6.298 −35.590 1.00 26.32 C

ATOM 4522 O GLY A 489 30.530 −6.783 −35.617 1.00 26.24 O

ATOM 4523 HN GLY A 489 30.129 −5.528 −32.997 1.00 0.00 H

ATOM 4524 HA1 GLY A 489 27.966 −7.164 −34.261 1.00 26.27 H

ATOM 4525 HA2 GLY A 489 28.005 −5.385 −34.251 1.00 26.27 H

ATOM 4526 N GLN A 490 28.922 −5.534 −36.564 1.00 26.42 N

ATOM 4527 CA GLN A 490 29.726 −4.814 −37.552 1.00 26.44 C

ATOM 4528 C GLN A 490 29.271 −3.339 −37.549 1.00 26.54 C

ATOM 4529 O GLN A 490 28.921 −2.813 −36.492 1.00 26.57 O

ATOM 4530 CB GLN A 490 29.648 −5.525 −38.919 1.00 26.48 C

ATOM 4531 CG GLN A 490 30.257 −6.936 −38.840 1.00 26.41 C

ATOM 4532 CD GLN A 490 30.789 −7.450 −40.171 1.00 26.47 C

ATOM 4533 OE1 GLN A 490 30.216 −7.275 −41.233 1.00 26.55 O

ATOM 4534 NE2 GLN A 490 31.918 −8.122 −40.164 1.00 26.45 N

ATOM 4535 HN GLN A 490 27.972 −5.177 −36.465 1.00 0.00 H

ATOM 4536 1HE2 GLN A 490 32.434 −8.213 −39.304 1.00 0.00 H

ATOM 4537 2HE2 GLN A 490 32.272 −8.410 −41.055 1.00 0.00 H

ATOM 4538 HA GLN A 490 30.764 −4.843 −37.221 1.00 26.48 H

ATOM 4539 HB1 GLN A 490 28.604 −5.604 −39.221 1.00 26.48 H

ATOM 4540 HB2 GLN A 490 30.197 −4.940 −39.657 1.00 26.48 H

ATOM 4541 HG1 GLN A 490 31.080 −6.916 −38.126 1.00 26.48 H

ATOM 4542 HG2 GLN A 490 29.487 −7.623 −38.489 1.00 26.48 H

ATOM 4543 N SER A 491 29.408 −2.608 −38.657 1.00 35.03 N

ATOM 4544 CA SER A 491 29.243 −1.145 −38.700 1.00 35.13 C

ATOM 4545 C SER A 491 27.787 −0.633 −38.693 1.00 35.22 C

ATOM 4546 O SER A 491 27.565 0.519 −39.059 1.00 35.29 O
ATOM 4547 CB SER A 491 30.008 −0.592 −39.907 1.00 35.18 C
ATOM 4548 OG SER A 491 29.399 −1.019 −41.111 1.00 35.16 O
ATOM 4549 HN SER A 491 29.699 −3.063 −39.510 1.00 0.00 H
ATOM 4550 HG SER A 491 28.564 −0.541 −41.192 1.00 0.00 H
ATOM 4551 HA SER A 491 29.725 −0.745 −37.808 1.00 35.17 H
ATOM 4552 HB1 SER A 491 30.003 0.497 −39.868 1.00 35.17 H
ATOM 4553 HB2 SER A 491 31.036 −0.952 −39.877 1.00 35.17 H
ATOM 4554 N GLN A 492 26.794 −1.485 −38.413 1.00 26.80 N
ATOM 4555 CA GLN A 492 25.361 −1.137 −38.420 1.00 26.91 C
ATOM 4556 C GLN A 492 24.715 −1.346 −37.036 1.00 26.93 C
ATOM 4557 O GLN A 492 24.024 −0.459 −36.542 1.00 27.05 O
ATOM 4558 CB GLN A 492 24.688 −1.946 −39.546 1.00 26.94 C
ATOM 4559 CG GLN A 492 23.149 −1.946 −39.565 1.00 27.06 C
ATOM 4560 CD GLN A 492 22.488 −0.656 −40.040 1.00 27.18 C
ATOM 4561 OE1 GLN A 492 23.092 0.389 −40.211 1.00 27.18 O
ATOM 4562 NE2 GLN A 492 21.204 −0.705 −40.324 1.00 27.28 N
ATOM 4563 HN GLN A 492 27.040 −2.430 −38.149 1.00 0.00 H
ATOM 4564 1HE2 GLN A 492 20.720 −1.590 −40.282 1.00 0.00 H
ATOM 4565 2HE2 GLN A 492 20.749 0.175 −40.493 1.00 0.00 H
ATOM 4566 HA GLN A 492 25.276 −0.080 −38.672 1.00 27.04 H
ATOM 4567 HB1 GLN A 492 25.031 −1.539 −40.497 1.00 27.04 H
ATOM 4568 HB2 GLN A 492 25.018 −2.981 −39.453 1.00 27.04 H
ATOM 4569 HG1 GLN A 492 22.823 −2.750 −40.225 1.00 27.04 H
ATOM 4570 HG2 GLN A 492 22.803 −2.145 −38.551 1.00 27.04 H
ATOM 4571 N CYS A 493 25.108 −2.403 −36.321 1.00 26.82 N
ATOM 4572 CA CYS A 493 24.735 −2.694 −34.934 1.00 26.83 C

ATOM	4573	C	CYS	A	493	25.978	−3.091	−34.091	1.00	26.69	C
ATOM	4574	O	CYS	A	493	26.039	−4.225	−33.597	1.00	26.61	O
ATOM	4575	CB	CYS	A	493	23.617	−3.757	−34.951	1.00	26.88	C
ATOM	4576	SG	CYS	A	493	22.037	−3.172	−35.630	1.00	27.07	S
ATOM	4577	HN	CYS	A	493	25.664	−3.112	−36.796	1.00	0.00	H
ATOM	4578	HA	CYS	A	493	24.320	−1.783	−34.504	1.00	26.82	H
ATOM	4579	HB1	CYS	A	493	23.956	−4.599	−35.554	1.00	26.82	H
ATOM	4580	HB2	CYS	A	493	23.446	−4.088	−33.927	1.00	26.82	H
ATOM	4581	N	PRO	A	494	26.996	−2.210	−33.938	1.00	26.66	N
ATOM	4582	CA	PRO	A	494	28.217	−2.450	−33.145	1.00	26.53	C
ATOM	4583	C	PRO	A	494	27.943	−2.540	−31.629	1.00	26.51	C
ATOM	4584	O	PRO	A	494	26.814	−2.340	−31.185	1.00	26.63	O
ATOM	4585	CB	PRO	A	494	29.138	−1.266	−33.471	1.00	26.57	C
ATOM	4586	CG	PRO	A	494	28.155	−0.132	−33.746	1.00	26.74	C
ATOM	4587	CD	PRO	A	494	27.021	−0.852	−34.469	1.00	26.78	C
ATOM	4588	HA	PRO	A	494	28.687	−3.374	−33.481	1.00	26.63	H
ATOM	4589	HB1	PRO	A	494	29.784	−1.028	−32.626	1.00	26.63	H
ATOM	4590	HB2	PRO	A	494	29.750	−1.476	−34.349	1.00	26.63	H
ATOM	4591	HG1	PRO	A	494	27.806	0.321	−32.818	1.00	26.63	H
ATOM	4592	HG2	PRO	A	494	28.603	0.634	−34.379	1.00	26.63	H
ATOM	4593	HD1	PRO	A	494	26.072	−0.353	−34.274	1.00	26.63	H
ATOM	4594	HD2	PRO	A	494	27.209	−0.870	−35.543	1.00	26.63	H
ATOM	4595	N	ARG	A	495	28.974	−2.818	−30.809	1.00	29.12	N
ATOM	4596	CA	ARG	A	495	28.834	−2.867	−29.333	1.00	29.08	C
ATOM	4597	C	ARG	A	495	28.155	−1.619	−28.756	1.00	29.24	C
ATOM	4598	O	ARG	A	495	28.538	−0.498	−29.078	1.00	29.36	O
ATOM	4599	CB	ARG	A	495	30.183	−3.090	−28.614	1.00	28.96	C

ATOM 4600 CG ARG A 495 30.550 −4.571 −28.566 1.00 28.78 C

ATOM 4601 CD ARG A 495 31.876 −4.925 −27.890 1.00 28.67 C

ATOM 4602 NE ARG A 495 31.867 −4.848 −26.422 1.00 28.62 N

ATOM 4603 CZ ARG A 495 32.892 −4.379 −25.732 1.00 28.53 C

ATOM 4604 NH1 ARG A 495 33.262 −4.972 −24.637 1.00 28.48 N

ATOM 4605 NH2 ARG A 495 33.638 −3.397 −26.145 1.00 28.51 N

ATOM 4606 HN ARG A 495 29.881 −2.976 −31.228 1.00 0.00 H

ATOM 4607 HE ARG A 495 31.346 −5.594 −25.979 1.00 0.00 H

ATOM 4608 1HH1 ARG A 495 32.906 −5.888 −24.439 1.00 0.00 H

ATOM 4609 2HH1 ARG A 495 34.176 −4.727 −24.260 1.00 0.00 H

ATOM 4610 1HH2 ARG A 495 33.468 −3.022 −27.072 1.00 0.00 H

ATOM 4611 2HH2 ARG A 495 34.607 −3.434 −25.861 1.00 0.00 H

ATOM 4612 HA ARG A 495 28.197 −3.720 −29.097 1.00 28.85 H

ATOM 4613 HB1 ARG A 495 30.963 −2.548 −29.149 1.00 28.85 H

ATOM 4614 HB2 ARG A 495 30.109 −2.710 −27.595 1.00 28.85 H

ATOM 4615 HG1 ARG A 495 29.756 −5.091 −28.030 1.00 28.85 H

ATOM 4616 HG2 ARG A 495 30.595 −4.934 −29.593 1.00 28.85 H

ATOM 4617 HD1 ARG A 495 32.138 −5.945 −28.173 1.00 28.85 H

ATOM 4618 HD2 ARG A 495 32.637 −4.238 −28.259 1.00 28.85 H

ATOM 4619 N PHE A 496 27.319 −1.858 −27.745 1.00 44.38 N

ATOM 4620 CA PHE A 496 26.547 −0.867 −26.987 1.00 44.56 C

ATOM 4621 C PHE A 496 25.545 −0.047 −27.820 1.00 44.76 C

ATOM 4622 O PHE A 496 25.055 0.979 −27.346 1.00 44.93 O

ATOM 4623 CB PHE A 496 27.466 −0.017 −26.091 1.00 44.56 C

ATOM 4624 CG PHE A 496 28.371 −0.830 −25.182 1.00 44.39 C

ATOM 4625 CD1 PHE A 496 27.847 −1.411 −24.013 1.00 44.42 C

ATOM 4626 CD2 PHE A 496 29.730 −1.010 −25.501 1.00 44.21 C

ATOM	4627	CE1	PHE	A	496	28.674	−2.178	−23.173	1.00	44.26	C
ATOM	4628	CE2	PHE	A	496	30.557	−1.778	−24.660	1.00	44.05	C
ATOM	4629	CZ	PHE	A	496	30.027	−2.367	−23.498	1.00	44.06	C
ATOM	4630	HN	PHE	A	496	27.116	−2.826	−27.543	1.00	0.00	H
ATOM	4631	HA	PHE	A	496	25.935	−1.449	−26.297	1.00	44.42	H
ATOM	4632	HB1	PHE	A	496	28.093	0.602	−26.733	1.00	44.42	H
ATOM	4633	HB2	PHE	A	496	26.840	0.622	−25.468	1.00	44.42	H
ATOM	4634	HD1	PHE	A	496	26.797	−1.266	−23.758	1.00	44.42	H
ATOM	4635	HD2	PHE	A	496	30.143	−0.554	−26.401	1.00	44.42	H
ATOM	4636	HE1	PHE	A	496	28.264	−2.627	−22.268	1.00	44.42	H
ATOM	4637	HE2	PHE	A	496	31.609	−1.916	−24.909	1.00	44.42	H
ATOM	4638	HZ	PHE	A	496	30.665	−2.969	−22.851	1.00	44.42	H
ATOM	4639	N	ASN	A	497	25.114	−0.550	−28.985	1.00	38.11	N
ATOM	4640	CA	ASN	A	497	23.877	−0.066	−29.588	1.00	38.29	C
ATOM	4641	C	ASN	A	497	22.645	−0.597	−28.830	1.00	38.39	C
ATOM	4642	O	ASN	A	497	22.563	−1.768	−28.462	1.00	38.31	O
ATOM	4643	CB	ASN	A	497	23.820	−0.390	−31.088	1.00	38.25	C
ATOM	4644	CG	ASN	A	497	22.542	0.156	−31.707	1.00	38.43	C
ATOM	4645	OD1	ASN	A	497	22.216	1.324	−31.545	1.00	38.55	O
ATOM	4646	ND2	ASN	A	497	21.611	−0.706	−32.031	1.00	38.45	N
ATOM	4647	HN	ASN	A	497	25.459	−1.454	−29.283	1.00	0.00	H
ATOM	4648	1HD2	ASN	A	497	21.680	−1.666	−31.722	1.00	0.00	H
ATOM	4649	2HD2	ASN	A	497	20.714	−0.331	−32.321	1.00	0.00	H
ATOM	4650	HA	ASN	A	497	23.873	1.020	−29.491	1.00	38.35	H
ATOM	4651	HB1	ASN	A	497	24.679	0.061	−31.584	1.00	38.35	H
ATOM	4652	HB2	ASN	A	497	23.849	−1.471	−31.222	1.00	38.35	H
ATOM	4653	N	VAL	A	498	21.638	0.266	−28.710	1.00	52.36	N

ATOM 4654 CA VAL A 498 20.312 −0.046 −28.146 1.00 52.51 C
ATOM 4655 C VAL A 498 19.185 0.477 −29.056 1.00 52.68 C
ATOM 4656 O VAL A 498 18.021 0.120 −28.886 1.00 52.79 O
ATOM 4657 CB VAL A 498 20.211 0.502 −26.699 1.00 52.65 C
ATOM 4658 CG1 VAL A 498 18.941 0.051 −25.964 1.00 52.78 C
ATOM 4659 CG2 VAL A 498 21.400 0.070 −25.822 1.00 52.48 C
ATOM 4660 HN VAL A 498 21.803 1.201 −29.052 1.00 0.00 H
ATOM 4661 HA VAL A 498 20.221 −1.131 −28.096 1.00 52.61 H
ATOM 4662 HB VAL A 498 20.205 1.591 −26.749 1.00 52.61 H
ATOM 4663 1HG1 VAL A 498 18.935 0.470 −24.958 1.00 52.61 H
ATOM 4664 2HG1 VAL A 498 18.923 −1.037 −25.904 1.00 52.61 H
ATOM 4665 3HG1 VAL A 498 18.063 0.399 −26.508 1.00 52.61 H
ATOM 4666 1HG2 VAL A 498 21.280 0.480 −24.819 1.00 52.61 H
ATOM 4667 2HG2 VAL A 498 22.327 0.442 −26.258 1.00 52.61 H
ATOM 4668 3HG2 VAL A 498 21.435 −1.018 −25.767 1.00 52.61 H
ATOM 4669 N CYS A 499 19.512 1.294 −30.065 1.00 27.71 N
ATOM 4670 CA CYS A 499 18.532 1.829 −31.003 1.00 27.88 C
ATOM 4671 C CYS A 499 18.097 0.776 −32.033 1.00 27.79 C
ATOM 4672 O CYS A 499 18.969 0.156 −32.644 1.00 27.61 O
ATOM 4673 CB CYS A 499 19.099 3.074 −31.687 1.00 27.92 C
ATOM 4674 SG CYS A 499 19.005 4.528 −30.615 1.00 28.18 S
ATOM 4675 HN CYS A 499 20.487 1.419 −30.299 1.00 0.00 H
ATOM 4676 HA CYS A 499 17.651 2.129 −30.435 1.00 27.85 H
ATOM 4677 HB1 CYS A 499 20.142 2.891 −31.944 1.00 27.85 H
ATOM 4678 HB2 CYS A 499 18.529 3.268 −32.596 1.00 27.85 H
ATOM 4679 N PRO A 500 16.788 0.623 −32.316 1.00 27.95 N
ATOM 4680 CA PRO A 500 16.325 −0.359 −33.287 1.00 27.92 C

ATOM	4681	C	PRO	A	500	16.806	−0.068	−34.707	1.00	27.81	C
ATOM	4682	O	PRO	A	500	16.348	0.886	−35.331	1.00	27.87	O
ATOM	4683	CB	PRO	A	500	14.800	−0.382	−33.182	1.00	28.16	C
ATOM	4684	CG	PRO	A	500	14.533	0.135	−31.772	1.00	28.30	C
ATOM	4685	CD	PRO	A	500	15.657	1.141	−31.557	1.00	28.19	C
ATOM	4686	HA	PRO	A	500	16.703	−1.338	−32.991	1.00	28.03	H
ATOM	4687	HB1	PRO	A	500	14.348	0.271	−33.929	1.00	28.03	H
ATOM	4688	HB2	PRO	A	500	14.416	−1.395	−33.305	1.00	28.03	H
ATOM	4689	HG1	PRO	A	500	13.558	0.618	−31.710	1.00	28.03	H
ATOM	4690	HG2	PRO	A	500	14.586	−0.673	−31.042	1.00	28.03	H
ATOM	4691	HD1	PRO	A	500	15.366	2.123	−31.929	1.00	28.03	H
ATOM	4692	HD2	PRO	A	500	15.908	1.212	−30.499	1.00	28.03	H
ATOM	4693	N	GLU	A	501	17.544	−1.007	−35.296	1.00	27.67	N
ATOM	4694	CA	GLU	A	501	18.062	−0.888	−36.660	1.00	27.58	C
ATOM	4695	C	GLU	A	501	17.939	−2.165	−37.486	1.00	27.53	C
ATOM	4696	O	GLU	A	501	17.790	−3.278	−36.972	1.00	27.52	O
ATOM	4697	CB	GLU	A	501	19.510	−0.356	−36.671	1.00	27.41	C
ATOM	4698	CG	GLU	A	501	19.604	1.177	−36.589	1.00	27.44	C
ATOM	4699	CD	GLU	A	501	18.743	1.918	−37.632	1.00	27.59	C
ATOM	4700	OE1	GLU	A	501	18.409	1.311	−38.683	1.00	27.60	O
ATOM	4701	OE2	GLU	A	501	18.120	2.926	−37.223	1.00	27.70	O
ATOM	4702	HN	GLU	A	501	17.859	−1.792	−34.746	1.00	0.00	H
ATOM	4703	HA	GLU	A	501	17.451	−0.136	−37.159	1.00	27.56	H
ATOM	4704	HB1	GLU	A	501	20.039	−0.780	−35.817	1.00	27.56	H
ATOM	4705	HB2	GLU	A	501	19.989	−0.682	−37.594	1.00	27.56	H
ATOM	4706	HG1	GLU	A	501	19.280	1.487	−35.596	1.00	27.56	H
ATOM	4707	HG2	GLU	A	501	20.645	1.464	−36.738	1.00	27.56	H

ATOM 4708 N VAL A 502 17.913 −1.979 −38.807 1.00 27.52 N

ATOM 4709 CA VAL A 502 17.830 −3.079 −39.772 1.00 27.49 C

ATOM 4710 C VAL A 502 19.177 −3.800 −39.834 1.00 27.33 C

ATOM 4711 O VAL A 502 20.052 −3.424 −40.611 1.00 27.25 O

ATOM 4712 CB VAL A 502 17.370 −2.587 −41.160 1.00 27.53 C

ATOM 4713 CG1 VAL A 502 17.185 −3.761 −42.131 1.00 27.50 C

ATOM 4714 CG2 VAL A 502 16.025 −1.855 −41.072 1.00 27.70 C

ATOM 4715 HN VAL A 502 18.055 −1.030 −39.147 1.00 0.00 H

ATOM 4716 HA VAL A 502 17.088 −3.789 −39.406 1.00 27.47 H

ATOM 4717 HB VAL A 502 18.120 −1.906 −41.563 1.00 27.47 H

ATOM 4718 1HG1 VAL A 502 16.861 −3.384 −43.101 1.00 27.47 H

ATOM 4719 2HG1 VAL A 502 16.432 −4.444 −41.737 1.00 27.47 H

ATOM 4720 3HG1 VAL A 502 18.131 −4.291 −42.246 1.00 27.47 H

ATOM 4721 1HG2 VAL A 502 15.727 −1.520 −42.066 1.00 27.47 H

ATOM 4722 2HG2 VAL A 502 16.123 −0.992 −40.413 1.00 27.47 H

ATOM 4723 3HG2 VAL A 502 15.268 −2.531 −40.675 1.00 27.47 H

ATOM 4724 N CYS A 503 19.344 −4.827 −39.003 1.00 28.33 N

ATOM 4725 CA CYS A 503 20.543 −5.663 −38.948 1.00 28.19 C

ATOM 4726 C CYS A 503 20.222 −7.121 −38.583 1.00 28.20 C

ATOM 4727 O CYS A 503 19.201 −7.409 −37.947 1.00 28.29 O

ATOM 4728 CB CYS A 503 21.556 −5.042 −37.975 1.00 28.09 C

ATOM 4729 SG CYS A 503 21.033 −4.838 −36.251 1.00 28.16 S

ATOM 4730 HN CYS A 503 18.661 −4.951 −38.268 1.00 0.00 H

ATOM 4731 HA CYS A 503 20.993 −5.662 −39.941 1.00 28.21 H

ATOM 4732 HB1 CYS A 503 22.442 −5.677 −37.976 1.00 28.21 H

ATOM 4733 HB2 CYS A 503 21.815 −4.055 −38.358 1.00 28.21 H

ATOM 4734 N TRP A 504 21.155 −8.009 −38.939 1.00 28.10 N

ATOM	4735	CA	TRP	A	504	21.322	−9.360	−38.399	1.00	28.10		C
ATOM	4736	C	TRP	A	504	22.824	−9.643	−38.282	1.00	27.95		C
ATOM	4737	O	TRP	A	504	23.486	−9.941	−39.273	1.00	27.94		O
ATOM	4738	CB	TRP	A	504	20.620	−10.421	−39.259	1.00	28.23		C
ATOM	4739	CG	TRP	A	504	20.896	−11.841	−38.839	1.00	28.24		C
ATOM	4740	CD1	TRP	A	504	20.707	−12.337	−37.594	1.00	28.21		C
ATOM	4741	CD2	TRP	A	504	21.484	−12.940	−39.605	1.00	28.31		C
ATOM	4742	NE1	TRP	A	504	21.059	−13.669	−37.555	1.00	28.25		N
ATOM	4743	CE2	TRP	A	504	21.547	−14.098	−38.770	1.00	28.32		C
ATOM	4744	CE3	TRP	A	504	21.986	−13.083	−40.920	1.00	28.36		C
ATOM	4745	CZ2	TRP	A	504	22.038	−15.331	−39.215	1.00	28.41		C
ATOM	4746	CZ3	TRP	A	504	22.491	−14.317	−41.377	1.00	28.45		C
ATOM	4747	CH2	TRP	A	504	22.510	−15.443	−40.534	1.00	28.48		C
ATOM	4748	HN	TRP	A	504	21.952	−7.655	−39.451	1.00	0.00		H
ATOM	4749	HE1	TRP	A	504	21.002	−14.237	−36.723	1.00	0.00		H
ATOM	4750	HA	TRP	A	504	20.889	−9.386	−37.399	1.00	28.24		H
ATOM	4751	HB1	TRP	A	504	19.545	−10.251	−39.201	1.00	28.24		H
ATOM	4752	HB2	TRP	A	504	20.952	−10.299	−40.290	1.00	28.24		H
ATOM	4753	HD1	TRP	A	504	20.331	−11.766	−36.745	1.00	28.24		H
ATOM	4754	HE3	TRP	A	504	21.982	−12.224	−41.591	1.00	28.24		H
ATOM	4755	HZ2	TRP	A	504	22.054	−16.193	−38.548	1.00	28.24		H
ATOM	4756	HZ3	TRP	A	504	22.871	−14.400	−42.395	1.00	28.24		H
ATOM	4757	HH2	TRP	A	504	22.889	−16.397	−40.902	1.00	28.24		H
ATOM	4758	N	GLU	A	505	23.391	−9.299	−37.128	1.00	44.40		N
ATOM	4759	CA	GLU	A	505	24.831	−9.325	−36.870	1.00	44.26		C
ATOM	4760	C	GLU	A	505	25.118	−9.435	−35.368	1.00	44.18		C
ATOM	4761	O	GLU	A	505	24.330	−9.000	−34.521	1.00	44.22		O

ATOM 4762 CB GLU A 505 25.526 −8.089 −37.475 1.00 44.21 C
ATOM 4763 CG GLU A 505 25.227 −6.777 −36.737 1.00 44.24 C
ATOM 4764 CD GLU A 505 25.537 −5.563 −37.617 1.00 44.26 C
ATOM 4765 OE1 GLU A 505 24.724 −5.305 −38.527 1.00 44.24 O
ATOM 4766 OE2 GLU A 505 26.351 −4.724 −37.166 1.00 44.33 O
ATOM 4767 HN GLU A 505 22.786 −9.091 −36.343 1.00 0.00 H
ATOM 4768 HA GLU A 505 25.242 −10.211 −37.355 1.00 44.26 H
ATOM 4769 HB1 GLU A 505 26.603 −8.257 −37.452 1.00 44.26 H
ATOM 4770 HB2 GLU A 505 25.196 −7.983 −38.508 1.00 44.26 H
ATOM 4771 HG1 GLU A 505 24.172 −6.756 −36.462 1.00 44.26 H
ATOM 4772 HG2 GLU A 505 25.839 −6.729 −35.836 1.00 44.26 H
ATOM 4773 N GLY A 506 26.263 −10.021 −35.038 1.00 26.50 N
ATOM 4774 CA GLY A 506 26.666 −10.348 −33.676 1.00 26.39 C
ATOM 4775 C GLY A 506 27.616 −11.544 −33.668 1.00 26.34 C
ATOM 4776 O GLY A 506 27.719 −12.250 −34.673 1.00 26.39 O
ATOM 4777 HN GLY A 506 26.848 −10.393 −35.774 1.00 0.00 H
ATOM 4778 HA1 GLY A 506 27.170 −9.488 −33.236 1.00 26.40 H
ATOM 4779 HA2 GLY A 506 25.780 −10.591 −33.089 1.00 26.40 H
ATOM 4780 N THR A 507 28.075 −11.935 −32.479 1.00 26.24 N
ATOM 4781 CA THR A 507 28.517 −13.313 −32.223 1.00 26.20 C
ATOM 4782 C THR A 507 27.970 −13.847 −30.899 1.00 26.19 C
ATOM 4783 O THR A 507 27.530 −13.095 −30.024 1.00 26.16 O
ATOM 4784 CB THR A 507 30.037 −13.532 −32.335 1.00 26.05 C
ATOM 4785 OG1 THR A 507 30.704 −13.230 −31.139 1.00 25.91 O
ATOM 4786 CG2 THR A 507 30.745 −12.780 −33.459 1.00 26.09 C
ATOM 4787 HN THR A 507 27.949 −11.332 −31.682 1.00 0.00 H
ATOM 4788 HG1 THR A 507 31.515 −13.770 −31.176 1.00 0.00 H

ATOM 4789 HA THR A 507 28.066 −13.925 −33.004 1.00 26.12 H
ATOM 4790 HB THR A 507 30.190 −14.595 −32.522 1.00 26.12 H
ATOM 4791 1HG2 THR A 507 31.810 −13.013 −33.438 1.00 26.12 H
ATOM 4792 2HG2 THR A 507 30.605 −11.707 −33.324 1.00 26.12 H
ATOM 4793 3HG2 THR A 507 30.326 −13.082 −34.419 1.00 26.12 H
ATOM 4794 N TYR A 508 27.905 −15.172 −30.779 1.00 26.23 N
ATOM 4795 CA TYR A 508 27.410 −15.864 −29.592 1.00 26.23 C
ATOM 4796 C TYR A 508 28.416 −15.743 −28.427 1.00 26.02 C
ATOM 4797 O TYR A 508 29.510 −16.304 −28.492 1.00 25.93 O
ATOM 4798 CB TYR A 508 27.116 −17.312 −30.014 1.00 26.38 C
ATOM 4799 CG TYR A 508 26.492 −18.197 −28.958 1.00 26.39 C
ATOM 4800 CD1 TYR A 508 25.155 −18.618 −29.069 1.00 26.60 C
ATOM 4801 CD2 TYR A 508 27.265 −18.636 −27.876 1.00 26.22 C
ATOM 4802 CE1 TYR A 508 24.593 −19.475 −28.099 1.00 26.63 C
ATOM 4803 CE2 TYR A 508 26.712 −19.482 −26.905 1.00 26.23 C
ATOM 4804 CZ TYR A 508 25.368 −19.888 −26.995 1.00 26.44 C
ATOM 4805 OH TYR A 508 24.827 −20.648 −26.015 1.00 26.46 O
ATOM 4806 HN TYR A 508 28.273 −15.733 −31.531 1.00 0.00 H
ATOM 4807 HH TYR A 508 25.380 −20.613 −25.198 1.00 0.00 H
ATOM 4808 HA TYR A 508 26.473 −15.397 −29.287 1.00 26.31 H
ATOM 4809 HB1 TYR A 508 26.437 −17.280 −30.866 1.00 26.31 H
ATOM 4810 HB2 TYR A 508 28.058 −17.769 −30.317 1.00 26.31 H
ATOM 4811 HD1 TYR A 508 24.549 −18.280 −29.910 1.00 26.31 H
ATOM 4812 HD2 TYR A 508 28.304 −18.318 −27.788 1.00 26.31 H
ATOM 4813 HE1 TYR A 508 23.563 −19.817 −28.202 1.00 26.31 H
ATOM 4814 HE2 TYR A 508 27.327 −19.828 −26.074 1.00 26.31 H
ATOM 4815 N ASN A 509 28.004 −15.100 −27.329 1.00 25.97 N

ATOM	4816	CA	ASN	A	509	28.809	−14.855	−26.125	1.00	25.77	C
ATOM	4817	C	ASN	A	509	27.963	−15.148	−24.871	1.00	25.80	C
ATOM	4818	O	ASN	A	509	27.151	−14.301	−24.498	1.00	25.82	O
ATOM	4819	CB	ASN	A	509	29.293	−13.388	−26.115	1.00	25.64	C
ATOM	4820	CG	ASN	A	509	30.307	−13.048	−27.188	1.00	25.59	C
ATOM	4821	OD1	ASN	A	509	31.470	−12.831	−26.910	1.00	25.46	O
ATOM	4822	ND2	ASN	A	509	29.876	−12.792	−28.398	1.00	25.70	N
ATOM	4823	HN	ASN	A	509	27.062	−14.731	−27.309	1.00	0.00	H
ATOM	4824	1HD2	ASN	A	509	28.911	−12.939	−28.649	1.00	0.00	H
ATOM	4825	2HD2	ASN	A	509	30.567	−12.678	−29.127	1.00	0.00	H
ATOM	4826	HA	ASN	A	509	29.675	−15.517	−26.134	1.00	25.72	H
ATOM	4827	HB1	ASN	A	509	28.425	−12.743	−26.252	1.00	25.72	H
ATOM	4828	HB2	ASN	A	509	29.746	−13.187	−25.144	1.00	25.72	H
ATOM	4829	N	ASP	A	510	28.012	−16.376	−24.333	1.00	25.84	N
ATOM	4830	CA	ASP	A	510	27.130	−16.788	−23.223	1.00	25.87	C
ATOM	4831	C	ASP	A	510	27.815	−16.835	−21.844	1.00	25.65	C
ATOM	4832	O	ASP	A	510	29.041	−16.904	−21.710	1.00	25.46	O
ATOM	4833	CB	ASP	A	510	26.279	−18.029	−23.576	1.00	26.03	C
ATOM	4834	CG	ASP	A	510	26.884	−19.413	−23.320	1.00	25.92	C
ATOM	4835	OD1	ASP	A	510	27.427	−19.634	−22.221	1.00	25.71	O
ATOM	4836	OD2	ASP	A	510	26.378	−20.368	−23.947	1.00	26.06	O
ATOM	4837	HN	ASP	A	510	28.786	−16.979	−24.581	1.00	0.00	H
ATOM	4838	HA	ASP	A	510	26.405	−15.978	−23.138	1.00	25.82	H
ATOM	4839	HB1	ASP	A	510	25.358	−17.966	−22.996	1.00	25.82	H
ATOM	4840	HB2	ASP	A	510	26.045	−17.969	−24.639	1.00	25.82	H
ATOM	4841	N	ALA	A	511	26.976	−16.691	−20.817	1.00	25.68	N
ATOM	4842	CA	ALA	A	511	27.347	−16.546	−19.416	1.00	25.49	C

ATOM	4843	C	ALA	A	511	26.333	−17.265	−18.512	1.00	25.56	C
ATOM	4844	O	ALA	A	511	25.180	−17.486	−18.898	1.00	25.77	O
ATOM	4845	CB	ALA	A	511	27.440	−15.049	−19.091	1.00	25.47	C
ATOM	4846	HN	ALA	A	511	25.987	−16.711	−21.012	1.00	0.00	H
ATOM	4847	HA	ALA	A	511	28.328	−16.997	−19.267	1.00	25.59	H
ATOM	4848	HB1	ALA	A	511	27.717	−14.920	−18.045	1.00	25.59	H
ATOM	4849	HB2	ALA	A	511	26.474	−14.577	−19.271	1.00	25.59	H
ATOM	4850	HB3	ALA	A	511	28.195	−14.585	−19.726	1.00	25.59	H
ATOM	4851	N	PHE	A	512	26.745	−17.555	−17.280	1.00	25.40	N
ATOM	4852	CA	PHE	A	512	26.056	−18.434	−16.338	1.00	25.44	C
ATOM	4853	C	PHE	A	512	25.835	−17.743	−14.986	1.00	25.38	C
ATOM	4854	O	PHE	A	512	26.763	−17.145	−14.444	1.00	25.18	O
ATOM	4855	CB	PHE	A	512	26.902	−19.701	−16.195	1.00	25.28	C
ATOM	4856	CG	PHE	A	512	26.203	−20.909	−15.605	1.00	25.41	C
ATOM	4857	CD1	PHE	A	512	25.847	−21.983	−16.442	1.00	25.60	C
ATOM	4858	CD2	PHE	A	512	25.968	−20.999	−14.220	1.00	25.36	C
ATOM	4859	CE1	PHE	A	512	25.321	−23.166	−15.894	1.00	25.75	C
ATOM	4860	CE2	PHE	A	512	25.403	−22.166	−13.677	1.00	25.50	C
ATOM	4861	CZ	PHE	A	512	25.095	−23.255	−14.510	1.00	25.71	C
ATOM	4862	HN	PHE	A	512	27.704	−17.317	−17.048	1.00	0.00	H
ATOM	4863	HA	PHE	A	512	25.086	−18.705	−16.756	1.00	25.46	H
ATOM	4864	HB1	PHE	A	512	27.260	−19.976	−17.187	1.00	25.46	H
ATOM	4865	HB2	PHE	A	512	27.752	−19.463	−15.555	1.00	25.46	H
ATOM	4866	HD1	PHE	A	512	25.980	−21.897	−17.520	1.00	25.46	H
ATOM	4867	HD2	PHE	A	512	26.224	−20.164	−13.568	1.00	25.46	H
ATOM	4868	HE1	PHE	A	512	25.089	−24.012	−16.541	1.00	25.46	H
ATOM	4869	HE2	PHE	A	512	25.203	−22.226	−12.607	1.00	25.46	H

```
ATOM   4870  HZ  PHE A 512      24.681 -24.168 -14.083  1.00 25.46           H
ATOM   4871  N   LEU A 513      24.639 -17.861 -14.404  1.00 25.57           N
ATOM   4872  CA  LEU A 513      24.291 -17.278 -13.103  1.00 25.55           C
ATOM   4873  C   LEU A 513      24.958 -18.041 -11.939  1.00 25.33           C
ATOM   4874  O   LEU A 513      24.559 -19.159 -11.616  1.00 25.37           O
ATOM   4875  CB  LEU A 513      22.757 -17.266 -13.002  1.00 25.85           C
ATOM   4876  CG  LEU A 513      22.193 -16.566 -11.754  1.00 25.88           C
ATOM   4877  CD1 LEU A 513      22.444 -15.063 -11.800  1.00 25.85           C
ATOM   4878  CD2 LEU A 513      20.685 -16.796 -11.695  1.00 26.20           C
ATOM   4879  HN  LEU A 513      23.916 -18.369 -14.889  1.00  0.00           H
ATOM   4880  HA  LEU A 513      24.644 -16.247 -13.084  1.00 25.70           H
ATOM   4881  HB1 LEU A 513      22.364 -16.756 -13.882  1.00 25.70           H
ATOM   4882  HB2 LEU A 513      22.411 -18.299 -12.995  1.00 25.70           H
ATOM   4883  HG  LEU A 513      22.659 -16.984 -10.862  1.00 25.70           H
ATOM   4884 1HD1 LEU A 513      22.033 -14.598 -10.904  1.00 25.70           H
ATOM   4885 2HD1 LEU A 513      21.962 -14.640 -12.682  1.00 25.70           H
ATOM   4886 3HD1 LEU A 513      23.517 -14.875 -11.848  1.00 25.70           H
ATOM   4887 1HD2 LEU A 513      20.276 -16.303 -10.813  1.00 25.70           H
ATOM   4888 2HD2 LEU A 513      20.482 -17.866 -11.640  1.00 25.70           H
ATOM   4889 3HD2 LEU A 513      20.219 -16.384 -12.590  1.00 25.70           H
ATOM   4890  N   ILE A 514      25.961 -17.432 -11.296  1.00 25.10           N
ATOM   4891  CA  ILE A 514      26.731 -18.035 -10.188  1.00 24.87           C
ATOM   4892  C   ILE A 514      26.261 -17.607  -8.787  1.00 24.86           C
ATOM   4893  O   ILE A 514      26.547 -18.307  -7.819  1.00 24.69           O
ATOM   4894  CB  ILE A 514      28.249 -17.820 -10.372  1.00 24.59           C
ATOM   4895  CG1 ILE A 514      28.657 -16.331 -10.332  1.00 24.54           C
ATOM   4896  CG2 ILE A 514      28.746 -18.514 -11.653  1.00 24.63           C
```

ATOM 4897 CD1 ILE A 514 30.156 −16.137 −10.082 1.00 24.27 C

ATOM 4898 HN ILE A 514 26.187 −16.482 −11.564 1.00 0.00 H

ATOM 4899 HA ILE A 514 26.565 −19.110 −10.251 1.00 24.69 H

ATOM 4900 HB ILE A 514 28.744 −18.308 −9.533 1.00 24.69 H

ATOM 4901 1HG1 ILE A 514 28.400 −15.873 −11.287 1.00 24.69 H

ATOM 4902 2HG1 ILE A 514 28.103 −15.839 −9.532 1.00 24.69 H

ATOM 4903 1HG2 ILE A 514 29.818 −18.350 −11.763 1.00 24.69 H

ATOM 4904 2HG2 ILE A 514 28.225 −18.100 −12.516 1.00 24.69 H

ATOM 4905 3HG2 ILE A 514 28.548 −19.584 −11.588 1.00 24.69 H

ATOM 4906 1HD1 ILE A 514 30.387 −15.072 −10.064 1.00 24.69 H

ATOM 4907 2HD1 ILE A 514 30.723 −16.618 −10.879 1.00 24.69 H

ATOM 4908 3HD1 ILE A 514 30.426 −16.583 −9.125 1.00 24.69 H

ATOM 4909 N ASP A 515 25.427 −16.568 −8.688 1.00 25.05 N

ATOM 4910 CA ASP A 515 24.532 −16.325 −7.549 1.00 25.09 C

ATOM 4911 C ASP A 515 23.263 −15.601 −8.019 1.00 25.41 C

ATOM 4912 O ASP A 515 23.348 −14.541 −8.635 1.00 25.46 O

ATOM 4913 CB ASP A 515 25.229 −15.516 −6.443 1.00 24.84 C

ATOM 4914 CG ASP A 515 24.283 −15.203 −5.274 1.00 24.86 C

ATOM 4915 OD1 ASP A 515 24.347 −14.050 −4.794 1.00 24.79 O

ATOM 4916 OD2 ASP A 515 23.290 −15.943 −5.090 1.00 24.97 O

ATOM 4917 HN ASP A 515 25.279 −16.001 −9.516 1.00 0.00 H

ATOM 4918 HA ASP A 515 24.240 −17.289 −7.133 1.00 25.06 H

ATOM 4919 HB1 ASP A 515 26.075 −16.091 −6.066 1.00 25.06 H

ATOM 4920 HB2 ASP A 515 25.587 −14.578 −6.866 1.00 25.06 H

ATOM 4921 N ARG A 516 22.081 −16.113 −7.654 1.00 25.65 N

ATOM 4922 CA ARG A 516 20.793 −15.452 −7.915 1.00 25.98 C

ATOM 4923 C ARG A 516 20.472 −14.332 −6.919 1.00 26.04 C

ATOM 4924 O ARG A 516 19.787 −13.388 −7.299 1.00 26.23 O

ATOM 4925 CB ARG A 516 19.680 −16.514 −7.971 1.00 26.22 C

ATOM 4926 CG ARG A 516 18.327 −15.915 −8.402 1.00 26.59 C

ATOM 4927 CD ARG A 516 17.270 −16.975 −8.732 1.00 26.83 C

ATOM 4928 NE ARG A 516 17.539 −17.627 −10.024 1.00 26.90 N

ATOM 4929 CZ ARG A 516 18.023 −18.831 −10.255 1.00 26.75 C

ATOM 4930 NH1 ARG A 516 18.361 −19.655 −9.309 1.00 26.51 N

ATOM 4931 NH2 ARG A 516 18.138 −19.257 −11.471 1.00 26.85 N

ATOM 4932 HN ARG A 516 22.112 −16.879 −6.994 1.00 0.00 H

ATOM 4933 HE ARG A 516 17.274 −17.097 −10.861 1.00 0.00 H

ATOM 4934 1HH1 ARG A 516 18.183 −19.434 −8.351 1.00 0.00 H

ATOM 4935 2HH1 ARG A 516 18.696 −20.572 −9.578 1.00 0.00 H

ATOM 4936 1HH2 ARG A 516 17.728 −18.651 −12.190 1.00 0.00 H

ATOM 4937 2HH2 ARG A 516 18.273 −20.242 −11.664 1.00 0.00 H

ATOM 4938 HA ARG A 516 20.857 −14.998 −8.904 1.00 26.41 H

ATOM 4939 HB1 ARG A 516 19.966 −17.285 −8.686 1.00 26.41 H

ATOM 4940 HB2 ARG A 516 19.568 −16.958 −6.982 1.00 26.41 H

ATOM 4941 HG1 ARG A 516 17.950 −15.293 −7.590 1.00 26.41 H

ATOM 4942 HG2 ARG A 516 18.488 −15.300 −9.287 1.00 26.41 H

ATOM 4943 HD1 ARG A 516 17.271 −17.732 −7.947 1.00 26.41 H

ATOM 4944 HD2 ARG A 516 16.291 −16.497 −8.775 1.00 26.41 H

ATOM 4945 N LEU A 517 20.974 −14.388 −5.683 1.00 48.11 N

ATOM 4946 CA LEU A 517 20.622 −13.433 −4.622 1.00 48.15 C

ATOM 4947 C LEU A 517 21.212 −12.036 −4.872 1.00 48.13 C

ATOM 4948 O LEU A 517 20.497 −11.042 −4.797 1.00 48.35 O

ATOM 4949 CB LEU A 517 21.071 −13.986 −3.256 1.00 47.91 C

ATOM 4950 CG LEU A 517 20.491 −15.365 −2.884 1.00 48.03 C

ATOM 4951 CD1 LEU A 517 21.051 −15.811 −1.532 1.00 47.84 C
ATOM 4952 CD2 LEU A 517 18.963 −15.344 −2.779 1.00 48.44 C
ATOM 4953 HN LEU A 517 21.657 −15.113 −5.465 1.00 0.00 H
ATOM 4954 HA LEU A 517 19.536 −13.339 −4.604 1.00 48.12 H
ATOM 4955 HB1 LEU A 517 22.158 −14.069 −3.267 1.00 48.12 H
ATOM 4956 HB2 LEU A 517 20.768 −13.274 −2.488 1.00 48.12 H
ATOM 4957 HG LEU A 517 20.784 −16.089 −3.645 1.00 48.12 H
ATOM 4958 1HD1 LEU A 517 20.639 −16.786 −1.271 1.00 48.12 H
ATOM 4959 2HD1 LEU A 517 20.776 −15.085 −0.767 1.00 48.12 H
ATOM 4960 3HD1 LEU A 517 22.137 −15.880 −1.593 1.00 48.12 H
ATOM 4961 1HD2 LEU A 517 18.603 −16.338 −2.515 1.00 48.12 H
ATOM 4962 2HD2 LEU A 517 18.537 −15.046 −3.737 1.00 48.12 H
ATOM 4963 3HD2 LEU A 517 18.661 −14.633 −2.010 1.00 48.12 H
ATOM 4964 N ASN A 518 22.463 −11.975 −5.331 1.00 51.68 N
ATOM 4965 CA ASN A 518 23.101 −10.761 −5.855 1.00 51.67 C
ATOM 4966 C ASN A 518 22.906 −10.579 −7.379 1.00 51.69 C
ATOM 4967 O ASN A 518 23.472 −9.645 −7.946 1.00 51.58 O
ATOM 4968 CB ASN A 518 24.593 −10.786 −5.468 1.00 51.37 C
ATOM 4969 CG ASN A 518 24.816 −10.698 −3.971 1.00 51.27 C
ATOM 4970 OD1 ASN A 518 25.105 −9.644 −3.434 1.00 51.49 O
ATOM 4971 ND2 ASN A 518 24.794 −11.802 −3.267 1.00 50.94 N
ATOM 4972 HN ASN A 518 23.014 −12.832 −5.309 1.00 0.00 H
ATOM 4973 1HD2 ASN A 518 24.602 −12.690 −3.739 1.00 0.00 H
ATOM 4974 2HD2 ASN A 518 24.938 −11.733 −2.282 1.00 0.00 H
ATOM 4975 HA ASN A 518 22.644 −9.905 −5.358 1.00 51.46 H
ATOM 4976 HB1 ASN A 518 25.031 −11.716 −5.831 1.00 51.46 H
ATOM 4977 HB2 ASN A 518 25.090 −9.940 −5.944 1.00 51.46 H

ATOM	4978	N	TRP	A	519	22.248	−11.541	−8.040	1.00	25.85	N
ATOM	4979	CA	TRP	A	519	22.129	−11.727	−9.497	1.00	25.89	C
ATOM	4980	C	TRP	A	519	23.455	−11.548	−10.268	1.00	25.65	C
ATOM	4981	O	TRP	A	519	23.581	−10.745	−11.195	1.00	25.72	O
ATOM	4982	CB	TRP	A	519	20.889	−11.013	−10.071	1.00	26.21	C
ATOM	4983	CG	TRP	A	519	20.049	−11.914	−10.935	1.00	26.35	C
ATOM	4984	CD1	TRP	A	519	18.989	−12.637	−10.500	1.00	26.48	C
ATOM	4985	CD2	TRP	A	519	20.267	−12.337	−12.320	1.00	26.37	C
ATOM	4986	NE1	TRP	A	519	18.524	−13.452	−11.516	1.00	26.59	N
ATOM	4987	CE2	TRP	A	519	19.303	−13.340	−12.645	1.00	26.52	C
ATOM	4988	CE3	TRP	A	519	21.202	−12.009	−13.328	1.00	26.28	C
ATOM	4989	CZ2	TRP	A	519	19.284	−14.005	−13.878	1.00	26.60	C
ATOM	4990	CZ3	TRP	A	519	21.202	−12.680	−14.568	1.00	26.34	C
ATOM	4991	CH2	TRP	A	519	20.251	−13.681	−14.844	1.00	26.50	C
ATOM	4992	HN	TRP	A	519	21.792	−12.237	−7.469	1.00	0.00	H
ATOM	4993	HE1	TRP	A	519	17.759	−14.133	−11.416	1.00	0.00	H
ATOM	4994	HA	TRP	A	519	21.899	−12.787	−9.610	1.00	26.24	H
ATOM	4995	HB1	TRP	A	519	20.278	−10.655	−9.242	1.00	26.24	H
ATOM	4996	HB2	TRP	A	519	21.221	−10.165	−10.670	1.00	26.24	H
ATOM	4997	HD1	TRP	A	519	18.565	−12.584	−9.497	1.00	26.24	H
ATOM	4998	HE3	TRP	A	519	21.935	−11.224	−13.144	1.00	26.24	H
ATOM	4999	HZ2	TRP	A	519	18.529	−14.764	−14.085	1.00	26.24	H
ATOM	5000	HZ3	TRP	A	519	21.946	−12.421	−15.321	1.00	26.24	H
ATOM	5001	HH2	TRP	A	519	20.265	−14.201	−15.802	1.00	26.24	H
ATOM	5002	N	VAL	A	520	24.496	−12.241	−9.793	1.00	25.37	N
ATOM	5003	CA	VAL	A	520	25.847	−12.242	−10.370	1.00	25.14	C
ATOM	5004	C	VAL	A	520	26.014	−13.417	−11.335	1.00	25.13	C

ATOM 5005 O VAL A 520 25.909 −14.580 −10.944 1.00 25.15 O

ATOM 5006 CB VAL A 520 26.925 −12.256 −9.269 1.00 24.85 C

ATOM 5007 CG1 VAL A 520 28.345 −12.373 −9.836 1.00 24.63 C

ATOM 5008 CG2 VAL A 520 26.877 −10.958 −8.453 1.00 24.87 C

ATOM 5009 HN VAL A 520 24.279 −12.953 −9.103 1.00 0.00 H

ATOM 5010 HA VAL A 520 25.966 −11.322 −10.942 1.00 25.02 H

ATOM 5011 HB VAL A 520 26.740 −13.099 −8.603 1.00 25.02 H

ATOM 5012 1HG1 VAL A 520 29.064 −12.378 −9.017 1.00 25.02 H

ATOM 5013 2HG1 VAL A 520 28.546 −11.525 −10.491 1.00 25.02 H

ATOM 5014 3HG1 VAL A 520 28.435 −13.299 −10.404 1.00 25.02 H

ATOM 5015 1HG2 VAL A 520 27.645 −10.985 −7.680 1.00 25.02 H

ATOM 5016 2HG2 VAL A 520 25.897 −10.857 −7.987 1.00 25.02 H

ATOM 5017 3HG2 VAL A 520 27.055 −10.108 −9.111 1.00 25.02 H

ATOM 5018 N SER A 521 26.391 −13.112 −12.574 1.00 25.13 N

ATOM 5019 CA SER A 521 26.748 −14.075 −13.622 1.00 25.14 C

ATOM 5020 C SER A 521 28.250 −14.068 −13.936 1.00 24.91 C

ATOM 5021 O SER A 521 28.964 −13.116 −13.618 1.00 24.76 O

ATOM 5022 CB SER A 521 25.935 −13.795 −14.889 1.00 25.37 C

ATOM 5023 OG SER A 521 24.554 −13.974 −14.634 1.00 25.58 O

ATOM 5024 HN SER A 521 26.569 −12.133 −12.774 1.00 0.00 H

ATOM 5025 HG SER A 521 24.279 −13.346 −13.956 1.00 0.00 H

ATOM 5026 HA SER A 521 26.484 −15.071 −13.267 1.00 25.15 H

ATOM 5027 HB1 SER A 521 26.111 −12.768 −15.210 1.00 25.15 H

ATOM 5028 HB2 SER A 521 26.247 −14.481 −15.676 1.00 25.15 H

ATOM 5029 N ALA A 522 28.724 −15.114 −14.614 1.00 24.89 N

ATOM 5030 CA ALA A 522 30.107 −15.283 −15.054 1.00 24.73 C

ATOM 5031 C ALA A 522 30.175 −15.861 −16.477 1.00 24.83 C

ATOM 5032 O ALA A 522 29.405 −16.760 −16.813 1.00 25.01 O

ATOM 5033 CB ALA A 522 30.824 −16.187 −14.049 1.00 24.46 C

ATOM 5034 HN ALA A 522 28.091 −15.888 −14.793 1.00 0.00 H

ATOM 5035 HA ALA A 522 30.593 −14.308 −15.052 1.00 24.78 H

ATOM 5036 HB1 ALA A 522 31.860 −16.326 −14.359 1.00 24.78 H

ATOM 5037 HB2 ALA A 522 30.324 −17.155 −14.009 1.00 24.78 H

ATOM 5038 HB3 ALA A 522 30.800 −15.725 −13.062 1.00 24.78 H

ATOM 5039 N GLY A 523 31.096 −15.380 −17.312 1.00 24.74 N

ATOM 5040 CA GLY A 523 31.263 −15.879 −18.680 1.00 24.82 C

ATOM 5041 C GLY A 523 32.318 −15.132 −19.487 1.00 24.76 C

ATOM 5042 O GLY A 523 32.798 −14.077 −19.073 1.00 24.65 O

ATOM 5043 HN GLY A 523 31.707 −14.629 −17.000 1.00 0.00 H

ATOM 5044 HA1 GLY A 523 31.550 −16.929 −18.629 1.00 24.74 H

ATOM 5045 HA2 GLY A 523 30.308 −15.788 −19.197 1.00 24.74 H

ATOM 5046 N VAL A 524 32.692 −15.693 −20.637 1.00 24.84 N

ATOM 5047 CA VAL A 524 33.604 −15.043 −21.592 1.00 24.80 C

ATOM 5048 C VAL A 524 32.809 −14.192 −22.578 1.00 24.97 C

ATOM 5049 O VAL A 524 31.868 −14.695 −23.200 1.00 25.12 O

ATOM 5050 CB VAL A 524 34.511 −16.048 −22.327 1.00 24.77 C

ATOM 5051 CG1 VAL A 524 35.590 −15.364 −23.183 1.00 24.84 C

ATOM 5052 CG2 VAL A 524 35.238 −16.966 −21.347 1.00 24.56 C

ATOM 5053 HN VAL A 524 32.219 −16.537 −20.929 1.00 0.00 H

ATOM 5054 HA VAL A 524 34.249 −14.372 −21.025 1.00 24.84 H

ATOM 5055 HB VAL A 524 33.890 −16.662 −22.979 1.00 24.84 H

ATOM 5056 1HG1 VAL A 524 36.198 −16.122 −23.676 1.00 24.84 H

ATOM 5057 2HG1 VAL A 524 36.225 −14.749 −22.545 1.00 24.84 H

ATOM 5058 3HG1 VAL A 524 35.113 −14.735 −23.935 1.00 24.84 H

ATOM 5059 1HG2 VAL A 524 35.869 −17.662 −21.900 1.00 24.84 H

ATOM 5060 2HG2 VAL A 524 34.508 −17.525 −20.762 1.00 24.84 H

ATOM 5061 3HG2 VAL A 524 35.857 −16.367 −20.679 1.00 24.84 H

ATOM 5062 N TYR A 525 33.285 −12.973 −22.831 1.00 24.94 N

ATOM 5063 CA TYR A 525 32.801 −12.090 −23.891 1.00 25.10 C

ATOM 5064 C TYR A 525 33.961 −11.477 −24.683 1.00 25.08 C

ATOM 5065 O TYR A 525 35.053 −11.266 −24.155 1.00 24.95 O

ATOM 5066 CB TYR A 525 31.830 −11.023 −23.342 1.00 25.14 C

ATOM 5067 CG TYR A 525 32.426 −9.796 −22.653 1.00 25.07 C

ATOM 5068 CD1 TYR A 525 32.019 −8.500 −23.042 1.00 25.21 C

ATOM 5069 CD2 TYR A 525 33.328 −9.940 −21.579 1.00 24.90 C

ATOM 5070 CE1 TYR A 525 32.497 −7.366 −22.349 1.00 25.19 C

ATOM 5071 CE2 TYR A 525 33.848 −8.809 −20.923 1.00 24.87 C

ATOM 5072 CZ TYR A 525 33.426 −7.519 −21.299 1.00 25.02 C

ATOM 5073 OH TYR A 525 33.890 −6.429 −20.636 1.00 25.02 O

ATOM 5074 HN TYR A 525 34.122 −12.672 −22.337 1.00 0.00 H

ATOM 5075 HH TYR A 525 34.391 −6.685 −19.843 1.00 0.00 H

ATOM 5076 HA TYR A 525 32.233 −12.709 −24.586 1.00 25.04 H

ATOM 5077 HB1 TYR A 525 31.234 −10.665 −24.181 1.00 25.04 H

ATOM 5078 HB2 TYR A 525 31.181 −11.517 −22.619 1.00 25.04 H

ATOM 5079 HD1 TYR A 525 31.333 −8.375 −23.880 1.00 25.04 H

ATOM 5080 HD2 TYR A 525 33.625 −10.937 −21.254 1.00 25.04 H

ATOM 5081 HE1 TYR A 525 32.147 −6.371 −22.626 1.00 25.04 H

ATOM 5082 HE2 TYR A 525 34.578 −8.931 −20.123 1.00 25.04 H

ATOM 5083 N LEU A 526 33.725 −11.244 −25.972 1.00 25.23 N

ATOM 5084 CA LEU A 526 34.641 −10.527 −26.855 1.00 25.24 C

ATOM 5085 C LEU A 526 34.562 −9.017 −26.599 1.00 25.29 C

ATOM 5086 O LEU A 526 33.475 −8.427 −26.583 1.00 25.42 O

ATOM 5087 CB LEU A 526 34.310 −10.854 −28.317 1.00 25.38 C

ATOM 5088 CG LEU A 526 34.385 −12.346 −28.675 1.00 25.42 C

ATOM 5089 CD1 LEU A 526 33.882 −12.540 −30.102 1.00 25.52 C

ATOM 5090 CD2 LEU A 526 35.795 −12.909 −28.552 1.00 25.28 C

ATOM 5091 HN LEU A 526 32.838 −11.545 −26.358 1.00 0.00 H

ATOM 5092 HA LEU A 526 35.657 −10.862 −26.646 1.00 25.35 H

ATOM 5093 HB1 LEU A 526 33.297 −10.507 −28.521 1.00 25.35 H

ATOM 5094 HB2 LEU A 526 35.014 −10.317 −28.953 1.00 25.35 H

ATOM 5095 HG LEU A 526 33.731 −12.898 −27.999 1.00 25.35 H

ATOM 5096 1HD1 LEU A 526 33.932 −13.597 −30.365 1.00 25.35 H

ATOM 5097 2HD1 LEU A 526 34.504 −11.966 −30.788 1.00 25.35 H

ATOM 5098 3HD1 LEU A 526 32.850 −12.196 −30.174 1.00 25.35 H

ATOM 5099 1HD2 LEU A 526 35.789 −13.966 −28.817 1.00 25.35 H

ATOM 5100 2HD2 LEU A 526 36.145 −12.794 −27.526 1.00 25.35 H

ATOM 5101 3HD2 LEU A 526 36.462 −12.370 −29.225 1.00 25.35 H

ATOM 5102 N ASN A 527 35.718 −8.384 −26.423 1.00 25.20 N

ATOM 5103 CA ASN A 527 35.811 −6.960 −26.107 1.00 25.28 C

ATOM 5104 C ASN A 527 35.888 −6.051 −27.351 1.00 25.40 C

ATOM 5105 O ASN A 527 35.729 −4.837 −27.228 1.00 25.38 O

ATOM 5106 CB ASN A 527 37.015 −6.773 −25.184 1.00 25.17 C

ATOM 5107 CG ASN A 527 37.027 −5.506 −24.359 1.00 25.29 C

ATOM 5108 OD1 ASN A 527 36.033 −4.807 −24.178 1.00 25.45 O

ATOM 5109 ND2 ASN A 527 38.176 −5.208 −23.802 1.00 25.21 N

ATOM 5110 HN ASN A 527 36.569 −8.943 −26.392 1.00 0.00 H

ATOM 5111 1HD2 ASN A 527 38.968 −5.823 −23.975 1.00 0.00 H

ATOM 5112 2HD2 ASN A 527 38.264 −4.352 −23.293 1.00 0.00 H

ATOM 5113 HA ASN A 527 34.917 −6.684 −25.548 1.00 25.30 H

ATOM 5114 HB1 ASN A 527 37.043 −7.619 −24.497 1.00 25.30 H

ATOM 5115 HB2 ASN A 527 37.913 −6.777 −25.802 1.00 25.30 H

ATOM 5116 N SER A 528 36.090 −6.606 −28.548 1.00 25.52 N

ATOM 5117 CA SER A 528 36.056 −5.843 −29.803 1.00 25.64 C

ATOM 5118 C SER A 528 34.652 −5.349 −30.168 1.00 25.78 C

ATOM 5119 O SER A 528 33.654 −6.021 −29.921 1.00 25.78 O

ATOM 5120 CB SER A 528 36.611 −6.699 −30.928 1.00 25.61 C

ATOM 5121 OG SER A 528 36.540 −6.004 −32.156 1.00 25.74 O

ATOM 5122 HN SER A 528 36.260 −7.605 −28.598 1.00 0.00 H

ATOM 5123 HG SER A 528 37.181 −6.512 −32.724 1.00 0.00 H

ATOM 5124 HA SER A 528 36.702 −4.973 −29.688 1.00 25.68 H

ATOM 5125 HB1 SER A 528 37.651 −6.944 −30.713 1.00 25.68 H

ATOM 5126 HB2 SER A 528 36.029 −7.618 −31.001 1.00 25.68 H

ATOM 5127 N ASN A 529 34.546 −4.165 −30.783 1.00 25.91 N

ATOM 5128 CA ASN A 529 33.255 −3.555 −31.131 1.00 26.06 C

ATOM 5129 C ASN A 529 32.624 −4.069 −32.441 1.00 26.09 C

ATOM 5130 O ASN A 529 31.402 −3.958 −32.584 1.00 26.15 O

ATOM 5131 CB ASN A 529 33.392 −2.019 −31.126 1.00 26.20 C

ATOM 5132 CG ASN A 529 33.468 −1.402 −29.734 1.00 26.29 C

ATOM 5133 OD1 ASN A 529 33.467 −2.061 −28.706 1.00 26.21 O

ATOM 5134 ND2 ASN A 529 33.310 −0.103 −29.648 1.00 26.46 N

ATOM 5135 HN ASN A 529 35.405 −3.686 −31.008 1.00 0.00 H

ATOM 5136 1HD2 ASN A 529 33.211 0.461 −30.472 1.00 0.00 H

ATOM 5137 2HD2 ASN A 529 33.266 0.283 −28.721 1.00 0.00 H

ATOM 5138 HA ASN A 529 32.562 −3.810 −30.329 1.00 26.17 H

ATOM 5139 HB1 ASN A 529 34.301 −1.755 −31.667 1.00 26.17 H

ATOM 5140 HB2 ASN A 529 32.528 −1.597 −31.640 1.00 26.17 H
ATOM 5141 N GLN A 530 33.425 −4.572 −33.390 1.00 26.08 N
ATOM 5142 CA GLN A 530 33.011 −4.841 −34.785 1.00 26.12 C
ATOM 5143 C GLN A 530 33.590 −6.134 −35.397 1.00 26.05 C
ATOM 5144 O GLN A 530 33.304 −6.448 −36.555 1.00 26.11 O
ATOM 5145 CB GLN A 530 33.422 −3.654 −35.676 1.00 26.24 C
ATOM 5146 CG GLN A 530 32.649 −2.357 −35.396 1.00 26.35 C
ATOM 5147 CD GLN A 530 32.961 −1.259 −36.412 1.00 26.46 C
ATOM 5148 OE1 GLN A 530 32.090 −0.560 −36.897 1.00 26.52 O
ATOM 5149 NE2 GLN A 530 34.198 −1.089 −36.828 1.00 26.49 N
ATOM 5150 HN GLN A 530 34.408 −4.677 −33.168 1.00 0.00 H
ATOM 5151 1HE2 GLN A 530 34.936 −1.695 −36.513 1.00 0.00 H
ATOM 5152 2HE2 GLN A 530 34.333 −0.395 −37.540 1.00 0.00 H
ATOM 5153 HA GLN A 530 31.924 −4.916 −34.805 1.00 26.27 H
ATOM 5154 HB1 GLN A 530 34.483 −3.460 −35.518 1.00 26.27 H
ATOM 5155 HB2 GLN A 530 33.254 −3.934 −36.716 1.00 26.27 H
ATOM 5156 HG1 GLN A 530 31.581 −2.572 −35.431 1.00 26.27 H
ATOM 5157 HG2 GLN A 530 32.916 −1.999 −34.402 1.00 26.27 H
ATOM 5158 N THR A 531 34.497 −6.803 −34.690 1.00 36.53 N
ATOM 5159 CA THR A 531 35.325 −7.900 −35.208 1.00 36.48 C
ATOM 5160 C THR A 531 35.672 −8.890 −34.083 1.00 36.37 C
ATOM 5161 O THR A 531 35.068 −8.845 −33.010 1.00 36.28 O
ATOM 5162 CB THR A 531 36.576 −7.358 −35.940 1.00 36.50 C
ATOM 5163 OG1 THR A 531 37.125 −6.235 −35.296 1.00 36.47 O
ATOM 5164 CG2 THR A 531 36.315 −6.944 −37.386 1.00 36.63 C
ATOM 5165 HN THR A 531 34.739 −6.488 −33.762 1.00 0.00 H
ATOM 5166 HG1 THR A 531 37.873 −6.609 −34.778 1.00 0.00 H

ATOM 5167 HA THR A 531 34.727 −8.439 −35.943 1.00 36.47 H
ATOM 5168 HB THR A 531 37.330 −8.145 −35.946 1.00 36.47 H
ATOM 5169 1HG2 THR A 531 37.239 −6.575 −37.831 1.00 36.47 H
ATOM 5170 2HG2 THR A 531 35.562 −6.156 −37.409 1.00 36.47 H
ATOM 5171 3HG2 THR A 531 35.958 −7.804 −37.952 1.00 36.47 H
ATOM 5172 N ALA A 532 36.316 −9.995 −34.460 1.00 25.79 N
ATOM 5173 CA ALA A 532 36.389 −11.229 −33.680 1.00 25.70 C
ATOM 5174 C ALA A 532 37.644 −11.292 −32.784 1.00 25.59 C
ATOM 5175 O ALA A 532 38.484 −12.167 −32.978 1.00 25.61 O
ATOM 5176 CB ALA A 532 36.271 −12.399 −34.674 1.00 25.80 C
ATOM 5177 HN ALA A 532 36.841 −9.969 −35.319 1.00 0.00 H
ATOM 5178 HA ALA A 532 35.516 −11.260 −33.028 1.00 25.70 H
ATOM 5179 HB1 ALA A 532 36.321 −13.343 −34.132 1.00 25.70 H
ATOM 5180 HB2 ALA A 532 37.089 −12.350 −35.393 1.00 25.70 H
ATOM 5181 HB3 ALA A 532 35.320 −12.333 −35.202 1.00 25.70 H
ATOM 5182 N GLU A 533 37.823 −10.332 −31.868 1.00 25.49 N
ATOM 5183 CA GLU A 533 39.034 −10.212 −31.031 1.00 25.38 C
ATOM 5184 C GLU A 533 38.749 −10.050 −29.522 1.00 25.24 C
ATOM 5185 O GLU A 533 37.687 −9.559 −29.118 1.00 25.25 O
ATOM 5186 CB GLU A 533 39.954 −9.064 −31.506 1.00 25.43 C
ATOM 5187 CG GLU A 533 40.225 −8.983 −33.021 1.00 25.56 C
ATOM 5188 CD GLU A 533 39.280 −8.040 −33.783 1.00 25.58 C
ATOM 5189 OE1 GLU A 533 39.478 −7.827 −34.996 1.00 25.70 O
ATOM 5190 OE2 GLU A 533 38.242 −7.589 −33.243 1.00 25.50 O
ATOM 5191 HN GLU A 533 37.152 −9.578 −31.829 1.00 0.00 H
ATOM 5192 HA GLU A 533 39.595 −11.139 −31.150 1.00 25.46 H
ATOM 5193 HB1 GLU A 533 39.495 −8.124 −31.201 1.00 25.46 H

ATOM	5194	HB2	GLU	A	533	40.914	−9.180	−31.004	1.00	25.46	H
ATOM	5195	HG1	GLU	A	533	41.247	−8.633	−33.167	1.00	25.46	H
ATOM	5196	HG2	GLU	A	533	40.120	−9.984	−33.440	1.00	25.46	H
ATOM	5197	N	ASN	A	534	39.808	−10.259	−28.724	1.00	25.12	N
ATOM	5198	CA	ASN	A	534	39.939	−9.916	−27.298	1.00	24.99	C
ATOM	5199	C	ASN	A	534	38.999	−10.724	−26.362	1.00	24.90	C
ATOM	5200	O	ASN	A	534	37.965	−10.212	−25.916	1.00	24.87	O
ATOM	5201	CB	ASN	A	534	39.848	−8.384	−27.157	1.00	25.07	C
ATOM	5202	CG	ASN	A	534	40.426	−7.858	−25.851	1.00	24.96	C
ATOM	5203	OD1	ASN	A	534	39.806	−7.889	−24.800	1.00	24.83	O
ATOM	5204	ND2	ASN	A	534	41.492	−7.110	−25.944	1.00	25.03	N
ATOM	5205	HN	ASN	A	534	40.632	−10.655	−29.167	1.00	0.00	H
ATOM	5206	1HD2	ASN	A	534	42.014	−7.151	−26.814	1.00	0.00	H
ATOM	5207	2HD2	ASN	A	534	42.019	−6.945	−25.079	1.00	0.00	H
ATOM	5208	HA	ASN	A	534	40.956	−10.187	−27.013	1.00	24.97	H
ATOM	5209	HB1	ASN	A	534	40.394	−7.929	−27.984	1.00	24.97	H
ATOM	5210	HB2	ASN	A	534	38.798	−8.095	−27.211	1.00	24.97	H
ATOM	5211	N	PRO	A	535	39.288	−12.022	−26.119	1.00	24.87	N
ATOM	5212	CA	PRO	A	535	38.470	−12.911	−25.288	1.00	24.80	C
ATOM	5213	C	PRO	A	535	38.708	−12.694	−23.780	1.00	24.63	C
ATOM	5214	O	PRO	A	535	39.639	−13.249	−23.187	1.00	24.51	O
ATOM	5215	CB	PRO	A	535	38.854	−14.324	−25.746	1.00	24.82	C
ATOM	5216	CG	PRO	A	535	40.320	−14.183	−26.162	1.00	24.83	C
ATOM	5217	CD	PRO	A	535	40.396	−12.762	−26.716	1.00	24.92	C
ATOM	5218	HA	PRO	A	535	37.417	−12.738	−25.511	1.00	24.77	H
ATOM	5219	HB1	PRO	A	535	38.748	−15.039	−24.930	1.00	24.77	H
ATOM	5220	HB2	PRO	A	535	38.239	−14.640	−26.589	1.00	24.77	H

```
ATOM   5221  HG1 PRO A 535      40.982 −14.303 −25.304  1.00 24.77           H
ATOM   5222  HG2 PRO A 535      40.580 −14.914 −26.927  1.00 24.77           H
ATOM   5223  HD1 PRO A 535      41.346 −12.302 −26.443  1.00 24.77           H
ATOM   5224  HD2 PRO A 535      40.297 −12.776 −27.801  1.00 24.77           H
ATOM   5225  N   VAL A 536      37.755 −12.043 −23.107  1.00 24.61           N
ATOM   5226  CA  VAL A 536      37.817 −11.748 −21.663  1.00 24.46           C
ATOM   5227  C   VAL A 536      36.796 −12.574 −20.883  1.00 24.45           C
ATOM   5228  O   VAL A 536      35.596 −12.459 −21.121  1.00 24.60           O
ATOM   5229  CB  VAL A 536      37.629 −10.237 −21.417  1.00 24.50           C
ATOM   5230  CG1 VAL A 536      37.601  −9.885 −19.925  1.00 24.36           C
ATOM   5231  CG2 VAL A 536      38.771  −9.436 −22.052  1.00 24.55           C
ATOM   5232  HN  VAL A 536      36.988 −11.634 −23.636  1.00  0.00           H
ATOM   5233  HA  VAL A 536      38.810 −12.023 −21.308  1.00 24.50           H
ATOM   5234  HB  VAL A 536      36.688  −9.923 −21.869  1.00 24.50           H
ATOM   5235 1HG1 VAL A 536      37.467  −8.810 −19.807  1.00 24.50           H
ATOM   5236 2HG1 VAL A 536      38.541 −10.186 −19.462  1.00 24.50           H
ATOM   5237 3HG1 VAL A 536      36.775 −10.409 −19.443  1.00 24.50           H
ATOM   5238 1HG2 VAL A 536      38.617  −8.373 −21.866  1.00 24.50           H
ATOM   5239 2HG2 VAL A 536      38.789  −9.617 −23.127  1.00 24.50           H
ATOM   5240 3HG2 VAL A 536      39.720  −9.747 −21.616  1.00 24.50           H
ATOM   5241  N   PHE A 537      37.233 −13.305 −19.852  1.00 24.29           N
ATOM   5242  CA  PHE A 537      36.330 −13.835 −18.824  1.00 24.26           C
ATOM   5243  C   PHE A 537      35.964 −12.719 −17.835  1.00 24.26           C
ATOM   5244  O   PHE A 537      36.842 −12.129 −17.201  1.00 24.16           O
ATOM   5245  CB  PHE A 537      36.958 −15.051 −18.122  1.00 24.07           C
ATOM   5246  CG  PHE A 537      35.997 −15.842 −17.241  1.00 24.06           C
ATOM   5247  CD1 PHE A 537      35.513 −17.097 −17.663  1.00 24.21           C
```

ATOM 5248 CD2 PHE A 537 35.586 −15.337 −15.992 1.00 23.92 C
ATOM 5249 CE1 PHE A 537 34.633 −17.833 −16.848 1.00 24.24 C
ATOM 5250 CE2 PHE A 537 34.703 −16.071 −15.179 1.00 23.93 C
ATOM 5251 CZ PHE A 537 34.227 −17.322 −15.606 1.00 24.09 C
ATOM 5252 HN PHE A 537 38.221 −13.255 −19.621 1.00 0.00 H
ATOM 5253 HA PHE A 537 35.415 −14.164 −19.316 1.00 24.14 H
ATOM 5254 HB1 PHE A 537 37.347 −15.722 −18.888 1.00 24.14 H
ATOM 5255 HB2 PHE A 537 37.778 −14.697 −17.498 1.00 24.14 H
ATOM 5256 HD1 PHE A 537 35.823 −17.500 −18.627 1.00 24.14 H
ATOM 5257 HD2 PHE A 537 35.955 −14.369 −15.652 1.00 24.14 H
ATOM 5258 HE1 PHE A 537 34.266 −18.803 −17.183 1.00 24.14 H
ATOM 5259 HE2 PHE A 537 34.388 −15.668 −14.216 1.00 24.14 H
ATOM 5260 HZ PHE A 537 33.545 −17.893 −14.975 1.00 24.14 H
ATOM 5261 N ALA A 538 34.671 −12.507 −17.602 1.00 24.40 N
ATOM 5262 CA ALA A 538 34.141 −11.521 −16.666 1.00 24.47 C
ATOM 5263 C ALA A 538 33.212 −12.164 −15.628 1.00 24.47 C
ATOM 5264 O ALA A 538 32.470 −13.099 −15.933 1.00 24.57 O
ATOM 5265 CB ALA A 538 33.431 −10.421 −17.464 1.00 24.69 C
ATOM 5266 HN ALA A 538 33.994 −13.052 −18.129 1.00 0.00 H
ATOM 5267 HA ALA A 538 34.980 −11.069 −16.137 1.00 24.52 H
ATOM 5268 HB1 ALA A 538 33.029 −9.676 −16.778 1.00 24.52 H
ATOM 5269 HB2 ALA A 538 32.617 −10.859 −18.042 1.00 24.52 H
ATOM 5270 HB3 ALA A 538 34.142 −9.946 −18.140 1.00 24.52 H
ATOM 5271 N VAL A 539 33.139 −11.547 −14.447 1.00 24.36 N
ATOM 5272 CA VAL A 539 32.083 −11.777 −13.449 1.00 24.36 C
ATOM 5273 C VAL A 539 31.351 −10.462 −13.197 1.00 24.52 C
ATOM 5274 O VAL A 539 31.989 −9.424 −13.007 1.00 24.48 O

ATOM	5275	CB	VAL	A	539	32.650	−12.377	−12.151	1.00	24.11	C
ATOM	5276	CG1	VAL	A	539	31.546	−12.621	−11.119	1.00	24.11	C
ATOM	5277	CG2	VAL	A	539	33.333	−13.723	−12.418	1.00	23.95	C
ATOM	5278	HN	VAL	A	539	33.774	−10.772	−14.276	1.00	0.00	H
ATOM	5279	HA	VAL	A	539	31.370	−12.487	−13.868	1.00	24.27	H
ATOM	5280	HB	VAL	A	539	33.381	−11.686	−11.731	1.00	24.27	H
ATOM	5281	1HG1	VAL	A	539	31.981	−13.045	−10.214	1.00	24.27	H
ATOM	5282	2HG1	VAL	A	539	30.812	−13.315	−11.529	1.00	24.27	H
ATOM	5283	3HG1	VAL	A	539	31.058	−11.677	−10.878	1.00	24.27	H
ATOM	5284	1HG2	VAL	A	539	33.724	−14.124	−11.483	1.00	24.27	H
ATOM	5285	2HG2	VAL	A	539	34.152	−13.582	−13.123	1.00	24.27	H
ATOM	5286	3HG2	VAL	A	539	32.609	−14.422	−12.838	1.00	24.27	H
ATOM	5287	N	PHE	A	540	30.023	−10.468	−13.299	1.00	24.72	N
ATOM	5288	CA	PHE	A	540	29.228	−9.250	−13.457	1.00	24.94	C
ATOM	5289	C	PHE	A	540	27.805	−9.372	−12.899	1.00	25.14	C
ATOM	5290	O	PHE	A	540	27.212	−10.445	−12.858	1.00	25.11	O
ATOM	5291	CB	PHE	A	540	29.207	−8.850	−14.944	1.00	25.07	C
ATOM	5292	CG	PHE	A	540	28.480	−9.814	−15.865	1.00	25.16	C
ATOM	5293	CD1	PHE	A	540	27.113	−9.623	−16.147	1.00	25.41	C
ATOM	5294	CD2	PHE	A	540	29.167	−10.898	−16.447	1.00	25.03	C
ATOM	5295	CE1	PHE	A	540	26.438	−10.507	−17.008	1.00	25.50	C
ATOM	5296	CE2	PHE	A	540	28.491	−11.785	−17.303	1.00	25.14	C
ATOM	5297	CZ	PHE	A	540	27.127	−11.588	−17.585	1.00	25.37	C
ATOM	5298	HN	PHE	A	540	29.551	−11.355	−13.465	1.00	0.00	H
ATOM	5299	HA	PHE	A	540	29.731	−8.454	−12.908	1.00	25.14	H
ATOM	5300	HB1	PHE	A	540	28.722	−7.877	−15.024	1.00	25.14	H
ATOM	5301	HB2	PHE	A	540	30.239	−8.771	−15.286	1.00	25.14	H

ATOM	5302	HD1	PHE	A	540	26.577	−8.788	−15.697	1.00	25.14	H
ATOM	5303	HD2	PHE	A	540	30.225	−11.049	−16.233	1.00	25.14	H
ATOM	5304	HE1	PHE	A	540	25.381	−10.354	−17.228	1.00	25.14	H
ATOM	5305	HE2	PHE	A	540	29.024	−12.625	−17.748	1.00	25.14	H
ATOM	5306	HZ	PHE	A	540	26.604	−12.274	−18.251	1.00	25.14	H
ATOM	5307	N	LYS	A	541	27.240	−8.220	−12.553	1.00	25.36	N
ATOM	5308	CA	LYS	A	541	25.839	−7.947	−12.214	1.00	25.60	C
ATOM	5309	C	LYS	A	541	25.263	−7.008	−13.290	1.00	25.85	C
ATOM	5310	O	LYS	A	541	26.030	−6.417	−14.050	1.00	25.83	O
ATOM	5311	CB	LYS	A	541	25.860	−7.346	−10.797	1.00	25.60	C
ATOM	5312	CG	LYS	A	541	24.499	−7.194	−10.108	1.00	25.88	C
ATOM	5313	CD	LYS	A	541	24.577	−6.475	−8.746	1.00	25.86	C
ATOM	5314	CE	LYS	A	541	25.443	−7.150	−7.667	1.00	25.60	C
ATOM	5315	NZ	LYS	A	541	26.884	−6.802	−7.778	1.00	25.30	N1+
ATOM	5316	HN	LYS	A	541	27.814	−7.392	−12.688	1.00	0.00	H
ATOM	5317	HZ1	LYS	A	541	27.421	−7.108	−6.980	1.00	0.00	H
ATOM	5318	HZ2	LYS	A	541	27.036	−5.788	−7.866	1.00	0.00	H
ATOM	5319	HZ3	LYS	A	541	27.294	−7.172	−8.620	1.00	0.00	H
ATOM	5320	HA	LYS	A	541	25.277	−8.881	−12.206	1.00	25.65	H
ATOM	5321	HB1	LYS	A	541	26.479	−7.989	−10.172	1.00	25.65	H
ATOM	5322	HB2	LYS	A	541	26.313	−6.356	−10.862	1.00	25.65	H
ATOM	5323	HG1	LYS	A	541	23.843	−6.622	−10.763	1.00	25.65	H
ATOM	5324	HG2	LYS	A	541	24.079	−8.187	−9.950	1.00	25.65	H
ATOM	5325	HD1	LYS	A	541	24.981	−5.478	−8.920	1.00	25.65	H
ATOM	5326	HD2	LYS	A	541	23.563	−6.394	−8.355	1.00	25.65	H
ATOM	5327	HE1	LYS	A	541	25.084	−6.836	−6.687	1.00	25.65	H
ATOM	5328	HE2	LYS	A	541	25.338	−8.231	−7.763	1.00	25.65	H

ATOM 5329 N ASP A 542 23.944 −6.818 −13.330 1.00 36.37 N

ATOM 5330 CA ASP A 542 23.222 −6.048 −14.366 1.00 36.63 C

ATOM 5331 C ASP A 542 23.896 −4.734 −14.809 1.00 36.65 C

ATOM 5332 O ASP A 542 24.173 −4.557 −15.994 1.00 36.70 O

ATOM 5333 CB ASP A 542 21.796 −5.737 −13.875 1.00 36.90 C

ATOM 5334 CG ASP A 542 20.981 −6.974 −13.498 1.00 36.90 C

ATOM 5335 OD1 ASP A 542 21.248 −8.068 −14.038 1.00 36.81 O

ATOM 5336 OD2 ASP A 542 20.002 −6.837 −12.738 1.00 36.99 O

ATOM 5337 HN ASP A 542 23.380 −7.414 −12.740 1.00 0.00 H

ATOM 5338 HA ASP A 542 23.134 −6.685 −15.246 1.00 36.74 H

ATOM 5339 HB1 ASP A 542 21.869 −5.095 −12.997 1.00 36.74 H

ATOM 5340 HB2 ASP A 542 21.268 −5.209 −14.669 1.00 36.74 H

ATOM 5341 N ASN A 543 24.298 −3.885 −13.860 1.00 26.35 N

ATOM 5342 CA ASN A 543 24.885 −2.570 −14.147 1.00 26.44 C

ATOM 5343 C ASN A 543 26.396 −2.501 −13.831 1.00 26.21 C

ATOM 5344 O ASN A 543 26.945 −1.414 −13.664 1.00 26.30 O

ATOM 5345 CB ASN A 543 24.076 −1.470 −13.425 1.00 26.73 C

ATOM 5346 CG ASN A 543 22.606 −1.368 −13.813 1.00 27.00 C

ATOM 5347 OD1 ASN A 543 21.953 −2.294 −14.255 1.00 26.98 O

ATOM 5348 ND2 ASN A 543 21.981 −0.257 −13.505 1.00 27.26 N

ATOM 5349 HN ASN A 543 24.028 −4.088 −12.912 1.00 0.00 H

ATOM 5350 1HD2 ASN A 543 22.481 0.518 −13.116 1.00 0.00 H

ATOM 5351 2HD2 ASN A 543 21.007 −0.232 −13.745 1.00 0.00 H

ATOM 5352 HA ASN A 543 24.774 −2.397 −15.218 1.00 26.66 H

ATOM 5353 HB1 ASN A 543 24.127 −1.666 −12.354 1.00 26.66 H

ATOM 5354 HB2 ASN A 543 24.547 −0.511 −13.642 1.00 26.66 H

ATOM 5355 N GLU A 544 27.044 −3.641 −13.567 1.00 25.94 N

ATOM	5356	CA	GLU	A	544	28.320	−3.684	−12.845	1.00	25.71	C
ATOM	5357	C	GLU	A	544	29.136	−4.944	−13.186	1.00	25.44	C
ATOM	5358	O	GLU	A	544	28.926	−6.011	−12.610	1.00	25.36	O
ATOM	5359	CB	GLU	A	544	28.009	−3.592	−11.337	1.00	25.69	C
ATOM	5360	CG	GLU	A	544	29.250	−3.611	−10.429	1.00	25.43	C
ATOM	5361	CD	GLU	A	544	28.901	−3.792	−8.942	1.00	25.50	C
ATOM	5362	OE1	GLU	A	544	27.930	−4.535	−8.646	1.00	25.63	O
ATOM	5363	OE2	GLU	A	544	29.795	−3.486	−8.126	1.00	25.44	O
ATOM	5364	HN	GLU	A	544	26.555	−4.518	−13.728	1.00	0.00	H
ATOM	5365	HA	GLU	A	544	28.905	−2.809	−13.129	1.00	25.57	H
ATOM	5366	HB1	GLU	A	544	27.469	−2.662	−11.157	1.00	25.57	H
ATOM	5367	HB2	GLU	A	544	27.377	−4.438	−11.067	1.00	25.57	H
ATOM	5368	HG1	GLU	A	544	29.894	−4.434	−10.738	1.00	25.57	H
ATOM	5369	HG2	GLU	A	544	29.783	−2.668	−10.549	1.00	25.57	H
ATOM	5370	N	ILE	A	545	30.198	−4.801	−13.985	1.00	25.30	N
ATOM	5371	CA	ILE	A	545	31.266	−5.814	−14.034	1.00	25.04	C
ATOM	5372	C	ILE	A	545	32.076	−5.717	−12.738	1.00	24.84	C
ATOM	5373	O	ILE	A	545	32.664	−4.669	−12.454	1.00	24.84	O
ATOM	5374	CB	ILE	A	545	32.156	−5.677	−15.287	1.00	25.00	C
ATOM	5375	CG1	ILE	A	545	31.302	−5.785	−16.568	1.00	25.14	C
ATOM	5376	CG2	ILE	A	545	33.254	−6.755	−15.266	1.00	24.73	C
ATOM	5377	CD1	ILE	A	545	32.078	−5.541	−17.866	1.00	25.16	C
ATOM	5378	HN	ILE	A	545	30.361	−3.908	−14.423	1.00	0.00	H
ATOM	5379	HA	ILE	A	545	30.795	−6.797	−14.063	1.00	25.01	H
ATOM	5380	HB	ILE	A	545	32.632	−4.697	−15.269	1.00	25.01	H
ATOM	5381	1HG1	ILE	A	545	30.874	−6.787	−16.609	1.00	25.01	H
ATOM	5382	2HG1	ILE	A	545	30.500	−5.049	−16.506	1.00	25.01	H

ATOM 5383 1HG2 ILE A 545 33.880 −6.654 −16.153 1.00 25.01 H

ATOM 5384 2HG2 ILE A 545 32.794 −7.743 −15.257 1.00 25.01 H

ATOM 5385 3HG2 ILE A 545 33.867 −6.632 −14.373 1.00 25.01 H

ATOM 5386 1HD1 ILE A 545 31.403 −5.636 −18.717 1.00 25.01 H

ATOM 5387 2HD1 ILE A 545 32.879 −6.275 −17.955 1.00 25.01 H

ATOM 5388 3HD1 ILE A 545 32.505 −4.538 −17.852 1.00 25.01 H

ATOM 5389 N LEU A 546 32.133 −6.804 −11.970 1.00 24.68 N

ATOM 5390 CA LEU A 546 32.840 −6.872 −10.693 1.00 24.48 C

ATOM 5391 C LEU A 546 34.351 −7.012 −10.895 1.00 24.28 C

ATOM 5392 O LEU A 546 35.111 −6.214 −10.352 1.00 24.26 O

ATOM 5393 CB LEU A 546 32.286 −8.039 −9.850 1.00 24.36 C

ATOM 5394 CG LEU A 546 30.801 −7.923 −9.469 1.00 24.55 C

ATOM 5395 CD1 LEU A 546 30.341 −9.201 −8.769 1.00 24.40 C

ATOM 5396 CD2 LEU A 546 30.583 −6.765 −8.503 1.00 24.77 C

ATOM 5397 HN LEU A 546 31.714 −7.659 −12.319 1.00 0.00 H

ATOM 5398 HA LEU A 546 32.654 −5.944 −10.152 1.00 24.47 H

ATOM 5399 HB1 LEU A 546 32.417 −8.959 −10.420 1.00 24.47 H

ATOM 5400 HB2 LEU A 546 32.867 −8.096 −8.929 1.00 24.47 H

ATOM 5401 HG LEU A 546 30.205 −7.765 −10.368 1.00 24.47 H

ATOM 5402 1HD1 LEU A 546 29.288 −9.111 −8.502 1.00 24.47 H

ATOM 5403 2HD1 LEU A 546 30.932 −9.354 −7.866 1.00 24.47 H

ATOM 5404 3HD1 LEU A 546 30.474 −10.051 −9.439 1.00 24.47 H

ATOM 5405 1HD2 LEU A 546 29.526 −6.700 −8.246 1.00 24.47 H

ATOM 5406 2HD2 LEU A 546 30.900 −5.834 −8.973 1.00 24.47 H

ATOM 5407 3HD2 LEU A 546 31.167 −6.931 −7.598 1.00 24.47 H

ATOM 5408 N TYR A 547 34.778 −7.973 −11.715 1.00 24.15 N

ATOM 5409 CA TYR A 547 36.187 −8.288 −11.978 1.00 23.98 C

ATOM	5410	C	TYR	A	547	36.325	−9.150	−13.250	1.00	24.01		C
ATOM	5411	O	TYR	A	547	35.342	−9.718	−13.734	1.00	24.08		O
ATOM	5412	CB	TYR	A	547	36.816	−8.955	−10.734	1.00	23.71		C
ATOM	5413	CG	TYR	A	547	35.981	−10.021	−10.046	1.00	23.64		C
ATOM	5414	CD1	TYR	A	547	36.069	−11.354	−10.476	1.00	23.49		C
ATOM	5415	CD2	TYR	A	547	35.151	−9.691	−8.956	1.00	23.75		C
ATOM	5416	CE1	TYR	A	547	35.326	−12.359	−9.829	1.00	23.44		C
ATOM	5417	CE2	TYR	A	547	34.375	−10.683	−8.329	1.00	23.71		C
ATOM	5418	CZ	TYR	A	547	34.464	−12.022	−8.763	1.00	23.55		C
ATOM	5419	OH	TYR	A	547	33.758	−12.990	−8.126	1.00	23.53		O
ATOM	5420	HN	TYR	A	547	34.087	−8.579	−12.143	1.00	0.00		H
ATOM	5421	HH	TYR	A	547	33.155	−12.609	−7.463	1.00	0.00		H
ATOM	5422	HA	TYR	A	547	36.711	−7.349	−12.153	1.00	23.75		H
ATOM	5423	HB1	TYR	A	547	37.754	−9.417	−11.043	1.00	23.75		H
ATOM	5424	HB2	TYR	A	547	37.020	−8.171	−10.005	1.00	23.75		H
ATOM	5425	HD1	TYR	A	547	36.716	−11.612	−11.315	1.00	23.75		H
ATOM	5426	HD2	TYR	A	547	35.110	−8.663	−8.597	1.00	23.75		H
ATOM	5427	HE1	TYR	A	547	35.416	−13.397	−10.151	1.00	23.75		H
ATOM	5428	HE2	TYR	A	547	33.707	−10.417	−7.510	1.00	23.75		H
ATOM	5429	N	GLN	A	548	37.521	−9.172	−13.848	1.00	37.99		N
ATOM	5430	CA	GLN	A	548	37.803	−9.771	−15.164	1.00	38.04		C
ATOM	5431	C	GLN	A	548	39.182	−10.448	−15.201	1.00	37.85		C
ATOM	5432	O	GLN	A	548	40.060	−10.084	−14.419	1.00	37.67		O
ATOM	5433	CB	GLN	A	548	37.769	−8.695	−16.268	1.00	38.24		C
ATOM	5434	CG	GLN	A	548	36.420	−7.986	−16.448	1.00	38.45		C
ATOM	5435	CD	GLN	A	548	36.324	−7.177	−17.745	1.00	38.64		C
ATOM	5436	OE1	GLN	A	548	35.285	−7.105	−18.378	1.00	38.81		O

ATOM	5437	NE2	GLN	A	548	37.386	−6.568	−18.228	1.00	38.63	N
ATOM	5438	HN	GLN	A	548	38.307	−8.741	−13.381	1.00	0.00	H
ATOM	5439	1HE2	GLN	A	548	38.290	−6.686	−17.807	1.00	0.00	H
ATOM	5440	2HE2	GLN	A	548	37.279	−6.137	−19.132	1.00	0.00	H
ATOM	5441	HA	GLN	A	548	37.040	−10.519	−15.380	1.00	38.26	H
ATOM	5442	HB1	GLN	A	548	38.517	−7.940	−16.026	1.00	38.26	H
ATOM	5443	HB2	GLN	A	548	38.027	−9.173	−17.213	1.00	38.26	H
ATOM	5444	HG1	GLN	A	548	35.632	−8.739	−16.452	1.00	38.26	H
ATOM	5445	HG2	GLN	A	548	36.271	−7.309	−15.607	1.00	38.26	H
ATOM	5446	N	VAL	A	549	39.417	−11.281	−16.218	1.00	23.90	N
ATOM	5447	CA	VAL	A	549	40.751	−11.695	−16.682	1.00	23.78	C
ATOM	5448	C	VAL	A	549	40.706	−12.018	−18.191	1.00	23.92	C
ATOM	5449	O	VAL	A	549	39.733	−12.636	−18.636	1.00	24.02	O
ATOM	5450	CB	VAL	A	549	41.277	−12.878	−15.835	1.00	23.57	C
ATOM	5451	CG1	VAL	A	549	40.557	−14.206	−16.109	1.00	23.62	C
ATOM	5452	CG2	VAL	A	549	42.782	−13.096	−16.004	1.00	23.45	C
ATOM	5453	HN	VAL	A	549	38.631	−11.555	−16.804	1.00	0.00	H
ATOM	5454	HA	VAL	A	549	41.430	−10.854	−16.540	1.00	23.75	H
ATOM	5455	HB	VAL	A	549	41.103	−12.627	−14.789	1.00	23.75	H
ATOM	5456	1HG1	VAL	A	549	40.981	−14.987	−15.478	1.00	23.75	H
ATOM	5457	2HG1	VAL	A	549	40.682	−14.478	−17.157	1.00	23.75	H
ATOM	5458	3HG1	VAL	A	549	39.495	−14.097	−15.887	1.00	23.75	H
ATOM	5459	1HG2	VAL	A	549	43.099	−13.938	−15.388	1.00	23.75	H
ATOM	5460	2HG2	VAL	A	549	43.317	−12.198	−15.694	1.00	23.75	H
ATOM	5461	3HG2	VAL	A	549	43.004	−13.308	−17.050	1.00	23.75	H
ATOM	5462	N	PRO	A	550	41.671	−11.567	−19.015	1.00	47.09	N
ATOM	5463	CA	PRO	A	550	41.814	−12.059	−20.388	1.00	47.23	C

ATOM 5464 C PRO A 550 42.198 −13.549 −20.396 1.00 47.14 C

ATOM 5465 O PRO A 550 42.815 −14.042 −19.449 1.00 47.00 O

ATOM 5466 CB PRO A 550 42.892 −11.179 −21.026 1.00 47.30 C

ATOM 5467 CG PRO A 550 43.774 −10.790 −19.843 1.00 47.15 C

ATOM 5468 CD PRO A 550 42.778 −10.674 −18.689 1.00 47.02 C

ATOM 5469 HA PRO A 550 40.873 −11.924 −20.921 1.00 47.13 H

ATOM 5470 HB1 PRO A 550 43.461 −11.736 −21.770 1.00 47.13 H

ATOM 5471 HB2 PRO A 550 42.451 −10.297 −21.491 1.00 47.13 H

ATOM 5472 HG1 PRO A 550 44.519 −11.560 −19.641 1.00 47.13 H

ATOM 5473 HG2 PRO A 550 44.274 −9.839 −20.025 1.00 47.13 H

ATOM 5474 HD1 PRO A 550 43.247 −10.979 −17.753 1.00 47.13 H

ATOM 5475 HD2 PRO A 550 42.422 −9.648 −18.599 1.00 47.13 H

ATOM 5476 N LEU A 551 41.780 −14.292 −21.424 1.00 45.69 N

ATOM 5477 CA LEU A 551 42.152 −15.708 −21.600 1.00 45.66 C

ATOM 5478 C LEU A 551 43.313 −15.930 −22.584 1.00 45.74 C

ATOM 5479 O LEU A 551 43.915 −17.003 −22.566 1.00 45.70 O

ATOM 5480 CB LEU A 551 40.908 −16.515 −21.998 1.00 45.79 C

ATOM 5481 CG LEU A 551 39.814 −16.563 −20.916 1.00 45.70 C

ATOM 5482 CD1 LEU A 551 38.662 −17.422 −21.430 1.00 45.87 C

ATOM 5483 CD2 LEU A 551 40.292 −17.178 −19.594 1.00 45.57 C

ATOM 5484 HN LEU A 551 41.217 −13.848 −22.145 1.00 0.00 H

ATOM 5485 HA LEU A 551 42.478 −16.078 −20.628 1.00 45.72 H

ATOM 5486 HB1 LEU A 551 40.482 −16.066 −22.895 1.00 45.72 H

ATOM 5487 HB2 LEU A 551 41.219 −17.537 −22.215 1.00 45.72 H

ATOM 5488 HG LEU A 551 39.451 −15.552 −20.730 1.00 45.72 H

ATOM 5489 1HD1 LEU A 551 37.877 −17.467 −20.675 1.00 45.72 H

ATOM 5490 2HD1 LEU A 551 39.024 −18.429 −21.638 1.00 45.72 H

ATOM 5491 3HD1 LEU A 551 38.262 −16.984 −22.344 1.00 45.72 H
ATOM 5492 1HD2 LEU A 551 39.472 −17.181 −18.876 1.00 45.72 H
ATOM 5493 2HD2 LEU A 551 41.119 −16.590 −19.197 1.00 45.72 H
ATOM 5494 3HD2 LEU A 551 40.625 −18.201 −19.768 1.00 45.72 H
ATOM 5495 N ALA A 552 43.634 −14.908 −23.375 1.00 24.27 N
ATOM 5496 CA ALA A 552 44.817 −14.755 −24.218 1.00 24.37 C
ATOM 5497 C ALA A 552 44.986 −13.252 −24.541 1.00 24.41 C
ATOM 5498 O ALA A 552 44.158 −12.438 −24.119 1.00 24.35 O
ATOM 5499 CB ALA A 552 44.644 −15.607 −25.482 1.00 24.56 C
ATOM 5500 HN ALA A 552 43.098 −14.058 −23.282 1.00 0.00 H
ATOM 5501 HA ALA A 552 45.693 −15.103 −23.670 1.00 24.39 H
ATOM 5502 HB1 ALA A 552 45.523 −15.498 −26.117 1.00 24.39 H
ATOM 5503 HB2 ALA A 552 43.760 −15.275 −26.026 1.00 24.39 H
ATOM 5504 HB3 ALA A 552 44.526 −16.654 −25.202 1.00 24.39 H
ATOM 5505 N GLU A 553 46.078 −12.879 −25.203 1.00 35.47 N
ATOM 5506 CA GLU A 553 46.497 −11.494 −25.452 1.00 35.58 C
ATOM 5507 C GLU A 553 45.467 −10.642 −26.232 1.00 35.70 C
ATOM 5508 O GLU A 553 44.558 −11.151 −26.893 1.00 35.71 O
ATOM 5509 CB GLU A 553 47.876 −11.500 −26.146 1.00 35.73 C
ATOM 5510 CG GLU A 553 49.066 −11.778 −25.203 1.00 35.68 C
ATOM 5511 CD GLU A 553 49.009 −13.103 −24.417 1.00 35.83 C
ATOM 5512 OE1 GLU A 553 48.527 −14.117 −24.974 1.00 35.88 O
ATOM 5513 OE2 GLU A 553 49.403 −13.083 −23.228 1.00 35.90 O
ATOM 5514 HN GLU A 553 46.786 −13.593 −25.380 1.00 0.00 H
ATOM 5515 HA GLU A 553 46.631 −11.021 −24.479 1.00 35.72 H
ATOM 5516 HB1 GLU A 553 47.866 −12.270 −26.917 1.00 35.72 H
ATOM 5517 HB2 GLU A 553 48.029 −10.525 −26.608 1.00 35.72 H

ATOM	5518	HG1	GLU	A	553	49.974	−11.786	−25.806	1.00	35.72	H
ATOM	5519	HG2	GLU	A	553	49.116	−10.963	−24.480	1.00	35.72	H
ATOM	5520	N	ASP	A	554	45.566	−9.315	−26.093	1.00	27.42	N
ATOM	5521	CA	ASP	A	554	44.496	−8.365	−26.440	1.00	27.55	C
ATOM	5522	C	ASP	A	554	44.093	−8.301	−27.930	1.00	27.68	C
ATOM	5523	O	ASP	A	554	42.989	−7.836	−28.225	1.00	27.74	O
ATOM	5524	CB	ASP	A	554	44.880	−6.958	−25.936	1.00	27.65	C
ATOM	5525	CG	ASP	A	554	44.638	−6.757	−24.434	1.00	27.56	C
ATOM	5526	OD1	ASP	A	554	43.468	−6.950	−24.023	1.00	27.40	O
ATOM	5527	OD2	ASP	A	554	45.489	−6.093	−23.804	1.00	27.67	O
ATOM	5528	HN	ASP	A	554	46.338	−8.953	−25.555	1.00	0.00	H
ATOM	5529	HA	ASP	A	554	43.609	−8.670	−25.884	1.00	27.58	H
ATOM	5530	HB1	ASP	A	554	45.939	−6.796	−26.139	1.00	27.58	H
ATOM	5531	HB2	ASP	A	554	44.288	−6.223	−26.482	1.00	27.58	H
ATOM	5532	N	ASP	A	555	44.924	−8.767	−28.865	1.00	27.90	N
ATOM	5533	CA	ASP	A	555	44.605	−8.863	−30.299	1.00	28.05	C
ATOM	5534	C	ASP	A	555	44.078	−10.250	−30.725	1.00	28.03	C
ATOM	5535	O	ASP	A	555	43.693	−10.434	−31.881	1.00	28.16	O
ATOM	5536	CB	ASP	A	555	45.842	−8.477	−31.135	1.00	28.16	C
ATOM	5537	CG	ASP	A	555	47.061	−9.401	−30.975	1.00	28.10	C
ATOM	5538	OD1	ASP	A	555	47.123	−10.148	−29.970	1.00	27.96	O
ATOM	5539	OD2	ASP	A	555	47.971	−9.284	−31.826	1.00	28.20	O
ATOM	5540	HN	ASP	A	555	45.802	−9.192	−28.581	1.00	0.00	H
ATOM	5541	HA	ASP	A	555	43.823	−8.134	−30.512	1.00	28.07	H
ATOM	5542	HB1	ASP	A	555	45.552	−8.482	−32.186	1.00	28.07	H
ATOM	5543	HB2	ASP	A	555	46.143	−7.470	−30.845	1.00	28.07	H
ATOM	5544	N	THR	A	556	44.065	−11.241	−29.827	1.00	25.17	N

ATOM	5545	CA	THR	A	556	43.853	−12.647	−30.208	1.00	25.16	C
ATOM	5546	C	THR	A	556	42.453	−12.929	−30.762	1.00	25.25	C
ATOM	5547	O	THR	A	556	41.425	−12.483	−30.239	1.00	25.21	O
ATOM	5548	CB	THR	A	556	44.199	−13.636	−29.086	1.00	24.98	C
ATOM	5549	OG1	THR	A	556	43.480	−13.376	−27.903	1.00	24.90	O
ATOM	5550	CG2	THR	A	556	45.696	−13.631	−28.785	1.00	24.89	C
ATOM	5551	HN	THR	A	556	44.424	−11.039	−28.901	1.00	0.00	H
ATOM	5552	HG1	THR	A	556	43.869	−12.569	−27.509	1.00	0.00	H
ATOM	5553	HA	THR	A	556	44.550	−12.851	−31.021	1.00	25.08	H
ATOM	5554	HB	THR	A	556	43.930	−14.636	−29.427	1.00	25.08	H
ATOM	5555	1HG2	THR	A	556	45.909	−14.341	−27.986	1.00	25.08	H
ATOM	5556	2HG2	THR	A	556	46.001	−12.632	−28.473	1.00	25.08	H
ATOM	5557	3HG2	THR	A	556	46.248	−13.916	−29.681	1.00	25.08	H
ATOM	5558	N	ASN	A	557	42.428	−13.715	−31.844	1.00	25.38	N
ATOM	5559	CA	ASN	A	557	41.236	−14.024	−32.630	1.00	25.47	C
ATOM	5560	C	ASN	A	557	40.294	−14.957	−31.851	1.00	25.41	C
ATOM	5561	O	ASN	A	557	40.629	−16.120	−31.625	1.00	25.38	O
ATOM	5562	CB	ASN	A	557	41.617	−14.676	−33.982	1.00	25.65	C
ATOM	5563	CG	ASN	A	557	42.867	−14.140	−34.657	1.00	25.72	C
ATOM	5564	OD1	ASN	A	557	42.810	−13.580	−35.745	1.00	25.68	O
ATOM	5565	ND2	ASN	A	557	44.016	−14.604	−34.222	1.00	25.86	N
ATOM	5566	HN	ASN	A	557	43.313	−14.023	−32.213	1.00	0.00	H
ATOM	5567	1HD2	ASN	A	557	44.017	−15.343	−33.524	1.00	0.00	H
ATOM	5568	2HD2	ASN	A	557	44.873	−14.262	−34.637	1.00	0.00	H
ATOM	5569	HA	ASN	A	557	40.707	−13.093	−32.834	1.00	25.57	H
ATOM	5570	HB1	ASN	A	557	41.765	−15.742	−33.807	1.00	25.57	H
ATOM	5571	HB2	ASN	A	557	40.782	−14.532	−34.667	1.00	25.57	H

ATOM 5572 N ALA A 558 39.043 −14.557 −31.629 1.00 25.42 N

ATOM 5573 CA ALA A 558 38.017 −15.404 −31.025 1.00 25.40 C

ATOM 5574 C ALA A 558 36.608 −15.069 −31.541 1.00 25.54 C

ATOM 5575 O ALA A 558 36.220 −13.909 −31.642 1.00 25.61 O

ATOM 5576 CB ALA A 558 38.141 −15.320 −29.498 1.00 25.23 C

ATOM 5577 HN ALA A 558 38.783 −13.618 −31.919 1.00 0.00 H

ATOM 5578 HA ALA A 558 38.233 −16.433 −31.313 1.00 25.44 H

ATOM 5579 HB1 ALA A 558 37.380 −15.949 −29.036 1.00 25.44 H

ATOM 5580 HB2 ALA A 558 38.002 −14.287 −29.178 1.00 25.44 H

ATOM 5581 HB3 ALA A 558 39.129 −15.664 −29.194 1.00 25.44 H

ATOM 5582 N GLN A 559 35.828 −16.103 −31.873 1.00 25.58 N

ATOM 5583 CA GLN A 559 34.526 −15.982 −32.543 1.00 25.73 C

ATOM 5584 C GLN A 559 33.333 −16.170 −31.593 1.00 25.74 C

ATOM 5585 O GLN A 559 32.337 −15.453 −31.701 1.00 25.69 O

ATOM 5586 CB GLN A 559 34.480 −16.985 −33.714 1.00 25.89 C

ATOM 5587 CG GLN A 559 33.157 −16.936 −34.499 1.00 26.05 C

ATOM 5588 CD GLN A 559 33.195 −17.799 −35.759 1.00 26.20 C

ATOM 5589 OE1 GLN A 559 33.313 −17.314 −36.873 1.00 26.18 O

ATOM 5590 NE2 GLN A 559 33.064 −19.101 −35.648 1.00 26.37 N

ATOM 5591 HN GLN A 559 36.214 −17.030 −31.724 1.00 0.00 H

ATOM 5592 1HE2 GLN A 559 33.000 −19.557 −34.731 1.00 0.00 H

ATOM 5593 2HE2 GLN A 559 33.138 −19.652 −36.479 1.00 0.00 H

ATOM 5594 HA GLN A 559 34.461 −14.978 −32.963 1.00 25.94 H

ATOM 5595 HB1 GLN A 559 35.298 −16.757 −34.398 1.00 25.94 H

ATOM 5596 HB2 GLN A 559 34.609 −17.991 −33.314 1.00 25.94 H

ATOM 5597 HG1 GLN A 559 32.354 −17.293 −33.854 1.00 25.94 H

ATOM 5598 HG2 GLN A 559 32.960 −15.904 −34.788 1.00 25.94 H

ATOM 5599 N LYS A 560 33.388 −17.173 −30.710 1.00 25.84 N

ATOM 5600 CA LYS A 560 32.240 −17.681 −29.938 1.00 25.93 C

ATOM 5601 C LYS A 560 32.682 −18.168 −28.564 1.00 25.81 C

ATOM 5602 O LYS A 560 33.741 −18.776 −28.438 1.00 25.84 O

ATOM 5603 CB LYS A 560 31.547 −18.790 −30.760 1.00 26.15 C

ATOM 5604 CG LYS A 560 30.540 −19.664 −29.994 1.00 26.30 C

ATOM 5605 CD LYS A 560 29.769 −20.596 −30.941 1.00 26.56 C

ATOM 5606 CE LYS A 560 29.078 −21.752 −30.200 1.00 26.73 C

ATOM 5607 NZ LYS A 560 28.161 −21.295 −29.125 1.00 26.76 N1+

ATOM 5608 HN LYS A 560 34.251 −17.700 −30.663 1.00 0.00 H

ATOM 5609 HZ1 LYS A 560 27.730 −22.091 −28.676 1.00 0.00 H

ATOM 5610 HZ2 LYS A 560 28.666 −20.774 −28.414 1.00 0.00 H

ATOM 5611 HZ3 LYS A 560 27.435 −20.693 −29.488 1.00 0.00 H

ATOM 5612 HA LYS A 560 31.532 −16.864 −29.800 1.00 26.21 H

ATOM 5613 HB1 LYS A 560 31.016 −18.313 −31.584 1.00 26.21 H

ATOM 5614 HB2 LYS A 560 32.322 −19.445 −31.158 1.00 26.21 H

ATOM 5615 HG1 LYS A 560 31.079 −20.268 −29.264 1.00 26.21 H

ATOM 5616 HG2 LYS A 560 29.830 −19.017 −29.478 1.00 26.21 H

ATOM 5617 HD1 LYS A 560 29.010 −20.013 −31.463 1.00 26.21 H

ATOM 5618 HD2 LYS A 560 30.468 −21.014 −31.665 1.00 26.21 H

ATOM 5619 HE1 LYS A 560 28.503 −22.331 −30.922 1.00 26.21 H

ATOM 5620 HE2 LYS A 560 29.845 −22.385 −29.754 1.00 26.21 H

ATOM 5621 N THR A 561 31.786 −18.091 −27.583 1.00 25.67 N

ATOM 5622 CA THR A 561 32.006 −18.621 −26.226 1.00 25.51 C

ATOM 5623 C THR A 561 30.860 −19.538 −25.787 1.00 25.62 C

ATOM 5624 O THR A 561 29.787 −19.525 −26.393 1.00 25.77 O

ATOM 5625 CB THR A 561 32.270 −17.490 −25.211 1.00 25.34 C

ATOM	5626	OG1	THR	A	561	31.074	−16.897	−24.764	1.00	25.43	O
ATOM	5627	CG2	THR	A	561	33.141	−16.368	−25.789	1.00	25.28	C
ATOM	5628	HN	THR	A	561	30.932	−17.574	−27.755	1.00	0.00	H
ATOM	5629	HG1	THR	A	561	31.319	−16.152	−24.181	1.00	0.00	H
ATOM	5630	HA	THR	A	561	32.907	−19.233	−26.264	1.00	25.52	H
ATOM	5631	HB	THR	A	561	32.785	−17.915	−24.350	1.00	25.52	H
ATOM	5632	1HG2	THR	A	561	33.294	−15.599	−25.031	1.00	25.52	H
ATOM	5633	2HG2	THR	A	561	32.643	−15.930	−26.654	1.00	25.52	H
ATOM	5634	3HG2	THR	A	561	34.105	−16.776	−26.093	1.00	25.52	H
ATOM	5635	N	ILE	A	562	31.105	−20.429	−24.824	1.00	35.26	N
ATOM	5636	CA	ILE	A	562	30.079	−21.182	−24.076	1.00	35.34	C
ATOM	5637	C	ILE	A	562	30.595	−21.341	−22.644	1.00	35.14	C
ATOM	5638	O	ILE	A	562	31.689	−21.875	−22.481	1.00	34.97	O
ATOM	5639	CB	ILE	A	562	29.778	−22.581	−24.682	1.00	35.57	C
ATOM	5640	CG1	ILE	A	562	29.423	−22.537	−26.186	1.00	35.76	C
ATOM	5641	CG2	ILE	A	562	28.645	−23.257	−23.883	1.00	35.71	C
ATOM	5642	CD1	ILE	A	562	29.248	−23.908	−26.846	1.00	36.03	C
ATOM	5643	HN	ILE	A	562	32.060	−20.505	−24.483	1.00	0.00	H
ATOM	5644	HA	ILE	A	562	29.158	−20.600	−24.057	1.00	35.47	H
ATOM	5645	HB	ILE	A	562	30.674	−23.192	−24.570	1.00	35.47	H
ATOM	5646	1HG1	ILE	A	562	28.489	−21.986	−26.298	1.00	35.47	H
ATOM	5647	2HG1	ILE	A	562	30.222	−22.010	−26.707	1.00	35.47	H
ATOM	5648	1HG2	ILE	A	562	28.435	−24.238	−24.309	1.00	35.47	H
ATOM	5649	2HG2	ILE	A	562	27.747	−22.640	−23.932	1.00	35.47	H
ATOM	5650	3HG2	ILE	A	562	28.951	−23.371	−22.843	1.00	35.47	H
ATOM	5651	1HD1	ILE	A	562	29.001	−23.776	−27.899	1.00	35.47	H
ATOM	5652	2HD1	ILE	A	562	28.443	−24.451	−26.350	1.00	35.47	H

ATOM 5653 3HD1 ILE A 562 30.175 −24.475 −26.759 1.00 35.47 H

ATOM 5654 N THR A 563 29.848 −20.909 −21.625 1.00 25.46 N

ATOM 5655 CA THR A 563 30.239 −21.043 −20.209 1.00 25.29 C

ATOM 5656 C THR A 563 29.170 −21.768 −19.396 1.00 25.43 C

ATOM 5657 O THR A 563 28.071 −21.240 −19.220 1.00 25.61 O

ATOM 5658 CB THR A 563 30.554 −19.680 −19.570 1.00 25.10 C

ATOM 5659 OG1 THR A 563 31.510 −18.986 −20.351 1.00 24.97 O

ATOM 5660 CG2 THR A 563 31.101 −19.840 −18.148 1.00 24.92 C

ATOM 5661 HN THR A 563 28.953 −20.464 −21.827 1.00 0.00 H

ATOM 5662 HG1 THR A 563 31.009 −18.688 −21.123 1.00 0.00 H

ATOM 5663 HA THR A 563 31.148 −21.644 −20.172 1.00 25.25 H

ATOM 5664 HB THR A 563 29.638 −19.090 −19.530 1.00 25.25 H

ATOM 5665 1HG2 THR A 563 31.313 −18.857 −17.727 1.00 25.25 H

ATOM 5666 2HG2 THR A 563 32.018 −20.428 −18.175 1.00 25.25 H

ATOM 5667 3HG2 THR A 563 30.362 −20.348 −17.529 1.00 25.25 H

ATOM 5668 N ASP A 564 29.554 −22.875 −18.757 1.00 25.36 N

ATOM 5669 CA ASP A 564 28.732 −23.626 −17.797 1.00 25.49 C

ATOM 5670 C ASP A 564 29.445 −23.727 −16.432 1.00 25.26 C

ATOM 5671 O ASP A 564 30.665 −23.899 −16.383 1.00 25.16 O

ATOM 5672 CB ASP A 564 28.341 −25.012 −18.352 1.00 25.74 C

ATOM 5673 CG ASP A 564 27.499 −25.017 −19.644 1.00 25.95 C

ATOM 5674 OD1 ASP A 564 26.931 −23.966 −20.022 1.00 26.05 O

ATOM 5675 OD2 ASP A 564 27.259 −26.122 −20.172 1.00 26.04 O

ATOM 5676 HN ASP A 564 30.515 −23.185 −18.872 1.00 0.00 H

ATOM 5677 HA ASP A 564 27.811 −23.063 −17.644 1.00 25.63 H

ATOM 5678 HB1 ASP A 564 29.261 −25.561 −18.553 1.00 25.63 H

ATOM 5679 HB2 ASP A 564 27.770 −25.529 −17.581 1.00 25.63 H

ATOM 5680 N CYS A 565 28.711 −23.600 −15.317 1.00 25.19 N

ATOM 5681 CA CYS A 565 29.288 −23.544 −13.963 1.00 24.91 C

ATOM 5682 C CYS A 565 28.628 −24.526 −12.979 1.00 24.97 C

ATOM 5683 O CYS A 565 27.448 −24.856 −13.087 1.00 25.23 O

ATOM 5684 CB CYS A 565 29.248 −22.110 −13.412 1.00 24.75 C

ATOM 5685 SG CYS A 565 29.929 −20.789 −14.458 1.00 24.74 S

ATOM 5686 HN CYS A 565 27.710 −23.484 −15.398 1.00 0.00 H

ATOM 5687 HA CYS A 565 30.337 −23.828 −14.047 1.00 24.97 H

ATOM 5688 HB1 CYS A 565 28.204 −21.865 −13.215 1.00 24.97 H

ATOM 5689 HB2 CYS A 565 29.806 −22.105 −12.475 1.00 24.97 H

ATOM 5690 N PHE A 566 29.408 −24.998 −12.005 1.00 24.71 N

ATOM 5691 CA PHE A 566 29.070 −26.101 −11.101 1.00 24.74 C

ATOM 5692 C PHE A 566 29.947 −26.077 −9.832 1.00 24.42 C

ATOM 5693 O PHE A 566 30.757 −25.170 −9.634 1.00 24.18 O

ATOM 5694 CB PHE A 566 29.207 −27.430 −11.875 1.00 24.88 C

ATOM 5695 CG PHE A 566 30.536 −27.636 −12.581 1.00 24.74 C

ATOM 5696 CD1 PHE A 566 31.601 −28.282 −11.925 1.00 24.45 C

ATOM 5697 CD2 PHE A 566 30.700 −27.194 −13.909 1.00 24.89 C

ATOM 5698 CE1 PHE A 566 32.823 −28.480 −12.591 1.00 24.33 C

ATOM 5699 CE2 PHE A 566 31.923 −27.385 −14.572 1.00 24.78 C

ATOM 5700 CZ PHE A 566 32.984 −28.030 −13.914 1.00 24.51 C

ATOM 5701 HN PHE A 566 30.362 −24.648 −11.955 1.00 0.00 H

ATOM 5702 HA PHE A 566 28.029 −25.988 −10.799 1.00 24.60 H

ATOM 5703 HB1 PHE A 566 29.072 −28.247 −11.167 1.00 24.60 H

ATOM 5704 HB2 PHE A 566 28.418 −27.465 −12.626 1.00 24.60 H

ATOM 5705 HD1 PHE A 566 31.478 −28.629 −10.899 1.00 24.60 H

ATOM 5706 HD2 PHE A 566 29.874 −26.702 −14.423 1.00 24.60 H

```
ATOM   5707  HE1 PHE A 566      33.646 −28.982 −12.082  1.00 24.60           H
ATOM   5708  HE2 PHE A 566      32.049 −27.033 −15.596  1.00 24.60           H
ATOM   5709  HZ  PHE A 566      33.933 −28.182 −14.429  1.00 24.60           H
ATOM   5710  N   LEU A 567      29.728 −27.028  −8.919  1.00 24.42           N
ATOM   5711  CA  LEU A 567      30.560 −27.230  −7.728  1.00 24.10           C
ATOM   5712  C   LEU A 567      31.562 −28.372  −7.941  1.00 24.02           C
ATOM   5713  O   LEU A 567      31.174 −29.437  −8.414  1.00 24.26           O
ATOM   5714  CB  LEU A 567      29.673 −27.522  −6.502  1.00 24.11           C
ATOM   5715  CG  LEU A 567      28.669 −26.417  −6.127  1.00 23.97           C
ATOM   5716  CD1 LEU A 567      27.853 −26.858  −4.912  1.00 24.06           C
ATOM   5717  CD2 LEU A 567      29.362 −25.101  −5.780  1.00 23.59           C
ATOM   5718  HN  LEU A 567      29.099 −27.778  −9.164  1.00  0.00           H
ATOM   5719  HA  LEU A 567      31.118 −26.313  −7.538  1.00 24.07           H
ATOM   5720  HB1 LEU A 567      29.109 −28.432  −6.706  1.00 24.07           H
ATOM   5721  HB2 LEU A 567      30.328 −27.684  −5.646  1.00 24.07           H
ATOM   5722  HG  LEU A 567      27.994 −26.252  −6.967  1.00 24.07           H
ATOM   5723 1HD1 LEU A 567      27.142 −26.075  −4.647  1.00 24.07           H
ATOM   5724 2HD1 LEU A 567      28.522 −27.040  −4.071  1.00 24.07           H
ATOM   5725 3HD1 LEU A 567      27.312 −27.774  −5.150  1.00 24.07           H
ATOM   5726 1HD2 LEU A 567      28.613 −24.352  −5.522  1.00 24.07           H
ATOM   5727 2HD2 LEU A 567      29.939 −24.756  −6.638  1.00 24.07           H
ATOM   5728 3HD2 LEU A 567      30.029 −25.254  −4.932  1.00 24.07           H
ATOM   5729  N   LEU A 568      32.764 −28.232  −7.381  1.00 37.61           N
ATOM   5730  CA  LEU A 568      33.765 −29.298  −7.254  1.00 37.49           C
ATOM   5731  C   LEU A 568      34.573 −29.096  −5.958  1.00 37.18           C
ATOM   5732  O   LEU A 568      34.895 −27.959  −5.620  1.00 36.95           O
ATOM   5733  CB  LEU A 568      34.638 −29.307  −8.520  1.00 37.38           C
```

ATOM	5734	CG	LEU	A	568	35.660	−30.462	−8.538	1.00	37.30	C
ATOM	5735	CD1	LEU	A	568	35.408	−31.381	−9.734	1.00	37.65	C
ATOM	5736	CD2	LEU	A	568	37.081	−29.901	−8.594	1.00	37.12	C
ATOM	5737	HN	LEU	A	568	33.019	−27.317	−7.019	1.00	0.00	H
ATOM	5738	HA	LEU	A	568	33.244	−30.253	−7.189	1.00	37.34	H
ATOM	5739	HB1	LEU	A	568	33.988	−29.405	−9.390	1.00	37.34	H
ATOM	5740	HB2	LEU	A	568	35.180	−28.363	−8.575	1.00	37.34	H
ATOM	5741	HG	LEU	A	568	35.549	−31.042	−7.622	1.00	37.33	H
ATOM	5742	1HD1	LEU	A	568	36.138	−32.190	−9.731	1.00	37.33	H
ATOM	5743	2HD1	LEU	A	568	35.503	−30.810	−10.658	1.00	37.33	H
ATOM	5744	3HD1	LEU	A	568	34.403	−31.798	−9.667	1.00	37.33	H
ATOM	5745	1HD2	LEU	A	568	37.797	−30.723	−8.606	1.00	37.34	H
ATOM	5746	2HD2	LEU	A	568	37.261	−29.277	−7.719	1.00	37.34	H
ATOM	5747	3HD2	LEU	A	568	37.201	−29.302	−9.497	1.00	37.34	H
ATOM	5748	N	GLU	A	569	34.601	−30.117	−5.091	1.00	54.42	N
ATOM	5749	CA	GLU	A	569	34.884	−29.982	−3.643	1.00	54.12	C
ATOM	5750	C	GLU	A	569	34.048	−28.886	−2.936	1.00	53.97	C
ATOM	5751	O	GLU	A	569	34.477	−28.274	−1.959	1.00	53.64	O
ATOM	5752	CB	GLU	A	569	36.395	−29.860	−3.355	1.00	53.83	C
ATOM	5753	CG	GLU	A	569	37.145	−31.197	−3.465	1.00	53.98	C
ATOM	5754	CD	GLU	A	569	38.189	−31.377	−2.348	1.00	53.70	C
ATOM	5755	OE1	GLU	A	569	38.820	−30.375	−1.927	1.00	53.38	O
ATOM	5756	OE2	GLU	A	569	38.680	−32.515	−2.173	1.00	53.81	O
ATOM	5757	HN	GLU	A	569	34.370	−31.036	−5.440	1.00	0.00	H
ATOM	5758	HA	GLU	A	569	34.574	−30.925	−3.193	1.00	53.87	H
ATOM	5759	HB1	GLU	A	569	36.828	−29.161	−4.070	1.00	53.87	H
ATOM	5760	HB2	GLU	A	569	36.524	−29.473	−2.344	1.00	53.87	H

ATOM 5761 HG1 GLU A 569 36.422 −32.010 −3.403 1.00 53.87 H

ATOM 5762 HG2 GLU A 569 37.653 −31.235 −4.429 1.00 53.87 H

ATOM 5763 N ASN A 570 32.802 −28.675 −3.380 1.00 45.28 N

ATOM 5764 CA ASN A 570 31.907 −27.593 −2.925 1.00 45.21 C

ATOM 5765 C ASN A 570 32.383 −26.165 −3.288 1.00 45.11 C

ATOM 5766 O ASN A 570 31.709 −25.190 −2.958 1.00 45.02 O

ATOM 5767 CB ASN A 570 31.563 −27.725 −1.424 1.00 44.92 C

ATOM 5768 CG ASN A 570 31.368 −29.150 −0.942 1.00 44.92 C

ATOM 5769 OD1 ASN A 570 30.305 −29.729 −1.059 1.00 45.19 O

ATOM 5770 ND2 ASN A 570 32.399 −29.763 −0.407 1.00 44.61 N

ATOM 5771 HN ASN A 570 32.498 −29.235 −4.161 1.00 0.00 H

ATOM 5772 1HD2 ASN A 570 33.303 −29.311 −0.454 1.00 0.00 H

ATOM 5773 2HD2 ASN A 570 32.270 −30.719 −0.134 1.00 0.00 H

ATOM 5774 HA ASN A 570 30.968 −27.739 −3.459 1.00 45.03 H

ATOM 5775 HB1 ASN A 570 32.375 −27.280 −0.849 1.00 45.03 H

ATOM 5776 HB2 ASN A 570 30.641 −27.174 −1.237 1.00 45.03 H

ATOM 5777 N VAL A 571 33.491 −26.020 −4.022 1.00 22.99 N

ATOM 5778 CA VAL A 571 33.969 −24.747 −4.578 1.00 22.91 C

ATOM 5779 C VAL A 571 33.324 −24.503 −5.943 1.00 23.18 C

ATOM 5780 O VAL A 571 33.290 −25.400 −6.783 1.00 23.36 O

ATOM 5781 CB VAL A 571 35.508 −24.732 −4.680 1.00 22.60 C

ATOM 5782 CG1 VAL A 571 36.033 −23.389 −5.208 1.00 22.53 C

ATOM 5783 CG2 VAL A 571 36.159 −24.977 −3.311 1.00 22.33 C

ATOM 5784 HN VAL A 571 33.989 −26.857 −4.310 1.00 0.00 H

ATOM 5785 HA VAL A 571 33.663 −23.944 −3.907 1.00 22.84 H

ATOM 5786 HB VAL A 571 35.821 −25.522 −5.362 1.00 22.84 H

ATOM 5787 1HG1 VAL A 571 37.121 −23.421 −5.265 1.00 22.84 H

ATOM 5788 2HG1 VAL A 571 35.728 −22.589 −4.534 1.00 22.84 H

ATOM 5789 3HG1 VAL A 571 35.623 −23.203 −6.201 1.00 22.84 H

ATOM 5790 1HG2 VAL A 571 37.244 −24.961 −3.416 1.00 22.84 H

ATOM 5791 2HG2 VAL A 571 35.846 −25.948 −2.928 1.00 22.84 H

ATOM 5792 3HG2 VAL A 571 35.850 −24.196 −2.616 1.00 22.84 H

ATOM 5793 N ILE A 572 32.876 −23.270 −6.200 1.00 36.75 N

ATOM 5794 CA ILE A 572 32.316 −22.881 −7.499 1.00 37.02 C

ATOM 5795 C ILE A 572 33.428 −22.871 −8.564 1.00 36.99 C

ATOM 5796 O ILE A 572 34.371 −22.074 −8.495 1.00 36.81 O

ATOM 5797 CB ILE A 572 31.559 −21.530 −7.438 1.00 37.10 C

ATOM 5798 CG1 ILE A 572 30.408 −21.464 −6.404 1.00 37.10 C

ATOM 5799 CG2 ILE A 572 30.949 −21.216 −8.819 1.00 37.41 C

ATOM 5800 CD1 ILE A 572 30.844 −21.176 −4.960 1.00 36.79 C

ATOM 5801 HN ILE A 572 32.924 −22.578 −5.473 1.00 0.00 H

ATOM 5802 HA ILE A 572 31.595 −23.647 −7.786 1.00 37.00 H

ATOM 5803 HB ILE A 572 32.278 −20.747 −7.197 1.00 37.00 H

ATOM 5804 1HG1 ILE A 572 29.722 −20.675 −6.713 1.00 37.00 H

ATOM 5805 2HG1 ILE A 572 29.889 −22.423 −6.415 1.00 37.00 H

ATOM 5806 1HG2 ILE A 572 30.417 −20.266 −8.774 1.00 37.00 H

ATOM 5807 2HG2 ILE A 572 30.254 −22.008 −9.098 1.00 37.00 H

ATOM 5808 3HG2 ILE A 572 31.744 −21.152 −9.562 1.00 37.00 H

ATOM 5809 1HD1 ILE A 572 29.967 −21.150 −4.313 1.00 37.00 H

ATOM 5810 2HD1 ILE A 572 31.354 −20.213 −4.919 1.00 37.00 H

ATOM 5811 3HD1 ILE A 572 31.521 −21.960 −4.621 1.00 37.00 H

ATOM 5812 N TRP A 573 33.221 −23.646 −9.624 1.00 23.66 N

ATOM 5813 CA TRP A 573 34.004 −23.652 −10.859 1.00 23.67 C

ATOM 5814 C TRP A 573 33.111 −23.305 −12.051 1.00 23.94 C

ATOM	5815	O	TRP	A	573	31.931	−23.647	−12.076	1.00	24.17	O
ATOM	5816	CB	TRP	A	573	34.646	−25.033	−11.057	1.00	23.66	C
ATOM	5817	CG	TRP	A	573	35.935	−25.278	−10.333	1.00	23.37	C
ATOM	5818	CD1	TRP	A	573	36.061	−25.804	−9.094	1.00	23.16	C
ATOM	5819	CD2	TRP	A	573	37.299	−25.058	−10.811	1.00	23.23	C
ATOM	5820	NE1	TRP	A	573	37.400	−25.981	−8.797	1.00	22.91	N
ATOM	5821	CE2	TRP	A	573	38.209	−25.521	−9.814	1.00	22.95	C
ATOM	5822	CE3	TRP	A	573	37.861	−24.560	−12.008	1.00	23.33	C
ATOM	5823	CZ2	TRP	A	573	39.600	−25.486	−9.992	1.00	22.78	C
ATOM	5824	CZ3	TRP	A	573	39.259	−24.502	−12.189	1.00	23.17	C
ATOM	5825	CH2	TRP	A	573	40.127	−24.969	−11.188	1.00	22.90	C
ATOM	5826	HN	TRP	A	573	32.381	−24.220	−9.628	1.00	0.00	H
ATOM	5827	HE1	TRP	A	573	37.723	−26.535	−8.013	1.00	0.00	H
ATOM	5828	HA	TRP	A	573	34.793	−22.904	−10.780	1.00	23.35	H
ATOM	5829	HB1	TRP	A	573	33.930	−25.783	−10.722	1.00	23.35	H
ATOM	5830	HB2	TRP	A	573	34.835	−25.161	−12.123	1.00	23.35	H
ATOM	5831	HD1	TRP	A	573	35.233	−26.051	−8.430	1.00	23.35	H
ATOM	5832	HE3	TRP	A	573	37.203	−24.215	−12.805	1.00	23.35	H
ATOM	5833	HZ2	TRP	A	573	40.265	−25.856	−9.211	1.00	23.35	H
ATOM	5834	HZ3	TRP	A	573	39.669	−24.092	−13.112	1.00	23.35	H
ATOM	5835	HH2	TRP	A	573	41.206	−24.931	−11.338	1.00	23.35	H
ATOM	5836	N	CYS	A	574	33.726	−22.774	−13.102	1.00	23.94	N
ATOM	5837	CA	CYS	A	574	33.159	−22.695	−14.442	1.00	24.19	C
ATOM	5838	C	CYS	A	574	34.091	−23.385	−15.442	1.00	24.21	C
ATOM	5839	O	CYS	A	574	35.315	−23.285	−15.323	1.00	24.06	O
ATOM	5840	CB	CYS	A	574	32.919	−21.229	−14.810	1.00	24.24	C
ATOM	5841	SG	CYS	A	574	31.775	−20.338	−13.723	1.00	24.36	S

ATOM 5842 HN CYS A 574 34.693 −22.492 −13.002 1.00 0.00 H

ATOM 5843 HA CYS A 574 32.201 −23.215 −14.445 1.00 24.17 H

ATOM 5844 HB1 CYS A 574 33.879 −20.713 −14.784 1.00 24.17 H

ATOM 5845 HB2 CYS A 574 32.516 −21.197 −15.822 1.00 24.17 H

ATOM 5846 N ILE A 575 33.514 −23.989 −16.478 1.00 24.44 N

ATOM 5847 CA ILE A 575 34.205 −24.331 −17.721 1.00 24.55 C

ATOM 5848 C ILE A 575 33.719 −23.359 −18.793 1.00 24.67 C

ATOM 5849 O ILE A 575 32.521 −23.297 −19.075 1.00 24.76 O

ATOM 5850 CB ILE A 575 33.947 −25.805 −18.112 1.00 24.79 C

ATOM 5851 CG1 ILE A 575 34.562 −26.794 −17.094 1.00 24.69 C

ATOM 5852 CG2 ILE A 575 34.447 −26.109 −19.538 1.00 25.03 C

ATOM 5853 CD1 ILE A 575 36.087 −26.936 −17.155 1.00 24.50 C

ATOM 5854 HN ILE A 575 32.496 −23.990 −16.515 1.00 0.00 H

ATOM 5855 HA ILE A 575 35.276 −24.188 −17.578 1.00 24.68 H

ATOM 5856 HB ILE A 575 32.868 −25.960 −18.105 1.00 24.68 H

ATOM 5857 1HG1 ILE A 575 34.296 −26.455 −16.093 1.00 24.68 H

ATOM 5858 2HG1 ILE A 575 34.125 −27.776 −17.274 1.00 24.68 H

ATOM 5859 1HG2 ILE A 575 34.249 −27.154 −19.778 1.00 24.68 H

ATOM 5860 2HG2 ILE A 575 35.519 −25.920 −19.596 1.00 24.68 H

ATOM 5861 3HG2 ILE A 575 33.927 −25.468 −20.250 1.00 24.68 H

ATOM 5862 1HD1 ILE A 575 36.416 −27.652 −16.402 1.00 24.68 H

ATOM 5863 2HD1 ILE A 575 36.550 −25.968 −16.963 1.00 24.68 H

ATOM 5864 3HD1 ILE A 575 36.380 −27.289 −18.144 1.00 24.68 H

ATOM 5865 N SER A 576 34.655 −22.674 −19.445 1.00 24.68 N

ATOM 5866 CA SER A 576 34.400 −21.909 −20.662 1.00 24.85 C

ATOM 5867 C SER A 576 35.076 −22.576 −21.855 1.00 25.00 C

ATOM 5868 O SER A 576 36.304 −22.649 −21.925 1.00 24.96 O

ATOM 5869 CB SER A 576 34.860 −20.458 −20.526 1.00 24.71 C
ATOM 5870 OG SER A 576 34.121 −19.796 −19.517 1.00 24.50 O
ATOM 5871 HN SER A 576 35.622 −22.790 −19.160 1.00 0.00 H
ATOM 5872 HG SER A 576 33.221 −19.612 −19.845 1.00 0.00 H
ATOM 5873 HA SER A 576 33.325 −21.907 −20.841 1.00 24.78 H
ATOM 5874 HB1 SER A 576 35.918 −20.440 −20.265 1.00 24.78 H
ATOM 5875 HB2 SER A 576 34.711 −19.944 −21.476 1.00 24.78 H
ATOM 5876 N LEU A 577 34.289 −22.959 −22.857 1.00 25.18 N
ATOM 5877 CA LEU A 577 34.793 −23.130 −24.215 1.00 25.31 C
ATOM 5878 C LEU A 577 34.934 −21.749 −24.864 1.00 25.22 C
ATOM 5879 O LEU A 577 33.976 −20.972 −24.865 1.00 25.02 O
ATOM 5880 CB LEU A 577 33.834 −24.034 −25.012 1.00 25.60 C
ATOM 5881 CG LEU A 577 34.391 −24.484 −26.375 1.00 25.77 C
ATOM 5882 CD1 LEU A 577 35.487 −25.538 −26.207 1.00 25.78 C
ATOM 5883 CD2 LEU A 577 33.282 −25.103 −27.221 1.00 26.05 C
ATOM 5884 HN LEU A 577 33.295 −22.780 −22.749 1.00 0.00 H
ATOM 5885 HA LEU A 577 35.774 −23.604 −24.172 1.00 25.49 H
ATOM 5886 HB1 LEU A 577 33.625 −24.923 −24.416 1.00 25.49 H
ATOM 5887 HB2 LEU A 577 32.908 −23.485 −25.184 1.00 25.49 H
ATOM 5888 HG LEU A 577 34.801 −23.620 −26.898 1.00 25.49 H
ATOM 5889 1HD1 LEU A 577 35.860 −25.835 −27.187 1.00 25.49 H
ATOM 5890 2HD1 LEU A 577 35.078 −26.409 −25.695 1.00 25.49 H
ATOM 5891 3HD1 LEU A 577 36.305 −25.122 −25.619 1.00 25.49 H
ATOM 5892 1HD2 LEU A 577 33.690 −25.417 −28.182 1.00 25.49 H
ATOM 5893 2HD2 LEU A 577 32.495 −24.367 −27.384 1.00 25.49 H
ATOM 5894 3HD2 LEU A 577 32.869 −25.968 −26.702 1.00 25.49 H
ATOM 5895 N VAL A 578 36.043 −21.521 −25.564 1.00 25.36 N

```
ATOM   5896  CA  VAL A 578      36.190 −20.403 −26.506  1.00 25.33           C
ATOM   5897  C   VAL A 578      36.578 −20.959 −27.872  1.00 25.51           C
ATOM   5898  O   VAL A 578      37.483 −21.783 −27.970  1.00 25.62           O
ATOM   5899  CB  VAL A 578      37.206 −19.351 −26.013  1.00 25.10           C
ATOM   5900  CG1 VAL A 578      37.117 −18.073 −26.856  1.00 25.13           C
ATOM   5901  CG2 VAL A 578      36.976 −18.946 −24.551  1.00 24.92           C
ATOM   5902  HN  VAL A 578      36.812 −22.183 −25.504  1.00  0.00           H
ATOM   5903  HA  VAL A 578      35.220 −19.915 −26.604  1.00 25.28           H
ATOM   5904  HB  VAL A 578      38.210 −19.763 −26.108  1.00 25.28           H
ATOM   5905 1HG1 VAL A 578      37.842 −17.345 −26.491  1.00 25.28           H
ATOM   5906 2HG1 VAL A 578      36.113 −17.655 −26.779  1.00 25.28           H
ATOM   5907 3HG1 VAL A 578      37.333 −18.309 −27.898  1.00 25.28           H
ATOM   5908 1HG2 VAL A 578      37.719 −18.204 −24.258  1.00 25.28           H
ATOM   5909 2HG2 VAL A 578      37.068 −19.824 −23.911  1.00 25.28           H
ATOM   5910 3HG2 VAL A 578      35.978 −18.522 −24.444  1.00 25.28           H
ATOM   5911  N   GLU A 579      35.851 −20.579 −28.918  1.00 25.56           N
ATOM   5912  CA  GLU A 579      36.264 −20.747 −30.312  1.00 25.69           C
ATOM   5913  C   GLU A 579      37.272 −19.642 −30.656  1.00 25.57           C
ATOM   5914  O   GLU A 579      36.887 −18.483 −30.800  1.00 25.53           O
ATOM   5915  CB  GLU A 579      35.017 −20.720 −31.210  1.00 25.90           C
ATOM   5916  CG  GLU A 579      35.308 −21.117 −32.666  1.00 26.11           C
ATOM   5917  CD  GLU A 579      34.017 −21.277 −33.487  1.00 26.31           C
ATOM   5918  OE1 GLU A 579      33.056 −20.506 −33.261  1.00 26.33           O
ATOM   5919  OE2 GLU A 579      33.985 −22.125 −34.408  1.00 26.46           O
ATOM   5920  HN  GLU A 579      35.124 −19.892 −28.746  1.00  0.00           H
ATOM   5921  HA  GLU A 579      36.753 −21.715 −30.419  1.00 25.94           H
ATOM   5922  HB1 GLU A 579      34.282 −21.414 −30.802  1.00 25.94           H
```

ATOM 5923 HB2 GLU A 579 34.607 −19.710 −31.202 1.00 25.94 H
ATOM 5924 HG1 GLU A 579 35.924 −20.344 −33.125 1.00 25.94 H
ATOM 5925 HG2 GLU A 579 35.848 −22.064 −32.672 1.00 25.94 H
ATOM 5926 N ILE A 580 38.559 −19.974 −30.550 1.00 25.51 N
ATOM 5927 CA ILE A 580 39.715 −19.063 −30.510 1.00 25.37 C
ATOM 5928 C ILE A 580 40.825 −19.616 −31.408 1.00 25.48 C
ATOM 5929 O ILE A 580 40.955 −20.835 −31.532 1.00 25.59 O
ATOM 5930 CB ILE A 580 40.192 −18.856 −29.043 1.00 25.15 C
ATOM 5931 CG1 ILE A 580 41.369 −17.861 −28.894 1.00 25.01 C
ATOM 5932 CG2 ILE A 580 40.575 −20.189 −28.380 1.00 25.13 C
ATOM 5933 CD1 ILE A 580 41.759 −17.543 −27.444 1.00 24.80 C
ATOM 5934 HN ILE A 580 38.792 −20.946 −30.416 1.00 0.00 H
ATOM 5935 HA ILE A 580 39.403 −18.097 −30.908 1.00 25.26 H
ATOM 5936 HB ILE A 580 39.350 −18.447 −28.484 1.00 25.26 H
ATOM 5937 1HG1 ILE A 580 42.239 −18.287 −29.394 1.00 25.26 H
ATOM 5938 2HG1 ILE A 580 41.089 −16.928 −29.383 1.00 25.26 H
ATOM 5939 1HG2 ILE A 580 40.903 −20.005 −27.357 1.00 25.26 H
ATOM 5940 2HG2 ILE A 580 41.384 −20.656 −28.942 1.00 25.26 H
ATOM 5941 3HG2 ILE A 580 39.710 −20.852 −28.370 1.00 25.26 H
ATOM 5942 1HD1 ILE A 580 42.591 −16.839 −27.437 1.00 25.26 H
ATOM 5943 2HD1 ILE A 580 42.057 −18.461 −26.938 1.00 25.26 H
ATOM 5944 3HD1 ILE A 580 40.907 −17.103 −26.926 1.00 25.26 H
ATOM 5945 N TYR A 581 41.666 −18.748 −31.962 1.00 25.49 N
ATOM 5946 CA TYR A 581 42.958 −19.143 −32.510 1.00 25.58 C
ATOM 5947 C TYR A 581 44.013 −18.052 −32.336 1.00 25.54 C
ATOM 5948 O TYR A 581 43.752 −16.851 −32.447 1.00 25.48 O
ATOM 5949 CB TYR A 581 42.828 −19.608 −33.962 1.00 25.82 C

ATOM 5950 CG TYR A 581 42.440 −18.546 −34.973 1.00 25.94 C
ATOM 5951 CD1 TYR A 581 43.449 −17.841 −35.653 1.00 26.01 C
ATOM 5952 CD2 TYR A 581 41.085 −18.255 −35.222 1.00 25.99 C
ATOM 5953 CE1 TYR A 581 43.110 −16.807 −36.545 1.00 26.13 C
ATOM 5954 CE2 TYR A 581 40.739 −17.229 −36.126 1.00 26.10 C
ATOM 5955 CZ TYR A 581 41.754 −16.488 −36.769 1.00 26.16 C
ATOM 5956 OH TYR A 581 41.434 −15.394 −37.503 1.00 26.27 O
ATOM 5957 HN TYR A 581 41.513 −17.751 −31.821 1.00 0.00 H
ATOM 5958 HH TYR A 581 41.964 −14.639 −37.196 1.00 0.00 H
ATOM 5959 HA TYR A 581 43.296 −20.003 −31.932 1.00 25.88 H
ATOM 5960 HB1 TYR A 581 43.790 −20.021 −34.266 1.00 25.88 H
ATOM 5961 HB2 TYR A 581 42.070 −20.390 −33.994 1.00 25.88 H
ATOM 5962 HD1 TYR A 581 44.496 −18.096 −35.489 1.00 25.88 H
ATOM 5963 HD2 TYR A 581 40.304 −18.822 −34.716 1.00 25.88 H
ATOM 5964 HE1 TYR A 581 43.894 −16.253 −37.062 1.00 25.88 H
ATOM 5965 HE2 TYR A 581 39.691 −17.009 −36.328 1.00 25.88 H
ATOM 5966 N ASP A 582 45.228 −18.505 −32.080 1.00 27.79 N
ATOM 5967 CA ASP A 582 46.408 −17.666 −31.940 1.00 27.78 C
ATOM 5968 C ASP A 582 46.944 −17.392 −33.359 1.00 28.00 C
ATOM 5969 O ASP A 582 46.770 −18.225 −34.254 1.00 28.14 O
ATOM 5970 CB ASP A 582 47.445 −18.347 −31.016 1.00 27.66 C
ATOM 5971 CG ASP A 582 46.905 −18.974 −29.705 1.00 27.49 C
ATOM 5972 OD1 ASP A 582 45.723 −18.749 −29.346 1.00 27.55 O
ATOM 5973 OD2 ASP A 582 47.460 −20.027 −29.312 1.00 27.31 O
ATOM 5974 HN ASP A 582 45.379 −19.510 −32.128 1.00 0.00 H
ATOM 5975 HA ASP A 582 46.110 −16.718 −31.491 1.00 27.72 H
ATOM 5976 HB1 ASP A 582 47.926 −19.141 −31.588 1.00 27.72 H

ATOM	5977	HB2	ASP	A	582	48.186	−17.596	−30.742	1.00	27.72	H
ATOM	5978	N	THR	A	583	47.504	−16.206	−33.626	1.00	25.85	N
ATOM	5979	CA	THR	A	583	47.854	−15.758	−34.995	1.00	26.06	C
ATOM	5980	C	THR	A	583	49.079	−16.505	−35.557	1.00	26.14	C
ATOM	5981	O	THR	A	583	50.195	−15.993	−35.595	1.00	26.04	O
ATOM	5982	CB	THR	A	583	47.993	−14.225	−35.054	1.00	26.10	C
ATOM	5983	OG1	THR	A	583	46.803	−13.651	−34.555	1.00	26.02	O
ATOM	5984	CG2	THR	A	583	48.146	−13.683	−36.477	1.00	26.32	C
ATOM	5985	HN	THR	A	583	47.551	−15.520	−32.887	1.00	0.00	H
ATOM	5986	HG1	THR	A	583	47.004	−12.751	−34.266	1.00	0.00	H
ATOM	5987	HA	THR	A	583	47.010	−16.019	−35.633	1.00	26.08	H
ATOM	5988	HB	THR	A	583	48.839	−13.907	−34.445	1.00	26.08	H
ATOM	5989	1HG2	THR	A	583	48.239	−12.597	−36.445	1.00	26.08	H
ATOM	5990	2HG2	THR	A	583	47.270	−13.955	−37.066	1.00	26.08	H
ATOM	5991	3HG2	THR	A	583	49.038	−14.110	−36.935	1.00	26.08	H
ATOM	5992	N	GLY	A	584	48.871	−17.784	−35.880	1.00	35.45	N
ATOM	5993	CA	GLY	A	584	49.892	−18.808	−36.121	1.00	35.55	C
ATOM	5994	C	GLY	A	584	49.375	−20.258	−36.022	1.00	35.59	C
ATOM	5995	O	GLY	A	584	50.027	−21.162	−36.540	1.00	35.78	O
ATOM	5996	HN	GLY	A	584	47.930	−18.116	−35.706	1.00	0.00	H
ATOM	5997	HA1	GLY	A	584	50.296	−18.658	−37.122	1.00	35.59	H
ATOM	5998	HA2	GLY	A	584	50.686	−18.678	−35.385	1.00	35.59	H
ATOM	5999	N	ASP	A	585	48.167	−20.490	−35.487	1.00	26.30	N
ATOM	6000	CA	ASP	A	585	47.480	−21.792	−35.520	1.00	26.32	C
ATOM	6001	C	ASP	A	585	47.143	−22.237	−36.962	1.00	26.58	C
ATOM	6002	O	ASP	A	585	46.094	−21.890	−37.506	1.00	26.67	O
ATOM	6003	CB	ASP	A	585	46.180	−21.726	−34.689	1.00	26.11	C

ATOM 6004 CG ASP A 585 46.355 −21.843 −33.173 1.00 25.87 C

ATOM 6005 OD1 ASP A 585 47.051 −22.799 −32.763 1.00 25.85 O

ATOM 6006 OD2 ASP A 585 45.465 −21.325 −32.459 1.00 25.71 O

ATOM 6007 HN ASP A 585 47.670 −19.725 −35.044 1.00 0.00 H

ATOM 6008 HA ASP A 585 48.136 −22.539 −35.074 1.00 26.18 H

ATOM 6009 HB1 ASP A 585 45.697 −20.771 −34.898 1.00 26.18 H

ATOM 6010 HB2 ASP A 585 45.532 −22.540 −35.015 1.00 26.18 H

ATOM 6011 N SER A 586 47.909 −23.191 −37.506 1.00 35.84 N

ATOM 6012 CA SER A 586 47.547 −23.910 −38.747 1.00 36.14 C

ATOM 6013 C SER A 586 46.276 −24.760 −38.596 1.00 36.23 C

ATOM 6014 O SER A 586 45.555 −24.997 −39.567 1.00 36.41 O

ATOM 6015 CB SER A 586 48.679 −24.854 −39.168 1.00 36.26 C

ATOM 6016 OG SER A 586 49.908 −24.161 −39.273 1.00 36.32 O

ATOM 6017 HN SER A 586 48.842 −23.308 −37.135 1.00 0.00 H

ATOM 6018 HG SER A 586 50.606 −24.788 −39.475 1.00 0.00 H

ATOM 6019 HA SER A 586 47.387 −23.180 −39.540 1.00 36.20 H

ATOM 6020 HB1 SER A 586 48.779 −25.644 −38.424 1.00 36.20 H

ATOM 6021 HB2 SER A 586 48.435 −25.294 −40.135 1.00 36.20 H

ATOM 6022 N VAL A 587 46.025 −25.245 −37.374 1.00 42.69 N

ATOM 6023 CA VAL A 587 44.874 −26.073 −36.992 1.00 42.75 C

ATOM 6024 C VAL A 587 44.000 −25.266 −36.030 1.00 42.46 C

ATOM 6025 O VAL A 587 44.064 −25.443 −34.816 1.00 42.23 O

ATOM 6026 CB VAL A 587 45.325 −27.433 −36.411 1.00 42.92 C

ATOM 6027 CG1 VAL A 587 44.134 −28.338 −36.051 1.00 42.99 C

ATOM 6028 CG2 VAL A 587 46.196 −28.214 −37.405 1.00 43.25 C

ATOM 6029 HN VAL A 587 46.630 −24.924 −36.632 1.00 0.00 H

ATOM 6030 HA VAL A 587 44.290 −26.270 −37.891 1.00 42.76 H

ATOM 6031 HB VAL A 587 45.907 −27.249 −35.508 1.00 42.76 H

ATOM 6032 1HG1 VAL A 587 44.503 −29.281 −35.647 1.00 42.76 H

ATOM 6033 2HG1 VAL A 587 43.542 −28.533 −36.945 1.00 42.76 H

ATOM 6034 3HG1 VAL A 587 43.513 −27.842 −35.305 1.00 42.76 H

ATOM 6035 1HG2 VAL A 587 46.493 −29.164 −36.960 1.00 42.76 H

ATOM 6036 2HG2 VAL A 587 47.086 −27.632 −37.645 1.00 42.76 H

ATOM 6037 3HG2 VAL A 587 45.629 −28.402 −38.317 1.00 42.76 H

ATOM 6038 N ILE A 588 43.249 −24.310 −36.576 1.00 42.73 N

ATOM 6039 CA ILE A 588 42.336 −23.437 −35.825 1.00 42.49 C

ATOM 6040 C ILE A 588 41.344 −24.305 −35.043 1.00 42.51 C

ATOM 6041 O ILE A 588 40.645 −25.134 −35.634 1.00 42.72 O

ATOM 6042 CB ILE A 588 41.624 −22.470 −36.804 1.00 42.47 C

ATOM 6043 CG1 ILE A 588 42.651 −21.452 −37.352 1.00 42.41 C

ATOM 6044 CG2 ILE A 588 40.400 −21.784 −36.164 1.00 42.29 C

ATOM 6045 CD1 ILE A 588 42.103 −20.467 −38.392 1.00 42.43 C

ATOM 6046 HN ILE A 588 43.241 −24.251 −37.588 1.00 0.00 H

ATOM 6047 HA ILE A 588 42.919 −22.849 −35.116 1.00 42.51 H

ATOM 6048 HB ILE A 588 41.266 −23.061 −37.647 1.00 42.51 H

ATOM 6049 1HG1 ILE A 588 43.035 −20.875 −36.511 1.00 42.51 H

ATOM 6050 2HG1 ILE A 588 43.466 −22.010 −37.813 1.00 42.51 H

ATOM 6051 1HG2 ILE A 588 39.936 −21.117 −36.890 1.00 42.51 H

ATOM 6052 2HG2 ILE A 588 40.719 −21.209 −35.295 1.00 42.51 H

ATOM 6053 3HG2 ILE A 588 39.679 −22.541 −35.854 1.00 42.51 H

ATOM 6054 1HD1 ILE A 588 42.900 −19.796 −38.713 1.00 42.51 H

ATOM 6055 2HD1 ILE A 588 41.294 −19.884 −37.951 1.00 42.51 H

ATOM 6056 3HD1 ILE A 588 41.725 −21.019 −39.252 1.00 42.51 H

ATOM 6057 N ARG A 589 41.247 −24.131 −33.719 1.00 26.32 N

ATOM	6058	CA	ARG	A	589	40.341	−24.943	−32.892	1.00	26.34	C
ATOM	6059	C	ARG	A	589	39.897	−24.269	−31.590	1.00	26.07	C
ATOM	6060	O	ARG	A	589	40.740	−23.708	−30.899	1.00	25.86	O
ATOM	6061	CB	ARG	A	589	40.968	−26.324	−32.607	1.00	26.47	C
ATOM	6062	CG	ARG	A	589	42.338	−26.281	−31.894	1.00	26.31	C
ATOM	6063	CD	ARG	A	589	42.590	−27.542	−31.065	1.00	26.46	C
ATOM	6064	NE	ARG	A	589	41.671	−27.601	−29.910	1.00	26.36	N
ATOM	6065	CZ	ARG	A	589	41.283	−28.664	−29.238	1.00	26.51	C
ATOM	6066	NH1	ARG	A	589	41.759	−29.853	−29.479	1.00	26.80	N
ATOM	6067	NH2	ARG	A	589	40.387	−28.535	−28.307	1.00	26.40	N
ATOM	6068	HN	ARG	A	589	41.839	−23.442	−33.269	1.00	0.00	H
ATOM	6069	HE	ARG	A	589	41.225	−26.729	−29.639	1.00	0.00	H
ATOM	6070	1HH1	ARG	A	589	42.465	−29.941	−30.188	1.00	0.00	H
ATOM	6071	2HH1	ARG	A	589	41.449	−30.643	−28.950	1.00	0.00	H
ATOM	6072	1HH2	ARG	A	589	40.008	−27.600	−28.161	1.00	0.00	H
ATOM	6073	2HH2	ARG	A	589	40.035	−29.314	−27.790	1.00	0.00	H
ATOM	6074	HA	ARG	A	589	39.442	−25.118	−33.483	1.00	26.35	H
ATOM	6075	HB1	ARG	A	589	40.277	−26.887	−31.979	1.00	26.35	H
ATOM	6076	HB2	ARG	A	589	41.097	−26.840	−33.558	1.00	26.35	H
ATOM	6077	HG1	ARG	A	589	43.123	−26.190	−32.645	1.00	26.35	H
ATOM	6078	HG2	ARG	A	589	42.364	−25.414	−31.234	1.00	26.35	H
ATOM	6079	HD1	ARG	A	589	42.433	−28.420	−31.692	1.00	26.35	H
ATOM	6080	HD2	ARG	A	589	43.618	−27.533	−30.704	1.00	26.35	H
ATOM	6081	N	PRO	A	590	38.705	−24.627	−31.070	1.00	26.11	N
ATOM	6082	CA	PRO	A	590	38.286	−24.243	−29.730	1.00	25.90	C
ATOM	6083	C	PRO	A	590	39.253	−24.707	−28.634	1.00	25.71	C
ATOM	6084	O	PRO	A	590	39.733	−25.847	−28.656	1.00	25.80	O

ATOM 6085 CB PRO A 590 36.887 −24.833 −29.540 1.00 26.07 C
ATOM 6086 CG PRO A 590 36.346 −24.948 −30.960 1.00 26.35 C
ATOM 6087 CD PRO A 590 37.601 −25.271 −31.765 1.00 26.39 C
ATOM 6088 HA PRO A 590 38.212 −23.156 −29.689 1.00 26.05 H
ATOM 6089 HB1 PRO A 590 36.940 −25.812 −29.064 1.00 26.05 H
ATOM 6090 HB2 PRO A 590 36.264 −24.170 −28.940 1.00 26.05 H
ATOM 6091 HG1 PRO A 590 35.611 −25.749 −31.038 1.00 26.05 H
ATOM 6092 HG2 PRO A 590 35.898 −24.010 −31.288 1.00 26.05 H
ATOM 6093 HD1 PRO A 590 37.757 −26.349 −31.805 1.00 26.05 H
ATOM 6094 HD2 PRO A 590 37.512 −24.878 −32.778 1.00 26.05 H
ATOM 6095 N LYS A 591 39.444 −23.868 −27.615 1.00 25.46 N
ATOM 6096 CA LYS A 591 40.323 −24.111 −26.461 1.00 25.26 C
ATOM 6097 C LYS A 591 39.483 −24.012 −25.177 1.00 25.15 C
ATOM 6098 O LYS A 591 38.646 −23.117 −25.046 1.00 25.18 O
ATOM 6099 CB LYS A 591 41.543 −23.149 −26.503 1.00 25.06 C
ATOM 6100 CG LYS A 591 42.323 −23.218 −27.840 1.00 25.19 C
ATOM 6101 CD LYS A 591 43.554 −22.292 −27.974 1.00 25.02 C
ATOM 6102 CE LYS A 591 43.984 −22.263 −29.456 1.00 25.17 C
ATOM 6103 NZ LYS A 591 45.116 −21.350 −29.753 1.00 25.02 N1+
ATOM 6104 HN LYS A 591 38.938 −22.985 −27.638 1.00 0.00 H
ATOM 6105 HZ1 LYS A 591 45.355 −21.372 −30.749 1.00 0.00 H
ATOM 6106 HZ2 LYS A 591 44.950 −20.363 −29.541 1.00 0.00 H
ATOM 6107 HZ3 LYS A 591 45.995 −21.528 −29.278 1.00 0.00 H
ATOM 6108 HA LYS A 591 40.699 −25.131 −26.536 1.00 25.17 H
ATOM 6109 HB1 LYS A 591 41.186 −22.129 −26.362 1.00 25.17 H
ATOM 6110 HB2 LYS A 591 42.221 −23.413 −25.691 1.00 25.17 H
ATOM 6111 HG1 LYS A 591 42.666 −24.244 −27.969 1.00 25.17 H

ATOM 6112 HG2 LYS A 591 41.629 −22.963 −28.641 1.00 25.17 H
ATOM 6113 HD1 LYS A 591 43.294 −21.285 −27.647 1.00 25.17 H
ATOM 6114 HD2 LYS A 591 44.370 −22.675 −27.362 1.00 25.17 H
ATOM 6115 HE1 LYS A 591 44.277 −23.272 −29.745 1.00 25.17 H
ATOM 6116 HE2 LYS A 591 43.128 −21.948 −30.052 1.00 25.17 H
ATOM 6117 N LEU A 592 39.510 −25.070 −24.363 1.00 25.04 N
ATOM 6118 CA LEU A 592 38.758 −25.163 −23.103 1.00 24.93 C
ATOM 6119 C LEU A 592 39.537 −24.504 −21.960 1.00 24.62 C
ATOM 6120 O LEU A 592 40.650 −24.934 −21.652 1.00 24.49 O
ATOM 6121 CB LEU A 592 38.483 −26.636 −22.747 1.00 25.04 C
ATOM 6122 CG LEU A 592 37.369 −27.324 −23.547 1.00 25.31 C
ATOM 6123 CD1 LEU A 592 37.495 −28.842 −23.407 1.00 25.46 C
ATOM 6124 CD2 LEU A 592 35.992 −26.919 −23.016 1.00 25.25 C
ATOM 6125 HN LEU A 592 40.160 −25.814 −24.582 1.00 0.00 H
ATOM 6126 HA LEU A 592 37.806 −24.647 −23.223 1.00 25.02 H
ATOM 6127 HB1 LEU A 592 39.404 −27.196 −22.909 1.00 25.02 H
ATOM 6128 HB2 LEU A 592 38.211 −26.679 −21.692 1.00 25.02 H
ATOM 6129 HG LEU A 592 37.450 −27.047 −24.598 1.00 25.02 H
ATOM 6130 1HD1 LEU A 592 36.702 −29.327 −23.976 1.00 25.02 H
ATOM 6131 2HD1 LEU A 592 37.408 −29.119 −22.356 1.00 25.02 H
ATOM 6132 3HD1 LEU A 592 38.464 −29.163 −23.788 1.00 25.02 H
ATOM 6133 1HD2 LEU A 592 35.217 −27.418 −23.597 1.00 25.02 H
ATOM 6134 2HD2 LEU A 592 35.872 −25.839 −23.103 1.00 25.02 H
ATOM 6135 3HD2 LEU A 592 35.905 −27.211 −21.969 1.00 25.02 H
ATOM 6136 N PHE A 593 38.858 −23.665 −21.182 1.00 24.52 N
ATOM 6137 CA PHE A 593 39.402 −23.004 −19.998 1.00 24.25 C
ATOM 6138 C PHE A 593 38.589 −23.365 −18.750 1.00 24.15 C

ATOM	6139	O	PHE	A	593	37.381	−23.135	−18.698	1.00	24.24	O
ATOM	6140	CB	PHE	A	593	39.443	−21.485	−20.222	1.00	24.18	C
ATOM	6141	CG	PHE	A	593	40.314	−21.040	−21.385	1.00	24.24	C
ATOM	6142	CD1	PHE	A	593	41.638	−20.616	−21.158	1.00	24.07	C
ATOM	6143	CD2	PHE	A	593	39.789	−21.012	−22.692	1.00	24.47	C
ATOM	6144	CE1	PHE	A	593	42.424	−20.152	−22.228	1.00	24.15	C
ATOM	6145	CE2	PHE	A	593	40.578	−20.555	−23.762	1.00	24.53	C
ATOM	6146	CZ	PHE	A	593	41.894	−20.119	−23.530	1.00	24.38	C
ATOM	6147	HN	PHE	A	593	37.923	−23.388	−21.468	1.00	0.00	H
ATOM	6148	HA	PHE	A	593	40.424	−23.354	−19.852	1.00	24.29	H
ATOM	6149	HB1	PHE	A	593	38.426	−21.141	−20.409	1.00	24.29	H
ATOM	6150	HB2	PHE	A	593	39.824	−21.018	−19.314	1.00	24.29	H
ATOM	6151	HD1	PHE	A	593	42.054	−20.648	−20.151	1.00	24.29	H
ATOM	6152	HD2	PHE	A	593	38.768	−21.346	−22.874	1.00	24.29	H
ATOM	6153	HE1	PHE	A	593	43.446	−19.817	−22.048	1.00	24.29	H
ATOM	6154	HE2	PHE	A	593	40.169	−20.539	−24.772	1.00	24.29	H
ATOM	6155	HZ	PHE	A	593	42.503	−19.756	−24.358	1.00	24.29	H
ATOM	6156	N	ALA	A	594	39.261	−23.851	−17.708	1.00	23.97	N
ATOM	6157	CA	ALA	A	594	38.684	−24.025	−16.378	1.00	23.82	C
ATOM	6158	C	ALA	A	594	38.946	−22.784	−15.510	1.00	23.65	C
ATOM	6159	O	ALA	A	594	40.083	−22.312	−15.401	1.00	23.62	O
ATOM	6160	CB	ALA	A	594	39.237	−25.317	−15.764	1.00	23.65	C
ATOM	6161	HN	ALA	A	594	40.266	−23.955	−17.799	1.00	0.00	H
ATOM	6162	HA	ALA	A	594	37.606	−24.138	−16.489	1.00	23.74	H
ATOM	6163	HB1	ALA	A	594	38.813	−25.458	−14.770	1.00	23.74	H
ATOM	6164	HB2	ALA	A	594	40.322	−25.249	−15.689	1.00	23.74	H
ATOM	6165	HB3	ALA	A	594	38.969	−26.164	−16.396	1.00	23.74	H

ATOM 6166 N VAL A 595 37.915 −22.295 −14.817 1.00 23.58 N
ATOM 6167 CA VAL A 595 37.955 −21.059 −14.023 1.00 23.42 C
ATOM 6168 C VAL A 595 37.324 −21.284 −12.649 1.00 23.31 C
ATOM 6169 O VAL A 595 36.104 −21.292 −12.494 1.00 23.45 O
ATOM 6170 CB VAL A 595 37.279 −19.888 −14.772 1.00 23.57 C
ATOM 6171 CG1 VAL A 595 37.432 −18.581 −13.980 1.00 23.50 C
ATOM 6172 CG2 VAL A 595 37.892 −19.675 −16.162 1.00 23.58 C
ATOM 6173 HN VAL A 595 37.001 −22.710 −14.988 1.00 0.00 H
ATOM 6174 HA VAL A 595 39.002 −20.796 −13.869 1.00 23.49 H
ATOM 6175 HB VAL A 595 36.218 −20.109 −14.885 1.00 23.49 H
ATOM 6176 1HG1 VAL A 595 36.950 −17.768 −14.523 1.00 23.49 H
ATOM 6177 2HG1 VAL A 595 38.491 −18.355 −13.853 1.00 23.49 H
ATOM 6178 3HG1 VAL A 595 36.964 −18.692 −13.002 1.00 23.49 H
ATOM 6179 1HG2 VAL A 595 37.391 −18.844 −16.657 1.00 23.49 H
ATOM 6180 2HG2 VAL A 595 37.768 −20.580 −16.757 1.00 23.49 H
ATOM 6181 3HG2 VAL A 595 38.954 −19.450 −16.061 1.00 23.49 H
ATOM 6182 N LYS A 596 38.160 −21.395 −11.612 1.00 23.05 N
ATOM 6183 CA LYS A 596 37.708 −21.398 −10.214 1.00 22.94 C
ATOM 6184 C LYS A 596 37.272 −19.984 −9.827 1.00 22.97 C
ATOM 6185 O LYS A 596 38.136 −19.117 −9.663 1.00 22.96 O
ATOM 6186 CB LYS A 596 38.837 −21.910 −9.306 1.00 22.65 C
ATOM 6187 CG LYS A 596 38.355 −22.085 −7.853 1.00 22.52 C
ATOM 6188 CD LYS A 596 39.454 −21.895 −6.795 1.00 22.26 C
ATOM 6189 CE LYS A 596 40.247 −20.574 −6.872 1.00 22.06 C
ATOM 6190 NZ LYS A 596 39.403 −19.388 −7.178 1.00 21.94 N1+
ATOM 6191 HN LYS A 596 39.149 −21.439 −11.805 1.00 0.00 H
ATOM 6192 HZ1 LYS A 596 39.901 −18.520 −7.033 1.00 0.00 H

ATOM	6193	HZ2	LYS	A	596	39.105	−19.392	−8.153	1.00	0.00	H
ATOM	6194	HZ3	LYS	A	596	38.534	−19.393	−6.650	1.00	0.00	H
ATOM	6195	HA	LYS	A	596	36.852	−22.067	−10.123	1.00	22.59	H
ATOM	6196	HB1	LYS	A	596	39.186	−22.872	−9.682	1.00	22.59	H
ATOM	6197	HB2	LYS	A	596	39.658	−21.193	−9.323	1.00	22.59	H
ATOM	6198	HG1	LYS	A	596	37.569	−21.354	−7.664	1.00	22.59	H
ATOM	6199	HG2	LYS	A	596	37.949	−23.091	−7.747	1.00	22.59	H
ATOM	6200	HD1	LYS	A	596	38.983	−21.944	−5.813	1.00	22.59	H
ATOM	6201	HD2	LYS	A	596	40.163	−22.716	−6.900	1.00	22.59	H
ATOM	6202	HE1	LYS	A	596	40.736	−20.410	−5.912	1.00	22.59	H
ATOM	6203	HE2	LYS	A	596	41.001	−20.670	−7.653	1.00	22.59	H
ATOM	6204	N	ILE	A	597	36.023	−19.817	−9.404	1.00	27.88	N
ATOM	6205	CA	ILE	A	597	35.492	−18.542	−8.896	1.00	27.91	C
ATOM	6206	C	ILE	A	597	36.153	−18.149	−7.549	1.00	27.66	C
ATOM	6207	O	ILE	A	597	36.655	−19.019	−6.823	1.00	27.53	O
ATOM	6208	CB	ILE	A	597	33.949	−18.638	−8.842	1.00	28.14	C
ATOM	6209	CG1	ILE	A	597	33.322	−18.816	−10.249	1.00	28.39	C
ATOM	6210	CG2	ILE	A	597	33.250	−17.485	−8.104	1.00	28.13	C
ATOM	6211	CD1	ILE	A	597	33.636	−17.720	−11.281	1.00	28.37	C
ATOM	6212	HN	ILE	A	597	35.406	−20.624	−9.400	1.00	0.00	H
ATOM	6213	HA	ILE	A	597	35.746	−17.770	−9.622	1.00	28.00	H
ATOM	6214	HB	ILE	A	597	33.717	−19.546	−8.285	1.00	28.00	H
ATOM	6215	1HG1	ILE	A	597	33.678	−19.764	−10.653	1.00	28.00	H
ATOM	6216	2HG1	ILE	A	597	32.240	−18.856	−10.127	1.00	28.00	H
ATOM	6217	1HG2	ILE	A	597	32.172	−17.643	−8.121	1.00	28.00	H
ATOM	6218	2HG2	ILE	A	597	33.486	−16.542	−8.597	1.00	28.00	H
ATOM	6219	3HG2	ILE	A	597	33.596	−17.451	−7.071	1.00	28.00	H

```
ATOM   6220 1HD1 ILE A 597      33.142 −17.956 −12.224  1.00 28.00           H
ATOM   6221 2HD1 ILE A 597      34.713 −17.667 −11.439  1.00 28.00           H
ATOM   6222 3HD1 ILE A 597      33.275 −16.760 −10.913  1.00 28.00           H
ATOM   6223  N   PRO A 598      36.334 −16.849  −7.234  1.00 22.73           N
ATOM   6224  CA  PRO A 598      37.074 −16.429  −6.043  1.00 22.49           C
ATOM   6225  C   PRO A 598      36.364 −16.782  −4.730  1.00 22.50           C
ATOM   6226  O   PRO A 598      35.196 −16.439  −4.533  1.00 22.72           O
ATOM   6227  CB  PRO A 598      37.262 −14.919  −6.169  1.00 22.53           C
ATOM   6228  CG  PRO A 598      37.105 −14.661  −7.664  1.00 22.72           C
ATOM   6229  CD  PRO A 598      36.056 −15.684  −8.064  1.00 22.86           C
ATOM   6230  HA  PRO A 598      38.054 −16.907  −6.052  1.00 22.65           H
ATOM   6231  HB1 PRO A 598      36.502 −14.384  −5.600  1.00 22.65           H
ATOM   6232  HB2 PRO A 598      38.252 −14.620  −5.825  1.00 22.65           H
ATOM   6233  HG1 PRO A 598      36.758 −13.645  −7.854  1.00 22.65           H
ATOM   6234  HG2 PRO A 598      38.043 −14.830  −8.194  1.00 22.65           H
ATOM   6235  HD1 PRO A 598      35.054 −15.303  −7.867  1.00 22.65           H
ATOM   6236  HD2 PRO A 598      36.148 −15.935  −9.121  1.00 22.65           H
ATOM   6237  N   ALA A 599      37.124 −17.283  −3.752  1.00 51.52           N
ATOM   6238  CA  ALA A 599      36.645 −17.460  −2.380  1.00 51.48           C
ATOM   6239  C   ALA A 599      36.486 −16.114  −1.646  1.00 51.51           C
ATOM   6240  O   ALA A 599      35.415 −15.854  −1.093  1.00 51.74           O
ATOM   6241  CB  ALA A 599      37.609 −18.396  −1.641  1.00 51.20           C
ATOM   6242  HN  ALA A 599      38.092 −17.485  −3.943  1.00  0.00           H
ATOM   6243  HA  ALA A 599      35.668 −17.942  −2.421  1.00 51.49           H
ATOM   6244  HB1 ALA A 599      37.265 −18.538  −0.617  1.00 51.49           H
ATOM   6245  HB2 ALA A 599      38.607 −17.957  −1.631  1.00 51.49           H
ATOM   6246  HB3 ALA A 599      37.642 −19.360  −2.149  1.00 51.49           H
```

ATOM	6247	N	GLN	A	600	37.464	−15.219	−1.819	1.00	22.03	N
ATOM	6248	CA	GLN	A	600	37.531	−13.847	−1.299	1.00	22.07	C
ATOM	6249	C	GLN	A	600	37.956	−12.869	−2.408	1.00	22.26	C
ATOM	6250	O	GLN	A	600	38.484	−13.291	−3.435	1.00	22.27	O
ATOM	6251	CB	GLN	A	600	38.543	−13.777	−0.135	1.00	21.78	C
ATOM	6252	CG	GLN	A	600	38.166	−14.568	1.130	1.00	21.59	C
ATOM	6253	CD	GLN	A	600	37.017	−13.948	1.924	1.00	21.49	C
ATOM	6254	OE1	GLN	A	600	36.178	−13.225	1.415	1.00	21.31	O
ATOM	6255	NE2	GLN	A	600	36.925	−14.213	3.207	1.00	21.62	N
ATOM	6256	HN	GLN	A	600	38.250	−15.493	−2.391	1.00	0.00	H
ATOM	6257	1HE2	GLN	A	600	37.627	−14.756	3.675	1.00	0.00	H
ATOM	6258	2HE2	GLN	A	600	36.191	−13.739	3.704	1.00	0.00	H
ATOM	6259	HA	GLN	A	600	36.546	−13.559	−0.931	1.00	21.82	H
ATOM	6260	HB1	GLN	A	600	39.496	−14.161	−0.498	1.00	21.82	H
ATOM	6261	HB2	GLN	A	600	38.656	−12.730	0.147	1.00	21.82	H
ATOM	6262	HG1	GLN	A	600	37.874	−15.575	0.832	1.00	21.82	H
ATOM	6263	HG2	GLN	A	600	39.042	−14.620	1.777	1.00	21.82	H
ATOM	6264	N	CYS	A	601	37.772	−11.566	−2.191	1.00	42.68	N
ATOM	6265	CA	CYS	A	601	38.159	−10.496	−3.125	1.00	42.85	C
ATOM	6266	C	CYS	A	601	39.631	−10.045	−2.981	1.00	42.70	C
ATOM	6267	O	CYS	A	601	39.887	−8.853	−2.844	1.00	42.84	O
ATOM	6268	CB	CYS	A	601	37.187	−9.321	−2.957	1.00	43.15	C
ATOM	6269	SG	CYS	A	601	35.435	−9.738	−3.106	1.00	43.37	S
ATOM	6270	HN	CYS	A	601	37.355	−11.281	−1.317	1.00	0.00	H
ATOM	6271	HA	CYS	A	601	38.033	−10.883	−4.136	1.00	42.93	H
ATOM	6272	HB1	CYS	A	601	37.347	−8.891	−1.968	1.00	42.93	H
ATOM	6273	HB2	CYS	A	601	37.422	−8.578	−3.720	1.00	42.93	H

ATOM	6274	N	SER	A	602	40.534	−11.001	−2.749	1.00	26.10	N
ATOM	6275	CA	SER	A	602	41.901	−10.772	−2.258	1.00	25.93	C
ATOM	6276	C	SER	A	602	42.910	−10.475	−3.391	1.00	25.95	C
ATOM	6277	O	SER	A	602	42.968	−9.329	−3.838	1.00	26.14	O
ATOM	6278	CB	SER	A	602	42.249	−11.915	−1.295	1.00	25.61	C
ATOM	6279	OG	SER	A	602	43.589	−11.928	−0.882	1.00	25.48	O
ATOM	6280	HN	SER	A	602	40.257	−11.957	−2.913	1.00	0.00	H
ATOM	6281	HG	SER	A	602	44.011	−12.649	−1.406	1.00	0.00	H
ATOM	6282	HA	SER	A	602	41.856	−9.869	−1.650	1.00	25.87	H
ATOM	6283	HB1	SER	A	602	41.619	−11.821	−0.410	1.00	25.87	H
ATOM	6284	HB2	SER	A	602	42.036	−12.860	−1.794	1.00	25.87	H
ATOM	6285	N	GLU	A	603	43.591	−11.479	−3.961	1.00	29.34	N
ATOM	6286	CA	GLU	A	603	44.657	−11.334	−4.988	1.00	29.44	C
ATOM	6287	C	GLU	A	603	44.332	−11.947	−6.357	1.00	29.48	C
ATOM	6288	O	GLU	A	603	44.816	−11.410	−7.383	1.00	29.30	O
ATOM	6289	CB	GLU	A	603	46.038	−11.811	−4.466	1.00	29.25	C
ATOM	6290	CG	GLU	A	603	46.126	−12.750	−3.244	1.00	29.06	C
ATOM	6291	CD	GLU	A	603	45.186	−13.971	−3.281	1.00	28.76	C
ATOM	6292	OE1	GLU	A	603	45.598	−15.037	−3.796	1.00	28.62	O
ATOM	6293	OE2	GLU	A	603	44.157	−13.899	−2.558	1.00	28.68	O
ATOM	6294	OXT	GLU	A	603	43.919	−13.121	−6.402	1.00	0.00	O
ATOM	6295	HN	GLU	A	603	43.492	−12.407	−3.555	1.00	0.00	H
ATOM	6296	HA	GLU	A	603	44.759	−10.262	−5.160	1.00	29.10	H
ATOM	6297	HB1	GLU	A	603	46.524	−12.327	−5.294	1.00	29.10	H
ATOM	6298	HB2	GLU	A	603	46.604	−10.914	−4.214	1.00	29.10	H
ATOM	6299	HG1	GLU	A	603	47.150	−13.116	−3.174	1.00	29.10	H
ATOM	6300	HG2	GLU	A	603	45.886	−12.168	−2.354	1.00	29.10	H

TER 6301 GLU A 603

ATOM 6302 N ILE B 31 49.221 −5.258 −37.647 1.00 50.96 N1+

ATOM 6303 CA ILE B 31 49.286 −3.776 −37.451 1.00 51.06 C

ATOM 6304 C ILE B 31 47.926 −3.173 −37.787 1.00 51.06 C

ATOM 6305 O ILE B 31 47.183 −3.729 −38.600 1.00 51.05 O

ATOM 6306 CB ILE B 31 50.336 −3.112 −38.395 1.00 51.29 C

ATOM 6307 CG1 ILE B 31 51.758 −3.536 −38.028 1.00 51.33 C

ATOM 6308 CG2 ILE B 31 50.208 −1.546 −38.391 1.00 51.44 C

ATOM 6309 CD1 ILE B 31 52.608 −2.386 −37.518 1.00 51.46 C

ATOM 6310 HT1 ILE B 31 50.171 −5.691 −37.414 1.00 51.21 H

ATOM 6311 HT2 ILE B 31 48.972 −5.473 −38.665 1.00 51.21 H

ATOM 6312 HT3 ILE B 31 48.473 −5.671 −37.002 1.00 51.21 H

ATOM 6313 HA ILE B 31 49.537 −3.556 −36.413 1.00 51.21 H

ATOM 6314 HB ILE B 31 50.137 −3.459 −39.409 1.00 51.21 H

ATOM 6315 1HG1 ILE B 31 51.703 −4.298 −37.251 1.00 51.21 H

ATOM 6316 2HG1 ILE B 31 52.235 −3.954 −38.914 1.00 51.21 H

ATOM 6317 1HG2 ILE B 31 50.955 −1.118 −39.060 1.00 51.21 H

ATOM 6318 2HG2 ILE B 31 50.368 −1.170 −37.380 1.00 51.21 H

ATOM 6319 3HG2 ILE B 31 49.212 −1.261 −38.730 1.00 51.21 H

ATOM 6320 1HD1 ILE B 31 53.606 −2.751 −37.274 1.00 51.21 H

ATOM 6321 2HD1 ILE B 31 52.149 −1.962 −36.625 1.00 51.21 H

ATOM 6322 3HD1 ILE B 31 52.681 −1.618 −38.288 1.00 51.21 H

ATOM 6323 N VAL B 32 47.623 −2.030 −37.167 1.00 33.20 N

ATOM 6324 CA VAL B 32 46.446 −1.238 −37.480 1.00 33.25 C

ATOM 6325 C VAL B 32 46.813 −0.295 −38.611 1.00 33.47 C

ATOM 6326 O VAL B 32 47.472 0.727 −38.365 1.00 33.61 O

ATOM 6327 CB VAL B 32 46.005 −0.420 −36.211 1.00 33.21 C

ATOM 6328 CG1 VAL B 32 44.722 0.371 −36.465 1.00 33.26 C
ATOM 6329 CG2 VAL B 32 45.851 −1.363 −35.004 1.00 32.99 C
ATOM 6330 HN VAL B 32 48.281 −1.679 −36.400 1.00 33.28 H
ATOM 6331 HA VAL B 32 45.634 −1.894 −37.796 1.00 33.28 H
ATOM 6332 HB VAL B 32 46.797 0.292 −35.980 1.00 33.28 H
ATOM 6333 1HG1 VAL B 32 44.450 0.923 −35.565 1.00 33.28 H
ATOM 6334 2HG1 VAL B 32 43.917 −0.316 −36.727 1.00 33.28 H
ATOM 6335 3HG1 VAL B 32 44.882 1.071 −37.285 1.00 33.28 H
ATOM 6336 1HG2 VAL B 32 45.545 −0.788 −34.130 1.00 33.28 H
ATOM 6337 2HG2 VAL B 32 46.803 −1.853 −34.801 1.00 33.28 H
ATOM 6338 3HG2 VAL B 32 45.095 −2.116 −35.225 1.00 33.28 H
ATOM 6339 N LEU B 33 46.383 −0.632 −39.831 1.00 26.95 N
ATOM 6340 CA LEU B 33 46.721 0.160 −41.038 1.00 27.15 C
ATOM 6341 C LEU B 33 45.926 1.448 −41.050 1.00 27.24 C
ATOM 6342 O LEU B 33 45.022 1.643 −40.202 1.00 27.15 O
ATOM 6343 CB LEU B 33 46.438 −0.587 −42.333 1.00 27.16 C
ATOM 6344 CG LEU B 33 46.835 −2.031 −42.184 1.00 27.05 C
ATOM 6345 CD1 LEU B 33 46.549 −2.702 −43.470 1.00 27.10 C
ATOM 6346 CD2 LEU B 33 48.295 −2.169 −41.717 1.00 27.11 C
ATOM 6347 HN LEU B 33 45.773 −1.504 −39.940 1.00 27.11 H
ATOM 6348 HA LEU B 33 47.782 0.407 −41.005 1.00 27.11 H
ATOM 6349 HB1 LEU B 33 45.374 −0.526 −42.561 1.00 27.11 H
ATOM 6350 HB2 LEU B 33 47.010 −0.136 −43.144 1.00 27.11 H
ATOM 6351 HG LEU B 33 46.196 −2.478 −41.423 1.00 27.11 H
ATOM 6352 1HD1 LEU B 33 46.825 −3.754 −43.401 1.00 27.11 H
ATOM 6353 2HD1 LEU B 33 47.126 −2.228 −44.264 1.00 27.11 H
ATOM 6354 3HD1 LEU B 33 45.486 −2.620 −43.695 1.00 27.11 H

ATOM	6355	1HD2	LEU	B	33	48.548	−3.225	−41.620	1.00	27.11	H
ATOM	6356	2HD2	LEU	B	33	48.416	−1.677	−40.752	1.00	27.11	H
ATOM	6357	3HD2	LEU	B	33	48.956	−1.703	−42.448	1.00	27.11	H
ATOM	6358	N	GLU	B	34	46.260	2.312	−42.020	1.00	36.14	N
ATOM	6359	CA	GLU	B	34	45.668	3.660	−42.136	1.00	36.28	C
ATOM	6360	C	GLU	B	34	44.172	3.679	−42.510	1.00	36.20	C
ATOM	6361	O	GLU	B	34	43.845	3.369	−43.682	1.00	36.20	O
ATOM	6362	CB	GLU	B	34	46.409	4.502	−43.171	1.00	36.52	C
ATOM	6363	CG	GLU	B	34	45.752	5.863	−43.333	1.00	36.67	C
ATOM	6364	CD	GLU	B	34	46.491	6.793	−44.246	1.00	36.93	C
ATOM	6365	OE1	GLU	B	34	45.966	7.901	−44.469	1.00	37.08	O
ATOM	6366	OE2	GLU	B	34	47.576	6.437	−44.744	1.00	37.01	O
ATOM	6367	HN	GLU	B	34	46.989	2.011	−42.743	1.00	36.56	H
ATOM	6368	HA	GLU	B	34	45.774	4.153	−41.170	1.00	36.56	H
ATOM	6369	HB1	GLU	B	34	47.440	4.640	−42.847	1.00	36.56	H
ATOM	6370	HB2	GLU	B	34	46.395	3.983	−44.129	1.00	36.56	H
ATOM	6371	HG1	GLU	B	34	44.750	5.714	−43.734	1.00	36.56	H
ATOM	6372	HG2	GLU	B	34	45.685	6.330	−42.350	1.00	36.56	H
ATOM	6373	N	PRO	B	35	43.281	4.088	−41.532	1.00	27.44	N
ATOM	6374	CA	PRO	B	35	41.828	4.229	−41.798	1.00	27.39	C
ATOM	6375	C	PRO	B	35	41.502	4.773	−43.219	1.00	27.51	C
ATOM	6376	O	PRO	B	35	42.136	5.734	−43.692	1.00	27.70	O
ATOM	6377	CB	PRO	B	35	41.354	5.183	−40.679	1.00	27.45	C
ATOM	6378	CG	PRO	B	35	42.261	4.907	−39.560	1.00	27.40	C
ATOM	6379	CD	PRO	B	35	43.587	4.443	−40.123	1.00	27.43	C
ATOM	6380	HA	PRO	B	35	41.346	3.260	−41.668	1.00	27.47	H
ATOM	6381	HB1	PRO	B	35	41.438	6.222	−40.997	1.00	27.47	H

ATOM	6382	HB2	PRO	B	35	40.322	4.969	−40.401	1.00	27.47	H
ATOM	6383	HG1	PRO	B	35	42.408	5.814	−38.973	1.00	27.47	H
ATOM	6384	HG2	PRO	B	35	41.837	4.128	−38.926	1.00	27.47	H
ATOM	6385	HD1	PRO	B	35	44.327	5.242	−40.078	1.00	27.47	H
ATOM	6386	HD2	PRO	B	35	43.956	3.575	−39.577	1.00	27.47	H
ATOM	6387	N	ILE	B	36	40.555	4.137	−43.904	1.00	33.35	N
ATOM	6388	CA	ILE	B	36	40.287	4.524	−45.294	1.00	33.44	C
ATOM	6389	C	ILE	B	36	38.923	5.196	−45.428	1.00	33.45	C
ATOM	6390	O	ILE	B	36	37.874	4.578	−45.228	1.00	33.32	O
ATOM	6391	CB	ILE	B	36	40.453	3.380	−46.337	1.00	33.36	C
ATOM	6392	CG1	ILE	B	36	41.890	2.840	−46.303	1.00	33.39	C
ATOM	6393	CG2	ILE	B	36	40.124	3.930	−47.746	1.00	33.45	C
ATOM	6394	CD1	ILE	B	36	42.121	1.644	−47.180	1.00	33.34	C
ATOM	6395	HN	ILE	B	36	39.994	3.350	−43.445	1.00	33.39	H
ATOM	6396	HA	ILE	B	36	41.028	5.281	−45.549	1.00	33.39	H
ATOM	6397	HB	ILE	B	36	39.761	2.573	−46.097	1.00	33.39	H
ATOM	6398	1HG1	ILE	B	36	42.561	3.635	−46.627	1.00	33.39	H
ATOM	6399	2HG1	ILE	B	36	42.125	2.561	−45.276	1.00	33.39	H
ATOM	6400	1HG2	ILE	B	36	40.238	3.135	−48.483	1.00	33.39	H
ATOM	6401	2HG2	ILE	B	36	40.805	4.747	−47.986	1.00	33.39	H
ATOM	6402	3HG2	ILE	B	36	39.097	4.296	−47.763	1.00	33.39	H
ATOM	6403	1HD1	ILE	B	36	43.161	1.327	−47.097	1.00	33.39	H
ATOM	6404	2HD1	ILE	B	36	41.903	1.905	−48.216	1.00	33.39	H
ATOM	6405	3HD1	ILE	B	36	41.467	0.830	−46.865	1.00	33.39	H
ATOM	6406	N	TYR	B	37	38.956	6.467	−45.789	1.00	32.53	N
ATOM	6407	CA	TYR	B	37	37.753	7.260	−45.795	1.00	32.58	C
ATOM	6408	C	TYR	B	37	37.085	7.074	−47.144	1.00	32.55	C

ATOM 6409 O TYR B 37 37.670 7.404 −48.220 1.00 32.64 O
ATOM 6410 CB TYR B 37 38.076 8.723 −45.537 1.00 32.82 C
ATOM 6411 CG TYR B 37 38.590 8.984 −44.142 1.00 32.86 C
ATOM 6412 CD1 TYR B 37 37.821 9.718 −43.239 1.00 32.94 C
ATOM 6413 CD2 TYR B 37 39.841 8.494 −43.709 1.00 32.84 C
ATOM 6414 CE1 TYR B 37 38.268 9.983 −41.938 1.00 33.00 C
ATOM 6415 CE2 TYR B 37 40.302 8.738 −42.398 1.00 32.88 C
ATOM 6416 CZ TYR B 37 39.504 9.499 −41.514 1.00 32.96 C
ATOM 6417 OH TYR B 37 39.896 9.795 −40.210 1.00 33.01 O
ATOM 6418 HN TYR B 37 39.885 6.911 −46.080 1.00 32.80 H
ATOM 6419 HA TYR B 37 37.084 6.899 −45.013 1.00 32.80 H
ATOM 6420 HB1 TYR B 37 38.836 9.040 −46.251 1.00 32.80 H
ATOM 6421 HB2 TYR B 37 37.169 9.309 −45.687 1.00 32.80 H
ATOM 6422 HD1 TYR B 37 36.848 10.095 −43.553 1.00 32.80 H
ATOM 6423 HD2 TYR B 37 40.460 7.919 −44.398 1.00 32.80 H
ATOM 6424 HE1 TYR B 37 37.649 10.568 −41.257 1.00 32.80 H
ATOM 6425 HE2 TYR B 37 41.264 8.344 −42.070 1.00 32.80 H
ATOM 6426 HH TYR B 37 39.949 10.837 −40.088 1.00 32.80 H
ATOM 6427 N TRP B 38 35.878 6.510 −47.097 1.00 27.57 N
ATOM 6428 CA TRP B 38 35.093 6.315 −48.302 1.00 27.53 C
ATOM 6429 C TRP B 38 34.406 7.614 −48.565 1.00 27.67 C
ATOM 6430 O TRP B 38 33.454 7.969 −47.871 1.00 27.68 O
ATOM 6431 CB TRP B 38 34.084 5.211 −48.140 1.00 27.37 C
ATOM 6432 CG TRP B 38 33.455 4.871 −49.413 1.00 27.33 C
ATOM 6433 CD1 TRP B 38 32.468 5.548 −50.005 1.00 27.37 C
ATOM 6434 CD2 TRP B 38 33.768 3.756 −50.276 1.00 27.25 C
ATOM 6435 NE1 TRP B 38 32.124 4.949 −51.190 1.00 27.31 N

ATOM 6436 CE2 TRP B 38 32.904 3.843 −51.385 1.00 27.24 C
ATOM 6437 CE3 TRP B 38 34.683 2.694 −50.209 1.00 27.20 C
ATOM 6438 CZ2 TRP B 38 32.914 2.918 −52.435 1.00 27.19 C
ATOM 6439 CZ3 TRP B 38 34.700 1.777 −51.239 1.00 27.16 C
ATOM 6440 CH2 TRP B 38 33.808 1.896 −52.349 1.00 27.16 C
ATOM 6441 HN TRP B 38 35.481 6.196 −46.154 1.00 27.36 H
ATOM 6442 HA TRP B 38 35.757 6.081 −49.134 1.00 27.36 H
ATOM 6443 HB1 TRP B 38 34.586 4.327 −47.746 1.00 27.36 H
ATOM 6444 HB2 TRP B 38 33.314 5.535 −47.440 1.00 27.36 H
ATOM 6445 HD1 TRP B 38 32.002 6.448 −49.604 1.00 27.36 H
ATOM 6446 HE1 TRP B 38 31.363 5.294 −51.858 1.00 27.36 H
ATOM 6447 HE3 TRP B 38 35.364 2.596 −49.363 1.00 27.36 H
ATOM 6448 HZ2 TRP B 38 32.236 3.012 −53.283 1.00 27.36 H
ATOM 6449 HZ3 TRP B 38 35.406 0.947 −51.206 1.00 27.36 H
ATOM 6450 HH2 TRP B 38 33.845 1.152 −53.144 1.00 27.36 H
ATOM 6451 N ASN B 39 34.935 8.324 −49.565 1.00 27.79 N
ATOM 6452 CA ASN B 39 34.608 9.731 −49.861 1.00 27.97 C
ATOM 6453 C ASN B 39 35.036 10.078 −51.309 1.00 28.03 C
ATOM 6454 O ASN B 39 35.979 9.479 −51.831 1.00 28.00 O
ATOM 6455 CB ASN B 39 35.345 10.612 −48.854 1.00 28.14 C
ATOM 6456 CG ASN B 39 34.580 11.799 −48.494 1.00 28.30 C
ATOM 6457 OD1 ASN B 39 33.433 11.930 −48.871 1.00 28.27 O
ATOM 6458 ND2 ASN B 39 35.197 12.688 −47.746 1.00 28.50 N
ATOM 6459 HN ASN B 39 35.648 7.837 −50.197 1.00 28.13 H
ATOM 6460 HA ASN B 39 33.534 9.882 −49.756 1.00 28.13 H
ATOM 6461 HB1 ASN B 39 35.534 10.030 −47.952 1.00 28.13 H
ATOM 6462 HB2 ASN B 39 36.293 10.925 −49.291 1.00 28.13 H

ATOM	6463	1HD2	ASN	B	39	36.207	12.518	−47.436	1.00	28.13	H
ATOM	6464	2HD2	ASN	B	39	34.689	13.581	−47.448	1.00	28.13	H
ATOM	6465	N	SER	B	40	34.374	11.024	−51.966	1.00	28.13	N
ATOM	6466	CA	SER	B	40	34.701	11.300	−53.377	1.00	28.17	C
ATOM	6467	C	SER	B	40	36.033	11.980	−53.531	1.00	28.37	C
ATOM	6468	O	SER	B	40	36.599	12.003	−54.621	1.00	28.41	O
ATOM	6469	CB	SER	B	40	33.688	12.245	−53.993	1.00	28.24	C
ATOM	6470	OG	SER	B	40	33.741	13.474	−53.299	1.00	28.46	O
ATOM	6471	HN	SER	B	40	33.607	11.583	−51.473	1.00	28.30	H
ATOM	6472	HA	SER	B	40	34.707	10.364	−53.936	1.00	28.30	H
ATOM	6473	HB1	SER	B	40	33.927	12.406	−55.044	1.00	28.30	H
ATOM	6474	HB2	SER	B	40	32.689	11.818	−53.909	1.00	28.30	H
ATOM	6475	HG	SER	B	40	33.513	13.315	−52.287	1.00	28.30	H
ATOM	6476	N	SER	B	41	36.480	12.578	−52.431	1.00	28.51	N
ATOM	6477	CA	SER	B	41	37.696	13.392	−52.364	1.00	28.75	C
ATOM	6478	C	SER	B	41	38.971	12.588	−52.276	1.00	28.72	C
ATOM	6479	O	SER	B	41	40.064	13.090	−52.603	1.00	28.91	O
ATOM	6480	CB	SER	B	41	37.637	14.268	−51.105	1.00	28.92	C
ATOM	6481	OG	SER	B	41	37.350	13.467	−49.970	1.00	28.75	O
ATOM	6482	HN	SER	B	41	35.912	12.456	−51.532	1.00	28.76	H
ATOM	6483	HA	SER	B	41	37.742	14.035	−53.243	1.00	28.76	H
ATOM	6484	HB1	SER	B	41	38.598	14.762	−50.964	1.00	28.76	H
ATOM	6485	HB2	SER	B	41	36.856	15.019	−51.224	1.00	28.76	H
ATOM	6486	HG	SER	B	41	36.424	12.990	−50.103	1.00	28.76	H
ATOM	6487	N	ASN	B	42	38.826	11.351	−51.808	1.00	69.62	N
ATOM	6488	CA	ASN	B	42	39.988	10.553	−51.394	1.00	69.59	C
ATOM	6489	C	ASN	B	42	40.954	10.192	−52.569	1.00	69.64	C

ATOM	6490	O	ASN	B	42	40.805	9.167	−53.271	1.00	69.49	O
ATOM	6491	CB	ASN	B	42	39.546	9.314	−50.548	1.00	69.35	C
ATOM	6492	CG	ASN	B	42	40.695	8.728	−49.685	1.00	69.34	C
ATOM	6493	OD1	ASN	B	42	41.878	9.081	−49.873	1.00	69.50	O
ATOM	6494	ND2	ASN	B	42	40.342	7.821	−48.742	1.00	69.16	N
ATOM	6495	HN	ASN	B	42	37.845	10.930	−51.732	1.00	69.46	H
ATOM	6496	HA	ASN	B	42	40.562	11.188	−50.719	1.00	69.46	H
ATOM	6497	HB1	ASN	B	42	38.735	9.617	−49.886	1.00	69.46	H
ATOM	6498	HB2	ASN	B	42	39.192	8.539	−51.227	1.00	69.46	H
ATOM	6499	1HD2	ASN	B	42	39.315	7.544	−48.624	1.00	69.46	H
ATOM	6500	2HD2	ASN	B	42	41.090	7.382	−48.116	1.00	69.46	H
ATOM	6501	N	SER	B	43	41.915	11.102	−52.757	1.00	37.97	N
ATOM	6502	CA	SER	B	43	43.149	10.912	−53.539	1.00	38.10	C
ATOM	6503	C	SER	B	43	43.836	9.539	−53.531	1.00	37.97	C
ATOM	6504	O	SER	B	43	44.881	9.398	−54.157	1.00	38.09	O
ATOM	6505	CB	SER	B	43	44.213	11.921	−53.050	1.00	38.38	C
ATOM	6506	OG	SER	B	43	43.850	13.290	−53.303	1.00	38.58	O
ATOM	6507	HN	SER	B	43	41.775	12.058	−52.297	1.00	38.18	H
ATOM	6508	HA	SER	B	43	42.919	11.156	−54.576	1.00	38.18	H
ATOM	6509	HB1	SER	B	43	44.347	11.790	−51.976	1.00	38.18	H
ATOM	6510	HB2	SER	B	43	45.152	11.711	−53.562	1.00	38.18	H
ATOM	6511	HG	SER	B	43	43.726	13.435	−54.336	1.00	38.18	H
ATOM	6512	N	LYS	B	44	43.320	8.560	−52.782	1.00	28.53	N
ATOM	6513	CA	LYS	B	44	43.884	7.192	−52.785	1.00	28.41	C
ATOM	6514	C	LYS	B	44	43.153	6.300	−53.782	1.00	28.26	C
ATOM	6515	O	LYS	B	44	43.718	5.332	−54.260	1.00	28.24	O
ATOM	6516	CB	LYS	B	44	43.857	6.565	−51.384	1.00	28.28	C

ATOM	6517	CG	LYS	B	44	45.026	6.978	−50.481	1.00	28.42	C
ATOM	6518	CD	LYS	B	44	45.298	5.906	−49.388	1.00	28.25	C
ATOM	6519	CE	LYS	B	44	46.060	6.460	−48.187	1.00	28.35	C
ATOM	6520	NZ	LYS	B	44	45.178	7.321	−47.339	1.00	28.34	N1+
ATOM	6521	HN	LYS	B	44	42.474	8.772	−52.162	1.00	28.34	H
ATOM	6522	HA	LYS	B	44	44.925	7.261	−53.100	1.00	28.34	H
ATOM	6523	HB1	LYS	B	44	42.929	6.861	−50.896	1.00	28.34	H
ATOM	6524	HB2	LYS	B	44	43.879	5.481	−51.496	1.00	28.34	H
ATOM	6525	HG1	LYS	B	44	45.921	7.099	−51.091	1.00	28.34	H
ATOM	6526	HG2	LYS	B	44	44.785	7.925	−49.998	1.00	28.34	H
ATOM	6527	HD1	LYS	B	44	44.342	5.513	−49.042	1.00	28.34	H
ATOM	6528	HD2	LYS	B	44	45.884	5.100	−49.830	1.00	28.34	H
ATOM	6529	HE1	LYS	B	44	46.430	5.629	−47.586	1.00	28.34	H
ATOM	6530	HE2	LYS	B	44	46.901	7.054	−48.543	1.00	28.34	H
ATOM	6531	HZ1	LYS	B	44	45.737	7.695	−46.507	1.00	28.34	H
ATOM	6532	HZ2	LYS	B	44	44.351	6.743	−46.982	1.00	28.34	H
ATOM	6533	HZ3	LYS	B	44	44.814	8.142	−47.921	1.00	28.34	H
ATOM	6534	N	PHE	B	45	41.888	6.615	−54.063	1.00	30.56	N
ATOM	6535	CA	PHE	B	45	41.142	5.924	−55.118	1.00	30.45	C
ATOM	6536	C	PHE	B	45	41.637	6.438	−56.463	1.00	30.59	C
ATOM	6537	O	PHE	B	45	41.175	7.443	−57.019	1.00	30.66	O
ATOM	6538	CB	PHE	B	45	39.628	6.107	−54.969	1.00	30.31	C
ATOM	6539	CG	PHE	B	45	39.047	5.411	−53.759	1.00	30.16	C
ATOM	6540	CD1	PHE	B	45	38.600	4.094	−53.849	1.00	30.01	C
ATOM	6541	CD2	PHE	B	45	38.941	6.075	−52.536	1.00	30.19	C
ATOM	6542	CE1	PHE	B	45	38.069	3.455	−52.752	1.00	29.89	C
ATOM	6543	CE2	PHE	B	45	38.398	5.429	−51.417	1.00	30.05	C

ATOM 6544 CZ PHE B 45 37.971 4.128 −51.528 1.00 29.90 C
ATOM 6545 HN PHE B 45 41.406 7.391 −53.505 1.00 30.25 H
ATOM 6546 HA PHE B 45 41.366 4.859 −55.055 1.00 30.25 H
ATOM 6547 HB1 PHE B 45 39.417 7.173 −54.887 1.00 30.25 H
ATOM 6548 HB2 PHE B 45 39.144 5.708 −55.860 1.00 30.25 H
ATOM 6549 HD1 PHE B 45 38.672 3.564 −54.799 1.00 30.25 H
ATOM 6550 HD2 PHE B 45 39.284 7.106 −52.450 1.00 30.25 H
ATOM 6551 HE1 PHE B 45 37.724 2.424 −52.836 1.00 30.25 H
ATOM 6552 HE2 PHE B 45 38.315 5.955 −50.466 1.00 30.25 H
ATOM 6553 HZ PHE B 45 37.554 3.618 −50.660 1.00 30.25 H
ATOM 6554 N LEU B 46 42.612 5.731 −56.971 1.00 28.27 N
ATOM 6555 CA LEU B 46 43.269 6.144 −58.174 1.00 28.43 C
ATOM 6556 C LEU B 46 42.418 5.659 −59.331 1.00 28.32 C
ATOM 6557 O LEU B 46 41.747 4.638 −59.229 1.00 28.16 O
ATOM 6558 CB LEU B 46 44.688 5.592 −58.180 1.00 28.56 C
ATOM 6559 CG LEU B 46 45.358 5.882 −56.825 1.00 28.62 C
ATOM 6560 CD1 LEU B 46 46.769 5.282 −56.665 1.00 28.74 C
ATOM 6561 CD2 LEU B 46 45.334 7.382 −56.580 1.00 28.77 C
ATOM 6562 HN LEU B 46 42.923 4.832 −56.481 1.00 28.48 H
ATOM 6563 HA LEU B 46 43.312 7.233 −58.198 1.00 28.48 H
ATOM 6564 HB1 LEU B 46 44.658 4.515 −58.347 1.00 28.48 H
ATOM 6565 HB2 LEU B 46 45.259 6.068 −58.977 1.00 28.48 H
ATOM 6566 HG LEU B 46 44.732 5.426 −56.058 1.00 28.48 H
ATOM 6567 1HD1 LEU B 46 47.163 5.536 −55.681 1.00 28.48 H
ATOM 6568 2HD1 LEU B 46 47.426 5.687 −57.435 1.00 28.48 H
ATOM 6569 3HD1 LEU B 46 46.717 4.198 −56.766 1.00 28.48 H
ATOM 6570 1HD2 LEU B 46 45.806 7.602 −55.622 1.00 28.48 H

ATOM	6571	2HD2	LEU	B	46	44.302	7.732	−56.564	1.00	28.48	H
ATOM	6572	3HD2	LEU	B	46	45.877	7.890	−57.377	1.00	28.48	H
ATOM	6573	N	PRO	B	47	42.473	6.374	−60.455	1.00	50.87	N
ATOM	6574	CA	PRO	B	47	41.393	6.378	−61.454	1.00	50.76	C
ATOM	6575	C	PRO	B	47	41.316	5.105	−62.288	1.00	50.68	C
ATOM	6576	O	PRO	B	47	40.215	4.694	−62.685	1.00	50.53	O
ATOM	6577	CB	PRO	B	47	41.777	7.554	−62.367	1.00	50.93	C
ATOM	6578	CG	PRO	B	47	43.281	7.564	−62.319	1.00	51.14	C
ATOM	6579	CD	PRO	B	47	43.639	7.157	−60.904	1.00	51.11	C
ATOM	6580	HA	PRO	B	47	40.435	6.572	−60.972	1.00	50.86	H
ATOM	6581	HB1	PRO	B	47	41.421	7.389	−63.384	1.00	50.86	H
ATOM	6582	HB2	PRO	B	47	41.370	8.491	−61.987	1.00	50.86	H
ATOM	6583	HG1	PRO	B	47	43.690	6.852	−63.036	1.00	50.86	H
ATOM	6584	HG2	PRO	B	47	43.663	8.561	−62.537	1.00	50.86	H
ATOM	6585	HD1	PRO	B	47	44.543	6.548	−60.893	1.00	50.86	H
ATOM	6586	HD2	PRO	B	47	43.783	8.034	−60.273	1.00	50.86	H
ATOM	6587	N	GLY	B	48	42.477	4.509	−62.555	1.00	28.37	N
ATOM	6588	CA	GLY	B	48	42.559	3.243	−63.269	1.00	28.35	C
ATOM	6589	C	GLY	B	48	42.433	2.024	−62.358	1.00	28.24	C
ATOM	6590	O	GLY	B	48	41.724	1.067	−62.689	1.00	28.14	O
ATOM	6591	HN	GLY	B	48	43.383	4.978	−62.233	1.00	28.27	H
ATOM	6592	HA1	GLY	B	48	41.756	3.209	−64.005	1.00	28.27	H
ATOM	6593	HA2	GLY	B	48	43.521	3.195	−63.778	1.00	28.27	H
ATOM	6594	N	GLN	B	49	43.114	2.043	−61.214	1.00	28.27	N
ATOM	6595	CA	GLN	B	49	43.201	0.844	−60.369	1.00	28.19	C
ATOM	6596	C	GLN	B	49	42.697	1.049	−58.918	1.00	28.06	C
ATOM	6597	O	GLN	B	49	43.001	0.257	−58.005	1.00	28.01	O

ATOM 6598 CB GLN B 49 44.639 0.307 −60.399 1.00 28.36 C

ATOM 6599 CG GLN B 49 45.655 1.209 −59.673 1.00 28.48 C

ATOM 6600 CD GLN B 49 46.786 1.714 −60.577 1.00 28.73 C

ATOM 6601 OE1 GLN B 49 47.735 0.974 −60.930 1.00 28.86 O

ATOM 6602 NE2 GLN B 49 46.692 2.996 −60.949 1.00 28.80 N

ATOM 6603 HN GLN B 49 43.604 2.943 −60.906 1.00 28.42 H

ATOM 6604 HA GLN B 49 42.566 0.084 −60.824 1.00 28.42 H

ATOM 6605 HB1 GLN B 49 44.649 −0.674 −59.924 1.00 28.42 H

ATOM 6606 HB2 GLN B 49 44.948 0.210 −61.440 1.00 28.42 H

ATOM 6607 HG1 GLN B 49 45.124 2.072 −59.272 1.00 28.42 H

ATOM 6608 HG2 GLN B 49 46.096 0.641 −58.854 1.00 28.42 H

ATOM 6609 1HE2 GLN B 49 45.868 3.591 −60.615 1.00 28.42 H

ATOM 6610 2HE2 GLN B 49 47.441 3.426 −61.580 1.00 28.42 H

ATOM 6611 N GLY B 50 41.921 2.109 −58.714 1.00 28.01 N

ATOM 6612 CA GLY B 50 41.274 2.334 −57.428 1.00 27.89 C

ATOM 6613 C GLY B 50 42.292 2.429 −56.317 1.00 27.95 C

ATOM 6614 O GLY B 50 43.433 2.763 −56.582 1.00 28.11 O

ATOM 6615 HN GLY B 50 41.769 2.809 −59.508 1.00 27.99 H

ATOM 6616 HA1 GLY B 50 40.708 3.264 −57.472 1.00 27.99 H

ATOM 6617 HA2 GLY B 50 40.597 1.505 −57.220 1.00 27.99 H

ATOM 6618 N LEU B 51 41.878 2.136 −55.083 1.00 27.82 N

ATOM 6619 CA LEU B 51 42.781 2.102 −53.932 1.00 27.85 C

ATOM 6620 C LEU B 51 43.190 0.660 −53.795 1.00 27.80 C

ATOM 6621 O LEU B 51 42.336 −0.192 −53.500 1.00 27.65 O

ATOM 6622 CB LEU B 51 42.072 2.579 −52.647 1.00 27.75 C

ATOM 6623 CG LEU B 51 42.863 2.902 −51.368 1.00 27.79 C

ATOM 6624 CD1 LEU B 51 41.990 3.760 −50.479 1.00 27.75 C

ATOM 6625 CD2 LEU B 51 43.330 1.685 −50.606 1.00 27.69 C

ATOM 6626 HN LEU B 51 40.842 1.917 −54.928 1.00 27.76 H

ATOM 6627 HA LEU B 51 43.657 2.721 −54.127 1.00 27.76 H

ATOM 6628 HB1 LEU B 51 41.532 3.488 −52.913 1.00 27.76 H

ATOM 6629 HB2 LEU B 51 41.360 1.798 −52.380 1.00 27.76 H

ATOM 6630 HG LEU B 51 43.740 3.485 −51.649 1.00 27.76 H

ATOM 6631 1HD1 LEU B 51 42.530 4.003 −49.564 1.00 27.76 H

ATOM 6632 2HD1 LEU B 51 41.079 3.216 −50.229 1.00 27.76 H

ATOM 6633 3HD1 LEU B 51 41.731 4.680 −51.002 1.00 27.76 H

ATOM 6634 1HD2 LEU B 51 43.879 2.000 −49.719 1.00 27.76 H

ATOM 6635 2HD2 LEU B 51 43.981 1.085 −51.242 1.00 27.76 H

ATOM 6636 3HD2 LEU B 51 42.467 1.090 −50.307 1.00 27.76 H

ATOM 6637 N VAL B 52 44.471 0.373 −54.053 1.00 37.58 N

ATOM 6638 CA VAL B 52 45.048 −0.957 −53.747 1.00 37.55 C

ATOM 6639 C VAL B 52 45.842 −0.904 −52.419 1.00 37.54 C

ATOM 6640 O VAL B 52 46.563 0.084 −52.140 1.00 37.65 O

ATOM 6641 CB VAL B 52 45.887 −1.505 −54.900 1.00 37.71 C

ATOM 6642 CG1 VAL B 52 46.605 −2.775 −54.467 1.00 37.72 C

ATOM 6643 CG2 VAL B 52 44.973 −1.766 −56.111 1.00 37.69 C

ATOM 6644 HN VAL B 52 45.095 1.123 −54.492 1.00 37.63 H

ATOM 6645 HA VAL B 52 44.215 −1.644 −53.598 1.00 37.63 H

ATOM 6646 HB VAL B 52 46.631 −0.759 −55.177 1.00 37.63 H

ATOM 6647 1HG1 VAL B 52 47.200 −3.157 −55.296 1.00 37.63 H

ATOM 6648 2HG1 VAL B 52 45.871 −3.525 −54.171 1.00 37.63 H

ATOM 6649 3HG1 VAL B 52 47.259 −2.554 −53.623 1.00 37.63 H

ATOM 6650 1HG2 VAL B 52 45.567 −2.157 −56.937 1.00 37.63 H

ATOM 6651 2HG2 VAL B 52 44.498 −0.834 −56.416 1.00 37.63 H

ATOM	6652	3HG2	VAL	B	52	44.207	−2.492	−55.839	1.00	37.63	H
ATOM	6653	N	LEU	B	53	45.684	−1.946	−51.599	1.00	27.76	N
ATOM	6654	CA	LEU	B	53	46.359	−2.010	−50.321	1.00	27.72	C
ATOM	6655	C	LEU	B	53	46.774	−3.471	−50.061	1.00	27.68	C
ATOM	6656	O	LEU	B	53	46.086	−4.409	−50.489	1.00	27.62	O
ATOM	6657	CB	LEU	B	53	45.438	−1.415	−49.242	1.00	27.58	C
ATOM	6658	CG	LEU	B	53	45.867	−1.413	−47.764	1.00	27.50	C
ATOM	6659	CD1	LEU	B	53	47.116	−0.570	−47.566	1.00	27.65	C
ATOM	6660	CD2	LEU	B	53	44.726	−0.864	−46.960	1.00	27.37	C
ATOM	6661	HN	LEU	B	53	45.044	−2.751	−51.894	1.00	27.61	H
ATOM	6662	HA	LEU	B	53	47.261	−1.401	−50.377	1.00	27.61	H
ATOM	6663	HB1	LEU	B	53	45.265	−0.375	−49.518	1.00	27.61	H
ATOM	6664	HB2	LEU	B	53	44.499	−1.967	−49.294	1.00	27.61	H
ATOM	6665	HG	LEU	B	53	46.070	−2.436	−47.447	1.00	27.61	H
ATOM	6666	1HD1	LEU	B	53	47.401	−0.583	−46.514	1.00	27.61	H
ATOM	6667	2HD1	LEU	B	53	46.915	0.456	−47.875	1.00	27.61	H
ATOM	6668	3HD1	LEU	B	53	47.929	−0.977	−48.167	1.00	27.61	H
ATOM	6669	1HD2	LEU	B	53	44.998	−0.849	−45.905	1.00	27.61	H
ATOM	6670	2HD2	LEU	B	53	43.847	−1.493	−47.099	1.00	27.61	H
ATOM	6671	3HD2	LEU	B	53	44.502	0.150	−47.291	1.00	27.61	H
ATOM	6672	N	TYR	B	54	47.907	−3.648	−49.376	1.00	29.37	N
ATOM	6673	CA	TYR	B	54	48.516	−4.965	−49.157	1.00	29.36	C
ATOM	6674	C	TYR	B	54	48.367	−5.461	−47.713	1.00	29.18	C
ATOM	6675	O	TYR	B	54	49.374	−5.643	−47.037	1.00	29.20	O
ATOM	6676	CB	TYR	B	54	50.041	−4.925	−49.482	1.00	29.57	C
ATOM	6677	CG	TYR	B	54	50.475	−4.506	−50.908	1.00	29.78	C
ATOM	6678	CD1	TYR	B	54	49.552	−4.437	−51.969	1.00	29.79	C

ATOM	6679	CD2	TYR	B	54	51.819	−4.197	−51.189	1.00	30.00	C
ATOM	6680	CE1	TYR	B	54	49.935	−4.046	−53.233	1.00	29.98	C
ATOM	6681	CE2	TYR	B	54	52.208	−3.808	−52.467	1.00	30.21	C
ATOM	6682	CZ	TYR	B	54	51.255	−3.743	−53.483	1.00	30.19	C
ATOM	6683	OH	TYR	B	54	51.592	−3.390	−54.776	1.00	30.38	O
ATOM	6684	HN	TYR	B	54	48.394	−2.788	−48.967	1.00	29.75	H
ATOM	6685	HA	TYR	B	54	48.038	−5.684	−49.822	1.00	29.75	H
ATOM	6686	HB1	TYR	B	54	50.501	−4.224	−48.785	1.00	29.75	H
ATOM	6687	HB2	TYR	B	54	50.435	−5.926	−49.305	1.00	29.75	H
ATOM	6688	HD1	TYR	B	54	48.510	−4.699	−51.786	1.00	29.75	H
ATOM	6689	HD2	TYR	B	54	52.564	−4.263	−50.396	1.00	29.75	H
ATOM	6690	HE1	TYR	B	54	49.197	−3.977	−54.032	1.00	29.75	H
ATOM	6691	HE2	TYR	B	54	53.248	−3.556	−52.672	1.00	29.75	H
ATOM	6692	HH	TYR	B	54	51.360	−4.180	−55.427	1.00	29.75	H
ATOM	6693	N	PRO	B	55	47.132	−5.692	−47.210	1.00	37.09	N
ATOM	6694	CA	PRO	B	55	47.173	−6.055	−45.778	1.00	36.93	C
ATOM	6695	C	PRO	B	55	47.896	−7.383	−45.593	1.00	36.94	C
ATOM	6696	O	PRO	B	55	48.082	−8.150	−46.567	1.00	37.06	O
ATOM	6697	CB	PRO	B	55	45.699	−6.151	−45.360	1.00	36.78	C
ATOM	6698	CG	PRO	B	55	44.907	−6.272	−46.628	1.00	36.84	C
ATOM	6699	CD	PRO	B	55	45.760	−5.692	−47.758	1.00	37.03	C
ATOM	6700	HA	PRO	B	55	47.674	−5.273	−45.207	1.00	36.95	H
ATOM	6701	HB1	PRO	B	55	45.542	−7.028	−44.732	1.00	36.95	H
ATOM	6702	HB2	PRO	B	55	45.403	−5.255	−44.815	1.00	36.95	H
ATOM	6703	HG1	PRO	B	55	44.685	−7.320	−46.830	1.00	36.95	H
ATOM	6704	HG2	PRO	B	55	43.976	−5.713	−46.540	1.00	36.95	H
ATOM	6705	HD1	PRO	B	55	45.699	−6.317	−48.649	1.00	36.95	H

ATOM 6706 HD2 PRO B 55 45.441 −4.679 −48.002 1.00 36.95 H
ATOM 6707 N GLN B 56 48.352 −7.624 −44.372 1.00 27.12 N
ATOM 6708 CA GLN B 56 48.898 −8.924 −44.003 1.00 27.10 C
ATOM 6709 C GLN B 56 47.963 −9.541 −43.000 1.00 26.90 C
ATOM 6710 O GLN B 56 47.247 −8.804 −42.303 1.00 26.78 O
ATOM 6711 CB GLN B 56 50.299 −8.786 −43.405 1.00 27.14 C
ATOM 6712 CG GLN B 56 51.379 −8.392 −44.404 1.00 27.37 C
ATOM 6713 CD GLN B 56 51.463 −9.353 −45.558 1.00 27.52 C
ATOM 6714 OE1 GLN B 56 51.285 −8.967 −46.723 1.00 27.66 O
ATOM 6715 NE2 GLN B 56 51.774 −10.615 −45.249 1.00 27.52 N
ATOM 6716 HN GLN B 56 48.318 −6.845 −43.639 1.00 27.23 H
ATOM 6717 HA GLN B 56 48.946 −9.558 −44.888 1.00 27.23 H
ATOM 6718 HB1 GLN B 56 50.262 −8.024 −42.626 1.00 27.23 H
ATOM 6719 HB2 GLN B 56 50.576 −9.744 −42.965 1.00 27.23 H
ATOM 6720 HG1 GLN B 56 51.154 −7.398 −44.791 1.00 27.23 H
ATOM 6721 HG2 GLN B 56 52.341 −8.374 −43.892 1.00 27.23 H
ATOM 6722 1HE2 GLN B 56 51.946 −10.889 −44.229 1.00 27.23 H
ATOM 6723 2HE2 GLN B 56 51.851 −11.352 −46.021 1.00 27.23 H
ATOM 6724 N ILE B 57 47.947 −10.878 −42.927 1.00 26.90 N
ATOM 6725 CA ILE B 57 47.113 −11.560 −41.912 1.00 26.72 C
ATOM 6726 C ILE B 57 47.480 −11.069 −40.484 1.00 26.56 C
ATOM 6727 O ILE B 57 48.656 −11.017 −40.100 1.00 26.58 O
ATOM 6728 CB ILE B 57 47.176 −13.112 −41.961 1.00 26.77 C
ATOM 6729 CG1 ILE B 57 46.622 −13.660 −43.268 1.00 26.93 C
ATOM 6730 CG2 ILE B 57 46.361 −13.718 −40.797 1.00 26.59 C
ATOM 6731 CD1 ILE B 57 45.126 −13.717 −43.302 1.00 26.86 C
ATOM 6732 HN ILE B 57 48.540 −11.456 −43.605 1.00 26.74 H

ATOM 6733 HA ILE B 57 46.078 −11.272 −42.097 1.00 26.74 H

ATOM 6734 HB ILE B 57 48.216 −13.425 −41.862 1.00 26.74 H

ATOM 6735 1HG1 ILE B 57 46.962 −13.020 −44.082 1.00 26.74 H

ATOM 6736 2HG1 ILE B 57 47.009 −14.669 −43.412 1.00 26.74 H

ATOM 6737 1HG2 ILE B 57 46.414 −14.806 −40.843 1.00 26.74 H

ATOM 6738 2HG2 ILE B 57 45.321 −13.401 −40.877 1.00 26.74 H

ATOM 6739 3HG2 ILE B 57 46.772 −13.375 −39.848 1.00 26.74 H

ATOM 6740 1HD1 ILE B 57 44.799 −14.117 −44.262 1.00 26.74 H

ATOM 6741 2HD1 ILE B 57 44.721 −12.714 −43.170 1.00 26.74 H

ATOM 6742 3HD1 ILE B 57 44.768 −14.362 −42.499 1.00 26.74 H

ATOM 6743 N GLY B 58 46.460 −10.688 −39.720 1.00 42.67 N

ATOM 6744 CA GLY B 58 46.659 −10.070 −38.412 1.00 42.53 C

ATOM 6745 C GLY B 58 46.482 −8.542 −38.365 1.00 42.55 C

ATOM 6746 O GLY B 58 46.496 −7.915 −37.274 1.00 42.45 O

ATOM 6747 HN GLY B 58 45.462 −10.839 −40.075 1.00 42.55 H

ATOM 6748 HA1 GLY B 58 45.942 −10.512 −37.720 1.00 42.55 H

ATOM 6749 HA2 GLY B 58 47.672 −10.300 −38.083 1.00 42.55 H

ATOM 6750 N ASP B 59 46.328 −7.920 −39.532 1.00 51.40 N

ATOM 6751 CA ASP B 59 46.115 −6.469 −39.567 1.00 51.44 C

ATOM 6752 C ASP B 59 44.653 −6.144 −39.234 1.00 51.36 C

ATOM 6753 O ASP B 59 43.812 −7.052 −39.115 1.00 51.28 O

ATOM 6754 CB ASP B 59 46.486 −5.882 −40.937 1.00 51.63 C

ATOM 6755 CG ASP B 59 47.870 −6.294 −41.395 1.00 51.75 C

ATOM 6756 OD1 ASP B 59 48.540 −7.040 −40.653 1.00 51.68 O

ATOM 6757 OD2 ASP B 59 48.282 −5.901 −42.511 1.00 51.92 O

ATOM 6758 HN ASP B 59 46.360 −8.478 −40.444 1.00 51.56 H

ATOM 6759 HA ASP B 59 46.751 −6.009 −38.811 1.00 51.56 H

ATOM 6760 HB1 ASP B 59 45.759 −6.226 −41.672 1.00 51.56 H
ATOM 6761 HB2 ASP B 59 46.450 −4.795 −40.871 1.00 51.56 H
ATOM 6762 N LYS B 60 44.362 −4.857 −39.059 1.00 26.43 N
ATOM 6763 CA LYS B 60 42.987 −4.387 −39.003 1.00 26.41 C
ATOM 6764 C LYS B 60 42.851 −3.002 −39.667 1.00 26.55 C
ATOM 6765 O LYS B 60 43.737 −2.141 −39.556 1.00 26.64 O
ATOM 6766 CB LYS B 60 42.464 −4.379 −37.561 1.00 26.27 C
ATOM 6767 CG LYS B 60 43.426 −3.754 −36.557 1.00 26.26 C
ATOM 6768 CD LYS B 60 42.775 −3.472 −35.243 1.00 26.15 C
ATOM 6769 CE LYS B 60 42.503 −4.747 −34.458 1.00 25.98 C
ATOM 6770 NZ LYS B 60 41.463 −4.561 −33.413 1.00 25.90 N1+
ATOM 6771 HN LYS B 60 45.159 −4.151 −38.957 1.00 26.29 H
ATOM 6772 HA LYS B 60 42.375 −5.087 −39.572 1.00 26.29 H
ATOM 6773 HB1 LYS B 60 41.531 −3.816 −37.538 1.00 26.29 H
ATOM 6774 HB2 LYS B 60 42.275 −5.409 −37.259 1.00 26.29 H
ATOM 6775 HG1 LYS B 60 44.257 −4.440 −36.395 1.00 26.29 H
ATOM 6776 HG2 LYS B 60 43.802 −2.818 −36.969 1.00 26.29 H
ATOM 6777 HD1 LYS B 60 43.431 −2.830 −34.655 1.00 26.29 H
ATOM 6778 HD2 LYS B 60 41.829 −2.960 −35.421 1.00 26.29 H
ATOM 6779 HE1 LYS B 60 42.168 −5.519 −35.151 1.00 26.29 H
ATOM 6780 HE2 LYS B 60 43.428 −5.066 −33.978 1.00 26.29 H
ATOM 6781 HZ1 LYS B 60 41.311 −5.483 −32.893 1.00 26.29 H
ATOM 6782 HZ2 LYS B 60 40.546 −4.255 −33.872 1.00 26.29 H
ATOM 6783 HZ3 LYS B 60 41.783 −3.810 −32.721 1.00 26.29 H
ATOM 6784 N LEU B 61 41.711 −2.812 −40.336 1.00 26.57 N
ATOM 6785 CA LEU B 61 41.493 −1.716 −41.269 1.00 26.71 C
ATOM 6786 C LEU B 61 40.137 −1.129 −41.043 1.00 26.70 C

ATOM	6787	O	LEU	B	61	39.152	−1.865	−41.044	1.00	26.62	O
ATOM	6788	CB	LEU	B	61	41.527	−2.225	−42.730	1.00	26.78	C
ATOM	6789	CG	LEU	B	61	41.606	−1.143	−43.837	1.00	26.93	C
ATOM	6790	CD1	LEU	B	61	43.054	−0.696	−43.896	1.00	27.05	C
ATOM	6791	CD2	LEU	B	61	41.119	−1.565	−45.232	1.00	26.97	C
ATOM	6792	HN	LEU	B	61	40.912	−3.505	−40.177	1.00	26.79	H
ATOM	6793	HA	LEU	B	61	42.256	−0.951	−41.126	1.00	26.79	H
ATOM	6794	HB1	LEU	B	61	42.399	−2.871	−42.835	1.00	26.79	H
ATOM	6795	HB2	LEU	B	61	40.621	−2.806	−42.899	1.00	26.79	H
ATOM	6796	HG	LEU	B	61	41.006	−0.292	−43.516	1.00	26.79	H
ATOM	6797	1HD1	LEU	B	61	43.167	0.070	−44.663	1.00	26.79	H
ATOM	6798	2HD1	LEU	B	61	43.688	−1.549	−44.138	1.00	26.79	H
ATOM	6799	3HD1	LEU	B	61	43.348	−0.287	−42.929	1.00	26.79	H
ATOM	6800	1HD2	LEU	B	61	41.222	−0.728	−45.922	1.00	26.79	H
ATOM	6801	2HD2	LEU	B	61	40.072	−1.863	−45.177	1.00	26.79	H
ATOM	6802	3HD2	LEU	B	61	41.717	−2.404	−45.588	1.00	26.79	H
ATOM	6803	N	ASP	B	62	40.079	0.187	−40.881	1.00	26.81	N
ATOM	6804	CA	ASP	B	62	38.803	0.887	−40.796	1.00	26.83	C
ATOM	6805	C	ASP	B	62	38.449	1.432	−42.205	1.00	26.95	C
ATOM	6806	O	ASP	B	62	39.273	2.054	−42.890	1.00	27.07	O
ATOM	6807	CB	ASP	B	62	38.846	2.025	−39.745	1.00	26.91	C
ATOM	6808	CG	ASP	B	62	38.912	1.518	−38.245	1.00	26.78	C
ATOM	6809	OD1	ASP	B	62	39.049	0.306	−37.950	1.00	26.63	O
ATOM	6810	OD2	ASP	B	62	38.821	2.372	−37.328	1.00	26.84	O
ATOM	6811	HN	ASP	B	62	40.988	0.746	−40.811	1.00	26.85	H
ATOM	6812	HA	ASP	B	62	38.036	0.172	−40.499	1.00	26.85	H
ATOM	6813	HB1	ASP	B	62	39.727	2.637	−39.939	1.00	26.85	H

ATOM	6814	HB2	ASP	B	62	37.949	2.633	−39.862	1.00	26.85	H
ATOM	6815	N	ILE	B	63	37.222	1.170	−42.650	1.00	37.00	N
ATOM	6816	CA	ILE	B	63	36.613	1.959	−43.755	1.00	37.11	C
ATOM	6817	C	ILE	B	63	35.496	2.855	−43.161	1.00	37.15	C
ATOM	6818	O	ILE	B	63	34.620	2.374	−42.405	1.00	37.07	O
ATOM	6819	CB	ILE	B	63	36.097	1.090	−44.922	1.00	37.06	C
ATOM	6820	CG1	ILE	B	63	37.235	0.886	−45.947	1.00	37.11	C
ATOM	6821	CG2	ILE	B	63	34.865	1.734	−45.559	1.00	37.11	C
ATOM	6822	CD1	ILE	B	63	36.882	−0.008	−47.123	1.00	37.09	C
ATOM	6823	HN	ILE	B	63	36.662	0.375	−42.204	1.00	37.09	H
ATOM	6824	HA	ILE	B	63	37.385	2.617	−44.153	1.00	37.09	H
ATOM	6825	HB	ILE	B	63	35.811	0.116	−44.526	1.00	37.09	H
ATOM	6826	1HG1	ILE	B	63	37.518	1.863	−46.338	1.00	37.09	H
ATOM	6827	2HG1	ILE	B	63	38.084	0.442	−45.427	1.00	37.09	H
ATOM	6828	1HG2	ILE	B	63	34.512	1.110	−46.380	1.00	37.09	H
ATOM	6829	2HG2	ILE	B	63	35.126	2.721	−45.940	1.00	37.09	H
ATOM	6830	3HG2	ILE	B	63	34.077	1.830	−44.812	1.00	37.09	H
ATOM	6831	1HD1	ILE	B	63	37.742	−0.092	−47.787	1.00	37.09	H
ATOM	6832	2HD1	ILE	B	63	36.043	0.423	−47.669	1.00	37.09	H
ATOM	6833	3HD1	ILE	B	63	36.608	−0.998	−46.758	1.00	37.09	H
ATOM	6834	N	ILE	B	64	35.569	4.156	−43.466	1.00	27.21	N
ATOM	6835	CA	ILE	B	64	34.886	5.204	−42.706	1.00	27.31	C
ATOM	6836	C	ILE	B	64	34.228	6.182	−43.652	1.00	27.44	C
ATOM	6837	O	ILE	B	64	34.925	6.826	−44.415	1.00	27.55	O
ATOM	6838	CB	ILE	B	64	35.955	5.989	−41.845	1.00	27.42	C
ATOM	6839	CG1	ILE	B	64	36.356	5.197	−40.598	1.00	27.29	C
ATOM	6840	CG2	ILE	B	64	35.469	7.359	−41.411	1.00	27.61	C

ATOM	6841	CD1	ILE	B	64	36.982	6.058	−39.519	1.00	27.40	C
ATOM	6842	HN	ILE	B	64	36.158	4.446	−44.311	1.00	27.40	H
ATOM	6843	HA	ILE	B	64	34.136	4.759	−42.051	1.00	27.40	H
ATOM	6844	HB	ILE	B	64	36.846	6.126	−42.458	1.00	27.40	H
ATOM	6845	1HG1	ILE	B	64	35.465	4.722	−40.188	1.00	27.40	H
ATOM	6846	2HG1	ILE	B	64	37.075	4.431	−40.890	1.00	27.40	H
ATOM	6847	1HG2	ILE	B	64	36.244	7.851	−40.824	1.00	27.40	H
ATOM	6848	2HG2	ILE	B	64	34.569	7.251	−40.806	1.00	27.40	H
ATOM	6849	3HG2	ILE	B	64	35.244	7.961	−42.291	1.00	27.40	H
ATOM	6850	1HD1	ILE	B	64	37.243	5.436	−38.663	1.00	27.40	H
ATOM	6851	2HD1	ILE	B	64	36.272	6.824	−39.208	1.00	27.40	H
ATOM	6852	3HD1	ILE	B	64	37.882	6.534	−39.910	1.00	27.40	H
ATOM	6853	N	CYS	B	65	32.906	6.307	−43.621	1.00	48.51	N
ATOM	6854	CA	CYS	B	65	32.260	7.447	−44.291	1.00	48.66	C
ATOM	6855	C	CYS	B	65	32.056	8.582	−43.296	1.00	48.83	C
ATOM	6856	O	CYS	B	65	31.347	8.416	−42.280	1.00	48.81	O
ATOM	6857	CB	CYS	B	65	30.922	7.108	−44.897	1.00	48.60	C
ATOM	6858	SG	CYS	B	65	30.887	5.504	−45.519	1.00	48.40	S
ATOM	6859	HN	CYS	B	65	32.311	5.579	−43.109	1.00	48.63	H
ATOM	6860	HA	CYS	B	65	32.919	7.799	−45.085	1.00	48.64	H
ATOM	6861	HB1	CYS	B	65	30.153	7.205	−44.130	1.00	48.64	H
ATOM	6862	HB2	CYS	B	65	30.716	7.805	−45.709	1.00	48.64	H
ATOM	6863	N	PRO	B	66	32.646	9.754	−43.594	1.00	45.59	N
ATOM	6864	CA	PRO	B	66	32.628	10.799	−42.605	1.00	45.79	C
ATOM	6865	C	PRO	B	66	31.234	11.383	−42.559	1.00	45.88	C
ATOM	6866	O	PRO	B	66	30.445	11.127	−43.468	1.00	45.81	O
ATOM	6867	CB	PRO	B	66	33.661	11.800	−43.136	1.00	45.98	C

ATOM 6868 CG PRO B 66 33.612 11.661 −44.575 1.00 45.94 C

ATOM 6869 CD PRO B 66 33.302 10.203 −44.838 1.00 45.67 C

ATOM 6870 HA PRO B 66 32.923 10.416 −41.628 1.00 45.81 H

ATOM 6871 HB1 PRO B 66 33.397 12.815 −42.840 1.00 45.81 H

ATOM 6872 HB2 PRO B 66 34.656 11.559 −42.762 1.00 45.81 H

ATOM 6873 HG1 PRO B 66 32.830 12.296 −44.991 1.00 45.81 H

ATOM 6874 HG2 PRO B 66 34.572 11.930 −45.015 1.00 45.81 H

ATOM 6875 HD1 PRO B 66 32.632 10.096 −45.691 1.00 45.81 H

ATOM 6876 HD2 PRO B 66 34.216 9.638 −45.021 1.00 45.81 H

ATOM 6877 N LYS B 67 30.933 12.097 −41.477 1.00 28.40 N

ATOM 6878 CA LYS B 67 29.701 12.889 −41.363 1.00 28.56 C

ATOM 6879 C LYS B 67 29.888 14.292 −41.942 1.00 28.83 C

ATOM 6880 O LYS B 67 30.983 14.816 −41.905 1.00 28.95 O

ATOM 6881 CB LYS B 67 29.230 12.969 −39.896 1.00 28.63 C

ATOM 6882 CG LYS B 67 30.001 13.910 −39.000 1.00 28.85 C

ATOM 6883 CD LYS B 67 29.736 13.590 −37.524 1.00 28.84 C

ATOM 6884 CE LYS B 67 30.537 14.491 −36.601 1.00 29.06 C

ATOM 6885 NZ LYS B 67 31.878 14.813 −37.179 1.00 29.10 N1+

ATOM 6886 HN LYS B 67 31.618 12.094 −40.655 1.00 28.80 H

ATOM 6887 HA LYS B 67 28.924 12.388 −41.939 1.00 28.80 H

ATOM 6888 HB1 LYS B 67 28.188 13.290 −39.898 1.00 28.80 H

ATOM 6889 HB2 LYS B 67 29.302 11.969 −39.468 1.00 28.80 H

ATOM 6890 HG1 LYS B 67 31.067 13.805 −39.203 1.00 28.80 H

ATOM 6891 HG2 LYS B 67 29.691 14.934 −39.205 1.00 28.80 H

ATOM 6892 HD1 LYS B 67 28.675 13.728 −37.318 1.00 28.80 H

ATOM 6893 HD2 LYS B 67 30.012 12.553 −37.332 1.00 28.80 H

ATOM 6894 HE1 LYS B 67 29.986 15.419 −36.446 1.00 28.80 H

ATOM	6895	HE2	LYS	B	67	30.675	13.986	−35.645	1.00	28.80	H
ATOM	6896	HZ1	LYS	B	67	32.414	15.448	−36.505	1.00	28.80	H
ATOM	6897	HZ2	LYS	B	67	31.752	15.314	−38.116	1.00	28.80	H
ATOM	6898	HZ3	LYS	B	67	32.428	13.908	−37.330	1.00	28.80	H
ATOM	6899	N	VAL	B	68	28.810	14.897	−42.444	1.00	40.30	N
ATOM	6900	CA	VAL	B	68	28.866	16.227	−43.111	1.00	40.57	C
ATOM	6901	C	VAL	B	68	29.258	17.386	−42.164	1.00	40.89	C
ATOM	6902	O	VAL	B	68	28.473	17.822	−41.314	1.00	41.04	O
ATOM	6903	CB	VAL	B	68	27.510	16.580	−43.820	1.00	40.61	C
ATOM	6904	CG1	VAL	B	68	26.430	17.043	−42.770	1.00	40.79	C
ATOM	6905	CG2	VAL	B	68	27.721	17.623	−44.953	1.00	40.78	C
ATOM	6906	HN	VAL	B	68	27.863	14.405	−42.364	1.00	40.71	H
ATOM	6907	HA	VAL	B	68	29.631	16.169	−43.885	1.00	40.71	H
ATOM	6908	HC	VAL	B	68	30.250	17.829	−42.248	1.00	40.71	H
ATOM	6909	HB	VAL	B	68	27.140	15.666	−44.285	1.00	40.71	H
ATOM	6910	1HG1	VAL	B	68	25.500	17.282	−43.286	1.00	40.71	H
ATOM	6911	2HG1	VAL	B	68	26.790	17.927	−42.243	1.00	40.71	H
ATOM	6912	3HG1	VAL	B	68	26.252	16.241	−42.054	1.00	40.71	H
ATOM	6913	1HG2	VAL	B	68	26.764	17.847	−45.424	1.00	40.71	H
ATOM	6914	2HG2	VAL	B	68	28.406	17.218	−45.697	1.00	40.71	H
ATOM	6915	3HG2	VAL	B	68	28.141	18.537	−44.533	1.00	40.71	H
ATOM	6916	N	TYR	B	76	26.885	15.715	−47.918	1.00	28.84	N
ATOM	6917	CA	TYR	B	76	26.954	14.289	−47.549	1.00	28.59	C
ATOM	6918	C	TYR	B	76	26.778	13.367	−48.753	1.00	28.36	C
ATOM	6919	O	TYR	B	76	25.781	13.423	−49.478	1.00	28.34	O
ATOM	6920	CB	TYR	B	76	25.891	13.927	−46.484	1.00	28.58	C
ATOM	6921	CG	TYR	B	76	26.111	12.589	−45.810	1.00	28.37	C

ATOM	6922	CD1	TYR	B	76	27.098	12.448	−44.858	1.00	28.38	C
ATOM	6923	CD2	TYR	B	76	25.339	11.466	−46.126	1.00	28.18	C
ATOM	6924	CE1	TYR	B	76	27.332	11.206	−44.209	1.00	28.18	C
ATOM	6925	CE2	TYR	B	76	25.562	10.209	−45.489	1.00	28.01	C
ATOM	6926	CZ	TYR	B	76	26.575	10.101	−44.518	1.00	28.01	C
ATOM	6927	OH	TYR	B	76	26.874	8.948	−43.825	1.00	27.85	O
ATOM	6928	HN1	TYR	B	76	25.932	15.924	−48.358	1.00	28.31	H
ATOM	6929	HN2	TYR	B	76	27.659	15.940	−48.622	1.00	28.31	H
ATOM	6930	HA	TYR	B	76	27.938	14.099	−47.121	1.00	28.31	H
ATOM	6931	HB1	TYR	B	76	25.900	14.701	−45.716	1.00	28.31	H
ATOM	6932	HB2	TYR	B	76	24.915	13.908	−46.969	1.00	28.31	H
ATOM	6933	HD1	TYR	B	76	27.713	13.309	−44.597	1.00	28.31	H
ATOM	6934	HD2	TYR	B	76	24.551	11.553	−46.874	1.00	28.31	H
ATOM	6935	HE1	TYR	B	76	28.119	11.129	−43.459	1.00	28.31	H
ATOM	6936	HE2	TYR	B	76	24.955	9.343	−45.751	1.00	28.31	H
ATOM	6937	HH	TYR	B	76	27.872	8.677	−44.009	1.00	28.31	H
ATOM	6938	N	GLU	B	77	27.747	12.488	−48.913	1.00	28.22	N
ATOM	6939	CA	GLU	B	77	27.691	11.490	−49.932	1.00	28.02	C
ATOM	6940	C	GLU	B	77	27.014	10.203	−49.386	1.00	27.85	C
ATOM	6941	O	GLU	B	77	27.346	9.738	−48.299	1.00	27.82	O
ATOM	6942	CB	GLU	B	77	29.122	11.235	−50.409	1.00	27.99	C
ATOM	6943	CG	GLU	B	77	29.884	12.493	−50.822	1.00	28.19	C
ATOM	6944	CD	GLU	B	77	31.183	12.183	−51.593	1.00	28.16	C
ATOM	6945	OE1	GLU	B	77	31.202	11.212	−52.382	1.00	27.99	O
ATOM	6946	OE2	GLU	B	77	32.201	12.907	−51.420	1.00	28.32	O
ATOM	6947	HN	GLU	B	77	28.596	12.523	−48.263	1.00	28.06	H
ATOM	6948	HA	GLU	B	77	27.103	11.871	−50.767	1.00	28.06	H

ATOM 6949 HB1 GLU B 77 29.671 10.755 −49.599 1.00 28.06 H

ATOM 6950 HB2 GLU B 77 29.081 10.565 −51.267 1.00 28.06 H

ATOM 6951 HG1 GLU B 77 29.238 13.097 −51.459 1.00 28.06 H

ATOM 6952 HG2 GLU B 77 30.139 13.055 −49.924 1.00 28.06 H

ATOM 6953 N TYR B 78 26.059 9.659 −50.146 1.00 27.75 N

ATOM 6954 CA TYR B 78 25.361 8.406 −49.807 1.00 27.61 C

ATOM 6955 C TYR B 78 25.804 7.144 −50.548 1.00 27.45 C

ATOM 6956 O TYR B 78 25.752 7.071 −51.794 1.00 27.41 O

ATOM 6957 CB TYR B 78 23.878 8.565 −50.068 1.00 27.64 C

ATOM 6958 CG TYR B 78 23.266 9.579 −49.177 1.00 27.81 C

ATOM 6959 CD1 TYR B 78 22.974 9.268 −47.852 1.00 27.84 C

ATOM 6960 CD2 TYR B 78 23.001 10.847 −49.634 1.00 27.96 C

ATOM 6961 CE1 TYR B 78 22.411 10.198 −47.006 1.00 28.03 C

ATOM 6962 CE2 TYR B 78 22.446 11.787 −48.800 1.00 28.15 C

ATOM 6963 CZ TYR B 78 22.141 11.460 −47.486 1.00 28.19 C

ATOM 6964 OH TYR B 78 21.586 12.414 −46.664 1.00 28.41 O

ATOM 6965 HN TYR B 78 25.786 10.161 −51.051 1.00 27.85 H

ATOM 6966 HA TYR B 78 25.494 8.229 −48.740 1.00 27.85 H

ATOM 6967 HB1 TYR B 78 23.734 8.873 −51.104 1.00 27.85 H

ATOM 6968 HB2 TYR B 78 23.386 7.607 −49.901 1.00 27.85 H

ATOM 6969 HD1 TYR B 78 23.195 8.269 −47.476 1.00 27.85 H

ATOM 6970 HD2 TYR B 78 23.233 11.109 −50.666 1.00 27.85 H

ATOM 6971 HE1 TYR B 78 22.183 9.939 −45.972 1.00 27.85 H

ATOM 6972 HE2 TYR B 78 22.245 12.792 −49.171 1.00 27.85 H

ATOM 6973 HH TYR B 78 20.656 12.076 −46.313 1.00 27.85 H

ATOM 6974 N TYR B 79 26.158 6.134 −49.747 1.00 27.37 N

ATOM 6975 CA TYR B 79 26.808 4.931 −50.229 1.00 27.25 C

ATOM	6976	C	TYR	B	79	26.356	3.734	−49.485	1.00	27.17	C
ATOM	6977	O	TYR	B	79	26.253	3.780	−48.266	1.00	27.19	O
ATOM	6978	CB	TYR	B	79	28.324	4.989	−49.956	1.00	27.25	C
ATOM	6979	CG	TYR	B	79	29.076	6.033	−50.732	1.00	27.35	C
ATOM	6980	CD1	TYR	B	79	29.150	5.971	−52.134	1.00	27.32	C
ATOM	6981	CD2	TYR	B	79	29.713	7.085	−50.075	1.00	27.47	C
ATOM	6982	CE1	TYR	B	79	29.842	6.937	−52.861	1.00	27.42	C
ATOM	6983	CE2	TYR	B	79	30.409	8.057	−50.793	1.00	27.58	C
ATOM	6984	CZ	TYR	B	79	30.469	7.978	−52.184	1.00	27.56	C
ATOM	6985	OH	TYR	B	79	31.157	8.938	−52.885	1.00	27.68	O
ATOM	6986	HN	TYR	B	79	25.952	6.217	−48.700	1.00	27.38	H
ATOM	6987	HA	TYR	B	79	26.622	4.804	−51.295	1.00	27.38	H
ATOM	6988	HB1	TYR	B	79	28.468	5.190	−48.894	1.00	27.38	H
ATOM	6989	HB2	TYR	B	79	28.748	4.016	−50.203	1.00	27.38	H
ATOM	6990	HD1	TYR	B	79	28.658	5.154	−52.661	1.00	27.38	H
ATOM	6991	HD2	TYR	B	79	29.666	7.148	−48.988	1.00	27.38	H
ATOM	6992	HE1	TYR	B	79	29.892	6.878	−53.948	1.00	27.38	H
ATOM	6993	HE2	TYR	B	79	30.904	8.875	−50.269	1.00	27.38	H
ATOM	6994	HH	TYR	B	79	31.933	8.486	−53.429	1.00	27.38	H
ATOM	6995	N	LYS	B	80	26.149	2.659	−50.231	1.00	27.09	N
ATOM	6996	CA	LYS	B	80	26.174	1.319	−49.699	1.00	27.03	C
ATOM	6997	C	LYS	B	80	27.419	0.655	−50.288	1.00	26.98	C
ATOM	6998	O	LYS	B	80	27.601	0.670	−51.477	1.00	26.98	O
ATOM	6999	CB	LYS	B	80	24.842	0.610	−50.026	1.00	27.03	C
ATOM	7000	CG	LYS	B	80	23.669	1.005	−49.030	1.00	27.09	C
ATOM	7001	CD	LYS	B	80	22.217	0.665	−49.500	1.00	27.13	C
ATOM	7002	CE	LYS	B	80	21.189	0.605	−48.323	1.00	27.20	C

ATOM	7003	NZ	LYS	B	80	19.899	−0.102	−48.640	1.00	27.24	N1+
ATOM	7004	HN	LYS	B	80	25.955	2.791	−51.275	1.00	27.09	H
ATOM	7005	HA	LYS	B	80	26.285	1.374	−48.616	1.00	27.09	H
ATOM	7006	HB1	LYS	B	80	24.544	0.881	−51.039	1.00	27.09	H
ATOM	7007	HB2	LYS	B	80	24.999	−0.467	−49.969	1.00	27.09	H
ATOM	7008	HG1	LYS	B	80	23.844	0.484	−48.088	1.00	27.09	H
ATOM	7009	HG2	LYS	B	80	23.721	2.081	−48.866	1.00	27.09	H
ATOM	7010	HD1	LYS	B	80	21.893	1.430	−50.205	1.00	27.09	H
ATOM	7011	HD2	LYS	B	80	22.232	−0.305	−49.997	1.00	27.09	H
ATOM	7012	HE1	LYS	B	80	21.661	0.088	−47.488	1.00	27.09	H
ATOM	7013	HE2	LYS	B	80	20.951	1.627	−48.030	1.00	27.09	H
ATOM	7014	HZ1	LYS	B	80	19.265	−0.091	−47.778	1.00	27.09	H
ATOM	7015	HZ2	LYS	B	80	20.106	−1.115	−48.918	1.00	27.09	H
ATOM	7016	HZ3	LYS	B	80	19.408	0.396	−49.450	1.00	27.09	H
ATOM	7017	N	VAL	B	81	28.310	0.147	−49.452	1.00	26.95	N
ATOM	7018	CA	VAL	B	81	29.563	−0.478	−49.918	1.00	26.93	C
ATOM	7019	C	VAL	B	81	29.536	−1.982	−49.674	1.00	26.88	C
ATOM	7020	O	VAL	B	81	29.226	−2.426	−48.581	1.00	26.85	O
ATOM	7021	CB	VAL	B	81	30.832	0.075	−49.197	1.00	26.94	C
ATOM	7022	CG1	VAL	B	81	32.006	−0.859	−49.388	1.00	26.91	C
ATOM	7023	CG2	VAL	B	81	31.208	1.427	−49.707	1.00	27.02	C
ATOM	7024	HN	VAL	B	81	28.115	0.191	−48.401	1.00	26.93	H
ATOM	7025	HA	VAL	B	81	29.665	−0.300	−50.989	1.00	26.93	H
ATOM	7026	HB	VAL	B	81	30.620	0.153	−48.131	1.00	26.93	H
ATOM	7027	1HG1	VAL	B	81	32.880	−0.454	−48.877	1.00	26.93	H
ATOM	7028	2HG1	VAL	B	81	32.222	−0.959	−50.452	1.00	26.93	H
ATOM	7029	3HG1	VAL	B	81	31.764	−1.837	−48.973	1.00	26.93	H

ATOM 7030 1HG2 VAL B 81 32.096 1.780 −49.182 1.00 26.93 H
ATOM 7031 2HG2 VAL B 81 30.386 2.122 −49.537 1.00 26.93 H
ATOM 7032 3HG2 VAL B 81 31.418 1.368 −50.775 1.00 26.93 H
ATOM 7033 N TYR B 82 29.903 −2.765 −50.676 1.00 26.89 N
ATOM 7034 CA TYR B 82 29.839 −4.219 −50.579 1.00 26.89 C
ATOM 7035 C TYR B 82 31.189 −4.854 −50.884 1.00 26.90 C
ATOM 7036 O TYR B 82 31.872 −4.453 −51.833 1.00 26.94 O
ATOM 7037 CB TYR B 82 28.863 −4.811 −51.603 1.00 26.93 C
ATOM 7038 CG TYR B 82 27.402 −4.375 −51.572 1.00 26.93 C
ATOM 7039 CD1 TYR B 82 26.401 −5.303 −51.508 1.00 26.97 C
ATOM 7040 CD2 TYR B 82 27.035 −3.043 −51.676 1.00 26.93 C
ATOM 7041 CE1 TYR B 82 25.104 −4.927 −51.519 1.00 26.99 C
ATOM 7042 CE2 TYR B 82 25.733 −2.661 −51.677 1.00 26.95 C
ATOM 7043 CZ TYR B 82 24.778 −3.604 −51.598 1.00 26.98 C
ATOM 7044 OH TYR B 82 23.468 −3.225 −51.584 1.00 27.02 O
ATOM 7045 HN TYR B 82 30.256 −2.319 −51.582 1.00 26.94 H
ATOM 7046 HA TYR B 82 29.525 −4.501 −49.574 1.00 26.94 H
ATOM 7047 HB1 TYR B 82 29.249 −4.563 −52.592 1.00 26.94 H
ATOM 7048 HB2 TYR B 82 28.876 −5.893 −51.468 1.00 26.94 H
ATOM 7049 HD1 TYR B 82 26.653 −6.362 −51.447 1.00 26.94 H
ATOM 7050 HD2 TYR B 82 27.812 −2.283 −51.759 1.00 26.94 H
ATOM 7051 HE1 TYR B 82 24.318 −5.680 −51.465 1.00 26.94 H
ATOM 7052 HE2 TYR B 82 25.466 −1.606 −51.741 1.00 26.94 H
ATOM 7053 HH TYR B 82 22.969 −3.659 −52.399 1.00 26.94 H
ATOM 7054 N MET B 83 31.555 −5.868 −50.097 1.00 26.89 N
ATOM 7055 CA MET B 83 32.622 −6.796 −50.474 1.00 26.93 C
ATOM 7056 C MET B 83 32.072 −7.588 −51.634 1.00 27.01 C

ATOM 7057 O MET B 83 31.008 −8.192 −51.490 1.00 27.02 O

ATOM 7058 CB MET B 83 32.952 −7.781 −49.335 1.00 26.90 C

ATOM 7059 CG MET B 83 33.591 −9.122 −49.819 1.00 26.98 C

ATOM 7060 SD MET B 83 35.030 −9.728 −48.865 1.00 26.96 S

ATOM 7061 CE MET B 83 35.924 −8.193 −48.537 1.00 26.89 C

ATOM 7062 HN MET B 83 31.053 −6.007 −49.162 1.00 26.95 H

ATOM 7063 HA MET B 83 33.515 −6.248 −50.775 1.00 26.95 H

ATOM 7064 HB1 MET B 83 33.650 −7.295 −48.654 1.00 26.95 H

ATOM 7065 HB2 MET B 83 32.028 −8.013 −48.805 1.00 26.95 H

ATOM 7066 HG1 MET B 83 32.819 −9.891 −49.779 1.00 26.95 H

ATOM 7067 HG2 MET B 83 33.912 −8.983 −50.851 1.00 26.95 H

ATOM 7068 HE1 MET B 83 36.824 −8.412 −47.962 1.00 26.95 H

ATOM 7069 HE2 MET B 83 35.287 −7.515 −47.969 1.00 26.95 H

ATOM 7070 HE3 MET B 83 36.201 −7.725 −49.481 1.00 26.95 H

ATOM 7071 N VAL B 84 32.770 −7.594 −52.766 1.00 27.08 N

ATOM 7072 CA VAL B 84 32.257 −8.270 −53.948 1.00 27.16 C

ATOM 7073 C VAL B 84 33.186 −9.411 −54.253 1.00 27.27 C

ATOM 7074 O VAL B 84 34.019 −9.779 −53.403 1.00 27.26 O

ATOM 7075 CB VAL B 84 32.115 −7.330 −55.181 1.00 27.18 C

ATOM 7076 CG1 VAL B 84 31.337 −6.097 −54.810 1.00 27.09 C

ATOM 7077 CG2 VAL B 84 33.465 −6.958 −55.788 1.00 27.23 C

ATOM 7078 HN VAL B 84 33.717 −7.097 −52.808 1.00 27.18 H

ATOM 7079 HA VAL B 84 31.275 −8.679 −53.712 1.00 27.18 H

ATOM 7080 HB VAL B 84 31.546 −7.865 −55.941 1.00 27.18 H

ATOM 7081 1HG1 VAL B 84 31.245 −5.449 −55.681 1.00 27.18 H

ATOM 7082 2HG1 VAL B 84 31.858 −5.564 −54.014 1.00 27.18 H

ATOM 7083 3HG1 VAL B 84 30.344 −6.384 −54.465 1.00 27.18 H

```
ATOM   7084 1HG2 VAL B  84      33.310  −6.302 −56.644  1.00 27.18           H
ATOM   7085 2HG2 VAL B  84      33.980  −7.862 −56.112  1.00 27.18           H
ATOM   7086 3HG2 VAL B  84      34.070  −6.444 −55.041  1.00 27.18           H
ATOM   7087  N   ASP B  85      32.988 −10.008 −55.431  1.00 52.19           N
ATOM   7088  CA  ASP B  85      34.028 −10.797 −56.071  1.00 52.32           C
ATOM   7089  C   ASP B  85      34.680  −9.954 −57.168  1.00 52.35           C
ATOM   7090  O   ASP B  85      34.316  −8.801 −57.353  1.00 52.27           O
ATOM   7091  CB  ASP B  85      33.451 −12.098 −56.627  1.00 52.47           C
ATOM   7092  CG  ASP B  85      32.658 −11.896 −57.915  1.00 52.51           C
ATOM   7093  OD1 ASP B  85      33.290 −11.658 −58.979  1.00 52.58           O
ATOM   7094  OD2 ASP B  85      31.406 −12.004 −57.860  1.00 52.49           O
ATOM   7095  HN  ASP B  85      32.040  −9.903 −55.916  1.00 52.40           H
ATOM   7096  HA  ASP B  85      34.785 −11.043 −55.326  1.00 52.40           H
ATOM   7097  HB1 ASP B  85      34.273 −12.784 −56.830  1.00 52.40           H
ATOM   7098  HB2 ASP B  85      32.790 −12.533 −55.877  1.00 52.40           H
ATOM   7099  N   LYS B  86      35.640 −10.542 −57.882  1.00 27.68           N
ATOM   7100  CA  LYS B  86      36.378  −9.834 −58.933  1.00 27.74           C
ATOM   7101  C   LYS B  86      35.485  −9.360 −60.051  1.00 27.73           C
ATOM   7102  O   LYS B  86      35.465  −8.165 −60.350  1.00 27.66           O
ATOM   7103  CB  LYS B  86      37.456 −10.717 −59.588  1.00 27.93           C
ATOM   7104  CG  LYS B  86      38.352  −9.929 −60.585  1.00 28.01           C
ATOM   7105  CD  LYS B  86      39.155 −10.818 −61.525  1.00 28.23           C
ATOM   7106  CE  LYS B  86      40.481 −10.173 −61.960  1.00 28.32           C
ATOM   7107  NZ  LYS B  86      40.395  −8.913 −62.776  1.00 28.28           N1+
ATOM   7108  HN  LYS B  86      35.880 −11.565 −57.681  1.00 27.95           H
ATOM   7109  HA  LYS B  86      36.865  −8.966 −58.488  1.00 27.95           H
ATOM   7110  HB1 LYS B  86      38.089 −11.133 −58.804  1.00 27.95           H
```

ATOM 7111 HB2 LYS B 86 36.964 −11.527 −60.127 1.00 27.95 H
ATOM 7112 HG1 LYS B 86 37.712 −9.284 −61.186 1.00 27.95 H
ATOM 7113 HG2 LYS B 86 39.049 −9.318 −60.011 1.00 27.95 H
ATOM 7114 HD1 LYS B 86 39.374 −11.756 −61.016 1.00 27.95 H
ATOM 7115 HD2 LYS B 86 38.556 −11.017 −62.414 1.00 27.95 H
ATOM 7116 HE1 LYS B 86 41.045 −9.941 −61.057 1.00 27.95 H
ATOM 7117 HE2 LYS B 86 41.026 −10.910 −62.550 1.00 27.95 H
ATOM 7118 HZ1 LYS B 86 41.381 −8.571 −63.010 1.00 27.95 H
ATOM 7119 HZ2 LYS B 86 39.880 −8.160 −62.217 1.00 27.95 H
ATOM 7120 HZ3 LYS B 86 39.862 −9.110 −63.683 1.00 27.95 H
ATOM 7121 N ASP B 87 34.787 −10.307 −60.688 1.00 36.30 N
ATOM 7122 CA ASP B 87 33.968 −10.015 −61.865 1.00 36.31 C
ATOM 7123 C ASP B 87 33.058 −8.858 −61.565 1.00 36.13 C
ATOM 7124 O ASP B 87 32.911 −7.951 −62.389 1.00 36.09 O
ATOM 7125 CB ASP B 87 33.091 −11.186 −62.225 1.00 36.41 C
ATOM 7126 CG ASP B 87 33.876 −12.389 −62.560 1.00 36.61 C
ATOM 7127 OD1 ASP B 87 34.908 −12.258 −63.253 1.00 36.71 O
ATOM 7128 OD2 ASP B 87 33.465 −13.476 −62.110 1.00 36.70 O
ATOM 7129 HN ASP B 87 34.829 −11.312 −60.324 1.00 36.41 H
ATOM 7130 HA ASP B 87 34.612 −9.766 −62.708 1.00 36.41 H
ATOM 7131 HB1 ASP B 87 32.445 −11.414 −61.377 1.00 36.41 H
ATOM 7132 HB2 ASP B 87 32.480 −10.915 −63.086 1.00 36.41 H
ATOM 7133 N GLN B 88 32.449 −8.912 −60.376 1.00 44.70 N
ATOM 7134 CA GLN B 88 31.612 −7.824 −59.847 1.00 44.56 C
ATOM 7135 C GLN B 88 32.400 −6.546 −59.687 1.00 44.50 C
ATOM 7136 O GLN B 88 31.902 −5.474 −59.978 1.00 44.43 O
ATOM 7137 CB GLN B 88 30.946 −8.209 −58.513 1.00 44.50 C

ATOM	7138	CG	GLN	B	88	29.632	−9.001	−58.695	1.00	44.55	C
ATOM	7139	CD	GLN	B	88	29.005	−9.526	−57.402	1.00	44.52	C
ATOM	7140	OE1	GLN	B	88	29.701	−9.920	−56.442	1.00	44.52	O
ATOM	7141	NE2	GLN	B	88	27.673	−9.570	−57.389	1.00	44.52	N
ATOM	7142	HN	GLN	B	88	32.577	−9.791	−59.779	1.00	44.53	H
ATOM	7143	HA	GLN	B	88	30.819	−7.635	−60.570	1.00	44.53	H
ATOM	7144	HB1	GLN	B	88	31.643	−8.822	−57.942	1.00	44.53	H
ATOM	7145	HB2	GLN	B	88	30.726	−7.296	−57.959	1.00	44.53	H
ATOM	7146	HG1	GLN	B	88	28.908	−8.346	−59.179	1.00	44.53	H
ATOM	7147	HG2	GLN	B	88	29.839	−9.855	−59.340	1.00	44.53	H
ATOM	7148	1HE2	GLN	B	88	27.119	−9.250	−58.247	1.00	44.53	H
ATOM	7149	2HE2	GLN	B	88	27.158	−9.925	−56.521	1.00	44.53	H
ATOM	7150	N	ALA	B	89	33.630	−6.649	−59.212	1.00	48.80	N
ATOM	7151	CA	ALA	B	89	34.520	−5.487	−59.196	1.00	48.79	C
ATOM	7152	C	ALA	B	89	34.700	−4.925	−60.611	1.00	48.85	C
ATOM	7153	O	ALA	B	89	34.418	−3.747	−60.867	1.00	48.80	O
ATOM	7154	CB	ALA	B	89	35.879	−5.857	−58.590	1.00	48.85	C
ATOM	7155	HN	ALA	B	89	33.977	−7.589	−58.837	1.00	48.82	H
ATOM	7156	HA	ALA	B	89	34.066	−4.715	−58.575	1.00	48.82	H
ATOM	7157	HB1	ALA	B	89	36.527	−4.980	−58.587	1.00	48.82	H
ATOM	7158	HB2	ALA	B	89	36.340	−6.646	−59.184	1.00	48.82	H
ATOM	7159	HB3	ALA	B	89	35.738	−6.208	−57.568	1.00	48.82	H
ATOM	7160	N	ASP	B	90	35.121	−5.807	−61.522	1.00	41.47	N
ATOM	7161	CA	ASP	B	90	35.574	−5.442	−62.865	1.00	41.55	C
ATOM	7162	C	ASP	B	90	34.439	−5.134	−63.805	1.00	41.49	C
ATOM	7163	O	ASP	B	90	34.679	−4.632	−64.893	1.00	41.53	O
ATOM	7164	CB	ASP	B	90	36.451	−6.561	−63.426	1.00	41.71	C

ATOM 7165 CG ASP B 90 37.599 −6.952 −62.458 1.00 41.77 C

ATOM 7166 OD1 ASP B 90 37.807 −6.248 −61.446 1.00 41.68 O

ATOM 7167 OD2 ASP B 90 38.288 −7.968 −62.685 1.00 41.92 O

ATOM 7168 HN ASP B 90 35.127 −6.843 −61.254 1.00 41.64 H

ATOM 7169 HA ASP B 90 36.189 −4.546 −62.779 1.00 41.64 H

ATOM 7170 HB1 ASP B 90 35.829 −7.438 −63.604 1.00 41.64 H

ATOM 7171 HB2 ASP B 90 36.886 −6.226 −64.368 1.00 41.64 H

ATOM 7172 N ARG B 91 33.212 −5.429 −63.372 1.00 48.08 N

ATOM 7173 CA ARG B 91 31.973 −5.028 −64.068 1.00 48.01 C

ATOM 7174 C ARG B 91 31.143 −3.907 −63.350 1.00 47.88 C

ATOM 7175 O ARG B 91 30.234 −3.325 −63.962 1.00 47.82 O

ATOM 7176 CB ARG B 91 31.126 −6.288 −64.322 1.00 48.06 C

ATOM 7177 CG ARG B 91 31.786 −7.226 −65.344 1.00 48.21 C

ATOM 7178 CD ARG B 91 31.404 −8.698 −65.210 1.00 48.33 C

ATOM 7179 NE ARG B 91 32.220 −9.550 −66.097 1.00 48.51 N

ATOM 7180 CZ ARG B 91 32.275 −10.890 −66.077 1.00 48.67 C

ATOM 7181 NH1 ARG B 91 31.566 −11.590 −65.205 1.00 48.68 N

ATOM 7182 NH2 ARG B 91 33.058 −11.546 −66.937 1.00 48.86 N

ATOM 7183 HN ARG B 91 33.120 −5.990 −62.466 1.00 48.28 H

ATOM 7184 HA ARG B 91 32.265 −4.636 −65.042 1.00 48.28 H

ATOM 7185 HB1 ARG B 91 31.001 −6.824 −63.381 1.00 48.28 H

ATOM 7186 HB2 ARG B 91 30.150 −5.986 −64.701 1.00 48.28 H

ATOM 7187 HG1 ARG B 91 31.501 −6.892 −66.342 1.00 48.28 H

ATOM 7188 HG2 ARG B 91 32.867 −7.145 −65.228 1.00 48.28 H

ATOM 7189 HD1 ARG B 91 31.558 −9.011 −64.177 1.00 48.28 H

ATOM 7190 HD2 ARG B 91 30.353 −8.817 −65.473 1.00 48.28 H

ATOM 7191 HE ARG B 91 32.825 −9.051 −66.825 1.00 48.28 H

ATOM 7192 1HH1 ARG B 91 30.936 −11.087 −64.501 1.00 48.28 H
ATOM 7193 2HH1 ARG B 91 31.623 −12.658 −65.204 1.00 48.28 H
ATOM 7194 1HH2 ARG B 91 33.646 −11.003 −67.647 1.00 48.28 H
ATOM 7195 2HH2 ARG B 91 33.098 −12.615 −66.916 1.00 48.28 H
ATOM 7196 N CYS B 92 31.516 −3.589 −62.093 1.00 43.45 N
ATOM 7197 CA CYS B 92 30.774 −2.703 −61.136 1.00 43.36 C
ATOM 7198 C CYS B 92 29.274 −2.990 −60.964 1.00 43.31 C
ATOM 7199 O CYS B 92 28.410 −2.149 −61.224 1.00 43.26 O
ATOM 7200 CB CYS B 92 31.041 −1.233 −61.426 1.00 43.35 C
ATOM 7201 SG CYS B 92 32.793 −0.866 −61.202 1.00 43.44 S
ATOM 7202 HN CYS B 92 32.435 −4.012 −61.743 1.00 43.36 H
ATOM 7203 HA CYS B 92 31.220 −2.892 −60.160 1.00 43.36 H
ATOM 7204 HB1 CYS B 92 30.755 −1.011 −62.454 1.00 43.36 H
ATOM 7205 HB2 CYS B 92 30.454 −0.618 −60.744 1.00 43.36 H
ATOM 7206 N THR B 93 29.027 −4.205 −60.477 1.00 27.78 N
ATOM 7207 CA THR B 93 27.717 −4.822 −60.331 1.00 27.78 C
ATOM 7208 C THR B 93 27.638 −5.485 −58.930 1.00 27.78 C
ATOM 7209 O THR B 93 28.583 −6.129 −58.463 1.00 27.82 O
ATOM 7210 CB THR B 93 27.469 −5.842 −61.525 1.00 27.86 C
ATOM 7211 OG1 THR B 93 28.150 −7.096 −61.336 1.00 27.96 O
ATOM 7212 CG2 THR B 93 27.974 −5.244 −62.821 1.00 27.86 C
ATOM 7213 HN THR B 93 29.872 −4.782 −60.163 1.00 27.83 H
ATOM 7214 HA THR B 93 26.960 −4.039 −60.384 1.00 27.83 H
ATOM 7215 HB THR B 93 26.399 −6.030 −61.614 1.00 27.83 H
ATOM 7216 HG1 THR B 93 29.184 −6.927 −61.268 1.00 27.83 H
ATOM 7217 1HG2 THR B 93 27.802 −5.946 −63.637 1.00 27.83 H
ATOM 7218 2HG2 THR B 93 29.041 −5.040 −62.736 1.00 27.83 H

ATOM	7219	3HG2	THR	B	93	27.442	−4.314	−63.024	1.00	27.83	H
ATOM	7220	N	ILE	B	94	26.518	−5.291	−58.249	1.00	27.11	N
ATOM	7221	CA	ILE	B	94	26.222	−5.975	−56.974	1.00	27.12	C
ATOM	7222	C	ILE	B	94	24.857	−6.686	−57.088	1.00	27.17	C
ATOM	7223	O	ILE	B	94	23.842	−6.075	−57.415	1.00	27.15	O
ATOM	7224	CB	ILE	B	94	26.177	−4.993	−55.799	1.00	27.05	C
ATOM	7225	CG1	ILE	B	94	25.569	−3.656	−56.275	1.00	27.01	C
ATOM	7226	CG2	ILE	B	94	27.559	−4.836	−55.219	1.00	27.02	C
ATOM	7227	CD1	ILE	B	94	24.892	−2.838	−55.250	1.00	26.99	C
ATOM	7228	HN	ILE	B	94	25.795	−4.607	−58.641	1.00	27.08	H
ATOM	7229	HA	ILE	B	94	26.995	−6.720	−56.784	1.00	27.08	H
ATOM	7230	HB	ILE	B	94	25.528	−5.410	−55.029	1.00	27.08	H
ATOM	7231	1HG1	ILE	B	94	26.376	−3.057	−56.697	1.00	27.08	H
ATOM	7232	2HG1	ILE	B	94	24.840	−3.882	−57.053	1.00	27.08	H
ATOM	7233	1HG2	ILE	B	94	27.526	−4.137	−54.383	1.00	27.08	H
ATOM	7234	2HG2	ILE	B	94	28.234	−4.454	−55.985	1.00	27.08	H
ATOM	7235	3HG2	ILE	B	94	27.919	−5.804	−54.869	1.00	27.08	H
ATOM	7236	1HD1	ILE	B	94	24.508	−1.926	−55.707	1.00	27.08	H
ATOM	7237	2HD1	ILE	B	94	25.602	−2.579	−54.464	1.00	27.08	H
ATOM	7238	3HD1	ILE	B	94	24.066	−3.404	−54.820	1.00	27.08	H
ATOM	7239	N	LYS	B	95	24.809	−7.977	−56.814	1.00	27.26	N
ATOM	7240	CA	LYS	B	95	23.609	−8.713	−57.135	1.00	27.35	C
ATOM	7241	C	LYS	B	95	22.760	−8.879	−55.916	1.00	27.36	C
ATOM	7242	O	LYS	B	95	23.268	−8.822	−54.795	1.00	27.32	O
ATOM	7243	CB	LYS	B	95	23.968	−10.019	−57.773	1.00	27.48	C
ATOM	7244	CG	LYS	B	95	24.696	−9.785	−59.115	1.00	27.48	C
ATOM	7245	CD	LYS	B	95	23.754	−9.478	−60.311	1.00	27.49	C

ATOM	7246	CE	LYS	B	95	24.484	−8.685	−61.374	1.00	27.43	C
ATOM	7247	NZ	LYS	B	95	25.981	−8.812	−61.272	1.00	27.44	N1+
ATOM	7248	HN	LYS	B	95	25.645	−8.469	−56.362	1.00	27.40	H
ATOM	7249	HA	LYS	B	95	23.042	−8.130	−57.861	1.00	27.40	H
ATOM	7250	HB1	LYS	B	95	24.622	−10.577	−57.103	1.00	27.40	H
ATOM	7251	HB2	LYS	B	95	23.058	−10.591	−57.954	1.00	27.40	H
ATOM	7252	HG1	LYS	B	95	25.376	−8.942	−58.991	1.00	27.40	H
ATOM	7253	HG2	LYS	B	95	25.268	−10.682	−59.353	1.00	27.40	H
ATOM	7254	HD1	LYS	B	95	23.404	−10.416	−60.741	1.00	27.40	H
ATOM	7255	HD2	LYS	B	95	22.901	−8.900	−59.956	1.00	27.40	H
ATOM	7256	HE1	LYS	B	95	24.170	−9.045	−62.354	1.00	27.40	H
ATOM	7257	HE2	LYS	B	95	24.216	−7.634	−61.270	1.00	27.40	H
ATOM	7258	HZ1	LYS	B	95	26.442	−8.233	−62.045	1.00	27.40	H
ATOM	7259	HZ2	LYS	B	95	26.261	−9.839	−61.381	1.00	27.40	H
ATOM	7260	HZ3	LYS	B	95	26.306	−8.454	−60.317	1.00	27.40	H
ATOM	7261	N	LYS	B	96	21.457	−9.033	−56.139	1.00	27.43	N
ATOM	7262	CA	LYS	B	96	20.456	−8.973	−55.059	1.00	27.46	C
ATOM	7263	C	LYS	B	96	20.928	−9.637	−53.746	1.00	27.47	C
ATOM	7264	O	LYS	B	96	20.911	−8.992	−52.671	1.00	27.40	O
ATOM	7265	CB	LYS	B	96	19.104	−9.541	−55.524	1.00	27.59	C
ATOM	7266	CG	LYS	B	96	19.138	−10.998	−56.027	1.00	27.75	C
ATOM	7267	CD	LYS	B	96	18.604	−12.020	−54.992	1.00	27.88	C
ATOM	7268	CE	LYS	B	96	19.008	−13.448	−55.356	1.00	28.05	C
ATOM	7269	NZ	LYS	B	96	20.498	−13.642	−55.323	1.00	27.98	N1+
ATOM	7270	HN	LYS	B	96	21.123	−9.207	−57.141	1.00	27.67	H
ATOM	7271	HA	LYS	B	96	20.295	−7.918	−54.838	1.00	27.67	H
ATOM	7272	HB1	LYS	B	96	18.411	−9.491	−54.684	1.00	27.67	H

ATOM	7273	HB2	LYS	B	96	18.737	−8.913	−56.336	1.00	27.67	H
ATOM	7274	HG1	LYS	B	96	18.527	−11.067	−56.927	1.00	27.67	H
ATOM	7275	HG2	LYS	B	96	20.169	−11.256	−56.267	1.00	27.67	H
ATOM	7276	HD1	LYS	B	96	19.011	−11.776	−54.011	1.00	27.67	H
ATOM	7277	HD2	LYS	B	96	17.516	−11.957	−54.961	1.00	27.67	H
ATOM	7278	HE1	LYS	B	96	18.549	−14.135	−54.645	1.00	27.67	H
ATOM	7279	HE2	LYS	B	96	18.647	−13.670	−56.360	1.00	27.67	H
ATOM	7280	HZ1	LYS	B	96	20.732	−14.653	−55.584	1.00	27.67	H
ATOM	7281	HZ2	LYS	B	96	20.864	−13.434	−54.339	1.00	27.67	H
ATOM	7282	HZ3	LYS	B	96	20.960	−12.977	−56.023	1.00	27.67	H
ATOM	7283	N	GLU	B	97	21.397	−10.889	−53.848	1.00	44.12	N
ATOM	7284	CA	GLU	B	97	21.974	−11.627	−52.705	1.00	44.13	C
ATOM	7285	C	GLU	B	97	22.855	−10.710	−51.857	1.00	43.98	C
ATOM	7286	O	GLU	B	97	22.878	−10.808	−50.627	1.00	43.95	O
ATOM	7287	CB	GLU	B	97	22.837	−12.802	−53.214	1.00	44.23	C
ATOM	7288	CG	GLU	B	97	23.311	−13.798	−52.137	1.00	44.28	C
ATOM	7289	CD	GLU	B	97	24.674	−14.427	−52.459	1.00	44.30	C
ATOM	7290	OE1	GLU	B	97	25.306	−14.970	−51.520	1.00	44.29	O
ATOM	7291	OE2	GLU	B	97	25.120	−14.375	−53.631	1.00	44.34	O
ATOM	7292	HN	GLU	B	97	21.351	−11.376	−54.800	1.00	44.18	H
ATOM	7293	HA	GLU	B	97	21.166	−12.018	−52.087	1.00	44.18	H
ATOM	7294	HB1	GLU	B	97	22.250	−13.356	−53.947	1.00	44.18	H
ATOM	7295	HB2	GLU	B	97	23.721	−12.384	−53.696	1.00	44.18	H
ATOM	7296	HG1	GLU	B	97	23.388	−13.271	−51.186	1.00	44.18	H
ATOM	7297	HG2	GLU	B	97	22.572	−14.595	−52.052	1.00	44.18	H
ATOM	7298	N	ASN	B	98	23.570	−9.814	−52.530	1.00	27.33	N
ATOM	7299	CA	ASN	B	98	24.638	−9.116	−51.905	1.00	27.21	C

ATOM	7300	C	ASN	B	98	24.078	−8.285	−50.772	1.00	27.15	C
ATOM	7301	O	ASN	B	98	23.067	−7.588	−50.943	1.00	27.17	O
ATOM	7302	CB	ASN	B	98	25.443	−8.322	−52.936	1.00	27.17	C
ATOM	7303	CG	ASN	B	98	26.585	−9.150	−53.560	1.00	27.21	C
ATOM	7304	OD1	ASN	B	98	27.100	−8.818	−54.623	1.00	27.22	O
ATOM	7305	ND2	ASN	B	98	26.998	−10.212	−52.873	1.00	27.26	N
ATOM	7306	HN	ASN	B	98	23.338	−9.616	−53.556	1.00	27.21	H
ATOM	7307	HA	ASN	B	98	25.305	−9.859	−51.469	1.00	27.21	H
ATOM	7308	HB1	ASN	B	98	24.771	−8.000	−53.731	1.00	27.22	H
ATOM	7309	HB2	ASN	B	98	25.873	−7.448	−52.446	1.00	27.22	H
ATOM	7310	1HD2	ASN	B	98	26.541	−10.454	−51.936	1.00	27.21	H
ATOM	7311	2HD2	ASN	B	98	27.787	−10.824	−53.257	1.00	27.21	H
ATOM	7312	N	THR	B	99	24.702	−8.468	−49.599	1.00	29.42	N
ATOM	7313	CA	THR	B	99	24.393	−7.738	−48.378	1.00	29.37	C
ATOM	7314	C	THR	B	99	25.587	−6.801	−48.164	1.00	29.27	C
ATOM	7315	O	THR	B	99	26.727	−7.221	−48.386	1.00	29.25	O
ATOM	7316	CB	THR	B	99	24.188	−8.698	−47.187	1.00	29.39	C
ATOM	7317	OG1	THR	B	99	23.068	−9.558	−47.452	1.00	29.51	O
ATOM	7318	CG2	THR	B	99	23.932	−7.927	−45.885	1.00	29.34	C
ATOM	7319	HN	THR	B	99	25.480	−9.201	−49.558	1.00	29.36	H
ATOM	7320	HA	THR	B	99	23.490	−7.147	−48.527	1.00	29.36	H
ATOM	7321	HB	THR	B	99	25.083	−9.309	−47.067	1.00	29.36	H
ATOM	7322	HG1	THR	B	99	22.204	−8.977	−47.585	1.00	29.36	H
ATOM	7323	1HG2	THR	B	99	23.792	−8.632	−45.066	1.00	29.36	H
ATOM	7324	2HG2	THR	B	99	23.036	−7.315	−45.994	1.00	29.36	H
ATOM	7325	3HG2	THR	B	99	24.786	−7.285	−45.669	1.00	29.36	H
ATOM	7326	N	PRO	B	100	25.327	−5.517	−47.800	1.00	44.48	N

ATOM	7327	CA	PRO	B	100	26.378	−4.505	−47.635	1.00	44.42	C
ATOM	7328	C	PRO	B	100	27.268	−4.634	−46.384	1.00	44.36	C
ATOM	7329	O	PRO	B	100	26.889	−5.280	−45.379	1.00	44.35	O
ATOM	7330	CB	PRO	B	100	25.585	−3.182	−47.546	1.00	44.45	C
ATOM	7331	CG	PRO	B	100	24.201	−3.496	−47.904	1.00	44.51	C
ATOM	7332	CD	PRO	B	100	24.000	−4.921	−47.565	1.00	44.53	C
ATOM	7333	HA	PRO	B	100	27.007	−4.490	−48.525	1.00	44.44	H
ATOM	7334	HB1	PRO	B	100	25.630	−2.785	−46.532	1.00	44.44	H
ATOM	7335	HB2	PRO	B	100	25.996	−2.450	−48.242	1.00	44.44	H
ATOM	7336	HG1	PRO	B	100	23.513	−2.872	−47.334	1.00	44.44	H
ATOM	7337	HG2	PRO	B	100	24.041	−3.335	−48.970	1.00	44.44	H
ATOM	7338	HD1	PRO	B	100	23.700	−5.036	−46.523	1.00	44.44	H
ATOM	7339	HD2	PRO	B	100	23.249	−5.374	−48.212	1.00	44.44	H
ATOM	7340	N	LEU	B	101	28.444	−4.015	−46.449	1.00	37.24	N
ATOM	7341	CA	LEU	B	101	29.297	−3.911	−45.287	1.00	37.19	C
ATOM	7342	C	LEU	B	101	29.244	−2.485	−44.733	1.00	37.21	C
ATOM	7343	O	LEU	B	101	29.488	−2.277	−43.521	1.00	37.18	O
ATOM	7344	CB	LEU	B	101	30.722	−4.352	−45.614	1.00	37.16	C
ATOM	7345	CG	LEU	B	101	31.497	−3.565	−46.665	1.00	37.20	C
ATOM	7346	CD1	LEU	B	101	32.085	−2.277	−46.100	1.00	37.22	C
ATOM	7347	CD2	LEU	B	101	32.595	−4.453	−47.287	1.00	37.21	C
ATOM	7348	HN	LEU	B	101	28.763	−3.587	−47.376	1.00	37.20	H
ATOM	7349	HA	LEU	B	101	28.905	−4.582	−44.523	1.00	37.20	H
ATOM	7350	HB1	LEU	B	101	31.295	−4.305	−44.688	1.00	37.20	H
ATOM	7351	HB2	LEU	B	101	30.670	−5.385	−45.958	1.00	37.20	H
ATOM	7352	HG	LEU	B	101	30.800	−3.293	−47.458	1.00	37.20	H
ATOM	7353	1HD1	LEU	B	101	32.628	−1.750	−46.885	1.00	37.20	H

ATOM	7354	2HD1	LEU	B	101	32.767	−2.516	−45.284	1.00	37.20	H
ATOM	7355	3HD1	LEU	B	101	31.281	−1.643	−45.727	1.00	37.20	H
ATOM	7356	1HD2	LEU	B	101	33.143	−3.882	−48.037	1.00	37.20	H
ATOM	7357	2HD2	LEU	B	101	32.136	−5.323	−47.757	1.00	37.20	H
ATOM	7358	3HD2	LEU	B	101	33.282	−4.782	−46.507	1.00	37.20	H
ATOM	7359	N	LEU	B	102	28.897	−1.510	−45.574	1.00	26.82	N
ATOM	7360	CA	LEU	B	102	28.701	−0.169	−45.065	1.00	26.88	C
ATOM	7361	C	LEU	B	102	27.486	0.515	−45.675	1.00	26.95	C
ATOM	7362	O	LEU	B	102	27.372	0.625	−46.879	1.00	26.97	O
ATOM	7363	CB	LEU	B	102	29.983	0.650	−45.203	1.00	26.91	C
ATOM	7364	CG	LEU	B	102	30.919	0.589	−43.973	1.00	26.87	C
ATOM	7365	CD1	LEU	B	102	32.101	1.552	−44.059	1.00	26.94	C
ATOM	7366	CD2	LEU	B	102	30.162	0.893	−42.694	1.00	26.88	C
ATOM	7367	HN	LEU	B	102	28.765	−1.717	−46.615	1.00	26.90	H
ATOM	7368	HA	LEU	B	102	28.504	−0.265	−43.997	1.00	26.90	H
ATOM	7369	HB1	LEU	B	102	30.533	0.278	−46.068	1.00	26.90	H
ATOM	7370	HB2	LEU	B	102	29.706	1.691	−45.368	1.00	26.90	H
ATOM	7371	HG	LEU	B	102	31.314	−0.424	−43.899	1.00	26.90	H
ATOM	7372	1HD1	LEU	B	102	32.715	1.456	−43.163	1.00	26.90	H
ATOM	7373	2HD1	LEU	B	102	31.732	2.575	−44.138	1.00	26.90	H
ATOM	7374	3HD1	LEU	B	102	32.701	1.314	−44.937	1.00	26.90	H
ATOM	7375	1HD2	LEU	B	102	30.845	0.843	−41.846	1.00	26.90	H
ATOM	7376	2HD2	LEU	B	102	29.364	0.162	−42.561	1.00	26.90	H
ATOM	7377	3HD2	LEU	B	102	29.732	1.893	−42.755	1.00	26.90	H
ATOM	7378	N	ASN	B	103	26.572	0.935	−44.802	1.00	31.56	N
ATOM	7379	CA	ASN	B	103	25.335	1.645	−45.172	1.00	31.65	C
ATOM	7380	C	ASN	B	103	25.491	3.107	−44.751	1.00	31.75	C

ATOM	7381	O	ASN	B	103	25.092	3.485	−43.623	1.00	31.82	O
ATOM	7382	CB	ASN	B	103	24.125	0.999	−44.446	1.00	31.67	C
ATOM	7383	CG	ASN	B	103	22.739	1.443	−44.987	1.00	31.76	C
ATOM	7384	OD1	ASN	B	103	22.698	2.326	−45.852	1.00	31.81	O
ATOM	7385	ND2	ASN	B	103	21.630	0.865	−44.487	1.00	31.81	N
ATOM	7386	HN	ASN	B	103	26.748	0.744	−43.764	1.00	31.73	H
ATOM	7387	HA	ASN	B	103	25.188	1.588	−46.251	1.00	31.73	H
ATOM	7388	HB1	ASN	B	103	24.202	−0.083	−44.553	1.00	31.73	H
ATOM	7389	HB2	ASN	B	103	24.180	1.265	−43.390	1.00	31.73	H
ATOM	7390	1HD2	ASN	B	103	21.713	0.113	−43.730	1.00	31.73	H
ATOM	7391	2HD2	ASN	B	103	20.666	1.157	−44.847	1.00	31.73	H
ATOM	7392	N	CYS	B	104	26.070	3.920	−45.645	1.00	27.24	N
ATOM	7393	CA	CYS	B	104	26.423	5.287	−45.312	1.00	27.37	C
ATOM	7394	C	CYS	B	104	25.285	6.213	−45.682	1.00	27.49	C
ATOM	7395	O	CYS	B	104	25.183	6.712	−46.788	1.00	27.52	O
ATOM	7396	CB	CYS	B	104	27.785	5.648	−45.898	1.00	27.37	C
ATOM	7397	SG	CYS	B	104	29.073	4.868	−44.831	1.00	27.28	S
ATOM	7398	HN	CYS	B	104	26.277	3.552	−46.628	1.00	27.38	H
ATOM	7399	HA	CYS	B	104	26.525	5.331	−44.228	1.00	27.38	H
ATOM	7400	HB1	CYS	B	104	27.865	5.267	−46.916	1.00	27.38	H
ATOM	7401	HB2	CYS	B	104	27.912	6.731	−45.904	1.00	27.38	H
ATOM	7402	N	ALA	B	105	24.413	6.383	−44.697	1.00	27.56	N
ATOM	7403	CA	ALA	B	105	23.171	7.088	−44.833	1.00	27.69	C
ATOM	7404	C	ALA	B	105	22.866	7.758	−43.513	1.00	27.84	C
ATOM	7405	O	ALA	B	105	21.708	7.772	−43.085	1.00	27.92	O
ATOM	7406	CB	ALA	B	105	22.076	6.114	−45.182	1.00	27.62	C
ATOM	7407	HN	ALA	B	105	24.651	5.968	−43.740	1.00	27.73	H

ATOM	7408	HA	ALA	B	105	23.258	7.841	−45.617	1.00	27.73	H
ATOM	7409	HB1	ALA	B	105	21.132	6.649	−45.285	1.00	27.73	H
ATOM	7410	HB2	ALA	B	105	21.986	5.369	−44.391	1.00	27.73	H
ATOM	7411	HB3	ALA	B	105	22.317	5.618	−46.122	1.00	27.73	H
ATOM	7412	N	LYS	B	106	23.896	8.309	−42.884	1.00	27.89	N
ATOM	7413	CA	LYS	B	106	23.766	9.126	−41.675	1.00	28.06	C
ATOM	7414	C	LYS	B	106	24.534	10.456	−41.733	1.00	28.23	C
ATOM	7415	O	LYS	B	106	25.645	10.582	−41.307	1.00	28.22	O
ATOM	7416	CB	LYS	B	106	24.095	8.321	−40.429	1.00	27.96	C
ATOM	7417	CG	LYS	B	106	23.065	7.303	−40.146	1.00	27.89	C
ATOM	7418	CD	LYS	B	106	23.111	6.860	−38.743	1.00	27.87	C
ATOM	7419	CE	LYS	B	106	22.369	5.555	−38.534	1.00	27.76	C
ATOM	7420	NZ	LYS	B	106	22.947	4.732	−37.446	1.00	27.64	N1+
ATOM	7421	HN	LYS	B	106	24.876	8.148	−43.282	1.00	27.95	H
ATOM	7422	HA	LYS	B	106	22.711	9.388	−41.597	1.00	27.95	H
ATOM	7423	HB1	LYS	B	106	25.053	7.822	−40.574	1.00	27.95	H
ATOM	7424	HB2	LYS	B	106	24.163	8.999	−39.579	1.00	27.95	H
ATOM	7425	HG1	LYS	B	106	22.082	7.728	−40.350	1.00	27.95	H
ATOM	7426	HG2	LYS	B	106	23.231	6.442	−40.794	1.00	27.95	H
ATOM	7427	HD1	LYS	B	106	24.152	6.723	−38.452	1.00	27.95	H
ATOM	7428	HD2	LYS	B	106	22.656	7.627	−38.117	1.00	27.95	H
ATOM	7429	HE1	LYS	B	106	21.332	5.780	−38.286	1.00	27.95	H
ATOM	7430	HE2	LYS	B	106	22.405	4.982	−39.460	1.00	27.95	H
ATOM	7431	HZ1	LYS	B	106	22.385	3.827	−37.345	1.00	27.95	H
ATOM	7432	HZ2	LYS	B	106	22.910	5.279	−36.527	1.00	27.95	H
ATOM	7433	HZ3	LYS	B	106	23.964	4.495	−37.679	1.00	27.95	H
ATOM	7434	N	PRO	B	107	23.912	11.458	−42.302	1.00	42.84	N

ATOM 7435 CA PRO B 107 24.602 12.714 −42.499 1.00 43.03 C
ATOM 7436 C PRO B 107 25.472 13.117 −41.326 1.00 43.14 C
ATOM 7437 O PRO B 107 26.635 13.435 −41.539 1.00 43.16 O
ATOM 7438 CB PRO B 107 23.450 13.707 −42.732 1.00 43.25 C
ATOM 7439 CG PRO B 107 22.447 12.894 −43.479 1.00 43.09 C
ATOM 7440 CD PRO B 107 22.539 11.509 −42.856 1.00 42.88 C
ATOM 7441 HA PRO B 107 25.214 12.653 −43.399 1.00 43.06 H
ATOM 7442 HB1 PRO B 107 23.041 14.060 −41.785 1.00 43.06 H
ATOM 7443 HB2 PRO B 107 23.782 14.559 −43.325 1.00 43.06 H
ATOM 7444 HG1 PRO B 107 21.446 13.307 −43.354 1.00 43.06 H
ATOM 7445 HG2 PRO B 107 22.695 12.856 −44.540 1.00 43.06 H
ATOM 7446 HD1 PRO B 107 21.798 11.389 −42.066 1.00 43.06 H
ATOM 7447 HD2 PRO B 107 22.396 10.735 −43.610 1.00 43.06 H
ATOM 7448 N ASP B 108 24.930 13.062 −40.110 1.00 56.47 N
ATOM 7449 CA ASP B 108 25.598 13.682 −38.932 1.00 56.63 C
ATOM 7450 C ASP B 108 26.256 12.670 −37.997 1.00 56.44 C
ATOM 7451 O ASP B 108 26.455 12.945 −36.792 1.00 56.54 O
ATOM 7452 CB ASP B 108 24.607 14.565 −38.145 1.00 56.90 C
ATOM 7453 CG ASP B 108 23.379 13.805 −37.695 1.00 56.83 C
ATOM 7454 OD1 ASP B 108 23.545 12.621 −37.321 1.00 56.59 O
ATOM 7455 OD2 ASP B 108 22.263 14.388 −37.737 1.00 57.02 O
ATOM 7456 HN ASP B 108 23.994 12.561 −39.977 1.00 56.68 H
ATOM 7457 HA ASP B 108 26.385 14.334 −39.311 1.00 56.68 H
ATOM 7458 HB1 ASP B 108 25.115 14.960 −37.265 1.00 56.68 H
ATOM 7459 HB2 ASP B 108 24.292 15.390 −38.784 1.00 56.68 H
ATOM 7460 N GLN B 109 26.587 11.510 −38.567 1.00 28.46 N
ATOM 7461 CA GLN B 109 27.214 10.437 −37.820 1.00 28.26 C

ATOM	7462	C	GLN	B	109	28.320	9.709	−38.630	1.00	28.07	C
ATOM	7463	O	GLN	B	109	28.089	9.243	−39.753	1.00	27.97	O
ATOM	7464	CB	GLN	B	109	26.129	9.478	−37.338	1.00	28.15	C
ATOM	7465	CG	GLN	B	109	26.335	8.989	−35.918	1.00	28.09	C
ATOM	7466	CD	GLN	B	109	25.492	7.768	−35.618	1.00	27.94	C
ATOM	7467	OE1	GLN	B	109	26.009	6.645	−35.580	1.00	27.73	O
ATOM	7468	NE2	GLN	B	109	24.181	7.972	−35.438	1.00	28.07	N
ATOM	7469	HN	GLN	B	109	26.383	11.367	−39.608	1.00	28.08	H
ATOM	7470	HA	GLN	B	109	27.683	10.876	−36.940	1.00	28.08	H
ATOM	7471	HB1	GLN	B	109	25.169	9.991	−37.389	1.00	28.08	H
ATOM	7472	HB2	GLN	B	109	26.115	8.613	−38.001	1.00	28.08	H
ATOM	7473	HG1	GLN	B	109	27.386	8.735	−35.781	1.00	28.08	H
ATOM	7474	HG2	GLN	B	109	26.061	9.786	−35.227	1.00	28.08	H
ATOM	7475	1HE2	GLN	B	109	23.779	8.961	−35.509	1.00	28.08	H
ATOM	7476	2HE2	GLN	B	109	23.536	7.146	−35.223	1.00	28.08	H
ATOM	7477	N	ASP	B	110	29.524	9.659	−38.046	1.00	34.03	N
ATOM	7478	CA	ASP	B	110	30.616	8.798	−38.521	1.00	33.85	C
ATOM	7479	C	ASP	B	110	30.165	7.326	−38.601	1.00	33.61	C
ATOM	7480	O	ASP	B	110	29.941	6.671	−37.566	1.00	33.52	O
ATOM	7481	CB	ASP	B	110	31.813	8.855	−37.555	1.00	33.84	C
ATOM	7482	CG	ASP	B	110	32.551	10.177	−37.581	1.00	34.08	C
ATOM	7483	OD1	ASP	B	110	32.719	10.757	−38.668	1.00	34.18	O
ATOM	7484	OD2	ASP	B	110	33.011	10.628	−36.517	1.00	34.18	O
ATOM	7485	HN	ASP	B	110	29.700	10.277	−37.190	1.00	33.91	H
ATOM	7486	HA	ASP	B	110	30.934	9.132	−39.509	1.00	33.91	H
ATOM	7487	HB1	ASP	B	110	31.447	8.685	−36.543	1.00	33.91	H
ATOM	7488	HB2	ASP	B	110	32.513	8.065	−37.827	1.00	33.91	H

ATOM 7489 N ILE B 111 30.031 6.803 −39.819 1.00 27.53 N

ATOM 7490 CA ILE B 111 29.692 5.394 −40.008 1.00 27.34 C

ATOM 7491 C ILE B 111 30.946 4.673 −40.400 1.00 27.23 C

ATOM 7492 O ILE B 111 31.517 4.953 −41.433 1.00 27.26 O

ATOM 7493 CB ILE B 111 28.599 5.197 −41.064 1.00 27.34 C

ATOM 7494 CG1 ILE B 111 27.233 5.383 −40.394 1.00 27.42 C

ATOM 7495 CG2 ILE B 111 28.700 3.821 −41.704 1.00 27.18 C

ATOM 7496 CD1 ILE B 111 26.054 5.392 −41.348 1.00 27.46 C

ATOM 7497 HN ILE B 111 30.174 7.427 −40.676 1.00 27.35 H

ATOM 7498 HA ILE B 111 29.338 4.990 −39.060 1.00 27.35 H

ATOM 7499 HB ILE B 111 28.717 5.955 −41.838 1.00 27.35 H

ATOM 7500 1HG1 ILE B 111 27.089 4.568 −39.685 1.00 27.35 H

ATOM 7501 2HG1 ILE B 111 27.244 6.333 −39.859 1.00 27.35 H

ATOM 7502 1HG2 ILE B 111 27.913 3.708 −42.449 1.00 27.35 H

ATOM 7503 2HG2 ILE B 111 28.587 3.054 −40.938 1.00 27.35 H

ATOM 7504 3HG2 ILE B 111 29.673 3.714 −42.184 1.00 27.35 H

ATOM 7505 1HD1 ILE B 111 25.131 5.529 −40.785 1.00 27.35 H

ATOM 7506 2HD1 ILE B 111 26.014 4.445 −41.886 1.00 27.35 H

ATOM 7507 3HD1 ILE B 111 26.169 6.209 −42.060 1.00 27.35 H

ATOM 7508 N LYS B 112 31.350 3.739 −39.544 1.00 27.10 N

ATOM 7509 CA LYS B 112 32.675 3.093 −39.596 1.00 27.00 C

ATOM 7510 C LYS B 112 32.590 1.536 −39.515 1.00 26.83 C

ATOM 7511 O LYS B 112 31.808 0.972 −38.714 1.00 26.77 O

ATOM 7512 CB LYS B 112 33.566 3.648 −38.458 1.00 27.03 C

ATOM 7513 CG LYS B 112 32.864 3.746 −37.080 1.00 27.03 C

ATOM 7514 CD LYS B 112 33.839 4.056 −35.958 1.00 27.04 C

ATOM 7515 CE LYS B 112 34.690 2.839 −35.564 1.00 26.85 C

ATOM	7516	NZ	LYS	B	112	33.987	1.903	−34.612	1.00	26.71	N1+
ATOM	7517	HN	LYS	B	112	30.674	3.438	−38.771	1.00	26.93	H
ATOM	7518	HA	LYS	B	112	33.140	3.356	−40.546	1.00	26.93	H
ATOM	7519	HB1	LYS	B	112	34.431	2.993	−38.353	1.00	26.93	H
ATOM	7520	HB2	LYS	B	112	33.898	4.647	−38.742	1.00	26.93	H
ATOM	7521	HG1	LYS	B	112	32.116	4.538	−37.123	1.00	26.93	H
ATOM	7522	HG2	LYS	B	112	32.375	2.795	−36.867	1.00	26.93	H
ATOM	7523	HD1	LYS	B	112	34.504	4.856	−36.284	1.00	26.93	H
ATOM	7524	HD2	LYS	B	112	33.274	4.385	−35.085	1.00	26.93	H
ATOM	7525	HE1	LYS	B	112	34.943	2.286	−36.469	1.00	26.93	H
ATOM	7526	HE2	LYS	B	112	35.603	3.195	−35.088	1.00	26.93	H
ATOM	7527	HZ1	LYS	B	112	34.631	1.081	−34.379	1.00	26.93	H
ATOM	7528	HZ2	LYS	B	112	33.091	1.536	−35.068	1.00	26.93	H
ATOM	7529	HZ3	LYS	B	112	33.739	2.428	−33.713	1.00	26.93	H
ATOM	7530	N	PHE	B	113	33.395	0.851	−40.334	1.00	26.78	N
ATOM	7531	CA	PHE	B	113	33.426	−0.613	−40.325	1.00	26.65	C
ATOM	7532	C	PHE	B	113	34.867	−1.117	−40.165	1.00	26.59	C
ATOM	7533	O	PHE	B	113	35.773	−0.642	−40.864	1.00	26.66	O
ATOM	7534	CB	PHE	B	113	32.774	−1.179	−41.592	1.00	26.66	C
ATOM	7535	CG	PHE	B	113	33.201	−2.580	−41.905	1.00	26.59	C
ATOM	7536	CD1	PHE	B	113	33.104	−3.580	−40.939	1.00	26.50	C
ATOM	7537	CD2	PHE	B	113	33.741	−2.899	−43.154	1.00	26.62	C
ATOM	7538	CE1	PHE	B	113	33.534	−4.886	−41.219	1.00	26.46	C
ATOM	7539	CE2	PHE	B	113	34.167	−4.205	−43.446	1.00	26.59	C
ATOM	7540	CZ	PHE	B	113	34.059	−5.198	−42.481	1.00	26.51	C
ATOM	7541	HN	PHE	B	113	34.030	1.384	−41.010	1.00	26.60	H
ATOM	7542	HA	PHE	B	113	32.850	−0.958	−39.466	1.00	26.60	H

ATOM	7543	HB1	PHE	B	113	31.692	−1.169	−41.458	1.00	26.60	H
ATOM	7544	HB2	PHE	B	113	33.042	−0.540	−42.433	1.00	26.60	H
ATOM	7545	HD1	PHE	B	113	32.691	−3.346	−39.958	1.00	26.60	H
ATOM	7546	HD2	PHE	B	113	33.833	−2.122	−43.913	1.00	26.60	H
ATOM	7547	HE1	PHE	B	113	33.460	−5.659	−40.454	1.00	26.60	H
ATOM	7548	HE2	PHE	B	113	34.581	−4.439	−44.427	1.00	26.60	H
ATOM	7549	HZ	PHE	B	113	34.381	−6.215	−42.704	1.00	26.60	H
ATOM	7550	N	THR	B	114	35.068	−2.068	−39.242	1.00	26.48	N
ATOM	7551	CA	THR	B	114	36.429	−2.605	−38.934	1.00	26.42	C
ATOM	7552	C	THR	B	114	36.640	−4.084	−39.388	1.00	26.36	C
ATOM	7553	O	THR	B	114	35.935	−5.030	−38.950	1.00	26.29	O
ATOM	7554	CB	THR	B	114	36.865	−2.446	−37.402	1.00	26.35	C
ATOM	7555	OG1	THR	B	114	37.134	−1.071	−37.068	1.00	26.44	O
ATOM	7556	CG2	THR	B	114	38.108	−3.273	−37.113	1.00	26.26	C
ATOM	7557	HN	THR	B	114	34.226	−2.456	−38.708	1.00	26.37	H
ATOM	7558	HA	THR	B	114	37.131	−2.006	−39.514	1.00	26.37	H
ATOM	7559	HB	THR	B	114	36.053	−2.805	−36.770	1.00	26.37	H
ATOM	7560	HG1	THR	B	114	37.912	−0.709	−37.673	1.00	26.37	H
ATOM	7561	1HG2	THR	B	114	38.390	−3.153	−36.067	1.00	26.37	H
ATOM	7562	2HG2	THR	B	114	38.926	−2.936	−37.750	1.00	26.37	H
ATOM	7563	3HG2	THR	B	114	37.901	−4.324	−37.314	1.00	26.37	H
ATOM	7564	N	ILE	B	115	37.631	−4.260	−40.256	1.00	26.40	N
ATOM	7565	CA	ILE	B	115	37.999	−5.562	−40.749	1.00	26.39	C
ATOM	7566	C	ILE	B	115	39.231	−5.917	−39.949	1.00	26.33	C
ATOM	7567	O	ILE	B	115	40.162	−5.130	−39.928	1.00	26.37	O
ATOM	7568	CB	ILE	B	115	38.393	−5.513	−42.292	1.00	26.50	C
ATOM	7569	CG1	ILE	B	115	37.222	−5.169	−43.211	1.00	26.56	C

ATOM 7570 CG2 ILE B 115 38.925 −6.852 −42.801 1.00 26.52 C
ATOM 7571 CD1 ILE B 115 37.601 −5.221 −44.715 1.00 26.66 C
ATOM 7572 HN ILE B 115 38.174 −3.405 −40.600 1.00 26.47 H
ATOM 7573 HA ILE B 115 37.201 −6.284 −40.574 1.00 26.47 H
ATOM 7574 HB ILE B 115 39.169 −4.760 −42.426 1.00 26.47 H
ATOM 7575 1HG1 ILE B 115 36.417 −5.881 −43.030 1.00 26.47 H
ATOM 7576 2HG1 ILE B 115 36.877 −4.163 −42.974 1.00 26.47 H
ATOM 7577 1HG2 ILE B 115 39.179 −6.765 −43.857 1.00 26.47 H
ATOM 7578 2HG2 ILE B 115 38.161 −7.619 −42.674 1.00 26.47 H
ATOM 7579 3HG2 ILE B 115 39.815 −7.129 −42.236 1.00 26.47 H
ATOM 7580 1HD1 ILE B 115 36.730 −4.968 −45.319 1.00 26.47 H
ATOM 7581 2HD1 ILE B 115 37.940 −6.225 −44.970 1.00 26.47 H
ATOM 7582 3HD1 ILE B 115 38.400 −4.507 −44.914 1.00 26.47 H
ATOM 7583 N LYS B 116 39.244 −7.060 −39.266 1.00 26.24 N
ATOM 7584 CA LYS B 116 40.523 −7.720 −38.904 1.00 26.20 C
ATOM 7585 C LYS B 116 40.716 −8.935 −39.820 1.00 26.27 C
ATOM 7586 O LYS B 116 39.816 −9.766 −39.977 1.00 26.27 O
ATOM 7587 CB LYS B 116 40.610 −8.158 −37.420 1.00 26.06 C
ATOM 7588 CG LYS B 116 41.878 −8.991 −37.130 1.00 26.02 C
ATOM 7589 CD LYS B 116 42.168 −9.222 −35.686 1.00 25.87 C
ATOM 7590 CE LYS B 116 43.599 −9.712 −35.468 1.00 25.85 C
ATOM 7591 NZ LYS B 116 44.582 −8.597 −35.384 1.00 25.89 N1+
ATOM 7592 HN LYS B 116 38.318 −7.510 −38.974 1.00 26.07 H
ATOM 7593 HA LYS B 116 41.333 −7.017 −39.098 1.00 26.07 H
ATOM 7594 HB1 LYS B 116 40.624 −7.267 −36.792 1.00 26.07 H
ATOM 7595 HB2 LYS B 116 39.733 −8.759 −37.180 1.00 26.07 H
ATOM 7596 HG1 LYS B 116 41.760 −9.963 −37.610 1.00 26.07 H

ATOM 7597 HG2 LYS B 116 42.731 −8.470 −37.566 1.00 26.07 H

ATOM 7598 HD1 LYS B 116 42.029 −8.287 −35.144 1.00 26.07 H

ATOM 7599 HD2 LYS B 116 41.476 −9.972 −35.302 1.00 26.07 H

ATOM 7600 HE1 LYS B 116 43.635 −10.279 −34.538 1.00 26.07 H

ATOM 7601 HE2 LYS B 116 43.876 −10.359 −36.300 1.00 26.07 H

ATOM 7602 HZ1 LYS B 116 45.563 −8.996 −35.232 1.00 26.07 H

ATOM 7603 HZ2 LYS B 116 44.327 −7.957 −34.565 1.00 26.07 H

ATOM 7604 HZ3 LYS B 116 44.564 −8.035 −36.295 1.00 26.07 H

ATOM 7605 N PHE B 117 41.900 −9.050 −40.399 1.00 26.54 N

ATOM 7606 CA PHE B 117 42.149 −10.081 −41.376 1.00 26.64 C

ATOM 7607 C PHE B 117 42.527 −11.402 −40.693 1.00 26.59 C

ATOM 7608 O PHE B 117 43.677 −11.625 −40.272 1.00 26.58 O

ATOM 7609 CB PHE B 117 43.189 −9.573 −42.341 1.00 26.77 C

ATOM 7610 CG PHE B 117 42.828 −8.243 −42.910 1.00 26.82 C

ATOM 7611 CD1 PHE B 117 41.996 −8.157 −44.017 1.00 26.89 C

ATOM 7612 CD2 PHE B 117 43.266 −7.075 −42.311 1.00 26.80 C

ATOM 7613 CE1 PHE B 117 41.652 −6.925 −44.537 1.00 26.94 C

ATOM 7614 CE2 PHE B 117 42.918 −5.841 −42.816 1.00 26.86 C

ATOM 7615 CZ PHE B 117 42.110 −5.761 −43.926 1.00 26.93 C

ATOM 7616 HN PHE B 117 42.683 −8.369 −40.139 1.00 26.76 H

ATOM 7617 HA PHE B 117 41.226 −10.244 −41.933 1.00 26.76 H

ATOM 7618 HB1 PHE B 117 44.140 −9.484 −41.817 1.00 26.76 H

ATOM 7619 HB2 PHE B 117 43.289 −10.288 −43.157 1.00 26.76 H

ATOM 7620 HD1 PHE B 117 41.612 −9.067 −44.479 1.00 26.76 H

ATOM 7621 HD2 PHE B 117 43.897 −7.132 −41.424 1.00 26.76 H

ATOM 7622 HE1 PHE B 117 41.023 −6.864 −45.425 1.00 26.76 H

ATOM 7623 HE2 PHE B 117 43.282 −4.932 −42.337 1.00 26.76 H

ATOM 7624 HZ PHE B 117 41.829 −4.787 −44.327 1.00 26.76 H
ATOM 7625 N GLN B 118 41.511 −12.257 −40.559 1.00 26.38 N
ATOM 7626 CA GLN B 118 41.626 −13.565 −39.921 1.00 26.35 C
ATOM 7627 C GLN B 118 40.372 −14.370 −40.232 1.00 26.41 C
ATOM 7628 O GLN B 118 39.479 −13.918 −40.911 1.00 26.46 O
ATOM 7629 CB GLN B 118 41.751 −13.403 −38.418 1.00 26.17 C
ATOM 7630 CG GLN B 118 40.596 −12.607 −37.819 1.00 26.08 C
ATOM 7631 CD GLN B 118 39.977 −13.246 −36.604 1.00 25.97 C
ATOM 7632 OE1 GLN B 118 40.210 −14.422 −36.286 1.00 25.96 O
ATOM 7633 NE2 GLN B 118 39.146 −12.476 −35.926 1.00 25.89 N
ATOM 7634 HN GLN B 118 40.555 −11.966 −40.942 1.00 26.19 H
ATOM 7635 HA GLN B 118 42.502 −14.086 −40.308 1.00 26.19 H
ATOM 7636 HB1 GLN B 118 41.769 −14.392 −37.960 1.00 26.19 H
ATOM 7637 HB2 GLN B 118 42.684 −12.884 −38.199 1.00 26.19 H
ATOM 7638 HG1 GLN B 118 40.969 −11.623 −37.536 1.00 26.19 H
ATOM 7639 HG2 GLN B 118 39.823 −12.499 −38.580 1.00 26.19 H
ATOM 7640 1HE2 GLN B 118 38.956 −11.475 −36.254 1.00 26.19 H
ATOM 7641 2HE2 GLN B 118 38.663 −12.853 −35.049 1.00 26.19 H
ATOM 7642 N GLU B 119 40.276 −15.551 −39.670 1.00 26.41 N
ATOM 7643 CA GLU B 119 39.191 −16.455 −40.000 1.00 26.51 C
ATOM 7644 C GLU B 119 37.916 −16.123 −39.222 1.00 26.41 C
ATOM 7645 O GLU B 119 36.843 −16.419 −39.693 1.00 26.51 O
ATOM 7646 CB GLU B 119 39.621 −17.918 −39.757 1.00 26.58 C
ATOM 7647 CG GLU B 119 41.125 −18.219 −40.079 1.00 26.64 C
ATOM 7648 CD GLU B 119 41.341 −19.399 −41.021 1.00 26.88 C
ATOM 7649 OE1 GLU B 119 42.318 −20.170 −40.817 1.00 26.93 O
ATOM 7650 OE2 GLU B 119 40.542 −19.544 −41.982 1.00 27.02 O

ATOM 7651 HN GLU B 119 41.012 −15.853 −38.955 1.00 26.65 H

ATOM 7652 HA GLU B 119 38.973 −16.342 −41.062 1.00 26.65 H

ATOM 7653 HB1 GLU B 119 39.444 −18.154 −38.708 1.00 26.65 H

ATOM 7654 HB2 GLU B 119 39.004 −18.562 −40.384 1.00 26.65 H

ATOM 7655 HG1 GLU B 119 41.560 −17.331 −40.539 1.00 26.65 H

ATOM 7656 HG2 GLU B 119 41.639 −18.432 −39.142 1.00 26.65 H

ATOM 7657 N PHE B 120 38.045 −15.512 −38.037 1.00 26.24 N

ATOM 7658 CA PHE B 120 36.899 −15.215 −37.124 1.00 26.16 C

ATOM 7659 C PHE B 120 36.279 −13.792 −37.251 1.00 26.13 C

ATOM 7660 O PHE B 120 36.930 −12.774 −36.949 1.00 26.05 O

ATOM 7661 CB PHE B 120 37.278 −15.435 −35.636 1.00 26.00 C

ATOM 7662 CG PHE B 120 37.710 −16.829 −35.294 1.00 26.02 C

ATOM 7663 CD1 PHE B 120 37.182 −17.942 −35.928 1.00 26.18 C

ATOM 7664 CD2 PHE B 120 38.630 −17.041 −34.307 1.00 25.88 C

ATOM 7665 CE1 PHE B 120 37.597 −19.260 −35.586 1.00 26.23 C

ATOM 7666 CE2 PHE B 120 39.044 −18.359 −33.968 1.00 25.91 C

ATOM 7667 CZ PHE B 120 38.524 −19.453 −34.615 1.00 26.09 C

ATOM 7668 HN PHE B 120 39.026 −15.219 −37.726 1.00 26.08 H

ATOM 7669 HA PHE B 120 36.110 −15.929 −37.361 1.00 26.08 H

ATOM 7670 HB1 PHE B 120 38.096 −14.758 −35.391 1.00 26.08 H

ATOM 7671 HB2 PHE B 120 36.409 −15.191 −35.026 1.00 26.08 H

ATOM 7672 HD1 PHE B 120 36.432 −17.805 −36.707 1.00 26.08 H

ATOM 7673 HD2 PHE B 120 39.052 −16.190 −33.773 1.00 26.08 H

ATOM 7674 HE1 PHE B 120 37.168 −20.117 −36.106 1.00 26.08 H

ATOM 7675 HE2 PHE B 120 39.786 −18.503 −33.182 1.00 26.08 H

ATOM 7676 HZ PHE B 120 38.851 −20.459 −34.353 1.00 26.08 H

ATOM 7677 N SER B 121 34.992 −13.748 −37.616 1.00 27.80 N

ATOM 7678 CA SER B 121 34.241 −12.497 −37.777 1.00 27.80 C

ATOM 7679 C SER B 121 32.791 −12.586 −37.218 1.00 27.82 C

ATOM 7680 O SER B 121 32.152 −13.635 −37.288 1.00 27.90 O

ATOM 7681 CB SER B 121 34.224 −12.130 −39.262 1.00 27.91 C

ATOM 7682 OG SER B 121 34.083 −13.283 −40.092 1.00 28.03 O

ATOM 7683 HN SER B 121 34.485 −14.672 −37.800 1.00 27.88 H

ATOM 7684 HA SER B 121 34.768 −11.710 −37.237 1.00 27.88 H

ATOM 7685 HB1 SER B 121 33.388 −11.456 −39.449 1.00 27.88 H

ATOM 7686 HB2 SER B 121 35.159 −11.628 −39.512 1.00 27.88 H

ATOM 7687 HG SER B 121 34.887 −13.937 −39.924 1.00 27.88 H

ATOM 7688 N PRO B 122 32.266 −11.488 −36.645 1.00 27.67 N

ATOM 7689 CA PRO B 122 30.842 −11.492 −36.329 1.00 27.73 C

ATOM 7690 C PRO B 122 29.970 −11.715 −37.567 1.00 27.88 C

ATOM 7691 O PRO B 122 28.779 −12.029 −37.411 1.00 27.96 O

ATOM 7692 CB PRO B 122 30.577 −10.079 −35.802 1.00 27.70 C

ATOM 7693 CG PRO B 122 31.859 −9.361 −35.788 1.00 27.62 C

ATOM 7694 CD PRO B 122 32.941 −10.231 −36.279 1.00 27.60 C

ATOM 7695 HA PRO B 122 30.620 −12.236 −35.564 1.00 27.74 H

ATOM 7696 HB1 PRO B 122 29.871 −9.566 −36.455 1.00 27.74 H

ATOM 7697 HB2 PRO B 122 30.169 −10.130 −34.793 1.00 27.74 H

ATOM 7698 HG1 PRO B 122 31.786 −8.483 −36.430 1.00 27.74 H

ATOM 7699 HG2 PRO B 122 32.084 −9.049 −34.768 1.00 27.74 H

ATOM 7700 HD1 PRO B 122 33.427 −9.788 −37.148 1.00 27.74 H

ATOM 7701 HD2 PRO B 122 33.681 −10.404 −35.497 1.00 27.74 H

ATOM 7702 N ASN B 123 30.548 −11.534 −38.769 1.00 33.40 N

ATOM 7703 CA ASN B 123 29.855 −11.735 −40.044 1.00 33.53 C

ATOM 7704 C ASN B 123 29.315 −13.139 −40.125 1.00 33.63 C

ATOM 7705 O ASN B 123 30.080 −14.071 −40.022 1.00 33.63 O

ATOM 7706 CB ASN B 123 30.847 −11.501 −41.193 1.00 33.54 C

ATOM 7707 CG ASN B 123 30.520 −12.314 −42.454 1.00 33.68 C

ATOM 7708 OD1 ASN B 123 31.215 −13.297 −42.756 1.00 33.73 O

ATOM 7709 ND2 ASN B 123 29.473 −11.908 −43.192 1.00 33.75 N

ATOM 7710 HN ASN B 123 31.572 −11.226 −38.795 1.00 33.61 H

ATOM 7711 HA ASN B 123 29.032 −11.025 −40.125 1.00 33.61 H

ATOM 7712 HB1 ASN B 123 30.833 −10.442 −41.451 1.00 33.61 H

ATOM 7713 HB2 ASN B 123 31.844 −11.779 −40.851 1.00 33.61 H

ATOM 7714 1HD2 ASN B 123 28.907 −11.051 −42.892 1.00 33.61 H

ATOM 7715 2HD2 ASN B 123 29.205 −12.442 −44.080 1.00 33.61 H

ATOM 7716 N LEU B 124 28.016 −13.300 −40.341 1.00 26.77 N

ATOM 7717 CA LEU B 124 27.388 −14.618 −40.249 1.00 26.90 C

ATOM 7718 C LEU B 124 27.165 −15.281 −41.608 1.00 27.05 C

ATOM 7719 O LEU B 124 26.306 −16.167 −41.766 1.00 27.21 O

ATOM 7720 CB LEU B 124 26.064 −14.498 −39.490 1.00 26.95 C

ATOM 7721 CG LEU B 124 26.129 −15.147 −38.126 1.00 26.90 C

ATOM 7722 CD1 LEU B 124 26.270 −16.663 −38.308 1.00 27.02 C

ATOM 7723 CD2 LEU B 124 27.294 −14.537 −37.307 1.00 26.70 C

ATOM 7724 HN LEU B 124 27.416 −12.449 −40.587 1.00 26.94 H

ATOM 7725 HA LEU B 124 28.048 −15.262 −39.668 1.00 26.94 H

ATOM 7726 HB1 LEU B 124 25.825 −13.442 −39.365 1.00 26.94 H

ATOM 7727 HB2 LEU B 124 25.280 −14.982 −40.072 1.00 26.94 H

ATOM 7728 HG LEU B 124 25.195 −14.946 −37.602 1.00 26.94 H

ATOM 7729 1HD1 LEU B 124 26.318 −17.144 −37.331 1.00 26.94 H

ATOM 7730 2HD1 LEU B 124 27.182 −16.879 −38.864 1.00 26.94 H

ATOM 7731 3HD1 LEU B 124 25.410 −17.045 −38.858 1.00 26.94 H

ATOM 7732 1HD2 LEU B 124 27.335 −15.009 −36.325 1.00 26.94 H

ATOM 7733 2HD2 LEU B 124 27.133 −13.466 −37.188 1.00 26.94 H

ATOM 7734 3HD2 LEU B 124 28.235 −14.707 −37.831 1.00 26.94 H

ATOM 7735 N TRP B 125 27.964 −14.864 −42.584 1.00 39.25 N

ATOM 7736 CA TRP B 125 27.805 −15.317 −43.944 1.00 39.40 C

ATOM 7737 C TRP B 125 29.042 −16.034 −44.441 1.00 39.43 C

ATOM 7738 O TRP B 125 29.168 −16.273 −45.642 1.00 39.54 O

ATOM 7739 CB TRP B 125 27.429 −14.137 −44.836 1.00 39.37 C

ATOM 7740 CG TRP B 125 25.961 −14.078 −45.031 1.00 39.47 C

ATOM 7741 CD1 TRP B 125 25.029 −13.554 −44.166 1.00 39.44 C

ATOM 7742 CD2 TRP B 125 25.227 −14.604 −46.147 1.00 39.63 C

ATOM 7743 NE1 TRP B 125 23.763 −13.705 −44.688 1.00 39.57 N

ATOM 7744 CE2 TRP B 125 23.854 −14.343 −45.905 1.00 39.69 C

ATOM 7745 CE3 TRP B 125 25.598 −15.241 −47.351 1.00 39.74 C

ATOM 7746 CZ2 TRP B 125 22.840 −14.712 −46.832 1.00 39.85 C

ATOM 7747 CZ3 TRP B 125 24.590 −15.599 −48.274 1.00 39.90 C

ATOM 7748 CH2 TRP B 125 23.233 −15.334 −48.003 1.00 39.94 C

ATOM 7749 HN TRP B 125 28.748 −14.173 −42.355 1.00 39.59 H

ATOM 7750 HA TRP B 125 26.978 −16.027 −43.964 1.00 39.59 H

ATOM 7751 HB1 TRP B 125 27.766 −13.212 −44.367 1.00 39.59 H

ATOM 7752 HB2 TRP B 125 27.915 −14.251 −45.805 1.00 39.59 H

ATOM 7753 HD1 TRP B 125 25.258 −13.088 −43.208 1.00 39.59 H

ATOM 7754 HE1 TRP B 125 22.856 −13.379 −44.223 1.00 39.59 H

ATOM 7755 HE3 TRP B 125 26.645 −15.453 −47.565 1.00 39.59 H

ATOM 7756 HZ2 TRP B 125 21.789 −14.511 −46.626 1.00 39.59 H

ATOM 7757 HZ3 TRP B 125 24.866 −16.087 −49.209 1.00 39.59 H

ATOM 7758 HH2 TRP B 125 22.477 −15.625 −48.732 1.00 39.59 H

ATOM 7759 N GLY B 126 29.926 −16.419 −43.518 1.00 36.07 N
ATOM 7760 CA GLY B 126 31.108 −17.203 −43.857 1.00 36.13 C
ATOM 7761 C GLY B 126 32.140 −16.496 −44.731 1.00 36.09 C
ATOM 7762 O GLY B 126 32.898 −17.146 −45.483 1.00 36.20 O
ATOM 7763 HN GLY B 126 29.762 −16.144 −42.497 1.00 36.12 H
ATOM 7764 HA1 GLY B 126 31.597 −17.491 −42.927 1.00 36.12 H
ATOM 7765 HA2 GLY B 126 30.777 −18.097 −44.386 1.00 36.12 H
ATOM 7766 N LEU B 127 32.169 −15.164 −44.618 1.00 27.66 N
ATOM 7767 CA LEU B 127 33.179 −14.326 −45.293 1.00 27.62 C
ATOM 7768 C LEU B 127 34.330 −14.056 −44.281 1.00 27.49 C
ATOM 7769 O LEU B 127 34.119 −13.461 −43.177 1.00 27.36 O
ATOM 7770 CB LEU B 127 32.586 −12.994 −45.842 1.00 27.58 C
ATOM 7771 CG LEU B 127 31.526 −13.005 −46.970 1.00 27.69 C
ATOM 7772 CD1 LEU B 127 30.531 −11.856 −46.867 1.00 27.62 C
ATOM 7773 CD2 LEU B 127 32.167 −13.006 −48.350 1.00 27.78 C
ATOM 7774 HN LEU B 127 31.428 −14.685 −44.012 1.00 27.60 H
ATOM 7775 HA LEU B 127 33.586 −14.890 −46.132 1.00 27.60 H
ATOM 7776 HB1 LEU B 127 32.132 −12.483 −44.993 1.00 27.60 H
ATOM 7777 HB2 LEU B 127 33.427 −12.408 −46.212 1.00 27.60 H
ATOM 7778 HG LEU B 127 30.961 −13.932 −46.875 1.00 27.60 H
ATOM 7779 1HD1 LEU B 127 29.815 −11.920 −47.686 1.00 27.60 H
ATOM 7780 2HD1 LEU B 127 31.064 −10.907 −46.925 1.00 27.60 H
ATOM 7781 3HD1 LEU B 127 30.001 −11.918 −45.916 1.00 27.60 H
ATOM 7782 1HD2 LEU B 127 31.388 −13.014 −49.113 1.00 27.60 H
ATOM 7783 2HD2 LEU B 127 32.792 −13.892 −48.460 1.00 27.60 H
ATOM 7784 3HD2 LEU B 127 32.780 −12.113 −48.467 1.00 27.60 H
ATOM 7785 N GLU B 128 35.520 −14.560 −44.649 1.00 54.37 N

ATOM 7786 CA GLU B 128 36.760 −14.384 −43.869 1.00 54.27 C
ATOM 7787 C GLU B 128 37.913 −13.956 −44.795 1.00 54.34 C
ATOM 7788 O GLU B 128 37.694 −13.680 −45.983 1.00 54.44 O
ATOM 7789 CB GLU B 128 37.119 −15.670 −43.123 1.00 54.29 C
ATOM 7790 CG GLU B 128 38.119 −16.558 −43.830 1.00 54.44 C
ATOM 7791 CD GLU B 128 37.544 −17.913 −44.120 1.00 54.60 C
ATOM 7792 OE1 GLU B 128 37.619 −18.802 −43.227 1.00 54.59 O
ATOM 7793 OE2 GLU B 128 37.015 −18.076 −45.256 1.00 54.75 O
ATOM 7794 HN GLU B 128 35.571 −15.121 −45.559 1.00 54.45 H
ATOM 7795 HA GLU B 128 36.596 −13.595 −43.135 1.00 54.45 H
ATOM 7796 HB1 GLU B 128 37.536 −15.394 −42.155 1.00 54.45 H
ATOM 7797 HB2 GLU B 128 36.203 −16.242 −42.975 1.00 54.45 H
ATOM 7798 HG1 GLU B 128 38.408 −16.088 −44.770 1.00 54.45 H
ATOM 7799 HG2 GLU B 128 38.998 −16.675 −43.197 1.00 54.45 H
ATOM 7800 N PHE B 129 39.135 −13.924 −44.264 1.00 36.94 N
ATOM 7801 CA PHE B 129 40.296 −13.520 −45.049 1.00 37.02 C
ATOM 7802 C PHE B 129 41.467 −14.454 −44.866 1.00 37.08 C
ATOM 7803 O PHE B 129 41.718 −14.995 −43.758 1.00 37.00 O
ATOM 7804 CB PHE B 129 40.699 −12.074 −44.750 1.00 36.93 C
ATOM 7805 CG PHE B 129 39.632 −11.088 −45.095 1.00 36.90 C
ATOM 7806 CD1 PHE B 129 39.198 −10.957 −46.422 1.00 37.01 C
ATOM 7807 CD2 PHE B 129 39.029 −10.314 −44.108 1.00 36.77 C
ATOM 7808 CE1 PHE B 129 38.171 −10.049 −46.774 1.00 36.98 C
ATOM 7809 CE2 PHE B 129 37.997 −9.390 −44.439 1.00 36.76 C
ATOM 7810 CZ PHE B 129 37.563 −9.262 −45.772 1.00 36.86 C
ATOM 7811 HN PHE B 129 39.267 −14.201 −43.239 1.00 36.93 H
ATOM 7812 HA PHE B 129 40.008 −13.565 −46.099 1.00 36.93 H

ATOM 7813 HB1 PHE B 129 40.920 −11.988 −43.686 1.00 36.93 H
ATOM 7814 HB2 PHE B 129 41.592 −11.836 −45.328 1.00 36.93 H
ATOM 7815 HD1 PHE B 129 39.661 −11.567 −47.198 1.00 36.93 H
ATOM 7816 HD2 PHE B 129 39.350 −10.416 −43.071 1.00 36.93 H
ATOM 7817 HE1 PHE B 129 37.852 −9.958 −47.812 1.00 36.93 H
ATOM 7818 HE2 PHE B 129 37.542 −8.780 −43.658 1.00 36.93 H
ATOM 7819 HZ PHE B 129 36.767 −8.563 −46.028 1.00 36.93 H
ATOM 7820 N GLN B 130 42.171 −14.626 −45.989 1.00 40.19 N
ATOM 7821 CA GLN B 130 43.243 −15.613 −46.124 1.00 40.32 C
ATOM 7822 C GLN B 130 44.402 −15.100 −46.997 1.00 40.44 C
ATOM 7823 O GLN B 130 44.158 −14.381 −47.951 1.00 40.50 O
ATOM 7824 CB GLN B 130 42.661 −16.938 −46.670 1.00 40.48 C
ATOM 7825 CG GLN B 130 41.570 −17.562 −45.726 1.00 40.38 C
ATOM 7826 CD GLN B 130 41.649 −19.087 −45.579 1.00 40.53 C
ATOM 7827 OE1 GLN B 130 41.439 −19.835 −46.550 1.00 40.74 O
ATOM 7828 NE2 GLN B 130 41.931 −19.551 −44.362 1.00 40.42 N
ATOM 7829 HN GLN B 130 41.939 −14.010 −46.833 1.00 40.44 H
ATOM 7830 HA GLN B 130 43.640 −15.810 −45.128 1.00 40.44 H
ATOM 7831 HB1 GLN B 130 42.209 −16.745 −47.643 1.00 40.44 H
ATOM 7832 HB2 GLN B 130 43.474 −17.655 −46.783 1.00 40.44 H
ATOM 7833 HG1 GLN B 130 41.683 −17.119 −44.737 1.00 40.44 H
ATOM 7834 HG2 GLN B 130 40.589 −17.310 −46.128 1.00 40.44 H
ATOM 7835 1HE2 GLN B 130 42.088 −18.869 −43.553 1.00 40.44 H
ATOM 7836 2HE2 GLN B 130 42.000 −20.606 −44.194 1.00 40.44 H
ATOM 7837 N LYS B 131 45.643 −15.479 −46.636 1.00 43.97 N
ATOM 7838 CA LYS B 131 46.859 −15.086 −47.366 1.00 44.11 C
ATOM 7839 C LYS B 131 46.671 −15.344 −48.853 1.00 44.32 C

ATOM 7840 O LYS B 131 45.876 −16.217 −49.250 1.00 44.40 O

ATOM 7841 CB LYS B 131 48.131 −15.897 −47.005 1.00 44.21 C

ATOM 7842 CG LYS B 131 48.373 −16.374 −45.579 1.00 44.05 C

ATOM 7843 CD LYS B 131 49.830 −16.918 −45.408 1.00 44.17 C

ATOM 7844 CE LYS B 131 49.955 −18.454 −45.588 1.00 44.34 C

ATOM 7845 NZ LYS B 131 51.334 −18.905 −46.116 1.00 44.58 N1+

ATOM 7846 HN LYS B 131 45.751 −16.100 −45.771 1.00 44.24 H

ATOM 7847 HA LYS B 131 47.050 −14.025 −47.205 1.00 44.24 H

ATOM 7848 HB1 LYS B 131 48.120 −16.788 −47.633 1.00 44.24 H

ATOM 7849 HB2 LYS B 131 48.984 −15.273 −47.273 1.00 44.24 H

ATOM 7850 HG1 LYS B 131 48.222 −15.540 −44.894 1.00 44.24 H

ATOM 7851 HG2 LYS B 131 47.666 −17.170 −45.345 1.00 44.24 H

ATOM 7852 HD1 LYS B 131 50.466 −16.433 −46.149 1.00 44.24 H

ATOM 7853 HD2 LYS B 131 50.176 −16.661 −44.407 1.00 44.24 H

ATOM 7854 HE1 LYS B 131 49.784 −18.929 −44.622 1.00 44.24 H

ATOM 7855 HE2 LYS B 131 49.192 −18.781 −46.294 1.00 44.24 H

ATOM 7856 HZ1 LYS B 131 51.348 −19.970 −46.215 1.00 44.24 H

ATOM 7857 HZ2 LYS B 131 52.096 −18.601 −45.429 1.00 44.24 H

ATOM 7858 HZ3 LYS B 131 51.515 −18.456 −47.070 1.00 44.24 H

ATOM 7859 N ASN B 132 47.437 −14.605 −49.663 1.00 34.69 N

ATOM 7860 CA ASN B 132 47.532 −14.862 −51.079 1.00 34.91 C

ATOM 7861 C ASN B 132 46.152 −15.054 −51.662 1.00 34.89 C

ATOM 7862 O ASN B 132 45.887 −16.008 −52.380 1.00 35.06 O

ATOM 7863 CB ASN B 132 48.365 −16.119 −51.300 1.00 35.12 C

ATOM 7864 CG ASN B 132 49.651 −16.110 −50.505 1.00 35.12 C

ATOM 7865 OD1 ASN B 132 50.419 −15.146 −50.550 1.00 35.13 O

ATOM 7866 ND2 ASN B 132 49.902 −17.194 −49.783 1.00 35.14 N

ATOM	7867	HN	ASN	B	132	47.999	−13.795	−49.246	1.00	35.01	H
ATOM	7868	HA	ASN	B	132	48.016	−14.017	−51.568	1.00	35.01	H
ATOM	7869	HB1	ASN	B	132	47.776	−16.986	−51.001	1.00	35.01	H
ATOM	7870	HB2	ASN	B	132	48.610	−16.194	−52.359	1.00	35.01	H
ATOM	7871	1HD2	ASN	B	132	49.211	−18.011	−49.786	1.00	35.01	H
ATOM	7872	2HD2	ASN	B	132	50.796	−17.252	−49.197	1.00	35.01	H
ATOM	7873	N	LYS	B	133	45.254	−14.169	−51.276	1.00	27.91	N
ATOM	7874	CA	LYS	B	133	43.963	−14.088	−51.893	1.00	27.89	C
ATOM	7875	C	LYS	B	133	43.626	−12.607	−52.058	1.00	27.77	C
ATOM	7876	O	LYS	B	133	44.160	−11.743	−51.347	1.00	27.67	O
ATOM	7877	CB	LYS	B	133	42.918	−14.849	−51.076	1.00	27.79	C
ATOM	7878	CG	LYS	B	133	42.978	−16.348	−51.312	1.00	27.96	C
ATOM	7879	CD	LYS	B	133	41.932	−17.136	−50.511	1.00	27.89	C
ATOM	7880	CE	LYS	B	133	42.321	−18.678	−50.331	1.00	28.07	C
ATOM	7881	NZ	LYS	B	133	43.123	−19.106	−49.083	1.00	27.99	N1+
ATOM	7882	HN	LYS	B	133	45.495	−13.494	−50.482	1.00	27.88	H
ATOM	7883	HA	LYS	B	133	44.024	−14.541	−52.883	1.00	27.88	H
ATOM	7884	HB1	LYS	B	133	43.091	−14.654	−50.018	1.00	27.88	H
ATOM	7885	HB2	LYS	B	133	41.927	−14.490	−51.354	1.00	27.88	H
ATOM	7886	HG1	LYS	B	133	42.814	−16.537	−52.373	1.00	27.88	H
ATOM	7887	HG2	LYS	B	133	43.969	−16.703	−51.028	1.00	27.88	H
ATOM	7888	HD1	LYS	B	133	41.836	−16.684	−49.524	1.00	27.88	H
ATOM	7889	HD2	LYS	B	133	40.977	−17.077	−51.033	1.00	27.88	H
ATOM	7890	HE1	LYS	B	133	41.389	−19.244	−50.321	1.00	27.88	H
ATOM	7891	HE2	LYS	B	133	42.910	−18.963	−51.203	1.00	27.88	H
ATOM	7892	HZ1	LYS	B	133	43.299	−20.161	−49.116	1.00	27.88	H
ATOM	7893	HZ2	LYS	B	133	42.567	−18.865	−48.201	1.00	27.88	H

ATOM 7894 HZ3 LYS B 133 44.060 −18.589 −49.066 1.00 27.88 H
ATOM 7895 N ASP B 134 42.782 −12.329 −53.050 1.00 35.95 N
ATOM 7896 CA ASP B 134 42.359 −10.973 −53.370 1.00 35.87 C
ATOM 7897 C ASP B 134 40.869 −10.832 −53.059 1.00 35.73 C
ATOM 7898 O ASP B 134 40.073 −11.752 −53.290 1.00 35.77 O
ATOM 7899 CB ASP B 134 42.648 −10.652 −54.834 1.00 36.02 C
ATOM 7900 CG ASP B 134 44.089 −10.942 −55.213 1.00 36.20 C
ATOM 7901 OD1 ASP B 134 45.023 −10.550 −54.441 1.00 36.17 O
ATOM 7902 OD2 ASP B 134 44.271 −11.569 −56.287 1.00 36.37 O
ATOM 7903 HN ASP B 134 42.398 −13.137 −53.637 1.00 36.01 H
ATOM 7904 HA ASP B 134 42.915 −10.276 −52.743 1.00 36.01 H
ATOM 7905 HB1 ASP B 134 41.992 −11.256 −55.461 1.00 36.01 H
ATOM 7906 HB2 ASP B 134 42.446 −9.595 −55.008 1.00 36.01 H
ATOM 7907 N TYR B 135 40.520 −9.684 −52.487 1.00 28.04 N
ATOM 7908 CA TYR B 135 39.160 −9.382 −52.059 1.00 27.91 C
ATOM 7909 C TYR B 135 38.836 −7.921 −52.478 1.00 27.87 C
ATOM 7910 O TYR B 135 39.652 −7.008 −52.291 1.00 27.88 O
ATOM 7911 CB TYR B 135 39.017 −9.623 −50.536 1.00 27.79 C
ATOM 7912 CG TYR B 135 39.574 −10.981 −49.994 1.00 27.83 C
ATOM 7913 CD1 TYR B 135 38.712 −12.071 −49.659 1.00 27.83 C
ATOM 7914 CD2 TYR B 135 40.948 −11.166 −49.796 1.00 27.88 C
ATOM 7915 CE1 TYR B 135 39.223 −13.309 −49.162 1.00 27.88 C
ATOM 7916 CE2 TYR B 135 41.459 −12.391 −49.304 1.00 27.92 C
ATOM 7917 CZ TYR B 135 40.605 −13.452 −48.993 1.00 27.92 C
ATOM 7918 OH TYR B 135 41.187 −14.619 −48.518 1.00 27.98 O
ATOM 7919 HN TYR B 135 41.277 −8.943 −52.333 1.00 27.89 H
ATOM 7920 HA TYR B 135 38.476 −10.051 −52.581 1.00 27.89 H

ATOM 7921 HB1 TYR B 135 39.544 −8.819 −50.022 1.00 27.89 H
ATOM 7922 HB2 TYR B 135 37.956 −9.578 −50.291 1.00 27.89 H
ATOM 7923 HD1 TYR B 135 37.636 −11.954 −49.786 1.00 27.89 H
ATOM 7924 HD2 TYR B 135 41.636 −10.352 −50.025 1.00 27.89 H
ATOM 7925 HE1 TYR B 135 38.548 −14.129 −48.918 1.00 27.89 H
ATOM 7926 HE2 TYR B 135 42.534 −12.508 −49.166 1.00 27.89 H
ATOM 7927 HH TYR B 135 40.819 −14.829 −47.557 1.00 27.89 H
ATOM 7928 N TYR B 136 37.653 −7.727 −53.068 1.00 30.21 N
ATOM 7929 CA TYR B 136 37.272 −6.485 −53.806 1.00 30.20 C
ATOM 7930 C TYR B 136 36.092 −5.808 −53.114 1.00 30.09 C
ATOM 7931 O TYR B 136 35.016 −6.399 −52.974 1.00 30.05 O
ATOM 7932 CB TYR B 136 36.873 −6.764 −55.272 1.00 30.28 C
ATOM 7933 CG TYR B 136 37.802 −7.689 −56.000 1.00 30.42 C
ATOM 7934 CD1 TYR B 136 38.678 −7.210 −56.964 1.00 30.53 C
ATOM 7935 CD2 TYR B 136 37.802 −9.053 −55.713 1.00 30.47 C
ATOM 7936 CE1 TYR B 136 39.533 −8.077 −57.627 1.00 30.68 C
ATOM 7937 CE2 TYR B 136 38.646 −9.914 −56.329 1.00 30.63 C
ATOM 7938 CZ TYR B 136 39.517 −9.441 −57.292 1.00 30.73 C
ATOM 7939 OH TYR B 136 40.357 −10.356 −57.922 1.00 30.91 O
ATOM 7940 HN TYR B 136 36.930 −8.514 −53.008 1.00 30.43 H
ATOM 7941 HA TYR B 136 38.120 −5.801 −53.799 1.00 30.43 H
ATOM 7942 HB1 TYR B 136 35.877 −7.208 −55.276 1.00 30.43 H
ATOM 7943 HB2 TYR B 136 36.850 −5.814 −55.806 1.00 30.43 H
ATOM 7944 HD1 TYR B 136 38.694 −6.146 −57.201 1.00 30.43 H
ATOM 7945 HD2 TYR B 136 37.102 −9.438 −54.971 1.00 30.43 H
ATOM 7946 HE1 TYR B 136 40.208 −7.705 −58.398 1.00 30.43 H
ATOM 7947 HE2 TYR B 136 38.639 −10.972 −56.067 1.00 30.43 H

ATOM 7948 HH TYR B 136 40.159 −10.361 −58.953 1.00 30.43 H
ATOM 7949 N ILE B 137 36.293 −4.569 −52.690 1.00 27.11 N
ATOM 7950 CA ILE B 137 35.267 −3.834 −51.986 1.00 27.03 C
ATOM 7951 C ILE B 137 34.905 −2.676 −52.885 1.00 27.07 C
ATOM 7952 O ILE B 137 35.811 −1.960 −53.295 1.00 27.13 O
ATOM 7953 CB ILE B 137 35.792 −3.332 −50.589 1.00 26.98 C
ATOM 7954 CG1 ILE B 137 36.388 −4.499 −49.777 1.00 26.95 C
ATOM 7955 CG2 ILE B 137 34.694 −2.602 −49.809 1.00 26.93 C
ATOM 7956 CD1 ILE B 137 35.833 −4.670 −48.355 1.00 26.85 C
ATOM 7957 HN ILE B 137 37.238 −4.101 −52.873 1.00 27.01 H
ATOM 7958 HA ILE B 137 34.395 −4.471 −51.841 1.00 27.01 H
ATOM 7959 HB ILE B 137 36.594 −2.618 −50.776 1.00 27.01 H
ATOM 7960 1HG1 ILE B 137 36.196 −5.421 −50.325 1.00 27.01 H
ATOM 7961 2HG1 ILE B 137 37.463 −4.339 −49.700 1.00 27.01 H
ATOM 7962 1HG2 ILE B 137 35.090 −2.269 −48.850 1.00 27.01 H
ATOM 7963 2HG2 ILE B 137 33.856 −3.279 −49.641 1.00 27.01 H
ATOM 7964 3HG2 ILE B 137 34.354 −1.739 −50.381 1.00 27.01 H
ATOM 7965 1HD1 ILE B 137 36.320 −5.518 −47.874 1.00 27.01 H
ATOM 7966 2HD1 ILE B 137 34.759 −4.848 −48.403 1.00 27.01 H
ATOM 7967 3HD1 ILE B 137 36.026 −3.766 −47.778 1.00 27.01 H
ATOM 7968 N ILE B 138 33.618 −2.501 −53.216 1.00 30.53 N
ATOM 7969 CA ILE B 138 33.160 −1.381 −54.080 1.00 30.56 C
ATOM 7970 C ILE B 138 31.811 −0.801 −53.602 1.00 30.52 C
ATOM 7971 O ILE B 138 31.228 −1.275 −52.629 1.00 30.47 O
ATOM 7972 CB ILE B 138 32.992 −1.780 −55.647 1.00 30.60 C
ATOM 7973 CG1 ILE B 138 31.637 −2.461 −55.889 1.00 30.56 C
ATOM 7974 CG2 ILE B 138 34.189 −2.617 −56.218 1.00 30.67 C

ATOM	7975	CD1	ILE	B	138	31.677	−3.503	−56.923	1.00	30.60	C
ATOM	7976	HN	ILE	B	138	32.892	−3.194	−52.844	1.00	30.56	H
ATOM	7977	HA	ILE	B	138	33.904	−0.587	−54.017	1.00	30.56	H
ATOM	7978	HB	ILE	B	138	32.973	−0.844	−56.205	1.00	30.56	H
ATOM	7979	1HG1	ILE	B	138	31.309	−2.916	−54.954	1.00	30.56	H
ATOM	7980	2HG1	ILE	B	138	30.919	−1.700	−56.195	1.00	30.56	H
ATOM	7981	1HG2	ILE	B	138	34.005	−2.845	−57.268	1.00	30.56	H
ATOM	7982	2HG2	ILE	B	138	34.284	−3.546	−55.656	1.00	30.56	H
ATOM	7983	3HG2	ILE	B	138	35.111	−2.042	−56.128	1.00	30.56	H
ATOM	7984	1HD1	ILE	B	138	30.685	−3.939	−57.039	1.00	30.56	H
ATOM	7985	2HD1	ILE	B	138	32.383	−4.280	−56.629	1.00	30.56	H
ATOM	7986	3HD1	ILE	B	138	31.993	−3.064	−57.869	1.00	30.56	H
ATOM	7987	N	SER	B	139	31.322	0.222	−54.305	1.00	27.04	N
ATOM	7988	CA	SER	B	139	29.872	0.396	−54.429	1.00	27.02	C
ATOM	7989	C	SER	B	139	29.495	0.838	−55.832	1.00	27.03	C
ATOM	7990	O	SER	B	139	30.279	1.450	−56.534	1.00	27.08	O
ATOM	7991	CB	SER	B	139	29.258	1.326	−53.401	1.00	27.03	C
ATOM	7992	OG	SER	B	139	27.841	1.156	−53.449	1.00	27.01	O
ATOM	7993	HN	SER	B	139	31.987	0.914	−54.777	1.00	27.04	H
ATOM	7994	HA	SER	B	139	29.422	−0.585	−54.276	1.00	27.04	H
ATOM	7995	HB1	SER	B	139	29.628	1.075	−52.407	1.00	27.04	H
ATOM	7996	HB2	SER	B	139	29.515	2.359	−53.635	1.00	27.04	H
ATOM	7997	HG	SER	B	139	27.492	1.395	−54.410	1.00	27.03	H
ATOM	7998	N	THR	B	140	28.274	0.504	−56.213	1.00	27.00	N
ATOM	7999	CA	THR	B	140	27.692	0.961	−57.453	1.00	27.00	C
ATOM	8000	C	THR	B	140	26.838	2.187	−57.194	1.00	27.01	C
ATOM	8001	O	THR	B	140	26.196	2.703	−58.109	1.00	27.01	O

ATOM 8002 CB THR B 140 26.857 −0.156 −58.098 1.00 26.98 C

ATOM 8003 OG1 THR B 140 25.845 −0.622 −57.196 1.00 26.97 O

ATOM 8004 CG2 THR B 140 27.747 −1.327 −58.423 1.00 27.00 C

ATOM 8005 HN THR B 140 27.693 −0.131 −55.577 1.00 27.00 H

ATOM 8006 HA THR B 140 28.496 1.234 −58.136 1.00 27.00 H

ATOM 8007 HB THR B 140 26.391 0.215 −59.011 1.00 27.00 H

ATOM 8008 HG1 THR B 140 26.297 −0.998 −56.326 1.00 27.00 H

ATOM 8009 1HG2 THR B 140 27.154 −2.119 −58.880 1.00 27.00 H

ATOM 8010 2HG2 THR B 140 28.206 −1.700 −57.507 1.00 27.00 H

ATOM 8011 3HG2 THR B 140 28.526 −1.010 −59.117 1.00 27.00 H

ATOM 8012 N SER B 141 26.827 2.645 −55.943 1.00 27.04 N

ATOM 8013 CA SER B 141 26.100 3.850 −55.564 1.00 27.09 C

ATOM 8014 C SER B 141 26.525 5.016 −56.458 1.00 27.14 C

ATOM 8015 O SER B 141 27.704 5.086 −56.826 1.00 27.16 O

ATOM 8016 CB SER B 141 26.386 4.209 −54.100 1.00 27.14 C

ATOM 8017 OG SER B 141 26.502 3.041 −53.343 1.00 27.08 O

ATOM 8018 HN SER B 141 27.370 2.111 −55.192 1.00 27.11 H

ATOM 8019 HA SER B 141 25.031 3.676 −55.687 1.00 27.11 H

ATOM 8020 HB1 SER B 141 27.317 4.773 −54.041 1.00 27.11 H

ATOM 8021 HB2 SER B 141 25.568 4.814 −53.709 1.00 27.11 H

ATOM 8022 HG SER B 141 25.606 2.497 −53.399 1.00 27.11 H

ATOM 8023 N ASN B 142 25.580 5.916 −56.795 1.00 27.18 N

ATOM 8024 CA ASN B 142 25.900 7.187 −57.475 1.00 27.25 C

ATOM 8025 C ASN B 142 26.158 8.307 −56.498 1.00 27.38 C

ATOM 8026 O ASN B 142 26.479 9.419 −56.929 1.00 27.48 O

ATOM 8027 CB ASN B 142 24.787 7.672 −58.393 1.00 27.23 C

ATOM 8028 CG ASN B 142 23.984 8.801 −57.772 1.00 27.34 C

ATOM	8029	OD1	ASN	B	142	23.394	8.649	−56.700	1.00	27.37	O
ATOM	8030	ND2	ASN	B	142	23.989	9.949	−58.428	1.00	27.41	N
ATOM	8031	HN	ASN	B	142	24.558	5.701	−56.561	1.00	27.33	H
ATOM	8032	HA	ASN	B	142	26.799	7.039	−58.073	1.00	27.33	H
ATOM	8033	HB1	ASN	B	142	25.229	8.027	−59.324	1.00	27.33	H
ATOM	8034	HB2	ASN	B	142	24.117	6.838	−58.603	1.00	27.33	H
ATOM	8035	1HD2	ASN	B	142	24.539	10.041	−59.341	1.00	27.33	H
ATOM	8036	2HD2	ASN	B	142	23.443	10.786	−58.045	1.00	27.33	H
ATOM	8037	N	GLY	B	143	25.948	8.040	−55.206	1.00	27.39	N
ATOM	8038	CA	GLY	B	143	26.363	8.954	−54.151	1.00	27.53	C
ATOM	8039	C	GLY	B	143	25.207	9.746	−53.615	1.00	27.62	C
ATOM	8040	O	GLY	B	143	25.195	10.089	−52.450	1.00	27.71	O
ATOM	8041	HN	GLY	B	143	25.460	7.126	−54.938	1.00	27.56	H
ATOM	8042	HA1	GLY	B	143	26.803	8.377	−53.337	1.00	27.56	H
ATOM	8043	HA2	GLY	B	143	27.106	9.642	−54.553	1.00	27.56	H
ATOM	8044	N	SER	B	144	24.244	10.067	−54.472	1.00	27.62	N
ATOM	8045	CA	SER	B	144	23.079	10.792	−54.024	1.00	27.72	C
ATOM	8046	C	SER	B	144	22.290	9.852	−53.125	1.00	27.67	C
ATOM	8047	O	SER	B	144	22.567	8.652	−53.072	1.00	27.54	O
ATOM	8048	CB	SER	B	144	22.230	11.306	−55.215	1.00	27.72	C
ATOM	8049	OG	SER	B	144	21.660	10.271	−56.028	1.00	27.56	O
ATOM	8050	HN	SER	B	144	24.333	9.786	−55.501	1.00	27.64	H
ATOM	8051	HA	SER	B	144	23.406	11.648	−53.433	1.00	27.64	H
ATOM	8052	HB1	SER	B	144	21.417	11.913	−54.817	1.00	27.64	H
ATOM	8053	HB2	SER	B	144	22.869	11.923	−55.847	1.00	27.64	H
ATOM	8054	HG	SER	B	144	22.428	9.680	−56.433	1.00	27.64	H
ATOM	8055	N	LEU	B	145	21.327	10.412	−52.406	1.00	27.79	N

ATOM 8056 CA LEU B 145 20.351 9.618 −51.679 1.00 27.76 C

ATOM 8057 C LEU B 145 19.359 8.964 −52.653 1.00 27.67 C

ATOM 8058 O LEU B 145 18.666 8.022 −52.271 1.00 27.63 O

ATOM 8059 CB LEU B 145 19.599 10.511 −50.709 1.00 27.95 C

ATOM 8060 CG LEU B 145 18.879 9.902 −49.525 1.00 27.99 C

ATOM 8061 CD1 LEU B 145 19.854 9.275 −48.581 1.00 27.92 C

ATOM 8062 CD2 LEU B 145 18.113 11.023 −48.849 1.00 28.21 C

ATOM 8063 HN LEU B 145 21.267 11.479 −52.360 1.00 27.87 H

ATOM 8064 HA LEU B 145 20.869 8.839 −51.120 1.00 27.87 H

ATOM 8065 HB1 LEU B 145 20.323 11.219 −50.307 1.00 27.87 H

ATOM 8066 HB2 LEU B 145 18.850 11.048 −51.291 1.00 27.87 H

ATOM 8067 HG LEU B 145 18.178 9.145 −49.877 1.00 27.86 H

ATOM 8068 1HD1 LEU B 145 19.317 8.843 −47.737 1.00 27.86 H

ATOM 8069 2HD1 LEU B 145 20.549 10.033 −48.219 1.00 27.86 H

ATOM 8070 3HD1 LEU B 145 20.408 8.491 −49.098 1.00 27.86 H

ATOM 8071 1HD2 LEU B 145 17.576 10.628 −47.986 1.00 27.86 H

ATOM 8072 2HD2 LEU B 145 17.401 11.453 −49.553 1.00 27.86 H

ATOM 8073 3HD2 LEU B 145 18.810 11.794 −48.521 1.00 27.86 H

ATOM 8074 N GLU B 146 19.299 9.482 −53.895 1.00 33.93 N

ATOM 8075 CA GLU B 146 18.397 9.007 −54.971 1.00 33.84 C

ATOM 8076 C GLU B 146 18.884 7.747 −55.666 1.00 33.68 C

ATOM 8077 O GLU B 146 18.079 6.904 −56.054 1.00 33.63 O

ATOM 8078 CB GLU B 146 18.207 10.095 −56.052 1.00 33.88 C

ATOM 8079 CG GLU B 146 17.211 11.220 −55.662 1.00 34.05 C

ATOM 8080 CD GLU B 146 17.748 12.134 −54.549 1.00 34.22 C

ATOM 8081 OE1 GLU B 146 18.975 12.069 −54.292 1.00 34.20 O

ATOM 8082 OE2 GLU B 146 16.964 12.922 −53.949 1.00 34.40 O

ATOM 8083 HN GLU B 146 19.952 10.298 −54.122 1.00 33.98 H
ATOM 8084 HA GLU B 146 17.424 8.795 −54.528 1.00 33.98 H
ATOM 8085 HB1 GLU B 146 19.177 10.552 −56.250 1.00 33.98 H
ATOM 8086 HB2 GLU B 146 17.840 9.614 −56.959 1.00 33.98 H
ATOM 8087 HG1 GLU B 146 17.010 11.828 −56.544 1.00 33.98 H
ATOM 8088 HG2 GLU B 146 16.285 10.760 −55.318 1.00 33.98 H
ATOM 8089 N GLY B 147 20.200 7.644 −55.827 1.00 27.35 N
ATOM 8090 CA GLY B 147 20.835 6.508 −56.515 1.00 27.23 C
ATOM 8091 C GLY B 147 21.542 5.507 −55.601 1.00 27.20 C
ATOM 8092 O GLY B 147 22.267 4.619 −56.070 1.00 27.12 O
ATOM 8093 HN GLY B 147 20.825 8.421 −55.439 1.00 27.23 H
ATOM 8094 HA1 GLY B 147 20.062 5.973 −57.067 1.00 27.23 H
ATOM 8095 HA2 GLY B 147 21.571 6.906 −57.213 1.00 27.23 H
ATOM 8096 N LEU B 148 21.295 5.641 −54.295 1.00 27.26 N
ATOM 8097 CA LEU B 148 21.950 4.845 −53.269 1.00 27.24 C
ATOM 8098 C LEU B 148 21.999 3.372 −53.668 1.00 27.15 C
ATOM 8099 O LEU B 148 23.069 2.745 −53.674 1.00 27.10 O
ATOM 8100 CB LEU B 148 21.213 5.047 −51.924 1.00 27.32 C
ATOM 8101 CG LEU B 148 21.600 4.258 −50.658 1.00 27.30 C
ATOM 8102 CD1 LEU B 148 23.054 3.788 −50.661 1.00 27.22 C
ATOM 8103 CD2 LEU B 148 21.336 5.125 −49.445 1.00 27.41 C
ATOM 8104 HN LEU B 148 20.577 6.373 −53.987 1.00 27.25 H
ATOM 8105 HA LEU B 148 22.973 5.204 −53.157 1.00 27.25 H
ATOM 8106 HB1 LEU B 148 21.321 6.102 −51.672 1.00 27.25 H
ATOM 8107 HB2 LEU B 148 20.163 4.824 −52.114 1.00 27.25 H
ATOM 8108 HG LEU B 148 20.958 3.379 −50.595 1.00 27.25 H
ATOM 8109 1HD1 LEU B 148 23.261 3.239 −49.742 1.00 27.25 H

ATOM	8110	2HD1	LEU	B	148	23.716	4.652	−50.724	1.00	27.25	H
ATOM	8111	3HD1	LEU	B	148	23.224	3.137	−51.518	1.00	27.25	H
ATOM	8112	1HD2	LEU	B	148	21.606	4.578	−48.541	1.00	27.25	H
ATOM	8113	2HD2	LEU	B	148	20.279	5.387	−49.408	1.00	27.25	H
ATOM	8114	3HD2	LEU	B	148	21.933	6.034	−49.511	1.00	27.25	H
ATOM	8115	N	ASP	B	149	20.842	2.840	−54.045	1.00	27.15	N
ATOM	8116	CA	ASP	B	149	20.692	1.409	−54.264	1.00	27.10	C
ATOM	8117	C	ASP	B	149	20.833	1.042	−55.754	1.00	27.06	C
ATOM	8118	O	ASP	B	149	20.360	−0.037	−56.149	1.00	27.06	O
ATOM	8119	CB	ASP	B	149	19.332	0.950	−53.684	1.00	27.17	C
ATOM	8120	CG	ASP	B	149	19.469	−0.042	−52.508	1.00	27.18	C
ATOM	8121	OD1	ASP	B	149	20.458	−0.796	−52.453	1.00	27.13	O
ATOM	8122	OD2	ASP	B	149	18.564	−0.093	−51.655	1.00	27.25	O
ATOM	8123	HN	ASP	B	149	19.998	3.481	−54.192	1.00	27.14	H
ATOM	8124	HA	ASP	B	149	21.483	0.899	−53.714	1.00	27.14	H
ATOM	8125	HB1	ASP	B	149	18.792	1.829	−53.333	1.00	27.14	H
ATOM	8126	HB2	ASP	B	149	18.763	0.467	−54.479	1.00	27.14	H
ATOM	8127	N	ASN	B	150	21.457	1.933	−56.569	1.00	27.03	N
ATOM	8128	CA	ASN	B	150	21.755	1.649	−58.005	1.00	26.99	C
ATOM	8129	C	ASN	B	150	22.678	0.450	−58.008	1.00	26.97	C
ATOM	8130	O	ASN	B	150	23.762	0.528	−57.460	1.00	26.97	O
ATOM	8131	CB	ASN	B	150	22.489	2.800	−58.721	1.00	26.98	C
ATOM	8132	CG	ASN	B	150	21.562	3.911	−59.245	1.00	26.99	C
ATOM	8133	OD1	ASN	B	150	20.646	4.338	−58.560	1.00	27.04	O
ATOM	8134	ND2	ASN	B	150	21.850	4.413	−60.458	1.00	26.96	N
ATOM	8135	HN	ASN	B	150	21.748	2.880	−56.164	1.00	26.99	H
ATOM	8136	HA	ASN	B	150	20.833	1.409	−58.535	1.00	26.99	H

ATOM	8137	HB1	ASN	B	150	23.193	3.246	−58.018	1.00	26.99	H
ATOM	8138	HB2	ASN	B	150	23.035	2.383	−59.567	1.00	26.99	H
ATOM	8139	1HD2	ASN	B	150	22.688	4.038	−61.007	1.00	26.99	H
ATOM	8140	2HD2	ASN	B	150	21.241	5.188	−60.875	1.00	26.99	H
ATOM	8141	N	GLN	B	151	22.260	−0.671	−58.587	1.00	26.97	N
ATOM	8142	CA	GLN	B	151	23.041	−1.933	−58.453	1.00	26.99	C
ATOM	8143	C	GLN	B	151	24.070	−2.085	−59.593	1.00	26.98	C
ATOM	8144	O	GLN	B	151	24.684	−3.137	−59.774	1.00	27.02	O
ATOM	8145	CB	GLN	B	151	22.116	−3.176	−58.397	1.00	27.04	C
ATOM	8146	CG	GLN	B	151	21.007	−3.187	−57.287	1.00	27.08	C
ATOM	8147	CD	GLN	B	151	21.472	−3.554	−55.839	1.00	27.09	C
ATOM	8148	OE1	GLN	B	151	20.873	−3.090	−54.864	1.00	27.10	O
ATOM	8149	NE2	GLN	B	151	22.494	−4.407	−55.713	1.00	27.09	N
ATOM	8150	HN	GLN	B	151	21.353	−0.667	−59.155	1.00	27.04	H
ATOM	8151	HA	GLN	B	151	23.591	−1.885	−57.513	1.00	27.04	H
ATOM	8152	HB1	GLN	B	151	21.616	−3.258	−59.362	1.00	27.04	H
ATOM	8153	HB2	GLN	B	151	22.748	−4.050	−58.239	1.00	27.04	H
ATOM	8154	HG1	GLN	B	151	20.565	−2.191	−57.250	1.00	27.04	H
ATOM	8155	HG2	GLN	B	151	20.248	−3.911	−57.584	1.00	27.04	H
ATOM	8156	1HE2	GLN	B	151	22.963	−4.813	−56.585	1.00	27.04	H
ATOM	8157	2HE2	GLN	B	151	22.843	−4.684	−54.740	1.00	27.04	H
ATOM	8158	N	GLU	B	152	24.246	−1.028	−60.370	1.00	41.10	N
ATOM	8159	CA	GLU	B	152	24.967	−1.133	−61.601	1.00	41.10	C
ATOM	8160	C	GLU	B	152	25.490	0.265	−61.922	1.00	41.08	C
ATOM	8161	O	GLU	B	152	24.767	1.262	−61.750	1.00	41.06	O
ATOM	8162	CB	GLU	B	152	24.005	−1.651	−62.665	1.00	41.10	C
ATOM	8163	CG	GLU	B	152	24.545	−2.807	−63.455	1.00	41.15	C

ATOM	8164	CD	GLU	B	152	25.659	−2.372	−64.396	1.00	41.16	C
ATOM	8165	OE1	GLU	B	152	25.354	−1.699	−65.430	1.00	41.12	O
ATOM	8166	OE2	GLU	B	152	26.836	−2.700	−64.099	1.00	41.21	O
ATOM	8167	HN	GLU	B	152	23.843	−0.082	−60.073	1.00	41.12	H
ATOM	8168	HA	GLU	B	152	25.801	−1.825	−61.485	1.00	41.12	H
ATOM	8169	HB1	GLU	B	152	23.087	−1.970	−62.172	1.00	41.12	H
ATOM	8170	HB2	GLU	B	152	23.784	−0.836	−63.355	1.00	41.12	H
ATOM	8171	HG1	GLU	B	152	24.937	−3.554	−62.765	1.00	41.12	H
ATOM	8172	HG2	GLU	B	152	23.736	−3.242	−64.042	1.00	41.12	H
ATOM	8173	N	GLY	B	153	26.756	0.337	−62.342	1.00	26.97	N
ATOM	8174	CA	GLY	B	153	27.411	1.604	−62.652	1.00	26.98	C
ATOM	8175	C	GLY	B	153	28.032	2.228	−61.419	1.00	27.02	C
ATOM	8176	O	GLY	B	153	28.946	1.642	−60.795	1.00	27.05	O
ATOM	8177	HN	GLY	B	153	27.312	−0.570	−62.456	1.00	27.01	H
ATOM	8178	HA1	GLY	B	153	28.193	1.426	−63.390	1.00	27.01	H
ATOM	8179	HA2	GLY	B	153	26.673	2.292	−63.063	1.00	27.01	H
ATOM	8180	N	GLY	B	154	27.543	3.430	−61.102	1.00	51.59	N
ATOM	8181	CA	GLY	B	154	27.942	4.166	−59.902	1.00	51.64	C
ATOM	8182	C	GLY	B	154	29.330	4.795	−59.904	1.00	51.73	C
ATOM	8183	O	GLY	B	154	29.902	5.073	−60.974	1.00	51.76	O
ATOM	8184	HN	GLY	B	154	26.825	3.876	−61.757	1.00	51.68	H
ATOM	8185	HA1	GLY	B	154	27.218	4.967	−59.753	1.00	51.68	H
ATOM	8186	HA2	GLY	B	154	27.897	3.473	−59.062	1.00	51.68	H
ATOM	8187	N	VAL	B	155	29.867	5.006	−58.700	1.00	27.21	N
ATOM	8188	CA	VAL	B	155	31.159	5.652	−58.520	1.00	27.31	C
ATOM	8189	C	VAL	B	155	32.311	4.656	−58.524	1.00	27.32	C
ATOM	8190	O	VAL	B	155	33.446	5.055	−58.365	1.00	27.41	O

ATOM	8191	CB	VAL	B	155	31.177	6.430	−57.202	1.00	27.38	C
ATOM	8192	CG1	VAL	B	155	29.933	7.321	−57.071	1.00	27.40	C
ATOM	8193	CG2	VAL	B	155	31.258	5.471	−56.018	1.00	27.32	C
ATOM	8194	HN	VAL	B	155	29.326	4.688	−57.833	1.00	27.34	H
ATOM	8195	HA	VAL	B	155	31.309	6.356	−59.338	1.00	27.34	H
ATOM	8196	HB	VAL	B	155	32.061	7.068	−57.190	1.00	27.34	H
ATOM	8197	1HG1	VAL	B	155	29.971	7.862	−56.126	1.00	27.34	H
ATOM	8198	2HG1	VAL	B	155	29.037	6.701	−57.098	1.00	27.34	H
ATOM	8199	3HG1	VAL	B	155	29.907	8.033	−57.896	1.00	27.34	H
ATOM	8200	1HG2	VAL	B	155	31.270	6.040	−55.089	1.00	27.34	H
ATOM	8201	2HG2	VAL	B	155	32.170	4.878	−56.092	1.00	27.34	H
ATOM	8202	3HG2	VAL	B	155	30.392	4.809	−56.027	1.00	27.34	H
ATOM	8203	N	CYS	B	156	31.998	3.371	−58.660	1.00	36.62	N
ATOM	8204	CA	CYS	B	156	32.976	2.309	−58.919	1.00	36.63	C
ATOM	8205	C	CYS	B	156	33.490	2.416	−60.375	1.00	36.69	C
ATOM	8206	O	CYS	B	156	34.682	2.236	−60.670	1.00	36.78	O
ATOM	8207	CB	CYS	B	156	32.265	0.955	−58.665	1.00	36.55	C
ATOM	8208	SG	CYS	B	156	33.012	−0.698	−59.139	1.00	36.58	S
ATOM	8209	HN	CYS	B	156	30.968	3.094	−58.575	1.00	36.64	H
ATOM	8210	HA	CYS	B	156	33.814	2.413	−58.230	1.00	36.64	H
ATOM	8211	HB1	CYS	B	156	32.087	0.907	−57.591	1.00	36.64	H
ATOM	8212	HB2	CYS	B	156	31.311	1.014	−59.189	1.00	36.64	H
ATOM	8213	N	GLN	B	157	32.568	2.750	−61.274	1.00	50.23	N
ATOM	8214	CA	GLN	B	157	32.764	2.660	−62.710	1.00	50.26	C
ATOM	8215	C	GLN	B	157	32.935	4.025	−63.367	1.00	50.31	C
ATOM	8216	O	GLN	B	157	32.888	4.135	−64.581	1.00	50.32	O
ATOM	8217	CB	GLN	B	157	31.516	2.003	−63.307	1.00	50.16	C

ATOM	8218	CG	GLN	B	157	31.690	1.443	−64.713	1.00	50.18	C
ATOM	8219	CD	GLN	B	157	30.377	1.300	−65.511	1.00	50.10	C
ATOM	8220	OE1	GLN	B	157	29.399	0.719	−65.030	1.00	50.04	O
ATOM	8221	NE2	GLN	B	157	30.382	1.792	−66.756	1.00	50.10	N
ATOM	8222	HN	GLN	B	157	31.624	3.107	−60.917	1.00	50.19	H
ATOM	8223	HA	GLN	B	157	33.637	2.042	−62.921	1.00	50.19	H
ATOM	8224	HB1	GLN	B	157	31.220	1.184	−62.652	1.00	50.19	H
ATOM	8225	HB2	GLN	B	157	30.723	2.750	−63.337	1.00	50.19	H
ATOM	8226	HG1	GLN	B	157	32.353	2.109	−65.265	1.00	50.19	H
ATOM	8227	HG2	GLN	B	157	32.149	0.457	−64.633	1.00	50.19	H
ATOM	8228	1HE2	GLN	B	157	31.266	2.253	−67.146	1.00	50.19	H
ATOM	8229	2HE2	GLN	B	157	29.502	1.724	−67.362	1.00	50.19	H
ATOM	8230	N	THR	B	158	33.119	5.085	−62.594	1.00	27.41	N
ATOM	8231	CA	THR	B	158	33.163	6.423	−63.206	1.00	27.48	C
ATOM	8232	C	THR	B	158	34.036	7.332	−62.348	1.00	27.62	C
ATOM	8233	O	THR	B	158	34.845	8.103	−62.892	1.00	27.75	O
ATOM	8234	CB	THR	B	158	31.766	6.944	−63.470	1.00	27.40	C
ATOM	8235	OG1	THR	B	158	31.063	6.995	−62.229	1.00	27.36	O
ATOM	8236	CG2	THR	B	158	31.021	6.010	−64.452	1.00	27.28	C
ATOM	8237	HN	THR	B	158	33.234	4.970	−61.536	1.00	27.47	H
ATOM	8238	HA	THR	B	158	33.657	6.320	−64.172	1.00	27.47	H
ATOM	8239	HB	THR	B	158	31.826	7.946	−63.895	1.00	27.47	H
ATOM	8240	HG1	THR	B	158	31.015	6.032	−61.814	1.00	27.47	H
ATOM	8241	1HG2	THR	B	158	30.018	6.397	−64.632	1.00	27.47	H
ATOM	8242	2HG2	THR	B	158	30.952	5.011	−64.022	1.00	27.47	H
ATOM	8243	3HG2	THR	B	158	31.567	5.963	−65.394	1.00	27.47	H
ATOM	8244	N	ARG	B	159	33.911	7.213	−61.020	1.00	27.60	N

ATOM	8245	CA	ARG	B	159	35.115	7.324	−60.186	1.00	27.71	C
ATOM	8246	C	ARG	B	159	35.516	5.858	−59.979	1.00	27.63	C
ATOM	8247	O	ARG	B	159	34.706	4.951	−60.235	1.00	27.51	O
ATOM	8248	CB	ARG	B	159	34.893	8.071	−58.866	1.00	27.76	C
ATOM	8249	CG	ARG	B	159	35.938	9.168	−58.618	1.00	27.96	C
ATOM	8250	CD	ARG	B	159	35.629	9.970	−57.351	1.00	28.03	C
ATOM	8251	NE	ARG	B	159	34.849	11.193	−57.589	1.00	28.12	N
ATOM	8252	CZ	ARG	B	159	35.365	12.396	−57.849	1.00	28.32	C
ATOM	8253	NH1	ARG	B	159	36.672	12.581	−57.928	1.00	28.47	N
ATOM	8254	NH2	ARG	B	159	34.569	13.431	−58.042	1.00	28.40	N
ATOM	8255	HN	ARG	B	159	32.953	7.041	−60.575	1.00	27.96	H
ATOM	8256	HA	ARG	B	159	35.896	7.830	−60.754	1.00	27.96	H
ATOM	8257	HB1	ARG	B	159	33.905	8.531	−58.888	1.00	27.96	H
ATOM	8258	HB2	ARG	B	159	34.942	7.353	−58.048	1.00	27.96	H
ATOM	8259	HG1	ARG	B	159	36.918	8.704	−58.512	1.00	27.96	H
ATOM	8260	HG2	ARG	B	159	35.947	9.846	−59.471	1.00	27.96	H
ATOM	8261	HD1	ARG	B	159	35.065	9.331	−56.671	1.00	27.96	H
ATOM	8262	HD2	ARG	B	159	36.574	10.252	−56.886	1.00	27.96	H
ATOM	8263	HE	ARG	B	159	33.783	11.112	−57.551	1.00	27.96	H
ATOM	8264	1HH1	ARG	B	159	37.339	11.757	−57.782	1.00	27.96	H
ATOM	8265	2HH1	ARG	B	159	37.063	13.555	−58.138	1.00	27.96	H
ATOM	8266	1HH2	ARG	B	159	33.508	13.307	−57.990	1.00	27.96	H
ATOM	8267	2HH2	ARG	B	159	34.985	14.394	−58.250	1.00	27.96	H
ATOM	8268	N	ALA	B	160	36.763	5.627	−59.574	1.00	27.72	N
ATOM	8269	CA	ALA	B	160	37.261	4.286	−59.269	1.00	27.67	C
ATOM	8270	C	ALA	B	160	37.138	4.006	−57.764	1.00	27.61	C
ATOM	8271	O	ALA	B	160	38.081	3.553	−57.122	1.00	27.64	O

ATOM	8272	CB	ALA	B	160	38.703	4.149	−59.745	1.00	27.81	C
ATOM	8273	HN	ALA	B	160	37.429	6.458	−59.467	1.00	27.69	H
ATOM	8274	HA	ALA	B	160	36.650	3.561	−59.806	1.00	27.69	H
ATOM	8275	HB1	ALA	B	160	39.069	3.148	−59.515	1.00	27.69	H
ATOM	8276	HB2	ALA	B	160	39.325	4.887	−59.239	1.00	27.69	H
ATOM	8277	HB3	ALA	B	160	38.748	4.313	−60.822	1.00	27.69	H
ATOM	8278	N	MET	B	161	35.964	4.310	−57.214	1.00	27.53	N
ATOM	8279	CA	MET	B	161	35.635	3.992	−55.835	1.00	27.46	C
ATOM	8280	C	MET	B	161	35.528	2.478	−55.669	1.00	27.35	C
ATOM	8281	O	MET	B	161	34.439	1.897	−55.848	1.00	27.27	O
ATOM	8282	CB	MET	B	161	34.298	4.617	−55.437	1.00	27.41	C
ATOM	8283	CG	MET	B	161	34.371	6.054	−54.992	1.00	27.53	C
ATOM	8284	SD	MET	B	161	33.560	6.095	−53.391	1.00	27.48	S
ATOM	8285	CE	MET	B	161	34.649	7.195	−52.527	1.00	27.64	C
ATOM	8286	HN	MET	B	161	35.232	4.810	−57.813	1.00	27.46	H
ATOM	8287	HA	MET	B	161	36.419	4.372	−55.180	1.00	27.46	H
ATOM	8288	HB1	MET	B	161	33.631	4.564	−56.298	1.00	27.46	H
ATOM	8289	HB2	MET	B	161	33.882	4.031	−54.618	1.00	27.46	H
ATOM	8290	HG1	MET	B	161	35.409	6.374	−54.902	1.00	27.46	H
ATOM	8291	HG2	MET	B	161	33.851	6.700	−55.700	1.00	27.46	H
ATOM	8292	HE1	MET	B	161	34.292	7.334	−51.507	1.00	27.46	H
ATOM	8293	HE2	MET	B	161	34.673	8.158	−53.037	1.00	27.46	H
ATOM	8294	HE3	MET	B	161	35.653	6.771	−52.505	1.00	27.46	H
ATOM	8295	N	LYS	B	162	36.674	1.868	−55.338	1.00	37.41	N
ATOM	8296	CA	LYS	B	162	36.831	0.405	−55.218	1.00	37.35	C
ATOM	8297	C	LYS	B	162	38.155	0.083	−54.510	1.00	37.40	C
ATOM	8298	O	LYS	B	162	39.235	0.446	−54.995	1.00	37.52	O

ATOM 8299 CB LYS B 162 36.778 −0.287 −56.613 1.00 37.38 C

ATOM 8300 CG LYS B 162 37.892 0.135 −57.571 1.00 37.51 C

ATOM 8301 CD LYS B 162 37.432 0.220 −58.993 1.00 37.53 C

ATOM 8302 CE LYS B 162 37.721 −1.055 −59.763 1.00 37.57 C

ATOM 8303 NZ LYS B 162 37.441 −0.872 −61.218 1.00 37.61 N1+

ATOM 8304 HN LYS B 162 37.531 2.481 −55.148 1.00 37.48 H

ATOM 8305 HA LYS B 162 36.012 0.019 −54.610 1.00 37.48 H

ATOM 8306 HB1 LYS B 162 36.849 −1.364 −56.462 1.00 37.48 H

ATOM 8307 HB2 LYS B 162 35.821 −0.047 −57.076 1.00 37.48 H

ATOM 8308 HG1 LYS B 162 38.262 1.114 −57.266 1.00 37.48 H

ATOM 8309 HG2 LYS B 162 38.699 −0.595 −57.509 1.00 37.48 H

ATOM 8310 HD1 LYS B 162 36.357 0.402 −59.004 1.00 37.48 H

ATOM 8311 HD2 LYS B 162 37.946 1.049 −59.480 1.00 37.48 H

ATOM 8312 HE1 LYS B 162 38.770 −1.321 −59.632 1.00 37.48 H

ATOM 8313 HE2 LYS B 162 37.091 −1.856 −59.376 1.00 37.48 H

ATOM 8314 HZ1 LYS B 162 37.652 −1.782 −61.739 1.00 37.48 H

ATOM 8315 HZ2 LYS B 162 38.059 −0.089 −61.605 1.00 37.48 H

ATOM 8316 HZ3 LYS B 162 36.412 −0.614 −61.354 1.00 37.48 H

ATOM 8317 N ILE B 163 38.094 −0.592 −53.371 1.00 27.30 N

ATOM 8318 CA ILE B 163 39.331 −0.981 −52.711 1.00 27.34 C

ATOM 8319 C ILE B 163 39.722 −2.349 −53.247 1.00 27.35 C

ATOM 8320 O ILE B 163 38.879 −3.240 −53.323 1.00 27.29 O

ATOM 8321 CB ILE B 163 39.200 −0.965 −51.157 1.00 27.25 C

ATOM 8322 CG1 ILE B 163 38.616 0.354 −50.694 1.00 27.25 C

ATOM 8323 CG2 ILE B 163 40.540 −1.141 −50.480 1.00 27.29 C

ATOM 8324 CD1 ILE B 163 38.809 0.589 −49.249 1.00 27.21 C

ATOM 8325 HN ILE B 163 37.147 −0.848 −52.942 1.00 27.28 H

ATOM 8326 HA ILE B 163 40.107 −0.270 −52.995 1.00 27.28 H
ATOM 8327 HB ILE B 163 38.538 −1.774 −50.849 1.00 27.28 H
ATOM 8328 1HG1 ILE B 163 39.098 1.161 −51.246 1.00 27.29 H
ATOM 8329 2HG1 ILE B 163 37.547 0.356 −50.907 1.00 27.29 H
ATOM 8330 1HG2 ILE B 163 40.406 −1.124 −49.398 1.00 27.29 H
ATOM 8331 2HG2 ILE B 163 41.206 −0.331 −50.776 1.00 27.29 H
ATOM 8332 3HG2 ILE B 163 40.975 −2.095 −50.777 1.00 27.29 H
ATOM 8333 1HD1 ILE B 163 38.371 1.548 −48.975 1.00 27.29 H
ATOM 8334 2HD1 ILE B 163 39.875 0.599 −49.021 1.00 27.29 H
ATOM 8335 3HD1 ILE B 163 38.324 −0.207 −48.683 1.00 27.29 H
ATOM 8336 N LEU B 164 40.976 −2.493 −53.673 1.00 30.73 N
ATOM 8337 CA LEU B 164 41.593 −3.829 −53.848 1.00 30.77 C
ATOM 8338 C LEU B 164 42.534 −4.156 −52.643 1.00 30.76 C
ATOM 8339 O LEU B 164 43.562 −3.485 −52.405 1.00 30.84 O
ATOM 8340 CB LEU B 164 42.344 −3.979 −55.185 1.00 30.93 C
ATOM 8341 CG LEU B 164 43.205 −5.256 −55.369 1.00 31.03 C
ATOM 8342 CD1 LEU B 164 42.532 −6.568 −54.955 1.00 30.96 C
ATOM 8343 CD2 LEU B 164 43.648 −5.355 −56.807 1.00 31.20 C
ATOM 8344 HN LEU B 164 41.554 −1.620 −53.895 1.00 30.90 H
ATOM 8345 HA LEU B 164 40.788 −4.564 −53.842 1.00 30.90 H
ATOM 8346 HB1 LEU B 164 41.601 −3.965 −55.983 1.00 30.90 H
ATOM 8347 HB2 LEU B 164 43.005 −3.119 −55.289 1.00 30.90 H
ATOM 8348 HG LEU B 164 44.100 −5.139 −54.758 1.00 30.90 H
ATOM 8349 1HD1 LEU B 164 43.218 −7.398 −55.124 1.00 30.90 H
ATOM 8350 2HD1 LEU B 164 41.630 −6.717 −55.548 1.00 30.90 H
ATOM 8351 3HD1 LEU B 164 42.268 −6.524 −53.898 1.00 30.90 H
ATOM 8352 1HD2 LEU B 164 44.254 −6.251 −56.940 1.00 30.90 H

ATOM 8353 2HD2 LEU B 164 44.238 −4.476 −57.067 1.00 30.90 H

ATOM 8354 3HD2 LEU B 164 42.773 −5.409 −57.454 1.00 30.90 H

ATOM 8355 N MET B 165 42.168 −5.196 −51.894 1.00 27.40 N

ATOM 8356 CA MET B 165 42.939 −5.617 −50.750 1.00 27.36 C

ATOM 8357 C MET B 165 43.815 −6.776 −51.176 1.00 27.47 C

ATOM 8358 O MET B 165 43.306 −7.884 −51.313 1.00 27.46 O

ATOM 8359 CB MET B 165 41.993 −6.085 −49.643 1.00 27.20 C

ATOM 8360 CG MET B 165 41.354 −4.981 −48.797 1.00 27.11 C

ATOM 8361 SD MET B 165 40.278 −5.619 −47.476 1.00 26.94 S

ATOM 8362 CE MET B 165 38.761 −5.882 −48.377 1.00 26.94 C

ATOM 8363 HN MET B 165 41.278 −5.733 −52.148 1.00 27.24 H

ATOM 8364 HA MET B 165 43.556 −4.793 −50.391 1.00 27.24 H

ATOM 8365 HB1 MET B 165 41.190 −6.655 −50.110 1.00 27.24 H

ATOM 8366 HB2 MET B 165 42.558 −6.734 −48.973 1.00 27.24 H

ATOM 8367 HG1 MET B 165 42.149 −4.391 −48.341 1.00 27.24 H

ATOM 8368 HG2 MET B 165 40.758 −4.345 −49.451 1.00 27.24 H

ATOM 8369 HE1 MET B 165 38.000 −6.274 −47.702 1.00 27.23 H

ATOM 8370 HE2 MET B 165 38.935 −6.597 −49.181 1.00 27.23 H

ATOM 8371 HE3 MET B 165 38.420 −4.937 −48.799 1.00 27.23 H

ATOM 8372 N LYS B 166 45.113 −6.536 −51.390 1.00 27.60 N

ATOM 8373 CA LYS B 166 46.080 −7.617 −51.729 1.00 27.73 C

ATOM 8374 C LYS B 166 46.539 −8.292 −50.445 1.00 27.64 C

ATOM 8375 O LYS B 166 47.482 −7.838 −49.797 1.00 27.65 O

ATOM 8376 CB LYS B 166 47.325 −7.078 −52.446 1.00 27.92 C

ATOM 8377 CG LYS B 166 47.082 −6.255 −53.729 1.00 28.02 C

ATOM 8378 CD LYS B 166 47.596 −6.959 −55.006 1.00 28.22 C

ATOM 8379 CE LYS B 166 46.466 −7.593 −55.766 1.00 28.19 C

ATOM	8380	NZ	LYS	B	166	45.799	−6.560	−56.596	1.00	28.17		N1+
ATOM	8381	HN	LYS	B	166	45.470	−5.530	−51.315	1.00	27.90		H
ATOM	8382	HA	LYS	B	166	45.588	−8.352	−52.366	1.00	27.90		H
ATOM	8383	HB1	LYS	B	166	47.862	−6.442	−51.742	1.00	27.90		H
ATOM	8384	HB2	LYS	B	166	47.948	−7.932	−52.714	1.00	27.90		H
ATOM	8385	HG1	LYS	B	166	46.011	−6.084	−53.834	1.00	27.90		H
ATOM	8386	HG2	LYS	B	166	47.595	−5.298	−53.630	1.00	27.90		H
ATOM	8387	HD1	LYS	B	166	48.086	−6.224	−55.645	1.00	27.90		H
ATOM	8388	HD2	LYS	B	166	48.312	−7.731	−54.723	1.00	27.90		H
ATOM	8389	HE1	LYS	B	166	46.856	−8.382	−56.409	1.00	27.90		H
ATOM	8390	HE2	LYS	B	166	45.748	−8.017	−55.065	1.00	27.90		H
ATOM	8391	HZ1	LYS	B	166	44.993	−7.005	−57.140	1.00	27.90		H
ATOM	8392	HZ2	LYS	B	166	46.505	−6.143	−57.283	1.00	27.90		H
ATOM	8393	HZ3	LYS	B	166	45.417	−5.786	−55.964	1.00	27.90		H
ATOM	8394	N	VAL	B	167	45.856	−9.360	−50.048	1.00	65.77		N
ATOM	8395	CA	VAL	B	167	46.125	−9.959	−48.729	1.00	65.66		C
ATOM	8396	C	VAL	B	167	47.397	−10.805	−48.794	1.00	65.79		C
ATOM	8397	O	VAL	B	167	47.704	−11.416	−49.852	1.00	65.97		O
ATOM	8398	CB	VAL	B	167	44.918	−10.768	−48.161	1.00	65.53		C
ATOM	8399	CG1	VAL	B	167	45.386	−11.958	−47.272	1.00	65.50		C
ATOM	8400	CG2	VAL	B	167	44.015	−9.822	−47.379	1.00	65.36		C
ATOM	8401	HN	VAL	B	167	45.110	−9.785	−50.686	1.00	65.65		H
ATOM	8402	HA	VAL	B	167	46.318	−9.140	−48.036	1.00	65.65		H
ATOM	8403	HB	VAL	B	167	44.349	−11.170	−49.000	1.00	65.65		H
ATOM	8404	1HG1	VAL	B	167	44.516	−12.496	−46.896	1.00	65.65		H
ATOM	8405	2HG1	VAL	B	167	45.968	−11.578	−46.432	1.00	65.65		H
ATOM	8406	3HG1	VAL	B	167	46.002	−12.634	−47.864	1.00	65.65		H

```
ATOM   8407 1HG2 VAL B 167      43.167 −10.377 −46.977  1.00 65.65           H
ATOM   8408 2HG2 VAL B 167      43.653  −9.035 −48.041  1.00 65.65           H
ATOM   8409 3HG2 VAL B 167      44.578  −9.376 −46.559  1.00 65.65           H
ATOM   8410  N   GLY B 168      48.129 −10.808 −47.672  1.00 27.51           N
ATOM   8411  CA  GLY B 168      49.370 −11.557 −47.556  1.00 27.62           C
ATOM   8412  C   GLY B 168      50.449 −10.957 −48.447  1.00 27.83           C
ATOM   8413  O   GLY B 168      50.332 −10.914 −49.686  1.00 27.98           O
ATOM   8414  HN  GLY B 168      47.789 −10.239 −46.832  1.00 27.74           H
ATOM   8415  HA1 GLY B 168      49.707 −11.531 −46.520  1.00 27.74           H
ATOM   8416  HA2 GLY B 168      49.193 −12.590 −47.855  1.00 27.74           H
ATOM   8417  HC  GLY B 168      51.351 −10.553 −47.987  1.00 27.74           H
TER    8418      GLY B 168
HETATM    1  C1  LIG d   0      28.952 −17.994 −35.602  0.00  0.00           C
HETATM    2  C2  LIG d   0      29.030 −18.924 −36.788  0.00  0.00           C
HETATM    3  N4  LIG d   0      29.730 −18.444 −37.918  0.00  0.00           N
HETATM    4  C11 LIG d   0      29.479 −16.759 −35.727  0.00  0.00           C
HETATM    5  C14 LIG d   0      30.236 −17.153 −37.969  0.00  0.00           C
HETATM    6  O15 LIG d   0      28.529 −20.047 −36.721  0.00  0.00           O
HETATM    7  C16 LIG d   0      30.112 −16.316 −36.939  0.00  0.00           C
HETATM    8  C8  LIG d   0      29.949 −19.301 −39.114  0.00  0.00           C
HETATM    9  C5  LIG d   0      30.537 −20.692 −38.763  0.00  0.00           C
HETATM   10  N3  LIG d   0      29.899 −21.875 −39.196  0.00  0.00           N
HETATM   11  C6  LIG d   0      28.589 −21.959 −39.896  0.00  0.00           C
HETATM   12  C9  LIG d   0      30.342 −23.090 −38.496  0.00  0.00           C
HETATM   13  C13 LIG d   0      28.176 −23.414 −40.346  0.00  0.00           C
HETATM   14  N10 LIG d   0      28.501 −24.510 −39.406  0.00  0.00           N
HETATM   15  C20 LIG d   0      29.950 −24.426 −39.142  0.00  0.00           C
```

HETATM 16 C23 LIG d 0 28.146 −25.813 −39.976 0.00 0.00 C

HETATM 17 O17 LIG d 0 31.583 −20.749 −38.103 0.00 0.00 O

HETATM 18 C18 LIG d 0 28.493 −21.103 −41.156 0.00 0.00 C

HETATM 19 C24 LIG d 0 29.398 −21.318 −42.210 0.00 0.00 C

HETATM 20 C28 LIG d 0 29.358 −20.523 −43.356 0.00 0.00 C

HETATM 21 C33 LIG d 0 28.413 −19.506 −43.464 0.00 0.00 C

HETATM 22 C29 LIG d 0 27.521 −19.268 −42.420 0.00 0.00 C

HETATM 23 C25 LIG d 0 27.569 −20.051 −41.266 0.00 0.00 C

HETATM 24 C7 LIG d 0 28.424 −18.327 −34.232 0.00 0.00 C

HETATM 25 N12 LIG d 0 27.348 −19.173 −34.116 0.00 0.00 N

HETATM 26 H7 LIG d 0 26.937 −19.540 −34.971 0.00 0.00 H

HETATM 27 O19 LIG d 0 28.961 −17.857 −33.225 0.00 0.00 O

HETATM 28 C21 LIG d 0 26.870 −19.666 −32.832 0.00 0.00 C

HETATM 29 C22 LIG d 0 25.909 −20.825 −32.931 0.00 0.00 C

HETATM 30 C26 LIG d 0 25.298 −21.141 −34.155 0.00 0.00 C

HETATM 31 C30 LIG d 0 24.336 −22.151 −34.232 0.00 0.00 C

HETATM 32 C32 LIG d 0 23.985 −22.866 −33.089 0.00 0.00 C

HETATM 33 C31 LIG d 0 24.582 −22.567 −31.865 0.00 0.00 C

HETATM 34 C27 LIG d 0 25.546 −21.557 −31.788 0.00 0.00 C

END

Top2−combine−coordinates

ATOM 1 N ILE A 188 32.653 −16.268 −1.319 1.00 51.04 N1+

ATOM 2 CA ILE A 188 32.633 −15.941 −2.763 1.00 51.24 C

ATOM 3 C ILE A 188 32.837 −14.441 −2.909 1.00 51.38 C

ATOM 4 O ILE A 188 32.290 −13.705 −2.093 1.00 51.55 O

ATOM 5 CB ILE A 188 31.347 −16.438 −3.468 1.00 51.49 C

ATOM 6 CG1 ILE A 188 31.341 −16.009 −4.953 1.00 51.69 C

ATOM 7 CG2 ILE A 188 30.059 −15.978 −2.757 1.00 51.70 C

ATOM 8 CD1 ILE A 188 30.325 −16.768 −5.817 1.00 51.91 C

ATOM 9 1HD1 ILE A 188 30.420 −16.435 −6.849 1.00 0.00 H

ATOM 10 2HD1 ILE A 188 30.514 −17.841 −5.770 1.00 0.00 H

ATOM 11 H1 ILE A 188 32.449 −17.244 −1.163 1.00 0.00 H

ATOM 12 H2 ILE A 188 31.970 −15.687 −0.848 1.00 0.00 H

ATOM 13 H3 ILE A 188 33.568 −16.041 −0.940 1.00 0.00 H

ATOM 14 N CYS A 189 33.675 −13.977 −3.840 1.00 23.07 N

ATOM 15 CA CYS A 189 33.809 −12.535 −4.055 1.00 23.21 C

ATOM 16 C CYS A 189 32.574 −11.948 −4.761 1.00 23.54 C

ATOM 17 O CYS A 189 32.343 −12.226 −5.937 1.00 23.69 O

ATOM 18 CB CYS A 189 35.092 −12.191 −4.815 1.00 23.15 C

ATOM 19 SG CYS A 189 35.277 −10.404 −5.017 1.00 23.21 S

ATOM 20 H CYS A 189 34.138 −14.619 −4.473 1.00 0.00 H

ATOM 21 N LEU A 190 31.841 −11.084 −4.056 1.00 50.65 N

ATOM 22 CA LEU A 190 30.699 −10.317 −4.576 1.00 50.98 C

ATOM 23 C LEU A 190 31.032 −8.834 −4.838 1.00 51.13 C

ATOM 24 O LEU A 190 30.234 −8.131 −5.451 1.00 51.41 O

ATOM 25 CB LEU A 190 29.536 −10.434 −3.572 1.00 51.07 C

ATOM 26 CG LEU A 190 29.029 −11.868 −3.324 1.00 51.02 C

ATOM 27 CD1 LEU A 190 28.068 −11.866 −2.138 1.00 51.16 C

ATOM 28 CD2 LEU A 190 28.323 −12.447 −4.551 1.00 51.19 C

ATOM 29 H LEU A 190 32.062 −10.971 −3.078 1.00 0.00 H

ATOM 30 N GLN A 191 32.174 −8.356 −4.332 1.00 23.97 N

ATOM 31 CA GLN A 191 32.625 −6.966 −4.438 1.00 24.11 C

ATOM 32 C GLN A 191 33.318 −6.704 −5.783 1.00 24.14 C

ATOM 33 O GLN A 191 34.098 −7.532 −6.258 1.00 23.95 O

```
ATOM     34  CB  GLN A 191      33.568  −6.659  −3.256  1.00 23.92           C
ATOM     35  CG  GLN A 191      33.907  −5.174  −3.041  1.00 24.14           C
ATOM     36  CD  GLN A 191      32.715  −4.319  −2.625  1.00 24.38           C
ATOM     37  OE1 GLN A 191      31.658  −4.797  −2.257  1.00 24.39           O
ATOM     38  NE2 GLN A 191      32.833  −3.013  −2.682  1.00 24.59           N
ATOM     39  H   GLN A 191      32.809  −9.008  −3.905  1.00  0.00           H
ATOM     40 1HE2 GLN A 191      33.685  −2.587  −2.999  1.00  0.00           H
ATOM     41 2HE2 GLN A 191      32.019  −2.489  −2.414  1.00  0.00           H
ATOM     42  N   LYS A 192      33.179  −5.485  −6.311  1.00 24.41           N
ATOM     43  CA  LYS A 192      33.993  −4.990  −7.427  1.00 24.45           C
ATOM     44  C   LYS A 192      35.463  −4.805  −7.021  1.00 24.22           C
ATOM     45  O   LYS A 192      35.744  −4.157  −6.016  1.00 24.15           O
ATOM     46  CB  LYS A 192      33.343  −3.703  −7.956  1.00 24.77           C
ATOM     47  CG  LYS A 192      34.110  −3.120  −9.144  1.00 24.86           C
ATOM     48  CD  LYS A 192      33.252  −2.138  −9.952  1.00 25.21           C
ATOM     49  CE  LYS A 192      34.025  −1.589 −11.158  1.00 25.32           C
ATOM     50  NZ  LYS A 192      34.494  −2.678 −12.052  1.00 25.19           N1+
ATOM     51  H   LYS A 192      32.506  −4.854  −5.897  1.00  0.00           H
ATOM     52  HZ1 LYS A 192      34.932  −2.323 −12.889  1.00  0.00           H
ATOM     53  HZ2 LYS A 192      35.137  −3.292 −11.566  1.00  0.00           H
ATOM     54  HZ3 LYS A 192      33.708  −3.272 −12.317  1.00  0.00           H
ATOM     55  N   THR A 193      36.402  −5.279  −7.844  1.00 27.90           N
ATOM     56  CA  THR A 193      37.856  −5.126  −7.630  1.00 27.72           C
ATOM     57  C   THR A 193      38.646  −5.237  −8.944  1.00 27.67           C
ATOM     58  O   THR A 193      38.110  −5.652  −9.971  1.00 27.64           O
ATOM     59  CB  THR A 193      38.375  −6.122  −6.569  1.00 27.43           C
ATOM     60  OG1 THR A 193      39.738  −5.865  −6.316  1.00 27.25           O
```

```
ATOM     61  CG2 THR A 193      38.269  −7.592  −6.974  1.00 27.28           C
ATOM     62  H   THR A 193      36.107  −5.742  −8.700  1.00  0.00           H
ATOM     63  HG1 THR A 193      40.069  −6.475  −5.641  1.00  0.00           H
ATOM     64  N   THR A 194      39.918  −4.835  −8.924  1.00 23.90           N
ATOM     65  CA  THR A 194      40.904  −5.065  −9.996  1.00 23.87           C
ATOM     66  C   THR A 194      41.966  −6.108  −9.622  1.00 23.58           C
ATOM     67  O   THR A 194      42.909  −6.306 −10.383  1.00 23.54           O
ATOM     68  CB  THR A 194      41.565  −3.750 −10.439  1.00 24.10           C
ATOM     69  OG1 THR A 194      42.236  −3.148  −9.357  1.00 24.17           O
ATOM     70  CG2 THR A 194      40.543  −2.746 −10.975  1.00 24.37           C
ATOM     71  H   THR A 194      40.283  −4.513  −8.035  1.00  0.00           H
ATOM     72  HG1 THR A 194      43.081  −3.597  −9.254  1.00  0.00           H
ATOM     73  N   SER A 195      41.831  −6.792  −8.479  1.00 23.39           N
ATOM     74  CA  SER A 195      42.647  −7.967  −8.139  1.00 23.10           C
ATOM     75  C   SER A 195      42.491  −9.069  −9.196  1.00 23.09           C
ATOM     76  O   SER A 195      41.382  −9.338  −9.659  1.00 23.24           O
ATOM     77  CB  SER A 195      42.241  −8.541  −6.780  1.00 22.92           C
ATOM     78  OG  SER A 195      42.255  −7.570  −5.754  1.00 22.92           O
ATOM     79  H   SER A 195      41.086  −6.545  −7.842  1.00  0.00           H
ATOM     80  HG  SER A 195      42.543  −8.039  −4.942  1.00  0.00           H
ATOM     81  N   THR A 196      43.545  −9.851  −9.436  1.00 41.71           N
ATOM     82  CA  THR A 196      43.582 −10.945 −10.433  1.00 41.69           C
ATOM     83  C   THR A 196      42.945 −12.254  −9.931  1.00 41.49           C
ATOM     84  O   THR A 196      43.398 −13.354 −10.246  1.00 41.34           O
ATOM     85  CB  THR A 196      45.010 −11.166 −10.966  1.00 41.65           C
ATOM     86  OG1 THR A 196      45.888 −11.607  −9.943  1.00 41.41           O
ATOM     87  CG2 THR A 196      45.594  −9.902 −11.599  1.00 41.87           C
```

ATOM 88 H THR A 196 44.358 −9.740 −8.843 1.00 0.00 H

ATOM 89 HG1 THR A 196 45.580 −11.264 −9.081 1.00 0.00 H

ATOM 90 N ILE A 197 41.897 −12.159 −9.110 1.00 23.24 N

ATOM 91 CA ILE A 197 41.276 −13.273 −8.367 1.00 23.08 C

ATOM 92 C ILE A 197 40.673 −14.378 −9.256 1.00 23.12 C

ATOM 93 O ILE A 197 40.500 −15.519 −8.812 1.00 22.96 O

ATOM 94 CB ILE A 197 40.227 −12.716 −7.379 1.00 23.15 C

ATOM 95 CG1 ILE A 197 39.159 −11.831 −8.065 1.00 23.43 C

ATOM 96 CG2 ILE A 197 40.916 −12.004 −6.207 1.00 23.15 C

ATOM 97 CD1 ILE A 197 38.103 −11.279 −7.101 1.00 23.57 C

ATOM 98 H ILE A 197 41.583 −11.220 −8.901 1.00 0.00 H

ATOM 99 2HG2 ILE A 197 41.559 −12.711 −5.682 1.00 0.00 H

ATOM 100 3HG2 ILE A 197 40.194 −11.632 −5.487 1.00 0.00 H

ATOM 101 N LEU A 198 40.408 −14.076 −10.529 1.00 22.85 N

ATOM 102 CA LEU A 198 40.169 −15.065 −11.579 1.00 22.95 C

ATOM 103 C LEU A 198 41.512 −15.603 −12.095 1.00 22.89 C

ATOM 104 O LEU A 198 42.148 −14.989 −12.945 1.00 22.95 O

ATOM 105 CB LEU A 198 39.368 −14.411 −12.718 1.00 23.21 C

ATOM 106 CG LEU A 198 37.928 −14.021 −12.357 1.00 23.35 C

ATOM 107 CD1 LEU A 198 37.405 −13.062 −13.424 1.00 23.59 C

ATOM 108 CD2 LEU A 198 37.012 −15.243 −12.273 1.00 23.40 C

ATOM 109 H LEU A 198 40.670 −13.148 −10.830 1.00 0.00 H

ATOM 110 N LYS A 199 41.921 −16.783 −11.613 1.00 22.81 N

ATOM 111 CA LYS A 199 43.184 −17.445 −11.998 1.00 22.73 C

ATOM 112 C LYS A 199 42.898 −18.715 −12.827 1.00 22.86 C

ATOM 113 O LYS A 199 42.656 −19.768 −12.220 1.00 22.78 O

ATOM 114 CB LYS A 199 44.060 −17.686 −10.749 1.00 22.47 C

```
ATOM    115  CG  LYS A 199      44.320 −16.380  −9.971  1.00 22.32           C
ATOM    116  CD  LYS A 199      45.403 −16.508  −8.886  1.00 22.05           C
ATOM    117  CE  LYS A 199      45.335 −15.373  −7.849  1.00 21.96           C
ATOM    118  NZ  LYS A 199      45.433 −14.022  −8.445  1.00 22.09           N1+
ATOM    119  H   LYS A 199      41.367 −17.191 −10.876  1.00  0.00           H
ATOM    120  HZ1 LYS A 199      45.122 −13.338  −7.743  1.00  0.00           H
ATOM    121  HZ2 LYS A 199      46.353 −13.750  −8.749  1.00  0.00           H
ATOM    122  HZ3 LYS A 199      44.777 −13.890  −9.207  1.00  0.00           H
ATOM    123  N   PRO A 200      42.721 −18.603 −14.161  1.00 46.45           N
ATOM    124  CA  PRO A 200      42.211 −19.673 −15.031  1.00 46.62           C
ATOM    125  C   PRO A 200      43.206 −20.839 −15.209  1.00 46.57           C
ATOM    126  O   PRO A 200      44.264 −20.883 −14.575  1.00 46.50           O
ATOM    127  CB  PRO A 200      41.911 −18.964 −16.367  1.00 46.83           C
ATOM    128  CG  PRO A 200      42.965 −17.865 −16.425  1.00 46.78           C
ATOM    129  CD  PRO A 200      43.019 −17.421 −14.967  1.00 46.57           C
ATOM    130  N   ARG A 201      42.864 −21.803 −16.072  1.00 32.66           N
ATOM    131  CA  ARG A 201      43.782 −22.766 −16.709  1.00 32.66           C
ATOM    132  C   ARG A 201      43.226 −23.218 −18.056  1.00 32.93           C
ATOM    133  O   ARG A 201      42.065 −23.608 −18.128  1.00 33.05           O
ATOM    134  CB  ARG A 201      44.004 −23.992 −15.797  1.00 32.48           C
ATOM    135  CG  ARG A 201      45.385 −23.988 −15.114  1.00 32.20           C
ATOM    136  CD  ARG A 201      45.286 −24.303 −13.619  1.00 32.00           C
ATOM    137  NE  ARG A 201      44.661 −23.182 −12.895  1.00 31.86           N
ATOM    138  CZ  ARG A 201      44.368 −23.138 −11.612  1.00 31.60           C
ATOM    139  NH1 ARG A 201      44.608 −24.144 −10.816  1.00 31.41           N
ATOM    140  NH2 ARG A 201      43.820 −22.076 −11.098  1.00 31.54           N
ATOM    141  H   ARG A 201      41.900 −21.808 −16.395  1.00  0.00           H
```

ATOM 142 HE ARG A 201 44.493 −22.336 −13.437 1.00 0.00 H

ATOM 143 1HH1 ARG A 201 45.012 −24.978 −11.206 1.00 0.00 H

ATOM 144 2HH1 ARG A 201 44.381 −24.111 −9.841 1.00 0.00 H

ATOM 145 1HH2 ARG A 201 43.557 −21.300 −11.706 1.00 0.00 H

ATOM 146 2HH2 ARG A 201 43.639 −22.022 −10.120 1.00 0.00 H

ATOM 147 N LEU A 202 44.095 −23.335 −19.059 1.00 23.67 N

ATOM 148 CA LEU A 202 43.834 −24.121 −20.266 1.00 23.95 C

ATOM 149 C LEU A 202 43.808 −25.615 −19.898 1.00 24.04 C

ATOM 150 O LEU A 202 44.800 −26.138 −19.397 1.00 23.97 O

ATOM 151 CB LEU A 202 44.930 −23.783 −21.300 1.00 24.03 C

ATOM 152 CG LEU A 202 44.926 −24.630 −22.587 1.00 24.32 C

ATOM 153 CD1 LEU A 202 43.662 −24.422 −23.420 1.00 24.44 C

ATOM 154 CD2 LEU A 202 46.127 −24.262 −23.455 1.00 24.39 C

ATOM 155 H LEU A 202 45.047 −23.052 −18.898 1.00 0.00 H

ATOM 156 N ILE A 203 42.720 −26.314 −20.227 1.00 41.12 N

ATOM 157 CA ILE A 203 42.558 −27.771 −20.036 1.00 41.21 C

ATOM 158 C ILE A 203 42.485 −28.521 −21.382 1.00 41.54 C

ATOM 159 O ILE A 203 41.862 −29.575 −21.499 1.00 41.69 O

ATOM 160 CB ILE A 203 41.384 −28.092 −19.078 1.00 41.12 C

ATOM 161 CG1 ILE A 203 40.023 −27.655 −19.658 1.00 41.28 C

ATOM 162 CG2 ILE A 203 41.614 −27.485 −17.683 1.00 40.79 C

ATOM 163 CD1 ILE A 203 38.851 −28.479 −19.122 1.00 41.28 C

ATOM 164 H ILE A 203 41.935 −25.803 −20.624 1.00 0.00 H

ATOM 165 N SER A 204 43.075 −27.945 −22.436 1.00 45.79 N

ATOM 166 CA SER A 204 42.951 −28.358 −23.845 1.00 46.10 C

ATOM 167 C SER A 204 41.503 −28.349 −24.342 1.00 46.35 C

ATOM 168 O SER A 204 41.101 −27.443 −25.079 1.00 46.59 O

ATOM	169	CB	SER	A	204	43.609	−29.722	−24.096	1.00	46.13	C
ATOM	170	OG	SER	A	204	42.722	−30.771	−23.762	1.00	46.07	O
ATOM	171	H	SER	A	204	43.557	−27.078	−22.251	1.00	0.00	H
ATOM	172	HG	SER	A	204	42.459	−30.658	−22.829	1.00	0.00	H
ATOM	173	N	GLU	A	213	47.851	−30.099	−40.412	1.00	28.22	N
ATOM	174	CA	GLU	A	213	47.876	−29.395	−41.698	1.00	28.20	C
ATOM	175	C	GLU	A	213	46.934	−28.166	−41.645	1.00	28.01	C
ATOM	176	O	GLU	A	213	46.824	−27.560	−40.580	1.00	27.70	O
ATOM	177	CB	GLU	A	213	47.623	−30.413	−42.828	1.00	28.58	C
ATOM	178	CG	GLU	A	213	48.077	−29.927	−44.223	1.00	28.63	C
ATOM	179	CD	GLU	A	213	46.945	−29.883	−45.261	1.00	28.79	C
ATOM	180	OE1	GLU	A	213	45.826	−29.477	−44.874	1.00	29.02	O
ATOM	181	OE2	GLU	A	213	47.247	−30.082	−46.457	1.00	28.69	O
ATOM	182	H	GLU	A	213	47.470	−29.570	−39.634	1.00	0.00	H
ATOM	183	N	GLY	A	214	46.355	−27.704	−42.759	1.00	28.20	N
ATOM	184	CA	GLY	A	214	45.509	−26.507	−42.815	1.00	28.08	C
ATOM	185	C	GLY	A	214	44.053	−26.823	−42.474	1.00	28.09	C
ATOM	186	O	GLY	A	214	43.267	−27.200	−43.347	1.00	28.25	O
ATOM	187	H	GLY	A	214	46.346	−28.301	−43.586	1.00	0.00	H
ATOM	188	N	VAL	A	215	43.654	−26.625	−41.217	1.00	27.92	N
ATOM	189	CA	VAL	A	215	42.374	−27.122	−40.678	1.00	27.94	C
ATOM	190	C	VAL	A	215	41.659	−26.060	−39.842	1.00	27.61	C
ATOM	191	O	VAL	A	215	42.298	−25.317	−39.104	1.00	27.38	O
ATOM	192	CB	VAL	A	215	42.613	−28.437	−39.896	1.00	28.10	C
ATOM	193	CG1	VAL	A	215	41.448	−28.895	−39.004	1.00	27.94	C
ATOM	194	CG2	VAL	A	215	42.909	−29.592	−40.866	1.00	28.50	C
ATOM	195	H	VAL	A	215	44.332	−26.237	−40.561	1.00	0.00	H

ATOM 196 N CYS A 216 40.323 −26.107 −39.822 1.00 35.13 N
ATOM 197 CA CYS A 216 39.522 −25.474 −38.774 1.00 34.87 C
ATOM 198 C CYS A 216 38.521 −26.450 −38.133 1.00 34.94 C
ATOM 199 O CYS A 216 37.795 −27.162 −38.825 1.00 35.18 O
ATOM 200 CB CYS A 216 38.843 −24.203 −39.295 1.00 34.80 C
ATOM 201 SG CYS A 216 37.835 −23.395 −38.019 1.00 34.59 S
ATOM 202 H CYS A 216 39.865 −26.774 −40.433 1.00 0.00 H
ATOM 203 N ILE A 217 38.426 −26.415 −36.805 1.00 27.23 N
ATOM 204 CA ILE A 217 37.384 −27.046 −35.988 1.00 27.24 C
ATOM 205 C ILE A 217 36.337 −25.982 −35.618 1.00 27.06 C
ATOM 206 O ILE A 217 36.694 −24.978 −35.010 1.00 26.81 O
ATOM 207 CB ILE A 217 38.037 −27.659 −34.726 1.00 27.10 C
ATOM 208 CG1 ILE A 217 39.206 −28.632 −35.011 1.00 27.29 C
ATOM 209 CG2 ILE A 217 36.981 −28.338 −33.851 1.00 27.10 C
ATOM 210 CD1 ILE A 217 38.840 −29.866 −35.842 1.00 27.63 C
ATOM 211 H ILE A 217 39.069 −25.793 −36.320 1.00 0.00 H
ATOM 212 N THR A 218 35.061 −26.173 −35.972 1.00 27.21 N
ATOM 213 CA THR A 218 34.011 −25.139 −35.809 1.00 27.08 C
ATOM 214 C THR A 218 32.593 −25.736 −35.676 1.00 27.25 C
ATOM 215 O THR A 218 32.461 −26.906 −35.325 1.00 27.47 O
ATOM 216 CB THR A 218 34.154 −24.107 −36.946 1.00 27.09 C
ATOM 217 OG1 THR A 218 33.341 −22.973 −36.757 1.00 26.93 O
ATOM 218 CG2 THR A 218 33.810 −24.689 −38.316 1.00 27.38 C
ATOM 219 H THR A 218 34.825 −26.999 −36.511 1.00 0.00 H
ATOM 220 HG1 THR A 218 33.615 −22.575 −35.888 1.00 0.00 H
ATOM 221 N ASP A 219 31.544 −24.905 −35.744 1.00 39.43 N
ATOM 222 CA ASP A 219 30.172 −25.150 −35.240 1.00 39.58 C

ATOM	223	C	ASP	A	219	30.130	−25.782	−33.822	1.00	39.54	C
ATOM	224	O	ASP	A	219	29.601	−26.890	−33.654	1.00	39.76	O
ATOM	225	CB	ASP	A	219	29.320	−25.911	−36.276	1.00	39.91	C
ATOM	226	CG	ASP	A	219	27.824	−25.942	−35.911	1.00	40.10	C
ATOM	227	OD1	ASP	A	219	27.267	−24.883	−35.554	1.00	40.00	O
ATOM	228	OD2	ASP	A	219	27.179	−26.974	−36.223	1.00	40.36	O
ATOM	229	H	ASP	A	219	31.771	−23.951	−36.008	1.00	0.00	H
ATOM	230	N	PRO	A	220	30.851	−25.203	−32.834	1.00	27.00	N
ATOM	231	CA	PRO	A	220	31.111	−25.870	−31.567	1.00	26.94	C
ATOM	232	C	PRO	A	220	29.889	−25.889	−30.639	1.00	26.98	C
ATOM	233	O	PRO	A	220	29.296	−24.848	−30.334	1.00	26.93	O
ATOM	234	CB	PRO	A	220	32.292	−25.131	−30.941	1.00	26.63	C
ATOM	235	CG	PRO	A	220	32.151	−23.713	−31.482	1.00	26.51	C
ATOM	236	CD	PRO	A	220	31.562	−23.927	−32.873	1.00	26.74	C
ATOM	237	N	LEU	A	221	29.713	−27.034	−29.980	1.00	27.07	N
ATOM	238	CA	LEU	A	221	28.813	−27.256	−28.848	1.00	27.10	C
ATOM	239	C	LEU	A	221	29.625	−27.688	−27.623	1.00	26.91	C
ATOM	240	O	LEU	A	221	30.477	−28.568	−27.730	1.00	26.93	O
ATOM	241	CB	LEU	A	221	27.745	−28.286	−29.271	1.00	27.48	C
ATOM	242	CG	LEU	A	221	26.740	−28.757	−28.192	1.00	27.62	C
ATOM	243	CD1	LEU	A	221	25.454	−29.220	−28.875	1.00	28.03	C
ATOM	244	CD2	LEU	A	221	27.221	−29.953	−27.367	1.00	27.55	C
ATOM	245	H	LEU	A	221	30.322	−27.806	−30.238	1.00	0.00	H
ATOM	246	N	LEU	A	222	29.206	−27.209	−26.453	1.00	26.73	N
ATOM	247	CA	LEU	A	222	29.513	−27.755	−25.132	1.00	26.61	C
ATOM	248	C	LEU	A	222	28.234	−27.672	−24.287	1.00	26.66	C
ATOM	249	O	LEU	A	222	27.495	−26.694	−24.405	1.00	26.58	O

ATOM	250	CB	LEU	A	222	30.689	−26.983	−24.495	1.00	26.27	C
ATOM	251	CG	LEU	A	222	31.011	−27.381	−23.037	1.00	26.07	C
ATOM	252	CD1	LEU	A	222	31.580	−28.798	−22.937	1.00	26.22	C
ATOM	253	CD2	LEU	A	222	32.040	−26.429	−22.434	1.00	25.72	C
ATOM	254	H	LEU	A	222	28.448	−26.543	−26.460	1.00	0.00	H
ATOM	255	N	ALA	A	223	28.009	−28.657	−23.426	1.00	26.80	N
ATOM	256	CA	ALA	A	223	26.996	−28.628	−22.378	1.00	26.85	C
ATOM	257	C	ALA	A	223	27.507	−29.389	−21.151	1.00	26.69	C
ATOM	258	O	ALA	A	223	28.128	−30.440	−21.321	1.00	26.66	O
ATOM	259	CB	ALA	A	223	25.713	−29.267	−22.918	1.00	27.24	C
ATOM	260	H	ALA	A	223	28.619	−29.466	−23.443	1.00	0.00	H
ATOM	261	N	VAL	A	224	27.206	−28.915	−19.942	1.00	26.59	N
ATOM	262	CA	VAL	A	224	27.513	−29.596	−18.675	1.00	26.40	C
ATOM	263	C	VAL	A	224	26.244	−29.750	−17.836	1.00	26.52	C
ATOM	264	O	VAL	A	224	25.599	−28.771	−17.465	1.00	26.41	O
ATOM	265	CB	VAL	A	224	28.634	−28.874	−17.896	1.00	26.00	C
ATOM	266	CG1	VAL	A	224	28.977	−29.597	−16.585	1.00	25.79	C
ATOM	267	CG2	VAL	A	224	29.927	−28.768	−18.719	1.00	25.90	C
ATOM	268	H	VAL	A	224	26.836	−27.965	−19.892	1.00	0.00	H
ATOM	269	N	ASP	A	225	25.979	−30.975	−17.386	1.00	26.75	N
ATOM	270	CA	ASP	A	225	24.855	−31.337	−16.523	1.00	26.90	C
ATOM	271	C	ASP	A	225	25.327	−32.278	−15.413	1.00	26.77	C
ATOM	272	O	ASP	A	225	25.739	−33.407	−15.671	1.00	26.89	O
ATOM	273	CB	ASP	A	225	23.759	−32.039	−17.332	1.00	27.35	C
ATOM	274	CG	ASP	A	225	22.716	−31.106	−17.950	1.00	27.50	C
ATOM	275	OD1	ASP	A	225	21.921	−30.638	−17.098	1.00	27.50	O
ATOM	276	OD2	ASP	A	225	22.293	−31.477	−19.071	1.00	27.63	O

ATOM 277 H ASP A 225 26.607 −31.722 −17.667 1.00 0.00 H

ATOM 278 N ASN A 226 25.123 −31.875 −14.157 1.00 28.10 N

ATOM 279 CA ASN A 226 25.235 −32.733 −12.966 1.00 28.01 C

ATOM 280 C ASN A 226 26.608 −33.416 −12.719 1.00 27.81 C

ATOM 281 O ASN A 226 26.746 −34.096 −11.708 1.00 27.68 O

ATOM 282 CB ASN A 226 24.060 −33.741 −12.963 1.00 28.41 C

ATOM 283 CG ASN A 226 22.694 −33.090 −13.103 1.00 28.65 C

ATOM 284 OD1 ASN A 226 22.000 −33.202 −14.105 1.00 29.05 O

ATOM 285 ND2 ASN A 226 22.285 −32.320 −12.120 1.00 28.43 N

ATOM 286 H ASN A 226 24.757 −30.943 −14.033 1.00 0.00 H

ATOM 287 1HD2 ASN A 226 22.831 −32.247 −11.281 1.00 0.00 H

ATOM 288 2HD2 ASN A 226 21.389 −31.884 −12.224 1.00 0.00 H

ATOM 289 N GLY A 227 27.624 −33.165 −13.555 1.00 34.27 N

ATOM 290 CA GLY A 227 28.937 −33.834 −13.541 1.00 34.17 C

ATOM 291 C GLY A 227 29.290 −34.541 −14.861 1.00 34.37 C

ATOM 292 O GLY A 227 30.462 −34.809 −15.122 1.00 34.28 O

ATOM 293 H GLY A 227 27.401 −32.629 −14.381 1.00 0.00 H

ATOM 294 N PHE A 228 28.309 −34.709 −15.750 1.00 26.62 N

ATOM 295 CA PHE A 228 28.478 −35.165 −17.130 1.00 26.86 C

ATOM 296 C PHE A 228 28.590 −33.987 −18.105 1.00 26.81 C

ATOM 297 O PHE A 228 28.134 −32.881 −17.805 1.00 26.69 O

ATOM 298 CB PHE A 228 27.300 −36.078 −17.500 1.00 27.32 C

ATOM 299 CG PHE A 228 27.084 −37.198 −16.501 1.00 27.40 C

ATOM 300 CD1 PHE A 228 28.071 −38.187 −16.349 1.00 27.62 C

ATOM 301 CD2 PHE A 228 25.950 −37.210 −15.669 1.00 27.27 C

ATOM 302 CE1 PHE A 228 27.928 −39.189 −15.375 1.00 27.72 C

ATOM 303 CE2 PHE A 228 25.804 −38.215 −14.695 1.00 27.36 C

ATOM	304	CZ	PHE	A	228	26.791	−39.207	−14.550	1.00	27.58	C
ATOM	305	H	PHE	A	228	27.377	−34.401	−15.500	1.00	0.00	H
ATOM	306	N	PHE	A	229	29.056	−34.250	−19.327	1.00	26.92	N
ATOM	307	CA	PHE	A	229	29.075	−33.267	−20.414	1.00	26.87	C
ATOM	308	C	PHE	A	229	28.679	−33.860	−21.772	1.00	27.24	C
ATOM	309	O	PHE	A	229	28.847	−35.059	−22.012	1.00	27.48	O
ATOM	310	CB	PHE	A	229	30.447	−32.569	−20.477	1.00	26.52	C
ATOM	311	CG	PHE	A	229	31.565	−33.382	−21.106	1.00	26.56	C
ATOM	312	CD1	PHE	A	229	31.845	−33.249	−22.480	1.00	26.79	C
ATOM	313	CD2	PHE	A	229	32.323	−34.274	−20.326	1.00	26.39	C
ATOM	314	CE1	PHE	A	229	32.858	−34.021	−23.074	1.00	26.87	C
ATOM	315	CE2	PHE	A	229	33.350	−35.030	−20.916	1.00	26.46	C
ATOM	316	CZ	PHE	A	229	33.609	−34.914	−22.291	1.00	26.71	C
ATOM	317	H	PHE	A	229	29.396	−35.186	−19.525	1.00	0.00	H
ATOM	318	N	ALA	A	230	28.321	−32.971	−22.701	1.00	27.28	N
ATOM	319	CA	ALA	A	230	28.228	−33.226	−24.136	1.00	27.59	C
ATOM	320	C	ALA	A	230	29.031	−32.200	−24.946	1.00	27.40	C
ATOM	321	O	ALA	A	230	29.260	−31.073	−24.510	1.00	27.06	O
ATOM	322	CB	ALA	A	230	26.762	−33.244	−24.573	1.00	27.81	C
ATOM	323	H	ALA	A	230	28.244	−32.003	−22.401	1.00	0.00	H
ATOM	324	N	TYR	A	231	29.423	−32.589	−26.157	1.00	27.63	N
ATOM	325	CA	TYR	A	231	30.275	−31.818	−27.056	1.00	27.48	C
ATOM	326	C	TYR	A	231	29.952	−32.123	−28.528	1.00	27.78	C
ATOM	327	O	TYR	A	231	29.590	−33.249	−28.874	1.00	28.10	O
ATOM	328	CB	TYR	A	231	31.739	−32.123	−26.711	1.00	27.22	C
ATOM	329	CG	TYR	A	231	32.755	−31.481	−27.634	1.00	27.17	C
ATOM	330	CD1	TYR	A	231	33.206	−30.171	−27.383	1.00	26.87	C

ATOM	331	CD2	TYR	A	231	33.223	−32.183	−28.762	1.00	27.44	C
ATOM	332	CE1	TYR	A	231	34.138	−29.566	−28.249	1.00	26.83	C
ATOM	333	CE2	TYR	A	231	34.147	−31.578	−29.632	1.00	27.40	C
ATOM	334	CZ	TYR	A	231	34.613	−30.270	−29.375	1.00	27.10	C
ATOM	335	OH	TYR	A	231	35.520	−29.692	−30.205	1.00	27.08	O
ATOM	336	H	TYR	A	231	29.238	−33.553	−26.419	1.00	0.00	H
ATOM	337	HH	TYR	A	231	35.609	−30.200	−31.028	1.00	0.00	H
ATOM	338	N	SER	A	232	30.176	−31.160	−29.423	1.00	27.69	N
ATOM	339	CA	SER	A	232	30.195	−31.384	−30.878	1.00	27.91	C
ATOM	340	C	SER	A	232	31.152	−30.420	−31.570	1.00	27.68	C
ATOM	341	O	SER	A	232	31.192	−29.246	−31.205	1.00	27.38	O
ATOM	342	CB	SER	A	232	28.771	−31.323	−31.478	1.00	28.17	C
ATOM	343	OG	SER	A	232	28.649	−30.496	−32.635	1.00	28.36	O
ATOM	344	H	SER	A	232	30.533	−30.272	−29.079	1.00	0.00	H
ATOM	345	HG	SER	A	232	28.840	−29.585	−32.366	1.00	0.00	H
ATOM	346	N	HIS	A	233	31.698	−30.844	−32.713	1.00	27.87	N
ATOM	347	CA	HIS	A	233	32.311	−29.949	−33.700	1.00	27.74	C
ATOM	348	C	HIS	A	233	32.132	−30.462	−35.129	1.00	28.06	C
ATOM	349	O	HIS	A	233	31.985	−31.663	−35.358	1.00	28.37	O
ATOM	350	CB	HIS	A	233	33.795	−29.706	−33.367	1.00	27.50	C
ATOM	351	CG	HIS	A	233	34.754	−30.874	−33.522	1.00	27.68	C
ATOM	352	ND1	HIS	A	233	35.715	−31.238	−32.573	1.00	27.55	N
ATOM	353	CD2	HIS	A	233	35.013	−31.575	−34.669	1.00	27.99	C
ATOM	354	CE1	HIS	A	233	36.486	−32.172	−33.149	1.00	27.78	C
ATOM	355	NE2	HIS	A	233	36.082	−32.401	−34.407	1.00	28.06	N
ATOM	356	H	HIS	A	233	31.507	−31.798	−33.003	1.00	0.00	H
ATOM	357	HE2	HIS	A	233	36.518	−33.043	−35.056	1.00	0.00	H

ATOM	358	N	LEU	A	234	32.341	−29.570	−36.092	1.00	27.98	N
ATOM	359	CA	LEU	A	234	32.568	−29.847	−37.505	1.00	28.25	C
ATOM	360	C	LEU	A	234	34.018	−29.492	−37.875	1.00	28.15	C
ATOM	361	O	LEU	A	234	34.403	−28.324	−37.859	1.00	27.88	O
ATOM	362	CB	LEU	A	234	31.531	−29.038	−38.306	1.00	28.33	C
ATOM	363	CG	LEU	A	234	31.610	−29.229	−39.829	1.00	28.61	C
ATOM	364	CD1	LEU	A	234	31.315	−30.675	−40.227	1.00	28.96	C
ATOM	365	CD2	LEU	A	234	30.582	−28.326	−40.512	1.00	28.65	C
ATOM	366	H	LEU	A	234	32.463	−28.604	−35.801	1.00	0.00	H
ATOM	367	N	GLU	A	235	34.829	−30.497	−38.200	1.00	28.42	N
ATOM	368	CA	GLU	A	235	36.171	−30.314	−38.760	1.00	28.40	C
ATOM	369	C	GLU	A	235	36.102	−29.971	−40.253	1.00	28.55	C
ATOM	370	O	GLU	A	235	35.412	−30.653	−41.018	1.00	28.84	O
ATOM	371	CB	GLU	A	235	37.008	−31.586	−38.536	1.00	28.63	C
ATOM	372	CG	GLU	A	235	38.425	−31.464	−39.122	1.00	28.53	C
ATOM	373	CD	GLU	A	235	39.310	−32.675	−38.823	1.00	28.76	C
ATOM	374	OE1	GLU	A	235	38.842	−33.781	−39.174	1.00	28.81	O
ATOM	375	OE2	GLU	A	235	40.538	−32.459	−38.883	1.00	28.89	O
ATOM	376	H	GLU	A	235	34.438	−31.433	−38.230	1.00	0.00	H
ATOM	377	N	LYS	A	236	36.969	−29.057	−40.696	1.00	28.34	N
ATOM	378	CA	LYS	A	236	37.114	−28.605	−42.083	1.00	28.45	C
ATOM	379	C	LYS	A	236	38.570	−28.599	−42.540	1.00	28.50	C
ATOM	380	O	LYS	A	236	39.458	−28.232	−41.778	1.00	28.31	O
ATOM	381	CB	LYS	A	236	36.531	−27.192	−42.214	1.00	28.19	C
ATOM	382	CG	LYS	A	236	35.000	−27.188	−42.131	1.00	28.17	C
ATOM	383	CD	LYS	A	236	34.505	−25.755	−41.941	1.00	27.94	C
ATOM	384	CE	LYS	A	236	32.984	−25.682	−42.086	1.00	28.02	C

ATOM 385 NZ LYS A 236 32.604 −25.277 −43.459 1.00 28.25 N1+

ATOM 386 H LYS A 236 37.493 −28.535 −39.996 1.00 0.00 H

ATOM 387 HZ1 LYS A 236 31.619 −25.409 −43.623 1.00 0.00 H

ATOM 388 HZ2 LYS A 236 32.785 −24.283 −43.583 1.00 0.00 H

ATOM 389 HZ3 LYS A 236 33.148 −25.784 −44.156 1.00 0.00 H

ATOM 390 N ILE A 237 38.781 −28.846 −43.831 1.00 35.45 N

ATOM 391 CA ILE A 237 39.982 −28.435 −44.571 1.00 35.50 C

ATOM 392 C ILE A 237 39.874 −26.930 −44.848 1.00 35.28 C

ATOM 393 O ILE A 237 38.910 −26.489 −45.482 1.00 35.36 O

ATOM 394 CB ILE A 237 40.128 −29.225 −45.895 1.00 35.90 C

ATOM 395 CG1 ILE A 237 40.050 −30.751 −45.663 1.00 36.20 C

ATOM 396 CG2 ILE A 237 41.446 −28.838 −46.593 1.00 35.95 C

ATOM 397 CD1 ILE A 237 40.091 −31.598 −46.941 1.00 36.62 C

ATOM 398 H ILE A 237 37.971 −29.107 −44.380 1.00 0.00 H

ATOM 399 1HD1 ILE A 237 39.877 −32.638 −46.690 1.00 0.00 H

ATOM 400 2HD1 ILE A 237 39.339 −31.244 −47.648 1.00 0.00 H

ATOM 401 N GLY A 238 40.853 −26.149 −44.399 1.00 28.33 N

ATOM 402 CA GLY A 238 40.871 −24.693 −44.529 1.00 28.13 C

ATOM 403 C GLY A 238 39.777 −23.973 −43.725 1.00 27.87 C

ATOM 404 O GLY A 238 39.363 −24.418 −42.657 1.00 27.74 O

ATOM 405 H GLY A 238 41.642 −26.589 −43.931 1.00 0.00 H

ATOM 406 N SER A 239 39.376 −22.792 −44.213 1.00 28.81 N

ATOM 407 CA SER A 239 38.617 −21.785 −43.447 1.00 28.58 C

ATOM 408 C SER A 239 37.361 −22.322 −42.739 1.00 28.53 C

ATOM 409 O SER A 239 36.545 −23.020 −43.337 1.00 28.72 O

ATOM 410 CB SER A 239 38.240 −20.607 −44.360 1.00 28.62 C

ATOM 411 OG SER A 239 37.289 −20.990 −45.341 1.00 28.85 O

ATOM	412	H	SER	A	239	39.825	−22.488	−45.061	1.00	0.00	H
ATOM	413	HG	SER	A	239	37.612	−21.795	−45.762	1.00	0.00	H
ATOM	414	N	CYS	A	240	37.074	−21.793	−41.548	1.00	27.28	N
ATOM	415	CA	CYS	A	240	35.934	−22.219	−40.728	1.00	27.23	C
ATOM	416	C	CYS	A	240	34.570	−22.018	−41.414	1.00	27.33	C
ATOM	417	O	CYS	A	240	33.711	−22.895	−41.368	1.00	27.37	O
ATOM	418	CB	CYS	A	240	35.988	−21.445	−39.407	1.00	26.95	C
ATOM	419	SG	CYS	A	240	37.607	−21.463	−38.601	1.00	26.84	S
ATOM	420	H	CYS	A	240	37.786	−21.248	−41.081	1.00	0.00	H
ATOM	421	N	THR	A	241	34.412	−20.958	−42.211	1.00	27.37	N
ATOM	422	CA	THR	A	241	33.191	−20.738	−43.000	1.00	27.45	C
ATOM	423	C	THR	A	241	33.122	−21.675	−44.213	1.00	27.73	C
ATOM	424	O	THR	A	241	32.301	−22.593	−44.244	1.00	27.88	O
ATOM	425	CB	THR	A	241	33.042	−19.262	−43.406	1.00	27.30	C
ATOM	426	OG1	THR	A	241	34.166	−18.816	−44.132	1.00	27.40	O
ATOM	427	CG2	THR	A	241	32.906	−18.338	−42.198	1.00	27.06	C
ATOM	428	H	THR	A	241	35.167	−20.294	−42.290	1.00	0.00	H
ATOM	429	HG1	THR	A	241	34.385	−17.941	−43.791	1.00	0.00	H
ATOM	430	N	ARG	A	242	34.016	−21.517	−45.197	1.00	27.82	N
ATOM	431	CA	ARG	A	242	33.886	−22.128	−46.539	1.00	28.08	C
ATOM	432	C	ARG	A	242	34.731	−23.388	−46.767	1.00	28.31	C
ATOM	433	O	ARG	A	242	34.648	−23.979	−47.836	1.00	28.57	O
ATOM	434	CB	ARG	A	242	34.132	−21.052	−47.613	1.00	28.06	C
ATOM	435	CG	ARG	A	242	33.023	−19.987	−47.590	1.00	28.01	C
ATOM	436	CD	ARG	A	242	33.265	−18.883	−48.624	1.00	27.82	C
ATOM	437	NE	ARG	A	242	32.190	−17.870	−48.564	1.00	27.72	N
ATOM	438	CZ	ARG	A	242	32.310	−16.559	−48.696	1.00	27.59	C

ATOM 439 NH1 ARG A 242 33.448 −15.983 −48.964 1.00 27.55 N
ATOM 440 NH2 ARG A 242 31.267 −15.790 −48.556 1.00 27.52 N
ATOM 441 H ARG A 242 34.702 −20.778 −45.070 1.00 0.00 H
ATOM 442 HE ARG A 242 31.265 −18.219 −48.376 1.00 0.00 H
ATOM 443 1HH1 ARG A 242 34.266 −16.554 −49.080 1.00 0.00 H
ATOM 444 2HH1 ARG A 242 33.516 −14.985 −49.045 1.00 0.00 H
ATOM 445 1HH2 ARG A 242 30.372 −16.184 −48.326 1.00 0.00 H
ATOM 446 2HH2 ARG A 242 31.361 −14.793 −48.632 1.00 0.00 H
ATOM 447 N GLY A 243 35.482 −23.836 −45.764 1.00 28.24 N
ATOM 448 CA GLY A 243 36.289 −25.056 −45.808 1.00 28.47 C
ATOM 449 C GLY A 243 35.466 −26.342 −45.929 1.00 28.74 C
ATOM 450 O GLY A 243 34.333 −26.424 −45.435 1.00 28.70 O
ATOM 451 H GLY A 243 35.528 −23.289 −44.911 1.00 0.00 H
ATOM 452 N ILE A 244 36.051 −27.347 −46.587 1.00 29.04 N
ATOM 453 CA ILE A 244 35.428 −28.644 −46.885 1.00 29.36 C
ATOM 454 C ILE A 244 35.351 −29.481 −45.607 1.00 29.31 C
ATOM 455 O ILE A 244 36.378 −29.747 −44.991 1.00 29.18 O
ATOM 456 CB ILE A 244 36.208 −29.404 −47.989 1.00 29.73 C
ATOM 457 CG1 ILE A 244 36.279 −28.617 −49.320 1.00 29.85 C
ATOM 458 CG2 ILE A 244 35.559 −30.776 −48.262 1.00 30.08 C
ATOM 459 CD1 ILE A 244 37.524 −27.728 −49.446 1.00 29.71 C
ATOM 460 H ILE A 244 37.018 −27.217 −46.856 1.00 0.00 H
ATOM 461 N ALA A 245 34.159 −29.948 −45.236 1.00 29.42 N
ATOM 462 CA ALA A 245 33.963 −30.790 −44.056 1.00 29.35 C
ATOM 463 C ALA A 245 34.711 −32.135 −44.170 1.00 29.63 C
ATOM 464 O ALA A 245 34.429 −32.908 −45.084 1.00 30.00 O
ATOM 465 CB ALA A 245 32.458 −31.010 −43.870 1.00 29.41 C

ATOM 466 H ALA A 245 33.364 −29.765 −45.824 1.00 0.00 H

ATOM 467 N LYS A 246 35.543 −32.472 −43.174 1.00 29.46 N

ATOM 468 CA LYS A 246 36.100 −33.827 −42.992 1.00 29.72 C

ATOM 469 C LYS A 246 35.198 −34.707 −42.126 1.00 29.91 C

ATOM 470 O LYS A 246 34.843 −35.805 −42.534 1.00 30.30 O

ATOM 471 CB LYS A 246 37.522 −33.785 −42.403 1.00 29.50 C

ATOM 472 CG LYS A 246 38.550 −33.093 −43.319 1.00 29.57 C

ATOM 473 CD LYS A 246 39.937 −33.764 −43.287 1.00 29.86 C

ATOM 474 CE LYS A 246 40.583 −33.826 −41.896 1.00 29.66 C

ATOM 475 NZ LYS A 246 41.146 −32.527 −41.461 1.00 29.22 N1+

ATOM 476 H LYS A 246 35.676 −31.791 −42.432 1.00 0.00 H

ATOM 477 HA LYS A 246 36.151 −34.326 −43.962 1.00 0.00 H

ATOM 478 HZ1 LYS A 246 42.027 −32.307 −41.900 1.00 0.00 H

ATOM 479 HZ2 LYS A 246 40.487 −31.776 −41.592 1.00 0.00 H

ATOM 480 HZ3 LYS A 246 41.280 −32.562 −40.442 1.00 0.00 H

ATOM 481 N GLN A 247 34.765 −34.201 −40.969 1.00 29.65 N

ATOM 482 CA GLN A 247 33.913 −34.935 −40.025 1.00 29.82 C

ATOM 483 C GLN A 247 33.124 −33.981 −39.117 1.00 29.51 C

ATOM 484 O GLN A 247 33.687 −33.059 −38.531 1.00 29.17 O

ATOM 485 CB GLN A 247 34.737 −35.945 −39.200 1.00 29.98 C

ATOM 486 CG GLN A 247 35.890 −35.326 −38.395 1.00 29.64 C

ATOM 487 CD GLN A 247 36.834 −36.375 −37.824 1.00 29.83 C

ATOM 488 OE1 GLN A 247 36.427 −37.392 −37.286 1.00 29.67 O

ATOM 489 NE2 GLN A 247 38.125 −36.137 −37.864 1.00 30.19 N

ATOM 490 H GLN A 247 35.093 −33.285 −40.697 1.00 0.00 H

ATOM 491 1HE2 GLN A 247 38.475 −35.300 −38.342 1.00 0.00 H

ATOM 492 2HE2 GLN A 247 38.735 −36.844 −37.503 1.00 0.00 H

ATOM 493 N ARG A 248 31.837 −34.276 −38.907 1.00 32.41 N
ATOM 494 CA ARG A 248 31.103 −33.890 −37.695 1.00 32.19 C
ATOM 495 C ARG A 248 31.421 −34.949 −36.644 1.00 32.21 C
ATOM 496 O ARG A 248 31.179 −36.117 −36.925 1.00 32.54 O
ATOM 497 CB ARG A 248 29.593 −33.830 −38.011 1.00 32.42 C
ATOM 498 CG ARG A 248 28.643 −33.750 −36.793 1.00 32.30 C
ATOM 499 CD ARG A 248 28.634 −32.399 −36.070 1.00 31.87 C
ATOM 500 NE ARG A 248 28.074 −31.348 −36.936 1.00 31.85 N
ATOM 501 CZ ARG A 248 27.935 −30.070 −36.650 1.00 31.55 C
ATOM 502 NH1 ARG A 248 27.457 −29.278 −37.554 1.00 31.55 N
ATOM 503 NH2 ARG A 248 28.225 −29.537 −35.499 1.00 31.24 N
ATOM 504 H ARG A 248 31.490 −35.099 −39.379 1.00 0.00 H
ATOM 505 HE ARG A 248 27.794 −31.610 −37.867 1.00 0.00 H
ATOM 506 1HH1 ARG A 248 27.018 −29.651 −38.388 1.00 0.00 H
ATOM 507 2HH1 ARG A 248 27.295 −28.313 −37.258 1.00 0.00 H
ATOM 508 1HH2 ARG A 248 28.509 −30.101 −34.711 1.00 0.00 H
ATOM 509 2HH2 ARG A 248 28.038 −28.542 −35.383 1.00 0.00 H
ATOM 510 N ILE A 249 31.771 −34.553 −35.425 1.00 29.12 N
ATOM 511 CA ILE A 249 31.750 −35.455 −34.263 1.00 29.14 C
ATOM 512 C ILE A 249 30.752 −34.939 −33.231 1.00 29.00 C
ATOM 513 O ILE A 249 30.565 −33.728 −33.089 1.00 28.78 O
ATOM 514 CB ILE A 249 33.154 −35.736 −33.670 1.00 28.90 C
ATOM 515 CG1 ILE A 249 33.655 −34.642 −32.698 1.00 28.47 C
ATOM 516 CG2 ILE A 249 34.173 −36.033 −34.787 1.00 28.89 C
ATOM 517 CD1 ILE A 249 34.960 −35.011 −31.981 1.00 28.27 C
ATOM 518 H ILE A 249 31.884 −33.559 −35.252 1.00 0.00 H
ATOM 519 N ILE A 250 30.161 −35.863 −32.480 1.00 34.08 N

ATOM 520 CA ILE A 250 29.270 −35.601 −31.348 1.00 33.97 C
ATOM 521 C ILE A 250 29.651 −36.582 −30.236 1.00 33.98 C
ATOM 522 O ILE A 250 29.774 −37.774 −30.502 1.00 34.32 O
ATOM 523 CB ILE A 250 27.786 −35.752 −31.761 1.00 34.29 C
ATOM 524 CG1 ILE A 250 27.451 −35.004 −33.077 1.00 34.21 C
ATOM 525 CG2 ILE A 250 26.899 −35.273 −30.599 1.00 34.23 C
ATOM 526 CD1 ILE A 250 25.974 −35.031 −33.481 1.00 34.57 C
ATOM 527 H ILE A 250 30.328 −36.842 −32.707 1.00 0.00 H
ATOM 528 N GLY A 251 29.941 −36.107 −29.028 1.00 28.69 N
ATOM 529 CA GLY A 251 30.489 −36.946 −27.958 1.00 28.65 C
ATOM 530 C GLY A 251 30.115 −36.480 −26.557 1.00 28.38 C
ATOM 531 O GLY A 251 29.592 −35.380 −26.385 1.00 28.18 O
ATOM 532 H GLY A 251 29.799 −35.119 −28.835 1.00 0.00 H
ATOM 533 N VAL A 252 30.293 −37.366 −25.580 1.00 28.40 N
ATOM 534 CA VAL A 252 29.871 −37.189 −24.182 1.00 28.19 C
ATOM 535 C VAL A 252 30.884 −37.810 −23.218 1.00 28.01 C
ATOM 536 O VAL A 252 31.718 −38.635 −23.610 1.00 28.16 O
ATOM 537 CB VAL A 252 28.455 −37.761 −23.924 1.00 28.54 C
ATOM 538 CG1 VAL A 252 27.394 −37.154 −24.846 1.00 28.82 C
ATOM 539 CG2 VAL A 252 28.369 −39.279 −24.088 1.00 28.88 C
ATOM 540 H VAL A 252 30.743 −38.248 −25.812 1.00 0.00 H
ATOM 541 N GLY A 253 30.778 −37.454 −21.939 1.00 27.71 N
ATOM 542 CA GLY A 253 31.604 −38.038 −20.888 1.00 27.52 C
ATOM 543 C GLY A 253 31.428 −37.386 −19.521 1.00 27.15 C
ATOM 544 O GLY A 253 30.374 −36.827 −19.219 1.00 27.12 O
ATOM 545 H GLY A 253 30.100 −36.745 −21.678 1.00 0.00 H
ATOM 546 N GLU A 254 32.484 −37.430 −18.709 1.00 34.09 N

ATOM 547 CA GLU A 254 32.478 −37.058 −17.286 1.00 33.75 C

ATOM 548 C GLU A 254 33.516 −35.946 −16.996 1.00 33.31 C

ATOM 549 O GLU A 254 34.571 −35.894 −17.633 1.00 33.21 O

ATOM 550 CB GLU A 254 32.691 −38.323 −16.420 1.00 33.76 C

ATOM 551 CG GLU A 254 31.831 −39.526 −16.879 1.00 34.15 C

ATOM 552 CD GLU A 254 31.691 −40.657 −15.847 1.00 34.03 C

ATOM 553 OE1 GLU A 254 32.668 −40.912 −15.109 1.00 33.63 O

ATOM 554 OE2 GLU A 254 30.652 −41.362 −15.901 1.00 34.34 O

ATOM 555 H GLU A 254 33.330 −37.865 −19.064 1.00 0.00 H

ATOM 556 N VAL A 255 33.189 −34.980 −16.124 1.00 25.87 N

ATOM 557 CA VAL A 255 33.978 −33.741 −15.898 1.00 25.47 C

ATOM 558 C VAL A 255 34.874 −33.844 −14.647 1.00 25.20 C

ATOM 559 O VAL A 255 34.716 −33.103 −13.677 1.00 25.14 O

ATOM 560 CB VAL A 255 33.088 −32.473 −15.905 1.00 25.38 C

ATOM 561 CG1 VAL A 255 33.923 −31.183 −15.995 1.00 24.99 C

ATOM 562 CG2 VAL A 255 32.144 −32.431 −17.114 1.00 25.63 C

ATOM 563 H VAL A 255 32.287 −35.043 −15.658 1.00 0.00 H

ATOM 564 N LEU A 256 35.668 −34.914 −14.577 1.00 25.04 N

ATOM 565 CA LEU A 256 36.375 −35.344 −13.362 1.00 24.83 C

ATOM 566 C LEU A 256 37.560 −34.429 −12.982 1.00 24.43 C

ATOM 567 O LEU A 256 38.222 −33.832 −13.839 1.00 24.39 O

ATOM 568 CB LEU A 256 36.827 −36.814 −13.502 1.00 25.04 C

ATOM 569 CG LEU A 256 35.766 −37.805 −14.024 1.00 25.50 C

ATOM 570 CD1 LEU A 256 36.387 −39.191 −14.190 1.00 25.67 C

ATOM 571 CD2 LEU A 256 34.568 −37.914 −13.081 1.00 25.62 C

ATOM 572 H LEU A 256 35.735 −35.486 −15.405 1.00 0.00 H

ATOM 573 N ASP A 257 37.946 −34.451 −11.706 1.00 24.14 N

ATOM 574 CA ASP A 257 39.196 −33.878 −11.194 1.00 23.75 C
ATOM 575 C ASP A 257 40.246 −34.976 −10.893 1.00 23.70 C
ATOM 576 O ASP A 257 40.280 −36.018 −11.554 1.00 23.88 O
ATOM 577 CB ASP A 257 38.898 −32.979 −9.981 1.00 23.51 C
ATOM 578 CG ASP A 257 38.367 −33.704 −8.737 1.00 23.67 C
ATOM 579 OD1 ASP A 257 38.344 −33.037 −7.682 1.00 23.58 O
ATOM 580 OD2 ASP A 257 38.141 −34.932 −8.817 1.00 23.93 O
ATOM 581 H ASP A 257 37.426 −35.012 −11.041 1.00 0.00 H
ATOM 582 N ARG A 258 41.282 −34.591 −10.139 1.00 31.69 N
ATOM 583 CA ARG A 258 42.198 −35.448 −9.365 1.00 31.57 C
ATOM 584 C ARG A 258 42.535 −34.675 −8.076 1.00 31.15 C
ATOM 585 O ARG A 258 42.035 −33.573 −7.877 1.00 31.05 O
ATOM 586 CB ARG A 258 43.478 −35.822 −10.155 1.00 31.63 C
ATOM 587 CG ARG A 258 43.325 −36.719 −11.398 1.00 32.06 C
ATOM 588 CD ARG A 258 43.109 −36.073 −12.781 1.00 32.29 C
ATOM 589 NE ARG A 258 43.115 −34.595 −12.795 1.00 32.04 N
ATOM 590 CZ ARG A 258 42.185 −33.803 −13.287 1.00 32.01 C
ATOM 591 NH1 ARG A 258 41.069 −34.259 −13.779 1.00 32.22 N
ATOM 592 NH2 ARG A 258 42.341 −32.522 −13.233 1.00 31.79 N
ATOM 593 H ARG A 258 41.182 −33.660 −9.752 1.00 0.00 H
ATOM 594 HE ARG A 258 43.899 −34.109 −12.353 1.00 0.00 H
ATOM 595 1HH1 ARG A 258 40.810 −35.185 −13.468 1.00 0.00 H
ATOM 596 2HH1 ARG A 258 40.301 −33.622 −13.952 1.00 0.00 H
ATOM 597 1HH2 ARG A 258 43.031 −32.183 −12.557 1.00 0.00 H
ATOM 598 2HH2 ARG A 258 41.523 −31.931 −13.333 1.00 0.00 H
ATOM 599 N GLY A 259 43.596 −35.062 −7.362 1.00 41.93 N
ATOM 600 CA GLY A 259 44.176 −34.263 −6.269 1.00 41.54 C

ATOM	601	C	GLY	A	259	44.690	−32.857	−6.646	1.00	41.30	C
ATOM	602	O	GLY	A	259	44.931	−32.060	−5.744	1.00	41.02	O
ATOM	603	H	GLY	A	259	44.026	−35.941	−7.599	1.00	0.00	H
ATOM	604	N	ASP	A	260	44.791	−32.516	−7.939	1.00	50.86	N
ATOM	605	CA	ASP	A	260	45.023	−31.142	−8.429	1.00	50.70	C
ATOM	606	C	ASP	A	260	43.776	−30.226	−8.348	1.00	50.79	C
ATOM	607	O	ASP	A	260	43.902	−28.999	−8.368	1.00	50.71	O
ATOM	608	CB	ASP	A	260	45.555	−31.212	−9.873	1.00	50.84	C
ATOM	609	CG	ASP	A	260	44.575	−31.843	−10.870	1.00	50.90	C
ATOM	610	OD1	ASP	A	260	44.907	−32.873	−11.493	1.00	50.67	O
ATOM	611	OD2	ASP	A	260	43.434	−31.354	−11.002	1.00	51.19	O
ATOM	612	H	ASP	A	260	44.544	−33.209	−8.628	1.00	0.00	H
ATOM	613	N	LYS	A	261	42.591	−30.830	−8.185	1.00	22.34	N
ATOM	614	CA	LYS	A	261	41.266	−30.221	−7.978	1.00	22.44	C
ATOM	615	C	LYS	A	261	40.802	−29.293	−9.107	1.00	22.62	C
ATOM	616	O	LYS	A	261	40.002	−28.384	−8.881	1.00	22.63	O
ATOM	617	CB	LYS	A	261	41.139	−29.638	−6.559	1.00	22.15	C
ATOM	618	CG	LYS	A	261	41.757	−30.591	−5.520	1.00	21.90	C
ATOM	619	CD	LYS	A	261	41.205	−30.392	−4.115	1.00	21.66	C
ATOM	620	CE	LYS	A	261	41.977	−31.307	−3.158	1.00	21.44	C
ATOM	621	NZ	LYS	A	261	41.169	−31.616	−1.960	1.00	21.17	N1+
ATOM	622	H	LYS	A	261	42.608	−31.843	−8.160	1.00	0.00	H
ATOM	623	HZ1	LYS	A	261	41.681	−31.991	−1.183	1.00	0.00	H
ATOM	624	HZ2	LYS	A	261	40.403	−32.259	−2.193	1.00	0.00	H
ATOM	625	HZ3	LYS	A	261	40.583	−30.822	−1.686	1.00	0.00	H
ATOM	626	N	VAL	A	262	41.310	−29.508	−10.324	1.00	46.68	N
ATOM	627	CA	VAL	A	262	40.851	−28.860	−11.563	1.00	46.91	C

ATOM 628 C VAL A 262 39.850 −29.779 −12.285 1.00 47.22 C

ATOM 629 O VAL A 262 40.228 −30.897 −12.642 1.00 47.27 O

ATOM 630 CB VAL A 262 42.044 −28.531 −12.488 1.00 46.93 C

ATOM 631 CG1 VAL A 262 41.592 −27.774 −13.744 1.00 47.26 C

ATOM 632 CG2 VAL A 262 43.100 −27.665 −11.783 1.00 46.71 C

ATOM 633 H VAL A 262 42.001 −30.251 −10.416 1.00 0.00 H

ATOM 634 N PRO A 263 38.633 −29.325 −12.636 1.00 23.53 N

ATOM 635 CA PRO A 263 37.737 −30.097 −13.493 1.00 23.88 C

ATOM 636 C PRO A 263 38.317 −30.226 −14.910 1.00 24.06 C

ATOM 637 O PRO A 263 38.679 −29.220 −15.524 1.00 23.93 O

ATOM 638 CB PRO A 263 36.395 −29.355 −13.495 1.00 24.04 C

ATOM 639 CG PRO A 263 36.512 −28.293 −12.401 1.00 23.78 C

ATOM 640 CD PRO A 263 38.012 −28.077 −12.239 1.00 23.49 C

ATOM 641 N SER A 264 38.291 −31.436 −15.468 1.00 24.37 N

ATOM 642 CA SER A 264 38.810 −31.763 −16.805 1.00 24.59 C

ATOM 643 C SER A 264 37.825 −32.657 −17.558 1.00 24.99 C

ATOM 644 O SER A 264 37.125 −33.456 −16.944 1.00 25.12 O

ATOM 645 CB SER A 264 40.164 −32.477 −16.705 1.00 24.49 C

ATOM 646 OG SER A 264 41.124 −31.675 −16.042 1.00 24.25 O

ATOM 647 H SER A 264 37.953 −32.215 −14.909 1.00 0.00 H

ATOM 648 HG SER A 264 41.895 −32.214 −15.835 1.00 0.00 H

ATOM 649 N MET A 265 37.794 −32.583 −18.893 1.00 25.19 N

ATOM 650 CA MET A 265 36.953 −33.487 −19.689 1.00 25.58 C

ATOM 651 C MET A 265 37.548 −34.907 −19.730 1.00 25.81 C

ATOM 652 O MET A 265 38.710 −35.079 −20.101 1.00 25.75 O

ATOM 653 CB MET A 265 36.683 −32.908 −21.096 1.00 25.71 C

ATOM 654 CG MET A 265 37.801 −33.132 −22.126 1.00 26.00 C

ATOM 655 SD MET A 265 37.475 −32.467 −23.779 1.00 26.23 S

ATOM 656 CE MET A 265 38.726 −33.401 −24.702 1.00 26.60 C

ATOM 657 H MET A 265 38.430 −31.965 −19.370 1.00 0.00 H

ATOM 658 N PHE A 266 36.693 −35.919 −19.593 1.00 34.60 N

ATOM 659 CA PHE A 266 36.995 −37.317 −19.899 1.00 34.90 C

ATOM 660 C PHE A 266 35.923 −37.859 −20.844 1.00 35.34 C

ATOM 661 O PHE A 266 34.844 −38.255 −20.409 1.00 35.52 O

ATOM 662 CB PHE A 266 37.115 −38.134 −18.601 1.00 34.77 C

ATOM 663 CG PHE A 266 38.318 −37.750 −17.761 1.00 34.36 C

ATOM 664 CD1 PHE A 266 39.526 −38.464 −17.878 1.00 34.35 C

ATOM 665 CD2 PHE A 266 38.245 −36.648 −16.892 1.00 33.99 C

ATOM 666 CE1 PHE A 266 40.654 −38.076 −17.131 1.00 33.96 C

ATOM 667 CE2 PHE A 266 39.373 −36.255 −16.155 1.00 33.61 C

ATOM 668 CZ PHE A 266 40.578 −36.967 −16.270 1.00 33.59 C

ATOM 669 H PHE A 266 35.799 −35.736 −19.143 1.00 0.00 H

ATOM 670 N MET A 267 36.181 −37.771 −22.155 1.00 32.46 N

ATOM 671 CA MET A 267 35.320 −38.365 −23.188 1.00 32.90 C

ATOM 672 C MET A 267 35.211 −39.876 −22.971 1.00 33.24 C

ATOM 673 O MET A 267 36.225 −40.571 −22.988 1.00 33.24 O

ATOM 674 CB MET A 267 35.903 −38.117 −24.590 1.00 33.04 C

ATOM 675 CG MET A 267 35.610 −36.726 −25.154 1.00 32.94 C

ATOM 676 SD MET A 267 33.900 −36.525 −25.725 1.00 33.35 S

ATOM 677 CE MET A 267 34.105 −35.118 −26.844 1.00 33.21 C

ATOM 678 H MET A 267 37.089 −37.440 −22.431 1.00 0.00 H

ATOM 679 N THR A 268 33.985 −40.387 −22.891 1.00 28.08 N

ATOM 680 CA THR A 268 33.703 −41.827 −22.769 1.00 28.46 C

ATOM 681 C THR A 268 33.118 −42.422 −24.052 1.00 28.95 C

ATOM 682 O THR A 268 33.195 −43.633 −24.258 1.00 29.26 O

ATOM 683 CB THR A 268 32.724 −42.088 −21.619 1.00 28.53 C

ATOM 684 OG1 THR A 268 31.494 −41.477 −21.922 1.00 28.68 O

ATOM 685 CG2 THR A 268 33.207 −41.548 −20.274 1.00 28.08 C

ATOM 686 H THR A 268 33.187 −39.755 −22.898 1.00 0.00 H

ATOM 687 HG1 THR A 268 30.965 −41.423 −21.100 1.00 0.00 H

ATOM 688 N ASN A 269 32.483 −41.596 −24.892 1.00 29.03 N

ATOM 689 CA ASN A 269 31.578 −42.047 −25.944 1.00 29.52 C

ATOM 690 C ASN A 269 31.463 −40.992 −27.059 1.00 29.43 C

ATOM 691 O ASN A 269 31.212 −39.821 −26.777 1.00 29.07 O

ATOM 692 CB ASN A 269 30.227 −42.297 −25.259 1.00 29.73 C

ATOM 693 CG ASN A 269 29.172 −42.950 −26.119 1.00 30.31 C

ATOM 694 OD1 ASN A 269 29.389 −43.365 −27.246 1.00 30.61 O

ATOM 695 ND2 ASN A 269 28.011 −43.149 −25.555 1.00 30.49 N

ATOM 696 H ASN A 269 32.390 −40.630 −24.597 1.00 0.00 H

ATOM 697 1HD2 ASN A 269 27.888 −42.862 −24.587 1.00 0.00 H

ATOM 698 2HD2 ASN A 269 27.297 −43.654 −26.061 1.00 0.00 H

ATOM 699 N VAL A 270 31.651 −41.389 −28.324 1.00 29.75 N

ATOM 700 CA VAL A 270 31.692 −40.483 −29.488 1.00 29.65 C

ATOM 701 C VAL A 270 31.021 −41.129 −30.704 1.00 30.11 C

ATOM 702 O VAL A 270 31.187 −42.323 −30.953 1.00 30.49 O

ATOM 703 CB VAL A 270 33.138 −40.040 −29.823 1.00 29.46 C

ATOM 704 CG1 VAL A 270 33.187 −39.019 −30.971 1.00 29.48 C

ATOM 705 CG2 VAL A 270 33.861 −39.398 −28.627 1.00 28.96 C

ATOM 706 H VAL A 270 31.775 −42.375 −28.506 1.00 0.00 H

ATOM 707 N TRP A 271 30.305 −40.328 −31.492 1.00 30.07 N

ATOM 708 CA TRP A 271 29.620 −40.709 −32.726 1.00 30.51 C

ATOM	709	C	TRP	A	271	29.911	−39.712	−33.859	1.00	30.47	C
ATOM	710	O	TRP	A	271	30.058	−38.509	−33.628	1.00	30.13	O
ATOM	711	CB	TRP	A	271	28.120	−40.844	−32.439	1.00	30.64	C
ATOM	712	CG	TRP	A	271	27.261	−41.177	−33.621	1.00	31.10	C
ATOM	713	CD1	TRP	A	271	27.079	−42.416	−34.125	1.00	31.61	C
ATOM	714	CD2	TRP	A	271	26.498	−40.279	−34.487	1.00	31.10	C
ATOM	715	NE1	TRP	A	271	26.255	−42.354	−35.231	1.00	31.93	N
ATOM	716	CE2	TRP	A	271	25.853	−41.062	−35.491	1.00	31.62	C
ATOM	717	CE3	TRP	A	271	26.269	−38.887	−34.514	1.00	30.73	C
ATOM	718	CZ2	TRP	A	271	25.015	−40.498	−36.465	1.00	31.75	C
ATOM	719	CZ3	TRP	A	271	25.418	−38.311	−35.477	1.00	30.87	C
ATOM	720	CH2	TRP	A	271	24.795	−39.111	−36.453	1.00	31.36	C
ATOM	721	H	TRP	A	271	30.218	−39.355	−31.218	1.00	0.00	H
ATOM	722	HE1	TRP	A	271	26.002	−43.166	−35.774	1.00	0.00	H
ATOM	723	N	THR	A	272	29.888	−40.214	−35.095	1.00	30.85	N
ATOM	724	CA	THR	A	272	30.163	−39.468	−36.332	1.00	30.86	C
ATOM	725	C	THR	A	272	29.091	−39.822	−37.372	1.00	31.24	C
ATOM	726	O	THR	A	272	28.882	−41.012	−37.619	1.00	31.69	O
ATOM	727	CB	THR	A	272	31.572	−39.808	−36.852	1.00	31.00	C
ATOM	728	OG1	THR	A	272	32.533	−39.287	−35.963	1.00	30.68	O
ATOM	729	CG2	THR	A	272	31.900	−39.245	−38.235	1.00	30.94	C
ATOM	730	H	THR	A	272	29.656	−41.190	−35.201	1.00	0.00	H
ATOM	731	HG1	THR	A	272	33.399	−39.393	−36.365	1.00	0.00	H
ATOM	732	N	PRO	A	273	28.410	−38.849	−38.012	1.00	31.08	N
ATOM	733	CA	PRO	A	273	27.413	−39.142	−39.040	1.00	31.46	C
ATOM	734	C	PRO	A	273	28.034	−39.779	−40.297	1.00	31.70	C
ATOM	735	O	PRO	A	273	29.108	−39.344	−40.714	1.00	31.47	O

ATOM 736 CB PRO A 273 26.752 −37.806 −39.394 1.00 31.19 C
ATOM 737 CG PRO A 273 27.151 −36.850 −38.276 1.00 30.68 C
ATOM 738 CD PRO A 273 28.472 −37.419 −37.768 1.00 30.60 C
ATOM 739 N PRO A 274 27.249 −40.544 −41.084 1.00 32.17 N
ATOM 740 CA PRO A 274 27.665 −41.048 −42.400 1.00 32.50 C
ATOM 741 C PRO A 274 27.792 −39.963 −43.490 1.00 32.32 C
ATOM 742 O PRO A 274 28.243 −40.257 −44.594 1.00 32.50 O
ATOM 743 CB PRO A 274 26.621 −42.109 −42.767 1.00 33.03 C
ATOM 744 CG PRO A 274 25.361 −41.620 −42.056 1.00 32.87 C
ATOM 745 CD PRO A 274 25.912 −41.024 −40.762 1.00 32.44 C
ATOM 746 N ASN A 275 27.436 −38.706 −43.198 1.00 32.01 N
ATOM 747 CA ASN A 275 27.847 −37.545 −43.986 1.00 31.73 C
ATOM 748 C ASN A 275 28.017 −36.307 −43.074 1.00 31.21 C
ATOM 749 O ASN A 275 27.078 −35.951 −42.359 1.00 31.14 O
ATOM 750 CB ASN A 275 26.834 −37.294 −45.114 1.00 31.96 C
ATOM 751 CG ASN A 275 27.359 −36.265 −46.096 1.00 31.71 C
ATOM 752 OD1 ASN A 275 27.651 −35.133 −45.749 1.00 31.31 O
ATOM 753 ND2 ASN A 275 27.756 −36.688 −47.272 1.00 31.95 N
ATOM 754 H ASN A 275 27.096 −38.522 −42.268 1.00 0.00 H
ATOM 755 1HD2 ASN A 275 27.652 −37.654 −47.523 1.00 0.00 H
ATOM 756 2HD2 ASN A 275 28.212 −36.014 −47.860 1.00 0.00 H
ATOM 757 N PRO A 276 29.185 −35.635 −43.072 1.00 30.86 N
ATOM 758 CA PRO A 276 29.442 −34.504 −42.181 1.00 30.41 C
ATOM 759 C PRO A 276 28.842 −33.176 −42.668 1.00 30.19 C
ATOM 760 O PRO A 276 28.701 −32.247 −41.877 1.00 29.83 O
ATOM 761 CB PRO A 276 30.966 −34.435 −42.124 1.00 30.19 C
ATOM 762 CG PRO A 276 31.396 −34.856 −43.527 1.00 30.41 C

ATOM 763 CD PRO A 276 30.382 −35.954 −43.845 1.00 30.88 C

ATOM 764 N SER A 277 28.540 −33.051 −43.964 1.00 30.43 N

ATOM 765 CA SER A 277 28.065 −31.815 −44.598 1.00 30.27 C

ATOM 766 C SER A 277 26.572 −31.546 −44.394 1.00 30.24 C

ATOM 767 O SER A 277 26.124 −30.444 −44.695 1.00 29.99 O

ATOM 768 CB SER A 277 28.344 −31.868 −46.105 1.00 30.54 C

ATOM 769 OG SER A 277 29.722 −32.085 −46.352 1.00 30.71 O

ATOM 770 H SER A 277 28.644 −33.861 −44.568 1.00 0.00 H

ATOM 771 HG SER A 277 30.202 −31.284 −46.138 1.00 0.00 H

ATOM 772 N THR A 278 25.807 −32.544 −43.939 1.00 30.51 N

ATOM 773 CA THR A 278 24.331 −32.536 −43.903 1.00 30.62 C

ATOM 774 C THR A 278 23.748 −32.494 −42.482 1.00 30.50 C

ATOM 775 O THR A 278 22.591 −32.870 −42.298 1.00 30.61 O

ATOM 776 CB THR A 278 23.777 −33.748 −44.678 1.00 31.11 C

ATOM 777 OG1 THR A 278 24.192 −34.949 −44.066 1.00 31.31 O

ATOM 778 CG2 THR A 278 24.235 −33.792 −46.136 1.00 31.28 C

ATOM 779 H THR A 278 26.250 −33.442 −43.800 1.00 0.00 H

ATOM 780 HG1 THR A 278 23.721 −35.016 −43.230 1.00 0.00 H

ATOM 781 N ILE A 279 24.568 −32.199 −41.463 1.00 30.29 N

ATOM 782 CA ILE A 279 24.195 −32.189 −40.035 1.00 30.12 C

ATOM 783 C ILE A 279 24.608 −30.855 −39.400 1.00 29.68 C

ATOM 784 O ILE A 279 25.792 −30.502 −39.419 1.00 29.47 O

ATOM 785 CB ILE A 279 24.820 −33.400 −39.298 1.00 30.15 C

ATOM 786 CG1 ILE A 279 24.274 −34.758 −39.802 1.00 30.62 C

ATOM 787 CG2 ILE A 279 24.653 −33.302 −37.769 1.00 29.89 C

ATOM 788 CD1 ILE A 279 22.788 −35.028 −39.525 1.00 30.76 C

ATOM 789 H ILE A 279 25.499 −31.886 −41.699 1.00 0.00 H

ATOM 790 N HIS A 280 23.664 −30.163 −38.754 1.00 29.56 N

ATOM 791 CA HIS A 280 23.795 −28.751 −38.365 1.00 29.19 C

ATOM 792 C HIS A 280 23.184 −28.450 −36.985 1.00 29.06 C

ATOM 793 O HIS A 280 22.183 −29.059 −36.603 1.00 29.28 O

ATOM 794 CB HIS A 280 23.124 −27.860 −39.431 1.00 29.17 C

ATOM 795 CG HIS A 280 23.448 −28.223 −40.857 1.00 29.42 C

ATOM 796 ND1 HIS A 280 24.583 −27.875 −41.550 1.00 29.30 N

ATOM 797 CD2 HIS A 280 22.744 −29.094 −41.644 1.00 29.80 C

ATOM 798 CE1 HIS A 280 24.558 −28.511 −42.732 1.00 29.59 C

ATOM 799 NE2 HIS A 280 23.461 −29.275 −42.829 1.00 29.90 N

ATOM 800 H HIS A 280 22.721 −30.544 −38.757 1.00 0.00 H

ATOM 801 HD1 HIS A 280 25.298 −27.238 −41.234 1.00 0.00 H

ATOM 802 N HIS A 281 23.709 −27.410 −36.323 1.00 28.71 N

ATOM 803 CA HIS A 281 23.065 −26.677 −35.218 1.00 28.56 C

ATOM 804 C HIS A 281 22.537 −27.561 −34.071 1.00 28.69 C

ATOM 805 O HIS A 281 21.420 −27.382 −33.587 1.00 28.78 O

ATOM 806 CB HIS A 281 22.010 −25.694 −35.755 1.00 28.58 C

ATOM 807 CG HIS A 281 22.453 −24.893 −36.955 1.00 28.41 C

ATOM 808 ND1 HIS A 281 23.614 −24.157 −37.089 1.00 28.11 N

ATOM 809 CD2 HIS A 281 21.812 −24.856 −38.163 1.00 28.50 C

ATOM 810 CE1 HIS A 281 23.649 −23.667 −38.342 1.00 28.04 C

ATOM 811 NE2 HIS A 281 22.577 −24.080 −39.035 1.00 28.27 N

ATOM 812 H HIS A 281 24.543 −26.980 −36.703 1.00 0.00 H

ATOM 813 HD1 HIS A 281 24.350 −24.049 −36.402 1.00 0.00 H

ATOM 814 N CYS A 282 23.316 −28.577 −33.691 1.00 28.70 N

ATOM 815 CA CYS A 282 22.938 −29.526 −32.648 1.00 28.83 C

ATOM 816 C CYS A 282 22.659 −28.844 −31.294 1.00 28.57 C

ATOM 817 O CYS A 282 23.218 −27.791 −30.988 1.00 28.26 O
ATOM 818 CB CYS A 282 24.023 −30.599 −32.525 1.00 28.90 C
ATOM 819 SG CYS A 282 24.293 −31.607 −34.011 1.00 29.22 S
ATOM 820 H CYS A 282 24.207 −28.690 −34.145 1.00 0.00 H
ATOM 821 N SER A 283 21.868 −29.506 −30.448 1.00 28.73 N
ATOM 822 CA SER A 283 21.390 −28.989 −29.163 1.00 28.52 C
ATOM 823 C SER A 283 21.182 −30.115 −28.154 1.00 28.71 C
ATOM 824 O SER A 283 20.615 −31.136 −28.525 1.00 29.07 O
ATOM 825 CB SER A 283 20.058 −28.282 −29.402 1.00 28.54 C
ATOM 826 OG SER A 283 19.662 −27.658 −28.204 1.00 28.27 O
ATOM 827 H SER A 283 21.394 −30.333 −30.802 1.00 0.00 H
ATOM 828 HG SER A 283 20.285 −26.913 −28.102 1.00 0.00 H
ATOM 829 N SER A 284 21.634 −29.978 −26.903 1.00 28.47 N
ATOM 830 CA SER A 284 21.833 −31.135 −26.009 1.00 28.60 C
ATOM 831 C SER A 284 21.270 −30.981 −24.588 1.00 28.50 C
ATOM 832 O SER A 284 20.991 −29.874 −24.130 1.00 28.17 O
ATOM 833 CB SER A 284 23.321 −31.492 −25.992 1.00 28.41 C
ATOM 834 OG SER A 284 24.068 −30.502 −25.322 1.00 28.02 O
ATOM 835 H SER A 284 22.062 −29.099 −26.635 1.00 0.00 H
ATOM 836 HG SER A 284 23.939 −29.631 −25.742 1.00 0.00 H
ATOM 837 N THR A 285 20.986 −32.107 −23.921 1.00 28.82 N
ATOM 838 CA THR A 285 20.414 −32.199 −22.558 1.00 28.79 C
ATOM 839 C THR A 285 20.697 −33.579 −21.938 1.00 28.95 C
ATOM 840 O THR A 285 20.427 −34.594 −22.582 1.00 29.31 O
ATOM 841 CB THR A 285 18.891 −31.979 −22.629 1.00 29.06 C
ATOM 842 OG1 THR A 285 18.615 −30.635 −22.942 1.00 28.88 O
ATOM 843 CG2 THR A 285 18.153 −32.260 −21.324 1.00 29.12 C

ATOM 844 H THR A 285 21.140 −32.988 −24.405 1.00 0.00 H

ATOM 845 HG1 THR A 285 19.369 −30.296 −23.451 1.00 0.00 H

ATOM 846 N TYR A 286 21.145 −33.649 −20.677 1.00 28.68 N

ATOM 847 CA TYR A 286 21.287 −34.919 −19.943 1.00 28.79 C

ATOM 848 C TYR A 286 19.981 −35.369 −19.274 1.00 29.13 C

ATOM 849 O TYR A 286 19.447 −34.636 −18.437 1.00 29.15 O

ATOM 850 CB TYR A 286 22.385 −34.801 −18.881 1.00 28.37 C

ATOM 851 CG TYR A 286 22.529 −36.010 −17.974 1.00 28.43 C

ATOM 852 CD1 TYR A 286 23.097 −37.194 −18.477 1.00 28.48 C

ATOM 853 CD2 TYR A 286 22.058 −35.969 −16.646 1.00 28.45 C

ATOM 854 CE1 TYR A 286 23.179 −38.336 −17.660 1.00 28.55 C

ATOM 855 CE2 TYR A 286 22.133 −37.109 −15.827 1.00 28.52 C

ATOM 856 CZ TYR A 286 22.692 −38.298 −16.337 1.00 28.56 C

ATOM 857 OH TYR A 286 22.755 −39.404 −15.553 1.00 28.63 O

ATOM 858 H TYR A 286 21.417 −32.791 −20.195 1.00 0.00 H

ATOM 859 HH TYR A 286 22.729 −39.201 −14.618 1.00 0.00 H

ATOM 860 N HIS A 287 19.656 −36.656 −19.397 1.00 29.43 N

ATOM 861 CA HIS A 287 18.632 −37.364 −18.623 1.00 29.74 C

ATOM 862 C HIS A 287 19.028 −38.844 −18.440 1.00 29.98 C

ATOM 863 O HIS A 287 19.458 −39.489 −19.389 1.00 30.22 O

ATOM 864 CB HIS A 287 17.262 −37.162 −19.303 1.00 30.15 C

ATOM 865 CG HIS A 287 16.217 −38.179 −18.927 1.00 30.49 C

ATOM 866 ND1 HIS A 287 16.001 −39.400 −19.576 1.00 31.01 N

ATOM 867 CD2 HIS A 287 15.449 −38.126 −17.804 1.00 30.40 C

ATOM 868 CE1 HIS A 287 15.092 −40.045 −18.822 1.00 31.24 C

ATOM 869 NE2 HIS A 287 14.749 −39.307 −17.754 1.00 30.87 N

ATOM 870 H HIS A 287 20.220 −37.235 −20.017 1.00 0.00 H

ATOM 871 HE2 HIS A 287 14.274 −39.679 −16.923 1.00 0.00 H

ATOM 872 N GLU A 288 19.054 −39.299 −17.181 1.00 49.70 N

ATOM 873 CA GLU A 288 19.219 −40.702 −16.737 1.00 49.93 C

ATOM 874 C GLU A 288 20.207 −41.560 −17.562 1.00 49.97 C

ATOM 875 O GLU A 288 19.822 −42.364 −18.404 1.00 50.41 O

ATOM 876 CB GLU A 288 17.835 −41.355 −16.555 1.00 50.49 C

ATOM 877 CG GLU A 288 17.053 −40.650 −15.429 1.00 50.53 C

ATOM 878 CD GLU A 288 15.624 −41.182 −15.266 1.00 51.14 C

ATOM 879 OE1 GLU A 288 15.458 −42.419 −15.209 1.00 51.54 O

ATOM 880 OE2 GLU A 288 14.707 −40.331 −15.183 1.00 51.23 O

ATOM 881 H GLU A 288 18.656 −38.686 −16.491 1.00 0.00 H

ATOM 882 N ASP A 289 21.507 −41.382 −17.299 1.00 33.41 N

ATOM 883 CA ASP A 289 22.657 −41.975 −18.005 1.00 33.44 C

ATOM 884 C ASP A 289 22.823 −41.630 −19.495 1.00 33.66 C

ATOM 885 O ASP A 289 23.880 −41.935 −20.052 1.00 33.88 O

ATOM 886 CB ASP A 289 22.789 −43.491 −17.757 1.00 33.75 C

ATOM 887 CG ASP A 289 23.340 −43.883 −16.386 1.00 33.52 C

ATOM 888 OD1 ASP A 289 24.017 −43.045 −15.742 1.00 33.39 O

ATOM 889 OD2 ASP A 289 23.561 −45.103 −16.227 1.00 33.49 O

ATOM 890 H ASP A 289 21.744 −40.703 −16.588 1.00 0.00 H

ATOM 891 N PHE A 290 21.958 −40.812 −20.097 1.00 29.92 N

ATOM 892 CA PHE A 290 22.097 −40.378 −21.490 1.00 29.96 C

ATOM 893 C PHE A 290 22.221 −38.859 −21.614 1.00 29.56 C

ATOM 894 O PHE A 290 21.340 −38.112 −21.194 1.00 29.39 O

ATOM 895 CB PHE A 290 20.932 −40.909 −22.335 1.00 30.46 C

ATOM 896 CG PHE A 290 20.602 −42.373 −22.111 1.00 30.94 C

ATOM 897 CD1 PHE A 290 21.484 −43.378 −22.546 1.00 31.25 C

ATOM	898	CD2	PHE	A	290	19.443	−42.724	−21.395	1.00	31.12	C
ATOM	899	CE1	PHE	A	290	21.209	−44.727	−22.267	1.00	31.74	C
ATOM	900	CE2	PHE	A	290	19.175	−44.072	−21.102	1.00	31.60	C
ATOM	901	CZ	PHE	A	290	20.058	−45.075	−21.537	1.00	31.92	C
ATOM	902	H	PHE	A	290	21.096	−40.572	−19.612	1.00	0.00	H
ATOM	903	HZ	PHE	A	290	19.870	−46.103	−21.268	1.00	0.00	H
ATOM	904	N	TYR	A	291	23.187	−38.398	−22.408	1.00	29.46	N
ATOM	905	CA	TYR	A	291	23.070	−37.092	−23.052	1.00	29.29	C
ATOM	906	C	TYR	A	291	22.317	−37.260	−24.377	1.00	29.70	C
ATOM	907	O	TYR	A	291	22.768	−37.947	−25.300	1.00	29.98	O
ATOM	908	CB	TYR	A	291	24.439	−36.414	−23.192	1.00	28.88	C
ATOM	909	CG	TYR	A	291	24.534	−35.090	−22.456	1.00	28.44	C
ATOM	910	CD1	TYR	A	291	25.375	−34.949	−21.332	1.00	28.12	C
ATOM	911	CD2	TYR	A	291	23.805	−33.983	−22.932	1.00	28.34	C
ATOM	912	CE1	TYR	A	291	25.460	−33.708	−20.668	1.00	27.73	C
ATOM	913	CE2	TYR	A	291	23.900	−32.741	−22.275	1.00	27.97	C
ATOM	914	CZ	TYR	A	291	24.709	−32.609	−21.131	1.00	27.66	C
ATOM	915	OH	TYR	A	291	24.703	−31.442	−20.443	1.00	27.31	O
ATOM	916	H	TYR	A	291	23.824	−39.069	−22.825	1.00	0.00	H
ATOM	917	HH	TYR	A	291	23.810	−31.266	−20.096	1.00	0.00	H
ATOM	918	N	TYR	A	292	21.108	−36.708	−24.427	1.00	37.87	N
ATOM	919	CA	TYR	A	292	20.309	−36.565	−25.639	1.00	38.21	C
ATOM	920	C	TYR	A	292	20.795	−35.329	−26.406	1.00	37.92	C
ATOM	921	O	TYR	A	292	21.166	−34.321	−25.799	1.00	37.57	O
ATOM	922	CB	TYR	A	292	18.822	−36.436	−25.274	1.00	38.48	C
ATOM	923	CG	TYR	A	292	18.215	−37.617	−24.528	1.00	38.79	C
ATOM	924	CD1	TYR	A	292	17.333	−38.504	−25.181	1.00	39.29	C

ATOM 925 CD2 TYR A 292 18.480 −37.795 −23.155 1.00 38.61 C
ATOM 926 CE1 TYR A 292 16.731 −39.565 −24.472 1.00 39.62 C
ATOM 927 CE2 TYR A 292 17.884 −38.856 −22.451 1.00 38.91 C
ATOM 928 CZ TYR A 292 17.000 −39.735 −23.098 1.00 39.42 C
ATOM 929 OH TYR A 292 16.403 −40.727 −22.390 1.00 39.75 O
ATOM 930 H TYR A 292 20.815 −36.154 −23.628 1.00 0.00 H
ATOM 931 HH TYR A 292 16.318 −40.465 −21.458 1.00 0.00 H
ATOM 932 N THR A 293 20.795 −35.383 −27.738 1.00 29.96 N
ATOM 933 CA THR A 293 21.269 −34.285 −28.587 1.00 29.69 C
ATOM 934 C THR A 293 20.570 −34.263 −29.950 1.00 29.95 C
ATOM 935 O THR A 293 20.570 −35.239 −30.697 1.00 30.26 O
ATOM 936 CB THR A 293 22.803 −34.280 −28.675 1.00 29.41 C
ATOM 937 OG1 THR A 293 23.254 −33.191 −29.445 1.00 29.45 O
ATOM 938 CG2 THR A 293 23.419 −35.541 −29.274 1.00 29.54 C
ATOM 939 H THR A 293 20.556 −36.258 −28.195 1.00 0.00 H
ATOM 940 HG1 THR A 293 24.213 −33.217 −29.434 1.00 0.00 H
ATOM 941 N LEU A 294 19.791 −33.206 −30.166 1.00 29.82 N
ATOM 942 CA LEU A 294 18.923 −32.956 −31.311 1.00 30.04 C
ATOM 943 C LEU A 294 19.660 −32.108 −32.360 1.00 29.83 C
ATOM 944 O LEU A 294 20.055 −30.983 −32.064 1.00 29.50 O
ATOM 945 CB LEU A 294 17.666 −32.257 −30.754 1.00 30.03 C
ATOM 946 CG LEU A 294 16.650 −31.785 −31.804 1.00 30.29 C
ATOM 947 CD1 LEU A 294 16.073 −32.945 −32.607 1.00 30.80 C
ATOM 948 CD2 LEU A 294 15.499 −31.050 −31.119 1.00 30.22 C
ATOM 949 H LEU A 294 19.866 −32.450 −29.493 1.00 0.00 H
ATOM 950 N CYS A 295 19.743 −32.583 −33.602 1.00 30.05 N
ATOM 951 CA CYS A 295 20.414 −31.915 −34.723 1.00 29.93 C

ATOM	952	C	CYS	A	295	19.472	−31.741	−35.926	1.00	30.19	C
ATOM	953	O	CYS	A	295	18.683	−32.635	−36.254	1.00	30.50	O
ATOM	954	CB	CYS	A	295	21.642	−32.731	−35.146	1.00	30.05	C
ATOM	955	SG	CYS	A	295	22.896	−33.093	−33.883	1.00	29.80	S
ATOM	956	H	CYS	A	295	19.356	−33.503	−33.784	1.00	0.00	H
ATOM	957	N	ALA	A	296	19.672	−30.664	−36.686	1.00	30.06	N
ATOM	958	CA	ALA	A	296	19.045	−30.474	−37.992	1.00	30.32	C
ATOM	959	C	ALA	A	296	19.756	−31.302	−39.076	1.00	30.46	C
ATOM	960	O	ALA	A	296	20.987	−31.283	−39.164	1.00	30.28	O
ATOM	961	CB	ALA	A	296	19.064	−28.979	−38.317	1.00	30.10	C
ATOM	962	H	ALA	A	296	20.419	−30.021	−36.438	1.00	0.00	H
ATOM	963	N	VAL	A	297	18.984	−31.950	−39.951	1.00	30.78	N
ATOM	964	CA	VAL	A	297	19.468	−32.678	−41.134	1.00	30.98	C
ATOM	965	C	VAL	A	297	19.127	−31.875	−42.389	1.00	30.87	C
ATOM	966	O	VAL	A	297	18.004	−31.389	−42.514	1.00	30.84	O
ATOM	967	CB	VAL	A	297	18.851	−34.089	−41.227	1.00	31.52	C
ATOM	968	CG1	VAL	A	297	19.548	−34.931	−42.304	1.00	31.76	C
ATOM	969	CG2	VAL	A	297	18.906	−34.861	−39.903	1.00	31.70	C
ATOM	970	H	VAL	A	297	17.974	−31.892	−39.848	1.00	0.00	H
ATOM	971	N	SER	A	298	20.038	−31.756	−43.360	1.00	30.82	N
ATOM	972	CA	SER	A	298	19.801	−30.929	−44.555	1.00	30.79	C
ATOM	973	C	SER	A	298	20.377	−31.516	−45.841	1.00	30.94	C
ATOM	974	O	SER	A	298	21.553	−31.866	−45.908	1.00	30.88	O
ATOM	975	CB	SER	A	298	20.364	−29.529	−44.336	1.00	30.35	C
ATOM	976	OG	SER	A	298	20.047	−28.697	−45.428	1.00	30.29	O
ATOM	977	H	SER	A	298	20.962	−32.151	−43.211	1.00	0.00	H
ATOM	978	HG	SER	A	298	20.532	−27.860	−45.252	1.00	0.00	H

ATOM 979 N HIS A 299 19.592 −31.447 −46.922 1.00 31.13 N
ATOM 980 CA HIS A 299 20.047 −31.680 −48.302 1.00 31.31 C
ATOM 981 C HIS A 299 20.580 −30.411 −48.994 1.00 30.95 C
ATOM 982 O HIS A 299 21.123 −30.503 −50.090 1.00 30.98 O
ATOM 983 CB HIS A 299 18.896 −32.314 −49.115 1.00 31.69 C
ATOM 984 CG HIS A 299 19.210 −33.639 −49.768 1.00 32.16 C
ATOM 985 ND1 HIS A 299 18.514 −34.157 −50.860 1.00 32.47 N
ATOM 986 CD2 HIS A 299 20.090 −34.588 −49.327 1.00 32.40 C
ATOM 987 CE1 HIS A 299 19.014 −35.379 −51.081 1.00 32.89 C
ATOM 988 NE2 HIS A 299 19.964 −35.666 −50.176 1.00 32.86 N
ATOM 989 H HIS A 299 18.648 −31.115 −46.793 1.00 0.00 H
ATOM 990 HE2 HIS A 299 20.477 −36.534 −50.123 1.00 0.00 H
ATOM 991 N VAL A 300 20.430 −29.234 −48.373 1.00 37.47 N
ATOM 992 CA VAL A 300 20.701 −27.912 −48.982 1.00 37.19 C
ATOM 993 C VAL A 300 21.814 −27.133 −48.265 1.00 36.77 C
ATOM 994 O VAL A 300 22.018 −25.951 −48.526 1.00 36.52 O
ATOM 995 CB VAL A 300 19.407 −27.081 −49.113 1.00 37.21 C
ATOM 996 CG1 VAL A 300 18.363 −27.818 −49.963 1.00 37.63 C
ATOM 997 CG2 VAL A 300 18.773 −26.731 −47.760 1.00 37.10 C
ATOM 998 H VAL A 300 20.024 −29.237 −47.446 1.00 0.00 H
ATOM 999 N GLY A 301 22.529 −27.778 −47.340 1.00 38.24 N
ATOM 1000 CA GLY A 301 23.466 −27.114 −46.436 1.00 37.87 C
ATOM 1001 C GLY A 301 22.743 −26.305 −45.353 1.00 37.71 C
ATOM 1002 O GLY A 301 21.701 −26.724 −44.860 1.00 37.88 O
ATOM 1003 H GLY A 301 22.290 −28.738 −47.149 1.00 0.00 H
ATOM 1004 N ASP A 302 23.335 −25.183 −44.948 1.00 29.01 N
ATOM 1005 CA ASP A 302 22.908 −24.343 −43.820 1.00 28.85 C

ATOM 1006 C ASP A 302 21.398 −23.977 −43.869 1.00 28.88 C

ATOM 1007 O ASP A 302 20.968 −23.246 −44.769 1.00 28.76 O

ATOM 1008 CB ASP A 302 23.832 −23.116 −43.788 1.00 28.51 C

ATOM 1009 CG ASP A 302 23.573 −22.135 −42.638 1.00 28.35 C

ATOM 1010 OD1 ASP A 302 22.392 −21.792 −42.417 1.00 28.51 O

ATOM 1011 OD2 ASP A 302 24.564 −21.501 −42.228 1.00 28.09 O

ATOM 1012 H ASP A 302 24.144 −24.864 −45.455 1.00 0.00 H

ATOM 1013 N PRO A 303 20.582 −24.454 −42.904 1.00 29.07 N

ATOM 1014 CA PRO A 303 19.149 −24.176 −42.857 1.00 29.18 C

ATOM 1015 C PRO A 303 18.764 −22.710 −42.663 1.00 28.93 C

ATOM 1016 O PRO A 303 17.672 −22.324 −43.071 1.00 28.97 O

ATOM 1017 CB PRO A 303 18.629 −24.989 −41.674 1.00 29.41 C

ATOM 1018 CG PRO A 303 19.568 −26.185 −41.626 1.00 29.52 C

ATOM 1019 CD PRO A 303 20.900 −25.519 −41.955 1.00 29.21 C

ATOM 1020 N ILE A 304 19.603 −21.880 −42.038 1.00 28.67 N

ATOM 1021 CA ILE A 304 19.321 −20.449 −41.842 1.00 28.45 C

ATOM 1022 C ILE A 304 19.552 −19.687 −43.149 1.00 28.38 C

ATOM 1023 O ILE A 304 18.659 −18.987 −43.634 1.00 28.33 O

ATOM 1024 CB ILE A 304 20.190 −19.891 −40.689 1.00 28.19 C

ATOM 1025 CG1 ILE A 304 19.950 −20.631 −39.354 1.00 28.25 C

ATOM 1026 CG2 ILE A 304 19.987 −18.375 −40.510 1.00 27.99 C

ATOM 1027 CD1 ILE A 304 18.536 −20.497 −38.768 1.00 28.30 C

ATOM 1028 H ILE A 304 20.554 −22.197 −41.856 1.00 0.00 H

ATOM 1029 N LEU A 305 20.673 −19.970 −43.819 1.00 28.40 N

ATOM 1030 CA LEU A 305 21.036 −19.400 −45.118 1.00 28.32 C

ATOM 1031 C LEU A 305 20.267 −20.019 −46.303 1.00 28.53 C

ATOM 1032 O LEU A 305 20.334 −19.488 −47.414 1.00 28.48 O

```
ATOM   1033  CB  LEU A 305      22.561 −19.507 −45.306  1.00 28.17           C
ATOM   1034  CG  LEU A 305      23.409 −18.823 −44.211  1.00 27.94           C
ATOM   1035  CD1 LEU A 305      24.893 −19.021 −44.521  1.00 27.86           C
ATOM   1036  CD2 LEU A 305      23.146 −17.316 −44.122  1.00 27.76           C
ATOM   1037  H   LEU A 305      21.330 −20.614 −43.370  1.00  0.00           H
ATOM   1038  N   ASN A 306      19.490 −21.088 −46.092  1.00 28.79           N
ATOM   1039  CA  ASN A 306      18.670 −21.736 −47.125  1.00 29.02           C
ATOM   1040  C   ASN A 306      17.295 −22.229 −46.621  1.00 29.27           C
ATOM   1041  O   ASN A 306      16.687 −23.113 −47.223  1.00 29.56           O
ATOM   1042  CB  ASN A 306      19.506 −22.812 −47.861  1.00 29.14           C
ATOM   1043  CG  ASN A 306      19.685 −22.480 −49.331  1.00 29.16           C
ATOM   1044  OD1 ASN A 306      19.054 −23.053 −50.204  1.00 29.42           O
ATOM   1045  ND2 ASN A 306      20.260 −21.338 −49.629  1.00 28.91           N
ATOM   1046  H   ASN A 306      19.628 −21.581 −45.214  1.00  0.00           H
ATOM   1047 1HD2 ASN A 306      20.570 −20.723 −48.883  1.00  0.00           H
ATOM   1048 2HD2 ASN A 306      20.264 −21.069 −50.593  1.00  0.00           H
ATOM   1049  N   SER A 307      16.679 −21.504 −45.680  1.00 37.31           N
ATOM   1050  CA  SER A 307      15.384 −21.867 −45.067  1.00 37.51           C
ATOM   1051  C   SER A 307      14.205 −22.026 −46.044  1.00 37.80           C
ATOM   1052  O   SER A 307      13.201 −22.648 −45.695  1.00 38.09           O
ATOM   1053  CB  SER A 307      15.010 −20.798 −44.036  1.00 37.32           C
ATOM   1054  OG  SER A 307      13.794 −21.127 −43.399  1.00 37.52           O
ATOM   1055  H   SER A 307      17.258 −20.848 −45.167  1.00  0.00           H
ATOM   1056  HG  SER A 307      13.181 −21.505 −44.043  1.00  0.00           H
ATOM   1057  N   THR A 308      14.250 −21.373 −47.211  1.00 47.88           N
ATOM   1058  CA  THR A 308      13.223 −21.512 −48.267  1.00 48.11           C
ATOM   1059  C   THR A 308      13.362 −22.796 −49.094  1.00 48.41           C
```

ATOM 1060 O THR A 308 12.437 −23.143 −49.823 1.00 48.70 O

ATOM 1061 CB THR A 308 13.214 −20.278 −49.183 1.00 47.94 C

ATOM 1062 OG1 THR A 308 12.090 −20.308 −50.026 1.00 48.12 O

ATOM 1063 CG2 THR A 308 14.452 −20.149 −50.074 1.00 47.91 C

ATOM 1064 H THR A 308 15.103 −20.884 −47.434 1.00 0.00 H

ATOM 1065 HG1 THR A 308 11.995 −21.216 −50.354 1.00 0.00 H

ATOM 1066 N SER A 309 14.512 −23.465 −49.007 1.00 30.05 N

ATOM 1067 CA SER A 309 14.844 −24.720 −49.694 1.00 30.35 C

ATOM 1068 C SER A 309 14.860 −25.911 −48.725 1.00 30.55 C

ATOM 1069 O SER A 309 14.755 −27.060 −49.146 1.00 30.83 O

ATOM 1070 CB SER A 309 16.241 −24.607 −50.322 1.00 30.22 C

ATOM 1071 OG SER A 309 16.469 −23.358 −50.947 1.00 30.03 O

ATOM 1072 H SER A 309 15.252 −23.065 −48.445 1.00 0.00 H

ATOM 1073 HG SER A 309 17.429 −23.190 −50.863 1.00 0.00 H

ATOM 1074 N TRP A 310 15.109 −25.654 −47.436 1.00 30.42 N

ATOM 1075 CA TRP A 310 15.312 −26.683 −46.416 1.00 30.56 C

ATOM 1076 C TRP A 310 14.010 −27.414 −46.048 1.00 30.94 C

ATOM 1077 O TRP A 310 13.085 −26.836 −45.478 1.00 30.94 O

ATOM 1078 CB TRP A 310 15.995 −26.054 −45.190 1.00 30.25 C

ATOM 1079 CG TRP A 310 16.273 −26.997 −44.058 1.00 30.32 C

ATOM 1080 CD1 TRP A 310 17.028 −28.116 −44.136 1.00 30.36 C

ATOM 1081 CD2 TRP A 310 15.765 −26.965 −42.687 1.00 30.34 C

ATOM 1082 NE1 TRP A 310 17.006 −28.780 −42.926 1.00 30.40 N

ATOM 1083 CE2 TRP A 310 16.231 −28.124 −41.998 1.00 30.38 C

ATOM 1084 CE3 TRP A 310 14.969 −26.065 −41.947 1.00 30.34 C

ATOM 1085 CZ2 TRP A 310 15.877 −28.414 −40.674 1.00 30.41 C

ATOM 1086 CZ3 TRP A 310 14.600 −26.343 −40.617 1.00 30.39 C

ATOM 1087 CH2 TRP A 310 15.039 −27.521 −39.985 1.00 30.42 C

ATOM 1088 H TRP A 310 15.291 −24.696 −47.174 1.00 0.00 H

ATOM 1089 HE1 TRP A 310 17.381 −29.717 −42.787 1.00 0.00 H

ATOM 1090 N THR A 311 13.988 −28.730 −46.259 1.00 63.68 N

ATOM 1091 CA THR A 311 13.044 −29.658 −45.620 1.00 64.05 C

ATOM 1092 C THR A 311 13.395 −29.802 −44.137 1.00 63.88 C

ATOM 1093 O THR A 311 14.513 −30.223 −43.848 1.00 63.62 O

ATOM 1094 CB THR A 311 13.144 −31.044 −46.283 1.00 64.38 C

ATOM 1095 OG1 THR A 311 14.486 −31.487 −46.273 1.00 64.15 O

ATOM 1096 CG2 THR A 311 12.672 −31.043 −47.736 1.00 64.60 C

ATOM 1097 H THR A 311 14.766 −29.147 −46.749 1.00 0.00 H

ATOM 1098 HG1 THR A 311 14.822 −31.301 −45.382 1.00 0.00 H

ATOM 1099 N GLU A 312 12.437 −29.637 −43.215 1.00 31.64 N

ATOM 1100 CA GLU A 312 12.686 −29.657 −41.754 1.00 31.46 C

ATOM 1101 C GLU A 312 12.916 −31.056 −41.137 1.00 31.70 C

ATOM 1102 O GLU A 312 12.334 −31.428 −40.120 1.00 31.93 O

ATOM 1103 CB GLU A 312 11.648 −28.810 −40.996 1.00 31.48 C

ATOM 1104 CG GLU A 312 10.209 −29.360 −40.955 1.00 31.91 C

ATOM 1105 CD GLU A 312 9.523 −29.123 −39.596 1.00 31.86 C

ATOM 1106 OE1 GLU A 312 8.442 −29.734 −39.427 1.00 32.21 O

ATOM 1107 OE2 GLU A 312 9.649 −27.990 −39.087 1.00 31.49 O

ATOM 1108 H GLU A 312 11.525 −29.334 −43.514 1.00 0.00 H

ATOM 1109 N SER A 313 13.772 −31.859 −41.768 1.00 31.66 N

ATOM 1110 CA SER A 313 14.220 −33.147 −41.247 1.00 31.95 C

ATOM 1111 C SER A 313 15.060 −32.943 −39.983 1.00 31.68 C

ATOM 1112 O SER A 313 16.156 −32.379 −40.026 1.00 31.28 O

ATOM 1113 CB SER A 313 15.004 −33.891 −42.327 1.00 32.07 C

ATOM 1114 OG SER A 313 15.368 −35.179 −41.871 1.00 32.36 O
ATOM 1115 H SER A 313 14.290 −31.450 −42.536 1.00 0.00 H
ATOM 1116 HG SER A 313 15.892 −35.609 −42.551 1.00 0.00 H
ATOM 1117 N LEU A 314 14.518 −33.353 −38.838 1.00 31.91 N
ATOM 1118 CA LEU A 314 15.201 −33.325 −37.547 1.00 31.66 C
ATOM 1119 C LEU A 314 15.597 −34.742 −37.129 1.00 31.94 C
ATOM 1120 O LEU A 314 14.848 −35.702 −37.334 1.00 32.37 O
ATOM 1121 CB LEU A 314 14.324 −32.622 −36.493 1.00 31.53 C
ATOM 1122 CG LEU A 314 13.955 −31.159 −36.822 1.00 31.20 C
ATOM 1123 CD1 LEU A 314 13.219 −30.532 −35.636 1.00 31.20 C
ATOM 1124 CD2 LEU A 314 15.189 −30.303 −37.113 1.00 30.76 C
ATOM 1125 H LEU A 314 13.597 −33.764 −38.874 1.00 0.00 H
ATOM 1126 N SER A 315 16.735 −34.866 −36.448 1.00 31.68 N
ATOM 1127 CA SER A 315 17.180 −36.144 −35.890 1.00 31.89 C
ATOM 1128 C SER A 315 17.784 −35.980 −34.504 1.00 31.54 C
ATOM 1129 O SER A 315 18.568 −35.062 −34.265 1.00 31.11 O
ATOM 1130 CB SER A 315 18.134 −36.856 −36.846 1.00 32.00 C
ATOM 1131 OG SER A 315 19.362 −36.162 −36.999 1.00 31.65 O
ATOM 1132 H SER A 315 17.292 −34.038 −36.256 1.00 0.00 H
ATOM 1133 HG SER A 315 19.170 −35.217 −36.925 1.00 0.00 H
ATOM 1134 N LEU A 316 17.384 −36.851 −33.583 1.00 31.74 N
ATOM 1135 CA LEU A 316 17.939 −36.928 −32.240 1.00 31.44 C
ATOM 1136 C LEU A 316 18.898 −38.114 −32.151 1.00 31.58 C
ATOM 1137 O LEU A 316 18.556 −39.240 −32.513 1.00 32.03 O
ATOM 1138 CB LEU A 316 16.792 −36.954 −31.214 1.00 31.53 C
ATOM 1139 CG LEU A 316 17.273 −36.978 −29.750 1.00 31.25 C
ATOM 1140 CD1 LEU A 316 16.301 −36.196 −28.865 1.00 31.16 C

ATOM 1141 CD2 LEU A 316 17.364 −38.403 −29.197 1.00 31.53 C
ATOM 1142 H LEU A 316 16.810 −37.629 −33.893 1.00 0.00 H
ATOM 1143 N ILE A 317 20.070 −37.872 −31.582 1.00 31.20 N
ATOM 1144 CA ILE A 317 20.992 −38.898 −31.109 1.00 31.26 C
ATOM 1145 C ILE A 317 20.883 −38.945 −29.583 1.00 31.02 C
ATOM 1146 O ILE A 317 20.764 −37.904 −28.939 1.00 30.58 O
ATOM 1147 CB ILE A 317 22.433 −38.618 −31.601 1.00 31.04 C
ATOM 1148 CG1 ILE A 317 22.492 −38.330 −33.122 1.00 31.28 C
ATOM 1149 CG2 ILE A 317 23.350 −39.814 −31.288 1.00 31.13 C
ATOM 1150 CD1 ILE A 317 22.469 −36.836 −33.474 1.00 31.03 C
ATOM 1151 H ILE A 317 20.258 −36.920 −31.278 1.00 0.00 H
ATOM 1152 N ARG A 318 20.995 −40.126 −28.977 1.00 31.34 N
ATOM 1153 CA ARG A 318 21.327 −40.251 −27.550 1.00 31.16 C
ATOM 1154 C ARG A 318 22.633 −41.012 −27.393 1.00 31.10 C
ATOM 1155 O ARG A 318 22.848 −42.006 −28.089 1.00 31.47 O
ATOM 1156 CB ARG A 318 20.135 −40.799 −26.742 1.00 31.56 C
ATOM 1157 CG ARG A 318 19.896 −42.318 −26.838 1.00 32.13 C
ATOM 1158 CD ARG A 318 20.722 −43.107 −25.809 1.00 32.27 C
ATOM 1159 NE ARG A 318 20.310 −44.519 −25.708 1.00 32.85 N
ATOM 1160 CZ ARG A 318 19.320 −44.995 −24.975 1.00 33.29 C
ATOM 1161 NH1 ARG A 318 18.534 −44.231 −24.269 1.00 33.24 N
ATOM 1162 NH2 ARG A 318 19.083 −46.273 −24.972 1.00 33.82 N
ATOM 1163 H ARG A 318 21.082 −40.947 −29.570 1.00 0.00 H
ATOM 1164 HE ARG A 318 20.950 −45.211 −26.078 1.00 0.00 H
ATOM 1165 1HH1 ARG A 318 18.831 −43.284 −24.106 1.00 0.00 H
ATOM 1166 2HH1 ARG A 318 17.925 −44.634 −23.582 1.00 0.00 H
ATOM 1167 1HH2 ARG A 318 19.667 −46.870 −25.556 1.00 0.00 H

ATOM	1168	2HH2	ARG	A	318	18.344	−46.657	−24.418	1.00	0.00	H
ATOM	1169	N	LEU	A	319	23.464	−40.576	−26.455	1.00	30.64	N
ATOM	1170	CA	LEU	A	319	24.765	−41.166	−26.144	1.00	30.54	C
ATOM	1171	C	LEU	A	319	24.841	−41.404	−24.632	1.00	30.35	C
ATOM	1172	O	LEU	A	319	24.603	−40.485	−23.849	1.00	29.99	O
ATOM	1173	CB	LEU	A	319	25.889	−40.227	−26.632	1.00	30.17	C
ATOM	1174	CG	LEU	A	319	25.898	−39.897	−28.139	1.00	30.26	C
ATOM	1175	CD1	LEU	A	319	26.962	−38.844	−28.449	1.00	29.80	C
ATOM	1176	CD2	LEU	A	319	26.195	−41.124	−28.999	1.00	30.72	C
ATOM	1177	H	LEU	A	319	23.208	−39.735	−25.942	1.00	0.00	H
ATOM	1178	N	ALA	A	320	25.099	−42.644	−24.213	1.00	30.61	N
ATOM	1179	CA	ALA	A	320	25.290	−42.964	−22.800	1.00	30.44	C
ATOM	1180	C	ALA	A	320	26.560	−42.285	−22.263	1.00	29.90	C
ATOM	1181	O	ALA	A	320	27.632	−42.450	−22.847	1.00	29.81	O
ATOM	1182	CB	ALA	A	320	25.332	−44.484	−22.612	1.00	30.84	C
ATOM	1183	H	ALA	A	320	25.276	−43.367	−24.898	1.00	0.00	H
ATOM	1184	N	VAL	A	321	26.457	−41.558	−21.150	1.00	55.87	N
ATOM	1185	CA	VAL	A	321	27.574	−40.804	−20.543	1.00	55.36	C
ATOM	1186	C	VAL	A	321	28.640	−41.714	−19.925	1.00	55.36	C
ATOM	1187	O	VAL	A	321	29.812	−41.350	−19.889	1.00	55.04	O
ATOM	1188	CB	VAL	A	321	27.066	−39.794	−19.493	1.00	54.95	C
ATOM	1189	CG1	VAL	A	321	26.022	−38.846	−20.097	1.00	54.90	C
ATOM	1190	CG2	VAL	A	321	26.477	−40.467	−18.244	1.00	55.05	C
ATOM	1191	H	VAL	A	321	25.541	−41.496	−20.710	1.00	0.00	H
ATOM	1192	N	ARG	A	322	28.264	−42.956	−19.596	1.00	37.18	N
ATOM	1193	CA	ARG	A	322	29.091	−43.948	−18.894	1.00	37.18	C
ATOM	1194	C	ARG	A	322	28.913	−45.340	−19.536	1.00	37.74	C

ATOM 1195 O ARG A 322 28.294 −46.225 −18.950 1.00 38.00 O

ATOM 1196 CB ARG A 322 28.709 −43.858 −17.410 1.00 37.05 C

ATOM 1197 CG ARG A 322 29.694 −44.531 −16.445 1.00 36.97 C

ATOM 1198 CD ARG A 322 29.337 −44.217 −14.981 1.00 36.47 C

ATOM 1199 NE ARG A 322 28.027 −44.784 −14.591 1.00 36.59 N

ATOM 1200 CZ ARG A 322 26.842 −44.204 −14.700 1.00 36.64 C

ATOM 1201 NH1 ARG A 322 26.672 −42.964 −15.050 1.00 36.58 N

ATOM 1202 NH2 ARG A 322 25.764 −44.904 −14.536 1.00 36.77 N

ATOM 1203 H ARG A 322 27.278 −43.156 −19.682 1.00 0.00 H

ATOM 1204 HE ARG A 322 28.020 −45.745 −14.308 1.00 0.00 H

ATOM 1205 1HH1 ARG A 322 27.463 −42.387 −15.274 1.00 0.00 H

ATOM 1206 2HH1 ARG A 322 25.712 −42.687 −15.276 1.00 0.00 H

ATOM 1207 1HH2 ARG A 322 25.793 −45.899 −14.511 1.00 0.00 H

ATOM 1208 2HH2 ARG A 322 24.901 −44.498 −14.924 1.00 0.00 H

ATOM 1209 N PRO A 323 29.234 −45.477 −20.837 1.00 30.14 N

ATOM 1210 CA PRO A 323 28.738 −46.534 −21.722 1.00 30.74 C

ATOM 1211 C PRO A 323 29.149 −47.934 −21.237 1.00 31.12 C

ATOM 1212 O PRO A 323 29.228 −48.878 −22.028 1.00 31.05 O

ATOM 1213 CB PRO A 323 29.303 −46.174 −23.105 1.00 30.74 C

ATOM 1214 CG PRO A 323 30.610 −45.469 −22.760 1.00 30.17 C

ATOM 1215 CD PRO A 323 30.194 −44.646 −21.547 1.00 29.79 C

ATOM 1216 N ASP A 329 22.436 −50.639 −28.123 1.00 34.57 N

ATOM 1217 CA ASP A 329 21.606 −49.435 −28.176 1.00 34.22 C

ATOM 1218 C ASP A 329 21.352 −48.781 −26.805 1.00 33.80 C

ATOM 1219 O ASP A 329 20.647 −47.770 −26.711 1.00 33.55 O

ATOM 1220 CB ASP A 329 20.295 −49.716 −28.922 1.00 34.68 C

ATOM 1221 CG ASP A 329 20.503 −49.898 −30.427 1.00 34.80 C

ATOM	1222	OD1	ASP	A	329	21.168	−50.880	−30.804	1.00	34.33	O
ATOM	1223	OD2	ASP	A	329	19.705	−49.262	−31.160	1.00	35.36	O
ATOM	1224	H	ASP	A	329	22.454	−51.205	−28.975	1.00	0.00	H
ATOM	1225	N	TYR	A	330	21.992	−49.276	−25.740	1.00	33.72	N
ATOM	1226	CA	TYR	A	330	22.263	−48.463	−24.546	1.00	33.20	C
ATOM	1227	C	TYR	A	330	23.263	−47.349	−24.886	1.00	32.60	C
ATOM	1228	O	TYR	A	330	22.895	−46.175	−24.919	1.00	32.21	O
ATOM	1229	CB	TYR	A	330	22.771	−49.346	−23.390	1.00	33.19	C
ATOM	1230	CG	TYR	A	330	23.259	−48.568	−22.172	1.00	32.61	C
ATOM	1231	CD1	TYR	A	330	22.338	−47.885	−21.351	1.00	32.26	C
ATOM	1232	CD2	TYR	A	330	24.634	−48.509	−21.866	1.00	32.45	C
ATOM	1233	CE1	TYR	A	330	22.785	−47.122	−20.250	1.00	31.77	C
ATOM	1234	CE2	TYR	A	330	25.080	−47.761	−20.757	1.00	31.93	C
ATOM	1235	CZ	TYR	A	330	24.162	−47.060	−19.947	1.00	31.61	C
ATOM	1236	OH	TYR	A	330	24.632	−46.334	−18.897	1.00	31.12	O
ATOM	1237	H	TYR	A	330	22.543	−50.109	−25.900	1.00	0.00	H
ATOM	1238	HH	TYR	A	330	23.968	−46.062	−18.248	1.00	0.00	H
ATOM	1239	N	ASN	A	331	24.436	−47.752	−25.383	1.00	32.56	N
ATOM	1240	CA	ASN	A	331	25.605	−46.900	−25.601	1.00	32.02	C
ATOM	1241	C	ASN	A	331	25.331	−45.725	−26.551	1.00	31.95	C
ATOM	1242	O	ASN	A	331	25.723	−44.597	−26.258	1.00	31.51	O
ATOM	1243	CB	ASN	A	331	26.769	−47.770	−26.130	1.00	32.05	C
ATOM	1244	CG	ASN	A	331	27.141	−48.938	−25.224	1.00	32.09	C
ATOM	1245	OD1	ASN	A	331	26.299	−49.681	−24.751	1.00	31.93	O
ATOM	1246	ND2	ASN	A	331	28.402	−49.104	−24.911	1.00	32.30	N
ATOM	1247	H	ASN	A	331	24.631	−48.746	−25.324	1.00	0.00	H
ATOM	1248	1HD2	ASN	A	331	29.108	−48.481	−25.252	1.00	0.00	H

ATOM 1249 2HD2 ASN A 331 28.594 −49.729 −24.140 1.00 0.00 H

ATOM 1250 N GLN A 332 24.650 −45.976 −27.671 1.00 32.39 N

ATOM 1251 CA GLN A 332 24.299 −44.978 −28.685 1.00 32.30 C

ATOM 1252 C GLN A 332 22.949 −45.341 −29.316 1.00 32.80 C

ATOM 1253 O GLN A 332 22.684 −46.521 −29.535 1.00 33.32 O

ATOM 1254 CB GLN A 332 25.379 −44.901 −29.786 1.00 32.24 C

ATOM 1255 CG GLN A 332 26.811 −44.674 −29.270 1.00 31.71 C

ATOM 1256 CD GLN A 332 27.807 −44.297 −30.363 1.00 31.54 C

ATOM 1257 OE1 GLN A 332 27.565 −44.419 −31.553 1.00 31.87 O

ATOM 1258 NE2 GLN A 332 28.975 −43.841 −29.977 1.00 31.04 N

ATOM 1259 H GLN A 332 24.312 −46.915 −27.826 1.00 0.00 H

ATOM 1260 1HE2 GLN A 332 29.169 −43.717 −28.988 1.00 0.00 H

ATOM 1261 2HE2 GLN A 332 29.648 −43.549 −30.674 1.00 0.00 H

ATOM 1262 N LYS A 333 22.153 −44.351 −29.735 1.00 32.66 N

ATOM 1263 CA LYS A 333 21.011 −44.566 −30.644 1.00 33.07 C

ATOM 1264 C LYS A 333 20.738 −43.320 −31.483 1.00 32.84 C

ATOM 1265 O LYS A 333 20.755 −42.214 −30.951 1.00 32.33 O

ATOM 1266 CB LYS A 333 19.764 −45.008 −29.853 1.00 33.24 C

ATOM 1267 CG LYS A 333 18.697 −45.631 −30.764 1.00 33.61 C

ATOM 1268 CD LYS A 333 17.467 −46.065 −29.953 1.00 33.92 C

ATOM 1269 CE LYS A 333 16.652 −47.132 −30.697 1.00 34.58 C

ATOM 1270 NZ LYS A 333 17.302 −48.463 −30.637 1.00 34.67 N1+

ATOM 1271 H LYS A 333 22.411 −43.396 −29.493 1.00 0.00 H

ATOM 1272 HZ1 LYS A 333 16.801 −49.177 −31.142 1.00 0.00 H

ATOM 1273 HZ2 LYS A 333 17.484 −48.775 −29.692 1.00 0.00 H

ATOM 1274 HZ3 LYS A 333 18.233 −48.447 −31.072 1.00 0.00 H

ATOM 1275 N TYR A 334 20.411 −43.515 −32.759 1.00 33.24 N

ATOM 1276 CA TYR A 334 19.962 −42.473 −33.691 1.00 33.11 C
ATOM 1277 C TYR A 334 18.449 −42.581 −33.944 1.00 33.44 C
ATOM 1278 O TYR A 334 17.930 −43.687 −34.101 1.00 33.93 O
ATOM 1279 CB TYR A 334 20.763 −42.611 −34.996 1.00 33.28 C
ATOM 1280 CG TYR A 334 20.545 −41.494 −35.999 1.00 32.90 C
ATOM 1281 CD1 TYR A 334 19.536 −41.593 −36.979 1.00 33.04 C
ATOM 1282 CD2 TYR A 334 21.367 −40.353 −35.954 1.00 32.40 C
ATOM 1283 CE1 TYR A 334 19.352 −40.550 −37.910 1.00 32.68 C
ATOM 1284 CE2 TYR A 334 21.163 −39.294 −36.858 1.00 32.06 C
ATOM 1285 CZ TYR A 334 20.167 −39.400 −37.850 1.00 32.19 C
ATOM 1286 OH TYR A 334 19.969 −38.378 −38.722 1.00 31.85 O
ATOM 1287 H TYR A 334 20.363 −44.464 −33.091 1.00 0.00 H
ATOM 1288 HH TYR A 334 20.019 −37.527 −38.262 1.00 0.00 H
ATOM 1289 N ILE A 335 17.752 −41.448 −34.044 1.00 33.18 N
ATOM 1290 CA ILE A 335 16.310 −41.345 −34.314 1.00 33.48 C
ATOM 1291 C ILE A 335 16.084 −40.233 −35.345 1.00 33.30 C
ATOM 1292 O ILE A 335 16.319 −39.064 −35.050 1.00 32.82 O
ATOM 1293 CB ILE A 335 15.519 −41.071 −33.004 1.00 33.41 C
ATOM 1294 CG1 ILE A 335 15.700 −42.218 −31.980 1.00 33.82 C
ATOM 1295 CG2 ILE A 335 14.023 −40.839 −33.304 1.00 33.54 C
ATOM 1296 CD1 ILE A 335 15.024 −41.975 −30.623 1.00 33.89 C
ATOM 1297 H ILE A 335 18.227 −40.578 −33.815 1.00 0.00 H
ATOM 1298 N ALA A 336 15.507 −40.563 −36.503 1.00 48.91 N
ATOM 1299 CA ALA A 336 14.855 −39.574 −37.365 1.00 48.81 C
ATOM 1300 C ALA A 336 13.444 −39.276 −36.825 1.00 48.88 C
ATOM 1301 O ALA A 336 12.671 −40.204 −36.576 1.00 49.36 O
ATOM 1302 CB ALA A 336 14.820 −40.108 −38.801 1.00 49.23 C

ATOM 1303 H ALA A 336 15.260 −41.528 −36.650 1.00 0.00 H

ATOM 1304 N ILE A 337 13.132 −38.007 −36.555 1.00 33.24 N

ATOM 1305 CA ILE A 337 11.854 −37.619 −35.943 1.00 33.27 C

ATOM 1306 C ILE A 337 10.745 −37.584 −36.999 1.00 33.57 C

ATOM 1307 O ILE A 337 10.852 −36.909 −38.017 1.00 33.45 O

ATOM 1308 CB ILE A 337 12.017 −36.291 −35.177 1.00 32.73 C

ATOM 1309 CG1 ILE A 337 12.875 −36.522 −33.908 1.00 32.41 C

ATOM 1310 CG2 ILE A 337 10.665 −35.690 −34.761 1.00 32.81 C

ATOM 1311 CD1 ILE A 337 13.977 −35.475 −33.772 1.00 31.85 C

ATOM 1312 H ILE A 337 13.774 −37.267 −36.827 1.00 0.00 H

ATOM 1313 N THR A 338 9.645 −38.289 −36.725 1.00 33.99 N

ATOM 1314 CA THR A 338 8.498 −38.439 −37.643 1.00 34.40 C

ATOM 1315 C THR A 338 7.315 −37.519 −37.323 1.00 34.37 C

ATOM 1316 O THR A 338 6.333 −37.512 −38.064 1.00 34.43 O

ATOM 1317 CB THR A 338 8.022 −39.899 −37.683 1.00 35.02 C

ATOM 1318 OG1 THR A 338 7.709 −40.356 −36.386 1.00 35.10 O

ATOM 1319 CG2 THR A 338 9.081 −40.844 −38.252 1.00 35.14 C

ATOM 1320 H THR A 338 9.632 −38.830 −35.874 1.00 0.00 H

ATOM 1321 HG1 THR A 338 8.436 −40.915 −36.101 1.00 0.00 H

ATOM 1322 N LYS A 339 7.394 −36.723 −36.246 1.00 44.67 N

ATOM 1323 CA LYS A 339 6.368 −35.754 −35.830 1.00 44.64 C

ATOM 1324 C LYS A 339 7.001 −34.518 −35.195 1.00 44.06 C

ATOM 1325 O LYS A 339 7.706 −34.636 −34.193 1.00 43.81 O

ATOM 1326 CB LYS A 339 5.399 −36.389 −34.823 1.00 45.08 C

ATOM 1327 CG LYS A 339 4.450 −37.428 −35.434 1.00 45.68 C

ATOM 1328 CD LYS A 339 3.465 −37.887 −34.356 1.00 46.21 C

ATOM 1329 CE LYS A 339 2.395 −38.812 −34.929 1.00 46.84 C

ATOM 1330 NZ LYS A 339 1.403 −39.148 −33.878 1.00 47.34 N1+

ATOM 1331 H LYS A 339 8.243 −36.763 −35.699 1.00 0.00 H

ATOM 1332 HZ1 LYS A 339 0.676 −39.745 −34.247 1.00 0.00 H

ATOM 1333 HZ2 LYS A 339 0.983 −38.297 −33.520 1.00 0.00 H

ATOM 1334 HZ3 LYS A 339 1.858 −39.618 −33.106 1.00 0.00 H

ATOM 1335 N VAL A 340 6.590 −33.344 −35.671 1.00 33.47 N

ATOM 1336 CA VAL A 340 6.974 −32.042 −35.117 1.00 32.97 C

ATOM 1337 C VAL A 340 5.751 −31.124 −35.114 1.00 33.00 C

ATOM 1338 O VAL A 340 5.136 −30.895 −36.154 1.00 33.08 O

ATOM 1339 CB VAL A 340 8.144 −31.405 −35.896 1.00 32.55 C

ATOM 1340 CG1 VAL A 340 8.675 −30.188 −35.137 1.00 32.09 C

ATOM 1341 CG2 VAL A 340 9.329 −32.359 −36.108 1.00 32.46 C

ATOM 1342 H VAL A 340 5.960 −33.333 −36.458 1.00 0.00 H

ATOM 1343 N GLU A 341 5.341 −30.652 −33.941 1.00 49.50 N

ATOM 1344 CA GLU A 341 4.078 −29.938 −33.728 1.00 49.55 C

ATOM 1345 C GLU A 341 4.362 −28.475 −33.355 1.00 49.06 C

ATOM 1346 O GLU A 341 4.981 −28.189 −32.331 1.00 48.77 O

ATOM 1347 CB GLU A 341 3.234 −30.675 −32.672 1.00 49.79 C

ATOM 1348 CG GLU A 341 2.808 −32.075 −33.165 1.00 50.32 C

ATOM 1349 CD GLU A 341 2.043 −32.921 −32.134 1.00 50.60 C

ATOM 1350 OE1 GLU A 341 2.082 −32.579 −30.928 1.00 50.33 O

ATOM 1351 OE2 GLU A 341 1.618 −34.032 −32.534 1.00 51.10 O

ATOM 1352 H GLU A 341 5.866 −30.920 −33.111 1.00 0.00 H

ATOM 1353 N ARG A 342 4.044 −27.543 −34.267 1.00 32.42 N

ATOM 1354 CA ARG A 342 4.571 −26.161 −34.234 1.00 31.99 C

ATOM 1355 C ARG A 342 3.576 −25.054 −34.609 1.00 32.01 C

ATOM 1356 O ARG A 342 3.972 −23.962 −35.009 1.00 31.69 O

ATOM 1357 CB ARG A 342 5.925 −26.076 −34.970 1.00 31.67 C
ATOM 1358 CG ARG A 342 5.940 −26.320 −36.489 1.00 31.83 C
ATOM 1359 CD ARG A 342 5.754 −27.792 −36.898 1.00 32.12 C
ATOM 1360 NE ARG A 342 6.327 −28.106 −38.220 1.00 32.19 N
ATOM 1361 CZ ARG A 342 6.017 −27.592 −39.394 1.00 32.47 C
ATOM 1362 NH1 ARG A 342 5.168 −26.627 −39.571 1.00 32.71 N
ATOM 1363 NH2 ARG A 342 6.628 −27.982 −40.456 1.00 32.50 N
ATOM 1364 H ARG A 342 3.613 −27.868 −35.119 1.00 0.00 H
ATOM 1365 HE ARG A 342 7.083 −28.795 −38.267 1.00 0.00 H
ATOM 1366 1HH1 ARG A 342 4.839 −26.086 −38.798 1.00 0.00 H
ATOM 1367 2HH1 ARG A 342 5.221 −26.165 −40.461 1.00 0.00 H
ATOM 1368 1HH2 ARG A 342 7.363 −28.665 −40.272 1.00 0.00 H
ATOM 1369 2HH2 ARG A 342 6.716 −27.339 −41.231 1.00 0.00 H
ATOM 1370 N GLY A 343 2.279 −25.299 −34.424 1.00 32.41 N
ATOM 1371 CA GLY A 343 1.241 −24.266 −34.533 1.00 32.51 C
ATOM 1372 C GLY A 343 1.241 −23.542 −35.887 1.00 32.44 C
ATOM 1373 O GLY A 343 1.014 −24.167 −36.920 1.00 32.74 O
ATOM 1374 H GLY A 343 2.026 −26.199 −34.049 1.00 0.00 H
ATOM 1375 N LYS A 344 1.447 −22.216 −35.878 1.00 32.05 N
ATOM 1376 CA LYS A 344 1.487 −21.372 −37.090 1.00 31.95 C
ATOM 1377 C LYS A 344 2.871 −21.250 −37.749 1.00 31.64 C
ATOM 1378 O LYS A 344 2.976 −20.586 −38.778 1.00 31.67 O
ATOM 1379 CB LYS A 344 0.877 −19.984 −36.783 1.00 31.81 C
ATOM 1380 CG LYS A 344 −0.319 −19.627 −37.685 1.00 32.03 C
ATOM 1381 CD LYS A 344 0.033 −19.432 −39.170 1.00 31.88 C
ATOM 1382 CE LYS A 344 −1.245 −19.149 −39.969 1.00 32.20 C
ATOM 1383 NZ LYS A 344 −0.968 −18.970 −41.414 1.00 32.04 N1+

ATOM	1384	H	LYS	A	344	1.653	−21.781	−34.989	1.00	0.00	H
ATOM	1385	HZ1	LYS	A	344	−0.380	−19.695	−41.793	1.00	0.00	H
ATOM	1386	HZ2	LYS	A	344	−0.503	−18.074	−41.591	1.00	0.00	H
ATOM	1387	HZ3	LYS	A	344	−1.813	−18.886	−41.958	1.00	0.00	H
ATOM	1388	N	TYR	A	345	3.925	−21.834	−37.175	1.00	31.63	N
ATOM	1389	CA	TYR	A	345	5.253	−21.852	−37.799	1.00	31.32	C
ATOM	1390	C	TYR	A	345	5.272	−22.804	−38.998	1.00	31.52	C
ATOM	1391	O	TYR	A	345	4.770	−23.925	−38.926	1.00	31.84	O
ATOM	1392	CB	TYR	A	345	6.342	−22.182	−36.768	1.00	31.08	C
ATOM	1393	CG	TYR	A	345	6.509	−21.056	−35.772	1.00	30.90	C
ATOM	1394	CD1	TYR	A	345	5.715	−21.007	−34.612	1.00	31.05	C
ATOM	1395	CD2	TYR	A	345	7.365	−19.982	−36.078	1.00	30.61	C
ATOM	1396	CE1	TYR	A	345	5.729	−19.858	−33.799	1.00	30.91	C
ATOM	1397	CE2	TYR	A	345	7.389	−18.837	−35.264	1.00	30.48	C
ATOM	1398	CZ	TYR	A	345	6.543	−18.758	−34.144	1.00	30.63	C
ATOM	1399	OH	TYR	A	345	6.492	−17.603	−33.439	1.00	30.51	O
ATOM	1400	H	TYR	A	345	3.782	−22.425	−36.367	1.00	0.00	H
ATOM	1401	HH	TYR	A	345	5.833	−17.687	−32.699	1.00	0.00	H
ATOM	1402	N	ASP	A	346	5.869	−22.350	−40.101	1.00	31.10	N
ATOM	1403	CA	ASP	A	346	6.030	−23.118	−41.345	1.00	31.28	C
ATOM	1404	C	ASP	A	346	6.996	−24.298	−41.149	1.00	31.32	C
ATOM	1405	O	ASP	A	346	6.691	−25.415	−41.580	1.00	31.66	O
ATOM	1406	CB	ASP	A	346	6.495	−22.127	−42.427	1.00	31.04	C
ATOM	1407	CG	ASP	A	346	6.955	−22.724	−43.768	1.00	31.26	C
ATOM	1408	OD1	ASP	A	346	7.858	−23.583	−43.717	1.00	31.53	O
ATOM	1409	OD2	ASP	A	346	6.922	−21.945	−44.746	1.00	31.17	O
ATOM	1410	H	ASP	A	346	6.360	−21.466	−40.034	1.00	0.00	H

ATOM	1411	HB2	ASP	A	346	5.683	−21.425	−42.615	1.00	0.00	H
ATOM	1412	HB3	ASP	A	346	7.331	−21.560	−42.017	1.00	0.00	H
ATOM	1413	N	LYS	A	347	8.019	−24.088	−40.309	1.00	31.00	N
ATOM	1414	CA	LYS	A	347	9.024	−25.069	−39.880	1.00	31.01	C
ATOM	1415	C	LYS	A	347	9.731	−24.635	−38.585	1.00	30.69	C
ATOM	1416	O	LYS	A	347	9.635	−23.466	−38.201	1.00	30.42	O
ATOM	1417	CB	LYS	A	347	10.021	−25.317	−41.029	1.00	30.92	C
ATOM	1418	CG	LYS	A	347	10.678	−24.031	−41.554	1.00	30.70	C
ATOM	1419	CD	LYS	A	347	11.762	−24.261	−42.609	1.00	30.51	C
ATOM	1420	CE	LYS	A	347	11.314	−24.966	−43.893	1.00	30.82	C
ATOM	1421	NZ	LYS	A	347	10.284	−24.214	−44.642	1.00	31.19	N1+
ATOM	1422	H	LYS	A	347	8.125	−23.148	−39.943	1.00	0.00	H
ATOM	1423	HZ1	LYS	A	347	10.019	−24.687	−45.490	1.00	0.00	H
ATOM	1424	HZ2	LYS	A	347	10.584	−23.277	−44.885	1.00	0.00	H
ATOM	1425	HZ3	LYS	A	347	9.424	−24.097	−44.097	1.00	0.00	H
ATOM	1426	N	VAL	A	348	10.462	−25.542	−37.932	1.00	30.74	N
ATOM	1427	CA	VAL	A	348	11.216	−25.325	−36.672	1.00	30.45	C
ATOM	1428	C	VAL	A	348	12.532	−26.126	−36.629	1.00	30.37	C
ATOM	1429	O	VAL	A	348	12.715	−27.076	−37.387	1.00	30.60	O
ATOM	1430	CB	VAL	A	348	10.369	−25.668	−35.427	1.00	30.60	C
ATOM	1431	CG1	VAL	A	348	9.206	−24.701	−35.198	1.00	30.99	C
ATOM	1432	CG2	VAL	A	348	9.812	−27.091	−35.479	1.00	30.67	C
ATOM	1433	H	VAL	A	348	10.440	−26.496	−38.302	1.00	0.00	H
ATOM	1434	N	MET	A	349	13.474	−25.744	−35.756	1.00	30.05	N
ATOM	1435	CA	MET	A	349	14.831	−26.317	−35.709	1.00	29.89	C
ATOM	1436	C	MET	A	349	15.505	−26.168	−34.324	1.00	29.58	C
ATOM	1437	O	MET	A	349	15.299	−25.136	−33.682	1.00	29.38	O

ATOM	1438	CB	MET	A	349	15.657	−25.606	−36.795	1.00	29.75	C
ATOM	1439	CG	MET	A	349	16.999	−26.261	−37.103	1.00	29.69	C
ATOM	1440	SD	MET	A	349	18.024	−25.355	−38.282	1.00	29.34	S
ATOM	1441	CE	MET	A	349	18.574	−23.966	−37.256	1.00	28.98	C
ATOM	1442	H	MET	A	349	13.302	−24.907	−35.207	1.00	0.00	H
ATOM	1443	HB2	MET	A	349	15.092	−25.600	−37.726	1.00	0.00	H
ATOM	1444	HB3	MET	A	349	15.822	−24.574	−36.491	1.00	0.00	H
ATOM	1445	N	PRO	A	350	16.368	−27.107	−33.871	1.00	29.54	N
ATOM	1446	CA	PRO	A	350	17.294	−26.876	−32.750	1.00	29.23	C
ATOM	1447	C	PRO	A	350	18.264	−25.722	−33.044	1.00	28.89	C
ATOM	1448	O	PRO	A	350	18.670	−25.519	−34.188	1.00	28.87	O
ATOM	1449	CB	PRO	A	350	18.069	−28.187	−32.573	1.00	29.24	C
ATOM	1450	CG	PRO	A	350	18.061	−28.772	−33.984	1.00	29.53	C
ATOM	1451	CD	PRO	A	350	16.663	−28.402	−34.472	1.00	29.80	C
ATOM	1452	N	TYR	A	351	18.590	−24.917	−32.030	1.00	28.63	N
ATOM	1453	CA	TYR	A	351	19.341	−23.674	−32.232	1.00	28.32	C
ATOM	1454	C	TYR	A	351	20.016	−23.186	−30.940	1.00	28.05	C
ATOM	1455	O	TYR	A	351	19.713	−22.114	−30.418	1.00	27.95	O
ATOM	1456	CB	TYR	A	351	18.403	−22.623	−32.867	1.00	28.34	C
ATOM	1457	CG	TYR	A	351	19.048	−21.560	−33.742	1.00	28.15	C
ATOM	1458	CD1	TYR	A	351	18.568	−20.241	−33.675	1.00	28.01	C
ATOM	1459	CD2	TYR	A	351	19.979	−21.910	−34.744	1.00	28.14	C
ATOM	1460	CE1	TYR	A	351	18.960	−19.297	−34.639	1.00	27.86	C
ATOM	1461	CE2	TYR	A	351	20.447	−20.939	−35.652	1.00	27.98	C
ATOM	1462	CZ	TYR	A	351	19.924	−19.630	−35.609	1.00	27.84	C
ATOM	1463	OH	TYR	A	351	20.328	−18.683	−36.496	1.00	27.70	O
ATOM	1464	H	TYR	A	351	18.203	−25.107	−31.110	1.00	0.00	H

ATOM 1465 HH TYR A 351 19.667 −17.995 −36.600 1.00 0.00 H

ATOM 1466 N GLY A 352 20.890 −24.009 −30.358 1.00 61.59 N

ATOM 1467 CA GLY A 352 21.552 −23.673 −29.096 1.00 61.31 C

ATOM 1468 C GLY A 352 22.239 −24.860 −28.425 1.00 61.32 C

ATOM 1469 O GLY A 352 21.687 −25.952 −28.479 1.00 61.57 O

ATOM 1470 H GLY A 352 21.040 −24.929 −30.753 1.00 0.00 H

ATOM 1471 N PRO A 353 23.405 −24.702 −27.775 1.00 27.41 N

ATOM 1472 CA PRO A 353 24.228 −25.843 −27.368 1.00 27.40 C

ATOM 1473 C PRO A 353 23.640 −26.672 −26.211 1.00 27.59 C

ATOM 1474 O PRO A 353 23.677 −27.905 −26.267 1.00 27.81 O

ATOM 1475 CB PRO A 353 25.579 −25.225 −26.999 1.00 27.05 C

ATOM 1476 CG PRO A 353 25.225 −23.812 −26.536 1.00 26.92 C

ATOM 1477 CD PRO A 353 24.061 −23.444 −27.456 1.00 27.12 C

ATOM 1478 N SER A 354 23.019 −26.020 −25.222 1.00 27.51 N

ATOM 1479 CA SER A 354 22.569 −26.648 −23.974 1.00 27.64 C

ATOM 1480 C SER A 354 21.151 −26.224 −23.567 1.00 27.79 C

ATOM 1481 O SER A 354 20.751 −25.060 −23.686 1.00 27.72 O

ATOM 1482 CB SER A 354 23.597 −26.414 −22.855 1.00 27.36 C

ATOM 1483 OG SER A 354 23.932 −25.055 −22.653 1.00 27.06 O

ATOM 1484 H SER A 354 23.007 −25.012 −25.237 1.00 0.00 H

ATOM 1485 HG SER A 354 24.684 −25.048 −22.043 1.00 0.00 H

ATOM 1486 N GLY A 355 20.356 −27.239 −23.227 1.00 28.03 N

ATOM 1487 CA GLY A 355 18.976 −27.155 −22.758 1.00 28.21 C

ATOM 1488 C GLY A 355 18.775 −27.849 −21.408 1.00 28.15 C

ATOM 1489 O GLY A 355 19.743 −28.164 −20.716 1.00 27.85 O

ATOM 1490 H GLY A 355 20.767 −28.166 −23.214 1.00 0.00 H

ATOM 1491 N ILE A 356 17.518 −28.117 −21.034 1.00 28.45 N

ATOM 1492 CA ILE A 356 17.182 −28.738 −19.741 1.00 28.46 C
ATOM 1493 C ILE A 356 16.191 −29.893 −19.859 1.00 28.89 C
ATOM 1494 O ILE A 356 15.198 −29.823 −20.574 1.00 29.19 O
ATOM 1495 CB ILE A 356 16.704 −27.705 −18.693 1.00 28.26 C
ATOM 1496 CG1 ILE A 356 15.406 −26.972 −19.102 1.00 28.43 C
ATOM 1497 CG2 ILE A 356 17.847 −26.733 −18.368 1.00 27.82 C
ATOM 1498 CD1 ILE A 356 14.821 −26.095 −17.991 1.00 28.26 C
ATOM 1499 H ILE A 356 16.773 −27.945 −21.701 1.00 0.00 H
ATOM 1500 N LYS A 357 16.353 −30.862 −18.959 1.00 42.34 N
ATOM 1501 CA LYS A 357 15.305 −31.792 −18.525 1.00 42.77 C
ATOM 1502 C LYS A 357 14.466 −31.186 −17.398 1.00 42.88 C
ATOM 1503 O LYS A 357 15.002 −30.493 −16.528 1.00 42.60 O
ATOM 1504 CB LYS A 357 15.896 −33.169 −18.159 1.00 42.82 C
ATOM 1505 CG LYS A 357 16.745 −33.253 −16.876 1.00 42.57 C
ATOM 1506 CD LYS A 357 18.001 −32.366 −16.897 1.00 42.06 C
ATOM 1507 CE LYS A 357 19.039 −32.862 −15.886 1.00 41.78 C
ATOM 1508 NZ LYS A 357 20.409 −32.719 −16.420 1.00 41.37 N1+
ATOM 1509 H LYS A 357 17.180 −30.804 −18.389 1.00 0.00 H
ATOM 1510 HZ1 LYS A 357 21.110 −32.990 −15.734 1.00 0.00 H
ATOM 1511 HZ2 LYS A 357 20.655 −31.760 −16.687 1.00 0.00 H
ATOM 1512 HZ3 LYS A 357 20.513 −33.277 −17.262 1.00 0.00 H
ATOM 1513 N GLN A 358 13.197 −31.573 −17.347 1.00 29.91 N
ATOM 1514 CA GLN A 358 12.217 −31.262 −16.303 1.00 30.11 C
ATOM 1515 C GLN A 358 11.470 −32.549 −15.928 1.00 30.55 C
ATOM 1516 O GLN A 358 10.349 −32.794 −16.373 1.00 30.95 O
ATOM 1517 CB GLN A 358 11.275 −30.135 −16.765 1.00 30.25 C
ATOM 1518 CG GLN A 358 12.010 −28.797 −16.962 1.00 29.87 C

ATOM	1519	CD	GLN	A	358	11.070	−27.595	−17.027	1.00	30.05	C
ATOM	1520	OE1	GLN	A	358	9.859	−27.693	−17.077	1.00	30.45	O
ATOM	1521	NE2	GLN	A	358	11.590	−26.390	−16.974	1.00	29.77	N
ATOM	1522	H	GLN	A	358	12.863	−32.132	−18.128	1.00	0.00	H
ATOM	1523	1HE2	GLN	A	358	12.582	−26.256	−16.929	1.00	0.00	H
ATOM	1524	2HE2	GLN	A	358	10.924	−25.639	−17.010	1.00	0.00	H
ATOM	1525	N	GLY	A	359	12.221	−33.489	−15.345	1.00	30.49	N
ATOM	1526	CA	GLY	A	359	11.855	−34.906	−15.367	1.00	30.89	C
ATOM	1527	C	GLY	A	359	11.900	−35.470	−16.789	1.00	31.08	C
ATOM	1528	O	GLY	A	359	12.805	−35.150	−17.557	1.00	30.84	O
ATOM	1529	H	GLY	A	359	13.179	−33.262	−15.148	1.00	0.00	H
ATOM	1530	N	ASP	A	360	10.856	−36.211	−17.148	1.00	31.53	N
ATOM	1531	CA	ASP	A	360	10.718	−36.949	−18.411	1.00	31.77	C
ATOM	1532	C	ASP	A	360	10.544	−36.056	−19.668	1.00	31.71	C
ATOM	1533	O	ASP	A	360	10.648	−36.532	−20.800	1.00	32.07	O
ATOM	1534	CB	ASP	A	360	9.501	−37.875	−18.246	1.00	32.39	C
ATOM	1535	CG	ASP	A	360	9.617	−39.194	−19.004	1.00	32.55	C
ATOM	1536	OD1	ASP	A	360	10.722	−39.772	−19.039	1.00	32.33	O
ATOM	1537	OD2	ASP	A	360	8.542	−39.696	−19.402	1.00	32.94	O
ATOM	1538	H	ASP	A	360	10.151	−36.390	−16.452	1.00	0.00	H
ATOM	1539	N	THR	A	361	10.279	−34.751	−19.498	1.00	31.27	N
ATOM	1540	CA	THR	A	361	10.316	−33.761	−20.586	1.00	31.23	C
ATOM	1541	C	THR	A	361	11.706	−33.151	−20.735	1.00	30.74	C
ATOM	1542	O	THR	A	361	12.170	−32.457	−19.830	1.00	30.38	O
ATOM	1543	CB	THR	A	361	9.261	−32.660	−20.393	1.00	31.43	C
ATOM	1544	OG1	THR	A	361	7.967	−33.191	−20.554	1.00	31.83	O
ATOM	1545	CG2	THR	A	361	9.338	−31.580	−21.477	1.00	31.61	C

ATOM 1546 H THR A 361 10.264 −34.400 −18.550 1.00 0.00 H
ATOM 1547 HG1 THR A 361 7.968 −34.122 −20.262 1.00 0.00 H
ATOM 1548 N LEU A 362 12.286 −33.229 −21.937 1.00 30.73 N
ATOM 1549 CA LEU A 362 13.448 −32.433 −22.352 1.00 30.30 C
ATOM 1550 C LEU A 362 12.988 −31.128 −23.028 1.00 30.21 C
ATOM 1551 O LEU A 362 11.986 −31.129 −23.744 1.00 30.52 O
ATOM 1552 CB LEU A 362 14.363 −33.254 −23.286 1.00 30.34 C
ATOM 1553 CG LEU A 362 14.598 −34.727 −22.893 1.00 30.50 C
ATOM 1554 CD1 LEU A 362 15.581 −35.351 −23.882 1.00 30.55 C
ATOM 1555 CD2 LEU A 362 15.179 −34.896 −21.489 1.00 30.16 C
ATOM 1556 H LEU A 362 11.832 −33.794 −22.648 1.00 0.00 H
ATOM 1557 N TYR A 363 13.800 −30.073 −22.943 1.00 29.80 N
ATOM 1558 CA TYR A 363 13.611 −28.778 −23.601 1.00 29.69 C
ATOM 1559 C TYR A 363 14.925 −28.317 −24.240 1.00 29.35 C
ATOM 1560 O TYR A 363 15.799 −27.756 −23.572 1.00 29.03 O
ATOM 1561 CB TYR A 363 13.137 −27.738 −22.580 1.00 29.57 C
ATOM 1562 CG TYR A 363 11.701 −27.871 −22.127 1.00 29.95 C
ATOM 1563 CD1 TYR A 363 10.684 −27.299 −22.913 1.00 30.19 C
ATOM 1564 CD2 TYR A 363 11.393 −28.466 −20.889 1.00 30.09 C
ATOM 1565 CE1 TYR A 363 9.363 −27.265 −22.439 1.00 30.57 C
ATOM 1566 CE2 TYR A 363 10.066 −28.449 −20.419 1.00 30.48 C
ATOM 1567 CZ TYR A 363 9.057 −27.832 −21.187 1.00 30.72 C
ATOM 1568 OH TYR A 363 7.783 −27.790 −20.732 1.00 31.13 O
ATOM 1569 H TYR A 363 14.566 −30.126 −22.281 1.00 0.00 H
ATOM 1570 HH TYR A 363 7.313 −27.014 −21.130 1.00 0.00 H
ATOM 1571 N PHE A 364 15.043 −28.501 −25.554 1.00 29.44 N
ATOM 1572 CA PHE A 364 16.165 −28.004 −26.352 1.00 29.16 C

ATOM 1573 C PHE A 364 15.931 −26.532 −26.752 1.00 29.02 C

ATOM 1574 O PHE A 364 14.851 −26.237 −27.270 1.00 29.22 O

ATOM 1575 CB PHE A 364 16.309 −28.888 −27.601 1.00 29.37 C

ATOM 1576 CG PHE A 364 16.519 −30.364 −27.308 1.00 29.56 C

ATOM 1577 CD1 PHE A 364 17.792 −30.828 −26.930 1.00 29.38 C

ATOM 1578 CD2 PHE A 364 15.443 −31.271 −27.392 1.00 29.95 C

ATOM 1579 CE1 PHE A 364 17.994 −32.190 −26.644 1.00 29.58 C

ATOM 1580 CE2 PHE A 364 15.644 −32.634 −27.104 1.00 30.15 C

ATOM 1581 CZ PHE A 364 16.919 −33.092 −26.729 1.00 29.96 C

ATOM 1582 H PHE A 364 14.272 −28.939 −26.049 1.00 0.00 H

ATOM 1583 N PRO A 365 16.905 −25.607 −26.630 1.00 28.68 N

ATOM 1584 CA PRO A 365 16.816 −24.278 −27.247 1.00 28.60 C

ATOM 1585 C PRO A 365 16.688 −24.370 −28.776 1.00 28.76 C

ATOM 1586 O PRO A 365 17.375 −25.156 −29.433 1.00 28.80 O

ATOM 1587 CB PRO A 365 18.091 −23.538 −26.831 1.00 28.21 C

ATOM 1588 CG PRO A 365 19.075 −24.682 −26.613 1.00 28.10 C

ATOM 1589 CD PRO A 365 18.198 −25.757 −25.981 1.00 28.38 C

ATOM 1590 N ALA A 366 15.809 −23.551 −29.351 1.00 28.87 N

ATOM 1591 CA ALA A 366 15.298 −23.750 −30.705 1.00 29.07 C

ATOM 1592 C ALA A 366 14.928 −22.440 −31.419 1.00 29.01 C

ATOM 1593 O ALA A 366 14.850 −21.373 −30.809 1.00 28.83 O

ATOM 1594 CB ALA A 366 14.084 −24.678 −30.594 1.00 29.47 C

ATOM 1595 H ALA A 366 15.276 −22.924 −28.756 1.00 0.00 H

ATOM 1596 N VAL A 367 14.563 −22.559 −32.698 1.00 29.17 N

ATOM 1597 CA VAL A 367 14.020 −21.481 −33.529 1.00 29.11 C

ATOM 1598 C VAL A 367 12.843 −21.965 −34.376 1.00 29.41 C

ATOM 1599 O VAL A 367 12.872 −23.062 −34.933 1.00 29.61 O

ATOM 1600 CB VAL A 367 15.137 −20.835 −34.372 1.00 28.84 C

ATOM 1601 CG1 VAL A 367 15.714 −21.752 −35.456 1.00 28.96 C

ATOM 1602 CG2 VAL A 367 14.710 −19.527 −35.043 1.00 28.75 C

ATOM 1603 H VAL A 367 14.650 −23.477 −33.127 1.00 0.00 H

ATOM 1604 N GLY A 368 11.819 −21.123 −34.498 1.00 29.46 N

ATOM 1605 CA GLY A 368 10.718 −21.270 −35.444 1.00 29.75 C

ATOM 1606 C GLY A 368 10.811 −20.262 −36.583 1.00 29.61 C

ATOM 1607 O GLY A 368 11.202 −19.110 −36.382 1.00 29.36 O

ATOM 1608 H GLY A 368 11.868 −20.253 −33.980 1.00 0.00 H

ATOM 1609 N PHE A 369 10.417 −20.698 −37.778 1.00 29.79 N

ATOM 1610 CA PHE A 369 10.414 −19.909 −39.006 1.00 29.68 C

ATOM 1611 C PHE A 369 8.964 −19.639 −39.428 1.00 29.92 C

ATOM 1612 O PHE A 369 8.154 −20.566 −39.540 1.00 30.18 O

ATOM 1613 CB PHE A 369 11.165 −20.655 −40.116 1.00 29.68 C

ATOM 1614 CG PHE A 369 12.571 −21.156 −39.802 1.00 29.51 C

ATOM 1615 CD1 PHE A 369 13.682 −20.589 −40.451 1.00 29.32 C

ATOM 1616 CD2 PHE A 369 12.778 −22.253 −38.942 1.00 29.56 C

ATOM 1617 CE1 PHE A 369 14.973 −21.115 −40.256 1.00 29.18 C

ATOM 1618 CE2 PHE A 369 14.065 −22.768 −38.732 1.00 29.41 C

ATOM 1619 CZ PHE A 369 15.166 −22.208 −39.396 1.00 29.23 C

ATOM 1620 H PHE A 369 10.095 −21.659 −37.854 1.00 0.00 H

ATOM 1621 N LEU A 370 8.610 −18.370 −39.630 1.00 29.85 N

ATOM 1622 CA LEU A 370 7.235 −17.944 −39.910 1.00 30.06 C

ATOM 1623 C LEU A 370 7.226 −16.895 −41.035 1.00 29.90 C

ATOM 1624 O LEU A 370 7.999 −15.938 −40.952 1.00 29.61 O

ATOM 1625 CB LEU A 370 6.611 −17.465 −38.586 1.00 30.07 C

ATOM 1626 CG LEU A 370 5.220 −16.811 −38.688 1.00 30.32 C

ATOM 1627 CD1 LEU A 370 4.343 −17.228 −37.506 1.00 30.46 C
ATOM 1628 CD2 LEU A 370 5.334 −15.287 −38.657 1.00 30.17 C
ATOM 1629 H LEU A 370 9.313 −17.648 −39.493 1.00 0.00 H
ATOM 1630 N PRO A 371 6.494 −17.104 −42.148 1.00 30.08 N
ATOM 1631 CA PRO A 371 6.512 −16.161 −43.261 1.00 29.90 C
ATOM 1632 C PRO A 371 6.012 −14.780 −42.818 1.00 29.87 C
ATOM 1633 O PRO A 371 5.013 −14.667 −42.110 1.00 30.10 O
ATOM 1634 CB PRO A 371 5.670 −16.776 −44.382 1.00 30.11 C
ATOM 1635 CG PRO A 371 4.951 −17.976 −43.764 1.00 30.46 C
ATOM 1636 CD PRO A 371 5.685 −18.272 −42.457 1.00 30.45 C
ATOM 1637 N ARG A 372 6.701 −13.705 −43.220 1.00 39.74 N
ATOM 1638 CA ARG A 372 6.431 −12.338 −42.733 1.00 39.73 C
ATOM 1639 C ARG A 372 5.151 −11.691 −43.288 1.00 39.94 C
ATOM 1640 O ARG A 372 4.867 −10.549 −42.948 1.00 40.06 O
ATOM 1641 CB ARG A 372 7.687 −11.449 −42.828 1.00 39.44 C
ATOM 1642 CG ARG A 372 8.079 −10.972 −44.232 1.00 39.24 C
ATOM 1643 CD ARG A 372 9.132 −9.846 −44.200 1.00 39.00 C
ATOM 1644 NE ARG A 372 10.407 −10.198 −43.526 1.00 38.78 N
ATOM 1645 CZ ARG A 372 10.953 −9.577 −42.488 1.00 38.60 C
ATOM 1646 NH1 ARG A 372 10.243 −8.821 −41.698 1.00 38.61 N
ATOM 1647 NH2 ARG A 372 12.215 −9.714 −42.203 1.00 38.43 N
ATOM 1648 H ARG A 372 7.532 −13.866 −43.783 1.00 0.00 H
ATOM 1649 HE ARG A 372 11.009 −10.840 −44.030 1.00 0.00 H
ATOM 1650 1HH1 ARG A 372 9.255 −8.798 −41.836 1.00 0.00 H
ATOM 1651 2HH1 ARG A 372 10.639 −8.449 −40.840 1.00 0.00 H
ATOM 1652 1HH2 ARG A 372 12.810 −10.224 −42.849 1.00 0.00 H
ATOM 1653 2HH2 ARG A 372 12.662 −9.093 −41.538 1.00 0.00 H

ATOM 1654 N THR A 373 4.352 −12.439 −44.049 1.00 29.86 N

ATOM 1655 CA THR A 373 2.921 −12.186 −44.301 1.00 30.10 C

ATOM 1656 C THR A 373 2.055 −12.459 −43.066 1.00 30.40 C

ATOM 1657 O THR A 373 1.123 −11.712 −42.791 1.00 30.61 O

ATOM 1658 CB THR A 373 2.430 −13.098 −45.440 1.00 30.20 C

ATOM 1659 OG1 THR A 373 2.840 −14.431 −45.220 1.00 30.34 O

ATOM 1660 CG2 THR A 373 3.014 −12.677 −46.788 1.00 29.92 C

ATOM 1661 H THR A 373 4.659 −13.378 −44.257 1.00 0.00 H

ATOM 1662 HG1 THR A 373 2.075 −14.915 −44.812 1.00 0.00 H

ATOM 1663 N GLU A 374 2.429 −13.447 −42.251 1.00 30.43 N

ATOM 1664 CA GLU A 374 1.701 −13.846 −41.040 1.00 30.71 C

ATOM 1665 C GLU A 374 2.125 −13.032 −39.808 1.00 30.59 C

ATOM 1666 O GLU A 374 1.435 −13.051 −38.788 1.00 30.80 O

ATOM 1667 CB GLU A 374 1.941 −15.341 −40.749 1.00 30.92 C

ATOM 1668 CG GLU A 374 1.946 −16.266 −41.971 1.00 31.03 C

ATOM 1669 CD GLU A 374 0.715 −16.072 −42.853 1.00 31.30 C

ATOM 1670 OE1 GLU A 374 −0.327 −16.638 −42.450 1.00 31.63 O

ATOM 1671 OE2 GLU A 374 0.905 −15.677 −44.023 1.00 31.19 O

ATOM 1672 H GLU A 374 3.217 −14.023 −42.528 1.00 0.00 H

ATOM 1673 N PHE A 375 3.310 −12.413 −39.854 1.00 30.27 N

ATOM 1674 CA PHE A 375 3.981 −11.826 −38.695 1.00 30.14 C

ATOM 1675 C PHE A 375 3.301 −10.538 −38.208 1.00 30.19 C

ATOM 1676 O PHE A 375 3.645 −9.430 −38.620 1.00 30.11 O

ATOM 1677 CB PHE A 375 5.469 −11.610 −39.009 1.00 29.79 C

ATOM 1678 CG PHE A 375 6.268 −11.037 −37.850 1.00 29.62 C

ATOM 1679 CD1 PHE A 375 6.228 −11.653 −36.582 1.00 29.76 C

ATOM 1680 CD2 PHE A 375 7.054 −9.884 −38.036 1.00 29.34 C

ATOM	1681	CE1	PHE	A	375	6.949	−11.107	−35.506	1.00	29.61	C
ATOM	1682	CE2	PHE	A	375	7.789	−9.350	−36.964	1.00	29.19	C
ATOM	1683	CZ	PHE	A	375	7.728	−9.954	−35.696	1.00	29.33	C
ATOM	1684	H	PHE	A	375	3.806	−12.439	−40.731	1.00	0.00	H
ATOM	1685	N	GLN	A	376	2.383	−10.694	−37.255	1.00	30.34	N
ATOM	1686	CA	GLN	A	376	1.738	−9.579	−36.566	1.00	30.47	C
ATOM	1687	C	GLN	A	376	2.737	−8.891	−35.624	1.00	30.27	C
ATOM	1688	O	GLN	A	376	3.091	−9.432	−34.578	1.00	30.24	O
ATOM	1689	CB	GLN	A	376	0.488	−10.068	−35.805	1.00	30.84	C
ATOM	1690	CG	GLN	A	376	−0.487	−10.906	−36.652	1.00	31.14	C
ATOM	1691	CD	GLN	A	376	−0.809	−10.270	−38.001	1.00	31.05	C
ATOM	1692	OE1	GLN	A	376	−1.278	−9.150	−38.091	1.00	31.11	O
ATOM	1693	NE2	GLN	A	376	−0.492	−10.926	−39.094	1.00	30.92	N
ATOM	1694	H	GLN	A	376	2.120	−11.633	−36.996	1.00	0.00	H
ATOM	1695	1HE2	GLN	A	376	0.033	−11.789	−39.024	1.00	0.00	H
ATOM	1696	2HE2	GLN	A	376	−0.625	−10.447	−39.966	1.00	0.00	H
ATOM	1697	N	TYR	A	377	3.134	−7.664	−35.962	1.00	50.03	N
ATOM	1698	CA	TYR	A	377	3.960	−6.806	−35.115	1.00	49.87	C
ATOM	1699	C	TYR	A	377	3.527	−5.347	−35.266	1.00	49.97	C
ATOM	1700	O	TYR	A	377	3.466	−4.825	−36.378	1.00	49.92	O
ATOM	1701	CB	TYR	A	377	5.446	−6.992	−35.455	1.00	49.53	C
ATOM	1702	CG	TYR	A	377	6.376	−6.200	−34.551	1.00	49.40	C
ATOM	1703	CD1	TYR	A	377	6.861	−6.783	−33.362	1.00	49.50	C
ATOM	1704	CD2	TYR	A	377	6.705	−4.865	−34.862	1.00	49.20	C
ATOM	1705	CE1	TYR	A	377	7.661	−6.029	−32.481	1.00	49.40	C
ATOM	1706	CE2	TYR	A	377	7.506	−4.112	−33.983	1.00	49.12	C
ATOM	1707	CZ	TYR	A	377	7.970	−4.687	−32.783	1.00	49.21	C

ATOM	1708	OH	TYR	A	377	8.698	−3.943	−31.912	1.00	49.15	O
ATOM	1709	H	TYR	A	377	2.846	−7.297	−36.857	1.00	0.00	H
ATOM	1710	HH	TYR	A	377	8.548	−3.005	−32.036	1.00	0.00	H
ATOM	1711	N	ASN	A	378	3.233	−4.682	−34.148	1.00	34.98	N
ATOM	1712	CA	ASN	A	378	2.982	−3.245	−34.127	1.00	35.08	C
ATOM	1713	C	ASN	A	378	4.315	−2.486	−34.127	1.00	34.82	C
ATOM	1714	O	ASN	A	378	4.971	−2.394	−33.090	1.00	34.72	O
ATOM	1715	CB	ASN	A	378	2.116	−2.907	−32.902	1.00	35.37	C
ATOM	1716	CG	ASN	A	378	1.811	−1.422	−32.770	1.00	35.45	C
ATOM	1717	OD1	ASN	A	378	1.848	−0.646	−33.715	1.00	35.27	O
ATOM	1718	ND2	ASN	A	378	1.605	−0.944	−31.570	1.00	35.74	N
ATOM	1719	H	ASN	A	378	3.358	−5.150	−33.265	1.00	0.00	H
ATOM	1720	1HD2	ASN	A	378	1.918	−1.486	−30.768	1.00	0.00	H
ATOM	1721	2HD2	ASN	A	378	1.418	0.036	−31.482	1.00	0.00	H
ATOM	1722	N	ASP	A	379	4.616	−1.790	−35.223	1.00	29.95	N
ATOM	1723	CA	ASP	A	379	5.781	−0.901	−35.331	1.00	29.73	C
ATOM	1724	C	ASP	A	379	5.823	0.182	−34.233	1.00	29.83	C
ATOM	1725	O	ASP	A	379	6.901	0.489	−33.731	1.00	29.65	O
ATOM	1726	CB	ASP	A	379	5.811	−0.257	−36.722	1.00	29.67	C
ATOM	1727	CG	ASP	A	379	6.158	−1.207	−37.860	1.00	29.51	C
ATOM	1728	OD1	ASP	A	379	6.438	−2.407	−37.646	1.00	29.27	O
ATOM	1729	OD2	ASP	A	379	6.246	−0.712	−39.002	1.00	29.66	O
ATOM	1730	H	ASP	A	379	4.087	−1.975	−36.063	1.00	0.00	H
ATOM	1731	N	SER	A	380	4.669	0.594	−33.691	1.00	30.51	N
ATOM	1732	CA	SER	A	380	4.578	1.555	−32.574	1.00	30.65	C
ATOM	1733	C	SER	A	380	5.143	1.023	−31.244	1.00	30.54	C
ATOM	1734	O	SER	A	380	5.414	1.814	−30.345	1.00	30.55	O

ATOM 1735 CB SER A 380 3.135 2.033 −32.336 1.00 31.03 C
ATOM 1736 OG SER A 380 2.353 2.057 −33.518 1.00 31.14 O
ATOM 1737 H SER A 380 3.809 0.241 −34.086 1.00 0.00 H
ATOM 1738 HG SER A 380 2.073 1.147 −33.726 1.00 0.00 H
ATOM 1739 N ASN A 381 5.360 −0.294 −31.109 1.00 30.08 N
ATOM 1740 CA ASN A 381 6.080 −0.889 −29.976 1.00 29.95 C
ATOM 1741 C ASN A 381 7.609 −0.696 −30.073 1.00 29.61 C
ATOM 1742 O ASN A 381 8.325 −1.043 −29.135 1.00 29.48 O
ATOM 1743 CB ASN A 381 5.755 −2.398 −29.865 1.00 29.99 C
ATOM 1744 CG ASN A 381 4.333 −2.748 −29.452 1.00 30.34 C
ATOM 1745 OD1 ASN A 381 3.362 −2.050 −29.694 1.00 30.56 O
ATOM 1746 ND2 ASN A 381 4.150 −3.883 −28.818 1.00 30.43 N
ATOM 1747 H ASN A 381 5.129 −0.897 −31.891 1.00 0.00 H
ATOM 1748 1HD2 ASN A 381 4.931 −4.471 −28.584 1.00 0.00 H
ATOM 1749 2HD2 ASN A 381 3.217 −4.090 −28.513 1.00 0.00 H
ATOM 1750 N CYS A 382 8.142 −0.330 −31.245 1.00 29.47 N
ATOM 1751 CA CYS A 382 9.580 −0.286 −31.496 1.00 29.18 C
ATOM 1752 C CYS A 382 10.253 0.877 −30.719 1.00 29.19 C
ATOM 1753 O CYS A 382 9.856 2.022 −30.939 1.00 29.32 O
ATOM 1754 CB CYS A 382 9.796 −0.203 −33.013 1.00 29.06 C
ATOM 1755 SG CYS A 382 11.476 −0.555 −33.575 1.00 28.72 S
ATOM 1756 H CYS A 382 7.524 −0.020 −31.989 1.00 0.00 H
ATOM 1757 N PRO A 383 11.278 0.652 −29.859 1.00 54.27 N
ATOM 1758 CA PRO A 383 11.807 1.655 −28.903 1.00 54.34 C
ATOM 1759 C PRO A 383 12.537 2.911 −29.443 1.00 54.19 C
ATOM 1760 O PRO A 383 13.381 3.477 −28.751 1.00 53.96 O
ATOM 1761 CB PRO A 383 12.718 0.874 −27.944 1.00 54.27 C

ATOM 1762 CG PRO A 383 12.134 −0.530 −27.965 1.00 54.10 C

ATOM 1763 CD PRO A 383 11.715 −0.676 −29.422 1.00 54.07 C

ATOM 1764 N ILE A 384 12.220 3.398 −30.642 1.00 33.61 N

ATOM 1765 CA ILE A 384 12.958 4.432 −31.394 1.00 33.52 C

ATOM 1766 C ILE A 384 12.998 5.847 −30.774 1.00 33.74 C

ATOM 1767 O ILE A 384 13.517 6.759 −31.407 1.00 33.73 O

ATOM 1768 CB ILE A 384 12.403 4.515 −32.836 1.00 33.48 C

ATOM 1769 CG1 ILE A 384 10.954 5.064 −32.856 1.00 33.76 C

ATOM 1770 CG2 ILE A 384 12.518 3.153 −33.544 1.00 33.33 C

ATOM 1771 CD1 ILE A 384 10.458 5.442 −34.252 1.00 33.73 C

ATOM 1772 H ILE A 384 11.435 2.960 −31.113 1.00 0.00 H

ATOM 1773 HA ILE A 384 13.996 4.105 −31.457 1.00 0.00 H

ATOM 1774 N ILE A 385 12.392 6.090 −29.609 1.00 41.53 N

ATOM 1775 CA ILE A 385 12.001 7.441 −29.145 1.00 41.81 C

ATOM 1776 C ILE A 385 13.188 8.421 −29.027 1.00 41.76 C

ATOM 1777 O ILE A 385 13.058 9.583 −29.402 1.00 41.95 O

ATOM 1778 CB ILE A 385 11.196 7.326 −27.824 1.00 42.09 C

ATOM 1779 CG1 ILE A 385 9.871 6.565 −28.089 1.00 42.20 C

ATOM 1780 CG2 ILE A 385 10.911 8.706 −27.198 1.00 42.42 C

ATOM 1781 CD1 ILE A 385 9.036 6.268 −26.836 1.00 42.55 C

ATOM 1782 H ILE A 385 12.039 5.288 −29.107 1.00 0.00 H

ATOM 1783 N HIS A 386 14.368 7.941 −28.625 1.00 29.46 N

ATOM 1784 CA HIS A 386 15.611 8.740 −28.571 1.00 29.42 C

ATOM 1785 C HIS A 386 16.465 8.635 −29.851 1.00 29.17 C

ATOM 1786 O HIS A 386 17.497 9.294 −29.994 1.00 29.07 O

ATOM 1787 CB HIS A 386 16.415 8.312 −27.334 1.00 29.35 C

ATOM 1788 CG HIS A 386 15.611 8.358 −26.058 1.00 29.60 C

ATOM 1789 ND1 HIS A 386 15.133 9.520 −25.451 1.00 29.94 N

ATOM 1790 CD2 HIS A 386 15.178 7.278 −25.344 1.00 29.60 C

ATOM 1791 CE1 HIS A 386 14.431 9.118 −24.383 1.00 30.13 C

ATOM 1792 NE2 HIS A 386 14.440 7.776 −24.292 1.00 29.92 N

ATOM 1793 H HIS A 386 14.411 6.969 −28.362 1.00 0.00 H

ATOM 1794 HE2 HIS A 386 13.986 7.236 −23.570 1.00 0.00 H

ATOM 1795 N CYS A 387 16.106 7.719 −30.746 1.00 35.54 N

ATOM 1796 CA CYS A 387 16.943 7.215 −31.827 1.00 35.30 C

ATOM 1797 C CYS A 387 16.730 8.024 −33.109 1.00 35.39 C

ATOM 1798 O CYS A 387 15.987 7.623 −33.998 1.00 35.38 O

ATOM 1799 CB CYS A 387 16.640 5.727 −31.995 1.00 35.10 C

ATOM 1800 SG CYS A 387 16.989 4.785 −30.493 1.00 34.90 S

ATOM 1801 H CYS A 387 15.189 7.300 −30.650 1.00 0.00 H

ATOM 1802 N LYS A 388 17.468 9.137 −33.222 1.00 29.04 N

ATOM 1803 CA LYS A 388 17.288 10.247 −34.189 1.00 29.18 C

ATOM 1804 C LYS A 388 17.003 9.892 −35.668 1.00 29.02 C

ATOM 1805 O LYS A 388 16.627 10.788 −36.418 1.00 29.16 O

ATOM 1806 CB LYS A 388 18.555 11.125 −34.131 1.00 29.19 C

ATOM 1807 CG LYS A 388 18.271 12.622 −34.345 1.00 29.50 C

ATOM 1808 CD LYS A 388 19.472 13.439 −34.853 1.00 29.52 C

ATOM 1809 CE LYS A 388 20.773 13.279 −34.049 1.00 29.35 C

ATOM 1810 NZ LYS A 388 21.578 12.114 −34.500 1.00 29.01 N1+

ATOM 1811 H LYS A 388 17.979 9.359 −32.376 1.00 0.00 H

ATOM 1812 HZ1 LYS A 388 22.433 12.032 −33.967 1.00 0.00 H

ATOM 1813 HZ2 LYS A 388 21.825 12.216 −35.483 1.00 0.00 H

ATOM 1814 HZ3 LYS A 388 21.054 11.254 −34.407 1.00 0.00 H

ATOM 1815 N TYR A 389 17.363 8.693 −36.127 1.00 28.75 N

ATOM	1816	CA	TYR	A	389	17.205	8.242	−37.520	1.00	28.60	C
ATOM	1817	C	TYR	A	389	16.382	6.947	−37.671	1.00	28.49	C
ATOM	1818	O	TYR	A	389	16.008	6.594	−38.789	1.00	28.40	O
ATOM	1819	CB	TYR	A	389	18.598	8.056	−38.151	1.00	28.39	C
ATOM	1820	CG	TYR	A	389	19.576	9.209	−37.973	1.00	28.48	C
ATOM	1821	CD1	TYR	A	389	19.188	10.530	−38.273	1.00	28.48	C
ATOM	1822	CD2	TYR	A	389	20.883	8.952	−37.513	1.00	28.60	C
ATOM	1823	CE1	TYR	A	389	20.093	11.594	−38.093	1.00	28.59	C
ATOM	1824	CE2	TYR	A	389	21.793	10.011	−37.332	1.00	28.72	C
ATOM	1825	CZ	TYR	A	389	21.396	11.331	−37.624	1.00	28.72	C
ATOM	1826	OH	TYR	A	389	22.249	12.355	−37.363	1.00	28.86	O
ATOM	1827	H	TYR	A	389	17.625	8.005	−35.440	1.00	0.00	H
ATOM	1828	HH	TYR	A	389	22.741	12.588	−38.160	1.00	0.00	H
ATOM	1829	N	SER	A	390	16.131	6.226	−36.576	1.00	28.50	N
ATOM	1830	CA	SER	A	390	15.590	4.867	−36.597	1.00	28.40	C
ATOM	1831	C	SER	A	390	14.095	4.848	−36.898	1.00	28.59	C
ATOM	1832	O	SER	A	390	13.304	5.553	−36.270	1.00	28.81	O
ATOM	1833	CB	SER	A	390	15.873	4.168	−35.268	1.00	28.34	C
ATOM	1834	OG	SER	A	390	17.270	4.118	−35.053	1.00	28.20	O
ATOM	1835	H	SER	A	390	16.345	6.610	−35.666	1.00	0.00	H
ATOM	1836	HG	SER	A	390	17.654	3.627	−35.826	1.00	0.00	H
ATOM	1837	N	LYS	A	391	13.677	3.990	−37.832	1.00	28.50	N
ATOM	1838	CA	LYS	A	391	12.264	3.831	−38.198	1.00	28.66	C
ATOM	1839	C	LYS	A	391	11.568	2.893	−37.217	1.00	28.73	C
ATOM	1840	O	LYS	A	391	12.111	1.845	−36.883	1.00	28.63	O
ATOM	1841	CB	LYS	A	391	12.112	3.321	−39.640	1.00	28.55	C
ATOM	1842	CG	LYS	A	391	12.939	4.121	−40.663	1.00	28.49	C

ATOM	1843	CD	LYS	A	391	12.654	3.665	−42.099	1.00	28.44	C
ATOM	1844	CE	LYS	A	391	11.297	4.189	−42.584	1.00	28.65	C
ATOM	1845	NZ	LYS	A	391	10.905	3.574	−43.873	1.00	28.62	N1+
ATOM	1846	H	LYS	A	391	14.360	3.343	−38.214	1.00	0.00	H
ATOM	1847	HZ1	LYS	A	391	10.055	3.980	−44.234	1.00	0.00	H
ATOM	1848	HZ2	LYS	A	391	11.641	3.610	−44.559	1.00	0.00	H
ATOM	1849	HZ3	LYS	A	391	10.650	2.590	−43.716	1.00	0.00	H
ATOM	1850	N	ALA	A	392	10.265	3.094	−37.036	1.00	38.67	N
ATOM	1851	CA	ALA	A	392	9.406	2.165	−36.294	1.00	38.80	C
ATOM	1852	C	ALA	A	392	9.332	0.763	−36.949	1.00	38.67	C
ATOM	1853	O	ALA	A	392	9.303	−0.252	−36.256	1.00	38.68	O
ATOM	1854	CB	ALA	A	392	8.033	2.837	−36.170	1.00	39.08	C
ATOM	1855	H	ALA	A	392	9.870	3.969	−37.334	1.00	0.00	H
ATOM	1856	N	GLU	A	393	9.560	0.701	−38.267	1.00	32.27	N
ATOM	1857	CA	GLU	A	393	9.720	−0.543	−39.029	1.00	32.19	C
ATOM	1858	C	GLU	A	393	10.927	−1.393	−38.601	1.00	31.99	C
ATOM	1859	O	GLU	A	393	10.941	−2.596	−38.855	1.00	31.95	O
ATOM	1860	CB	GLU	A	393	9.937	−0.226	−40.516	1.00	32.12	C
ATOM	1861	CG	GLU	A	393	8.928	0.727	−41.165	1.00	32.18	C
ATOM	1862	CD	GLU	A	393	8.927	0.524	−42.685	1.00	31.98	C
ATOM	1863	OE1	GLU	A	393	8.488	−0.569	−43.125	1.00	31.89	O
ATOM	1864	OE2	GLU	A	393	9.553	1.363	−43.371	1.00	31.92	O
ATOM	1865	H	GLU	A	393	9.592	1.566	−38.776	1.00	0.00	H
ATOM	1866	N	ASN	A	394	11.999	−0.778	−38.087	1.00	32.64	N
ATOM	1867	CA	ASN	A	394	13.317	−1.412	−37.982	1.00	32.44	C
ATOM	1868	C	ASN	A	394	13.295	−2.685	−37.126	1.00	32.45	C
ATOM	1869	O	ASN	A	394	13.847	−3.702	−37.547	1.00	32.34	O

ATOM	1870	CB	ASN	A	394	14.334	−0.401	−37.431	1.00	32.41	C
ATOM	1871	CG	ASN	A	394	14.736	0.694	−38.408	1.00	32.39	C
ATOM	1872	OD1	ASN	A	394	14.377	0.694	−39.577	1.00	32.47	O
ATOM	1873	ND2	ASN	A	394	15.578	1.597	−37.971	1.00	32.30	N
ATOM	1874	H	ASN	A	394	11.936	0.209	−37.866	1.00	0.00	H
ATOM	1875	1HD2	ASN	A	394	15.926	1.499	−37.018	1.00	0.00	H
ATOM	1876	2HD2	ASN	A	394	16.283	1.933	−38.623	1.00	0.00	H
ATOM	1877	N	CYS	A	395	12.455	−2.704	−36.084	1.00	31.44	N
ATOM	1878	CA	CYS	A	395	12.134	−3.904	−35.318	1.00	31.44	C
ATOM	1879	C	CYS	A	395	11.551	−5.013	−36.212	1.00	31.49	C
ATOM	1880	O	CYS	A	395	12.182	−6.054	−36.387	1.00	31.38	O
ATOM	1881	CB	CYS	A	395	11.164	−3.548	−34.182	1.00	31.64	C
ATOM	1882	SG	CYS	A	395	11.826	−2.451	−32.904	1.00	31.62	S
ATOM	1883	H	CYS	A	395	11.987	−1.841	−35.843	1.00	0.00	H
ATOM	1884	N	ARG	A	396	10.420	−4.751	−36.882	1.00	28.58	N
ATOM	1885	CA	ARG	A	396	9.716	−5.702	−37.763	1.00	28.67	C
ATOM	1886	C	ARG	A	396	10.585	−6.213	−38.915	1.00	28.50	C
ATOM	1887	O	ARG	A	396	10.500	−7.387	−39.284	1.00	28.50	O
ATOM	1888	CB	ARG	A	396	8.444	−5.004	−38.281	1.00	28.89	C
ATOM	1889	CG	ARG	A	396	7.640	−5.814	−39.316	1.00	29.00	C
ATOM	1890	CD	ARG	A	396	6.301	−5.154	−39.681	1.00	29.22	C
ATOM	1891	NE	ARG	A	396	6.446	−3.741	−40.065	1.00	29.17	N
ATOM	1892	CZ	ARG	A	396	6.895	−3.205	−41.179	1.00	29.02	C
ATOM	1893	NH1	ARG	A	396	7.261	−3.903	−42.212	1.00	28.90	N
ATOM	1894	NH2	ARG	A	396	6.977	−1.924	−41.281	1.00	29.01	N
ATOM	1895	H	ARG	A	396	10.031	−3.820	−36.798	1.00	0.00	H
ATOM	1896	HE	ARG	A	396	6.292	−3.071	−39.304	1.00	0.00	H

ATOM 1897 1HH1 ARG A 396 7.090 −4.884 −42.203 1.00 0.00 H

ATOM 1898 2HH1 ARG A 396 7.563 −3.401 −43.027 1.00 0.00 H

ATOM 1899 1HH2 ARG A 396 6.688 −1.368 −40.471 1.00 0.00 H

ATOM 1900 2HH2 ARG A 396 7.436 −1.455 −42.061 1.00 0.00 H

ATOM 1901 N LEU A 397 11.412 −5.347 −39.495 1.00 28.38 N

ATOM 1902 CA LEU A 397 12.327 −5.696 −40.581 1.00 28.20 C

ATOM 1903 C LEU A 397 13.477 −6.595 −40.095 1.00 28.06 C

ATOM 1904 O LEU A 397 13.667 −7.663 −40.681 1.00 28.01 O

ATOM 1905 CB LEU A 397 12.843 −4.406 −41.245 1.00 28.09 C

ATOM 1906 CG LEU A 397 11.758 −3.560 −41.946 1.00 28.20 C

ATOM 1907 CD1 LEU A 397 12.386 −2.273 −42.480 1.00 28.15 C

ATOM 1908 CD2 LEU A 397 11.077 −4.288 −43.108 1.00 28.17 C

ATOM 1909 H LEU A 397 11.393 −4.387 −39.158 1.00 0.00 H

ATOM 1910 N SER A 398 14.107 −6.260 −38.960 1.00 29.91 N

ATOM 1911 CA SER A 398 15.251 −6.986 −38.378 1.00 29.76 C

ATOM 1912 C SER A 398 14.941 −8.426 −37.954 1.00 29.83 C

ATOM 1913 O SER A 398 15.838 −9.256 −38.020 1.00 29.71 O

ATOM 1914 CB SER A 398 15.818 −6.197 −37.185 1.00 29.68 C

ATOM 1915 OG SER A 398 16.835 −6.905 −36.489 1.00 29.55 O

ATOM 1916 H SER A 398 13.827 −5.400 −38.494 1.00 0.00 H

ATOM 1917 HG SER A 398 17.565 −7.113 −37.107 1.00 0.00 H

ATOM 1918 N MET A 399 13.676 −8.795 −37.707 1.00 28.13 N

ATOM 1919 CA MET A 399 13.304 −10.161 −37.282 1.00 28.23 C

ATOM 1920 C MET A 399 13.583 −11.284 −38.308 1.00 28.22 C

ATOM 1921 O MET A 399 13.324 −12.448 −38.010 1.00 28.30 O

ATOM 1922 CB MET A 399 11.833 −10.229 −36.831 1.00 28.49 C

ATOM 1923 CG MET A 399 11.420 −9.198 −35.781 1.00 28.56 C

ATOM 1924 SD MET A 399 12.495 −9.055 −34.327 1.00 28.44 S

ATOM 1925 CE MET A 399 11.635 −7.723 −33.449 1.00 28.56 C

ATOM 1926 H MET A 399 12.984 −8.061 −37.632 1.00 0.00 H

ATOM 1927 N GLY A 400 14.090 −10.986 −39.509 1.00 28.13 N

ATOM 1928 CA GLY A 400 14.535 −11.989 −40.482 1.00 28.11 C

ATOM 1929 C GLY A 400 15.872 −11.627 −41.134 1.00 27.89 C

ATOM 1930 O GLY A 400 16.190 −10.452 −41.288 1.00 27.75 O

ATOM 1931 H GLY A 400 14.437 −10.043 −39.637 1.00 0.00 H

ATOM 1932 N VAL A 401 16.570 −12.642 −41.660 1.00 27.88 N

ATOM 1933 CA VAL A 401 17.931 −12.569 −42.254 1.00 27.71 C

ATOM 1934 C VAL A 401 18.082 −11.520 −43.373 1.00 27.63 C

ATOM 1935 O VAL A 401 19.172 −11.009 −43.615 1.00 27.49 O

ATOM 1936 CB VAL A 401 18.325 −13.981 −42.755 1.00 27.78 C

ATOM 1937 CG1 VAL A 401 19.585 −14.037 −43.628 1.00 27.64 C

ATOM 1938 CG2 VAL A 401 18.532 −14.928 −41.566 1.00 27.85 C

ATOM 1939 H VAL A 401 16.219 −13.569 −41.473 1.00 0.00 H

ATOM 1940 N ASN A 402 16.979 −11.096 −43.993 1.00 27.75 N

ATOM 1941 CA ASN A 402 16.870 −9.793 −44.649 1.00 27.71 C

ATOM 1942 C ASN A 402 15.631 −9.063 −44.100 1.00 27.82 C

ATOM 1943 O ASN A 402 14.590 −9.697 −43.925 1.00 27.97 O

ATOM 1944 CB ASN A 402 16.735 −9.944 −46.179 1.00 27.78 C

ATOM 1945 CG ASN A 402 17.873 −10.554 −46.982 1.00 27.74 C

ATOM 1946 OD1 ASN A 402 17.722 −10.733 −48.179 1.00 27.60 O

ATOM 1947 ND2 ASN A 402 19.032 −10.814 −46.430 1.00 27.89 N

ATOM 1948 H ASN A 402 16.115 −11.462 −43.623 1.00 0.00 H

ATOM 1949 HB3 ASN A 402 15.851 −10.539 −46.392 1.00 0.00 H

ATOM 1950 1HD2 ASN A 402 19.178 −10.720 −45.428 1.00 0.00 H

ATOM 1951 2HD2 ASN A 402 19.747 −11.200 −47.019 1.00 0.00 H

ATOM 1952 N SER A 403 15.602 −7.739 −44.269 1.00 27.76 N

ATOM 1953 CA SER A 403 14.370 −6.929 −44.220 1.00 27.88 C

ATOM 1954 C SER A 403 13.289 −7.416 −45.202 1.00 28.04 C

ATOM 1955 O SER A 403 12.124 −7.542 −44.833 1.00 28.19 O

ATOM 1956 CB SER A 403 14.743 −5.479 −44.546 1.00 27.82 C

ATOM 1957 OG SER A 403 15.415 −5.410 −45.795 1.00 27.78 O

ATOM 1958 H SER A 403 16.455 −7.263 −44.524 1.00 0.00 H

ATOM 1959 HG SER A 403 15.723 −4.504 −45.923 1.00 0.00 H

ATOM 1960 N LYS A 404 13.710 −7.847 −46.400 1.00 28.00 N

ATOM 1961 CA LYS A 404 12.878 −8.440 −47.465 1.00 28.14 C

ATOM 1962 C LYS A 404 12.785 −9.978 −47.448 1.00 28.29 C

ATOM 1963 O LYS A 404 12.532 −10.576 −48.493 1.00 28.45 O

ATOM 1964 CB LYS A 404 13.310 −7.858 −48.829 1.00 28.05 C

ATOM 1965 CG LYS A 404 14.747 −8.213 −49.280 1.00 27.95 C

ATOM 1966 CD LYS A 404 14.869 −9.300 −50.361 1.00 28.03 C

ATOM 1967 CE LYS A 404 14.234 −8.861 −51.688 1.00 27.98 C

ATOM 1968 NZ LYS A 404 14.296 −9.943 −52.701 1.00 27.94 N1+

ATOM 1969 H LYS A 404 14.678 −7.649 −46.607 1.00 0.00 H

ATOM 1970 HZ1 LYS A 404 13.897 −9.633 −53.578 1.00 0.00 H

ATOM 1971 HZ2 LYS A 404 13.776 −10.749 −52.377 1.00 0.00 H

ATOM 1972 HZ3 LYS A 404 15.256 −10.223 −52.859 1.00 0.00 H

ATOM 1973 N SER A 405 13.146 −10.635 −46.342 1.00 28.23 N

ATOM 1974 CA SER A 405 13.092 −12.102 −46.225 1.00 28.36 C

ATOM 1975 C SER A 405 11.665 −12.640 −46.380 1.00 28.62 C

ATOM 1976 O SER A 405 10.704 −11.955 −46.041 1.00 28.71 O

ATOM 1977 CB SER A 405 13.662 −12.563 −44.878 1.00 28.30 C

ATOM 1978 OG SER A 405 12.888 −12.063 −43.806 1.00 28.39 O

ATOM 1979 H SER A 405 13.349 −10.104 −45.504 1.00 0.00 H

ATOM 1980 HG SER A 405 12.640 −12.825 −43.233 1.00 0.00 H

ATOM 1981 N HIS A 406 11.546 −13.915 −46.762 1.00 28.77 N

ATOM 1982 CA HIS A 406 10.296 −14.679 −46.657 1.00 29.05 C

ATOM 1983 C HIS A 406 9.943 −14.948 −45.184 1.00 29.17 C

ATOM 1984 O HIS A 406 9.006 −14.345 −44.672 1.00 29.36 O

ATOM 1985 CB HIS A 406 10.432 −15.959 −47.501 1.00 29.22 C

ATOM 1986 CG HIS A 406 9.279 −16.924 −47.405 1.00 29.54 C

ATOM 1987 ND1 HIS A 406 8.969 −17.668 −46.272 1.00 29.74 N

ATOM 1988 CD2 HIS A 406 8.496 −17.350 −48.439 1.00 29.72 C

ATOM 1989 CE1 HIS A 406 8.020 −18.540 −46.644 1.00 30.04 C

ATOM 1990 NE2 HIS A 406 7.702 −18.357 −47.935 1.00 30.03 N

ATOM 1991 H HIS A 406 12.379 −14.419 −47.019 1.00 0.00 H

ATOM 1992 HE2 HIS A 406 7.050 −18.931 −48.453 1.00 0.00 H

ATOM 1993 N TYR A 407 10.840 −15.602 −44.441 1.00 29.08 N

ATOM 1994 CA TYR A 407 10.692 −15.844 −43.002 1.00 29.17 C

ATOM 1995 C TYR A 407 11.130 −14.660 −42.128 1.00 28.96 C

ATOM 1996 O TYR A 407 12.218 −14.113 −42.338 1.00 28.74 O

ATOM 1997 CB TYR A 407 11.478 −17.109 −42.624 1.00 29.25 C

ATOM 1998 CG TYR A 407 10.986 −18.339 −43.352 1.00 29.54 C

ATOM 1999 CD1 TYR A 407 9.708 −18.833 −43.049 1.00 29.82 C

ATOM 2000 CD2 TYR A 407 11.751 −18.941 −44.369 1.00 29.57 C

ATOM 2001 CE1 TYR A 407 9.187 −19.924 −43.756 1.00 30.10 C

ATOM 2002 CE2 TYR A 407 11.227 −20.040 −45.080 1.00 29.87 C

ATOM 2003 CZ TYR A 407 9.943 −20.535 −44.775 1.00 30.13 C

ATOM 2004 OH TYR A 407 9.435 −21.585 −45.473 1.00 30.46 O

ATOM	2005	H	TYR	A	407	11.551	−16.125	−44.922	1.00	0.00	H
ATOM	2006	HH	TYR	A	407	8.488	−21.742	−45.240	1.00	0.00	H
ATOM	2007	N	ILE	A	408	10.432	−14.453	−41.006	1.00	29.04	N
ATOM	2008	CA	ILE	A	408	11.074	−14.082	−39.735	1.00	28.85	C
ATOM	2009	C	ILE	A	408	11.494	−15.341	−38.970	1.00	28.90	C
ATOM	2010	O	ILE	A	408	10.919	−16.417	−39.154	1.00	29.12	O
ATOM	2011	CB	ILE	A	408	10.209	−13.143	−38.859	1.00	28.90	C
ATOM	2012	CG1	ILE	A	408	8.884	−13.727	−38.317	1.00	29.19	C
ATOM	2013	CG2	ILE	A	408	9.940	−11.835	−39.617	1.00	28.83	C
ATOM	2014	CD1	ILE	A	408	9.015	−14.513	−37.004	1.00	29.25	C
ATOM	2015	H	ILE	A	408	9.570	−14.983	−40.911	1.00	0.00	H
ATOM	2016	N	LEU	A	409	12.480	−15.180	−38.090	1.00	28.70	N
ATOM	2017	CA	LEU	A	409	12.918	−16.181	−37.123	1.00	28.69	C
ATOM	2018	C	LEU	A	409	12.405	−15.803	−35.724	1.00	28.72	C
ATOM	2019	O	LEU	A	409	12.503	−14.639	−35.333	1.00	28.54	O
ATOM	2020	CB	LEU	A	409	14.457	−16.280	−37.145	1.00	28.44	C
ATOM	2021	CG	LEU	A	409	15.110	−16.514	−38.519	1.00	28.40	C
ATOM	2022	CD1	LEU	A	409	16.625	−16.636	−38.356	1.00	28.20	C
ATOM	2023	CD2	LEU	A	409	14.604	−17.787	−39.189	1.00	28.67	C
ATOM	2024	H	LEU	A	409	12.833	−14.237	−37.957	1.00	0.00	H
ATOM	2025	N	ARG	A	410	12.060	−16.798	−34.903	1.00	28.95	N
ATOM	2026	CA	ARG	A	410	11.633	−16.638	−33.499	1.00	29.00	C
ATOM	2027	C	ARG	A	410	12.342	−17.667	−32.618	1.00	28.96	C
ATOM	2028	O	ARG	A	410	12.143	−18.859	−32.825	1.00	29.08	O
ATOM	2029	CB	ARG	A	410	10.104	−16.832	−33.447	1.00	29.32	C
ATOM	2030	CG	ARG	A	410	9.463	−16.556	−32.075	1.00	29.43	C
ATOM	2031	CD	ARG	A	410	9.222	−15.060	−31.837	1.00	29.22	C

ATOM	2032	NE	ARG	A	410	8.336	−14.813	−30.682	1.00	29.41	N
ATOM	2033	CZ	ARG	A	410	7.015	−14.863	−30.656	1.00	29.65	C
ATOM	2034	NH1	ARG	A	410	6.290	−15.248	−31.659	1.00	29.72	N
ATOM	2035	NH2	ARG	A	410	6.388	−14.676	−29.541	1.00	29.82	N
ATOM	2036	H	ARG	A	410	11.967	−17.724	−35.318	1.00	0.00	H
ATOM	2037	HE	ARG	A	410	8.762	−14.671	−29.773	1.00	0.00	H
ATOM	2038	1HH1	ARG	A	410	6.741	−15.705	−32.430	1.00	0.00	H
ATOM	2039	2HH1	ARG	A	410	5.372	−15.615	−31.393	1.00	0.00	H
ATOM	2040	1HH2	ARG	A	410	6.952	−14.687	−28.702	1.00	0.00	H
ATOM	2041	2HH2	ARG	A	410	5.451	−15.085	−29.498	1.00	0.00	H
ATOM	2042	N	SER	A	411	13.165	−17.241	−31.656	1.00	28.78	N
ATOM	2043	CA	SER	A	411	13.763	−18.166	−30.674	1.00	28.71	C
ATOM	2044	C	SER	A	411	12.689	−18.807	−29.784	1.00	28.96	C
ATOM	2045	O	SER	A	411	11.583	−18.282	−29.643	1.00	29.14	O
ATOM	2046	CB	SER	A	411	14.818	−17.475	−29.799	1.00	28.40	C
ATOM	2047	OG	SER	A	411	15.889	−16.997	−30.590	1.00	28.18	O
ATOM	2048	H	SER	A	411	13.305	−16.253	−31.525	1.00	0.00	H
ATOM	2049	HG	SER	A	411	16.656	−17.591	−30.482	1.00	0.00	H
ATOM	2050	N	GLY	A	412	13.016	−19.934	−29.160	1.00	28.98	N
ATOM	2051	CA	GLY	A	412	12.067	−20.702	−28.365	1.00	29.24	C
ATOM	2052	C	GLY	A	412	12.660	−21.996	−27.815	1.00	29.23	C
ATOM	2053	O	GLY	A	412	13.880	−22.133	−27.698	1.00	28.98	O
ATOM	2054	H	GLY	A	412	13.903	−20.379	−29.382	1.00	0.00	H
ATOM	2055	N	LEU	A	413	11.789	−22.969	−27.558	1.00	29.53	N
ATOM	2056	CA	LEU	A	413	12.137	−24.318	−27.113	1.00	29.59	C
ATOM	2057	C	LEU	A	413	11.505	−25.368	−28.032	1.00	29.95	C
ATOM	2058	O	LEU	A	413	10.344	−25.238	−28.415	1.00	30.22	O

ATOM	2059	CB	LEU	A	413	11.678	−24.547	−25.659	1.00	29.66	C
ATOM	2060	CG	LEU	A	413	12.202	−23.538	−24.624	1.00	29.39	C
ATOM	2061	CD1	LEU	A	413	11.518	−23.746	−23.274	1.00	29.64	C
ATOM	2062	CD2	LEU	A	413	13.712	−23.665	−24.417	1.00	29.11	C
ATOM	2063	H	LEU	A	413	10.806	−22.779	−27.741	1.00	0.00	H
ATOM	2064	N	LEU	A	414	12.201	−26.486	−28.234	1.00	29.98	N
ATOM	2065	CA	LEU	A	414	11.608	−27.748	−28.673	1.00	30.37	C
ATOM	2066	C	LEU	A	414	11.495	−28.692	−27.469	1.00	30.54	C
ATOM	2067	O	LEU	A	414	12.492	−29.215	−26.968	1.00	30.32	O
ATOM	2068	CB	LEU	A	414	12.415	−28.337	−29.843	1.00	30.34	C
ATOM	2069	CG	LEU	A	414	12.166	−27.599	−31.171	1.00	30.36	C
ATOM	2070	CD1	LEU	A	414	13.267	−27.907	−32.186	1.00	30.22	C
ATOM	2071	CD2	LEU	A	414	10.832	−27.982	−31.813	1.00	30.80	C
ATOM	2072	H	LEU	A	414	13.141	−26.525	−27.850	1.00	0.00	H
ATOM	2073	N	LYS	A	415	10.266	−28.811	−26.966	1.00	30.93	N
ATOM	2074	CA	LYS	A	415	9.820	−29.746	−25.929	1.00	31.15	C
ATOM	2075	C	LYS	A	415	9.801	−31.174	−26.492	1.00	31.47	C
ATOM	2076	O	LYS	A	415	9.338	−31.381	−27.614	1.00	31.77	O
ATOM	2077	CB	LYS	A	415	8.419	−29.274	−25.498	1.00	31.44	C
ATOM	2078	CG	LYS	A	415	7.795	−30.021	−24.314	1.00	31.88	C
ATOM	2079	CD	LYS	A	415	6.318	−29.618	−24.170	1.00	32.14	C
ATOM	2080	CE	LYS	A	415	5.649	−30.297	−22.969	1.00	32.69	C
ATOM	2081	NZ	LYS	A	415	5.777	−29.485	−21.736	1.00	32.84	N1+
ATOM	2082	H	LYS	A	415	9.527	−28.344	−27.485	1.00	0.00	H
ATOM	2083	HZ1	LYS	A	415	5.371	−29.935	−20.932	1.00	0.00	H
ATOM	2084	HZ2	LYS	A	415	6.738	−29.237	−21.523	1.00	0.00	H
ATOM	2085	HZ3	LYS	A	415	5.300	−28.584	−21.849	1.00	0.00	H

ATOM	2086	N	TYR	A	416	10.211	−32.163	−25.702	1.00	31.42	N
ATOM	2087	CA	TYR	A	416	10.307	−33.573	−26.105	1.00	31.73	C
ATOM	2088	C	TYR	A	416	10.047	−34.480	−24.895	1.00	31.90	C
ATOM	2089	O	TYR	A	416	10.918	−34.631	−24.040	1.00	31.62	O
ATOM	2090	CB	TYR	A	416	11.706	−33.806	−26.697	1.00	31.48	C
ATOM	2091	CG	TYR	A	416	11.950	−35.166	−27.330	1.00	31.86	C
ATOM	2092	CD1	TYR	A	416	11.496	−35.426	−28.638	1.00	32.12	C
ATOM	2093	CD2	TYR	A	416	12.696	−36.142	−26.643	1.00	31.95	C
ATOM	2094	CE1	TYR	A	416	11.835	−36.631	−29.284	1.00	32.49	C
ATOM	2095	CE2	TYR	A	416	13.027	−37.355	−27.278	1.00	32.33	C
ATOM	2096	CZ	TYR	A	416	12.609	−37.595	−28.605	1.00	32.61	C
ATOM	2097	OH	TYR	A	416	12.942	−38.760	−29.219	1.00	33.01	O
ATOM	2098	H	TYR	A	416	10.663	−31.896	−24.832	1.00	0.00	H
ATOM	2099	HH	TYR	A	416	13.212	−39.419	−28.579	1.00	0.00	H
ATOM	2100	N	ASN	A	417	8.797	−34.913	−24.707	1.00	32.37	N
ATOM	2101	CA	ASN	A	417	8.398	−35.716	−23.545	1.00	32.60	C
ATOM	2102	C	ASN	A	417	8.552	−37.220	−23.813	1.00	32.91	C
ATOM	2103	O	ASN	A	417	7.847	−37.779	−24.656	1.00	33.38	O
ATOM	2104	CB	ASN	A	417	6.977	−35.330	−23.114	1.00	32.98	C
ATOM	2105	CG	ASN	A	417	6.643	−35.996	−21.792	1.00	33.03	C
ATOM	2106	OD1	ASN	A	417	7.339	−35.828	−20.807	1.00	32.78	O
ATOM	2107	ND2	ASN	A	417	5.697	−36.900	−21.775	1.00	33.34	N
ATOM	2108	H	ASN	A	417	8.115	−34.751	−25.431	1.00	0.00	H
ATOM	2109	1HD2	ASN	A	417	5.228	−37.168	−22.615	1.00	0.00	H
ATOM	2110	2HD2	ASN	A	417	5.710	−37.504	−20.954	1.00	0.00	H
ATOM	2111	N	LEU	A	418	9.434	−37.879	−23.062	1.00	32.67	N
ATOM	2112	CA	LEU	A	418	9.832	−39.272	−23.280	1.00	32.94	C

ATOM 2113 C LEU A 418 8.716 −40.292 −22.976 1.00 33.45 C
ATOM 2114 O LEU A 418 8.602 −41.266 −23.722 1.00 33.83 O
ATOM 2115 CB LEU A 418 11.102 −39.546 −22.455 1.00 32.53 C
ATOM 2116 CG LEU A 418 12.344 −38.734 −22.875 1.00 32.07 C
ATOM 2117 CD1 LEU A 418 13.437 −38.837 −21.815 1.00 31.67 C
ATOM 2118 CD2 LEU A 418 12.925 −39.241 −24.198 1.00 32.31 C
ATOM 2119 H LEU A 418 9.926 −37.369 −22.330 1.00 0.00 H
ATOM 2120 N SER A 419 7.728 −39.935 −22.141 1.00 33.50 N
ATOM 2121 CA SER A 419 6.552 −40.781 −21.838 1.00 34.04 C
ATOM 2122 C SER A 419 5.671 −41.084 −23.058 1.00 34.59 C
ATOM 2123 O SER A 419 4.808 −41.957 −23.001 1.00 35.09 O
ATOM 2124 CB SER A 419 5.637 −40.177 −20.751 1.00 33.95 C
ATOM 2125 OG SER A 419 6.194 −39.091 −20.042 1.00 33.55 O
ATOM 2126 H SER A 419 7.941 −39.200 −21.476 1.00 0.00 H
ATOM 2127 HG SER A 419 7.087 −39.379 −19.691 1.00 0.00 H
ATOM 2128 N LEU A 420 5.814 −40.320 −24.147 1.00 34.51 N
ATOM 2129 CA LEU A 420 5.107 −40.550 −25.406 1.00 35.00 C
ATOM 2130 C LEU A 420 5.605 −41.846 −26.064 1.00 35.44 C
ATOM 2131 O LEU A 420 5.358 −42.082 −27.250 1.00 35.95 O
ATOM 2132 CB LEU A 420 5.281 −39.320 −26.317 1.00 34.74 C
ATOM 2133 CG LEU A 420 4.695 −38.006 −25.765 1.00 34.51 C
ATOM 2134 CD1 LEU A 420 5.059 −36.856 −26.704 1.00 33.93 C
ATOM 2135 CD2 LEU A 420 3.170 −38.063 −25.652 1.00 34.98 C
ATOM 2136 H LEU A 420 6.579 −39.654 −24.142 1.00 0.00 H
ATOM 2137 N ILE A 424 9.238 −40.377 −30.929 1.00 34.45 N
ATOM 2138 CA ILE A 424 8.601 −39.459 −29.981 1.00 34.27 C
ATOM 2139 C ILE A 424 8.507 −38.057 −30.613 1.00 33.88 C

ATOM 2140 O ILE A 424 9.183 −37.753 −31.599 1.00 33.54 O

ATOM 2141 CB ILE A 424 9.332 −39.509 −28.610 1.00 34.01 C

ATOM 2142 CG1 ILE A 424 8.897 −40.726 −27.756 1.00 34.49 C

ATOM 2143 CG2 ILE A 424 9.106 −38.264 −27.730 1.00 33.66 C

ATOM 2144 CD1 ILE A 424 9.225 −42.106 −28.340 1.00 35.04 C

ATOM 2145 H ILE A 424 9.789 −39.945 −31.663 1.00 0.00 H

ATOM 2146 N ILE A 425 7.499 −37.294 −30.185 1.00 33.98 N

ATOM 2147 CA ILE A 425 7.091 −36.014 −30.779 1.00 33.71 C

ATOM 2148 C ILE A 425 7.971 −34.870 −30.257 1.00 33.14 C

ATOM 2149 O ILE A 425 8.118 −34.718 −29.044 1.00 33.01 O

ATOM 2150 CB ILE A 425 5.590 −35.753 −30.492 1.00 33.99 C

ATOM 2151 CG1 ILE A 425 4.731 −36.969 −30.919 1.00 34.60 C

ATOM 2152 CG2 ILE A 425 5.124 −34.464 −31.185 1.00 33.68 C

ATOM 2153 CD1 ILE A 425 3.213 −36.799 −30.783 1.00 34.81 C

ATOM 2154 H ILE A 425 7.020 −37.585 −29.348 1.00 0.00 H

ATOM 2155 N LEU A 426 8.415 −33.980 −31.151 1.00 32.81 N

ATOM 2156 CA LEU A 426 8.852 −32.628 −30.777 1.00 32.29 C

ATOM 2157 C LEU A 426 7.671 −31.650 −30.799 1.00 32.29 C

ATOM 2158 O LEU A 426 6.949 −31.592 −31.792 1.00 32.54 O

ATOM 2159 CB LEU A 426 9.948 −32.131 −31.733 1.00 31.95 C

ATOM 2160 CG LEU A 426 11.311 −32.820 −31.585 1.00 31.92 C

ATOM 2161 CD1 LEU A 426 12.212 −32.365 −32.729 1.00 31.80 C

ATOM 2162 CD2 LEU A 426 12.032 −32.443 −30.291 1.00 31.56 C

ATOM 2163 H LEU A 426 8.192 −34.143 −32.127 1.00 0.00 H

ATOM 2164 N GLN A 427 7.596 −30.751 −29.819 1.00 32.01 N

ATOM 2165 CA GLN A 427 6.581 −29.691 −29.747 1.00 32.05 C

ATOM 2166 C GLN A 427 7.254 −28.324 −29.546 1.00 31.59 C

ATOM 2167 O GLN A 427 8.116 −28.184 −28.679 1.00 31.34 O
ATOM 2168 CB GLN A 427 5.561 −30.007 −28.637 1.00 32.34 C
ATOM 2169 CG GLN A 427 4.902 −31.389 −28.795 1.00 32.77 C
ATOM 2170 CD GLN A 427 3.775 −31.614 −27.795 1.00 33.14 C
ATOM 2171 OE1 GLN A 427 3.917 −31.395 −26.600 1.00 33.09 O
ATOM 2172 NE2 GLN A 427 2.643 −32.127 −28.222 1.00 33.55 N
ATOM 2173 H GLN A 427 8.243 −30.835 −29.040 1.00 0.00 H
ATOM 2174 1HE2 GLN A 427 2.484 −32.304 −29.225 1.00 0.00 H
ATOM 2175 2HE2 GLN A 427 1.918 −32.267 −27.550 1.00 0.00 H
ATOM 2176 N PHE A 428 6.965 −27.338 −30.400 1.00 31.50 N
ATOM 2177 CA PHE A 428 7.614 −26.020 −30.342 1.00 31.09 C
ATOM 2178 C PHE A 428 6.866 −25.024 −29.445 1.00 31.08 C
ATOM 2179 O PHE A 428 5.677 −24.774 −29.646 1.00 31.41 O
ATOM 2180 CB PHE A 428 7.821 −25.455 −31.750 1.00 31.02 C
ATOM 2181 CG PHE A 428 8.544 −24.116 −31.778 1.00 30.60 C
ATOM 2182 CD1 PHE A 428 9.917 −24.045 −31.474 1.00 30.28 C
ATOM 2183 CD2 PHE A 428 7.844 −22.933 −32.087 1.00 30.54 C
ATOM 2184 CE1 PHE A 428 10.584 −22.806 −31.472 1.00 29.91 C
ATOM 2185 CE2 PHE A 428 8.512 −21.694 −32.092 1.00 30.18 C
ATOM 2186 CZ PHE A 428 9.881 −21.629 −31.781 1.00 29.87 C
ATOM 2187 H PHE A 428 6.244 −27.497 −31.098 1.00 0.00 H
ATOM 2188 N ILE A 429 7.625 −24.304 −28.617 1.00 30.74 N
ATOM 2189 CA ILE A 429 7.180 −23.154 −27.827 1.00 30.70 C
ATOM 2190 C ILE A 429 7.976 −21.914 −28.253 1.00 30.33 C
ATOM 2191 O ILE A 429 9.201 −21.909 −28.167 1.00 30.02 O
ATOM 2192 CB ILE A 429 7.353 −23.469 −26.323 1.00 30.69 C
ATOM 2193 CG1 ILE A 429 6.435 −24.610 −25.817 1.00 31.13 C

ATOM 2194 CG2 ILE A 429 7.161 −22.206 −25.476 1.00 30.51 C

ATOM 2195 CD1 ILE A 429 4.926 −24.325 −25.866 1.00 31.38 C

ATOM 2196 H ILE A 429 8.612 −24.539 −28.561 1.00 0.00 H

ATOM 2197 N GLU A 430 7.288 −20.835 −28.625 1.00 30.39 N

ATOM 2198 CA GLU A 430 7.908 −19.535 −28.906 1.00 30.08 C

ATOM 2199 C GLU A 430 8.277 −18.760 −27.627 1.00 29.90 C

ATOM 2200 O GLU A 430 7.578 −18.840 −26.616 1.00 30.07 O

ATOM 2201 CB GLU A 430 6.991 −18.713 −29.824 1.00 30.21 C

ATOM 2202 CG GLU A 430 5.584 −18.406 −29.269 1.00 30.38 C

ATOM 2203 CD GLU A 430 4.750 −17.576 −30.262 1.00 30.63 C

ATOM 2204 OE1 GLU A 430 4.931 −17.794 −31.480 1.00 30.68 O

ATOM 2205 OE2 GLU A 430 4.396 −16.433 −29.901 1.00 30.79 O

ATOM 2206 H GLU A 430 6.282 −20.874 −28.593 1.00 0.00 H

ATOM 2207 N ILE A 431 9.330 −17.936 −27.699 1.00 29.56 N

ATOM 2208 CA ILE A 431 9.667 −16.948 −26.660 1.00 29.38 C

ATOM 2209 C ILE A 431 8.743 −15.723 −26.718 1.00 29.53 C

ATOM 2210 O ILE A 431 8.392 −15.267 −27.808 1.00 29.62 O

ATOM 2211 CB ILE A 431 11.162 −16.557 −26.752 1.00 29.00 C

ATOM 2212 CG1 ILE A 431 11.657 −15.952 −25.421 1.00 28.83 C

ATOM 2213 CG2 ILE A 431 11.480 −15.590 −27.912 1.00 28.92 C

ATOM 2214 CD1 ILE A 431 13.176 −16.067 −25.259 1.00 28.52 C

ATOM 2215 H ILE A 431 9.904 −17.948 −28.534 1.00 0.00 H

ATOM 2216 N ALA A 432 8.451 −15.112 −25.568 1.00 29.57 N

ATOM 2217 CA ALA A 432 7.614 −13.914 −25.440 1.00 29.73 C

ATOM 2218 C ALA A 432 8.048 −12.710 −26.314 1.00 29.55 C

ATOM 2219 O ALA A 432 9.199 −12.581 −26.733 1.00 29.25 O

ATOM 2220 CB ALA A 432 7.552 −13.532 −23.954 1.00 29.72 C

ATOM 2221 H ALA A 432 8.679 −15.614 −24.716 1.00 0.00 H

ATOM 2222 N ASP A 433 7.120 −11.771 −26.516 1.00 29.73 N

ATOM 2223 CA ASP A 433 7.316 −10.492 −27.217 1.00 29.58 C

ATOM 2224 C ASP A 433 8.240 −9.513 −26.461 1.00 29.37 C

ATOM 2225 O ASP A 433 8.782 −8.571 −27.044 1.00 29.27 O

ATOM 2226 CB ASP A 433 5.942 −9.817 −27.386 1.00 29.85 C

ATOM 2227 CG ASP A 433 5.325 −9.431 −26.032 1.00 30.11 C

ATOM 2228 OD1 ASP A 433 5.072 −10.388 −25.266 1.00 30.09 O

ATOM 2229 OD2 ASP A 433 5.645 −8.305 −25.586 1.00 30.34 O

ATOM 2230 H ASP A 433 6.227 −11.859 −26.035 1.00 0.00 H

ATOM 2231 N ASN A 434 8.360 −9.683 −25.141 1.00 29.30 N

ATOM 2232 CA ASN A 434 9.047 −8.740 −24.266 1.00 29.19 C

ATOM 2233 C ASN A 434 10.544 −8.696 −24.638 1.00 28.85 C

ATOM 2234 O ASN A 434 11.232 −9.709 −24.519 1.00 28.68 O

ATOM 2235 CB ASN A 434 8.842 −9.156 −22.793 1.00 29.31 C

ATOM 2236 CG ASN A 434 7.414 −9.377 −22.302 1.00 29.69 C

ATOM 2237 OD1 ASN A 434 7.223 −9.968 −21.249 1.00 29.84 O

ATOM 2238 ND2 ASN A 434 6.375 −8.931 −22.963 1.00 29.87 N

ATOM 2239 H ASN A 434 7.796 −10.411 −24.729 1.00 0.00 H

ATOM 2240 1HD2 ASN A 434 6.424 −8.600 −23.932 1.00 0.00 H

ATOM 2241 2HD2 ASN A 434 5.475 −9.282 −22.690 1.00 0.00 H

ATOM 2242 N ARG A 435 11.042 −7.553 −25.143 1.00 30.70 N

ATOM 2243 CA ARG A 435 12.352 −7.442 −25.836 1.00 30.41 C

ATOM 2244 C ARG A 435 12.522 −8.508 −26.935 1.00 30.33 C

ATOM 2245 O ARG A 435 13.543 −9.192 −27.006 1.00 30.15 O

ATOM 2246 CB ARG A 435 13.546 −7.390 −24.849 1.00 30.20 C

ATOM 2247 CG ARG A 435 13.652 −6.054 −24.096 1.00 30.27 C

ATOM 2248 CD ARG A 435 14.786 −6.038 −23.057 1.00 30.09 C
ATOM 2249 NE ARG A 435 16.146 −6.118 −23.636 1.00 29.80 N
ATOM 2250 CZ ARG A 435 17.259 −6.313 −22.952 1.00 29.70 C
ATOM 2251 NH1 ARG A 435 18.393 −6.455 −23.565 1.00 29.45 N
ATOM 2252 NH2 ARG A 435 17.264 −6.371 −21.649 1.00 29.88 N
ATOM 2253 H ARG A 435 10.377 −6.805 −25.280 1.00 0.00 H
ATOM 2254 HE ARG A 435 16.274 −6.116 −24.652 1.00 0.00 H
ATOM 2255 1HH1 ARG A 435 18.430 −6.224 −24.571 1.00 0.00 H
ATOM 2256 2HH1 ARG A 435 19.255 −6.499 −23.066 1.00 0.00 H
ATOM 2257 1HH2 ARG A 435 16.464 −6.020 −21.135 1.00 0.00 H
ATOM 2258 2HH2 ARG A 435 18.084 −6.677 −21.145 1.00 0.00 H
ATOM 2259 N LEU A 436 11.526 −8.657 −27.810 1.00 28.55 N
ATOM 2260 CA LEU A 436 11.640 −9.473 −29.018 1.00 28.54 C
ATOM 2261 C LEU A 436 12.813 −8.994 −29.888 1.00 28.30 C
ATOM 2262 O LEU A 436 12.936 −7.812 −30.198 1.00 28.26 O
ATOM 2263 CB LEU A 436 10.307 −9.446 −29.791 1.00 28.79 C
ATOM 2264 CG LEU A 436 10.280 −10.286 −31.080 1.00 28.94 C
ATOM 2265 CD1 LEU A 436 10.450 −11.781 −30.805 1.00 28.93 C
ATOM 2266 CD2 LEU A 436 8.944 −10.092 −31.800 1.00 29.25 C
ATOM 2267 H LEU A 436 10.642 −8.197 −27.628 1.00 0.00 H
ATOM 2268 N THR A 437 13.595 −9.950 −30.379 1.00 28.80 N
ATOM 2269 CA THR A 437 14.648 −9.774 −31.389 1.00 28.61 C
ATOM 2270 C THR A 437 14.591 −10.953 −32.356 1.00 28.65 C
ATOM 2271 O THR A 437 13.956 −11.966 −32.047 1.00 28.76 O
ATOM 2272 CB THR A 437 16.049 −9.672 −30.754 1.00 28.36 C
ATOM 2273 OG1 THR A 437 16.340 −10.798 −29.951 1.00 28.32 O
ATOM 2274 CG2 THR A 437 16.215 −8.444 −29.866 1.00 28.33 C

ATOM 2275 H THR A 437 13.367 −10.906 −30.152 1.00 0.00 H

ATOM 2276 HG1 THR A 437 16.567 −11.540 −30.531 1.00 0.00 H

ATOM 2277 N ILE A 438 15.323 −10.871 −33.473 1.00 27.92 N

ATOM 2278 CA ILE A 438 15.451 −11.970 −34.440 1.00 27.96 C

ATOM 2279 C ILE A 438 15.783 −13.296 −33.751 1.00 27.93 C

ATOM 2280 O ILE A 438 16.641 −13.347 −32.868 1.00 27.78 O

ATOM 2281 CB ILE A 438 16.486 −11.627 −35.538 1.00 27.81 C

ATOM 2282 CG1 ILE A 438 16.565 −12.770 −36.577 1.00 27.92 C

ATOM 2283 CG2 ILE A 438 17.888 −11.313 −34.977 1.00 27.59 C

ATOM 2284 CD1 ILE A 438 17.174 −12.369 −37.922 1.00 27.82 C

ATOM 2285 H ILE A 438 15.847 −10.028 −33.653 1.00 0.00 H

ATOM 2286 N GLY A 439 15.112 −14.368 −34.177 1.00 28.10 N

ATOM 2287 CA GLY A 439 15.406 −15.726 −33.741 1.00 28.11 C

ATOM 2288 C GLY A 439 16.870 −16.076 −33.983 1.00 27.85 C

ATOM 2289 O GLY A 439 17.353 −16.100 −35.114 1.00 27.73 O

ATOM 2290 H GLY A 439 14.345 −14.232 −34.827 1.00 0.00 H

ATOM 2291 N SER A 440 17.580 −16.295 −32.887 1.00 42.44 N

ATOM 2292 CA SER A 440 19.033 −16.396 −32.800 1.00 42.21 C

ATOM 2293 C SER A 440 19.416 −17.476 −31.782 1.00 42.22 C

ATOM 2294 O SER A 440 18.564 −17.853 −30.966 1.00 42.34 O

ATOM 2295 CB SER A 440 19.610 −15.037 −32.418 1.00 42.01 C

ATOM 2296 OG SER A 440 19.157 −14.633 −31.145 1.00 42.03 O

ATOM 2297 H SER A 440 17.090 −16.249 −32.001 1.00 0.00 H

ATOM 2298 HG SER A 440 19.886 −14.136 −30.718 1.00 0.00 H

ATOM 2299 N PRO A 441 20.635 −18.048 −31.852 1.00 27.43 N

ATOM 2300 CA PRO A 441 20.950 −19.217 −31.048 1.00 27.40 C

ATOM 2301 C PRO A 441 20.926 −18.900 −29.550 1.00 27.28 C

ATOM	2302	O	PRO	A	441	21.413	−17.858	−29.105	1.00	27.10	O
ATOM	2303	CB	PRO	A	441	22.297	−19.750	−31.537	1.00	27.22	C
ATOM	2304	CG	PRO	A	441	22.463	−19.121	−32.920	1.00	27.30	C
ATOM	2305	CD	PRO	A	441	21.701	−17.800	−32.812	1.00	27.32	C
ATOM	2306	N	SER	A	442	20.348	−19.803	−28.768	1.00	27.41	N
ATOM	2307	CA	SER	A	442	19.942	−19.567	−27.377	1.00	27.34	C
ATOM	2308	C	SER	A	442	20.324	−20.732	−26.458	1.00	27.40	C
ATOM	2309	O	SER	A	442	20.978	−21.674	−26.888	1.00	27.55	O
ATOM	2310	CB	SER	A	442	18.436	−19.250	−27.352	1.00	27.52	C
ATOM	2311	OG	SER	A	442	17.650	−20.206	−28.043	1.00	27.81	O
ATOM	2312	H	SER	A	442	19.967	−20.631	−29.219	1.00	0.00	H
ATOM	2313	HG	SER	A	442	17.740	−21.064	−27.623	1.00	0.00	H
ATOM	2314	N	LYS	A	443	20.030	−20.626	−25.163	1.00	27.29	N
ATOM	2315	CA	LYS	A	443	20.440	−21.590	−24.132	1.00	27.31	C
ATOM	2316	C	LYS	A	443	19.489	−21.507	−22.944	1.00	27.35	C
ATOM	2317	O	LYS	A	443	19.059	−20.413	−22.584	1.00	27.20	O
ATOM	2318	CB	LYS	A	443	21.907	−21.288	−23.770	1.00	27.03	C
ATOM	2319	CG	LYS	A	443	22.463	−21.918	−22.488	1.00	26.89	C
ATOM	2320	CD	LYS	A	443	23.974	−21.646	−22.417	1.00	26.60	C
ATOM	2321	CE	LYS	A	443	24.557	−21.807	−21.009	1.00	26.37	C
ATOM	2322	NZ	LYS	A	443	26.030	−21.700	−21.039	1.00	26.08	N1+
ATOM	2323	H	LYS	A	443	19.565	−19.778	−24.848	1.00	0.00	H
ATOM	2324	HZ1	LYS	A	443	26.447	−22.624	−21.121	1.00	0.00	H
ATOM	2325	HZ2	LYS	A	443	26.382	−21.071	−21.756	1.00	0.00	H
ATOM	2326	HZ3	LYS	A	443	26.420	−21.388	−20.160	1.00	0.00	H
ATOM	2327	N	ILE	A	444	19.182	−22.642	−22.323	1.00	27.58	N
ATOM	2328	CA	ILE	A	444	18.385	−22.719	−21.089	1.00	27.62	C

ATOM 2329 C ILE A 444 19.063 −23.682 −20.113 1.00 27.48 C

ATOM 2330 O ILE A 444 19.596 −24.700 −20.544 1.00 27.53 O

ATOM 2331 CB ILE A 444 16.902 −23.058 −21.380 1.00 28.00 C

ATOM 2332 CG1 ILE A 444 16.067 −22.967 −20.080 1.00 28.08 C

ATOM 2333 CG2 ILE A 444 16.719 −24.417 −22.072 1.00 28.23 C

ATOM 2334 CD1 ILE A 444 14.549 −23.078 −20.279 1.00 28.47 C

ATOM 2335 H ILE A 444 19.635 −23.499 −22.632 1.00 0.00 H

ATOM 2336 N TYR A 445 19.190 −23.295 −18.840 1.00 27.31 N

ATOM 2337 CA TYR A 445 20.089 −23.972 −17.897 1.00 27.14 C

ATOM 2338 C TYR A 445 19.654 −23.806 −16.429 1.00 27.08 C

ATOM 2339 O TYR A 445 19.313 −22.713 −15.976 1.00 27.00 O

ATOM 2340 CB TYR A 445 21.533 −23.505 −18.147 1.00 26.82 C

ATOM 2341 CG TYR A 445 21.815 −22.020 −17.985 1.00 26.58 C

ATOM 2342 CD1 TYR A 445 22.528 −21.574 −16.862 1.00 26.32 C

ATOM 2343 CD2 TYR A 445 21.433 −21.089 −18.973 1.00 26.63 C

ATOM 2344 CE1 TYR A 445 22.873 −20.219 −16.732 1.00 26.13 C

ATOM 2345 CE2 TYR A 445 21.788 −19.730 −18.857 1.00 26.45 C

ATOM 2346 CZ TYR A 445 22.519 −19.293 −17.733 1.00 26.21 C

ATOM 2347 OH TYR A 445 22.879 −17.990 −17.604 1.00 26.05 O

ATOM 2348 H TYR A 445 18.831 −22.380 −18.577 1.00 0.00 H

ATOM 2349 HH TYR A 445 23.598 −17.760 −18.221 1.00 0.00 H

ATOM 2350 N ASN A 446 19.658 −24.910 −15.672 1.00 48.90 N

ATOM 2351 CA ASN A 446 19.094 −25.023 −14.315 1.00 48.86 C

ATOM 2352 C ASN A 446 19.984 −24.412 −13.205 1.00 48.47 C

ATOM 2353 O ASN A 446 20.363 −25.095 −12.251 1.00 48.24 O

ATOM 2354 CB ASN A 446 18.737 −26.503 −14.042 1.00 49.01 C

ATOM 2355 CG ASN A 446 17.537 −27.004 −14.826 1.00 49.43 C

ATOM 2356 OD1 ASN A 446 16.602 −26.276 −15.103 1.00 49.56 O

ATOM 2357 ND2 ASN A 446 17.488 −28.277 −15.142 1.00 49.67 N

ATOM 2358 H ASN A 446 19.974 −25.762 −16.111 1.00 0.00 H

ATOM 2359 1HD2 ASN A 446 18.243 −28.889 −14.904 1.00 0.00 H

ATOM 2360 2HD2 ASN A 446 16.638 −28.603 −15.579 1.00 0.00 H

ATOM 2361 N SER A 447 20.320 −23.122 −13.300 1.00 26.66 N

ATOM 2362 CA SER A 447 21.146 −22.438 −12.292 1.00 26.31 C

ATOM 2363 C SER A 447 20.483 −22.451 −10.914 1.00 26.35 C

ATOM 2364 O SER A 447 19.441 −21.827 −10.715 1.00 26.61 O

ATOM 2365 CB SER A 447 21.423 −20.979 −12.658 1.00 26.16 C

ATOM 2366 OG SER A 447 22.041 −20.887 −13.917 1.00 26.11 O

ATOM 2367 H SER A 447 19.959 −22.596 −14.087 1.00 0.00 H

ATOM 2368 HG SER A 447 21.383 −20.965 −14.616 1.00 0.00 H

ATOM 2369 N LEU A 448 21.168 −23.003 −9.908 1.00 26.11 N

ATOM 2370 CA LEU A 448 20.837 −22.810 −8.487 1.00 26.08 C

ATOM 2371 C LEU A 448 19.348 −23.088 −8.158 1.00 26.46 C

ATOM 2372 O LEU A 448 18.684 −22.270 −7.524 1.00 26.55 O

ATOM 2373 CB LEU A 448 21.321 −21.415 −8.024 1.00 25.87 C

ATOM 2374 CG LEU A 448 22.781 −21.057 −8.365 1.00 25.61 C

ATOM 2375 CD1 LEU A 448 23.047 −19.604 −7.985 1.00 25.35 C

ATOM 2376 CD2 LEU A 448 23.780 −21.952 −7.631 1.00 25.44 C

ATOM 2377 H LEU A 448 22.026 −23.483 −10.137 1.00 0.00 H

ATOM 2378 N GLY A 449 18.781 −24.152 −8.742 1.00 26.72 N

ATOM 2379 CA GLY A 449 17.403 −24.604 −8.489 1.00 27.11 C

ATOM 2380 C GLY A 449 16.291 −23.976 −9.343 1.00 27.46 C

ATOM 2381 O GLY A 449 15.135 −24.330 −9.145 1.00 27.80 O

ATOM 2382 H GLY A 449 19.359 −24.719 −9.345 1.00 0.00 H

ATOM 2383 N GLN A 450 16.608 −23.114 −10.314 1.00 27.37 N

ATOM 2384 CA GLN A 450 15.624 −22.408 −11.156 1.00 27.66 C

ATOM 2385 C GLN A 450 16.215 −22.161 −12.562 1.00 27.50 C

ATOM 2386 O GLN A 450 17.385 −21.771 −12.643 1.00 27.16 O

ATOM 2387 CB GLN A 450 15.210 −21.115 −10.416 1.00 27.78 C

ATOM 2388 CG GLN A 450 14.780 −19.906 −11.266 1.00 27.81 C

ATOM 2389 CD GLN A 450 13.436 −20.042 −11.966 1.00 28.20 C

ATOM 2390 OE1 GLN A 450 12.672 −20.968 −11.755 1.00 28.43 O

ATOM 2391 NE2 GLN A 450 13.119 −19.091 −12.809 1.00 28.29 N

ATOM 2392 H GLN A 450 17.581 −22.865 −10.440 1.00 0.00 H

ATOM 2393 1HE2 GLN A 450 13.765 −18.350 −13.000 1.00 0.00 H

ATOM 2394 2HE2 GLN A 450 12.212 −19.109 −13.265 1.00 0.00 H

ATOM 2395 N PRO A 451 15.478 −22.358 −13.675 1.00 27.73 N

ATOM 2396 CA PRO A 451 16.040 −22.151 −15.006 1.00 27.60 C

ATOM 2397 C PRO A 451 16.356 −20.681 −15.306 1.00 27.50 C

ATOM 2398 O PRO A 451 15.513 −19.795 −15.154 1.00 27.64 O

ATOM 2399 CB PRO A 451 15.046 −22.736 −16.012 1.00 27.91 C

ATOM 2400 CG PRO A 451 14.166 −23.658 −15.169 1.00 28.25 C

ATOM 2401 CD PRO A 451 14.169 −22.986 −13.798 1.00 28.15 C

ATOM 2402 N VAL A 452 17.515 −20.472 −15.924 1.00 27.28 N

ATOM 2403 CA VAL A 452 17.921 −19.226 −16.585 1.00 27.17 C

ATOM 2404 C VAL A 452 17.888 −19.452 −18.093 1.00 27.20 C

ATOM 2405 O VAL A 452 18.285 −20.518 −18.568 1.00 27.13 O

ATOM 2406 CB VAL A 452 19.322 −18.783 −16.121 1.00 26.83 C

ATOM 2407 CG1 VAL A 452 19.739 −17.423 −16.695 1.00 26.82 C

ATOM 2408 CG2 VAL A 452 19.403 −18.657 −14.599 1.00 26.76 C

ATOM 2409 H VAL A 452 18.123 −21.272 −16.060 1.00 0.00 H

ATOM 2410 N PHE A 453 17.418 −18.464 −18.848 1.00 27.32 N

ATOM 2411 CA PHE A 453 17.481 −18.426 −20.305 1.00 27.39 C

ATOM 2412 C PHE A 453 18.518 −17.397 −20.778 1.00 27.15 C

ATOM 2413 O PHE A 453 18.691 −16.342 −20.165 1.00 27.02 O

ATOM 2414 CB PHE A 453 16.084 −18.132 −20.869 1.00 27.70 C

ATOM 2415 CG PHE A 453 15.937 −18.457 −22.344 1.00 27.81 C

ATOM 2416 CD1 PHE A 453 15.575 −19.760 −22.728 1.00 28.03 C

ATOM 2417 CD2 PHE A 453 16.180 −17.485 −23.333 1.00 27.70 C

ATOM 2418 CE1 PHE A 453 15.489 −20.107 −24.088 1.00 28.15 C

ATOM 2419 CE2 PHE A 453 16.076 −17.827 −24.694 1.00 27.80 C

ATOM 2420 CZ PHE A 453 15.729 −19.136 −25.072 1.00 28.02 C

ATOM 2421 H PHE A 453 17.135 −17.608 −18.380 1.00 0.00 H

ATOM 2422 N TYR A 454 19.100 −17.638 −21.950 1.00 27.11 N

ATOM 2423 CA TYR A 454 19.944 −16.706 −22.695 1.00 26.94 C

ATOM 2424 C TYR A 454 19.599 −16.759 −24.189 1.00 27.06 C

ATOM 2425 O TYR A 454 19.381 −17.837 −24.744 1.00 27.13 O

ATOM 2426 CB TYR A 454 21.429 −17.005 −22.415 1.00 26.67 C

ATOM 2427 CG TYR A 454 22.399 −16.590 −23.511 1.00 26.56 C

ATOM 2428 CD1 TYR A 454 23.106 −15.372 −23.449 1.00 26.46 C

ATOM 2429 CD2 TYR A 454 22.575 −17.439 −24.619 1.00 26.58 C

ATOM 2430 CE1 TYR A 454 24.017 −15.034 −24.473 1.00 26.38 C

ATOM 2431 CE2 TYR A 454 23.468 −17.100 −25.643 1.00 26.49 C

ATOM 2432 CZ TYR A 454 24.214 −15.911 −25.562 1.00 26.39 C

ATOM 2433 OH TYR A 454 25.143 −15.666 −26.522 1.00 26.32 O

ATOM 2434 H TYR A 454 18.900 −18.531 −22.395 1.00 0.00 H

ATOM 2435 HH TYR A 454 25.482 −16.507 −26.837 1.00 0.00 H

ATOM 2436 N GLN A 455 19.701 −15.618 −24.869 1.00 27.06 N

ATOM	2437	CA	GLN	A	455	19.637	−15.494	−26.323	1.00	27.15	C
ATOM	2438	C	GLN	A	455	20.860	−14.718	−26.822	1.00	26.94	C
ATOM	2439	O	GLN	A	455	21.095	−13.594	−26.386	1.00	26.82	O
ATOM	2440	CB	GLN	A	455	18.308	−14.816	−26.705	1.00	27.36	C
ATOM	2441	CG	GLN	A	455	18.171	−14.571	−28.214	1.00	27.48	C
ATOM	2442	CD	GLN	A	455	16.823	−13.982	−28.630	1.00	27.72	C
ATOM	2443	OE1	GLN	A	455	16.031	−13.501	−27.829	1.00	27.83	O
ATOM	2444	NE2	GLN	A	455	16.584	−13.846	−29.914	1.00	27.80	N
ATOM	2445	H	GLN	A	455	19.973	−14.785	−24.352	1.00	0.00	H
ATOM	2446	1HE2	GLN	A	455	17.261	−14.193	−30.591	1.00	0.00	H
ATOM	2447	2HE2	GLN	A	455	15.667	−13.558	−30.205	1.00	0.00	H
ATOM	2448	N	ALA	A	456	21.560	−15.232	−27.835	1.00	26.92	N
ATOM	2449	CA	ALA	A	456	22.638	−14.482	−28.467	1.00	26.76	C
ATOM	2450	C	ALA	A	456	22.082	−13.266	−29.225	1.00	26.83	C
ATOM	2451	O	ALA	A	456	21.237	−13.405	−30.113	1.00	27.02	O
ATOM	2452	CB	ALA	A	456	23.439	−15.415	−29.374	1.00	26.74	C
ATOM	2453	H	ALA	A	456	21.343	−16.157	−28.192	1.00	0.00	H
ATOM	2454	N	SER	A	457	22.593	−12.080	−28.904	1.00	57.19	N
ATOM	2455	CA	SER	A	457	22.084	−10.776	−29.348	1.00	57.25	C
ATOM	2456	C	SER	A	457	22.418	−10.437	−30.815	1.00	57.25	C
ATOM	2457	O	SER	A	457	23.145	−9.493	−31.104	1.00	57.14	O
ATOM	2458	CB	SER	A	457	22.608	−9.713	−28.376	1.00	57.13	C
ATOM	2459	OG	SER	A	457	22.256	−10.059	−27.049	1.00	57.13	O
ATOM	2460	H	SER	A	457	23.167	−12.049	−28.073	1.00	0.00	H
ATOM	2461	HG	SER	A	457	22.183	−9.229	−26.529	1.00	0.00	H
ATOM	2462	N	TYR	A	458	21.943	−11.249	−31.762	1.00	26.89	N
ATOM	2463	CA	TYR	A	458	22.236	−11.165	−33.204	1.00	26.89	C

ATOM 2464 C TYR A 458 21.496 −10.041 −33.968 1.00 26.95 C

ATOM 2465 O TYR A 458 21.383 −10.106 −35.191 1.00 26.95 O

ATOM 2466 CB TYR A 458 22.029 −12.550 −33.847 1.00 27.01 C

ATOM 2467 CG TYR A 458 23.259 −13.434 −33.823 1.00 26.91 C

ATOM 2468 CD1 TYR A 458 24.161 −13.391 −34.904 1.00 26.84 C

ATOM 2469 CD2 TYR A 458 23.505 −14.296 −32.740 1.00 26.88 C

ATOM 2470 CE1 TYR A 458 25.306 −14.211 −34.903 1.00 26.77 C

ATOM 2471 CE2 TYR A 458 24.648 −15.120 −32.736 1.00 26.80 C

ATOM 2472 CZ TYR A 458 25.552 −15.076 −33.817 1.00 26.75 C

ATOM 2473 OH TYR A 458 26.658 −15.866 −33.801 1.00 26.68 O

ATOM 2474 H TYR A 458 21.406 −12.045 −31.440 1.00 0.00 H

ATOM 2475 HH TYR A 458 27.137 −15.798 −34.632 1.00 0.00 H

ATOM 2476 N SER A 459 21.010 −8.994 −33.299 1.00 36.02 N

ATOM 2477 CA SER A 459 20.677 −7.727 −33.971 1.00 36.09 C

ATOM 2478 C SER A 459 20.798 −6.541 −33.003 1.00 36.08 C

ATOM 2479 O SER A 459 21.832 −6.417 −32.353 1.00 35.99 O

ATOM 2480 CB SER A 459 19.353 −7.796 −34.761 1.00 36.27 C

ATOM 2481 OG SER A 459 18.171 −7.810 −33.982 1.00 36.38 O

ATOM 2482 H SER A 459 21.242 −8.931 −32.318 1.00 0.00 H

ATOM 2483 HG SER A 459 17.454 −7.611 −34.606 1.00 0.00 H

ATOM 2484 N TRP A 460 19.880 −5.576 −33.060 1.00 27.19 N

ATOM 2485 CA TRP A 460 20.076 −4.195 −32.607 1.00 27.20 C

ATOM 2486 C TRP A 460 20.078 −3.988 −31.082 1.00 27.19 C

ATOM 2487 O TRP A 460 20.686 −3.028 −30.619 1.00 27.14 O

ATOM 2488 CB TRP A 460 19.001 −3.336 −33.282 1.00 27.38 C

ATOM 2489 CG TRP A 460 17.585 −3.776 −33.051 1.00 27.55 C

ATOM 2490 CD1 TRP A 460 16.830 −4.473 −33.930 1.00 27.66 C

ATOM	2491	CD2	TRP	A	460	16.740	−3.570	−31.875	1.00	27.66	C
ATOM	2492	NE1	TRP	A	460	15.603	−4.758	−33.363	1.00	27.84	N
ATOM	2493	CE2	TRP	A	460	15.489	−4.219	−32.098	1.00	27.85	C
ATOM	2494	CE3	TRP	A	460	16.891	−2.866	−30.659	1.00	27.63	C
ATOM	2495	CZ2	TRP	A	460	14.461	−4.217	−31.144	1.00	28.01	C
ATOM	2496	CZ3	TRP	A	460	15.861	−2.843	−29.701	1.00	27.79	C
ATOM	2497	CH2	TRP	A	460	14.653	−3.525	−29.934	1.00	27.98	C
ATOM	2498	H	TRP	A	460	19.040	−5.767	−33.587	1.00	0.00	H
ATOM	2499	HE1	TRP	A	460	14.892	−5.302	−33.828	1.00	0.00	H
ATOM	2500	N	ASP	A	461	19.457	−4.875	−30.302	1.00	27.26	N
ATOM	2501	CA	ASP	A	461	19.566	−4.928	−28.832	1.00	27.24	C
ATOM	2502	C	ASP	A	461	20.871	−5.653	−28.449	1.00	27.02	C
ATOM	2503	O	ASP	A	461	20.862	−6.870	−28.265	1.00	26.96	O
ATOM	2504	CB	ASP	A	461	18.299	−5.613	−28.265	1.00	27.40	C
ATOM	2505	CG	ASP	A	461	18.248	−5.793	−26.737	1.00	27.43	C
ATOM	2506	OD1	ASP	A	461	19.117	−5.276	−26.004	1.00	27.29	O
ATOM	2507	OD2	ASP	A	461	17.266	−6.389	−26.226	1.00	27.62	O
ATOM	2508	H	ASP	A	461	18.995	−5.644	−30.756	1.00	0.00	H
ATOM	2509	N	THR	A	462	22.026	−4.993	−28.635	1.00	26.91	N
ATOM	2510	CA	THR	A	462	23.347	−5.671	−28.668	1.00	26.71	C
ATOM	2511	C	THR	A	462	23.848	−6.126	−27.296	1.00	26.62	C
ATOM	2512	O	THR	A	462	24.853	−6.842	−27.204	1.00	26.44	O
ATOM	2513	CB	THR	A	462	24.438	−4.816	−29.335	1.00	26.65	C
ATOM	2514	OG1	THR	A	462	24.910	−3.772	−28.515	1.00	26.72	O
ATOM	2515	CG2	THR	A	462	23.978	−4.224	−30.662	1.00	26.73	C
ATOM	2516	H	THR	A	462	21.989	−4.007	−28.885	1.00	0.00	H
ATOM	2517	HG1	THR	A	462	25.388	−4.172	−27.776	1.00	0.00	H

ATOM	2518	N	MET	A	463	23.191	−5.654	−26.236	1.00	26.74	N
ATOM	2519	CA	MET	A	463	23.445	−6.013	−24.842	1.00	26.67	C
ATOM	2520	C	MET	A	463	23.123	−7.489	−24.591	1.00	26.61	C
ATOM	2521	O	MET	A	463	22.294	−8.077	−25.286	1.00	26.70	O
ATOM	2522	CB	MET	A	463	22.605	−5.113	−23.917	1.00	26.86	C
ATOM	2523	CG	MET	A	463	22.806	−3.613	−24.183	1.00	26.94	C
ATOM	2524	SD	MET	A	463	24.529	−3.038	−24.209	1.00	26.74	S
ATOM	2525	CE	MET	A	463	24.994	−3.363	−22.491	1.00	26.56	C
ATOM	2526	H	MET	A	463	22.361	−5.113	−26.429	1.00	0.00	H
ATOM	2527	N	ILE	A	464	23.755	−8.113	−23.593	1.00	54.73	N
ATOM	2528	CA	ILE	A	464	23.518	−9.533	−23.294	1.00	54.68	C
ATOM	2529	C	ILE	A	464	22.059	−9.792	−22.871	1.00	54.89	C
ATOM	2530	O	ILE	A	464	21.601	−9.360	−21.812	1.00	55.00	O
ATOM	2531	CB	ILE	A	464	24.561	−10.088	−22.299	1.00	54.48	C
ATOM	2532	CG1	ILE	A	464	24.378	−11.617	−22.164	1.00	54.44	C
ATOM	2533	CG2	ILE	A	464	24.552	−9.373	−20.933	1.00	54.53	C
ATOM	2534	CD1	ILE	A	464	25.534	−12.326	−21.448	1.00	54.22	C
ATOM	2535	H	ILE	A	464	24.461	−7.605	−23.069	1.00	0.00	H
ATOM	2536	N	LYS	A	465	21.324	−10.557	−23.685	1.00	26.70	N
ATOM	2537	CA	LYS	A	465	19.965	−11.004	−23.362	1.00	26.91	C
ATOM	2538	C	LYS	A	465	20.021	−12.295	−22.544	1.00	26.86	C
ATOM	2539	O	LYS	A	465	20.094	−13.389	−23.097	1.00	26.83	O
ATOM	2540	CB	LYS	A	465	19.143	−11.166	−24.647	1.00	27.06	C
ATOM	2541	CG	LYS	A	465	18.958	−9.846	−25.414	1.00	27.19	C
ATOM	2542	CD	LYS	A	465	18.036	−10.048	−26.621	1.00	27.39	C
ATOM	2543	CE	LYS	A	465	16.576	−10.145	−26.167	1.00	27.64	C
ATOM	2544	NZ	LYS	A	465	15.732	−10.797	−27.187	1.00	27.83	N1+

ATOM	2545	H	LYS	A	465	21.719	−10.837	−24.575	1.00	0.00	H
ATOM	2546	HZ1	LYS	A	465	15.982	−10.487	−28.122	1.00	0.00	H
ATOM	2547	HZ2	LYS	A	465	15.824	−11.811	−27.177	1.00	0.00	H
ATOM	2548	HZ3	LYS	A	465	14.760	−10.538	−27.037	1.00	0.00	H
ATOM	2549	N	LEU	A	466	19.926	−12.165	−21.222	1.00	26.86	N
ATOM	2550	CA	LEU	A	466	19.771	−13.278	−20.276	1.00	26.84	C
ATOM	2551	C	LEU	A	466	18.744	−12.944	−19.185	1.00	27.00	C
ATOM	2552	O	LEU	A	466	18.462	−11.769	−18.959	1.00	27.10	O
ATOM	2553	CB	LEU	A	466	21.144	−13.706	−19.709	1.00	26.55	C
ATOM	2554	CG	LEU	A	466	21.687	−12.914	−18.499	1.00	26.47	C
ATOM	2555	CD1	LEU	A	466	23.035	−13.501	−18.074	1.00	26.18	C
ATOM	2556	CD2	LEU	A	466	21.903	−11.426	−18.774	1.00	26.53	C
ATOM	2557	H	LEU	A	466	19.902	−11.228	−20.845	1.00	0.00	H
ATOM	2558	N	GLY	A	467	18.201	−13.947	−18.498	1.00	27.06	N
ATOM	2559	CA	GLY	A	467	17.263	−13.753	−17.386	1.00	27.20	C
ATOM	2560	C	GLY	A	467	16.701	−15.063	−16.836	1.00	27.36	C
ATOM	2561	O	GLY	A	467	16.761	−16.093	−17.500	1.00	27.44	O
ATOM	2562	H	GLY	A	467	18.392	−14.902	−18.793	1.00	0.00	H
ATOM	2563	N	ASP	A	468	16.158	−15.038	−15.620	1.00	27.42	N
ATOM	2564	CA	ASP	A	468	15.366	−16.158	−15.088	1.00	27.61	C
ATOM	2565	C	ASP	A	468	14.077	−16.373	−15.905	1.00	27.96	C
ATOM	2566	O	ASP	A	468	13.507	−15.413	−16.431	1.00	28.13	O
ATOM	2567	CB	ASP	A	468	15.066	−15.909	−13.599	1.00	27.64	C
ATOM	2568	CG	ASP	A	468	16.284	−16.202	−12.721	1.00	27.32	C
ATOM	2569	OD1	ASP	A	468	16.769	−17.349	−12.790	1.00	27.07	O
ATOM	2570	OD2	ASP	A	468	16.570	−15.445	−11.768	1.00	27.33	O
ATOM	2571	H	ASP	A	468	16.061	−14.148	−15.159	1.00	0.00	H

ATOM	2572	N	VAL	A	469	13.608	−17.621	−16.024	1.00	28.08	N
ATOM	2573	CA	VAL	A	469	12.295	−17.905	−16.636	1.00	28.43	C
ATOM	2574	C	VAL	A	469	11.166	−17.600	−15.648	1.00	28.70	C
ATOM	2575	O	VAL	A	469	11.154	−18.108	−14.529	1.00	28.67	O
ATOM	2576	CB	VAL	A	469	12.182	−19.337	−17.208	1.00	28.48	C
ATOM	2577	CG1	VAL	A	469	13.321	−19.630	−18.194	1.00	28.26	C
ATOM	2578	CG2	VAL	A	469	12.133	−20.456	−16.160	1.00	28.42	C
ATOM	2579	H	VAL	A	469	14.120	−18.388	−15.598	1.00	0.00	H
ATOM	2580	N	ASP	A	470	10.173	−16.818	−16.063	1.00	39.49	N
ATOM	2581	CA	ASP	A	470	8.910	−16.665	−15.328	1.00	39.78	C
ATOM	2582	C	ASP	A	470	7.862	−17.700	−15.777	1.00	40.13	C
ATOM	2583	O	ASP	A	470	6.863	−17.916	−15.092	1.00	40.29	O
ATOM	2584	CB	ASP	A	470	8.352	−15.234	−15.444	1.00	39.91	C
ATOM	2585	CG	ASP	A	470	9.416	−14.160	−15.680	1.00	39.59	C
ATOM	2586	OD1	ASP	A	470	9.761	−13.988	−16.869	1.00	39.27	O
ATOM	2587	OD2	ASP	A	470	9.451	−13.186	−14.897	1.00	39.68	O
ATOM	2588	H	ASP	A	470	10.232	−16.412	−16.993	1.00	0.00	H
ATOM	2589	N	THR	A	471	8.035	−18.305	−16.959	1.00	29.72	N
ATOM	2590	CA	THR	A	471	7.220	−19.432	−17.452	1.00	30.01	C
ATOM	2591	C	THR	A	471	7.951	−20.164	−18.589	1.00	29.86	C
ATOM	2592	O	THR	A	471	8.837	−19.582	−19.218	1.00	29.67	O
ATOM	2593	CB	THR	A	471	5.803	−19.002	−17.913	1.00	30.44	C
ATOM	2594	OG1	THR	A	471	5.441	−17.708	−17.480	1.00	30.39	O
ATOM	2595	CG2	THR	A	471	4.748	−19.935	−17.320	1.00	30.66	C
ATOM	2596	H	THR	A	471	8.834	−18.035	−17.523	1.00	0.00	H
ATOM	2597	HG1	THR	A	471	5.708	−17.665	−16.547	1.00	0.00	H
ATOM	2598	N	VAL	A	472	7.686	−21.466	−18.772	1.00	29.99	N

ATOM	2599	CA	VAL	A	472	8.402	−22.337	−19.738	1.00	29.91	C
ATOM	2600	C	VAL	A	472	7.494	−22.853	−20.860	1.00	30.22	C
ATOM	2601	O	VAL	A	472	7.849	−22.692	−22.024	1.00	30.11	O
ATOM	2602	CB	VAL	A	472	9.124	−23.493	−19.014	1.00	29.92	C
ATOM	2603	CG1	VAL	A	472	9.828	−24.459	−19.977	1.00	29.85	C
ATOM	2604	CG2	VAL	A	472	10.195	−22.941	−18.063	1.00	29.58	C
ATOM	2605	H	VAL	A	472	6.951	−21.878	−18.221	1.00	0.00	H
ATOM	2606	N	ASP	A	473	6.255	−23.232	−20.532	1.00	51.41	N
ATOM	2607	CA	ASP	A	473	5.142	−23.327	−21.482	1.00	51.73	C
ATOM	2608	C	ASP	A	473	4.130	−22.195	−21.182	1.00	52.06	C
ATOM	2609	O	ASP	A	473	3.421	−22.291	−20.176	1.00	52.38	O
ATOM	2610	CB	ASP	A	473	4.431	−24.700	−21.402	1.00	51.95	C
ATOM	2611	CG	ASP	A	473	5.237	−25.949	−21.790	1.00	51.66	C
ATOM	2612	OD1	ASP	A	473	6.480	−25.913	−21.896	1.00	51.33	O
ATOM	2613	OD2	ASP	A	473	4.629	−27.043	−21.839	1.00	51.80	O
ATOM	2614	H	ASP	A	473	6.017	−23.307	−19.560	1.00	0.00	H
ATOM	2615	N	PRO	A	474	3.998	−21.149	−22.025	1.00	31.22	N
ATOM	2616	CA	PRO	A	474	4.957	−20.696	−23.039	1.00	30.90	C
ATOM	2617	C	PRO	A	474	6.233	−20.106	−22.405	1.00	30.47	C
ATOM	2618	O	PRO	A	474	6.238	−19.761	−21.225	1.00	30.41	O
ATOM	2619	CB	PRO	A	474	4.200	−19.625	−23.829	1.00	31.07	C
ATOM	2620	CG	PRO	A	474	3.339	−18.966	−22.752	1.00	31.32	C
ATOM	2621	CD	PRO	A	474	2.956	−20.143	−21.855	1.00	31.52	C
ATOM	2622	N	LEU	A	475	7.292	−19.889	−23.193	1.00	30.19	N
ATOM	2623	CA	LEU	A	475	8.548	−19.352	−22.668	1.00	29.79	C
ATOM	2624	C	LEU	A	475	8.460	−17.831	−22.454	1.00	29.62	C
ATOM	2625	O	LEU	A	475	8.470	−17.048	−23.409	1.00	29.51	O

ATOM 2626 CB LEU A 475 9.727 −19.773 −23.562 1.00 29.57 C

ATOM 2627 CG LEU A 475 11.075 −19.184 −23.098 1.00 29.17 C

ATOM 2628 CD1 LEU A 475 11.530 −19.709 −21.734 1.00 29.09 C

ATOM 2629 CD2 LEU A 475 12.164 −19.495 −24.117 1.00 29.00 C

ATOM 2630 H LEU A 475 7.242 −20.124 −24.174 1.00 0.00 H

ATOM 2631 N ARG A 476 8.621 −17.405 −21.201 1.00 29.61 N

ATOM 2632 CA ARG A 476 8.668 −15.999 −20.776 1.00 29.46 C

ATOM 2633 C ARG A 476 9.810 −15.802 −19.775 1.00 29.09 C

ATOM 2634 O ARG A 476 10.035 −16.637 −18.903 1.00 29.03 O

ATOM 2635 CB ARG A 476 7.262 −15.604 −20.274 1.00 29.73 C

ATOM 2636 CG ARG A 476 7.163 −14.381 −19.354 1.00 29.60 C

ATOM 2637 CD ARG A 476 7.777 −13.066 −19.870 1.00 29.61 C

ATOM 2638 NE ARG A 476 8.600 −12.473 −18.803 1.00 29.28 N

ATOM 2639 CZ ARG A 476 8.393 −11.370 −18.118 1.00 29.29 C

ATOM 2640 NH1 ARG A 476 7.639 −10.399 −18.545 1.00 29.59 N

ATOM 2641 NH2 ARG A 476 8.920 −11.253 −16.936 1.00 29.00 N

ATOM 2642 H ARG A 476 8.672 −18.118 −20.474 1.00 0.00 H

ATOM 2643 HE ARG A 476 9.198 −13.126 −18.294 1.00 0.00 H

ATOM 2644 1HH1 ARG A 476 7.356 −10.416 −19.523 1.00 0.00 H

ATOM 2645 2HH1 ARG A 476 7.552 −9.560 −18.016 1.00 0.00 H

ATOM 2646 1HH2 ARG A 476 9.345 −12.100 −16.530 1.00 0.00 H

ATOM 2647 2HH2 ARG A 476 8.623 −10.545 −16.303 1.00 0.00 H

ATOM 2648 N VAL A 477 10.613 −14.763 −20.018 1.00 28.85 N

ATOM 2649 CA VAL A 477 11.924 −14.529 −19.394 1.00 28.51 C

ATOM 2650 C VAL A 477 11.989 −13.105 −18.848 1.00 28.47 C

ATOM 2651 O VAL A 477 11.765 −12.147 −19.593 1.00 28.48 O

ATOM 2652 CB VAL A 477 13.061 −14.764 −20.417 1.00 28.28 C

ATOM	2653	CG1	VAL	A	477	14.453	−14.578	−19.799	1.00	27.96	C
ATOM	2654	CG2	VAL	A	477	13.002	−16.166	−21.039	1.00	28.42	C
ATOM	2655	H	VAL	A	477	10.353	−14.131	−20.757	1.00	0.00	H
ATOM	2656	N	GLN	A	478	12.409	−12.939	−17.594	1.00	28.42	N
ATOM	2657	CA	GLN	A	478	12.701	−11.630	−17.008	1.00	28.38	C
ATOM	2658	C	GLN	A	478	14.114	−11.186	−17.397	1.00	28.05	C
ATOM	2659	O	GLN	A	478	15.071	−11.307	−16.628	1.00	27.83	O
ATOM	2660	CB	GLN	A	478	12.414	−11.589	−15.498	1.00	28.45	C
ATOM	2661	CG	GLN	A	478	12.926	−12.780	−14.676	1.00	28.17	C
ATOM	2662	CD	GLN	A	478	12.600	−12.608	−13.202	1.00	28.23	C
ATOM	2663	OE1	GLN	A	478	13.440	−12.240	−12.394	1.00	28.04	O
ATOM	2664	NE2	GLN	A	478	11.341	−12.699	−12.834	1.00	28.52	N
ATOM	2665	H	GLN	A	478	12.590	−13.771	−17.037	1.00	0.00	H
ATOM	2666	1HE2	GLN	A	478	10.622	−12.923	−13.530	1.00	0.00	H
ATOM	2667	2HE2	GLN	A	478	11.133	−12.578	−11.863	1.00	0.00	H
ATOM	2668	N	TRP	A	479	14.245	−10.732	−18.647	1.00	28.03	N
ATOM	2669	CA	TRP	A	479	15.504	−10.239	−19.202	1.00	27.76	C
ATOM	2670	C	TRP	A	479	16.131	−9.175	−18.297	1.00	27.66	C
ATOM	2671	O	TRP	A	479	15.516	−8.140	−18.027	1.00	27.86	O
ATOM	2672	CB	TRP	A	479	15.290	−9.624	−20.589	1.00	27.82	C
ATOM	2673	CG	TRP	A	479	14.655	−10.480	−21.637	1.00	27.93	C
ATOM	2674	CD1	TRP	A	479	13.444	−10.248	−22.186	1.00	28.21	C
ATOM	2675	CD2	TRP	A	479	15.197	−11.638	−22.346	1.00	27.79	C
ATOM	2676	NE1	TRP	A	479	13.223	−11.134	−23.215	1.00	28.25	N
ATOM	2677	CE2	TRP	A	479	14.281	−11.998	−23.379	1.00	28.00	C
ATOM	2678	CE3	TRP	A	479	16.379	−12.403	−22.245	1.00	27.52	C
ATOM	2679	CZ2	TRP	A	479	14.544	−13.021	−24.297	1.00	27.97	C

ATOM	2680	CZ3	TRP	A	479	16.636	−13.464	−23.137	1.00	27.49	C
ATOM	2681	CH2	TRP	A	479	15.730	−13.761	−24.171	1.00	27.72	C
ATOM	2682	H	TRP	A	479	13.437	−10.783	−19.256	1.00	0.00	H
ATOM	2683	HE1	TRP	A	479	12.414	−11.051	−23.832	1.00	0.00	H
ATOM	2684	N	ARG	A	480	17.423	−9.323	−17.997	1.00	27.38	N
ATOM	2685	CA	ARG	A	480	18.202	−8.260	−17.361	1.00	27.28	C
ATOM	2686	C	ARG	A	480	18.344	−7.103	−18.347	1.00	27.35	C
ATOM	2687	O	ARG	A	480	18.833	−7.284	−19.465	1.00	27.32	O
ATOM	2688	CB	ARG	A	480	19.578	−8.760	−16.886	1.00	26.96	C
ATOM	2689	CG	ARG	A	480	19.602	−10.100	−16.125	1.00	26.85	C
ATOM	2690	CD	ARG	A	480	18.531	−10.307	−15.045	1.00	26.99	C
ATOM	2691	NE	ARG	A	480	18.764	−9.476	−13.856	1.00	26.84	N
ATOM	2692	CZ	ARG	A	480	18.156	−9.603	−12.693	1.00	26.92	C
ATOM	2693	NH1	ARG	A	480	17.198	−10.467	−12.496	1.00	27.17	N
ATOM	2694	NH2	ARG	A	480	18.486	−8.830	−11.706	1.00	26.78	N
ATOM	2695	H	ARG	A	480	17.893	−10.170	−18.300	1.00	0.00	H
ATOM	2696	HE	ARG	A	480	19.579	−8.856	−13.866	1.00	0.00	H
ATOM	2697	1HH1	ARG	A	480	16.963	−11.096	−13.240	1.00	0.00	H
ATOM	2698	2HH1	ARG	A	480	16.833	−10.625	−11.578	1.00	0.00	H
ATOM	2699	1HH2	ARG	A	480	19.139	−8.069	−11.927	1.00	0.00	H
ATOM	2700	2HH2	ARG	A	480	18.040	−8.887	−10.818	1.00	0.00	H
ATOM	2701	N	ASN	A	481	18.054	−5.883	−17.907	1.00	39.58	N
ATOM	2702	CA	ASN	A	481	18.405	−4.664	−18.644	1.00	39.63	C
ATOM	2703	C	ASN	A	481	19.880	−4.295	−18.407	1.00	39.36	C
ATOM	2704	O	ASN	A	481	20.185	−3.249	−17.843	1.00	39.37	O
ATOM	2705	CB	ASN	A	481	17.381	−3.558	−18.338	1.00	39.93	C
ATOM	2706	CG	ASN	A	481	15.995	−3.900	−18.867	1.00	40.20	C

ATOM	2707	OD1	ASN	A	481	15.818	−4.607	−19.851	1.00	40.19	O
ATOM	2708	ND2	ASN	A	481	14.959	−3.418	−18.221	1.00	40.44	N
ATOM	2709	H	ASN	A	481	17.696	−5.791	−16.970	1.00	0.00	H
ATOM	2710	1HD2	ASN	A	481	15.089	−2.844	−17.409	1.00	0.00	H
ATOM	2711	2HD2	ASN	A	481	14.053	−3.684	−18.562	1.00	0.00	H
ATOM	2712	N	ASN	A	482	20.765	−5.264	−18.670	1.00	27.02	N
ATOM	2713	CA	ASN	A	482	22.193	−5.176	−18.399	1.00	26.76	C
ATOM	2714	C	ASN	A	482	22.821	−4.008	−19.175	1.00	26.76	C
ATOM	2715	O	ASN	A	482	22.593	−3.875	−20.378	1.00	26.82	O
ATOM	2716	CB	ASN	A	482	22.844	−6.532	−18.740	1.00	26.54	C
ATOM	2717	CG	ASN	A	482	24.335	−6.559	−18.440	1.00	26.27	C
ATOM	2718	OD1	ASN	A	482	25.143	−5.925	−19.100	1.00	26.24	O
ATOM	2719	ND2	ASN	A	482	24.755	−7.296	−17.440	1.00	26.09	N
ATOM	2720	H	ASN	A	482	20.426	−6.101	−19.126	1.00	0.00	H
ATOM	2721	1HD2	ASN	A	482	24.094	−7.747	−16.838	1.00	0.00	H
ATOM	2722	2HD2	ASN	A	482	25.715	−7.166	−17.171	1.00	0.00	H
ATOM	2723	N	SER	A	483	23.633	−3.194	−18.499	1.00	30.82	N
ATOM	2724	CA	SER	A	483	24.206	−1.961	−19.054	1.00	30.88	C
ATOM	2725	C	SER	A	483	25.717	−2.041	−19.327	1.00	30.65	C
ATOM	2726	O	SER	A	483	26.358	−0.996	−19.424	1.00	30.70	O
ATOM	2727	CB	SER	A	483	23.839	−0.764	−18.164	1.00	31.07	C
ATOM	2728	OG	SER	A	483	24.672	−0.685	−17.025	1.00	30.95	O
ATOM	2729	H	SER	A	483	23.784	−3.386	−17.512	1.00	0.00	H
ATOM	2730	HG	SER	A	483	25.550	−0.433	−17.341	1.00	0.00	H
ATOM	2731	N	VAL	A	484	26.322	−3.240	−19.298	1.00	26.29	N
ATOM	2732	CA	VAL	A	484	27.792	−3.401	−19.234	1.00	26.09	C
ATOM	2733	C	VAL	A	484	28.392	−4.503	−20.120	1.00	25.96	C

ATOM 2734 O VAL A 484 29.519 −4.331 −20.575 1.00 25.91 O

ATOM 2735 CB VAL A 484 28.276 −3.578 −17.780 1.00 25.92 C

ATOM 2736 CG1 VAL A 484 27.994 −2.347 −16.913 1.00 26.08 C

ATOM 2737 CG2 VAL A 484 27.683 −4.806 −17.074 1.00 25.72 C

ATOM 2738 H VAL A 484 25.741 −4.058 −19.142 1.00 0.00 H

ATOM 2739 N ILE A 485 27.653 −5.558 −20.482 1.00 25.93 N

ATOM 2740 CA ILE A 485 28.118 −6.590 −21.429 1.00 25.83 C

ATOM 2741 C ILE A 485 27.390 −6.438 −22.773 1.00 25.99 C

ATOM 2742 O ILE A 485 26.171 −6.586 −22.852 1.00 26.18 O

ATOM 2743 CB ILE A 485 27.965 −8.011 −20.837 1.00 25.74 C

ATOM 2744 CG1 ILE A 485 28.742 −8.247 −19.521 1.00 25.55 C

ATOM 2745 CG2 ILE A 485 28.343 −9.100 −21.862 1.00 25.69 C

ATOM 2746 CD1 ILE A 485 30.271 −8.168 −19.617 1.00 25.41 C

ATOM 2747 H ILE A 485 26.713 −5.645 −20.108 1.00 0.00 H

ATOM 2748 N SER A 486 28.164 −6.314 −23.855 1.00 25.92 N

ATOM 2749 CA SER A 486 27.695 −6.274 −25.248 1.00 26.04 C

ATOM 2750 C SER A 486 28.661 −7.033 −26.160 1.00 25.93 C

ATOM 2751 O SER A 486 29.875 −6.905 −25.994 1.00 25.79 O

ATOM 2752 CB SER A 486 27.566 −4.819 −25.715 1.00 26.14 C

ATOM 2753 OG SER A 486 27.142 −4.757 −27.067 1.00 26.27 O

ATOM 2754 H SER A 486 29.161 −6.257 −23.708 1.00 0.00 H

ATOM 2755 HG SER A 486 27.785 −5.220 −27.619 1.00 0.00 H

ATOM 2756 N ARG A 487 28.136 −7.689 −27.206 1.00 26.00 N

ATOM 2757 CA ARG A 487 28.914 −8.449 −28.214 1.00 25.93 C

ATOM 2758 C ARG A 487 29.420 −7.589 −29.396 1.00 25.98 C

ATOM 2759 O ARG A 487 28.713 −6.634 −29.741 1.00 26.08 O

ATOM 2760 CB ARG A 487 28.085 −9.670 −28.680 1.00 26.02 C

ATOM 2761 CG ARG A 487 27.147 −9.478 −29.888 1.00 26.18 C

ATOM 2762 CD ARG A 487 26.108 −8.354 −29.781 1.00 26.31 C

ATOM 2763 NE ARG A 487 25.513 −8.047 −31.096 1.00 26.45 N

ATOM 2764 CZ ARG A 487 25.760 −7.007 −31.872 1.00 26.49 C

ATOM 2765 NH1 ARG A 487 26.658 −6.110 −31.602 1.00 26.40 N

ATOM 2766 NH2 ARG A 487 25.081 −6.808 −32.955 1.00 26.62 N

ATOM 2767 H ARG A 487 27.124 −7.683 −27.286 1.00 0.00 H

ATOM 2768 HE ARG A 487 24.695 −8.588 −31.359 1.00 0.00 H

ATOM 2769 1HH1 ARG A 487 27.328 −6.265 −30.861 1.00 0.00 H

ATOM 2770 2HH1 ARG A 487 26.705 −5.297 −32.203 1.00 0.00 H

ATOM 2771 1HH2 ARG A 487 24.466 −7.528 −33.320 1.00 0.00 H

ATOM 2772 2HH2 ARG A 487 25.262 −5.967 −33.487 1.00 0.00 H

ATOM 2773 N PRO A 488 30.507 −7.957 −30.108 1.00 25.91 N

ATOM 2774 CA PRO A 488 30.891 −7.349 −31.392 1.00 25.97 C

ATOM 2775 C PRO A 488 29.763 −7.421 −32.423 1.00 26.10 C

ATOM 2776 O PRO A 488 29.118 −8.457 −32.562 1.00 26.12 O

ATOM 2777 CB PRO A 488 32.114 −8.123 −31.904 1.00 25.88 C

ATOM 2778 CG PRO A 488 32.098 −9.419 −31.100 1.00 25.78 C

ATOM 2779 CD PRO A 488 31.452 −9.011 −29.778 1.00 25.79 C

ATOM 2780 N GLY A 489 29.500 −6.314 −33.118 1.00 26.20 N

ATOM 2781 CA GLY A 489 28.614 −6.288 −34.288 1.00 26.31 C

ATOM 2782 C GLY A 489 29.406 −6.298 −35.590 1.00 26.32 C

ATOM 2783 O GLY A 489 30.530 −6.783 −35.617 1.00 26.24 O

ATOM 2784 H GLY A 489 30.129 −5.528 −32.997 1.00 0.00 H

ATOM 2785 N GLN A 490 28.922 −5.534 −36.564 1.00 26.42 N

ATOM 2786 CA GLN A 490 29.726 −4.814 −37.552 1.00 26.44 C

ATOM 2787 C GLN A 490 29.271 −3.339 −37.549 1.00 26.54 C

ATOM 2788 O GLN A 490 28.921 −2.813 −36.492 1.00 26.57 O
ATOM 2789 CB GLN A 490 29.648 −5.525 −38.919 1.00 26.48 C
ATOM 2790 CG GLN A 490 30.257 −6.936 −38.840 1.00 26.41 C
ATOM 2791 CD GLN A 490 30.789 −7.450 −40.171 1.00 26.47 C
ATOM 2792 OE1 GLN A 490 30.216 −7.275 −41.233 1.00 26.55 O
ATOM 2793 NE2 GLN A 490 31.918 −8.122 −40.164 1.00 26.45 N
ATOM 2794 H GLN A 490 27.972 −5.177 −36.465 1.00 0.00 H
ATOM 2795 1HE2 GLN A 490 32.434 −8.213 −39.304 1.00 0.00 H
ATOM 2796 2HE2 GLN A 490 32.272 −8.410 −41.055 1.00 0.00 H
ATOM 2797 N SER A 491 29.408 −2.608 −38.657 1.00 35.03 N
ATOM 2798 CA SER A 491 29.243 −1.145 −38.700 1.00 35.13 C
ATOM 2799 C SER A 491 27.787 −0.633 −38.693 1.00 35.22 C
ATOM 2800 O SER A 491 27.565 0.519 −39.059 1.00 35.29 O
ATOM 2801 CB SER A 491 30.008 −0.592 −39.907 1.00 35.18 C
ATOM 2802 OG SER A 491 29.399 −1.019 −41.111 1.00 35.16 O
ATOM 2803 H SER A 491 29.699 −3.063 −39.510 1.00 0.00 H
ATOM 2804 HG SER A 491 28.564 −0.541 −41.192 1.00 0.00 H
ATOM 2805 N GLN A 492 26.794 −1.485 −38.413 1.00 26.80 N
ATOM 2806 CA GLN A 492 25.361 −1.137 −38.420 1.00 26.91 C
ATOM 2807 C GLN A 492 24.715 −1.346 −37.036 1.00 26.93 C
ATOM 2808 O GLN A 492 24.024 −0.459 −36.542 1.00 27.05 O
ATOM 2809 CB GLN A 492 24.688 −1.946 −39.546 1.00 26.94 C
ATOM 2810 CG GLN A 492 23.149 −1.946 −39.565 1.00 27.06 C
ATOM 2811 CD GLN A 492 22.488 −0.656 −40.040 1.00 27.18 C
ATOM 2812 OE1 GLN A 492 23.092 0.389 −40.211 1.00 27.18 O
ATOM 2813 NE2 GLN A 492 21.204 −0.705 −40.324 1.00 27.28 N
ATOM 2814 H GLN A 492 27.040 −2.430 −38.149 1.00 0.00 H

ATOM 2815 1HE2 GLN A 492 20.720 −1.590 −40.282 1.00 0.00 H

ATOM 2816 2HE2 GLN A 492 20.749 0.175 −40.493 1.00 0.00 H

ATOM 2817 N CYS A 493 25.108 −2.403 −36.321 1.00 26.82 N

ATOM 2818 CA CYS A 493 24.735 −2.694 −34.934 1.00 26.83 C

ATOM 2819 C CYS A 493 25.978 −3.091 −34.091 1.00 26.69 C

ATOM 2820 O CYS A 493 26.039 −4.225 −33.597 1.00 26.61 O

ATOM 2821 CB CYS A 493 23.617 −3.757 −34.951 1.00 26.88 C

ATOM 2822 SG CYS A 493 22.037 −3.172 −35.630 1.00 27.07 S

ATOM 2823 H CYS A 493 25.664 −3.112 −36.796 1.00 0.00 H

ATOM 2824 N PRO A 494 26.996 −2.210 −33.938 1.00 26.66 N

ATOM 2825 CA PRO A 494 28.217 −2.450 −33.145 1.00 26.53 C

ATOM 2826 C PRO A 494 27.943 −2.540 −31.629 1.00 26.51 C

ATOM 2827 O PRO A 494 26.814 −2.340 −31.185 1.00 26.63 O

ATOM 2828 CB PRO A 494 29.138 −1.266 −33.471 1.00 26.57 C

ATOM 2829 CG PRO A 494 28.155 −0.132 −33.746 1.00 26.74 C

ATOM 2830 CD PRO A 494 27.021 −0.852 −34.469 1.00 26.78 C

ATOM 2831 N ARG A 495 28.974 −2.818 −30.809 1.00 29.12 N

ATOM 2832 CA ARG A 495 28.834 −2.867 −29.333 1.00 29.08 C

ATOM 2833 C ARG A 495 28.155 −1.619 −28.756 1.00 29.24 C

ATOM 2834 O ARG A 495 28.538 −0.498 −29.078 1.00 29.36 O

ATOM 2835 CB ARG A 495 30.183 −3.090 −28.614 1.00 28.96 C

ATOM 2836 CG ARG A 495 30.550 −4.571 −28.566 1.00 28.78 C

ATOM 2837 CD ARG A 495 31.876 −4.925 −27.890 1.00 28.67 C

ATOM 2838 NE ARG A 495 31.867 −4.848 −26.422 1.00 28.62 N

ATOM 2839 CZ ARG A 495 32.892 −4.379 −25.732 1.00 28.53 C

ATOM 2840 NH1 ARG A 495 33.262 −4.972 −24.637 1.00 28.48 N

ATOM 2841 NH2 ARG A 495 33.638 −3.397 −26.145 1.00 28.51 N

ATOM 2842 H ARG A 495 29.881 −2.976 −31.228 1.00 0.00 H

ATOM 2843 HE ARG A 495 31.346 −5.594 −25.979 1.00 0.00 H

ATOM 2844 1HH1 ARG A 495 32.906 −5.888 −24.439 1.00 0.00 H

ATOM 2845 2HH1 ARG A 495 34.176 −4.727 −24.260 1.00 0.00 H

ATOM 2846 1HH2 ARG A 495 33.468 −3.022 −27.072 1.00 0.00 H

ATOM 2847 2HH2 ARG A 495 34.607 −3.434 −25.861 1.00 0.00 H

ATOM 2848 N PHE A 496 27.319 −1.858 −27.745 1.00 44.38 N

ATOM 2849 CA PHE A 496 26.547 −0.867 −26.987 1.00 44.56 C

ATOM 2850 C PHE A 496 25.545 −0.047 −27.820 1.00 44.76 C

ATOM 2851 O PHE A 496 25.055 0.979 −27.346 1.00 44.93 O

ATOM 2852 CB PHE A 496 27.466 −0.017 −26.091 1.00 44.56 C

ATOM 2853 CG PHE A 496 28.371 −0.830 −25.182 1.00 44.39 C

ATOM 2854 CD1 PHE A 496 27.847 −1.411 −24.013 1.00 44.42 C

ATOM 2855 CD2 PHE A 496 29.730 −1.010 −25.501 1.00 44.21 C

ATOM 2856 CE1 PHE A 496 28.674 −2.178 −23.173 1.00 44.26 C

ATOM 2857 CE2 PHE A 496 30.557 −1.778 −24.660 1.00 44.05 C

ATOM 2858 CZ PHE A 496 30.027 −2.367 −23.498 1.00 44.06 C

ATOM 2859 H PHE A 496 27.116 −2.826 −27.543 1.00 0.00 H

ATOM 2860 N ASN A 497 25.114 −0.550 −28.985 1.00 38.11 N

ATOM 2861 CA ASN A 497 23.877 −0.066 −29.588 1.00 38.29 C

ATOM 2862 C ASN A 497 22.645 −0.597 −28.830 1.00 38.39 C

ATOM 2863 O ASN A 497 22.563 −1.768 −28.462 1.00 38.31 O

ATOM 2864 CB ASN A 497 23.820 −0.390 −31.088 1.00 38.25 C

ATOM 2865 CG ASN A 497 22.542 0.156 −31.707 1.00 38.43 C

ATOM 2866 OD1 ASN A 497 22.216 1.324 −31.545 1.00 38.55 O

ATOM 2867 ND2 ASN A 497 21.611 −0.706 −32.031 1.00 38.45 N

ATOM 2868 H ASN A 497 25.459 −1.454 −29.283 1.00 0.00 H

ATOM	2869	1HD2	ASN	A	497	21.680	−1.666	−31.722	1.00	0.00	H
ATOM	2870	2HD2	ASN	A	497	20.714	−0.331	−32.321	1.00	0.00	H
ATOM	2871	N	VAL	A	498	21.638	0.266	−28.710	1.00	52.36	N
ATOM	2872	CA	VAL	A	498	20.312	−0.046	−28.146	1.00	52.51	C
ATOM	2873	C	VAL	A	498	19.185	0.477	−29.056	1.00	52.68	C
ATOM	2874	O	VAL	A	498	18.021	0.120	−28.886	1.00	52.79	O
ATOM	2875	CB	VAL	A	498	20.211	0.502	−26.699	1.00	52.65	C
ATOM	2876	CG1	VAL	A	498	18.941	0.051	−25.964	1.00	52.78	C
ATOM	2877	CG2	VAL	A	498	21.400	0.070	−25.822	1.00	52.48	C
ATOM	2878	H	VAL	A	498	21.803	1.201	−29.052	1.00	0.00	H
ATOM	2879	N	CYS	A	499	19.512	1.294	−30.065	1.00	27.71	N
ATOM	2880	CA	CYS	A	499	18.532	1.829	−31.003	1.00	27.88	C
ATOM	2881	C	CYS	A	499	18.097	0.776	−32.033	1.00	27.79	C
ATOM	2882	O	CYS	A	499	18.969	0.156	−32.644	1.00	27.61	O
ATOM	2883	CB	CYS	A	499	19.099	3.074	−31.687	1.00	27.92	C
ATOM	2884	SG	CYS	A	499	19.005	4.528	−30.615	1.00	28.18	S
ATOM	2885	H	CYS	A	499	20.487	1.419	−30.299	1.00	0.00	H
ATOM	2886	N	PRO	A	500	16.788	0.623	−32.316	1.00	27.95	N
ATOM	2887	CA	PRO	A	500	16.325	−0.359	−33.287	1.00	27.92	C
ATOM	2888	C	PRO	A	500	16.806	−0.068	−34.707	1.00	27.81	C
ATOM	2889	O	PRO	A	500	16.348	0.886	−35.331	1.00	27.87	O
ATOM	2890	CB	PRO	A	500	14.800	−0.382	−33.182	1.00	28.16	C
ATOM	2891	CG	PRO	A	500	14.533	0.135	−31.772	1.00	28.30	C
ATOM	2892	CD	PRO	A	500	15.657	1.141	−31.557	1.00	28.19	C
ATOM	2893	N	GLU	A	501	17.544	−1.007	−35.296	1.00	27.67	N
ATOM	2894	CA	GLU	A	501	18.062	−0.888	−36.660	1.00	27.58	C
ATOM	2895	C	GLU	A	501	17.939	−2.165	−37.486	1.00	27.53	C

ATOM	2896	O	GLU	A	501	17.790	−3.278	−36.972	1.00	27.52	O
ATOM	2897	CB	GLU	A	501	19.510	−0.356	−36.671	1.00	27.41	C
ATOM	2898	CG	GLU	A	501	19.604	1.177	−36.589	1.00	27.44	C
ATOM	2899	CD	GLU	A	501	18.743	1.918	−37.632	1.00	27.59	C
ATOM	2900	OE1	GLU	A	501	18.409	1.311	−38.683	1.00	27.60	O
ATOM	2901	OE2	GLU	A	501	18.120	2.926	−37.223	1.00	27.70	O
ATOM	2902	H	GLU	A	501	17.859	−1.792	−34.746	1.00	0.00	H
ATOM	2903	N	VAL	A	502	17.913	−1.979	−38.807	1.00	27.52	N
ATOM	2904	CA	VAL	A	502	17.830	−3.079	−39.772	1.00	27.49	C
ATOM	2905	C	VAL	A	502	19.177	−3.800	−39.834	1.00	27.33	C
ATOM	2906	O	VAL	A	502	20.052	−3.424	−40.611	1.00	27.25	O
ATOM	2907	CB	VAL	A	502	17.370	−2.587	−41.160	1.00	27.53	C
ATOM	2908	CG1	VAL	A	502	17.185	−3.761	−42.131	1.00	27.50	C
ATOM	2909	CG2	VAL	A	502	16.025	−1.855	−41.072	1.00	27.70	C
ATOM	2910	H	VAL	A	502	18.055	−1.030	−39.147	1.00	0.00	H
ATOM	2911	N	CYS	A	503	19.344	−4.827	−39.003	1.00	28.33	N
ATOM	2912	CA	CYS	A	503	20.543	−5.663	−38.948	1.00	28.19	C
ATOM	2913	C	CYS	A	503	20.222	−7.121	−38.583	1.00	28.20	C
ATOM	2914	O	CYS	A	503	19.201	−7.409	−37.947	1.00	28.29	O
ATOM	2915	CB	CYS	A	503	21.556	−5.042	−37.975	1.00	28.09	C
ATOM	2916	SG	CYS	A	503	21.033	−4.838	−36.251	1.00	28.16	S
ATOM	2917	H	CYS	A	503	18.661	−4.951	−38.268	1.00	0.00	H
ATOM	2918	N	TRP	A	504	21.155	−8.009	−38.939	1.00	28.10	N
ATOM	2919	CA	TRP	A	504	21.322	−9.360	−38.399	1.00	28.10	C
ATOM	2920	C	TRP	A	504	22.824	−9.643	−38.282	1.00	27.95	C
ATOM	2921	O	TRP	A	504	23.486	−9.941	−39.273	1.00	27.94	O
ATOM	2922	CB	TRP	A	504	20.620	−10.421	−39.259	1.00	28.23	C

ATOM	2923	CG	TRP	A	504	20.896	−11.841	−38.839	1.00	28.24	C
ATOM	2924	CD1	TRP	A	504	20.707	−12.337	−37.594	1.00	28.21	C
ATOM	2925	CD2	TRP	A	504	21.484	−12.940	−39.605	1.00	28.31	C
ATOM	2926	NE1	TRP	A	504	21.059	−13.669	−37.555	1.00	28.25	N
ATOM	2927	CE2	TRP	A	504	21.547	−14.098	−38.770	1.00	28.32	C
ATOM	2928	CE3	TRP	A	504	21.986	−13.083	−40.920	1.00	28.36	C
ATOM	2929	CZ2	TRP	A	504	22.038	−15.331	−39.215	1.00	28.41	C
ATOM	2930	CZ3	TRP	A	504	22.491	−14.317	−41.377	1.00	28.45	C
ATOM	2931	CH2	TRP	A	504	22.510	−15.443	−40.534	1.00	28.48	C
ATOM	2932	H	TRP	A	504	21.952	−7.655	−39.451	1.00	0.00	H
ATOM	2933	HE1	TRP	A	504	21.002	−14.237	−36.723	1.00	0.00	H
ATOM	2934	N	GLU	A	505	23.391	−9.299	−37.128	1.00	44.40	N
ATOM	2935	CA	GLU	A	505	24.831	−9.325	−36.870	1.00	44.26	C
ATOM	2936	C	GLU	A	505	25.118	−9.435	−35.368	1.00	44.18	C
ATOM	2937	O	GLU	A	505	24.330	−9.000	−34.521	1.00	44.22	O
ATOM	2938	CB	GLU	A	505	25.526	−8.089	−37.475	1.00	44.21	C
ATOM	2939	CG	GLU	A	505	25.227	−6.777	−36.737	1.00	44.24	C
ATOM	2940	CD	GLU	A	505	25.537	−5.563	−37.617	1.00	44.26	C
ATOM	2941	OE1	GLU	A	505	24.724	−5.305	−38.527	1.00	44.24	O
ATOM	2942	OE2	GLU	A	505	26.351	−4.724	−37.166	1.00	44.33	O
ATOM	2943	H	GLU	A	505	22.786	−9.091	−36.343	1.00	0.00	H
ATOM	2944	N	GLY	A	506	26.263	−10.021	−35.038	1.00	26.50	N
ATOM	2945	CA	GLY	A	506	26.666	−10.348	−33.676	1.00	26.39	C
ATOM	2946	C	GLY	A	506	27.616	−11.544	−33.668	1.00	26.34	C
ATOM	2947	O	GLY	A	506	27.719	−12.250	−34.673	1.00	26.39	O
ATOM	2948	H	GLY	A	506	26.848	−10.393	−35.774	1.00	0.00	H
ATOM	2949	N	THR	A	507	28.075	−11.935	−32.479	1.00	26.24	N

ATOM	2950	CA	THR	A	507	28.517	−13.313	−32.223	1.00	26.20	C
ATOM	2951	C	THR	A	507	27.970	−13.847	−30.899	1.00	26.19	C
ATOM	2952	O	THR	A	507	27.530	−13.095	−30.024	1.00	26.16	O
ATOM	2953	CB	THR	A	507	30.037	−13.532	−32.335	1.00	26.05	C
ATOM	2954	OG1	THR	A	507	30.704	−13.230	−31.139	1.00	25.91	O
ATOM	2955	CG2	THR	A	507	30.745	−12.780	−33.459	1.00	26.09	C
ATOM	2956	H	THR	A	507	27.949	−11.332	−31.682	1.00	0.00	H
ATOM	2957	HG1	THR	A	507	31.515	−13.770	−31.176	1.00	0.00	H
ATOM	2958	N	TYR	A	508	27.905	−15.172	−30.779	1.00	26.23	N
ATOM	2959	CA	TYR	A	508	27.410	−15.864	−29.592	1.00	26.23	C
ATOM	2960	C	TYR	A	508	28.416	−15.743	−28.427	1.00	26.02	C
ATOM	2961	O	TYR	A	508	29.510	−16.304	−28.492	1.00	25.93	O
ATOM	2962	CB	TYR	A	508	27.116	−17.312	−30.014	1.00	26.38	C
ATOM	2963	CG	TYR	A	508	26.492	−18.197	−28.958	1.00	26.39	C
ATOM	2964	CD1	TYR	A	508	25.155	−18.618	−29.069	1.00	26.60	C
ATOM	2965	CD2	TYR	A	508	27.265	−18.636	−27.876	1.00	26.22	C
ATOM	2966	CE1	TYR	A	508	24.593	−19.475	−28.099	1.00	26.63	C
ATOM	2967	CE2	TYR	A	508	26.712	−19.482	−26.905	1.00	26.23	C
ATOM	2968	CZ	TYR	A	508	25.368	−19.888	−26.995	1.00	26.44	C
ATOM	2969	OH	TYR	A	508	24.827	−20.648	−26.015	1.00	26.46	O
ATOM	2970	H	TYR	A	508	28.273	−15.733	−31.531	1.00	0.00	H
ATOM	2971	HH	TYR	A	508	25.380	−20.613	−25.198	1.00	0.00	H
ATOM	2972	N	ASN	A	509	28.004	−15.100	−27.329	1.00	25.97	N
ATOM	2973	CA	ASN	A	509	28.809	−14.855	−26.125	1.00	25.77	C
ATOM	2974	C	ASN	A	509	27.963	−15.148	−24.871	1.00	25.80	C
ATOM	2975	O	ASN	A	509	27.151	−14.301	−24.498	1.00	25.82	O
ATOM	2976	CB	ASN	A	509	29.293	−13.388	−26.115	1.00	25.64	C

ATOM 2977 CG ASN A 509 30.307 −13.048 −27.188 1.00 25.59 C

ATOM 2978 OD1 ASN A 509 31.470 −12.831 −26.910 1.00 25.46 O

ATOM 2979 ND2 ASN A 509 29.876 −12.792 −28.398 1.00 25.70 N

ATOM 2980 H ASN A 509 27.062 −14.731 −27.309 1.00 0.00 H

ATOM 2981 1HD2 ASN A 509 28.911 −12.939 −28.649 1.00 0.00 H

ATOM 2982 2HD2 ASN A 509 30.567 −12.678 −29.127 1.00 0.00 H

ATOM 2983 N ASP A 510 28.012 −16.376 −24.333 1.00 25.84 N

ATOM 2984 CA ASP A 510 27.130 −16.788 −23.223 1.00 25.87 C

ATOM 2985 C ASP A 510 27.815 −16.835 −21.844 1.00 25.65 C

ATOM 2986 O ASP A 510 29.041 −16.904 −21.710 1.00 25.46 O

ATOM 2987 CB ASP A 510 26.279 −18.029 −23.576 1.00 26.03 C

ATOM 2988 CG ASP A 510 26.884 −19.413 −23.320 1.00 25.92 C

ATOM 2989 OD1 ASP A 510 27.427 −19.634 −22.221 1.00 25.71 O

ATOM 2990 OD2 ASP A 510 26.378 −20.368 −23.947 1.00 26.06 O

ATOM 2991 H ASP A 510 28.786 −16.979 −24.581 1.00 0.00 H

ATOM 2992 N ALA A 511 26.976 −16.691 −20.817 1.00 25.68 N

ATOM 2993 CA ALA A 511 27.347 −16.546 −19.416 1.00 25.49 C

ATOM 2994 C ALA A 511 26.333 −17.265 −18.512 1.00 25.56 C

ATOM 2995 O ALA A 511 25.180 −17.486 −18.898 1.00 25.77 O

ATOM 2996 CB ALA A 511 27.440 −15.049 −19.091 1.00 25.47 C

ATOM 2997 H ALA A 511 25.987 −16.711 −21.012 1.00 0.00 H

ATOM 2998 N PHE A 512 26.745 −17.555 −17.280 1.00 25.40 N

ATOM 2999 CA PHE A 512 26.056 −18.434 −16.338 1.00 25.44 C

ATOM 3000 C PHE A 512 25.835 −17.743 −14.986 1.00 25.38 C

ATOM 3001 O PHE A 512 26.763 −17.145 −14.444 1.00 25.18 O

ATOM 3002 CB PHE A 512 26.902 −19.701 −16.195 1.00 25.28 C

ATOM 3003 CG PHE A 512 26.203 −20.909 −15.605 1.00 25.41 C

ATOM 3004 CD1 PHE A 512 25.847 −21.983 −16.442 1.00 25.60 C
ATOM 3005 CD2 PHE A 512 25.968 −20.999 −14.220 1.00 25.36 C
ATOM 3006 CE1 PHE A 512 25.321 −23.166 −15.894 1.00 25.75 C
ATOM 3007 CE2 PHE A 512 25.403 −22.166 −13.677 1.00 25.50 C
ATOM 3008 CZ PHE A 512 25.095 −23.255 −14.510 1.00 25.71 C
ATOM 3009 H PHE A 512 27.704 −17.317 −17.048 1.00 0.00 H
ATOM 3010 N LEU A 513 24.639 −17.861 −14.404 1.00 25.57 N
ATOM 3011 CA LEU A 513 24.291 −17.278 −13.103 1.00 25.55 C
ATOM 3012 C LEU A 513 24.958 −18.041 −11.939 1.00 25.33 C
ATOM 3013 O LEU A 513 24.559 −19.159 −11.616 1.00 25.37 O
ATOM 3014 CB LEU A 513 22.757 −17.266 −13.002 1.00 25.85 C
ATOM 3015 CG LEU A 513 22.193 −16.566 −11.754 1.00 25.88 C
ATOM 3016 CD1 LEU A 513 22.444 −15.063 −11.800 1.00 25.85 C
ATOM 3017 CD2 LEU A 513 20.685 −16.796 −11.695 1.00 26.20 C
ATOM 3018 H LEU A 513 23.916 −18.369 −14.889 1.00 0.00 H
ATOM 3019 N ILE A 514 25.961 −17.432 −11.296 1.00 25.10 N
ATOM 3020 CA ILE A 514 26.731 −18.035 −10.188 1.00 24.87 C
ATOM 3021 C ILE A 514 26.261 −17.607 −8.787 1.00 24.86 C
ATOM 3022 O ILE A 514 26.547 −18.307 −7.819 1.00 24.69 O
ATOM 3023 CB ILE A 514 28.249 −17.820 −10.372 1.00 24.59 C
ATOM 3024 CG1 ILE A 514 28.657 −16.331 −10.332 1.00 24.54 C
ATOM 3025 CG2 ILE A 514 28.746 −18.514 −11.653 1.00 24.63 C
ATOM 3026 CD1 ILE A 514 30.156 −16.137 −10.082 1.00 24.27 C
ATOM 3027 H ILE A 514 26.187 −16.482 −11.564 1.00 0.00 H
ATOM 3028 N ASP A 515 25.427 −16.568 −8.688 1.00 25.05 N
ATOM 3029 CA ASP A 515 24.532 −16.325 −7.549 1.00 25.09 C
ATOM 3030 C ASP A 515 23.263 −15.601 −8.019 1.00 25.41 C

```
ATOM   3031  O   ASP A 515      23.348 −14.541  −8.635  1.00 25.46           O
ATOM   3032  CB  ASP A 515      25.229 −15.516  −6.443  1.00 24.84           C
ATOM   3033  CG  ASP A 515      24.283 −15.203  −5.274  1.00 24.86           C
ATOM   3034  OD1 ASP A 515      24.347 −14.050  −4.794  1.00 24.79           O
ATOM   3035  OD2 ASP A 515      23.290 −15.943  −5.090  1.00 24.97           O
ATOM   3036  H   ASP A 515      25.279 −16.001  −9.516  1.00  0.00           H
ATOM   3037  N   ARG A 516      22.081 −16.113  −7.654  1.00 25.65           N
ATOM   3038  CA  ARG A 516      20.793 −15.452  −7.915  1.00 25.98           C
ATOM   3039  C   ARG A 516      20.472 −14.332  −6.919  1.00 26.04           C
ATOM   3040  O   ARG A 516      19.787 −13.388  −7.299  1.00 26.23           O
ATOM   3041  CB  ARG A 516      19.680 −16.514  −7.971  1.00 26.22           C
ATOM   3042  CG  ARG A 516      18.327 −15.915  −8.402  1.00 26.59           C
ATOM   3043  CD  ARG A 516      17.270 −16.975  −8.732  1.00 26.83           C
ATOM   3044  NE  ARG A 516      17.539 −17.627 −10.024  1.00 26.90           N
ATOM   3045  CZ  ARG A 516      18.023 −18.831 −10.255  1.00 26.75           C
ATOM   3046  NH1 ARG A 516      18.361 −19.655  −9.309  1.00 26.51           N
ATOM   3047  NH2 ARG A 516      18.138 −19.257 −11.471  1.00 26.85           N
ATOM   3048  H   ARG A 516      22.112 −16.879  −6.994  1.00  0.00           H
ATOM   3049  HE  ARG A 516      17.274 −17.097 −10.861  1.00  0.00           H
ATOM   3050 1HH1 ARG A 516      18.183 −19.434  −8.351  1.00  0.00           H
ATOM   3051 2HH1 ARG A 516      18.696 −20.572  −9.578  1.00  0.00           H
ATOM   3052 1HH2 ARG A 516      17.728 −18.651 −12.190  1.00  0.00           H
ATOM   3053 2HH2 ARG A 516      18.273 −20.242 −11.664  1.00  0.00           H
ATOM   3054  N   LEU A 517      20.974 −14.388  −5.683  1.00 48.11           N
ATOM   3055  CA  LEU A 517      20.622 −13.433  −4.622  1.00 48.15           C
ATOM   3056  C   LEU A 517      21.212 −12.036  −4.872  1.00 48.13           C
ATOM   3057  O   LEU A 517      20.497 −11.042  −4.797  1.00 48.35           O
```

ATOM	3058	CB	LEU	A	517	21.071	−13.986	−3.256	1.00	47.91	C
ATOM	3059	CG	LEU	A	517	20.491	−15.365	−2.884	1.00	48.03	C
ATOM	3060	CD1	LEU	A	517	21.051	−15.811	−1.532	1.00	47.84	C
ATOM	3061	CD2	LEU	A	517	18.963	−15.344	−2.779	1.00	48.44	C
ATOM	3062	H	LEU	A	517	21.657	−15.113	−5.465	1.00	0.00	H
ATOM	3063	N	ASN	A	518	22.463	−11.975	−5.331	1.00	51.68	N
ATOM	3064	CA	ASN	A	518	23.101	−10.761	−5.855	1.00	51.67	C
ATOM	3065	C	ASN	A	518	22.906	−10.579	−7.379	1.00	51.69	C
ATOM	3066	O	ASN	A	518	23.472	−9.645	−7.946	1.00	51.58	O
ATOM	3067	CB	ASN	A	518	24.593	−10.786	−5.468	1.00	51.37	C
ATOM	3068	CG	ASN	A	518	24.816	−10.698	−3.971	1.00	51.27	C
ATOM	3069	OD1	ASN	A	518	25.105	−9.644	−3.434	1.00	51.49	O
ATOM	3070	ND2	ASN	A	518	24.794	−11.802	−3.267	1.00	50.94	N
ATOM	3071	H	ASN	A	518	23.014	−12.832	−5.309	1.00	0.00	H
ATOM	3072	1HD2	ASN	A	518	24.602	−12.690	−3.739	1.00	0.00	H
ATOM	3073	2HD2	ASN	A	518	24.938	−11.733	−2.282	1.00	0.00	H
ATOM	3074	N	TRP	A	519	22.248	−11.541	−8.040	1.00	25.85	N
ATOM	3075	CA	TRP	A	519	22.129	−11.727	−9.497	1.00	25.89	C
ATOM	3076	C	TRP	A	519	23.455	−11.548	−10.268	1.00	25.65	C
ATOM	3077	O	TRP	A	519	23.581	−10.745	−11.195	1.00	25.72	O
ATOM	3078	CB	TRP	A	519	20.889	−11.013	−10.071	1.00	26.21	C
ATOM	3079	CG	TRP	A	519	20.049	−11.914	−10.935	1.00	26.35	C
ATOM	3080	CD1	TRP	A	519	18.989	−12.637	−10.500	1.00	26.48	C
ATOM	3081	CD2	TRP	A	519	20.267	−12.337	−12.320	1.00	26.37	C
ATOM	3082	NE1	TRP	A	519	18.524	−13.452	−11.516	1.00	26.59	N
ATOM	3083	CE2	TRP	A	519	19.303	−13.340	−12.645	1.00	26.52	C
ATOM	3084	CE3	TRP	A	519	21.202	−12.009	−13.328	1.00	26.28	C

ATOM	3085	CZ2	TRP	A	519	19.284	−14.005	−13.878	1.00	26.60	C
ATOM	3086	CZ3	TRP	A	519	21.202	−12.680	−14.568	1.00	26.34	C
ATOM	3087	CH2	TRP	A	519	20.251	−13.681	−14.844	1.00	26.50	C
ATOM	3088	H	TRP	A	519	21.792	−12.237	−7.469	1.00	0.00	H
ATOM	3089	HE1	TRP	A	519	17.759	−14.133	−11.416	1.00	0.00	H
ATOM	3090	N	VAL	A	520	24.496	−12.241	−9.793	1.00	25.37	N
ATOM	3091	CA	VAL	A	520	25.847	−12.242	−10.370	1.00	25.14	C
ATOM	3092	C	VAL	A	520	26.014	−13.417	−11.335	1.00	25.13	C
ATOM	3093	O	VAL	A	520	25.909	−14.580	−10.944	1.00	25.15	O
ATOM	3094	CB	VAL	A	520	26.925	−12.256	−9.269	1.00	24.85	C
ATOM	3095	CG1	VAL	A	520	28.345	−12.373	−9.836	1.00	24.63	C
ATOM	3096	CG2	VAL	A	520	26.877	−10.958	−8.453	1.00	24.87	C
ATOM	3097	H	VAL	A	520	24.279	−12.953	−9.103	1.00	0.00	H
ATOM	3098	N	SER	A	521	26.391	−13.112	−12.574	1.00	25.13	N
ATOM	3099	CA	SER	A	521	26.748	−14.075	−13.622	1.00	25.14	C
ATOM	3100	C	SER	A	521	28.250	−14.068	−13.936	1.00	24.91	C
ATOM	3101	O	SER	A	521	28.964	−13.116	−13.618	1.00	24.76	O
ATOM	3102	CB	SER	A	521	25.935	−13.795	−14.889	1.00	25.37	C
ATOM	3103	OG	SER	A	521	24.554	−13.974	−14.634	1.00	25.58	O
ATOM	3104	H	SER	A	521	26.569	−12.133	−12.774	1.00	0.00	H
ATOM	3105	HG	SER	A	521	24.279	−13.346	−13.956	1.00	0.00	H
ATOM	3106	N	ALA	A	522	28.724	−15.114	−14.614	1.00	24.89	N
ATOM	3107	CA	ALA	A	522	30.107	−15.283	−15.054	1.00	24.73	C
ATOM	3108	C	ALA	A	522	30.175	−15.861	−16.477	1.00	24.83	C
ATOM	3109	O	ALA	A	522	29.405	−16.760	−16.813	1.00	25.01	O
ATOM	3110	CB	ALA	A	522	30.824	−16.187	−14.049	1.00	24.46	C
ATOM	3111	H	ALA	A	522	28.091	−15.888	−14.793	1.00	0.00	H

ATOM	3112	N	GLY	A	523	31.096	−15.380	−17.312	1.00	24.74	N
ATOM	3113	CA	GLY	A	523	31.263	−15.879	−18.680	1.00	24.82	C
ATOM	3114	C	GLY	A	523	32.318	−15.132	−19.487	1.00	24.76	C
ATOM	3115	O	GLY	A	523	32.798	−14.077	−19.073	1.00	24.65	O
ATOM	3116	H	GLY	A	523	31.707	−14.629	−17.000	1.00	0.00	H
ATOM	3117	N	VAL	A	524	32.692	−15.693	−20.637	1.00	24.84	N
ATOM	3118	CA	VAL	A	524	33.604	−15.043	−21.592	1.00	24.80	C
ATOM	3119	C	VAL	A	524	32.809	−14.192	−22.578	1.00	24.97	C
ATOM	3120	O	VAL	A	524	31.868	−14.695	−23.200	1.00	25.12	O
ATOM	3121	CB	VAL	A	524	34.511	−16.048	−22.327	1.00	24.77	C
ATOM	3122	CG1	VAL	A	524	35.590	−15.364	−23.183	1.00	24.84	C
ATOM	3123	CG2	VAL	A	524	35.238	−16.966	−21.347	1.00	24.56	C
ATOM	3124	H	VAL	A	524	32.219	−16.537	−20.929	1.00	0.00	H
ATOM	3125	N	TYR	A	525	33.285	−12.973	−22.831	1.00	24.94	N
ATOM	3126	CA	TYR	A	525	32.801	−12.090	−23.891	1.00	25.10	C
ATOM	3127	C	TYR	A	525	33.961	−11.477	−24.683	1.00	25.08	C
ATOM	3128	O	TYR	A	525	35.053	−11.266	−24.155	1.00	24.95	O
ATOM	3129	CB	TYR	A	525	31.830	−11.023	−23.342	1.00	25.14	C
ATOM	3130	CG	TYR	A	525	32.426	−9.796	−22.653	1.00	25.07	C
ATOM	3131	CD1	TYR	A	525	32.019	−8.500	−23.042	1.00	25.21	C
ATOM	3132	CD2	TYR	A	525	33.328	−9.940	−21.579	1.00	24.90	C
ATOM	3133	CE1	TYR	A	525	32.497	−7.366	−22.349	1.00	25.19	C
ATOM	3134	CE2	TYR	A	525	33.848	−8.809	−20.923	1.00	24.87	C
ATOM	3135	CZ	TYR	A	525	33.426	−7.519	−21.299	1.00	25.02	C
ATOM	3136	OH	TYR	A	525	33.890	−6.429	−20.636	1.00	25.02	O
ATOM	3137	H	TYR	A	525	34.122	−12.672	−22.337	1.00	0.00	H
ATOM	3138	HH	TYR	A	525	34.391	−6.685	−19.843	1.00	0.00	H

ATOM 3139 N LEU A 526 33.725 −11.244 −25.972 1.00 25.23 N

ATOM 3140 CA LEU A 526 34.641 −10.527 −26.855 1.00 25.24 C

ATOM 3141 C LEU A 526 34.562 −9.017 −26.599 1.00 25.29 C

ATOM 3142 O LEU A 526 33.475 −8.427 −26.583 1.00 25.42 O

ATOM 3143 CB LEU A 526 34.310 −10.854 −28.317 1.00 25.38 C

ATOM 3144 CG LEU A 526 34.385 −12.346 −28.675 1.00 25.42 C

ATOM 3145 CD1 LEU A 526 33.882 −12.540 −30.102 1.00 25.52 C

ATOM 3146 CD2 LEU A 526 35.795 −12.909 −28.552 1.00 25.28 C

ATOM 3147 H LEU A 526 32.838 −11.545 −26.358 1.00 0.00 H

ATOM 3148 N ASN A 527 35.718 −8.384 −26.423 1.00 25.20 N

ATOM 3149 CA ASN A 527 35.811 −6.960 −26.107 1.00 25.28 C

ATOM 3150 C ASN A 527 35.888 −6.051 −27.351 1.00 25.40 C

ATOM 3151 O ASN A 527 35.729 −4.837 −27.228 1.00 25.38 O

ATOM 3152 CB ASN A 527 37.015 −6.773 −25.184 1.00 25.17 C

ATOM 3153 CG ASN A 527 37.027 −5.506 −24.359 1.00 25.29 C

ATOM 3154 OD1 ASN A 527 36.033 −4.807 −24.178 1.00 25.45 O

ATOM 3155 ND2 ASN A 527 38.176 −5.208 −23.802 1.00 25.21 N

ATOM 3156 H ASN A 527 36.569 −8.943 −26.392 1.00 0.00 H

ATOM 3157 1HD2 ASN A 527 38.968 −5.823 −23.975 1.00 0.00 H

ATOM 3158 2HD2 ASN A 527 38.264 −4.352 −23.293 1.00 0.00 H

ATOM 3159 N SER A 528 36.090 −6.606 −28.548 1.00 25.52 N

ATOM 3160 CA SER A 528 36.056 −5.843 −29.803 1.00 25.64 C

ATOM 3161 C SER A 528 34.652 −5.349 −30.168 1.00 25.78 C

ATOM 3162 O SER A 528 33.654 −6.021 −29.921 1.00 25.78 O

ATOM 3163 CB SER A 528 36.611 −6.699 −30.928 1.00 25.61 C

ATOM 3164 OG SER A 528 36.540 −6.004 −32.156 1.00 25.74 O

ATOM 3165 H SER A 528 36.260 −7.605 −28.598 1.00 0.00 H

ATOM	3166	HG	SER	A	528	37.181	−6.512	−32.724	1.00	0.00	H
ATOM	3167	N	ASN	A	529	34.546	−4.165	−30.783	1.00	25.91	N
ATOM	3168	CA	ASN	A	529	33.255	−3.555	−31.131	1.00	26.06	C
ATOM	3169	C	ASN	A	529	32.624	−4.069	−32.441	1.00	26.09	C
ATOM	3170	O	ASN	A	529	31.402	−3.958	−32.584	1.00	26.15	O
ATOM	3171	CB	ASN	A	529	33.392	−2.019	−31.126	1.00	26.20	C
ATOM	3172	CG	ASN	A	529	33.468	−1.402	−29.734	1.00	26.29	C
ATOM	3173	OD1	ASN	A	529	33.467	−2.061	−28.706	1.00	26.21	O
ATOM	3174	ND2	ASN	A	529	33.310	−0.103	−29.648	1.00	26.46	N
ATOM	3175	H	ASN	A	529	35.405	−3.686	−31.008	1.00	0.00	H
ATOM	3176	1HD2	ASN	A	529	33.211	0.461	−30.472	1.00	0.00	H
ATOM	3177	2HD2	ASN	A	529	33.266	0.283	−28.721	1.00	0.00	H
ATOM	3178	N	GLN	A	530	33.425	−4.572	−33.390	1.00	26.08	N
ATOM	3179	CA	GLN	A	530	33.011	−4.841	−34.785	1.00	26.12	C
ATOM	3180	C	GLN	A	530	33.590	−6.134	−35.397	1.00	26.05	C
ATOM	3181	O	GLN	A	530	33.304	−6.448	−36.555	1.00	26.11	O
ATOM	3182	CB	GLN	A	530	33.422	−3.654	−35.676	1.00	26.24	C
ATOM	3183	CG	GLN	A	530	32.649	−2.357	−35.396	1.00	26.35	C
ATOM	3184	CD	GLN	A	530	32.961	−1.259	−36.412	1.00	26.46	C
ATOM	3185	OE1	GLN	A	530	32.090	−0.560	−36.897	1.00	26.52	O
ATOM	3186	NE2	GLN	A	530	34.198	−1.089	−36.828	1.00	26.49	N
ATOM	3187	H	GLN	A	530	34.408	−4.677	−33.168	1.00	0.00	H
ATOM	3188	1HE2	GLN	A	530	34.936	−1.695	−36.513	1.00	0.00	H
ATOM	3189	2HE2	GLN	A	530	34.333	−0.395	−37.540	1.00	0.00	H
ATOM	3190	N	THR	A	531	34.497	−6.803	−34.690	1.00	36.53	N
ATOM	3191	CA	THR	A	531	35.325	−7.900	−35.208	1.00	36.48	C
ATOM	3192	C	THR	A	531	35.672	−8.890	−34.083	1.00	36.37	C

ATOM 3193 O THR A 531 35.068 −8.845 −33.010 1.00 36.28 O

ATOM 3194 CB THR A 531 36.576 −7.358 −35.940 1.00 36.50 C

ATOM 3195 OG1 THR A 531 37.125 −6.235 −35.296 1.00 36.47 O

ATOM 3196 CG2 THR A 531 36.315 −6.944 −37.386 1.00 36.63 C

ATOM 3197 H THR A 531 34.739 −6.488 −33.762 1.00 0.00 H

ATOM 3198 HG1 THR A 531 37.873 −6.609 −34.778 1.00 0.00 H

ATOM 3199 N ALA A 532 36.316 −9.995 −34.460 1.00 25.79 N

ATOM 3200 CA ALA A 532 36.389 −11.229 −33.680 1.00 25.70 C

ATOM 3201 C ALA A 532 37.644 −11.292 −32.784 1.00 25.59 C

ATOM 3202 O ALA A 532 38.484 −12.167 −32.978 1.00 25.61 O

ATOM 3203 CB ALA A 532 36.271 −12.399 −34.674 1.00 25.80 C

ATOM 3204 H ALA A 532 36.841 −9.969 −35.319 1.00 0.00 H

ATOM 3205 N GLU A 533 37.823 −10.332 −31.868 1.00 25.49 N

ATOM 3206 CA GLU A 533 39.034 −10.212 −31.031 1.00 25.38 C

ATOM 3207 C GLU A 533 38.749 −10.050 −29.522 1.00 25.24 C

ATOM 3208 O GLU A 533 37.687 −9.559 −29.118 1.00 25.25 O

ATOM 3209 CB GLU A 533 39.954 −9.064 −31.506 1.00 25.43 C

ATOM 3210 CG GLU A 533 40.225 −8.983 −33.021 1.00 25.56 C

ATOM 3211 CD GLU A 533 39.280 −8.040 −33.783 1.00 25.58 C

ATOM 3212 OE1 GLU A 533 39.478 −7.827 −34.996 1.00 25.70 O

ATOM 3213 OE2 GLU A 533 38.242 −7.589 −33.243 1.00 25.50 O

ATOM 3214 H GLU A 533 37.152 −9.578 −31.829 1.00 0.00 H

ATOM 3215 N ASN A 534 39.808 −10.259 −28.724 1.00 25.12 N

ATOM 3216 CA ASN A 534 39.939 −9.916 −27.298 1.00 24.99 C

ATOM 3217 C ASN A 534 38.999 −10.724 −26.362 1.00 24.90 C

ATOM 3218 O ASN A 534 37.965 −10.212 −25.916 1.00 24.87 O

ATOM 3219 CB ASN A 534 39.848 −8.384 −27.157 1.00 25.07 C

ATOM	3220	CG	ASN	A	534	40.426	−7.858	−25.851	1.00	24.96	C
ATOM	3221	OD1	ASN	A	534	39.806	−7.889	−24.800	1.00	24.83	O
ATOM	3222	ND2	ASN	A	534	41.492	−7.110	−25.944	1.00	25.03	N
ATOM	3223	H	ASN	A	534	40.632	−10.655	−29.167	1.00	0.00	H
ATOM	3224	1HD2	ASN	A	534	42.014	−7.151	−26.814	1.00	0.00	H
ATOM	3225	2HD2	ASN	A	534	42.019	−6.945	−25.079	1.00	0.00	H
ATOM	3226	N	PRO	A	535	39.288	−12.022	−26.119	1.00	24.87	N
ATOM	3227	CA	PRO	A	535	38.470	−12.911	−25.288	1.00	24.80	C
ATOM	3228	C	PRO	A	535	38.708	−12.694	−23.780	1.00	24.63	C
ATOM	3229	O	PRO	A	535	39.639	−13.249	−23.187	1.00	24.51	O
ATOM	3230	CB	PRO	A	535	38.854	−14.324	−25.746	1.00	24.82	C
ATOM	3231	CG	PRO	A	535	40.320	−14.183	−26.162	1.00	24.83	C
ATOM	3232	CD	PRO	A	535	40.396	−12.762	−26.716	1.00	24.92	C
ATOM	3233	N	VAL	A	536	37.755	−12.043	−23.107	1.00	24.61	N
ATOM	3234	CA	VAL	A	536	37.817	−11.748	−21.663	1.00	24.46	C
ATOM	3235	C	VAL	A	536	36.796	−12.574	−20.883	1.00	24.45	C
ATOM	3236	O	VAL	A	536	35.596	−12.459	−21.121	1.00	24.60	O
ATOM	3237	CB	VAL	A	536	37.629	−10.237	−21.417	1.00	24.50	C
ATOM	3238	CG1	VAL	A	536	37.601	−9.885	−19.925	1.00	24.36	C
ATOM	3239	CG2	VAL	A	536	38.771	−9.436	−22.052	1.00	24.55	C
ATOM	3240	H	VAL	A	536	36.988	−11.634	−23.636	1.00	0.00	H
ATOM	3241	N	PHE	A	537	37.233	−13.305	−19.852	1.00	24.29	N
ATOM	3242	CA	PHE	A	537	36.330	−13.835	−18.824	1.00	24.26	C
ATOM	3243	C	PHE	A	537	35.964	−12.719	−17.835	1.00	24.26	C
ATOM	3244	O	PHE	A	537	36.842	−12.129	−17.201	1.00	24.16	O
ATOM	3245	CB	PHE	A	537	36.958	−15.051	−18.122	1.00	24.07	C
ATOM	3246	CG	PHE	A	537	35.997	−15.842	−17.241	1.00	24.06	C

ATOM 3247 CD1 PHE A 537 35.513 −17.097 −17.663 1.00 24.21 C

ATOM 3248 CD2 PHE A 537 35.586 −15.337 −15.992 1.00 23.92 C

ATOM 3249 CE1 PHE A 537 34.633 −17.833 −16.848 1.00 24.24 C

ATOM 3250 CE2 PHE A 537 34.703 −16.071 −15.179 1.00 23.93 C

ATOM 3251 CZ PHE A 537 34.227 −17.322 −15.606 1.00 24.09 C

ATOM 3252 H PHE A 537 38.221 −13.255 −19.621 1.00 0.00 H

ATOM 3253 N ALA A 538 34.671 −12.507 −17.602 1.00 24.40 N

ATOM 3254 CA ALA A 538 34.141 −11.521 −16.666 1.00 24.47 C

ATOM 3255 C ALA A 538 33.212 −12.164 −15.628 1.00 24.47 C

ATOM 3256 O ALA A 538 32.470 −13.099 −15.933 1.00 24.57 O

ATOM 3257 CB ALA A 538 33.431 −10.421 −17.464 1.00 24.69 C

ATOM 3258 H ALA A 538 33.994 −13.052 −18.129 1.00 0.00 H

ATOM 3259 N VAL A 539 33.139 −11.547 −14.447 1.00 24.36 N

ATOM 3260 CA VAL A 539 32.083 −11.777 −13.449 1.00 24.36 C

ATOM 3261 C VAL A 539 31.351 −10.462 −13.197 1.00 24.52 C

ATOM 3262 O VAL A 539 31.989 −9.424 −13.007 1.00 24.48 O

ATOM 3263 CB VAL A 539 32.650 −12.377 −12.151 1.00 24.11 C

ATOM 3264 CG1 VAL A 539 31.546 −12.621 −11.119 1.00 24.11 C

ATOM 3265 CG2 VAL A 539 33.333 −13.723 −12.418 1.00 23.95 C

ATOM 3266 H VAL A 539 33.774 −10.772 −14.276 1.00 0.00 H

ATOM 3267 N PHE A 540 30.023 −10.468 −13.299 1.00 24.72 N

ATOM 3268 CA PHE A 540 29.228 −9.250 −13.457 1.00 24.94 C

ATOM 3269 C PHE A 540 27.805 −9.372 −12.899 1.00 25.14 C

ATOM 3270 O PHE A 540 27.212 −10.445 −12.858 1.00 25.11 O

ATOM 3271 CB PHE A 540 29.207 −8.850 −14.944 1.00 25.07 C

ATOM 3272 CG PHE A 540 28.480 −9.814 −15.865 1.00 25.16 C

ATOM 3273 CD1 PHE A 540 27.113 −9.623 −16.147 1.00 25.41 C

ATOM 3274 CD2 PHE A 540 29.167 −10.898 −16.447 1.00 25.03 C
ATOM 3275 CE1 PHE A 540 26.438 −10.507 −17.008 1.00 25.50 C
ATOM 3276 CE2 PHE A 540 28.491 −11.785 −17.303 1.00 25.14 C
ATOM 3277 CZ PHE A 540 27.127 −11.588 −17.585 1.00 25.37 C
ATOM 3278 H PHE A 540 29.551 −11.355 −13.465 1.00 0.00 H
ATOM 3279 N LYS A 541 27.240 −8.220 −12.553 1.00 25.36 N
ATOM 3280 CA LYS A 541 25.839 −7.947 −12.214 1.00 25.60 C
ATOM 3281 C LYS A 541 25.263 −7.008 −13.290 1.00 25.85 C
ATOM 3282 O LYS A 541 26.030 −6.417 −14.050 1.00 25.83 O
ATOM 3283 CB LYS A 541 25.860 −7.346 −10.797 1.00 25.60 C
ATOM 3284 CG LYS A 541 24.499 −7.194 −10.108 1.00 25.88 C
ATOM 3285 CD LYS A 541 24.577 −6.475 −8.746 1.00 25.86 C
ATOM 3286 CE LYS A 541 25.443 −7.150 −7.667 1.00 25.60 C
ATOM 3287 NZ LYS A 541 26.884 −6.802 −7.778 1.00 25.30 N1+
ATOM 3288 H LYS A 541 27.814 −7.392 −12.688 1.00 0.00 H
ATOM 3289 HZ1 LYS A 541 27.421 −7.108 −6.980 1.00 0.00 H
ATOM 3290 HZ2 LYS A 541 27.036 −5.788 −7.866 1.00 0.00 H
ATOM 3291 HZ3 LYS A 541 27.294 −7.172 −8.620 1.00 0.00 H
ATOM 3292 N ASP A 542 23.944 −6.818 −13.330 1.00 36.37 N
ATOM 3293 CA ASP A 542 23.222 −6.048 −14.366 1.00 36.63 C
ATOM 3294 C ASP A 542 23.896 −4.734 −14.809 1.00 36.65 C
ATOM 3295 O ASP A 542 24.173 −4.557 −15.994 1.00 36.70 O
ATOM 3296 CB ASP A 542 21.796 −5.737 −13.875 1.00 36.90 C
ATOM 3297 CG ASP A 542 20.981 −6.974 −13.498 1.00 36.90 C
ATOM 3298 OD1 ASP A 542 21.248 −8.068 −14.038 1.00 36.81 O
ATOM 3299 OD2 ASP A 542 20.002 −6.837 −12.738 1.00 36.99 O
ATOM 3300 H ASP A 542 23.380 −7.414 −12.740 1.00 0.00 H

ATOM	3301	N	ASN	A	543	24.298	−3.885	−13.860	1.00	26.35	N
ATOM	3302	CA	ASN	A	543	24.885	−2.570	−14.147	1.00	26.44	C
ATOM	3303	C	ASN	A	543	26.396	−2.501	−13.831	1.00	26.21	C
ATOM	3304	O	ASN	A	543	26.945	−1.414	−13.664	1.00	26.30	O
ATOM	3305	CB	ASN	A	543	24.076	−1.470	−13.425	1.00	26.73	C
ATOM	3306	CG	ASN	A	543	22.606	−1.368	−13.813	1.00	27.00	C
ATOM	3307	OD1	ASN	A	543	21.953	−2.294	−14.255	1.00	26.98	O
ATOM	3308	ND2	ASN	A	543	21.981	−0.257	−13.505	1.00	27.26	N
ATOM	3309	H	ASN	A	543	24.028	−4.088	−12.912	1.00	0.00	H
ATOM	3310	1HD2	ASN	A	543	22.481	0.518	−13.116	1.00	0.00	H
ATOM	3311	2HD2	ASN	A	543	21.007	−0.232	−13.745	1.00	0.00	H
ATOM	3312	N	GLU	A	544	27.044	−3.641	−13.567	1.00	25.94	N
ATOM	3313	CA	GLU	A	544	28.320	−3.684	−12.845	1.00	25.71	C
ATOM	3314	C	GLU	A	544	29.136	−4.944	−13.186	1.00	25.44	C
ATOM	3315	O	GLU	A	544	28.926	−6.011	−12.610	1.00	25.36	O
ATOM	3316	CB	GLU	A	544	28.009	−3.592	−11.337	1.00	25.69	C
ATOM	3317	CG	GLU	A	544	29.250	−3.611	−10.429	1.00	25.43	C
ATOM	3318	CD	GLU	A	544	28.901	−3.792	−8.942	1.00	25.50	C
ATOM	3319	OE1	GLU	A	544	27.930	−4.535	−8.646	1.00	25.63	O
ATOM	3320	OE2	GLU	A	544	29.795	−3.486	−8.126	1.00	25.44	O
ATOM	3321	H	GLU	A	544	26.555	−4.518	−13.728	1.00	0.00	H
ATOM	3322	N	ILE	A	545	30.198	−4.801	−13.985	1.00	25.30	N
ATOM	3323	CA	ILE	A	545	31.266	−5.814	−14.034	1.00	25.04	C
ATOM	3324	C	ILE	A	545	32.076	−5.717	−12.738	1.00	24.84	C
ATOM	3325	O	ILE	A	545	32.664	−4.669	−12.454	1.00	24.84	O
ATOM	3326	CB	ILE	A	545	32.156	−5.677	−15.287	1.00	25.00	C
ATOM	3327	CG1	ILE	A	545	31.302	−5.785	−16.568	1.00	25.14	C

ATOM 3328 CG2 ILE A 545 33.254 −6.755 −15.266 1.00 24.73 C
ATOM 3329 CD1 ILE A 545 32.078 −5.541 −17.866 1.00 25.16 C
ATOM 3330 H ILE A 545 30.361 −3.908 −14.423 1.00 0.00 H
ATOM 3331 N LEU A 546 32.133 −6.804 −11.970 1.00 24.68 N
ATOM 3332 CA LEU A 546 32.840 −6.872 −10.693 1.00 24.48 C
ATOM 3333 C LEU A 546 34.351 −7.012 −10.895 1.00 24.28 C
ATOM 3334 O LEU A 546 35.111 −6.214 −10.352 1.00 24.26 O
ATOM 3335 CB LEU A 546 32.286 −8.039 −9.850 1.00 24.36 C
ATOM 3336 CG LEU A 546 30.801 −7.923 −9.469 1.00 24.55 C
ATOM 3337 CD1 LEU A 546 30.341 −9.201 −8.769 1.00 24.40 C
ATOM 3338 CD2 LEU A 546 30.583 −6.765 −8.503 1.00 24.77 C
ATOM 3339 H LEU A 546 31.714 −7.659 −12.319 1.00 0.00 H
ATOM 3340 N TYR A 547 34.778 −7.973 −11.715 1.00 24.15 N
ATOM 3341 CA TYR A 547 36.187 −8.288 −11.978 1.00 23.98 C
ATOM 3342 C TYR A 547 36.325 −9.150 −13.250 1.00 24.01 C
ATOM 3343 O TYR A 547 35.342 −9.718 −13.734 1.00 24.08 O
ATOM 3344 CB TYR A 547 36.816 −8.955 −10.734 1.00 23.71 C
ATOM 3345 CG TYR A 547 35.981 −10.021 −10.046 1.00 23.64 C
ATOM 3346 CD1 TYR A 547 36.069 −11.354 −10.476 1.00 23.49 C
ATOM 3347 CD2 TYR A 547 35.151 −9.691 −8.956 1.00 23.75 C
ATOM 3348 CE1 TYR A 547 35.326 −12.359 −9.829 1.00 23.44 C
ATOM 3349 CE2 TYR A 547 34.375 −10.683 −8.329 1.00 23.71 C
ATOM 3350 CZ TYR A 547 34.464 −12.022 −8.763 1.00 23.55 C
ATOM 3351 OH TYR A 547 33.758 −12.990 −8.126 1.00 23.53 O
ATOM 3352 H TYR A 547 34.087 −8.579 −12.143 1.00 0.00 H
ATOM 3353 HH TYR A 547 33.155 −12.609 −7.463 1.00 0.00 H
ATOM 3354 N GLN A 548 37.521 −9.172 −13.848 1.00 37.99 N

ATOM 3355 CA GLN A 548 37.803 −9.771 −15.164 1.00 38.04 C
ATOM 3356 C GLN A 548 39.182 −10.448 −15.201 1.00 37.85 C
ATOM 3357 O GLN A 548 40.060 −10.084 −14.419 1.00 37.67 O
ATOM 3358 CB GLN A 548 37.769 −8.695 −16.268 1.00 38.24 C
ATOM 3359 CG GLN A 548 36.420 −7.986 −16.448 1.00 38.45 C
ATOM 3360 CD GLN A 548 36.324 −7.177 −17.745 1.00 38.64 C
ATOM 3361 OE1 GLN A 548 35.285 −7.105 −18.378 1.00 38.81 O
ATOM 3362 NE2 GLN A 548 37.386 −6.568 −18.228 1.00 38.63 N
ATOM 3363 H GLN A 548 38.307 −8.741 −13.381 1.00 0.00 H
ATOM 3364 1HE2 GLN A 548 38.290 −6.686 −17.807 1.00 0.00 H
ATOM 3365 2HE2 GLN A 548 37.279 −6.137 −19.132 1.00 0.00 H
ATOM 3366 N VAL A 549 39.417 −11.281 −16.218 1.00 23.90 N
ATOM 3367 CA VAL A 549 40.751 −11.695 −16.682 1.00 23.78 C
ATOM 3368 C VAL A 549 40.706 −12.018 −18.191 1.00 23.92 C
ATOM 3369 O VAL A 549 39.733 −12.636 −18.636 1.00 24.02 O
ATOM 3370 CB VAL A 549 41.277 −12.878 −15.835 1.00 23.57 C
ATOM 3371 CG1 VAL A 549 40.557 −14.206 −16.109 1.00 23.62 C
ATOM 3372 CG2 VAL A 549 42.782 −13.096 −16.004 1.00 23.45 C
ATOM 3373 H VAL A 549 38.631 −11.555 −16.804 1.00 0.00 H
ATOM 3374 N PRO A 550 41.671 −11.567 −19.015 1.00 47.09 N
ATOM 3375 CA PRO A 550 41.814 −12.059 −20.388 1.00 47.23 C
ATOM 3376 C PRO A 550 42.198 −13.549 −20.396 1.00 47.14 C
ATOM 3377 O PRO A 550 42.815 −14.042 −19.449 1.00 47.00 O
ATOM 3378 CB PRO A 550 42.892 −11.179 −21.026 1.00 47.30 C
ATOM 3379 CG PRO A 550 43.774 −10.790 −19.843 1.00 47.15 C
ATOM 3380 CD PRO A 550 42.778 −10.674 −18.689 1.00 47.02 C
ATOM 3381 N LEU A 551 41.780 −14.292 −21.424 1.00 45.69 N

ATOM	3382	CA	LEU	A	551	42.152	−15.708	−21.600	1.00	45.66	C
ATOM	3383	C	LEU	A	551	43.313	−15.930	−22.584	1.00	45.74	C
ATOM	3384	O	LEU	A	551	43.915	−17.003	−22.566	1.00	45.70	O
ATOM	3385	CB	LEU	A	551	40.908	−16.515	−21.998	1.00	45.79	C
ATOM	3386	CG	LEU	A	551	39.814	−16.563	−20.916	1.00	45.70	C
ATOM	3387	CD1	LEU	A	551	38.662	−17.422	−21.430	1.00	45.87	C
ATOM	3388	CD2	LEU	A	551	40.292	−17.178	−19.594	1.00	45.57	C
ATOM	3389	H	LEU	A	551	41.217	−13.848	−22.145	1.00	0.00	H
ATOM	3390	N	ALA	A	552	43.634	−14.908	−23.375	1.00	24.27	N
ATOM	3391	CA	ALA	A	552	44.817	−14.755	−24.218	1.00	24.37	C
ATOM	3392	C	ALA	A	552	44.986	−13.252	−24.541	1.00	24.41	C
ATOM	3393	O	ALA	A	552	44.158	−12.438	−24.119	1.00	24.35	O
ATOM	3394	CB	ALA	A	552	44.644	−15.607	−25.482	1.00	24.56	C
ATOM	3395	H	ALA	A	552	43.098	−14.058	−23.282	1.00	0.00	H
ATOM	3396	N	GLU	A	553	46.078	−12.879	−25.203	1.00	35.47	N
ATOM	3397	CA	GLU	A	553	46.497	−11.494	−25.452	1.00	35.58	C
ATOM	3398	C	GLU	A	553	45.467	−10.642	−26.232	1.00	35.70	C
ATOM	3399	O	GLU	A	553	44.558	−11.151	−26.893	1.00	35.71	O
ATOM	3400	CB	GLU	A	553	47.876	−11.500	−26.146	1.00	35.73	C
ATOM	3401	CG	GLU	A	553	49.066	−11.778	−25.203	1.00	35.68	C
ATOM	3402	CD	GLU	A	553	49.009	−13.103	−24.417	1.00	35.83	C
ATOM	3403	OE1	GLU	A	553	48.527	−14.117	−24.974	1.00	35.88	O
ATOM	3404	OE2	GLU	A	553	49.403	−13.083	−23.228	1.00	35.90	O
ATOM	3405	H	GLU	A	553	46.786	−13.593	−25.380	1.00	0.00	H
ATOM	3406	N	ASP	A	554	45.566	−9.315	−26.093	1.00	27.42	N
ATOM	3407	CA	ASP	A	554	44.496	−8.365	−26.440	1.00	27.55	C
ATOM	3408	C	ASP	A	554	44.093	−8.301	−27.930	1.00	27.68	C

ATOM 3409 O ASP A 554 42.989 −7.836 −28.225 1.00 27.74 O

ATOM 3410 CB ASP A 554 44.880 −6.958 −25.936 1.00 27.65 C

ATOM 3411 CG ASP A 554 44.638 −6.757 −24.434 1.00 27.56 C

ATOM 3412 OD1 ASP A 554 43.468 −6.950 −24.023 1.00 27.40 O

ATOM 3413 OD2 ASP A 554 45.489 −6.093 −23.804 1.00 27.67 O

ATOM 3414 H ASP A 554 46.338 −8.953 −25.555 1.00 0.00 H

ATOM 3415 N ASP A 555 44.924 −8.767 −28.865 1.00 27.90 N

ATOM 3416 CA ASP A 555 44.605 −8.863 −30.299 1.00 28.05 C

ATOM 3417 C ASP A 555 44.078 −10.250 −30.725 1.00 28.03 C

ATOM 3418 O ASP A 555 43.693 −10.434 −31.881 1.00 28.16 O

ATOM 3419 CB ASP A 555 45.842 −8.477 −31.135 1.00 28.16 C

ATOM 3420 CG ASP A 555 47.061 −9.401 −30.975 1.00 28.10 C

ATOM 3421 OD1 ASP A 555 47.123 −10.148 −29.970 1.00 27.96 O

ATOM 3422 OD2 ASP A 555 47.971 −9.284 −31.826 1.00 28.20 O

ATOM 3423 H ASP A 555 45.802 −9.192 −28.581 1.00 0.00 H

ATOM 3424 N THR A 556 44.065 −11.241 −29.827 1.00 25.17 N

ATOM 3425 CA THR A 556 43.853 −12.647 −30.208 1.00 25.16 C

ATOM 3426 C THR A 556 42.453 −12.929 −30.762 1.00 25.25 C

ATOM 3427 O THR A 556 41.425 −12.483 −30.239 1.00 25.21 O

ATOM 3428 CB THR A 556 44.199 −13.636 −29.086 1.00 24.98 C

ATOM 3429 OG1 THR A 556 43.480 −13.376 −27.903 1.00 24.90 O

ATOM 3430 CG2 THR A 556 45.696 −13.631 −28.785 1.00 24.89 C

ATOM 3431 H THR A 556 44.424 −11.039 −28.901 1.00 0.00 H

ATOM 3432 HG1 THR A 556 43.869 −12.569 −27.509 1.00 0.00 H

ATOM 3433 N ASN A 557 42.428 −13.715 −31.844 1.00 25.38 N

ATOM 3434 CA ASN A 557 41.236 −14.024 −32.630 1.00 25.47 C

ATOM 3435 C ASN A 557 40.294 −14.957 −31.851 1.00 25.41 C

```
ATOM   3436  O   ASN A 557      40.629 −16.120 −31.625  1.00 25.38           O
ATOM   3437  CB  ASN A 557      41.617 −14.676 −33.982  1.00 25.65           C
ATOM   3438  CG  ASN A 557      42.867 −14.140 −34.657  1.00 25.72           C
ATOM   3439  OD1 ASN A 557      42.810 −13.580 −35.745  1.00 25.68           O
ATOM   3440  ND2 ASN A 557      44.016 −14.604 −34.222  1.00 25.86           N
ATOM   3441  H   ASN A 557      43.313 −14.023 −32.213  1.00  0.00           H
ATOM   3442 1HD2 ASN A 557      44.017 −15.343 −33.524  1.00  0.00           H
ATOM   3443 2HD2 ASN A 557      44.873 −14.262 −34.637  1.00  0.00           H
ATOM   3444  N   ALA A 558      39.043 −14.557 −31.629  1.00 25.42           N
ATOM   3445  CA  ALA A 558      38.017 −15.404 −31.025  1.00 25.40           C
ATOM   3446  C   ALA A 558      36.608 −15.069 −31.541  1.00 25.54           C
ATOM   3447  O   ALA A 558      36.220 −13.909 −31.642  1.00 25.61           O
ATOM   3448  CB  ALA A 558      38.141 −15.320 −29.498  1.00 25.23           C
ATOM   3449  H   ALA A 558      38.783 −13.618 −31.919  1.00  0.00           H
ATOM   3450  N   GLN A 559      35.828 −16.103 −31.873  1.00 25.58           N
ATOM   3451  CA  GLN A 559      34.526 −15.982 −32.543  1.00 25.73           C
ATOM   3452  C   GLN A 559      33.333 −16.170 −31.593  1.00 25.74           C
ATOM   3453  O   GLN A 559      32.337 −15.453 −31.701  1.00 25.69           O
ATOM   3454  CB  GLN A 559      34.480 −16.985 −33.714  1.00 25.89           C
ATOM   3455  CG  GLN A 559      33.157 −16.936 −34.499  1.00 26.05           C
ATOM   3456  CD  GLN A 559      33.195 −17.799 −35.759  1.00 26.20           C
ATOM   3457  OE1 GLN A 559      33.313 −17.314 −36.873  1.00 26.18           O
ATOM   3458  NE2 GLN A 559      33.064 −19.101 −35.648  1.00 26.37           N
ATOM   3459  H   GLN A 559      36.214 −17.030 −31.724  1.00  0.00           H
ATOM   3460 1HE2 GLN A 559      33.000 −19.557 −34.731  1.00  0.00           H
ATOM   3461 2HE2 GLN A 559      33.138 −19.652 −36.479  1.00  0.00           H
ATOM   3462  N   LYS A 560      33.388 −17.173 −30.710  1.00 25.84           N
```

ATOM 3463 CA LYS A 560 32.240 −17.681 −29.938 1.00 25.93 C

ATOM 3464 C LYS A 560 32.682 −18.168 −28.564 1.00 25.81 C

ATOM 3465 O LYS A 560 33.741 −18.776 −28.438 1.00 25.84 O

ATOM 3466 CB LYS A 560 31.547 −18.790 −30.760 1.00 26.15 C

ATOM 3467 CG LYS A 560 30.540 −19.664 −29.994 1.00 26.30 C

ATOM 3468 CD LYS A 560 29.769 −20.596 −30.941 1.00 26.56 C

ATOM 3469 CE LYS A 560 29.078 −21.752 −30.200 1.00 26.73 C

ATOM 3470 NZ LYS A 560 28.161 −21.295 −29.125 1.00 26.76 N1+

ATOM 3471 H LYS A 560 34.251 −17.700 −30.663 1.00 0.00 H

ATOM 3472 HZ1 LYS A 560 27.730 −22.091 −28.676 1.00 0.00 H

ATOM 3473 HZ2 LYS A 560 28.666 −20.774 −28.414 1.00 0.00 H

ATOM 3474 HZ3 LYS A 560 27.435 −20.693 −29.488 1.00 0.00 H

ATOM 3475 N THR A 561 31.786 −18.091 −27.583 1.00 25.67 N

ATOM 3476 CA THR A 561 32.006 −18.621 −26.226 1.00 25.51 C

ATOM 3477 C THR A 561 30.860 −19.538 −25.787 1.00 25.62 C

ATOM 3478 O THR A 561 29.787 −19.525 −26.393 1.00 25.77 O

ATOM 3479 CB THR A 561 32.270 −17.490 −25.211 1.00 25.34 C

ATOM 3480 OG1 THR A 561 31.074 −16.897 −24.764 1.00 25.43 O

ATOM 3481 CG2 THR A 561 33.141 −16.368 −25.789 1.00 25.28 C

ATOM 3482 H THR A 561 30.932 −17.574 −27.755 1.00 0.00 H

ATOM 3483 HG1 THR A 561 31.319 −16.152 −24.181 1.00 0.00 H

ATOM 3484 N ILE A 562 31.105 −20.429 −24.824 1.00 35.26 N

ATOM 3485 CA ILE A 562 30.079 −21.182 −24.076 1.00 35.34 C

ATOM 3486 C ILE A 562 30.595 −21.341 −22.644 1.00 35.14 C

ATOM 3487 O ILE A 562 31.689 −21.875 −22.481 1.00 34.97 O

ATOM 3488 CB ILE A 562 29.778 −22.581 −24.682 1.00 35.57 C

ATOM 3489 CG1 ILE A 562 29.423 −22.537 −26.186 1.00 35.76 C

ATOM 3490 CG2 ILE A 562 28.645 −23.257 −23.883 1.00 35.71 C
ATOM 3491 CD1 ILE A 562 29.248 −23.908 −26.846 1.00 36.03 C
ATOM 3492 H ILE A 562 32.060 −20.505 −24.483 1.00 0.00 H
ATOM 3493 N THR A 563 29.848 −20.909 −21.625 1.00 25.46 N
ATOM 3494 CA THR A 563 30.239 −21.043 −20.209 1.00 25.29 C
ATOM 3495 C THR A 563 29.170 −21.768 −19.396 1.00 25.43 C
ATOM 3496 O THR A 563 28.071 −21.240 −19.220 1.00 25.61 O
ATOM 3497 CB THR A 563 30.554 −19.680 −19.570 1.00 25.10 C
ATOM 3498 OG1 THR A 563 31.510 −18.986 −20.351 1.00 24.97 O
ATOM 3499 CG2 THR A 563 31.101 −19.840 −18.148 1.00 24.92 C
ATOM 3500 H THR A 563 28.953 −20.464 −21.827 1.00 0.00 H
ATOM 3501 HG1 THR A 563 31.009 −18.688 −21.123 1.00 0.00 H
ATOM 3502 N ASP A 564 29.554 −22.875 −18.757 1.00 25.36 N
ATOM 3503 CA ASP A 564 28.732 −23.626 −17.797 1.00 25.49 C
ATOM 3504 C ASP A 564 29.445 −23.727 −16.432 1.00 25.26 C
ATOM 3505 O ASP A 564 30.665 −23.899 −16.383 1.00 25.16 O
ATOM 3506 CB ASP A 564 28.341 −25.012 −18.352 1.00 25.74 C
ATOM 3507 CG ASP A 564 27.499 −25.017 −19.644 1.00 25.95 C
ATOM 3508 OD1 ASP A 564 26.931 −23.966 −20.022 1.00 26.05 O
ATOM 3509 OD2 ASP A 564 27.259 −26.122 −20.172 1.00 26.04 O
ATOM 3510 H ASP A 564 30.515 −23.185 −18.872 1.00 0.00 H
ATOM 3511 N CYS A 565 28.711 −23.600 −15.317 1.00 25.19 N
ATOM 3512 CA CYS A 565 29.288 −23.544 −13.963 1.00 24.91 C
ATOM 3513 C CYS A 565 28.628 −24.526 −12.979 1.00 24.97 C
ATOM 3514 O CYS A 565 27.448 −24.856 −13.087 1.00 25.23 O
ATOM 3515 CB CYS A 565 29.248 −22.110 −13.412 1.00 24.75 C
ATOM 3516 SG CYS A 565 29.929 −20.789 −14.458 1.00 24.74 S

```
ATOM   3517  H   CYS A 565      27.710 −23.484 −15.398  1.00  0.00           H
ATOM   3518  N   PHE A 566      29.408 −24.998 −12.005  1.00 24.71           N
ATOM   3519  CA  PHE A 566      29.070 −26.101 −11.101  1.00 24.74           C
ATOM   3520  C   PHE A 566      29.947 −26.077  −9.832  1.00 24.42           C
ATOM   3521  O   PHE A 566      30.757 −25.170  −9.634  1.00 24.18           O
ATOM   3522  CB  PHE A 566      29.207 −27.430 −11.875  1.00 24.88           C
ATOM   3523  CG  PHE A 566      30.536 −27.636 −12.581  1.00 24.74           C
ATOM   3524  CD1 PHE A 566      31.601 −28.282 −11.925  1.00 24.45           C
ATOM   3525  CD2 PHE A 566      30.700 −27.194 −13.909  1.00 24.89           C
ATOM   3526  CE1 PHE A 566      32.823 −28.480 −12.591  1.00 24.33           C
ATOM   3527  CE2 PHE A 566      31.923 −27.385 −14.572  1.00 24.78           C
ATOM   3528  CZ  PHE A 566      32.984 −28.030 −13.914  1.00 24.51           C
ATOM   3529  H   PHE A 566      30.362 −24.648 −11.955  1.00  0.00           H
ATOM   3530  N   LEU A 567      29.728 −27.028  −8.919  1.00 24.42           N
ATOM   3531  CA  LEU A 567      30.560 −27.230  −7.728  1.00 24.10           C
ATOM   3532  C   LEU A 567      31.562 −28.372  −7.941  1.00 24.02           C
ATOM   3533  O   LEU A 567      31.174 −29.437  −8.414  1.00 24.26           O
ATOM   3534  CB  LEU A 567      29.673 −27.522  −6.502  1.00 24.11           C
ATOM   3535  CG  LEU A 567      28.669 −26.417  −6.127  1.00 23.97           C
ATOM   3536  CD1 LEU A 567      27.853 −26.858  −4.912  1.00 24.06           C
ATOM   3537  CD2 LEU A 567      29.362 −25.101  −5.780  1.00 23.59           C
ATOM   3538  H   LEU A 567      29.099 −27.778  −9.164  1.00  0.00           H
ATOM   3539  N   LEU A 568      32.764 −28.232  −7.381  1.00 37.61           N
ATOM   3540  CA  LEU A 568      33.765 −29.298  −7.254  1.00 37.49           C
ATOM   3541  C   LEU A 568      34.573 −29.096  −5.958  1.00 37.18           C
ATOM   3542  O   LEU A 568      34.895 −27.959  −5.620  1.00 36.95           O
ATOM   3543  CB  LEU A 568      34.638 −29.307  −8.520  1.00 37.38           C
```

ATOM	3544	CG	LEU	A	568	35.660	−30.462	−8.538	1.00	37.30	C
ATOM	3545	CD1	LEU	A	568	35.408	−31.381	−9.734	1.00	37.65	C
ATOM	3546	CD2	LEU	A	568	37.081	−29.901	−8.594	1.00	37.12	C
ATOM	3547	H	LEU	A	568	33.019	−27.317	−7.019	1.00	0.00	H
ATOM	3548	N	GLU	A	569	34.601	−30.117	−5.091	1.00	54.42	N
ATOM	3549	CA	GLU	A	569	34.884	−29.982	−3.643	1.00	54.12	C
ATOM	3550	C	GLU	A	569	34.048	−28.886	−2.936	1.00	53.97	C
ATOM	3551	O	GLU	A	569	34.477	−28.274	−1.959	1.00	53.64	O
ATOM	3552	CB	GLU	A	569	36.395	−29.860	−3.355	1.00	53.83	C
ATOM	3553	CG	GLU	A	569	37.145	−31.197	−3.465	1.00	53.98	C
ATOM	3554	CD	GLU	A	569	38.189	−31.377	−2.348	1.00	53.70	C
ATOM	3555	OE1	GLU	A	569	38.820	−30.375	−1.927	1.00	53.38	O
ATOM	3556	OE2	GLU	A	569	38.680	−32.515	−2.173	1.00	53.81	O
ATOM	3557	H	GLU	A	569	34.370	−31.036	−5.440	1.00	0.00	H
ATOM	3558	N	ASN	A	570	32.802	−28.675	−3.380	1.00	45.28	N
ATOM	3559	CA	ASN	A	570	31.907	−27.593	−2.925	1.00	45.21	C
ATOM	3560	C	ASN	A	570	32.383	−26.165	−3.288	1.00	45.11	C
ATOM	3561	O	ASN	A	570	31.709	−25.190	−2.958	1.00	45.02	O
ATOM	3562	CB	ASN	A	570	31.563	−27.725	−1.424	1.00	44.92	C
ATOM	3563	CG	ASN	A	570	31.368	−29.150	−0.942	1.00	44.92	C
ATOM	3564	OD1	ASN	A	570	30.305	−29.729	−1.059	1.00	45.19	O
ATOM	3565	ND2	ASN	A	570	32.399	−29.763	−0.407	1.00	44.61	N
ATOM	3566	H	ASN	A	570	32.498	−29.235	−4.161	1.00	0.00	H
ATOM	3567	1HD2	ASN	A	570	33.303	−29.311	−0.454	1.00	0.00	H
ATOM	3568	2HD2	ASN	A	570	32.270	−30.719	−0.134	1.00	0.00	H
ATOM	3569	N	VAL	A	571	33.491	−26.020	−4.022	1.00	22.99	N
ATOM	3570	CA	VAL	A	571	33.969	−24.747	−4.578	1.00	22.91	C

ATOM	3571	C	VAL	A	571	33.324	−24.503	−5.943	1.00	23.18	C
ATOM	3572	O	VAL	A	571	33.290	−25.400	−6.783	1.00	23.36	O
ATOM	3573	CB	VAL	A	571	35.508	−24.732	−4.680	1.00	22.60	C
ATOM	3574	CG1	VAL	A	571	36.033	−23.389	−5.208	1.00	22.53	C
ATOM	3575	CG2	VAL	A	571	36.159	−24.977	−3.311	1.00	22.33	C
ATOM	3576	H	VAL	A	571	33.989	−26.857	−4.310	1.00	0.00	H
ATOM	3577	N	ILE	A	572	32.876	−23.270	−6.200	1.00	36.75	N
ATOM	3578	CA	ILE	A	572	32.316	−22.881	−7.499	1.00	37.02	C
ATOM	3579	C	ILE	A	572	33.428	−22.871	−8.564	1.00	36.99	C
ATOM	3580	O	ILE	A	572	34.371	−22.074	−8.495	1.00	36.81	O
ATOM	3581	CB	ILE	A	572	31.559	−21.530	−7.438	1.00	37.10	C
ATOM	3582	CG1	ILE	A	572	30.408	−21.464	−6.404	1.00	37.10	C
ATOM	3583	CG2	ILE	A	572	30.949	−21.216	−8.819	1.00	37.41	C
ATOM	3584	CD1	ILE	A	572	30.844	−21.176	−4.960	1.00	36.79	C
ATOM	3585	H	ILE	A	572	32.924	−22.578	−5.473	1.00	0.00	H
ATOM	3586	N	TRP	A	573	33.221	−23.646	−9.624	1.00	23.66	N
ATOM	3587	CA	TRP	A	573	34.004	−23.652	−10.859	1.00	23.67	C
ATOM	3588	C	TRP	A	573	33.111	−23.305	−12.051	1.00	23.94	C
ATOM	3589	O	TRP	A	573	31.931	−23.647	−12.076	1.00	24.17	O
ATOM	3590	CB	TRP	A	573	34.646	−25.033	−11.057	1.00	23.66	C
ATOM	3591	CG	TRP	A	573	35.935	−25.278	−10.333	1.00	23.37	C
ATOM	3592	CD1	TRP	A	573	36.061	−25.804	−9.094	1.00	23.16	C
ATOM	3593	CD2	TRP	A	573	37.299	−25.058	−10.811	1.00	23.23	C
ATOM	3594	NE1	TRP	A	573	37.400	−25.981	−8.797	1.00	22.91	N
ATOM	3595	CE2	TRP	A	573	38.209	−25.521	−9.814	1.00	22.95	C
ATOM	3596	CE3	TRP	A	573	37.861	−24.560	−12.008	1.00	23.33	C
ATOM	3597	CZ2	TRP	A	573	39.600	−25.486	−9.992	1.00	22.78	C

ATOM 3598 CZ3 TRP A 573 39.259 −24.502 −12.189 1.00 23.17 C
ATOM 3599 CH2 TRP A 573 40.127 −24.969 −11.188 1.00 22.90 C
ATOM 3600 H TRP A 573 32.381 −24.220 −9.628 1.00 0.00 H
ATOM 3601 HE1 TRP A 573 37.723 −26.535 −8.013 1.00 0.00 H
ATOM 3602 N CYS A 574 33.726 −22.774 −13.102 1.00 23.94 N
ATOM 3603 CA CYS A 574 33.159 −22.695 −14.442 1.00 24.19 C
ATOM 3604 C CYS A 574 34.091 −23.385 −15.442 1.00 24.21 C
ATOM 3605 O CYS A 574 35.315 −23.285 −15.323 1.00 24.06 O
ATOM 3606 CB CYS A 574 32.919 −21.229 −14.810 1.00 24.24 C
ATOM 3607 SG CYS A 574 31.775 −20.338 −13.723 1.00 24.36 S
ATOM 3608 H CYS A 574 34.693 −22.492 −13.002 1.00 0.00 H
ATOM 3609 N ILE A 575 33.514 −23.989 −16.478 1.00 24.44 N
ATOM 3610 CA ILE A 575 34.205 −24.331 −17.721 1.00 24.55 C
ATOM 3611 C ILE A 575 33.719 −23.359 −18.793 1.00 24.67 C
ATOM 3612 O ILE A 575 32.521 −23.297 −19.075 1.00 24.76 O
ATOM 3613 CB ILE A 575 33.947 −25.805 −18.112 1.00 24.79 C
ATOM 3614 CG1 ILE A 575 34.562 −26.794 −17.094 1.00 24.69 C
ATOM 3615 CG2 ILE A 575 34.447 −26.109 −19.538 1.00 25.03 C
ATOM 3616 CD1 ILE A 575 36.087 −26.936 −17.155 1.00 24.50 C
ATOM 3617 H ILE A 575 32.496 −23.990 −16.515 1.00 0.00 H
ATOM 3618 N SER A 576 34.655 −22.674 −19.445 1.00 24.68 N
ATOM 3619 CA SER A 576 34.400 −21.909 −20.662 1.00 24.85 C
ATOM 3620 C SER A 576 35.076 −22.576 −21.855 1.00 25.00 C
ATOM 3621 O SER A 576 36.304 −22.649 −21.925 1.00 24.96 O
ATOM 3622 CB SER A 576 34.860 −20.458 −20.526 1.00 24.71 C
ATOM 3623 OG SER A 576 34.121 −19.796 −19.517 1.00 24.50 O
ATOM 3624 H SER A 576 35.622 −22.790 −19.160 1.00 0.00 H

ATOM	3625	HG	SER	A	576	33.221	−19.612	−19.845	1.00	0.00	H
ATOM	3626	N	LEU	A	577	34.289	−22.959	−22.857	1.00	25.18	N
ATOM	3627	CA	LEU	A	577	34.793	−23.130	−24.215	1.00	25.31	C
ATOM	3628	C	LEU	A	577	34.934	−21.749	−24.864	1.00	25.22	C
ATOM	3629	O	LEU	A	577	33.976	−20.972	−24.865	1.00	25.02	O
ATOM	3630	CB	LEU	A	577	33.834	−24.034	−25.012	1.00	25.60	C
ATOM	3631	CG	LEU	A	577	34.391	−24.484	−26.375	1.00	25.77	C
ATOM	3632	CD1	LEU	A	577	35.487	−25.538	−26.207	1.00	25.78	C
ATOM	3633	CD2	LEU	A	577	33.282	−25.103	−27.221	1.00	26.05	C
ATOM	3634	H	LEU	A	577	33.295	−22.780	−22.749	1.00	0.00	H
ATOM	3635	N	VAL	A	578	36.043	−21.521	−25.564	1.00	25.36	N
ATOM	3636	CA	VAL	A	578	36.190	−20.403	−26.506	1.00	25.33	C
ATOM	3637	C	VAL	A	578	36.578	−20.959	−27.872	1.00	25.51	C
ATOM	3638	O	VAL	A	578	37.483	−21.783	−27.970	1.00	25.62	O
ATOM	3639	CB	VAL	A	578	37.206	−19.351	−26.013	1.00	25.10	C
ATOM	3640	CG1	VAL	A	578	37.117	−18.073	−26.856	1.00	25.13	C
ATOM	3641	CG2	VAL	A	578	36.976	−18.946	−24.551	1.00	24.92	C
ATOM	3642	H	VAL	A	578	36.812	−22.183	−25.504	1.00	0.00	H
ATOM	3643	N	GLU	A	579	35.851	−20.579	−28.918	1.00	25.56	N
ATOM	3644	CA	GLU	A	579	36.264	−20.747	−30.312	1.00	25.69	C
ATOM	3645	C	GLU	A	579	37.272	−19.642	−30.656	1.00	25.57	C
ATOM	3646	O	GLU	A	579	36.887	−18.483	−30.800	1.00	25.53	O
ATOM	3647	CB	GLU	A	579	35.017	−20.720	−31.210	1.00	25.90	C
ATOM	3648	CG	GLU	A	579	35.308	−21.117	−32.666	1.00	26.11	C
ATOM	3649	CD	GLU	A	579	34.017	−21.277	−33.487	1.00	26.31	C
ATOM	3650	OE1	GLU	A	579	33.056	−20.506	−33.261	1.00	26.33	O
ATOM	3651	OE2	GLU	A	579	33.985	−22.125	−34.408	1.00	26.46	O

ATOM	3652	H	GLU	A	579	35.124	−19.892	−28.746	1.00	0.00	H
ATOM	3653	N	ILE	A	580	38.559	−19.974	−30.550	1.00	25.51	N
ATOM	3654	CA	ILE	A	580	39.715	−19.063	−30.510	1.00	25.37	C
ATOM	3655	C	ILE	A	580	40.825	−19.616	−31.408	1.00	25.48	C
ATOM	3656	O	ILE	A	580	40.955	−20.835	−31.532	1.00	25.59	O
ATOM	3657	CB	ILE	A	580	40.192	−18.856	−29.043	1.00	25.15	C
ATOM	3658	CG1	ILE	A	580	41.369	−17.861	−28.894	1.00	25.01	C
ATOM	3659	CG2	ILE	A	580	40.575	−20.189	−28.380	1.00	25.13	C
ATOM	3660	CD1	ILE	A	580	41.759	−17.543	−27.444	1.00	24.80	C
ATOM	3661	H	ILE	A	580	38.792	−20.946	−30.416	1.00	0.00	H
ATOM	3662	N	TYR	A	581	41.666	−18.748	−31.962	1.00	25.49	N
ATOM	3663	CA	TYR	A	581	42.958	−19.143	−32.510	1.00	25.58	C
ATOM	3664	C	TYR	A	581	44.013	−18.052	−32.336	1.00	25.54	C
ATOM	3665	O	TYR	A	581	43.752	−16.851	−32.447	1.00	25.48	O
ATOM	3666	CB	TYR	A	581	42.828	−19.608	−33.962	1.00	25.82	C
ATOM	3667	CG	TYR	A	581	42.440	−18.546	−34.973	1.00	25.94	C
ATOM	3668	CD1	TYR	A	581	43.449	−17.841	−35.653	1.00	26.01	C
ATOM	3669	CD2	TYR	A	581	41.085	−18.255	−35.222	1.00	25.99	C
ATOM	3670	CE1	TYR	A	581	43.110	−16.807	−36.545	1.00	26.13	C
ATOM	3671	CE2	TYR	A	581	40.739	−17.229	−36.126	1.00	26.10	C
ATOM	3672	CZ	TYR	A	581	41.754	−16.488	−36.769	1.00	26.16	C
ATOM	3673	OH	TYR	A	581	41.434	−15.394	−37.503	1.00	26.27	O
ATOM	3674	H	TYR	A	581	41.513	−17.751	−31.821	1.00	0.00	H
ATOM	3675	HH	TYR	A	581	41.964	−14.639	−37.196	1.00	0.00	H
ATOM	3676	N	ASP	A	582	45.228	−18.505	−32.080	1.00	27.79	N
ATOM	3677	CA	ASP	A	582	46.408	−17.666	−31.940	1.00	27.78	C
ATOM	3678	C	ASP	A	582	46.944	−17.392	−33.359	1.00	28.00	C

ATOM 3679 O ASP A 582 46.770 −18.225 −34.254 1.00 28.14 O

ATOM 3680 CB ASP A 582 47.445 −18.347 −31.016 1.00 27.66 C

ATOM 3681 CG ASP A 582 46.905 −18.974 −29.705 1.00 27.49 C

ATOM 3682 OD1 ASP A 582 45.723 −18.749 −29.346 1.00 27.55 O

ATOM 3683 OD2 ASP A 582 47.460 −20.027 −29.312 1.00 27.31 O

ATOM 3684 H ASP A 582 45.379 −19.510 −32.128 1.00 0.00 H

ATOM 3685 N THR A 583 47.504 −16.206 −33.626 1.00 25.85 N

ATOM 3686 CA THR A 583 47.854 −15.758 −34.995 1.00 26.06 C

ATOM 3687 C THR A 583 49.079 −16.505 −35.557 1.00 26.14 C

ATOM 3688 O THR A 583 50.195 −15.993 −35.595 1.00 26.04 O

ATOM 3689 CB THR A 583 47.993 −14.225 −35.054 1.00 26.10 C

ATOM 3690 OG1 THR A 583 46.803 −13.651 −34.555 1.00 26.02 O

ATOM 3691 CG2 THR A 583 48.146 −13.683 −36.477 1.00 26.32 C

ATOM 3692 H THR A 583 47.551 −15.520 −32.887 1.00 0.00 H

ATOM 3693 HG1 THR A 583 47.004 −12.751 −34.266 1.00 0.00 H

ATOM 3694 N GLY A 584 48.871 −17.784 −35.880 1.00 35.45 N

ATOM 3695 CA GLY A 584 49.892 −18.808 −36.121 1.00 35.55 C

ATOM 3696 C GLY A 584 49.375 −20.258 −36.022 1.00 35.59 C

ATOM 3697 O GLY A 584 50.027 −21.162 −36.540 1.00 35.78 O

ATOM 3698 H GLY A 584 47.930 −18.116 −35.706 1.00 0.00 H

ATOM 3699 N ASP A 585 48.167 −20.490 −35.487 1.00 26.30 N

ATOM 3700 CA ASP A 585 47.480 −21.792 −35.520 1.00 26.32 C

ATOM 3701 C ASP A 585 47.143 −22.237 −36.962 1.00 26.58 C

ATOM 3702 O ASP A 585 46.094 −21.890 −37.506 1.00 26.67 O

ATOM 3703 CB ASP A 585 46.180 −21.726 −34.689 1.00 26.11 C

ATOM 3704 CG ASP A 585 46.355 −21.843 −33.173 1.00 25.87 C

ATOM 3705 OD1 ASP A 585 47.051 −22.799 −32.763 1.00 25.85 O

ATOM 3706 OD2 ASP A 585 45.465 −21.325 −32.459 1.00 25.71 O
ATOM 3707 H ASP A 585 47.670 −19.725 −35.044 1.00 0.00 H
ATOM 3708 N SER A 586 47.909 −23.191 −37.506 1.00 35.84 N
ATOM 3709 CA SER A 586 47.547 −23.910 −38.747 1.00 36.14 C
ATOM 3710 C SER A 586 46.276 −24.760 −38.596 1.00 36.23 C
ATOM 3711 O SER A 586 45.555 −24.997 −39.567 1.00 36.41 O
ATOM 3712 CB SER A 586 48.679 −24.854 −39.168 1.00 36.26 C
ATOM 3713 OG SER A 586 49.908 −24.161 −39.273 1.00 36.32 O
ATOM 3714 H SER A 586 48.842 −23.308 −37.135 1.00 0.00 H
ATOM 3715 HG SER A 586 50.606 −24.788 −39.475 1.00 0.00 H
ATOM 3716 N VAL A 587 46.025 −25.245 −37.374 1.00 42.69 N
ATOM 3717 CA VAL A 587 44.874 −26.073 −36.992 1.00 42.75 C
ATOM 3718 C VAL A 587 44.000 −25.266 −36.030 1.00 42.46 C
ATOM 3719 O VAL A 587 44.064 −25.443 −34.816 1.00 42.23 O
ATOM 3720 CB VAL A 587 45.325 −27.433 −36.411 1.00 42.92 C
ATOM 3721 CG1 VAL A 587 44.134 −28.338 −36.051 1.00 42.99 C
ATOM 3722 CG2 VAL A 587 46.196 −28.214 −37.405 1.00 43.25 C
ATOM 3723 H VAL A 587 46.630 −24.924 −36.632 1.00 0.00 H
ATOM 3724 N ILE A 588 43.249 −24.310 −36.576 1.00 42.73 N
ATOM 3725 CA ILE A 588 42.336 −23.437 −35.825 1.00 42.49 C
ATOM 3726 C ILE A 588 41.344 −24.305 −35.043 1.00 42.51 C
ATOM 3727 O ILE A 588 40.645 −25.134 −35.634 1.00 42.72 O
ATOM 3728 CB ILE A 588 41.624 −22.470 −36.804 1.00 42.47 C
ATOM 3729 CG1 ILE A 588 42.651 −21.452 −37.352 1.00 42.41 C
ATOM 3730 CG2 ILE A 588 40.400 −21.784 −36.164 1.00 42.29 C
ATOM 3731 CD1 ILE A 588 42.103 −20.467 −38.392 1.00 42.43 C
ATOM 3732 H ILE A 588 43.241 −24.251 −37.588 1.00 0.00 H

ATOM 3733 N ARG A 589 41.247 −24.131 −33.719 1.00 26.32 N
ATOM 3734 CA ARG A 589 40.341 −24.943 −32.892 1.00 26.34 C
ATOM 3735 C ARG A 589 39.897 −24.269 −31.590 1.00 26.07 C
ATOM 3736 O ARG A 589 40.740 −23.708 −30.899 1.00 25.86 O
ATOM 3737 CB ARG A 589 40.968 −26.324 −32.607 1.00 26.47 C
ATOM 3738 CG ARG A 589 42.338 −26.281 −31.894 1.00 26.31 C
ATOM 3739 CD ARG A 589 42.590 −27.542 −31.065 1.00 26.46 C
ATOM 3740 NE ARG A 589 41.671 −27.601 −29.910 1.00 26.36 N
ATOM 3741 CZ ARG A 589 41.283 −28.664 −29.238 1.00 26.51 C
ATOM 3742 NH1 ARG A 589 41.759 −29.853 −29.479 1.00 26.80 N
ATOM 3743 NH2 ARG A 589 40.387 −28.535 −28.307 1.00 26.40 N
ATOM 3744 H ARG A 589 41.839 −23.442 −33.269 1.00 0.00 H
ATOM 3745 HE ARG A 589 41.225 −26.729 −29.639 1.00 0.00 H
ATOM 3746 1HH1 ARG A 589 42.465 −29.941 −30.188 1.00 0.00 H
ATOM 3747 2HH1 ARG A 589 41.449 −30.643 −28.950 1.00 0.00 H
ATOM 3748 1HH2 ARG A 589 40.008 −27.600 −28.161 1.00 0.00 H
ATOM 3749 2HH2 ARG A 589 40.035 −29.314 −27.790 1.00 0.00 H
ATOM 3750 N PRO A 590 38.705 −24.627 −31.070 1.00 26.11 N
ATOM 3751 CA PRO A 590 38.286 −24.243 −29.730 1.00 25.90 C
ATOM 3752 C PRO A 590 39.253 −24.707 −28.634 1.00 25.71 C
ATOM 3753 O PRO A 590 39.733 −25.847 −28.656 1.00 25.80 O
ATOM 3754 CB PRO A 590 36.887 −24.833 −29.540 1.00 26.07 C
ATOM 3755 CG PRO A 590 36.346 −24.948 −30.960 1.00 26.35 C
ATOM 3756 CD PRO A 590 37.601 −25.271 −31.765 1.00 26.39 C
ATOM 3757 N LYS A 591 39.444 −23.868 −27.615 1.00 25.46 N
ATOM 3758 CA LYS A 591 40.323 −24.111 −26.461 1.00 25.26 C
ATOM 3759 C LYS A 591 39.483 −24.012 −25.177 1.00 25.15 C

ATOM 3760 O LYS A 591 38.646 −23.117 −25.046 1.00 25.18 O

ATOM 3761 CB LYS A 591 41.543 −23.149 −26.503 1.00 25.06 C

ATOM 3762 CG LYS A 591 42.323 −23.218 −27.840 1.00 25.19 C

ATOM 3763 CD LYS A 591 43.554 −22.292 −27.974 1.00 25.02 C

ATOM 3764 CE LYS A 591 43.984 −22.263 −29.456 1.00 25.17 C

ATOM 3765 NZ LYS A 591 45.116 −21.350 −29.753 1.00 25.02 N1+

ATOM 3766 H LYS A 591 38.938 −22.985 −27.638 1.00 0.00 H

ATOM 3767 HZ1 LYS A 591 45.355 −21.372 −30.749 1.00 0.00 H

ATOM 3768 HZ2 LYS A 591 44.950 −20.363 −29.541 1.00 0.00 H

ATOM 3769 HZ3 LYS A 591 45.995 −21.528 −29.278 1.00 0.00 H

ATOM 3770 N LEU A 592 39.510 −25.070 −24.363 1.00 25.04 N

ATOM 3771 CA LEU A 592 38.758 −25.163 −23.103 1.00 24.93 C

ATOM 3772 C LEU A 592 39.537 −24.504 −21.960 1.00 24.62 C

ATOM 3773 O LEU A 592 40.650 −24.934 −21.652 1.00 24.49 O

ATOM 3774 CB LEU A 592 38.483 −26.636 −22.747 1.00 25.04 C

ATOM 3775 CG LEU A 592 37.369 −27.324 −23.547 1.00 25.31 C

ATOM 3776 CD1 LEU A 592 37.495 −28.842 −23.407 1.00 25.46 C

ATOM 3777 CD2 LEU A 592 35.992 −26.919 −23.016 1.00 25.25 C

ATOM 3778 H LEU A 592 40.160 −25.814 −24.582 1.00 0.00 H

ATOM 3779 N PHE A 593 38.858 −23.665 −21.182 1.00 24.52 N

ATOM 3780 CA PHE A 593 39.402 −23.004 −19.998 1.00 24.25 C

ATOM 3781 C PHE A 593 38.589 −23.365 −18.750 1.00 24.15 C

ATOM 3782 O PHE A 593 37.381 −23.135 −18.698 1.00 24.24 O

ATOM 3783 CB PHE A 593 39.443 −21.485 −20.222 1.00 24.18 C

ATOM 3784 CG PHE A 593 40.314 −21.040 −21.385 1.00 24.24 C

ATOM 3785 CD1 PHE A 593 41.638 −20.616 −21.158 1.00 24.07 C

ATOM 3786 CD2 PHE A 593 39.789 −21.012 −22.692 1.00 24.47 C

ATOM	3787	CE1	PHE	A	593	42.424	−20.152	−22.228	1.00	24.15	C
ATOM	3788	CE2	PHE	A	593	40.578	−20.555	−23.762	1.00	24.53	C
ATOM	3789	CZ	PHE	A	593	41.894	−20.119	−23.530	1.00	24.38	C
ATOM	3790	H	PHE	A	593	37.923	−23.388	−21.468	1.00	0.00	H
ATOM	3791	N	ALA	A	594	39.261	−23.851	−17.708	1.00	23.97	N
ATOM	3792	CA	ALA	A	594	38.684	−24.025	−16.378	1.00	23.82	C
ATOM	3793	C	ALA	A	594	38.946	−22.784	−15.510	1.00	23.65	C
ATOM	3794	O	ALA	A	594	40.083	−22.312	−15.401	1.00	23.62	O
ATOM	3795	CB	ALA	A	594	39.237	−25.317	−15.764	1.00	23.65	C
ATOM	3796	H	ALA	A	594	40.266	−23.955	−17.799	1.00	0.00	H
ATOM	3797	N	VAL	A	595	37.915	−22.295	−14.817	1.00	23.58	N
ATOM	3798	CA	VAL	A	595	37.955	−21.059	−14.023	1.00	23.42	C
ATOM	3799	C	VAL	A	595	37.324	−21.284	−12.649	1.00	23.31	C
ATOM	3800	O	VAL	A	595	36.104	−21.292	−12.494	1.00	23.45	O
ATOM	3801	CB	VAL	A	595	37.279	−19.888	−14.772	1.00	23.57	C
ATOM	3802	CG1	VAL	A	595	37.432	−18.581	−13.980	1.00	23.50	C
ATOM	3803	CG2	VAL	A	595	37.892	−19.675	−16.162	1.00	23.58	C
ATOM	3804	H	VAL	A	595	37.001	−22.710	−14.988	1.00	0.00	H
ATOM	3805	N	LYS	A	596	38.160	−21.395	−11.612	1.00	23.05	N
ATOM	3806	CA	LYS	A	596	37.708	−21.398	−10.214	1.00	22.94	C
ATOM	3807	C	LYS	A	596	37.272	−19.984	−9.827	1.00	22.97	C
ATOM	3808	O	LYS	A	596	38.136	−19.117	−9.663	1.00	22.96	O
ATOM	3809	CB	LYS	A	596	38.837	−21.910	−9.306	1.00	22.65	C
ATOM	3810	CG	LYS	A	596	38.355	−22.085	−7.853	1.00	22.52	C
ATOM	3811	CD	LYS	A	596	39.454	−21.895	−6.795	1.00	22.26	C
ATOM	3812	CE	LYS	A	596	40.247	−20.574	−6.872	1.00	22.06	C
ATOM	3813	NZ	LYS	A	596	39.403	−19.388	−7.178	1.00	21.94	N1+

ATOM	3814	H	LYS	A	596	39.149	−21.439	−11.805	1.00	0.00	H
ATOM	3815	HZ1	LYS	A	596	39.901	−18.520	−7.033	1.00	0.00	H
ATOM	3816	HZ2	LYS	A	596	39.105	−19.392	−8.153	1.00	0.00	H
ATOM	3817	HZ3	LYS	A	596	38.534	−19.393	−6.650	1.00	0.00	H
ATOM	3818	N	ILE	A	597	36.023	−19.817	−9.404	1.00	27.88	N
ATOM	3819	CA	ILE	A	597	35.492	−18.542	−8.896	1.00	27.91	C
ATOM	3820	C	ILE	A	597	36.153	−18.149	−7.549	1.00	27.66	C
ATOM	3821	O	ILE	A	597	36.655	−19.019	−6.823	1.00	27.53	O
ATOM	3822	CB	ILE	A	597	33.949	−18.638	−8.842	1.00	28.14	C
ATOM	3823	CG1	ILE	A	597	33.322	−18.816	−10.249	1.00	28.39	C
ATOM	3824	CG2	ILE	A	597	33.250	−17.485	−8.104	1.00	28.13	C
ATOM	3825	CD1	ILE	A	597	33.636	−17.720	−11.281	1.00	28.37	C
ATOM	3826	H	ILE	A	597	35.406	−20.624	−9.400	1.00	0.00	H
ATOM	3827	N	PRO	A	598	36.334	−16.849	−7.234	1.00	22.73	N
ATOM	3828	CA	PRO	A	598	37.074	−16.429	−6.043	1.00	22.49	C
ATOM	3829	C	PRO	A	598	36.364	−16.782	−4.730	1.00	22.50	C
ATOM	3830	O	PRO	A	598	35.196	−16.439	−4.533	1.00	22.72	O
ATOM	3831	CB	PRO	A	598	37.262	−14.919	−6.169	1.00	22.53	C
ATOM	3832	CG	PRO	A	598	37.105	−14.661	−7.664	1.00	22.72	C
ATOM	3833	CD	PRO	A	598	36.056	−15.684	−8.064	1.00	22.86	C
ATOM	3834	N	ALA	A	599	37.124	−17.283	−3.752	1.00	51.52	N
ATOM	3835	CA	ALA	A	599	36.645	−17.460	−2.380	1.00	51.48	C
ATOM	3836	C	ALA	A	599	36.486	−16.114	−1.646	1.00	51.51	C
ATOM	3837	O	ALA	A	599	35.415	−15.854	−1.093	1.00	51.74	O
ATOM	3838	CB	ALA	A	599	37.609	−18.396	−1.641	1.00	51.20	C
ATOM	3839	H	ALA	A	599	38.092	−17.485	−3.943	1.00	0.00	H
ATOM	3840	N	GLN	A	600	37.464	−15.219	−1.819	1.00	22.03	N

ATOM	3841	CA	GLN	A	600	37.531	−13.847	−1.299	1.00	22.07	C
ATOM	3842	C	GLN	A	600	37.956	−12.869	−2.408	1.00	22.26	C
ATOM	3843	O	GLN	A	600	38.484	−13.291	−3.435	1.00	22.27	O
ATOM	3844	CB	GLN	A	600	38.543	−13.777	−0.135	1.00	21.78	C
ATOM	3845	CG	GLN	A	600	38.166	−14.568	1.130	1.00	21.59	C
ATOM	3846	CD	GLN	A	600	37.017	−13.948	1.924	1.00	21.49	C
ATOM	3847	OE1	GLN	A	600	36.178	−13.225	1.415	1.00	21.31	O
ATOM	3848	NE2	GLN	A	600	36.925	−14.213	3.207	1.00	21.62	N
ATOM	3849	H	GLN	A	600	38.250	−15.493	−2.391	1.00	0.00	H
ATOM	3850	1HE2	GLN	A	600	37.627	−14.756	3.675	1.00	0.00	H
ATOM	3851	2HE2	GLN	A	600	36.191	−13.739	3.704	1.00	0.00	H
ATOM	3852	N	CYS	A	601	37.772	−11.566	−2.191	1.00	42.68	N
ATOM	3853	CA	CYS	A	601	38.159	−10.496	−3.125	1.00	42.85	C
ATOM	3854	C	CYS	A	601	39.631	−10.045	−2.981	1.00	42.70	C
ATOM	3855	O	CYS	A	601	39.887	−8.853	−2.844	1.00	42.84	O
ATOM	3856	CB	CYS	A	601	37.187	−9.321	−2.957	1.00	43.15	C
ATOM	3857	SG	CYS	A	601	35.435	−9.738	−3.106	1.00	43.37	S
ATOM	3858	H	CYS	A	601	37.355	−11.281	−1.317	1.00	0.00	H
ATOM	3859	N	SER	A	602	40.534	−11.001	−2.749	1.00	26.10	N
ATOM	3860	CA	SER	A	602	41.901	−10.772	−2.258	1.00	25.93	C
ATOM	3861	C	SER	A	602	42.910	−10.475	−3.391	1.00	25.95	C
ATOM	3862	O	SER	A	602	42.968	−9.329	−3.838	1.00	26.14	O
ATOM	3863	CB	SER	A	602	42.249	−11.915	−1.295	1.00	25.61	C
ATOM	3864	OG	SER	A	602	43.589	−11.928	−0.882	1.00	25.48	O
ATOM	3865	H	SER	A	602	40.257	−11.957	−2.913	1.00	0.00	H
ATOM	3866	HG	SER	A	602	44.011	−12.649	−1.406	1.00	0.00	H
ATOM	3867	N	GLU	A	603	43.591	−11.479	−3.961	1.00	29.34	N

ATOM 3868 CA GLU A 603 44.657 −11.334 −4.988 1.00 29.44 C

ATOM 3869 C GLU A 603 44.332 −11.947 −6.357 1.00 29.48 C

ATOM 3870 O GLU A 603 44.816 −11.410 −7.383 1.00 29.30 O

ATOM 3871 CB GLU A 603 46.038 −11.811 −4.466 1.00 29.25 C

ATOM 3872 CG GLU A 603 46.126 −12.750 −3.244 1.00 29.06 C

ATOM 3873 CD GLU A 603 45.186 −13.971 −3.281 1.00 28.76 C

ATOM 3874 OE1 GLU A 603 45.598 −15.037 −3.796 1.00 28.62 O

ATOM 3875 OE2 GLU A 603 44.157 −13.899 −2.558 1.00 28.68 O

ATOM 3876 OXT GLU A 603 43.919 −13.121 −6.402 1.00 0.00 O

ATOM 3877 H GLU A 603 43.492 −12.407 −3.555 1.00 0.00 H

TER 3878 GLU A 603

ATOM 3879 N ILE B 31 49.221 −5.258 −37.647 1.00 50.96 N1+

ATOM 3880 CA ILE B 31 49.286 −3.776 −37.451 1.00 51.06 C

ATOM 3881 C ILE B 31 47.926 −3.173 −37.787 1.00 51.06 C

ATOM 3882 O ILE B 31 47.183 −3.729 −38.600 1.00 51.05 O

ATOM 3883 CB ILE B 31 50.336 −3.112 −38.395 1.00 51.29 C

ATOM 3884 CG1 ILE B 31 51.758 −3.536 −38.028 1.00 51.33 C

ATOM 3885 CG2 ILE B 31 50.208 −1.546 −38.391 1.00 51.44 C

ATOM 3886 CD1 ILE B 31 52.608 −2.386 −37.518 1.00 51.46 C

ATOM 3887 N VAL B 32 47.623 −2.030 −37.167 1.00 33.20 N

ATOM 3888 CA VAL B 32 46.446 −1.238 −37.480 1.00 33.25 C

ATOM 3889 C VAL B 32 46.813 −0.295 −38.611 1.00 33.47 C

ATOM 3890 O VAL B 32 47.472 0.727 −38.365 1.00 33.61 O

ATOM 3891 CB VAL B 32 46.005 −0.420 −36.211 1.00 33.21 C

ATOM 3892 CG1 VAL B 32 44.722 0.371 −36.465 1.00 33.26 C

ATOM 3893 CG2 VAL B 32 45.851 −1.363 −35.004 1.00 32.99 C

ATOM 3894 N LEU B 33 46.383 −0.632 −39.831 1.00 26.95 N

ATOM	3895	CA	LEU	B	33	46.721	0.160	−41.038	1.00	27.15	C
ATOM	3896	C	LEU	B	33	45.926	1.448	−41.050	1.00	27.24	C
ATOM	3897	O	LEU	B	33	45.022	1.643	−40.202	1.00	27.15	O
ATOM	3898	CB	LEU	B	33	46.438	−0.587	−42.333	1.00	27.16	C
ATOM	3899	CG	LEU	B	33	46.835	−2.031	−42.184	1.00	27.05	C
ATOM	3900	CD1	LEU	B	33	46.549	−2.702	−43.470	1.00	27.10	C
ATOM	3901	CD2	LEU	B	33	48.295	−2.169	−41.717	1.00	27.11	C
ATOM	3902	N	GLU	B	34	46.260	2.312	−42.020	1.00	36.14	N
ATOM	3903	CA	GLU	B	34	45.668	3.660	−42.136	1.00	36.28	C
ATOM	3904	C	GLU	B	34	44.172	3.679	−42.510	1.00	36.20	C
ATOM	3905	O	GLU	B	34	43.845	3.369	−43.682	1.00	36.20	O
ATOM	3906	CB	GLU	B	34	46.409	4.502	−43.171	1.00	36.52	C
ATOM	3907	CG	GLU	B	34	45.752	5.863	−43.333	1.00	36.67	C
ATOM	3908	CD	GLU	B	34	46.491	6.793	−44.246	1.00	36.93	C
ATOM	3909	OE1	GLU	B	34	45.966	7.901	−44.469	1.00	37.08	O
ATOM	3910	OE2	GLU	B	34	47.576	6.437	−44.744	1.00	37.01	O
ATOM	3911	N	PRO	B	35	43.281	4.088	−41.532	1.00	27.44	N
ATOM	3912	CA	PRO	B	35	41.828	4.229	−41.798	1.00	27.39	C
ATOM	3913	C	PRO	B	35	41.502	4.773	−43.219	1.00	27.51	C
ATOM	3914	O	PRO	B	35	42.136	5.734	−43.692	1.00	27.70	O
ATOM	3915	CB	PRO	B	35	41.354	5.183	−40.679	1.00	27.45	C
ATOM	3916	CG	PRO	B	35	42.261	4.907	−39.560	1.00	27.40	C
ATOM	3917	CD	PRO	B	35	43.587	4.443	−40.123	1.00	27.43	C
ATOM	3918	N	ILE	B	36	40.555	4.137	−43.904	1.00	33.35	N
ATOM	3919	CA	ILE	B	36	40.287	4.524	−45.294	1.00	33.44	C
ATOM	3920	C	ILE	B	36	38.923	5.196	−45.428	1.00	33.45	C
ATOM	3921	O	ILE	B	36	37.874	4.578	−45.228	1.00	33.32	O

ATOM 3922 CB ILE B 36 40.453 3.380 −46.337 1.00 33.36 C

ATOM 3923 CG1 ILE B 36 41.890 2.840 −46.303 1.00 33.39 C

ATOM 3924 CG2 ILE B 36 40.124 3.930 −47.746 1.00 33.45 C

ATOM 3925 CD1 ILE B 36 42.121 1.644 −47.180 1.00 33.34 C

ATOM 3926 N TYR B 37 38.956 6.467 −45.789 1.00 32.53 N

ATOM 3927 CA TYR B 37 37.753 7.260 −45.795 1.00 32.58 C

ATOM 3928 C TYR B 37 37.085 7.074 −47.144 1.00 32.55 C

ATOM 3929 O TYR B 37 37.670 7.404 −48.220 1.00 32.64 O

ATOM 3930 CB TYR B 37 38.076 8.723 −45.537 1.00 32.82 C

ATOM 3931 CG TYR B 37 38.590 8.984 −44.142 1.00 32.86 C

ATOM 3932 CD1 TYR B 37 37.821 9.718 −43.239 1.00 32.94 C

ATOM 3933 CD2 TYR B 37 39.841 8.494 −43.709 1.00 32.84 C

ATOM 3934 CE1 TYR B 37 38.268 9.983 −41.938 1.00 33.00 C

ATOM 3935 CE2 TYR B 37 40.302 8.738 −42.398 1.00 32.88 C

ATOM 3936 CZ TYR B 37 39.504 9.499 −41.514 1.00 32.96 C

ATOM 3937 OH TYR B 37 39.896 9.795 −40.210 1.00 33.01 O

ATOM 3938 N TRP B 38 35.878 6.510 −47.097 1.00 27.57 N

ATOM 3939 CA TRP B 38 35.093 6.315 −48.302 1.00 27.53 C

ATOM 3940 C TRP B 38 34.406 7.614 −48.565 1.00 27.67 C

ATOM 3941 O TRP B 38 33.454 7.969 −47.871 1.00 27.68 O

ATOM 3942 CB TRP B 38 34.084 5.211 −48.140 1.00 27.37 C

ATOM 3943 CG TRP B 38 33.455 4.871 −49.413 1.00 27.33 C

ATOM 3944 CD1 TRP B 38 32.468 5.548 −50.005 1.00 27.37 C

ATOM 3945 CD2 TRP B 38 33.768 3.756 −50.276 1.00 27.25 C

ATOM 3946 NE1 TRP B 38 32.124 4.949 −51.190 1.00 27.31 N

ATOM 3947 CE2 TRP B 38 32.904 3.843 −51.385 1.00 27.24 C

ATOM 3948 CE3 TRP B 38 34.683 2.694 −50.209 1.00 27.20 C

ATOM	3949	CZ2	TRP	B	38	32.914	2.918	−52.435	1.00	27.19	C
ATOM	3950	CZ3	TRP	B	38	34.700	1.777	−51.239	1.00	27.16	C
ATOM	3951	CH2	TRP	B	38	33.808	1.896	−52.349	1.00	27.16	C
ATOM	3952	N	ASN	B	39	34.935	8.324	−49.565	1.00	27.79	N
ATOM	3953	CA	ASN	B	39	34.608	9.731	−49.861	1.00	27.97	C
ATOM	3954	C	ASN	B	39	35.036	10.078	−51.309	1.00	28.03	C
ATOM	3955	O	ASN	B	39	35.979	9.479	−51.831	1.00	28.00	O
ATOM	3956	CB	ASN	B	39	35.345	10.612	−48.854	1.00	28.14	C
ATOM	3957	CG	ASN	B	39	34.580	11.799	−48.494	1.00	28.30	C
ATOM	3958	OD1	ASN	B	39	33.433	11.930	−48.871	1.00	28.27	O
ATOM	3959	ND2	ASN	B	39	35.197	12.688	−47.746	1.00	28.50	N
ATOM	3960	N	SER	B	40	34.374	11.024	−51.966	1.00	28.13	N
ATOM	3961	CA	SER	B	40	34.701	11.300	−53.377	1.00	28.17	C
ATOM	3962	C	SER	B	40	36.033	11.980	−53.531	1.00	28.37	C
ATOM	3963	O	SER	B	40	36.599	12.003	−54.621	1.00	28.41	O
ATOM	3964	CB	SER	B	40	33.688	12.245	−53.993	1.00	28.24	C
ATOM	3965	OG	SER	B	40	33.741	13.474	−53.299	1.00	28.46	O
ATOM	3966	N	SER	B	41	36.480	12.578	−52.431	1.00	28.51	N
ATOM	3967	CA	SER	B	41	37.696	13.392	−52.364	1.00	28.75	C
ATOM	3968	C	SER	B	41	38.971	12.588	−52.276	1.00	28.72	C
ATOM	3969	O	SER	B	41	40.064	13.090	−52.603	1.00	28.91	O
ATOM	3970	CB	SER	B	41	37.637	14.268	−51.105	1.00	28.92	C
ATOM	3971	OG	SER	B	41	37.350	13.467	−49.970	1.00	28.75	O
ATOM	3972	N	ASN	B	42	38.826	11.351	−51.808	1.00	69.62	N
ATOM	3973	CA	ASN	B	42	39.988	10.553	−51.394	1.00	69.59	C
ATOM	3974	C	ASN	B	42	40.954	10.192	−52.569	1.00	69.64	C
ATOM	3975	O	ASN	B	42	40.805	9.167	−53.271	1.00	69.49	O

ATOM 3976 CB ASN B 42 39.546 9.314 −50.548 1.00 69.35 C

ATOM 3977 CG ASN B 42 40.695 8.728 −49.685 1.00 69.34 C

ATOM 3978 OD1 ASN B 42 41.878 9.081 −49.873 1.00 69.50 O

ATOM 3979 ND2 ASN B 42 40.342 7.821 −48.742 1.00 69.16 N

ATOM 3980 N SER B 43 41.915 11.102 −52.757 1.00 37.97 N

ATOM 3981 CA SER B 43 43.149 10.912 −53.539 1.00 38.10 C

ATOM 3982 C SER B 43 43.836 9.539 −53.531 1.00 37.97 C

ATOM 3983 O SER B 43 44.881 9.398 −54.157 1.00 38.09 O

ATOM 3984 CB SER B 43 44.213 11.921 −53.050 1.00 38.38 C

ATOM 3985 OG SER B 43 43.850 13.290 −53.303 1.00 38.58 O

ATOM 3986 N LYS B 44 43.320 8.560 −52.782 1.00 28.53 N

ATOM 3987 CA LYS B 44 43.884 7.192 −52.785 1.00 28.41 C

ATOM 3988 C LYS B 44 43.153 6.300 −53.782 1.00 28.26 C

ATOM 3989 O LYS B 44 43.718 5.332 −54.260 1.00 28.24 O

ATOM 3990 CB LYS B 44 43.857 6.565 −51.384 1.00 28.28 C

ATOM 3991 CG LYS B 44 45.026 6.978 −50.481 1.00 28.42 C

ATOM 3992 CD LYS B 44 45.298 5.906 −49.388 1.00 28.25 C

ATOM 3993 CE LYS B 44 46.060 6.460 −48.187 1.00 28.35 C

ATOM 3994 NZ LYS B 44 45.178 7.321 −47.339 1.00 28.34 N1+

ATOM 3995 N PHE B 45 41.888 6.615 −54.063 1.00 30.56 N

ATOM 3996 CA PHE B 45 41.142 5.924 −55.118 1.00 30.45 C

ATOM 3997 C PHE B 45 41.637 6.438 −56.463 1.00 30.59 C

ATOM 3998 O PHE B 45 41.175 7.443 −57.019 1.00 30.66 O

ATOM 3999 CB PHE B 45 39.628 6.107 −54.969 1.00 30.31 C

ATOM 4000 CG PHE B 45 39.047 5.411 −53.759 1.00 30.16 C

ATOM 4001 CD1 PHE B 45 38.600 4.094 −53.849 1.00 30.01 C

ATOM 4002 CD2 PHE B 45 38.941 6.075 −52.536 1.00 30.19 C

ATOM	4003	CE1	PHE	B	45	38.069	3.455	−52.752	1.00	29.89	C
ATOM	4004	CE2	PHE	B	45	38.398	5.429	−51.417	1.00	30.05	C
ATOM	4005	CZ	PHE	B	45	37.971	4.128	−51.528	1.00	29.90	C
ATOM	4006	N	LEU	B	46	42.612	5.731	−56.971	1.00	28.27	N
ATOM	4007	CA	LEU	B	46	43.269	6.144	−58.174	1.00	28.43	C
ATOM	4008	C	LEU	B	46	42.418	5.659	−59.331	1.00	28.32	C
ATOM	4009	O	LEU	B	46	41.747	4.638	−59.229	1.00	28.16	O
ATOM	4010	CB	LEU	B	46	44.688	5.592	−58.180	1.00	28.56	C
ATOM	4011	CG	LEU	B	46	45.358	5.882	−56.825	1.00	28.62	C
ATOM	4012	CD1	LEU	B	46	46.769	5.282	−56.665	1.00	28.74	C
ATOM	4013	CD2	LEU	B	46	45.334	7.382	−56.580	1.00	28.77	C
ATOM	4014	N	PRO	B	47	42.473	6.374	−60.455	1.00	50.87	N
ATOM	4015	CA	PRO	B	47	41.393	6.378	−61.454	1.00	50.76	C
ATOM	4016	C	PRO	B	47	41.316	5.105	−62.288	1.00	50.68	C
ATOM	4017	O	PRO	B	47	40.215	4.694	−62.685	1.00	50.53	O
ATOM	4018	CB	PRO	B	47	41.777	7.554	−62.367	1.00	50.93	C
ATOM	4019	CG	PRO	B	47	43.281	7.564	−62.319	1.00	51.14	C
ATOM	4020	CD	PRO	B	47	43.639	7.157	−60.904	1.00	51.11	C
ATOM	4021	N	GLY	B	48	42.477	4.509	−62.555	1.00	28.37	N
ATOM	4022	CA	GLY	B	48	42.559	3.243	−63.269	1.00	28.35	C
ATOM	4023	C	GLY	B	48	42.433	2.024	−62.358	1.00	28.24	C
ATOM	4024	O	GLY	B	48	41.724	1.067	−62.689	1.00	28.14	O
ATOM	4025	N	GLN	B	49	43.114	2.043	−61.214	1.00	28.27	N
ATOM	4026	CA	GLN	B	49	43.201	0.844	−60.369	1.00	28.19	C
ATOM	4027	C	GLN	B	49	42.697	1.049	−58.918	1.00	28.06	C
ATOM	4028	O	GLN	B	49	43.001	0.257	−58.005	1.00	28.01	O
ATOM	4029	CB	GLN	B	49	44.639	0.307	−60.399	1.00	28.36	C

ATOM	4030	CG	GLN	B	49	45.655	1.209	−59.673	1.00	28.48	C
ATOM	4031	CD	GLN	B	49	46.786	1.714	−60.577	1.00	28.73	C
ATOM	4032	OE1	GLN	B	49	47.735	0.974	−60.930	1.00	28.86	O
ATOM	4033	NE2	GLN	B	49	46.692	2.996	−60.949	1.00	28.80	N
ATOM	4034	N	GLY	B	50	41.921	2.109	−58.714	1.00	28.01	N
ATOM	4035	CA	GLY	B	50	41.274	2.334	−57.428	1.00	27.89	C
ATOM	4036	C	GLY	B	50	42.292	2.429	−56.317	1.00	27.95	C
ATOM	4037	O	GLY	B	50	43.433	2.763	−56.582	1.00	28.11	O
ATOM	4038	N	LEU	B	51	41.878	2.136	−55.083	1.00	27.82	N
ATOM	4039	CA	LEU	B	51	42.781	2.102	−53.932	1.00	27.85	C
ATOM	4040	C	LEU	B	51	43.190	0.660	−53.795	1.00	27.80	C
ATOM	4041	O	LEU	B	51	42.336	−0.192	−53.500	1.00	27.65	O
ATOM	4042	CB	LEU	B	51	42.072	2.579	−52.647	1.00	27.75	C
ATOM	4043	CG	LEU	B	51	42.863	2.902	−51.368	1.00	27.79	C
ATOM	4044	CD1	LEU	B	51	41.990	3.760	−50.479	1.00	27.75	C
ATOM	4045	CD2	LEU	B	51	43.330	1.685	−50.606	1.00	27.69	C
ATOM	4046	N	VAL	B	52	44.471	0.373	−54.053	1.00	37.58	N
ATOM	4047	CA	VAL	B	52	45.048	−0.957	−53.747	1.00	37.55	C
ATOM	4048	C	VAL	B	52	45.842	−0.904	−52.419	1.00	37.54	C
ATOM	4049	O	VAL	B	52	46.563	0.084	−52.140	1.00	37.65	O
ATOM	4050	CB	VAL	B	52	45.887	−1.505	−54.900	1.00	37.71	C
ATOM	4051	CG1	VAL	B	52	46.605	−2.775	−54.467	1.00	37.72	C
ATOM	4052	CG2	VAL	B	52	44.973	−1.766	−56.111	1.00	37.69	C
ATOM	4053	N	LEU	B	53	45.684	−1.946	−51.599	1.00	27.76	N
ATOM	4054	CA	LEU	B	53	46.359	−2.010	−50.321	1.00	27.72	C
ATOM	4055	C	LEU	B	53	46.774	−3.471	−50.061	1.00	27.68	C
ATOM	4056	O	LEU	B	53	46.086	−4.409	−50.489	1.00	27.62	O

ATOM 4057 CB LEU B 53 45.438 −1.415 −49.242 1.00 27.58 C
ATOM 4058 CG LEU B 53 45.867 −1.413 −47.764 1.00 27.50 C
ATOM 4059 CD1 LEU B 53 47.116 −0.570 −47.566 1.00 27.65 C
ATOM 4060 CD2 LEU B 53 44.726 −0.864 −46.960 1.00 27.37 C
ATOM 4061 N TYR B 54 47.907 −3.648 −49.376 1.00 29.37 N
ATOM 4062 CA TYR B 54 48.516 −4.965 −49.157 1.00 29.36 C
ATOM 4063 C TYR B 54 48.367 −5.461 −47.713 1.00 29.18 C
ATOM 4064 O TYR B 54 49.374 −5.643 −47.037 1.00 29.20 O
ATOM 4065 CB TYR B 54 50.041 −4.925 −49.482 1.00 29.57 C
ATOM 4066 CG TYR B 54 50.475 −4.506 −50.908 1.00 29.78 C
ATOM 4067 CD1 TYR B 54 49.552 −4.437 −51.969 1.00 29.79 C
ATOM 4068 CD2 TYR B 54 51.819 −4.197 −51.189 1.00 30.00 C
ATOM 4069 CE1 TYR B 54 49.935 −4.046 −53.233 1.00 29.98 C
ATOM 4070 CE2 TYR B 54 52.208 −3.808 −52.467 1.00 30.21 C
ATOM 4071 CZ TYR B 54 51.255 −3.743 −53.483 1.00 30.19 C
ATOM 4072 OH TYR B 54 51.592 −3.390 −54.776 1.00 30.38 O
ATOM 4073 N PRO B 55 47.132 −5.692 −47.210 1.00 37.09 N
ATOM 4074 CA PRO B 55 47.173 −6.055 −45.778 1.00 36.93 C
ATOM 4075 C PRO B 55 47.896 −7.383 −45.593 1.00 36.94 C
ATOM 4076 O PRO B 55 48.082 −8.150 −46.567 1.00 37.06 O
ATOM 4077 CB PRO B 55 45.699 −6.151 −45.360 1.00 36.78 C
ATOM 4078 CG PRO B 55 44.907 −6.272 −46.628 1.00 36.84 C
ATOM 4079 CD PRO B 55 45.760 −5.692 −47.758 1.00 37.03 C
ATOM 4080 N GLN B 56 48.352 −7.624 −44.372 1.00 27.12 N
ATOM 4081 CA GLN B 56 48.898 −8.924 −44.003 1.00 27.10 C
ATOM 4082 C GLN B 56 47.963 −9.541 −43.000 1.00 26.90 C
ATOM 4083 O GLN B 56 47.247 −8.804 −42.303 1.00 26.78 O

ATOM 4084 CB GLN B 56 50.299 −8.786 −43.405 1.00 27.14 C

ATOM 4085 CG GLN B 56 51.379 −8.392 −44.404 1.00 27.37 C

ATOM 4086 CD GLN B 56 51.463 −9.353 −45.558 1.00 27.52 C

ATOM 4087 OE1 GLN B 56 51.285 −8.967 −46.723 1.00 27.66 O

ATOM 4088 NE2 GLN B 56 51.774 −10.615 −45.249 1.00 27.52 N

ATOM 4089 N ILE B 57 47.947 −10.878 −42.927 1.00 26.90 N

ATOM 4090 CA ILE B 57 47.113 −11.560 −41.912 1.00 26.72 C

ATOM 4091 C ILE B 57 47.480 −11.069 −40.484 1.00 26.56 C

ATOM 4092 O ILE B 57 48.656 −11.017 −40.100 1.00 26.58 O

ATOM 4093 CB ILE B 57 47.176 −13.112 −41.961 1.00 26.77 C

ATOM 4094 CG1 ILE B 57 46.622 −13.660 −43.268 1.00 26.93 C

ATOM 4095 CG2 ILE B 57 46.361 −13.718 −40.797 1.00 26.59 C

ATOM 4096 CD1 ILE B 57 45.126 −13.717 −43.302 1.00 26.86 C

ATOM 4097 N GLY B 58 46.460 −10.688 −39.720 1.00 42.67 N

ATOM 4098 CA GLY B 58 46.659 −10.070 −38.412 1.00 42.53 C

ATOM 4099 C GLY B 58 46.482 −8.542 −38.365 1.00 42.55 C

ATOM 4100 O GLY B 58 46.496 −7.915 −37.274 1.00 42.45 O

ATOM 4101 N ASP B 59 46.328 −7.920 −39.532 1.00 51.40 N

ATOM 4102 CA ASP B 59 46.115 −6.469 −39.567 1.00 51.44 C

ATOM 4103 C ASP B 59 44.653 −6.144 −39.234 1.00 51.36 C

ATOM 4104 O ASP B 59 43.812 −7.052 −39.115 1.00 51.28 O

ATOM 4105 CB ASP B 59 46.486 −5.882 −40.937 1.00 51.63 C

ATOM 4106 CG ASP B 59 47.870 −6.294 −41.395 1.00 51.75 C

ATOM 4107 OD1 ASP B 59 48.540 −7.040 −40.653 1.00 51.68 O

ATOM 4108 OD2 ASP B 59 48.282 −5.901 −42.511 1.00 51.92 O

ATOM 4109 N LYS B 60 44.362 −4.857 −39.059 1.00 26.43 N

ATOM 4110 CA LYS B 60 42.987 −4.387 −39.003 1.00 26.41 C

ATOM	4111	C	LYS	B	60	42.851	−3.002	−39.667	1.00	26.55	C
ATOM	4112	O	LYS	B	60	43.737	−2.141	−39.556	1.00	26.64	O
ATOM	4113	CB	LYS	B	60	42.464	−4.379	−37.561	1.00	26.27	C
ATOM	4114	CG	LYS	B	60	43.426	−3.754	−36.557	1.00	26.26	C
ATOM	4115	CD	LYS	B	60	42.775	−3.472	−35.243	1.00	26.15	C
ATOM	4116	CE	LYS	B	60	42.503	−4.747	−34.458	1.00	25.98	C
ATOM	4117	NZ	LYS	B	60	41.463	−4.561	−33.413	1.00	25.90	N1+
ATOM	4118	N	LEU	B	61	41.711	−2.812	−40.336	1.00	26.57	N
ATOM	4119	CA	LEU	B	61	41.493	−1.716	−41.269	1.00	26.71	C
ATOM	4120	C	LEU	B	61	40.137	−1.129	−41.043	1.00	26.70	C
ATOM	4121	O	LEU	B	61	39.152	−1.865	−41.044	1.00	26.62	O
ATOM	4122	CB	LEU	B	61	41.527	−2.225	−42.730	1.00	26.78	C
ATOM	4123	CG	LEU	B	61	41.606	−1.143	−43.837	1.00	26.93	C
ATOM	4124	CD1	LEU	B	61	43.054	−0.696	−43.896	1.00	27.05	C
ATOM	4125	CD2	LEU	B	61	41.119	−1.565	−45.232	1.00	26.97	C
ATOM	4126	N	ASP	B	62	40.079	0.187	−40.881	1.00	26.81	N
ATOM	4127	CA	ASP	B	62	38.803	0.887	−40.796	1.00	26.83	C
ATOM	4128	C	ASP	B	62	38.449	1.432	−42.205	1.00	26.95	C
ATOM	4129	O	ASP	B	62	39.273	2.054	−42.890	1.00	27.07	O
ATOM	4130	CB	ASP	B	62	38.846	2.025	−39.745	1.00	26.91	C
ATOM	4131	CG	ASP	B	62	38.912	1.518	−38.245	1.00	26.78	C
ATOM	4132	OD1	ASP	B	62	39.049	0.306	−37.950	1.00	26.63	O
ATOM	4133	OD2	ASP	B	62	38.821	2.372	−37.328	1.00	26.84	O
ATOM	4134	N	ILE	B	63	37.222	1.170	−42.650	1.00	37.00	N
ATOM	4135	CA	ILE	B	63	36.613	1.959	−43.755	1.00	37.11	C
ATOM	4136	C	ILE	B	63	35.496	2.855	−43.161	1.00	37.15	C
ATOM	4137	O	ILE	B	63	34.620	2.374	−42.405	1.00	37.07	O

ATOM 4138 CB ILE B 63 36.097 1.090 −44.922 1.00 37.06 C

ATOM 4139 CG1 ILE B 63 37.235 0.886 −45.947 1.00 37.11 C

ATOM 4140 CG2 ILE B 63 34.865 1.734 −45.559 1.00 37.11 C

ATOM 4141 CD1 ILE B 63 36.882 −0.008 −47.123 1.00 37.09 C

ATOM 4142 N ILE B 64 35.569 4.156 −43.466 1.00 27.21 N

ATOM 4143 CA ILE B 64 34.886 5.204 −42.706 1.00 27.31 C

ATOM 4144 C ILE B 64 34.228 6.182 −43.652 1.00 27.44 C

ATOM 4145 O ILE B 64 34.925 6.826 −44.415 1.00 27.55 O

ATOM 4146 CB ILE B 64 35.955 5.989 −41.845 1.00 27.42 C

ATOM 4147 CG1 ILE B 64 36.356 5.197 −40.598 1.00 27.29 C

ATOM 4148 CG2 ILE B 64 35.469 7.359 −41.411 1.00 27.61 C

ATOM 4149 CD1 ILE B 64 36.982 6.058 −39.519 1.00 27.40 C

ATOM 4150 N CYS B 65 32.906 6.307 −43.621 1.00 48.51 N

ATOM 4151 CA CYS B 65 32.260 7.447 −44.291 1.00 48.66 C

ATOM 4152 C CYS B 65 32.056 8.582 −43.296 1.00 48.83 C

ATOM 4153 O CYS B 65 31.347 8.416 −42.280 1.00 48.81 O

ATOM 4154 CB CYS B 65 30.922 7.108 −44.897 1.00 48.60 C

ATOM 4155 SG CYS B 65 30.887 5.504 −45.519 1.00 48.40 S

ATOM 4156 N PRO B 66 32.646 9.754 −43.594 1.00 45.59 N

ATOM 4157 CA PRO B 66 32.628 10.799 −42.605 1.00 45.79 C

ATOM 4158 C PRO B 66 31.234 11.383 −42.559 1.00 45.88 C

ATOM 4159 O PRO B 66 30.445 11.127 −43.468 1.00 45.81 O

ATOM 4160 CB PRO B 66 33.661 11.800 −43.136 1.00 45.98 C

ATOM 4161 CG PRO B 66 33.612 11.661 −44.575 1.00 45.94 C

ATOM 4162 CD PRO B 66 33.302 10.203 −44.838 1.00 45.67 C

ATOM 4163 N LYS B 67 30.933 12.097 −41.477 1.00 28.40 N

ATOM 4164 CA LYS B 67 29.701 12.889 −41.363 1.00 28.56 C

ATOM	4165	C	LYS	B	67	29.888	14.292	−41.942	1.00	28.83	C
ATOM	4166	O	LYS	B	67	30.983	14.816	−41.905	1.00	28.95	O
ATOM	4167	CB	LYS	B	67	29.230	12.969	−39.896	1.00	28.63	C
ATOM	4168	CG	LYS	B	67	30.001	13.910	−39.000	1.00	28.85	C
ATOM	4169	CD	LYS	B	67	29.736	13.590	−37.524	1.00	28.84	C
ATOM	4170	CE	LYS	B	67	30.537	14.491	−36.601	1.00	29.06	C
ATOM	4171	NZ	LYS	B	67	31.878	14.813	−37.179	1.00	29.10	N1+
ATOM	4172	N	VAL	B	68	28.810	14.897	−42.444	1.00	40.30	N
ATOM	4173	CA	VAL	B	68	28.866	16.227	−43.111	1.00	40.57	C
ATOM	4174	C	VAL	B	68	29.258	17.386	−42.164	1.00	40.89	C
ATOM	4175	O	VAL	B	68	28.473	17.822	−41.314	1.00	41.04	O
ATOM	4176	CB	VAL	B	68	27.510	16.580	−43.820	1.00	40.61	C
ATOM	4177	CG1	VAL	B	68	26.430	17.043	−42.770	1.00	40.79	C
ATOM	4178	CG2	VAL	B	68	27.721	17.623	−44.953	1.00	40.78	C
ATOM	4179	N	TYR	B	76	26.885	15.715	−47.918	1.00	28.84	N
ATOM	4180	CA	TYR	B	76	26.954	14.289	−47.549	1.00	28.59	C
ATOM	4181	C	TYR	B	76	26.778	13.367	−48.753	1.00	28.36	C
ATOM	4182	O	TYR	B	76	25.781	13.423	−49.478	1.00	28.34	O
ATOM	4183	CB	TYR	B	76	25.891	13.927	−46.484	1.00	28.58	C
ATOM	4184	CG	TYR	B	76	26.111	12.589	−45.810	1.00	28.37	C
ATOM	4185	CD1	TYR	B	76	27.098	12.448	−44.858	1.00	28.38	C
ATOM	4186	CD2	TYR	B	76	25.339	11.466	−46.126	1.00	28.18	C
ATOM	4187	CE1	TYR	B	76	27.332	11.206	−44.209	1.00	28.18	C
ATOM	4188	CE2	TYR	B	76	25.562	10.209	−45.489	1.00	28.01	C
ATOM	4189	CZ	TYR	B	76	26.575	10.101	−44.518	1.00	28.01	C
ATOM	4190	OH	TYR	B	76	26.874	8.948	−43.825	1.00	27.85	O
ATOM	4191	N	GLU	B	77	27.747	12.488	−48.913	1.00	28.22	N

ATOM 4192 CA GLU B 77 27.691 11.490 −49.932 1.00 28.02 C

ATOM 4193 C GLU B 77 27.014 10.203 −49.386 1.00 27.85 C

ATOM 4194 O GLU B 77 27.346 9.738 −48.299 1.00 27.82 O

ATOM 4195 CB GLU B 77 29.122 11.235 −50.409 1.00 27.99 C

ATOM 4196 CG GLU B 77 29.884 12.493 −50.822 1.00 28.19 C

ATOM 4197 CD GLU B 77 31.183 12.183 −51.593 1.00 28.16 C

ATOM 4198 OE1 GLU B 77 31.202 11.212 −52.382 1.00 27.99 O

ATOM 4199 OE2 GLU B 77 32.201 12.907 −51.420 1.00 28.32 O

ATOM 4200 N TYR B 78 26.059 9.659 −50.146 1.00 27.75 N

ATOM 4201 CA TYR B 78 25.361 8.406 −49.807 1.00 27.61 C

ATOM 4202 C TYR B 78 25.804 7.144 −50.548 1.00 27.45 C

ATOM 4203 O TYR B 78 25.752 7.071 −51.794 1.00 27.41 O

ATOM 4204 CB TYR B 78 23.878 8.565 −50.068 1.00 27.64 C

ATOM 4205 CG TYR B 78 23.266 9.579 −49.177 1.00 27.81 C

ATOM 4206 CD1 TYR B 78 22.974 9.268 −47.852 1.00 27.84 C

ATOM 4207 CD2 TYR B 78 23.001 10.847 −49.634 1.00 27.96 C

ATOM 4208 CE1 TYR B 78 22.411 10.198 −47.006 1.00 28.03 C

ATOM 4209 CE2 TYR B 78 22.446 11.787 −48.800 1.00 28.15 C

ATOM 4210 CZ TYR B 78 22.141 11.460 −47.486 1.00 28.19 C

ATOM 4211 OH TYR B 78 21.586 12.414 −46.664 1.00 28.41 O

ATOM 4212 N TYR B 79 26.158 6.134 −49.747 1.00 27.37 N

ATOM 4213 CA TYR B 79 26.808 4.931 −50.229 1.00 27.25 C

ATOM 4214 C TYR B 79 26.356 3.734 −49.485 1.00 27.17 C

ATOM 4215 O TYR B 79 26.253 3.780 −48.266 1.00 27.19 O

ATOM 4216 CB TYR B 79 28.324 4.989 −49.956 1.00 27.25 C

ATOM 4217 CG TYR B 79 29.076 6.033 −50.732 1.00 27.35 C

ATOM 4218 CD1 TYR B 79 29.150 5.971 −52.134 1.00 27.32 C

ATOM	4219	CD2	TYR	B	79	29.713	7.085	−50.075	1.00	27.47	C
ATOM	4220	CE1	TYR	B	79	29.842	6.937	−52.861	1.00	27.42	C
ATOM	4221	CE2	TYR	B	79	30.409	8.057	−50.793	1.00	27.58	C
ATOM	4222	CZ	TYR	B	79	30.469	7.978	−52.184	1.00	27.56	C
ATOM	4223	OH	TYR	B	79	31.157	8.938	−52.885	1.00	27.68	O
ATOM	4224	N	LYS	B	80	26.149	2.659	−50.231	1.00	27.09	N
ATOM	4225	CA	LYS	B	80	26.174	1.319	−49.699	1.00	27.03	C
ATOM	4226	C	LYS	B	80	27.419	0.655	−50.288	1.00	26.98	C
ATOM	4227	O	LYS	B	80	27.601	0.670	−51.477	1.00	26.98	O
ATOM	4228	CB	LYS	B	80	24.842	0.610	−50.026	1.00	27.03	C
ATOM	4229	CG	LYS	B	80	23.669	1.005	−49.030	1.00	27.09	C
ATOM	4230	CD	LYS	B	80	22.217	0.665	−49.500	1.00	27.13	C
ATOM	4231	CE	LYS	B	80	21.189	0.605	−48.323	1.00	27.20	C
ATOM	4232	NZ	LYS	B	80	19.899	−0.102	−48.640	1.00	27.24	N1+
ATOM	4233	N	VAL	B	81	28.310	0.147	−49.452	1.00	26.95	N
ATOM	4234	CA	VAL	B	81	29.563	−0.478	−49.918	1.00	26.93	C
ATOM	4235	C	VAL	B	81	29.536	−1.982	−49.674	1.00	26.88	C
ATOM	4236	O	VAL	B	81	29.226	−2.426	−48.581	1.00	26.85	O
ATOM	4237	CB	VAL	B	81	30.832	0.075	−49.197	1.00	26.94	C
ATOM	4238	CG1	VAL	B	81	32.006	−0.859	−49.388	1.00	26.91	C
ATOM	4239	CG2	VAL	B	81	31.208	1.427	−49.707	1.00	27.02	C
ATOM	4240	N	TYR	B	82	29.903	−2.765	−50.676	1.00	26.89	N
ATOM	4241	CA	TYR	B	82	29.839	−4.219	−50.579	1.00	26.89	C
ATOM	4242	C	TYR	B	82	31.189	−4.854	−50.884	1.00	26.90	C
ATOM	4243	O	TYR	B	82	31.872	−4.453	−51.833	1.00	26.94	O
ATOM	4244	CB	TYR	B	82	28.863	−4.811	−51.603	1.00	26.93	C
ATOM	4245	CG	TYR	B	82	27.402	−4.375	−51.572	1.00	26.93	C

ATOM	4246	CD1	TYR	B	82	26.401	−5.303	−51.508	1.00	26.97	C
ATOM	4247	CD2	TYR	B	82	27.035	−3.043	−51.676	1.00	26.93	C
ATOM	4248	CE1	TYR	B	82	25.104	−4.927	−51.519	1.00	26.99	C
ATOM	4249	CE2	TYR	B	82	25.733	−2.661	−51.677	1.00	26.95	C
ATOM	4250	CZ	TYR	B	82	24.778	−3.604	−51.598	1.00	26.98	C
ATOM	4251	OH	TYR	B	82	23.468	−3.225	−51.584	1.00	27.02	O
ATOM	4252	N	MET	B	83	31.555	−5.868	−50.097	1.00	26.89	N
ATOM	4253	CA	MET	B	83	32.622	−6.796	−50.474	1.00	26.93	C
ATOM	4254	C	MET	B	83	32.072	−7.588	−51.634	1.00	27.01	C
ATOM	4255	O	MET	B	83	31.008	−8.192	−51.490	1.00	27.02	O
ATOM	4256	CB	MET	B	83	32.952	−7.781	−49.335	1.00	26.90	C
ATOM	4257	CG	MET	B	83	33.591	−9.122	−49.819	1.00	26.98	C
ATOM	4258	SD	MET	B	83	35.030	−9.728	−48.865	1.00	26.96	S
ATOM	4259	CE	MET	B	83	35.924	−8.193	−48.537	1.00	26.89	C
ATOM	4260	N	VAL	B	84	32.770	−7.594	−52.766	1.00	27.08	N
ATOM	4261	CA	VAL	B	84	32.257	−8.270	−53.948	1.00	27.16	C
ATOM	4262	C	VAL	B	84	33.186	−9.411	−54.253	1.00	27.27	C
ATOM	4263	O	VAL	B	84	34.019	−9.779	−53.403	1.00	27.26	O
ATOM	4264	CB	VAL	B	84	32.115	−7.330	−55.181	1.00	27.18	C
ATOM	4265	CG1	VAL	B	84	31.337	−6.097	−54.810	1.00	27.09	C
ATOM	4266	CG2	VAL	B	84	33.465	−6.958	−55.788	1.00	27.23	C
ATOM	4267	N	ASP	B	85	32.988	−10.008	−55.431	1.00	52.19	N
ATOM	4268	CA	ASP	B	85	34.028	−10.797	−56.071	1.00	52.32	C
ATOM	4269	C	ASP	B	85	34.680	−9.954	−57.168	1.00	52.35	C
ATOM	4270	O	ASP	B	85	34.316	−8.801	−57.353	1.00	52.27	O
ATOM	4271	CB	ASP	B	85	33.451	−12.098	−56.627	1.00	52.47	C
ATOM	4272	CG	ASP	B	85	32.658	−11.896	−57.915	1.00	52.51	C

ATOM 4273 OD1 ASP B 85 33.290 −11.658 −58.979 1.00 52.58 O

ATOM 4274 OD2 ASP B 85 31.406 −12.004 −57.860 1.00 52.49 O

ATOM 4275 N LYS B 86 35.640 −10.542 −57.882 1.00 27.68 N

ATOM 4276 CA LYS B 86 36.378 −9.834 −58.933 1.00 27.74 C

ATOM 4277 C LYS B 86 35.485 −9.360 −60.051 1.00 27.73 C

ATOM 4278 O LYS B 86 35.465 −8.165 −60.350 1.00 27.66 O

ATOM 4279 CB LYS B 86 37.456 −10.717 −59.588 1.00 27.93 C

ATOM 4280 CG LYS B 86 38.352 −9.929 −60.585 1.00 28.01 C

ATOM 4281 CD LYS B 86 39.155 −10.818 −61.525 1.00 28.23 C

ATOM 4282 CE LYS B 86 40.481 −10.173 −61.960 1.00 28.32 C

ATOM 4283 NZ LYS B 86 40.395 −8.913 −62.776 1.00 28.28 N1+

ATOM 4284 N ASP B 87 34.787 −10.307 −60.688 1.00 36.30 N

ATOM 4285 CA ASP B 87 33.968 −10.015 −61.865 1.00 36.31 C

ATOM 4286 C ASP B 87 33.058 −8.858 −61.565 1.00 36.13 C

ATOM 4287 O ASP B 87 32.911 −7.951 −62.389 1.00 36.09 O

ATOM 4288 CB ASP B 87 33.091 −11.186 −62.225 1.00 36.41 C

ATOM 4289 CG ASP B 87 33.876 −12.389 −62.560 1.00 36.61 C

ATOM 4290 OD1 ASP B 87 34.908 −12.258 −63.253 1.00 36.71 O

ATOM 4291 OD2 ASP B 87 33.465 −13.476 −62.110 1.00 36.70 O

ATOM 4292 N GLN B 88 32.449 −8.912 −60.376 1.00 44.70 N

ATOM 4293 CA GLN B 88 31.612 −7.824 −59.847 1.00 44.56 C

ATOM 4294 C GLN B 88 32.400 −6.546 −59.687 1.00 44.50 C

ATOM 4295 O GLN B 88 31.902 −5.474 −59.978 1.00 44.43 O

ATOM 4296 CB GLN B 88 30.946 −8.209 −58.513 1.00 44.50 C

ATOM 4297 CG GLN B 88 29.632 −9.001 −58.695 1.00 44.55 C

ATOM 4298 CD GLN B 88 29.005 −9.526 −57.402 1.00 44.52 C

ATOM 4299 OE1 GLN B 88 29.701 −9.920 −56.442 1.00 44.52 O

ATOM	4300	NE2	GLN	B	88	27.673	−9.570	−57.389	1.00	44.52	N
ATOM	4301	N	ALA	B	89	33.630	−6.649	−59.212	1.00	48.80	N
ATOM	4302	CA	ALA	B	89	34.520	−5.487	−59.196	1.00	48.79	C
ATOM	4303	C	ALA	B	89	34.700	−4.925	−60.611	1.00	48.85	C
ATOM	4304	O	ALA	B	89	34.418	−3.747	−60.867	1.00	48.80	O
ATOM	4305	CB	ALA	B	89	35.879	−5.857	−58.590	1.00	48.85	C
ATOM	4306	N	ASP	B	90	35.121	−5.807	−61.522	1.00	41.47	N
ATOM	4307	CA	ASP	B	90	35.574	−5.442	−62.865	1.00	41.55	C
ATOM	4308	C	ASP	B	90	34.439	−5.134	−63.805	1.00	41.49	C
ATOM	4309	O	ASP	B	90	34.679	−4.632	−64.893	1.00	41.53	O
ATOM	4310	CB	ASP	B	90	36.451	−6.561	−63.426	1.00	41.71	C
ATOM	4311	CG	ASP	B	90	37.599	−6.952	−62.458	1.00	41.77	C
ATOM	4312	OD1	ASP	B	90	37.807	−6.248	−61.446	1.00	41.68	O
ATOM	4313	OD2	ASP	B	90	38.288	−7.968	−62.685	1.00	41.92	O
ATOM	4314	N	ARG	B	91	33.212	−5.429	−63.372	1.00	48.08	N
ATOM	4315	CA	ARG	B	91	31.973	−5.028	−64.068	1.00	48.01	C
ATOM	4316	C	ARG	B	91	31.143	−3.907	−63.350	1.00	47.88	C
ATOM	4317	O	ARG	B	91	30.234	−3.325	−63.962	1.00	47.82	O
ATOM	4318	CB	ARG	B	91	31.126	−6.288	−64.322	1.00	48.06	C
ATOM	4319	CG	ARG	B	91	31.786	−7.226	−65.344	1.00	48.21	C
ATOM	4320	CD	ARG	B	91	31.404	−8.698	−65.210	1.00	48.33	C
ATOM	4321	NE	ARG	B	91	32.220	−9.550	−66.097	1.00	48.51	N
ATOM	4322	CZ	ARG	B	91	32.275	−10.890	−66.077	1.00	48.67	C
ATOM	4323	NH1	ARG	B	91	31.566	−11.590	−65.205	1.00	48.68	N
ATOM	4324	NH2	ARG	B	91	33.058	−11.546	−66.937	1.00	48.86	N
ATOM	4325	N	CYS	B	92	31.516	−3.589	−62.093	1.00	43.45	N
ATOM	4326	CA	CYS	B	92	30.774	−2.703	−61.136	1.00	43.36	C

ATOM	4327	C	CYS	B	92	29.274	−2.990	−60.964	1.00	43.31	C
ATOM	4328	O	CYS	B	92	28.410	−2.149	−61.224	1.00	43.26	O
ATOM	4329	CB	CYS	B	92	31.041	−1.233	−61.426	1.00	43.35	C
ATOM	4330	SG	CYS	B	92	32.793	−0.866	−61.202	1.00	43.44	S
ATOM	4331	N	THR	B	93	29.027	−4.205	−60.477	1.00	27.78	N
ATOM	4332	CA	THR	B	93	27.717	−4.822	−60.331	1.00	27.78	C
ATOM	4333	C	THR	B	93	27.638	−5.485	−58.930	1.00	27.78	C
ATOM	4334	O	THR	B	93	28.583	−6.129	−58.463	1.00	27.82	O
ATOM	4335	CB	THR	B	93	27.469	−5.842	−61.525	1.00	27.86	C
ATOM	4336	OG1	THR	B	93	28.150	−7.096	−61.336	1.00	27.96	O
ATOM	4337	CG2	THR	B	93	27.974	−5.244	−62.821	1.00	27.86	C
ATOM	4338	N	ILE	B	94	26.518	−5.291	−58.249	1.00	27.11	N
ATOM	4339	CA	ILE	B	94	26.222	−5.975	−56.974	1.00	27.12	C
ATOM	4340	C	ILE	B	94	24.857	−6.686	−57.088	1.00	27.17	C
ATOM	4341	O	ILE	B	94	23.842	−6.075	−57.415	1.00	27.15	O
ATOM	4342	CB	ILE	B	94	26.177	−4.993	−55.799	1.00	27.05	C
ATOM	4343	CG1	ILE	B	94	25.569	−3.656	−56.275	1.00	27.01	C
ATOM	4344	CG2	ILE	B	94	27.559	−4.836	−55.219	1.00	27.02	C
ATOM	4345	CD1	ILE	B	94	24.892	−2.838	−55.250	1.00	26.99	C
ATOM	4346	N	LYS	B	95	24.809	−7.977	−56.814	1.00	27.26	N
ATOM	4347	CA	LYS	B	95	23.609	−8.713	−57.135	1.00	27.35	C
ATOM	4348	C	LYS	B	95	22.760	−8.879	−55.916	1.00	27.36	C
ATOM	4349	O	LYS	B	95	23.268	−8.822	−54.795	1.00	27.32	O
ATOM	4350	CB	LYS	B	95	23.968	−10.019	−57.773	1.00	27.48	C
ATOM	4351	CG	LYS	B	95	24.696	−9.785	−59.115	1.00	27.48	C
ATOM	4352	CD	LYS	B	95	23.754	−9.478	−60.311	1.00	27.49	C
ATOM	4353	CE	LYS	B	95	24.484	−8.685	−61.374	1.00	27.43	C

ATOM	4354	NZ	LYS	B	95	25.981	−8.812	−61.272	1.00	27.44	N1+
ATOM	4355	N	LYS	B	96	21.457	−9.033	−56.139	1.00	27.43	N
ATOM	4356	CA	LYS	B	96	20.456	−8.973	−55.059	1.00	27.46	C
ATOM	4357	C	LYS	B	96	20.928	−9.637	−53.746	1.00	27.47	C
ATOM	4358	O	LYS	B	96	20.911	−8.992	−52.671	1.00	27.40	O
ATOM	4359	CB	LYS	B	96	19.104	−9.541	−55.524	1.00	27.59	C
ATOM	4360	CG	LYS	B	96	19.138	−10.998	−56.027	1.00	27.75	C
ATOM	4361	CD	LYS	B	96	18.604	−12.020	−54.992	1.00	27.88	C
ATOM	4362	CE	LYS	B	96	19.008	−13.448	−55.356	1.00	28.05	C
ATOM	4363	NZ	LYS	B	96	20.498	−13.642	−55.323	1.00	27.98	N1+
ATOM	4364	N	GLU	B	97	21.397	−10.889	−53.848	1.00	44.12	N
ATOM	4365	CA	GLU	B	97	21.974	−11.627	−52.705	1.00	44.13	C
ATOM	4366	C	GLU	B	97	22.855	−10.710	−51.857	1.00	43.98	C
ATOM	4367	O	GLU	B	97	22.878	−10.808	−50.627	1.00	43.95	O
ATOM	4368	CB	GLU	B	97	22.837	−12.802	−53.214	1.00	44.23	C
ATOM	4369	CG	GLU	B	97	23.311	−13.798	−52.137	1.00	44.28	C
ATOM	4370	CD	GLU	B	97	24.674	−14.427	−52.459	1.00	44.30	C
ATOM	4371	OE1	GLU	B	97	25.306	−14.970	−51.520	1.00	44.29	O
ATOM	4372	OE2	GLU	B	97	25.120	−14.375	−53.631	1.00	44.34	O
ATOM	4373	N	ASN	B	98	23.570	−9.814	−52.530	1.00	27.33	N
ATOM	4374	CA	ASN	B	98	24.638	−9.116	−51.905	1.00	27.21	C
ATOM	4375	C	ASN	B	98	24.078	−8.285	−50.772	1.00	27.15	C
ATOM	4376	O	ASN	B	98	23.067	−7.588	−50.943	1.00	27.17	O
ATOM	4377	CB	ASN	B	98	25.443	−8.322	−52.936	1.00	27.17	C
ATOM	4378	CG	ASN	B	98	26.585	−9.150	−53.560	1.00	27.21	C
ATOM	4379	OD1	ASN	B	98	27.100	−8.818	−54.623	1.00	27.22	O
ATOM	4380	ND2	ASN	B	98	26.998	−10.212	−52.873	1.00	27.26	N

ATOM	4381	N	THR	B	99	24.702	−8.468	−49.599	1.00	29.42	N
ATOM	4382	CA	THR	B	99	24.393	−7.738	−48.378	1.00	29.37	C
ATOM	4383	C	THR	B	99	25.587	−6.801	−48.164	1.00	29.27	C
ATOM	4384	O	THR	B	99	26.727	−7.221	−48.386	1.00	29.25	O
ATOM	4385	CB	THR	B	99	24.188	−8.698	−47.187	1.00	29.39	C
ATOM	4386	OG1	THR	B	99	23.068	−9.558	−47.452	1.00	29.51	O
ATOM	4387	CG2	THR	B	99	23.932	−7.927	−45.885	1.00	29.34	C
ATOM	4388	N	PRO	B	100	25.327	−5.517	−47.800	1.00	44.48	N
ATOM	4389	CA	PRO	B	100	26.378	−4.505	−47.635	1.00	44.42	C
ATOM	4390	C	PRO	B	100	27.268	−4.634	−46.384	1.00	44.36	C
ATOM	4391	O	PRO	B	100	26.889	−5.280	−45.379	1.00	44.35	O
ATOM	4392	CB	PRO	B	100	25.585	−3.182	−47.546	1.00	44.45	C
ATOM	4393	CG	PRO	B	100	24.201	−3.496	−47.904	1.00	44.51	C
ATOM	4394	CD	PRO	B	100	24.000	−4.921	−47.565	1.00	44.53	C
ATOM	4395	N	LEU	B	101	28.444	−4.015	−46.449	1.00	37.24	N
ATOM	4396	CA	LEU	B	101	29.297	−3.911	−45.287	1.00	37.19	C
ATOM	4397	C	LEU	B	101	29.244	−2.485	−44.733	1.00	37.21	C
ATOM	4398	O	LEU	B	101	29.488	−2.277	−43.521	1.00	37.18	O
ATOM	4399	CB	LEU	B	101	30.722	−4.352	−45.614	1.00	37.16	C
ATOM	4400	CG	LEU	B	101	31.497	−3.565	−46.665	1.00	37.20	C
ATOM	4401	CD1	LEU	B	101	32.085	−2.277	−46.100	1.00	37.22	C
ATOM	4402	CD2	LEU	B	101	32.595	−4.453	−47.287	1.00	37.21	C
ATOM	4403	N	LEU	B	102	28.897	−1.510	−45.574	1.00	26.82	N
ATOM	4404	CA	LEU	B	102	28.701	−0.169	−45.065	1.00	26.88	C
ATOM	4405	C	LEU	B	102	27.486	0.515	−45.675	1.00	26.95	C
ATOM	4406	O	LEU	B	102	27.372	0.625	−46.879	1.00	26.97	O
ATOM	4407	CB	LEU	B	102	29.983	0.650	−45.203	1.00	26.91	C

ATOM	4408	CG	LEU	B	102	30.919	0.589	−43.973	1.00	26.87		C
ATOM	4409	CD1	LEU	B	102	32.101	1.552	−44.059	1.00	26.94		C
ATOM	4410	CD2	LEU	B	102	30.162	0.893	−42.694	1.00	26.88		C
ATOM	4411	N	ASN	B	103	26.572	0.935	−44.802	1.00	31.56		N
ATOM	4412	CA	ASN	B	103	25.335	1.645	−45.172	1.00	31.65		C
ATOM	4413	C	ASN	B	103	25.491	3.107	−44.751	1.00	31.75		C
ATOM	4414	O	ASN	B	103	25.092	3.485	−43.623	1.00	31.82		O
ATOM	4415	CB	ASN	B	103	24.125	0.999	−44.446	1.00	31.67		C
ATOM	4416	CG	ASN	B	103	22.739	1.443	−44.987	1.00	31.76		C
ATOM	4417	OD1	ASN	B	103	22.698	2.326	−45.852	1.00	31.81		O
ATOM	4418	ND2	ASN	B	103	21.630	0.865	−44.487	1.00	31.81		N
ATOM	4419	N	CYS	B	104	26.070	3.920	−45.645	1.00	27.24		N
ATOM	4420	CA	CYS	B	104	26.423	5.287	−45.312	1.00	27.37		C
ATOM	4421	C	CYS	B	104	25.285	6.213	−45.682	1.00	27.49		C
ATOM	4422	O	CYS	B	104	25.183	6.712	−46.788	1.00	27.52		O
ATOM	4423	CB	CYS	B	104	27.785	5.648	−45.898	1.00	27.37		C
ATOM	4424	SG	CYS	B	104	29.073	4.868	−44.831	1.00	27.28		S
ATOM	4425	N	ALA	B	105	24.413	6.383	−44.697	1.00	27.56		N
ATOM	4426	CA	ALA	B	105	23.171	7.088	−44.833	1.00	27.69		C
ATOM	4427	C	ALA	B	105	22.866	7.758	−43.513	1.00	27.84		C
ATOM	4428	O	ALA	B	105	21.708	7.772	−43.085	1.00	27.92		O
ATOM	4429	CB	ALA	B	105	22.076	6.114	−45.182	1.00	27.62		C
ATOM	4430	N	LYS	B	106	23.896	8.309	−42.884	1.00	27.89		N
ATOM	4431	CA	LYS	B	106	23.766	9.126	−41.675	1.00	28.06		C
ATOM	4432	C	LYS	B	106	24.534	10.456	−41.733	1.00	28.23		C
ATOM	4433	O	LYS	B	106	25.645	10.582	−41.307	1.00	28.22		O
ATOM	4434	CB	LYS	B	106	24.095	8.321	−40.429	1.00	27.96		C

ATOM	4435	CG	LYS	B	106	23.065	7.303	−40.146	1.00	27.89	C
ATOM	4436	CD	LYS	B	106	23.111	6.860	−38.743	1.00	27.87	C
ATOM	4437	CE	LYS	B	106	22.369	5.555	−38.534	1.00	27.76	C
ATOM	4438	NZ	LYS	B	106	22.947	4.732	−37.446	1.00	27.64	N1+
ATOM	4439	N	PRO	B	107	23.912	11.458	−42.302	1.00	42.84	N
ATOM	4440	CA	PRO	B	107	24.602	12.714	−42.499	1.00	43.03	C
ATOM	4441	C	PRO	B	107	25.472	13.117	−41.326	1.00	43.14	C
ATOM	4442	O	PRO	B	107	26.635	13.435	−41.539	1.00	43.16	O
ATOM	4443	CB	PRO	B	107	23.450	13.707	−42.732	1.00	43.25	C
ATOM	4444	CG	PRO	B	107	22.447	12.894	−43.479	1.00	43.09	C
ATOM	4445	CD	PRO	B	107	22.539	11.509	−42.856	1.00	42.88	C
ATOM	4446	N	ASP	B	108	24.930	13.062	−40.110	1.00	56.47	N
ATOM	4447	CA	ASP	B	108	25.598	13.682	−38.932	1.00	56.63	C
ATOM	4448	C	ASP	B	108	26.256	12.670	−37.997	1.00	56.44	C
ATOM	4449	O	ASP	B	108	26.455	12.945	−36.792	1.00	56.54	O
ATOM	4450	CB	ASP	B	108	24.607	14.565	−38.145	1.00	56.90	C
ATOM	4451	CG	ASP	B	108	23.379	13.805	−37.695	1.00	56.83	C
ATOM	4452	OD1	ASP	B	108	23.545	12.621	−37.321	1.00	56.59	O
ATOM	4453	OD2	ASP	B	108	22.263	14.388	−37.737	1.00	57.02	O
ATOM	4454	N	GLN	B	109	26.587	11.510	−38.567	1.00	28.46	N
ATOM	4455	CA	GLN	B	109	27.214	10.437	−37.820	1.00	28.26	C
ATOM	4456	C	GLN	B	109	28.320	9.709	−38.630	1.00	28.07	C
ATOM	4457	O	GLN	B	109	28.089	9.243	−39.753	1.00	27.97	O
ATOM	4458	CB	GLN	B	109	26.129	9.478	−37.338	1.00	28.15	C
ATOM	4459	CG	GLN	B	109	26.335	8.989	−35.918	1.00	28.09	C
ATOM	4460	CD	GLN	B	109	25.492	7.768	−35.618	1.00	27.94	C
ATOM	4461	OE1	GLN	B	109	26.009	6.645	−35.580	1.00	27.73	O

ATOM 4462 NE2 GLN B 109 24.181 7.972 −35.438 1.00 28.07 N

ATOM 4463 N ASP B 110 29.524 9.659 −38.046 1.00 34.03 N

ATOM 4464 CA ASP B 110 30.616 8.798 −38.521 1.00 33.85 C

ATOM 4465 C ASP B 110 30.165 7.326 −38.601 1.00 33.61 C

ATOM 4466 O ASP B 110 29.941 6.671 −37.566 1.00 33.52 O

ATOM 4467 CB ASP B 110 31.813 8.855 −37.555 1.00 33.84 C

ATOM 4468 CG ASP B 110 32.551 10.177 −37.581 1.00 34.08 C

ATOM 4469 OD1 ASP B 110 32.719 10.757 −38.668 1.00 34.18 O

ATOM 4470 OD2 ASP B 110 33.011 10.628 −36.517 1.00 34.18 O

ATOM 4471 N ILE B 111 30.031 6.803 −39.819 1.00 27.53 N

ATOM 4472 CA ILE B 111 29.692 5.394 −40.008 1.00 27.34 C

ATOM 4473 C ILE B 111 30.946 4.673 −40.400 1.00 27.23 C

ATOM 4474 O ILE B 111 31.517 4.953 −41.433 1.00 27.26 O

ATOM 4475 CB ILE B 111 28.599 5.197 −41.064 1.00 27.34 C

ATOM 4476 CG1 ILE B 111 27.233 5.383 −40.394 1.00 27.42 C

ATOM 4477 CG2 ILE B 111 28.700 3.821 −41.704 1.00 27.18 C

ATOM 4478 CD1 ILE B 111 26.054 5.392 −41.348 1.00 27.46 C

ATOM 4479 N LYS B 112 31.350 3.739 −39.544 1.00 27.10 N

ATOM 4480 CA LYS B 112 32.675 3.093 −39.596 1.00 27.00 C

ATOM 4481 C LYS B 112 32.590 1.536 −39.515 1.00 26.83 C

ATOM 4482 O LYS B 112 31.808 0.972 −38.714 1.00 26.77 O

ATOM 4483 CB LYS B 112 33.566 3.648 −38.458 1.00 27.03 C

ATOM 4484 CG LYS B 112 32.864 3.746 −37.080 1.00 27.03 C

ATOM 4485 CD LYS B 112 33.839 4.056 −35.958 1.00 27.04 C

ATOM 4486 CE LYS B 112 34.690 2.839 −35.564 1.00 26.85 C

ATOM 4487 NZ LYS B 112 33.987 1.903 −34.612 1.00 26.71 N1+

ATOM 4488 N PHE B 113 33.395 0.851 −40.334 1.00 26.78 N

ATOM	4489	CA	PHE	B	113	33.426	−0.613	−40.325	1.00	26.65	C
ATOM	4490	C	PHE	B	113	34.867	−1.117	−40.165	1.00	26.59	C
ATOM	4491	O	PHE	B	113	35.773	−0.642	−40.864	1.00	26.66	O
ATOM	4492	CB	PHE	B	113	32.774	−1.179	−41.592	1.00	26.66	C
ATOM	4493	CG	PHE	B	113	33.201	−2.580	−41.905	1.00	26.59	C
ATOM	4494	CD1	PHE	B	113	33.104	−3.580	−40.939	1.00	26.50	C
ATOM	4495	CD2	PHE	B	113	33.741	−2.899	−43.154	1.00	26.62	C
ATOM	4496	CE1	PHE	B	113	33.534	−4.886	−41.219	1.00	26.46	C
ATOM	4497	CE2	PHE	B	113	34.167	−4.205	−43.446	1.00	26.59	C
ATOM	4498	CZ	PHE	B	113	34.059	−5.198	−42.481	1.00	26.51	C
ATOM	4499	N	THR	B	114	35.068	−2.068	−39.242	1.00	26.48	N
ATOM	4500	CA	THR	B	114	36.429	−2.605	−38.934	1.00	26.42	C
ATOM	4501	C	THR	B	114	36.640	−4.084	−39.388	1.00	26.36	C
ATOM	4502	O	THR	B	114	35.935	−5.030	−38.950	1.00	26.29	O
ATOM	4503	CB	THR	B	114	36.865	−2.446	−37.402	1.00	26.35	C
ATOM	4504	OG1	THR	B	114	37.134	−1.071	−37.068	1.00	26.44	O
ATOM	4505	CG2	THR	B	114	38.108	−3.273	−37.113	1.00	26.26	C
ATOM	4506	N	ILE	B	115	37.631	−4.260	−40.256	1.00	26.40	N
ATOM	4507	CA	ILE	B	115	37.999	−5.562	−40.749	1.00	26.39	C
ATOM	4508	C	ILE	B	115	39.231	−5.917	−39.949	1.00	26.33	C
ATOM	4509	O	ILE	B	115	40.162	−5.130	−39.928	1.00	26.37	O
ATOM	4510	CB	ILE	B	115	38.393	−5.513	−42.292	1.00	26.50	C
ATOM	4511	CG1	ILE	B	115	37.222	−5.169	−43.211	1.00	26.56	C
ATOM	4512	CG2	ILE	B	115	38.925	−6.852	−42.801	1.00	26.52	C
ATOM	4513	CD1	ILE	B	115	37.601	−5.221	−44.715	1.00	26.66	C
ATOM	4514	N	LYS	B	116	39.244	−7.060	−39.266	1.00	26.24	N
ATOM	4515	CA	LYS	B	116	40.523	−7.720	−38.904	1.00	26.20	C

ATOM	4516	C	LYS	B	116	40.716	−8.935	−39.820	1.00	26.27	C
ATOM	4517	O	LYS	B	116	39.816	−9.766	−39.977	1.00	26.27	O
ATOM	4518	CB	LYS	B	116	40.610	−8.158	−37.420	1.00	26.06	C
ATOM	4519	CG	LYS	B	116	41.878	−8.991	−37.130	1.00	26.02	C
ATOM	4520	CD	LYS	B	116	42.168	−9.222	−35.686	1.00	25.87	C
ATOM	4521	CE	LYS	B	116	43.599	−9.712	−35.468	1.00	25.85	C
ATOM	4522	NZ	LYS	B	116	44.582	−8.597	−35.384	1.00	25.89	N1+
ATOM	4523	N	PHE	B	117	41.900	−9.050	−40.399	1.00	26.54	N
ATOM	4524	CA	PHE	B	117	42.149	−10.081	−41.376	1.00	26.64	C
ATOM	4525	C	PHE	B	117	42.527	−11.402	−40.693	1.00	26.59	C
ATOM	4526	O	PHE	B	117	43.677	−11.625	−40.272	1.00	26.58	O
ATOM	4527	CB	PHE	B	117	43.189	−9.573	−42.341	1.00	26.77	C
ATOM	4528	CG	PHE	B	117	42.828	−8.243	−42.910	1.00	26.82	C
ATOM	4529	CD1	PHE	B	117	41.996	−8.157	−44.017	1.00	26.89	C
ATOM	4530	CD2	PHE	B	117	43.266	−7.075	−42.311	1.00	26.80	C
ATOM	4531	CE1	PHE	B	117	41.652	−6.925	−44.537	1.00	26.94	C
ATOM	4532	CE2	PHE	B	117	42.918	−5.841	−42.816	1.00	26.86	C
ATOM	4533	CZ	PHE	B	117	42.110	−5.761	−43.926	1.00	26.93	C
ATOM	4534	N	GLN	B	118	41.511	−12.257	−40.559	1.00	26.38	N
ATOM	4535	CA	GLN	B	118	41.626	−13.565	−39.921	1.00	26.35	C
ATOM	4536	C	GLN	B	118	40.372	−14.370	−40.232	1.00	26.41	C
ATOM	4537	O	GLN	B	118	39.479	−13.918	−40.911	1.00	26.46	O
ATOM	4538	CB	GLN	B	118	41.751	−13.403	−38.418	1.00	26.17	C
ATOM	4539	CG	GLN	B	118	40.596	−12.607	−37.819	1.00	26.08	C
ATOM	4540	CD	GLN	B	118	39.977	−13.246	−36.604	1.00	25.97	C
ATOM	4541	OE1	GLN	B	118	40.210	−14.422	−36.286	1.00	25.96	O
ATOM	4542	NE2	GLN	B	118	39.146	−12.476	−35.926	1.00	25.89	N

ATOM	4543	N	GLU	B	119	40.276	−15.551	−39.670	1.00	26.41	N
ATOM	4544	CA	GLU	B	119	39.191	−16.455	−40.000	1.00	26.51	C
ATOM	4545	C	GLU	B	119	37.916	−16.123	−39.222	1.00	26.41	C
ATOM	4546	O	GLU	B	119	36.843	−16.419	−39.693	1.00	26.51	O
ATOM	4547	CB	GLU	B	119	39.621	−17.918	−39.757	1.00	26.58	C
ATOM	4548	CG	GLU	B	119	41.125	−18.219	−40.079	1.00	26.64	C
ATOM	4549	CD	GLU	B	119	41.341	−19.399	−41.021	1.00	26.88	C
ATOM	4550	OE1	GLU	B	119	42.318	−20.170	−40.817	1.00	26.93	O
ATOM	4551	OE2	GLU	B	119	40.542	−19.544	−41.982	1.00	27.02	O
ATOM	4552	N	PHE	B	120	38.045	−15.512	−38.037	1.00	26.24	N
ATOM	4553	CA	PHE	B	120	36.899	−15.215	−37.124	1.00	26.16	C
ATOM	4554	C	PHE	B	120	36.279	−13.792	−37.251	1.00	26.13	C
ATOM	4555	O	PHE	B	120	36.930	−12.774	−36.949	1.00	26.05	O
ATOM	4556	CB	PHE	B	120	37.278	−15.435	−35.636	1.00	26.00	C
ATOM	4557	CG	PHE	B	120	37.710	−16.829	−35.294	1.00	26.02	C
ATOM	4558	CD1	PHE	B	120	37.182	−17.942	−35.928	1.00	26.18	C
ATOM	4559	CD2	PHE	B	120	38.630	−17.041	−34.307	1.00	25.88	C
ATOM	4560	CE1	PHE	B	120	37.597	−19.260	−35.586	1.00	26.23	C
ATOM	4561	CE2	PHE	B	120	39.044	−18.359	−33.968	1.00	25.91	C
ATOM	4562	CZ	PHE	B	120	38.524	−19.453	−34.615	1.00	26.09	C
ATOM	4563	N	SER	B	121	34.992	−13.748	−37.616	1.00	27.80	N
ATOM	4564	CA	SER	B	121	34.241	−12.497	−37.777	1.00	27.80	C
ATOM	4565	C	SER	B	121	32.791	−12.586	−37.218	1.00	27.82	C
ATOM	4566	O	SER	B	121	32.152	−13.635	−37.288	1.00	27.90	O
ATOM	4567	CB	SER	B	121	34.224	−12.130	−39.262	1.00	27.91	C
ATOM	4568	OG	SER	B	121	34.083	−13.283	−40.092	1.00	28.03	O
ATOM	4569	N	PRO	B	122	32.266	−11.488	−36.645	1.00	27.67	N

ATOM 4570 CA PRO B 122 30.842 −11.492 −36.329 1.00 27.73 C
ATOM 4571 C PRO B 122 29.970 −11.715 −37.567 1.00 27.88 C
ATOM 4572 O PRO B 122 28.779 −12.029 −37.411 1.00 27.96 O
ATOM 4573 CB PRO B 122 30.577 −10.079 −35.802 1.00 27.70 C
ATOM 4574 CG PRO B 122 31.859 −9.361 −35.788 1.00 27.62 C
ATOM 4575 CD PRO B 122 32.941 −10.231 −36.279 1.00 27.60 C
ATOM 4576 N ASN B 123 30.548 −11.534 −38.769 1.00 33.40 N
ATOM 4577 CA ASN B 123 29.855 −11.735 −40.044 1.00 33.53 C
ATOM 4578 C ASN B 123 29.315 −13.139 −40.125 1.00 33.63 C
ATOM 4579 O ASN B 123 30.080 −14.071 −40.022 1.00 33.63 O
ATOM 4580 CB ASN B 123 30.847 −11.501 −41.193 1.00 33.54 C
ATOM 4581 CG ASN B 123 30.520 −12.314 −42.454 1.00 33.68 C
ATOM 4582 OD1 ASN B 123 31.215 −13.297 −42.756 1.00 33.73 O
ATOM 4583 ND2 ASN B 123 29.473 −11.908 −43.192 1.00 33.75 N
ATOM 4584 N LEU B 124 28.016 −13.300 −40.341 1.00 26.77 N
ATOM 4585 CA LEU B 124 27.388 −14.618 −40.249 1.00 26.90 C
ATOM 4586 C LEU B 124 27.165 −15.281 −41.608 1.00 27.05 C
ATOM 4587 O LEU B 124 26.306 −16.167 −41.766 1.00 27.21 O
ATOM 4588 CB LEU B 124 26.064 −14.498 −39.490 1.00 26.95 C
ATOM 4589 CG LEU B 124 26.129 −15.147 −38.126 1.00 26.90 C
ATOM 4590 CD1 LEU B 124 26.270 −16.663 −38.308 1.00 27.02 C
ATOM 4591 CD2 LEU B 124 27.294 −14.537 −37.307 1.00 26.70 C
ATOM 4592 N TRP B 125 27.964 −14.864 −42.584 1.00 39.25 N
ATOM 4593 CA TRP B 125 27.805 −15.317 −43.944 1.00 39.40 C
ATOM 4594 C TRP B 125 29.042 −16.034 −44.441 1.00 39.43 C
ATOM 4595 O TRP B 125 29.168 −16.273 −45.642 1.00 39.54 O
ATOM 4596 CB TRP B 125 27.429 −14.137 −44.836 1.00 39.37 C

ATOM	4597	CG	TRP	B	125	25.961	−14.078	−45.031	1.00	39.47	C
ATOM	4598	CD1	TRP	B	125	25.029	−13.554	−44.166	1.00	39.44	C
ATOM	4599	CD2	TRP	B	125	25.227	−14.604	−46.147	1.00	39.63	C
ATOM	4600	NE1	TRP	B	125	23.763	−13.705	−44.688	1.00	39.57	N
ATOM	4601	CE2	TRP	B	125	23.854	−14.343	−45.905	1.00	39.69	C
ATOM	4602	CE3	TRP	B	125	25.598	−15.241	−47.351	1.00	39.74	C
ATOM	4603	CZ2	TRP	B	125	22.840	−14.712	−46.832	1.00	39.85	C
ATOM	4604	CZ3	TRP	B	125	24.590	−15.599	−48.274	1.00	39.90	C
ATOM	4605	CH2	TRP	B	125	23.233	−15.334	−48.003	1.00	39.94	C
ATOM	4606	N	GLY	B	126	29.926	−16.419	−43.518	1.00	36.07	N
ATOM	4607	CA	GLY	B	126	31.108	−17.203	−43.857	1.00	36.13	C
ATOM	4608	C	GLY	B	126	32.140	−16.496	−44.731	1.00	36.09	C
ATOM	4609	O	GLY	B	126	32.898	−17.146	−45.483	1.00	36.20	O
ATOM	4610	N	LEU	B	127	32.169	−15.164	−44.618	1.00	27.66	N
ATOM	4611	CA	LEU	B	127	33.179	−14.326	−45.293	1.00	27.62	C
ATOM	4612	C	LEU	B	127	34.330	−14.056	−44.281	1.00	27.49	C
ATOM	4613	O	LEU	B	127	34.119	−13.461	−43.177	1.00	27.36	O
ATOM	4614	CB	LEU	B	127	32.586	−12.994	−45.842	1.00	27.58	C
ATOM	4615	CG	LEU	B	127	31.526	−13.005	−46.970	1.00	27.69	C
ATOM	4616	CD1	LEU	B	127	30.531	−11.856	−46.867	1.00	27.62	C
ATOM	4617	CD2	LEU	B	127	32.167	−13.006	−48.350	1.00	27.78	C
ATOM	4618	N	GLU	B	128	35.520	−14.560	−44.649	1.00	54.37	N
ATOM	4619	CA	GLU	B	128	36.760	−14.384	−43.869	1.00	54.27	C
ATOM	4620	C	GLU	B	128	37.913	−13.956	−44.795	1.00	54.34	C
ATOM	4621	O	GLU	B	128	37.694	−13.680	−45.983	1.00	54.44	O
ATOM	4622	CB	GLU	B	128	37.119	−15.670	−43.123	1.00	54.29	C
ATOM	4623	CG	GLU	B	128	38.119	−16.558	−43.830	1.00	54.44	C

ATOM	4624	CD	GLU	B	128	37.544	−17.913	−44.120	1.00	54.60	C
ATOM	4625	OE1	GLU	B	128	37.619	−18.802	−43.227	1.00	54.59	O
ATOM	4626	OE2	GLU	B	128	37.015	−18.076	−45.256	1.00	54.75	O
ATOM	4627	N	PHE	B	129	39.135	−13.924	−44.264	1.00	36.94	N
ATOM	4628	CA	PHE	B	129	40.296	−13.520	−45.049	1.00	37.02	C
ATOM	4629	C	PHE	B	129	41.467	−14.454	−44.866	1.00	37.08	C
ATOM	4630	O	PHE	B	129	41.718	−14.995	−43.758	1.00	37.00	O
ATOM	4631	CB	PHE	B	129	40.699	−12.074	−44.750	1.00	36.93	C
ATOM	4632	CG	PHE	B	129	39.632	−11.088	−45.095	1.00	36.90	C
ATOM	4633	CD1	PHE	B	129	39.198	−10.957	−46.422	1.00	37.01	C
ATOM	4634	CD2	PHE	B	129	39.029	−10.314	−44.108	1.00	36.77	C
ATOM	4635	CE1	PHE	B	129	38.171	−10.049	−46.774	1.00	36.98	C
ATOM	4636	CE2	PHE	B	129	37.997	−9.390	−44.439	1.00	36.76	C
ATOM	4637	CZ	PHE	B	129	37.563	−9.262	−45.772	1.00	36.86	C
ATOM	4638	N	GLN	B	130	42.171	−14.626	−45.989	1.00	40.19	N
ATOM	4639	CA	GLN	B	130	43.243	−15.613	−46.124	1.00	40.32	C
ATOM	4640	C	GLN	B	130	44.402	−15.100	−46.997	1.00	40.44	C
ATOM	4641	O	GLN	B	130	44.158	−14.381	−47.951	1.00	40.50	O
ATOM	4642	CB	GLN	B	130	42.661	−16.938	−46.670	1.00	40.48	C
ATOM	4643	CG	GLN	B	130	41.570	−17.562	−45.726	1.00	40.38	C
ATOM	4644	CD	GLN	B	130	41.649	−19.087	−45.579	1.00	40.53	C
ATOM	4645	OE1	GLN	B	130	41.439	−19.835	−46.550	1.00	40.74	O
ATOM	4646	NE2	GLN	B	130	41.931	−19.551	−44.362	1.00	40.42	N
ATOM	4647	N	LYS	B	131	45.643	−15.479	−46.636	1.00	43.97	N
ATOM	4648	CA	LYS	B	131	46.859	−15.086	−47.366	1.00	44.11	C
ATOM	4649	C	LYS	B	131	46.671	−15.344	−48.853	1.00	44.32	C
ATOM	4650	O	LYS	B	131	45.876	−16.217	−49.250	1.00	44.40	O

ATOM	4651	CB	LYS	B	131	48.131	−15.897	−47.005	1.00	44.21	C
ATOM	4652	CG	LYS	B	131	48.373	−16.374	−45.579	1.00	44.05	C
ATOM	4653	CD	LYS	B	131	49.830	−16.918	−45.408	1.00	44.17	C
ATOM	4654	CE	LYS	B	131	49.955	−18.454	−45.588	1.00	44.34	C
ATOM	4655	NZ	LYS	B	131	51.334	−18.905	−46.116	1.00	44.58	N1+
ATOM	4656	N	ASN	B	132	47.437	−14.605	−49.663	1.00	34.69	N
ATOM	4657	CA	ASN	B	132	47.532	−14.862	−51.079	1.00	34.91	C
ATOM	4658	C	ASN	B	132	46.152	−15.054	−51.662	1.00	34.89	C
ATOM	4659	O	ASN	B	132	45.887	−16.008	−52.380	1.00	35.06	O
ATOM	4660	CB	ASN	B	132	48.365	−16.119	−51.300	1.00	35.12	C
ATOM	4661	CG	ASN	B	132	49.651	−16.110	−50.505	1.00	35.12	C
ATOM	4662	OD1	ASN	B	132	50.419	−15.146	−50.550	1.00	35.13	O
ATOM	4663	ND2	ASN	B	132	49.902	−17.194	−49.783	1.00	35.14	N
ATOM	4664	N	LYS	B	133	45.254	−14.169	−51.276	1.00	27.91	N
ATOM	4665	CA	LYS	B	133	43.963	−14.088	−51.893	1.00	27.89	C
ATOM	4666	C	LYS	B	133	43.626	−12.607	−52.058	1.00	27.77	C
ATOM	4667	O	LYS	B	133	44.160	−11.743	−51.347	1.00	27.67	O
ATOM	4668	CB	LYS	B	133	42.918	−14.849	−51.076	1.00	27.79	C
ATOM	4669	CG	LYS	B	133	42.978	−16.348	−51.312	1.00	27.96	C
ATOM	4670	CD	LYS	B	133	41.932	−17.136	−50.511	1.00	27.89	C
ATOM	4671	CE	LYS	B	133	42.321	−18.678	−50.331	1.00	28.07	C
ATOM	4672	NZ	LYS	B	133	43.123	−19.106	−49.083	1.00	27.99	N1+
ATOM	4673	N	ASP	B	134	42.782	−12.329	−53.050	1.00	35.95	N
ATOM	4674	CA	ASP	B	134	42.359	−10.973	−53.370	1.00	35.87	C
ATOM	4675	C	ASP	B	134	40.869	−10.832	−53.059	1.00	35.73	C
ATOM	4676	O	ASP	B	134	40.073	−11.752	−53.290	1.00	35.77	O
ATOM	4677	CB	ASP	B	134	42.648	−10.652	−54.834	1.00	36.02	C

ATOM	4678	CG	ASP	B	134	44.089	−10.942	−55.213	1.00	36.20	C
ATOM	4679	OD1	ASP	B	134	45.023	−10.550	−54.441	1.00	36.17	O
ATOM	4680	OD2	ASP	B	134	44.271	−11.569	−56.287	1.00	36.37	O
ATOM	4681	N	TYR	B	135	40.520	−9.684	−52.487	1.00	28.04	N
ATOM	4682	CA	TYR	B	135	39.160	−9.382	−52.059	1.00	27.91	C
ATOM	4683	C	TYR	B	135	38.836	−7.921	−52.478	1.00	27.87	C
ATOM	4684	O	TYR	B	135	39.652	−7.008	−52.291	1.00	27.88	O
ATOM	4685	CB	TYR	B	135	39.017	−9.623	−50.536	1.00	27.79	C
ATOM	4686	CG	TYR	B	135	39.574	−10.981	−49.994	1.00	27.83	C
ATOM	4687	CD1	TYR	B	135	38.712	−12.071	−49.659	1.00	27.83	C
ATOM	4688	CD2	TYR	B	135	40.948	−11.166	−49.796	1.00	27.88	C
ATOM	4689	CE1	TYR	B	135	39.223	−13.309	−49.162	1.00	27.88	C
ATOM	4690	CE2	TYR	B	135	41.459	−12.391	−49.304	1.00	27.92	C
ATOM	4691	CZ	TYR	B	135	40.605	−13.452	−48.993	1.00	27.92	C
ATOM	4692	OH	TYR	B	135	41.187	−14.619	−48.518	1.00	27.98	O
ATOM	4693	N	TYR	B	136	37.653	−7.727	−53.068	1.00	30.21	N
ATOM	4694	CA	TYR	B	136	37.272	−6.485	−53.806	1.00	30.20	C
ATOM	4695	C	TYR	B	136	36.092	−5.808	−53.114	1.00	30.09	C
ATOM	4696	O	TYR	B	136	35.016	−6.399	−52.974	1.00	30.05	O
ATOM	4697	CB	TYR	B	136	36.873	−6.764	−55.272	1.00	30.28	C
ATOM	4698	CG	TYR	B	136	37.802	−7.689	−56.000	1.00	30.42	C
ATOM	4699	CD1	TYR	B	136	38.678	−7.210	−56.964	1.00	30.53	C
ATOM	4700	CD2	TYR	B	136	37.802	−9.053	−55.713	1.00	30.47	C
ATOM	4701	CE1	TYR	B	136	39.533	−8.077	−57.627	1.00	30.68	C
ATOM	4702	CE2	TYR	B	136	38.646	−9.914	−56.329	1.00	30.63	C
ATOM	4703	CZ	TYR	B	136	39.517	−9.441	−57.292	1.00	30.73	C
ATOM	4704	OH	TYR	B	136	40.357	−10.356	−57.922	1.00	30.91	O

ATOM	4705	N	ILE	B	137	36.293	−4.569	−52.690	1.00	27.11	N
ATOM	4706	CA	ILE	B	137	35.267	−3.834	−51.986	1.00	27.03	C
ATOM	4707	C	ILE	B	137	34.905	−2.676	−52.885	1.00	27.07	C
ATOM	4708	O	ILE	B	137	35.811	−1.960	−53.295	1.00	27.13	O
ATOM	4709	CB	ILE	B	137	35.792	−3.332	−50.589	1.00	26.98	C
ATOM	4710	CG1	ILE	B	137	36.388	−4.499	−49.777	1.00	26.95	C
ATOM	4711	CG2	ILE	B	137	34.694	−2.602	−49.809	1.00	26.93	C
ATOM	4712	CD1	ILE	B	137	35.833	−4.670	−48.355	1.00	26.85	C
ATOM	4713	N	ILE	B	138	33.618	−2.501	−53.216	1.00	30.53	N
ATOM	4714	CA	ILE	B	138	33.160	−1.381	−54.080	1.00	30.56	C
ATOM	4715	C	ILE	B	138	31.811	−0.801	−53.602	1.00	30.52	C
ATOM	4716	O	ILE	B	138	31.228	−1.275	−52.629	1.00	30.47	O
ATOM	4717	CB	ILE	B	138	32.992	−1.780	−55.647	1.00	30.60	C
ATOM	4718	CG1	ILE	B	138	31.637	−2.461	−55.889	1.00	30.56	C
ATOM	4719	CG2	ILE	B	138	34.189	−2.617	−56.218	1.00	30.67	C
ATOM	4720	CD1	ILE	B	138	31.677	−3.503	−56.923	1.00	30.60	C
ATOM	4721	N	SER	B	139	31.322	0.222	−54.305	1.00	27.04	N
ATOM	4722	CA	SER	B	139	29.872	0.396	−54.429	1.00	27.02	C
ATOM	4723	C	SER	B	139	29.495	0.838	−55.832	1.00	27.03	C
ATOM	4724	O	SER	B	139	30.279	1.450	−56.534	1.00	27.08	O
ATOM	4725	CB	SER	B	139	29.258	1.326	−53.401	1.00	27.03	C
ATOM	4726	OG	SER	B	139	27.841	1.156	−53.449	1.00	27.01	O
ATOM	4727	N	THR	B	140	28.274	0.504	−56.213	1.00	27.00	N
ATOM	4728	CA	THR	B	140	27.692	0.961	−57.453	1.00	27.00	C
ATOM	4729	C	THR	B	140	26.838	2.187	−57.194	1.00	27.01	C
ATOM	4730	O	THR	B	140	26.196	2.703	−58.109	1.00	27.01	O
ATOM	4731	CB	THR	B	140	26.857	−0.156	−58.098	1.00	26.98	C

ATOM 4732 OG1 THR B 140 25.845 −0.622 −57.196 1.00 26.97 O
ATOM 4733 CG2 THR B 140 27.747 −1.327 −58.423 1.00 27.00 C
ATOM 4734 N SER B 141 26.827 2.645 −55.943 1.00 27.04 N
ATOM 4735 CA SER B 141 26.100 3.850 −55.564 1.00 27.09 C
ATOM 4736 C SER B 141 26.525 5.016 −56.458 1.00 27.14 C
ATOM 4737 O SER B 141 27.704 5.086 −56.826 1.00 27.16 O
ATOM 4738 CB SER B 141 26.386 4.209 −54.100 1.00 27.14 C
ATOM 4739 OG SER B 141 26.502 3.041 −53.343 1.00 27.08 O
ATOM 4740 N ASN B 142 25.580 5.916 −56.795 1.00 27.18 N
ATOM 4741 CA ASN B 142 25.900 7.187 −57.475 1.00 27.25 C
ATOM 4742 C ASN B 142 26.158 8.307 −56.498 1.00 27.38 C
ATOM 4743 O ASN B 142 26.479 9.419 −56.929 1.00 27.48 O
ATOM 4744 CB ASN B 142 24.787 7.672 −58.393 1.00 27.23 C
ATOM 4745 CG ASN B 142 23.984 8.801 −57.772 1.00 27.34 C
ATOM 4746 OD1 ASN B 142 23.394 8.649 −56.700 1.00 27.37 O
ATOM 4747 ND2 ASN B 142 23.989 9.949 −58.428 1.00 27.41 N
ATOM 4748 N GLY B 143 25.948 8.040 −55.206 1.00 27.39 N
ATOM 4749 CA GLY B 143 26.363 8.954 −54.151 1.00 27.53 C
ATOM 4750 C GLY B 143 25.207 9.746 −53.615 1.00 27.62 C
ATOM 4751 O GLY B 143 25.195 10.089 −52.450 1.00 27.71 O
ATOM 4752 N SER B 144 24.244 10.067 −54.472 1.00 27.62 N
ATOM 4753 CA SER B 144 23.079 10.792 −54.024 1.00 27.72 C
ATOM 4754 C SER B 144 22.290 9.852 −53.125 1.00 27.67 C
ATOM 4755 O SER B 144 22.567 8.652 −53.072 1.00 27.54 O
ATOM 4756 CB SER B 144 22.230 11.306 −55.215 1.00 27.72 C
ATOM 4757 OG SER B 144 21.660 10.271 −56.028 1.00 27.56 O
ATOM 4758 N LEU B 145 21.327 10.412 −52.406 1.00 27.79 N

ATOM 4759 CA LEU B 145 20.351 9.618 −51.679 1.00 27.76 C

ATOM 4760 C LEU B 145 19.359 8.964 −52.653 1.00 27.67 C

ATOM 4761 O LEU B 145 18.666 8.022 −52.271 1.00 27.63 O

ATOM 4762 CB LEU B 145 19.599 10.511 −50.709 1.00 27.95 C

ATOM 4763 CG LEU B 145 18.879 9.902 −49.525 1.00 27.99 C

ATOM 4764 CD1 LEU B 145 19.854 9.275 −48.581 1.00 27.92 C

ATOM 4765 CD2 LEU B 145 18.113 11.023 −48.849 1.00 28.21 C

ATOM 4766 N GLU B 146 19.299 9.482 −53.895 1.00 33.93 N

ATOM 4767 CA GLU B 146 18.397 9.007 −54.971 1.00 33.84 C

ATOM 4768 C GLU B 146 18.884 7.747 −55.666 1.00 33.68 C

ATOM 4769 O GLU B 146 18.079 6.904 −56.054 1.00 33.63 O

ATOM 4770 CB GLU B 146 18.207 10.095 −56.052 1.00 33.88 C

ATOM 4771 CG GLU B 146 17.211 11.220 −55.662 1.00 34.05 C

ATOM 4772 CD GLU B 146 17.748 12.134 −54.549 1.00 34.22 C

ATOM 4773 OE1 GLU B 146 18.975 12.069 −54.292 1.00 34.20 O

ATOM 4774 OE2 GLU B 146 16.964 12.922 −53.949 1.00 34.40 O

ATOM 4775 N GLY B 147 20.200 7.644 −55.827 1.00 27.35 N

ATOM 4776 CA GLY B 147 20.835 6.508 −56.515 1.00 27.23 C

ATOM 4777 C GLY B 147 21.542 5.507 −55.601 1.00 27.20 C

ATOM 4778 O GLY B 147 22.267 4.619 −56.070 1.00 27.12 O

ATOM 4779 N LEU B 148 21.295 5.641 −54.295 1.00 27.26 N

ATOM 4780 CA LEU B 148 21.950 4.845 −53.269 1.00 27.24 C

ATOM 4781 C LEU B 148 21.999 3.372 −53.668 1.00 27.15 C

ATOM 4782 O LEU B 148 23.069 2.745 −53.674 1.00 27.10 O

ATOM 4783 CB LEU B 148 21.213 5.047 −51.924 1.00 27.32 C

ATOM 4784 CG LEU B 148 21.600 4.258 −50.658 1.00 27.30 C

ATOM 4785 CD1 LEU B 148 23.054 3.788 −50.661 1.00 27.22 C

ATOM 4786 CD2 LEU B 148 21.336 5.125 −49.445 1.00 27.41 C
ATOM 4787 N ASP B 149 20.842 2.840 −54.045 1.00 27.15 N
ATOM 4788 CA ASP B 149 20.692 1.409 −54.264 1.00 27.10 C
ATOM 4789 C ASP B 149 20.833 1.042 −55.754 1.00 27.06 C
ATOM 4790 O ASP B 149 20.360 −0.037 −56.149 1.00 27.06 O
ATOM 4791 CB ASP B 149 19.332 0.950 −53.684 1.00 27.17 C
ATOM 4792 CG ASP B 149 19.469 −0.042 −52.508 1.00 27.18 C
ATOM 4793 OD1 ASP B 149 20.458 −0.796 −52.453 1.00 27.13 O
ATOM 4794 OD2 ASP B 149 18.564 −0.093 −51.655 1.00 27.25 O
ATOM 4795 N ASN B 150 21.457 1.933 −56.569 1.00 27.03 N
ATOM 4796 CA ASN B 150 21.755 1.649 −58.005 1.00 26.99 C
ATOM 4797 C ASN B 150 22.678 0.450 −58.008 1.00 26.97 C
ATOM 4798 O ASN B 150 23.762 0.528 −57.460 1.00 26.97 O
ATOM 4799 CB ASN B 150 22.489 2.800 −58.721 1.00 26.98 C
ATOM 4800 CG ASN B 150 21.562 3.911 −59.245 1.00 26.99 C
ATOM 4801 OD1 ASN B 150 20.646 4.338 −58.560 1.00 27.04 O
ATOM 4802 ND2 ASN B 150 21.850 4.413 −60.458 1.00 26.96 N
ATOM 4803 N GLN B 151 22.260 −0.671 −58.587 1.00 26.97 N
ATOM 4804 CA GLN B 151 23.041 −1.933 −58.453 1.00 26.99 C
ATOM 4805 C GLN B 151 24.070 −2.085 −59.593 1.00 26.98 C
ATOM 4806 O GLN B 151 24.684 −3.137 −59.774 1.00 27.02 O
ATOM 4807 CB GLN B 151 22.116 −3.176 −58.397 1.00 27.04 C
ATOM 4808 CG GLN B 151 21.007 −3.187 −57.287 1.00 27.08 C
ATOM 4809 CD GLN B 151 21.472 −3.554 −55.839 1.00 27.09 C
ATOM 4810 OE1 GLN B 151 20.873 −3.090 −54.864 1.00 27.10 O
ATOM 4811 NE2 GLN B 151 22.494 −4.407 −55.713 1.00 27.09 N
ATOM 4812 N GLU B 152 24.246 −1.028 −60.370 1.00 41.10 N

ATOM	4813	CA	GLU	B	152	24.967	−1.133	−61.601	1.00	41.10	C
ATOM	4814	C	GLU	B	152	25.490	0.265	−61.922	1.00	41.08	C
ATOM	4815	O	GLU	B	152	24.767	1.262	−61.750	1.00	41.06	O
ATOM	4816	CB	GLU	B	152	24.005	−1.651	−62.665	1.00	41.10	C
ATOM	4817	CG	GLU	B	152	24.545	−2.807	−63.455	1.00	41.15	C
ATOM	4818	CD	GLU	B	152	25.659	−2.372	−64.396	1.00	41.16	C
ATOM	4819	OE1	GLU	B	152	25.354	−1.699	−65.430	1.00	41.12	O
ATOM	4820	OE2	GLU	B	152	26.836	−2.700	−64.099	1.00	41.21	O
ATOM	4821	N	GLY	B	153	26.756	0.337	−62.342	1.00	26.97	N
ATOM	4822	CA	GLY	B	153	27.411	1.604	−62.652	1.00	26.98	C
ATOM	4823	C	GLY	B	153	28.032	2.228	−61.419	1.00	27.02	C
ATOM	4824	O	GLY	B	153	28.946	1.642	−60.795	1.00	27.05	O
ATOM	4825	N	GLY	B	154	27.543	3.430	−61.102	1.00	51.59	N
ATOM	4826	CA	GLY	B	154	27.942	4.166	−59.902	1.00	51.64	C
ATOM	4827	C	GLY	B	154	29.330	4.795	−59.904	1.00	51.73	C
ATOM	4828	O	GLY	B	154	29.902	5.073	−60.974	1.00	51.76	O
ATOM	4829	N	VAL	B	155	29.867	5.006	−58.700	1.00	27.21	N
ATOM	4830	CA	VAL	B	155	31.159	5.652	−58.520	1.00	27.31	C
ATOM	4831	C	VAL	B	155	32.311	4.656	−58.524	1.00	27.32	C
ATOM	4832	O	VAL	B	155	33.446	5.055	−58.365	1.00	27.41	O
ATOM	4833	CB	VAL	B	155	31.177	6.430	−57.202	1.00	27.38	C
ATOM	4834	CG1	VAL	B	155	29.933	7.321	−57.071	1.00	27.40	C
ATOM	4835	CG2	VAL	B	155	31.258	5.471	−56.018	1.00	27.32	C
ATOM	4836	N	CYS	B	156	31.998	3.371	−58.660	1.00	36.62	N
ATOM	4837	CA	CYS	B	156	32.976	2.309	−58.919	1.00	36.63	C
ATOM	4838	C	CYS	B	156	33.490	2.416	−60.375	1.00	36.69	C
ATOM	4839	O	CYS	B	156	34.682	2.236	−60.670	1.00	36.78	O

ATOM	4840	CB	CYS	B	156	32.265	0.955	−58.665	1.00	36.55	C
ATOM	4841	SG	CYS	B	156	33.012	−0.698	−59.139	1.00	36.58	S
ATOM	4842	N	GLN	B	157	32.568	2.750	−61.274	1.00	50.23	N
ATOM	4843	CA	GLN	B	157	32.764	2.660	−62.710	1.00	50.26	C
ATOM	4844	C	GLN	B	157	32.935	4.025	−63.367	1.00	50.31	C
ATOM	4845	O	GLN	B	157	32.888	4.135	−64.581	1.00	50.32	O
ATOM	4846	CB	GLN	B	157	31.516	2.003	−63.307	1.00	50.16	C
ATOM	4847	CG	GLN	B	157	31.690	1.443	−64.713	1.00	50.18	C
ATOM	4848	CD	GLN	B	157	30.377	1.300	−65.511	1.00	50.10	C
ATOM	4849	OE1	GLN	B	157	29.399	0.719	−65.030	1.00	50.04	O
ATOM	4850	NE2	GLN	B	157	30.382	1.792	−66.756	1.00	50.10	N
ATOM	4851	N	THR	B	158	33.119	5.085	−62.594	1.00	27.41	N
ATOM	4852	CA	THR	B	158	33.163	6.423	−63.206	1.00	27.48	C
ATOM	4853	C	THR	B	158	34.036	7.332	−62.348	1.00	27.62	C
ATOM	4854	O	THR	B	158	34.845	8.103	−62.892	1.00	27.75	O
ATOM	4855	CB	THR	B	158	31.766	6.944	−63.470	1.00	27.40	C
ATOM	4856	OG1	THR	B	158	31.063	6.995	−62.229	1.00	27.36	O
ATOM	4857	CG2	THR	B	158	31.021	6.010	−64.452	1.00	27.28	C
ATOM	4858	N	ARG	B	159	33.911	7.213	−61.020	1.00	27.60	N
ATOM	4859	CA	ARG	B	159	35.115	7.324	−60.186	1.00	27.71	C
ATOM	4860	C	ARG	B	159	35.516	5.858	−59.979	1.00	27.63	C
ATOM	4861	O	ARG	B	159	34.706	4.951	−60.235	1.00	27.51	O
ATOM	4862	CB	ARG	B	159	34.893	8.071	−58.866	1.00	27.76	C
ATOM	4863	CG	ARG	B	159	35.938	9.168	−58.618	1.00	27.96	C
ATOM	4864	CD	ARG	B	159	35.629	9.970	−57.351	1.00	28.03	C
ATOM	4865	NE	ARG	B	159	34.849	11.193	−57.589	1.00	28.12	N
ATOM	4866	CZ	ARG	B	159	35.365	12.396	−57.849	1.00	28.32	C

ATOM	4867	NH1	ARG	B	159	36.672	12.581	−57.928	1.00	28.47	N
ATOM	4868	NH2	ARG	B	159	34.569	13.431	−58.042	1.00	28.40	N
ATOM	4869	N	ALA	B	160	36.763	5.627	−59.574	1.00	27.72	N
ATOM	4870	CA	ALA	B	160	37.261	4.286	−59.269	1.00	27.67	C
ATOM	4871	C	ALA	B	160	37.138	4.006	−57.764	1.00	27.61	C
ATOM	4872	O	ALA	B	160	38.081	3.553	−57.122	1.00	27.64	O
ATOM	4873	CB	ALA	B	160	38.703	4.149	−59.745	1.00	27.81	C
ATOM	4874	N	MET	B	161	35.964	4.310	−57.214	1.00	27.53	N
ATOM	4875	CA	MET	B	161	35.635	3.992	−55.835	1.00	27.46	C
ATOM	4876	C	MET	B	161	35.528	2.478	−55.669	1.00	27.35	C
ATOM	4877	O	MET	B	161	34.439	1.897	−55.848	1.00	27.27	O
ATOM	4878	CB	MET	B	161	34.298	4.617	−55.437	1.00	27.41	C
ATOM	4879	CG	MET	B	161	34.371	6.054	−54.992	1.00	27.53	C
ATOM	4880	SD	MET	B	161	33.560	6.095	−53.391	1.00	27.48	S
ATOM	4881	CE	MET	B	161	34.649	7.195	−52.527	1.00	27.64	C
ATOM	4882	N	LYS	B	162	36.674	1.868	−55.338	1.00	37.41	N
ATOM	4883	CA	LYS	B	162	36.831	0.405	−55.218	1.00	37.35	C
ATOM	4884	C	LYS	B	162	38.155	0.083	−54.510	1.00	37.40	C
ATOM	4885	O	LYS	B	162	39.235	0.446	−54.995	1.00	37.52	O
ATOM	4886	CB	LYS	B	162	36.778	−0.287	−56.613	1.00	37.38	C
ATOM	4887	CG	LYS	B	162	37.892	0.135	−57.571	1.00	37.51	C
ATOM	4888	CD	LYS	B	162	37.432	0.220	−58.993	1.00	37.53	C
ATOM	4889	CE	LYS	B	162	37.721	−1.055	−59.763	1.00	37.57	C
ATOM	4890	NZ	LYS	B	162	37.441	−0.872	−61.218	1.00	37.61	N1+
ATOM	4891	N	ILE	B	163	38.094	−0.592	−53.371	1.00	27.30	N
ATOM	4892	CA	ILE	B	163	39.331	−0.981	−52.711	1.00	27.34	C
ATOM	4893	C	ILE	B	163	39.722	−2.349	−53.247	1.00	27.35	C

ATOM	4894	O	ILE	B	163	38.879	−3.240	−53.323	1.00	27.29	O
ATOM	4895	CB	ILE	B	163	39.200	−0.965	−51.157	1.00	27.25	C
ATOM	4896	CG1	ILE	B	163	38.616	0.354	−50.694	1.00	27.25	C
ATOM	4897	CG2	ILE	B	163	40.540	−1.141	−50.480	1.00	27.29	C
ATOM	4898	CD1	ILE	B	163	38.809	0.589	−49.249	1.00	27.21	C
ATOM	4899	N	LEU	B	164	40.976	−2.493	−53.673	1.00	30.73	N
ATOM	4900	CA	LEU	B	164	41.593	−3.829	−53.848	1.00	30.77	C
ATOM	4901	C	LEU	B	164	42.534	−4.156	−52.643	1.00	30.76	C
ATOM	4902	O	LEU	B	164	43.562	−3.485	−52.405	1.00	30.84	O
ATOM	4903	CB	LEU	B	164	42.344	−3.979	−55.185	1.00	30.93	C
ATOM	4904	CG	LEU	B	164	43.205	−5.256	−55.369	1.00	31.03	C
ATOM	4905	CD1	LEU	B	164	42.532	−6.568	−54.955	1.00	30.96	C
ATOM	4906	CD2	LEU	B	164	43.648	−5.355	−56.807	1.00	31.20	C
ATOM	4907	N	MET	B	165	42.168	−5.196	−51.894	1.00	27.40	N
ATOM	4908	CA	MET	B	165	42.939	−5.617	−50.750	1.00	27.36	C
ATOM	4909	C	MET	B	165	43.815	−6.776	−51.176	1.00	27.47	C
ATOM	4910	O	MET	B	165	43.306	−7.884	−51.313	1.00	27.46	O
ATOM	4911	CB	MET	B	165	41.993	−6.085	−49.643	1.00	27.20	C
ATOM	4912	CG	MET	B	165	41.354	−4.981	−48.797	1.00	27.11	C
ATOM	4913	SD	MET	B	165	40.278	−5.619	−47.476	1.00	26.94	S
ATOM	4914	CE	MET	B	165	38.761	−5.882	−48.377	1.00	26.94	C
ATOM	4915	N	LYS	B	166	45.113	−6.536	−51.390	1.00	27.60	N
ATOM	4916	CA	LYS	B	166	46.080	−7.617	−51.729	1.00	27.73	C
ATOM	4917	C	LYS	B	166	46.539	−8.292	−50.445	1.00	27.64	C
ATOM	4918	O	LYS	B	166	47.482	−7.838	−49.797	1.00	27.65	O
ATOM	4919	CB	LYS	B	166	47.325	−7.078	−52.446	1.00	27.92	C
ATOM	4920	CG	LYS	B	166	47.082	−6.255	−53.729	1.00	28.02	C

ATOM 4921 CD LYS B 166 47.596 −6.959 −55.006 1.00 28.22 C

ATOM 4922 CE LYS B 166 46.466 −7.593 −55.766 1.00 28.19 C

ATOM 4923 NZ LYS B 166 45.799 −6.560 −56.596 1.00 28.17 N1+

ATOM 4924 N VAL B 167 45.856 −9.360 −50.048 1.00 65.77 N

ATOM 4925 CA VAL B 167 46.125 −9.959 −48.729 1.00 65.66 C

ATOM 4926 C VAL B 167 47.397 −10.805 −48.794 1.00 65.79 C

ATOM 4927 O VAL B 167 47.704 −11.416 −49.852 1.00 65.97 O

ATOM 4928 CB VAL B 167 44.918 −10.768 −48.161 1.00 65.53 C

ATOM 4929 CG1 VAL B 167 45.386 −11.958 −47.272 1.00 65.50 C

ATOM 4930 CG2 VAL B 167 44.015 −9.822 −47.379 1.00 65.36 C

ATOM 4931 N GLY B 168 48.129 −10.808 −47.672 1.00 27.51 N

ATOM 4932 CA GLY B 168 49.370 −11.557 −47.556 1.00 27.62 C

ATOM 4933 C GLY B 168 50.449 −10.957 −48.447 1.00 27.83 C

ATOM 4934 O GLY B 168 50.332 −10.914 −49.686 1.00 27.98 O

TER 4935 GLY B 168

HETATM 1 C1 LIG d 0 29.947 −20.867 −37.837 0.00 0.00 C

HETATM 2 C2 LIG d 0 29.878 −20.441 −36.398 0.00 0.00 C

HETATM 3 N3 LIG d 0 29.760 −19.068 −36.156 0.00 0.00 N

HETATM 4 C6 LIG d 0 29.949 −19.916 −38.788 0.00 0.00 C

HETATM 5 C9 LIG d 0 29.739 −18.140 −37.185 0.00 0.00 C

HETATM 6 O12 LIG d 0 29.907 −21.282 −35.498 0.00 0.00 O

HETATM 7 C13 LIG d 0 29.848 −18.519 −38.460 0.00 0.00 C

HETATM 8 C7 LIG d 0 29.641 −18.592 −34.774 0.00 0.00 C

HETATM 9 C8 LIG d 0 28.218 −18.722 −34.214 0.00 0.00 C

HETATM 10 N15 LIG d 0 27.295 −19.292 −35.068 0.00 0.00 N

HETATM 11 H7 LIG d 0 27.602 −19.646 −35.967 0.00 0.00 H

HETATM 12 O18 LIG d 0 27.930 −18.365 −33.073 0.00 0.00 O

```
HETATM   13  C16 LIG d   0      25.903 −19.491 −34.679  0.00  0.00           C
HETATM   14  C10 LIG d   0      25.328 −20.923 −34.952  0.00  0.00           C
HETATM   15  C24 LIG d   0      26.372 −21.704 −35.787  0.00  0.00           C
HETATM   16  C25 LIG d   0      24.020 −20.805 −35.765  0.00  0.00           C
HETATM   17  C11 LIG d   0      25.021 −21.711 −33.652  0.00  0.00           C
HETATM   18  C17 LIG d   0      24.158 −22.826 −33.683  0.00  0.00           C
HETATM   19  C19 LIG d   0      23.861 −23.542 −32.524  0.00  0.00           C
HETATM   20  F21 LIG d   0      23.036 −24.597 −32.588  0.00  0.00           F
HETATM   21  C26 LIG d   0      24.405 −23.176 −31.303  0.00  0.00           C
HETATM   22  C23 LIG d   0      25.253 −22.077 −31.239  0.00  0.00           C
HETATM   23  C22 LIG d   0      25.555 −21.354 −32.398  0.00  0.00           C
HETATM   24  C4  LIG d   0      29.975 −22.289 −38.299  0.00  0.00           C
HETATM   25  N5  LIG d   0      28.876 −22.721 −39.001  0.00  0.00           N
HETATM   26  H1  LIG d   0      28.107 −22.064 −39.135  0.00  0.00           H
HETATM   27  O14 LIG d   0      30.946 −23.015 −38.094  0.00  0.00           O
HETATM   28  C20 LIG d   0      28.786 −24.045 −39.597  0.00  0.00           C
HETATM   29  C27 LIG d   0      29.129 −23.985 −41.090  0.00  0.00           C
HETATM   30  C29 LIG d   0      28.973 −25.348 −41.760  0.00  0.00           C
HETATM   31  C31 LIG d   0      27.583 −25.933 −41.532  0.00  0.00           C
HETATM   32  C30 LIG d   0      27.237 −25.996 −40.046  0.00  0.00           C
HETATM   33  C28 LIG d   0      27.388 −24.633 −39.376  0.00  0.00           C
END
```
